# Structural insights into DNA sequence recognition by Type ISP restriction-modification enzymes

**DOI:** 10.1093/nar/gkw154

**Published:** 2016-03-14

**Authors:** Manasi Kulkarni, Neha Nirwan, Kara van Aelst, Mark D. Szczelkun, Kayarat Saikrishnan

**Affiliations:** 1 Division of Biology, Indian Institute of Science Education and Research, Pune 411008, India; 2 DNA-Protein Interactions Unit, School of Biochemistry, Medical Sciences Building, University of Bristol, Bristol BS8 1TD, UK

## Abstract

Engineering restriction enzymes with new sequence specificity has been an unaccomplished challenge, presumably because of the complexity of target recognition. Here we report detailed analyses of target recognition by Type ISP restriction-modification enzymes. We determined the structure of the Type ISP enzyme LlaGI bound to its target and compared it with the previously reported structure of a close homologue that binds to a distinct target, LlaBIII. The comparison revealed that, although the two enzymes use almost a similar set of structural elements for target recognition, the residues that read the bases vary. Change in specificity resulted not only from appropriate substitution of amino acids that contacted the bases but also from new contacts made by positionally distinct residues directly or through a water bridge. Sequence analyses of 552 Type ISP enzymes showed that the structural elements involved in target recognition of LlaGI and LlaBIII were structurally well-conserved but sequentially less-conserved. In addition, the residue positions within these structural elements were under strong evolutionary constraint, highlighting the functional importance of these regions. The comparative study helped decipher a partial consensus code for target recognition by Type ISP enzymes.

## INTRODUCTION

Protein–nucleic acid interactions are central to a large number of important cellular functions. The interactions can be broadly classified into either sequence specific or sequence independent interaction. Sequence independent binding is primarily established by protein residues via ionic and hydrogen bonds with the sugar-phosphate DNA backbone, and/or by stacking interactions with bases. Sequence specificity is established via base-specific interaction made by protein residues (1). Understanding the molecular details of how proteins bind to nucleic acid has not only contributed to the understanding of the biology of the system, but has also contributed to our ability to engineer proteins with new binding specificity.

Engineering proteins to bind specific nucleic acid sequences has been in the forefront of the research activities driving biotechnological breakthroughs. Successful examples of reagents generated from nucleic acid binding proteins include zinc-finger domains coupled to an endonuclease, where the zinc-fingers have been engineered to recognize different target sites, and transcription activator-like effector nucleases (TALENs), made of nuclease-fused arrays of TAL domains that recognize specific sequences (2). Furthermore, Pumilio FBF homology (3) and pentatricopeptide proteins (4) have been engineered for new RNA sequence specificity. However, engineering new specificities has been hitherto successful using repeating modular and non-catalytic units that recognize short nucleic acid sequences (a single or a few nucleobases).

Engineering specificities of other nucleic acid binding proteins has in general had poor success. This is despite detailed understanding of how a large variety of proteins that participate in different cellular functions recognize specific DNA sequences. A limited exception to this is the engineering of new specificities in meganucleases (5). Among DNA binding proteins, restriction enzymes were amongst the first that were studied biochemically, biophysically and structurally towards rationally designing new specificities. For example, detailed studies of the enzymes BamHI, BglII and BstYI, which recognize similar sequences, demonstrated that the enzymes use different recognition strategies, which hindered rational design of new specificities (6–8).

Subsequent efforts included use of the MmeI family of Type IIL restriction-modification (RM) enzymes, which have both endonuclease and methyltransferase activities within the same polypeptide. This offers the advantage that change in specificity is produced in both the destructive nuclease and protective methyltransferase. Based on sequence analyses of known Type IIL enzymes and their target sequences, mutations were carried out to generate changes at three positions of their six base pair sequences (9). However, change in specificity at other positions was less successful (9,10). Here, we report the structure of the single polypeptide Type ISP RM enzyme LlaGI, allowing a structural comparison of DNA recognition elements with the homologous Type ISP enzyme LlaBIII (11).

**Figure 1.**
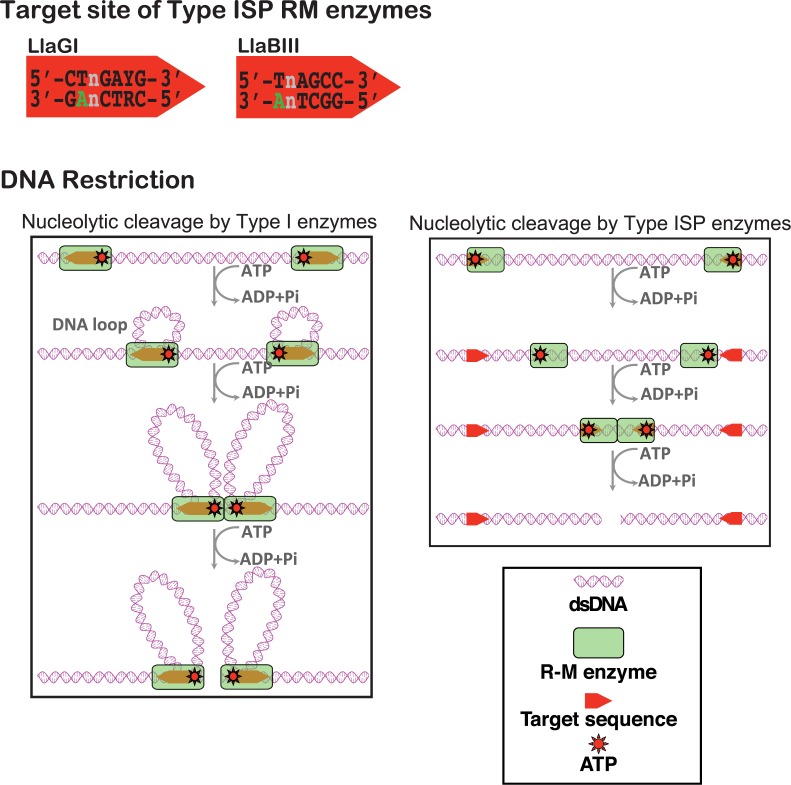
Cartoon illustrating the target sequence of LlaGI and LlaBIII and the translocation-coupled nucleolytic cleavage of DNA by Type I and Type ISP enzymes. Type I enzyme complexes have two MTase, ATPase and nuclease domains, and hence can translocate and cleave DNA either upstream or downstream of the target. For clarity, translocation upstream is not illustrated. Note that in the schematic of Type ISP enzymes ATP is located at the far end to indicate that the ATPase domain is upstream of the target.

**Figure 2.**
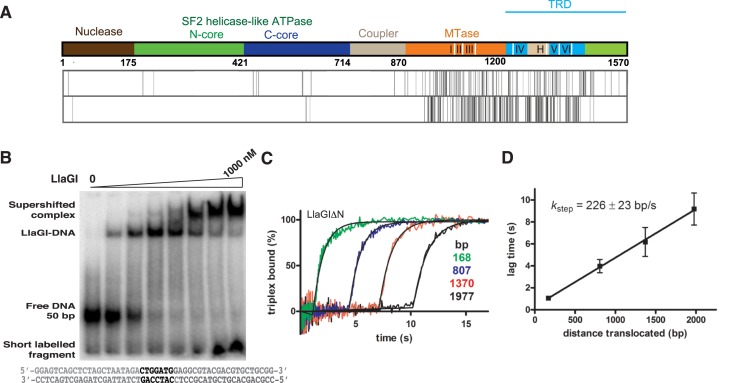
(**A**) The domain organization of LlaGI. The positions of the structural elements that recognize the targets are shown as white lines. The amino acid sequence of LlaGI and LlaBIII were aligned pairwise to identify regions of homology, similarity and difference (4). The vertical lines show positions that have amino acid substitutions that are similar (upper row) or different (lower row)—all other positions are identical. (**B**) EMSA assays of LlaGI binding to 10 nM of a 50 bp DNA substrate with 0, 10, 25, 50, 100, 250, 500 and 1000 nM protein. (**C**) Triplex displacement reactions on linear DNA. Triplex DNA (with different spacing between the LlaGI site and fluorescent triplex) were pre-incubated with enzyme and the reactions initiated with ATP, to give a final concentration of 1 nM DNA, 100 nM LlaGIΔN and 4 mM ATP at 25°C. The triplex displacement profiles, which have lag phases characteristic of a translocating motor protein, were fitted to Equation (1) to obtain the lag time (*T*_app_). (**D**) The linear relationship between *T*_app_ and *d* was used to estimate the translocation rate. The points are the mean and SD for repeat reactions measured using two different preparations of LlaGIΔN.

Type ISP enzymes are similar in domain organization to Type IIL enzymes, except for the insertion of a helicase-like ATPase domain motor that plays an essential role in nucleolytic activity (Figures 1 and 2A). RM enzymes prevent the integration, replication and expression of foreign DNA in the host bacterium by introducing double-strand (ds) DNA breaks into the invading genomes. In case of Type ISP enzymes, the entire gamut of tasks is carried out by the coordinated action of target recognition, methyltransferase (MTase), helicase-like ATPase and nuclease domains that constitute the single polypeptide chain (12). The target recognition domain (TRD) recognizes a specific DNA sequence and the methyltransferase modifies the target adenine. Nucleolytic cleavage of unmodified DNA is triggered by recognition of two DNA binding sites on the same DNA, ATP-dependent long-range communication, and collision of two RM enzymes (Figure 1) (13,14). LlaBIII and LlaGI are the two prototypes of Type ISP RM enzymes (15,16). LlaGI recognizes 5′-CTnGAyG-3′, while LlaBIII recognizes 5′-TnAGCC-3′ (Figure 1) (15,16). DNA cleavage requires the presence of two sites on the same DNA, in an inverted, head-to-head repeat as defined by the arrowheads in Figure 1. Cleavage occurs at a distant location between the two sites, with the distribution of locations concentrated on the midpoint position (11,17).

We recently reported a high-resolution crystal structure of LlaBIII (11). The structure of LlaBIII revealed the molecular architecture and domain organization in a Type ISP enzyme; the mode of target recognition; a mechanism for coupling target recognition and ATPase domain activation; a unique mechanism for translocation coupled nucleolytic activity (11). We found that the LlaBIII nuclease domain was located upstream of the DNA target and ATPase domain. This architecture was inconsistent with the model proposed for DNA cleavage (13), which, akin to Type I enzymes (Figure 1), involved translocation-mediated DNA looping resulting in convergence and collision of two enzymes leading to DNA cleavage. Based on complementary single-molecule magnetic tweezers assay and single cleavage sequence analysis, we found that Type ISP enzymes translocated along DNA without looping, and proposed a nucleolytic mechanism in which DNA break formation resulted from multiple nicks caused by distal nucleases in the collision complex of the enzyme (Figure 1) (11,18).

In continuation of our study of the mechanism of action Type ISP enzymes, we report here the co-crystal structures of a nuclease mutant of LlaGI and a nuclease-deleted mutant LlaGIΔN, bound to DNA substrate mimics. As mentioned earlier, the target of LlaGI is distinct from LlaBIII. But the two enzymes share an amino acid sequence identity of ∼80%, with the target binding MTase-TRD unit having an identity of 58% (Figure 2A). The structures of LlaGI and LlaBIII provided us with a unique opportunity to understand and compare in atomic detail how the two closely related RM enzymes recognized disparate target sequences and characterize the recognition region. Similar studies helped understand the complexities of DNA sequence recognition by zinc-finger proteins and served as a platform in engineering novel zinc-fingers (19).

We also analysed 552 amino acid sequences of Type ISP enzymes to study the conservation of the target recognition region in this family of enzymes. Amino acid sequence comparison of 11 Type ISP enzymes whose targets are known obtained from the REBASE database (20) provided further insights into the correlation between target sequence and amino acids involved in their recognition. This structure comparison and sequence analyses show that target recognition by Type ISP enzymes is complex and that change in target recognition cannot be achieved only by corresponding change in the contacting amino acids. Changes at additional positions are often required to generate specific interactions with the new base. Though a simple code for target recognition could not be obtained, the study led to a consensus and a predictive code that may facilitate engineering new specificities.

## MATERIALS AND METHODS

### Purification of LlaGI and LlaGIΔN

Native 180 kDa LlaGI was overexpressed in 10L of *Escherichia coli* BL21 (DE3) from a recombinant clone of the *llagi* gene in the pRSF vector (15). The highest amount of soluble protein in the crude lysate was observed upon inducing the culture at 25°C with 0.5 mM IPTG at OD_600_ = 0.6. The incubation temperature was lowered from 37 to 25°C before addition of IPTG. Induced cells were harvested after 5 h further incubation. To avoid proteolytic degradation of LlaGI, protease inhibitors (Roche, UK) were added to the lysis buffer (50 mM Tris-HCl pH 8.0, 150 mM NaCl, 10 mM MgCl_2_, 1 mM EDTA and 1 mM DTT), all steps of purification were carried out at 4°C, and the protein was purified to homogeneity within 24 h of lysis. The cells were lysed by sonication. LlaGI was salted-out using 70% w/v ammonium sulphate. The re-suspended protein pellet was further purified by column chromatography using heparin followed by MonoQ. The strongly anionic MonoQ column not only removed protein impurities but also any cellular DNA bound to the enzyme. Finally, size exclusion chromatography using Superdex 200 10/300 (GE Healthcare) ensured homogenous monomeric LlaGI. Equivalent strategies were followed for purification of LlaGIΔN (11). Purified LlaGI and LlaGIΔN were stored in a buffer containing 10 mM Tris-HCl pH 7.4, 100 mM NaCl and 1 mM DTT. Purified samples stored at −80°C remained intact and could be thawed and used for crystallization later.

### Purification of DNA for crystallization

The duplex DNA substrate mimics used for co-crystallization were obtained by annealing the oligos 5′ - TTAGCTAATAGACTGGATGGAGG-3′ and 5′-TCCTCCATCCAGTCTATTAGCTA-3′ for LlaGIΔN-DNA, and 5′-GCTCTAGCTAATAGACTGGATGGAGGTG-3′ and 5′ -CACCTCCATCCAGTCTATTAGCTAGAGC-3′ for LlaGI-DNA complex. The chemically synthesized and PAGE-purified oligos were purchased from Integrated DNA Technologies, USA. The single-strand DNA were annealed and the duplex DNA was purified using a MonoQ column. The pure duplex DNA was concentrated and stored in sterile water at −20°C.

### Electrophoretic mobility shift assay

7% w/v polyacrylamide gels were used to study binding of LlaGI to DNA by electrophoretic mobility shift assay (EMSA). Duplex DNA was generated from complementary single-strand DNA as described above. The 5′-ends of the duplex DNA substrates were labelled with ^32^P using T4 polynucleotide kinase from New England Biolabs, USA. The binding reaction buffer contained 50 mM Tris-HCl pH 7.4, 100 mM NaCl, 10 mM MgCl_2_, 0.01 mg/ml BSA, 1 mM DTT and 10% (v/v) glycerol. The reactions were incubated at 4°C, and the DNA–protein complexes resolved by electrophoresis at room temperature. The gels were dried using a gel dryer, exposed to a phosphor screen, and images recorded using a Typhoon Imager (GE healthcare).

### Translocase assay

Triplex displacement measurements were carried out in an SF61-DX2 stopped-flow fluorimeter as described previously (16). The sample temperature was maintained at 25°C by a water bath connected to the chamber housing the syringes and flow cell. Reactions were started by adding ATP to a DNA/enzyme mix with the final reaction conditions as 1 nM linear DNA (0.5 nM tetramethylrhodamine triplex), 100 nM LlaGIΔN and 4 mM ATP in 50 mM Tris-HCl, pH 8.0, 10 mM MgCl_2_, 1 mM DTT. Data averages were collected from at least three individual time-courses and analysed in KinetAsyst 3.11 (Hi-Tech Scientific) and Prism 4 (GraphPad software, Inc., San Diego). Triplex displacement data were fitted to Equation (1) (modified from Gilhooly and Dillingham, 2014) which defines displacement (*Y*) as a function of time (*t*), as the sum of a background linear rate (with gradient m) and an offset exponential with an x-axis offset at times *T*_app_ (the apparent ‘lag time’), with amplitude *A*, an apparent rate constant of triplex displacement of *k*, and an offset (*C*1) which is the value for free triplex at *t* = *T*_app_:

(1) \begin{equation*} Y = (x < T_{{\rm app}} )(mx) + (x >T_{{\rm app}} )\left( {\left( {A\left( {1 - \left( {\exp ^{ - k(t - T_{{\rm app}} )} } \right)} \right)} \right) + C1} \right) \end{equation*}

The variation of *T*_app_ (s) with distance (*d*, bp) was fitted to:

(2) \begin{equation*} Y = \left( {\frac{1}{{k_{{\rm step}} }}} \right)d + C2 \end{equation*}

Where *k*_step_ is the rate of translocation and *C*2 is the intercept with the y-axis. Although C2 can be used to estimate the initiation time, there are limitations in doing this (16), so this value is not reported here. Note, this uncertainty has no effect on the quality of the *k*_step_ value.

### Crystallization and data collection

A complex of the purified protein and DNA substrate was formed by mixing the two in 1:1.3 molar ratio at 4°C. A protein concentration of 5 mg/ml and a crystallization drop size of 200 nl were used for all the initial crystallization trials by sitting drop vapour diffusion method. The nanodrops were set using a robotic liquid handler. Over 4000 different conditions of varying buffers, additives, precipitants, DNA substrates and temperatures were screened for crystallization of LlaGI-DNA and LlaGIΔN-DNA complex. The most promising conditions were further optimized for growing single crystals using an optimization grid. Crystals grown in nanodrops did not diffract well. Improvement in the diffraction quality of LlaGI-DNA crystals was achieved from larger crystals grown in 2 μl drops at 291 K by the sitting drop method. One such crystal of LlaGI-DNA grown from a 1:1 ratio of protein:reservoir buffer (100 mM MES pH 5.6, 300 mM KCl, 6.5% w/v PEG 20,000) diffracted to 7.40 Å at 100 K, with glycerol as a cryoprotectant. Suitable diffracting crystals of LlaGIΔN were grown in 4 μl hanging drops at 291 K by the vapour diffusion method. The reservoir buffer contained 100 mM Tris-HCl pH 7.4, 20% w/v PEG 20 000, 4% w/v PEG 550 MME and 150–250 mM sodium acetate. One of these crystals diffracted to 2.84 Å at 100 K, with ethylene glycol as cryoprotectant.

### Structure determination

The LlaGI-DNA and LlaGIΔN-DNA diffraction data were collected at the European Synchrotron Radiation Facility ID23–1 and the Diamond Light Source I03 beamlines, and processed using MOSFLM (21) and XDS (22), respectively. The intensities were scaled and merged using AIMLESS (23). The structure solution for LlaGIΔN-DNA crystal was obtained by molecular replacement using the program PHASER (24) and the structure of partially built LlaGIΔN^Se^-DNA (11) as the search model. The coordinates of the ATPase domain of LlaBIII (PDB ID: 4XQK) were used as a guide for building the ATPase domain. The structure solution for LlaGI-DNA crystal was obtained by molecular replacement using the structures of coupler-MTase-TRD unit of LlaGIΔN and nuclease-ATPase unit of LlaBIII (PDB ID: 4XQK) as separate search models.

Initial cycles of structure refinements were carried out by REFMAC5 (25) and subsequently by *phenix.refine* (26). The maps were visualized and model building carried out using COOT (27). In case of LlaGIΔN-DNA structure, positional and isotropic B-factor refinement was carried out. Due to the low resolution of LlaGI-DNA crystal data, only a domain-wise rigid-body refinement was carried out keeping the B-factors constant, and side chain atoms beyond Cβ were truncated and not included in the refinement.

### Amino acid sequence analysis

A database of amino acid sequences of 552 Type ISP enzymes were generated by BLAST (28) using the LlaGI sequence and the non-redundant protein sequences database. Multiple sequences with 100% conservation were reduced to a single sequence. Many of the sequences showed very high sequence conservation in the first half of the ORF. However, much higher levels of variation were observed in the C-terminal half of the MTase domain and in the TRD. Therefore the sequences were not further reduced for redundancy. The sequences were aligned using Clustal Omega (29) and Jalview (30) (Supplementary Data 1). The evolutionary conservation scores for the residues in the MTase-TRD unit were calculated using ConSurf (31). To plot, the scores obtained from ConSurf were multiplied by −1 and scaled up by addition of a constant value of 3.505 so that the lowest conservation score was zero. Evolutionary constraint (EC) strengths for the residues were calculated using the program EVcouplings available online (32). As the program could only handle a sequence length of 600 residues, residues starting from LlaGI-868 to LlaGI-1428 were used for the calculations. Recognition sequences for a subset of the Type ISP enzymes were obtained from REBASE (20) and are based on DNA methylation patterns determined from PacBio sequencing.

## RESULTS

### A nuclease-dead N-terminal deletion mutant of LlaGI is an active translocase

During characterization of LlaGI-DNA complex by EMSA prior to co-crystallization studies, a nucleotide-independent cleavage of single-site substrates resulted in release of short-labelled fragment (Figure 2B). This suggested that even in the absence of ATP, wild-type LlaGI carried a residual nucleolytic activity. To protect the integrity of the DNA during crystallization of LlaGI-DNA complexes, two different nuclease-dead mutants of LlaGI were generated. One of these was a previously characterized point mutant in which the catalytic aspartate at position 78 was mutated to alanine (13). This mutant has wild-type translocation activity. In addition, we sought to generate truncations of LlaGI that removed the nuclease activity whilst retaining translocase activity. Identification of suitable exposed inter-domain loops using partial proteolytic digestion proved unsuccessful (data not shown). As an alternative, we generated a secondary structure prediction of LlaGI using the PSIPRED server (Supplementary Figure S2) (33). From this the region 160–180 was predicted to be a loop between the nuclease and ATPase domains. We chose to delete the residues 2–165, producing the recombinant protein LlaGIΔN (11).

Purification of LlaGIΔN was achieved using the same protocol as with wild-type LlaGI, and the protein did not show any differences in solubility/stability during the preparation. To check for the retention of translocase activity, we utilized the triplex displacement assay (Figure 2B). The displacement profiles show the distance-dependent lag time (*T*_app_) characteristic of a translocating motor and we were able to measure a stepping rate of 226 ± 23 bp/s at 25°C (Figure 2C), in good agreement with the value for the wild-type enzyme under these conditions (250 ± 10 bp/s at 25°C) (17). Moreover, LlaGIΔN was also able to cooperate with Type ISP enzymes to activate DNA nicking at distant non-specific sites (17), consistent with DNA translocation. Both the D78A and ΔN mutants were used for crystallographic studies.

### Molecular architecture of LlaGI bound to DNA

**Figure 3.**
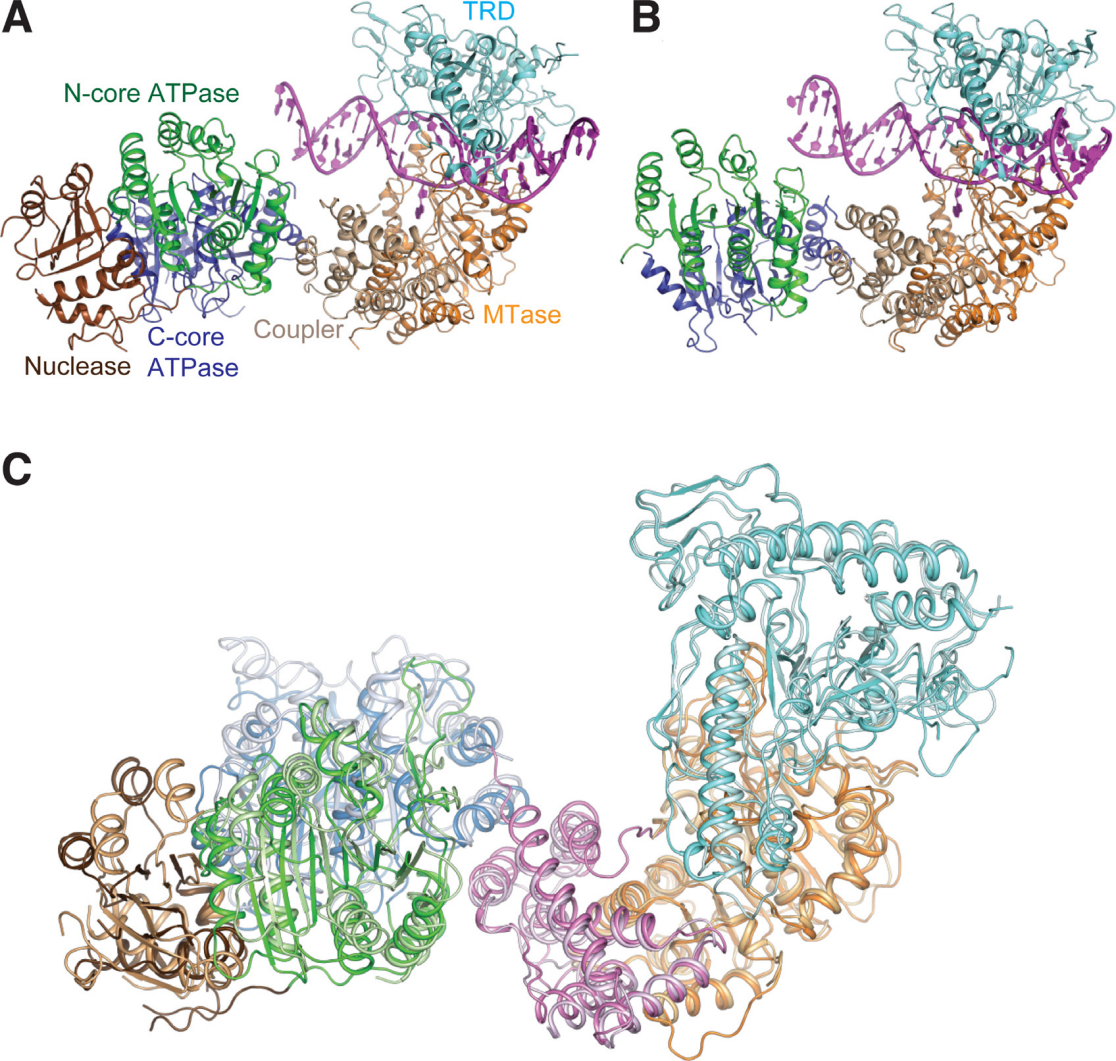
Ribbon representation of (**A**) LlaGI-DNA complex at 7.4 Å resolution and (**B**) LlaGIΔN-DNA complex at 2.8 Å resolution. The six structural domains are coloured distinctly. The nuclease is coloured brown, the N-core of the ATPase domain is coloured green, the C-core is in blue, the coupler in beige, the MTase domain in orange and the TRD in cyan. (**C**) The superposition of the Cα-trace of LlaGI at 7.4 Å and LlaBIII (PDB ID: 4XQK) reveals domain movement. LlaGI was superposed on to LlaBIII with respect to the coupler using Coot (27).

The structures of LlaGIΔN bound to a 22-bp DNA and full-length LlaGI bound to a 28-bp DNA were determined to a resolution of 2.84 and 7.4 Å, respectively (Table 1). In the LlaGIΔN-DNA crystals there were two molecules in the asymmetric unit, while in LlaGI-DNA there were four. Like LlaBIII, LlaGI contained six structural domains: the N-terminal Mrr-family nuclease, followed by the N-core and C-core RecA folds of the SF2 helicase-like ATPase domain, connected to the γ-class of N6-adenine MTase domain and the C-terminal TRD by an all α-helical coupler domain (Figure 3A and B). Comparison of LlaGI-DNA, LlaGIΔN-DNA and LlaBIII-DNA structures revealed interdomain conformational mobility (Figure 3C).

**Table 1.** Data collection and refinement statistics

	LlaGI-DNA	LlaGIΔN-DNA
**Data collection**		
Space group	I2	P2_1_
Cell dimensions		
*a, b, c* (Å)	267.9,203.4,291.5	87.4,222.3,117.4
α, β,γ (°)	90.0,96.2,90.0	90.0,105.1,90.0
Resolution (Å)	50.0–7.4 (8.11–7.40)	50.0–2.84 (2.99–2.84)
*R*_sym_	8.0 (50.7)	8.9 (58.8)
*I* / σ(*I*)	8.0 (2.0)	9.6 (2.0)
Completeness (%)	96.7 (98.4)	99.8 (99.9)
Redundancy	2.0	2.0
**Refinement**		
Resolution (Å)	50–7.4	50–2.84
No. of reflections (total/test)	20176/1040	101504/5107
*R*_work_/*R*_free_	34.6/37.4	22.9/26.2
R.m.s. deviations		
Bond lengths (Å)	0.015	0.004
Bond angles (°)	1.281	0.767
Ramachandran plot (%)		
Favoured	95.4	95.4
Outliers	0.2	0.2

**Figure 4.**
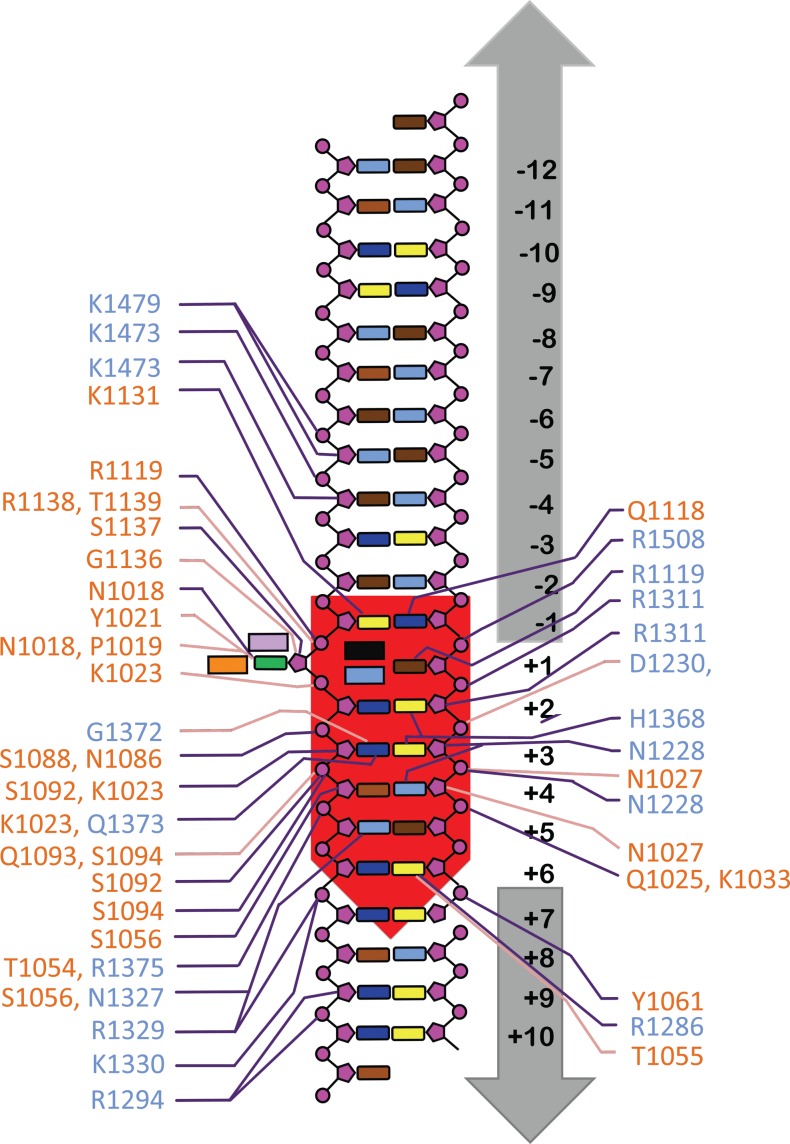
A schematic diagram of protein–DNA interactions in LlaGIΔN-DNA complex. Amino acid colours are according to domains in Figure 2A. DNA bases are cyan (A), brown (T), blue (C) and yellow (G), main chain interactions are pink lines, side chain interactions are purple lines. The target adenine (green) is +1, with downstream positions defined as positive and upstream positions defined as negative. Residues Y1021, F1133, R1119 and R1138 are depicted as lavender, orange, sky blue and black coloured boxes, respectively.

Like in the case of the LlaBIII-DNA complex (11), in both LlaGI-DNA and LlaGIΔN-DNA structures, the MTase domain and TRD held the target in a vice-like grip. The T:A base pair of the LlaGI target that is methylated was designated as position +1 based on the convention used by Chand *et al*. (11) to describe the LlaBIII target. Therefore the first C:G base pair of the LlaGI target was designated as −1. In LlaGIΔN-DNA, the upstream end of the oligonucleotide was not long enough to interact with the ATPase domain (Figures 3B and 4). Instead, this end interacted with the ATPase domain of the neighbouring molecule of the asymmetric unit. In the LlaGI-DNA structure, the base pairs at the ends of the DNA could not be built because of poor density. In the LlaGIΔN structure, only a part of the C-core of chain B could be built with confidence, while the C-core of chain A could not be built due to poor electron density. The lack of electron density could be the result of conformational flexibility of the domains in the crystal lattice.

The structures of two close homologues, LlaGI and LlaBIII, and those of other evolutionarily related multidomain restriction enzymes (Type I and Type IIG), provided us with an opportunity to carry out a detailed comparison of their structure to understand target recognition. This is detailed in the following sections. All the residue numbering in the following sections are based on LlaGI sequence unless specified otherwise.

### The target binding MTase-TRD unit

**Figure 5.**
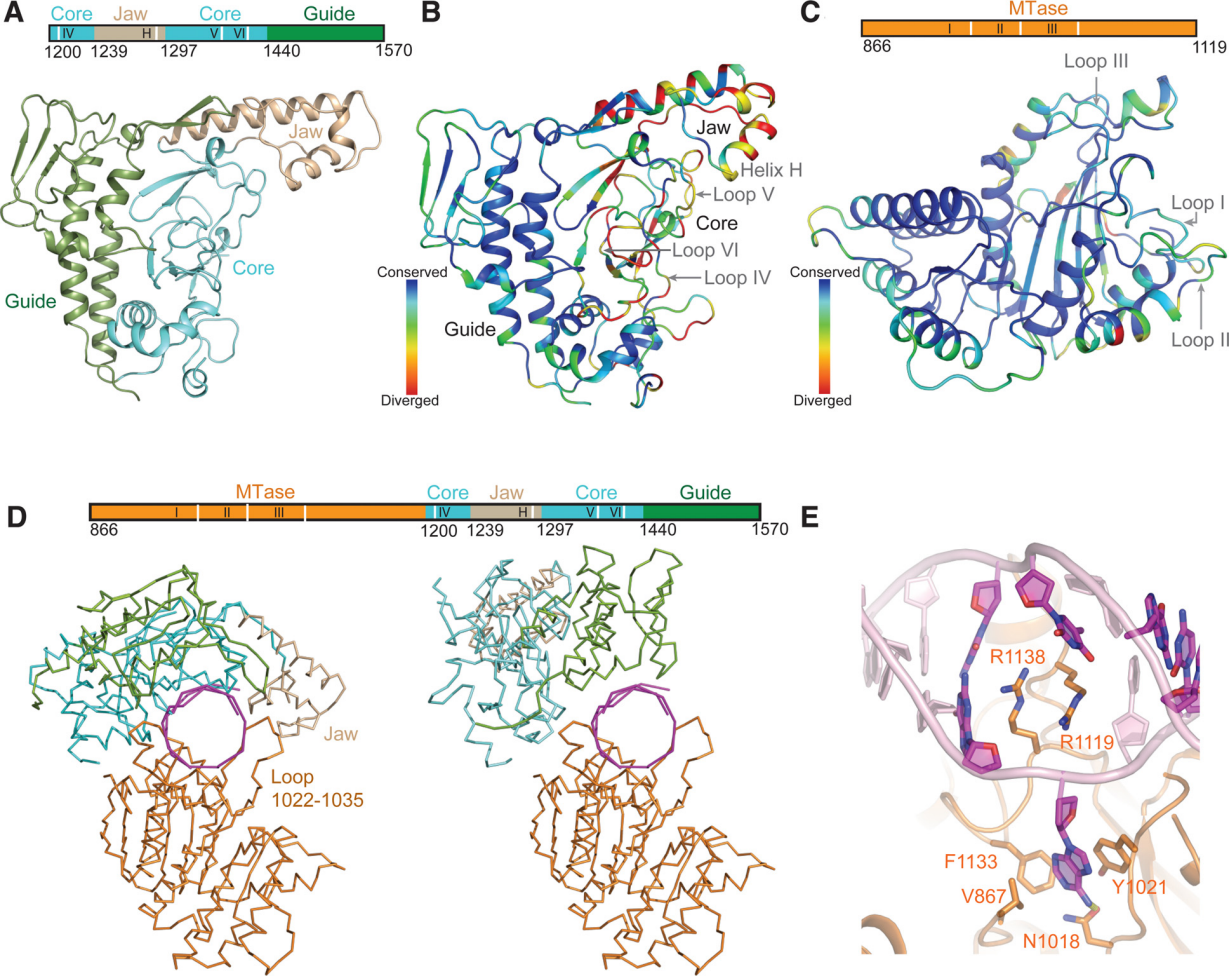
TRD of Type ISP enzyme. (**A**) The three subdomains seen in TRD of LlaGIΔN: the core is coloured cyan, the jaw is coloured wheat and the guide is coloured green. The positions of the structural elements that recognize the targets are shown as white lines in the cartoon above the ribbon diagram. (**B**) Mapping of the evolutionary conservation scores of the Type ISP TRD residues onto the LlaBIII-TRD and (**C**) onto the LlaBIII-MTase domain structure. The positions of the structural elements that recognize the targets are shown as white lines in the cartoon above the ribbon diagram. (**D**) Left: the closed structure of the clamp formed by MTase domain and TRD in LlaGIΔN around the DNA (magenta). Right: the open structure of the clamp modelled based on the apo structure of BpuSI (PDB ID: 3S1S) (36). (**E**) The active site of the LlaGIΔN MTase domain with the target adenine flipped. The structural elements of the MTase domain and TRD that are involved in target recognition are marked.

The structures of LlaGI and LlaBIII showed a conserved mode of DNA binding at the target by a clamp formed by the MTase domain and TRD. The structures of the TRDs of LlaGI and LlaBIII are very similar (RMSD = 1 Å) despite the relatively low amino acid sequence identity of ∼52% (Figure 2A). Structural variation in the form of insertion or deletion of amino acid residues are primarily located in regions not directly involved in DNA recognition (Supplementary Figure S3A). A similar mode of target binding is employed by the prototypical γ-class of N6-adenine MTase domain M. *Taq*I (34). However, the TRD of the Type ISP enzymes makes much more extensive interactions with the DNA (Figure 4) (11). Additionally, the TRD and MTase domain of the Type ISP enzymes form a closed clamp around the target, while in M. *Taq*I the two domains form an open DNA binding cleft. The TRD of LlaGI is larger than that of M.*Taq*I by ∼200 residues and can be divided into three structural subdomains—the core (1200–1239 and 1297–1440), the jaw (1240–1296) and the guide (1440–1578) as illustrated in Figure 5A. Similarly, LlaBIII TRD (Figure 5B) can be delineated into the core (1205–1244) and (1291–1448), the jaw (1245–1288) and the guide (1448–1578).

An analysis of a multiple sequence alignment (MSA) of 552 Type ISP enzyme sequences across the target binding MTase-TRD unit showed that the MTase domain was more strongly conserved in comparison to the TRD (Figure 5B and C). Amongst the TRD subdomains, the jaw appeared to be the least conserved, followed by the core, while the guide appeared to be the most conserved. Structurally, the core has the same fold as the TRD of M.*Taq*I (Supplementary Figure S3B), TRD1 or TRD2 of Type I enzymes (Figure 5B) (35) and the TRD of the Type IIG enzyme BpuSI (Supplementary Figure S3C) (36). This subdomain along with the MTase domain is the primary reader of the target sequence (see below). Visual inspection of the amino acid conservation score mapped onto MTase-TRD unit suggested that the surface of the TRD core that contacted the DNA was not strongly conserved, unlike the recognition elements of the MTase domain (Figure 5B and C). Amino acids of the central β-sheet of the TRD also showed moderate conservation. However, the helices (I1395–I1428) stacked on the central β-sheet were relatively well conserved.

The jaw, an insertion in the core, is made of a three-helix bundle (Figure 5A), which, based on structural similarity search using DALI server (37), was not found in other RM enzymes. The sequence alignment indicated the amino acids of this subdomain to be the least conserved. LlaGI also has inserts in this region compared to LlaBIII (Supplementary Figure S3A). The three helices of the jaw along with the MTase loop S1022-I1035 seal the clamp around the DNA (Figure 5D). One of the jaw helices (1286–1295) fits into the major grove of the deformed target and interacts with the phosphate backbone at +9 (a non-specific position). The clamp may aid the processivity of the translocating enzyme.

The structure of the closed clamp bound to the DNA raises the question as to how the enzyme would assemble on its DNA substrates, which could be a long linear or a closed circular DNA. Comparison of the Type ISP-DNA structures with the apo-structure of BpuSI (36) provided an insight into this question. Superposition of the MTase domain of LlaGI and BpuSI revealed that the TRDs of the two enzymes were rotated by 92° with respect to one another. Consequently, the TRD in the apo-structure of BpuSI would be predicted to be in an open conformation to allow entry of DNA. We suggest that in the apo-form of the Type ISP enzymes, the TRD-MTase interdomain conformation would be in a similar open conformation, facilitating the DNA to slide into a cleft created by the two domains (Figure 5D).

With the limited structural information on Type ISP enzymes, it is difficult to postulate on the transition from the open to the closed conformation. However, deriving inspiration from the studies on the crystal structures of the Type II enzyme BamHI in its apo form, bound to non-specific DNA and specific DNA (38), it may be envisaged that MTase-TRD unit of Type ISP enzyme would transit from the open conformation when not bound to DNA, to a conformationally distinct, and as yet unobserved, state when bound to non-specific DNA, to the closed conformation seen in the crystal structure when bound to specific DNA. The trigger for these conformational changes could be the interactions made by the protein with non-specific and specific DNA. We had earlier postulated that translocation initiation would be accompanied by remodelling of the target bound Type ISP enzyme (11,18).

The C-terminus of the core continues into the guide. The guide has a two layer open-faced β-sandwich structure with a four-stranded N-type Greek key motif layer stacked against a layer of three helices with a β-hairpin loop inserted between two of them (Figure 5A). Based on a Dali search, the fold of the guide was not found in other RM enzymes. Amongst the three subdomains, amino acids of the guide were found to have the highest conservation scores (Figure 5B). The helices at the N-terminus and C-terminus of the subdomain form a bundle, reminiscent of the helical bundle formed by the Central and Distal Conserved Regions of the HsdS subunit of classical Type I enzymes (35,39).

In Type I enzymes, the helical bundle acts as a molecular ruler, spacing the two TRDs for recognition of the two halves of the bipartite target sequences (e.g. GAANNNNNNRTCG for EcoR124I). In the Type ISP enzymes, the helical bundle together with the hairpin loop insertion in the guide forms a concave surface against which the core is packed (Figure 5A). The guide does not participate in target recognition. Instead, the Greek-key motif located at the end of the helical bundle and the hairpin loop steers the DNA upstream of the target. K1473, K1479 and R1508 of the LlaGI guide interact with the phosphate backbone (Figure 4). As in case of LlaBIII (11), the DNA is steered towards the ATPase domain.

The MTase domain is structurally similar to other γ-class adenine-N6 MTases, including the prototypical M. *Taq*I, and the MTase domains of Type I and the Type IIG protein BpuSI. As in the case of LlaBIII, the target adenine to be methylated is flipped out via the minor groove into the active site of the LlaGI MTase domain despite the absence of AdoMet (Figure 5E). The interactions made by the flipped adenine are similar to that made with AdoMet-bound M. *Taq*I. The adenine stacks against Y1021 and makes an edge-to-surface T-interaction with the aromatic F1133. The adenine-N6 is hydrogen bonded to the catalytic N1018. The cavity formed by the base flip is filled by the intercalation of two residues R1119 and R1138 (Figure 5E). The two residues along with the flanking bases at positions +2 and −1 form a continuous stack. Intercalation by two positionally identical residues R1119 and M1137 fills the cavity in the LlaBIII-DNA complex.

The use of two residues to fill the cavity arising from the extrahelical base is unique to Type ISP enzymes. In case of the prototype M. *Taq*I, domain movement compresses the DNA locally (34) and P393 partially intercalates at the position of base flip. In most other enzymes that employ base flipping to catalyse DNA modification, such as other classes of DNA methyltransferases and DNA repair enzymes (40), the cavity is filled in by a single, often hydrophobic residue. However, the cavity formed upon base flipping by Type ISP enzymes appears larger than that seen in case of other enzymes due to the large distortion in the structure (Supplementary Figure S4). Like the DNA bound to LlaBIII (11), the DNA is bent by ∼34° at the target. The width of both the major and minor grooves at the target is increased significantly (Supplementary Figure S4). In contrast, the binding of M. *Taq*I to its target DNA increased the width of only the minor groove by 3 Å (34). Structurally, the additional residue required to plug the DNA is accommodated in a long loop (R1115 to I1141). F1133, discussed above, is also in this loop. In the prototypical M.*Taq*I, this loop is much shorter and lacks the two intercalating residues but has the conserved phenylalanine involved in T-interaction with the adenine.

### Structural elements involved in target recognition

**Figure 6.**
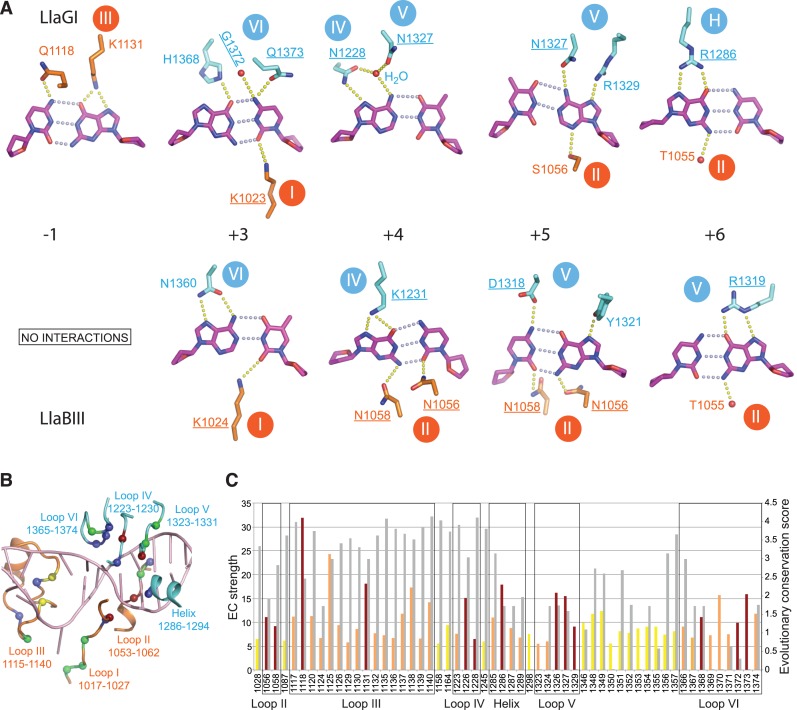
Target recognition. (**A**) Base-specific interactions made by LlaGI (top) and LlaBIII (bottom). Hydrogen bond acceptors and donors within 3.5 Å are connected by yellow dotted lines. Positionally non-equivalent residues that read the equivalent base pairs of LlaGI and LlaBIII targets are underlined. Note that the interactions made by LlaGIΔN with the base pair at +1 are illustrated in Figure 5E. (**B**) The structural elements involved in target recognition (residue numbers based on LlaGI sequence). The spheres represent amino acid residues whose side chains are involved in target recognition in case of LlaGI and LlaBIII. Residues involved in target recognition of LlaGI are coloured blue, residues involved in recognition of LlaBIII target are coloured red and those involved in recognition of both the targets are coloured green. The two intercalating residues that fill the cavity formed upon flipping of the adenine are coloured yellow. (**C**) The top 10% of the residues of the MTase-TRD unit (LlaGI residues 868–1428) with the highest EC strengths. The EC strengths (coloured bars) and the conservation scores (grey bars) for each of these residues are plotted. The EC strengths and the conservation scores were computed using EVcouplings (32,40) and ConSurf (31). The red coloured bars are the EC strengths of residues that interact with a target base; the orange bars are of residues that do not interact with the target but are part of the structural elements involved in target recognition; the yellow bars are of residues in other regions of the MTase-TRD unit. The residue numbering is based on LlaGI sequence. Residues LlaGI-K1023, LlaBIII-K1024 and LlaGI/LlaBIII-T1055, which interact with target bases, are not shown because their score is less than the top 10% (see text).

The structures of LlaGI and LlaBIII bound to their target sites provided a means to understand the structural basis of target recognition by Type ISP enzymes. LlaGI recognizes the 7 bp target sequence CTnAGYG, while LlaBIII recognizes the 6 bp target sequence TnGACC. The overall mode of target binding by the two enzymes is very similar, involving DNA bending and partial unwinding resulting in widening of both the major and minor groove at the target, and flipping of the adenine at +1 position into the MTase catalytic pocket to be methylated. Interestingly, target sequence specificity is achieved by two distinct means—(i) by substitution of a positionally conserved amino acid to ensure correct reading of a target base and (ii) through base-specific interactions by amino acids located at positionally distinct positions (Figure 6A and B). Nonetheless, the LlaGI and LlaBIII residues involved in target recognition are located in one of the following conserved structural elements (Figure 5B, C and 6B): Loop I (1017–1027; residue numbers based on LlaGI sequence), Loop II (1053–1062) and Loop III (1115–1138) in the MTase domain; and Loop IV (1223–1230), Loop V (1324–1331) and Loop VI (1365–1374) in the TRD (Figure 6B). Particular loops are involved in recognition of particular sequence positions. In addition, LlaGI utilizes a helix (H) spanning residues 1286–1294 in the TRD. The equivalent helix in LlaBIII does not play a recognition role. Figure 6A and B reveals the hydrogen-bonded interactions that the residues of LlaGI and LlaBIII use for readout of the different targets.

### Evolutionary constraints at the target-binding region

The above comparison revealed that though not all the amino acids involved in target recognition of the disparate LlaGI and LlaBIII targets are positionally equivalent, all but one amino acid are located within one of the six structurally conserved loops. The conservation scores of the amino acids in the target-binding region derived from the MSA were comparatively lower (Figures 5B, C and 6C). At first glance, this indicated that the structural elements and amino acid positions used by LlaGI and LlaBIII for target recognition are not functionally conserved amongst other Type ISP enzymes. Alternatively, the lower conservation score could be a result of substitutions occurring at these positions to facilitate recognition of different target sequences. If latter were true, then the position, rather than the identity of the amino acid, should be under strict EC, and would co-evolve with one or more positions in the protein. Accordingly, there would also be a correlation between the amino acids at these positions and the target sequence.

Using the program EVcouplings (32), an evolutionary coupling score was calculated for all pairs of residues from 868 to 1428 (LlaGI numbering), which encompasses the target binding MTase domain, core and jaw. Based on the number of pairs a residue was associated with and the corresponding coupling scores, the program calculated strength of EC for that residue, as defined by Hopf *et al*. (2012) (41). It has been shown that the EC strength of a residue is linked to its functional importance, such as binding to ligands (32). Through this calculation, we sought to find if the residues of LlaGI and LlaBIII involved in target recognition were evolutionarily constrained. The top 10% of residue positions, i.e. 56 of 561, with a high EC strength is shown in Figure 6C (for the complete list see Supplementary Data 2). Out of the 17 residues of LlaGI and/or LlaBIII that participate in direct base-specific interactions, 11 were in the top 5% and 3 more within the top 10%. Among the remaining three positions, 1023 (LlaGI numbering) was within top 15%, while 1024 and 1055 were below the top 65% (Supplementary Data 2).

In the top 10%, 28 residues, though not involved in base-specific interactions, were still part of the structural elements involved in target recognition, while 11 of them formed a single β-hairpin loop (1347–1358) in the core subdomain. The two strands of the hairpin are connected by a Ω loop, and make extensive interactions with other structural elements including Loop VI, in addition to directly or indirectly interacting with the phosphate backbone of the DNA. This loop appears structurally conserved in M.*Taq*I, Type I HsdS subunits and TRD of BpuSI. Based on the conservation and its location, we speculate that the loop may impart structural stability to the core.

### Correlation between amino acids and target sequence

**Figure 7.**
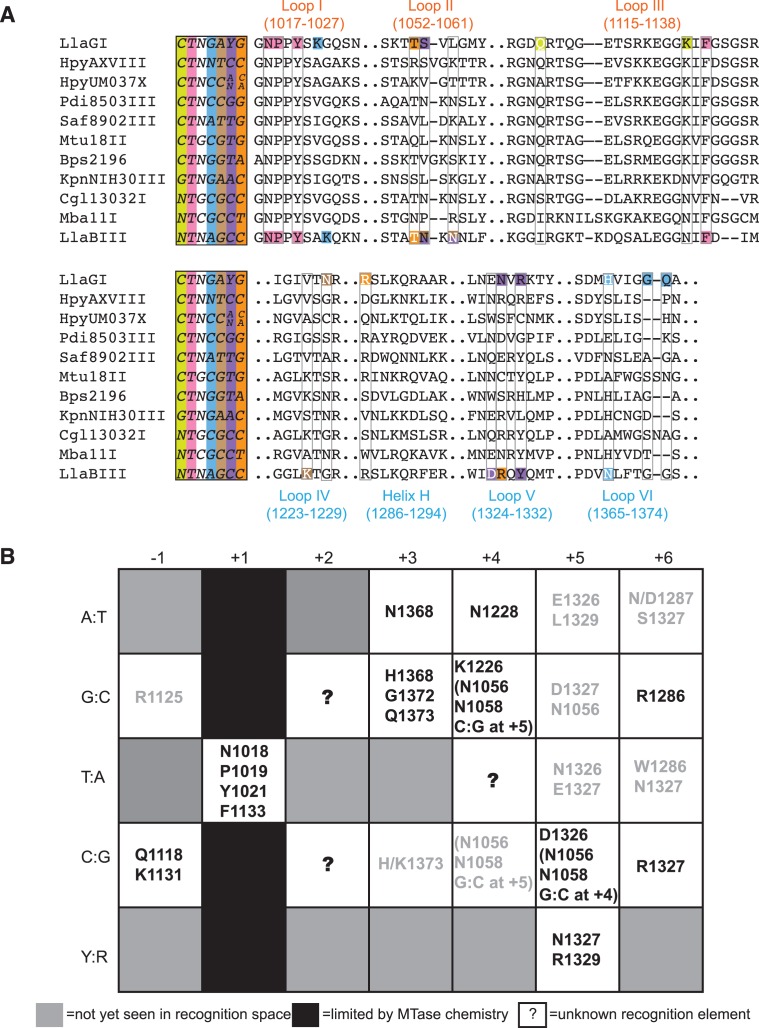
Consensus amino acid code for target recognition. (**A**) An MSA of the amino acid sequences of the seven structural elements involved in target recognition. The sequences are of those Type ISP enzymes whose targets are known. On the left of the alignment are the name of the enzyme and the target sequence. Each position of the target is coloured distinctly. The residues involved in direct interactions with the target bases of LlaGI and LlaBIII are coloured according to the position of the base they interact with. Residues that interact with the top strand are in white font, while those that interact with the bottom strand are in black. The residue numbering is based on LlaGI sequence. (**B**) A tabulation of the amino acids of the Type ISP MTase-TRD involved in recognition of the different base pairs at six of the seven target positions. Amino acids that interact with two neighbouring bases of a base pair step are in parenthesis. The neighbouring base pair, which is part of the step, and its position is also mentioned. Amino acids predicted by us to interact with target bases are in grey font.

Having found that the structural elements involved in target recognition are under strong EC, we proceeded to find if there is a correlation between the target sequence and the amino acid substitutions at positions involved in its recognition. In addition to LlaBIII and LlaGI, the targets of nine other Type ISP enzymes are known (Figure 7A) (20). (In addition, Mtu10134II, MboBCG21III and MtuHN878II have the same amino acid sequence as Mtu18II and recognize the same target sequence.) We compared the amino acid and the target sequences of these enzymes along with LlaGI and LlaBIII to gain insights into this question and investigate if there is a consensus code for target recognition (Figure 7B). The sequence identity amongst the MTase-TRD unit of the 11 enzymes ranged from ∼87% (HpyAXVIII versus HpyUM037X) to ∼33% (HpyUM037X versus LlaBIII). The summary of the analyses is given below and is elaborated in the Supplementary Text.

Position −1: LlaGI recognizes C:G (CTNGAYG). Cytosine and guanine interact with Q1118 and K1131 of Loop III, respectively, via the major groove (Figure 6A). The sequence analysis revealed a very strong correlation between the occurrence of lysine at 1131 and C:G at −1 (Figure 7A). In enzymes that do not recognize −1, including LlaBIII, the glutamine is substituted by a small- or medium-sized hydrophobic residue and the lysine by asparagine (Figure 7A). We predict that a G:C at this position could be read by arginine at 1125 (see Supplementary Text for details).

Position +1: By convention, +1 represents the location of the adenine that is methylated by the MTase domain. Consequently, all the enzymes recognize a T:A at this position (for example, LlaGI-CTNGAYG and LlaBIII-TNAGCC). In the LlaGIΔN and LlaBIII structures, specificity of the T:A base pair is ensured by the interactions made by the flipped adenine with the active site residues of the MTase domain located primarily on Loop I, which catalyse the transfer of methyl group (Figure 5E). It is possible that enzymes which modify cytosine at this position may be discovered, or even engineered in future.

Position +2: Among the 11 Type ISP enzymes whose targets are known, only three recognize this position in a sequence-specific manner. In both LlaGI and LlaBIII structures, the +2 base pair lies at the mouth of the MTase-TRD clamp. The bases do not make any sequence-specific contacts with the protein, thus making this position sequence non-specific. From the sequence alignment, it was not obvious how the other three enzymes read this position.

Position +3: LlaGI recognizes G:C (CTNGAYG) while LlaBIII recognizes A:T (TNAGCC) at this position. The top strand guanine of the LlaGI target and the adenine at the corresponding position of LlaBIII target are recognized by interactions made by the positionally equivalent residues, H1368 and N1360, respectively. The bottom-strand cytosine of the LlaGI target is recognized by the TRD residues G1372 and Q1373, and the MTase residue K1023. However, the corresponding bottom-strand thymine of the LlaBIII target interacts only with the LlaBIII MTase residue K1024 (Loop I).

Position +4: LlaGI recognizes A:T (CTNGAYG) while LlaBIII recognizes G:C (TNAGCC) at this position. The A:T base pair at the LlaGI target is recognized by the interaction between N1228 with the adenine. A water molecule assigned based on a weak 2F_O_-F_C_ but a strong F_O_-F_C_ electron density bridged interactions between the base and N1228 and N1327 (Figure 6A). In LlaBIII, the guanine at the top strand interacts with K1231 and N1058, and the complementary cytosine interacts with the MTase residue N1056 via the minor groove. An A:T base pair at +4 correlated with asparagine at 1228, while G:C correlated strongly with lysine at 1226 (Figure 7A). We noticed that a GC:GC or CG:CG base pair step at positions +4 and +5 correlated with asparagine at 1056 and 1058.

Position +5: LlaGI recognizes T:A or C:G (CTNGAYG). In the crystal structure, the DNA bound to LlaGI has T:A at this position. The bottom strand adenine interacts with the TRD residues N1327 and R1329, and the MTase residue S1056. At this position, LlaBIII recognizes only C:G (TNAGCC). Specificity for cytosine is achieved through contact with the LlaBIII residue D1318. LlaBIII has asparagine at 1056 and 1058, which interact with the guanine and cytosine via the minor groove, respectively, and LlaBIII-Y1321 interacting with guanine via the major groove. Based on the sequence alignment of 11 Type ISP enzymes whose targets are known, we predict that asparagine at 1326 or glutamate at 1327 could contribute to recognition of T:A; glutamate at 1326 could contribute to recognition of A:T; aspartate at 1327 could read G:C at +5 (see Supplementary Text for details).

Position +6: LlaGI recognizes a G:C (CTNGAYG) while LlaBIII recognizes a C:G (TNAGCC). The top strand guanine of LlaGI target is recognized through a bidentate interaction with R1286 located on helix H and the interaction by the backbone carbonyl of the MTase residue T1055 (Figure 6A). At the corresponding position of LlaBIII target, the bottom strand guanine is recognized by bidentate hydrogen bonds with LlaBIII-R1319 in Loop V (Figure 6A). We found a strong correlation between G:C at +6 and arginine at 1286, and C:G and arginine at 1327 (Figure 7A). Based on our analyses we predict that recognition of A:T at +6 could involve aspartate at 1287 and serine at 1327; T:A at +6 by tryptophan at 1286 and asparagine at 1327 (see Supplementary Text for details).

## DISCUSSION

The structure determination and analysis of LlaGI along with the structure of LlaBIII and amino acid sequences of other Type ISP enzymes, described above, provided us with valuable insights into the recognition of target sequence. It has been a long-standing endeavour to rationally design restriction enzymes with new specificities. Single-polypeptide restriction enzymes with fused nuclease and methyltransferase are good candidates for engineering new specificities, as change in specificity alters simultaneously the targets of both the nuclease and methyltransferase (9). Perplexingly, irrespective of the enzyme system, strategies such as rational replacement of amino acids contacting the bases have only been partially successful and required multiple attempts to find active enzymes (8–10).

The comparison of target recognition by LlaGI and LlaBIII provided an alternative to understanding this issue, as we could compare two very closely related RM enzymes that recognize different target sequences. Upon comparison, we found that most of the structural elements employed by the two enzymes for target recognition were identical. However, changes in target sequence did not always involve appropriate substitution of amino acids at the same positions. Corresponding amino acid changes also occurred at positionally distinct locations, with possible compensatory mutations in regions beyond to maintain the integrity of the structural elements. The non-equivalent positions were either on the same or one of the other target-binding structural elements. In addition, indirect interactions, such as the water bridge between LlaGI-N1228 and adenine at +4, appeared to contribute to specificity. These results emphasize the complexities of target recognition and that it may not suffice to change the identity of amino acids contacting the target bases to generate new specificity. Additional substitution of amino acids at new positions may be required to read the new set of bases.

Analyses of the MSA of a large set of Type ISP enzyme sequences revealed that the amino acid positions that are involved in target recognition are not strongly conserved. The lower conservation possibly reflects the change in amino acid identity complementary to the target sequence. Among the target binding structural elements, those belonging to the MTase domain were found to be strikingly more conserved than those from TRD (Figures 5B, C and 6C). The MTase residues play crucial role in specificity of −1 via the major grove and the flipped adenine of +1. The contacts made by the relatively less conserved TRD residues are the primary sequence readers of the base pairs downstream from +3 onwards, while interactions by the MTase domain via the minor groove provide additional stability. The differential conservation score suggests positions −1 and +1 to be least plastic. This observation highlights the constraints in engineering the DNA binding elements that are part of or in the near vicinity of catalytic regions, in this case the MTase active site. Despite relatively lower conservation, we found that the residues of the structural elements recognizing the target, including those that interact with the bases, were under strong EC. Our analysis using the program EVcouplings identified the target recognition region as the primary functional ‘hotspot’ in the MTase-TRD unit, suggesting this to be a powerful tool in identifying specificity-determining regions in DNA binding proteins.

Based on our analysis, we have arrived at a consensus code for target recognition by Type ISP enzymes (Figure 7B). This, we believe, will help in engineering enzymes with new specificity. We recognize that the engineering of new specificity will be limited by the catalytic requirement of a T:A base pair at +1. It will be interesting to find if the binding pocket of the adenine methyltransferase can be engineered to accommodate and recognize other bases. The translocation-active nuclease deletion mutant of LlaGI reported here highlights the modular nature of these enzymes. The modularity of the Type ISP enzymes is an additional advantage in engineering sequence-specific methyltransferases, DNA translocases or nickases. These would be useful biotechnological reagents where N6-adenine methylation is to be carried out; sequence-dependent remodelling of nucleoprotein complexes is required; or sequence-specific nicking of dsDNA is to be performed. The conserved architecture of the MTase-TRD unit of the γ-class of N6-adenine methyltransferase, Type I, Type IIG and L enzymes suggests that the insights on target recognition by Type ISP enzymes may find use in engineering specificity in the above enzyme types too.

## ACCESSION NUMBER

The atomic coordinates and structure factors have been deposited with accession code 5FFJ.

## Supplementary Material

SUPPLEMENTARY DATA
