# Supplementary material for: Structural insights into DNA sequence recognition by Type ISP restriction-modification enzymes
Source: Nucleic Acids Res. 2016 Mar 14;44(9):4396–408. doi: 10.1093/nar/gkw154 (PMC4872093; doi:10.1093/nar/gkw154)
Supplement: SUPPLEMENTARY DATA [file supp_44_9_4396__index.html]

Structural insights into DNA sequence recognition by Type ISP restriction-modification enzymes — Structural insights into DNA sequence recognition by Type ISP restriction-modification enzymes — SUPPLEMENTARY DATA 

# Structural insights into DNA sequence recognition by Type ISP restriction-modification enzymes

## SUPPLEMENTARY DATA

- SUPPLEMENTARY DATA
- SUPPLEMENTARY DATA
- SUPPLEMENTARY DATA
- SUPPLEMENTARY DATA
