# Supplementary material for: Structural insights into DNA sequence recognition by Type ISP restriction-modification enzymes
Source: Nucleic Acids Res. 2016 Mar 14;44(9):4396–408. doi: 10.1093/nar/gkw154 (PMC4872093; doi:10.1093/nar/gkw154)
Supplement: SUPPLEMENTARY DATA [file supp_gkw154_nar-03673-z-2015-File004.pdf]

Supplementary Information

Structural insights into DNA sequence recognition by Type ISP restriction-modification enzymes

Manasi Kulkarni[1,3], Neha Nirwan[1,3], Kara van Aelst[2], Mark D. Szczelkun[2], Kayarat Saikrishnan [1*]

[1]Division of Biology, Indian Institute of Science Education and Research, Pune, 411008, India

[2]DNA-Protein Interactions Unit, School of Biochemistry, Medical Sciences Building, University of Bristol, Bristol BS8 1TD, UK

Supplementary Text

Interactions between Type ISP enzyme and target

Position -1: LlaGI recognises C:G (CTNGAYG). The cytosine and guanine interacts with Q1118 and K1131 of Loop III, respectively, via the major groove (Figure 6A). The -1 position is recognised as part of the target by eight of the eleven enzymes (Figure 7A). Strikingly, Q1118 and K1131 occur together in all these enzymes, except HpyUM037X in which the glutamine is replaced by alanine. In the three enzymes that did not recognise -1, including LlaBIII, the glutamine is substituted by a small or medium sized hydrophobic residue and the lysine by asparagine (Figure 6A). These substitutions possibly make -1 non-specific. In the MSA of 552 Type ISP enzymes, we found a strong correlation between Q1118 and K1131, and similarly between hydrophobic residue at 1118 and asparagine at 1131. The evolutionary coupling score obtained from EVcouplings was fourth highest for this pair of positions (Supplementary Data 2). There was a small set of enzymes, including HpyUM037X, in which lysine was observed at 1131, even if glutamine was substituted at 1118, suggesting that the bidentate interaction between lysine and guanine may be sufficient to recognise C:G at -1. Almost ~57% of the sequences in the MSA had lysine at the equivalent 1131 position. Therefore, we predict that these enzymes would require C:G at -1 for target recognition.

KpnNIH30III recognises G:C at -1 and the enzyme has an alanine and asparagine at 1118 and 1131. A careful examination of an atomic model of KpnNIH30III generated using LlaGI as template, indicated that an arginine in KpnNIH30III at the position equivalent to LlaGI-S1125 (Loop III) was proximal to the guanine

and could potentially form a bidentate interaction with the base. Interestingly, S1125 had the second highest EC strength (Figure 6C). In LlaGI, the hydroxyl group of S1125 hydrogen bonded with the side chain carbonyl of Q1118 and the main chain carbonyl hydrogen bonded with the side chain amino group of the same residue thus positioning the glutamine for base specific interaction with cytosine at -1. We also noted a correlation between arginine at 1125 and alanine at 1118, possibly a requirement to facilitate the positioning of the bulky side chain. The 1118:1125 pair also had the second highest evolutionary coupling score.

Position +1: The enzymes recognise T:A at this position (LlaGI-CTNGAYG and LlaBIII-TNAGCC). In LlaGI and LlaBIII, specificity of the T:A base pair is ensured by the interactions made by the flipped adenine with the active site residues of the MTase domain located primarily on Loop I. Specificity is ensured by catalytic N1018 that makes a hydrogen bond with adenine, along with F1133, which makes an edge-to-edge T-shaped interaction with the flipped base and sterically prevents guanine from entering the pocket. In addition, the main chain carbonyl group of N1018 and P1019 make hydrogen bonds with the N6 of adenine, while the main chain amino group of Y1021 hydrogen bonds with N7. These residues are conserved in all the 552 sequences. These residues, which are part of the MTase active site, also catalyse the transfer of methyl group. By definition, completely conserved residues fail to provide information on evolutionary pair coupling (33). Hence, despite their functional importance, these residues did not appear in the list of evolutionarily constrained residues.

Position +2: Among the eleven Type ISP enzymes whose targets are known, only three recognise this position in a sequence specific manner. This position lies at the mouth of the MTase-TRD clamp. In both LlaGI and LlaBIII structures, the bases at +2 does not make any sequence specific contacts with the protein, thus making this position sequence non-specific. From the sequence alignment, it was not obvious how the other three enzymes read this position. However, we noted that Loop III, which is in the vicinity of this position, was longer in Mba11I by two residues in comparison to most other enzymes, and could play a role in recognition (Figure 7A). Similarly, Cgl13032I and Mtu18II had a longer Loop VI (Figure 7A).

Position +3: LlaGI recognises G:C (CTNGAYG) while LlaBIII recognises A:T (TNAGCC) at this position. The top strand guanine of the LlaGI target and the adenine at the corresponding position of LlaBIII target are recognised by interactions made by the positionally-equivalent residues, N1360 and H1368, respectively. The histidine forms a hydrogen bond with the guanine, while the asparagine forms bidentate hydrogen bonds with the adenine. The bottom-strand cytosine of the LlaGI target is recognised via the major groove by the interactions made by the side chain of the TRD residue Q1373 (Loop VI) and main chain carbonyl oxygen of G1372 (Loop VI), and via the minor groove by the side chain of the MTase residue K1023 (Loop I). However, the corresponding bottom-strand thymine of the LlaBIII target interacts only with the LlaBIII MTase residue K1024 (Loop I).

As evident from Figure 7A, whenever G:C or A:T base pair occurrs in the target at +3, there is the guanine-specific histidine or adenine-specific asparagine at position 1368. The lysine and glutamine at 1023 and 1373 of LlaGI that contacts the cytosine in G:C, and the lysine in LlaBIII at 1024 that contacts the bottom strand of A:T at -3 are poorly conserved (Figure 6A and 7A). Accordingly, 1023 and 1024 had relatively poor EC strength (Figure 6C), suggesting that the lysine-mediated interactions via the minor groove are not essential for base recognition. In contrast, a high EC strength at position 1373 indicated its importance in recognition of other bases. For example, in the enzymes HpyUM037Z and Pdi8503III a histidine and a lysine occurs at 1373, respectively, which could be located proximal to the guanine of C:G at +3 of their targets, and potentially facilitating base recognition. The target of HpyAXVIII can have any base pair at this position. Correspondingly, we find substitution to amino acids with short side chains (serine) and no side chains (proline) at 1368 and 1373, respectively.

Position +4: LlaGI recognises A:T (CTNGAYG) while LlaBIII recognises G:C (TNAGCC) at this position. The A:T base pair at the LlaGI target is recognised by the interaction between N1228 of Loop III with the adenine, while the complementary thymine makes no hydrogen bond with the protein. We found a weak $2F_O$-F_C but a strong F_O-F_C electron density neigbouring N1228 and adenine. We interpreted this as water, which bridges additional interactions between the base and N1228 and N1327 (Figure 6A). The side chain of M1367 is within van der Waal's distance of the complementary thymine. At the corresponding position of LlaBIII target, the guanine at the top strand interacts with K1231 of

Loop III and N1058 of Loop II, and the complementary cytosine interacts with the MTase residue N1056 of Loop II via the minor groove. An A:T base pair at +4 correlated with asparagine at 1228, while G:C correlated strongly with lysine at 1226 (Figure 7A). We found that a GC:GC or CG:CG base pairs step at positions +4 and +5 correlated with asparagine at 1056 and 1058. The carboxamide group of the two asparagines can hydrogen bond with the two consecutive base pairs simultaneously via the minor groove, as seen in the structure of LlaBIII (Figure 6A).

It was not obvious how T:A or C:G base pair would be recognised. But we found the occurrence of polar or positively charged residues at 1056 and 1058, which could specifically recognise bases through inter-base pair interactions.

Position +5: LlaGI recognises T:A or C:G (CTNGAYG). In the crystal structure, the DNA bound to LlaGI has T:A at this position. The bottom strand adenine interacts with the TRD residues N1327 and R1329 of Loop V via the major groove, and the MTase residue S1056 of Loop II via the minor groove. The hydrogen bonds formed by N1327 and R1329 with the adenine resembles that of a bidentate interaction made by an asparagine. However, unlike the adenine-specific asparagine bidentate interaction, replacement of the adenine with guanine could also lead to favourable interactions with N1327 and R1329 on flipping of the carboxamide group of the asparagine. We found that the carboxamide group of N1327 did not interact with any other neighbouring atoms, implying that the flip could occur. In addition, the serine at 1056 could interact with either of the purines.

At this position, LlaBIII recognises C:G (TNAGCC). Specificity for cytosine is achieved through contact with the LlaBIII residues N1058 and D1318 (positionally-equivalent to LlaGI-E1326). The interaction formed by LlaGI-R1329 with the N7 of purine is made by the positionally-equivalent residue LlaBIII-Y1321. LlaBIII has asparagine at 1056, which interacts with the bottom-strand guanine via the minor groove. HpyAXVIII, Cgl13032I and Mba11I also have C:G at +5, and possibly utilise the potential interaction made by glutamate, glutamine or asparagine at 1326, respectively, to read cytosine. Though LlaGI also has glutamate at 1326, it is not competent to interact with cytosine as its side chain is tethered away from the base by hydrogen bonds with S1285 and S1287. The coupling of 1326 with 1285 and 1287 appeared as a high score in the EVcouplings calculation (Supplementary Data 2). LlaBIII-R1319, which is positionally-equivalent to LlaGI-N1327 (involved in recognition of +5 of LlaGI target; see above), is positioned to interact with +6 rather than +5 of the LlaBIII target (see below).

Saf8902III, Mtu18II and Bps2196ORF3045 recognise T:A at this position. Saf8902III has glutamate at 1327, which could hydrogen bond with the bottom strand adenine N6, and thus may contribute to specificity. Mtu18II has asparagine at 1326, which may facilitate the recognition of top strand thymine, while Bps2196ORF3045 has tryptophan that we predict could ensure selection of thymine through hydrophobic interaction with C5 methyl group. In contrast, HpyUM037X, which recognises A:T or any base pair at +5 in combination with C:G or A:T at +6, respectively, also has a tryptophan at 1326, which makes the

role of the residue in recognition unclear. In this enzyme, positions 1327 and 1329 have the short side chained serine and cysteine, respectively, which could facilitate accommodation of any base pair.

KpnNIH30III recognises an A:T base pair at this position. As in case of HpyUM037X, this A:T is in combination with C:G at +6. In this enzyme there is a glutamate at 1326, which could interact specifically with the adenine N6, while leucine at 1329 could make hydrophobic contact with the bottom strand thymine's C5 methyl group. Though, both HpyUM037X and KpnNIG30III could recognise A:T at +5, from the sequence analysis the basis of recognition is not apparent. The enzyme Pdi8503III recognises G:C at this position. In this case, we predict that the specificity could be achieved by interaction between aspartate at 1327 with the cytosine, and asparagine at 1056 with the guanine.

Position +6: LlaGI recognises a G:C (CTNGAYG) while LlaBIII recognises a C:G (TNAGCC). At +6 position of LlaGI target, the top strand guanine is recognised through a bidentate interaction with the TRD residue R1286 located on helix H and the interaction by the backbone carbonyl of the MTase residue T1055 via the minor groove. At the corresponding position of LlaBIII target, the bottom strand guanine is recognised by bidentate hydrogen bonds with LlaBIII-R1319 (Loop V; positionally-equivalent to LlaGI-N1327) (Figure 6A and 6B). It is interesting to note that LlaGI-R1286 is highly conserved and is also found in LlaBIII (LlaBIII-R1278) where it does not play a role in recognition. In LlaBIII, the guanidinium group of LlaBIII-R1278 is away from the target and towards the solvent, thus making way for LlaBIII-R1319 to contact the guanine.

We found a strong correlation between G:C at +6 and arginine at 1286, and C:G and arginine at 1327 (Figure 7A). In REBASE, HpyUM037X is assigned two targets – CTNCCAC and CTNCCNA. The enzyme has glutamine and serine at the equivalent positions of 1286 and 1327. The absence of arginine at 1327 positions failed to explain recognition of C:G at +6 by HpyUM037X using the above logic. HpyUM037X and Bps2196ORF3045 recognise an A:T at +6. It was not obvious from the sequence analysis how these enzymes specifically recognised the base pair. We note that both HpyUM037X and Bps2196ORF3045 have serine at 1327. A model of the two enzymes generated using LlaGI as template indicated that asparagine or aspartate at 1287 and serine at 1327 were in the vicinity of the base pair, which could together specifically read A:T. The two residues had a high EC strength (Figure 6C) and coupling score (Supplementary Data 2).

Mba11I target has T:A at +6. The enzyme has tryptophan and asparagine at positions equivalent to 1286 and 1327. We predict that the amino group of asparagine side chain could hydrogen bond with the O6 of guanine at +5, while the carbonyl group could hydrogen bond with the N6 of adenine at +6; the hydrophobic tryptophan through contact with the complementary thymine C5 methyl group could contribute to specificity.

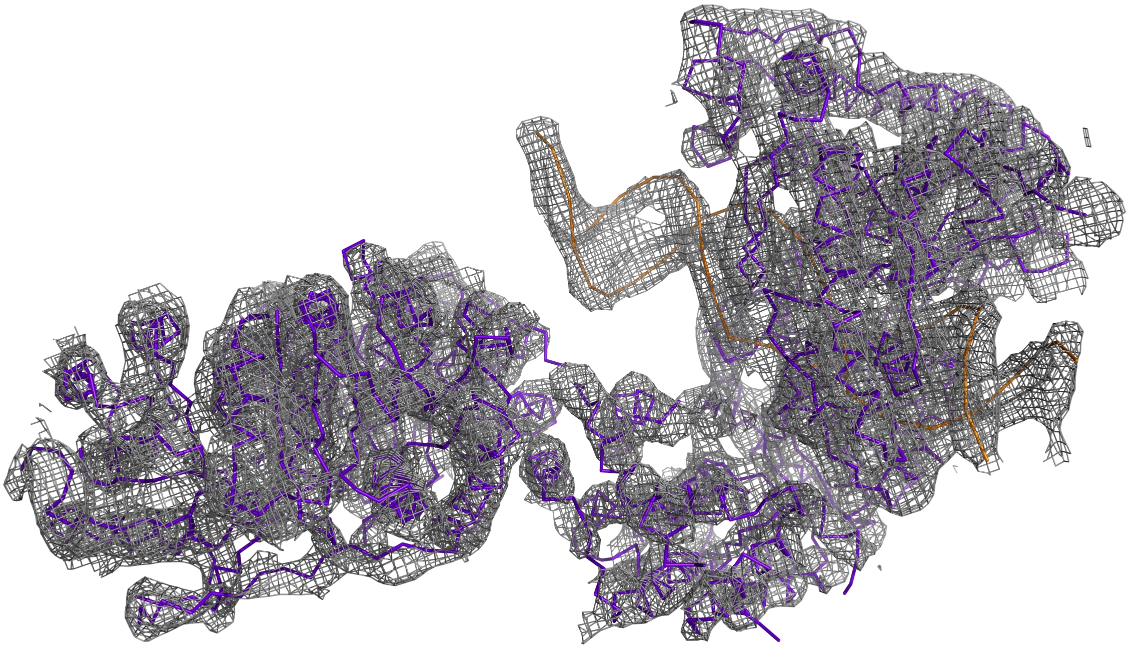

Supplementary Figure 1: A $2F_O$-F_C electron density map (grey) of LlaGI (purple; chain A) bound to DNA (orange, chain E and F) at a contour level of 1 σ.

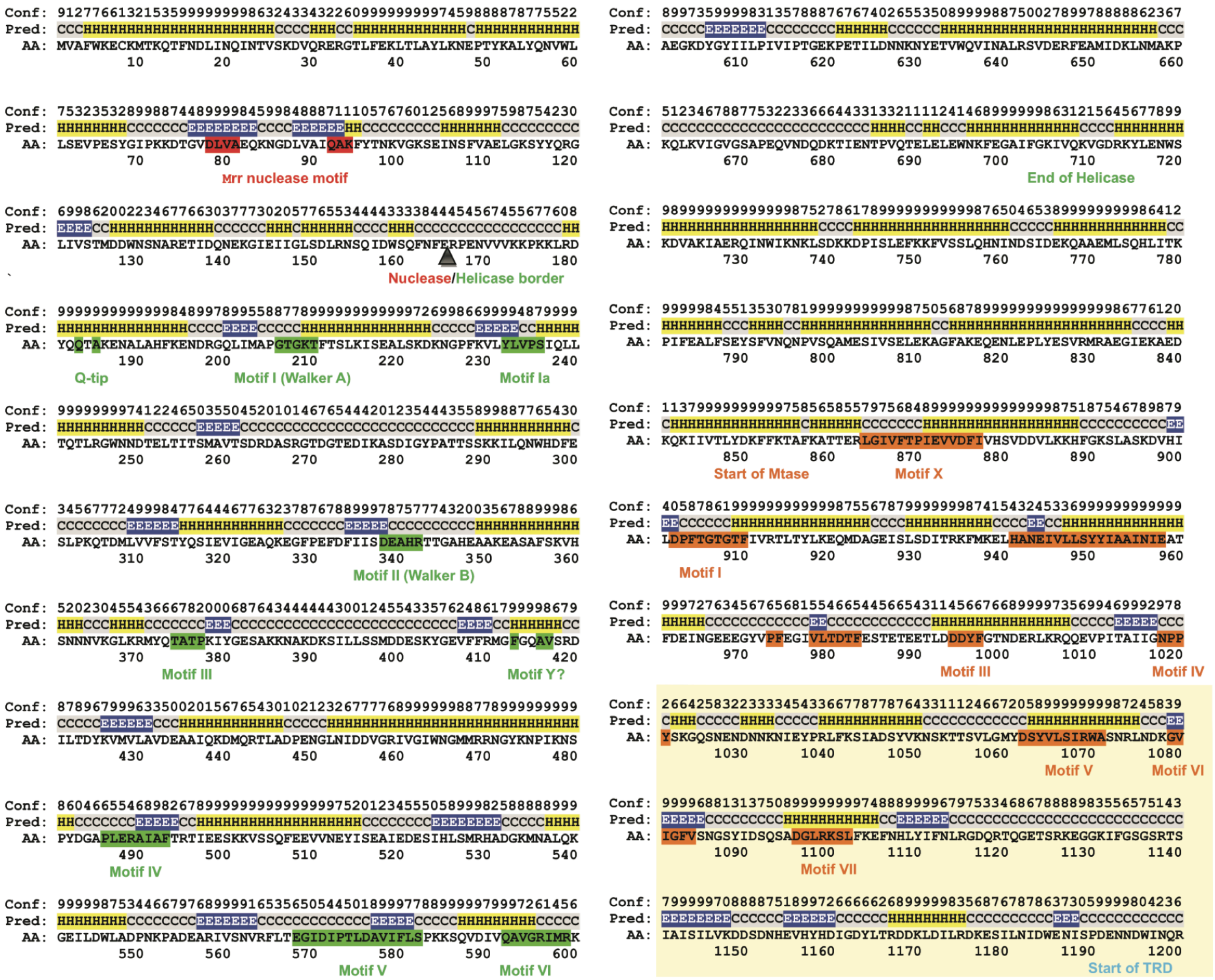

Supplementary Figure 2: Secondary structure prediction of LlaGI to determine domain boundaries. The amino acid sequence of LlaGI was submitted to the PSIPRED server (34). Yellow (H) = alpha helix; Blue (E) = beta strand; Grey (C) = random coil. Numbers above each line are the confidence levels (0 = low, 9 = high). The arrowhead indicates the truncated position of LlaGIΔN. Nuclease, helicase and methyltransferase motifs are indicated. The yellow boxes indicate the regions of the LlaGI sequence that show most divergence from LlaBIII (see Figure 2A).

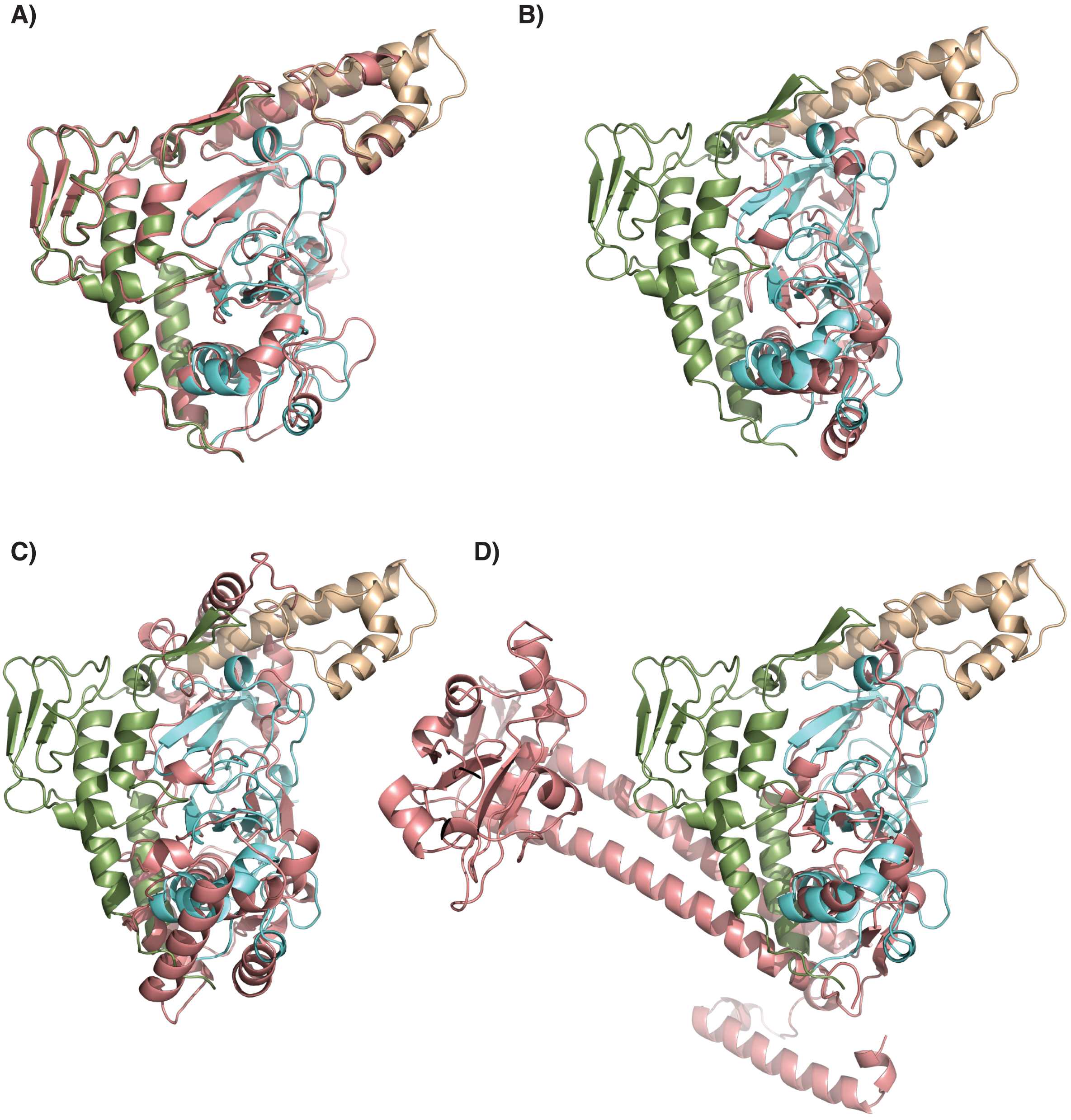

Supplementary Figure 3: Superposition of the C^{α}-trace of A) TRD of LlaBIII (salmon), B) TRD of M.*TaqI* (salmon; PDB code:1G38) (35), C) TRD of BpuSI (salmon; PDB code:3SIS) (37), D) TRD of a Type I enzyme (salmon; PDB code:1YF2) (36) superposed on TRD of LlaGI, which is coloured subdomain-wise as in Figure 3A. The structures were superposed on the TRDa subdomain of LlaGI using COOT (28).

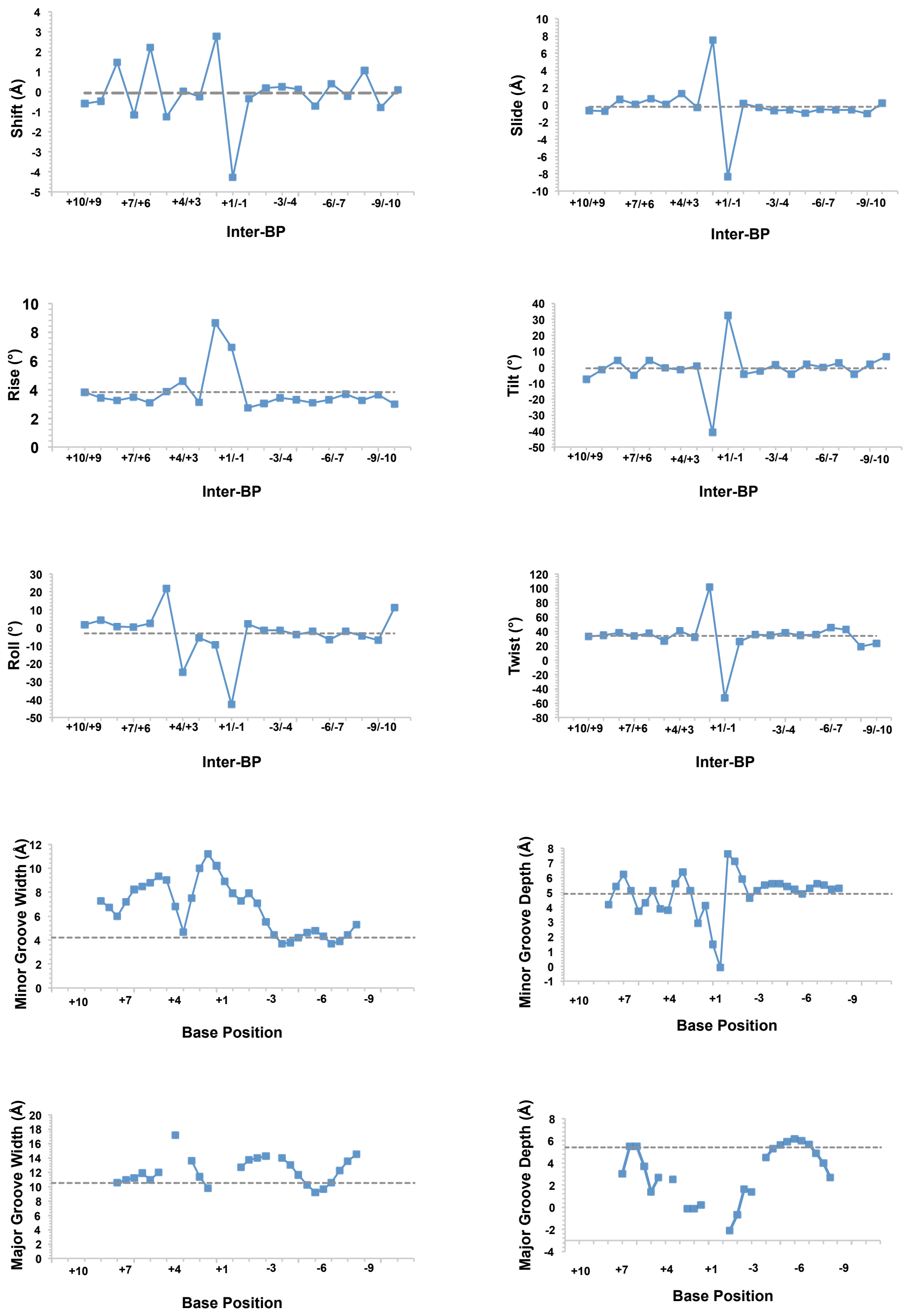

Supplementary Figure 4: Geometrical parameters of the DNA bound to LlaGIΔN. The parameters were calculated for the DNA chains C and D using the program Curves+ (43). Curves+ calculated DNA bending angle to be 34°. There is a significant increase in the width of both the major and minor grooves. The major groove width and depth at some of the regions could not be calculated due to large deformation. The grey lines indicate the average parameter values for a B-DNA obtained by Curves+ using the coordinates 1BDN (43).

43. Lavery, R., Moakher, M., Maddocks, J. H., Petkeviciute, D. & Zakrzewska, K. (2009). Conformational analysis of nucleic acids revisited: Curves+. *Nucleic Acids Res*. **37**, 5917-5929.
