# Supplementary material for: Structural insights into DNA sequence recognition by Type ISP restriction-modification enzymes
Source: Nucleic Acids Res. 2016 Mar 14;44(9):4396–408. doi: 10.1093/nar/gkw154 (PMC4872093; doi:10.1093/nar/gkw154)
Supplement: SUPPLEMENTARY DATA [file supp_gkw154_nar-03673-z-2015-File005.pdf]

CLUSTAL O(1.2.1) multiple sequence alignment

```
gi|490190857|ref|WP_004089451.1|hypotheticalprotein[Helicobacterbilis]_gi|476630638|gb|EMZ37196.1|hypotheticalproteinC826_02193      ------------------------------------------------------------
gi|488896133|ref|WP_002807242.1|MULTISPECIES:restrictionendonucleaseEco57I[Campylobacter]_gi|380541096|gb|EIA65377.1|hypothetic      ------------------------------------------------------------
gi|746602240|ref|WP_039628365.1|restrictionendonucleaseEco57I[Campylobacterlari]_gi|744819395|gb|AJD03391.1|putativehelicase[Ca      ------------------------------------------------------------
gi|727317135|ref|WP_033772558.1|restrictionendonucleaseEco57I[Helicobacterpylori]/11622                                              ------------------------------------------------------------
gi|727103490|ref|WP_033618954.1|restrictionendonucleaseEco57I[Helicobacterpylori]/11616                                              ------------------------------------------------------------
gi|727340205|ref|WP_033794408.1|restrictionendonucleaseEco57I[Helicobacterpylori]/11609                                              ------------------------------------------------------------
gi|727292407|ref|WP_033749975.1|restrictionendonucleaseEco57I[Helicobacterpylori]/11611                                              ------------------------------------------------------------
gi|727289795|ref|WP_033747591.1|restrictionendonucleaseEco57I[Helicobacterpylori]/11612                                              ------------------------------------------------------------
gi|727319676|ref|WP_033775047.1|restrictionendonucleaseEco57I[Helicobacterpylori]/11612                                              ------------------------------------------------------------
gi|447212910|ref|WP_001290166.1|restrictionendonucleaseEco57I[Helicobacterpylori]_gi|308063540|gb|ADO05427.1|hypotheticalprotei      ------------------------------------------------------------
gi|447212901|ref|WP_001290157.1|restrictionendonucleaseEco57I[Helicobacterpylori]_gi|384557329|gb|AFH97797.1|hypotheticalprotei      ------------------------------------------------------------
gi|515849671|ref|WP_017280424.1|hypotheticalprotein[Helicobacterpylori]/11615                                                        ------------------------------------------------------------
gi|447212900|ref|WP_001290156.1|restrictionendonucleaseEco57I[Helicobacterpylori]_gi|317178970|dbj|BAJ56758.1|TypeIIGrestrictio      ------------------------------------------------------------
gi|447079018|ref|WP_001156274.1|hypotheticalprotein[Helicobacterpylori]/11614                                                        ------------------------------------------------------------
gi|447212800|ref|WP_001290056.1|hypotheticalprotein[Helicobacterpylori]_gi|532153248|gb|EQL67316.1|restrictionendonucleaseEco57      ------------------------------------------------------------
gi|505457237|ref|WP_015644339.1|hypotheticalprotein[Helicobacterpylori]_gi|499061160|gb|AGL70074.1|restrictionendonucleaseEco57      ------------------------------------------------------------
HPYUM037X/11591                                                                                                                       ------------------------------------------------------------
gi|920026297|ref|WP_052937176.1|restrictionendonuclease[Helicobacterpylori]_gi|912528678|gb|KNX48238.1|restrictionendonucleaseE      ------------------------------------------------------------
gi|915539050|ref|WP_050841327.1|restrictionendonuclease[Helicobacterpylori]_gi|908890887|gb|KNE06183.1|restrictionendonucleaseE      ------------------------------------------------------------
gi|727101397|ref|WP_033616957.1|restrictionendonucleaseEco57I[Helicobacterpylori]/11592                                              ------------------------------------------------------------
gi|562814875|emb|CDK13002.1|TypeISPRMsystem[Helicobacterpylori26695]/11588                                                           ------------------------------------------------------------
HPYAXVIII/11588                                                                                                                       ------------------------------------------------------------
gi|447079029|ref|WP_001156285.1|restrictionendonucleaseEco57I[Helicobacterpylori]_gi|393085956|gb|EJB86635.1|hypotheticalprotei      ------------------------------------------------------------
gi|489105953|ref|WP_003015814.1|restrictionendonuclease[Helicobacterpylori]_gi|477503388|gb|ENH59950.1|restrictionendonucleasef      ------------------------------------------------------------
gi|447079006|ref|WP_001156262.1|restrictionendonucleaseEco57I[Helicobacterpylori]_gi|393067470|gb|EJB68280.1|hypotheticalprotei      ------------------------------------------------------------
gi|727314589|ref|WP_033770298.1|restrictionendonucleaseEco57I[Helicobacterpylori]/11586                                              ------------------------------------------------------------
gi|447079019|ref|WP_001156275.1|restrictionendonucleaseEco57I[Helicobacterpylori]_gi|308065028|gb|ADO06920.1|hypotheticalprotei      ------------------------------------------------------------
gi|447079028|ref|WP_001156284.1|restrictionendonucleaseEco57I[Helicobacterpylori]_gi|393076030|gb|EJB76784.1|hypotheticalprotei      ------------------------------------------------------------
gi|393034009|gb|EJB35070.1|hypotheticalproteinHPNQ4053_0631[HelicobacterpyloriNQ4053]/11514                                          ------------------------------------------------------------
gi|447079004|ref|WP_001156260.1|restrictionendonucleaseEco57I[Helicobacterpylori]_gi|384552418|gb|AFI07366.1|hypotheticalprotei      ------------------------------------------------------------
gi|746011306|ref|WP_039088215.1|restrictionendonucleaseEco57I[Helicobacterpylori]_gi|733488720|gb|KHL85979.1|restrictionendonuc      ------------------------------------------------------------
gi|531081162|gb|EQD92166.1|restrictionendonucleaseEco57I[HelicobacterpyloriPZ5080]/11509                                             ------------------------------------------------------------
gi|544760796|ref|WP_021187395.1|restrictionendonucleaseEco57I[Helicobacterpylori]_gi|531086508|gb|EQD96745.1|restrictionendonuc      ------------------------------------------------------------
gi|447079012|ref|WP_001156268.1|restrictionendonucleaseEco57I[Helicobacterpylori]_gi|393028199|gb|EJB29286.1|hypotheticalprotei      ------------------------------------------------------------
gi|746008458|ref|WP_039085555.1|restrictionendonucleaseEco57I[Helicobacterpylori]_gi|733480358|gb|KHL78026.1|restrictionendonuc      ------------------------------------------------------------
gi|447079011|ref|WP_001156267.1|restrictionendonucleaseEco57I[Helicobacterpylori]_gi|393038534|gb|EJB39568.1|hypotheticalprotei      ------------------------------------------------------------
gi|393037163|gb|EJB38199.1|hypotheticalproteinHPNQ4161_0422[HelicobacterpyloriNQ4161]/11556                                          ------------------------------------------------------------
gi|447079007|ref|WP_001156263.1|restrictionendonucleaseEco57I[Helicobacterpylori]_gi|393051817|gb|EJB52768.1|hypotheticalprotei      ------------------------------------------------------------
gi|487867094|ref|WP_001940560.1|helicase[Helicobacterpylori]_gi|459505657|gb|EMG98418.1|helicaseprotein[HelicobacterpyloriGAM21      ------------------------------------------------------------
gi|487872094|ref|WP_001945560.1|helicase[Helicobacterpylori]_gi|459512104|gb|EMH04523.1|helicaseprotein[HelicobacterpyloriGAM24      ------------------------------------------------------------
gi|487880228|ref|WP_001953694.1|helicase[Helicobacterpylori]_gi|459529131|gb|EMH20734.1|helicaseprotein[HelicobacterpyloriGAM26      ------------------------------------------------------------
gi|487882593|ref|WP_001956059.1|helicase[Helicobacterpylori]_gi|459533462|gb|EMH24859.1|helicaseprotein[HelicobacterpyloriGAM26      ------------------------------------------------------------
gi|487845430|ref|WP_001918896.1|helicase[Helicobacterpylori]_gi|410713871|gb|EKQ71363.1|helicaseprotein[HelicobacterpyloriGAM10      ------------------------------------------------------------
gi|447079005|ref|WP_001156261.1|restrictionendonucleaseEco57I[Helicobacterpylori]_gi|393069830|gb|EJB70625.1|hypotheticalprotei      ------------------------------------------------------------
gi|459506808|gb|EMG99528.1|helicaseprotein[HelicobacterpyloriGAM121Aii]/11545                                                        ------------------------------------------------------------
gi|487825283|ref|WP_001898749.1|restrictionendonucleaseEco57I[Helicobacterpylori]_gi|393107526|gb|EJC08068.1|hypotheticalprotei      ------------------------------------------------------------
gi|447079008|ref|WP_001156264.1|restrictionendonucleaseEco57I[Helicobacterpylori]_gi|393058636|gb|EJB59524.1|hypotheticalprotei      ------------------------------------------------------------
gi|447079015|ref|WP_001156271.1|restrictionendonucleaseEco57I[Helicobacterpylori]_gi|307637357|gb|ADN79807.1|hypotheticalprotei      ------------------------------------------------------------
gi|487884861|ref|WP_001958327.1|helicase[Helicobacterpylori]_gi|459535535|gb|EMH26834.1|helicaseprotein[HelicobacterpyloriGAM26      ------------------------------------------------------------
gi|487886365|ref|WP_001959831.1|helicase[Helicobacterpylori]_gi|459538727|gb|EMH29861.1|helicaseprotein[HelicobacterpyloriGAM27      ------------------------------------------------------------
gi|545301637|ref|WP_021581412.1|helicase[Helicobacterpylori]_gi|535931889|gb|ERA61745.1|helicaseprotein[HelicobacterpyloriGAM11      ------------------------------------------------------------
gi|447079010|ref|WP_001156266.1|restrictionendonucleaseEco57I[Helicobacterpylori]_gi|393095477|gb|EJB96081.1|hypotheticalprotei      ------------------------------------------------------------
gi|447079009|ref|WP_001156265.1|restrictionendonucleaseEco57I[Helicobacterpylori]_gi|393056294|gb|EJB57206.1|hypotheticalprotei      ------------------------------------------------------------
gi|471386985|gb|EMR58906.1|restrictionendonucleasefamilyprotein[HelicobacterpyloriHpH1]/11504                                        ------------------------------------------------------------
gi|487875679|ref|WP_001949145.1|helicase[Helicobacterpylori]_gi|459516928|gb|EMH09091.1|helicaseprotein[HelicobacterpyloriGAM25      ------------------------------------------------------------
gi|496130066|ref|WP_008854573.1|DNAdamageinducibleprotein[Commensalibacterintestini]_gi|353671689|gb|EHD13391.1|helicasedomain/      ------------------------------------------------------------
gi|924898894|ref|WP_053556546.1|damageinducibleprotein[Sphingopyxissp.113P3]_gi|924523996|gb|ALC14732.1|damageinducibleprotei/1      ------------------------------------------------------------
gi|780802950|gb|KJS08562.1|damageinducibleprotein[Hoefleasp.BRH_c9]/11633                                                            ------------------------------------------------------------
gi|944671432|gb|KQN22886.1|damageinducibleprotein[Sphingomonassp.Leaf32]/11415                                                       ------------------------------------------------------------
gi|944612953|gb|KQM64772.1|damageinducibleprotein[Sphingomonassp.Leaf16]/11726                                                       ------------------------------------------------------------
gi|764353851|ref|WP_044331468.1|damageinducibleprotein[Sphingomonashengshuiensis]_gi|761897338|gb|AJP71707.1|damageinduciblep/1      ------------------------------------------------------------
gi|945874472|gb|KQZ27973.1|damageinducibleprotein[Mesorhizobiumsp.Root552]/11651                                                     ------------------------------------------------------------
gi|759408081|ref|WP_043132403.1|damageinducibleprotein[Paracoccussanguinis]_gi|694217086|gb|KGJ14281.1|damageinducibleprotein/1      ------------------------------------------------------------
gi|654342671|ref|WP_027836116.1|damageinducibleprotein[Maritaleamyrionectae]/11632                                                   ------------------------------------------------------------
```

```
gi|126717687|gb|EBA14409.1|hypotheticalproteinRSK20926_02032[Roseobactersp.SK20926]/11603           ------------------------------------------------------------
gi|765301116|ref|WP_044628780.1|damageinducibleprotein[Halomonasmeridiana]_gi|764089679|gb|KJD19875.1|damageinducibleprotein[/1    ------------------------------------------------------------
gi|518053006|ref|WP_019223214.1|DNAdamageinducibleprotein[Bartonellarattaustraliani]/11654           ------------------------------------------------------------
gi|493637806|ref|WP_006589578.1|DNAdamageinducibleprotein[Bartonellabirtlesii]_gi|395410345|gb|EJF76900.1|hypotheticalproteinM/    ------------------------------------------------------------
gi|501188603|ref|WP_012231622.1|DNAdamageinducibleprotein[Bartonellatribocorum]_gi|161017843|emb|CAK01401.1|helicase/methyltra/    ------------------------------------------------------------
gi|651388616|ref|WP_026500615.1|damageinducibleprotein[Bartonellagrahamii]/11655           ------------------------------------------------------------
gi|501188669|ref|WP_012231687.1|DNAdamageinducibleprotein[Bartonellatribocorum]_gi|161017922|emb|CAK01480.1|helicase/methyltra/    ------------------------------------------------------------
gi|518048832|ref|WP_019219040.1|DNAdamageinducibleprotein[Bartonellaflorencae]/11655           ------------------------------------------------------------
gi|518053617|ref|WP_019223825.1|DNAdamageinducibleprotein[Bartonellarattaustraliani]/11607           ------------------------------------------------------------
gi|506336449|ref|WP_015856168.1|DNAdamageinducibleprotein[Bartonellagrahamii]_gi|240267492|gb|ACS51080.1|helicase/methyltransf/    ------------------------------------------------------------
gi|494588832|ref|WP_007347427.1|DNAdamageinducibleprotein[Bartonellarattimassiliensis]_gi|395419053|gb|EJF85361.1|hypothetical/    ------------------------------------------------------------
gi|501188650|ref|WP_012231668.1|DNAdamageinducibleprotein[Bartonellatribocorum]_gi|161017902|emb|CAK01460.1|helicase/methyltra/    ------------------------------------------------------------
gi|501188624|ref|WP_012231643.1|DNAdamageinducibleprotein[Bartonellatribocorum]_gi|161017866|emb|CAK01424.1|helicase/methyltra/    ------------------------------------------------------------
gi|518053856|ref|WP_019224064.1|DNAdamageinducibleprotein[Bartonellarattaustraliani]/11625           ------------------------------------------------------------
gi|501188053|ref|WP_012231081.1|DNAdamageinducibleprotein[Bartonellatribocorum]_gi|161017434|emb|CAK00992.1|helicase/methyltra/    ------------------------------------------------------------
gi|651388630|ref|WP_026500626.1|damageinducibleprotein[Bartonellagrahamii]/11657           ------------------------------------------------------------
gi|518048855|ref|WP_019219063.1|DNAdamageinducibleprotein[Bartonellaflorencae]/11658           ------------------------------------------------------------
gi|490995101|ref|WP_004856829.1|DNAdamageinducibleprotein[Bartonelladoshiae]_gi|395413167|gb|EJF79645.1|hypotheticalproteinMCS/    ------------------------------------------------------------
gi|491002558|ref|WP_004864279.1|DNAdamageinducibleprotein[Bartonellavinsonii]_gi|395421392|gb|EJF87643.1|hypotheticalproteinME/    ------------------------------------------------------------
gi|491003373|ref|WP_004865094.1|DNAdamageinducibleprotein[Bartonellavinsonii]_gi|395432864|gb|EJF98838.1|hypotheticalproteinME/    ------------------------------------------------------------
gi|491002426|ref|WP_004864148.1|DNAdamageinducibleprotein[Bartonellavinsonii]_gi|395421767|gb|EJF88003.1|hypotheticalproteinME/    ------------------------------------------------------------
gi|491004024|ref|WP_004865745.1|DNAdamageinducibleprotein[Bartonellavinsonii]_gi|395432542|gb|EJF98522.1|hypotheticalproteinME/    ------------------------------------------------------------
gi|648396442|ref|WP_026088193.1|damageinducibleprotein[Bartonellarattaustraliani]/11593           ------------------------------------------------------------
gi|748190174|ref|WP_039761290.1|damageinducibleprotein[Bartonellaqueenslandensis]/11658           ------------------------------------------------------------
gi|494817599|ref|WP_007553007.1|DNAdamageinducibleprotein[Bartonellasp.DB56]_gi|395411312|gb|EJF77837.1|hypotheticalproteinMC/1    ------------------------------------------------------------
gi|515763376|ref|WP_017195976.1|DNAdamageinducibleprotein[Bartonellabirtlesii]/11656           ------------------------------------------------------------
gi|494589420|ref|WP_007348014.1|DNAdamageinducibleprotein[Bartonellarattimassiliensis]_gi|395416278|gb|EJF82667.1|hypothetical/    ------------------------------------------------------------
gi|506336469|ref|WP_015856188.1|DNAdamageinducibleprotein[Bartonellagrahamii]_gi|240267512|gb|ACS51100.1|helicase/methyltransf/    ------------------------------------------------------------
gi|749308087|ref|WP_040132227.1|damageinducibleprotein[Bartonellatribocorum]_gi|605051878|emb|CDO49988.1|helicase/methyltransf/    ------------------------------------------------------------
gi|515763800|ref|WP_017196400.1|DNAdamageinducibleprotein[Bartonellabirtlesii]/11658           ------------------------------------------------------------
gi|492186994|ref|WP_005773380.1|DNAdamageinducibleprotein[Bartonellaelizabethae]_gi|395418361|gb|EJF84688.1|hypotheticalprotei/    ------------------------------------------------------------
gi|501188615|ref|WP_012231634.1|DNAdamageinducibleprotein[Bartonellatribocorum]_gi|161017856|emb|CAK01414.1|helicase/methyltra/    ------------------------------------------------------------
gi|501187470|ref|WP_012230499.1|DNAdamageinducibleprotein[Bartonellatribocorum]_gi|161017090|emb|CAK00648.1|helicase/methyltra/    ------------------------------------------------------------
gi|501188636|ref|WP_012231655.1|DNAdamageinducibleprotein[Bartonellatribocorum]_gi|161017879|emb|CAK01437.1|helicase/methyltra/    ------------------------------------------------------------
gi|930151941|emb|CTQ34818.1|UvsWhelicase[Jannaschiarubra]/11641           ------------------------------------------------------------
gi|928977106|ref|WP_054001461.1|damageinducibleprotein[Shimiasp.SK013]_gi|927698852|gb|KPA22698.1|typeIrestrictionenzymeEcoKIs/    ------------------------------------------------------------
gi|738872465|ref|WP_036760884.1|damageinducibleprotein[Leisingeradaeponensis]/11643           ------------------------------------------------------------
gi|518050881|ref|WP_019221089.1|DNAdamageinducibleprotein[Bartonellasenegalensis]/11652           ------------------------------------------------------------
gi|493980596|ref|WP_006923523.1|DNAdamageinducibleprotein[Bartonellawashoensis]_gi|395413322|gb|EJF79796.1|hypotheticalprotein/    ------------------------------------------------------------
gi|493983106|ref|WP_006925984.1|DNAdamageinducibleprotein[Bartonellawashoensis]_gi|395418127|gb|EJF84455.1|hypotheticalprotein/    ------------------------------------------------------------
gi|499494671|ref|WP_011181311.1|DNAdamageinducibleprotein[Bartonellahenselae]_gi|49239008|emb|CAF28308.1|hypotheticalproteinBH/    ------------------------------------------------------------
gi|651388426|ref|WP_026500441.1|damageinducibleprotein[Bartonellagrahamii]/11652           ------------------------------------------------------------
gi|506337280|ref|WP_015856999.1|DNAdamageinducibleprotein[Bartonellagrahamii]_gi|240268402|gb|ACS51990.1|helicase/methyltransf/    ------------------------------------------------------------
gi|494817535|ref|WP_007552943.1|DNAdamageinducibleprotein[Bartonellasp.DB56]_gi|395412002|gb|EJF78518.1|hypotheticalproteinMC/1    ------------------------------------------------------------
gi|493638099|ref|WP_006589864.1|DNAdamageinducibleprotein[Bartonellabirtlesii]_gi|395409809|gb|EJF76394.1|hypotheticalproteinM/    ------------------------------------------------------------
gi|748189589|ref|WP_039760707.1|damageinducibleprotein[Bartonellaqueenslandensis]/11651           ------------------------------------------------------------
gi|501189483|ref|WP_012232501.1|DNAdamageinducibleprotein[Bartonellatribocorum]_gi|161018902|emb|CAK02460.1|helicase/methyltra/    ------------------------------------------------------------
gi|518049181|ref|WP_019219389.1|DNAdamageinducibleprotein[Bartonellaflorencae]/11652           ------------------------------------------------------------
gi|492193255|ref|WP_005774528.1|DNAdamageinducibleprotein[Bartonellaelizabethae]_gi|395416403|gb|EJF82779.1|hypotheticalprotei/    ------------------------------------------------------------
gi|498387325|ref|WP_010701288.1|helicase/methyltransferase[Bartonellabovis]_gi|478418724|gb|ENN92426.1|helicase/methyltransfera    ------------------------------------------------------------
gi|319408697|emb|CBI82352.1|conservedhypotheticalprotein[BartonellaschoenbuchensisR1]/11661           ------------------------------------------------------------
gi|494741726|ref|WP_007477134.1|DNAdamageinducibleprotein[Bartonellamelophagi]_gi|395423925|gb|EJF90113.1|hypotheticalproteinM/    ------------------------------------------------------------
gi|737261405|ref|WP_035245349.1|damageinducibleprotein[Alcanivoraxjadensis]_gi|686989709|gb|KGD62556.1|hypotheticalproteinT9A_/    ------------------------------------------------------------
gi|916687564|ref|WP_051294655.1|damageinducibleprotein[Gemmobacternectariphilus]/11641           ------------------------------------------------------------
gi|740294127|ref|WP_038132495.1|damageinducibleprotein[Thioclavasp.DT234]_gi|660524826|gb|KEO54689.1|hypotheticalproteinDT23_/1    ------------------------------------------------------------
gi|568331062|ref|WP_024099615.1|helicase[Phaeobactergallaeciensis]_gi|568232121|gb|AHD12007.1|putativehelicase[Phaeobactergalla    ------------------------------------------------------------
gi|518810459|ref|WP_019966413.1|DNAdamageinducibleprotein[Pannonibacterphragmitetus]/11635           ------------------------------------------------------------
gi|495305760|ref|WP_008030510.1|DNAdamageinducibleprotein[Rhodobactersp.SW2]_gi|259021698|gb|EEW25000.1|typeIIIrestrictionprot/    ------------------------------------------------------------
gi|506403303|ref|WP_015923022.1|DNAdamageinducibleprotein[Rhodobactersphaeroides]_gi|221162951|gb|ACM03922.1|HypotheticalProte/    ------------------------------------------------------------
gi|738818386|ref|WP_036708790.1|damageinducibleprotein[Paracoccussanguinis]_gi|694226786|gb|KGJ22541.1|damageinducibleprotein/1    ------------------------------------------------------------
gi|746096057|ref|WP_039162453.1|damageinducibleprotein[Gallibacteriumanatis]_gi|703617775|gb|KGQ63868.1|damageinducibleprotei/1    ------------------------------------------------------------
gi|737716785|ref|WP_035685631.1|damageinducibleprotein[Avibacteriumparagallinarum]/11643           ------------------------------------------------------------
gi|540072553|gb|ERF78672.1|hypotheticalproteinN561_04930[Gallibacteriumanatis12656/12}/11651           ------------------------------------------------------------
gi|917509519|ref|WP_052115936.1|damageinducibleprotein[Gallibacteriumanatis]_gi|702414161|gb|KGQ42233.1|damageinducibleprotei/1    ------------------------------------------------------------
gi|746082942|ref|WP_039150268.1|damageinducibleprotein[Gallibacteriumanatis]_gi|703617078|gb|KGQ63194.1|damageinducibleprotei/1    ------------------------------------------------------------
gi|822522885|ref|WP_046952840.1|damageinducibleprotein[Haemophilushaemolyticus]_gi|821501762|gb|KKZ59267.1|damageinduciblepro/1    ------------------------------------------------------------
gi|404381014|gb|EJZ77501.1|putativehelicase[Pasteurellamultocidasubsp.gallicidaP1059]/11681           ------------------------------------------------------------
```

```
gi|298281660|gb|EFI23149.1|helicaseCterminaldomainprotein[Neisseriasp.oraltaxon014str.F0314]/11493 ------------------------------------------------------------
gi|348013055|gb|EGY51980.1|helicasedomainprotein[Neisseriashayeganii871]/11481 ------------------------------------------------------------
gi|496407140|ref|WP_009116004.1|DNAdamageinducibleprotein[Neisseriawadsworthii]_gi|349794756|gb|EGZ48566.1|helicasedomainprote/ ------------------------------------------------------------
gi|517216441|ref|WP_018405259.1|DNAdamageinducibleprotein[Marinobacterlipolyticus]/11656 ------------------------------------------------------------
gi|738230264|ref|WP_036185691.1|damageinducibleprotein[Marinobactersp.ELB17]/11671 ------------------------------------------------------------
gi|447197741|ref|WP_001274997.1|DNAdamageinducibleprotein[Acinetobacterbaumannii]_gi|408512324|gb|EKK13969.1|typeIIIrestrictio/ ------------------------------------------------------------
gi|820775897|ref|WP_046739909.1|damageinducibleprotein[Acinetobactersp.AG1]_gi|819909758|gb|KKW75938.1|damageinducibleprotein/1 ------------------------------------------------------------
gi|584595163|ref|WP_024160846.1|helicase[Acinetobacterpittii]_gi|576702530|emb|CDI28049.1|helicasedomaincontainingprotein[Acin/ ------------------------------------------------------------
gi|514963042|ref|WP_016651366.1|hypotheticalprotein[Acinetobactersp.NIPH2036]_gi|514162665|gb|EPG41391.1|hypotheticalproteinF90 ------------------------------------------------------------
gi|515965564|ref|WP_017396147.1|DNAdamageinducibleprotein[Acinetobacterhaemolyticus]/11667 ------------------------------------------------------------
gi|491346810|ref|WP_005204747.1|MULTISPECIES:hypotheticalprotein[Acinetobacter]_gi|480335823|gb|ENX56033.1|hypotheticalproteinF ------------------------------------------------------------
gi|693044472|ref|WP_032215268.1|damageinducibleprotein[Escherichiacoli]_gi|607725731|gb|EZE08230.1|damageinducibleprotein[Esc/1 ------------------------------------------------------------
gi|742382177|ref|WP_038861304.1|damageinducibleprotein[Cronobactermuytjensii]_gi|924333747|gb|ALB69766.1|damageinducibleprote/1 ------------------------------------------------------------
gi|915912804|ref|WP_050927035.1|damageinducibleprotein[Yersiniaenterocolitica]_gi|899805802|emb|CQH32042.1|typeIrestrictionenz/ ------------------------------------------------------------
gi|921111327|gb|KON62781.1|typeIrestrictionenzymeEcoKIsubunitR[Komagataeibactereuropaeus]/11492 ------------------------------------------------------------
gi|582024278|gb|AHI27615.1|DNAhelicaserestrictionenzymeTypeIIIRsubunit[KomagataeibacterxylinusE25]/11636 ------------------------------------------------------------
gi|347662673|dbj|BAK86128.1|DNAhelicaserestrictionenzymeTypeIIIRsubunit[KomagataeibactermedellinensisNBRC3288]/11737 ------------------------------------------------------------
gi|917445053|ref|WP_052051471.1|damageinducibleprotein[Acetobactertropicalis]/11429 ------------------------------------------------------------
gi|453329445|dbj|GAC88472.1|DNAhelicaserestrictionenzymetypeIIIRsubunit[GluconobacterthailandicusNBRC3255]_gi|530287981|dbj|GAD ------------------------------------------------------------
gi|889770922|ref|WP_048844278.1|damageinducibleprotein[Gluconobacterfrateurii]/11419 ------------------------------------------------------------
gi|504886208|ref|WP_015073310.1|hypotheticalprotein[Gluconobacteroxydans]_gi|411027954|gb|AFW01209.1|hypotheticalproteinB932_16 ------------------------------------------------------------
gi|480040211|gb|ENV79751.1|hypotheticalproteinF942_01641[AcinetobacterursingiiANC3649]/11432 ------------------------------------------------------------
gi|822498222|ref|WP_046936504.1|damageinducibleprotein[Xanthomonasgardneri]_gi|821751224|gb|KLB07223.1|damageinducibleprotein/1 ------------------------------------------------------------
gi|654059559|ref|WP_027705556.1|damageinducibleprotein[Zymobacterpalmae]/11635 ------------------------------------------------------------
gi|643535620|ref|WP_025224315.1|damageinducibleprotein[Methylophilaceaebacterium11]_gi|577704400|gb|EUJ10546.1|putativehelicas/ ------------------------------------------------------------
MBA11I/11635 ------------------------------------------------------------
gi|655355857|ref|WP_028762287.1|damageinducibleprotein[Shewanellacolwelliana]/11632 ------------------------------------------------------------
gi|753204236|ref|WP_041510579.1|damageinducibleprotein[Shewanellasp.cp20]_gi|752457765|gb|KIO36326.1|damageinducibleprotein[S/1 ------------------------------------------------------------
KPNNIH30III/11619 ------------------------------------------------------------
gi|749295994|ref|WP_040120352.1|damageinducibleprotein[Klebsiellapneumoniae]_gi|723249300|gb|AIX81926.1|damageinducibleprotei/1 ------------------------------------------------------------
gi|641639739|emb|CDQ54010.1|unnamedproteinproduct[Klebsiellapneumoniae]/11641 ------------------------------------------------------------
gi|490797922|ref|WP_004660060.1|hypotheticalprotein[Acinetobactersp.NIPH236]_gi|479876779|gb|ENU27973.1|hypotheticalproteinF992 ------------------------------------------------------------
gi|916750926|ref|WP_051357982.1|damageinducibleprotein[Acinetobactertjernbergiae]/11453 ------------------------------------------------------------
gi|691098662|ref|WP_032025060.1|damageinducibleprotein[Acinetobacterbaumannii]_gi|593567521|gb|EXR50735.1|restrictionendonucle/ ------------------------------------------------------------
gi|736669405|ref|WP_034676049.1|damageinducibleprotein[Acinetobacterbaumannii]/11643 ------------------------------------------------------------
gi|491368928|ref|WP_005226826.1|hypotheticalprotein[Acinetobactersp.NIPH1847]_gi|480173259|gb|ENX09797.1|hypotheticalproteinF89 ------------------------------------------------------------
gi|796853993|ref|WP_045795953.1|damageinducibleprotein[Acinetobacterindicus]_gi|787929761|gb|KJV44219.1|damageinducibleprotei/1 ------------------------------------------------------------
gi|295797463|gb|ADG32253.1|typeIIIrestrictionproteinressubunit[ThiomonasintermediaK12]/11676 ------------------------------------------------------------
gi|499871179|ref|WP_011551913.1|DNAdamageinducibleprotein[Myxococcusxanthus]_gi|108467544|gb|ABF92729.1|putativerestriction/mo/ ------------------------------------------------------------
gi|495313852|ref|WP_008038600.1|DNAdamageinducibleprotein[Bartonellatamiae]_gi|395424181|gb|EJF90368.1|hypotheticalproteinME5_/ ------------------------------------------------------------
gi|501453335|ref|WP_012476784.1|DNAdamageinducibleprotein[Leptospirabiflexa]_gi|167777824|gb|ABZ96123.1|Helicase[Leptospirabif/ ------------------------------------------------------------
gi|738104613|ref|WP_036063193.1|damageinducibleprotein[Leptospiranoguchii]/11652 ------------------------------------------------------------
gi|410001039|gb|EKO51661.1|typeIIIrestrictionenzyme,ressubunit[Leptospirakirschneristr.200802841]/11651 ------------------------------------------------------------
gi|490901851|ref|WP_004763774.1|typeIIIrestrictionenzyme,ressubunit[Leptospirakirschneri]_gi|464209249|gb|EMN24014.1|typeIIIres ------------------------------------------------------------
gi|464398131|gb|EMO65902.1|typeIIIrestrictionenzyme,ressubunit[Leptospirakirschneristr.200803703]/11648 ------------------------------------------------------------
gi|945029024|gb|KQQ84900.1|damageinducibleprotein[Xanthomonassp.Leaf131]/11423 ------------------------------------------------------------
gi|896511314|ref|WP_049418183.1|damageinducibleprotein[Stenotrophomonasmaltophilia]/11639 ------------------------------------------------------------
gi|896513082|ref|WP_049419616.1|damageinducibleprotein[Stenotrophomonasmaltophilia]/11638 ------------------------------------------------------------
gi|896580616|ref|WP_049469425.1|damageinducibleprotein[Stenotrophomonasmaltophilia]/11641 ------------------------------------------------------------
gi|648289350|ref|WP_026070499.1|damageinducibleprotein[Stenotrophomonasmaltophilia]/11639 ------------------------------------------------------------
gi|656272786|ref|WP_029218442.1|damageinducibleprotein[Xanthomonascassavae]/11639 ------------------------------------------------------------
gi|406976236|gb|EKD98747.1|hypotheticalproteinACD_23C00290G0002[unculturedbacterium]/11679 ------------------------------------------------------------
gi|828145737|ref|WP_047238120.1|damageinducibleprotein[Chromobacteriumsubtsugae]/11645 ------------------------------------------------------------
gi|654311174|ref|WP_027824327.1|damageinducibleprotein[Laribacterhongkongensis]/11641 ------------------------------------------------------------
gi|757242173|gb|KIR15516.1|typeIrestrictionenzymeEcoKIsubunitR[Pseudomonasfluorescens]/11778 ------------------------------------------------------------
gi|653605161|ref|WP_027616682.1|damageinducibleprotein[Pseudomonassp.URHB0015]/11639 ------------------------------------------------------------
gi|520868025|ref|WP_020308060.1|helicase[Pseudomonassyringae]_gi|519159034|gb|EPM49935.1|helicasedomaincontainingprotein[Pseud/ ------------------------------------------------------------
gi|940156567|gb|KPX71668.1|Helicasedomaincontainingprotein[Pseudomonasamygdalipv.lachrymans]/11677 ------------------------------------------------------------
gi|505460508|ref|WP_015647610.1|helicase[Pseudomonassp.GLE121]_gi|499110212|ref|YP_007974374.1|helicasedomaincontainingprotein/ ------------------------------------------------------------
gi|940235551|gb|KPY45311.1|hypotheticalproteinALO48_200154[Pseudomonassyringaepv.rhaphiolepidis]/11631 ------------------------------------------------------------
gi|520880808|ref|WP_020312235.1|helicase[Pseudomonassyringae]_gi|519172158|gb|EPM62027.1|helicasedomaincontainingprotein[Pseud/ ------------------------------------------------------------
gi|829046479|ref|WP_047282259.1|damageinducibleprotein[Pseudomonasfluorescens]/11630 ------------------------------------------------------------
gi|499415493|ref|WP_011102960.1|DNAdamageinducibleprotein[Pseudomonassyringaegroupgenomosp.3]_gi|28867279|ref|NP_789898.1|heli/ ------------------------------------------------------------
gi|730238299|ref|WP_033942897.1|damageinducibleprotein[Pseudomonasaeruginosa]/11629 ------------------------------------------------------------
gi|495310125|ref|WP_008034873.1|DNAdamageinducibleprotein[Pseudomonassp.GM60]_gi|398186685|gb|EJM74052.1|putativehelicase[Pseu/ ------------------------------------------------------------
gi|779992653|ref|WP_045428690.1|damageinducibleprotein[Pseudomonassp.MT1]_gi|699967872|dbj|BAP80334.1|DNAhelicaserestrictione/1 ------------------------------------------------------------
gi|786037289|ref|WP_045733274.1|damageinducibleprotein[Pseudomonaspseudoalcaligenes]_gi|782987904|gb|KJU79152.1|damageinducib/1 ------------------------------------------------------------
```

```
gi|749666366|ref|WP_040265445.1|damageinducibleprotein[Pseudomonasrhodesiae]/11632          ------------------------------------------------------------
gi|734902433|ref|WP_034133698.1|damageinducibleprotein[Pseudomonasfluorescens]/11635          ------------------------------------------------------------
gi|490551031|ref|WP_004416146.1|typeIIrestrictionmodificationsystemrestriction/helicasesubunit[Pseudomonassyringae]_gi|4432823/          ------------------------------------------------------------
gi|489199911|ref|WP_003109152.1|DNAdamageinducibleprotein[Pseudomonasaeruginosa]_gi|552648708|gb|ERY63184.1|hypotheticalprotei/          ------------------------------------------------------------
gi|730641237|ref|WP_034061328.1|damageinducibleprotein[Pseudomonasaeruginosa]/11636          ------------------------------------------------------------
gi|751304980|ref|WP_041013386.1|damageinducibleprotein[Pseudomonasxanthomarina]_gi|703407611|emb|CEG51014.1|Helicasedomaincon/1          ------------------------------------------------------------
gi|939335207|ref|WP_054881520.1|damageinducibleprotein[Pseudomonassp.NBRC111128]/11632          ------------------------------------------------------------
gi|757693555|ref|WP_042923792.1|damageinducibleprotein[Pseudomonaschloritidismutans]_gi|780910610|gb|KJS78302.1|damageinducib/1          ------------------------------------------------------------
gi|665953944|ref|WP_031319989.1|damageinducibleprotein[Pseudomonastolaasii]/11626          ------------------------------------------------------------
gi|771600573|ref|WP_045213746.1|damageinducibleprotein[Pseudomonassp.21]_gi|770081123|gb|KJJ98634.1|damageinducibleprotein[Ps/1          ------------------------------------------------------------
gi|740871396|ref|WP_038656644.1|damageinducibleprotein[Pseudomonasstutzeri]_gi|627772214|gb|AHY41437.1|damageinducibleprotein/1          ------------------------------------------------------------
gi|946242205|gb|KRC93782.1|damageinducibleprotein[Pseudomonassp.Root9]/11634          ------------------------------------------------------------
gi|553777322|ref|WP_023109107.1|MULTISPECIES:hypotheticalprotein[Pseudomonas]_gi|552638674|gb|ERY53184.1|hypotheticalproteinQ05          ------------------------------------------------------------
gi|896383370|ref|WP_049324618.1|damageinducibleprotein[Pseudomonasstutzeri]/11627          ------------------------------------------------------------
gi|696158510|gb|AIT41787.1|putativehelicase[Pseudomonassp.LM8]/11630          ------------------------------------------------------------
gi|504634892|ref|WP_014821994.1|DNAdamageinducibleprotein[Pseudomonasstutzeri]_gi|390985249|gb|AFM35242.1|helicasedomainconta/1          ------------------------------------------------------------
gi|544783974|ref|WP_021206729.1|DNAdamageinducibleprotein[Pseudomonasstutzeri]_gi|533224779|gb|EQM79688.1|DNAdamageinduciblep/1          ------------------------------------------------------------
gi|504567385|ref|WP_014754487.1|MULTISPECIES:DNAdamageinducibleprotein[Pseudomonas]_gi|388559493|gb|AFK68634.1|helicasedomain/1          ------------------------------------------------------------
gi|654441469|ref|WP_027912052.1|damageinducibleprotein[Pseudomonassp.URIL14HWK12:I7]/11630          ------------------------------------------------------------
gi|941470066|ref|WP_055127894.1|damageinducibleprotein[Pseudomonasmediterranea]/11631          ------------------------------------------------------------
gi|557815599|ref|WP_023446184.1|DNAdamageinducibleprotein[Pseudomonaschloritidismutans]_gi|557364081|gb|ESQ98190.1|DNAdamagei/1          ------------------------------------------------------------
gi|727814791|ref|WP_033898247.1|damageinducibleprotein[Pseudomonassp.FH1]_gi|567648005|gb|ETK23229.1|helicasedomaincontaining/1          ------------------------------------------------------------
gi|503278702|ref|WP_013513363.1|DNAdamageinducibleprotein[Desulfovibrioaespoeensis]_gi|316942375|gb|ADU61426.1|typeIIrestrict/          ------------------------------------------------------------
gi|505228796|ref|WP_015415898.1|typeIIrestrictionendonucleasesubunitR[Desulfovibriopiezophilus]_gi|451896976|emb|CCH49855.1|Ty          ------------------------------------------------------------
gi|517732543|ref|WP_018902751.1|hypotheticalprotein[Variovoraxparadoxus]/11675          ------------------------------------------------------------
gi|550909475|ref|WP_022662570.1|DNAdamageinducibleprotein[Desulfovibriolongus]/11637          ------------------------------------------------------------
gi|945494542|gb|KQV50024.1|damageinducibleprotein[Massiliasp.Root335]/11647          ------------------------------------------------------------
gi|334196917|gb|AEG70102.1|helicasedomainprotein[RalstoniasolanacearumPo82]/11663          ------------------------------------------------------------
gi|656006746|ref|WP_029048170.1|damageinducibleprotein[Cupriavidussp.amp6]/11661          ------------------------------------------------------------
BPS2196ORF3054P/11659          ------------------------------------------------------------
gi|740932312|ref|WP_038717332.1|damageinducibleprotein[Burkholderiapseudomallei]_gi|760211882|gb|KIX58314.1|damageinduciblepr/1          ------------------------------------------------------------
gi|755810813|ref|WP_042583861.1|damageinducibleprotein[Burkholderiasp.MSHR3999]_gi|755174952|gb|KIP19790.1|DEAD/DEAHboxhelicas/          ------------------------------------------------------------
gi|484586570|gb|AGK48195.1|DEAD/DEAHboxhelicasefamilyprotein[BurkholderiathailandensisMSMB121]/11646          ------------------------------------------------------------
gi|737582935|ref|WP_035553941.1|damageinducibleprotein[Burkholderiasp.9120]/11658          ------------------------------------------------------------
gi|740989872|ref|WP_038773225.1|damageinducibleprotein[Burkholderiapseudomallei]_gi|705645291|gb|KGS70691.1|DEAD/DEAHboxhelica/          ------------------------------------------------------------
gi|654912525|ref|WP_028363424.1|damageinducibleprotein[Burkholderiasp.JPY366]/11659          ------------------------------------------------------------
gi|740955879|ref|WP_038740512.1|damageinducibleprotein[Burkholderiapseudomallei]_gi|714684932|gb|KGW74438.1|DEAD/DEAHboxhelica/          ------------------------------------------------------------
gi|919203714|ref|WP_052759215.1|damageinducibleprotein[Xanthomonasaxonopodis]/11388          ------------------------------------------------------------
gi|522152381|ref|WP_020663589.1|hypotheticalprotein[Amycolatopsisbenzoatilytica]/11605          ------------------------------------------------------------
gi|663196013|ref|WP_030233899.1|damageinducibleprotein[Streptomycessp.NRRLS455]/11641          ------------------------------------------------------------
gi|750429553|ref|WP_040711444.1|damageinducibleprotein[Nocardiopsispotens]/11622          ------------------------------------------------------------
gi|518656139|ref|WP_019819475.1|hypotheticalprotein[Saccharomonosporasaliphila]/11623          ------------------------------------------------------------
gi|759722933|ref|WP_043433758.1|damageinducibleprotein[Streptomycespluripotens]_gi|733404978|gb|KHK94361.1|damageinduciblepro/1          ------------------------------------------------------------
gi|663424167|ref|WP_030419030.1|damageinducibleprotein[Streptomycessp.NRRLF5065]/11627          ------------------------------------------------------------
gi|663412917|ref|WP_030408353.1|damageinducibleprotein[Streptomycessp.NRRLF5917]/11630          ------------------------------------------------------------
gi|671521483|ref|WP_031506489.1|damageinducibleprotein[Streptomycesmegasporus]/11630          ------------------------------------------------------------
gi|740053144|ref|WP_037902776.1|damageinducibleprotein[Streptomycessp.TAA204]/11626          ------------------------------------------------------------
gi|663409375|ref|WP_030404974.1|MULTISPECIES:damageinducibleprotein[Streptomyces]/11627          ------------------------------------------------------------
gi|746628608|ref|WP_039654277.1|damageinducibleprotein[Streptomycessp.MUSC125]_gi|745644381|gb|KIE25557.1|damageinducibleprot/1          ------------------------------------------------------------
gi|727322066|ref|WP_033777320.1|damageinducibleprotein[Salinisporapacifica]/11627          ------------------------------------------------------------
gi|653653242|ref|WP_027655726.1|damageinducibleprotein[Salinisporapacifica]/11618          ------------------------------------------------------------
gi|648632543|ref|WP_026324294.1|damageinducibleprotein[Salinisporapacifica]/11621          ------------------------------------------------------------
gi|881018002|ref|WP_048738463.1|damageinducibleprotein[Corynebacteriumfalsenii]/11676          ------------------------------------------------------------
gi|505465526|ref|WP_015652417.1|helicase[Corynebacteriumcallunae]_gi|459385704|gb|AGG67995.1|helicase[CorynebacteriumcallunaeDS          ------------------------------------------------------------
gi|41223062|emb|CAF19007.1|DNAorRNAhelicaseofsuperfamilyII[CorynebacteriumglutamicumATCC13032]/11646          ------------------------------------------------------------
CGL13032I/11643          ------------------------------------------------------------
gi|751257081|ref|WP_040967675.1|damageinducibleprotein[Corynebacteriumglutamicum]_gi|748811620|gb|AJE67931.1|damageinduciblep/1          ------------------------------------------------------------
gi|749040539|ref|WP_040086524.1|damageinducibleprotein[Corynebacteriumhumireducens]/11663          ------------------------------------------------------------
gi|491662899|ref|WP_005519615.1|helicase[Corynebacteriummatruchotii]_gi|224946756|gb|EEG27965.1|helicaseCterminaldomainprotein/          ------------------------------------------------------------
gi|746717312|ref|WP_039676231.1|damageinducibleprotein[Corynebacteriumminutissimum]_gi|734991118|gb|KHO29219.1|damageinducibl/1          ------------------------------------------------------------
gi|908654892|emb|CQD15529.1|SuperfamilyIIDNA/RNAhelicase(modularprotein)[Corynebacteriumstriatum]/11674          ------------------------------------------------------------
gi|895994446|ref|WP_049064418.1|damageinducibleprotein[Corynebacteriumaurimucosum]/11667          ------------------------------------------------------------
gi|896123331|ref|WP_049145390.1|damageinducibleprotein[Corynebacteriumaurimucosum]/11669          ------------------------------------------------------------
gi|817681178|ref|WP_046645422.1|damageinducibleprotein[Corynebacteriumstriatum]_gi|817551679|gb|KKO79868.1|damageinduciblepro/1          ------------------------------------------------------------
gi|896139239|ref|WP_049159146.1|damageinducibleprotein[Corynebacteriumaurimucosum]/11675          ------------------------------------------------------------
gi|489943947|ref|WP_003847254.1|helicase[Corynebacteriumammoniagenes]_gi|295968218|gb|EFG81467.1|helicaseCterminaldomainprotei/          ------------------------------------------------------------
gi|516653464|ref|WP_018022453.1|helicase[Corynebacteriumdoosanense]_gi|697778282|gb|AIT61018.1|damageinducibleprotein[Coryneba/          ------------------------------------------------------------
```

```
gi|493894179|ref|WP_006840129.1|helicase[Corynebacteriumlipophiloflavum]_gi|227078819|gb|EEI16782.1|helicaseCterminaldomainpro/      ------------------------------------------------------------
gi|740831472|ref|WP_038616755.1|damageinducibleprotein[Corynebacteriumpseudotuberculosis]_gi|666103058|gb|AIG06935.1|putativeh/      ------------------------------------------------------------
gi|518023322|ref|WP_019193530.1|helicase[Corynebacteriumtimonense]/11655      ------------------------------------------------------------
gi|552767697|ref|WP_023019559.1|MULTISPECIES:hypotheticalprotein[Corynebacterium]_gi|550739013|gb|ERS38609.1|hypotheticalprotei      ------------------------------------------------------------
gi|652659121|ref|WP_027017812.1|damageinducibleprotein[Corynebacteriumpseudodiphtheriticum]/11644      ------------------------------------------------------------
gi|749039309|ref|WP_040085390.1|damageinducibleprotein[Corynebacteriumhumireducens]/11657      ------------------------------------------------------------
gi|740835591|ref|WP_038620874.1|damageinducibleprotein[Corynebacteriumulcerans]_gi|698973854|gb|AIU33664.1|HelicaseCterminald/1      ------------------------------------------------------------
gi|805307599|ref|WP_046096452.1|damageinducibleprotein[Corynebacteriumulcerans]_gi|803357323|gb|AKA97638.1|Damageinduciblepro/1      ------------------------------------------------------------
gi|504083555|ref|WP_014317549.1|helicase[Corynebacteriumdiphtheriae]_gi|371584249|gb|AEX47914.1|superfamilyIIDNA/RNAhelicase[Co      ------------------------------------------------------------
gi|896182193|ref|WP_049193580.1|damageinducibleprotein[Corynebacteriumjeikeium]/11645      ------------------------------------------------------------
gi|917636459|ref|WP_052205460.1|damageinducibleprotein[Corynebacteriumriegelii]_gi|914295811|gb|AKV59187.1|damageinduciblepro/1      ------------------------------------------------------------
gi|737013522|ref|WP_035009205.1|damageinducibleprotein[Corynebacteriumjeikeium]/11651      ------------------------------------------------------------
gi|737006291|ref|WP_035002094.1|damageinducibleprotein[Corynebacteriumjeikeium]/11658      ------------------------------------------------------------
gi|737010378|ref|WP_035006133.1|damageinducibleprotein[Corynebacteriumjeikeium]/11650      ------------------------------------------------------------
gi|737010473|ref|WP_035006228.1|damageinducibleprotein[Corynebacteriumjeikeium]/11657      ------------------------------------------------------------
gi|737006250|ref|WP_035002053.1|damageinducibleprotein[Corynebacteriumjeikeium]/11658      ------------------------------------------------------------
gi|923018168|ref|WP_053412254.1|damageinducibleprotein[Corynebacteriumlactis]_gi|922845184|gb|ALA67504.1|helicase[Corynebacter/      ------------------------------------------------------------
gi|923018885|ref|WP_053412970.1|damageinducibleprotein[Corynebacteriumlactis]_gi|922845845|gb|ALA68165.1|helicase[Corynebacter/      ------------------------------------------------------------
gi|512464870|ref|WP_016422869.1|hypotheticalprotein[Corynebacteriumsp.HFH0082]_gi|512047830|gb|EPD47505.1|hypotheticalproteinHM      ------------------------------------------------------------
gi|881012697|ref|WP_048733406.1|damageinducibleprotein[Corynebacteriumurealyticum]/11670      ------------------------------------------------------------
gi|755608478|ref|WP_042530519.1|damageinducibleprotein[Corynebacteriumsingulare]_gi|754340239|gb|AJI78730.1|putativehelicase[C/      ------------------------------------------------------------
gi|491421796|ref|WP_005279593.1|helicase[Corynebacteriumaccolens]_gi|227077180|gb|EEI15143.1|helicaseCterminaldomainprotein[Co/      ------------------------------------------------------------
gi|550752286|gb|ERS51451.1|hypotheticalproteinHMPREF1282_00033[Corynebacteriumsp.KPL1856]_gi|550759961|gb|ERS58964.1|hypothetic      ------------------------------------------------------------
gi|497955053|ref|WP_010269209.1|helicase[Corynebacteriumbovis]/11599      ------------------------------------------------------------
gi|860580040|ref|WP_048470569.1|hypotheticalprotein[Mycobacteriumchlorophenolicum]_gi|859098575|gb|KMO76871.1|typeIrestrictione      ------------------------------------------------------------
gi|262085905|gb|ACY21873.1|typeIIIrestrictionproteinressubunit[GordoniabronchialisDSM43247]/11632      ------------------------------------------------------------
gi|495996338|ref|WP_008720917.1|typeIIIrestrictionendonucleasesubunitR[Rhodococcussp.AW25M09]_gi|443412350|emb|CCQ18455.1|TypeI      ------------------------------------------------------------
gi|739284636|ref|WP_037147410.1|damageinducibleprotein[Rhodococcusfascians]/11641      ------------------------------------------------------------
gi|739328195|ref|WP_037190574.1|damageinducibleprotein[Rhodococcusfascians]/11643      ------------------------------------------------------------
gi|811698335|emb|CMN21041.1|helicase[Mycobacteriumtuberculosis]/11725      ------------------------------------------------------------
gi|801577898|emb|CFK23773.1|helicase[Mycobacteriumtuberculosis]/11675      ------------------------------------------------------------
MTU18II/11606      ------------------------------------------------------------
gi|887719858|emb|CMT27886.1|helicase[Mycobacteriumtuberculosis]/11667      ------------------------------------------------------------
gi|621694743|gb|KAS87156.1|hypotheticalproteinAL70_01801[MycobacteriumtuberculosisTKK_03_0115]/11604      ------------------------------------------------------------
gi|888128072|emb|CLQ84422.1|helicase[Mycobacteriumtuberculosis]_gi|889917090|emb|CLY86893.1|helicase[Mycobacteriumtuberculosis]      ------------------------------------------------------------
gi|884823424|emb|CKL96672.1|helicase[Mycobacteriumtuberculosis]/11466      ------------------------------------------------------------
gi|505103239|ref|WP_015290341.1|hypotheticalprotein[Mycobacteriumcanettii]_gi|432162733|emb|CCK60117.1|Conservedproteinofunknow      ------------------------------------------------------------
gi|505116250|ref|WP_015303352.1|hypotheticalprotein[Mycobacteriumcanettii]_gi|432158726|emb|CCK56026.1|Conservedproteinofunknow      ------------------------------------------------------------
gi|432154735|emb|CCK51974.1|Conservedproteinofunknownfunction(part1)[MycobacteriumcanettiiCIPT140060008]/11588      ------------------------------------------------------------
gi|809008027|ref|WP_046295375.1|hypotheticalprotein[Mycobacteriumsp.UM_Kg17]/11624      ------------------------------------------------------------
gi|808802633|ref|WP_046284139.1|hypotheticalprotein[Mycobacteriumsp.UM_NZ2]/11624      ------------------------------------------------------------
gi|750125774|ref|WP_040429852.1|damageinducibleprotein[Corynebacteriumnuruki]/11668      ------------------------------------------------------------
gi|602237614|gb|EYT60960.1|damageinducibleprotein[Dietziasp.UCDTHP]/11473      ------------------------------------------------------------
gi|665897412|ref|WP_031264618.1|damageinducibleprotein[Dietziasp.UCDTHP]_gi|602237599|gb|EYT60945.1|helicase[Dietziasp.UCDTH/11      ------------------------------------------------------------
gi|502903674|ref|WP_013138650.1|helicase[Segniliparusrotundus]_gi|296181291|gb|ADG98197.1|typeIIIrestrictionproteinressubunit[S      ------------------------------------------------------------
gi|780248306|ref|WP_045538930.1|hypotheticalprotein[Gordoniasp.no.9]_gi|778142514|gb|KJR06917.1|hypotheticalproteinUG54_12320[G      ------------------------------------------------------------
gi|665897368|ref|WP_031264574.1|damageinducibleprotein[Dietziasp.UCDTHP]_gi|602239137|gb|EYT62483.1|helicase[Dietziasp.UCDTH/11      ------------------------------------------------------------
gi|493372923|ref|WP_006329236.1|helicase[Gordoniarhizosphera]_gi|403207917|dbj|GAB88168.1|hypotheticalproteinGORHZ_006_00370[Go      ------------------------------------------------------------
gi|764904870|ref|WP_044487821.1|hypotheticalprotein[Mycobacteriumtuberculosis]_gi|625005353|gb|KBZ57120.1|hypotheticalproteinK8      ------------------------------------------------------------
gi|927068212|ref|WP_053778441.1|damageinducibleprotein[Gordoniasp.NB41Y]_gi|926660541|gb|EMP14526.2|damageinducibleprotein[G/11      ------------------------------------------------------------
gi|935514169|ref|WP_054429894.1|damageinducibleprotein[Mycobacteriumimmunogenum]_gi|929801120|gb|KPG29247.1|damageinduciblepr/1      ------------------------------------------------------------
gi|941317728|ref|WP_055118574.1|hypotheticalprotein[Mycobacteriumperegrinum]/11657      ------------------------------------------------------------
gi|933683673|gb|ALG86377.1|hypotheticalproteinACH46_20120[Gordoniasp.QH11]/11636      ------------------------------------------------------------
gi|873553947|emb|CRY84110.1|Predictedhelicase[Nocardiafarcinica]/11688      ------------------------------------------------------------
gi|499525749|ref|WP_011212389.1|helicase[Nocardiafarcinica]_gi|54019339|dbj|BAD60707.1|hypotheticalprotein[NocardiafarcinicaIFM      ------------------------------------------------------------
gi|640109609|ref|WP_024794187.1|damageinducibleprotein[Tomitellabiformata]/11653      ------------------------------------------------------------
gi|640109991|ref|WP_024794334.1|hypotheticalprotein[Tomitellabiformata]/11639      ------------------------------------------------------------
gi|939125496|ref|WP_054831438.1|damageinducibleprotein[Rhodococcuserythropolis]/11669      ------------------------------------------------------------
gi|910258550|ref|WP_050062711.1|hypotheticalprotein[Rhodococcussp.RD6.2]_gi|846871967|emb|CRK49225.1|conservedhypotheticalprote      ------------------------------------------------------------
gi|797148189|gb|KJY52358.1|putativeHelicase[Bifidobacteriumasteroides]/11801      ------------------------------------------------------------
gi|535476570|gb|EFG27357.2|hypotheticalproteinHMPREF9020_00999[ScardoviainopinataF0304]/11827      ------------------------------------------------------------
gi|737482729|ref|WP_035462496.1|hypotheticalprotein[Alicyclobacillusmacrosporangiidus]/11512      ------------------------------------------------------------
gi|685434354|gb|KGA95679.1|helicase[BacillusalcalophilusATCC27647=CGMCC1.3604]/11575      ------------------------------------------------------------
gi|945370253|gb|KQU27251.1|helicase[Bacillussp.Leaf13]/11557      ------------------------------------------------------------
gi|880997015|ref|WP_048719116.1|helicase[Enterococcushirae]/11563      ------------------------------------------------------------
gi|945356929|gb|KQU14100.1|helicase[Bacillussp.Leaf49]/11547      ------------------------------------------------------------
gi|763482876|gb|KIZ53217.1|helicase[Bacillussafensis]/11563      ------------------------------------------------------------
```

gi|852223292|ref|WP_048309707.1|helicase[Anaerobacillusmacyae]_gi|850322910|gb|KMM38590.1|helicase[Anaerobacillusmacyae]/11573 --
gi|739708665|ref|WP_037562619.1|helicase[Sporolactobacillusterrae]/11573 --
gi|496687561|ref|WP_009329104.1|MULTISPECIES:helicase[Bacillus]_gi|317390867|gb|EFV71668.1|endonucleaseandmethylaseLlaGI[Bacill --
gi|930213599|gb|KPH74225.1|helicase[Oceanobacilluscaeni]/11569 --
gi|516366003|ref|WP_017756036.1|helicase[Bacillussp.ZYK]/11571 --
gi|488909977|ref|WP_002821052.1|helicase[Oenococcusoeni]_gi|399965230|gb|EJN99855.1|endonucleaseandmethylaseLlaGI[Oenococcusoen --
gi|495019967|ref|WP_007745780.1|helicase[Oenococcuskitaharae]_gi|366983682|gb|EHN59081.1|typeIIIrestrictionenzyme,ressubunit[Oe --
gi|489666119|ref|WP_003570406.1|typeIIIrestrictionendonuclease[Lactobacilluscasei]_gi|410527834|gb|EKQ02696.1|typeIIIrestrictio --
gi|511447623|gb|EPC78110.1|superfamilyIIDNA/RNAhelicase[Lactobacillusparacaseisubsp.paracaseiLpp221}/11615 --
gi|497700411|ref|WP_010014595.1|helicase[Lactobacilluscoryniformis]/11585 --
gi|489784048|ref|WP_003687939.1|helicase[Lactobacillusmali]_gi|394428539|gb|EJF01080.1|superfamilyIIDNA/RNAhelicase[Lactobacill --
gi|446780825|ref|WP_000858081.1|helicase[Staphylococcusaureus]_gi|374396604|gb|EHQ67835.1|typeIIIrestrictionenzyme,ressubunit[S --
gi|510796658|ref|WP_016171456.1|hypotheticalprotein[Enterococcusdispar]_gi|508167532|gb|EOT44083.1|hypotheticalproteinOMK_00227 --
gi|612613473|gb|EZS30411.1|hypotheticalproteinW603_02482[StaphylococcusaureusVET0353R]_gi|612881917|gb|EZU93992.1|hypotheticalp --
gi|488332893|ref|WP_002402278.1|helicase[Enterococcusfaecalis]_gi|306513864|gb|EFM82466.1|helicaseCterminaldomainprotein[Enter/ --
gi|921166029|ref|WP_053111806.1|helicase[Enterococcusdurans]_gi|915771660|gb|AKX85928.1|helicase[Enterococcusdurans]/11562 --
gi|498525358|ref|WP_010825030.1|hypotheticalprotein[Enterococcusfaecalis]_gi|487059631|gb|EOJ86727.1|hypotheticalproteinWOC_005 --
gi|498412624|ref|WP_010722070.1|hypotheticalprotein[Enterococcusfaecium]_gi|486634654|gb|EOF75253.1|hypotheticalproteinSGC_0253 --
gi|498408534|ref|WP_010718364.1|hypotheticalprotein[Enterococcusfaecium]_gi|486609608|gb|EOF50873.1|hypotheticalproteinSCW_0257 --
gi|498407475|ref|WP_010717483.1|hypotheticalprotein[Enterococcusfaecalis]_gi|486594855|gb|EOF36348.1|hypotheticalproteinSCK_005 --
gi|514885392|ref|WP_016615344.1|helicase[Enterococcusfaecalis]_gi|514505843|gb|EPH71676.1|helicaseprotein[Enterococcusfaecalis0 --
gi|325157603|gb|EGC69759.1|helicaseCterminaldomainprotein[EnterococcuscasseliflavusATCC12755]/11561 --
gi|895856206|ref|WP_048967668.1|helicase[Enterococcusfaecalis]/11551 --
gi|639458836|ref|WP_024636366.1|helicase[Enterococcusfaecium]_gi|658176502|gb|KEI51159.1|helicase[EnterococcusfaeciumUC7256]/1/ --
gi|488266511|ref|WP_002337719.1|hypotheticalprotein[Enterococcusfaecium]_gi|430581409|gb|ELB19854.1|hypotheticalproteinOIQ_0351 --
gi|446353356|ref|WP_000431211.1|helicase[Staphylococcusaureus]_gi|253726329|gb|EES95058.1|helicaseCterminaldomainprotein[Staph/ --
gi|672712068|emb|CDM14336.1|DNAorRNAhelicaseofsuperfamilyII[StaphylococcusepidermidisPM221]/11464 --
gi|896298990|ref|WP_049279905.1|helicase[Staphylococcusaureus]/11571 --
gi|541903432|gb|ERH36190.1|hypotheticalproteinSEQU_00085[StaphylococcusequorumUMCCNS924]/11578 --
gi|488289747|ref|WP_002360955.1|helicase[Enterococcusfaecalis]_gi|256682216|gb|EEU21911.1|endonucleaseandmethylaseLlaGI[Enteroc --
gi|494392045|ref|WP_007207894.1|helicase[Enterococcusitalicus]_gi|315483798|gb|EFU74284.1|helicaseCterminaldomainprotein[Enter/ --
gi|446350692|ref|WP_000428547.1|helicase[Bacilluscereus]_gi|228727580|gb|EEL78721.1|RestrictionmodificationsystemLlaBIII[Bacil/ --
gi|488255302|ref|WP_002326510.1|hypotheticalprotein[Enterococcusfaecium]_gi|430480276|gb|ELA57459.1|hypotheticalproteinOGE_0473 --
gi|843253770|ref|WP_047916698.1|helicase[Lactococcuspiscium]_gi|802124677|emb|CEN29502.1|EndonucleaseandmethylaseLlaGI,TypeISPr --
LlaBIII/11578 --
gi|499320531|ref|WP_011011023.1|helicase[Listeriainnocua]_gi|16415805|emb|CAC42019.1|pli0021[ListeriainnocuaClip11262]/11569 --
gi|760026859|ref|WP_043709973.1|helicase[Weissellacibaria]_gi|758337165|gb|KIU20061.1|UvsWhelicase[Weissellacibaria]/11567 --
LlaGI/11570 --
gi|499988414|ref|WP_011669132.1|helicase[Lactococcuslactis]_gi|116109019|gb|ABJ74139.1|DNAorRNAhelicaseofsuperfamilyII[Lactococ --
gi|685938360|ref|WP_031670319.1|helicase[Listeriamonocytogenes]_gi|590403387|gb|EXL23261.1|hypotheticalproteinX846_1956[Listeri --
gi|530793219|gb|EQC56240.1|helicase[Lactococcuslactissubsp.cremorisTIFN5]_gi|530793247|gb|EQC56263.1|helicase[Lactococcuslactis --
gi|695270006|ref|WP_032492075.1|hypotheticalprotein[Listeriamonocytogenes]_gi|695212872|ref|YP_009081522.1|hypotheticalproteinp --
gi|504101310|ref|WP_014335297.1|helicase[Leuconostocmesenteroides]_gi|859247905|gb|AKO63885.1|helicase(plasmid)[Leuconostocmese --
gi|488905190|ref|WP_002816265.1|helicase[Leuconostocmesenteroides]_gi|227351522|gb|EEJ41777.1|helicaseCterminaldomainprotein[L/ --
gi|518121285|ref|WP_019291493.1|MULTISPECIES:hypotheticalprotein[Lactococcus]/11588 --
gi|754857213|ref|WP_042217902.1|helicase[Lactococcusgarvieae]/11549 --
gi|640048455|ref|WP_024784762.1|helicase[Streptococcusmutans]/11564 --
gi|512695361|ref|WP_016476323.1|hypotheticalprotein[Veillonellasp.HPA0037]_gi|512079853|gb|EPD79018.1|hypotheticalproteinHMPREF --
gi|636963514|ref|WP_024403747.1|helicase[Streptococcussuis]/11574 --
gi|881115312|ref|WP_048802949.1|helicase[Staphylococcuspasteuri]/11573 --
gi|446063996|ref|WP_000141851.1|helicase[Streptococcusmitis]_gi|307618725|gb|EFN97864.1|endonucleaseandmethylaseLlaGI[Streptoco --
gi|488206609|ref|WP_002277817.1|restrictionmodificationsystemLlaBIII[Streptococcusmutans]_gi|449173033|gb|EMB75626.1|restricti/ --
gi|640023290|ref|WP_024781621.1|helicase[Streptococcusmutans]/11566 --
gi|814946581|ref|WP_046393781.1|helicase[Streptococcusuberis]_gi|810600885|gb|KKF57499.1|helicase[StreptococcusuberisB362]/1156 --
gi|765470762|ref|WP_044720336.1|helicase[Streptococcussuis]/11559 --
gi|654507127|ref|WP_027976443.1|helicase[Streptococcusdevriesei]/11558 --
gi|530812902|gb|EQC72481.1|DNAorRNAhelicaseofsuperfamilyII[Streptococcussp.HSISS2]/11570 --
gi|489158846|ref|WP_003068504.1|helicase[Streptococcusintermedius]_gi|355367114|gb|EHG14827.1|hypotheticalproteinHMPREF9682_002 --
RM.Lru27782ORF13200P/11620 --
gi|492676546|ref|WP_005919236.1|DNAphosphorothioationsystemrestrictionenzyme[Pediococcusacidilactici]_gi|482517924|gb|EOA08103. --
gi|498183116|ref|WP_010497272.1|helicase[Lactobacillusacidipiscis]/11563 --
gi|822100485|ref|WP_046921865.1|helicase[Lactobacillusruminis]_gi|821684442|gb|KLA43321.1|restrictionmodificationsystemLlaBIII/ --
gi|935565832|ref|WP_054450311.1|helicase[Lactobacilluskunkeei]/11573 --
gi|752296887|ref|WP_041152901.1|helicase[Lactobacilluskunkeei]_gi|751663520|gb|KIM18358.1|helicase[Lactobacilluskunkeei]/11577 --
gi|495745291|ref|WP_008469870.1|helicase[Lactobacillushominis]_gi|394484442|emb|CCI81207.1|RestrictionmodificationsystemLlaBII/ --
gi|918672414|ref|WP_052543134.1|helicase[Lactobacillussp.]/11564 --
gi|799141631|ref|WP_045927261.1|helicase[Lactobacilluskullabergensis]_gi|797155335|gb|KJY59264.1|putativerestrictionendonucleas --

```
gi|504379990|ref|WP_014567092.1|helicase[Lactobacillusjohnsonii]_gi|329666726|gb|AEB92674.1|putativerestrictionendonuclease[Lac      ------------------------------------------------------------
gi|489880179|ref|WP_003783646.1|DNAdamageinducibleprotein[Actinomycesnaeslundii]_gi|399901561|gb|EJN84440.1|typeIIIrestriction/      ------------------------------------------------------------
gi|496524497|ref|WP_009232780.1|DNAdamageinducibleprotein[Actinomycessp.oraltaxon849]_gi|365265413|gb|EHM95177.1|hypotheticalp/      ------------------------------------------------------------
gi|915397782|ref|WP_050783167.1|damageinducibleprotein[Actinomycesoris]/11694                                                         ------------------------------------------------------------
gi|502925248|ref|WP_013160224.1|helicase[Propionibacteriumfreudenreichii]_gi|296921272|emb|CBL55824.1|helicase[Propionibacteriu      ------------------------------------------------------------
gi|732164352|emb|CEG87481.1|helicase[Propionibacteriumfreudenreichii]/11618                                                           ------------------------------------------------------------
gi|739047402|ref|WP_036925058.1|damageinducibleprotein[Propionibacteriumthoenii]/11596                                                ------------------------------------------------------------
gi|739112839|ref|WP_036982533.1|damageinducibleprotein[Propionibacteriumjensenii]/11594                                               ------------------------------------------------------------
gi|918023558|ref|WP_052314121.1|damageinducibleprotein[Nocardiathailandica]/11612                                                     ------------------------------------------------------------
gi|498380271|ref|WP_010694427.1|DNAdamageinducibleprotein[Saccharopolysporaspinosa]/11605                                             ------------------------------------------------------------
gi|749991459|ref|WP_040339809.1|damageinducibleprotein[CandidatusBlastococcusmassiliensis]/11597                                      ------------------------------------------------------------
gi|754177801|ref|WP_041796231.1|damageinducibleprotein[Modestobactermarinus]/11592                                                    ------------------------------------------------------------
gi|504188248|ref|WP_014375350.1|DNAdamageinducibleprotein[Blastococcussaxobsidens]_gi|378782790|emb|CCG02456.1|putativeHelicas/      ------------------------------------------------------------
gi|946476713|gb|KRF26798.1|damageinducibleprotein[Phycicoccussp.Soil803]/11609                                                        ------------------------------------------------------------
gi|700176672|gb|KGN37509.1|DNAdamageinducibleprotein[KnoelliasubterraneaKCTC19937]/11633                                              ------------------------------------------------------------
gi|700168841|gb|KGN29914.1|hypotheticalproteinN802_10215[KnoelliasinensisKCTC19936]/11631                                             ------------------------------------------------------------
gi|946426241|gb|KRE76610.1|damageinducibleprotein[Arthrobactersp.Soil763]/11606                                                       ------------------------------------------------------------
gi|517318525|ref|WP_018500927.1|hypotheticalprotein[Frankiasp.BCU110501]/11592                                                        ------------------------------------------------------------
gi|499072429|ref|WP_010851327.1|Helicase[Tetrasphaeraelongata]_gi|478756579|emb|CCH71501.1|putativeHelicase[Tetrasphaeraelongat      ------------------------------------------------------------
gi|750080666|ref|WP_040385350.1|damageinducibleprotein[Demetriaterragena]/11625                                                       ------------------------------------------------------------
gi|752558292|ref|WP_041229523.1|damageinducibleprotein[Dermacoccusnishinomiyaensis]_gi|664689315|gb|AIF41904.1|damageinducibl/1      ------------------------------------------------------------
gi|829089213|ref|WP_047310701.1|damageinducibleprotein[Dermacoccussp.PE3]_gi|828384812|gb|KLO63770.1|damageinducibleprotein[D/1      ------------------------------------------------------------
gi|748756635|ref|WP_040014750.1|damageinducibleprotein[Dermacoccussp.Ellin185]/11596                                                  ------------------------------------------------------------
gi|916534724|ref|WP_051141848.1|damageinducibleprotein[Actinomycesneuii]/11632                                                        ------------------------------------------------------------
gi|921140030|ref|WP_053090276.1|damageinducibleprotein[Rothiamucilaginosa]/11616                                                      ------------------------------------------------------------
gi|896406751|ref|WP_049343749.1|damageinducibleprotein[Rothiamucilaginosa]/11647                                                      ------------------------------------------------------------
gi|723608234|gb|AIY01610.1|putativehelicase[Arthrobactersp.PAMC25486]/11613                                                           ------------------------------------------------------------
gi|946394527|gb|KRE45160.1|damageinducibleprotein[Arthrobactersp.Soil736]/11616                                                       ------------------------------------------------------------
gi|515765304|ref|WP_017197904.1|hypotheticalprotein[Arthrobactersp.M2012083]/11648                                                    ------------------------------------------------------------
gi|945315587|gb|KQT73127.1|damageinducibleprotein[Microbacteriumsp.Leaf436]/11612                                                     ------------------------------------------------------------
gi|759908497|ref|WP_043594599.1|damageinducibleprotein[Frigoribacteriumsp.MEB024]_gi|758271359|gb|KIU03346.1|damageinduciblep/1      ------------------------------------------------------------
gi|945072850|gb|KQR34237.1|hypotheticalproteinASF75_00055[Curtobacteriumsp.Leaf154]/11597                                             ------------------------------------------------------------
gi|551263152|ref|WP_022883363.1|DNAdamageinducibleprotein[Glaciibactersuperstes]/11621                                                ------------------------------------------------------------
gi|945452341|gb|KQV08491.1|hypotheticalproteinASC63_10170[Leifsoniasp.Root112D2]/11623                                                ------------------------------------------------------------
gi|742759483|gb|KIC69912.1|damageinducibleprotein[Kocuriarhizophila]/11610                                                           ------------------------------------------------------------
gi|737919995|ref|WP_035885304.1|damageinducibleprotein[Kocuriarhizophila]/11611                                                       ------------------------------------------------------------
gi|737997701|ref|WP_035959325.1|damageinducibleprotein[Kocuriamarina]_gi|725823884|gb|KHE75603.1|damageinducibleprotein[Kocur/1      ------------------------------------------------------------
gi|664521070|ref|WP_031037650.1|damageinducibleprotein[Streptomycessp.NRRLWC3774]/11624                                               ------------------------------------------------------------
gi|500101034|ref|WP_011777041.1|DNAdamageinducibleprotein[Arthrobacteraurescens]_gi|119951547|gb|ABM10457.1|putativeHelicase[A/      ------------------------------------------------------------
gi|916871953|ref|WP_051479009.1|damageinducibleprotein[Arthrobactersp.H5]/11588                                                       ------------------------------------------------------------
gi|612084649|gb|EZP43369.1|TypeIIIrestrictionenzyme,ressubunit[Micrococcusluteus]/11625                                               ------------------------------------------------------------
gi|759739708|ref|WP_043450422.1|damageinducibleprotein[Arthrobacterphenanthrenivorans]_gi|742758264|gb|KIC68716.1|damageinduc/1      ------------------------------------------------------------
gi|636845448|ref|WP_024366823.1|damageinducibleprotein[Arthrobactersp.TB26]/11605                                                     ------------------------------------------------------------
gi|500009025|ref|WP_011689743.1|DNAdamageinducibleprotein[Arthrobactersp.FB24]_gi|116613051|gb|ABK05760.1|typeIIIrestrictionen/      ------------------------------------------------------------
gi|821249847|gb|KKZ12083.1|hypotheticalproteinTQ37_06090[CandidatusSynechococcusspongiarum15L]/11436                                  ------------------------------------------------------------
gi|739446902|ref|WP_037306885.1|damageinducibleprotein[Ruegeriahalocynthiae]/11592                                                    ------------------------------------------------------------
gi|640620081|ref|WP_025048131.1|damageinducibleprotein[Sulfitobactermediterraneus]_gi|751956650|gb|KIN77213.1|TypeIIIrestricti/      ------------------------------------------------------------
gi|740247634|ref|WP_038088583.1|damageinducibleprotein[Thiobacillusprosperus]_gi|684049748|gb|KFZ90853.1|putativehelicase[Thio/      ------------------------------------------------------------
gi|518420321|ref|WP_019590528.1|hypotheticalprotein[Thioalkalivibriosp.ALE20]/11603                                                   ------------------------------------------------------------
gi|500156826|ref|WP_011831496.1|DNAdamageinducibleprotein[Methylibiumpetroleiphilum]_gi|124261892|gb|ABM96881.1|adeninespecifi/      ------------------------------------------------------------
gi|769978537|ref|WP_045094522.1|damageinducibleprotein[Legionellafallonii]_gi|698841736|emb|CEG55682.1|Putativeproteinwithboxh/      ------------------------------------------------------------
gi|553752541|ref|WP_023085344.1|hypotheticalprotein[Pseudomonasaeruginosa]_gi|552201952|gb|ERU62653.1|hypotheticalproteinQ088_0      ------------------------------------------------------------
gi|880980418|ref|WP_048704461.1|damageinducibleprotein[Thauerahumireducens]_gi|875132785|gb|KMU70944.1|damageinducibleprotein/1      ------------------------------------------------------------
gi|740135732|ref|WP_037982815.1|damageinducibleprotein[Thauerasp.27]/11603                                                            ------------------------------------------------------------
gi|738017371|ref|WP_035978332.1|damageinducibleprotein[Burkholderiaglumae]/11380                                                      ------------------------------------------------------------
gi|498506003|ref|WP_010806671.1|typeIIIrestrictionenzyme,ressubunit[Pandoraeasp.SD62]_gi|490314561|gb|EON12879.1|typeIIIrestri/      ------------------------------------------------------------
gi|500117748|ref|WP_011793753.1|DNAdamageinducibleprotein[Acidovoraxcitrulli]_gi|120587742|gb|ABM31182.1|typeIIIrestrictionenz/      ------------------------------------------------------------
gi|651617328|ref|WP_026611444.1|damageinducibleprotein[Methylocaldumszegediense]/11601                                                ------------------------------------------------------------
gi|737210208|ref|WP_035194994.1|damageinducibleprotein[Acidithiobacillusferrivorans]_gi|669953220|emb|CDQ11755.1|Adeninespecif/      ------------------------------------------------------------
gi|935697386|ref|WP_054506180.1|damageinducibleprotein[Achromobactersp.]_gi|928716153|emb|CUK12836.1|Hefnuclease[Achromobacter/      ------------------------------------------------------------
gi|429502517|gb|ELA00827.1|adeninespecificDNAmethyltransferase[Cupriavidussp.HMR1]/11599                                              ------------------------------------------------------------
gi|506356642|ref|WP_015876361.1|DNAdamageinducibleprotein[Burkholderiaglumae]_gi|237877158|gb|ACR29491.1|AdeninespecificDNAmet/      ------------------------------------------------------------
gi|696406787|ref|WP_032978003.1|damageinducibleprotein[Bordetellahinzii]_gi|625335076|gb|KCB49343.1|typeIIIrestrictionenzyme,r/      ------------------------------------------------------------
gi|817529125|gb|KKO72587.1|damageinducibleprotein[Kerstersiagyiorum]/11592                                                            ------------------------------------------------------------
gi|737048910|ref|WP_035044118.1|damageinducibleprotein[Aquabacteriumsp.NJ1]_gi|699052896|gb|KGM38706.1|damageinducibleprotein/1      ------------------------------------------------------------
gi|822492519|ref|WP_046933319.1|MULTISPECIES:damageinducibleprotein[Xanthomonas]_gi|821703525|gb|KLA61196.1|damageinduciblepr/1      ------------------------------------------------------------
gi|751252861|ref|WP_040963740.1|damageinducibleprotein[Xanthomonasfuscans]_gi|749808363|gb|KIJ03887.1|damageinducibleprotein[/1      ------------------------------------------------------------
```

```
gi|496774980|ref|WP_009368792.1|DNAdamageinducibleprotein[Bilophilasp.4_1_30]_gi|345039902|gb|EGW44205.1|hypotheticalproteinHM/    ------------------------------------------------------------
gi|499241912|ref|WP_010939452.1|DNAdamageinducibleprotein[Desulfovibriovulgaris]_gi|46580580|ref|YP_011388.1|adeninespecificDN/    ------------------------------------------------------------
gi|504274913|ref|WP_014462015.1|DNAdamageinducibleprotein[Bacillusmegaterium]_gi|345446919|gb|AEN91936.1|AdeninespecificDNAmet/    ------------------------------------------------------------
gi|504895966|ref|WP_015083068.1|typeIIrestrictionenzymeandmethyltransferasesubtypeG[Anabaenasp.90]_gi|413972424|gb|AFW96512.1|t    ------------------------------------------------------------
gi|518327765|ref|WP_019497972.1|hypotheticalprotein[Calothrixsp.PCC7103]/11600    ------------------------------------------------------------
gi|652765503|ref|WP_027079917.1|damageinducibleprotein[Luteimonasmephitis]/11593    ------------------------------------------------------------
gi|896539951|ref|WP_049442123.1|damageinducibleprotein[Stenotrophomonasmaltophilia]/11591    ------------------------------------------------------------
gi|500217900|ref|WP_011888043.1|DNAdamageinducibleprotein[Geobacillusthermodenitrificans]_gi|134267954|gb|ABO68149.1|Conserved/    ------------------------------------------------------------
gi|696570298|ref|WP_033102014.1|damageinducibleprotein[Thermoactinomycesdaqus]/11594    ------------------------------------------------------------
gi|251772067|gb|EES52637.1|putativehelicase[Leptospirillumferrodiazotrophum]/11618    ------------------------------------------------------------
gi|738124101|ref|WP_036082108.1|damageinducibleprotein[Leptospirillumferriphilum]_gi|685243306|gb|KGA94177.1|putativehelicase[/    ------------------------------------------------------------
gi|558699223|ref|WP_023524766.1|helicase[Leptospirillumsp.GroupII'CF1']_gi|206603764|gb|EDZ40244.1|Putativehelicase[Leptospiri/    ------------------------------------------------------------
gi|406774462|gb|AFS53887.1|putativehelicase[LeptospirillumferriphilumML04]/11610    ------------------------------------------------------------
gi|740719463|ref|WP_038504749.1|damageinducibleprotein[Leptospirillumferriphilum]_gi|640838125|gb|AIA30173.1|damageinduciblep/1    ------------------------------------------------------------
gi|517810766|ref|WP_018980974.1|hypotheticalprotein[Saccharibacterfloricola]/11733    ------------------------------------------------------------
gi|502471675|ref|WP_012798533.1|helicase[Slackiaheliotrinireducens]_gi|256791761|gb|ACV22431.1|predictedhelicase[Slackiaheliotr    MASVTHITSRAHQPRGGYINLERFDEHAFEDGLPFHGEYSIVPGTLGTTVDYLSRALLGF
gi|657871713|ref|WP_029576297.1|hypotheticalprotein[Bifidobacteriumthermacidophilum]_gi|673007521|gb|KFJ02535.1|helicase[Bifido    ------------------------------------------------------------
gi|673004757|gb|KFI99791.1|DNAhelicaserestrictionenzymetypeIIIRessubunit[Bifidobacteriumthermacidophilumsubsp.porcinum]/11682    ------------------------------------------------------------
gi|917316092|ref|WP_051922804.1|hypotheticalprotein[Bifidobacteriumstellenboschense]_gi|673003774|gb|KFI98812.1|helicase[Bifido    ------------------------------------------------------------
gi|705415694|ref|WP_033502332.1|hypotheticalprotein[Bifidobacteriumpseudolongum]_gi|672982411|gb|KFI77685.1|DNAdamageinducible/    ------------------------------------------------------------
gi|916591418|ref|WP_051198509.1|hypotheticalprotein[Bifidobacteriumsp.AGR2158]/11704    ------------------------------------------------------------
gi|772242982|gb|AJW78255.1|hypotheticalproteinVO01_03165[Clavibactermichiganensissubsp.insidiosus]/11642    ------------------------------------------------------------
gi|916649818|ref|WP_051256909.1|damageinducibleprotein[Actinomycesgraevenitzii]/11677    ------------------------------------------------------------
gi|496504578|ref|WP_009212861.1|helicase[Actinomycessp.oraltaxon180]_gi|315312695|gb|EFU60776.1|helicase[Actinomycessp.oraltaxo    ------------------------------------------------------------
gi|494862206|ref|WP_007588306.1|helicase[Actinomycessp.ICM39]_gi|398362799|gb|EJN46470.1|typeIIrestrictionenzyme,ressubunit[Ac    ------------------------------------------------------------
gi|881057111|ref|WP_048774974.1|damageinducibleprotein[Sanguibacterkeddieii]/11697    ------------------------------------------------------------
gi|881020459|ref|WP_048740808.1|damageinducibleprotein[Kytococcussedentarius]/11704    ------------------------------------------------------------
gi|881052313|ref|WP_048770555.1|damageinducibleprotein[Cellulomonasflavigena]/11705    ------------------------------------------------------------
gi|490108682|ref|WP_004009424.1|helicase[Mobiluncuscurtisii]_gi|315490465|gb|EFU80089.1|helicaseCterminaldomainprotein[Mobilun/    ------------------------------------------------------------
gi|490111935|ref|WP_004012603.1|helicase[Mobiluncusmulieris]_gi|227843812|gb|EEJ53982.1|helicaseCterminaldomainprotein[Mobilun/    ------------------------------------------------------------
gi|490114228|ref|WP_004014848.1|helicase[Mobiluncusmulieris]_gi|269934432|gb|EEZ90993.1|helicaseCterminaldomainprotein[Mobilun/    ------------------------------------------------------------
gi|493333551|ref|WP_006290636.1|helicase[Parascardoviadenticolens]_gi|315119487|gb|EFT82620.1|helicaseCterminaldomainprotein[P/    ------------------------------------------------------------
gi|495817067|ref|WP_008541646.1|helicase,partial[Sutterellaparvirubra]_gi|378598583|gb|EHY31712.1|helicaseprotein,partial[Sutte    ------------------------------------------------------------
gi|491572696|ref|WP_005430278.1|helicase[Sutterellawadsworthensis]_gi|319805116|gb|EFW01943.1|DNAhelicaserestrictionenzymeTypeI    ------------------------------------------------------------
gi|748686321|ref|WP_039944445.1|hypotheticalprotein[Thermicanusaegyptius]/11573    ------------------------------------------------------------
gi|916875698|ref|WP_051482754.1|hypotheticalprotein[Thioalkalivibriosp.HK1]/11366    ------------------------------------------------------------
gi|821248604|gb|KKZ10960.1|hypotheticalproteinTE42_09075[CandidatusSynechococcusspongiarumSP3]/11598    ------------------------------------------------------------
gi|737409843|ref|WP_035390956.1|hypotheticalprotein[Ferrimicrobiumacidiphilum]_gi|765544266|gb|KJE76241.1|typeIrestrictionenzym    ------------------------------------------------------------
gi|654906925|ref|WP_028358281.1|damageinducibleprotein[Brackiellaoedipodis]/11679    ------------------------------------------------------------
gi|502809901|ref|WP_013044877.1|hypotheticalprotein[CandidatusPuniceispirillummarinum]_gi|292663146|gb|ADE38247.1|hypotheticalp    ------------------------------------------------------------
gi|251771370|gb|EES51950.1|typeIIrestrictionenzyme,ressubunit[Leptospirillumferrodiazotrophum]/11628    ------------------------------------------------------------
gi|498328480|ref|WP_010642636.1|DNAdamageinducibleprotein[Acidithiobacillusthiooxidans]/11627    ------------------------------------------------------------
gi|647633864|ref|WP_025906698.1|damageinducibleprotein[Prochlorococcussp.scB245a_519B7]/11622    ------------------------------------------------------------
gi|647619599|ref|WP_025893729.1|damageinducibleprotein[Prochlorococcussp.scB241_527N11]/11621    ------------------------------------------------------------
gi|755034887|ref|WP_042387498.1|hypotheticalprotein[Clostridiumsp.CL6]/11606    ------------------------------------------------------------
gi|498139798|ref|WP_010453954.1|helicase[SuccinivibrionaceaebacteriumWG1]/11662    ------------------------------------------------------------
gi|527501386|gb|EPT34571.1|typeIIrestrictionenzyme,ressubunit[Bacteroidetesbacteriumoraltaxon272str.F0290]/11789    ------------------------------------------------------------
gi|496047275|ref|WP_008771782.1|helicase[Bacteroidessp.2_1_33B]_gi|262296443|gb|EEY84373.1|helicaseCterminaldomainprotein[Bact/    ------------------------------------------------------------
PDI8503III/11664    ------------------------------------------------------------
gi|738719431|ref|WP_036613715.1|helicase[Parabacteroidesdistasonis]_gi|649542518|gb|KDS49387.1|restrictionendonucleasefamilypro    ------------------------------------------------------------
gi|659417033|gb|KEJ86133.1|hypotheticalproteinHMPREF1002_00341[Porphyromonassp.31_2]/11656    ------------------------------------------------------------
gi|567456807|gb|ETK11358.1|hypotheticalproteinT235_15845[Tannerellasp.oraltaxonBU063isolateCell8/11]/11433    ------------------------------------------------------------
gi|545440279|ref|WP_021677877.1|helicase[Porphyromonasgingivalis]_gi|543985765|gb|ERJ89108.1|helicaseprotein[Porphyromonasgingi    ------------------------------------------------------------
gi|497438056|ref|WP_009752254.1|helicase[Prevotellasp.oraltaxon473]_gi|429144866|gb|EKX87968.1|helicaseprotein[Prevotellasp.ora    ------------------------------------------------------------
gi|490423474|ref|WP_004295683.1|MULTISPECIES:helicase[Bacteroides]_gi|156110098|gb|EDO11843.1|helicaseCterminaldomainprotein[B/    ------------------------------------------------------------
gi|941777229|ref|WP_055173506.1|helicase[Bacteroidescaccae]_gi|933263395|emb|CUQ00567.1|Predictedhelicase[Bacteroidescaccae]/1/    ------------------------------------------------------------
gi|492413898|ref|WP_005836892.1|helicase[Bacteroidesuniformis]_gi|392678824|gb|EIY72229.1|hypotheticalproteinHMPREF1072_03583[B    ------------------------------------------------------------
gi|695455095|ref|WP_032566191.1|helicase[Bacteroidesfragilis]_gi|596095426|gb|EXZ59996.1|restrictionendonucleasefamilyprotein[B    ------------------------------------------------------------
gi|933227941|emb|CUQ02348.1|Predictedhelicase[Bacteroidesuniformis]/11658    ------------------------------------------------------------
gi|494823013|ref|WP_007558421.1|helicase[Bacteroidesplebeius]_gi|198272167|gb|EDY96436.1|helicaseCterminaldomainprotein[Bacter/    ------------------------------------------------------------
gi|495427212|ref|WP_008151909.1|helicase[Parabacteroidesjohnsonii]_gi|218222381|gb|EEC95031.1|helicaseCterminaldomainprotein[P/    ------------------------------------------------------------
gi|933196855|emb|CUO94545.1|typeIrestrictionenzymeEcoKIsubunitR[Bacteroidesuniformis]_gi|933220098|emb|CUN86874.1|typeIrestrict    ------------------------------------------------------------
gi|823687748|ref|WP_047101121.1|helicase[Brachyspirahyodysenteriae]_gi|823334537|gb|KLI17932.1|helicase[Brachyspirahyodysenteri    ------------------------------------------------------------
gi|496436691|ref|WP_009145538.1|helicase[Phascolarctobacteriumsuccinatutens]_gi|322414007|gb|EFY04839.1|helicaseCterminaldomai/    ------------------------------------------------------------
gi|503372880|ref|WP_013607541.1|helicase[Sphaerochaetaglobosa]_gi|324026933|gb|ADY13692.1|typeIIrestrictionproteinressubunit[S    ------------------------------------------------------------
gi|505821748|ref|WP_015707145.1|helicase[Treponemaprimitia]_gi|299483503|gb|ADJ19584.1|helicasedomaincontainingprotein[Trepone/    ------------------------------------------------------------
gi|505822741|ref|WP_015708134.1|helicase[Treponemaprimitia]_gi|333739639|gb|AEF85129.1|endonucleaseandmethylaseLlaGI[Treponemap    ------------------------------------------------------------
```

gi|488791994|ref|WP_002704400.1|helicase[Treponemasaccharophilum]_gi|380767691|gb|EIC01689.1|typeIIIrestrictionproteinressubuni --
gi|524671454|emb|CDD81750.1|putativeseptumsitedeterminingproteinMinC[Bacteroidessp.CAG:462]/11642 --
gi|524573865|emb|CDC95766.1|putativeseptumsitedeterminingproteinMinC[Alistipessp.CAG:268]/11639 --
gi|658617970|ref|WP_029759328.1|helicase[Fusobacteriumnucleatum]/11606 --
gi|494412037|ref|WP_007216782.1|helicase[Bacteroidescellulosilyticus]_gi|392638921|gb|EIY32752.1|hypotheticalproteinHMPREF1062_ --
gi|383107102|gb|AFG37435.1|putativehelicase[SpirochaetaafricanaDSM8902]/11616 --
SAF8902III/11616 --
gi|652538894|ref|WP_026932796.1|helicase[Gramellaechinicola]/11633 --
gi|639238223|ref|WP_024568839.1|helicase[Elizabethkingiaanophelis]/11628 --
gi|652520381|ref|WP_026914582.1|helicase[Gramellaportivictoriae]/11623 --
gi|494922183|ref|WP_007648219.1|Helicase[Formosasp.AK20]_gi|469825489|gb|EMQ95550.1|Helicasedomainprotein[Formosasp.AK20]/11626 --
gi|522083382|ref|WP_020594591.1|hypotheticalprotein[Spirosomapanaciterrae]/11637 --
gi|490455352|ref|WP_004326157.1|helicase[Bacteroidesovatus]_gi|392669277|gb|EIY62768.1|hypotheticalproteinHMPREF1070_03635[Bact --
gi|499516035|ref|WP_011202675.1|helicase[Bacteroidesfragilis]_gi|53713270|ref|YP_099262.1|helicasedomainprotein[Bacteroidesfrag --
gi|651338332|ref|WP_026461320.1|helicase[Adhaeribacteraquaticus]/11620 --
gi|806964642|gb|AKC17139.1|helicase[Flavobacteriumpsychrophilum]/11639 --
gi|752684374|ref|WP_041332322.1|helicase[Flavobacteriumpsychrophilum]_gi|666882889|gb|AIG30715.1|helicase[Flavobacteriumpsychro --
gi|933196595|emb|CUP04894.1|DNAorRNAhelicasesofsuperfamilyII[Bacteroidesuniformis]/11631 --
gi|670509109|ref|WP_031456918.1|helicase[Flavobacteriumchungangense]/11625 --

gi|490190857|ref|WP_004089451.1|hypotheticalprotein[Helicobacterbilis]_gi|476630638|gb|EMZ37196.1|hypotheticalproteinC826_02193 --
gi|488896133|ref|WP_002807242.1|MULTISPECIES:restrictionendonucleaseEco57I[Campylobacter]_gi|380541096|gb|EIA65377.1|hypothetic --
gi|746602240|ref|WP_039628365.1|restrictionendonucleaseEco57I[Campylobacterlari]_gi|744819395|gb|AJD03391.1|putativehelicase[Ca --
gi|727317135|ref|WP_033772558.1|restrictionendonucleaseEco57I[Helicobacterpylori]/11622 --
gi|727103490|ref|WP_033618954.1|restrictionendonucleaseEco57I[Helicobacterpylori]/11616 --
gi|727340205|ref|WP_033794408.1|restrictionendonucleaseEco57I[Helicobacterpylori]/11609 --
gi|727292407|ref|WP_033749975.1|restrictionendonucleaseEco57I[Helicobacterpylori]/11611 --
gi|727289795|ref|WP_033747591.1|restrictionendonucleaseEco57I[Helicobacterpylori]/11612 --
gi|727319676|ref|WP_033775047.1|restrictionendonucleaseEco57I[Helicobacterpylori]/11612 --
gi|447212910|ref|WP_001290166.1|restrictionendonucleaseEco57I[Helicobacterpylori]_gi|308063540|gb|ADO05427.1|hypotheticalprotei --
gi|447212901|ref|WP_001290157.1|restrictionendonucleaseEco57I[Helicobacterpylori]_gi|384557329|gb|AFH97797.1|hypotheticalprotei --
gi|515849671|ref|WP_017280424.1|hypotheticalprotein[Helicobacterpylori]/11615 --
gi|447212900|ref|WP_001290156.1|restrictionendonucleaseEco57I[Helicobacterpylori]_gi|317178970|dbj|BAJ56758.1|TypeIIGrestrictio --
gi|447079018|ref|WP_001156274.1|hypotheticalprotein[Helicobacterpylori]/11614 --
gi|447212800|ref|WP_001290056.1|hypotheticalprotein[Helicobacterpylori]_gi|532153248|gb|EQL67316.1|restrictionendonucleaseEco57 --
gi|505457237|ref|WP_015644339.1|hypotheticalprotein[Helicobacterpylori]_gi|499061160|gb|AGL70074.1|restrictionendonucleaseEco57 --
HPYUM037X/11591 --
gi|920026297|ref|WP_052937176.1|restrictionendonuclease[Helicobacterpylori]_gi|912528678|gb|KNX48238.1|restrictionendonucleaseE --
gi|915539050|ref|WP_050841327.1|restrictionendonuclease[Helicobacterpylori]_gi|908890887|gb|KNE06183.1|restrictionendonucleaseE --
gi|727101397|ref|WP_033616957.1|restrictionendonucleaseEco57I[Helicobacterpylori]/11592 --
gi|562814875|emb|CDK13002.1|TypeISPRMsystem[Helicobacterpylori26695]/11588 --
HPYAXVIII/11588 --
gi|447079029|ref|WP_001156285.1|restrictionendonucleaseEco57I[Helicobacterpylori]_gi|393085956|gb|EJB86635.1|hypotheticalprotei --
gi|489105953|ref|WP_003015814.1|restrictionendonuclease[Helicobacterpylori]_gi|477503388|gb|ENH59950.1|restrictionendonucleasef --
gi|447079006|ref|WP_001156262.1|restrictionendonucleaseEco57I[Helicobacterpylori]_gi|393067470|gb|EJB68280.1|hypotheticalprotei --
gi|727314589|ref|WP_033770298.1|restrictionendonucleaseEco57I[Helicobacterpylori]/11586 --
gi|447079019|ref|WP_001156275.1|restrictionendonucleaseEco57I[Helicobacterpylori]_gi|308065028|gb|ADO06920.1|hypotheticalprotei --
gi|447079028|ref|WP_001156284.1|restrictionendonucleaseEco57I[Helicobacterpylori]_gi|393076030|gb|EJB76784.1|hypotheticalprotei --
gi|393034009|gb|EJB35070.1|hypotheticalproteinHPNQ4053_0631[HelicobacterpyloriNQ4053]/11514 --
gi|447079004|ref|WP_001156260.1|restrictionendonucleaseEco57I[Helicobacterpylori]_gi|384552418|gb|AFI07366.1|hypotheticalprotei --
gi|746011306|ref|WP_039088215.1|restrictionendonucleaseEco57I[Helicobacterpylori]_gi|733488720|gb|KHL85979.1|restrictionendonuc --
gi|531081162|gb|EQD92166.1|restrictionendonucleaseEco57I[HelicobacterpyloriPZ5080]/11509 --
gi|544760796|ref|WP_021187395.1|restrictionendonucleaseEco57I[Helicobacterpylori]_gi|531086508|gb|EQD96745.1|restrictionendonuc --
gi|447079012|ref|WP_001156268.1|restrictionendonucleaseEco57I[Helicobacterpylori]_gi|393028199|gb|EJB29286.1|hypotheticalprotei --
gi|746008458|ref|WP_039085555.1|restrictionendonucleaseEco57I[Helicobacterpylori]_gi|733480358|gb|KHL78026.1|restrictionendonuc --
gi|447079011|ref|WP_001156267.1|restrictionendonucleaseEco57I[Helicobacterpylori]_gi|393038534|gb|EJB39568.1|hypotheticalprotei --
gi|393037163|gb|EJB38199.1|hypotheticalproteinHPNQ4161_0422[HelicobacterpyloriNQ4161]/11556 --
gi|447079007|ref|WP_001156263.1|restrictionendonucleaseEco57I[Helicobacterpylori]_gi|393051817|gb|EJB52768.1|hypotheticalprotei --
gi|487867094|ref|WP_001940560.1|helicase[Helicobacterpylori]_gi|459505657|gb|EMG98418.1|helicaseprotein[HelicobacterpyloriGAM21 --
gi|487872094|ref|WP_001945560.1|helicase[Helicobacterpylori]_gi|459512104|gb|EMH04523.1|helicaseprotein[HelicobacterpyloriGAM24 --
gi|487880228|ref|WP_001953694.1|helicase[Helicobacterpylori]_gi|459529131|gb|EMH20734.1|helicaseprotein[HelicobacterpyloriGAM26 --
gi|487882593|ref|WP_001956059.1|helicase[Helicobacterpylori]_gi|459533462|gb|EMH24859.1|helicaseprotein[HelicobacterpyloriGAM26 --
gi|487845430|ref|WP_001918896.1|helicase[Helicobacterpylori]_gi|410713871|gb|EKQ71363.1|helicaseprotein[HelicobacterpyloriGAM10 --
gi|447079005|ref|WP_001156261.1|restrictionendonucleaseEco57I[Helicobacterpylori]_gi|393069830|gb|EJB70625.1|hypotheticalprotei --
gi|459506808|gb|EMG99528.1|helicaseprotein[HelicobacterpyloriGAM121Aii]/11545 --
gi|487825283|ref|WP_001898749.1|restrictionendonucleaseEco57I[Helicobacterpylori]_gi|393107526|gb|EJC08068.1|hypotheticalprotei --

gi|447079008|ref|WP_001156264.1|restrictionendonucleaseEco57I[Helicobacterpylori]_gi|393058636|gb|EJB59524.1|hypotheticalprotei --
gi|447079015|ref|WP_001156271.1|restrictionendonucleaseEco57I[Helicobacterpylori]_gi|307637357|gb|ADN79807.1|hypotheticalprotei --
gi|487884861|ref|WP_001958327.1|helicase[Helicobacterpylori]_gi|459535535|gb|EMH26834.1|helicaseprotein[HelicobacterpyloriGAM26 --
gi|487886365|ref|WP_001959831.1|helicase[Helicobacterpylori]_gi|459538727|gb|EMH29861.1|helicaseprotein[HelicobacterpyloriGAM27 --
gi|545301637|ref|WP_021581412.1|helicase[Helicobacterpylori]_gi|535931889|gb|ERA61745.1|helicaseprotein[HelicobacterpyloriGAM11 --
gi|447079010|ref|WP_001156266.1|restrictionendonucleaseEco57I[Helicobacterpylori]_gi|393095477|gb|EJB96081.1|hypotheticalprotei --
gi|447079009|ref|WP_001156265.1|restrictionendonucleaseEco57I[Helicobacterpylori]_gi|393056294|gb|EJB57206.1|hypotheticalprotei --
gi|471386985|gb|EMR58906.1|restrictionendonucleasefamilyprotein[HelicobacterpyloriHpH1]/11504 --
gi|487875679|ref|WP_001949145.1|helicase[Helicobacterpylori]_gi|459516928|gb|EMH09091.1|helicaseprotein[HelicobacterpyloriGAM25 --
gi|496130066|ref|WP_008854573.1|DNAdamageinducibleprotein[Commensalibacterintestini]_gi|353671689|gb|EHD13391.1|helicasedomain/ --
gi|924898894|ref|WP_053556546.1|damageinducibleprotein[Sphingopyxissp.113P3]_gi|924523996|gb|ALC14732.1|damageinducibleprotei/1 --
gi|780802950|gb|KJS08562.1|damageinducibleprotein[Hoefleasp.BRH_c9]/11633 --
gi|944671432|gb|KQN22886.1|damageinducibleprotein[Sphingomonassp.Leaf32]/11415 --
gi|944612953|gb|KQM64772.1|damageinducibleprotein[Sphingomonassp.Leaf16]/11726 --
gi|764353851|ref|WP_044331468.1|damageinducibleprotein[Sphingomonashengshuiensis]_gi|761897338|gb|AJP71707.1|damageinduciblep/1 --
gi|945874472|gb|KQZ27973.1|damageinducibleprotein[Mesorhizobiumsp.Root552]/11651 --
gi|759408081|ref|WP_043132403.1|damageinducibleprotein[Paracoccussanguinis]_gi|694217086|gb|KGJ14281.1|damageinducibleprotein/1 --
gi|654342671|ref|WP_027836116.1|damageinducibleprotein[Maritaleamyrionectae]/11632 --
gi|126717687|gb|EBA14409.1|hypotheticalproteinRSK20926_02032[Roseobactersp.SK20926]/11603 --
gi|765301116|ref|WP_044628780.1|damageinducibleprotein[Halomonasmeridiana]_gi|764089679|gb|KJD19875.1|damageinducibleprotein[/1 --
gi|518053006|ref|WP_019223214.1|DNAdamageinducibleprotein[Bartonellarattaustraliani]/11654 --
gi|493637806|ref|WP_006589578.1|DNAdamageinducibleprotein[Bartonellabirtlesii]_gi|395410345|gb|EJF76900.1|hypotheticalproteinM/ --
gi|501188603|ref|WP_012231622.1|DNAdamageinducibleprotein[Bartonellatribocorum]_gi|161017843|emb|CAK01401.1|helicase/methyltra/ --
gi|651388616|ref|WP_026500615.1|damageinducibleprotein[Bartonellagrahamii]/11655 --
gi|501188669|ref|WP_012231687.1|DNAdamageinducibleprotein[Bartonellatribocorum]_gi|161017922|emb|CAK01480.1|helicase/methyltra/ --
gi|518048832|ref|WP_019219040.1|DNAdamageinducibleprotein[Bartonellaflorencae]/11655 --
gi|518053617|ref|WP_019223825.1|DNAdamageinducibleprotein[Bartonellarattaustraliani]/11607 --
gi|506336449|ref|WP_015856168.1|DNAdamageinducibleprotein[Bartonellagrahamii]_gi|240267492|gb|ACS51080.1|helicase/methyltransf/ --
gi|494588832|ref|WP_007347427.1|DNAdamageinducibleprotein[Bartonellarattimassiliensis]_gi|395419053|gb|EJF85361.1|hypothetical/ --
gi|501188650|ref|WP_012231668.1|DNAdamageinducibleprotein[Bartonellatribocorum]_gi|161017902|emb|CAK01460.1|helicase/methyltra/ --
gi|501188624|ref|WP_012231643.1|DNAdamageinducibleprotein[Bartonellatribocorum]_gi|161017866|emb|CAK01424.1|helicase/methyltra/ --
gi|518053856|ref|WP_019224064.1|DNAdamageinducibleprotein[Bartonellarattaustraliani]/11625 --
gi|501188053|ref|WP_012231081.1|DNAdamageinducibleprotein[Bartonellatribocorum]_gi|161017434|emb|CAK00992.1|helicase/methyltra/ --
gi|651388630|ref|WP_026500626.1|damageinducibleprotein[Bartonellagrahamii]/11657 --
gi|518048855|ref|WP_019219063.1|DNAdamageinducibleprotein[Bartonellaflorencae]/11658 --
gi|490995101|ref|WP_004856829.1|DNAdamageinducibleprotein[Bartonelladoshiae]_gi|395413167|gb|EJF79645.1|hypotheticalproteinMCS/ --
gi|491002558|ref|WP_004864279.1|DNAdamageinducibleprotein[Bartonellavinsonii]_gi|395421392|gb|EJF87643.1|hypotheticalproteinME/ --
gi|491003373|ref|WP_004865094.1|DNAdamageinducibleprotein[Bartonellavinsonii]_gi|395432864|gb|EJF98838.1|hypotheticalproteinME/ --
gi|491002426|ref|WP_004864148.1|DNAdamageinducibleprotein[Bartonellavinsonii]_gi|395421767|gb|EJF88003.1|hypotheticalproteinME/ --
gi|491004024|ref|WP_004865745.1|DNAdamageinducibleprotein[Bartonellavinsonii]_gi|395432542|gb|EJF98522.1|hypotheticalproteinME/ --
gi|648396442|ref|WP_026088193.1|damageinducibleprotein[Bartonellarattaustraliani]/11593 --
gi|748190174|ref|WP_039761290.1|damageinducibleprotein[Bartonellaqueenslandensis]/11658 --
gi|494817599|ref|WP_007553007.1|DNAdamageinducibleprotein[Bartonellasp.DB56]_gi|395411312|gb|EJF77837.1|hypotheticalproteinMC/1 --
gi|515763376|ref|WP_017195976.1|DNAdamageinducibleprotein[Bartonellabirtlesii]/11656 --
gi|494589420|ref|WP_007348014.1|DNAdamageinducibleprotein[Bartonellarattimassiliensis]_gi|395416278|gb|EJF82667.1|hypothetical/ --
gi|506336469|ref|WP_015856188.1|DNAdamageinducibleprotein[Bartonellagrahamii]_gi|240267512|gb|ACS51100.1|helicase/methyltransf/ --
gi|749308087|ref|WP_040132227.1|damageinducibleprotein[Bartonellatribocorum]_gi|605051878|emb|CDO49988.1|helicase/methyltransf/ --
gi|515763800|ref|WP_017196400.1|DNAdamageinducibleprotein[Bartonellabirtlesii]/11658 --
gi|492186994|ref|WP_005773380.1|DNAdamageinducibleprotein[Bartonellaelizabethae]_gi|395418361|gb|EJF84688.1|hypotheticalprotei/ --
gi|501188615|ref|WP_012231634.1|DNAdamageinducibleprotein[Bartonellatribocorum]_gi|161017856|emb|CAK01414.1|helicase/methyltra/ --
gi|501187470|ref|WP_012230499.1|DNAdamageinducibleprotein[Bartonellatribocorum]_gi|161017090|emb|CAK00648.1|helicase/methyltra/ --
gi|501188636|ref|WP_012231655.1|DNAdamageinducibleprotein[Bartonellatribocorum]_gi|161017879|emb|CAK01437.1|helicase/methyltra/ --
gi|930151941|emb|CTQ34818.1|UvsWhelicase[Jannaschiarubra]/11641 --
gi|928977106|ref|WP_054001461.1|damageinducibleprotein[Shimiasp.SK013]_gi|927698852|gb|KPA22698.1|typeIrestrictionenzymeEcoKIs/ --
gi|738872465|ref|WP_036760884.1|damageinducibleprotein[Leisingeradaeponensis]/11643 --
gi|518050881|ref|WP_019221089.1|DNAdamageinducibleprotein[Bartonellasenegalensis]/11652 --
gi|493980596|ref|WP_006923523.1|DNAdamageinducibleprotein[Bartonellawashoensis]_gi|395413322|gb|EJF79796.1|hypotheticalprotein/ --
gi|493983106|ref|WP_006925984.1|DNAdamageinducibleprotein[Bartonellawashoensis]_gi|395418127|gb|EJF84455.1|hypotheticalprotein/ --
gi|499494671|ref|WP_011181311.1|DNAdamageinducibleprotein[Bartonellahenselae]_gi|49239008|emb|CAF28308.1|hypotheticalproteinBH/ --
gi|651388426|ref|WP_026500441.1|damageinducibleprotein[Bartonellagrahamii]/11652 --
gi|506337280|ref|WP_015856999.1|DNAdamageinducibleprotein[Bartonellagrahamii]_gi|240268402|gb|ACS51990.1|helicase/methyltransf/ --
gi|494817535|ref|WP_007552943.1|DNAdamageinducibleprotein[Bartonellasp.DB56]_gi|395412002|gb|EJF78518.1|hypotheticalproteinMC/1 --
gi|493638099|ref|WP_006589864.1|DNAdamageinducibleprotein[Bartonellabirtlesii]_gi|395409809|gb|EJF76394.1|hypotheticalproteinM/ --
gi|748189589|ref|WP_039760707.1|damageinducibleprotein[Bartonellaqueenslandensis]/11651 --
gi|501189483|ref|WP_012232501.1|DNAdamageinducibleprotein[Bartonellatribocorum]_gi|161018902|emb|CAK02460.1|helicase/methyltra/ --
gi|518049181|ref|WP_019219389.1|DNAdamageinducibleprotein[Bartonellaflorencae]/11652 --
gi|492193255|ref|WP_005774528.1|DNAdamageinducibleprotein[Bartonellaelizabethae]_gi|395416403|gb|EJF82779.1|hypotheticalprotei/ --

```
gi|498387325|ref|WP_010701288.1|helicase/methyltransferase[Bartonellabovis]_gi|478418724|gb|ENN92426.1|helicase/methyltransfera      ------------------------------------------------------------
gi|319408697|emb|CBI82352.1|conservedhypotheticalprotein[BartonellaschoenbuchensisR1]/11661                                           ------------------------------------------------------------
gi|494741726|ref|WP_007477134.1|DNAdamageinducibleprotein[Bartonellamelophagi]_gi|395423925|gb|EJF90113.1|hypotheticalproteinM/      ------------------------------------------------------------
gi|737261405|ref|WP_035245349.1|damageinducibleprotein[Alcanivoraxjadensis]_gi|686989709|gb|KGD62556.1|hypotheticalproteinT9A_/      ------------------------------------------------------------
gi|916687564|ref|WP_051294655.1|damageinducibleprotein[Gemmobacternectariphilus]/11641                                                ------------------------------------------------------------
gi|740294127|ref|WP_038132495.1|damageinducibleprotein[Thioclavasp.DT234]_gi|660524826|gb|KEO54689.1|hypotheticalproteinDT23_/1      ------------------------------------------------------------
gi|568331062|ref|WP_024099615.1|helicase[Phaeobactergallaeciensis]_gi|568232121|gb|AHD12007.1|putativehelicase[Phaeobactergalla      ------------------------------------------------------------
gi|518810459|ref|WP_019966413.1|DNAdamageinducibleprotein[Pannonibacterphragmitetus]/11635                                            ------------------------------------------------------------
gi|495305760|ref|WP_008030510.1|DNAdamageinducibleprotein[Rhodobactersp.SW2]_gi|259021698|gb|EEW25000.1|typeIIIrestrictionprot/      ------------------------------------------------------------
gi|506403303|ref|WP_015923022.1|DNAdamageinducibleprotein[Rhodobactersphaeroides]_gi|221162951|gb|ACM03922.1|HypotheticalProte/      ------------------------------------------------------------
gi|738818386|ref|WP_036708790.1|damageinducibleprotein[Paracoccussanguinis]_gi|694226786|gb|KGJ22541.1|damageinducibleprotein/1      ------------------------------------------------------------
gi|746096057|ref|WP_039162453.1|damageinducibleprotein[Gallibacteriumanatis]_gi|703617775|gb|KGQ63868.1|damageinducibleprotei/1      ------------------------------------------------------------
gi|737716785|ref|WP_035685631.1|damageinducibleprotein[Avibacteriumparagallinarum]/11643                                              ------------------------------------------------------------
gi|540072553|gb|ERF78672.1|hypotheticalproteinN561_04930[Gallibacteriumanatis12656/12}/11651                                          ------------------------------------------------------------
gi|917509519|ref|WP_052115936.1|damageinducibleprotein[Gallibacteriumanatis]_gi|702414161|gb|KGQ42233.1|damageinducibleprotei/1      ------------------------------------------------------------
gi|746082942|ref|WP_039150268.1|damageinducibleprotein[Gallibacteriumanatis]_gi|703617078|gb|KGQ63194.1|damageinducibleprotei/1      ------------------------------------------------------------
gi|822522885|ref|WP_046952840.1|damageinducibleprotein[Haemophilushaemolyticus]_gi|821501762|gb|KKZ59267.1|damageinduciblepro/1      ------------------------------------------------------------
gi|404381014|gb|EJZ77501.1|putativehelicase[Pasteurellamultocidasubsp.gallicidaP1059]/11681                                           ------------------------------------------------------------
gi|298281660|gb|EFI23149.1|helicaseCterminaldomainprotein[Neisseriasp.oraltaxon014str.F0314]/11493                                    ------------------------------------------------------------
gi|348013055|gb|EGY51980.1|helicasedomainprotein[Neisseriashayeganii871]/11481                                                        ------------------------------------------------------------
gi|496407140|ref|WP_009116004.1|DNAdamageinducibleprotein[Neisseriawadsworthii]_gi|349794756|gb|EGZ48566.1|helicasedomainprote/      ------------------------------------------------------------
gi|517216441|ref|WP_018405259.1|DNAdamageinducibleprotein[Marinobacterlipolyticus]/11656                                              ------------------------------------------------------------
gi|738230264|ref|WP_036185691.1|damageinducibleprotein[Marinobactersp.ELB17]/11671                                                   ------------------------------------------------------------
gi|447197741|ref|WP_001274997.1|DNAdamageinducibleprotein[Acinetobacterbaumannii]_gi|408512324|gb|EKK13969.1|typeIIIrestrictio/      ------------------------------------------------------------
gi|820775897|ref|WP_046739909.1|damageinducibleprotein[Acinetobactersp.AG1]_gi|819909758|gb|KKW75938.1|damageinducibleprotein/1      ------------------------------------------------------------
gi|584595163|ref|WP_024160846.1|helicase[Acinetobacterpittii]_gi|576702530|emb|CDI28049.1|helicasedomaincontainingprotein[Acin/      ------------------------------------------------------------
gi|514963042|ref|WP_016651366.1|hypotheticalprotein[Acinetobactersp.NIPH2036]_gi|514162665|gb|EPG41391.1|hypotheticalproteinF90      ------------------------------------------------------------
gi|515965564|ref|WP_017396147.1|DNAdamageinducibleprotein[Acinetobacterhaemolyticus]/11667                                            ------------------------------------------------------------
gi|491346810|ref|WP_005204747.1|MULTISPECIES:hypotheticalprotein[Acinetobacter]_gi|480335823|gb|ENX56033.1|hypotheticalproteinF      ------------------------------------------------------------
gi|693044472|ref|WP_032215268.1|damageinducibleprotein[Escherichiacoli]_gi|607725731|gb|EZE08230.1|damageinducibleprotein[Esc/1      ------------------------------------------------------------
gi|742382177|ref|WP_038861304.1|damageinducibleprotein[Cronobactermuytjensii]_gi|924333747|gb|ALB69766.1|damageinducibleprote/1      ------------------------------------------------------------
gi|915912804|ref|WP_050927035.1|damageinducibleprotein[Yersiniaenterocolitica]_gi|899805802|emb|CQH32042.1|typeIrestrictionenz/      ------------------------------------------------------------
gi|921111327|gb|KON62781.1|typeIrestrictionenzymeEcoKIsubunitR[Komagataeibactereuropaeus]/11492                                        ------------------------------------------------------------
gi|582024278|gb|AHI27615.1|DNAhelicaserestrictionenzymeTypeIIIRsubunit[KomagataeibacterxylinusE25]/11636                              ------------------------------------------------------------
gi|347662673|dbj|BAK86128.1|DNAhelicaserestrictionenzymeTypeIIIRsubunit[KomagataeibactermedellinensisNBRC3288]/11737                  ------------------------------------------------------------
gi|917445053|ref|WP_052051471.1|damageinducibleprotein[Acetobactertropicalis]/11429                                                   ------------------------------------------------------------
gi|453329445|dbj|GAC88472.1|DNAhelicaserestrictionenzymetypeIIIRsubunit[GluconobacterthailandicusNBRC3255]_gi|530287981|dbj|GAD      ------------------------------------------------------------
gi|889770922|ref|WP_048844278.1|damageinducibleprotein[Gluconobacterfrateurii]/11419                                                  ------------------------------------------------------------
gi|504886208|ref|WP_015073310.1|hypotheticalprotein[Gluconobacteroxydans]_gi|411027954|gb|AFW01209.1|hypotheticalproteinB932_16      ------------------------------------------------------------
gi|480040211|gb|ENV79751.1|hypotheticalproteinF942_01641[AcinetobacterursingiiANC3649]/11432                                          ------------------------------------------------------------
gi|822498222|ref|WP_046936504.1|damageinducibleprotein[Xanthomonasgardneri]_gi|821751224|gb|KLB07223.1|damageinducibleprotein/1      ------------------------------------------------------------
gi|654059559|ref|WP_027705556.1|damageinducibleprotein[Zymobacterpalmae]/11635                                                        ------------------------------------------------------------
gi|643535620|ref|WP_025224315.1|damageinducibleprotein[Methylophilaceaebacterium11]_gi|577704400|gb|EUJ10546.1|putativehelicas/      ------------------------------------------------------------
MBA11I/11635                                                                                                                          ------------------------------------------------------------
gi|655355857|ref|WP_028762287.1|damageinducibleprotein[Shewanellacolwelliana]/11632                                                   ------------------------------------------------------------
gi|753204236|ref|WP_041510579.1|damageinducibleprotein[Shewanellasp.cp20]_gi|752457765|gb|KIO36326.1|damageinducibleprotein[S/1      ------------------------------------------------------------
KPNNIH30III/11619                                                                                                                     ------------------------------------------------------------
gi|749295994|ref|WP_040120352.1|damageinducibleprotein[Klebsiellapneumoniae]_gi|723249300|gb|AIX81926.1|damageinducibleprotei/1      ------------------------------------------------------------
gi|641639739|emb|CDQ54010.1|unnamedproteinproduct[Klebsiellapneumoniae]/11641                                                         ------------------------------------------------------------
gi|490797922|ref|WP_004660060.1|hypotheticalprotein[Acinetobactersp.NIPH236]_gi|479876779|gb|ENU27973.1|hypotheticalproteinF992      ------------------------------------------------------------
gi|916750926|ref|WP_051357982.1|damageinducibleprotein[Acinetobactertjernbergiae]/11453                                               ------------------------------------------------------------
gi|691098662|ref|WP_032025060.1|damageinducibleprotein[Acinetobacterbaumannii]_gi|593567521|gb|EXR50735.1|restrictionendonucle/      ------------------------------------------------------------
gi|736669405|ref|WP_034676049.1|damageinducibleprotein[Acinetobacterbaumannii]/11643                                                  ------------------------------------------------------------
gi|491368928|ref|WP_005226826.1|hypotheticalprotein[Acinetobactersp.NIPH1847]_gi|480173259|gb|ENX09797.1|hypotheticalproteinF89      ------------------------------------------------------------
gi|796853993|ref|WP_045795953.1|damageinducibleprotein[Acinetobacterindicus]_gi|787929761|gb|KJV44219.1|damageinducibleprotei/1      ------------------------------------------------------------
gi|295797463|gb|ADG32253.1|typeIIIrestrictionproteinressubunit[ThiomonasintermediaK12]/11676                                          ------------------------------------------------------------
gi|499871179|ref|WP_011551913.1|DNAdamageinducibleprotein[Myxococcusxanthus]_gi|108467544|gb|ABF92729.1|putativerestriction/mo/      ------------------------------------------------------------
gi|495313852|ref|WP_008038600.1|DNAdamageinducibleprotein[Bartonellatamiae]_gi|395424181|gb|EJF90368.1|hypotheticalproteinME5_/      ------------------------------------------------------------
gi|501453335|ref|WP_012476784.1|DNAdamageinducibleprotein[Leptospirabiflexa]_gi|167777824|gb|ABZ96123.1|Helicase[Leptospirabif/      ------------------------------------------------------------
gi|738104613|ref|WP_036063193.1|damageinducibleprotein[Leptospiranoguchii]/11652                                                      ------------------------------------------------------------
gi|410001039|gb|EKO51661.1|typeIIIrestrictionenzyme,ressubunit[Leptospirakirschneristr.200802841]/11651                               ------------------------------------------------------------
gi|490901851|ref|WP_004763774.1|typeIIIrestrictionenzyme,ressubunit[Leptospirakirschneri]_gi|464209249|gb|EMN24014.1|typeIIIres      ------------------------------------------------------------
gi|464398131|gb|EMO65902.1|typeIIIrestrictionenzyme,ressubunit[Leptospirakirschneristr.200803703]/11648                               ------------------------------------------------------------
gi|945029024|gb|KQQ84900.1|damageinducibleprotein[Xanthomonassp.Leaf131]/11423                                                        ------------------------------------------------------------
gi|896511314|ref|WP_049418183.1|damageinducibleprotein[Stenotrophomonasmaltophilia]/11639                                             ------------------------------------------------------------
gi|896513082|ref|WP_049419616.1|damageinducibleprotein[Stenotrophomonasmaltophilia]/11638                                             ------------------------------------------------------------
gi|896580616|ref|WP_049469425.1|damageinducibleprotein[Stenotrophomonasmaltophilia]/11641                                             ------------------------------------------------------------
```

```
gi|648289350|ref|WP_026070499.1|damageinducibleprotein[Stenotrophomonasmaltophilia]/11639    ------------------------------------------------------------
gi|656272786|ref|WP_029218442.1|damageinducibleprotein[Xanthomonascassavae]/11639    ------------------------------------------------------------
gi|406976236|gb|EKD98747.1|hypotheticalproteinACD_23C00290G0002[unculturedbacterium]/11679    ------------------------------------------------------------
gi|828145737|ref|WP_047238120.1|damageinducibleprotein[Chromobacteriumsubtsugae]/11645    ------------------------------------------------------------
gi|654311174|ref|WP_027824327.1|damageinducibleprotein[Laribacterhongkongensis]/11641    ------------------------------------------------------------
gi|757242173|gb|KIR15516.1|typeIrestrictionenzymeEcoKIsubunitR[Pseudomonasfluorescens]/11778    ------------------------------------------------------------
gi|653605161|ref|WP_027616682.1|damageinducibleprotein[Pseudomonassp.URHB0015]/11639    ------------------------------------------------------------
gi|520868025|ref|WP_020308060.1|helicase[Pseudomonassyringae]_gi|519159034|gb|EPM49935.1|helicasedomaincontainingprotein[Pseud/    ------------------------------------------------------------
gi|940156567|gb|KPX71668.1|Helicasedomaincontainingprotein[Pseudomonasamygdalipv.lachrymans]/11677    ------------------------------------------------------------
gi|505460508|ref|WP_015647610.1|helicase[Pseudomonassp.GLE121]_gi|499110212|ref|YP_007974374.1|helicasedomaincontainingprotein/    ------------------------------------------------------------
gi|940235551|gb|KPY45311.1|hypotheticalproteinALO48_200154[Pseudomonassyringaepv.rhaphiolepidis]/11631    ------------------------------------------------------------
gi|520880808|ref|WP_020312235.1|helicase[Pseudomonassyringae]_gi|519172158|gb|EPM62027.1|helicasedomaincontainingprotein[Pseud/    ------------------------------------------------------------
gi|829046479|ref|WP_047282259.1|damageinducibleprotein[Pseudomonasfluorescens]/11630    ------------------------------------------------------------
gi|499415493|ref|WP_011102960.1|DNAdamageinducibleprotein[Pseudomonassyringaegroupgenomosp.3]_gi|28867279|ref|NP_789898.1|heli/    ------------------------------------------------------------
gi|730238299|ref|WP_033942897.1|damageinducibleprotein[Pseudomonasaeruginosa]/11629    ------------------------------------------------------------
gi|495310125|ref|WP_008034873.1|DNAdamageinducibleprotein[Pseudomonassp.GM60]_gi|398186685|gb|EJM74052.1|putativehelicase[Pseu/    ------------------------------------------------------------
gi|779992653|ref|WP_045428690.1|damageinducibleprotein[Pseudomonassp.MT1]_gi|699967872|dbj|BAP80334.1|DNAhelicaserestrictione/1    ------------------------------------------------------------
gi|786037289|ref|WP_045733274.1|damageinducibleprotein[Pseudomonaspseudoalcaligenes]_gi|782987904|gb|KJU79152.1|damageinducib/1    ------------------------------------------------------------
gi|749666366|ref|WP_040265445.1|damageinducibleprotein[Pseudomonasrhodesiae]/11632    ------------------------------------------------------------
gi|734902433|ref|WP_034133698.1|damageinducibleprotein[Pseudomonasfluorescens]/11635    ------------------------------------------------------------
gi|490551031|ref|WP_004416146.1|typeIIrestrictionmodificationsystemrestriction/helicasesubunit[Pseudomonassyringae]_gi|4432823/    ------------------------------------------------------------
gi|489199911|ref|WP_003109152.1|DNAdamageinducibleprotein[Pseudomonasaeruginosa]_gi|552648708|gb|ERY63184.1|hypotheticalprotei/    ------------------------------------------------------------
gi|730641237|ref|WP_034061328.1|damageinducibleprotein[Pseudomonasaeruginosa]/11636    ------------------------------------------------------------
gi|751304980|ref|WP_041013386.1|damageinducibleprotein[Pseudomonasxanthomarina]_gi|703407611|emb|CEG51014.1|Helicasedomaincon/1    ------------------------------------------------------------
gi|939335207|ref|WP_054881520.1|damageinducibleprotein[Pseudomonassp.NBRC111128]/11632    ------------------------------------------------------------
gi|757693555|ref|WP_042923792.1|damageinducibleprotein[Pseudomonaschloritidismutans]_gi|780910610|gb|KJS78302.1|damageinducib/1    ------------------------------------------------------------
gi|665953944|ref|WP_031319989.1|damageinducibleprotein[Pseudomonastolaasii]/11626    ------------------------------------------------------------
gi|771600573|ref|WP_045213746.1|damageinducibleprotein[Pseudomonassp.21]_gi|770081123|gb|KJJ98634.1|damageinducibleprotein[Ps/1    ------------------------------------------------------------
gi|740871396|ref|WP_038656644.1|damageinducibleprotein[Pseudomonasstutzeri]_gi|627772214|gb|AHY41437.1|damageinducibleprotein/1    ------------------------------------------------------------
gi|946242205|gb|KRC93782.1|damageinducibleprotein[Pseudomonassp.Root9]/11634    ------------------------------------------------------------
gi|553777322|ref|WP_023109107.1|MULTISPECIES:hypotheticalprotein[Pseudomonas]_gi|552638674|gb|ERY53184.1|hypotheticalproteinQ05    ------------------------------------------------------------
gi|896383370|ref|WP_049324618.1|damageinducibleprotein[Pseudomonasstutzeri]/11627    ------------------------------------------------------------
gi|696158510|gb|AIT41787.1|putativehelicase[Pseudomonassp.LM8]/11630    ------------------------------------------------------------
gi|504634892|ref|WP_014821994.1|DNAdamageinducibleprotein[Pseudomonasstutzeri]_gi|390985249|gb|AFM35242.1|helicasedomainconta/1    ------------------------------------------------------------
gi|544783974|ref|WP_021206729.1|DNAdamageinducibleprotein[Pseudomonasstutzeri]_gi|533224779|gb|EQM79688.1|DNAdamageinduciblep/1    ------------------------------------------------------------
gi|504567385|ref|WP_014754487.1|MULTISPECIES:DNAdamageinducibleprotein[Pseudomonas]_gi|388559493|gb|AFK68634.1|helicasedomain/1    ------------------------------------------------------------
gi|654441469|ref|WP_027912052.1|damageinducibleprotein[Pseudomonassp.URIL14HWK12:I7]/11630    ------------------------------------------------------------
gi|941470066|ref|WP_055127894.1|damageinducibleprotein[Pseudomonasmediterranea]/11631    ------------------------------------------------------------
gi|557815599|ref|WP_023446184.1|DNAdamageinducibleprotein[Pseudomonaschloritidismutans]_gi|557364081|gb|ESQ98190.1|DNAdamagei/1    ------------------------------------------------------------
gi|727814791|ref|WP_033898247.1|damageinducibleprotein[Pseudomonassp.FH1]_gi|567648005|gb|ETK23229.1|helicasedomaincontaining/1    ------------------------------------------------------------
gi|503278702|ref|WP_013513363.1|DNAdamageinducibleprotein[Desulfovibrioaespoeensis]_gi|316942375|gb|ADU61426.1|typeIIrestrict/    ------------------------------------------------------------
gi|505228796|ref|WP_015415898.1|typeIIIrestrictionendonucleasesubunitR[Desulfovibriopiezophilus]_gi|451896976|emb|CCH49855.1|Ty    ------------------------------------------------------------
gi|517732543|ref|WP_018902751.1|hypotheticalprotein[Variovoraxparadoxus]/11675    ------------------------------------------------------------
gi|550909475|ref|WP_022662570.1|DNAdamageinducibleprotein[Desulfovibriolongus]/11637    ------------------------------------------------------------
gi|945494542|gb|KQV50024.1|damageinducibleprotein[Massiliasp.Root335]/11647    ------------------------------------------------------------
gi|334196917|gb|AEG70102.1|helicasedomainprotein[RalstoniasolanacearumPo82]/11663    ------------------------------------------------------------
gi|656006746|ref|WP_029048170.1|damageinducibleprotein[Cupriavidussp.amp6]/11661    ------------------------------------------------------------
BPS2196ORF3054P/11659    ------------------------------------------------------------
gi|740932312|ref|WP_038717332.1|damageinducibleprotein[Burkholderiapseudomallei]_gi|760211882|gb|KIX58314.1|damageinduciblepr/1    ------------------------------------------------------------
gi|755810813|ref|WP_042583861.1|damageinducibleprotein[Burkholderiasp.MSHR3999]_gi|755174952|gb|KIP19790.1|DEAD/DEAHboxhelicas/    ------------------------------------------------------------
gi|484586570|gb|AGK48195.1|DEAD/DEAHboxhelicasefamilyprotein[BurkholderiathailandensisMSMB121]/11646    ------------------------------------------------------------
gi|737582935|ref|WP_035553941.1|damageinducibleprotein[Burkholderiasp.9120]/11658    ------------------------------------------------------------
gi|740989872|ref|WP_038773225.1|damageinducibleprotein[Burkholderiapseudomallei]_gi|705645291|gb|KGS70691.1|DEAD/DEAHboxhelica/    ------------------------------------------------------------
gi|654912525|ref|WP_028363424.1|damageinducibleprotein[Burkholderiasp.JPY366]/11659    ------------------------------------------------------------
gi|740955879|ref|WP_038740512.1|damageinducibleprotein[Burkholderiapseudomallei]_gi|714684932|gb|KGW74438.1|DEAD/DEAHboxhelica/    ------------------------------------------------------------
gi|919203714|ref|WP_052759215.1|damageinducibleprotein[Xanthomonasaxonopodis]/11388    ------------------------------------------------------------
gi|522152381|ref|WP_020663589.1|hypotheticalprotein[Amycolatopsisbenzoatilytica]/11605    ------------------------------------------------------------
gi|663196013|ref|WP_030233899.1|damageinducibleprotein[Streptomycessp.NRRLS455]/11641    ------------------------------------------------------------
gi|750429553|ref|WP_040711444.1|damageinducibleprotein[Nocardiopsispotens]/11622    ------------------------------------------------------------
gi|518656139|ref|WP_019819475.1|hypotheticalprotein[Saccharomonosporasaliphila]/11623    ------------------------------------------------------------
gi|759722933|ref|WP_043433758.1|damageinducibleprotein[Streptomycespluripotens]_gi|733404978|gb|KHK94361.1|damageinduciblepro/1    ------------------------------------------------------------
gi|663424167|ref|WP_030419030.1|damageinducibleprotein[Streptomycessp.NRRLF5065]/11627    ------------------------------------------------------------
gi|663412917|ref|WP_030408353.1|damageinducibleprotein[Streptomycessp.NRRLF5917]/11630    ------------------------------------------------------------
gi|671521483|ref|WP_031506489.1|damageinducibleprotein[Streptomycesmegasporus]/11630    ------------------------------------------------------------
gi|740053144|ref|WP_037902776.1|damageinducibleprotein[Streptomycessp.TAA204]/11626    ------------------------------------------------------------
gi|663409375|ref|WP_030404974.1|MULTISPECIES:damageinducibleprotein[Streptomyces]/11627    ------------------------------------------------------------
gi|746628608|ref|WP_039654277.1|damageinducibleprotein[Streptomycessp.MUSC125]_gi|745644381|gb|KIE25557.1|damageinducibleprot/1    ------------------------------------------------------------
```

```
gi|727322066|ref|WP_033777320.1|damageinducibleprotein[Salinisporapacifica]/11627                                                                    ------------------------------------------------------------
gi|653653242|ref|WP_027655726.1|damageinducibleprotein[Salinisporapacifica]/11618                                                                    ------------------------------------------------------------
gi|648632543|ref|WP_026324294.1|damageinducibleprotein[Salinisporapacifica]/11621                                                                    ------------------------------------------------------------
gi|881018002|ref|WP_048738463.1|damageinducibleprotein[Corynebacteriumfalsenii]/11676                                                                ------------------------------------------------------------
gi|505465526|ref|WP_015652417.1|helicase[Corynebacteriumcallunae]_gi|459385704|gb|AGG67995.1|helicase[CorynebacteriumcallunaeDS       ------------------------------------------------------------
gi|41223062|emb|CAF19007.1|DNAorRNAhelicaseofsuperfamilyII[CorynebacteriumglutamicumATCC13032]/11646                                 ------------------------------------------------------------
CGL13032I/11643                                                                                                                        ------------------------------------------------------------
gi|751257081|ref|WP_040967675.1|damageinducibleprotein[Corynebacteriumglutamicum]_gi|748811620|gb|AJE67931.1|damageinduciblep/1       ------------------------------------------------------------
gi|749040539|ref|WP_040086524.1|damageinducibleprotein[Corynebacteriumhumireducens]/11663                                             ------------------------------------------------------------
gi|491662899|ref|WP_005519615.1|helicase[Corynebacteriummatruchotii]_gi|224946756|gb|EEG27965.1|helicaseCterminaldomainprotein/       ------------------------------------------------------------
gi|746717312|ref|WP_039676231.1|damageinducibleprotein[Corynebacteriumminutissimum]_gi|734991118|gb|KHO29219.1|damageinducibl/1       ------------------------------------------------------------
gi|908654892|emb|CQD15529.1|SuperfamilyIIDNA/RNAhelicase(modularprotein)[Corynebacteriumstriatum]/11674                              ------------------------------------------------------------
gi|895994446|ref|WP_049064418.1|damageinducibleprotein[Corynebacteriumaurimucosum]/11667                                              ------------------------------------------------------------
gi|896123331|ref|WP_049145390.1|damageinducibleprotein[Corynebacteriumaurimucosum]/11669                                              ------------------------------------------------------------
gi|817681178|ref|WP_046645422.1|damageinducibleprotein[Corynebacteriumstriatum]_gi|817551679|gb|KKO79868.1|damageinduciblepro/1       ------------------------------------------------------------
gi|896139239|ref|WP_049159146.1|damageinducibleprotein[Corynebacteriumaurimucosum]/11675                                              ------------------------------------------------------------
gi|489943947|ref|WP_003847254.1|helicase[Corynebacteriumammoniagenes]_gi|295968218|gb|EFG81467.1|helicaseCterminaldomainprotei/       ------------------------------------------------------------
gi|516653464|ref|WP_018022453.1|helicase[Corynebacteriumdoosanense]_gi|697778282|gb|AIT61018.1|damageinducibleprotein[Coryneba/       ------------------------------------------------------------
gi|493894179|ref|WP_006840129.1|helicase[Corynebacteriumlipophiloflavum]_gi|227078819|gb|EEI16782.1|helicaseCterminaldomainpro/       ------------------------------------------------------------
gi|740831472|ref|WP_038616755.1|damageinducibleprotein[Corynebacteriumpseudotuberculosis]_gi|666103058|gb|AIG06935.1|putativeh/       ------------------------------------------------------------
gi|518023322|ref|WP_019193530.1|helicase[Corynebacteriumtimonense]/11655                                                              ------------------------------------------------------------
gi|552767697|ref|WP_023019559.1|MULTISPECIES:hypotheticalprotein[Corynebacterium]_gi|550739013|gb|ERS38609.1|hypotheticalprotei       ------------------------------------------------------------
gi|652659121|ref|WP_027017812.1|damageinducibleprotein[Corynebacteriumpseudodiphtheriticum]/11644                                     ------------------------------------------------------------
gi|749039309|ref|WP_040085390.1|damageinducibleprotein[Corynebacteriumhumireducens]/11657                                             ------------------------------------------------------------
gi|740835591|ref|WP_038620874.1|damageinducibleprotein[Corynebacteriumulcerans]_gi|698973854|gb|AIU33664.1|HelicaseCterminald/1       ------------------------------------------------------------
gi|805307599|ref|WP_046096452.1|damageinducibleprotein[Corynebacteriumulcerans]_gi|803357323|gb|AKA97638.1|Damageinduciblepro/1       ------------------------------------------------------------
gi|504083555|ref|WP_014317549.1|helicase[Corynebacteriumdiphtheriae]_gi|371584249|gb|AEX47914.1|superfamilyIIDNA/RNAhelicase[Co       ------------------------------------------------------------
gi|896182193|ref|WP_049193580.1|damageinducibleprotein[Corynebacteriumjeikeium]/11645                                                 ------------------------------------------------------------
gi|917636459|ref|WP_052205460.1|damageinducibleprotein[Corynebacteriumriegelii]_gi|914295811|gb|AKV59187.1|damageinduciblepro/1       ------------------------------------------------------------
gi|737013522|ref|WP_035009205.1|damageinducibleprotein[Corynebacteriumjeikeium]/11651                                                 ------------------------------------------------------------
gi|737006291|ref|WP_035002094.1|damageinducibleprotein[Corynebacteriumjeikeium]/11658                                                 ------------------------------------------------------------
gi|737010378|ref|WP_035006133.1|damageinducibleprotein[Corynebacteriumjeikeium]/11650                                                 ------------------------------------------------------------
gi|737010473|ref|WP_035006228.1|damageinducibleprotein[Corynebacteriumjeikeium]/11657                                                 ------------------------------------------------------------
gi|737006250|ref|WP_035002053.1|damageinducibleprotein[Corynebacteriumjeikeium]/11658                                                 ------------------------------------------------------------
gi|923018168|ref|WP_053412254.1|damageinducibleprotein[Corynebacteriumlactis]_gi|922845184|gb|ALA67504.1|helicase[Corynebacter/       ------------------------------------------------------------
gi|923018885|ref|WP_053412970.1|damageinducibleprotein[Corynebacteriumlactis]_gi|922845845|gb|ALA68165.1|helicase[Corynebacter/       ------------------------------------------------------------
gi|512464870|ref|WP_016422869.1|hypotheticalprotein[Corynebacteriumsp.HFH0082]_gi|512047830|gb|EPD47505.1|hypotheticalproteinHM       ------------------------------------------------------------
gi|881012697|ref|WP_048733406.1|damageinducibleprotein[Corynebacteriumurealyticum]/11670                                              ------------------------------------------------------------
gi|755608478|ref|WP_042530519.1|damageinducibleprotein[Corynebacteriumsingulare]_gi|754340239|gb|AJI78730.1|putativehelicase[C/       ------------------------------------------------------------
gi|491421796|ref|WP_005279593.1|helicase[Corynebacteriumaccolens]_gi|227077180|gb|EEI15143.1|helicaseCterminaldomainprotein[Co/       ------------------------------------------------------------
gi|550752286|gb|ERS51451.1|hypotheticalproteinHMPREF1282_00033[Corynebacteriumsp.KPL1856]_gi|550759961|gb|ERS58964.1|hypothetic       ------------------------------------------------------------
gi|497955053|ref|WP_010269209.1|helicase[Corynebacteriumbovis]/11599                                                                  ------------------------------------------------------------
gi|860580040|ref|WP_048470569.1|hypotheticalprotein[Mycobacteriumchlorophenolicum]_gi|859098575|gb|KMO76871.1|typeIrestrictione       ------------------------------------------------------------
gi|262085905|gb|ACY21873.1|typeIIIrestrictionproteinressubunit[GordoniabronchialisDSM43247]/11632                                      ------------------------------------------------------------
gi|495996338|ref|WP_008720917.1|typeIIIrestrictionendonucleasesubunitR[Rhodococcussp.AW25M09]_gi|443412350|emb|CCQ18455.1|TypeI       ------------------------------------------------------------
gi|739284636|ref|WP_037147410.1|damageinducibleprotein[Rhodococcusfascians]/11641                                                     ------------------------------------------------------------
gi|739328195|ref|WP_037190574.1|damageinducibleprotein[Rhodococcusfascians]/11643                                                     ------------------------------------------------------------
gi|811698335|emb|CMN21041.1|helicase[Mycobacteriumtuberculosis]/11725                                                                 ------------------------------------------------------------
gi|801577898|emb|CFK23773.1|helicase[Mycobacteriumtuberculosis]/11675                                                                 ------------------------------------------------------------
MTU18II/11606                                                                                                                          ------------------------------------------------------------
gi|887719858|emb|CMT27886.1|helicase[Mycobacteriumtuberculosis]/11667                                                                 ------------------------------------------------------------
gi|621694743|gb|KAS87156.1|hypotheticalproteinAL70_01801[MycobacteriumtuberculosisTKK_03_0115]/11604                                  ------------------------------------------------------------
gi|888128072|emb|CLQ84422.1|helicase[Mycobacteriumtuberculosis]_gi|889917090|emb|CLY86893.1|helicase[Mycobacteriumtuberculosis]       ------------------------------------------------------------
gi|884823424|emb|CKL96672.1|helicase[Mycobacteriumtuberculosis]/11466                                                                 ------------------------------------------------------------
gi|505103239|ref|WP_015290341.1|hypotheticalprotein[Mycobacteriumcanettii]_gi|432162733|emb|CCK60117.1|Conservedproteinofunknow       ------------------------------------------------------------
gi|505116250|ref|WP_015303352.1|hypotheticalprotein[Mycobacteriumcanettii]_gi|432158726|emb|CCK56026.1|Conservedproteinofunknow       ------------------------------------------------------------
gi|432154735|emb|CCK51974.1|Conservedproteinofunknownfunction(part1)[MycobacteriumcanettiiCIPT140060008]/11588                         ------------------------------------------------------------
gi|809008027|ref|WP_046295375.1|hypotheticalprotein[Mycobacteriumsp.UM_Kg17]/11624                                                    ------------------------------------------------------------
gi|808802633|ref|WP_046284139.1|hypotheticalprotein[Mycobacteriumsp.UM_NZ2]/11624                                                     ------------------------------------------------------------
gi|750125774|ref|WP_040429852.1|damageinducibleprotein[Corynebacteriumnuruki]/11668                                                   ------------------------------------------------------------
gi|602237614|gb|EYT60960.1|damageinducibleprotein[Dietziasp.UCDTHP]/11473                                                             ------------------------------------------------------------
gi|665897412|ref|WP_031264618.1|damageinducibleprotein[Dietziasp.UCDTHP]_gi|602237599|gb|EYT60945.1|helicase[Dietziasp.UCDTH/11       ------------------------------------------------------------
gi|502903674|ref|WP_013138650.1|helicase[Segniliparusrotundus]_gi|296181291|gb|ADG98197.1|typeIIIrestrictionproteinressubunit[S       ------------------------------------------------------------
gi|780248306|ref|WP_045538930.1|hypotheticalprotein[Gordoniasp.no.9]_gi|778142514|gb|KJR06917.1|hypotheticalproteinUG54_12320[G       ------------------------------------------------------------
gi|665897368|ref|WP_031264574.1|damageinducibleprotein[Dietziasp.UCDTHP]_gi|602239137|gb|EYT62483.1|helicase[Dietziasp.UCDTH/11       ------------------------------------------------------------
gi|493372923|ref|WP_006329236.1|helicase[Gordoniarhizosphera]_gi|403207917|dbj|GAB88168.1|hypotheticalproteinGORHZ_006_00370[Go       ------------------------------------------------------------
gi|764904870|ref|WP_044487821.1|hypotheticalprotein[Mycobacteriumtuberculosis]_gi|625005353|gb|KBZ57120.1|hypotheticalproteinK8       ------------------------------------------------------------
```

```
gi|927068212|ref|WP_053778441.1|damageinducibleprotein[Gordoniasp.NB41Y]_gi|926660541|gb|EMP14526.2|damageinducibleprotein[G/11      ------------------------------------------------------------
gi|935514169|ref|WP_054429894.1|damageinducibleprotein[Mycobacteriumimmunogenum]_gi|929801120|gb|KPG29247.1|damageinduciblepr/1      ------------------------------------------------------------
gi|941317728|ref|WP_055118574.1|hypotheticalprotein[Mycobacteriumperegrinum]/11657      ------------------------------------------------------------
gi|933683673|gb|ALG86377.1|hypotheticalproteinACH46_20120[Gordoniasp.QH11]/11636      ------------------------------------------------------------
gi|873553947|emb|CRY84110.1|Predictedhelicase[Nocardiafarcinica]/11688      ------------------------------------------------------------
gi|499525749|ref|WP_011212389.1|helicase[Nocardiafarcinica]_gi|54019339|dbj|BAD60707.1|hypotheticalprotein[NocardiafarcinicaIFM      ------------------------------------------------------------
gi|640109609|ref|WP_024794187.1|damageinducibleprotein[Tomitellabiformata]/11653      ------------------------------------------------------------
gi|640109991|ref|WP_024794334.1|hypotheticalprotein[Tomitellabiformata]/11639      ------------------------------------------------------------
gi|939125496|ref|WP_054831438.1|damageinducibleprotein[Rhodococcuserythropolis]/11669      ------------------------------------------------------------
gi|910258550|ref|WP_050062711.1|hypotheticalprotein[Rhodococcussp.RD6.2]_gi|846871967|emb|CRK49225.1|conservedhypotheticalprote      ------------------------------------------------------------
gi|797148189|gb|KJY52358.1|putativeHelicase[Bifidobacteriumasteroides]/11801      ------------------------------------------------------------
gi|535476570|gb|EFG27357.2|hypotheticalproteinHMPREF9020_00999[ScardoviainopinataF0304]/11827      ------------------------------------------------------------
gi|737482729|ref|WP_035462496.1|hypotheticalprotein[Alicyclobacillusmacrosporangiidus]/11512      ------------------------------------------------------------
gi|685434354|gb|KGA95679.1|helicase[BacillusalcalophilusATCC27647=CGMCC1.3604]/11575      ------------------------------------------------------------
gi|945370253|gb|KQU27251.1|helicase[Bacillussp.Leaf13]/11557      ------------------------------------------------------------
gi|880997015|ref|WP_048719116.1|helicase[Enterococcushirae]/11563      ------------------------------------------------------------
gi|945356929|gb|KQU14100.1|helicase[Bacillussp.Leaf49]/11547      ------------------------------------------------------------
gi|763482876|gb|KIZ53217.1|helicase[Bacillussafensis]/11563      ------------------------------------------------------------
gi|852223292|ref|WP_048309707.1|helicase[Anaerobacillusmacyae]_gi|850322910|gb|KMM38590.1|helicase[Anaerobacillusmacyae]/11573      ------------------------------------------------------------
gi|739708665|ref|WP_037562619.1|helicase[Sporolactobacillusterrae]/11573      ------------------------------------------------------------
gi|496687561|ref|WP_009329104.1|MULTISPECIES:helicase[Bacillus]_gi|317390867|gb|EFV71668.1|endonucleaseandmethylaseLlaGI[Bacill      ------------------------------------------------------------
gi|930213599|gb|KPH74225.1|helicase[Oceanobacilluscaeni]/11569      ------------------------------------------------------------
gi|516366003|ref|WP_017756036.1|helicase[Bacillussp.ZYK]/11571      ------------------------------------------------------------
gi|488909977|ref|WP_002821052.1|helicase[Oenococcusoeni]_gi|399965230|gb|EJN99855.1|endonucleaseandmethylaseLlaGI[Oenococcusoen      ------------------------------------------------------------
gi|495019967|ref|WP_007745780.1|helicase[Oenococcuskitaharae]_gi|366983682|gb|EHN59081.1|typeIIIrestrictionenzyme,ressubunit[Oe      ------------------------------------------------------------
gi|489666119|ref|WP_003570406.1|typeIIIrestrictionendonuclease[Lactobacilluscasei]_gi|410527834|gb|EKQ02696.1|typeIIIrestrictio      ------------------------------------------------------------
gi|511447623|gb|EPC78110.1|superfamilyIIDNA/RNAhelicase[Lactobacillusparacaseisubsp.paracaseiLpp221}/11615      ------------------------------------------------------------
gi|497700411|ref|WP_010014595.1|helicase[Lactobacilluscoryniformis]/11585      ------------------------------------------------------------
gi|489784048|ref|WP_003687939.1|helicase[Lactobacillusmali]_gi|394428539|gb|EJF01080.1|superfamilyIIDNA/RNAhelicase[Lactobacill      ------------------------------------------------------------
gi|446780825|ref|WP_000858081.1|helicase[Staphylococcusaureus]_gi|374396604|gb|EHQ67835.1|typeIIIrestrictionenzyme,ressubunit[S      ------------------------------------------------------------
gi|510796658|ref|WP_016171456.1|hypotheticalprotein[Enterococcusdispar]_gi|508167532|gb|EOT44083.1|hypotheticalproteinOMK_00227      ------------------------------------------------------------
gi|612613473|gb|EZS30411.1|hypotheticalproteinW603_02482[StaphylococcusaureusVET0353R]_gi|612881917|gb|EZU93992.1|hypotheticalp      ------------------------------------------------------------
gi|488332893|ref|WP_002402278.1|helicase[Enterococcusfaecalis]_gi|306513864|gb|EFM82466.1|helicaseCterminaldomainprotein[Enter/      ------------------------------------------------------------
gi|921166029|ref|WP_053111806.1|helicase[Enterococcusdurans]_gi|915771660|gb|AKX85928.1|helicase[Enterococcusdurans]/11562      ------------------------------------------------------------
gi|498525358|ref|WP_010825030.1|hypotheticalprotein[Enterococcusfaecalis]_gi|487059631|gb|EOJ86727.1|hypotheticalproteinWOC_005      ------------------------------------------------------------
gi|498412624|ref|WP_010722070.1|hypotheticalprotein[Enterococcusfaecium]_gi|486634654|gb|EOF75253.1|hypotheticalproteinSGC_0253      ------------------------------------------------------------
gi|498408534|ref|WP_010718364.1|hypotheticalprotein[Enterococcusfaecium]_gi|486609608|gb|EOF50873.1|hypotheticalproteinSCW_0257      ------------------------------------------------------------
gi|498407475|ref|WP_010717483.1|hypotheticalprotein[Enterococcusfaecalis]_gi|486594855|gb|EOF36348.1|hypotheticalproteinSCK_005      ------------------------------------------------------------
gi|514885392|ref|WP_016615344.1|helicase[Enterococcusfaecalis]_gi|514505843|gb|EPH71676.1|helicaseprotein[Enterococcusfaecalis0      ------------------------------------------------------------
gi|325157603|gb|EGC69759.1|helicaseCterminaldomainprotein[EnterococcuscasseliflavusATCC12755]/11561      ------------------------------------------------------------
gi|895856206|ref|WP_048967668.1|helicase[Enterococcusfaecalis]/11551      ------------------------------------------------------------
gi|639458836|ref|WP_024636366.1|helicase[Enterococcusfaecium]_gi|658176502|gb|KEI51159.1|helicase[EnterococcusfaeciumUC7256]/1/      ------------------------------------------------------------
gi|488266511|ref|WP_002337719.1|hypotheticalprotein[Enterococcusfaecium]_gi|430581409|gb|ELB19854.1|hypotheticalproteinOIQ_0351      ------------------------------------------------------------
gi|446353356|ref|WP_000431211.1|helicase[Staphylococcusaureus]_gi|253726329|gb|EES95058.1|helicaseCterminaldomainprotein[Staph/      ------------------------------------------------------------
gi|672712068|emb|CDM14336.1|DNAorRNAhelicaseofsuperfamilyII[StaphylococcusepidermidisPM221]/11464      ------------------------------------------------------------
gi|896298990|ref|WP_049279905.1|helicase[Staphylococcusaureus]/11571      ------------------------------------------------------------
gi|541903432|gb|ERH36190.1|hypotheticalproteinSEQU_00085[StaphylococcusequorumUMCCNS924]/11578      ------------------------------------------------------------
gi|488289747|ref|WP_002360955.1|helicase[Enterococcusfaecalis]_gi|256682216|gb|EEU21911.1|endonucleaseandmethylaseLlaGI[Enteroc      ------------------------------------------------------------
gi|494392045|ref|WP_007207894.1|helicase[Enterococcusitalicus]_gi|315483798|gb|EFU74284.1|helicaseCterminaldomainprotein[Enter/      ------------------------------------------------------------
gi|446350692|ref|WP_000428547.1|helicase[Bacilluscereus]_gi|228727580|gb|EEL78721.1|RestrictionmodificationsystemLlaBIII[Bacil/      ------------------------------------------------------------
gi|488255302|ref|WP_002326510.1|hypotheticalprotein[Enterococcusfaecium]_gi|430480276|gb|ELA57459.1|hypotheticalproteinOGE_0473      ------------------------------------------------------------
gi|843253770|ref|WP_047916698.1|helicase[Lactococcuspiscium]_gi|802124677|emb|CEN29502.1|EndonucleaseandmethylaseLlaGI,TypeISPr      ------------------------------------------------------------
LlaBIII/11578      ------------------------------------------------------------
gi|499320531|ref|WP_011011023.1|helicase[Listeriainnocua]_gi|16415805|emb|CAC42019.1|pli0021[ListeriainnocuaClip11262]/11569      ------------------------------------------------------------
gi|760026859|ref|WP_043709973.1|helicase[Weissellacibaria]_gi|758337165|gb|KIU20061.1|UvsWhelicase[Weissellacibaria]/11567      ------------------------------------------------------------
LlaGI/11570      ------------------------------------------------------------
gi|499988414|ref|WP_011669132.1|helicase[Lactococcuslactis]_gi|116109019|gb|ABJ74139.1|DNAorRNAhelicaseofsuperfamilyII[Lactococ      ------------------------------------------------------------
gi|685938360|ref|WP_031670319.1|helicase[Listeriamonocytogenes]_gi|590403387|gb|EXL23261.1|hypotheticalproteinX846_1956[Listeri      ------------------------------------------------------------
gi|530793219|gb|EQC56240.1|helicase[Lactococcuslactissubsp.cremorisTIFN5]_gi|530793247|gb|EQC56263.1|helicase[Lactococcuslactis      ------------------------------------------------------------
gi|695270006|ref|WP_032492075.1|hypotheticalprotein[Listeriamonocytogenes]_gi|695212872|ref|YP_009081522.1|hypotheticalproteinp      ------------------------------------------------------------
gi|504101310|ref|WP_014335297.1|helicase[Leuconostocmesenteroides]_gi|859247905|gb|AKO63885.1|helicase(plasmid)[Leuconostocmese      ------------------------------------------------------------
gi|488905190|ref|WP_002816265.1|helicase[Leuconostocmesenteroides]_gi|227351522|gb|EEJ41777.1|helicaseCterminaldomainprotein[L/      ------------------------------------------------------------
gi|518121285|ref|WP_019291493.1|MULTISPECIES:hypotheticalprotein[Lactococcus]/11588      ------------------------------------------------------------
gi|754857213|ref|WP_042217902.1|helicase[Lactococcusgarvieae]/11549      ------------------------------------------------------------
gi|640048455|ref|WP_024784762.1|helicase[Streptococcusmutans]/11564      ------------------------------------------------------------
gi|512695361|ref|WP_016476323.1|hypotheticalprotein[Veillonellasp.HPA0037]_gi|512079853|gb|EPD79018.1|hypotheticalproteinHMPREF      ------------------------------------------------------------
gi|636963514|ref|WP_024403747.1|helicase[Streptococcussuis]/11574
```

```
gi|881115312|ref|WP_048802949.1|helicase[Staphylococcuspasteuri]/11573      ------------------------------------------------------------
gi|446063996|ref|WP_000141851.1|helicase[Streptococcusmitis]_gi|307618725|gb|EFN97864.1|endonucleaseandmethylaseLlaGI[Streptoco      ------------------------------------------------------------
gi|488206609|ref|WP_002277817.1|restrictionmodificationsystemLlaBIII[Streptococcusmutans]_gi|449173033|gb|EMB75626.1|restricti/      ------------------------------------------------------------
gi|640023290|ref|WP_024781621.1|helicase[Streptococcusmutans]/11566      ------------------------------------------------------------
gi|814946581|ref|WP_046393781.1|helicase[Streptococcusuberis]_gi|810600885|gb|KKF57499.1|helicase[StreptococcusuberisB362]/1156      ------------------------------------------------------------
gi|765470762|ref|WP_044720336.1|helicase[Streptococcussuis]/11559      ------------------------------------------------------------
gi|654507127|ref|WP_027976443.1|helicase[Streptococcusdevriesei]/11558      ------------------------------------------------------------
gi|530812902|gb|EQC72481.1|DNAorRNAhelicaseofsuperfamilyII[Streptococcussp.HSISS2]/11570      ------------------------------------------------------------
gi|489158846|ref|WP_003068504.1|helicase[Streptococcusintermedius]_gi|355367114|gb|EHG14827.1|hypotheticalproteinHMPREF9682_002      ------------------------------------------------------------
RM.Lru27782ORF13200P/11620      ------------------------------------------------------------
gi|492676546|ref|WP_005919236.1|DNAphosphorothioationsystemrestrictionenzyme[Pediococcusacidilactici]_gi|482517924|gb|EOA08103.      ------------------------------------------------------------
gi|498183116|ref|WP_010497272.1|helicase[Lactobacillusacidipiscis]/11563      ------------------------------------------------------------
gi|822100485|ref|WP_046921865.1|helicase[Lactobacillusruminis]_gi|821684442|gb|KLA43321.1|restrictionmodificationsystemLlaBIII/      ------------------------------------------------------------
gi|935565832|ref|WP_054450311.1|helicase[Lactobacilluskunkeei]/11573      ------------------------------------------------------------
gi|752296887|ref|WP_041152901.1|helicase[Lactobacilluskunkeei]_gi|751663520|gb|KIM18358.1|helicase[Lactobacilluskunkeei]/11577      ------------------------------------------------------------
gi|495745291|ref|WP_008469870.1|helicase[Lactobacillushominis]_gi|394484442|emb|CCI81207.1|RestrictionmodificationsystemLlaBII/      ------------------------------------------------------------
gi|918672414|ref|WP_052543134.1|helicase[Lactobacillussp.]/11564      ------------------------------------------------------------
gi|799141631|ref|WP_045927261.1|helicase[Lactobacilluskullabergensis]_gi|797155335|gb|KJY59264.1|putativerestrictionendonucleas      ------------------------------------------------------------
gi|504379990|ref|WP_014567092.1|helicase[Lactobacillusjohnsonii]_gi|329666726|gb|AEB92674.1|putativerestrictionendonuclease[Lac      ------------------------------------------------------------
gi|489880179|ref|WP_003783646.1|DNAdamageinducibleprotein[Actinomycesnaeslundii]_gi|399901561|gb|EJN84440.1|typeIIIrestriction/      ------------------------------------------------------------
gi|496524497|ref|WP_009232780.1|DNAdamageinducibleprotein[Actinomycessp.oraltaxon849]_gi|365265413|gb|EHM95177.1|hypotheticalp/      ------------------------------------------------------------
gi|915397782|ref|WP_050783167.1|damageinducibleprotein[Actinomycesoris]/11694      ------------------------------------------------------------
gi|502925248|ref|WP_013160224.1|helicase[Propionibacteriumfreudenreichii]_gi|296921272|emb|CBL55824.1|helicase[Propionibacteriu      ------------------------------------------------------------
gi|732164352|emb|CEG87481.1|helicase[Propionibacteriumfreudenreichii]/11618      ------------------------------------------------------------
gi|739047402|ref|WP_036925058.1|damageinducibleprotein[Propionibacteriumthoenii]/11596      ------------------------------------------------------------
gi|739112839|ref|WP_036982533.1|damageinducibleprotein[Propionibacteriumjensenii]/11594      ------------------------------------------------------------
gi|918023558|ref|WP_052314121.1|damageinducibleprotein[Nocardiathailandica]/11612      ------------------------------------------------------------
gi|498380271|ref|WP_010694427.1|DNAdamageinducibleprotein[Saccharopolysporaspinosa]/11605      ------------------------------------------------------------
gi|749991459|ref|WP_040339809.1|damageinducibleprotein[CandidatusBlastococcusmassiliensis]/11597      ------------------------------------------------------------
gi|754177801|ref|WP_041796231.1|damageinducibleprotein[Modestobactermarinus]/11592      ------------------------------------------------------------
gi|504188248|ref|WP_014375350.1|DNAdamageinducibleprotein[Blastococcussaxobsidens]_gi|378782790|emb|CCG02456.1|putativeHelicas/      ------------------------------------------------------------
gi|946476713|gb|KRF26798.1|damageinducibleprotein[Phycicoccussp.Soil803]/11609      ------------------------------------------------------------
gi|700176672|gb|KGN37509.1|DNAdamageinducibleprotein[KnoelliasubterraneaKCTC19937]/11633      ------------------------------------------------------------
gi|700168841|gb|KGN29914.1|hypotheticalproteinN802_10215[KnoelliasinensisKCTC19936]/11631      ------------------------------------------------------------
gi|946426241|gb|KRE76610.1|damageinducibleprotein[Arthrobactersp.Soil763]/11606      ------------------------------------------------------------
gi|517318525|ref|WP_018500927.1|hypotheticalprotein[Frankiasp.BCU110501]/11592      ------------------------------------------------------------
gi|499072429|ref|WP_010851327.1|Helicase[Tetrasphaeraelongata]_gi|478756579|emb|CCH71501.1|putativeHelicase[Tetrasphaeraelongat      ------------------------------------------------------------
gi|750080666|ref|WP_040385350.1|damageinducibleprotein[Demetriaterragena]/11625      ------------------------------------------------------------
gi|752558292|ref|WP_041229523.1|damageinducibleprotein[Dermacoccusnishinomiyaensis]_gi|664689315|gb|AIF41904.1|damageinducibl/1      ------------------------------------------------------------
gi|829089213|ref|WP_047310701.1|damageinducibleprotein[Dermacoccussp.PE3]_gi|828384812|gb|KLO63770.1|damageinducibleprotein[D/1      ------------------------------------------------------------
gi|748756635|ref|WP_040014750.1|damageinducibleprotein[Dermacoccussp.Ellin185]/11596      ------------------------------------------------------------
gi|916534724|ref|WP_051141848.1|damageinducibleprotein[Actinomycesneuii]/11632      ------------------------------------------------------------
gi|921140030|ref|WP_053090276.1|damageinducibleprotein[Rothiamucilaginosa]/11616      ------------------------------------------------------------
gi|896406751|ref|WP_049343749.1|damageinducibleprotein[Rothiamucilaginosa]/11647      ------------------------------------------------------------
gi|723608234|gb|AIY01610.1|putativehelicase[Arthrobactersp.PAMC25486]/11613      ------------------------------------------------------------
gi|946394527|gb|KRE45160.1|damageinducibleprotein[Arthrobactersp.Soil736]/11616      ------------------------------------------------------------
gi|515765304|ref|WP_017197904.1|hypotheticalprotein[Arthrobactersp.M2012083]/11648      ------------------------------------------------------------
gi|945315587|gb|KQT73127.1|damageinducibleprotein[Microbacteriumsp.Leaf436]/11612      ------------------------------------------------------------
gi|759908497|ref|WP_043594599.1|damageinducibleprotein[Frigoribacteriumsp.MEB024]_gi|758271359|gb|KIU03346.1|damageinduciblep/1      ------------------------------------------------------------
gi|945072850|gb|KQR34237.1|hypotheticalproteinASF75_00055[Curtobacteriumsp.Leaf154]/11597      ------------------------------------------------------------
gi|551263152|ref|WP_022883363.1|DNAdamageinducibleprotein[Glaciibactersuperstes]/11621      ------------------------------------------------------------
gi|945452341|gb|KQV08491.1|hypotheticalproteinASC63_10170[Leifsoniasp.Root112D2]/11623      ------------------------------------------------------------
gi|742759483|gb|KIC69912.1|damageinducibleprotein[Kocuriarhizophila]/11610      ------------------------------------------------------------
gi|737919995|ref|WP_035885304.1|damageinducibleprotein[Kocuriarhizophila]/11611      ------------------------------------------------------------
gi|737997701|ref|WP_035959325.1|damageinducibleprotein[Kocuriamarina]_gi|725823884|gb|KHE75603.1|damageinducibleprotein[Kocur/1      ------------------------------------------------------------
gi|664521070|ref|WP_031037650.1|damageinducibleprotein[Streptomycessp.NRRLWC3774]/11624      ------------------------------------------------------------
gi|500101034|ref|WP_011777041.1|DNAdamageinducibleprotein[Arthrobacteraurescens]_gi|119951547|gb|ABM10457.1|putativeHelicase[A/      ------------------------------------------------------------
gi|916871953|ref|WP_051479009.1|damageinducibleprotein[Arthrobactersp.H5]/11588      ------------------------------------------------------------
gi|612084649|gb|EZP43369.1|TypeIIIrestrictionenzyme,ressubunit[Micrococcusluteus]/11625      ------------------------------------------------------------
gi|759739708|ref|WP_043450422.1|damageinducibleprotein[Arthrobacterphenanthrenivorans]_gi|742758264|gb|KIC68716.1|damageinduc/1      ------------------------------------------------------------
gi|636845448|ref|WP_024366823.1|damageinducibleprotein[Arthrobactersp.TB26]/11605      ------------------------------------------------------------
gi|500009025|ref|WP_011689743.1|DNAdamageinducibleprotein[Arthrobactersp.FB24]_gi|116613051|gb|ABK05760.1|typeIIIrestrictionen/      ------------------------------------------------------------
gi|821249847|gb|KKZ12083.1|hypotheticalproteinTQ37_06090[CandidatusSynechococcusspongiarum15L]/11436      ------------------------------------------------------------
gi|739446902|ref|WP_037306885.1|damageinducibleprotein[Ruegeriahalocynthiae]/11592      ------------------------------------------------------------
gi|640620081|ref|WP_025048131.1|damageinducibleprotein[Sulfitobactermediterraneus]_gi|751956650|gb|KIN77213.1|TypeIIIrestricti/      ------------------------------------------------------------
gi|740247634|ref|WP_038088583.1|damageinducibleprotein[Thiobacillusprosperus]_gi|684049748|gb|KFZ90853.1|putativehelicase[Thio/      ------------------------------------------------------------
gi|518420321|ref|WP_019590528.1|hypotheticalprotein[Thioalkalivibriosp.ALE20]/11603      ------------------------------------------------------------
```

```
gi|500156826|ref|WP_011831496.1|DNAdamageinducibleprotein[Methylibiumpetroleiphilum]_gi|124261892|gb|ABM96881.1|adeninespecifi/    ------------------------------------------------------------
gi|769978537|ref|WP_045094522.1|damageinducibleprotein[Legionellafallonii]_gi|698841736|emb|CEG55682.1|Putativeproteinwithboxh/     ------------------------------------------------------------
gi|553752541|ref|WP_023085344.1|hypotheticalprotein[Pseudomonasaeruginosa]_gi|552201952|gb|ERU62653.1|hypotheticalproteinQ088_0    ------------------------------------------------------------
gi|880980418|ref|WP_048704461.1|damageinducibleprotein[Thauerahumireducens]_gi|875132785|gb|KMU70944.1|damageinducibleprotein/1    ------------------------------------------------------------
gi|740135732|ref|WP_037982815.1|damageinducibleprotein[Thauerasp.27]/11603                                                         ------------------------------------------------------------
gi|738017371|ref|WP_035978332.1|damageinducibleprotein[Burkholderiaglumae]/11380                                                   ------------------------------------------------------------
gi|498506003|ref|WP_010806671.1|typeIIIrestrictionenzyme,ressubunit[Pandoraeasp.SD62]_gi|490314561|gb|EON12879.1|typeIIIrestri/    ------------------------------------------------------------
gi|500117748|ref|WP_011793753.1|DNAdamageinducibleprotein[Acidovoraxcitrulli]_gi|120587742|gb|ABM31182.1|typeIIIrestrictionenz/    ------------------------------------------------------------
gi|651617328|ref|WP_026611444.1|damageinducibleprotein[Methylocaldumszegediense]/11601                                              ------------------------------------------------------------
gi|737210208|ref|WP_035194994.1|damageinducibleprotein[Acidithiobacillusferrivorans]_gi|669953220|emb|CDQ11755.1|Adeninespecif/    ------------------------------------------------------------
gi|935697386|ref|WP_054506180.1|damageinducibleprotein[Achromobactersp.]_gi|928716153|emb|CUK12836.1|Hefnuclease[Achromobacter/    ------------------------------------------------------------
gi|429502517|gb|ELA00827.1|adeninespecificDNAmethyltransferase[Cupriavidussp.HMR1]/11599                                           ------------------------------------------------------------
gi|506356642|ref|WP_015876361.1|DNAdamageinducibleprotein[Burkholderiaglumae]_gi|237877158|gb|ACR29491.1|AdeninespecificDNAmet/    ------------------------------------------------------------
gi|696406787|ref|WP_032978003.1|damageinducibleprotein[Bordetellahinzii]_gi|625335076|gb|KCB49343.1|typeIIIrestrictionenzyme,r/    ------------------------------------------------------------
gi|817529125|gb|KKO72587.1|damageinducibleprotein[Kerstersiagyiorum]/11592                                                         ------------------------------------------------------------
gi|737048910|ref|WP_035044118.1|damageinducibleprotein[Aquabacteriumsp.NJ1]_gi|699052896|gb|KGM38706.1|damageinducibleprotein/1    ------------------------------------------------------------
gi|822492519|ref|WP_046933319.1|MULTISPECIES:damageinducibleprotein[Xanthomonas]_gi|821703525|gb|KLA61196.1|damageinduciblepr/1    ------------------------------------------------------------
gi|751252861|ref|WP_040963740.1|damageinducibleprotein[Xanthomonasfuscans]_gi|749808363|gb|KIJ03887.1|damageinducibleprotein[/1    ------------------------------------------------------------
gi|496774980|ref|WP_009368792.1|DNAdamageinducibleprotein[Bilophilasp.4_1_30]_gi|345039902|gb|EGW44205.1|hypotheticalproteinHM/    ------------------------------------------------------------
gi|499241912|ref|WP_010939452.1|DNAdamageinducibleprotein[Desulfovibriovulgaris]_gi|46580580|ref|YP_011388.1|adeninespecificDN/    ------------------------------------------------------------
gi|504274913|ref|WP_014462015.1|DNAdamageinducibleprotein[Bacillusmegaterium]_gi|345446919|gb|AEN91936.1|AdeninespecificDNAmet/    ------------------------------------------------------------
gi|504895966|ref|WP_015083068.1|typeIIrestrictionenzymeandmethyltransferasesubtypeG[Anabaenasp.90]_gi|413972424|gb|AFW96512.1|t    ------------------------------------------------------------
gi|518327765|ref|WP_019497972.1|hypotheticalprotein[Calothrixsp.PCC7103]/11600                                                     ------------------------------------------------------------
gi|652765503|ref|WP_027079917.1|damageinducibleprotein[Luteimonasmephitis]/11593                                                   ------------------------------------------------------------
gi|896539951|ref|WP_049442123.1|damageinducibleprotein[Stenotrophomonasmaltophilia]/11591                                          ------------------------------------------------------------
gi|500217900|ref|WP_011888043.1|DNAdamageinducibleprotein[Geobacillusthermodenitrificans]_gi|134267954|gb|ABO68149.1|Conserved/    ------------------------------------------------------------
gi|696570298|ref|WP_033102014.1|damageinducibleprotein[Thermoactinomycesdaqus]/11594                                               ------------------------------------------------------------
gi|251772067|gb|EES52637.1|putativehelicase[Leptospirillumferrodiazotrophum]/11618                                                 ------------------------------------------------------------
gi|738124101|ref|WP_036082108.1|damageinducibleprotein[Leptospirillumferriphilum]_gi|685243306|gb|KGA94177.1|putativehelicase[/    ------------------------------------------------------------
gi|558699223|ref|WP_023524766.1|helicase[Leptospirillumsp.GroupII'CF1']_gi|206603764|gb|EDZ40244.1|Putativehelicase[Leptospiri/    ------------------------------------------------------------
gi|406774462|gb|AFS53887.1|putativehelicase[LeptospirillumferriphilumML04]/11610                                                   ------------------------------------------------------------
gi|740719463|ref|WP_038504749.1|damageinducibleprotein[Leptospirillumferriphilum]_gi|640838125|gb|AIA30173.1|damageinduciblep/1    ------------------------------------------------------------
gi|517810766|ref|WP_018980974.1|hypotheticalprotein[Saccharibacterfloricola]/11733                                                 ------------------------------------------------------------
gi|502471675|ref|WP_012798533.1|helicase[Slackiaheliotrinireducens]_gi|256791761|gb|ACV22431.1|predictedhelicase[Slackiaheliotr    SVKKAFEIAIMGAHIIGAKETGYANDLANQLQDAIDSGELTDNDIRNAVRLARYDAAYRA
gi|657871713|ref|WP_029576297.1|hypotheticalprotein[Bifidobacteriumthermacidophilum]_gi|673007521|gb|KFJ02535.1|helicase[Bifido    ------------------------------------------------------------
gi|673004757|gb|KFI99791.1|DNAhelicaserestrictionenzymetypeIIIRessubunit[Bifidobacteriumthermacidophilumsubsp.porcinum]/11682      ------------------------------------------------------------
gi|917316092|ref|WP_051922804.1|hypotheticalprotein[Bifidobacteriumstellenboschense]_gi|673003774|gb|KFI98812.1|helicase[Bifido    ------------------------------------------------------------
gi|705415694|ref|WP_033502332.1|hypotheticalprotein[Bifidobacteriumpseudolongum]_gi|672982411|gb|KFI77685.1|DNAdamageinducible/    ------------------------------------------------------------
gi|916591418|ref|WP_051198509.1|hypotheticalprotein[Bifidobacteriumsp.AGR2158]/11704                                               ------------------------------------------------------------
gi|772242982|gb|AJW78255.1|hypotheticalproteinVO01_03165[Clavibactermichiganensissubsp.insidiosus]/11642                           ------------------------------------------------------------
gi|916649818|ref|WP_051256909.1|damageinducibleprotein[Actinomycesgraevenitzii]/11677                                              ------------------------------------------------------------
gi|496504578|ref|WP_009212861.1|helicase[Actinomycessp.oraltaxon180]_gi|315312695|gb|EFU60776.1|helicase[Actinomycessp.oraltaxo    ------------------------------------------------------------
gi|494862206|ref|WP_007588306.1|helicase[Actinomycessp.ICM39]_gi|398362799|gb|EJN46470.1|typeIIIrestrictionenzyme,ressubunit[Ac    ------------------------------------------------------------
gi|881057111|ref|WP_048774974.1|damageinducibleprotein[Sanguibacterkeddieii]/11697                                                 ------------------------------------------------------------
gi|881020459|ref|WP_048740808.1|damageinducibleprotein[Kytococcussedentarius]/11704                                                ------------------------------------------------------------
gi|881052313|ref|WP_048770555.1|damageinducibleprotein[Cellulomonasflavigena]/11705                                                ------------------------------------------------------------
gi|490108682|ref|WP_004009424.1|helicase[Mobiluncuscurtisii]_gi|315490465|gb|EFU80089.1|helicaseCterminaldomainprotein[Mobilun/    ------------------------------------------------------------
gi|490111935|ref|WP_004012603.1|helicase[Mobiluncusmulieris]_gi|227843812|gb|EEJ53982.1|helicaseCterminaldomainprotein[Mobilun/    ------------------------------------------------------------
gi|490114228|ref|WP_004014848.1|helicase[Mobiluncusmulieris]_gi|269934432|gb|EEZ90993.1|helicaseCterminaldomainprotein[Mobilun/    ------------------------------------------------------------
gi|493333551|ref|WP_006290636.1|helicase[Parascardoviadenticolens]_gi|315119487|gb|EFT82620.1|helicaseCterminaldomainprotein[P/    ------------------------------------------------------------
gi|495817067|ref|WP_008541646.1|helicase,partial[Sutterellaparvirubra]_gi|378598583|gb|EHY31712.1|helicaseprotein,partial[Sutte    ------------------------------------------------------------
gi|491572696|ref|WP_005430278.1|helicase[Sutterellawadsworthensis]_gi|319805116|gb|EFW01943.1|DNAhelicaserestrictionenzymeTypeI    ------------------------------------------------------------
gi|748686321|ref|WP_039944445.1|hypotheticalprotein[Thermicanusaegyptius]/11573                                                    ------------------------------------------------------------
gi|916875698|ref|WP_051482754.1|hypotheticalprotein[Thioalkalivibriosp.HK1]/11366                                                  ------------------------------------------------------------
gi|821248604|gb|KKZ10960.1|hypotheticalproteinTE42_09075[CandidatusSynechococcusspongiarumSP3]/11598                               ------------------------------------------------------------
gi|737409843|ref|WP_035390956.1|hypotheticalprotein[Ferrimicrobiumacidiphilum]_gi|765544266|gb|KJE76241.1|typeIrestrictionenzym    ------------------------------------------------------------
gi|654906925|ref|WP_028358281.1|damageinducibleprotein[Brackiellaoedipodis]/11679                                                  ------------------------------------------------------------
gi|502809901|ref|WP_013044877.1|hypotheticalprotein[CandidatusPuniceispirillummarinum]_gi|292663146|gb|ADE38247.1|hypotheticalp    ------------------------------------------------------------
gi|251771370|gb|EES51950.1|typeIIIrestrictionenzyme,ressubunit[Leptospirillumferrodiazotrophum]/11628                              ------------------------------------------------------------
gi|498328480|ref|WP_010642636.1|DNAdamageinducibleprotein[Acidithiobacillusthiooxidans]/11627                                      ------------------------------------------------------------
gi|647633864|ref|WP_025906698.1|damageinducibleprotein[Prochlorococcussp.scB245a_519B7]/11622                                      ------------------------------------------------------------
gi|647619599|ref|WP_025893729.1|damageinducibleprotein[Prochlorococcussp.scB241_527N11]/11621                                      ------------------------------------------------------------
gi|755034887|ref|WP_042387498.1|hypotheticalprotein[Clostridiumsp.CL6]/11606                                                      ------------------------------------------------------------
gi|498139798|ref|WP_010453954.1|helicase[SuccinivibrionaceaebacteriumWG1]/11662                                                   ------------------------------------------------------------
gi|527501386|gb|EPT34571.1|typeIIIrestrictionenzyme,ressubunit[Bacteroidetesbacteriumoraltaxon272str.F0290]/11789                  ------------------------------------------------------------
gi|496047275|ref|WP_008771782.1|helicase[Bacteroidessp.2_1_33B]_gi|262296443|gb|EEY84373.1|helicaseCterminaldomainprotein[Bact/    ------------------------------------------------------------
PDI8503III/11664                                                                                                                   ------------------------------------------------------------
```

```
gi|738719431|ref|WP_036613715.1|helicase[Parabacteroidesdistasonis]_gi|649542518|gb|KDS49387.1|restrictionendonucleasefamilypro      ------------------------------------------------------------
gi|659417033|gb|KEJ86133.1|hypotheticalproteinHMPREF1002_00341[Porphyromonassp.31_2]/11656                                           ------------------------------------------------------------
gi|567456807|gb|ETK11358.1|hypotheticalproteinT235_15845[Tannerellasp.oraltaxonBU063isolateCell8/11]/11433                          ------------------------------------------------------------
gi|545440279|ref|WP_021677877.1|helicase[Porphyromonasgingivalis]_gi|543985765|gb|ERJ89108.1|helicaseprotein[Porphyromonasgingi      ------------------------------------------------------------
gi|497438056|ref|WP_009752254.1|helicase[Prevotellasp.oraltaxon473]_gi|429144866|gb|EKX87968.1|helicaseprotein[Prevotellasp.ora      ------------------------------------------------------------
gi|490423474|ref|WP_004295683.1|MULTISPECIES:helicase[Bacteroides]_gi|156110098|gb|EDO11843.1|helicaseCterminaldomainprotein[B/      ------------------------------------------------------------
gi|941777229|ref|WP_055173506.1|helicase[Bacteroidescaccae]_gi|933263395|emb|CUQ00567.1|Predictedhelicase[Bacteroidescaccae]/1/      ------------------------------------------------------------
gi|492413898|ref|WP_005836892.1|helicase[Bacteroidesuniformis]_gi|392678824|gb|EIY72229.1|hypotheticalproteinHMPREF1072_03583[B      ------------------------------------------------------------
gi|695455095|ref|WP_032566191.1|helicase[Bacteroidesfragilis]_gi|596095426|gb|EXZ59996.1|restrictionendonucleasefamilyprotein[B      ------------------------------------------------------------
gi|933227941|emb|CUQ02348.1|Predictedhelicase[Bacteroidesuniformis]/11658                                                            ------------------------------------------------------------
gi|494823013|ref|WP_007558421.1|helicase[Bacteroidesplebeius]_gi|198272167|gb|EDY96436.1|helicaseCterminaldomainprotein[Bacter/      ------------------------------------------------------------
gi|495427212|ref|WP_008151909.1|helicase[Parabacteroidesjohnsonii]_gi|218222381|gb|EEC95031.1|helicaseCterminaldomainprotein[P/      ------------------------------------------------------------
gi|933196855|emb|CUO94545.1|typeIrestrictionenzymeEcoKIsubunitR[Bacteroidesuniformis]_gi|933220098|emb|CUN86874.1|typeIrestrict      ------------------------------------------------------------
gi|823687748|ref|WP_047101121.1|helicase[Brachyspirahyodysenteriae]_gi|823334537|gb|KLI17932.1|helicase[Brachyspirahyodysenteri      ------------------------------------------------------------
gi|496436691|ref|WP_009145538.1|helicase[Phascolarctobacteriumsuccinatutens]_gi|322414007|gb|EFY04839.1|helicaseCterminaldomai/      ------------------------------------------------------------
gi|503372880|ref|WP_013607541.1|helicase[Sphaerochaetaglobosa]_gi|324026933|gb|ADY13692.1|typeIIIrestrictionproteinressubunit[S      ------------------------------------------------------------
gi|505821748|ref|WP_015707145.1|helicase[Treponemaprimitia]_gi|299483503|gb|ADJ19584.1|helicasedomaincontainingprotein[Trepone/      ------------------------------------------------------------
gi|505822741|ref|WP_015708134.1|helicase[Treponemaprimitia]_gi|333739639|gb|AEF85129.1|endonucleaseandmethylaseLlaGI[Treponemap      ------------------------------------------------------------
gi|488791994|ref|WP_002704400.1|helicase[Treponemasaccharophilum]_gi|380767691|gb|EIC01689.1|typeIIIrestrictionproteinressubuni      ------------------------------------------------------------
gi|524671454|emb|CDD81750.1|putativeseptumsitedeterminingproteinMinC[Bacteroidessp.CAG:462]/11642                                   ------------------------------------------------------------
gi|524573865|emb|CDC95766.1|putativeseptumsitedeterminingproteinMinC[Alistipessp.CAG:268]/11639                                     ------------------------------------------------------------
gi|658617970|ref|WP_029759328.1|helicase[Fusobacteriumnucleatum]/11606                                                                ------------------------------------------------------------
gi|494412037|ref|WP_007216782.1|helicase[Bacteroidescellulosilyticus]_gi|392638921|gb|EIY32752.1|hypotheticalproteinHMPREF1062_      ------------------------------------------------------------
gi|383107102|gb|AFG37435.1|putativehelicase[SpirochaetaafricanaDSM8902]/11616                                                         ------------------------------------------------------------
SAF8902III/11616                                                                                                                      ------------------------------------------------------------
gi|652538894|ref|WP_026932796.1|helicase[Gramellaechinicola]/11633                                                                    ------------------------------------------------------------
gi|639238223|ref|WP_024568839.1|helicase[Elizabethkingiaanophelis]/11628                                                              ------------------------------------------------------------
gi|652520381|ref|WP_026914582.1|helicase[Gramellaportivictoriae]/11623                                                                ------------------------------------------------------------
gi|494922183|ref|WP_007648219.1|Helicase[Formosasp.AK20]_gi|469825489|gb|EMQ95550.1|Helicasedomainprotein[Formosasp.AK20]/11626      ------------------------------------------------------------
gi|522083382|ref|WP_020594591.1|hypotheticalprotein[Spirosomapanaciterrae]/11637                                                      ------------------------------------------------------------
gi|490455352|ref|WP_004326157.1|helicase[Bacteroidesovatus]_gi|392669277|gb|EIY62768.1|hypotheticalproteinHMPREF1070_03635[Bact      ------------------------------------------------------------
gi|499516035|ref|WP_011202675.1|helicase[Bacteroidesfragilis]_gi|53713270|ref|YP_099262.1|helicasedomainprotein[Bacteroidesfrag      ------------------------------------------------------------
gi|651338332|ref|WP_026461320.1|helicase[Adhaeribacteraquaticus]/11620                                                               ------------------------------------------------------------
gi|806964642|gb|AKC17139.1|helicase[Flavobacteriumpsychrophilum]/11639                                                                ------------------------------------------------------------
gi|752684374|ref|WP_041332322.1|helicase[Flavobacteriumpsychrophilum]_gi|666882889|gb|AIG30715.1|helicase[Flavobacteriumpsychro      ------------------------------------------------------------
gi|933196595|emb|CUP04894.1|DNAorRNAhelicasesofsuperfamilyII[Bacteroidesuniformis]/11631                                             ------------------------------------------------------------
gi|670509109|ref|WP_031456918.1|helicase[Flavobacteriumchungangense]/11625                                                            ------------------------------------------------------------


gi|490190857|ref|WP_004089451.1|hypotheticalprotein[Helicobacterbilis]_gi|476630638|gb|EMZ37196.1|hypotheticalproteinC826_02193      ------------------------------------------------------------
gi|488896133|ref|WP_002807242.1|MULTISPECIES:restrictionendonucleaseEco57I[Campylobacter]_gi|380541096|gb|EIA65377.1|hypothetic      ------------------------------------------------------------
gi|746602240|ref|WP_039628365.1|restrictionendonucleaseEco57I[Campylobacterlari]_gi|744819395|gb|AJD03391.1|putativehelicase[Ca      ------------------------------------------------------------
gi|727317135|ref|WP_033772558.1|restrictionendonucleaseEco57I[Helicobacterpylori]/11622                                               ------------------------------------------------------------
gi|727103490|ref|WP_033618954.1|restrictionendonucleaseEco57I[Helicobacterpylori]/11616                                               ------------------------------------------------------------
gi|727340205|ref|WP_033794408.1|restrictionendonucleaseEco57I[Helicobacterpylori]/11609                                               ------------------------------------------------------------
gi|727292407|ref|WP_033749975.1|restrictionendonucleaseEco57I[Helicobacterpylori]/11611                                               ------------------------------------------------------------
gi|727289795|ref|WP_033747591.1|restrictionendonucleaseEco57I[Helicobacterpylori]/11612                                               ------------------------------------------------------------
gi|727319676|ref|WP_033775047.1|restrictionendonucleaseEco57I[Helicobacterpylori]/11612                                               ------------------------------------------------------------
gi|447212910|ref|WP_001290166.1|restrictionendonucleaseEco57I[Helicobacterpylori]_gi|308063540|gb|ADO05427.1|hypotheticalprotei      ------------------------------------------------------------
gi|447212901|ref|WP_001290157.1|restrictionendonucleaseEco57I[Helicobacterpylori]_gi|384557329|gb|AFH97797.1|hypotheticalprotei      ------------------------------------------------------------
gi|515849671|ref|WP_017280424.1|hypotheticalprotein[Helicobacterpylori]/11615                                                         ------------------------------------------------------------
gi|447212900|ref|WP_001290156.1|restrictionendonucleaseEco57I[Helicobacterpylori]_gi|317178970|dbj|BAJ56758.1|TypeIIGrestrictio      ------------------------------------------------------------
gi|447079018|ref|WP_001156274.1|hypotheticalprotein[Helicobacterpylori]/11614                                                         ------------------------------------------------------------
gi|447212800|ref|WP_001290056.1|hypotheticalprotein[Helicobacterpylori]_gi|532153248|gb|EQL67316.1|restrictionendonucleaseEco57      ------------------------------------------------------------
gi|505457237|ref|WP_015644339.1|hypotheticalprotein[Helicobacterpylori]_gi|499061160|gb|AGL70074.1|restrictionendonucleaseEco57      ------------------------------------------------------------
HPYUM037X/11591                                                                                                                       ------------------------------------------------------------
gi|920026297|ref|WP_052937176.1|restrictionendonuclease[Helicobacterpylori]_gi|912528678|gb|KNX48238.1|restrictionendonucleaseE      ------------------------------------------------------------
gi|915539050|ref|WP_050841327.1|restrictionendonuclease[Helicobacterpylori]_gi|908890887|gb|KNE06183.1|restrictionendonucleaseE      ------------------------------------------------------------
gi|727101397|ref|WP_033616957.1|restrictionendonucleaseEco57I[Helicobacterpylori]/11592                                               ------------------------------------------------------------
gi|562814875|emb|CDK13002.1|TypeISPRMsystem[Helicobacterpylori26695]/11588                                                            ------------------------------------------------------------
HPYAXVIII/11588                                                                                                                       ------------------------------------------------------------
gi|447079029|ref|WP_001156285.1|restrictionendonucleaseEco57I[Helicobacterpylori]_gi|393085956|gb|EJB86635.1|hypotheticalprotei      ------------------------------------------------------------
gi|489105953|ref|WP_003015814.1|restrictionendonuclease[Helicobacterpylori]_gi|477503388|gb|ENH59950.1|restrictionendonucleasef      ------------------------------------------------------------
gi|447079006|ref|WP_001156262.1|restrictionendonucleaseEco57I[Helicobacterpylori]_gi|393067470|gb|EJB68280.1|hypotheticalprotei      ------------------------------------------------------------
gi|727314589|ref|WP_033770298.1|restrictionendonucleaseEco57I[Helicobacterpylori]/11586                                               ------------------------------------------------------------
gi|447079019|ref|WP_001156275.1|restrictionendonucleaseEco57I[Helicobacterpylori]_gi|308065028|gb|ADO06920.1|hypotheticalprotei      ------------------------------------------------------------
gi|447079028|ref|WP_001156284.1|restrictionendonucleaseEco57I[Helicobacterpylori]_gi|393076030|gb|EJB76784.1|hypotheticalprotei      ------------------------------------------------------------
```

```
gi|393034009|gb|EJB35070.1|hypotheticalproteinHPNQ4053_0631[HelicobacterpyloriNQ4053]/11514        ------------------------------------------------------------
gi|447079004|ref|WP_001156260.1|restrictionendonucleaseEco57I[Helicobacterpylori]_gi|384552418|gb|AFI07366.1|hypotheticalprotei        ------------------------------------------------------------
gi|746011306|ref|WP_039088215.1|restrictionendonucleaseEco57I[Helicobacterpylori]_gi|733488720|gb|KHL85979.1|restrictionendonuc        ------------------------------------------------------------
gi|531081162|gb|EQD92166.1|restrictionendonucleaseEco57I[HelicobacterpyloriPZ5080]/11509        ------------------------------------------------------------
gi|544760796|ref|WP_021187395.1|restrictionendonucleaseEco57I[Helicobacterpylori]_gi|531086508|gb|EQD96745.1|restrictionendonuc        ------------------------------------------------------------
gi|447079012|ref|WP_001156268.1|restrictionendonucleaseEco57I[Helicobacterpylori]_gi|393028199|gb|EJB29286.1|hypotheticalprotei        ------------------------------------------------------------
gi|746008458|ref|WP_039085555.1|restrictionendonucleaseEco57I[Helicobacterpylori]_gi|733480358|gb|KHL78026.1|restrictionendonuc        ------------------------------------------------------------
gi|447079011|ref|WP_001156267.1|restrictionendonucleaseEco57I[Helicobacterpylori]_gi|393038534|gb|EJB39568.1|hypotheticalprotei        ------------------------------------------------------------
gi|393037163|gb|EJB38199.1|hypotheticalproteinHPNQ4161_0422[HelicobacterpyloriNQ4161]/11556        ------------------------------------------------------------
gi|447079007|ref|WP_001156263.1|restrictionendonucleaseEco57I[Helicobacterpylori]_gi|393051817|gb|EJB52768.1|hypotheticalprotei        ------------------------------------------------------------
gi|487867094|ref|WP_001940560.1|helicase[Helicobacterpylori]_gi|459505657|gb|EMG98418.1|helicaseprotein[HelicobacterpyloriGAM21        ------------------------------------------------------------
gi|487872094|ref|WP_001945560.1|helicase[Helicobacterpylori]_gi|459512104|gb|EMH04523.1|helicaseprotein[HelicobacterpyloriGAM24        ------------------------------------------------------------
gi|487880228|ref|WP_001953694.1|helicase[Helicobacterpylori]_gi|459529131|gb|EMH20734.1|helicaseprotein[HelicobacterpyloriGAM26        ------------------------------------------------------------
gi|487882593|ref|WP_001956059.1|helicase[Helicobacterpylori]_gi|459533462|gb|EMH24859.1|helicaseprotein[HelicobacterpyloriGAM26        ------------------------------------------------------------
gi|487845430|ref|WP_001918896.1|helicase[Helicobacterpylori]_gi|410713871|gb|EKQ71363.1|helicaseprotein[HelicobacterpyloriGAM10        ------------------------------------------------------------
gi|447079005|ref|WP_001156261.1|restrictionendonucleaseEco57I[Helicobacterpylori]_gi|393069830|gb|EJB70625.1|hypotheticalprotei        ------------------------------------------------------------
gi|459506808|gb|EMG99528.1|helicaseprotein[HelicobacterpyloriGAM121Aii]/11545        ------------------------------------------------------------
gi|487825283|ref|WP_001898749.1|restrictionendonucleaseEco57I[Helicobacterpylori]_gi|393107526|gb|EJC08068.1|hypotheticalprotei        ------------------------------------------------------------
gi|447079008|ref|WP_001156264.1|restrictionendonucleaseEco57I[Helicobacterpylori]_gi|393058636|gb|EJB59524.1|hypotheticalprotei        ------------------------------------------------------------
gi|447079015|ref|WP_001156271.1|restrictionendonucleaseEco57I[Helicobacterpylori]_gi|307637357|gb|ADN79807.1|hypotheticalprotei        ------------------------------------------------------------
gi|487884861|ref|WP_001958327.1|helicase[Helicobacterpylori]_gi|459535535|gb|EMH26834.1|helicaseprotein[HelicobacterpyloriGAM26        ------------------------------------------------------------
gi|487886365|ref|WP_001959831.1|helicase[Helicobacterpylori]_gi|459538727|gb|EMH29861.1|helicaseprotein[HelicobacterpyloriGAM27        ------------------------------------------------------------
gi|545301637|ref|WP_021581412.1|helicase[Helicobacterpylori]_gi|535931889|gb|ERA61745.1|helicaseprotein[HelicobacterpyloriGAM11        ------------------------------------------------------------
gi|447079010|ref|WP_001156266.1|restrictionendonucleaseEco57I[Helicobacterpylori]_gi|393095477|gb|EJB96081.1|hypotheticalprotei        ------------------------------------------------------------
gi|447079009|ref|WP_001156265.1|restrictionendonucleaseEco57I[Helicobacterpylori]_gi|393056294|gb|EJB57206.1|hypotheticalprotei        ------------------------------------------------------------
gi|471386985|gb|EMR58906.1|restrictionendonucleasefamilyprotein[HelicobacterpyloriHpH1]/11504        ------------------------------------------------------------
gi|487875679|ref|WP_001949145.1|helicase[Helicobacterpylori]_gi|459516928|gb|EMH09091.1|helicaseprotein[HelicobacterpyloriGAM25        ------------------------------------------------------------
gi|496130066|ref|WP_008854573.1|DNAdamageinducibleprotein[Commensalibacterintestini]_gi|353671689|gb|EHD13391.1|helicasedomain/        ------------------------------------------------------------
gi|924898894|ref|WP_053556546.1|damageinducibleprotein[Sphingopyxissp.113P3]_gi|924523996|gb|ALC14732.1|damageinducibleprotei/1        ------------------------------------------------------------
gi|780802950|gb|KJS08562.1|damageinducibleprotein[Hoefleasp.BRH_c9]/11633        ------------------------------------------------------------
gi|944671432|gb|KQN22886.1|damageinducibleprotein[Sphingomonassp.Leaf32]/11415        ------------------------------------------------------------
gi|944612953|gb|KQM64772.1|damageinducibleprotein[Sphingomonassp.Leaf16]/11726        ------------------------------------------------------------
gi|764353851|ref|WP_044331468.1|damageinducibleprotein[Sphingomonashengshuiensis]_gi|761897338|gb|AJP71707.1|damageinduciblep/1        ------------------------------------------------------------
gi|945874472|gb|KQZ27973.1|damageinducibleprotein[Mesorhizobiumsp.Root552]/11651        ------------------------------------------------------------
gi|759408081|ref|WP_043132403.1|damageinducibleprotein[Paracoccussanguinis]_gi|694217086|gb|KGJ14281.1|damageinducibleprotein/1        ------------------------------------------------------------
gi|654342671|ref|WP_027836116.1|damageinducibleprotein[Maritaleamyrionectae]/11632        ------------------------------------------------------------
gi|126717687|gb|EBA14409.1|hypotheticalproteinRSK20926_02032[Roseobactersp.SK20926]/11603        ------------------------------------------------------------
gi|765301116|ref|WP_044628780.1|damageinducibleprotein[Halomonasmeridiana]_gi|764089679|gb|KJD19875.1|damageinducibleprotein[/1        ------------------------------------------------------------
gi|518053006|ref|WP_019223214.1|DNAdamageinducibleprotein[Bartonellarattaustraliani]/11654        ------------------------------------------------------------
gi|493637806|ref|WP_006589578.1|DNAdamageinducibleprotein[Bartonellabirtlesii]_gi|395410345|gb|EJF76900.1|hypotheticalproteinM/        ------------------------------------------------------------
gi|501188603|ref|WP_012231622.1|DNAdamageinducibleprotein[Bartonellatribocorum]_gi|161017843|emb|CAK01401.1|helicase/methyltra/        ------------------------------------------------------------
gi|651388616|ref|WP_026500615.1|damageinducibleprotein[Bartonellagrahamii]/11655        ------------------------------------------------------------
gi|501188669|ref|WP_012231687.1|DNAdamageinducibleprotein[Bartonellatribocorum]_gi|161017922|emb|CAK01480.1|helicase/methyltra/        ------------------------------------------------------------
gi|518048832|ref|WP_019219040.1|DNAdamageinducibleprotein[Bartonellaflorencae]/11655        ------------------------------------------------------------
gi|518053617|ref|WP_019223825.1|DNAdamageinducibleprotein[Bartonellarattaustraliani]/11607        ------------------------------------------------------------
gi|506336449|ref|WP_015856168.1|DNAdamageinducibleprotein[Bartonellagrahamii]_gi|240267492|gb|ACS51080.1|helicase/methyltransf/        ------------------------------------------------------------
gi|494588832|ref|WP_007347427.1|DNAdamageinducibleprotein[Bartonellarattimassiliensis]_gi|395419053|gb|EJF85361.1|hypothetical/        ------------------------------------------------------------
gi|501188650|ref|WP_012231668.1|DNAdamageinducibleprotein[Bartonellatribocorum]_gi|161017902|emb|CAK01460.1|helicase/methyltra/        ------------------------------------------------------------
gi|501188624|ref|WP_012231643.1|DNAdamageinducibleprotein[Bartonellatribocorum]_gi|161017866|emb|CAK01424.1|helicase/methyltra/        ------------------------------------------------------------
gi|518053856|ref|WP_019224064.1|DNAdamageinducibleprotein[Bartonellarattaustraliani]/11625        ------------------------------------------------------------
gi|501188053|ref|WP_012231081.1|DNAdamageinducibleprotein[Bartonellatribocorum]_gi|161017434|emb|CAK00992.1|helicase/methyltra/        ------------------------------------------------------------
gi|651388630|ref|WP_026500626.1|damageinducibleprotein[Bartonellagrahamii]/11657        ------------------------------------------------------------
gi|518048855|ref|WP_019219063.1|DNAdamageinducibleprotein[Bartonellaflorencae]/11658        ------------------------------------------------------------
gi|490995101|ref|WP_004856829.1|DNAdamageinducibleprotein[Bartonelladoshiae]_gi|395413167|gb|EJF79645.1|hypotheticalproteinMCS/        ------------------------------------------------------------
gi|491002558|ref|WP_004864279.1|DNAdamageinducibleprotein[Bartonellavinsonii]_gi|395421392|gb|EJF87643.1|hypotheticalproteinME/        ------------------------------------------------------------
gi|491003373|ref|WP_004865094.1|DNAdamageinducibleprotein[Bartonellavinsonii]_gi|395432864|gb|EJF98838.1|hypotheticalproteinME/        ------------------------------------------------------------
gi|491002426|ref|WP_004864148.1|DNAdamageinducibleprotein[Bartonellavinsonii]_gi|395421767|gb|EJF88003.1|hypotheticalproteinME/        ------------------------------------------------------------
gi|491004024|ref|WP_004865745.1|DNAdamageinducibleprotein[Bartonellavinsonii]_gi|395432542|gb|EJF98522.1|hypotheticalproteinME/        ------------------------------------------------------------
gi|648396442|ref|WP_026088193.1|damageinducibleprotein[Bartonellarattaustraliani]/11593        ------------------------------------------------------------
gi|748190174|ref|WP_039761290.1|damageinducibleprotein[Bartonellaqueenslandensis]/11658        ------------------------------------------------------------
gi|494817599|ref|WP_007553007.1|DNAdamageinducibleprotein[Bartonellasp.DB56]_gi|395411312|gb|EJF77837.1|hypotheticalproteinMC/1        ------------------------------------------------------------
gi|515763376|ref|WP_017195976.1|DNAdamageinducibleprotein[Bartonellabirtlesii]/11656        ------------------------------------------------------------
gi|494589420|ref|WP_007348014.1|DNAdamageinducibleprotein[Bartonellarattimassiliensis]_gi|395416278|gb|EJF82667.1|hypothetical/        ------------------------------------------------------------
gi|506336469|ref|WP_015856188.1|DNAdamageinducibleprotein[Bartonellagrahamii]_gi|240267512|gb|ACS51100.1|helicase/methyltransf/        ------------------------------------------------------------
gi|749308087|ref|WP_040132227.1|damageinducibleprotein[Bartonellatribocorum]_gi|605051878|emb|CDO49988.1|helicase/methyltransf/        ------------------------------------------------------------
gi|515763800|ref|WP_017196400.1|DNAdamageinducibleprotein[Bartonellabirtlesii]/11658        ------------------------------------------------------------
gi|492186994|ref|WP_005773380.1|DNAdamageinducibleprotein[Bartonellaelizabethae]_gi|395418361|gb|EJF84688.1|hypotheticalprotei/        ------------------------------------------------------------
```

```
gi|501188615|ref|WP_012231634.1|DNAdamageinducibleprotein[Bartonellatribocorum]_gi|161017856|emb|CAK01414.1|helicase/methyltra/      ------------------------------------------------------------
gi|501187470|ref|WP_012230499.1|DNAdamageinducibleprotein[Bartonellatribocorum]_gi|161017090|emb|CAK00648.1|helicase/methyltra/      ------------------------------------------------------------
gi|501188636|ref|WP_012231655.1|DNAdamageinducibleprotein[Bartonellatribocorum]_gi|161017879|emb|CAK01437.1|helicase/methyltra/      ------------------------------------------------------------
gi|930151941|emb|CTQ34818.1|UvsWhelicase[Jannaschiarubra]/11641                                                                      ------------------------------------------------------------
gi|928977106|ref|WP_054001461.1|damageinducibleprotein[Shimiasp.SK013]_gi|927698852|gb|KPA22698.1|typeIrestrictionenzymeEcoKIs/      ------------------------------------------------------------
gi|738872465|ref|WP_036760884.1|damageinducibleprotein[Leisingeradaeponensis]/11643                                                  ------------------------------------------------------------
gi|518050881|ref|WP_019221089.1|DNAdamageinducibleprotein[Bartonellasenegalensis]/11652                                              ------------------------------------------------------------
gi|493980596|ref|WP_006923523.1|DNAdamageinducibleprotein[Bartonellawashoensis]_gi|395413322|gb|EJF79796.1|hypotheticalprotein/      ------------------------------------------------------------
gi|493983106|ref|WP_006925984.1|DNAdamageinducibleprotein[Bartonellawashoensis]_gi|395418127|gb|EJF84455.1|hypotheticalprotein/      ------------------------------------------------------------
gi|499494671|ref|WP_011181311.1|DNAdamageinducibleprotein[Bartonellahenselae]_gi|49239008|emb|CAF28308.1|hypotheticalproteinBH/      ------------------------------------------------------------
gi|651388426|ref|WP_026500441.1|damageinducibleprotein[Bartonellagrahamii]/11652                                                     ------------------------------------------------------------
gi|506337280|ref|WP_015856999.1|DNAdamageinducibleprotein[Bartonellagrahamii]_gi|240268402|gb|ACS51990.1|helicase/methyltransf/      ------------------------------------------------------------
gi|494817535|ref|WP_007552943.1|DNAdamageinducibleprotein[Bartonellasp.DB56]_gi|395412002|gb|EJF78518.1|hypotheticalproteinMC/1      ------------------------------------------------------------
gi|493638099|ref|WP_006589864.1|DNAdamageinducibleprotein[Bartonellabirtlesii]_gi|395409809|gb|EJF76394.1|hypotheticalproteinM/      ------------------------------------------------------------
gi|748189589|ref|WP_039760707.1|damageinducibleprotein[Bartonellaqueenslandensis]/11651                                              ------------------------------------------------------------
gi|501189483|ref|WP_012232501.1|DNAdamageinducibleprotein[Bartonellatribocorum]_gi|161018902|emb|CAK02460.1|helicase/methyltra/      ------------------------------------------------------------
gi|518049181|ref|WP_019219389.1|DNAdamageinducibleprotein[Bartonellaflorencae]/11652                                                 ------------------------------------------------------------
gi|492193255|ref|WP_005774528.1|DNAdamageinducibleprotein[Bartonellaelizabethae]_gi|395416403|gb|EJF82779.1|hypotheticalprotei/      ------------------------------------------------------------
gi|498387325|ref|WP_010701288.1|helicase/methyltransferase[Bartonellabovis]_gi|478418724|gb|ENN92426.1|helicase/methyltransfera      ------------------------------------------------------------
gi|319408697|emb|CBI82352.1|conservedhypotheticalprotein[BartonellaschoenbuchensisR1]/11661                                          ------------------------------------------------------------
gi|494741726|ref|WP_007477134.1|DNAdamageinducibleprotein[Bartonellamelophagi]_gi|395423925|gb|EJF90113.1|hypotheticalproteinM/      ------------------------------------------------------------
gi|737261405|ref|WP_035245349.1|damageinducibleprotein[Alcanivoraxjadensis]_gi|686989709|gb|KGD62556.1|hypotheticalproteinT9A_/      ------------------------------------------------------------
gi|916687564|ref|WP_051294655.1|damageinducibleprotein[Gemmobacternectariphilus]/11641                                               ------------------------------------------------------------
gi|740294127|ref|WP_038132495.1|damageinducibleprotein[Thioclavasp.DT234]_gi|660524826|gb|KEO54689.1|hypotheticalproteinDT23_/1      ------------------------------------------------------------
gi|568331062|ref|WP_024099615.1|helicase[Phaeobactergallaeciensis]_gi|568232121|gb|AHD12007.1|putativehelicase[Phaeobactergalla      ------------------------------------------------------------
gi|518810459|ref|WP_019966413.1|DNAdamageinducibleprotein[Pannonibacterphragmitetus]/11635                                           ------------------------------------------------------------
gi|495305760|ref|WP_008030510.1|DNAdamageinducibleprotein[Rhodobactersp.SW2]_gi|259021698|gb|EEW25000.1|typeIIIrestrictionprot/      ------------------------------------------------------------
gi|506403303|ref|WP_015923022.1|DNAdamageinducibleprotein[Rhodobactersphaeroides]_gi|221162951|gb|ACM03922.1|HypotheticalProte/      ------------------------------------------------------------
gi|738818386|ref|WP_036708790.1|damageinducibleprotein[Paracoccussanguinis]_gi|694226786|gb|KGJ22541.1|damageinducibleprotein/1      ------------------------------------------------------------
gi|746096057|ref|WP_039162453.1|damageinducibleprotein[Gallibacteriumanatis]_gi|703617775|gb|KGQ63868.1|damageinducibleprotei/1      ------------------------------------------------------------
gi|737716785|ref|WP_035685631.1|damageinducibleprotein[Avibacteriumparagallinarum]/11643                                             ------------------------------------------------------------
gi|540072553|gb|ERF78672.1|hypotheticalproteinN561_04930[Gallibacteriumanatis12656/12}/11651                                         ------------------------------------------------------------
gi|917509519|ref|WP_052115936.1|damageinducibleprotein[Gallibacteriumanatis]_gi|702414161|gb|KGQ42233.1|damageinducibleprotei/1      ------------------------------------------------------------
gi|746082942|ref|WP_039150268.1|damageinducibleprotein[Gallibacteriumanatis]_gi|703617078|gb|KGQ63194.1|damageinducibleprotei/1      ------------------------------------------------------------
gi|822522885|ref|WP_046952840.1|damageinducibleprotein[Haemophilushaemolyticus]_gi|821501762|gb|KKZ59267.1|damageinduciblepro/1      ------------------------------------------------------------
gi|404381014|gb|EJZ77501.1|putativehelicase[Pasteurellamultocidasubsp.gallicidaP1059]/11681                                          ------------------------------------------------------------
gi|298281660|gb|EFI23149.1|helicaseCterminaldomainprotein[Neisseriasp.oraltaxon014str.F0314]/11493                                   ------------------------------------------------------------
gi|348013055|gb|EGY51980.1|helicasedomainprotein[Neisseriashayeganii871]/11481                                                       ------------------------------------------------------------
gi|496407140|ref|WP_009116004.1|DNAdamageinducibleprotein[Neisseriawadsworthii]_gi|349794756|gb|EGZ48566.1|helicasedomainprote/      ------------------------------------------------------------
gi|517216441|ref|WP_018405259.1|DNAdamageinducibleprotein[Marinobacterlipolyticus]/11656                                             ------------------------------------------------------------
gi|738230264|ref|WP_036185691.1|damageinducibleprotein[Marinobactersp.ELB17]/11671                                                   ------------------------------------------------------------
gi|447197741|ref|WP_001274997.1|DNAdamageinducibleprotein[Acinetobacterbaumannii]_gi|408512324|gb|EKK13969.1|typeIIIrestrictio/      ------------------------------------------------------------
gi|820775897|ref|WP_046739909.1|damageinducibleprotein[Acinetobactersp.AG1]_gi|819909758|gb|KKW75938.1|damageinducibleprotein/1      ------------------------------------------------------------
gi|584595163|ref|WP_024160846.1|helicase[Acinetobacterpittii]_gi|576702530|emb|CDI28049.1|helicasedomaincontainingprotein[Acin/      ------------------------------------------------------------
gi|514963042|ref|WP_016651366.1|hypotheticalprotein[Acinetobactersp.NIPH2036]_gi|514162665|gb|EPG41391.1|hypotheticalproteinF90      ------------------------------------------------------------
gi|515965564|ref|WP_017396147.1|DNAdamageinducibleprotein[Acinetobacterhaemolyticus]/11667                                           ------------------------------------------------------------
gi|491346810|ref|WP_005204747.1|MULTISPECIES:hypotheticalprotein[Acinetobacter]_gi|480335823|gb|ENX56033.1|hypotheticalproteinF      ------------------------------------------------------------
gi|693044472|ref|WP_032215268.1|damageinducibleprotein[Escherichiacoli]_gi|607725731|gb|EZE08230.1|damageinducibleprotein[Esc/1      ------------------------------------------------------------
gi|742382177|ref|WP_038861304.1|damageinducibleprotein[Cronobactermuytjensii]_gi|924333747|gb|ALB69766.1|damageinducibleprote/1      ------------------------------------------------------------
gi|915912804|ref|WP_050927035.1|damageinducibleprotein[Yersiniaenterocolitica]_gi|899805802|emb|CQH32042.1|typeIrestrictionenz/      ------------------------------------------------------------
gi|921111327|gb|KON62781.1|typeIrestrictionenzymeEcoKIsubunitR[Komagataeibactereuropaeus]/11492                                      ------------------------------------------------------------
gi|582024278|gb|AHI27615.1|DNAhelicaserestrictionenzymeTypeIIIRsubunit[KomagataeibacterxylinusE25]/11636                             ------------------------------------------------------------
gi|347662673|dbj|BAK86128.1|DNAhelicaserestrictionenzymeTypeIIIRsubunit[KomagataeibactermedellinensisNBRC3288]/11737                 ------------------------------------------------------------
gi|917445053|ref|WP_052051471.1|damageinducibleprotein[Acetobactertropicalis]/11429                                                  ------------------------------------------------------------
gi|453329445|dbj|GAC88472.1|DNAhelicaserestrictionenzymetypeIIIRsubunit[GluconobacterthailandicusNBRC3255]_gi|530287981|dbj|GAD      ------------------------------------------------------------
gi|889770922|ref|WP_048844278.1|damageinducibleprotein[Gluconobacterfrateurii]/11419                                                 ------------------------------------------------------------
gi|504886208|ref|WP_015073310.1|hypotheticalprotein[Gluconobacteroxydans]_gi|411027954|gb|AFW01209.1|hypotheticalproteinB932_16      ------------------------------------------------------------
gi|480040211|gb|ENV79751.1|hypotheticalproteinF942_01641[AcinetobacterursingiiANC3649]/11432                                         ------------------------------------------------------------
gi|822498222|ref|WP_046936504.1|damageinducibleprotein[Xanthomonasgardneri]_gi|821751224|gb|KLB07223.1|damageinducibleprotein/1      ------------------------------------------------------------
gi|654059559|ref|WP_027705556.1|damageinducibleprotein[Zymobacterpalmae]/11635                                                       ------------------------------------------------------------
gi|643535620|ref|WP_025224315.1|damageinducibleprotein[Methylophilaceaebacterium11]_gi|577704400|gb|EUJ10546.1|putativehelicas/      ------------------------------------------------------------
MBA11I/11635                                                                                                                          ------------------------------------------------------------
gi|655355857|ref|WP_028762287.1|damageinducibleprotein[Shewanellacolwelliana]/11632                                                  ------------------------------------------------------------
gi|753204236|ref|WP_041510579.1|damageinducibleprotein[Shewanellasp.cp20]_gi|752457765|gb|KIO36326.1|damageinducibleprotein[S/1      ------------------------------------------------------------
KPNNIH30III/11619                                                                                                                     ------------------------------------------------------------
gi|749295994|ref|WP_040120352.1|damageinducibleprotein[Klebsiellapneumoniae]_gi|723249300|gb|AIX81926.1|damageinducibleprotei/1      ------------------------------------------------------------
gi|641639739|emb|CDQ54010.1|unnamedproteinproduct[Klebsiellapneumoniae]/11641                                                        ------------------------------------------------------------
```

```
gi|490797922|ref|WP_004660060.1|hypotheticalprotein[Acinetobactersp.NIPH236]_gi|479876779|gb|ENU27973.1|hypotheticalproteinF992      ------------------------------------------------------------
gi|916750926|ref|WP_051357982.1|damageinducibleprotein[Acinetobactertjernbergiae]/11453      ------------------------------------------------------------
gi|691098662|ref|WP_032025060.1|damageinducibleprotein[Acinetobacterbaumannii]_gi|593567521|gb|EXR50735.1|restrictionendonucle/      ------------------------------------------------------------
gi|736669405|ref|WP_034676049.1|damageinducibleprotein[Acinetobacterbaumannii]/11643      ------------------------------------------------------------
gi|491368928|ref|WP_005226826.1|hypotheticalprotein[Acinetobactersp.NIPH1847]_gi|480173259|gb|ENX09797.1|hypotheticalproteinF89      ------------------------------------------------------------
gi|796853993|ref|WP_045795953.1|damageinducibleprotein[Acinetobacterindicus]_gi|787929761|gb|KJV44219.1|damageinducibleprotei/1      ------------------------------------------------------------
gi|295797463|gb|ADG32253.1|typeIIIrestrictionproteinressubunit[ThiomonasintermediaK12]/11676      ------------------------------------------------------------
gi|499871179|ref|WP_011551913.1|DNAdamageinducibleprotein[Myxococcusxanthus]_gi|108467544|gb|ABF92729.1|putativerestriction/mo/      ------------------------------------------------------------
gi|495313852|ref|WP_008038600.1|DNAdamageinducibleprotein[Bartonellatamiae]_gi|395424181|gb|EJF90368.1|hypotheticalproteinME5_/      ------------------------------------------------------------
gi|501453335|ref|WP_012476784.1|DNAdamageinducibleprotein[Leptospirabiflexa]_gi|167777824|gb|ABZ96123.1|Helicase[Leptospirabif/      ------------------------------------------------------------
gi|738104613|ref|WP_036063193.1|damageinducibleprotein[Leptospiranoguchii]/11652      ------------------------------------------------------------
gi|410001039|gb|EKO51661.1|typeIIIrestrictionenzyme,ressubunit[Leptospirakirschneristr.200802841]/11651      ------------------------------------------------------------
gi|490901851|ref|WP_004763774.1|typeIIIrestrictionenzyme,ressubunit[Leptospirakirschneri]_gi|464209249|gb|EMN24014.1|typeIIIres      ------------------------------------------------------------
gi|464398131|gb|EMO65902.1|typeIIIrestrictionenzyme,ressubunit[Leptospirakirschneristr.200803703]/11648      ------------------------------------------------------------
gi|945029024|gb|KQQ84900.1|damageinducibleprotein[Xanthomonassp.Leaf131]/11423      ------------------------------------------------------------
gi|896511314|ref|WP_049418183.1|damageinducibleprotein[Stenotrophomonasmaltophilia]/11639      ------------------------------------------------------------
gi|896513082|ref|WP_049419616.1|damageinducibleprotein[Stenotrophomonasmaltophilia]/11638      ------------------------------------------------------------
gi|896580616|ref|WP_049469425.1|damageinducibleprotein[Stenotrophomonasmaltophilia]/11641      ------------------------------------------------------------
gi|648289350|ref|WP_026070499.1|damageinducibleprotein[Stenotrophomonasmaltophilia]/11639      ------------------------------------------------------------
gi|656272786|ref|WP_029218442.1|damageinducibleprotein[Xanthomonascassavae]/11639      ------------------------------------------------------------
gi|406976236|gb|EKD98747.1|hypotheticalproteinACD_23C00290G0002[unculturedbacterium]/11679      ------------------------------------------------------------
gi|828145737|ref|WP_047238120.1|damageinducibleprotein[Chromobacteriumsubtsugae]/11645      ------------------------------------------------------------
gi|654311174|ref|WP_027824327.1|damageinducibleprotein[Laribacterhongkongensis]/11641      ------------------------------------------------------------
gi|757242173|gb|KIR15516.1|typeIrestrictionenzymeEcoKIsubunitR[Pseudomonasfluorescens]/11778      ------------------------------------------------------------
gi|653605161|ref|WP_027616682.1|damageinducibleprotein[Pseudomonassp.URHB0015]/11639      ------------------------------------------------------------
gi|520868025|ref|WP_020308060.1|helicase[Pseudomonassyringae]_gi|519159034|gb|EPM49935.1|helicasedomaincontainingprotein[Pseud/      ------------------------------------------------------------
gi|940156567|gb|KPX71668.1|Helicasedomaincontainingprotein[Pseudomonasamygdalipv.lachrymans]/11677      ------------------------------------------------------------
gi|505460508|ref|WP_015647610.1|helicase[Pseudomonassp.GLE121]_gi|499110212|ref|YP_007974374.1|helicasedomaincontainingprotein/      ------------------------------------------------------------
gi|940235551|gb|KPY45311.1|hypotheticalproteinALO48_200154[Pseudomonassyringaepv.rhaphiolepidis]/11631      ------------------------------------------------------------
gi|520880808|ref|WP_020312235.1|helicase[Pseudomonassyringae]_gi|519172158|gb|EPM62027.1|helicasedomaincontainingprotein[Pseud/      ------------------------------------------------------------
gi|829046479|ref|WP_047282259.1|damageinducibleprotein[Pseudomonasfluorescens]/11630      ------------------------------------------------------------
gi|499415493|ref|WP_011102960.1|DNAdamageinducibleprotein[Pseudomonassyringaegroupgenomosp.3]_gi|28867279|ref|NP_789898.1|heli/      ------------------------------------------------------------
gi|730238299|ref|WP_033942897.1|damageinducibleprotein[Pseudomonasaeruginosa]/11629      ------------------------------------------------------------
gi|495310125|ref|WP_008034873.1|DNAdamageinducibleprotein[Pseudomonassp.GM60]_gi|398186685|gb|EJM74052.1|putativehelicase[Pseu/      ------------------------------------------------------------
gi|779992653|ref|WP_045428690.1|damageinducibleprotein[Pseudomonassp.MT1]_gi|699967872|dbj|BAP80334.1|DNAhelicaserestrictione/1      ------------------------------------------------------------
gi|786037289|ref|WP_045733274.1|damageinducibleprotein[Pseudomonaspseudoalcaligenes]_gi|782987904|gb|KJU79152.1|damageinducib/1      ------------------------------------------------------------
gi|749666366|ref|WP_040265445.1|damageinducibleprotein[Pseudomonasrhodesiae]/11632      ------------------------------------------------------------
gi|734902433|ref|WP_034133698.1|damageinducibleprotein[Pseudomonasfluorescens]/11635      ------------------------------------------------------------
gi|490551031|ref|WP_004416146.1|typeIIrestrictionmodificationsystemrestriction/helicasesubunit[Pseudomonassyringae]_gi|4432823/      ------------------------------------------------------------
gi|489199911|ref|WP_003109152.1|DNAdamageinducibleprotein[Pseudomonasaeruginosa]_gi|552648708|gb|ERY63184.1|hypotheticalprotei/      ------------------------------------------------------------
gi|730641237|ref|WP_034061328.1|damageinducibleprotein[Pseudomonasaeruginosa]/11636      ------------------------------------------------------------
gi|751304980|ref|WP_041013386.1|damageinducibleprotein[Pseudomonasxanthomarina]_gi|703407611|emb|CEG51014.1|Helicasedomaincon/1      ------------------------------------------------------------
gi|939335207|ref|WP_054881520.1|damageinducibleprotein[Pseudomonassp.NBRC111128]/11632      ------------------------------------------------------------
gi|757693555|ref|WP_042923792.1|damageinducibleprotein[Pseudomonaschloritidismutans]_gi|780910610|gb|KJS78302.1|damageinducib/1      ------------------------------------------------------------
gi|665953944|ref|WP_031319989.1|damageinducibleprotein[Pseudomonastolaasii]/11626      ------------------------------------------------------------
gi|771600573|ref|WP_045213746.1|damageinducibleprotein[Pseudomonassp.21]_gi|770081123|gb|KJJ98634.1|damageinducibleprotein[Ps/1      ------------------------------------------------------------
gi|740871396|ref|WP_038656644.1|damageinducibleprotein[Pseudomonasstutzeri]_gi|627772214|gb|AHY41437.1|damageinducibleprotein/1      ------------------------------------------------------------
gi|946242205|gb|KRC93782.1|damageinducibleprotein[Pseudomonassp.Root9]/11634      ------------------------------------------------------------
gi|553777322|ref|WP_023109107.1|MULTISPECIES:hypotheticalprotein[Pseudomonas]_gi|552638674|gb|ERY53184.1|hypotheticalproteinQ05      ------------------------------------------------------------
gi|896383370|ref|WP_049324618.1|damageinducibleprotein[Pseudomonasstutzeri]/11627      ------------------------------------------------------------
gi|696158510|gb|AIT41787.1|putativehelicase[Pseudomonassp.LM8]/11630      ------------------------------------------------------------
gi|504634892|ref|WP_014821994.1|DNAdamageinducibleprotein[Pseudomonasstutzeri]_gi|390985249|gb|AFM35242.1|helicasedomainconta/1      ------------------------------------------------------------
gi|544783974|ref|WP_021206729.1|DNAdamageinducibleprotein[Pseudomonasstutzeri]_gi|533224779|gb|EQM79688.1|DNAdamageinduciblep/1      ------------------------------------------------------------
gi|504567385|ref|WP_014754487.1|MULTISPECIES:DNAdamageinducibleprotein[Pseudomonas]_gi|388559493|gb|AFK68634.1|helicasedomain/1      ------------------------------------------------------------
gi|654441469|ref|WP_027912052.1|damageinducibleprotein[Pseudomonassp.URIL14HWK12:I7]/11630      ------------------------------------------------------------
gi|941470066|ref|WP_055127894.1|damageinducibleprotein[Pseudomonasmediterranea]/11631      ------------------------------------------------------------
gi|557815599|ref|WP_023446184.1|DNAdamageinducibleprotein[Pseudomonaschloritidismutans]_gi|557364081|gb|ESQ98190.1|DNAdamagei/1      ------------------------------------------------------------
gi|727814791|ref|WP_033898247.1|damageinducibleprotein[Pseudomonassp.FH1]_gi|567648005|gb|ETK23229.1|helicasedomaincontaining/1      ------------------------------------------------------------
gi|503278702|ref|WP_013513363.1|DNAdamageinducibleprotein[Desulfovibrioaespoeensis]_gi|316942375|gb|ADU61426.1|typeIIIrestrict/      ------------------------------------------------------------
gi|505228796|ref|WP_015415898.1|typeIIIrestrictionendonucleasesubunitR[Desulfovibriopiezophilus]_gi|451896976|emb|CCH49855.1|Ty      ------------------------------------------------------------
gi|517732543|ref|WP_018902751.1|hypotheticalprotein[Variovoraxparadoxus]/11675      ------------------------------------------------------------
gi|550909475|ref|WP_022662570.1|DNAdamageinducibleprotein[Desulfovibriolongus]/11637      ------------------------------------------------------------
gi|945494542|gb|KQV50024.1|damageinducibleprotein[Massiliasp.Root335]/11647      ------------------------------------------------------------
gi|334196917|gb|AEG70102.1|helicasedomainprotein[RalstoniasolanacearumPo82]/11663      ------------------------------------------------------------
gi|656006746|ref|WP_029048170.1|damageinducibleprotein[Cupriavidussp.amp6]/11661      ------------------------------------------------------------
BPS2196ORF3054P/11659      ------------------------------------------------------------
gi|740932312|ref|WP_038717332.1|damageinducibleprotein[Burkholderiapseudomallei]_gi|760211882|gb|KIX58314.1|damageinduciblepr/1      ------------------------------------------------------------
```

```
gi|755810813|ref|WP_042583861.1|damageinducibleprotein[Burkholderiasp.MSHR3999]_gi|755174952|gb|KIP19790.1|DEAD/DEAHboxhelicas/      ------------------------------------------------------------
gi|484586570|gb|AGK48195.1|DEAD/DEAHboxhelicasefamilyprotein[BurkholderiathailandensisMSMB121]/11646                                  ------------------------------------------------------------
gi|737582935|ref|WP_035553941.1|damageinducibleprotein[Burkholderiasp.9120]/11658                                                     ------------------------------------------------------------
gi|740989872|ref|WP_038773225.1|damageinducibleprotein[Burkholderiapseudomallei]_gi|705645291|gb|KGS70691.1|DEAD/DEAHboxhelica/      ------------------------------------------------------------
gi|654912525|ref|WP_028363424.1|damageinducibleprotein[Burkholderiasp.JPY366]/11659                                                   ------------------------------------------------------------
gi|740955879|ref|WP_038740512.1|damageinducibleprotein[Burkholderiapseudomallei]_gi|714684932|gb|KGW74438.1|DEAD/DEAHboxhelica/      ------------------------------------------------------------
gi|919203714|ref|WP_052759215.1|damageinducibleprotein[Xanthomonasaxonopodis]/11388                                                   ------------------------------------------------------------
gi|522152381|ref|WP_020663589.1|hypotheticalprotein[Amycolatopsisbenzoatilytica]/11605                                               ------------------------------------------------------------
gi|663196013|ref|WP_030233899.1|damageinducibleprotein[Streptomycessp.NRRLS455]/11641                                                 ------------------------------------------------------------
gi|750429553|ref|WP_040711444.1|damageinducibleprotein[Nocardiopsispotens]/11622                                                      ------------------------------------------------------------
gi|518656139|ref|WP_019819475.1|hypotheticalprotein[Saccharomonosporasaliphila]/11623                                                 ------------------------------------------------------------
gi|759722933|ref|WP_043433758.1|damageinducibleprotein[Streptomycespluripotens]_gi|733404978|gb|KHK94361.1|damageinduciblepro/1      ------------------------------------------------------------
gi|663424167|ref|WP_030419030.1|damageinducibleprotein[Streptomycessp.NRRLF5065]/11627                                                ------------------------------------------------------------
gi|663412917|ref|WP_030408353.1|damageinducibleprotein[Streptomycessp.NRRLF5917]/11630                                                ------------------------------------------------------------
gi|671521483|ref|WP_031506489.1|damageinducibleprotein[Streptomycesmegasporus]/11630                                                  ------------------------------------------------------------
gi|740053144|ref|WP_037902776.1|damageinducibleprotein[Streptomycessp.TAA204]/11626                                                   ------------------------------------------------------------
gi|663409375|ref|WP_030404974.1|MULTISPECIES:damageinducibleprotein[Streptomyces]/11627                                               ------------------------------------------------------------
gi|746628608|ref|WP_039654277.1|damageinducibleprotein[Streptomycessp.MUSC125]_gi|745644381|gb|KIE25557.1|damageinducibleprot/1      ------------------------------------------------------------
gi|727322066|ref|WP_033777320.1|damageinducibleprotein[Salinisporapacifica]/11627                                                     ------------------------------------------------------------
gi|653653242|ref|WP_027655726.1|damageinducibleprotein[Salinisporapacifica]/11618                                                     ------------------------------------------------------------
gi|648632543|ref|WP_026324294.1|damageinducibleprotein[Salinisporapacifica]/11621                                                     ------------------------------------------------------------
gi|881018002|ref|WP_048738463.1|damageinducibleprotein[Corynebacteriumfalsenii]/11676                                                 ------------------------------------------------------------
gi|505465526|ref|WP_015652417.1|helicase[Corynebacteriumcallunae]_gi|459385704|gb|AGG67995.1|helicase[CorynebacteriumcallunaeDS      ------------------------------------------------------------
gi|41223062|emb|CAF19007.1|DNAorRNAhelicaseofsuperfamilyII[CorynebacteriumglutamicumATCC13032]/11646                                 ------------------------------------------------------------
CGL13032I/11643                                                                                                                       ------------------------------------------------------------
gi|751257081|ref|WP_040967675.1|damageinducibleprotein[Corynebacteriumglutamicum]_gi|748811620|gb|AJE67931.1|damageinduciblep/1      ------------------------------------------------------------
gi|749040539|ref|WP_040086524.1|damageinducibleprotein[Corynebacteriumhumireducens]/11663                                             ------------------------------------------------------------
gi|491662899|ref|WP_005519615.1|helicase[Corynebacteriummatruchotii]_gi|224946756|gb|EEG27965.1|helicaseCterminaldomainprotein/      ------------------------------------------------------------
gi|746717312|ref|WP_039676231.1|damageinducibleprotein[Corynebacteriumminutissimum]_gi|734991118|gb|KHO29219.1|damageinducibl/1      ------------------------------------------------------------
gi|908654892|emb|CQD15529.1|SuperfamilyIIDNA/RNAhelicase(modularprotein)[Corynebacteriumstriatum]/11674                              ------------------------------------------------------------
gi|895994446|ref|WP_049064418.1|damageinducibleprotein[Corynebacteriumaurimucosum]/11667                                              ------------------------------------------------------------
gi|896123331|ref|WP_049145390.1|damageinducibleprotein[Corynebacteriumaurimucosum]/11669                                              ------------------------------------------------------------
gi|817681178|ref|WP_046645422.1|damageinducibleprotein[Corynebacteriumstriatum]_gi|817551679|gb|KKO79868.1|damageinduciblepro/1      ------------------------------------------------------------
gi|896139239|ref|WP_049159146.1|damageinducibleprotein[Corynebacteriumaurimucosum]/11675                                              ------------------------------------------------------------
gi|489943947|ref|WP_003847254.1|helicase[Corynebacteriumammoniagenes]_gi|295968218|gb|EFG81467.1|helicaseCterminaldomainprotei/      ------------------------------------------------------------
gi|516653464|ref|WP_018022453.1|helicase[Corynebacteriumdoosanense]_gi|697778282|gb|AIT61018.1|damageinducibleprotein[Coryneba/      ------------------------------------------------------------
gi|493894179|ref|WP_006840129.1|helicase[Corynebacteriumlipophiloflavum]_gi|227078819|gb|EEI16782.1|helicaseCterminaldomainpro/      ------------------------------------------------------------
gi|740831472|ref|WP_038616755.1|damageinducibleprotein[Corynebacteriumpseudotuberculosis]_gi|666103058|gb|AIG06935.1|putativeh/      ------------------------------------------------------------
gi|518023322|ref|WP_019193530.1|helicase[Corynebacteriumtimonense]/11655                                                              ------------------------------------------------------------
gi|552767697|ref|WP_023019559.1|MULTISPECIES:hypotheticalprotein[Corynebacterium]_gi|550739013|gb|ERS38609.1|hypotheticalprotei      ------------------------------------------------------------
gi|652659121|ref|WP_027017812.1|damageinducibleprotein[Corynebacteriumpseudodiphtheriticum]/11644                                     ------------------------------------------------------------
gi|749039309|ref|WP_040085390.1|damageinducibleprotein[Corynebacteriumhumireducens]/11657                                             ------------------------------------------------------------
gi|740835591|ref|WP_038620874.1|damageinducibleprotein[Corynebacteriumulcerans]_gi|698973854|gb|AIU33664.1|HelicaseCterminald/1      ------------------------------------------------------------
gi|805307599|ref|WP_046096452.1|damageinducibleprotein[Corynebacteriumulcerans]_gi|803357323|gb|AKA97638.1|Damageinduciblepro/1      ------------------------------------------------------------
gi|504083555|ref|WP_014317549.1|helicase[Corynebacteriumdiphtheriae]_gi|371584249|gb|AEX47914.1|superfamilyIIDNA/RNAhelicase[Co      ------------------------------------------------------------
gi|896182193|ref|WP_049193580.1|damageinducibleprotein[Corynebacteriumjeikeium]/11645                                                 ------------------------------------------------------------
gi|917636459|ref|WP_052205460.1|damageinducibleprotein[Corynebacteriumriegelii]_gi|914295811|gb|AKV59187.1|damageinduciblepro/1      ------------------------------------------------------------
gi|737013522|ref|WP_035009205.1|damageinducibleprotein[Corynebacteriumjeikeium]/11651                                                 ------------------------------------------------------------
gi|737006291|ref|WP_035002094.1|damageinducibleprotein[Corynebacteriumjeikeium]/11658                                                 ------------------------------------------------------------
gi|737010378|ref|WP_035006133.1|damageinducibleprotein[Corynebacteriumjeikeium]/11650                                                 ------------------------------------------------------------
gi|737010473|ref|WP_035006228.1|damageinducibleprotein[Corynebacteriumjeikeium]/11657                                                 ------------------------------------------------------------
gi|737006250|ref|WP_035002053.1|damageinducibleprotein[Corynebacteriumjeikeium]/11658                                                 ------------------------------------------------------------
gi|923018168|ref|WP_053412254.1|damageinducibleprotein[Corynebacteriumlactis]_gi|922845184|gb|ALA67504.1|helicase[Corynebacter/      ------------------------------------------------------------
gi|923018885|ref|WP_053412970.1|damageinducibleprotein[Corynebacteriumlactis]_gi|922845845|gb|ALA68165.1|helicase[Corynebacter/      ------------------------------------------------------------
gi|512464870|ref|WP_016422869.1|hypotheticalprotein[Corynebacteriumsp.HFH0082]_gi|512047830|gb|EPD47505.1|hypotheticalproteinHM      ------------------------------------------------------------
gi|881012697|ref|WP_048733406.1|damageinducibleprotein[Corynebacteriumurealyticum]/11670                                              ------------------------------------------------------------
gi|755608478|ref|WP_042530519.1|damageinducibleprotein[Corynebacteriumsingulare]_gi|754340239|gb|AJI78730.1|putativehelicase[C/      ------------------------------------------------------------
gi|491421796|ref|WP_005279593.1|helicase[Corynebacteriumaccolens]_gi|227077180|gb|EEI15143.1|helicaseCterminaldomainprotein[Co/      ------------------------------------------------------------
gi|550752286|gb|ERS51451.1|hypotheticalproteinHMPREF1282_00033[Corynebacteriumsp.KPL1856]_gi|550759961|gb|ERS58964.1|hypothetic      ------------------------------------------------------------
gi|497955053|ref|WP_010269209.1|helicase[Corynebacteriumbovis]/11599                                                                  ------------------------------------------------------------
gi|860580040|ref|WP_048470569.1|hypotheticalprotein[Mycobacteriumchlorophenolicum]_gi|859098575|gb|KMO76871.1|typeIrestrictione      ------------------------------------------------------------
gi|262085905|gb|ACY21873.1|typeIIIrestrictionproteinressubunit[GordoniabronchialisDSM43247]/11632                                     ------------------------------------------------------------
gi|495996338|ref|WP_008720917.1|typeIIIrestrictionendonucleasesubunitR[Rhodococcussp.AW25M09]_gi|443412350|emb|CCQ18455.1|TypeI      ------------------------------------------------------------
gi|739284636|ref|WP_037147410.1|damageinducibleprotein[Rhodococcusfascians]/11641                                                     ------------------------------------------------------------
gi|739328195|ref|WP_037190574.1|damageinducibleprotein[Rhodococcusfascians]/11643                                                     ------------------------------------------------------------
gi|811698335|emb|CMN21041.1|helicase[Mycobacteriumtuberculosis]/11725                                                                 ------------------------------------------------------------
gi|801577898|emb|CFK23773.1|helicase[Mycobacteriumtuberculosis]/11675                                                                 ------------------------------------------------------------
```

```
MTU18II/11606                                                                                                                          ------------------------------------------------------------
gi|887719858|emb|CMT27886.1|helicase[Mycobacteriumtuberculosis]/11667                                                                  ------------------------------------------------------------
gi|621694743|gb|KAS87156.1|hypotheticalproteinAL70_01801[MycobacteriumtuberculosisTKK_03_0115]/11604                                   ------------------------------------------------------------
gi|888128072|emb|CLQ84422.1|helicase[Mycobacteriumtuberculosis]_gi|889917090|emb|CLY86893.1|helicase[Mycobacteriumtuberculosis]        ------------------------------------------------------------
gi|884823424|emb|CKL96672.1|helicase[Mycobacteriumtuberculosis]/11466                                                                  ------------------------------------------------------------
gi|505103239|ref|WP_015290341.1|hypotheticalprotein[Mycobacteriumcanettii]_gi|432162733|emb|CCK60117.1|Conservedproteinofunknow        ------------------------------------------------------------
gi|505116250|ref|WP_015303352.1|hypotheticalprotein[Mycobacteriumcanettii]_gi|432158726|emb|CCK56026.1|Conservedproteinofunknow        ------------------------------------------------------------
gi|432154735|emb|CCK51974.1|Conservedproteinofunknownfunction(part1)[MycobacteriumcanettiiCIPT140060008]/11588                          ------------------------------------------------------------
gi|809008027|ref|WP_046295375.1|hypotheticalprotein[Mycobacteriumsp.UM_Kg17]/11624                                                     ------------------------------------------------------------
gi|808802633|ref|WP_046284139.1|hypotheticalprotein[Mycobacteriumsp.UM_NZ2]/11624                                                      ------------------------------------------------------------
gi|750125774|ref|WP_040429852.1|damageinducibleprotein[Corynebacteriumnuruki]/11668                                                    ------------------------------------------------------------
gi|602237614|gb|EYT60960.1|damageinducibleprotein[Dietziasp.UCDTHP]/11473                                                              ------------------------------------------------------------
gi|665897412|ref|WP_031264618.1|damageinducibleprotein[Dietziasp.UCDTHP]_gi|602237599|gb|EYT60945.1|helicase[Dietziasp.UCDTH/11        ------------------------------------------------------------
gi|502903674|ref|WP_013138650.1|helicase[Segniliparusrotundus]_gi|296181291|gb|ADG98197.1|typeIIIrestrictionproteinressubunit[S        ------------------------------------------------------------
gi|780248306|ref|WP_045538930.1|hypotheticalprotein[Gordoniasp.no.9]_gi|778142514|gb|KJR06917.1|hypotheticalproteinUG54_12320[G        ------------------------------------------------------------
gi|665897368|ref|WP_031264574.1|damageinducibleprotein[Dietziasp.UCDTHP]_gi|602239137|gb|EYT62483.1|helicase[Dietziasp.UCDTH/11        ------------------------------------------------------------
gi|493372923|ref|WP_006329236.1|helicase[Gordoniarhizosphera]_gi|403207917|dbj|GAB88168.1|hypotheticalproteinGORHZ_006_00370[Go        ------------------------------------------------------------
gi|764904870|ref|WP_044487821.1|hypotheticalprotein[Mycobacteriumtuberculosis]_gi|625005353|gb|KBZ57120.1|hypotheticalproteinK8        ------------------------------------------------------------
gi|927068212|ref|WP_053778441.1|damageinducibleprotein[Gordoniasp.NB41Y]_gi|926660541|gb|EMP14526.2|damageinducibleprotein[G/11        ------------------------------------------------------------
gi|935514169|ref|WP_054429894.1|damageinducibleprotein[Mycobacteriumimmunogenum]_gi|929801120|gb|KPG29247.1|damageinduciblepr/1        ------------------------------------------------------------
gi|941317728|ref|WP_055118574.1|hypotheticalprotein[Mycobacteriumperegrinum]/11657                                                     ------------------------------------------------------------
gi|933683673|gb|ALG86377.1|hypotheticalproteinACH46_20120[Gordoniasp.QH11]/11636                                                       ------------------------------------------------------------
gi|873553947|emb|CRY84110.1|Predictedhelicase[Nocardiafarcinica]/11688                                                                 ------------------------------------------------------------
gi|499525749|ref|WP_011212389.1|helicase[Nocardiafarcinica]_gi|54019339|dbj|BAD60707.1|hypotheticalprotein[NocardiafarcinicaIFM        ------------------------------------------------------------
gi|640109609|ref|WP_024794187.1|damageinducibleprotein[Tomitellabiformata]/11653                                                       ------------------------------------------------------------
gi|640109991|ref|WP_024794334.1|hypotheticalprotein[Tomitellabiformata]/11639                                                          ------------------------------------------------------------
gi|939125496|ref|WP_054831438.1|damageinducibleprotein[Rhodococcuserythropolis]/11669                                                  ------------------------------------------------------------
gi|910258550|ref|WP_050062711.1|hypotheticalprotein[Rhodococcussp.RD6.2]_gi|846871967|emb|CRK49225.1|conservedhypotheticalprote        ------------------------------------------------------------
gi|797148189|gb|KJY52358.1|putativeHelicase[Bifidobacteriumasteroides]/11801                                                          ------------------------------------------------------------
gi|535476570|gb|EFG27357.2|hypotheticalproteinHMPREF9020_00999[ScardoviainopinataF0304]/11827                                          ------------------------------------------------------------
gi|737482729|ref|WP_035462496.1|hypotheticalprotein[Alicyclobacillusmacrosporangiidus]/11512                                           ------------------------------------------------------------
gi|685434354|gb|KGA95679.1|helicase[BacillusalcalophilusATCC27647=CGMCC1.3604]/11575                                                   ------------------------------------------------------------
gi|945370253|gb|KQU27251.1|helicase[Bacillussp.Leaf13]/11557                                                                           ------------------------------------------------------------
gi|880997015|ref|WP_048719116.1|helicase[Enterococcushirae]/11563                                                                      ------------------------------------------------------------
gi|945356929|gb|KQU14100.1|helicase[Bacillussp.Leaf49]/11547                                                                           ------------------------------------------------------------
gi|763482876|gb|KIZ53217.1|helicase[Bacillussafensis]/11563                                                                            ------------------------------------------------------------
gi|852223292|ref|WP_048309707.1|helicase[Anaerobacillusmacyae]_gi|850322910|gb|KMM38590.1|helicase[Anaerobacillusmacyae]/11573         ------------------------------------------------------------
gi|739708665|ref|WP_037562619.1|helicase[Sporolactobacillusterrae]/11573                                                               ------------------------------------------------------------
gi|496687561|ref|WP_009329104.1|MULTISPECIES:helicase[Bacillus]_gi|317390867|gb|EFV71668.1|endonucleaseandmethylaseLlaGI[Bacill        ------------------------------------------------------------
gi|930213599|gb|KPH74225.1|helicase[Oceanobacilluscaeni]/11569                                                                         ------------------------------------------------------------
gi|516366003|ref|WP_017756036.1|helicase[Bacillussp.ZYK]/11571                                                                         ------------------------------------------------------------
gi|488909977|ref|WP_002821052.1|helicase[Oenococcusoeni]_gi|399965230|gb|EJN99855.1|endonucleaseandmethylaseLlaGI[Oenococcusoen        ------------------------------------------------------------
gi|495019967|ref|WP_007745780.1|helicase[Oenococcuskitaharae]_gi|366983682|gb|EHN59081.1|typeIIIrestrictionenzyme,ressubunit[Oe        ------------------------------------------------------------
gi|489666119|ref|WP_003570406.1|typeIIIrestrictionendonuclease[Lactobacilluscasei]_gi|410527834|gb|EKQ02696.1|typeIIIrestrictio        ------------------------------------------------------------
gi|511447623|gb|EPC78110.1|superfamilyIIDNA/RNAhelicase[Lactobacillusparacaseisubsp.paracaseiLpp221}/11615                             ------------------------------------------------------------
gi|497700411|ref|WP_010014595.1|helicase[Lactobacilluscoryniformis]/11585                                                              ------------------------------------------------------------
gi|489784048|ref|WP_003687939.1|helicase[Lactobacillusmali]_gi|394428539|gb|EJF01080.1|superfamilyIIDNA/RNAhelicase[Lactobacill        ------------------------------------------------------------
gi|446780825|ref|WP_000858081.1|helicase[Staphylococcusaureus]_gi|374396604|gb|EHQ67835.1|typeIIIrestrictionenzyme,ressubunit[S        ------------------------------------------------------------
gi|510796658|ref|WP_016171456.1|hypotheticalprotein[Enterococcusdispar]_gi|508167532|gb|EOT44083.1|hypotheticalproteinOMK_00227        ------------------------------------------------------------
gi|612613473|gb|EZS30411.1|hypotheticalproteinW603_02482[StaphylococcusaureusVET0353R]_gi|612881917|gb|EZU93992.1|hypotheticalp        ------------------------------------------------------------
gi|488332893|ref|WP_002402278.1|helicase[Enterococcusfaecalis]_gi|306513864|gb|EFM82466.1|helicaseCterminaldomainprotein[Enter/        ------------------------------------------------------------
gi|921166029|ref|WP_053111806.1|helicase[Enterococcusdurans]_gi|915771660|gb|AKX85928.1|helicase[Enterococcusdurans]/11562            ------------------------------------------------------------
gi|498525358|ref|WP_010825030.1|hypotheticalprotein[Enterococcusfaecalis]_gi|487059631|gb|EOJ86727.1|hypotheticalproteinWOC_005        ------------------------------------------------------------
gi|498412624|ref|WP_010722070.1|hypotheticalprotein[Enterococcusfaecium]_gi|486634654|gb|EOF75253.1|hypotheticalproteinSGC_0253        ------------------------------------------------------------
gi|498408534|ref|WP_010718364.1|hypotheticalprotein[Enterococcusfaecium]_gi|486609608|gb|EOF50873.1|hypotheticalproteinSCW_0257        ------------------------------------------------------------
gi|498407475|ref|WP_010717483.1|hypotheticalprotein[Enterococcusfaecalis]_gi|486594855|gb|EOF36348.1|hypotheticalproteinSCK_005        ------------------------------------------------------------
gi|514885392|ref|WP_016615344.1|helicase[Enterococcusfaecalis]_gi|514505843|gb|EPH71676.1|helicaseprotein[Enterococcusfaecalis0        ------------------------------------------------------------
gi|325157603|gb|EGC69759.1|helicaseCterminaldomainprotein[EnterococcuscasseliflavusATCC12755]/11561                                    ------------------------------------------------------------
gi|895856206|ref|WP_048967668.1|helicase[Enterococcusfaecalis]/11551                                                                   ------------------------------------------------------------
gi|639458836|ref|WP_024636366.1|helicase[Enterococcusfaecium]_gi|658176502|gb|KEI51159.1|helicase[EnterococcusfaeciumUC7256]/1/        ------------------------------------------------------------
gi|488266511|ref|WP_002337719.1|hypotheticalprotein[Enterococcusfaecium]_gi|430581409|gb|ELB19854.1|hypotheticalproteinOIQ_0351        ------------------------------------------------------------
gi|446353356|ref|WP_000431211.1|helicase[Staphylococcusaureus]_gi|253726329|gb|EES95058.1|helicaseCterminaldomainprotein[Staph/        ------------------------------------------------------------
gi|672712068|emb|CDM14336.1|DNAorRNAhelicaseofsuperfamilyII[StaphylococcusepidermidisPM221]/11464                                      ------------------------------------------------------------
gi|896298990|ref|WP_049279905.1|helicase[Staphylococcusaureus]/11571                                                                   ------------------------------------------------------------
gi|541903432|gb|ERH36190.1|hypotheticalproteinSEQU_00085[StaphylococcusequorumUMCCNS924]/11578                                         ------------------------------------------------------------
gi|488289747|ref|WP_002360955.1|helicase[Enterococcusfaecalis]_gi|256682216|gb|EEU21911.1|endonucleaseandmethylaseLlaGI[Enteroc        ------------------------------------------------------------
gi|494392045|ref|WP_007207894.1|helicase[Enterococcusitalicus]_gi|315483798|gb|EFU74284.1|helicaseCterminaldomainprotein[Enter/        ------------------------------------------------------------
```

gi|446350692|ref|WP_000428547.1|helicase[Bacilluscereus]_gi|228727580|gb|EEL78721.1|RestrictionmodificationsystemLlaBIII[Bacil/ --
gi|488255302|ref|WP_002326510.1|hypotheticalprotein[Enterococcusfaecium]_gi|430480276|gb|ELA57459.1|hypotheticalproteinOGE_0473 --
gi|843253770|ref|WP_047916698.1|helicase[Lactococcuspiscium]_gi|802124677|emb|CEN29502.1|EndonucleaseandmethylaseLlaGI,TypeISPr --
LlaBIII/11578 --
gi|499320531|ref|WP_011011023.1|helicase[Listeriainnocua]_gi|16415805|emb|CAC42019.1|pli0021[ListeriainnocuaClip11262]/11569 --
gi|760026859|ref|WP_043709973.1|helicase[Weissellacibaria]_gi|758337165|gb|KIU20061.1|UvsWhelicase[Weissellacibaria]/11567 --
LlaGI/11570 --
gi|499988414|ref|WP_011669132.1|helicase[Lactococcuslactis]_gi|116109019|gb|ABJ74139.1|DNAorRNAhelicaseofsuperfamilyII[Lactococ --
gi|685938360|ref|WP_031670319.1|helicase[Listeriamonocytogenes]_gi|590403387|gb|EXL23261.1|hypotheticalproteinX846_1956[Listeri --
gi|530793219|gb|EQC56240.1|helicase[Lactococcuslactissubsp.cremorisTIFN5]_gi|530793247|gb|EQC56263.1|helicase[Lactococcuslactis --
gi|695270006|ref|WP_032492075.1|hypotheticalprotein[Listeriamonocytogenes]_gi|695212872|ref|YP_009081522.1|hypotheticalproteinp --
gi|504101310|ref|WP_014335297.1|helicase[Leuconostocmesenteroides]_gi|859247905|gb|AKO63885.1|helicase(plasmid)[Leuconostocmese --
gi|488905190|ref|WP_002816265.1|helicase[Leuconostocmesenteroides]_gi|227351522|gb|EEJ41777.1|helicaseCterminaldomainprotein[L/ --
gi|518121285|ref|WP_019291493.1|MULTISPECIES:hypotheticalprotein[Lactococcus]/11588 --
gi|754857213|ref|WP_042217902.1|helicase[Lactococcusgarvieae]/11549 --
gi|640048455|ref|WP_024784762.1|helicase[Streptococcusmutans]/11564 --
gi|512695361|ref|WP_016476323.1|hypotheticalprotein[Veillonellasp.HPA0037]_gi|512079853|gb|EPD79018.1|hypotheticalproteinHMPREF --
gi|636963514|ref|WP_024403747.1|helicase[Streptococcussuis]/11574 --
gi|881115312|ref|WP_048802949.1|helicase[Staphylococcuspasteuri]/11573 --
gi|446063996|ref|WP_000141851.1|helicase[Streptococcusmitis]_gi|307618725|gb|EFN97864.1|endonucleaseandmethylaseLlaGI[Streptoco --
gi|488206609|ref|WP_002277817.1|restrictionmodificationsystemLlaBIII[Streptococcusmutans]_gi|449173033|gb|EMB75626.1|restricti/ --
gi|640023290|ref|WP_024781621.1|helicase[Streptococcusmutans]/11566 --
gi|814946581|ref|WP_046393781.1|helicase[Streptococcusuberis]_gi|810600885|gb|KKF57499.1|helicase[StreptococcusuberisB362]/1156 --
gi|765470762|ref|WP_044720336.1|helicase[Streptococcussuis]/11559 --
gi|654507127|ref|WP_027976443.1|helicase[Streptococcusdevriesei]/11558 --
gi|530812902|gb|EQC72481.1|DNAorRNAhelicaseofsuperfamilyII[Streptococcussp.HSISS2]/11570 --
gi|489158846|ref|WP_003068504.1|helicase[Streptococcusintermedius]_gi|355367114|gb|EHG14827.1|hypotheticalproteinHMPREF9682_002 --
RM.Lru27782ORF13200P/11620 --
gi|492676546|ref|WP_005919236.1|DNAphosphorothioationsystemrestrictionenzyme[Pediococcusacidilactici]_gi|482517924|gb|EOA08103. --
gi|498183116|ref|WP_010497272.1|helicase[Lactobacillusacidipiscis]/11563 --
gi|822100485|ref|WP_046921865.1|helicase[Lactobacillusruminis]_gi|821684442|gb|KLA43321.1|restrictionmodificationsystemLlaBIII/ --
gi|935565832|ref|WP_054450311.1|helicase[Lactobacilluskunkeei]/11573 --
gi|752296887|ref|WP_041152901.1|helicase[Lactobacilluskunkeei]_gi|751663520|gb|KIM18358.1|helicase[Lactobacilluskunkeei]/11577 --
gi|495745291|ref|WP_008469870.1|helicase[Lactobacillushominis]_gi|394484442|emb|CCI81207.1|RestrictionmodificationsystemLlaBII/ --
gi|918672414|ref|WP_052543134.1|helicase[Lactobacillussp.]/11564 --
gi|799141631|ref|WP_045927261.1|helicase[Lactobacilluskullabergensis]_gi|797155335|gb|KJY59264.1|putativerestrictionendonucleas --
gi|504379990|ref|WP_014567092.1|helicase[Lactobacillusjohnsonii]_gi|329666726|gb|AEB92674.1|putativerestrictionendonuclease[Lac --
gi|489880179|ref|WP_003783646.1|DNAdamageinducibleprotein[Actinomycesnaeslundii]_gi|399901561|gb|EJN84440.1|typeIIIrestriction/ --
gi|496524497|ref|WP_009232780.1|DNAdamageinducibleprotein[Actinomycessp.oraltaxon849]_gi|365265413|gb|EHM95177.1|hypotheticalp/ --
gi|915397782|ref|WP_050783167.1|damageinducibleprotein[Actinomycesoris]/11694 --
gi|502925248|ref|WP_013160224.1|helicase[Propionibacteriumfreudenreichii]_gi|296921272|emb|CBL55824.1|helicase[Propionibacteriu --
gi|732164352|emb|CEG87481.1|helicase[Propionibacteriumfreudenreichii]/11618 --
gi|739047402|ref|WP_036925058.1|damageinducibleprotein[Propionibacteriumthoenii]/11596 --
gi|739112839|ref|WP_036982533.1|damageinducibleprotein[Propionibacteriumjensenii]/11594 --
gi|918023558|ref|WP_052314121.1|damageinducibleprotein[Nocardiathailandica]/11612 --
gi|498380271|ref|WP_010694427.1|DNAdamageinducibleprotein[Saccharopolysporaspinosa]/11605 --
gi|749991459|ref|WP_040339809.1|damageinducibleprotein[CandidatusBlastococcusmassiliensis]/11597 --
gi|754177801|ref|WP_041796231.1|damageinducibleprotein[Modestobactermarinus]/11592 --
gi|504188248|ref|WP_014375350.1|DNAdamageinducibleprotein[Blastococcussaxobsidens]_gi|378782790|emb|CCG02456.1|putativeHelicas/ --
gi|946476713|gb|KRF26798.1|damageinducibleprotein[Phycicoccussp.Soil803]/11609 --
gi|700176672|gb|KGN37509.1|DNAdamageinducibleprotein[KnoelliasubterraneaKCTC19937]/11633 --
gi|700168841|gb|KGN29914.1|hypotheticalproteinN802_10215[KnoelliasinensisKCTC19936]/11631 --
gi|946426241|gb|KRE76610.1|damageinducibleprotein[Arthrobactersp.Soil763]/11606 --
gi|517318525|ref|WP_018500927.1|hypotheticalprotein[Frankiasp.BCU110501]/11592 --
gi|499072429|ref|WP_010851327.1|Helicase[Tetrasphaeraelongata]_gi|478756579|emb|CCH71501.1|putativeHelicase[Tetrasphaeraelongat --
gi|750080666|ref|WP_040385350.1|damageinducibleprotein[Demetriaterragena]/11625 --
gi|752558292|ref|WP_041229523.1|damageinducibleprotein[Dermacoccusnishinomiyaensis]_gi|664689315|gb|AIF41904.1|damageinducibl/1 --
gi|829089213|ref|WP_047310701.1|damageinducibleprotein[Dermacoccussp.PE3]_gi|828384812|gb|KLO63770.1|damageinducibleprotein[D/1 --
gi|748756635|ref|WP_040014750.1|damageinducibleprotein[Dermacoccussp.Ellin185]/11596 --
gi|916534724|ref|WP_051141848.1|damageinducibleprotein[Actinomycesneuii]/11632 --
gi|921140030|ref|WP_053090276.1|damageinducibleprotein[Rothiamucilaginosa]/11616 --
gi|896406751|ref|WP_049343749.1|damageinducibleprotein[Rothiamucilaginosa]/11647 --
gi|723608234|gb|AIY01610.1|putativehelicase[Arthrobactersp.PAMC25486]/11613 --
gi|946394527|gb|KRE45160.1|damageinducibleprotein[Arthrobactersp.Soil736]/11616 --
gi|515765304|ref|WP_017197904.1|hypotheticalprotein[Arthrobactersp.M2012083]/11648 --
gi|945315587|gb|KQT73127.1|damageinducibleprotein[Microbacteriumsp.Leaf436]/11612 --
gi|759908497|ref|WP_043594599.1|damageinducibleprotein[Frigoribacteriumsp.MEB024]_gi|758271359|gb|KIU03346.1|damageinduciblep/1 --

```
gi|945072850|gb|KQR34237.1|hypotheticalproteinASF75_00055[Curtobacteriumsp.Leaf154]/11597 ------------------------------------------------------------
gi|551263152|ref|WP_022883363.1|DNAdamageinducibleprotein[Glaciibactersuperstes]/11621 ------------------------------------------------------------
gi|945452341|gb|KQV08491.1|hypotheticalproteinASC63_10170[Leifsoniasp.Root112D2]/11623 ------------------------------------------------------------
gi|742759483|gb|KIC69912.1|damageinducibleprotein[Kocuriarhizophila]/11610 ------------------------------------------------------------
gi|737919995|ref|WP_035885304.1|damageinducibleprotein[Kocuriarhizophila]/11611 ------------------------------------------------------------
gi|737997701|ref|WP_035959325.1|damageinducibleprotein[Kocuriamarina]_gi|725823884|gb|KHE75603.1|damageinducibleprotein[Kocur/1 ------------------------------------------------------------
gi|664521070|ref|WP_031037650.1|damageinducibleprotein[Streptomycessp.NRRLWC3774]/11624 ------------------------------------------------------------
gi|500101034|ref|WP_011777041.1|DNAdamageinducibleprotein[Arthrobacteraurescens]_gi|119951547|gb|ABM10457.1|putativeHelicase[A/ ------------------------------------------------------------
gi|916871953|ref|WP_051479009.1|damageinducibleprotein[Arthrobactersp.H5]/11588 ------------------------------------------------------------
gi|612084649|gb|EZP43369.1|TypeIIIrestrictionenzyme,ressubunit[Micrococcusluteus]/11625 ------------------------------------------------------------
gi|759739708|ref|WP_043450422.1|damageinducibleprotein[Arthrobacterphenanthrenivorans]_gi|742758264|gb|KIC68716.1|damageinduc/1 ------------------------------------------------------------
gi|636845448|ref|WP_024366823.1|damageinducibleprotein[Arthrobactersp.TB26]/11605 ------------------------------------------------------------
gi|500009025|ref|WP_011689743.1|DNAdamageinducibleprotein[Arthrobactersp.FB24]_gi|116613051|gb|ABK05760.1|typeIIIrestrictionen/ ------------------------------------------------------------
gi|821249847|gb|KKZ12083.1|hypotheticalproteinTQ37_06090[CandidatusSynechococcusspongiarum15L]/11436 ------------------------------------------------------------
gi|739446902|ref|WP_037306885.1|damageinducibleprotein[Ruegeriahalocynthiae]/11592 ------------------------------------------------------------
gi|640620081|ref|WP_025048131.1|damageinducibleprotein[Sulfitobactermediterraneus]_gi|751956650|gb|KIN77213.1|TypeIIIrestricti/ ------------------------------------------------------------
gi|740247634|ref|WP_038088583.1|damageinducibleprotein[Thiobacillusprosperus]_gi|684049748|gb|KFZ90853.1|putativehelicase[Thio/ ------------------------------------------------------------
gi|518420321|ref|WP_019590528.1|hypotheticalprotein[Thioalkalivibriosp.ALE20]/11603 ------------------------------------------------------------
gi|500156826|ref|WP_011831496.1|DNAdamageinducibleprotein[Methylibiumpetroleiphilum]_gi|124261892|gb|ABM96881.1|adeninespecifi/ ------------------------------------------------------------
gi|769978537|ref|WP_045094522.1|damageinducibleprotein[Legionellafallonii]_gi|698841736|emb|CEG55682.1|Putativeproteinwithboxh/ ------------------------------------------------------------
gi|553752541|ref|WP_023085344.1|hypotheticalprotein[Pseudomonasaeruginosa]_gi|552201952|gb|ERU62653.1|hypotheticalproteinQ088_0 ------------------------------------------------------------
gi|880980418|ref|WP_048704461.1|damageinducibleprotein[Thauerahumireducens]_gi|875132785|gb|KMU70944.1|damageinducibleprotein/1 ------------------------------------------------------------
gi|740135732|ref|WP_037982815.1|damageinducibleprotein[Thauerasp.27]/11603 ------------------------------------------------------------
gi|738017371|ref|WP_035978332.1|damageinducibleprotein[Burkholderiaglumae]/11380 ------------------------------------------------------------
gi|498506003|ref|WP_010806671.1|typeIIIrestrictionenzyme,ressubunit[Pandoraeasp.SD62]_gi|490314561|gb|EON12879.1|typeIIIrestri/ ------------------------------------------------------------
gi|500117748|ref|WP_011793753.1|DNAdamageinducibleprotein[Acidovoraxcitrulli]_gi|120587742|gb|ABM31182.1|typeIIIrestrictionenz/ ------------------------------------------------------------
gi|651617328|ref|WP_026611444.1|damageinducibleprotein[Methylocaldumszegediense]/11601 ------------------------------------------------------------
gi|737210208|ref|WP_035194994.1|damageinducibleprotein[Acidithiobacillusferrivorans]_gi|669953220|emb|CDQ11755.1|Adeninespecif/ ------------------------------------------------------------
gi|935697386|ref|WP_054506180.1|damageinducibleprotein[Achromobactersp.]_gi|928716153|emb|CUK12836.1|Hefnuclease[Achromobacter/ ------------------------------------------------------------
gi|429502517|gb|ELA00827.1|adeninespecificDNAmethyltransferase[Cupriavidussp.HMR1]/11599 ------------------------------------------------------------
gi|506356642|ref|WP_015876361.1|DNAdamageinducibleprotein[Burkholderiaglumae]_gi|237877158|gb|ACR29491.1|AdeninespecificDNAmet/ ------------------------------------------------------------
gi|696406787|ref|WP_032978003.1|damageinducibleprotein[Bordetellahinzii]_gi|625335076|gb|KCB49343.1|typeIIIrestrictionenzyme,r/ ------------------------------------------------------------
gi|817529125|gb|KKO72587.1|damageinducibleprotein[Kerstersiagyiorum]/11592 ------------------------------------------------------------
gi|737048910|ref|WP_035044118.1|damageinducibleprotein[Aquabacteriumsp.NJ1]_gi|699052896|gb|KGM38706.1|damageinducibleprotein/1 ------------------------------------------------------------
gi|822492519|ref|WP_046933319.1|MULTISPECIES:damageinducibleprotein[Xanthomonas]_gi|821703525|gb|KLA61196.1|damageinduciblepr/1 ------------------------------------------------------------
gi|751252861|ref|WP_040963740.1|damageinducibleprotein[Xanthomonasfuscans]_gi|749808363|gb|KIJ03887.1|damageinducibleprotein[/1 ------------------------------------------------------------
gi|496774980|ref|WP_009368792.1|DNAdamageinducibleprotein[Bilophilasp.4_1_30]_gi|345039902|gb|EGW44205.1|hypotheticalproteinHM/ ------------------------------------------------------------
gi|499241912|ref|WP_010939452.1|DNAdamageinducibleprotein[Desulfovibriovulgaris]_gi|46580580|ref|YP_011388.1|adeninespecificDN/ ------------------------------------------------------------
gi|504274913|ref|WP_014462015.1|DNAdamageinducibleprotein[Bacillusmegaterium]_gi|345446919|gb|AEN91936.1|AdeninespecificDNAmet/ ------------------------------------------------------------
gi|504895966|ref|WP_015083068.1|typeIIrestrictionenzymeandmethyltransferasesubtypeG[Anabaenasp.90]_gi|413972424|gb|AFW96512.1|t ------------------------------------------------------------
gi|518327765|ref|WP_019497972.1|hypotheticalprotein[Calothrixsp.PCC7103]/11600 ------------------------------------------------------------
gi|652765503|ref|WP_027079917.1|damageinducibleprotein[Luteimonasmephitis]/11593 ------------------------------------------------------------
gi|896539951|ref|WP_049442123.1|damageinducibleprotein[Stenotrophomonasmaltophilia]/11591 ------------------------------------------------------------
gi|500217900|ref|WP_011888043.1|DNAdamageinducibleprotein[Geobacillusthermodenitrificans]_gi|134267954|gb|ABO68149.1|Conserved/ ------------------------------------------------------------
gi|696570298|ref|WP_033102014.1|damageinducibleprotein[Thermoactinomycesdaqus]/11594 ------------------------------------------------------------
gi|251772067|gb|EES52637.1|putativehelicase[Leptospirillumferrodiazotrophum]/11618 ------------------------------------------------------------
gi|738124101|ref|WP_036082108.1|damageinducibleprotein[Leptospirillumferriphilum]_gi|685243306|gb|KGA94177.1|putativehelicase[/ ------------------------------------------------------------
gi|558699223|ref|WP_023524766.1|helicase[Leptospirillumsp.GroupII'CF1']_gi|206603764|gb|EDZ40244.1|Putativehelicase[Leptospiri/ ------------------------------------------------------------
gi|406774462|gb|AFS53887.1|putativehelicase[LeptospirillumferriphilumML04]/11610 ------------------------------------------------------------
gi|740719463|ref|WP_038504749.1|damageinducibleprotein[Leptospirillumferriphilum]_gi|640838125|gb|AIA30173.1|damageinduciblep/1 ------------------------------------------------------------
gi|517810766|ref|WP_018980974.1|hypotheticalprotein[Saccharibacterfloricola]/11733 ------------------------------------------------------------
gi|502471675|ref|WP_012798533.1|helicase[Slackiaheliotrinireducens]_gi|256791761|gb|ACV22431.1|predictedhelicase[Slackiaheliotr GAKSHNPNEGAEVDEKTVSDIRTLVRRTVSFFEENGPVVDAGITFEGGYGDVLDSGDADL
gi|657871713|ref|WP_029576297.1|hypotheticalprotein[Bifidobacteriumthermacidophilum]_gi|673007521|gb|KFJ02535.1|helicase[Bifido ------------------------------------------------------------
gi|673004757|gb|KFI99791.1|DNAhelicaserestrictionenzymetypeIIIRessubunit[Bifidobacteriumthermacidophilumsubsp.porcinum]/11682 ------------------------------------------------------------
gi|917316092|ref|WP_051922804.1|hypotheticalprotein[Bifidobacteriumstellenboschense]_gi|673003774|gb|KFI98812.1|helicase[Bifido ------------------------------------------------------------
gi|705415694|ref|WP_033502332.1|hypotheticalprotein[Bifidobacteriumpseudolongum]_gi|672982411|gb|KFI77685.1|DNAdamageinducible/ ------------------------------------------------------------
gi|916591418|ref|WP_051198509.1|hypotheticalprotein[Bifidobacteriumsp.AGR2158]/11704 ------------------------------------------------------------
gi|772242982|gb|AJW78255.1|hypotheticalproteinVO01_03165[Clavibactermichiganensissubsp.insidiosus]/11642 ------------------------------------------------------------
gi|916649818|ref|WP_051256909.1|damageinducibleprotein[Actinomycesgraevenitzii]/11677 ------------------------------------------------------------
gi|496504578|ref|WP_009212861.1|helicase[Actinomycessp.oraltaxon180]_gi|315312695|gb|EFU60776.1|helicase[Actinomycessp.oraltaxo ------------------------------------------------------------
gi|494862206|ref|WP_007588306.1|helicase[Actinomycessp.ICM39]_gi|398362799|gb|EJN46470.1|typeIIrestrictionenzyme,ressubunit[Ac ------------------------------------------------------------
gi|881057111|ref|WP_048774974.1|damageinducibleprotein[Sanguibacterkeddieii]/11697 ------------------------------------------------------------
gi|881020459|ref|WP_048740808.1|damageinducibleprotein[Kytococcussedentarius]/11704 ------------------------------------------------------------
gi|881052313|ref|WP_048770555.1|damageinducibleprotein[Cellulomonasflavigena]/11705 ------------------------------------------------------------
gi|490108682|ref|WP_004009424.1|helicase[Mobiluncuscurtisii]_gi|315490465|gb|EFU80089.1|helicaseCterminaldomainprotein[Mobilun/ ------------------------------------------------------------
gi|490111935|ref|WP_004012603.1|helicase[Mobiluncusmulieris]_gi|227843812|gb|EEJ53982.1|helicaseCterminaldomainprotein[Mobilun/ ------------------------------------------------------------
gi|490114228|ref|WP_004014848.1|helicase[Mobiluncusmulieris]_gi|269934432|gb|EEZ90993.1|helicaseCterminaldomainprotein[Mobilun/ ------------------------------------------------------------
```

```
gi|493333551|ref|WP_006290636.1|helicase[Parascardoviadenticolens]_gi|315119487|gb|EFT82620.1|helicaseCterminaldomainprotein[P/      ------------------------------------------------------------
gi|495817067|ref|WP_008541646.1|helicase,partial[Sutterellaparvirubra]_gi|378598583|gb|EHY31712.1|helicaseprotein,partial[Sutte      ------------------------------------------------------------
gi|491572696|ref|WP_005430278.1|helicase[Sutterellawadsworthensis]_gi|319805116|gb|EFW01943.1|DNAhelicaserestrictionenzymeTypeI      ------------------------------------------------------------
gi|748686321|ref|WP_039944445.1|hypotheticalprotein[Thermicanusaegyptius]/11573      ------------------------------------------------------------
gi|916875698|ref|WP_051482754.1|hypotheticalprotein[Thioalkalivibriosp.HK1]/11366      ------------------------------------------------------------
gi|821248604|gb|KKZ10960.1|hypotheticalproteinTE42_09075[CandidatusSynechococcusspongiarumSP3]/11598      ------------------------------------------------------------
gi|737409843|ref|WP_035390956.1|hypotheticalprotein[Ferrimicrobiumacidiphilum]_gi|765544266|gb|KJE76241.1|typeIrestrictionenzym      ------------------------------------------------------------
gi|654906925|ref|WP_028358281.1|damageinducibleprotein[Brackiellaoedipodis]/11679      ------------------------------------------------------------
gi|502809901|ref|WP_013044877.1|hypotheticalprotein[CandidatusPuniceispirillummarinum]_gi|292663146|gb|ADE38247.1|hypotheticalp      ------------------------------------------------------------
gi|251771370|gb|EES51950.1|typeIIIrestrictionenzyme,ressubunit[Leptospirillumferrodiazotrophum]/11628      ------------------------------------------------------------
gi|498328480|ref|WP_010642636.1|DNAdamageinducibleprotein[Acidithiobacillusthiooxidans]/11627      ------------------------------------------------------------
gi|647633864|ref|WP_025906698.1|damageinducibleprotein[Prochlorococcussp.scB245a_519B7]/11622      ------------------------------------------------------------
gi|647619599|ref|WP_025893729.1|damageinducibleprotein[Prochlorococcussp.scB241_527N11]/11621      ------------------------------------------------------------
gi|755034887|ref|WP_042387498.1|hypotheticalprotein[Clostridiumsp.CL6]/11606      ------------------------------------------------------------
gi|498139798|ref|WP_010453954.1|helicase[SuccinivibrionaceaebacteriumWG1]/11662      ------------------------------------------------------------
gi|527501386|gb|EPT34571.1|typeIIIrestrictionenzyme,ressubunit[Bacteroidetesbacteriumoraltaxon272str.F0290]/11789      ------------------------------------------------------------
gi|496047275|ref|WP_008771782.1|helicase[Bacteroidessp.2_1_33B]_gi|262296443|gb|EEY84373.1|helicaseCterminaldomainprotein[Bact/      ------------------------------------------------------------
PDI8503III/11664      ------------------------------------------------------------
gi|738719431|ref|WP_036613715.1|helicase[Parabacteroidesdistasonis]_gi|649542518|gb|KDS49387.1|restrictionendonucleasefamilypro      ------------------------------------------------------------
gi|659417033|gb|KEJ86133.1|hypotheticalproteinHMPREF1002_00341[Porphyromonassp.31_2]/11656      ------------------------------------------------------------
gi|567456807|gb|ETK11358.1|hypotheticalproteinT235_15845[Tannerellasp.oraltaxonBU063isolateCell8/11]/11433      ------------------------------------------------------------
gi|545440279|ref|WP_021677877.1|helicase[Porphyromonasgingivalis]_gi|543985765|gb|ERJ89108.1|helicaseprotein[Porphyromonasgingi      ------------------------------------------------------------
gi|497438056|ref|WP_009752254.1|helicase[Prevotellasp.oraltaxon473]_gi|429144866|gb|EKX87968.1|helicaseprotein[Prevotellasp.ora      ------------------------------------------------------------
gi|490423474|ref|WP_004295683.1|MULTISPECIES:helicase[Bacteroides]_gi|156110098|gb|EDO11843.1|helicaseCterminaldomainprotein[B/      ------------------------------------------------------------
gi|941777229|ref|WP_055173506.1|helicase[Bacteroidescaccae]_gi|933263395|emb|CUQ00567.1|Predictedhelicase[Bacteroidescaccae]/1/      ------------------------------------------------------------
gi|492413898|ref|WP_005836892.1|helicase[Bacteroidesuniformis]_gi|392678824|gb|EIY72229.1|hypotheticalproteinHMPREF1072_03583[B      ------------------------------------------------------------
gi|695455095|ref|WP_032566191.1|helicase[Bacteroidesfragilis]_gi|596095426|gb|EXZ59996.1|restrictionendonucleasefamilyprotein[B      ------------------------------------------------------------
gi|933227941|emb|CUQ02348.1|Predictedhelicase[Bacteroidesuniformis]/11658      ------------------------------------------------------------
gi|494823013|ref|WP_007558421.1|helicase[Bacteroidesplebeius]_gi|198272167|gb|EDY96436.1|helicaseCterminaldomainprotein[Bacter/      ------------------------------------------------------------
gi|495427212|ref|WP_008151909.1|helicase[Parabacteroidesjohnsonii]_gi|218222381|gb|EEC95031.1|helicaseCterminaldomainprotein[P/      ------------------------------------------------------------
gi|933196855|emb|CUO94545.1|typeIrestrictionenzymeEcoKIsubunitR[Bacteroidesuniformis]_gi|933220098|emb|CUN86874.1|typeIrestrict      ------------------------------------------------------------
gi|823687748|ref|WP_047101121.1|helicase[Brachyspirahyodysenteriae]_gi|823334537|gb|KLI17932.1|helicase[Brachyspirahyodysenteri      ------------------------------------------------------------
gi|496436691|ref|WP_009145538.1|helicase[Phascolarctobacteriumsuccinatutens]_gi|322414007|gb|EFY04839.1|helicaseCterminaldomai/      ------------------------------------------------------------
gi|503372880|ref|WP_013607541.1|helicase[Sphaerochaetaglobosa]_gi|324026933|gb|ADY13692.1|typeIIIrestrictionproteinressubunit[S      ------------------------------------------------------------
gi|505821748|ref|WP_015707145.1|helicase[Treponemaprimitia]_gi|299483503|gb|ADJ19584.1|helicasedomaincontainingprotein[Trepone/      ------------------------------------------------------------
gi|505822741|ref|WP_015708134.1|helicase[Treponemaprimitia]_gi|333739639|gb|AEF85129.1|endonucleaseandmethylaseLlaGI[Treponemap      ------------------------------------------------------------
gi|488791994|ref|WP_002704400.1|helicase[Treponemasaccharophilum]_gi|380767691|gb|EIC01689.1|typeIIIrestrictionproteinressubuni      ------------------------------------------------------------
gi|524671454|emb|CDD81750.1|putativeseptumsitedeterminingproteinMinC[Bacteroidessp.CAG:462]/11642      ------------------------------------------------------------
gi|524573865|emb|CDC95766.1|putativeseptumsitedeterminingproteinMinC[Alistipessp.CAG:268]/11639      ------------------------------------------------------------
gi|658617970|ref|WP_029759328.1|helicase[Fusobacteriumnucleatum]/11606      ------------------------------------------------------------
gi|494412037|ref|WP_007216782.1|helicase[Bacteroidescellulosilyticus]_gi|392638921|gb|EIY32752.1|hypotheticalproteinHMPREF1062_      ------------------------------------------------------------
gi|383107102|gb|AFG37435.1|putativehelicase[SpirochaetaafricanaDSM8902]/11616      ------------------------------------------------------------
SAF8902III/11616      ------------------------------------------------------------
gi|652538894|ref|WP_026932796.1|helicase[Gramellaechinicola]/11633      ------------------------------------------------------------
gi|639238223|ref|WP_024568839.1|helicase[Elizabethkingiaanophelis]/11628      ------------------------------------------------------------
gi|652520381|ref|WP_026914582.1|helicase[Gramellaportivictoriae]/11623      ------------------------------------------------------------
gi|494922183|ref|WP_007648219.1|Helicase[Formosasp.AK20]_gi|469825489|gb|EMQ95550.1|Helicasedomainprotein[Formosasp.AK20]/11626      ------------------------------------------------------------
gi|522083382|ref|WP_020594591.1|hypotheticalprotein[Spirosomapanaciterrae]/11637      ------------------------------------------------------------
gi|490455352|ref|WP_004326157.1|helicase[Bacteroidesovatus]_gi|392669277|gb|EIY62768.1|hypotheticalproteinHMPREF1070_03635[Bact      ------------------------------------------------------------
gi|499516035|ref|WP_011202675.1|helicase[Bacteroidesfragilis]_gi|53713270|ref|YP_099262.1|helicasedomainprotein[Bacteroidesfrag      ------------------------------------------------------------
gi|651338332|ref|WP_026461320.1|helicase[Adhaeribacteraquaticus]/11620      ------------------------------------------------------------
gi|806964642|gb|AKC17139.1|helicase[Flavobacteriumpsychrophilum]/11639      ------------------------------------------------------------
gi|752684374|ref|WP_041332322.1|helicase[Flavobacteriumpsychrophilum]_gi|666882889|gb|AIG30715.1|helicase[Flavobacteriumpsychro      ------------------------------------------------------------
gi|933196595|emb|CUP04894.1|DNAorRNAhelicasesofsuperfamilyII[Bacteroidesuniformis]/11631      ------------------------------------------------------------
gi|670509109|ref|WP_031456918.1|helicase[Flavobacteriumchungangense]/11625      ------------------------------------------------------------


gi|490190857|ref|WP_004089451.1|hypotheticalprotein[Helicobacterbilis]_gi|476630638|gb|EMZ37196.1|hypotheticalproteinC826_02193      ------------------------------------------------------------
gi|488896133|ref|WP_002807242.1|MULTISPECIES:restrictionendonucleaseEco57I[Campylobacter]_gi|380541096|gb|EIA65377.1|hypothetic      ------------------------------------------------------------
gi|746602240|ref|WP_039628365.1|restrictionendonucleaseEco57I[Campylobacterlari]_gi|744819395|gb|AJD03391.1|putativehelicase[Ca      ------------------------------------------------------------
gi|727317135|ref|WP_033772558.1|restrictionendonucleaseEco57I[Helicobacterpylori]/11622      ------------------------------------------------------------
gi|727103490|ref|WP_033618954.1|restrictionendonucleaseEco57I[Helicobacterpylori]/11616      ------------------------------------------------------------
gi|727340205|ref|WP_033794408.1|restrictionendonucleaseEco57I[Helicobacterpylori]/11609      ------------------------------------------------------------
gi|727292407|ref|WP_033749975.1|restrictionendonucleaseEco57I[Helicobacterpylori]/11611      ------------------------------------------------------------
gi|727289795|ref|WP_033747591.1|restrictionendonucleaseEco57I[Helicobacterpylori]/11612      ------------------------------------------------------------
gi|727319676|ref|WP_033775047.1|restrictionendonucleaseEco57I[Helicobacterpylori]/11612      ------------------------------------------------------------
gi|447212910|ref|WP_001290166.1|restrictionendonucleaseEco57I[Helicobacterpylori]_gi|308063540|gb|ADO05427.1|hypotheticalprotei      ------------------------------------------------------------
```

```
gi|447212901|ref|WP_001290157.1|restrictionendonucleaseEco57I[Helicobacterpylori]_gi|384557329|gb|AFH97797.1|hypotheticalprotei      ------------------------------------------------------------
gi|515849671|ref|WP_017280424.1|hypotheticalprotein[Helicobacterpylori]/11615      ------------------------------------------------------------
gi|447212900|ref|WP_001290156.1|restrictionendonucleaseEco57I[Helicobacterpylori]_gi|317178970|dbj|BAJ56758.1|TypeIIGrestrictio      ------------------------------------------------------------
gi|447079018|ref|WP_001156274.1|hypotheticalprotein[Helicobacterpylori]/11614      ------------------------------------------------------------
gi|447212800|ref|WP_001290056.1|hypotheticalprotein[Helicobacterpylori]_gi|532153248|gb|EQL67316.1|restrictionendonucleaseEco57      ------------------------------------------------------------
gi|505457237|ref|WP_015644339.1|hypotheticalprotein[Helicobacterpylori]_gi|499061160|gb|AGL70074.1|restrictionendonucleaseEco57      ------------------------------------------------------------
HPYUM037X/11591      ------------------------------------------------------------
gi|920026297|ref|WP_052937176.1|restrictionendonuclease[Helicobacterpylori]_gi|912528678|gb|KNX48238.1|restrictionendonucleaseE      ------------------------------------------------------------
gi|915539050|ref|WP_050841327.1|restrictionendonuclease[Helicobacterpylori]_gi|908890887|gb|KNE06183.1|restrictionendonucleaseE      ------------------------------------------------------------
gi|727101397|ref|WP_033616957.1|restrictionendonucleaseEco57I[Helicobacterpylori]/11592      ------------------------------------------------------------
gi|562814875|emb|CDK13002.1|TypeISPRMsystem[Helicobacterpylori26695]/11588      ------------------------------------------------------------
HPYAXVIII/11588      ------------------------------------------------------------
gi|447079029|ref|WP_001156285.1|restrictionendonucleaseEco57I[Helicobacterpylori]_gi|393085956|gb|EJB86635.1|hypotheticalprotei      ------------------------------------------------------------
gi|489105953|ref|WP_003015814.1|restrictionendonuclease[Helicobacterpylori]_gi|477503388|gb|ENH59950.1|restrictionendonucleasef      ------------------------------------------------------------
gi|447079006|ref|WP_001156262.1|restrictionendonucleaseEco57I[Helicobacterpylori]_gi|393067470|gb|EJB68280.1|hypotheticalprotei      ------------------------------------------------------------
gi|727314589|ref|WP_033770298.1|restrictionendonucleaseEco57I[Helicobacterpylori]/11586      ------------------------------------------------------------
gi|447079019|ref|WP_001156275.1|restrictionendonucleaseEco57I[Helicobacterpylori]_gi|308065028|gb|ADO06920.1|hypotheticalprotei      ------------------------------------------------------------
gi|447079028|ref|WP_001156284.1|restrictionendonucleaseEco57I[Helicobacterpylori]_gi|393076030|gb|EJB76784.1|hypotheticalprotei      ------------------------------------------------------------
gi|393034009|gb|EJB35070.1|hypotheticalproteinHPNQ4053_0631[HelicobacterpyloriNQ4053]/11514      ------------------------------------------------------------
gi|447079004|ref|WP_001156260.1|restrictionendonucleaseEco57I[Helicobacterpylori]_gi|384552418|gb|AFI07366.1|hypotheticalprotei      ------------------------------------------------------------
gi|746011306|ref|WP_039088215.1|restrictionendonucleaseEco57I[Helicobacterpylori]_gi|733488720|gb|KHL85979.1|restrictionendonuc      ------------------------------------------------------------
gi|531081162|gb|EQD92166.1|restrictionendonucleaseEco57I[HelicobacterpyloriPZ5080]/11509      ------------------------------------------------------------
gi|544760796|ref|WP_021187395.1|restrictionendonucleaseEco57I[Helicobacterpylori]_gi|531086508|gb|EQD96745.1|restrictionendonuc      ------------------------------------------------------------
gi|447079012|ref|WP_001156268.1|restrictionendonucleaseEco57I[Helicobacterpylori]_gi|393028199|gb|EJB29286.1|hypotheticalprotei      ------------------------------------------------------------
gi|746008458|ref|WP_039085555.1|restrictionendonucleaseEco57I[Helicobacterpylori]_gi|733480358|gb|KHL78026.1|restrictionendonuc      ------------------------------------------------------------
gi|447079011|ref|WP_001156267.1|restrictionendonucleaseEco57I[Helicobacterpylori]_gi|393038534|gb|EJB39568.1|hypotheticalprotei      ------------------------------------------------------------
gi|393037163|gb|EJB38199.1|hypotheticalproteinHPNQ4161_0422[HelicobacterpyloriNQ4161]/11556      ------------------------------------------------------------
gi|447079007|ref|WP_001156263.1|restrictionendonucleaseEco57I[Helicobacterpylori]_gi|393051817|gb|EJB52768.1|hypotheticalprotei      ------------------------------------------------------------
gi|487867094|ref|WP_001940560.1|helicase[Helicobacterpylori]_gi|459505657|gb|EMG98418.1|helicaseprotein[HelicobacterpyloriGAM21      ------------------------------------------------------------
gi|487872094|ref|WP_001945560.1|helicase[Helicobacterpylori]_gi|459512104|gb|EMH04523.1|helicaseprotein[HelicobacterpyloriGAM24      ------------------------------------------------------------
gi|487880228|ref|WP_001953694.1|helicase[Helicobacterpylori]_gi|459529131|gb|EMH20734.1|helicaseprotein[HelicobacterpyloriGAM26      ------------------------------------------------------------
gi|487882593|ref|WP_001956059.1|helicase[Helicobacterpylori]_gi|459533462|gb|EMH24859.1|helicaseprotein[HelicobacterpyloriGAM26      ------------------------------------------------------------
gi|487845430|ref|WP_001918896.1|helicase[Helicobacterpylori]_gi|410713871|gb|EKQ71363.1|helicaseprotein[HelicobacterpyloriGAM10      ------------------------------------------------------------
gi|447079005|ref|WP_001156261.1|restrictionendonucleaseEco57I[Helicobacterpylori]_gi|393069830|gb|EJB70625.1|hypotheticalprotei      ------------------------------------------------------------
gi|459506808|gb|EMG99528.1|helicaseprotein[HelicobacterpyloriGAM121Aii]/11545      ------------------------------------------------------------
gi|487825283|ref|WP_001898749.1|restrictionendonucleaseEco57I[Helicobacterpylori]_gi|393107526|gb|EJC08068.1|hypotheticalprotei      ------------------------------------------------------------
gi|447079008|ref|WP_001156264.1|restrictionendonucleaseEco57I[Helicobacterpylori]_gi|393058636|gb|EJB59524.1|hypotheticalprotei      ------------------------------------------------------------
gi|447079015|ref|WP_001156271.1|restrictionendonucleaseEco57I[Helicobacterpylori]_gi|307637357|gb|ADN79807.1|hypotheticalprotei      ------------------------------------------------------------
gi|487884861|ref|WP_001958327.1|helicase[Helicobacterpylori]_gi|459535535|gb|EMH26834.1|helicaseprotein[HelicobacterpyloriGAM26      ------------------------------------------------------------
gi|487886365|ref|WP_001959831.1|helicase[Helicobacterpylori]_gi|459538727|gb|EMH29861.1|helicaseprotein[HelicobacterpyloriGAM27      ------------------------------------------------------------
gi|545301637|ref|WP_021581412.1|helicase[Helicobacterpylori]_gi|535931889|gb|ERA61745.1|helicaseprotein[HelicobacterpyloriGAM11      ------------------------------------------------------------
gi|447079010|ref|WP_001156266.1|restrictionendonucleaseEco57I[Helicobacterpylori]_gi|393095477|gb|EJB96081.1|hypotheticalprotei      ------------------------------------------------------------
gi|447079009|ref|WP_001156265.1|restrictionendonucleaseEco57I[Helicobacterpylori]_gi|393056294|gb|EJB57206.1|hypotheticalprotei      ------------------------------------------------------------
gi|471386985|gb|EMR58906.1|restrictionendonucleasefamilyprotein[HelicobacterpyloriHpH1]/11504      ------------------------------------------------------------
gi|487875679|ref|WP_001949145.1|helicase[Helicobacterpylori]_gi|459516928|gb|EMH09091.1|helicaseprotein[HelicobacterpyloriGAM25      ------------------------------------------------------------
gi|496130066|ref|WP_008854573.1|DNAdamageinducibleprotein[Commensalibacterintestini]_gi|353671689|gb|EHD13391.1|helicasedomain/      ------------------------------------------------------------
gi|924898894|ref|WP_053556546.1|damageinducibleprotein[Sphingopyxissp.113P3]_gi|924523996|gb|ALC14732.1|damageinducibleprotei/1      ------------------------------------------------------------
gi|780802950|gb|KJS08562.1|damageinducibleprotein[Hoefleasp.BRH_c9]/11633      ------------------------------------------------------------
gi|944671432|gb|KQN22886.1|damageinducibleprotein[Sphingomonassp.Leaf32]/11415      ------------------------------------------------------------
gi|944612953|gb|KQM64772.1|damageinducibleprotein[Sphingomonassp.Leaf16]/11726      ------------------------------------------------------------
gi|764353851|ref|WP_044331468.1|damageinducibleprotein[Sphingomonashengshuiensis]_gi|761897338|gb|AJP71707.1|damageinduciblep/1      ------------------------------------------------------------
gi|945874472|gb|KQZ27973.1|damageinducibleprotein[Mesorhizobiumsp.Root552]/11651      ------------------------------------------------------------
gi|759408081|ref|WP_043132403.1|damageinducibleprotein[Paracoccussanguinis]_gi|694217086|gb|KGJ14281.1|damageinducibleprotein/1      ------------------------------------------------------------
gi|654342671|ref|WP_027836116.1|damageinducibleprotein[Maritaleamyrionectae]/11632      ------------------------------------------------------------
gi|126717687|gb|EBA14409.1|hypotheticalproteinRSK20926_02032[Roseobactersp.SK20926]/11603      ------------------------------------------------------------
gi|765301116|ref|WP_044628780.1|damageinducibleprotein[Halomonasmeridiana]_gi|764089679|gb|KJD19875.1|damageinducibleprotein[/1      ------------------------------------------------------------
gi|518053006|ref|WP_019223214.1|DNAdamageinducibleprotein[Bartonellarattaustraliani]/11654      ------------------------------------------------------------
gi|493637806|ref|WP_006589578.1|DNAdamageinducibleprotein[Bartonellabirtlesii]_gi|395410345|gb|EJF76900.1|hypotheticalproteinM/      ------------------------------------------------------------
gi|501188603|ref|WP_012231622.1|DNAdamageinducibleprotein[Bartonellatribocorum]_gi|161017843|emb|CAK01401.1|helicase/methyltra/      ------------------------------------------------------------
gi|651388616|ref|WP_026500615.1|damageinducibleprotein[Bartonellagrahamii]/11655      ------------------------------------------------------------
gi|501188669|ref|WP_012231687.1|DNAdamageinducibleprotein[Bartonellatribocorum]_gi|161017922|emb|CAK01480.1|helicase/methyltra/      ------------------------------------------------------------
gi|518048832|ref|WP_019219040.1|DNAdamageinducibleprotein[Bartonellaflorencae]/11655      ------------------------------------------------------------
gi|518053617|ref|WP_019223825.1|DNAdamageinducibleprotein[Bartonellarattaustraliani]/11607      ------------------------------------------------------------
gi|506336449|ref|WP_015856168.1|DNAdamageinducibleprotein[Bartonellagrahamii]_gi|240267492|gb|ACS51080.1|helicase/methyltransf/      ------------------------------------------------------------
gi|494588832|ref|WP_007347427.1|DNAdamageinducibleprotein[Bartonellarattimassiliensis]_gi|395419053|gb|EJF85361.1|hypothetical/      ------------------------------------------------------------
gi|501188650|ref|WP_012231668.1|DNAdamageinducibleprotein[Bartonellatribocorum]_gi|161017902|emb|CAK01460.1|helicase/methyltra/      ------------------------------------------------------------
gi|501188624|ref|WP_012231643.1|DNAdamageinducibleprotein[Bartonellatribocorum]_gi|161017866|emb|CAK01424.1|helicase/methyltra/      ------------------------------------------------------------
```

```
gi|518053856|ref|WP_019224064.1|DNAdamageinducibleprotein[Bartonellarattaustraliani]/11625          ------------------------------------------------------------
gi|501188053|ref|WP_012231081.1|DNAdamageinducibleprotein[Bartonellatribocorum]_gi|161017434|emb|CAK00992.1|helicase/methyltra/      ------------------------------------------------------------
gi|651388630|ref|WP_026500626.1|damageinducibleprotein[Bartonellagrahamii]/11657          ------------------------------------------------------------
gi|518048855|ref|WP_019219063.1|DNAdamageinducibleprotein[Bartonellaflorencae]/11658          ------------------------------------------------------------
gi|490995101|ref|WP_004856829.1|DNAdamageinducibleprotein[Bartonelladoshiae]_gi|395413167|gb|EJF79645.1|hypotheticalproteinMCS/      ------------------------------------------------------------
gi|491002558|ref|WP_004864279.1|DNAdamageinducibleprotein[Bartonellavinsonii]_gi|395421392|gb|EJF87643.1|hypotheticalproteinME/      ------------------------------------------------------------
gi|491003373|ref|WP_004865094.1|DNAdamageinducibleprotein[Bartonellavinsonii]_gi|395432864|gb|EJF98838.1|hypotheticalproteinME/      ------------------------------------------------------------
gi|491002426|ref|WP_004864148.1|DNAdamageinducibleprotein[Bartonellavinsonii]_gi|395421767|gb|EJF88003.1|hypotheticalproteinME/      ------------------------------------------------------------
gi|491004024|ref|WP_004865745.1|DNAdamageinducibleprotein[Bartonellavinsonii]_gi|395432542|gb|EJF98522.1|hypotheticalproteinME/      ------------------------------------------------------------
gi|648396442|ref|WP_026088193.1|damageinducibleprotein[Bartonellarattaustraliani]/11593          ------------------------------------------------------------
gi|748190174|ref|WP_039761290.1|damageinducibleprotein[Bartonellaqueenslandensis]/11658          ------------------------------------------------------------
gi|494817599|ref|WP_007553007.1|DNAdamageinducibleprotein[Bartonellasp.DB56]_gi|395411312|gb|EJF77837.1|hypotheticalproteinMC/1      ------------------------------------------------------------
gi|515763376|ref|WP_017195976.1|DNAdamageinducibleprotein[Bartonellabirtlesii]/11656          ------------------------------------------------------------
gi|494589420|ref|WP_007348014.1|DNAdamageinducibleprotein[Bartonellarattimassiliensis]_gi|395416278|gb|EJF82667.1|hypothetical/      ------------------------------------------------------------
gi|506336469|ref|WP_015856188.1|DNAdamageinducibleprotein[Bartonellagrahamii]_gi|240267512|gb|ACS51100.1|helicase/methyltransf/      ------------------------------------------------------------
gi|749308087|ref|WP_040132227.1|damageinducibleprotein[Bartonellatribocorum]_gi|605051878|emb|CDO49988.1|helicase/methyltransf/      ------------------------------------------------------------
gi|515763800|ref|WP_017196400.1|DNAdamageinducibleprotein[Bartonellabirtlesii]/11658          ------------------------------------------------------------
gi|492186994|ref|WP_005773380.1|DNAdamageinducibleprotein[Bartonellaelizabethae]_gi|395418361|gb|EJF84688.1|hypotheticalprotei/      ------------------------------------------------------------
gi|501188615|ref|WP_012231634.1|DNAdamageinducibleprotein[Bartonellatribocorum]_gi|161017856|emb|CAK01414.1|helicase/methyltra/      ------------------------------------------------------------
gi|501187470|ref|WP_012230499.1|DNAdamageinducibleprotein[Bartonellatribocorum]_gi|161017090|emb|CAK00648.1|helicase/methyltra/      ------------------------------------------------------------
gi|501188636|ref|WP_012231655.1|DNAdamageinducibleprotein[Bartonellatribocorum]_gi|161017879|emb|CAK01437.1|helicase/methyltra/      ------------------------------------------------------------
gi|930151941|emb|CTQ34818.1|UvsWhelicase[Jannaschiarubra]/11641          ------------------------------------------------------------
gi|928977106|ref|WP_054001461.1|damageinducibleprotein[Shimiasp.SK013]_gi|927698852|gb|KPA22698.1|typeIrestrictionenzymeEcoKIs/      ------------------------------------------------------------
gi|738872465|ref|WP_036760884.1|damageinducibleprotein[Leisingeradaeponensis]/11643          ------------------------------------------------------------
gi|518050881|ref|WP_019221089.1|DNAdamageinducibleprotein[Bartonellasenegalensis]/11652          ------------------------------------------------------------
gi|493980596|ref|WP_006923523.1|DNAdamageinducibleprotein[Bartonellawashoensis]_gi|395413322|gb|EJF79796.1|hypotheticalprotein/      ------------------------------------------------------------
gi|493983106|ref|WP_006925984.1|DNAdamageinducibleprotein[Bartonellawashoensis]_gi|395418127|gb|EJF84455.1|hypotheticalprotein/      ------------------------------------------------------------
gi|499494671|ref|WP_011181311.1|DNAdamageinducibleprotein[Bartonellahenselae]_gi|49239008|emb|CAF28308.1|hypotheticalproteinBH/      ------------------------------------------------------------
gi|651388426|ref|WP_026500441.1|damageinducibleprotein[Bartonellagrahamii]/11652          ------------------------------------------------------------
gi|506337280|ref|WP_015856999.1|DNAdamageinducibleprotein[Bartonellagrahamii]_gi|240268402|gb|ACS51990.1|helicase/methyltransf/      ------------------------------------------------------------
gi|494817535|ref|WP_007552943.1|DNAdamageinducibleprotein[Bartonellasp.DB56]_gi|395412002|gb|EJF78518.1|hypotheticalproteinMC/1      ------------------------------------------------------------
gi|493638099|ref|WP_006589864.1|DNAdamageinducibleprotein[Bartonellabirtlesii]_gi|395409809|gb|EJF76394.1|hypotheticalproteinM/      ------------------------------------------------------------
gi|748189589|ref|WP_039760707.1|damageinducibleprotein[Bartonellaqueenslandensis]/11651          ------------------------------------------------------------
gi|501189483|ref|WP_012232501.1|DNAdamageinducibleprotein[Bartonellatribocorum]_gi|161018902|emb|CAK02460.1|helicase/methyltra/      ------------------------------------------------------------
gi|518049181|ref|WP_019219389.1|DNAdamageinducibleprotein[Bartonellaflorencae]/11652          ------------------------------------------------------------
gi|492193255|ref|WP_005774528.1|DNAdamageinducibleprotein[Bartonellaelizabethae]_gi|395416403|gb|EJF82779.1|hypotheticalprotei/      ------------------------------------------------------------
gi|498387325|ref|WP_010701288.1|helicase/methyltransferase[Bartonellabovis]_gi|478418724|gb|ENN92426.1|helicase/methyltransfera      ------------------------------------------------------------
gi|319408697|emb|CBI82352.1|conservedhypotheticalprotein[BartonellaschoenbuchensisR1]/11661          ------------------------------------------------------------
gi|494741726|ref|WP_007477134.1|DNAdamageinducibleprotein[Bartonellamelophagi]_gi|395423925|gb|EJF90113.1|hypotheticalproteinM/      ------------------------------------------------------------
gi|737261405|ref|WP_035245349.1|damageinducibleprotein[Alcanivoraxjadensis]_gi|686989709|gb|KGD62556.1|hypotheticalproteinT9A_/      ------------------------------------------------------------
gi|916687564|ref|WP_051294655.1|damageinducibleprotein[Gemmobacternectariphilus]/11641          ------------------------------------------------------------
gi|740294127|ref|WP_038132495.1|damageinducibleprotein[Thioclavasp.DT234]_gi|660524826|gb|KEO54689.1|hypotheticalproteinDT23_/1      ------------------------------------------------------------
gi|568331062|ref|WP_024099615.1|helicase[Phaeobactergallaeciensis]_gi|568232121|gb|AHD12007.1|putativehelicase[Phaeobactergalla      ------------------------------------------------------------
gi|518810459|ref|WP_019966413.1|DNAdamageinducibleprotein[Pannonibacterphragmitetus]/11635          ------------------------------------------------------------
gi|495305760|ref|WP_008030510.1|DNAdamageinducibleprotein[Rhodobactersp.SW2]_gi|259021698|gb|EEW25000.1|typeIIIrestrictionprot/      ------------------------------------------------------------
gi|506403303|ref|WP_015923022.1|DNAdamageinducibleprotein[Rhodobactersphaeroides]_gi|221162951|gb|ACM03922.1|HypotheticalProte/      ------------------------------------------------------------
gi|738818386|ref|WP_036708790.1|damageinducibleprotein[Paracoccussanguinis]_gi|694226786|gb|KGJ22541.1|damageinducibleprotein/1      ------------------------------------------------------------
gi|746096057|ref|WP_039162453.1|damageinducibleprotein[Gallibacteriumanatis]_gi|703617775|gb|KGQ63868.1|damageinducibleprotei/1      ------------------------------------------------------------
gi|737716785|ref|WP_035685631.1|damageinducibleprotein[Avibacteriumparagallinarum]/11643          ------------------------------------------------------------
gi|540072553|gb|ERF78672.1|hypotheticalproteinN561_04930[Gallibacteriumanatis12656/12]/11651          ------------------------------------------------------------
gi|917509519|ref|WP_052115936.1|damageinducibleprotein[Gallibacteriumanatis]_gi|702414161|gb|KGQ42233.1|damageinducibleprotei/1      ------------------------------------------------------------
gi|746082942|ref|WP_039150268.1|damageinducibleprotein[Gallibacteriumanatis]_gi|703617078|gb|KGQ63194.1|damageinducibleprotei/1      ------------------------------------------------------------
gi|822522885|ref|WP_046952840.1|damageinducibleprotein[Haemophilushaemolyticus]_gi|821501762|gb|KKZ59267.1|damageinduciblepro/1      ------------------------------------------------------------
gi|404381014|gb|EJZ77501.1|putativehelicase[Pasteurellamultocidasubsp.gallicidaP1059]/11681          -----------------------------------------------------------M
gi|298281660|gb|EFI23149.1|helicaseCterminaldomainprotein[Neisseriasp.oraltaxon014str.F0314]/11493          ------------------------------------------------------------
gi|348013055|gb|EGY51980.1|helicasedomainprotein[Neisseriashayeganii871]/11481          ------------------------------------------------------------
gi|496407140|ref|WP_009116004.1|DNAdamageinducibleprotein[Neisseriawadsworthii]_gi|349794756|gb|EGZ48566.1|helicasedomainprote/      ------------------------------------------------------------
gi|517216441|ref|WP_018405259.1|DNAdamageinducibleprotein[Marinobacterlipolyticus]/11656          ------------------------------------------------------------
gi|738230264|ref|WP_036185691.1|damageinducibleprotein[Marinobactersp.ELB17]/11671          ------------------------------------------------------------
gi|447197741|ref|WP_001274997.1|DNAdamageinducibleprotein[Acinetobacterbaumannii]_gi|408512324|gb|EKK13969.1|typeIIIrestrictio/      ------------------------------------------------------------
gi|820775897|ref|WP_046739909.1|damageinducibleprotein[Acinetobactersp.AG1]_gi|819909758|gb|KKW75938.1|damageinducibleprotein/1      ------------------------------------------------------------
gi|584595163|ref|WP_024160846.1|helicase[Acinetobacterpittii]_gi|576702530|emb|CDI28049.1|helicasedomaincontainingprotein[Acin/      ------------------------------------------------------------
gi|514963042|ref|WP_016651366.1|hypotheticalprotein[Acinetobactersp.NIPH2036]_gi|514162665|gb|EPG41391.1|hypotheticalproteinF90      ------------------------------------------------------------
gi|515965564|ref|WP_017396147.1|DNAdamageinducibleprotein[Acinetobacterhaemolyticus]/11667          ------------------------------------------------------------
gi|491346810|ref|WP_005204747.1|MULTISPECIES:hypotheticalprotein[Acinetobacter]_gi|480335823|gb|ENX56033.1|hypotheticalproteinF      ------------------------------------------------------------
gi|693044472|ref|WP_032215268.1|damageinducibleprotein[Escherichiacoli]_gi|607725731|gb|EZE08230.1|damageinducibleprotein[Esc/1      ------------------------------------------------------------
gi|742382177|ref|WP_038861304.1|damageinducibleprotein[Cronobactermuytjensii]_gi|924333747|gb|ALB69766.1|damageinducibleprote/1      ------------------------------------------------------------
```

```
gi|915912804|ref|WP_050927035.1|damageinducibleprotein[Yersiniaenterocolitica]_gi|899805802|emb|CQH32042.1|typeIrestrictionenz/       ------------------------------------------------------------
gi|921111327|gb|KON62781.1|typeIrestrictionenzymeEcoKIsubunitR[Komagataeibactereuropaeus]/11492                                         ------------------------------------------------------------
gi|582024278|gb|AHI27615.1|DNAhelicaserestrictionenzymeTypeIIIRsubunit[KomagataeibacterxylinusE25]/11636                               ------------------------------------------------------------
gi|347662673|dbj|BAK86128.1|DNAhelicaserestrictionenzymeTypeIIIRsubunit[KomagataeibactermedellinensisNBRC3288]/11737                   ------------------------------------------------------------
gi|917445053|ref|WP_052051471.1|damageinducibleprotein[Acetobactertropicalis]/11429                                                     ------------------------------------------------------------
gi|453329445|dbj|GAC88472.1|DNAhelicaserestrictionenzymetypeIIIRsubunit[GluconobacterthailandicusNBRC3255]_gi|530287981|dbj|GAD        ------------------------------------------------------------
gi|889770922|ref|WP_048844278.1|damageinducibleprotein[Gluconobacterfrateurii]/11419                                                    ------------------------------------------------------------
gi|504886208|ref|WP_015073310.1|hypotheticalprotein[Gluconobacteroxydans]_gi|411027954|gb|AFW01209.1|hypotheticalproteinB932_16        ------------------------------------------------------------
gi|480040211|gb|ENV79751.1|hypotheticalproteinF942_01641[AcinetobacterursingiiANC3649]/11432                                            ------------------------------------------------------------
gi|822498222|ref|WP_046936504.1|damageinducibleprotein[Xanthomonasgardneri]_gi|821751224|gb|KLB07223.1|damageinducibleprotein/1        ------------------------------------------------------------
gi|654059559|ref|WP_027705556.1|damageinducibleprotein[Zymobacterpalmae]/11635                                                          ------------------------------------------------------------
gi|643535620|ref|WP_025224315.1|damageinducibleprotein[Methylophilaceaebacterium11]_gi|577704400|gb|EUJ10546.1|putativehelicas/        ------------------------------------------------------------
MBA11I/11635                                                                                                                            ------------------------------------------------------------
gi|655355857|ref|WP_028762287.1|damageinducibleprotein[Shewanellacolwelliana]/11632                                                     ------------------------------------------------------------
gi|753204236|ref|WP_041510579.1|damageinducibleprotein[Shewanellasp.cp20]_gi|752457765|gb|KIO36326.1|damageinducibleprotein[S/1        ------------------------------------------------------------
KPNNIH30III/11619                                                                                                                       ------------------------------------------------------------
gi|749295994|ref|WP_040120352.1|damageinducibleprotein[Klebsiellapneumoniae]_gi|723249300|gb|AIX81926.1|damageinducibleprotei/1        ------------------------------------------------------------
gi|641639739|emb|CDQ54010.1|unnamedproteinproduct[Klebsiellapneumoniae]/11641                                                           ------------------------------------------------------------
gi|490797922|ref|WP_004660060.1|hypotheticalprotein[Acinetobactersp.NIPH236]_gi|479876779|gb|ENU27973.1|hypotheticalproteinF992        ------------------------------------------------------------
gi|916750926|ref|WP_051357982.1|damageinducibleprotein[Acinetobactertjernbergiae]/11453                                                 ------------------------------------------------------------
gi|691098662|ref|WP_032025060.1|damageinducibleprotein[Acinetobacterbaumannii]_gi|593567521|gb|EXR50735.1|restrictionendonucle/        ------------------------------------------------------------
gi|736669405|ref|WP_034676049.1|damageinducibleprotein[Acinetobacterbaumannii]/11643                                                    ------------------------------------------------------------
gi|491368928|ref|WP_005226826.1|hypotheticalprotein[Acinetobactersp.NIPH1847]_gi|480173259|gb|ENX09797.1|hypotheticalproteinF89        ------------------------------------------------------------
gi|796853993|ref|WP_045795953.1|damageinducibleprotein[Acinetobacterindicus]_gi|787929761|gb|KJV44219.1|damageinducibleprotei/1        ------------------------------------------------------------
gi|295797463|gb|ADG32253.1|typeIIIrestrictionproteinressubunit[ThiomonasintermediaK12]/11676                                            ------------------------------------------------------------
gi|499871179|ref|WP_011551913.1|DNAdamageinducibleprotein[Myxococcusxanthus]_gi|108467544|gb|ABF92729.1|putativerestriction/mo/        ------------------------------------------------------------
gi|495313852|ref|WP_008038600.1|DNAdamageinducibleprotein[Bartonellatamiae]_gi|395424181|gb|EJF90368.1|hypotheticalproteinME5_/        ------------------------------------------------------------
gi|501453335|ref|WP_012476784.1|DNAdamageinducibleprotein[Leptospirabiflexa]_gi|167777824|gb|ABZ96123.1|Helicase[Leptospirabif/        ------------------------------------------------------------
gi|738104613|ref|WP_036063193.1|damageinducibleprotein[Leptospiranoguchii]/11652                                                        ------------------------------------------------------------
gi|410001039|gb|EKO51661.1|typeIIIrestrictionenzyme,ressubunit[Leptospirakirschneristr.200802841]/11651                                 ------------------------------------------------------------
gi|490901851|ref|WP_004763774.1|typeIIIrestrictionenzyme,ressubunit[Leptospirakirschneri]_gi|464209249|gb|EMN24014.1|typeIIIres        ------------------------------------------------------------
gi|464398131|gb|EMO65902.1|typeIIIrestrictionenzyme,ressubunit[Leptospirakirschneristr.200803703]/11648                                 ------------------------------------------------------------
gi|945029024|gb|KQQ84900.1|damageinducibleprotein[Xanthomonassp.Leaf131]/11423                                                         ------------------------------------------------------------
gi|896511314|ref|WP_049418183.1|damageinducibleprotein[Stenotrophomonasmaltophilia]/11639                                               ------------------------------------------------------------
gi|896513082|ref|WP_049419616.1|damageinducibleprotein[Stenotrophomonasmaltophilia]/11638                                               ------------------------------------------------------------
gi|896580616|ref|WP_049469425.1|damageinducibleprotein[Stenotrophomonasmaltophilia]/11641                                               ------------------------------------------------------------
gi|648289350|ref|WP_026070499.1|damageinducibleprotein[Stenotrophomonasmaltophilia]/11639                                               ------------------------------------------------------------
gi|656272786|ref|WP_029218442.1|damageinducibleprotein[Xanthomonascassavae]/11639                                                       ------------------------------------------------------------
gi|406976236|gb|EKD98747.1|hypotheticalproteinACD_23C00290G0002[unculturedbacterium]/11679                                              ------------------------------------------------------------
gi|828145737|ref|WP_047238120.1|damageinducibleprotein[Chromobacteriumsubtsugae]/11645                                                  ------------------------------------------------------------
gi|654311174|ref|WP_027824327.1|damageinducibleprotein[Laribacterhongkongensis]/11641                                                   ------------------------------------------------------------
gi|757242173|gb|KIR15516.1|typeIrestrictionenzymeEcoKIsubunitR[Pseudomonasfluorescens]/11778                                            ------------------------------------------------------------
gi|653605161|ref|WP_027616682.1|damageinducibleprotein[Pseudomonassp.URHB0015]/11639                                                    ------------------------------------------------------------
gi|520868025|ref|WP_020308060.1|helicase[Pseudomonassyringae]_gi|519159034|gb|EPM49935.1|helicasedomaincontainingprotein[Pseud/        ------------------------------------------------------------
gi|940156567|gb|KPX71668.1|Helicasedomaincontainingprotein[Pseudomonasamygdalipv.lachrymans]/11677                                      ---------------------------------------------------MKTNPGWPK
gi|505460508|ref|WP_015647610.1|helicase[Pseudomonassp.GLE121]_gi|499110212|ref|YP_007974374.1|helicasedomaincontainingprotein/        ------------------------------------------------------------
gi|940235551|gb|KPY45311.1|hypotheticalproteinALO48_200154[Pseudomonassyringaepv.rhaphiolepidis]/11631                                 ------------------------------------------------------------
gi|520880808|ref|WP_020312235.1|helicase[Pseudomonassyringae]_gi|519172158|gb|EPM62027.1|helicasedomaincontainingprotein[Pseud/        ------------------------------------------------------------
gi|829046479|ref|WP_047282259.1|damageinducibleprotein[Pseudomonasfluorescens]/11630                                                    ------------------------------------------------------------
gi|499415493|ref|WP_011102960.1|DNAdamageinducibleprotein[Pseudomonassyringaegroupgenomosp.3]_gi|28867279|ref|NP_789898.1|heli/        ------------------------------------------------------------
gi|730238299|ref|WP_033942897.1|damageinducibleprotein[Pseudomonasaeruginosa]/11629                                                     ------------------------------------------------------------
gi|495310125|ref|WP_008034873.1|DNAdamageinducibleprotein[Pseudomonassp.GM60]_gi|398186685|gb|EJM74052.1|putativehelicase[Pseu/        ------------------------------------------------------------
gi|779992653|ref|WP_045428690.1|damageinducibleprotein[Pseudomonassp.MT1]_gi|699967872|dbj|BAP80334.1|DNAhelicaserestrictione/1        ------------------------------------------------------------
gi|786037289|ref|WP_045733274.1|damageinducibleprotein[Pseudomonaspseudoalcaligenes]_gi|782987904|gb|KJU79152.1|damageinducib/1        ------------------------------------------------------------
gi|749666366|ref|WP_040265445.1|damageinducibleprotein[Pseudomonasrhodesiae]/11632                                                      ------------------------------------------------------------
gi|734902433|ref|WP_034133698.1|damageinducibleprotein[Pseudomonasfluorescens]/11635                                                    ------------------------------------------------------------
gi|490551031|ref|WP_004416146.1|typeIIrestrictionmodificationsystemrestriction/helicasesubunit[Pseudomonassyringae]_gi|4432823/        ------------------------------------------------------------
gi|489199911|ref|WP_003109152.1|DNAdamageinducibleprotein[Pseudomonasaeruginosa]_gi|552648708|gb|ERY63184.1|hypotheticalprotei/        ------------------------------------------------------------
gi|730641237|ref|WP_034061328.1|damageinducibleprotein[Pseudomonasaeruginosa]/11636                                                     ------------------------------------------------------------
gi|751304980|ref|WP_041013386.1|damageinducibleprotein[Pseudomonasxanthomarina]_gi|703407611|emb|CEG51014.1|Helicasedomaincon/1        ------------------------------------------------------------
gi|939335207|ref|WP_054881520.1|damageinducibleprotein[Pseudomonassp.NBRC111128]/11632                                                  ------------------------------------------------------------
gi|757693555|ref|WP_042923792.1|damageinducibleprotein[Pseudomonaschloritidismutans]_gi|780910610|gb|KJS78302.1|damageinducib/1        ------------------------------------------------------------
gi|665953944|ref|WP_031319989.1|damageinducibleprotein[Pseudomonastolaasii]/11626                                                       ------------------------------------------------------------
gi|771600573|ref|WP_045213746.1|damageinducibleprotein[Pseudomonassp.21]_gi|770081123|gb|KJJ98634.1|damageinducibleprotein[Ps/1        ------------------------------------------------------------
gi|740871396|ref|WP_038656644.1|damageinducibleprotein[Pseudomonasstutzeri]_gi|627772214|gb|AHY41437.1|damageinducibleprotein/1        ------------------------------------------------------------
gi|946242205|gb|KRC93782.1|damageinducibleprotein[Pseudomonassp.Root9]/11634                                                           ------------------------------------------------------------
gi|553777322|ref|WP_023109107.1|MULTISPECIES:hypotheticalprotein[Pseudomonas]_gi|552638674|gb|ERY53184.1|hypotheticalproteinQ05        ------------------------------------------------------------
```

```
gi|896383370|ref|WP_049324618.1|damageinducibleprotein[Pseudomonasstutzeri]/11627      ------------------------------------------------------------
gi|696158510|gb|AIT41787.1|putativehelicase[Pseudomonassp.LM8]/11630      ------------------------------------------------------------
gi|504634892|ref|WP_014821994.1|DNAdamageinducibleprotein[Pseudomonasstutzeri]_gi|390985249|gb|AFM35242.1|helicasedomainconta/1      ------------------------------------------------------------
gi|544783974|ref|WP_021206729.1|DNAdamageinducibleprotein[Pseudomonasstutzeri]_gi|533224779|gb|EQM79688.1|DNAdamageinduciblep/1      ------------------------------------------------------------
gi|504567385|ref|WP_014754487.1|MULTISPECIES:DNAdamageinducibleprotein[Pseudomonas]_gi|388559493|gb|AFK68634.1|helicasedomain/1      ------------------------------------------------------------
gi|654441469|ref|WP_027912052.1|damageinducibleprotein[Pseudomonassp.URIL14HWK12:I7]/11630      ------------------------------------------------------------
gi|941470066|ref|WP_055127894.1|damageinducibleprotein[Pseudomonasmediterranea]/11631      ------------------------------------------------------------
gi|557815599|ref|WP_023446184.1|DNAdamageinducibleprotein[Pseudomonaschloritidismutans]_gi|557364081|gb|ESQ98190.1|DNAdamagei/1      ------------------------------------------------------------
gi|727814791|ref|WP_033898247.1|damageinducibleprotein[Pseudomonassp.FH1]_gi|567648005|gb|ETK23229.1|helicasedomaincontaining/1      ------------------------------------------------------------
gi|503278702|ref|WP_013513363.1|DNAdamageinducibleprotein[Desulfovibrioaespoeensis]_gi|316942375|gb|ADU61426.1|typeIIIrestrict/      ------------------------------------------------------------
gi|505228796|ref|WP_015415898.1|typeIIIrestrictionendonucleasesubunitR[Desulfovibriopiezophilus]_gi|451896976|emb|CCH49855.1|Ty      ------------------------------------------------------------
gi|517732543|ref|WP_018902751.1|hypotheticalprotein[Variovoraxparadoxus]/11675      ------------------------------------------------------------
gi|550909475|ref|WP_022662570.1|DNAdamageinducibleprotein[Desulfovibriolongus]/11637      ------------------------------------------------------------
gi|945494542|gb|KQV50024.1|damageinducibleprotein[Massiliasp.Root335]/11647      ------------------------------------------------------------
gi|334196917|gb|AEG70102.1|helicasedomainprotein[RalstoniasolanacearumPo82]/11663      ------------------------------------------------------------
gi|656006746|ref|WP_029048170.1|damageinducibleprotein[Cupriavidussp.amp6]/11661      ------------------------------------------------------------
BPS2196ORF3054P/11659      ------------------------------------------------------------
gi|740932312|ref|WP_038717332.1|damageinducibleprotein[Burkholderiapseudomallei]_gi|760211882|gb|KIX58314.1|damageinduciblepr/1      ------------------------------------------------------------
gi|755810813|ref|WP_042583861.1|damageinducibleprotein[Burkholderiasp.MSHR3999]_gi|755174952|gb|KIP19790.1|DEAD/DEAHboxhelicas/      ------------------------------------------------------------
gi|484586570|gb|AGK48195.1|DEAD/DEAHboxhelicasefamilyprotein[BurkholderiathailandensisMSMB121]/11646      ------------------------------------------------------------
gi|737582935|ref|WP_035553941.1|damageinducibleprotein[Burkholderiasp.9120]/11658      ------------------------------------------------------------
gi|740989872|ref|WP_038773225.1|damageinducibleprotein[Burkholderiapseudomallei]_gi|705645291|gb|KGS70691.1|DEAD/DEAHboxhelica/      ------------------------------------------------------------
gi|654912525|ref|WP_028363424.1|damageinducibleprotein[Burkholderiasp.JPY366]/11659      ------------------------------------------------------------
gi|740955879|ref|WP_038740512.1|damageinducibleprotein[Burkholderiapseudomallei]_gi|714684932|gb|KGW74438.1|DEAD/DEAHboxhelica/      ------------------------------------------------------------
gi|919203714|ref|WP_052759215.1|damageinducibleprotein[Xanthomonasaxonopodis]/11388      ------------------------------------------------------------
gi|522152381|ref|WP_020663589.1|hypotheticalprotein[Amycolatopsisbenzoatilytica]/11605      ------------------------------------------------------------
gi|663196013|ref|WP_030233899.1|damageinducibleprotein[Streptomycessp.NRRLS455]/11641      ------------------------------------------------------------
gi|750429553|ref|WP_040711444.1|damageinducibleprotein[Nocardiopsispotens]/11622      ------------------------------------------------------------
gi|518656139|ref|WP_019819475.1|hypotheticalprotein[Saccharomonosporasaliphila]/11623      ------------------------------------------------------------
gi|759722933|ref|WP_043433758.1|damageinducibleprotein[Streptomycespluripotens]_gi|733404978|gb|KHK94361.1|damageinduciblepro/1      ------------------------------------------------------------
gi|663424167|ref|WP_030419030.1|damageinducibleprotein[Streptomycessp.NRRLF5065]/11627      ------------------------------------------------------------
gi|663412917|ref|WP_030408353.1|damageinducibleprotein[Streptomycessp.NRRLF5917]/11630      ------------------------------------------------------------
gi|671521483|ref|WP_031506489.1|damageinducibleprotein[Streptomycesmegasporus]/11630      ------------------------------------------------------------
gi|740053144|ref|WP_037902776.1|damageinducibleprotein[Streptomycessp.TAA204]/11626      ------------------------------------------------------------
gi|663409375|ref|WP_030404974.1|MULTISPECIES:damageinducibleprotein[Streptomyces]/11627      ------------------------------------------------------------
gi|746628608|ref|WP_039654277.1|damageinducibleprotein[Streptomycessp.MUSC125]_gi|745644381|gb|KIE25557.1|damageinducibleprot/1      ------------------------------------------------------------
gi|727322066|ref|WP_033777320.1|damageinducibleprotein[Salinisporapacifica]/11627      ------------------------------------------------------------
gi|653653242|ref|WP_027655726.1|damageinducibleprotein[Salinisporapacifica]/11618      ------------------------------------------------------------
gi|648632543|ref|WP_026324294.1|damageinducibleprotein[Salinisporapacifica]/11621      ------------------------------------------------------------
gi|881018002|ref|WP_048738463.1|damageinducibleprotein[Corynebacteriumfalsenii]/11676      ------------------------------------------------------------
gi|505465526|ref|WP_015652417.1|helicase[Corynebacteriumcallunae]_gi|459385704|gb|AGG67995.1|helicase[CorynebacteriumcallunaeDS      ------------------------------------------------------------
gi|41223062|emb|CAF19007.1|DNAorRNAhelicaseofsuperfamilyII[CorynebacteriumglutamicumATCC13032]/11646      ------------------------------------------------------------
CGL13032I/11643      ------------------------------------------------------------
gi|751257081|ref|WP_040967675.1|damageinducibleprotein[Corynebacteriumglutamicum]_gi|748811620|gb|AJE67931.1|damageinduciblep/1      ------------------------------------------------------------
gi|749040539|ref|WP_040086524.1|damageinducibleprotein[Corynebacteriumhumireducens]/11663      ------------------------------------------------------------
gi|491662899|ref|WP_005519615.1|helicase[Corynebacteriummatruchotii]_gi|224946756|gb|EEG27965.1|helicaseCterminaldomainprotein/      ------------------------------------------------------------
gi|746717312|ref|WP_039676231.1|damageinducibleprotein[Corynebacteriumminutissimum]_gi|734991118|gb|KHO29219.1|damageinducibl/1      ------------------------------------------------------------
gi|908654892|emb|CQD15529.1|SuperfamilyIIDNA/RNAhelicase(modularprotein)[Corynebacteriumstriatum]/11674      ------------------------------------------------------------
gi|895994446|ref|WP_049064418.1|damageinducibleprotein[Corynebacteriumaurimucosum]/11667      ------------------------------------------------------------
gi|896123331|ref|WP_049145390.1|damageinducibleprotein[Corynebacteriumaurimucosum]/11669      ------------------------------------------------------------
gi|817681178|ref|WP_046645422.1|damageinducibleprotein[Corynebacteriumstriatum]_gi|817551679|gb|KKO79868.1|damageinduciblepro/1      ------------------------------------------------------------
gi|896139239|ref|WP_049159146.1|damageinducibleprotein[Corynebacteriumaurimucosum]/11675      ------------------------------------------------------------
gi|489943947|ref|WP_003847254.1|helicase[Corynebacteriumammoniagenes]_gi|295968218|gb|EFG81467.1|helicaseCterminaldomainprotei/      ------------------------------------------------------------
gi|516653464|ref|WP_018022453.1|helicase[Corynebacteriumdoosanense]_gi|697778282|gb|AIT61018.1|damageinducibleprotein[Coryneba/      ------------------------------------------------------------
gi|493894179|ref|WP_006840129.1|helicase[Corynebacteriumlipophiloflavum]_gi|227078819|gb|EEI16782.1|helicaseCterminaldomainpro/      ------------------------------------------------------------
gi|740831472|ref|WP_038616755.1|damageinducibleprotein[Corynebacteriumpseudotuberculosis]_gi|666103058|gb|AIG06935.1|putativeh/      ------------------------------------------------------------
gi|518023322|ref|WP_019193530.1|helicase[Corynebacteriumtimonense]/11655      ------------------------------------------------------------
gi|552767697|ref|WP_023019559.1|MULTISPECIES:hypotheticalprotein[Corynebacterium]_gi|550739013|gb|ERS38609.1|hypotheticalprotei      ------------------------------------------------------------
gi|652659121|ref|WP_027017812.1|damageinducibleprotein[Corynebacteriumpseudodiphtheriticum]/11644      ------------------------------------------------------------
gi|749039309|ref|WP_040085390.1|damageinducibleprotein[Corynebacteriumhumireducens]/11657      ------------------------------------------------------------
gi|740835591|ref|WP_038620874.1|damageinducibleprotein[Corynebacteriumulcerans]_gi|698973854|gb|AIU33664.1|HelicaseCterminald/1      ------------------------------------------------------------
gi|805307599|ref|WP_046096452.1|damageinducibleprotein[Corynebacteriumulcerans]_gi|803357323|gb|AKA97638.1|Damageinduciblepro/1      ------------------------------------------------------------
gi|504083555|ref|WP_014317549.1|helicase[Corynebacteriumdiphtheriae]_gi|371584249|gb|AEX47914.1|superfamilyIIDNA/RNAhelicase[Co      ------------------------------------------------------------
gi|896182193|ref|WP_049193580.1|damageinducibleprotein[Corynebacteriumjeikeium]/11645      ------------------------------------------------------------
gi|917636459|ref|WP_052205460.1|damageinducibleprotein[Corynebacteriumriegelii]_gi|914295811|gb|AKV59187.1|damageinduciblepro/1      ------------------------------------------------------------
gi|737013522|ref|WP_035009205.1|damageinducibleprotein[Corynebacteriumjeikeium]/11651      ------------------------------------------------------------
gi|737006291|ref|WP_035002094.1|damageinducibleprotein[Corynebacteriumjeikeium]/11658      ------------------------------------------------------------
```

```
gi|737010378|ref|WP_035006133.1|damageinducibleprotein[Corynebacteriumjeikeium]/11650    ------------------------------------------------------------
gi|737010473|ref|WP_035006228.1|damageinducibleprotein[Corynebacteriumjeikeium]/11657    ------------------------------------------------------------
gi|737006250|ref|WP_035002053.1|damageinducibleprotein[Corynebacteriumjeikeium]/11658    ------------------------------------------------------------
gi|923018168|ref|WP_053412254.1|damageinducibleprotein[Corynebacteriumlactis]_gi|922845184|gb|ALA67504.1|helicase[Corynebacter/    ------------------------------------------------------------
gi|923018885|ref|WP_053412970.1|damageinducibleprotein[Corynebacteriumlactis]_gi|922845845|gb|ALA68165.1|helicase[Corynebacter/    ------------------------------------------------------------
gi|512464870|ref|WP_016422869.1|hypotheticalprotein[Corynebacteriumsp.HFH0082]_gi|512047830|gb|EPD47505.1|hypotheticalproteinHM    ------------------------------------------------------------
gi|881012697|ref|WP_048733406.1|damageinducibleprotein[Corynebacteriumurealyticum]/11670    ------------------------------------------------------------
gi|755608478|ref|WP_042530519.1|damageinducibleprotein[Corynebacteriumsingulare]_gi|754340239|gb|AJI78730.1|putativehelicase[C/    ------------------------------------------------------------
gi|491421796|ref|WP_005279593.1|helicase[Corynebacteriumaccolens]_gi|227077180|gb|EEI15143.1|helicaseCterminaldomainprotein[Co/    ------------------------------------------------------------
gi|550752286|gb|ERS51451.1|hypotheticalproteinHMPREF1282_00033[Corynebacteriumsp.KPL1856]_gi|550759961|gb|ERS58964.1|hypothetic    ------------------------------------------------------------
gi|497955053|ref|WP_010269209.1|helicase[Corynebacteriumbovis]/11599    ------------------------------------------------------------
gi|860580040|ref|WP_048470569.1|hypotheticalprotein[Mycobacteriumchlorophenolicum]_gi|859098575|gb|KMO76871.1|typeIrestrictione    ------------------------------------------------------------
gi|262085905|gb|ACY21873.1|typeIIIrestrictionproteinressubunit[GordoniabronchialisDSM43247]/11632    ------------------------------------------------------------
gi|495996338|ref|WP_008720917.1|typeIIIrestrictionendonucleasesubunitR[Rhodococcussp.AW25M09]_gi|443412350|emb|CCQ18455.1|TypeI    ------------------------------------------------------------
gi|739284636|ref|WP_037147410.1|damageinducibleprotein[Rhodococcusfascians]/11641    ------------------------------------------------------------
gi|739328195|ref|WP_037190574.1|damageinducibleprotein[Rhodococcusfascians]/11643    ------------------------------------------------------------
gi|811698335|emb|CMN21041.1|helicase[Mycobacteriumtuberculosis]/11725    ------------------------------------------------------------
gi|801577898|emb|CFK23773.1|helicase[Mycobacteriumtuberculosis]/11675    ------------------------------------------------------------
MTU18II/11606    ------------------------------------------------------------
gi|887719858|emb|CMT27886.1|helicase[Mycobacteriumtuberculosis]/11667    ------------------------------------------------------------
gi|621694743|gb|KAS87156.1|hypotheticalproteinAL70_01801[MycobacteriumtuberculosisTKK_03_0115]/11604    ------------------------------------------------------------
gi|888128072|emb|CLQ84422.1|helicase[Mycobacteriumtuberculosis]_gi|889917090|emb|CLY86893.1|helicase[Mycobacteriumtuberculosis]    ------------------------------------------------------------
gi|884823424|emb|CKL96672.1|helicase[Mycobacteriumtuberculosis]/11466    ------------------------------------------------------------
gi|505103239|ref|WP_015290341.1|hypotheticalprotein[Mycobacteriumcanettii]_gi|432162733|emb|CCK60117.1|Conservedproteinofunknow    ------------------------------------------------------------
gi|505116250|ref|WP_015303352.1|hypotheticalprotein[Mycobacteriumcanettii]_gi|432158726|emb|CCK56026.1|Conservedproteinofunknow    ------------------------------------------------------------
gi|432154735|emb|CCK51974.1|Conservedproteinofunknownfunction(part1)[MycobacteriumcanettiiCIPT140060008]/11588    ------------------------------------------------------------
gi|809008027|ref|WP_046295375.1|hypotheticalprotein[Mycobacteriumsp.UM_Kg17]/11624    ------------------------------------------------------------
gi|808802633|ref|WP_046284139.1|hypotheticalprotein[Mycobacteriumsp.UM_NZ2]/11624    ------------------------------------------------------------
gi|750125774|ref|WP_040429852.1|damageinducibleprotein[Corynebacteriumnuruki]/11668    ------------------------------------------------------------
gi|602237614|gb|EYT60960.1|damageinducibleprotein[Dietziasp.UCDTHP]/11473    ------------------------------------------------------------
gi|665897412|ref|WP_031264618.1|damageinducibleprotein[Dietziasp.UCDTHP]_gi|602237599|gb|EYT60945.1|helicase[Dietziasp.UCDTH/11    ------------------------------------------------------------
gi|502903674|ref|WP_013138650.1|helicase[Segniliparusrotundus]_gi|296181291|gb|ADG98197.1|typeIIIrestrictionproteinressubunit[S    ------------------------------------------------------------
gi|780248306|ref|WP_045538930.1|hypotheticalprotein[Gordoniasp.no.9]_gi|778142514|gb|KJR06917.1|hypotheticalproteinUG54_12320[G    ------------------------------------------------------------
gi|665897368|ref|WP_031264574.1|damageinducibleprotein[Dietziasp.UCDTHP]_gi|602239137|gb|EYT62483.1|helicase[Dietziasp.UCDTH/11    ------------------------------------------------------------
gi|493372923|ref|WP_006329236.1|helicase[Gordoniarhizosphera]_gi|403207917|dbj|GAB88168.1|hypotheticalproteinGORHZ_006_00370[Go    ------------------------------------------------------------
gi|764904870|ref|WP_044487821.1|hypotheticalprotein[Mycobacteriumtuberculosis]_gi|625005353|gb|KBZ57120.1|hypotheticalproteinK8    ------------------------------------------------------------
gi|927068212|ref|WP_053778441.1|damageinducibleprotein[Gordoniasp.NB41Y]_gi|926660541|gb|EMP14526.2|damageinducibleprotein[G/11    ------------------------------------------------------------
gi|935514169|ref|WP_054429894.1|damageinducibleprotein[Mycobacteriumimmunogenum]_gi|929801120|gb|KPG29247.1|damageinduciblepr/1    ------------------------------------------------------------
gi|941317728|ref|WP_055118574.1|hypotheticalprotein[Mycobacteriumperegrinum]/11657    ------------------------------------------------------------
gi|933683673|gb|ALG86377.1|hypotheticalproteinACH46_20120[Gordoniasp.QH11]/11636    ------------------------------------------------------------
gi|873553947|emb|CRY84110.1|Predictedhelicase[Nocardiafarcinica]/11688    -------------------------MPYTVLTA----------------YA---------
gi|499525749|ref|WP_011212389.1|helicase[Nocardiafarcinica]_gi|54019339|dbj|BAD60707.1|hypotheticalprotein[NocardiafarcinicaIFM    ------------------------------------------------------------
gi|640109609|ref|WP_024794187.1|damageinducibleprotein[Tomitellabiformata]/11653    ------------------------------------------------------------
gi|640109991|ref|WP_024794334.1|hypotheticalprotein[Tomitellabiformata]/11639    ------------------------------------------------------------
gi|939125496|ref|WP_054831438.1|damageinducibleprotein[Rhodococcuserythropolis]/11669    ------------------------------------------------------------
gi|910258550|ref|WP_050062711.1|hypotheticalprotein[Rhodococcussp.RD6.2]_gi|846871967|emb|CRK49225.1|conservedhypotheticalprote    ------------------------------------------------------------
gi|797148189|gb|KJY52358.1|putativeHelicase[Bifidobacteriumasteroides]/11801    ------------------------------------------------------------
gi|535476570|gb|EFG27357.2|hypotheticalproteinHMPREF9020_00999[ScardoviainopinataF0304]/11827    ----------------------------------------------MSMFS---------
gi|737482729|ref|WP_035462496.1|hypotheticalprotein[Alicyclobacillusmacrosporangiidus]/11512    ------------------------------------------------------------
gi|685434354|gb|KGA95679.1|helicase[BacillusalcalophilusATCC27647=CGMCC1.3604]/11575    ------------------------------------------------------------
gi|945370253|gb|KQU27251.1|helicase[Bacillussp.Leaf13]/11557    ------------------------------------------------------------
gi|880997015|ref|WP_048719116.1|helicase[Enterococcushirae]/11563    ------------------------------------------------------------
gi|945356929|gb|KQU14100.1|helicase[Bacillussp.Leaf49]/11547    ------------------------------------------------------------
gi|763482876|gb|KIZ53217.1|helicase[Bacillussafensis]/11563    ------------------------------------------------------------
gi|852223292|ref|WP_048309707.1|helicase[Anaerobacillusmacyae]_gi|850322910|gb|KMM38590.1|helicase[Anaerobacillusmacyae]/11573    ------------------------------------------------------------
gi|739708665|ref|WP_037562619.1|helicase[Sporolactobacillusterrae]/11573    ------------------------------------------------------------
gi|496687561|ref|WP_009329104.1|MULTISPECIES:helicase[Bacillus]_gi|317390867|gb|EFV71668.1|endonucleaseandmethylaseLlaGI[Bacill    ------------------------------------------------------------
gi|930213599|gb|KPH74225.1|helicase[Oceanobacilluscaeni]/11569    ------------------------------------------------------------
gi|516366003|ref|WP_017756036.1|helicase[Bacillussp.ZYK]/11571    ------------------------------------------------------------
gi|488909977|ref|WP_002821052.1|helicase[Oenococcusoeni]_gi|399965230|gb|EJN99855.1|endonucleaseandmethylaseLlaGI[Oenococcusoen    ------------------------------------------------------------
gi|495019967|ref|WP_007745780.1|helicase[Oenococcuskitaharae]_gi|366983682|gb|EHN59081.1|typeIIIrestrictionenzyme,ressubunit[Oe    ------------------------------------------------------------
gi|489666119|ref|WP_003570406.1|typeIIIrestrictionendonuclease[Lactobacilluscasei]_gi|410527834|gb|EKQ02696.1|typeIIIrestrictio    ------------------------------------------------------------
gi|511447623|gb|EPC78110.1|superfamilyIIDNA/RNAhelicase[Lactobacillusparacaseisubsp.paracaseiLpp221}/11615    ------------------------------TDRQTDRQTDRQTDRQTDRQT---------
gi|497700411|ref|WP_010014595.1|helicase[Lactobacilluscoryniformis]/11585    ------------------------------------------------------------
gi|489784048|ref|WP_003687939.1|helicase[Lactobacillusmali]_gi|394428539|gb|EJF01080.1|superfamilyIIDNA/RNAhelicase[Lactobacill    ------------------------------------------------------------
gi|446780825|ref|WP_000858081.1|helicase[Staphylococcusaureus]_gi|374396604|gb|EHQ67835.1|typeIIIrestrictionenzyme,ressubunit[S    ------------------------------------------------------------
gi|510796658|ref|WP_016171456.1|hypotheticalprotein[Enterococcusdispar]_gi|508167532|gb|EOT44083.1|hypotheticalproteinOMK_00227    ------------------------------------------------------------
```

```
gi|612613473|gb|EZS30411.1|hypotheticalproteinW603_02482[StaphylococcusaureusVET0353R]_gi|612881917|gb|EZU93992.1|hypotheticalp      ------------------------------------------------------------
gi|488332893|ref|WP_002402278.1|helicase[Enterococcusfaecalis]_gi|306513864|gb|EFM82466.1|helicaseCterminaldomainprotein[Enter/      ------------------------------------------------------------
gi|921166029|ref|WP_053111806.1|helicase[Enterococcusdurans]_gi|915771660|gb|AKX85928.1|helicase[Enterococcusdurans]/11562          ------------------------------------------------------------
gi|498525358|ref|WP_010825030.1|hypotheticalprotein[Enterococcusfaecalis]_gi|487059631|gb|EOJ86727.1|hypotheticalproteinWOC_005      ------------------------------------------------------------
gi|498412624|ref|WP_010722070.1|hypotheticalprotein[Enterococcusfaecium]_gi|486634654|gb|EOF75253.1|hypotheticalproteinSGC_0253      ------------------------------------------------------------
gi|498408534|ref|WP_010718364.1|hypotheticalprotein[Enterococcusfaecium]_gi|486609608|gb|EOF50873.1|hypotheticalproteinSCW_0257      ------------------------------------------------------------
gi|498407475|ref|WP_010717483.1|hypotheticalprotein[Enterococcusfaecalis]_gi|486594855|gb|EOF36348.1|hypotheticalproteinSCK_005      ------------------------------------------------------------
gi|514885392|ref|WP_016615344.1|helicase[Enterococcusfaecalis]_gi|514505843|gb|EPH71676.1|helicaseprotein[Enterococcusfaecalis0      ------------------------------------------------------------
gi|325157603|gb|EGC69759.1|helicaseCterminaldomainprotein[EnterococcuscasseliflavusATCC12755]/11561                                  ------------------------------------------------------------
gi|895856206|ref|WP_048967668.1|helicase[Enterococcusfaecalis]/11551                                                                 ------------------------------------------------------------
gi|639458836|ref|WP_024636366.1|helicase[Enterococcusfaecium]_gi|658176502|gb|KEI51159.1|helicase[EnterococcusfaeciumUC7256]/1/      ------------------------------------------------------------
gi|488266511|ref|WP_002337719.1|hypotheticalprotein[Enterococcusfaecium]_gi|430581409|gb|ELB19854.1|hypotheticalproteinOIQ_0351      ------------------------------------------------------------
gi|446353356|ref|WP_000431211.1|helicase[Staphylococcusaureus]_gi|253726329|gb|EES95058.1|helicaseCterminaldomainprotein[Staph/      ------------------------------------------------------------
gi|672712068|emb|CDM14336.1|DNAorRNAhelicaseofsuperfamilyII[StaphylococcusepidermidisPM221]/11464                                   ------------------------------------------------------------
gi|896298990|ref|WP_049279905.1|helicase[Staphylococcusaureus]/11571                                                                 ------------------------------------------------------------
gi|541903432|gb|ERH36190.1|hypotheticalproteinSEQU_00085[StaphylococcusequorumUMCCNS924]/11578                                       ------------------------------------------------------------
gi|488289747|ref|WP_002360955.1|helicase[Enterococcusfaecalis]_gi|256682216|gb|EEU21911.1|endonucleaseandmethylaseLlaGI[Enteroc      ------------------------------------------------------------
gi|494392045|ref|WP_007207894.1|helicase[Enterococcusitalicus]_gi|315483798|gb|EFU74284.1|helicaseCterminaldomainprotein[Enter/      ------------------------------------------------------------
gi|446350692|ref|WP_000428547.1|helicase[Bacilluscereus]_gi|228727580|gb|EEL78721.1|RestrictionmodificationsystemLlaBIII[Bacil/      ------------------------------------------------------------
gi|488255302|ref|WP_002326510.1|hypotheticalprotein[Enterococcusfaecium]_gi|430480276|gb|ELA57459.1|hypotheticalproteinOGE_0473      ------------------------------------------------------------
gi|843253770|ref|WP_047916698.1|helicase[Lactococcuspiscium]_gi|802124677|emb|CEN29502.1|EndonucleaseandmethylaseLlaGI,TypeISPr      ------------------------------------------------------------
LlaBIII/11578                                                                                                                        ------------------------------------------------------------
gi|499320531|ref|WP_011011023.1|helicase[Listeriainnocua]_gi|16415805|emb|CAC42019.1|pli0021[ListeriainnocuaClip11262]/11569         ------------------------------------------------------------
gi|760026859|ref|WP_043709973.1|helicase[Weissellacibaria]_gi|758337165|gb|KIU20061.1|UvsWhelicase[Weissellacibaria]/11567           ------------------------------------------------------------
LlaGI/11570                                                                                                                          ------------------------------------------------------------
gi|499988414|ref|WP_011669132.1|helicase[Lactococcuslactis]_gi|116109019|gb|ABJ74139.1|DNAorRNAhelicaseofsuperfamilyII[Lactococ      ------------------------------------------------------------
gi|685938360|ref|WP_031670319.1|helicase[Listeriamonocytogenes]_gi|590403387|gb|EXL23261.1|hypotheticalproteinX846_1956[Listeri      ------------------------------------------------------------
gi|530793219|gb|EQC56240.1|helicase[Lactococcuslactissubsp.cremorisTIFN5]_gi|530793247|gb|EQC56263.1|helicase[Lactococcuslactis      ------------------------------------------------------------
gi|695270006|ref|WP_032492075.1|hypotheticalprotein[Listeriamonocytogenes]_gi|695212872|ref|YP_009081522.1|hypotheticalproteinp      ------------------------------------------------------------
gi|504101310|ref|WP_014335297.1|helicase[Leuconostocmesenteroides]_gi|859247905|gb|AKO63885.1|helicase(plasmid)[Leuconostocmese      ------------------------------------------------------------
gi|488905190|ref|WP_002816265.1|helicase[Leuconostocmesenteroides]_gi|227351522|gb|EEJ41777.1|helicaseCterminaldomainprotein[L/      ------------------------------------------------------------
gi|518121285|ref|WP_019291493.1|MULTISPECIES:hypotheticalprotein[Lactococcus]/11588                                                  ------------------------------------------------------------
gi|754857213|ref|WP_042217902.1|helicase[Lactococcusgarvieae]/11549                                                                  ------------------------------------------------------------
gi|640048455|ref|WP_024784762.1|helicase[Streptococcusmutans]/11564                                                                  ------------------------------------------------------------
gi|512695361|ref|WP_016476323.1|hypotheticalprotein[Veillonellasp.HPA0037]_gi|512079853|gb|EPD79018.1|hypotheticalproteinHMPREF      ------------------------------------------------------------
gi|636963514|ref|WP_024403747.1|helicase[Streptococcussuis]/11574                                                                    ------------------------------------------------------------
gi|881115312|ref|WP_048802949.1|helicase[Staphylococcuspasteuri]/11573                                                               ------------------------------------------------------------
gi|446063996|ref|WP_000141851.1|helicase[Streptococcusmitis]_gi|307618725|gb|EFN97864.1|endonucleaseandmethylaseLlaGI[Streptoco      ------------------------------------------------------------
gi|488206609|ref|WP_002277817.1|restrictionmodificationsystemLlaBIII[Streptococcusmutans]_gi|449173033|gb|EMB75626.1|restricti/      ------------------------------------------------------------
gi|640023290|ref|WP_024781621.1|helicase[Streptococcusmutans]/11566                                                                  ------------------------------------------------------------
gi|814946581|ref|WP_046393781.1|helicase[Streptococcusuberis]_gi|810600885|gb|KKF57499.1|helicase[StreptococcusuberisB362]/1156      ------------------------------------------------------------
gi|765470762|ref|WP_044720336.1|helicase[Streptococcussuis]/11559                                                                    ------------------------------------------------------------
gi|654507127|ref|WP_027976443.1|helicase[Streptococcusdevriesei]/11558                                                               ------------------------------------------------------------
gi|530812902|gb|EQC72481.1|DNAorRNAhelicaseofsuperfamilyII[Streptococcussp.HSISS2]/11570                                             ------------------------------------------------------------
gi|489158846|ref|WP_003068504.1|helicase[Streptococcusintermedius]_gi|355367114|gb|EHG14827.1|hypotheticalproteinHMPREF9682_002      ------------------------------------------------------------
RM.Lru27782ORF13200P/11620                                                                                                           ---------------------MIREAFFLIISHLVSASGSII---AGEQFK---------
gi|492676546|ref|WP_005919236.1|DNAphosphorothioationsystemrestrictionenzyme[Pediococcusacidilactici]_gi|482517924|gb|EOA08103.      ------------------------------------------------------------
gi|498183116|ref|WP_010497272.1|helicase[Lactobacillusacidipiscis]/11563                                                             ------------------------------------------------------------
gi|822100485|ref|WP_046921865.1|helicase[Lactobacillusruminis]_gi|821684442|gb|KLA43321.1|restrictionmodificationsystemLlaBIII/      ------------------------------------------------------------
gi|935565832|ref|WP_054450311.1|helicase[Lactobacilluskunkeei]/11573                                                                 ------------------------------------------------------------
gi|752296887|ref|WP_041152901.1|helicase[Lactobacilluskunkeei]_gi|751663520|gb|KIM18358.1|helicase[Lactobacilluskunkeei]/11577      ------------------------------------------------------------
gi|495745291|ref|WP_008469870.1|helicase[Lactobacillushominis]_gi|394484442|emb|CCI81207.1|RestrictionmodificationsystemLlaBII/      ------------------------------------------------------------
gi|918672414|ref|WP_052543134.1|helicase[Lactobacillussp.]/11564                                                                     ------------------------------------------------------------
gi|799141631|ref|WP_045927261.1|helicase[Lactobacilluskullabergensis]_gi|797155335|gb|KJY59264.1|putativerestrictionendonucleas      ------------------------------------------------------------
gi|504379990|ref|WP_014567092.1|helicase[Lactobacillusjohnsonii]_gi|329666726|gb|AEB92674.1|putativerestrictionendonuclease[Lac      ------------------------------------------------------------
gi|489880179|ref|WP_003783646.1|DNAdamageinducibleprotein[Actinomycesnaeslundii]_gi|399901561|gb|EJN84440.1|typeIIrestriction/      ------------------------------------------------------------
gi|496524497|ref|WP_009232780.1|DNAdamageinducibleprotein[Actinomycessp.oraltaxon849]_gi|365265413|gb|EHM95177.1|hypotheticalp/      ------------------------------------------------------------
gi|915397782|ref|WP_050783167.1|damageinducibleprotein[Actinomycesoris]/11694                                                        ------------------------------------------------------------
gi|502925248|ref|WP_013160224.1|helicase[Propionibacteriumfreudenreichii]_gi|296921272|emb|CBL55824.1|helicase[Propionibacteriu      ------------------------------------------------------------
gi|732164352|emb|CEG87481.1|helicase[Propionibacteriumfreudenreichii]/11618                                                          ------------------------------------------------MK----------
gi|739047402|ref|WP_036925058.1|damageinducibleprotein[Propionibacteriumthoenii]/11596                                               ------------------------------------------------------------
gi|739112839|ref|WP_036982533.1|damageinducibleprotein[Propionibacteriumjensenii]/11594                                              ------------------------------------------------------------
gi|918023558|ref|WP_052314121.1|damageinducibleprotein[Nocardiathailandica]/11612                                                    --------------------------------------------MSVLIS----------
gi|498380271|ref|WP_010694427.1|DNAdamageinducibleprotein[Saccharopolysporaspinosa]/11605                                            ------------------------------------------------------------
gi|749991459|ref|WP_040339809.1|damageinducibleprotein[CandidatusBlastococcusmassiliensis]/11597                                     ------------------------------------------------------------
gi|754177801|ref|WP_041796231.1|damageinducibleprotein[Modestobactermarinus]/11592                                                   ------------------------------------------------------------
gi|504188248|ref|WP_014375350.1|DNAdamageinducibleprotein[Blastococcussaxobsidens]_gi|378782790|emb|CCG02456.1|putativeHelicas/      ------------------------------------------------------------
```

```
gi|946476713|gb|KRF26798.1|damageinducibleprotein[Phycicoccussp.Soil803]/11609      ------------------------------------------------------------
gi|700176672|gb|KGN37509.1|DNAdamageinducibleprotein[KnoelliasubterraneaKCTC19937]/11633      ------------------------------------------------------------
gi|700168841|gb|KGN29914.1|hypotheticalproteinN802_10215[KnoelliasinensisKCTC19936]/11631      ------------------------------------------------------------
gi|946426241|gb|KRE76610.1|damageinducibleprotein[Arthrobactersp.Soil763]/11606      ------------------------------------------------------------
gi|517318525|ref|WP_018500927.1|hypotheticalprotein[Frankiasp.BCU110501]/11592      ------------------------------------------------------------
gi|499072429|ref|WP_010851327.1|Helicase[Tetrasphaeraelongata]_gi|478756579|emb|CCH71501.1|putativeHelicase[Tetrasphaeraelongat      ------------------------------------------------------------
gi|750080666|ref|WP_040385350.1|damageinducibleprotein[Demetriaterragena]/11625      ------------------------------------------------------------
gi|752558292|ref|WP_041229523.1|damageinducibleprotein[Dermacoccusnishinomiyaensis]_gi|664689315|gb|AIF41904.1|damageinducibl/1      ------------------------------------------------------------
gi|829089213|ref|WP_047310701.1|damageinducibleprotein[Dermacoccussp.PE3]_gi|828384812|gb|KLO63770.1|damageinducibleprotein[D/1      ------------------------------------------------------------
gi|748756635|ref|WP_040014750.1|damageinducibleprotein[Dermacoccussp.Ellin185]/11596      ------------------------------------------------------------
gi|916534724|ref|WP_051141848.1|damageinducibleprotein[Actinomycesneuii]/11632      -----------------------------------------------MFYL---------
gi|921140030|ref|WP_053090276.1|damageinducibleprotein[Rothiamucilaginosa]/11616      ------------------------------------------------------------
gi|896406751|ref|WP_049343749.1|damageinducibleprotein[Rothiamucilaginosa]/11647      ----------------------------------------------MT-AS---------
gi|723608234|gb|AIY01610.1|putativehelicase[Arthrobactersp.PAMC25486]/11613      ------------------------------------------------------------
gi|946394527|gb|KRE45160.1|damageinducibleprotein[Arthrobactersp.Soil736]/11616      ------------------------------------------------------------
gi|515765304|ref|WP_017197904.1|hypotheticalprotein[Arthrobactersp.M2012083]/11648      ----------------------------------------------MIRSV---------
gi|945315587|gb|KQT73127.1|damageinducibleprotein[Microbacteriumsp.Leaf436]/11612      ------------------------------------------------------------
gi|759908497|ref|WP_043594599.1|damageinducibleprotein[Frigoribacteriumsp.MEB024]_gi|758271359|gb|KIU03346.1|damageinduciblep/1      ------------------------------------------------------------
gi|945072850|gb|KQR34237.1|hypotheticalproteinASF75_00055[Curtobacteriumsp.Leaf154]/11597      ------------------------------------------------------------
gi|551263152|ref|WP_022883363.1|DNAdamageinducibleprotein[Glaciibactersuperstes]/11621      ------------------------------------------------------------
gi|945452341|gb|KQV08491.1|hypotheticalproteinASC63_10170[Leifsoniasp.Root112D2]/11623      ------------------------------------------------------------
gi|742759483|gb|KIC69912.1|damageinducibleprotein[Kocuriarhizophila]/11610      ------------------------------------------------------------
gi|737919995|ref|WP_035885304.1|damageinducibleprotein[Kocuriarhizophila]/11611      ------------------------------------------------------------
gi|737997701|ref|WP_035959325.1|damageinducibleprotein[Kocuriamarina]_gi|725823884|gb|KHE75603.1|damageinducibleprotein[Kocur/1      ------------------------------------------------------------
gi|664521070|ref|WP_031037650.1|damageinducibleprotein[Streptomycessp.NRRLWC3774]/11624      ------------------------------------------------------------
gi|500101034|ref|WP_011777041.1|DNAdamageinducibleprotein[Arthrobacteraurescens]_gi|119951547|gb|ABM10457.1|putativeHelicase[A/      ------------------------------------------------------------
gi|916871953|ref|WP_051479009.1|damageinducibleprotein[Arthrobactersp.H5]/11588      ------------------------------------------------------------
gi|612084649|gb|EZP43369.1|TypeIIIrestrictionenzyme,ressubunit[Micrococcusluteus]/11625      -------------------------------------------MVRMTIST---------
gi|759739708|ref|WP_043450422.1|damageinducibleprotein[Arthrobacterphenanthrenivorans]_gi|742758264|gb|KIC68716.1|damageinduc/1      ------------------------------------------------------------
gi|636845448|ref|WP_024366823.1|damageinducibleprotein[Arthrobactersp.TB26]/11605      ------------------------------------------------------------
gi|500009025|ref|WP_011689743.1|DNAdamageinducibleprotein[Arthrobactersp.FB24]_gi|116613051|gb|ABK05760.1|typeIIIrestrictionen/      ------------------------------------------------------------
gi|821249847|gb|KKZ12083.1|hypotheticalproteinTQ37_06090[CandidatusSynechococcusspongiarum15L]/11436      ------------------------------------------------------------
gi|739446902|ref|WP_037306885.1|damageinducibleprotein[Ruegeriahalocynthiae]/11592      ------------------------------------------------------------
gi|640620081|ref|WP_025048131.1|damageinducibleprotein[Sulfitobactermediterraneus]_gi|751956650|gb|KIN77213.1|TypeIIIrestricti/      ------------------------------------------------------------
gi|740247634|ref|WP_038088583.1|damageinducibleprotein[Thiobacillusprosperus]_gi|684049748|gb|KFZ90853.1|putativehelicase[Thio/      ------------------------------------------------------------
gi|518420321|ref|WP_019590528.1|hypotheticalprotein[Thioalkalivibriosp.ALE20]/11603      ------------------------------------------------------------
gi|500156826|ref|WP_011831496.1|DNAdamageinducibleprotein[Methylibiumpetroleiphilum]_gi|124261892|gb|ABM96881.1|adeninespecifi/      ------------------------------------------------------------
gi|769978537|ref|WP_045094522.1|damageinducibleprotein[Legionellafallonii]_gi|698841736|emb|CEG55682.1|Putativeproteinwithboxh/      ------------------------------------------------------------
gi|553752541|ref|WP_023085344.1|hypotheticalprotein[Pseudomonasaeruginosa]_gi|552201952|gb|ERU62653.1|hypotheticalproteinQ088_0      ------------------------------------------------------------
gi|880980418|ref|WP_048704461.1|damageinducibleprotein[Thauerahumireducens]_gi|875132785|gb|KMU70944.1|damageinducibleprotein/1      ------------------------------------------------------------
gi|740135732|ref|WP_037982815.1|damageinducibleprotein[Thauerasp.27]/11603      ------------------------------------------------------------
gi|738017371|ref|WP_035978332.1|damageinducibleprotein[Burkholderiaglumae]/11380      ------------------------------------------------------------
gi|498506003|ref|WP_010806671.1|typeIIIrestrictionenzyme,ressubunit[Pandoraeasp.SD62]_gi|490314561|gb|EON12879.1|typeIIIrestri/      ------------------------------------------------------------
gi|500117748|ref|WP_011793753.1|DNAdamageinducibleprotein[Acidovoraxcitrulli]_gi|120587742|gb|ABM31182.1|typeIIIrestrictionenz/      ------------------------------------------------------------
gi|651617328|ref|WP_026611444.1|damageinducibleprotein[Methylocaldumszegediense]/11601      ------------------------------------------------------------
gi|737210208|ref|WP_035194994.1|damageinducibleprotein[Acidithiobacillusferrivorans]_gi|669953220|emb|CDQ11755.1|Adeninespecif/      ------------------------------------------------------------
gi|935697386|ref|WP_054506180.1|damageinducibleprotein[Achromobactersp.]_gi|928716153|emb|CUK12836.1|Hefnuclease[Achromobacter/      ------------------------------------------------------------
gi|429502517|gb|ELA00827.1|adeninespecificDNAmethyltransferase[Cupriavidussp.HMR1]/11599      ------------------------------------------------------------
gi|506356642|ref|WP_015876361.1|DNAdamageinducibleprotein[Burkholderiaglumae]_gi|237877158|gb|ACR29491.1|AdeninespecificDNAmet/      ------------------------------------------------------------
gi|696406787|ref|WP_032978003.1|damageinducibleprotein[Bordetellahinzii]_gi|625335076|gb|KCB49343.1|typeIIIrestrictionenzyme,r/      ------------------------------------------------------------
gi|817529125|gb|KKO72587.1|damageinducibleprotein[Kerstersiagyiorum]/11592      ------------------------------------------------------------
gi|737048910|ref|WP_035044118.1|damageinducibleprotein[Aquabacteriumsp.NJ1]_gi|699052896|gb|KGM38706.1|damageinducibleprotein/1      ------------------------------------------------------------
gi|822492519|ref|WP_046933319.1|MULTISPECIES:damageinducibleprotein[Xanthomonas]_gi|821703525|gb|KLA61196.1|damageinduciblepr/1      ------------------------------------------------------------
gi|751252861|ref|WP_040963740.1|damageinducibleprotein[Xanthomonasfuscans]_gi|749808363|gb|KIJ03887.1|damageinducibleprotein[/1      ------------------------------------------------------------
gi|496774980|ref|WP_009368792.1|DNAdamageinducibleprotein[Bilophilasp.4_1_30]_gi|345039902|gb|EGW44205.1|hypotheticalproteinHM/      ------------------------------------------------------------
gi|499241912|ref|WP_010939452.1|DNAdamageinducibleprotein[Desulfovibriovulgaris]_gi|46580580|ref|YP_011388.1|adeninespecificDN/      ------------------------------------------------------------
gi|504274913|ref|WP_014462015.1|DNAdamageinducibleprotein[Bacillusmegaterium]_gi|345446919|gb|AEN91936.1|AdeninespecificDNAmet/      ------------------------------------------------------------
gi|504895966|ref|WP_015083068.1|typeIIrestrictionenzymeandmethyltransferasesubtypeG[Anabaenasp.90]_gi|413972424|gb|AFW96512.1|t      ------------------------------------------------------------
gi|518327765|ref|WP_019497972.1|hypotheticalprotein[Calothrixsp.PCC7103]/11600      ------------------------------------------------------------
gi|652765503|ref|WP_027079917.1|damageinducibleprotein[Luteimonasmephitis]/11593      ------------------------------------------------------------
gi|896539951|ref|WP_049442123.1|damageinducibleprotein[Stenotrophomonasmaltophilia]/11591      ------------------------------------------------------------
gi|500217900|ref|WP_011888043.1|DNAdamageinducibleprotein[Geobacillusthermodenitrificans]_gi|134267954|gb|ABO68149.1|Conserved/      ------------------------------------------------------------
gi|696570298|ref|WP_033102014.1|damageinducibleprotein[Thermoactinomycesdaqus]/11594      ------------------------------------------------------------
gi|251772067|gb|EES52637.1|putativehelicase[Leptospirillumferrodiazotrophum]/11618      ------------------------------------------------------------
gi|738124101|ref|WP_036082108.1|damageinducibleprotein[Leptospirillumferriphilum]_gi|685243306|gb|KGA94177.1|putativehelicase[/      ------------------------------------------------------------
gi|558699223|ref|WP_023524766.1|helicase[Leptospirillumsp.GroupII'CF1']_gi|206603764|gb|EDZ40244.1|Putativehelicase[Leptospiri/      ------------------------------------------------------------
gi|406774462|gb|AFS53887.1|putativehelicase[LeptospirillumferriphilumML04]/11610      ------------------------------------------------------------
```

```
gi|740719463|ref|WP_038504749.1|damageinducibleprotein[Leptospirillumferriphilum]_gi|640838125|gb|AIA30173.1|damageinduciblep/1      ------------------------------------------------------------
gi|517810766|ref|WP_018980974.1|hypotheticalprotein[Saccharibacterfloricola]/11733                                                    ------------------------------------------------------------
gi|502471675|ref|WP_012798533.1|helicase[Slackiaheliotrinireducens]_gi|256791761|gb|ACV22431.1|predictedhelicase[Slackiaheliotr      LTEDTLWDLKTSKDKPSPADTLQLAVYYLLSLR--SSKEEFKPIRKLGFYNPRLNKSWTI
gi|657871713|ref|WP_029576297.1|hypotheticalprotein[Bifidobacteriumthermacidophilum]_gi|673007521|gb|KFJ02535.1|helicase[Bifido      ------------------------------------------------------------
gi|673004757|gb|KFI99791.1|DNAhelicaserestrictionenzymetypeIIIRessubunit[Bifidobacteriumthermacidophilumsubsp.porcinum]/11682       ------------------------------------------------------------
gi|917316092|ref|WP_051922804.1|hypotheticalprotein[Bifidobacteriumstellenboschense]_gi|673003774|gb|KFI98812.1|helicase[Bifido      ------------------------------------------------------------
gi|705415694|ref|WP_033502332.1|hypotheticalprotein[Bifidobacteriumpseudolongum]_gi|672982411|gb|KFI77685.1|DNAdamageinducible/      ------------------------------------------------------------
gi|916591418|ref|WP_051198509.1|hypotheticalprotein[Bifidobacteriumsp.AGR2158]/11704                                                  ------------------------------------------------------------
gi|772242982|gb|AJW78255.1|hypotheticalproteinVO01_03165[Clavibactermichiganensissubsp.insidiosus]/11642                              ------------------------------------------------------------
gi|916649818|ref|WP_051256909.1|damageinducibleprotein[Actinomycesgraevenitzii]/11677                                                 ---------------------------------------------MATNF----------
gi|496504578|ref|WP_009212861.1|helicase[Actinomycessp.oraltaxon180]_gi|315312695|gb|EFU60776.1|helicase[Actinomycessp.oraltaxo      ------------------------------MTV--GRDDDFMALPGMEADST--------
gi|494862206|ref|WP_007588306.1|helicase[Actinomycessp.ICM39]_gi|398362799|gb|EJN46470.1|typeIIIrestrictionenzyme,ressubunit[Ac      ------------------------------MTA--ARDDDFMALPGIEADSK--------
gi|881057111|ref|WP_048774974.1|damageinducibleprotein[Sanguibacterkeddieii]/11697                                                    ------------------------------MTA--ARDDDFMALPGMEADSN--------
gi|881020459|ref|WP_048740808.1|damageinducibleprotein[Kytococcussedentarius]/11704                                                   ------------------------------MTA--ARDDDFMALPGMEADSN--------
gi|881052313|ref|WP_048770555.1|damageinducibleprotein[Cellulomonasflavigena]/11705                                                   ------------------------------MTA--ARTDDFAALPGMETANP--------
gi|490108682|ref|WP_004009424.1|helicase[Mobiluncuscurtisii]_gi|315490465|gb|EFU80089.1|helicaseCterminaldomainprotein[Mobilun/      ------------------------------MKV----AGKYKVMPDISSSNQFLT-----
gi|490111935|ref|WP_004012603.1|helicase[Mobiluncusmulieris]_gi|227843812|gb|EEJ53982.1|helicaseCterminaldomainprotein[Mobilun/      ------------------------------------------------------------
gi|490114228|ref|WP_004014848.1|helicase[Mobiluncusmulieris]_gi|269934432|gb|EEZ90993.1|helicaseCterminaldomainprotein[Mobilun/      ------------------------------------------------------------
gi|493333551|ref|WP_006290636.1|helicase[Parascardoviadenticolens]_gi|315119487|gb|EFT82620.1|helicaseCterminaldomainprotein[P/      ------------------------------------------------------------
gi|495817067|ref|WP_008541646.1|helicase,partial[Sutterellaparvirubra]_gi|378598583|gb|EHY31712.1|helicaseprotein,partial[Sutte      ------------------------------------------------------------
gi|491572696|ref|WP_005430278.1|helicase[Sutterellawadsworthensis]_gi|319805116|gb|EFW01943.1|DNAhelicaserestrictionenzymeTypeI      ------------------------------------------------------------
gi|748686321|ref|WP_039944445.1|hypotheticalprotein[Thermicanusaegyptius]/11573                                                       ------------------------------------------------------------
gi|916875698|ref|WP_051482754.1|hypotheticalprotein[Thioalkalivibriosp.HK1]/11366                                                     ------------------------------------------------------------
gi|821248604|gb|KKZ10960.1|hypotheticalproteinTE42_09075[CandidatusSynechococcusspongiarumSP3]/11598                                  ------------------------------------------------------------
gi|737409843|ref|WP_035390956.1|hypotheticalprotein[Ferrimicrobiumacidiphilum]_gi|765544266|gb|KJE76241.1|typeIrestrictionenzym      ------------------------------------------------------------
gi|654906925|ref|WP_028358281.1|damageinducibleprotein[Brackiellaoedipodis]/11679                                                     ------------------------------------------------------------
gi|502809901|ref|WP_013044877.1|hypotheticalprotein[CandidatusPuniceispirillummarinum]_gi|292663146|gb|ADE38247.1|hypotheticalp      ------------------------------------------------------------
gi|251771370|gb|EES51950.1|typeIIIrestrictionenzyme,ressubunit[Leptospirillumferrodiazotrophum]/11628                                 ------------------------------------------------------------
gi|498328480|ref|WP_010642636.1|DNAdamageinducibleprotein[Acidithiobacillusthiooxidans]/11627                                         ------------------------------------------------------------
gi|647633864|ref|WP_025906698.1|damageinducibleprotein[Prochlorococcussp.scB245a_519B7]/11622                                         ------------------------------------------------------------
gi|647619599|ref|WP_025893729.1|damageinducibleprotein[Prochlorococcussp.scB241_527N11]/11621                                         ------------------------------------------------------------
gi|755034887|ref|WP_042387498.1|hypotheticalprotein[Clostridiumsp.CL6]/11606                                                          ------------------------------------------------------------
gi|498139798|ref|WP_010453954.1|helicase[SuccinivibrionaceaebacteriumWG1]/11662                                                       ------------------------------------------------------------
gi|527501386|gb|EPT34571.1|typeIIIrestrictionenzyme,ressubunit[Bacteroidetesbacteriumoraltaxon272str.F0290]/11789                     ------------------------------------------------------------
gi|496047275|ref|WP_008771782.1|helicase[Bacteroidessp.2_1_33B]_gi|262296443|gb|EEY84373.1|helicaseCterminaldomainprotein[Bact/      ------------------------------------------------------------
PDI8503III/11664                                                                                                                      ------------------------------------------------------------
gi|738719431|ref|WP_036613715.1|helicase[Parabacteroidesdistasonis]_gi|649542518|gb|KDS49387.1|restrictionendonucleasefamilypro      ------------------------------------------------------------
gi|659417033|gb|KEJ86133.1|hypotheticalproteinHMPREF1002_00341[Porphyromonassp.31_2]/11656                                            ------------------------------------------------------------
gi|567456807|gb|ETK11358.1|hypotheticalproteinT235_15845[Tannerellasp.oraltaxonBU063isolateCell8/11]/11433                            ------------------------------------------------------------
gi|545440279|ref|WP_021677877.1|helicase[Porphyromonasgingivalis]_gi|543985765|gb|ERJ89108.1|helicaseprotein[Porphyromonasgingi      ------------------------------------------------------------
gi|497438056|ref|WP_009752254.1|helicase[Prevotellasp.oraltaxon473]_gi|429144866|gb|EKX87968.1|helicaseprotein[Prevotellasp.ora      ------------------------------------------------------------
gi|490423474|ref|WP_004295683.1|MULTISPECIES:helicase[Bacteroides]_gi|156110098|gb|EDO11843.1|helicaseCterminaldomainprotein[B/      ------------------------------------------------------------
gi|941777229|ref|WP_055173506.1|helicase[Bacteroidescaccae]_gi|933263395|emb|CUQ00567.1|Predictedhelicase[Bacteroidescaccae]/1/      ------------------------------------------------------------
gi|492413898|ref|WP_005836892.1|helicase[Bacteroidesuniformis]_gi|392678824|gb|EIY72229.1|hypotheticalproteinHMPREF1072_03583[B      ------------------------------------------------------------
gi|695455095|ref|WP_032566191.1|helicase[Bacteroidesfragilis]_gi|596095426|gb|EXZ59996.1|restrictionendonucleasefamilyprotein[B      ------------------------------------------------------------
gi|933227941|emb|CUQ02348.1|Predictedhelicase[Bacteroidesuniformis]/11658                                                            ------------------------------------------------------------
gi|494823013|ref|WP_007558421.1|helicase[Bacteroidesplebeius]_gi|198272167|gb|EDY96436.1|helicaseCterminaldomainprotein[Bacter/      ------------------------------------------------------------
gi|495427212|ref|WP_008151909.1|helicase[Parabacteroidesjohnsonii]_gi|218222381|gb|EEC95031.1|helicaseCterminaldomainprotein[P/      ------------------------------------------------------------
gi|933196855|emb|CUO94545.1|typeIrestrictionenzymeEcoKIsubunitR[Bacteroidesuniformis]_gi|933220098|emb|CUN86874.1|typeIrestrict      ------------------------------------------------------------
gi|823687748|ref|WP_047101121.1|helicase[Brachyspirahyodysenteriae]_gi|823334537|gb|KLI17932.1|helicase[Brachyspirahyodysenteri      ------------------------------------------------------------
gi|496436691|ref|WP_009145538.1|helicase[Phascolarctobacteriumsuccinatutens]_gi|322414007|gb|EFY04839.1|helicaseCterminaldomai/      ------------------------------------------------------------
gi|503372880|ref|WP_013607541.1|helicase[Sphaerochaetaglobosa]_gi|324026933|gb|ADY13692.1|typeIIIrestrictionproteinressubunit[S      ------------------------------------------------------------
gi|505821748|ref|WP_015707145.1|helicase[Treponemaprimitia]_gi|299483503|gb|ADJ19584.1|helicasedomaincontainingprotein[Trepone/      ------------------------------------------------------------
gi|505822741|ref|WP_015708134.1|helicase[Treponemaprimitia]_gi|333739639|gb|AEF85129.1|endonucleaseandmethylaseLlaGI[Treponemap      ------------------------------------------------------------
gi|488791994|ref|WP_002704400.1|helicase[Treponemasaccharophilum]_gi|380767691|gb|EIC01689.1|typeIIIrestrictionproteinressubuni      ------------------------------------------------------------
gi|524671454|emb|CDD81750.1|putativeseptumsitedeterminingproteinMinC[Bacteroidessp.CAG:462]/11642                                     ------------------------------------------------------------
gi|524573865|emb|CDC95766.1|putativeseptumsitedeterminingproteinMinC[Alistipessp.CAG:268]/11639                                       ------------------------------------------------------------
gi|658617970|ref|WP_029759328.1|helicase[Fusobacteriumnucleatum]/11606                                                                ------------------------------------------------------------
gi|494412037|ref|WP_007216782.1|helicase[Bacteroidescellulosilyticus]_gi|392638921|gb|EIY32752.1|hypotheticalproteinHMPREF1062_      ------------------------------------------------------------
gi|383107102|gb|AFG37435.1|putativehelicase[SpirochaetaafricanaDSM8902]/11616                                                         ------------------------------------------------------------
SAF8902III/11616                                                                                                                      ------------------------------------------------------------
gi|652538894|ref|WP_026932796.1|helicase[Gramellaechinicola]/11633                                                                    ------------------------------------------------------------
gi|639238223|ref|WP_024568839.1|helicase[Elizabethkingiaanophelis]/11628                                                              ------------------------------------------------------------
gi|652520381|ref|WP_026914582.1|helicase[Gramellaportivictoriae]/11623                                                                ------------------------------------------------------------
gi|494922183|ref|WP_007648219.1|Helicase[Formosasp.AK20]_gi|469825489|gb|EMQ95550.1|Helicasedomainprotein[Formosasp.AK20]/11626      ------------------------------------------------------------
gi|522083382|ref|WP_020594591.1|hypotheticalprotein[Spirosomapanaciterrae]/11637                                                      ------------------------------------------------------------
gi|490455352|ref|WP_004326157.1|helicase[Bacteroidesovatus]_gi|392669277|gb|EIY62768.1|hypotheticalproteinHMPREF1070_03635[Bact      ------------------------------------------------------------
```

```
gi|499516035|ref|WP_011202675.1|helicase[Bacteroidesfragilis]_gi|53713270|ref|YP_099262.1|helicasedomainprotein[Bacteroidesfrag      ------------------------------------------------------------
gi|651338332|ref|WP_026461320.1|helicase[Adhaeribacteraquaticus]/11620                                                                ------------------------------------------------------------
gi|806964642|gb|AKC17139.1|helicase[Flavobacteriumpsychrophilum]/11639                                                                ------------------------------------------------------------
gi|752684374|ref|WP_041332322.1|helicase[Flavobacteriumpsychrophilum]_gi|666882889|gb|AIG30715.1|helicase[Flavobacteriumpsychro      ------------------------------------------------------------
gi|933196595|emb|CUP04894.1|DNAorRNAhelicasesofsuperfamilyII[Bacteroidesuniformis]/11631                                              ------------------------------------------------------------
gi|670509109|ref|WP_031456918.1|helicase[Flavobacteriumchungangense]/11625                                                            ------------------------------------------------------------


gi|490190857|ref|WP_004089451.1|hypotheticalprotein[Helicobacterbilis]_gi|476630638|gb|EMZ37196.1|hypotheticalproteinC826_02193      ---------------------------------------MNQF---QVILEKI-------
gi|488896133|ref|WP_002807242.1|MULTISPECIES:restrictionendonucleaseEco57I[Campylobacter]_gi|380541096|gb|EIA65377.1|hypothetic      ----------------------------------MSA-NETTF---EIILEKL-------
gi|746602240|ref|WP_039628365.1|restrictionendonucleaseEco57I[Campylobacterlari]_gi|744819395|gb|AJD03391.1|putativehelicase[Ca      ----------------------------------MSA-NETTF---EIILEKL-------
gi|727317135|ref|WP_033772558.1|restrictionendonucleaseEco57I[Helicobacterpylori]/11622                                               ------------------------------------MSEINTY---KLIKEKL-------
gi|727103490|ref|WP_033618954.1|restrictionendonucleaseEco57I[Helicobacterpylori]/11616                                               ------------------------------------MSEINTY---KLIKEKL-------
gi|727340205|ref|WP_033794408.1|restrictionendonucleaseEco57I[Helicobacterpylori]/11609                                               ------------------------------------MSEINTY---KLIKEKL-------
gi|727292407|ref|WP_033749975.1|restrictionendonucleaseEco57I[Helicobacterpylori]/11611                                               ------------------------------------MSEINTY---KLIKEKL-------
gi|727289795|ref|WP_033747591.1|restrictionendonucleaseEco57I[Helicobacterpylori]/11612                                               ------------------------------------MQEISAY---ELIKQKL-------
gi|727319676|ref|WP_033775047.1|restrictionendonucleaseEco57I[Helicobacterpylori]/11612                                               ------------------------------------MQEISAY---ELIKQKL-------
gi|447212910|ref|WP_001290166.1|restrictionendonucleaseEco57I[Helicobacterpylori]_gi|308063540|gb|ADO05427.1|hypotheticalprotei      ------------------------------------MSEISVY---KLIKEKL-------
gi|447212901|ref|WP_001290157.1|restrictionendonucleaseEco57I[Helicobacterpylori]_gi|384557329|gb|AFH97797.1|hypotheticalprotei      ------------------------------------MSEISTY---KLIKEKL-------
gi|515849671|ref|WP_017280424.1|hypotheticalprotein[Helicobacterpylori]/11615                                                         ----------------------------------MKNVRNQHF---TLIKEKL-------
gi|447212900|ref|WP_001290156.1|restrictionendonucleaseEco57I[Helicobacterpylori]_gi|317178970|dbj|BAJ56758.1|TypeIIGrestrictio      ------------------------------------MSEISTY---KLIKEKL-------
gi|447079018|ref|WP_001156274.1|hypotheticalprotein[Helicobacterpylori]/11614                                                         ------------------------------------MQEISAY---KFIKEKL-------
gi|447212800|ref|WP_001290056.1|hypotheticalprotein[Helicobacterpylori]_gi|532153248|gb|EQL67316.1|restrictionendonucleaseEco57      ------------------------------------MSEISAY---TLIKEKL-------
gi|505457237|ref|WP_015644339.1|hypotheticalprotein[Helicobacterpylori]_gi|499061160|gb|AGL70074.1|restrictionendonucleaseEco57      ------------------------------------MQEISAY---KLIKEKL-------
HPYUM037X/11591                                                                                                                        ------------------------------------MQEISAY---KLIKEKL-------
gi|920026297|ref|WP_052937176.1|restrictionendonuclease[Helicobacterpylori]_gi|912528678|gb|KNX48238.1|restrictionendonucleaseE      ------------------------------------MSEISTY---KLIKEKL-------
gi|915539050|ref|WP_050841327.1|restrictionendonuclease[Helicobacterpylori]_gi|908890887|gb|KNE06183.1|restrictionendonucleaseE      ------------------------------------MQEISAY---KLIKQKL-------
gi|727101397|ref|WP_033616957.1|restrictionendonucleaseEco57I[Helicobacterpylori]/11592                                               ------------------------------------MQEISAY---KLIKEKL-------
gi|562814875|emb|CDK13002.1|TypeISPRMsystem[Helicobacterpylori26695]/11588                                                            ------------------------------------MQEISAY---TLIKEKL-------
HPYAXVIII/11588                                                                                                                        ------------------------------------MQEISAY---TLIKEKL-------
gi|447079029|ref|WP_001156285.1|restrictionendonucleaseEco57I[Helicobacterpylori]_gi|393085956|gb|EJB86635.1|hypotheticalprotei      ------------------------------------MQEISAY---TLIKEKL-------
gi|489105953|ref|WP_003015814.1|restrictionendonuclease[Helicobacterpylori]_gi|477503388|gb|ENH59950.1|restrictionendonucleasef      ------------------------------------MQEISAY---KLIKEKL-------
gi|447079006|ref|WP_001156262.1|restrictionendonucleaseEco57I[Helicobacterpylori]_gi|393067470|gb|EJB68280.1|hypotheticalprotei      ------------------------------------MQEISAY---ELIKEKL-------
gi|727314589|ref|WP_033770298.1|restrictionendonucleaseEco57I[Helicobacterpylori]/11586                                               ------------------------------------MSEISAY---TLIKEKL-------
gi|447079019|ref|WP_001156275.1|restrictionendonucleaseEco57I[Helicobacterpylori]_gi|308065028|gb|ADO06920.1|hypotheticalprotei      ------------------------------------MQEISAY---KLIKEKL-------
gi|447079028|ref|WP_001156284.1|restrictionendonucleaseEco57I[Helicobacterpylori]_gi|393076030|gb|EJB76784.1|hypotheticalprotei      ------------------------------------MQEISAY---TLIKEKL-------
gi|393034009|gb|EJB35070.1|hypotheticalproteinHPNQ4053_0631[HelicobacterpyloriNQ4053]/11514                                           ------------------------------------MQEISAY---ELIKQKL-------
gi|447079004|ref|WP_001156260.1|restrictionendonucleaseEco57I[Helicobacterpylori]_gi|384552418|gb|AFI07366.1|hypotheticalprotei      ------------------------------------MQEISAY---ELIKEKL-------
gi|746011306|ref|WP_039088215.1|restrictionendonucleaseEco57I[Helicobacterpylori]_gi|733488720|gb|KHL85979.1|restrictionendonuc      ------------------------------------MQEISAY---ELIKEKL-------
gi|531081162|gb|EQD92166.1|restrictionendonucleaseEco57I[HelicobacterpyloriPZ5080]/11509                                              ------------------------------------MQEISAY---ELIKEKL-------
gi|544760796|ref|WP_021187395.1|restrictionendonucleaseEco57I[Helicobacterpylori]_gi|531086508|gb|EQD96745.1|restrictionendonuc      ------------------------------------MQEISAY---ELIKEKL-------
gi|447079012|ref|WP_001156268.1|restrictionendonucleaseEco57I[Helicobacterpylori]_gi|393028199|gb|EJB29286.1|hypotheticalprotei      ------------------------------------MQEISAY---ELIKQKL-------
gi|746008458|ref|WP_039085555.1|restrictionendonucleaseEco57I[Helicobacterpylori]_gi|733480358|gb|KHL78026.1|restrictionendonuc      ------------------------------------MQEISAY---KLIKEKL-------
gi|447079011|ref|WP_001156267.1|restrictionendonucleaseEco57I[Helicobacterpylori]_gi|393038534|gb|EJB39568.1|hypotheticalprotei      ------------------------------------MQEISAY---ELIKEKL-------
gi|393037163|gb|EJB38199.1|hypotheticalproteinHPNQ4161_0422[HelicobacterpyloriNQ4161]/11556                                           ------------------------------------MQEISTY---ELIKEKL-------
gi|447079007|ref|WP_001156263.1|restrictionendonucleaseEco57I[Helicobacterpylori]_gi|393051817|gb|EJB52768.1|hypotheticalprotei      ------------------------------------MQEISAY---ELIKEKL-------
gi|487867094|ref|WP_001940560.1|helicase[Helicobacterpylori]_gi|459505657|gb|EMG98418.1|helicaseprotein[HelicobacterpyloriGAM21      ------------------------------------MQEISAY---ELIKEKL-------
gi|487872094|ref|WP_001945560.1|helicase[Helicobacterpylori]_gi|459512104|gb|EMH04523.1|helicaseprotein[HelicobacterpyloriGAM24      ------------------------------------MQEISSY---ELIKEKL-------
gi|487880228|ref|WP_001953694.1|helicase[Helicobacterpylori]_gi|459529131|gb|EMH20734.1|helicaseprotein[HelicobacterpyloriGAM26      ------------------------------------MQEISAY---ELIKEKL-------
gi|487882593|ref|WP_001956059.1|helicase[Helicobacterpylori]_gi|459533462|gb|EMH24859.1|helicaseprotein[HelicobacterpyloriGAM26      ------------------------------------MQEISTY---ELIKQKL-------
gi|487845430|ref|WP_001918896.1|helicase[Helicobacterpylori]_gi|410713871|gb|EKQ71363.1|helicaseprotein[HelicobacterpyloriGAM10      ------------------------------------MQEISAY---ELIKEKL-------
gi|447079005|ref|WP_001156261.1|restrictionendonucleaseEco57I[Helicobacterpylori]_gi|393069830|gb|EJB70625.1|hypotheticalprotei      ------------------------------------MQEISAY---ELIKEKL-------
gi|459506808|gb|EMG99528.1|helicaseprotein[HelicobacterpyloriGAM121Aii]/11545                                                         ------------------------------------MQEISAY---ELIKEKL-------
gi|487825283|ref|WP_001898749.1|restrictionendonucleaseEco57I[Helicobacterpylori]_gi|393107526|gb|EJC08068.1|hypotheticalprotei      ------------------------------------MQEISAY---ELIKEKL-------
gi|447079008|ref|WP_001156264.1|restrictionendonucleaseEco57I[Helicobacterpylori]_gi|393058636|gb|EJB59524.1|hypotheticalprotei      ------------------------------------MQEISAY---ELIKEKL-------
gi|447079015|ref|WP_001156271.1|restrictionendonucleaseEco57I[Helicobacterpylori]_gi|307637357|gb|ADN79807.1|hypotheticalprotei      ------------------------------------MQEISAY---ELIKQKL-------
gi|487884861|ref|WP_001958327.1|helicase[Helicobacterpylori]_gi|459535535|gb|EMH26834.1|helicaseprotein[HelicobacterpyloriGAM26      ------------------------------------MQEISAY---ELIKEKL-------
gi|487886365|ref|WP_001959831.1|helicase[Helicobacterpylori]_gi|459538727|gb|EMH29861.1|helicaseprotein[HelicobacterpyloriGAM27      ------------------------------------MQEISAY---ELIKEKL-------
gi|545301637|ref|WP_021581412.1|helicase[Helicobacterpylori]_gi|535931889|gb|ERA61745.1|helicaseprotein[HelicobacterpyloriGAM11      ------------------------------------MQEISAY---ELIKQKL-------
gi|447079010|ref|WP_001156266.1|restrictionendonucleaseEco57I[Helicobacterpylori]_gi|393095477|gb|EJB96081.1|hypotheticalprotei      ------------------------------------MQEISAY---ELIKEKL-------
gi|447079009|ref|WP_001156265.1|restrictionendonucleaseEco57I[Helicobacterpylori]_gi|393056294|gb|EJB57206.1|hypotheticalprotei      ------------------------------------MQEISAY---ELIKEKL-------
gi|471386985|gb|EMR58906.1|restrictionendonucleasefamilyprotein[HelicobacterpyloriHpH1]/11504                                         ------------------------------------MQEISAY---ELIKEKL-------
gi|487875679|ref|WP_001949145.1|helicase[Helicobacterpylori]_gi|459516928|gb|EMH09091.1|helicaseprotein[HelicobacterpyloriGAM25      ------------------------------------MQEISAY---ELIKQKL-------
gi|496130066|ref|WP_008854573.1|DNAdamageinducibleprotein[Commensalibacterintestini]_gi|353671689|gb|EHD13391.1|helicasedomain/      --------------------------------------MTSSL---QTLLSQF-RKQ---
gi|924898894|ref|WP_053556546.1|damageinducibleprotein[Sphingopyxissp.113P3]_gi|924523996|gb|ALC14732.1|damageinducibleprotei/1      --------------------------------------MPTPL---RALLDHY-RSS---
gi|780802950|gb|KJS08562.1|damageinducibleprotein[Hoefleasp.BRH_c9]/11633                                                             --------------------------------------MSSAL---RDLFDRY-RTH---
gi|944671432|gb|KQN22886.1|damageinducibleprotein[Sphingomonassp.Leaf32]/11415                                                        ------------------------------------------------MLERY-RTG---
```

```
gi|944612953|gb|KQM64772.1|damageinducibleprotein[Sphingomonassp.Leaf16]/11726    --------------------------------------MASAI---RNLLERY-RTG---
gi|764353851|ref|WP_044331468.1|damageinducibleprotein[Sphingomonashengshuiensis]_gi|761897338|gb|AJP71707.1|damageinduciblep/1    --------------------------------------MTTPL---RELLAEF-RSS---
gi|945874472|gb|KQZ27973.1|damageinducibleprotein[Mesorhizobiumsp.Root552]/11651    ----------------------MNEQPRSLDQQSGVEAKTNAI---RELLAQF-RAS---
gi|759408081|ref|WP_043132403.1|damageinducibleprotein[Paracoccussanguinis]_gi|694217086|gb|KGJ14281.1|damageinducibleprotein/1    ----------------------------------------MQL---RDLLDSY-RQA---
gi|654342671|ref|WP_027836116.1|damageinducibleprotein[Maritaleamyrionectae]/11632    ----------------------------------------MQL---SELLNRY-REE---
gi|126717687|gb|EBA14409.1|hypotheticalproteinRSK20926_02032[Roseobactersp.SK20926]/11603    ----------------------------------------MQL---QEILNTY-RQA---
gi|765301116|ref|WP_044628780.1|damageinducibleprotein[Halomonasmeridiana]_gi|764089679|gb|KJD19875.1|damageinducibleprotein[/1    ----------------------------------------MQL---HELLESY-RRT---
gi|518053006|ref|WP_019223214.1|DNAdamageinducibleprotein[Bartonellarattaustraliani]/11654    -----------------------------MLQSHKDDNKPISL---RALLQFC-REG---
gi|493637806|ref|WP_006589578.1|DNAdamageinducibleprotein[Bartonellabirtlesii]_gi|395410345|gb|EJF76900.1|hypotheticalproteinM/    -----------------------------MPHFHHYDNEHVTL---RSLLEYY-RQQ---
gi|501188603|ref|WP_012231622.1|DNAdamageinducibleprotein[Bartonellatribocorum]_gi|161017843|emb|CAK01401.1|helicase/methyltra/    -----------------------------MLQSLNSDHEHVTL---RSLLQSY-REQ---
gi|651388616|ref|WP_026500615.1|damageinducibleprotein[Bartonellagrahamii]/11655    -----------------------------MHHFNKYDHEHVSL---RSLLEYY-RQQ---
gi|501188669|ref|WP_012231687.1|DNAdamageinducibleprotein[Bartonellatribocorum]_gi|161017922|emb|CAK01480.1|helicase/methyltra/    -----------------------------MLQSLKSDHEHVTL---RSLLEYY-RQQ---
gi|518048832|ref|WP_019219040.1|DNAdamageinducibleprotein[Bartonellaflorencae]/11655    -----------------------------MSHLHKSDHERVTL---RSLLEYY-RQQ---
gi|518053617|ref|WP_019223825.1|DNAdamageinducibleprotein[Bartonellarattaustraliani]/11607    -----------------------------MLQSDKSDNKPISL---RSLLQFC-REG---
gi|506336449|ref|WP_015856168.1|DNAdamageinducibleprotein[Bartonellagrahamii]_gi|240267492|gb|ACS51080.1|helicase/methyltransf/    -----------------------------MLRPHHSDHEHVSL---RSLLEYY-RQQ---
gi|494588832|ref|WP_007347427.1|DNAdamageinducibleprotein[Bartonellarattimassiliensis]_gi|395419053|gb|EJF85361.1|hypothetical/    -----------------------------MLQSLKSDHEHVTL---RSLLEYY-RGQ---
gi|501188650|ref|WP_012231668.1|DNAdamageinducibleprotein[Bartonellatribocorum]_gi|161017902|emb|CAK01460.1|helicase/methyltra/    -----------------------------MLQSVKYDNEHVTL---RSLLEYY-RQQ---
gi|501188624|ref|WP_012231643.1|DNAdamageinducibleprotein[Bartonellatribocorum]_gi|161017866|emb|CAK01424.1|helicase/methyltra/    -----------------------------MSHPLYSDNKHVSL---RALLQSY-REQ---
gi|518053856|ref|WP_019224064.1|DNAdamageinducibleprotein[Bartonellarattaustraliani]/11625    -----------------------------MSHPLYSDNKHVTL---RSLLQSY-RDQ---
gi|501188053|ref|WP_012231081.1|DNAdamageinducibleprotein[Bartonellatribocorum]_gi|161017434|emb|CAK00992.1|helicase/methyltra/    -----------------------------MSHPLYSDNKHVSL---RALLQSY-REQ---
gi|651388630|ref|WP_026500626.1|damageinducibleprotein[Bartonellagrahamii]/11657    -----------------------------MLRPHHSDHEHVSL---RSLLEYY-RQQ---
gi|518048855|ref|WP_019219063.1|DNAdamageinducibleprotein[Bartonellaflorencae]/11658    -----------------------------MLQSVKSDNEHVTL---RSLLEYY-RQR---
gi|490995101|ref|WP_004856829.1|DNAdamageinducibleprotein[Bartonelladoshiae]_gi|395413167|gb|EJF79645.1|hypotheticalproteinMCS/    -----------------------------MSHLHKSDHEHVTL---RSLLEYY-RQQ---
gi|491002558|ref|WP_004864279.1|DNAdamageinducibleprotein[Bartonellavinsonii]_gi|395421392|gb|EJF87643.1|hypotheticalproteinME/    --------------------------------MLQSHTKPLTL---RSLLQSY-RDH---
gi|491003373|ref|WP_004865094.1|DNAdamageinducibleprotein[Bartonellavinsonii]_gi|395432864|gb|EJF98838.1|hypotheticalproteinME/    --------------------------------MLQSHTKPLTL---RSLLQSY-RDH---
gi|491002426|ref|WP_004864148.1|DNAdamageinducibleprotein[Bartonellavinsonii]_gi|395421767|gb|EJF88003.1|hypotheticalproteinME/    -----------------------------MLQSLKSDNKQSSL---RSLLQSY-RQQ---
gi|491004024|ref|WP_004865745.1|DNAdamageinducibleprotein[Bartonellavinsonii]_gi|395432542|gb|EJF98522.1|hypotheticalproteinME/    -----------------------------MLQSLKSDNKQSSL---RSLLQSY-RQQ---
gi|648396442|ref|WP_026088193.1|damageinducibleprotein[Bartonellarattaustraliani]/11593    -----------------------------MLQSHKSDNKPISL---RSLLQFC-REG---
gi|748190174|ref|WP_039761290.1|damageinducibleprotein[Bartonellaqueenslandensis]/11658    -----------------------------MLQSHKSDNKPISL---RSLLQFC-RDG---
gi|494817599|ref|WP_007553007.1|DNAdamageinducibleprotein[Bartonellasp.DB56]_gi|395411312|gb|EJF77837.1|hypotheticalproteinMC/1    --------------------------------MLQSHTKSLTL---RSLLQSY-RDH---
gi|515763376|ref|WP_017195976.1|DNAdamageinducibleprotein[Bartonellabirtlesii]/11656    --------------------------------MLQSHTKPLTL---RSLLQSY-RDH---
gi|494589420|ref|WP_007348014.1|DNAdamageinducibleprotein[Bartonellarattimassiliensis]_gi|395416278|gb|EJF82667.1|hypothetical/    -----------------------------MSYSLCSDNEHVSL---HSLLEYY-RQK---
gi|506336469|ref|WP_015856188.1|DNAdamageinducibleprotein[Bartonellagrahamii]_gi|240267512|gb|ACS51100.1|helicase/methyltransf/    -----------------------------MLRPHHSDNEHVTL---RSLLEYY-RQR---
gi|749308087|ref|WP_040132227.1|damageinducibleprotein[Bartonellatribocorum]_gi|605051878|emb|CDO49988.1|helicase/methyltransf/    -----------------------------MLCSHHSDNELVTL---RSLLEYY-RQQ---
gi|515763800|ref|WP_017196400.1|DNAdamageinducibleprotein[Bartonellabirtlesii]/11658    -----------------------------MPHFHHYDNEHVTL---RSLLEYY-RKQ---
gi|492186994|ref|WP_005773380.1|DNAdamageinducibleprotein[Bartonellaelizabethae]_gi|395418361|gb|EJF84688.1|hypotheticalprotei/    -----------------------------MLQSVKSDNEHVTL---RSLLEYY-RKQ---
gi|501188615|ref|WP_012231634.1|DNAdamageinducibleprotein[Bartonellatribocorum]_gi|161017856|emb|CAK01414.1|helicase/methyltra/    -----------------------------MLQSLKSDHEHVTL---RSLLEYY-RGQ---
gi|501187470|ref|WP_012230499.1|DNAdamageinducibleprotein[Bartonellatribocorum]_gi|161017090|emb|CAK00648.1|helicase/methyltra/    -----------------------------MLQSLKSDHEHVTL---RSLLEYY-RQQ---
gi|501188636|ref|WP_012231655.1|DNAdamageinducibleprotein[Bartonellatribocorum]_gi|161017879|emb|CAK01437.1|helicase/methyltra/    -----------------------------MLQSLKSDHEHVTL---RSLLEYY-RQQ---
gi|930151941|emb|CTQ34818.1|UvsWhelicase[Jannaschiarubra]/11641    ----------------------------------MPRGMSDDL---RDLLERF-RET---
gi|928977106|ref|WP_054001461.1|damageinducibleprotein[Shimiasp.SK013]_gi|927698852|gb|KPA22698.1|typeIrestrictionenzymeEcoKIs/    ---------------------------------------MSAL---DRILDGF-RNA---
gi|738872465|ref|WP_036760884.1|damageinducibleprotein[Leisingeradaeponensis]/11643    --------------------------------------MSHSL---DAILESF-RDS---
gi|518050881|ref|WP_019221089.1|DNAdamageinducibleprotein[Bartonellasenegalensis]/11652    ---------------------------------------MTTL---QSLLQIY-RDK---
gi|493980596|ref|WP_006923523.1|DNAdamageinducibleprotein[Bartonellawashoensis]_gi|395413322|gb|EJF79796.1|hypotheticalprotein/    ----------------------------------------MRL---QSLLQTY-RDE---
gi|493983106|ref|WP_006925984.1|DNAdamageinducibleprotein[Bartonellawashoensis]_gi|395418127|gb|EJF84455.1|hypotheticalprotein/    ----------------------------------------MRL---QSLLQTY-RDE---
gi|499494671|ref|WP_011181311.1|DNAdamageinducibleprotein[Bartonellahenselae]_gi|49239008|emb|CAF28308.1|hypotheticalproteinBH/    ----------------------------------------MTL---QSLLQIY-RDE---
gi|651388426|ref|WP_026500441.1|damageinducibleprotein[Bartonellagrahamii]/11652    ----------------------------------------MTL---QSLLQTY-RDE---
gi|506337280|ref|WP_015856999.1|DNAdamageinducibleprotein[Bartonellagrahamii]_gi|240268402|gb|ACS51990.1|helicase/methyltransf/    ----------------------------------------MTL---QSLLQTY-RDE---
gi|494817535|ref|WP_007552943.1|DNAdamageinducibleprotein[Bartonellasp.DB56]_gi|395412002|gb|EJF78518.1|hypotheticalproteinMC/1    ----------------------------------------MTL---QSLLQTY-RNE---
gi|493638099|ref|WP_006589864.1|DNAdamageinducibleprotein[Bartonellabirtlesii]_gi|395409809|gb|EJF76394.1|hypotheticalproteinM/    ----------------------------------------MTL---QSLLQTY-RNE---
gi|748189589|ref|WP_039760707.1|damageinducibleprotein[Bartonellaqueenslandensis]/11651    ----------------------------------------MTL---QSLLQIY-RDE---
gi|501189483|ref|WP_012232501.1|DNAdamageinducibleprotein[Bartonellatribocorum]_gi|161018902|emb|CAK02460.1|helicase/methyltra/    ---------------------------------------MTTL---QSLLQTY-RDE---
gi|518049181|ref|WP_019219389.1|DNAdamageinducibleprotein[Bartonellaflorencae]/11652    ---------------------------------------MTTL---QSLLQIY-RDE---
gi|492193255|ref|WP_005774528.1|DNAdamageinducibleprotein[Bartonellaelizabethae]_gi|395416403|gb|EJF82779.1|hypotheticalprotei/    ---------------------------------------MTTL---QSLLQTY-REE---
gi|498387325|ref|WP_010701288.1|helicase/methyltransferase[Bartonellabovis]_gi|478418724|gb|ENN92426.1|helicase/methyltransfera    -------------------------------------MAEKTL---QSLLQTY-RDE---
gi|319408697|emb|CBI82352.1|conservedhypotheticalprotein[BartonellaschoenbuchensisR1]/11661    -------------------------------------MAEKTL---QSLLQTY-REE---
gi|494741726|ref|WP_007477134.1|DNAdamageinducibleprotein[Bartonellamelophagi]_gi|395423925|gb|EJF90113.1|hypotheticalproteinM/    -------------------------------------MAEKTL---QSLLQTY-REE---
gi|737261405|ref|WP_035245349.1|damageinducibleprotein[Alcanivoraxjadensis]_gi|686989709|gb|KGD62556.1|hypotheticalproteinT9A_/    --------------------------------------MSSPL---YELLERY-RTH---
gi|916687564|ref|WP_051294655.1|damageinducibleprotein[Gemmobacternectariphilus]/11641    -----------------------------MEDESRADADVATL---HDVLASY-RSA---
gi|740294127|ref|WP_038132495.1|damageinducibleprotein[Thioclavasp.DT234]_gi|660524826|gb|KEO54689.1|hypotheticalproteinDT23_/1    ---------------------------------------MSAI---DDLLRSY-REA---
gi|568331062|ref|WP_024099615.1|helicase[Phaeobactergallaeciensis]_gi|568232121|gb|AHD12007.1|putativehelicase[Phaeobactergalla    -------------------------------------MTTNSL---QDLLNSY-REA---
gi|518810459|ref|WP_019966413.1|DNAdamageinducibleprotein[Pannonibacterphragmitetus]/11635    ---------------------------------------MTAI---DRILQSY-RDA---
gi|495305760|ref|WP_008030510.1|DNAdamageinducibleprotein[Rhodobactersp.SW2]_gi|259021698|gb|EEW25000.1|typeIIIrestrictionprot/    ---------------------------------------MTAI---DRILQSY-RDA---
gi|506403303|ref|WP_015923022.1|DNAdamageinducibleprotein[Rhodobactersphaeroides]_gi|221162951|gb|ACM03922.1|HypotheticalProte/    ---------------------------------------MTAL---DRLLQSY-RDA---
gi|738818386|ref|WP_036708790.1|damageinducibleprotein[Paracoccussanguinis]_gi|694226786|gb|KGJ22541.1|damageinducibleprotein/1    ---------------------------------------MTAL---DRLLQSY-RDA---
gi|746096057|ref|WP_039162453.1|damageinducibleprotein[Gallibacteriumanatis]_gi|703617775|gb|KGQ63868.1|damageinducibleprotei/1    -------------------------------------MHPSGL---SHLLKTY-QDI---
gi|737716785|ref|WP_035685631.1|damageinducibleprotein[Avibacteriumparagallinarum]/11643    ------------------------------------------M---IPSIKTY-QDI---
```

```
gi|540072553|gb|ERF78672.1|hypotheticalproteinN561_04930[Gallibacteriumanatis12656/12}/11651    -----------------------------------THPSISGL---SQLLKTY-QDI---
gi|917509519|ref|WP_052115936.1|damageinducibleprotein[Gallibacteriumanatis]_gi|702414161|gb|KGQ42233.1|damageinducibleprotei/1    -------------------------------------MHPSGL---SQLLKTY-QDI---
gi|746082942|ref|WP_039150268.1|damageinducibleprotein[Gallibacteriumanatis]_gi|703617078|gb|KGQ63194.1|damageinducibleprotei/1    -------------------------------------MHPSGL---SQLLKTY-QDI---
gi|822522885|ref|WP_046952840.1|damageinducibleprotein[Haemophilushaemolyticus]_gi|821501762|gb|KKZ59267.1|damageinduciblepro/1    ------------------------------------------------------------
gi|404381014|gb|EJZ77501.1|putativehelicase[Pasteurellamultocidasubsp.gallicidaP1059]/11681    AISNQQS---AIS--NQQSAISNQQS--AISNQQSAISNQQGL---SRLLIEY-ENY---
gi|298281660|gb|EFI23149.1|helicaseCterminaldomainprotein[Neisseriasp.oraltaxon014str.F0314]/11493    ---------------------------------------MSAL---QTLLASF-REQ---
gi|348013055|gb|EGY51980.1|helicasedomainprotein[Neisseriashayeganii871]/11481    ----------------------------------MPKSMMSAL---QTLLSQF-REL---
gi|496407140|ref|WP_009116004.1|DNAdamageinducibleprotein[Neisseriawadsworthii]_gi|349794756|gb|EGZ48566.1|helicasedomainprote/    ---------------------------------------MSAL---QTLLSKF-REL---
gi|517216441|ref|WP_018405259.1|DNAdamageinducibleprotein[Marinobacterlipolyticus]/11656    ---------------------------------------MTAL---QTILAQF-REM---
gi|738230264|ref|WP_036185691.1|damageinducibleprotein[Marinobactersp.ELB17]/11671    ---------------------------------------MTPL---QTILAQF-REM---
gi|447197741|ref|WP_001274997.1|DNAdamageinducibleprotein[Acinetobacterbaumannii]_gi|408512324|gb|EKK13969.1|typeIIIrestrictio/    ---------------------------------------MSAL---IDLLQTI-EKN---
gi|820775897|ref|WP_046739909.1|damageinducibleprotein[Acinetobactersp.AG1]_gi|819909758|gb|KKW75938.1|damageinducibleprotei/1    ---------------------------------------MSAL---TNLLQSI-EKI---
gi|584595163|ref|WP_024160846.1|helicase[Acinetobacterpittii]_gi|576702530|emb|CDI28049.1|helicasedomaincontainingprotein[Acin/    ---------------------------------------MSAL---TNLLQSI-EKI---
gi|514963042|ref|WP_016651366.1|hypotheticalprotein[Acinetobactersp.NIPH2036]_gi|514162665|gb|EPG41391.1|hypotheticalproteinF90    ---------------------------------------MSAL---TNLLQSI-EKI---
gi|515965564|ref|WP_017396147.1|DNAdamageinducibleprotein[Acinetobacterhaemolyticus]/11667    ---------------------------------------MSAL---TNLLQSI-EKI---
gi|491346810|ref|WP_005204747.1|MULTISPECIES:hypotheticalprotein[Acinetobacter]_gi|480335823|gb|ENX56033.1|hypotheticalproteinF    ---------------------------------------MSAL---TNLLQSI-EKI---
gi|693044472|ref|WP_032215268.1|damageinducibleprotein[Escherichiacoli]_gi|607725731|gb|EZE08230.1|damageinducibleprotein[Esc/1    ---------------------------------------MNAL---SKLLTQI-ELA---
gi|742382177|ref|WP_038861304.1|damageinducibleprotein[Cronobactermuytjensii]_gi|924333747|gb|ALB69766.1|damageinducibleprote/1    ---------------------------------------MNAL---SELLTKI-EHA---
gi|915912804|ref|WP_050927035.1|damageinducibleprotein[Yersiniaenterocolitica]_gi|899805802|emb|CQH32042.1|typeIrestrictionenz/    ---------------------------------------MNAL---SELLTKI-EHA---
gi|921111327|gb|KON62781.1|typeIrestrictionenzymeEcoKIsubunitR[Komagataeibactereuropaeus]/11492    ---------------------------------------MSAL---SQLLDTY-RNL---
gi|582024278|gb|AHI27615.1|DNAhelicaserestrictionenzymeTypeIIIRsubunit[KomagataeibacterxylinusE25]/11636    ---------------------------------------MSAL---SQLLDTY-RNL---
gi|347662673|dbj|BAK86128.1|DNAhelicaserestrictionenzymeTypeIIIRsubunit[KomagataeibactermedellinensisNBRC3288]/11737    ---------------------------------------MSAL---SQLLDTY-RNL---
gi|917445053|ref|WP_052051471.1|damageinducibleprotein[Acetobactertropicalis]/11429    ---------------------------------------MSAL---STLLDTL-RHM---
gi|453329445|dbj|GAC88472.1|DNAhelicaserestrictionenzymetypeIIIRsubunit[GluconobacterthailandicusNBRC3255]_gi|530287981|dbj|GAD    ----------------------------------MKVLPMSAL---STLLDTF-RRM---
gi|889770922|ref|WP_048844278.1|damageinducibleprotein[Gluconobacterfrateurii]/11419    ---------------------------------------MSAL---STLLDTF-RQM---
gi|504886208|ref|WP_015073310.1|hypotheticalprotein[Gluconobacteroxydans]_gi|411027954|gb|AFW01209.1|hypotheticalproteinB932_16    ---------------------------------------MSAL---STLLDTF-RQM---
gi|480040211|gb|ENV79751.1|hypotheticalproteinF942_01641[AcinetobacterursingiiANC3649]/11432    ---------------------------------------MSSF---FQLIDKF-RRH---
gi|822498222|ref|WP_046936504.1|damageinducibleprotein[Xanthomonasgardneri]_gi|821751224|gb|KLB07223.1|damageinducibleprotein/1    ---------------------------------------MTNL---SILLDTF-RAA---
gi|654059559|ref|WP_027705556.1|damageinducibleprotein[Zymobacterpalmae]/11635    ---------------------------------------MKSL---HDLLDWL-RSS---
gi|643535620|ref|WP_025224315.1|damageinducibleprotein[Methylophilaceaebacterium11]_gi|577704400|gb|EUJ10546.1|putativehelicas/    ---------------------------------------MSAL---NDLLNNY-RSS---
MBA11I/11635    ---------------------------------------MSAL---NDLLNNY-RSS---
gi|655355857|ref|WP_028762287.1|damageinducibleprotein[Shewanellacolwelliana]/11632    ---------------------------------------MSAL---KNLLEVY-RQE---
gi|753204236|ref|WP_041510579.1|damageinducibleprotein[Shewanellasp.cp20]_gi|752457765|gb|KIO36326.1|damageinducibleprotein[S/1    ---------------------------------------MTAL---EKLLAEY-RQV---
KPNNIH30III/11619    ---------------------------------------MNAL---KSILNSY-RQN---
gi|749295994|ref|WP_040120352.1|damageinducibleprotein[Klebsiellapneumoniae]_gi|723249300|gb|AIX81926.1|damageinducibleprotei/1    ---------------------------------------MNAL---KSILNSY-RQN---
gi|641639739|emb|CDQ54010.1|unnamedproteinproduct[Klebsiellapneumoniae]/11641    -----------------MSAVQQRAEYISYNEIAYKAPPMNAL---KSILNSY-RQN---
gi|490797922|ref|WP_004660060.1|hypotheticalprotein[Acinetobactersp.NIPH236]_gi|479876779|gb|ENU27973.1|hypotheticalproteinF992    ---------------------------------------MSVF---FDLINTY-RTA---
gi|916750926|ref|WP_051357982.1|damageinducibleprotein[Acinetobactertjernbergiae]/11453    ----------------------------MQL-DTLDFIKMSSF---FDLINKY-RRT---
gi|691098662|ref|WP_032025060.1|damageinducibleprotein[Acinetobacterbaumannii]_gi|593567521|gb|EXR50735.1|restrictionendonucle/    ---------------------------------------MSSF---FDLINTY-RTT---
gi|736669405|ref|WP_034676049.1|damageinducibleprotein[Acinetobacterbaumannii]/11643    ---------------------------------------MSSF---FDLINTY-RTT---
gi|491368928|ref|WP_005226826.1|hypotheticalprotein[Acinetobactersp.NIPH1847]_gi|480173259|gb|ENX09797.1|hypotheticalproteinF89    ---------------------------------------MSSF---FDLITTY-KTI---
gi|796853993|ref|WP_045795953.1|damageinducibleprotein[Acinetobacterindicus]_gi|787929761|gb|KJV44219.1|damageinducibleprotei/1    ---------------------------------------MASF---FDLINIY-RTT---
gi|295797463|gb|ADG32253.1|typeIIIrestrictionproteinressubunit[ThiomonasintermediaK12]/11676    -----------------MPCLE------SPLDALRPDAPMTPL---SSLLDTY-RAA---
gi|499871179|ref|WP_011551913.1|DNAdamageinducibleprotein[Myxococcusxanthus]_gi|108467544|gb|ABF92729.1|putativerestriction/mo/    ---------------------------------------MSAL---LDLLEHF-RTT---
gi|495313852|ref|WP_008038600.1|DNAdamageinducibleprotein[Bartonellatamiae]_gi|395424181|gb|EJF90368.1|hypotheticalproteinME5_/    ---------------------------------MSEKLEQTPL---EKVLTRF-RNA---
gi|501453335|ref|WP_012476784.1|DNAdamageinducibleprotein[Leptospirabiflexa]_gi|167777824|gb|ABZ96123.1|Helicase[Leptospirabif/    ------------------------------MKESLKKDIHSPL---LSILFQF-RNA---
gi|738104613|ref|WP_036063193.1|damageinducibleprotein[Leptospiranoguchii]/11652    ------------------------------MSELNYPKTITEL---SQVLQIL-GIN---
gi|410001039|gb|EKO51661.1|typeIIIrestrictionenzyme,ressubunit[Leptospirakirschneristr.200802841]/11651    ------------------------------MSEMNHPKIATEL---DHILQSL-GIH---
gi|490901851|ref|WP_004763774.1|typeIIIrestrictionenzyme,ressubunit[Leptospirakirschneri]_gi|464209249|gb|EMN24014.1|typeIIIres    ---------------------------------MNHPKIATEL---DHILQSL-GIH---
gi|464398131|gb|EMO65902.1|typeIIIrestrictionenzyme,ressubunit[Leptospirakirschneristr.200803703]/11648    ------------------------------MSEMNHPKIATEL---DHILQSL-GIH---
gi|945029024|gb|KQQ84900.1|damageinducibleprotein[Xanthomonassp.Leaf131]/11423    ---------------------------------------MSPL---NELLGRF-RTA---
gi|896511314|ref|WP_049418183.1|damageinducibleprotein[Stenotrophomonasmaltophilia]/11639    --------------------------------------MTTPL---QGLLESL-RQS---
gi|896513082|ref|WP_049419616.1|damageinducibleprotein[Stenotrophomonasmaltophilia]/11638    ---------------------------------------MTAL---ETLLDTL-RQS---
gi|896580616|ref|WP_049469425.1|damageinducibleprotein[Stenotrophomonasmaltophilia]/11641    ---------------------------------------MTVL---ETLLDTL-RQS---
gi|648289350|ref|WP_026070499.1|damageinducibleprotein[Stenotrophomonasmaltophilia]/11639    ---------------------------------------MTAL---ETLLDTL-RQS---
gi|656272786|ref|WP_029218442.1|damageinducibleprotein[Xanthomonascassavae]/11639    ---------------------------------------MTAL---ETLLDTL-RQS---
gi|406976236|gb|EKD98747.1|hypotheticalproteinACD_23C00290G0002[unculturedbacterium]/11679    -----------------MSYCPCAVLLKAQYRETCTDTPVTAL---AQLLNTF-RTT---
gi|828145737|ref|WP_047238120.1|damageinducibleprotein[Chromobacteriumsubtsugae]/11645    ---------------------------------------MSAL---QNLLASF-RNA---
gi|654311174|ref|WP_027824327.1|damageinducibleprotein[Laribacterhongkongensis]/11641    ---------------------------------MTNASQTTAL---QSLLSTF-RAA---
gi|757242173|gb|KIR15516.1|typeIrestrictionenzymeEcoKIsubunitR[Pseudomonasfluorescens]/11778    ---------------------------------------MSAL---SALLNTY-RTA---
gi|653605161|ref|WP_027616682.1|damageinducibleprotein[Pseudomonassp.URHB0015]/11639    ---------------------------------------MSAL---TALLNTY-RTA---
gi|520868025|ref|WP_020308060.1|helicase[Pseudomonassyringae]_gi|519159034|gb|EPM49935.1|helicasedomaincontainingprotein[Pseud/    ---------------------------------------MSAL---TALLNTY-RTA---
gi|940156567|gb|KPX71668.1|Helicasedomaincontainingprotein[Pseudomonasamygdalipv.lachrymans]/11677    GLPGFSF---GCC--QHCQLIDCRDLASVPYQLYSKGIHMSAL---TALLNTY-RTA---
gi|505460508|ref|WP_015647610.1|helicase[Pseudomonassp.GLE121]_gi|499110212|ref|YP_007974374.1|helicasedomaincontainingprotein/    ---------------------------------------MSAL---STLLNTY-RAA---
gi|940235551|gb|KPY45311.1|hypotheticalproteinALO48_200154[Pseudomonassyringaepv.rhaphiolepidis]/11631    ---------------------------------------MSAL---STLLNTY-RAA---
gi|520880808|ref|WP_020312235.1|helicase[Pseudomonassyringae]_gi|519172158|gb|EPM62027.1|helicasedomaincontainingprotein[Pseud/    ---------------------------------------MSAL---IALLNTY-RTA---
gi|829046479|ref|WP_047282259.1|damageinducibleprotein[Pseudomonasfluorescens]/11630    ---------------------------------------MSVL---ATLLESY-RTA---
```

gi|499415493|ref|WP_011102960.1|DNAdamageinducibleprotein[Pseudomonassyringaegroupgenomosp.3]_gi|28867279|ref|NP_789898.1|heli/	---------------------------------------MSAL---SSLLDTY-RSA---
gi|730238299|ref|WP_033942897.1|damageinducibleprotein[Pseudomonasaeruginosa]/11629	---------------------------------------MSAL---SALLDTY-RSA---
gi|495310125|ref|WP_008034873.1|DNAdamageinducibleprotein[Pseudomonassp.GM60]_gi|398186685|gb|EJM74052.1|putativehelicase[Pseu/	---------------------------------------MSAL---QSLLNTY-RST---
gi|779992653|ref|WP_045428690.1|damageinducibleprotein[Pseudomonassp.MT1]_gi|699967872|dbj|BAP80334.1|DNAhelicaserestrictione/1	---------------------------------------MSAL---SALLDAY-RCA---
gi|786037289|ref|WP_045733274.1|damageinducibleprotein[Pseudomonaspseudoalcaligenes]_gi|782987904|gb|KJU79152.1|damageinducib/1	---------------------------------------MSSL---SALLDTY-RSA---
gi|749666366|ref|WP_040265445.1|damageinducibleprotein[Pseudomonasrhodesiae]/11632	---------------------------------------MSAL---SALLNTY-RSA---
gi|734902433|ref|WP_034133698.1|damageinducibleprotein[Pseudomonasfluorescens]/11635	---------------------------------------MSAL---SALLNTY-RSA---
gi|490551031|ref|WP_004416146.1|typeIIrestrictionmodificationsystemrestriction/helicasesubunit[Pseudomonassyringae]_gi|4432823/	---------------------------------------MSAL---SALLNAY-RSA---
gi|489199911|ref|WP_003109152.1|DNAdamageinducibleprotein[Pseudomonasaeruginosa]_gi|552648708|gb|ERY63184.1|hypotheticalprotei/	---------------------------------------MSAL---SALLHTF-RSA---
gi|730641237|ref|WP_034061328.1|damageinducibleprotein[Pseudomonasaeruginosa]/11636	---------------------------------------MSAL---SALLNTY-RSA---
gi|751304980|ref|WP_041013386.1|damageinducibleprotein[Pseudomonasxanthomarina]_gi|703407611|emb|CEG51014.1|Helicasedomaincon/1	---------------------------------------MSAL---STLLDTY-RDA---
gi|939335207|ref|WP_054881520.1|damageinducibleprotein[Pseudomonassp.NBRC111128]/11632	---------------------------------------MSAL---SALLNTY-RSA---
gi|757693555|ref|WP_042923792.1|damageinducibleprotein[Pseudomonaschloritidismutans]_gi|780910610|gb|KJS78302.1|damageinducib/1	---------------------------------------MSAL---SALLETY-RSA---
gi|665953944|ref|WP_031319989.1|damageinducibleprotein[Pseudomonastolaasii]/11626	---------------------------------------MSAL---TALLEAY-RAV---
gi|771600573|ref|WP_045213746.1|damageinducibleprotein[Pseudomonassp.21]_gi|770081123|gb|KJJ98634.1|damageinducibleprotein[Ps/1	---------------------------------------MSAL---SSLLDIY-RAA---
gi|740871396|ref|WP_038656644.1|damageinducibleprotein[Pseudomonasstutzeri]_gi|627772214|gb|AHY41437.1|damageinducibleprotein/1	---------------------------------------MSAL---SALLDAY-RSA---
gi|946242205|gb|KRC93782.1|damageinducibleprotein[Pseudomonassp.Root9]/11634	---------------------------------------MSAL---SALLTTY-RSA---
gi|553777322|ref|WP_023109107.1|MULTISPECIES:hypotheticalprotein[Pseudomonas]_gi|552638674|gb|ERY53184.1|hypotheticalproteinQ05	---------------------------------------MSAL---SALLNTY-RSA---
gi|896383370|ref|WP_049324618.1|damageinducibleprotein[Pseudomonasstutzeri]/11627	---------------------------------------MSAL---SALLDAY-RSA---
gi|696158510|gb|AIT41787.1|putativehelicase[Pseudomonassp.LM8]/11630	---------------------------------------MSAL---SALLNTY-RST---
gi|504634892|ref|WP_014821994.1|DNAdamageinducibleprotein[Pseudomonasstutzeri]_gi|390985249|gb|AFM35242.1|helicasedomainconta/1	---------------------------------------MSAL---SALLDTY-RST---
gi|544783974|ref|WP_021206729.1|DNAdamageinducibleprotein[Pseudomonasstutzeri]_gi|533224779|gb|EQM79688.1|DNAdamageinduciblep/1	---------------------------------------MSAL---SALLDAY-RSA---
gi|504567385|ref|WP_014754487.1|MULTISPECIES:DNAdamageinducibleprotein[Pseudomonas]_gi|388559493|gb|AFK68634.1|helicasedomain/1	---------------------------------------MSAL---SALLTTY-RSA---
gi|654441469|ref|WP_027912052.1|damageinducibleprotein[Pseudomonassp.URIL14HWK12:I7]/11630	---------------------------------------MSAL---SALLNTY-RSA---
gi|941470066|ref|WP_055127894.1|damageinducibleprotein[Pseudomonasmediterranea]/11631	---------------------------------------MSAL---SALLNTY-RSA---
gi|557815599|ref|WP_023446184.1|DNAdamageinducibleprotein[Pseudomonaschloritidismutans]_gi|557364081|gb|ESQ98190.1|DNAdamagei/1	---------------------------------------MSAL---SALLNTY-RSA---
gi|727814791|ref|WP_033898247.1|damageinducibleprotein[Pseudomonassp.FH1]_gi|567648005|gb|ETK23229.1|helicasedomaincontaining/1	---------------------------------------MSAL---SALLNTY-RSA---
gi|503278702|ref|WP_013513363.1|DNAdamageinducibleprotein[Desulfovibrioaespoeensis]_gi|316942375|gb|ADU61426.1|typeIIrestrict/	---------------------------------------MTAL---QNILDAY-RNA---
gi|505228796|ref|WP_015415898.1|typeIIrestrictionendonucleasesubunitR[Desulfovibriopiezophilus]_gi|451896976|emb|CCH49855.1|Ty	---------------------------------------MTAL---QKILETY-REA---
gi|517732543|ref|WP_018902751.1|hypotheticalprotein[Variovoraxparadoxus]/11675	---------------------------------------MTAL---SSLLNVY-RTA---
gi|550909475|ref|WP_022662570.1|DNAdamageinducibleprotein[Desulfovibriolongus]/11637	---------------------------------------MTAL---QKLLNAY-RRA---
gi|945494542|gb|KQV50024.1|damageinducibleprotein[Massiliasp.Root335]/11647	---------------------------------------MTAL---SSLLDHY-RQS---
gi|334196917|gb|AEG70102.1|helicasedomainprotein[RalstoniasolanacearumPo82]/11663	---------------------------------------MTAL---EQLLDTF-RTA---
gi|656006746|ref|WP_029048170.1|damageinducibleprotein[Cupriavidussp.amp6]/11661	---------------------------------------MTAL---EQLLDTF-RTA---
BPS2196ORF3054P/11659	---------------------------------------MTAL---EMLLDTF-RHA---
gi|740932312|ref|WP_038717332.1|damageinducibleprotein[Burkholderiapseudomallei]_gi|760211882|gb|KIX58314.1|damageinduciblepr/1	---------------------------------------MTAL---EMLLDTF-RHA---
gi|755810813|ref|WP_042583861.1|damageinducibleprotein[Burkholderiasp.MSHR3999]_gi|755174952|gb|KIP19790.1|DEAD/DEAHboxhelicas/	---------------------------------------MTAL---ELLLDTF-RHT---
gi|484586570|gb|AGK48195.1|DEAD/DEAHboxhelicasefamilyprotein[BurkholderiathailandensisMSMB121]/11646	---------------------------------------MTAL---EMLLDTF-RHT---
gi|737582935|ref|WP_035553941.1|damageinducibleprotein[Burkholderiasp.9120]/11658	---------------------------------------MTAL---ETLLDTF-RHA---
gi|740989872|ref|WP_038773225.1|damageinducibleprotein[Burkholderiapseudomallei]_gi|705645291|gb|KGS70691.1|DEAD/DEAHboxhelica/	---------------------------------------MTAL---EMLLDTF-RHA---
gi|654912525|ref|WP_028363424.1|damageinducibleprotein[Burkholderiasp.JPY366]/11659	---------------------------------------MTAL---EQLLDTF-RHA---
gi|740955879|ref|WP_038740512.1|damageinducibleprotein[Burkholderiapseudomallei]_gi|714684932|gb|KGW74438.1|DEAD/DEAHboxhelica/	---------------------------------------MTAL---EMLLDTF-RHA---
gi|919203714|ref|WP_052759215.1|damageinducibleprotein[Xanthomonasaxonopodis]/11388	-------------------------------------MAETTI---HHILDEL-AEA---
gi|522152381|ref|WP_020663589.1|hypotheticalprotein[Amycolatopsisbenzoatilytica]/11605	--------------------------------------MATTV---HDILEAF-RAL---
gi|663196013|ref|WP_030233899.1|damageinducibleprotein[Streptomycessp.NRRLS455]/11641	--------------------------------------MTTTV---HDVLRVI-REK---
gi|750429553|ref|WP_040711444.1|damageinducibleprotein[Nocardiopsispotens]/11622	-----------------------------------MTETTTTV---HDVLKAI-RED---
gi|518656139|ref|WP_019819475.1|hypotheticalprotein[Saccharomonosporasaliphila]/11623	-------------------------------------MTETTTV---HDVLQAI-RES---
gi|759722933|ref|WP_043433758.1|damageinducibleprotein[Streptomycespluripotens]_gi|733404978|gb|KHK94361.1|damageinduciblepro/1	-----------------------------------MAATTTTV---HDVLRAI-RHG---
gi|663424167|ref|WP_030419030.1|damageinducibleprotein[Streptomycessp.NRRLF5065]/11627	-----------------------------------MTATTTTV---HDVLKAI-RHG---
gi|663412917|ref|WP_030408353.1|damageinducibleprotein[Streptomycessp.NRRLF5917]/11630	-----------------------------------MATATTTV---HDVLRAI-RNG---
gi|671521483|ref|WP_031506489.1|damageinducibleprotein[Streptomycesmegasporus]/11630	-----------------------------------MATATTTV---HDVLRAI-RNG---
gi|740053144|ref|WP_037902776.1|damageinducibleprotein[Streptomycessp.TAA204]/11626	-------------------------------------MSTTTSV---HDVIRAI-RNG---
gi|663409375|ref|WP_030404974.1|MULTISPECIES:damageinducibleprotein[Streptomyces]/11627	-------------------------------------MATTTSV---HDVIRAI-RHG---
gi|746628608|ref|WP_039654277.1|damageinducibleprotein[Streptomycessp.MUSC125]_gi|745644381|gb|KIE25557.1|damageinducibleprot/1	-------------------------------------MATTTSV---HDVIRAI-RHG---
gi|727322066|ref|WP_033777320.1|damageinducibleprotein[Salinisporapacifica]/11627	-------------------------------------MTTTTTV---HDVIKAI-RDR---
gi|653653242|ref|WP_027655726.1|damageinducibleprotein[Salinisporapacifica]/11618	--------------------------------------MTTV---HDVIRAI-RDS---
gi|648632543|ref|WP_026324294.1|damageinducibleprotein[Salinisporapacifica]/11621	--------------------------------------MTTV---HDVMKAI-RDN---
gi|881018002|ref|WP_048738463.1|damageinducibleprotein[Corynebacteriumfalsenii]/11676	---------------------------------------MASF---VEALEQL-RRN---
gi|505465526|ref|WP_015652417.1|helicase[Corynebacteriumcallunae]_gi|459385704|gb|AGG67995.1|helicase[CorynebacteriumcallunaeDS	---------------------------------------MSTI---TDAVSAL-RTL---
gi|41223062|emb|CAF19007.1|DNAorRNAhelicaseofsuperfamilyII[CorynebacteriumglutamicumATCC13032]/11646	------------------------------------MECMSTI---TDAVSAL-RTL---
CGL13032I/11643	---------------------------------------MSTI---TDAVSAL-RTL---
gi|751257081|ref|WP_040967675.1|damageinducibleprotein[Corynebacteriumglutamicum]_gi|748811620|gb|AJE67931.1|damageinduciblep/1	---------------------------------------MSTI---NDAVAAL-REL---
gi|749040539|ref|WP_040086524.1|damageinducibleprotein[Corynebacteriumhumireducens]/11663	---------------------------------------MSSI---MDALSAL-REN---
gi|491662899|ref|WP_005519615.1|helicase[Corynebacteriummatruchotii]_gi|224946756|gb|EEG27965.1|helicaseCterminaldomainprotein/	---------------------------------------MASL---TDALDEL-RSL---
gi|746717312|ref|WP_039676231.1|damageinducibleprotein[Corynebacteriumminutissimum]_gi|734991118|gb|KHO29219.1|damageinducibl/1	---------------------------------------MSAF---GEVLSQL-REN---
gi|908654892|emb|CQD15529.1|SuperfamilyIIDNA/RNAhelicase(modularprotein)[Corynebacteriumstriatum]/11674	------------------------------------MDTMSAF---GEVLTQL-REN---
gi|895994446|ref|WP_049064418.1|damageinducibleprotein[Corynebacteriumaurimucosum]/11667	---------------------------------------MSAF---GEVLTQL-REN---

```
gi|896123331|ref|WP_049145390.1|damageinducibleprotein[Corynebacteriumaurimucosum]/11669    ---------------------------------------MSAF---GEVLTQL-REN---
gi|817681178|ref|WP_046645422.1|damageinducibleprotein[Corynebacteriumstriatum]_gi|817551679|gb|KKO79868.1|damageinduciblepro/1    ---------------------------------------MSAF---GEVLTQL-REN---
gi|896139239|ref|WP_049159146.1|damageinducibleprotein[Corynebacteriumaurimucosum]/11675    ---------------------------------------MSAF---GEVLTQL-REN---
gi|489943947|ref|WP_003847254.1|helicase[Corynebacteriumammoniagenes]_gi|295968218|gb|EFG81467.1|helicaseCterminaldomainprotei/    ---------------------------------------MTAF---GEVLEQL-REN---
gi|516653464|ref|WP_018022453.1|helicase[Corynebacteriumdoosanense]_gi|697778282|gb|AIT61018.1|damageinducibleprotein[Coryneba/    ---------------------------------------MSAF---EEVLHQL-REN---
gi|493894179|ref|WP_006840129.1|helicase[Corynebacteriumlipophiloflavum]_gi|227078819|gb|EEI16782.1|helicaseCterminaldomainpro/    ---------------------------------------MSSF---AQVMDQL-REN---
gi|740831472|ref|WP_038616755.1|damageinducibleprotein[Corynebacteriumpseudotuberculosis]_gi|666103058|gb|AIG06935.1|putativeh/    ---------------------------------------MASF---EQVLDQL-RDN---
gi|518023322|ref|WP_019193530.1|helicase[Corynebacteriumtimonense]/11655    ---------------------------------------MSYF---AEVLEQL-REN---
gi|552767697|ref|WP_023019559.1|MULTISPECIES:hypotheticalprotein[Corynebacterium]_gi|550739013|gb|ERS38609.1|hypotheticalprotei    ---------------------------------------MATF---EEVLDQL-REN---
gi|652659121|ref|WP_027017812.1|damageinducibleprotein[Corynebacteriumpseudodiphtheriticum]/11644    ---------------------------------------MATF---EEVLDQL-REN---
gi|749039309|ref|WP_040085390.1|damageinducibleprotein[Corynebacteriumhumireducens]/11657    ---------------------------------------MSSF---ADAIAQL-RNN---
gi|740835591|ref|WP_038620874.1|damageinducibleprotein[Corynebacteriumulcerans]_gi|698973854|gb|AIU33664.1|HelicaseCterminald/1    ---------------------------------------MATF---DEVLDQL-RDN---
gi|805307599|ref|WP_046096452.1|damageinducibleprotein[Corynebacteriumulcerans]_gi|803357323|gb|AKA97638.1|Damageinduciblepro/1    ---------------------------------------MATF---DEVLDQL-RDN---
gi|504083555|ref|WP_014317549.1|helicase[Corynebacteriumdiphtheriae]_gi|371584249|gb|AEX47914.1|superfamilyIIDNA/RNAhelicase[Co    ---------------------------------------MSKF---SEVLDQL-REN---
gi|896182193|ref|WP_049193580.1|damageinducibleprotein[Corynebacteriumjeikeium]/11645    ---------------------------------------MSKF---SEVLDQL-REN---
gi|917636459|ref|WP_052205460.1|damageinducibleprotein[Corynebacteriumriegelii]_gi|914295811|gb|AKV59187.1|damageinduciblepro/1    ---------------------------------------MSSF---GEVLDQL-RDN---
gi|737013522|ref|WP_035009205.1|damageinducibleprotein[Corynebacteriumjeikeium]/11651    ---------------------------------------MSTF---SEVLEQL-REN---
gi|737006291|ref|WP_035002094.1|damageinducibleprotein[Corynebacteriumjeikeium]/11658    ---------------------------------------MAKF---SEVLTQL-REN---
gi|737010378|ref|WP_035006133.1|damageinducibleprotein[Corynebacteriumjeikeium]/11650    ---------------------------------------MAKF---SEVLTQL-RDN---
gi|737010473|ref|WP_035006228.1|damageinducibleprotein[Corynebacteriumjeikeium]/11657    ---------------------------------------MSKF---SDVLEQL-RDN---
gi|737006250|ref|WP_035002053.1|damageinducibleprotein[Corynebacteriumjeikeium]/11658    ---------------------------------------MSKF---SDVLEQL-RDN---
gi|923018168|ref|WP_053412254.1|damageinducibleprotein[Corynebacteriumlactis]_gi|922845184|gb|ALA67504.1|helicase[Corynebacter/    ---------------------------------------MSKF---SEVLEQL-REN---
gi|923018885|ref|WP_053412970.1|damageinducibleprotein[Corynebacteriumlactis]_gi|922845845|gb|ALA68165.1|helicase[Corynebacter/    ---------------------------------------MSKF---SEVLEQL-REN---
gi|512464870|ref|WP_016422869.1|hypotheticalprotein[Corynebacteriumsp.HFH0082]_gi|512047830|gb|EPD47505.1|hypotheticalproteinHM    ---------------------------------------MSKF---SEVLEQL-REN---
gi|881012697|ref|WP_048733406.1|damageinducibleprotein[Corynebacteriumurealyticum]/11670    ---------------------------------------MSKF---SEVLEQL-REN---
gi|755608478|ref|WP_042530519.1|damageinducibleprotein[Corynebacteriumsingulare]_gi|754340239|gb|AJI78730.1|putativehelicase[C/    ---------------------------------------MSKF---SEVLEQL-REN---
gi|491421796|ref|WP_005279593.1|helicase[Corynebacteriumaccolens]_gi|227077180|gb|EEI15143.1|helicaseCterminaldomainprotein[Co/    ---------------------------------------MSKF---SEVLEQL-REN---
gi|550752286|gb|ERS51451.1|hypotheticalproteinHMPREF1282_00033[Corynebacteriumsp.KPL1856]_gi|550759961|gb|ERS58964.1|hypothetic    ----------------------------MDGWQVRHNAVMSKF---SEVLDQL-REN---
gi|497955053|ref|WP_010269209.1|helicase[Corynebacteriumbovis]/11599    ---------------------------------------MSTV---DDVYAAL-RDS---
gi|860580040|ref|WP_048470569.1|hypotheticalprotein[Mycobacteriumchlorophenolicum]_gi|859098575|gb|KMO76871.1|typeIrestrictione    ---------------------------------------MGSI---NDVIEAF-RQA---
gi|262085905|gb|ACY21873.1|typeIIIrestrictionproteinressubunit[GordoniabronchialisDSM43247]/11632    -------------------------------------MSMASV---HDVIEAF-RKE---
gi|495996338|ref|WP_008720917.1|typeIIIrestrictionendonucleasesubunitR[Rhodococcussp.AW25M09]_gi|443412350|emb|CCQ18455.1|TypeI    ---------------------------------------MASV---HEVIEAF-RGE---
gi|739284636|ref|WP_037147410.1|damageinducibleprotein[Rhodococcusfascians]/11641    ---------------------------------------MASV---HEVIEAF-RGE---
gi|739328195|ref|WP_037190574.1|damageinducibleprotein[Rhodococcusfascians]/11643    ---------------------------------------MASV---HEVIEAF-RGE---
gi|811698335|emb|CMN21041.1|helicase[Mycobacteriumtuberculosis]/11725    ---------------------------------------MGSV---HDVIEAF-RKA---
gi|801577898|emb|CFK23773.1|helicase[Mycobacteriumtuberculosis]/11675    ---------------------------------------MGSV---HDVIEAF-RKA---
MTU18II/11606    ---------------------------------------MGSV---HDVIEAF-RKA---
gi|887719858|emb|CMT27886.1|helicase[Mycobacteriumtuberculosis]/11667    ---------------------------------------MGSV---HDVIEAF-RKA---
gi|621694743|gb|KAS87156.1|hypotheticalproteinAL70_01801[MycobacteriumtuberculosisTKK_03_0115]/11604    ---------------------------------------MGSV---HDVIEAF-RKA---
gi|888128072|emb|CLQ84422.1|helicase[Mycobacteriumtuberculosis]_gi|889917090|emb|CLY86893.1|helicase[Mycobacteriumtuberculosis]    ---------------------------------------MGSV---HDVIEAF-RKA---
gi|884823424|emb|CKL96672.1|helicase[Mycobacteriumtuberculosis]/11466    ---------------------------------------MGSV---HDVIEAF-RKA---
gi|505103239|ref|WP_015290341.1|hypotheticalprotein[Mycobacteriumcanettii]_gi|432162733|emb|CCK60117.1|Conservedproteinofunknow    ---------------------------------------MGSV---HDVIEAF-RRA---
gi|505116250|ref|WP_015303352.1|hypotheticalprotein[Mycobacteriumcanettii]_gi|432158726|emb|CCK56026.1|Conservedproteinofunknow    ---------------------------------------MGSV---HDVIEAF-RKA---
gi|432154735|emb|CCK51974.1|Conservedproteinofunknownfunction(part1)[MycobacteriumcanettiiCIPT140060008]/11588    ---------------------------------------MGSV---HDVIEAF-RKA---
gi|809008027|ref|WP_046295375.1|hypotheticalprotein[Mycobacteriumsp.UM_Kg17]/11624    ---------------------------------------MGSI---HEVIEAF-RKA---
gi|808802633|ref|WP_046284139.1|hypotheticalprotein[Mycobacteriumsp.UM_NZ2]/11624    ---------------------------------------MGSI---HEVIEAF-RKA---
gi|750125774|ref|WP_040429852.1|damageinducibleprotein[Corynebacteriumnuruki]/11668    ---------------------------------------MASV---HDVIDAF-RTS---
gi|602237614|gb|EYT60960.1|damageinducibleprotein[Dietziasp.UCDTHP]/11473    ---------------------------------------MSSF---AQVIDAF-RQA---
gi|665897412|ref|WP_031264618.1|damageinducibleprotein[Dietziasp.UCDTHP]_gi|602237599|gb|EYT60945.1|helicase[Dietziasp.UCDTH/11    ---------------------------------------MSSF---AQVIDAF-RQA---
gi|502903674|ref|WP_013138650.1|helicase[Segniliparusrotundus]_gi|296181291|gb|ADG98197.1|typeIIIrestrictionproteinressubunit[S    ---------------------------------------MGSI---HEVIEAF-RQA---
gi|780248306|ref|WP_045538930.1|hypotheticalprotein[Gordoniasp.no.9]_gi|778142514|gb|KJR06917.1|hypotheticalproteinUG54_12320[G    ---------------------------------------MGSI---HEVIEAF-RQA---
gi|665897368|ref|WP_031264574.1|damageinducibleprotein[Dietziasp.UCDTHP]_gi|602239137|gb|EYT62483.1|helicase[Dietziasp.UCDTH/11    ---------------------------------------MASV---HEVIEAF-RQA---
gi|493372923|ref|WP_006329236.1|helicase[Gordoniarhizosphera]_gi|403207917|dbj|GAB88168.1|hypotheticalproteinGORHZ_006_00370[Go    ---------------------------------------MGSI---HQVIEAF-RQA---
gi|764904870|ref|WP_044487821.1|hypotheticalprotein[Mycobacteriumtuberculosis]_gi|625005353|gb|KBZ57120.1|hypotheticalproteinK8    ---------------------------------------MGSI---HEVMEAF-RKA---
gi|927068212|ref|WP_053778441.1|damageinducibleprotein[Gordoniasp.NB41Y]_gi|926660541|gb|EMP14526.2|damageinducibleprotein[G/11    ---------------------------------------MGSI---HEVMAAF-REA---
gi|935514169|ref|WP_054429894.1|damageinducibleprotein[Mycobacteriumimmunogenum]_gi|929801120|gb|KPG29247.1|damageinduciblepr/1    ---------------------------------------MGSI---HEVMEAF-REA---
gi|941317728|ref|WP_055118574.1|hypotheticalprotein[Mycobacteriumperegrinum]/11657    ---------------------------------------MGSI---HEVMEAF-RKA---
gi|933683673|gb|ALG86377.1|hypotheticalproteinACH46_20120[Gordoniasp.QH11]/11636    ---------------------------------------MGSV---HEVIEAF-RKE---
gi|873553947|emb|CRY84110.1|Predictedhelicase[Nocardiafarcinica]/11688    -ALAV-----GAG-LGRMV------GLGLLAWG-RCEWVMGSV---HEVIEAF-RQA---
gi|499525749|ref|WP_011212389.1|helicase[Nocardiafarcinica]_gi|54019339|dbj|BAD60707.1|hypotheticalprotein[NocardiafarcinicaIFM    ---------------------------------------MGSV---HEVIEAF-RLA---
gi|640109609|ref|WP_024794187.1|damageinducibleprotein[Tomitellabiformata]/11653    ---------------------------------------MASV---HEVIEAF-RKA---
gi|640109991|ref|WP_024794334.1|hypotheticalprotein[Tomitellabiformata]/11639    ---------------------------------------MASV---HEVIEAF-RTA---
gi|939125496|ref|WP_054831438.1|damageinducibleprotein[Rhodococcuserythropolis]/11669    ---------------------------------------MGSV---HEVIEAF-RQA---
gi|910258550|ref|WP_050062711.1|hypotheticalprotein[Rhodococcussp.RD6.2]_gi|846871967|emb|CRK49225.1|conservedhypotheticalprote    ---------------------------------------MGSV---HEVIEAF-RQA---
gi|797148189|gb|KJY52358.1|putativeHelicase[Bifidobacteriumasteroides]/11801    ----------------MT--------KGLIVEVMVDNNYMAVF---QDVLNVI-EKS---
gi|535476570|gb|EFG27357.2|hypotheticalproteinHMPREF9020_00999[ScardoviainopinataF0304]/11827    -----------KC-RDYF--------ELFFQNDIIANMATTTF---DDVLDAI-EKLDAV
gi|737482729|ref|WP_035462496.1|hypotheticalprotein[Alicyclobacillusmacrosporangiidus]/11512    ------------------------------------------------------------
```

```
gi|685434354|gb|KGA95679.1|helicase[BacillusalcalophilusATCC27647=CGMCC1.3604]/11575    -------------------------MDESKVRGLELKNHKSF---VALMNEI-------
gi|945370253|gb|KQU27251.1|helicase[Bacillussp.Leaf13]/11557    -------------------------MSEE---L---KSAKSF---ISLVKEM-------
gi|880997015|ref|WP_048719116.1|helicase[Enterococcushirae]/11563    ---------------------------------MESIQQKSF---ISLMEEI-------
gi|945356929|gb|KQU14100.1|helicase[Bacillussp.Leaf49]/11547    ------------------------------------------------------M----
gi|763482876|gb|KIZ53217.1|helicase[Bacillussafensis]/11563    ---------------------------------MKLANQKSF---VSLMNEI-------
gi|852223292|ref|WP_048309707.1|helicase[Anaerobacillusmacyae]_gi|850322910|gb|KMM38590.1|helicase[Anaerobacillusmacyae]/11573    ---------------------------MEE---IKGQVIRNSF---DKIIHDL-DI---
gi|739708665|ref|WP_037562619.1|helicase[Sporolactobacillusterrae]/11573    -------------------------MEEK---VSYNVTSQSF---DKIISQL-------
gi|496687561|ref|WP_009329104.1|MULTISPECIES:helicase[Bacillus]_gi|317390867|gb|EFV71668.1|endonucleaseandmethylaseLlaGI[Bacill    -------------------------MEEK---IGYH-VSHSF---DKIIRNL-------
gi|930213599|gb|KPH74225.1|helicase[Oceanobacilluscaeni]/11569    -------------------------MEET---VSYQ-VSHSF---DKIISNL-------
gi|516366003|ref|WP_017756036.1|helicase[Bacillussp.ZYK]/11571    -------------------------MEEK---IDYQVVSNAF---DKIIHDF-------
gi|488909977|ref|WP_002821052.1|helicase[Oenococcusoeni]_gi|399965230|gb|EJN99855.1|endonucleaseandmethylaseLlaGI[Oenococcusoen    ----------------------------------MNASTQSF---DRLVEEI-N-----
gi|495019967|ref|WP_007745780.1|helicase[Oenococcuskitaharae]_gi|366983682|gb|EHN59081.1|typeIIrestrictionenzyme,ressubunit[Oe    ----------------------------------MNASTQSF---DRLVEEI-N-----
gi|489666119|ref|WP_003570406.1|typeIIrestrictionendonuclease[Lactobacilluscasei]_gi|410527834|gb|EKQ02696.1|typeIIrestrictio    -----------------------------MSKTDHISVNSSF---DRLIEQI-D-----
gi|511447623|gb|EPC78110.1|superfamilyIIDNA/RNAhelicase[Lactobacillusparacaseisubsp.paracaseiLpp221}/11615    ----------DRQ-TDRQ-------TDRQTDRQTDRQTDHNF---DNLIAKI-D-----
gi|497700411|ref|WP_010014595.1|helicase[Lactobacilluscoryniformis]/11585    ------------------------------MRPDQTRPENDF---DSLIAQI-D-----
gi|489784048|ref|WP_003687939.1|helicase[Lactobacillusmali]_gi|394428539|gb|EJF01080.1|superfamilyIIDNA/RNAhelicase[Lactobacill    ------------------------------------MLKQSF---DNLMAQI-D-----
gi|446780825|ref|WP_000858081.1|helicase[Staphylococcusaureus]_gi|374396604|gb|EHQ67835.1|typeIIIrestrictionenzyme,ressubunit[S    ----------------------------------MKTSFKAF---NNIINTI-N-----
gi|510796658|ref|WP_016171456.1|hypotheticalprotein[Enterococcusdispar]_gi|508167532|gb|EOT44083.1|hypotheticalproteinOMK_00227    ----------------M--------SEENINYHVSKDSEKTF---DKLINQI-N-----
gi|612613473|gb|EZS30411.1|hypotheticalproteinW603_02482[StaphylococcusaureusVET0353R]_gi|612881917|gb|EZU93992.1|hypotheticalp    ----------------M--------SEEMRDYQVSNDSEKIF---DKILKQI-N-----
gi|488332893|ref|WP_002402278.1|helicase[Enterococcusfaecalis]_gi|306513684|gb|EFM82466.1|helicaseCterminaldomainprotein[Enter/    ---------------------------------------MTF---DELIQLA-R-----
gi|921166029|ref|WP_053111806.1|helicase[Enterococcusdurans]_gi|915771660|gb|AKX85928.1|helicase[Enterococcusdurans]/11562    ---------------------------------------MTF---DELIQLA-R-----
gi|498525358|ref|WP_010825030.1|hypotheticalprotein[Enterococcusfaecalis]_gi|487059631|gb|EOJ86727.1|hypotheticalproteinWOC_005    ---------------------------------------MTF---DELIQLA-R-----
gi|498412624|ref|WP_010722070.1|hypotheticalprotein[Enterococcusfaecium]_gi|486634654|gb|EOF75253.1|hypotheticalproteinSGC_0253    ---------------------------------------MTF---DELIQLA-R-----
gi|498408534|ref|WP_010718364.1|hypotheticalprotein[Enterococcusfaecium]_gi|486609608|gb|EOF50873.1|hypotheticalproteinSCW_0257    ---------------------------------------MTF---DELIQLA-R-----
gi|498407475|ref|WP_010717483.1|hypotheticalprotein[Enterococcusfaecalis]_gi|486594855|gb|EOF36348.1|hypotheticalproteinSCK_005    ---------------------------------------MTF---DELIQLA-R-----
gi|514885392|ref|WP_016615344.1|helicase[Enterococcusfaecalis]_gi|514505843|gb|EPH71676.1|helicaseprotein[Enterococcusfaecalis0    ---------------------------------------MTF---DELIQLA-R-----
gi|325157603|gb|EGC69759.1|helicaseCterminaldomainprotein[EnterococcuscasseliflavusATCC12755]/11561    ------------------------------MNILRRRKIMTF---DELIQLA-R-----
gi|895856206|ref|WP_048967668.1|helicase[Enterococcusfaecalis]/11551    ---------------------------------------MTF---DELIQLA-R-----
gi|639458836|ref|WP_024636366.1|helicase[Enterococcusfaecium]_gi|658176502|gb|KEI51159.1|helicase[EnterococcusfaeciumUC7256]/1/    ---------------------------------------MTF---DELIQLA-R-----
gi|488266511|ref|WP_002337719.1|hypotheticalprotein[Enterococcusfaecium]_gi|430581409|gb|ELB19854.1|hypotheticalproteinOIQ_0351    ---------------------------------------MTF---DELIQLA-R-----
gi|446353356|ref|WP_000431211.1|helicase[Staphylococcusaureus]_gi|253726329|gb|EES95058.1|helicaseCterminaldomainprotein[Staph/    ------------------------------MENQENYNASSF---DDLISQI-N-----
gi|672712068|emb|CDM14336.1|DNAorRNAhelicaseofsuperfamilyII[StaphylococcusepidermidisPM221]/11464    ------------------------------MKTRTEHNISSF---DNLLKQI-E-----
gi|896298990|ref|WP_049279905.1|helicase[Staphylococcusaureus]/11571    ------------------------------MKTKAEDNISSF---DNLLKQI-E-----
gi|541903432|gb|ERH36190.1|hypotheticalproteinSEQU_00085[StaphylococcusequorumUMCCNS924]/11578    ------------------------------MKMKAEDNISSF---DNLLKQI-E-----
gi|488289747|ref|WP_002360955.1|helicase[Enterococcusfaecalis]_gi|256682216|gb|EEU21911.1|endonucleaseandmethylaseLlaGI[Enteroc    ---------------------------------MSEKTSHTF---DDLIQQI-N-----
gi|494392045|ref|WP_007207894.1|helicase[Enterococcusitalicus]_gi|315483798|gb|EFU74284.1|helicaseCterminaldomainprotein[Enter/    ---------------------------------MGEKTSHTF---DDLIQQI-N-----
gi|446350692|ref|WP_000428547.1|helicase[Bacilluscereus]_gi|228727580|gb|EEL78721.1|RestrictionmodificationsystemLlaBIII[Bacil/    ---------------------------------MENKNINTF---DDLIEQI-N-----
gi|488255302|ref|WP_002326510.1|hypotheticalprotein[Enterococcusfaecium]_gi|430480276|gb|ELA57459.1|hypotheticalproteinOGE_0473    ---------------------------------MKEKIFNTF---DSLIQQI-N-----
gi|843253770|ref|WP_047916698.1|helicase[Lactococcuspiscium]_gi|802124677|emb|CEN29502.1|EndonucleaseandmethylaseLlaGI,TypeISPr    ------------------------------------MTKQTF---DDLINQI-N-----
LlaBIII/11578    ---------------------------MVAFWKECKMTKQTF---DDLINQI-N-----
gi|499320531|ref|WP_011011023.1|helicase[Listeriainnocua]_gi|16415805|emb|CAC42019.1|pli0021[ListeriainnocuaClip11262]/11569    ------------------------------------MTKQTF---DDLINQI-N-----
gi|760026859|ref|WP_043709973.1|helicase[Weissellacibaria]_gi|758337165|gb|KIU20061.1|UvsWhelicase[Weissellacibaria]/11567    ------------------------------------MSSQNF---NDLIEQI-N-----
LlaGI/11570    ---------------------------MVAFWKECKMTKQTF---NDLINQI-N-----
gi|499988414|ref|WP_011669132.1|helicase[Lactococcuslactis]_gi|116109019|gb|ABJ74139.1|DNAorRNAhelicaseofsuperfamilyII[Lactococ    ------------------------------------MTKQTF---DDLINQI-N-----
gi|685938360|ref|WP_031670319.1|helicase[Listeriamonocytogenes]_gi|590403387|gb|EXL23261.1|hypotheticalproteinX846_1956[Listeri    ------------------------------------MTKQTF---DDLINQI-N-----
gi|530793219|gb|EQC56240.1|helicase[Lactococcuslactissubsp.cremorisTIFN5]_gi|530793247|gb|EQC56263.1|helicase[Lactococcuslactis    ------------------------------------MTKQTF---DDLINQI-N-----
gi|695270006|ref|WP_032492075.1|hypotheticalprotein[Listeriamonocytogenes]_gi|695212872|ref|YP_009081522.1|hypotheticalproteinp    ------------------------------------MTKQTF---DDLINQI-N-----
gi|504101310|ref|WP_014335297.1|helicase[Leuconostocmesenteroides]_gi|859247905|gb|AKO63885.1|helicase(plasmid)[Leuconostocmese    ------------------------------------MKTQTF---NDLIEQI-N-----
gi|488905190|ref|WP_002816265.1|helicase[Leuconostocmesenteroides]_gi|227351522|gb|EEJ41777.1|helicaseCterminaldomainprotein[L/    ------------------------------------MKTQTF---NDLIEQI-N-----
gi|518121285|ref|WP_019291493.1|MULTISPECIES:hypotheticalprotein[Lactococcus]/11588    ---------------------------------------MSF---DTIIQEI-N-----
gi|754857213|ref|WP_042217902.1|helicase[Lactococcusgarvieae]/11549    ---------------------------------------MTF---DKLIQEI-N-----
gi|640048455|ref|WP_024784762.1|helicase[Streptococcusmutans]/11564    ---------------------------------------MSF---DNLVAQI-N-----
gi|512695361|ref|WP_016476323.1|hypotheticalprotein[Veillonellasp.HPA0037]_gi|512079853|gb|EPD79018.1|hypotheticalproteinHMPREF    ---------------------------------------MSF---DNLINQI-N-----
gi|636963514|ref|WP_024403747.1|helicase[Streptococcussuis]/11574    ---------------------------------------MSF---DYLVAQI-N-----
gi|881115312|ref|WP_048802949.1|helicase[Staphylococcuspasteuri]/11573    ---------------------------------------MSF---EKLVEQI-N-----
gi|446063996|ref|WP_000141851.1|helicase[Streptococcusmitis]_gi|307618725|gb|EFN97864.1|endonucleaseandmethylaseLlaGI[Streptoco    ---------------------------------------MTF---DNLVHQI-N-----
gi|488206609|ref|WP_002277817.1|restrictionmodificationsystemLlaBIII[Streptococcusmutans]_gi|449173033|gb|EMB75626.1|restricti/    ---------------------------------------MNF---NQLINQI-N-----
gi|640023290|ref|WP_024781621.1|helicase[Streptococcusmutans]/11566    ---------------------------------------MNF---NQLINQI-N-----
gi|814946581|ref|WP_046393781.1|helicase[Streptococcusuberis]_gi|810600885|gb|KKF57499.1|helicase[StreptococcusuberisB362]/1156    ---------------------------------------MSF---DDLVNQI-D-----
gi|765470762|ref|WP_044720336.1|helicase[Streptococcussuis]/11559    ---------------------------------------MTF---DTLVRQI-N-----
gi|654507127|ref|WP_027976443.1|helicase[Streptococcusdevriesei]/11558    ---------------------------------------MSF---DNLVAQI-N-----
gi|530812902|gb|EQC72481.1|DNAorRNAhelicaseofsuperfamilyII[Streptococcussp.HSISS2]/11570    ---------------------------------------MTF---DNLVAQI-N-----
gi|489158846|ref|WP_003068504.1|helicase[Streptococcusintermedius]_gi|355367114|gb|EHG14827.1|hypotheticalproteinHMPREF9682_002    ---------------------------------------MSF---DNLVNQI-N-----
RM.Lru27782ORF13200P/11620    ----------D---RVLL-------GMELRKFKGKADIMNAF---NHLIEHI-N-----
gi|492676546|ref|WP_005919236.1|DNAphosphorothioationsystemrestrictionenzyme[Pediococcusacidilactici]_gi|482517924|gb|EOA08103.    --------------------------------------MAKF---NELITQI-N-----
gi|498183116|ref|WP_010497272.1|helicase[Lactobacillusacidipiscis]/11563    --------------------------------------MATF---KELLQQI-D-----
gi|822100485|ref|WP_046921865.1|helicase[Lactobacillusruminis]_gi|821684442|gb|KLA43321.1|restrictionmodificationsystemLlaBIII/    --------------------------------------MATF---NELVNQI-D-----
```

```
gi|935565832|ref|WP_054450311.1|helicase[Lactobacilluskunkeei]/11573        ----------------------------------MTILSSKTF---NELIDEI-N-----
gi|752296887|ref|WP_041152901.1|helicase[Lactobacilluskunkeei]_gi|751663520|gb|KIM18358.1|helicase[Lactobacilluskunkeei]/11577        ----------------------------------MTILSSKTF---NELINEI-N-----
gi|495745291|ref|WP_008469870.1|helicase[Lactobacillushominis]_gi|394484442|emb|CCI81207.1|RestrictionmodificationsystemLlaBII/        ---------------------------------------MATF---EELIEQV-N-----
gi|918672414|ref|WP_052543134.1|helicase[Lactobacillussp.]/11564        ---------------------------------------MATF---DELVKQV-N-----
gi|799141631|ref|WP_045927261.1|helicase[Lactobacilluskullabergensis]_gi|797155335|gb|KJY59264.1|putativerestrictionendonucleas        ---------------------------------------MASF---DELVEQI-D-----
gi|504379990|ref|WP_014567092.1|helicase[Lactobacillusjohnsonii]_gi|329666726|gb|AEB92674.1|putativerestrictionendonuclease[Lac        ---------------------------------------MATF---DELVDQV-N-----
gi|489880179|ref|WP_003783646.1|DNAdamageinducibleprotein[Actinomycesnaeslundii]_gi|399901561|gb|EJN84440.1|typeIIIrestriction/        ------------------------------------MLLPVSQSRIAEILHAL-DFE---
gi|496524497|ref|WP_009232780.1|DNAdamageinducibleprotein[Actinomycessp.oraltaxon849]_gi|365265413|gb|EHM95177.1|hypotheticalp/        ------------------------------------MLLLVSQSRIAEILDGL-YFE---
gi|915397782|ref|WP_050783167.1|damageinducibleprotein[Actinomycesoris]/11694        ---------------------------------------------MKVILDGL-YFD---
gi|502925248|ref|WP_013160224.1|helicase[Propionibacteriumfreudenreichii]_gi|296921272|emb|CBL55824.1|helicase[Propionibacteriu        ------------------------------------MATRETI---HDVIERF--RE---
gi|732164352|emb|CEG87481.1|helicase[Propionibacteriumfreudenreichii]/11618        ----------GGT-----------------------VAKRSTI---HEVINQF--RQ---
gi|739047402|ref|WP_036925058.1|damageinducibleprotein[Propionibacteriumthoenii]/11596        ------------------------------------MAKVRTI---QDVIEEF--GC---
gi|739112839|ref|WP_036982533.1|damageinducibleprotein[Propionibacteriumjensenii]/11594        ------------------------------------MAKVRTI---QDVIEEF--GC---
gi|918023558|ref|WP_052314121.1|damageinducibleprotein[Nocardiathailandica]/11612        ----------GGT-----------------RRPGRLSSVPVTI---HELLDEL-RSS---
gi|498380271|ref|WP_010694427.1|DNAdamageinducibleprotein[Saccharopolysporaspinosa]/11605        --------------------------------------MPTTI---TDLLDDL-RAT---
gi|749991459|ref|WP_040339809.1|damageinducibleprotein[CandidatusBlastococcusmassiliensis]/11597        --------------------------------------MTGTI---HAVLDRL-RDE---
gi|754177801|ref|WP_041796231.1|damageinducibleprotein[Modestobactermarinus]/11592        -----------------------------------MRSVTGTI---HTVLDRL-RSE---
gi|504188248|ref|WP_014375350.1|DNAdamageinducibleprotein[Blastococcussaxobsidens]_gi|378782790|emb|CCG02456.1|putativeHelicas/        --------------------------------------MTGTI---HAVLDRL-RGE---
gi|946476713|gb|KRF26798.1|damageinducibleprotein[Phycicoccussp.Soil803]/11609        ----------------------------------------------MEKLDQIWTTY---
gi|700176672|gb|KGN37509.1|DNAdamageinducibleprotein[KnoelliasubterraneaKCTC19937]/11633        -------------------------------------MVETDRDFLLGKLDEIWNEF---
gi|700168841|gb|KGN29914.1|hypotheticalproteinN802_10215[KnoelliasinensisKCTC19936]/11631        ---------------------------------------------MLGKLDEIWNEF---
gi|946426241|gb|KRE76610.1|damageinducibleprotein[Arthrobactersp.Soil763]/11606        --------------------------------------MPTTV---YNVLDQL-RDS---
gi|517318525|ref|WP_018500927.1|hypotheticalprotein[Frankiasp.BCU110501]/11592        --------------------------------------MPTTI---YDVLDQL-RAS---
gi|499072429|ref|WP_010851327.1|Helicase[Tetrasphaeraelongata]_gi|478756579|emb|CCH71501.1|putativeHelicase[Tetrasphaeraelongat        --------------------------------------MSTSI---YEVLDEL-DGQ---
gi|750080666|ref|WP_040385350.1|damageinducibleprotein[Demetriaterragena]/11625        -------------------------------------MGTATV---YDVLDEL-RAQ---
gi|752558292|ref|WP_041229523.1|damageinducibleprotein[Dermacoccusnishinomiyaensis]_gi|664689315|gb|AIF41904.1|damageinducibl/1        -------------------------------------MSAVSV---AEVLEQL-RAT---
gi|829089213|ref|WP_047310701.1|damageinducibleprotein[Dermacoccussp.PE3]_gi|828384812|gb|KLO63770.1|damageinducibleprotein[D/1        --------------------------------------MSVTV---HEVLDDL-REK---
gi|748756635|ref|WP_040014750.1|damageinducibleprotein[Dermacoccussp.Ellin185]/11596        --------------------------------------MSVTV---HEVLDDM-RAK---
gi|916534724|ref|WP_051141848.1|damageinducibleprotein[Actinomycesneuii]/11632        ----------GGC-------------AVKT------VADLAAF---DALLDSY-RAL---
gi|921140030|ref|WP_053090276.1|damageinducibleprotein[Rothiamucilaginosa]/11616        ----------------------------------------MAF---DNLLHQL-RTT---
gi|896406751|ref|WP_049343749.1|damageinducibleprotein[Rothiamucilaginosa]/11647        ----------G-------------------TPADVSTKTSMAF---DNLLHQL-RTT---
gi|723608234|gb|AIY01610.1|putativehelicase[Arthrobactersp.PAMC25486]/11613        ------------------------------------------------------------
gi|946394527|gb|KRE45160.1|damageinducibleprotein[Arthrobactersp.Soil736]/11616        ----------------------------------MGAFVSTTF---DQLLDSY-RDL---
gi|515765304|ref|WP_017197904.1|hypotheticalprotein[Arthrobactersp.M2012083]/11648        ----------GSC-------------RLHLLLFSGVTLLATTF---DVLLDSY-RDL---
gi|945315587|gb|KQT73127.1|damageinducibleprotein[Microbacteriumsp.Leaf436]/11612        ------------------------------------------M---HDVLEQY-RKV---
gi|759908497|ref|WP_043594599.1|damageinducibleprotein[Frigoribacteriumsp.MEB024]_gi|758271359|gb|KIU03346.1|damageinduciblep/1        ---------------------------------------MKTI---HDLLAEY-ASI---
gi|945072850|gb|KQR34237.1|hypotheticalproteinASF75_00055[Curtobacteriumsp.Leaf154]/11597        --------------------------------------MAMSI---HDLLDVY-ATI---
gi|551263152|ref|WP_022883363.1|DNAdamageinducibleprotein[Glaciibactersuperstes]/11621        ---------------------------------------MTTI---HELLDEY-ARM---
gi|945452341|gb|KQV08491.1|hypotheticalproteinASC63_10170[Leifsoniasp.Root112D2]/11623        ------------------------------------------M---RGLLEQY-TRL---
gi|742759483|gb|KIC69912.1|damageinducibleprotein[Kocuriarhizophila]/11610        -------------------------------------MTQDAF---GELLDKL-YYG---
gi|737919995|ref|WP_035885304.1|damageinducibleprotein[Kocuriarhizophila]/11611        -------------------------------------MTQDAF---GELLDKL-YYG---
gi|737997701|ref|WP_035959325.1|damageinducibleprotein[Kocuriamarina]_gi|725823884|gb|KHE75603.1|damageinducibleprotein[Kocur/1        -------------------------------------MTQDAF---GELLDKL-YYG---
gi|664521070|ref|WP_031037650.1|damageinducibleprotein[Streptomycessp.NRRLWC3774]/11624        ------------------------------------MATTTSV---HDVIRAIRHGS---
gi|500101034|ref|WP_011777041.1|DNAdamageinducibleprotein[Arthrobacteraurescens]_gi|119951547|gb|ABM10457.1|putativeHelicase[A/        -------------------------------------MASSPF---AALLDRL-YFT---
gi|916871953|ref|WP_051479009.1|damageinducibleprotein[Arthrobactersp.H5]/11588        -------------------------------------MVSSSF---ATLLDKL-YFT---
gi|612084649|gb|EZP43369.1|TypeIIIrestrictionenzyme,ressubunit[Micrococcusluteus]/11625        ----------PDAAS--------STDPSAMDEGDPGSESRVGF---DEVLDRL-WLS---
gi|759739708|ref|WP_043450422.1|damageinducibleprotein[Arthrobacterphenanthrenivorans]_gi|742758264|gb|KIC68716.1|damageinduc/1        -------------------------------------MGSASF---EDVLDRL-YFS---
gi|636845448|ref|WP_024366823.1|damageinducibleprotein[Arthrobactersp.TB26]/11605        -------------------------------------MGSNTF---EQLLDRL-YFS---
gi|500009025|ref|WP_011689743.1|DNAdamageinducibleprotein[Arthrobactersp.FB24]_gi|116613051|gb|ABK05760.1|typeIIIrestrictionen/        -------------------------------------MGSNTF---EQLLDRL-YFS---
gi|821249847|gb|KKZ12083.1|hypotheticalproteinTQ37_06090[CandidatusSynechococcusspongiarum15L]/11436        ---------------------------------------MHPL---DEILQSW-RQE---
gi|739446902|ref|WP_037306885.1|damageinducibleprotein[Ruegeriahalocynthiae]/11592        ---------------------------------------MSAL---DRILQSY-RNA---
gi|640620081|ref|WP_025048131.1|damageinducibleprotein[Sulfitobactermediterraneus]_gi|751956650|gb|KIN77213.1|TypeIIIrestricti/        ---------------------------------------MSAL---DRILDGF-RNA---
gi|740247634|ref|WP_038088583.1|damageinducibleprotein[Thiobacillusprosperus]_gi|684049748|gb|KFZ90853.1|putativehelicase[Thio/        ---------------------------------MDEAARPDSI---DAVLDNL-RAR---
gi|518420321|ref|WP_019590528.1|hypotheticalprotein[Thioalkalivibriosp.ALE20]/11603        --------------------------------------MTTTV---DEVLERF-RER---
gi|500156826|ref|WP_011831496.1|DNAdamageinducibleprotein[Methylibiumpetroleiphilum]_gi|124261892|gb|ABM96881.1|adeninespecifi/        --------------------------------MSKRTPKVTSL---DDVLSHF-REA---
gi|769978537|ref|WP_045094522.1|damageinducibleprotein[Legionellafallonii]_gi|698841736|emb|CEG55682.1|Putativeproteinwithboxh/        ---------------------------------------MTTI---NDLLVDF-SKS---
gi|553752541|ref|WP_023085344.1|hypotheticalprotein[Pseudomonasaeruginosa]_gi|552201952|gb|ERU62653.1|hypotheticalproteinQ088_0        ---------------------------------------MTTL---NDILSAF-RDA---
gi|880980418|ref|WP_048704461.1|damageinducibleprotein[Thauerahumireducens]_gi|875132785|gb|KMU70944.1|damageinducibleprotein/1        ---------------------------------------MTTL---ESILTEF-REA---
gi|740135732|ref|WP_037982815.1|damageinducibleprotein[Thauerasp.27]/11603        ---------------------------------------MTTL---NDILDEL-REA---
gi|738017371|ref|WP_035978332.1|damageinducibleprotein[Burkholderiaglumae]/11380        ----------------------------------------MTF---QDLLHEL-TEA---
gi|498506003|ref|WP_010806671.1|typeIIIrestrictionenzyme,ressubunit[Pandoraeasp.SD62]_gi|490314561|gb|EON12879.1|typeIIIrestri/        ----------------------------------------MTF---QELLDEL-TEA---
gi|500117748|ref|WP_011793753.1|DNAdamageinducibleprotein[Acidovoraxcitrulli]_gi|120587742|gb|ABM31182.1|typeIIIrestrictionenz/        ----------------------------------------MTF---QELLDEL-AEA---
gi|651617328|ref|WP_026611444.1|damageinducibleprotein[Methylocaldumszegediense]/11601        ----------------------------------------MNF---QELLNEL-ASA---
gi|737210208|ref|WP_035194994.1|damageinducibleprotein[Acidithiobacillusferrivorans]_gi|669953220|emb|CDQ11755.1|Adeninespecif/        -------------------------------------MPVNTI---HDILAQF-RQY---
gi|935697386|ref|WP_054506180.1|damageinducibleprotein[Achromobactersp.]_gi|928716153|emb|CUK12836.1|Hefnuclease[Achromobacter/        -----------------------------------------MI---RTILQQY-RDN---
gi|429502517|gb|ELA00827.1|adeninespecificDNAmethyltransferase[Cupriavidussp.HMR1]/11599        -------------------------MRDFSEISTRTTDRHLMI---RTILQQY-RDN---
gi|506356642|ref|WP_015876361.1|DNAdamageinducibleprotein[Burkholderiaglumae]_gi|237877158|gb|ACR29491.1|AdeninespecificDNAmet/        -----------------------------------------MI---RTILQQY-RDE---
```

```
gi|696406787|ref|WP_032978003.1|damageinducibleprotein[Bordetellahinzii]_gi|625335076|gb|KCB49343.1|typeIIIrestrictionenzyme,r/ -----------------------------------------MI---RDILQQY-RDE---
gi|817529125|gb|KKO72587.1|damageinducibleprotein[Kerstersiagyiorum]/11592 -----------------------------------------MI---RDILQQY-RNE---
gi|737048910|ref|WP_035044118.1|damageinducibleprotein[Aquabacteriumsp.NJ1]_gi|699052896|gb|KGM38706.1|damageinducibleprotein/1 -----------------------------------------MI---RSILQQY-RDE---
gi|822492519|ref|WP_046933319.1|MULTISPECIES:damageinducibleprotein[Xanthomonas]_gi|821703525|gb|KLA61196.1|damageinduciblepr/1 -----------------------------------------MI---RTILQQY-RDE---
gi|751252861|ref|WP_040963740.1|damageinducibleprotein[Xanthomonasfuscans]_gi|749808363|gb|KIJ03887.1|damageinducibleprotein[/1 -----------------------------------------MI---RTILQQY-RDE---
gi|496774980|ref|WP_009368792.1|DNAdamageinducibleprotein[Bilophilasp.4_1_30]_gi|345039902|gb|EGW44205.1|hypotheticalproteinHM/ -----------------------------------------ML---RQILEDY-RK----
gi|499241912|ref|WP_010939452.1|DNAdamageinducibleprotein[Desulfovibriovulgaris]_gi|46580580|ref|YP_011388.1|adeninespecificDN/ -----------------------------------------ML---HQILQKY-RE----
gi|504274913|ref|WP_014462015.1|DNAdamageinducibleprotein[Bacillusmegaterium]_gi|345446919|gb|AEN91936.1|AdeninespecificDNAmet/ ---------------------------------------MNGF---NELLNEL-REI---
gi|504895966|ref|WP_015083068.1|typeIIrestrictionenzymeandmethyltransferasesubtypeG[Anabaenasp.90]_gi|413972424|gb|AFW96512.1|t ----------------------------------------MTI---NTILAEF-RQN---
gi|518327765|ref|WP_019497972.1|hypotheticalprotein[Calothrixsp.PCC7103]/11600 ---------------------------------------MATI---HTLLEEF-RQE---
gi|652765503|ref|WP_027079917.1|damageinducibleprotein[Luteimonasmephitis]/11593 -------------------------------------MAETTI---HDILEEL-AAA---
gi|896539951|ref|WP_049442123.1|damageinducibleprotein[Stenotrophomonasmaltophilia]/11591 -------------------------------------MAETTI---HHILDEL-AEA---
gi|500217900|ref|WP_011888043.1|DNAdamageinducibleprotein[Geobacillusthermodenitrificans]_gi|134267954|gb|ABO68149.1|Conserved/ ---------------------------------------MTTI---YDVLEQL-RNV---
gi|696570298|ref|WP_033102014.1|damageinducibleprotein[Thermoactinomycesdaqus]/11594 ---------------------------------------MTTI---YDILNQL-RQF---
gi|251772067|gb|EES52637.1|putativehelicase[Leptospirillumferrodiazotrophum]/11618 ----------------------------------------MQI---QDVLKSF-RRI---
gi|738124101|ref|WP_036082108.1|damageinducibleprotein[Leptospirillumferriphilum]_gi|685243306|gb|KGA94177.1|putativehelicase[/ ----------------------------------------MQI---QDVLDSF-RRN---
gi|558699223|ref|WP_023524766.1|helicase[Leptospirillumsp.GroupII'CF1']_gi|206603764|gb|EDZ40244.1|Putativehelicase[Leptospiri/ ----------------------------------------MQI---QDVLESF-RRI---
gi|406774462|gb|AFS53887.1|putativehelicase[LeptospirillumferriphilumML04]/11610 ---------------------------------------MTTL---HSILEQI-RQH---
gi|740719463|ref|WP_038504749.1|damageinducibleprotein[Leptospirillumferriphilum]_gi|640838125|gb|AIA30173.1|damageinduciblep/1 ---------------------------------------MTTL---HFILEQI-RQH---
gi|517810766|ref|WP_018980974.1|hypotheticalprotein[Saccharibacterfloricola]/11733 -----------------MP--------ENTLASTVLSSQETPL---EKLLGWI-DTS---
gi|502471675|ref|WP_012798533.1|helicase[Slackiaheliotrinireducens]_gi|256791761|gb|ACV22431.1|predictedhelicase[Slackiaheliotr DVLDLDEDVLDQIQTDILGFE--KHKAQRVNWSKEPSQTIYYV---IDW---I-RDT---
gi|657871713|ref|WP_029576297.1|hypotheticalprotein[Bifidobacteriumthermacidophilum]_gi|673007521|gb|KFJ02535.1|helicase[Bifido -------------------ME--HDETDE---TATPYDGHSIL---DEWMKNI-LKT---
gi|673004757|gb|KFI99791.1|DNAhelicaserestrictionenzymetypeIIIRessubunit[Bifidobacteriumthermacidophilumsubsp.porcinum]/11682 -----------------MVME--HDEADE---IATPNDGSSIL---DEWMKKI-LKT---
gi|917316092|ref|WP_051922804.1|hypotheticalprotein[Bifidobacteriumstellenboschense]_gi|673003774|gb|KFI98812.1|helicase[Bifido ----------------------------------------MSI---DEIFARI-VTE---
gi|705415694|ref|WP_033502332.1|hypotheticalprotein[Bifidobacteriumpseudolongum]_gi|672982411|gb|KFI77685.1|DNAdamageinducible/ ---------------------------------------MSNV---EELFARI-VAE---
gi|916591418|ref|WP_051198509.1|hypotheticalprotein[Bifidobacteriumsp.AGR2158]/11704 -----------------MAA------------EYREDGDRSFV---EDLFARI-VDE---
gi|772242982|gb|AJW78255.1|hypotheticalproteinVO01_03165[Clavibactermichiganensissubsp.insidiosus]/11642 -------------------------------MTPRRSEGNTSV---DRLLNEL-RGL---
gi|916649818|ref|WP_051256909.1|damageinducibleprotein[Actinomycesgraevenitzii]/11677 -----------------------RLMTNARTEALPTASSASAL---DSLLNEY-RTL---
gi|496504578|ref|WP_009212861.1|helicase[Actinomycessp.oraltaxon180]_gi|315312695|gb|EFU60776.1|helicase[Actinomycessp.oraltaxo -----------------ATHH-PEK---RLSDDAAPASSKSAL---DALLDEY-RAR---
gi|494862206|ref|WP_007588306.1|helicase[Actinomycessp.ICM39]_gi|398362799|gb|EJN46470.1|typeIIIrestrictionenzyme,ressubunit[Ac -----------------AHDT-ARHR----SDDTAPASSKSAL---DALLDEY-RAR---
gi|881057111|ref|WP_048774974.1|damageinducibleprotein[Sanguibacterkeddieii]/11697 -----------------AHDT-ARHR----SDDTAPASSKSAL---DALLDEY-RAR---
gi|881020459|ref|WP_048740808.1|damageinducibleprotein[Kytococcussedentarius]/11704 -----------------AHDT-ARHR----SDDTAPASSKSAL---DALLDEY-RAR---
gi|881052313|ref|WP_048770555.1|damageinducibleprotein[Cellulomonasflavigena]/11705 -----------------APPT-AIDRSPGDAANTGPVSSKSAL---DALLDEY-RAH---
gi|490108682|ref|WP_004009424.1|helicase[Mobiluncuscurtisii]_gi|315490465|gb|EFU80089.1|helicaseCterminaldomainprotein[Mobilun/ ---------RDATQSDTATAS-TDVNAL--DSQREAARSVKAF---DELLEHY-REF---
gi|490111935|ref|WP_004012603.1|helicase[Mobiluncusmulieris]_gi|227843812|gb|EEJ53982.1|helicaseCterminaldomainprotein[Mobilun/ -----------------MEAN-PETPAVNDQAIAEQGASAAAF---DRLLAQY-RGS---
gi|490114228|ref|WP_004014848.1|helicase[Mobiluncusmulieris]_gi|269934432|gb|EEZ90993.1|helicaseCterminaldomainprotein[Mobilun/ -----------------MEAN-PETPAVNDQAIAEQGASAAAF---DRLLAQY-RGS---
gi|493333551|ref|WP_006290636.1|helicase[Parascardoviadenticolens]_gi|315119487|gb|EFT82620.1|helicaseCterminaldomainprotein[P/ -----------------MVTI------MSTPVTDDVIPSIKTV---FDAVEYI-KQN---
gi|495817067|ref|WP_008541646.1|helicase,partial[Sutterellaparvirubra]_gi|378598583|gb|EHY31712.1|helicaseprotein,partial[Sutte ------------------------------------------------------MA----
gi|491572696|ref|WP_005430278.1|helicase[Sutterellawadsworthensis]_gi|319805116|gb|EFW01943.1|DNAhelicaserestrictionenzymeTypeI ------------------------------------MSEFANN---ESAFEAL-LES---
gi|748686321|ref|WP_039944445.1|hypotheticalprotein[Thermicanusaegyptius]/11573 ----------------------------------------MNV---QELLERL-EKT---
gi|916875698|ref|WP_051482754.1|hypotheticalprotein[Thioalkalivibriosp.HK1]/11366 -----------------MSFQ------ADSKVGGDWAMASDDF---EGLLQRL-RKS---
gi|821248604|gb|KKZ10960.1|hypotheticalproteinTE42_09075[CandidatusSynechococcusspongiarumSP3]/11598 ---------------------------------------MHPL---DQILQTW-RQE---
gi|737409843|ref|WP_035390956.1|hypotheticalprotein[Ferrimicrobiumacidiphilum]_gi|765544266|gb|KJE76241.1|typeIrestrictionenzym ----------------------------------------MNL---TDLLDKL-TRL---
gi|654906925|ref|WP_028358281.1|damageinducibleprotein[Brackiellaoedipodis]/11679 -----------------------------------MTTVQTPL---QKLLQQM-RTN---
gi|502809901|ref|WP_013044877.1|hypotheticalprotein[CandidatusPuniceispirillummarinum]_gi|292663146|gb|ADE38247.1|hypotheticalp ---------------------------------------MTTF---NDVLDAL-RAQ---
gi|251771370|gb|EES51950.1|typeIIIrestrictionenzyme,ressubunit[Leptospirillumferrodiazotrophum]/11628 ----------------------------------------MMI---EDVLKRL-RHD---
gi|498328480|ref|WP_010642636.1|DNAdamageinducibleprotein[Acidithiobacillusthiooxidans]/11627 --------------------------------------MPNSF---QEWMNEL-NRF---
gi|647633864|ref|WP_025906698.1|damageinducibleprotein[Prochlorococcussp.scB245a_519B7]/11622 --------------------------------------MSSTL---RKALEII-RQN---
gi|647619599|ref|WP_025893729.1|damageinducibleprotein[Prochlorococcussp.scB241_527N11]/11621 ---------------------------------------MSSF---QQALSLI-REK---
gi|755034887|ref|WP_042387498.1|hypotheticalprotein[Clostridiumsp.CL6]/11606 -----------------------------MEAVQKRVYDYTTV---NGLLEQF-KEL---
gi|498139798|ref|WP_010453954.1|helicase[SuccinivibrionaceaebacteriumWG1]/11662 ----------------------------------------MTI---FDILNLI-REQ---
gi|527501386|gb|EPT34571.1|typeIIIrestrictionenzyme,ressubunit[Bacteroidetesbacteriumoraltaxon272str.F0290]/11789 ---------------------------------------MKTF---DEILNLF-REY---
gi|496047275|ref|WP_008771782.1|helicase[Bacteroidessp.2_1_33B]_gi|262296443|gb|EEY84373.1|helicaseCterminaldomainprotein[Bact/ ----------------------------------------MTF---KEVLQRF-RTG---
PDI8503III/11664 ----------------------------------------MTF---KEVLQRF-RTG---
gi|738719431|ref|WP_036613715.1|helicase[Parabacteroidesdistasonis]_gi|649542518|gb|KDS49387.1|restrictionendonucleasefamilypro ----------------------------------------MTF---KEVLQRF-RTG---
gi|659417033|gb|KEJ86133.1|hypotheticalproteinHMPREF1002_00341[Porphyromonassp.31_2]/11656 ----------------------------------------MTF---KEVLQRF-RTG---
gi|567456807|gb|ETK11358.1|hypotheticalproteinT235_15845[Tannerellasp.oraltaxonBU063isolateCell8/11]/11433 ---------------------------------------MKTI---YDILKEF-RDE---
gi|545440279|ref|WP_021677877.1|helicase[Porphyromonasgingivalis]_gi|543985765|gb|ERJ89108.1|helicaseprotein[Porphyromonasgingi ---------------------------------------MNSI---KDILTHF-RNK---
gi|497438056|ref|WP_009752254.1|helicase[Prevotellasp.oraltaxon473]_gi|429144866|gb|EKX87968.1|helicaseprotein[Prevotellasp.ora ---------------------------------------MNNI---YDILSHF-REK---
gi|490423474|ref|WP_004295683.1|MULTISPECIES:helicase[Bacteroides]_gi|156110098|gb|EDO11843.1|helicaseCterminaldomainprotein[B/ ----------------------------------------MTF---NDILHKF-RSE---
gi|941777229|ref|WP_055173506.1|helicase[Bacteroidescaccae]_gi|933263395|emb|CUQ00567.1|Predictedhelicase[Bacteroidescaccae]/1/ ----------------------------------------MNF---KEILHKF-RTE---
gi|492413898|ref|WP_005836892.1|helicase[Bacteroidesuniformis]_gi|392678824|gb|EIY72229.1|hypotheticalproteinHMPREF1072_03583[B ----------------------------------------MNF---KEILHKF-RTE---
gi|695455095|ref|WP_032566191.1|helicase[Bacteroidesfragilis]_gi|596095426|gb|EXZ59996.1|restrictionendonucleasefamilyprotein[B ----------------------------------------MNF---KEILHKF-RTE---
gi|933227941|emb|CUQ02348.1|Predictedhelicase[Bacteroidesuniformis]/11658 ----------------------------------------MNF---KDILHKF-RTE---
gi|494823013|ref|WP_007558421.1|helicase[Bacteroidesplebeius]_gi|198272167|gb|EDY96436.1|helicaseCterminaldomainprotein[Bacter/ ----------------------------------------MNF---KEILHKF-RTE---
gi|495427212|ref|WP_008151909.1|helicase[Parabacteroidesjohnsonii]_gi|218222381|gb|EEC95031.1|helicaseCterminaldomainprotein[P/ ----------------------------------------MNF---KDILHKF-RTE---
gi|933196855|emb|CUO94545.1|typeIrestrictionenzymeEcoKIsubunitR[Bacteroidesuniformis]_gi|933220098|emb|CUN86874.1|typeIrestrict ----------------------------------------MNF---KEILHKF-RTE---
```

```
gi|823687748|ref|WP_047101121.1|helicase[Brachyspirahyodysenteriae]_gi|823334537|gb|KLI17932.1|helicase[Brachyspirahyodysenteri       ----------------------------------------MNL---DTIFDQF-NNN---
gi|496436691|ref|WP_009145538.1|helicase[Phascolarctobacteriumsuccinatutens]_gi|322414007|gb|EFY04839.1|helicaseCterminaldomai/       ---------------------------------------MATF---WSIIQDI-RKK---
gi|503372880|ref|WP_013607541.1|helicase[Sphaerochaetaglobosa]_gi|324026933|gb|ADY13692.1|typeIIIrestrictionproteinressubunit[S       ----------------------------------------MGL---QQILDKY-RKV---
gi|505821748|ref|WP_015707145.1|helicase[Treponemaprimitia]_gi|299483503|gb|ADJ19584.1|helicasedomaincontainingprotein[Trepone/       ---------------------------------------MNPF---ETVIQKY-RDT---
gi|505822741|ref|WP_015708134.1|helicase[Treponemaprimitia]_gi|333739639|gb|AEF85129.1|endonucleaseandmethylaseLlaGI[Treponemap       ---------------------------------------MNPF---ETVIQKY-RDT---
gi|488791994|ref|WP_002704400.1|helicase[Treponemasaccharophilum]_gi|380767691|gb|EIC01689.1|typeIIIrestrictionproteinressubuni       ----------------------------------MQNLSSTKF---DQIIDKY-RIE---
gi|524671454|emb|CDD81750.1|putativeseptumsitedeterminingproteinMinC[Bacteroidessp.CAG:462]/11642                                     ---------------------------------------MTTF---TKIIDKF-RRE---
gi|524573865|emb|CDC95766.1|putativeseptumsitedeterminingproteinMinC[Alistipessp.CAG:268]/11639                                      ---------------------------------------MSTF---AQILNKF-RQD---
gi|658617970|ref|WP_029759328.1|helicase[Fusobacteriumnucleatum]/11606                                                                ----------------------------------------MNL---KDILKEL-EEN---
gi|494412037|ref|WP_007216782.1|helicase[Bacteroidescellulosilyticus]_gi|392638921|gb|EIY32752.1|hypotheticalproteinHMPREF1062_       ---------------------------------------MTHL---HALLERF-RTL---
gi|383107102|gb|AFG37435.1|putativehelicase[SpirochaetaafricanaDSM8902]/11616                                                         --------------------------------------MAMTF---NQLLDKY-RKI---
SAF8902III/11616                                                                                                                      --------------------------------------MAMTF---NQLLDKY-RKI---
gi|652538894|ref|WP_026932796.1|helicase[Gramellaechinicola]/11633                                                                    ----------------------------------------MSF---QKVLEKY-RKY---
gi|639238223|ref|WP_024568839.1|helicase[Elizabethkingiaanophelis]/11628                                                              ---------------------------------------MTSF---NSILAKY-RKL---
gi|652520381|ref|WP_026914582.1|helicase[Gramellaportivictoriae]/11623                                                                ----------------------------------------MSF---KTILEHY-REF---
gi|494922183|ref|WP_007648219.1|Helicase[Formosasp.AK20]_gi|469825489|gb|EMQ95550.1|Helicasedomainprotein[Formosasp.AK20]/11626       ------------------------------------MATPNQF---NEILQDY-RNL---
gi|522083382|ref|WP_020594591.1|hypotheticalprotein[Spirosomapanaciterrae]/11637                                                      --------------------------------------MASNF---TTVLEKY-RKI---
gi|490455352|ref|WP_004326157.1|helicase[Bacteroidesovatus]_gi|392669277|gb|EIY62768.1|hypotheticalproteinHMPREF1070_03635[Bact       ----------------------------------------MTF---KNILDKF-RKY---
gi|499516035|ref|WP_011202675.1|helicase[Bacteroidesfragilis]_gi|53713270|ref|YP_099262.1|helicasedomainprotein[Bacteroidesfrag       ----------------------------------------MTF---KNILDKF-RKY---
gi|651338332|ref|WP_026461320.1|helicase[Adhaeribacteraquaticus]/11620                                                                ----------------------------------------MSF---QKILDKY-RKT---
gi|806964642|gb|AKC17139.1|helicase[Flavobacteriumpsychrophilum]/11639                                                                --------------------------------------MSKSF---NIILEKY-RKI---
gi|752684374|ref|WP_041332322.1|helicase[Flavobacteriumpsychrophilum]_gi|666882889|gb|AIG30715.1|helicase[Flavobacteriumpsychro       --------------------------------------MSKSF---NIILEKY-RKI---
gi|933196595|emb|CUP04894.1|DNAorRNAhelicasesofsuperfamilyII[Bacteroidesuniformis]/11631                                             ----------------------------------------MDF---NKVLEKY-RKY---
gi|670509109|ref|WP_031456918.1|helicase[Flavobacteriumchungangense]/11625                                                            ----------------------------------------MSF---NKILEKY-RKI---
```

```
gi|490190857|ref|WP_004089451.1|hypotheticalprotein[Helicobacterbilis]_gi|476630638|gb|EMZ37196.1|hypotheticalproteinC826_02193       ---SHVAMNNRTKGSIFEKLSTHLLRERD-TQGEYKS-IDLWTDWK---------YR---
gi|488896133|ref|WP_002807242.1|MULTISPECIES:restrictionendonucleaseEco57I[Campylobacter]_gi|380541096|gb|EIA65377.1|hypothetic       ---KKANLNTRSKGSMFEKLSKHLLKEKD-TANIYKN-IYLWDEWD---------KK---
gi|746602240|ref|WP_039628365.1|restrictionendonucleaseEco57I[Campylobacterlari]_gi|744819395|gb|AJD03391.1|putativehelicase[Ca       ---KKANLNTRSKGSMFEKLSKHLLKEKD-TANIYKN-IYLWDEWD---------KK---
gi|727317135|ref|WP_033772558.1|restrictionendonucleaseEco57I[Helicobacterpylori]/11622                                              ----QAIPNQRLKGSLFEKVCKRFLEEHD-SANEYES-IELWSDWK---------LR---
gi|727103490|ref|WP_033618954.1|restrictionendonucleaseEco57I[Helicobacterpylori]/11616                                              ----QAIPNQRLKGSLFEKVCKRFLEEHD-SANEYES-IELWSDWE---------LR---
gi|727340205|ref|WP_033794408.1|restrictionendonucleaseEco57I[Helicobacterpylori]/11609                                              ----QAIPNQRLKGSLFEKVCKRFLEEHD-SANEYES-IKLWSDWE---------LR---
gi|727292407|ref|WP_033749975.1|restrictionendonucleaseEco57I[Helicobacterpylori]/11611                                              ----QAIPNQRLKGSLFEKVCKRFLEEHD-SANEYES-IKLWSDWE---------LR---
gi|727289795|ref|WP_033747591.1|restrictionendonucleaseEco57I[Helicobacterpylori]/11612                                              ----HAIPNQRHKGSLFEKISKQFLQEHD-SANEYES-IDLWYDWE---------LR---
gi|727319676|ref|WP_033775047.1|restrictionendonucleaseEco57I[Helicobacterpylori]/11612                                              ----HAIPNQRHKGSLFEKVCKRFLEEHD-SANEYES-IELWSDWE---------LR---
gi|447212910|ref|WP_001290166.1|restrictionendonucleaseEco57I[Helicobacterpylori]_gi|308063540|gb|ADO05427.1|hypotheticalprotei       ----QAIPNQRLRGSWFEKVSRRFLIEHD-SANEYES-IELWSDWE---------LR---
gi|447212901|ref|WP_001290157.1|restrictionendonucleaseEco57I[Helicobacterpylori]_gi|384557329|gb|AFH97797.1|hypotheticalprotei       ----QAIPNQRLKGSWFEKVSRRFLIEHD-SANEYES-IELWSDWE---------LR---
gi|515849671|ref|WP_017280424.1|hypotheticalprotein[Helicobacterpylori]/11615                                                        ----QAIPNQRLKGSWFEKVSKRFLIEHD-SADEYES-IDLWNDWE---------LR---
gi|447212900|ref|WP_001290156.1|restrictionendonucleaseEco57I[Helicobacterpylori]_gi|317178970|dbj|BAJ56758.1|TypeIIGrestrictio       ----QAIPNQRLKGSWFEKVSKRFLKEHD-SADEYES-IDLWSDWK---------LN---
gi|447079018|ref|WP_001156274.1|hypotheticalprotein[Helicobacterpylori]/11614                                                        ----QAIPNLRHKGSWFEKISKQFLQEHD-SANEYES-IDLWNDWE---------LR---
gi|447212800|ref|WP_001290056.1|hypotheticalprotein[Helicobacterpylori]_gi|532153248|gb|EQL67316.1|restrictionendonucleaseEco57       ----QAIPPN-QKGSLFEKISKRFLIEHD-SANEYES-IDLWNDWE---------LR---
gi|505457237|ref|WP_015644339.1|hypotheticalprotein[Helicobacterpylori]_gi|499061160|gb|AGL70074.1|restrictionendonucleaseEco57       ----QAIPPN-QKGSLFEKISRRFLIEHD-SANEYES-IDLWNDWE---------LR---
HPYUM037X/11591                                                                                                                       ----QAIPPN-QKGSLFEKISRRFLIEHD-SANEYES-IDLWNDWE---------LR---
gi|920026297|ref|WP_052937176.1|restrictionendonuclease[Helicobacterpylori]_gi|912528678|gb|KNX48238.1|restrictionendonucleaseE       ----QAIPPN-QKGSLFEKISRRFLIEHD-SANEYES-IDLWNDWA---------LR---
gi|915539050|ref|WP_050841327.1|restrictionendonuclease[Helicobacterpylori]_gi|908890887|gb|KNE06183.1|restrictionendonucleaseE       ----HAIPNLRHKGSLFEKISKQFLQEHD-SANEYES-IELWGDWK---------LR---
gi|727101397|ref|WP_033616957.1|restrictionendonucleaseEco57I[Helicobacterpylori]/11592                                              ----QAIPNSRHKGSLFEKLSKRFLLEHD-SANEYES-IDLWSDFK---------LR---
gi|562814875|emb|CDK13002.1|TypeISPRMsystem[Helicobacterpylori26695]/11588                                                           ----QAIPNLRHKGILFEKISKQFLQEHD-SANEYES-IDLWYDWK---------LR---
HPYAXVIII/11588                                                                                                                       ----QAIPNLRHKGILFEKISKQFLQEHD-SANEYES-IDLWYDWK---------LR---
gi|447079029|ref|WP_001156285.1|restrictionendonucleaseEco57I[Helicobacterpylori]_gi|393085956|gb|EJB86635.1|hypotheticalprotei       ----QAIPNLRHKGSLFEKISRRFLQEHD-SANEYES-IDLWNDWE---------LR---
gi|489105953|ref|WP_003015814.1|restrictionendonuclease[Helicobacterpylori]_gi|477503388|gb|ENH59950.1|restrictionendonucleasef       ----QAIPNSHDKGSWFEKVSKHFLLEHD-SANEYES-IKLWNDWE---------LR---
gi|447079006|ref|WP_001156262.1|restrictionendonucleaseEco57I[Helicobacterpylori]_gi|393067470|gb|EJB68280.1|hypotheticalprotei       ----HAIPNLRHKGSLFEKISKQFLQEHD-SANEYES-IDLWYDWE---------LR---
gi|727314589|ref|WP_033770298.1|restrictionendonucleaseEco57I[Helicobacterpylori]/11586                                              ----QAIPNLRHKGSLFEKISKQFLQEHD-SANEYES-IKLWNDWE---------LR---
gi|447079019|ref|WP_001156275.1|restrictionendonucleaseEco57I[Helicobacterpylori]_gi|308065028|gb|ADO06920.1|hypotheticalprotei       ----HAIPNQRHKGSLFEKLSKRFLQEHD-SANEYES-IDLWYDWE---------LR---
gi|447079028|ref|WP_001156284.1|restrictionendonucleaseEco57I[Helicobacterpylori]_gi|393076030|gb|EJB76784.1|hypotheticalprotei       ----QAIPNLRHKGSLFEKISKQFLQEHD-SANEYES-IDLWYDWE---------LR---
gi|393034009|gb|EJB35070.1|hypotheticalproteinHPNQ4053_0631[HelicobacterpyloriNQ4053]/11514                                          ----HAIPNKRHKGSLFEKISKQFLQEHD-SANEYES-IDLWYDWE---------LR---
gi|447079004|ref|WP_001156260.1|restrictionendonucleaseEco57I[Helicobacterpylori]_gi|384552418|gb|AFI07366.1|hypotheticalprotei       ----HAIPNLRHKGSLFEKISKQFLLEHD-SANEYES-IDLWYDWE---------LR---
gi|746011306|ref|WP_039088215.1|restrictionendonucleaseEco57I[Helicobacterpylori]_gi|733488720|gb|KHL85979.1|restrictionendonuc       ----HAIPNLRHKGSLFEKISKQFLQEHD-SANEYES-IDLWYDWK---------LR---
gi|531081162|gb|EQD92166.1|restrictionendonucleaseEco57I[HelicobacterpyloriPZ5080]/11509                                             ----HAIPNSRHKGSLFEKISKQFLQEHD-SANEYES-IDLWYDWE---------LR---
gi|544760796|ref|WP_021187395.1|restrictionendonucleaseEco57I[Helicobacterpylori]_gi|531086508|gb|EQD96745.1|restrictionendonuc       ----HAIPNLRHKGSLFEKISKQFLQEHD-SANEYES-IDLWGDWK---------LR---
gi|447079012|ref|WP_001156268.1|restrictionendonucleaseEco57I[Helicobacterpylori]_gi|393028199|gb|EJB29286.1|hypotheticalprotei       ----HAIPNQRHKGSLFEKISKQFLQEHD-SANEYES-IDLWYDWE---------LR---
gi|746008458|ref|WP_039085555.1|restrictionendonucleaseEco57I[Helicobacterpylori]_gi|733480358|gb|KHL78026.1|restrictionendonuc       ----HAIPNLRHKGSLFEKISKQFLQEHD-SANEYES-IKLWSDWE---------LR---
gi|447079011|ref|WP_001156267.1|restrictionendonucleaseEco57I[Helicobacterpylori]_gi|393038534|gb|EJB39568.1|hypotheticalprotei       ----QAIPNLRHKGSLFEKISKQFLLEHD-SANEYES-IDLWYDWK---------LR---
gi|393037163|gb|EJB38199.1|hypotheticalproteinHPNQ4161_0422[HelicobacterpyloriNQ4161]/11556                                          ----HAIPNQRHKGSLFEKISKQFLQEHD-SANEYES-IDLWYDWE---------LR---
gi|447079007|ref|WP_001156263.1|restrictionendonucleaseEco57I[Helicobacterpylori]_gi|393051817|gb|EJB52768.1|hypotheticalprotei       ----HAIPNLRHKGSLFEKISKQFLQEHD-SANEYES-IDLWYDWE---------LR---
gi|487867094|ref|WP_001940560.1|helicase[Helicobacterpylori]_gi|459505657|gb|EMG98418.1|helicaseprotein[HelicobacterpyloriGAM21       ----HAIPNLRHKGSFFEKISKQFLQEHD-SANEYES-IDLWYDWE---------LR---
gi|487872094|ref|WP_001945560.1|helicase[Helicobacterpylori]_gi|459512104|gb|EMH04523.1|helicaseprotein[HelicobacterpyloriGAM24       ----HAIPNLRHKGSLFEKISKQFLQEHD-SANEYES-IDLWYDWK---------LR---
gi|487880228|ref|WP_001953694.1|helicase[Helicobacterpylori]_gi|459529131|gb|EMH20734.1|helicaseprotein[HelicobacterpyloriGAM26       ----HAIPNLRHKGSLFEKISKQFLQEHD-SANEYES-IDLWYDWE---------LR---
```

```
gi|487882593|ref|WP_001956059.1|helicase[Helicobacterpylori]_gi|459533462|gb|EMH24859.1|helicaseprotein[HelicobacterpyloriGAM26      ----HAIPNLRHKGSLFEKISKQFLQEHD-SANEYES-IDLWYDWK---------LR---
gi|487845430|ref|WP_001918896.1|helicase[Helicobacterpylori]_gi|410713871|gb|EKQ71363.1|helicaseprotein[HelicobacterpyloriGAM10      ----HAIPNLRHKGSLFEKISKQFLQEHD-SANEYES-IDLWYDWE---------LR---
gi|447079005|ref|WP_001156261.1|restrictionendonucleaseEco57I[Helicobacterpylori]_gi|393069830|gb|EJB70625.1|hypotheticalprotei      ----HAIPNLRHKGSLFEKISKQFLQEHD-SANEYES-IDLWYDWE---------LR---
gi|459506808|gb|EMG99528.1|helicaseprotein[HelicobacterpyloriGAM121Aii]/11545      ----HAIPNLRHKGSLFEKISKQFLQEHD-SANEYES-IDLWYDWE---------LR---
gi|487825283|ref|WP_001898749.1|restrictionendonucleaseEco57I[Helicobacterpylori]_gi|393107526|gb|EJC08068.1|hypotheticalprotei      ----HAIPNQRHKGSLFEKISKQFLQEHD-SANEYES-IDLWYDWE---------LR---
gi|447079008|ref|WP_001156264.1|restrictionendonucleaseEco57I[Helicobacterpylori]_gi|393058636|gb|EJB59524.1|hypotheticalprotei      ----HAIPNLRHKGSLFEKISKQFLQEHD-SANEYES-IDLWYDWE---------LR---
gi|447079015|ref|WP_001156271.1|restrictionendonucleaseEco57I[Helicobacterpylori]_gi|307637357|gb|ADN79807.1|hypotheticalprotei      ----HAIPNQRHKGSLFEKISKQFLQEHD-SANEYES-IDLWYDWK---------LR---
gi|487884861|ref|WP_001958327.1|helicase[Helicobacterpylori]_gi|459535535|gb|EMH26834.1|helicaseprotein[HelicobacterpyloriGAM26      ----HAIPNLRHKGSLFEKISKQFLQEHD-SANEYES-IDLWYDWE---------LR---
gi|487886365|ref|WP_001959831.1|helicase[Helicobacterpylori]_gi|459538727|gb|EMH29861.1|helicaseprotein[HelicobacterpyloriGAM27      ----HAIPNLRHKGSLFEKISKQFLQEHD-SANEYES-IDLWYDWE---------LR---
gi|545301637|ref|WP_021581412.1|helicase[Helicobacterpylori]_gi|535931889|gb|ERA61745.1|helicaseprotein[HelicobacterpyloriGAM11      ----HAIPNQRHKGSLFEKISKQFLQEHD-SANEYES-IDLWYDWE---------LR---
gi|447079010|ref|WP_001156266.1|restrictionendonucleaseEco57I[Helicobacterpylori]_gi|393095477|gb|EJB96081.1|hypotheticalprotei      ----HAIPNLRHKGSLFEKISKQFLQEHD-SANEYES-IDLWYDWK---------LR---
gi|447079009|ref|WP_001156265.1|restrictionendonucleaseEco57I[Helicobacterpylori]_gi|393056294|gb|EJB57206.1|hypotheticalprotei      ----HAIPNLRHKGSLFEKISKQFLQEHD-SANEYES-IDLWYDWK---------LR---
gi|471386985|gb|EMR58906.1|restrictionendonucleasefamilyprotein[HelicobacterpyloriHpH1]/11504      ----HAIPNLRHKGSLFEKISKQFLQEHD-SANEYES-IDLWYDWK---------LR---
gi|487875679|ref|WP_001949145.1|helicase[Helicobacterpylori]_gi|459516928|gb|EMH09091.1|helicaseprotein[HelicobacterpyloriGAM25      ----HAIPNQRHKGSLFEKISKQFLQEHD-SANEYES-IDLWYDWQ---------LR---
gi|496130066|ref|WP_008854573.1|DNAdamageinducibleprotein[Commensalibacterintestini]_gi|353671689|gb|EHD13391.1|helicasedomain/      -TK-----TERDKGTSFENLMVQYFKTEPVYKSQYKD-VLSYANWV---------DQYGN
gi|924898894|ref|WP_053556546.1|damageinducibleprotein[Sphingopyxissp.113P3]_gi|924523996|gb|ALC14732.1|damageinducibleprotei/1      -AR-----SQREKGTYFERLTKIWLEHAPTQAGRYAR-VLTYTDFG---------RERG-
gi|780802950|gb|KJS08562.1|damageinducibleprotein[Hoefleasp.BRH_c9]/11633      -SR-----GEREKGTYFENWAKVFFENDPQYTQRFDK-VWTFSEWA---------AERG-
gi|944671432|gb|KQN22886.1|damageinducibleprotein[Sphingomonassp.Leaf32]/11415      -AK-----SQREKGTYFERLCTEFLKHDPELAQQYED-AWTFRDWA---------EARG-
gi|944612953|gb|KQM64772.1|damageinducibleprotein[Sphingomonassp.Leaf16]/11726      -AK-----SQREKGTYFERLCTEFLKHDPELAQQYED-AWTFRDWA---------EARG-
gi|764353851|ref|WP_044331468.1|damageinducibleprotein[Sphingomonashengshuiensis]_gi|761897338|gb|AJP71707.1|damageinduciblep/1      -AQ-----TEREKGNYFERLAVAFAKNDHGMMQEYED-AWLYSEWA---------AAHN-
gi|945874472|gb|KQZ27973.1|damageinducibleprotein[Mesorhizobiumsp.Root552]/11651      -AR-----TEREKGNYFERLAVDFIKNDPGMAQEYED-AWLFSDWA---------ASHG-
gi|759408081|ref|WP_043132403.1|damageinducibleprotein[Paracoccussanguinis]_gi|694217086|gb|KGJ14281.1|damageinducibleprotein/1      -AR-----SEREKGDYFERVVRVFLEHDDAQKQYYSA-VVPFAQWA---------KEQG-
gi|654342671|ref|WP_027836116.1|damageinducibleprotein[Maritaleamyrionectae]/11632      -ST-----NEREKGDYFERVVKVYLENDDVQKQYYSA-VETYSEWA---------KKQG-
gi|126717687|gb|EBA14409.1|hypotheticalproteinRSK20926_02032[Roseobactersp.SK20926]/11603      -AR-----TEREKGDYFEKLVRVFLENDDTQKQFYSK-VITFADWA---------GAQG-
gi|765301116|ref|WP_044628780.1|damageinducibleprotein[Halomonasmeridiana]_gi|764089679|gb|KJD19875.1|damageinducibleprotein[/1      -ST-----NEREKGDYFEKLVRVFLEHDDTQKQFYSS-VVPFSVWA---------KEQG-
gi|518053006|ref|WP_019223214.1|DNAdamageinducibleprotein[Bartonellarattaustraliani]/11654      -AT-----NPRERGTVFEKLVKTYLTQDPIQKQEYET-VQTYCEWA---------RERGE
gi|493637806|ref|WP_006589578.1|DNAdamageinducibleprotein[Bartonellabirtlesii]_gi|395410345|gb|EJF76900.1|hypotheticalproteinM/      -AK-----SPRELGTLFENLVMAYLMHDPLHFGRYET-VESYYEWA---------TEREG
gi|501188603|ref|WP_012231622.1|DNAdamageinducibleprotein[Bartonellatribocorum]_gi|161017843|emb|CAK01401.1|helicase/methyltra/      -AT-----SNALRGKVFEDFVTKYLMHDPLHYGRYEK-VESYYEWA---------KERED
gi|651388616|ref|WP_026500615.1|damageinducibleprotein[Bartonellagrahamii]/11655      -AK-----SPRELGTLFENLVMAYLMHDPLHYGRYEK-VETYYEWA---------EEREG
gi|501188669|ref|WP_012231687.1|DNAdamageinducibleprotein[Bartonellatribocorum]_gi|161017922|emb|CAK01480.1|helicase/methyltra/      -AK-----SLRELGTLFENLVIAYLTKDPLHYGRYEK-VETYYEWA---------KEREG
gi|518048832|ref|WP_019219040.1|DNAdamageinducibleprotein[Bartonellaflorencae]/11655      -AK-----SPRELGTLFENLVMAYLMHDPLHFGRYET-VESYYEWA---------TEREG
gi|518053617|ref|WP_019223825.1|DNAdamageinducibleprotein[Bartonellarattaustraliani]/11607      -AT-----NPRERGTLFENLVKTYLTWDPIQKQEYET-VQTYCEWA---------KERGE
gi|506336449|ref|WP_015856168.1|DNAdamageinducibleprotein[Bartonellagrahamii]_gi|240267492|gb|ACS51080.1|helicase/methyltransf/      -AK-----SPRELGTLFENLVMAYLMHDPLHFGRYET-VESYYEWA---------KEREG
gi|494588832|ref|WP_007347427.1|DNAdamageinducibleprotein[Bartonellarattimassiliensis]_gi|395419053|gb|EJF85361.1|hypothetical/      -AK-----SPRELGTLFENLVKVYLAEDPLQKQEYEK-VQTYLEWA---------KEHDE
gi|501188650|ref|WP_012231668.1|DNAdamageinducibleprotein[Bartonellatribocorum]_gi|161017902|emb|CAK01460.1|helicase/methyltra/      -AQ-----SPRELGTMFENLVKVYLAEDPLQCQEYEK-VQTYLEWA---------QEHDE
gi|501188624|ref|WP_012231643.1|DNAdamageinducibleprotein[Bartonellatribocorum]_gi|161017866|emb|CAK01424.1|helicase/methyltra/      -AI-----SEQEKQAAFEKFVIAYLTQDPLQCQEYEK-VQTYREVA---------DEKG-
gi|518053856|ref|WP_019224064.1|DNAdamageinducibleprotein[Bartonellarattaustraliani]/11625      -AI-----SEQEKQAAFEKFVIAYLTWDPLQKQEYEK-IQTYREVA---------DEKG-
gi|501188053|ref|WP_012231081.1|DNAdamageinducibleprotein[Bartonellatribocorum]_gi|161017434|emb|CAK00992.1|helicase/methyltra/      -AI-----SEQEKQAAFEKFVIAYLTQDPLQCQEYEK-VQTYREVA---------DEKG-
gi|651388630|ref|WP_026500626.1|damageinducibleprotein[Bartonellagrahamii]/11657      -AK-----SPRELGTLFENLIMAYLTKDPLHYGRYEK-VETYYEWA---------KEHEG
gi|518048855|ref|WP_019219063.1|DNAdamageinducibleprotein[Bartonellaflorencae]/11658      -AK-----SPRELGTMFENLIMAYLTNDPLQKQEYEK-VQTYLEWA---------KEHGE
gi|490995101|ref|WP_004856829.1|DNAdamageinducibleprotein[Bartonelladoshiae]_gi|395413167|gb|EJF79645.1|hypotheticalproteinMCS/      -AK-----SPRELGTLFENLVKAYLSEDPLQKQEYEK-VQTYLEWA---------KEHDE
gi|491002558|ref|WP_004864279.1|DNAdamageinducibleprotein[Bartonellavinsonii]_gi|395421392|gb|EJF87643.1|hypotheticalproteinME/      -TG-----GARDRGTSFEKFVIAYLTHDPLQCQEYEK-VQTYYDWA---------CEHD-
gi|491003373|ref|WP_004865094.1|DNAdamageinducibleprotein[Bartonellavinsonii]_gi|395432864|gb|EJF98838.1|hypotheticalproteinME/      -TG-----GARDRGTSFEKFVIAYLTHDPLQCQEYEK-VQTYYDWA---------REHD-
gi|491002426|ref|WP_004864148.1|DNAdamageinducibleprotein[Bartonellavinsonii]_gi|395421767|gb|EJF88003.1|hypotheticalproteinME/      -AK-----SPRELGTLFENLVMAYLTQDPLQCQEYEQ-VQTYCDWA---------KERG-
gi|491004024|ref|WP_004865745.1|DNAdamageinducibleprotein[Bartonellavinsonii]_gi|395432542|gb|EJF98522.1|hypotheticalproteinME/      -AK-----SPRELGTLFENLVMAYLTQDPLQCQEYEQ-VQTYCDWA---------KERG-
gi|648396442|ref|WP_026088193.1|damageinducibleprotein[Bartonellarattaustraliani]/11593      -AT-----NPRERGTVFENLVKTYLTWDPIQKQEYET-VQTYCEWA---------KERG-
gi|748190174|ref|WP_039761290.1|damageinducibleprotein[Bartonellaqueenslandensis]/11658      -AT-----NPRERGTLFENLVKTYLTQDPIQKQEYET-VQTYCEWA---------RERG-
gi|494817599|ref|WP_007553007.1|DNAdamageinducibleprotein[Bartonellasp.DB56]_gi|395411312|gb|EJF77837.1|hypotheticalproteinMC/1      -TE-----GARDRGTSFEKFVIAYLTQDPLQKQEYEK-VQTYRDWA---------REHG-
gi|515763376|ref|WP_017195976.1|DNAdamageinducibleprotein[Bartonellabirtlesii]/11656      -TE-----GARDRGTSFEKFVIAYLTQDPLQCQEYEK-VQTYRDWA---------REHG-
gi|494589420|ref|WP_007348014.1|DNAdamageinducibleprotein[Bartonellarattimassiliensis]_gi|395416278|gb|EJF82667.1|hypothetical/      -AK-----SPRELGTLFENLVMDYLMHDPLQKQEYEK-VQTYYEWA---------KEHD-
gi|506336469|ref|WP_015856188.1|DNAdamageinducibleprotein[Bartonellagrahamii]_gi|240267512|gb|ACS51100.1|helicase/methyltransf/      -AK-----SPRELGTMFENLIMAYLTNDPLQKQEYEK-VQTYLEWA---------KEHG-
gi|749308087|ref|WP_040132227.1|damageinducibleprotein[Bartonellatribocorum]_gi|605051878|emb|CDO49988.1|helicase/methyltransf/      -AK-----SPRELGTMFENLVMVYLSEDPLQKQEYEK-VQTYLEWA---------QEHD-
gi|515763800|ref|WP_017196400.1|DNAdamageinducibleprotein[Bartonellabirtlesii]/11658      -AK-----SPRELGTLFENLVKAYLTNDPLQKQEYEK-VQTYCEWA---------KEHD-
gi|492186994|ref|WP_005773380.1|DNAdamageinducibleprotein[Bartonellaelizabethae]_gi|395418361|gb|EJF84688.1|hypotheticalprotei/      -AK-----SPRELGTLFENLVKTYLSEDPLQKQEYEK-VQTYSEWA---------EDHD-
gi|501188615|ref|WP_012231634.1|DNAdamageinducibleprotein[Bartonellatribocorum]_gi|161017856|emb|CAK01414.1|helicase/methyltra/      -AK-----SPRELGTLFENLVKVYLAEDPLQCQEYEK-VQTYLEWA---------KEHD-
gi|501187470|ref|WP_012230499.1|DNAdamageinducibleprotein[Bartonellatribocorum]_gi|161017090|emb|CAK00648.1|helicase/methyltra/      -AK-----SPRELGTLFENLVMVYLSEDPLQKQEYEK-VQTYLEWA---------KEHD-
gi|501188636|ref|WP_012231655.1|DNAdamageinducibleprotein[Bartonellatribocorum]_gi|161017879|emb|CAK01437.1|helicase/methyltra/      -AK-----SPRELGTLFENLVMVYLSEDPLQKQEYEK-VQTYLEWA---------KEHD-
gi|930151941|emb|CTQ34818.1|UvsWhelicase[Jannaschiarubra]/11641      -AR-----TEREKGTYFERIAAVYLRHDPIQAALFSK-VETYEEWA---------KGLG-
gi|928977106|ref|WP_054001461.1|damageinducibleprotein[Shimiasp.SK013]_gi|927698852|gb|KPA22698.1|typeIrestrictionenzymeEcoKIs/      -AV-----TEREKGTYFERLALAYLRNDPIQTQQFSE-VWTYGDWA---------KENG-
gi|738872465|ref|WP_036760884.1|damageinducibleprotein[Leisingeradaeponensis]/11643      -AR-----TEREKGTYFERLCVAYLTHDPMQAEHYED-VLSWTDWA---------KENG-
gi|518050881|ref|WP_019221089.1|DNAdamageinducibleprotein[Bartonellasenegalensis]/11652      -AR-----TERDKGTYFERFALAYLTHDPLQLDQYEK-VQTFKDWA---------HENG-
gi|493980596|ref|WP_006923523.1|DNAdamageinducibleprotein[Bartonellawashoensis]_gi|395413322|gb|EJF79796.1|hypotheticalprotein/      -AR-----TERDKGSYFERFALAYLTHDPLQFGCYEK-VQTFKDWA---------LENG-
gi|493983106|ref|WP_006925984.1|DNAdamageinducibleprotein[Bartonellawashoensis]_gi|395418127|gb|EJF84455.1|hypotheticalprotein/      -AR-----TERDKGSYFERFALAYLTHDPLQCEQYEK-VQTFKDWA---------HEND-
gi|499494671|ref|WP_011181311.1|DNAdamageinducibleprotein[Bartonellahenselae]_gi|49239008|emb|CAF28308.1|hypotheticalproteinBH/      -AR-----TERDKGTYFERFALAYLMHDPLQFDQYEK-VQTFKDWA---------YENG-
gi|651388426|ref|WP_026500441.1|damageinducibleprotein[Bartonellagrahamii]/11652      -AR-----TERDKGTYFERFALAYLTHDPLQFGCYEK-VQTFRDWA---------REND-
gi|506337280|ref|WP_015856999.1|DNAdamageinducibleprotein[Bartonellagrahamii]_gi|240268402|gb|ACS51990.1|helicase/methyltransf/      -AR-----TERDKGTYFERFALAYLTHDPLQSGCYEK-VQTFRDWA---------HEND-
gi|494817535|ref|WP_007552943.1|DNAdamageinducibleprotein[Bartonellasp.DB56]_gi|395412002|gb|EJF78518.1|hypotheticalproteinMC/1      -AR-----TERDKGTYFERFALAYLTHDPLQFDQYEK-VQTFKDWA---------HEND-
```

```
gi|493638099|ref|WP_006589864.1|DNAdamageinducibleprotein[Bartonellabirtlesii]_gi|395409809|gb|EJF76394.1|hypotheticalproteinM/    -AR-----TERDKGTYFERFALAYLRHDPLQFDQYEK-VQTFKDWA---------HENG-
gi|748189589|ref|WP_039760707.1|damageinducibleprotein[Bartonellaqueenslandensis]/11651    -AR-----TERDKGTYFERLALAYLTHDPLQSLCYEK-VQTFKDWA---------HENG-
gi|501189483|ref|WP_012232501.1|DNAdamageinducibleprotein[Bartonellatribocorum]_gi|161018902|emb|CAK02460.1|helicase/methyltra/    -AR-----TERDKGTYFERLALAYLTHDPLQSQCYEK-VQTFKDWA---------HENG-
gi|518049181|ref|WP_019219389.1|DNAdamageinducibleprotein[Bartonellaflorencae]/11652    -AR-----TERDKGTYFERFVLAYLTHDPLQSQCYEK-VQTFKDWA---------HENG-
gi|492193255|ref|WP_005774528.1|DNAdamageinducibleprotein[Bartonellaelizabethae]_gi|395416403|gb|EJF82779.1|hypotheticalprotei/    -AR-----TERDKGTYFERLALAYLTHDPLQSQCYEK-VQTFKDWA---------HENG-
gi|498387325|ref|WP_010701288.1|helicase/methyltransferase[Bartonellabovis]_gi|478418724|gb|ENN92426.1|helicase/methyltransfera    -AR-----TQRDKGTYFERFALAYLTHDPLQRREYEN-IQTFKDWA---------KENG-
gi|319408697|emb|CBI82352.1|conservedhypotheticalprotein[BartonellaschoenbuchensisR1]/11661    -AR-----TQRDKGTYFERLALTYLTHDPAQCDEYEN-VQTFKDWA---------KENG-
gi|494741726|ref|WP_007477134.1|DNAdamageinducibleprotein[Bartonellamelophagi]_gi|395423925|gb|EJF90113.1|hypotheticalproteinM/    -AR-----TQRDKGTYFERLAIAYLMHDPLQRKEYEN-VQTFKDWA---------KENG-
gi|737261405|ref|WP_035245349.1|damageinducibleprotein[Alcanivoraxjadensis]_gi|686989709|gb|KGD62556.1|hypotheticalproteinT9A_/    -AK-----TDREMGEYFELFVKAYFEHDDIQRQHYDQ-VWTYGDWA---------EEHG-
gi|916687564|ref|WP_051294655.1|damageinducibleprotein[Gemmobacternectariphilus]/11641    -AR-----TEREKGTYFERLATAYLTTDPIQAALYDD-VWAYAEWA---------KAQG-
gi|740294127|ref|WP_038132495.1|damageinducibleprotein[Thioclavasp.DT234]_gi|660524826|gb|KEO54689.1|hypotheticalproteinDT23_/1    -AV-----TEREKGTYFERLTCAYLTVDPVQAEEFSE-VWTWKDWA---------DQHG-
gi|568331062|ref|WP_024099615.1|helicase[Phaeobactergallaeciensis]_gi|568232121|gb|AHD12007.1|putativehelicase[Phaeobactergalla    -AR-----TERDKGTYFERLSIAYLTNDPVQVEQYED-VKPYAAWA---------IEQG-
gi|518810459|ref|WP_019966413.1|DNAdamageinducibleprotein[Pannonibacterphragmitetus]/11635    -AV-----TEREKGTYFERLALAFFQNDPVQSEEYEA-VWTWSEWA---------KANG-
gi|495305760|ref|WP_008030510.1|DNAdamageinducibleprotein[Rhodobactersp.SW2]_gi|259021698|gb|EEW25000.1|typeIIIrestrictionprot/    -AV-----TEREKGTYFERLALVFFMNDPVQSEEYEA-VWTWSDWA---------KTNG-
gi|506403303|ref|WP_015923022.1|DNAdamageinducibleprotein[Rhodobactersphaeroides]_gi|221162951|gb|ACM03922.1|HypotheticalProte/    -AV-----TEREKGTYFERLALAYFQNDPVQSEEYEA-VWTWSEWA---------KANG-
gi|738818386|ref|WP_036708790.1|damageinducibleprotein[Paracoccussanguinis]_gi|694226786|gb|KGJ22541.1|damageinducibleprotein/1    -AV-----TEREKGTYFERLALAYFQNDPVQSEEYEA-VWTWSEWA---------KANG-
gi|746096057|ref|WP_039162453.1|damageinducibleprotein[Gallibacteriumanatis]_gi|703617775|gb|KGQ63868.1|damageinducibleprotei/1    -SL-----TEREKGTYFEYLTIQYLTHEPAYRDLYQE-VLTFADWV---------EKYQP
gi|737716785|ref|WP_035685631.1|damageinducibleprotein[Avibacteriumparagallinarum]/11643    -SL-----TEREKGTYFEYLTIQYLTHEPAYRDLYQE-VLTFADWV---------EKYQP
gi|540072553|gb|ERF78672.1|hypotheticalproteinN561_04930[Gallibacteriumanatis12656/12]/11651    -SL-----TEREKGTYFEYLTIQYLTHEPAYLDLYQE-VLTFADWV---------EKYQP
gi|917509519|ref|WP_052115936.1|damageinducibleprotein[Gallibacteriumanatis]_gi|702414161|gb|KGQ42233.1|damageinducibleprotei/1    -SV-----TEREKGTYFEYLTIQYLTHEPAYRDLYQE-VLTFADWV---------EKYQP
gi|746082942|ref|WP_039150268.1|damageinducibleprotein[Gallibacteriumanatis]_gi|703617078|gb|KGQ63194.1|damageinducibleprotei/1    -SL-----TEREKGTYFEYLTIQYLTHEPAYHDLYQE-VLTFADWV---------EKYQP
gi|822522885|ref|WP_046952840.1|damageinducibleprotein[Haemophilushaemolyticus]_gi|821501762|gb|KKZ59267.1|damageinduciblepro/1    --------------------MQLYFQNEPFYSVYYKE-VLTFKDWI---------EKYYP
gi|404381014|gb|EJZ77501.1|putativehelicase[Pasteurellamultocidasubsp.gallicidaP1059]/11681    -SL-----TEREKGTYFEDLIQLYLQHEPFYKDLYQE-ILTFKQWI---------EKYQP
gi|298281660|gb|EFI23149.1|helicaseCterminaldomainprotein[Neisseriasp.oraltaxon014str.F0314]/11493    -TQNAAYQGRRDQGTAFEHLMVAYFQTEPCYKELYQN-VQPYGTWA---------AKHLN
gi|348013055|gb|EGY51980.1|helicasedomainprotein[Neisseriashayeganii871]/11481    -TQ-----TERDKGTAFEKLMIRYFEHEPYYRSQYAK-VQTYAEWV---------QEHFE
gi|496407140|ref|WP_009116004.1|DNAdamageinducibleprotein[Neisseriawadsworthii]_gi|349794756|gb|EGZ48566.1|helicasedomainprote/    -TQ-----TERDKGTAFENLIIRYFEHEPYYRSQYAK-VQTYAEWA---------QEHFE
gi|517216441|ref|WP_018405259.1|DNAdamageinducibleprotein[Marinobacterlipolyticus]/11656    -TL-----SERDKGTSFENLMVQYFKTEPFYKEQYAD-VLSYSGWV---------DQYGA
gi|738230264|ref|WP_036185691.1|damageinducibleprotein[Marinobactersp.ELB17]/11671    -TL-----SERDKGTSFENLMVQYFKTEPAYKQQYAD-VLSYSGWV---------EQYGA
gi|447197741|ref|WP_001274997.1|DNAdamageinducibleprotein[Acinetobacterbaumannii]_gi|408512324|gb|EKK13969.1|typeIIrestrictio/    -TL-----TNRDKGTSFENLMVQYFLNEPKYAEIYTE-VLSYSDWV---------EKYGE
gi|820775897|ref|WP_046739909.1|damageinducibleprotein[Acinetobactersp.AG1]_gi|819909758|gb|KKW75938.1|damageinducibleprotein/1    -TL-----TNRDKGTSFENLMVQYFLNEPKYAELYTE-VLSYADWV---------EKYGE
gi|584595163|ref|WP_024160846.1|helicase[Acinetobacterpittii]_gi|576702530|emb|CDI28049.1|helicasedomaincontainingprotein[Acin/    -AL-----TNRDKGTSFENLMVQYFLNEPKYAEIYTE-VLSYSDWV---------EKYGE
gi|514963042|ref|WP_016651366.1|hypotheticalprotein[Acinetobactersp.NIPH2036]_gi|514162665|gb|EPG41391.1|hypotheticalproteinF90    -SL-----TNRDKGTSFENLMIQYFLNEPKYAEIYTE-VLSYSDWV---------EKYGE
gi|515965564|ref|WP_017396147.1|DNAdamageinducibleprotein[Acinetobacterhaemolyticus]/11667    -TL-----TNRDKGTSFENLMVQYFLNEPKYAELYTE-VLSYADWV---------EKYGE
gi|491346810|ref|WP_005204747.1|MULTISPECIES:hypotheticalprotein[Acinetobacter]_gi|480335823|gb|ENX56033.1|hypotheticalproteinF    -TL-----TNRDKGTSFENLMVQYFLNEPKYAELYTE-VLSYADWV---------EKYGE
gi|693044472|ref|WP_032215268.1|damageinducibleprotein[Escherichiacoli]_gi|607725731|gb|EZE08230.1|damageinducibleprotein[Esc/1    -SP-----TQRDKGTTFENLCIQYFLNEPKYAELYSD-VLSYSEWS---------AQYGD
gi|742382177|ref|WP_038861304.1|damageinducibleprotein[Cronobactermuytjensii]_gi|924333747|gb|ALB69766.1|damageinducibleprote/1    -SP-----IQRDKGTSFENLCVQYFLHEPKYAELYSE-VLSYAGWI---------AQYGD
gi|915912804|ref|WP_050927035.1|damageinducibleprotein[Yersiniaenterocolitica]_gi|899805802|emb|CQH32042.1|typeIrestrictionenz/    -SP-----TQRDKGTTFENLCVQYFLHEPKYAELYSD-VLSYAGWI---------AQYGD
gi|921111327|gb|KON62781.1|typeIrestrictionenzymeEcoKIsubunitR[Komagataeibactereuropaeus]/11492    -SV-----TEREKGTYFEELIVCYLRTEPSYADLYDQ-VWTYKDWA---------T----
gi|582024278|gb|AHI27615.1|DNAhelicaserestrictionenzymeTypeIIIRsubunit[KomagataeibacterxylinusE25]/11636    -SV-----TEREKGTYFEELIVCYLRTEPSYADLYDQ-VWTYKDWA---------I----
gi|347662673|dbj|BAK86128.1|DNAhelicaserestrictionenzymeTypeIIIRsubunit[KomagataeibactermedellinensisNBRC3288]/11737    -SV-----TEREKGTYFEELIVCYLRTEPSYADLYDQ-VWTYKDWA---------T----
gi|917445053|ref|WP_052051471.1|damageinducibleprotein[Acetobactertropicalis]/11429    -AV-----TESEKGTYFEELVICYLRTEPSYVDLYDK-VWPYKRWA---------K----
gi|453329445|dbj|GAC88472.1|DNAhelicaserestrictionenzymetypeIIIRsubunit[GluconobacterthailandicusNBRC3255]_gi|530287981|dbj|GAD    -AV-----SESEKGTYFEELIVCYLRTEPSYADLYDM-VWPYKVWA---------G----
gi|889770922|ref|WP_048844278.1|damageinducibleprotein[Gluconobacterfrateurii]/11419    -AV-----SESEKGTYFEELIVCYLRTEPSYADLYDM-VWPYKVWA---------G----
gi|504886208|ref|WP_015073310.1|hypotheticalprotein[Gluconobacteroxydans]_gi|411027954|gb|AFW01209.1|hypotheticalproteinB932_16    -AV-----SESEKGTYFEELIVCYLRTEPSYADLYDM-VWPYKVWA---------G----
gi|480040211|gb|ENV79751.1|hypotheticalproteinF942_01641[AcinetobacterursingiiANC3649]/11432    -AK-----TQREKGNYFEDLMLRYFSNEPKYQELYNE-VFLWQGFR---------QYWLE
gi|822498222|ref|WP_046936504.1|damageinducibleprotein[Xanthomonasgardneri]_gi|821751224|gb|KLB07223.1|damageinducibleprotein/1    -AV-----SEREKGTYFEELILAYVRNEATYKDLYSQ-VWTWGDWA---------P----
gi|654059559|ref|WP_027705556.1|damageinducibleprotein[Zymobacterpalmae]/11635    -SK-----SEREKGTYFEELIVAYLRNEPAYKDRYSD-VWTWKEWA---------P----
gi|643535620|ref|WP_025224315.1|damageinducibleprotein[Methylophilaceaebacterium11]_gi|577704400|gb|EUJ10546.1|putativehelicas/    -SM-----SEREKGNYFEELIVQYFKNEASYQDLYSN-IWTYAAWA---------E----
MBA11I/11635    -SM-----SEREKGNYFEELIVQYFKNEASYQDLYSN-IWTYAAWA---------E----
gi|655355857|ref|WP_028762287.1|damageinducibleprotein[Shewanellacolwelliana]/11632    -SK-----TEREKGNYFEELIVTYFKNEPTYKDLYSK-VWLYADWA---------K----
gi|753204236|ref|WP_041510579.1|damageinducibleprotein[Shewanellasp.cp20]_gi|752457765|gb|KIO36326.1|damageinducibleprotein[S/1    -SR-----SEREKGTYFEELIQAYFKNEPTYADLYCD-VWMYKNWA---------K----
KPNNIH30III/11619    -SR-----SEREKGTYFEELVLSYLTNEPIYADLYSN-VWMYSDWA---------Q----
gi|749295994|ref|WP_040120352.1|damageinducibleprotein[Klebsiellapneumoniae]_gi|723249300|gb|AIX81926.1|damageinducibleprotei/1    -SR-----SEREKGTYFEELVLSYLTNEPIYADLYSN-VWMYSDWA---------Q----
gi|641639739|emb|CDQ54010.1|unnamedproteinproduct[Klebsiellapneumoniae]/11641    -SR-----SEREKGTYFEELVRSYLTNEPIYADLYSN-VWMYSDWA---------Q----
gi|490797922|ref|WP_004660060.1|hypotheticalprotein[Acinetobactersp.NIPH236]_gi|479876779|gb|ENU27973.1|hypotheticalproteinF992    -AK-----SEREKGTYFEALCIKYFENEPYYVDLFAQ-VQTYTDWA---------KA---
gi|916750926|ref|WP_051357982.1|damageinducibleprotein[Acinetobacterjernbergiae]/11453    -AK-----TEREKGTYFELLCIKYFKNEPFYADLFAD-VQTYTEWA---------KA---
gi|691098662|ref|WP_032025060.1|damageinducibleprotein[Acinetobacterbaumannii]_gi|593567521|gb|EXR50735.1|restrictionendonucle/    -AK-----TEREKGTYFELLCIKYFENEPFYADLFSK-VQTYSEWA---------KE---
gi|736669405|ref|WP_034676049.1|damageinducibleprotein[Acinetobacterbaumannii]/11643    -AK-----TEREKGTYFELLCIKYFSNEPFYADLFTE-VQTYTEWA---------KV---
gi|491368928|ref|WP_005226826.1|hypotheticalprotein[Acinetobactersp.NIPH1847]_gi|480173259|gb|ENX09797.1|hypotheticalproteinF89    -AK-----TEREKGTYFELLCIKYFLNEPFYADLYSE-VQPYSDWA---------KV---
gi|796853993|ref|WP_045795953.1|damageinducibleprotein[Acinetobacterindicus]_gi|787929761|gb|KJV44219.1|damageinducibleprotei/1    -AK-----TEREKGTYFELLCIKYFENEPFYADLFTK-IQTYSEWA---------KE---
gi|295797463|gb|ADG32253.1|typeIIIrestrictionproteinressubunit[ThiomonasintermediaK12]/11676    -AQ-----SEREKGFYFEKLVKAYLLAEPAWRDLFGGKVFLWEEWR---------TECRQ
gi|499871179|ref|WP_011551913.1|DNAdamageinducibleprotein[Myxococcusxanthus]_gi|108467544|gb|ABF92729.1|putativerestriction/mo/    -AA-----TNREQGTYFEELTVAFLKNEPAYRELYRS-VEPYAAWA---------ER---
gi|495313852|ref|WP_008038600.1|DNAdamageinducibleprotein[Bartonellatamiae]_gi|395424181|gb|EJF90368.1|hypotheticalproteinME5_/    -AV-----SEREKGTYFEELMIAYLKHEPFYADLYSD-VWTFKDWA---------LS---
gi|501453335|ref|WP_012476784.1|DNAdamageinducibleprotein[Leptospirabiflexa]_gi|167777824|gb|ABZ96123.1|Helicase[Leptospirabif/    -SK-----TEREKGTYFEELIQTYFRNEPYYKDYYSD-VWMYSDWA---------KL---
gi|738104613|ref|WP_036063193.1|damageinducibleprotein[Leptospiranoguchii]/11652    -AL-----TTREKGTYFEELVIAYLKNEPKYKDYYSD-VWMYSDWA---------SL---
gi|410001039|gb|EKO51661.1|typeIIIrestrictionenzyme,ressubunit[Leptospirakirschneristr.200802841]/11651    -AL-----TTREKGTYFEELVKAYLKNEPTYKDYYSD-VWMYSDWA---------DL---
gi|490901851|ref|WP_004763774.1|typeIIIrestrictionenzyme,ressubunit[Leptospirakirschneri]_gi|464209249|gb|EMN24014.1|typeIIIres    -AL-----TTREKGTYFEELVKAYLKNEPTYKDYYSD-VWMYSDWA---------DL---
```

```
gi|464398131|gb|EMO65902.1|typeIIIrestrictionenzyme,ressubunit[Leptospirakirschneristr.200803703]/11648        -AL-----TTREKGTYFEELVKAYLKNEPTYKDYYSD-VWMYSDWA---------DL---
gi|945029024|gb|KQQ84900.1|damageinducibleprotein[Xanthomonassp.Leaf131]/11423                                  -AL-----SEREKGTYFEELILAYLRTEATYRDLYS-QVWTWADWA---------PA---
gi|896511314|ref|WP_049418183.1|damageinducibleprotein[Stenotrophomonasmaltophilia]/11639                       -SQ-----TEREKGTYFEELIVAYLRHEASYRDLYS-NVWPWAQWA---------LA---
gi|896513082|ref|WP_049419616.1|damageinducibleprotein[Stenotrophomonasmaltophilia]/11638                       -SM-----TEREKGTYFEDLILAYLRNEATYRDLYS-KVQAWAEWA---------PA---
gi|896580616|ref|WP_049469425.1|damageinducibleprotein[Stenotrophomonasmaltophilia]/11641                       -SM-----TEREKGTYFEELILAYLRNEATYRDLYA-KVWTWSDWA---------PA---
gi|648289350|ref|WP_026070499.1|damageinducibleprotein[Stenotrophomonasmaltophilia]/11639                       -SM-----TEREKGTYFEELILAYLRNEATYRDLYR-QVWTWAEWA---------PA---
gi|656272786|ref|WP_029218442.1|damageinducibleprotein[Xanthomonascassavae]/11639                               -SM-----TEREKGTYFEELILAYLRNEATYRDLYR-QVWTWAEWA---------PA---
gi|406976236|gb|EKD98747.1|hypotheticalproteinACD_23C00290G0002[unculturedbacterium]/11679                      -AQ-----TEREKGTYFERLVKTYLQQEPSYQDLFGGQVYLWEEWR---------KQCLQ
gi|828145737|ref|WP_047238120.1|damageinducibleprotein[Chromobacteriumsubtsugae]/11645                          -AV-----TEREKGTYFEELIICYLKNEATYRDLYS-DVWTFAEWV---------EQY--
gi|654311174|ref|WP_027824327.1|damageinducibleprotein[Laribacterhongkongensis]/11641                           -AV-----TEREKGTYFEELIVCYLKNEATYRDLYS-DVWPFGEWA---------EQY--
gi|757242173|gb|KIR15516.1|typeIrestrictionenzymeEcoKIsubunitR[Pseudomonasfluorescens]/11778                   -SI-----SEREKGTYFEELICTYLRNEATYRDLYE-QLWTYSDWA---------RE---
gi|653605161|ref|WP_027616682.1|damageinducibleprotein[Pseudomonassp.URHB0015]/11639                            -SV-----SEREKGTYFEELICTYLRNEAAYCDLYA-QVWTYSDWS---------KE---
gi|520868025|ref|WP_020308060.1|helicase[Pseudomonassyringae]_gi|519159034|gb|EPM49935.1|helicasedomaincontainingprotein[Pseud/   -SV-----SEREKGTYFEELICTYLRNEATYCDLYA-QVWTYSAWS---------KE---
gi|940156567|gb|KPX71668.1|Helicasedomaincontainingprotein[Pseudomonasamygdalipv.lachrymans]/11677             -SV-----SEREKGTYFEELICTYLRNEATYRDLYE-QVWTYSDWA---------KE---
gi|505460508|ref|WP_015647610.1|helicase[Pseudomonassp.GLE121]_gi|499110212|ref|YP_007974374.1|helicasedomaincontainingprotein/   -SV-----SEREKGTYFEELICTYLRNEATYCDLYE-QVWTYSCWA---------KE---
gi|940235551|gb|KPY45311.1|hypotheticalproteinALO48_200154[Pseudomonassyringaepv.rhaphiolepidis]/11631         -SV-----SEREKGTYFEELICTYLRNEAAYRDLYE-QVWSYSCWA---------KE---
gi|520880808|ref|WP_020312235.1|helicase[Pseudomonassyringae]_gi|519172158|gb|EPM62027.1|helicasedomaincontainingprotein[Pseud/   -SV-----TEREKGTYFEELIYTYLHNEATYRDLYE-EVWTYADWA---------KE---
gi|829046479|ref|WP_047282259.1|damageinducibleprotein[Pseudomonasfluorescens]/11630                            -SV-----TEREKGTYFEELICAYLRNEATYRDLYD-KVWMYGDWA---------KE---
gi|499415493|ref|WP_011102960.1|DNAdamageinducibleprotein[Pseudomonassyringaegroupgenomosp.3]_gi|28867279|ref|NP_789898.1|heli/   -AV-----TEREKGTYFEELICGYLRNEATYRDLYE-KVWTYAEWA---------KE---
gi|730238299|ref|WP_033942897.1|damageinducibleprotein[Pseudomonasaeruginosa]/11629                             -SV-----TEREKGTYFEELICAYLRNEATYRDLYD-KVWMYADWA---------KE---
gi|495310125|ref|WP_008034873.1|DNAdamageinducibleprotein[Pseudomonassp.GM60]_gi|398186685|gb|EJM74052.1|putativehelicase[Pseu/   -SV-----TEREKGTYFEELICAYLRNEATYRDLYD-KVWMYSDWA---------KE---
gi|779992653|ref|WP_045428690.1|damageinducibleprotein[Pseudomonassp.MT1]_gi|699967872|dbj|BAP80334.1|DNAhelicaserestrictione/1   -SV-----TEREKGTYFEELICTYLRNEATYRDLYD-KVWTYASWA---------KE---
gi|786037289|ref|WP_045733274.1|damageinducibleprotein[Pseudomonaspseudoalcaligenes]_gi|782987904|gb|KJU79152.1|damageinducib/1   -SV-----TEREKGTYFEELICAYLRNEATYRDLYE-QVWTYAEWA---------KE---
gi|749666366|ref|WP_040265445.1|damageinducibleprotein[Pseudomonasrhodesiae]/11632                              -SV-----TEREKGTYFEELICTYLRNEATYRDLYD-KVWTYAEWA---------KE---
gi|734902433|ref|WP_034133698.1|damageinducibleprotein[Pseudomonasfluorescens]/11635                            -SV-----TEREKGTYFEELICTYLRNEATYRDLYD-KVWTYAEWA---------KE---
gi|490551031|ref|WP_004416146.1|typeIIrestrictionmodificationsystemrestriction/helicasesubunit[Pseudomonassyringae]_gi|4432823/   -SV-----TEREKGTYFEELICTYLRNEATYRDLYD-KVWTYAEWA---------KE---
gi|489199911|ref|WP_003109152.1|DNAdamageinducibleprotein[Pseudomonasaeruginosa]_gi|552648708|gb|ERY63184.1|hypotheticalprotei/   -SV-----TEREKGTYFEELICAYLRNEATYRDLYD-KVWTYADWA---------KE---
gi|730641237|ref|WP_034061328.1|damageinducibleprotein[Pseudomonasaeruginosa]/11636                             -SV-----TEREKGTYFEELICTYLRNEATYRDLYD-KVWTYAEWA---------KE---
gi|751304980|ref|WP_041013386.1|damageinducibleprotein[Pseudomonasxanthomarina]_gi|703407611|emb|CEG51014.1|Helicasedomaincon/1   -SV-----TEREKGTYFEELICAYLRNEATYRDLYD-KVWTYADWA---------KE---
gi|939335207|ref|WP_054881520.1|damageinducibleprotein[Pseudomonassp.NBRC111128]/11632                          -SV-----TEREKGTYFEELICTYLRNEATYRDLYD-KVWAYAEWA---------KE---
gi|757693555|ref|WP_042923792.1|damageinducibleprotein[Pseudomonaschloritidismutans]_gi|780910610|gb|KJS78302.1|damageinducib/1   -SV-----TEREKGTYFEELICAYLRNEATYRDLYD-KVWTYADWA---------KE---
gi|665953944|ref|WP_031319989.1|damageinducibleprotein[Pseudomonastolaasii]/11626                               -SV-----SEREKGTYFEELICVYLRNEATYSDLYD-RVWTYAEWA---------KE---
gi|771600573|ref|WP_045213746.1|damageinducibleprotein[Pseudomonassp.21]_gi|770081123|gb|KJJ98634.1|damageinducibleprotein[Ps/1   -SV-----SEREKGTYFEELICAYLRNEATYRDLYD-KVWMYADWA---------KE---
gi|740871396|ref|WP_038656644.1|damageinducibleprotein[Pseudomonasstutzeri]_gi|627772214|gb|AHY41437.1|damageinducibleprotein/1   -AL-----TEREKGTYFEELICAYLRNEATYRDLYD-KVWTYADWA---------KE---
gi|946242205|gb|KRC93782.1|damageinducibleprotein[Pseudomonassp.Root9]/11634                                   -SV-----TEREKGTYFEELVCTYLRNEATYRDLYE-KVWTYAQWA---------KE---
gi|553777322|ref|WP_023109107.1|MULTISPECIES:hypotheticalprotein[Pseudomonas]_gi|552638674|gb|ERY53184.1|hypotheticalproteinQ05   -SV-----TEREKGTYFEELICTYLRNEATYRDLYD-RVWTYAEWA---------KE---
gi|896383370|ref|WP_049324618.1|damageinducibleprotein[Pseudomonasstutzeri]/11627                               -AL-----TEREKGTYFEELICAYLRNEATYRDLYD-KVWTYADWA---------KE---
gi|696158510|gb|AIT41787.1|putativehelicase[Pseudomonassp.LM8]/11630                                           -SV-----TEREKGTYFEELICAYLRNEATYRDLYD-KVWMYGDWA---------KE---
gi|504634892|ref|WP_014821994.1|DNAdamageinducibleprotein[Pseudomonasstutzeri]_gi|390985249|gb|AFM35242.1|helicasedomainconta/1   -SV-----TEREKGTYFEELICTYLRNEATYRDLYD-KVWTYSNWA---------IE---
gi|544783974|ref|WP_021206729.1|DNAdamageinducibleprotein[Pseudomonasstutzeri]_gi|533224779|gb|EQM79688.1|DNAdamageinduciblep/1   -AL-----TEREKGTYFEELICAYLRNEATYRDLYD-KVWTYADWA---------KE---
gi|504567385|ref|WP_014754487.1|MULTISPECIES:DNAdamageinducibleprotein[Pseudomonas]_gi|388559493|gb|AFK68634.1|helicasedomain/1   -SV-----TEREKGTYFEELVCTYLRNEATYRDLYE-KVWTYAQWA---------KE---
gi|654441469|ref|WP_027912052.1|damageinducibleprotein[Pseudomonassp.URIL14HWK12:I7]/11630                      -SV-----TEREKGTYFEELICAYLRNEATYSDLYD-KVWTFANWA---------KE---
gi|941470066|ref|WP_055127894.1|damageinducibleprotein[Pseudomonasmediterranea]/11631                           -SV-----TEREKGTYFEELICTYLRNEATYRDLYD-KVWTYAEWA---------KE---
gi|557815599|ref|WP_023446184.1|DNAdamageinducibleprotein[Pseudomonaschloritidismutans]_gi|557364081|gb|ESQ98190.1|DNAdamagei/1   -SV-----TEREKGTYFEELVCTYLRNEATYRDLYD-KVWTYAEWA---------KE---
gi|727814791|ref|WP_033898247.1|damageinducibleprotein[Pseudomonassp.FH1]_gi|567648005|gb|ETK23229.1|helicasedomaincontaining/1   -SV-----TEREKGTYFEELICTYLRNEATYRDLYD-KVWTFAEWA---------KE---
gi|503278702|ref|WP_013513363.1|DNAdamageinducibleprotein[Desulfovibrioaespoeensis]_gi|316942375|gb|ADU61426.1|typeIIrestrict/   -AV-----SEREKGTYFEELTICYLRNEATYKDLYS-DVWRYAEWA---------EL---
gi|505228796|ref|WP_015415898.1|typeIIrestrictionendonucleasesubunitR[Desulfovibriopiezophilus]_gi|451896976|emb|CCH49855.1|Ty   -ST-----TEREKGTYFEELTICYLKNEATYKDLYS-DVWMYSEWA---------EL---
gi|517732543|ref|WP_018902751.1|hypotheticalprotein[Variovoraxparadoxus]/11675                                  -AA-----SEREKGTYFEELIVCYLRNEATYRDLYS-DVWTYAEWA---------KL---
gi|550909475|ref|WP_022662570.1|DNAdamageinducibleprotein[Desulfovibriolongus]/11637                            -AA-----TEREKGTYFEELIVCYLRNEASYRDLYS-DVWTYAEWA---------ET---
gi|945494542|gb|KQV50024.1|damageinducibleprotein[Massiliasp.Root335]/11647                                    -SQ-----SEREKGTYFEELMLCYLRNEATYRDLYS-DVWTYSDWA---------EL---
gi|334196917|gb|AEG70102.1|helicasedomainprotein[RalstoniasolanacearumPo82]/11663                               -AV-----TEREKGTYFEELIVCYLRNEATYRDLYS-DVWTYAEWA---------DL---
gi|656006746|ref|WP_029048170.1|damageinducibleprotein[Cupriavidussp.amp6]/11661                                -AV-----TEREKGTYFEELIVCYLRNEATYRDLYS-DVWTYAEWA---------DL---
BPS2196ORF3054P/11659                                                                                            -AV-----TEREKGTYFEELIVTYLRNEATYRDLYS-DVWTYAEWA---------DL---
gi|740932312|ref|WP_038717332.1|damageinducibleprotein[Burkholderiapseudomallei]_gi|760211882|gb|KIX58314.1|damageinduciblepr/1   -AV-----TEREKGTYFEELIVTYLRNEATYRDLYS-DVWTYAEWA---------DL---
gi|755810813|ref|WP_042583861.1|damageinducibleprotein[Burkholderiasp.MSHR3999]_gi|755174952|gb|KIP19790.1|DEAD/DEAHboxhelicas/   -AV-----TEREKGTYFEELIVCYLQNEATYRDLYS-DVWTYAEWA---------DL---
gi|484586570|gb|AGK48195.1|DEAD/DEAHboxhelicasefamilyprotein[BurkholderiathailandensisMSMB121]/11646           -AV-----TEREKGTYFEELIVCYLRNEATYRDLYS-DVWTYAEWA---------DL---
gi|737582935|ref|WP_035553941.1|damageinducibleprotein[Burkholderiasp.9120]/11658                               -AV-----TEREKGTYFEELIVCYLRNEADYRDRYS-DVWTYAEWA---------NL---
gi|740989872|ref|WP_038773225.1|damageinducibleprotein[Burkholderiapseudomallei]_gi|705645291|gb|KGS70691.1|DEAD/DEAHboxhelica/   -AV-----TEREKGTYFEELIVTYLRNEATYRDLYS-DVWTYAEWA---------DL---
gi|654912525|ref|WP_028363424.1|damageinducibleprotein[Burkholderiasp.JPY366]/11659                             -AV-----TEREKGTYFEELIVCYLRNEATYRDLYS-DVWTYGEWA---------DL---
gi|740955879|ref|WP_038740512.1|damageinducibleprotein[Burkholderiapseudomallei]_gi|714684932|gb|KGW74438.1|DEAD/DEAHboxhelica/   -AV-----TEREKGTYFEELIVTYLRNEATYRDLYS-DVWTYAEWA---------DL---
gi|919203714|ref|WP_052759215.1|damageinducibleprotein[Xanthomonasaxonopodis]/11388                              -A-----TDHRDKGGKFERLMKAYFLTDPLYLSRFSN-VWLWNEWP---------DRK--
gi|522152381|ref|WP_020663589.1|hypotheticalprotein[Amycolatopsisbenzoatilytica]/11605                          -P-----VSNRERGDKFERLMVQYLKLDPLYAEKFSE-VWMWADWP---------GRA--
gi|663196013|ref|WP_030233899.1|damageinducibleprotein[Streptomycessp.NRRLS455]/11641                           -S-----TTNRELGTAFERLMVRFLSTDPLWSEQFSR-VWMYGEWP---------GAA--
gi|750429553|ref|WP_040711444.1|damageinducibleprotein[Nocardiopsispotens]/11622                                -S-----TVNRDRGSRFEELMLQYLDTDPQWSDQFSR-TWLWTHWP---------GAE--
gi|518656139|ref|WP_019819475.1|hypotheticalprotein[Saccharomonosporasaliphila]/11623                           -S-----TVNRDRGTRFEELMVQYLSTDPQWSEQFSR-VWMWADWP---------GAE--
gi|759722933|ref|WP_043433758.1|damageinducibleprotein[Streptomycespluripotens]_gi|733404978|gb|KHK94361.1|damageinduciblepro/1   -A-----TGNRDRGTRFEELMVQYLSTDPQWTEQFTR-VWMWADWP---------GAE--
gi|663424167|ref|WP_030419030.1|damageinducibleprotein[Streptomycessp.NRRLF5065]/11627                          -A-----TGNRDRGTRFEELMVQYLSTDPQWTEQFTR-VWMWADWP---------GAE--
```

```
gi|663412917|ref|WP_030408353.1|damageinducibleprotein[Streptomycessp.NRRLF5917]/11630    -A-----TGNRDRGTRFEELMVQYLSTDPQWTEQFTR-VWMWADWP---------GAE--
gi|671521483|ref|WP_031506489.1|damageinducibleprotein[Streptomycesmegasporus]/11630    -A-----TGNRDRGTRFEELMVQYLSTDPQWTEQFTR-VWMWADWP---------GAE--
gi|740053144|ref|WP_037902776.1|damageinducibleprotein[Streptomycessp.TAA204]/11626    -SI----GQNRERGTRFEELMLQYLSTDPQWNEQFSR-VWMWADWP---------GAE--
gi|663409375|ref|WP_030404974.1|MULTISPECIES:damageinducibleprotein[Streptomyces]/11627    -SI----GKNRDRGTRFEELILQYLSTDPQWTEQFSR-VWMWADWP---------GAE--
gi|746628608|ref|WP_039654277.1|damageinducibleprotein[Streptomycessp.MUSC125]_gi|745644381|gb|KIE25557.1|damageinducibleprot/1    -SI----GKNRDRGTRFEELILQYLSTDPQWTEQFSR-VWMWTDWP---------GAE--
gi|727322066|ref|WP_033777320.1|damageinducibleprotein[Salinisporapacifica]/11627    -S-----SSNRDRGTGFEELMLQYLSTDPQWTEQFSR-VWMWADWP---------GTE--
gi|653653242|ref|WP_027655726.1|damageinducibleprotein[Salinisporapacifica]/11618    -S-----PTNRDRGTGFEELMLKYLSTDSQWTEQFSR-VWMWADWP---------GAA--
gi|648632543|ref|WP_026324294.1|damageinducibleprotein[Salinisporapacifica]/11621    -S-----PTNRDRGTGFEELMLKYLSIDPQWTEQFSR-VWMWADWP---------GAA--
gi|881018002|ref|WP_048738463.1|damageinducibleprotein[Corynebacteriumfalsenii]/11676    -R-----PKSTRYGIDFEKLIVNFFKQDPVLSNQYTD-VCRWTDWA---------FND--
gi|505465526|ref|WP_015652417.1|helicase[Corynebacteriumcallunae]_gi|459385704|gb|AGG67995.1|helicase[CorynebacteriumcallunaeDS    -P-----SQ-SAQGLAFEKLMVNFIKSDPTLSTEFDE-VHRWVDWP---------YNG--
gi|41223062|emb|CAF19007.1|DNAorRNAhelicaseofsuperfamilyII[CorynebacteriumglutamicumATCC13032]/11646    -P-----SQ-SAQGLAFEKLMVNFIKSDPTLSTEFDE-VHRWVDWP---------YNG--
CGL13032I/11643    -P-----SQ-SAQGLAFEKLMVNFIKSDPTLSTEFDE-VHRWVDWP---------YNG--
gi|751257081|ref|WP_040967675.1|damageinducibleprotein[Corynebacteriumglutamicum]_gi|748811620|gb|AJE67931.1|damageinduciblep/1    -P-----TK-AHQGIAFEKLMVNFIKADPTLAQQYGE-VYRWQDWK---------YNG--
gi|749040539|ref|WP_040086524.1|damageinducibleprotein[Corynebacteriumhumireducens]/11663    -P-----TH-GNHGLSFEKLMVNYFKLDPTLAKEYDE-VYEWSDWP---------YND--
gi|491662899|ref|WP_005519615.1|helicase[Corynebacteriummatruchotii]_gi|224946756|gb|EEG27965.1|helicaseCterminaldomainprotein/    -P-----SK-AKQGLAFEKLMVNYFKTDPVLRSEYDE-VYRWVDWP---------LNG--
gi|746717312|ref|WP_039676231.1|damageinducibleprotein[Corynebacteriumminutissimum]_gi|734991118|gb|KHO29219.1|damageinducibl/1    -Q-----PQ-GSYGIKFEKLMVNYFRTDPVLQEQFSE-VSRWSDWE---------FNG--
gi|908654892|emb|CQD15529.1|SuperfamilyIIDNA/RNAhelicase(modularprotein)[Corynebacteriumstriatum]/11674    -Q-----PQ-GSYGIKFEKLMVNYFRTDPVLQDQFSE-VSRWSDWE---------FNG--
gi|895994446|ref|WP_049064418.1|damageinducibleprotein[Corynebacteriumaurimucosum]/11667    -Q-----PQ-GSYGIKFEKLMVNYFRTDPVLQDQFSE-VSRWSDWE---------FNG--
gi|896123331|ref|WP_049145390.1|damageinducibleprotein[Corynebacteriumaurimucosum]/11669    -Q-----PQ-GSYGIKFEKLMVNYFRTDPVLQEQFSE-VSRWSDWE---------FNG--
gi|817681178|ref|WP_046645422.1|damageinducibleprotein[Corynebacteriumstriatum]_gi|817551679|gb|KKO79868.1|damageinduciblepro/1    -Q-----PQ-GSYGIKFEKLMVNYFRTDPVLQDQFSE-VSRWSDWE---------FNG--
gi|896139239|ref|WP_049159146.1|damageinducibleprotein[Corynebacteriumaurimucosum]/11675    -Q-----PQ-GSYGIKFEKLMVNYFRTDPVLQDQFSE-VSRWSDWE---------FNG--
gi|489943947|ref|WP_003847254.1|helicase[Corynebacteriumammoniagenes]_gi|295968218|gb|EFG81467.1|helicaseCterminaldomainprotei/    -Q-----PQ-GKYGIAFEKLMVNFFRTAPTLASQFDE-VYRWSDWR---------YNG--
gi|516653464|ref|WP_018022453.1|helicase[Corynebacteriumdoosanense]_gi|697778282|gb|AIT61018.1|damageinducibleprotein[Coryneba/    -Q-----PQ-GKYGIAFEKLMVNFFRTAPTLANQFDE-VYRWGDWR---------YNN--
gi|493894179|ref|WP_006840129.1|helicase[Corynebacteriumlipophiloflavum]_gi|227078819|gb|EEI16782.1|helicaseCterminaldomainpro/    -Q-----PA-GKYGSAFEKLMVNFFRSDPVLANEYED-VCRWVDWR---------YNG--
gi|740831472|ref|WP_038616755.1|damageinducibleprotein[Corynebacteriumpseudotuberculosis]_gi|666103058|gb|AIG06935.1|putativeh/    -Q-----PQ-GKYGIAFEKLMVNFFRMAPTLAEQFDE-VSRWGDWR---------YNG--
gi|518023322|ref|WP_019193530.1|helicase[Corynebacteriumtimonense]/11655    -Q-----PQ-AKYGIAFEKLMVNFFRTSPSLAEQFDE-VCRWADWR---------YNG--
gi|552767697|ref|WP_023019559.1|MULTISPECIES:hypotheticalprotein[Corynebacterium]_gi|550739013|gb|ERS38609.1|hypotheticalprotei    -Q-----PQ-GKYGIAFEKLMVNFFRTTPSLAEQFDE-VCRWSDWR---------YNN--
gi|652659121|ref|WP_027017812.1|damageinducibleprotein[Corynebacteriumpseudodiphtheriticum]/11644    -Q-----PQ-GKYGIAFEKLMVNFFRTTPSLAEQFDE-VCRWSDWR---------YNN--
gi|749039309|ref|WP_040085390.1|damageinducibleprotein[Corynebacteriumhumireducens]/11657    -Q-----PP-AKYGLAFEKLMVNYFRHDPTLSQEYDQ-VCRWVDWE---------FNG--
gi|740835591|ref|WP_038620874.1|damageinducibleprotein[Corynebacteriumulcerans]_gi|698973854|gb|AIU33664.1|HelicaseCterminald/1    -Q-----PQ-GKYGIAFEKLMVNFFRTDPTLANEFDE-VHRWVDWK---------CNG--
gi|805307599|ref|WP_046096452.1|damageinducibleprotein[Corynebacteriumulcerans]_gi|803357323|gb|AKA97638.1|Damageinduciblepro/1    -Q-----PQ-GKYGIAFEKLMVNFFRTDPTLANEFDE-VHRWVDWK---------CNG--
gi|504083555|ref|WP_014317549.1|helicase[Corynebacteriumdiphtheriae]_gi|371584249|gb|AEX47914.1|superfamilyIIDNA/RNAhelicase[Co    -Q-----PK-AKYGIAFEKLMVNYFRTDPTLRNEFDE-VVRWSDWR---------YNG--
gi|896182193|ref|WP_049193580.1|damageinducibleprotein[Corynebacteriumjeikeium]/11645    -Q-----PK-AKYGIAFEKLMVNYFRTDPTLKNEFDE-VVRWTDWR---------YNG--
gi|917636459|ref|WP_052205460.1|damageinducibleprotein[Corynebacteriumriegelii]_gi|914295811|gb|AKV59187.1|damageinduciblepro/1    -Q-----PQ-GKYGIAFEKLMVNFFQTDPVLSAEFDE-VCRWDKWR---------YNG--
gi|737013522|ref|WP_035009205.1|damageinducibleprotein[Corynebacteriumjeikeium]/11651    -Q-----PQ-GKYGIAFEKLMVNFFKTDPVLSEQFDE-VHRWDKWR---------YNG--
gi|737006291|ref|WP_035002094.1|damageinducibleprotein[Corynebacteriumjeikeium]/11658    -Q-----PK-AKYGIAFEKLMVNYFRTDPTLKAQFDE-VYRWADWR---------YNG--
gi|737010378|ref|WP_035006133.1|damageinducibleprotein[Corynebacteriumjeikeium]/11650    -Q-----PQ-AMYGIAFEKLMVNYFRTDPTLKAQFDE-VYRWTDWR---------YNG--
gi|737010473|ref|WP_035006228.1|damageinducibleprotein[Corynebacteriumjeikeium]/11657    -Q-----PT-AKYGIAFEKLMVNYFRADPVLSTTYDR-VVRWNDWE---------YNG--
gi|737006250|ref|WP_035002053.1|damageinducibleprotein[Corynebacteriumjeikeium]/11658    -Q-----PT-AKYGIAFEKLMVNYFRADPVLSTIYDR-VVRWNDWE---------YNG--
gi|923018168|ref|WP_053412254.1|damageinducibleprotein[Corynebacteriumlactis]_gi|922845184|gb|ALA67504.1|helicase[Corynebacter/    -Q-----PK-AKYGIAFEKLMVNYFRTDPTLRAQFDE-VYRWSDWR---------YNG--
gi|923018885|ref|WP_053412970.1|damageinducibleprotein[Corynebacteriumlactis]_gi|922845845|gb|ALA68165.1|helicase[Corynebacter/    -Q-----PK-AKYGIAFEKLMVNYFRTDPTLRAQFDE-VYRWSDWR---------YNG--
gi|512464870|ref|WP_016422869.1|hypotheticalprotein[Corynebacteriumsp.HFH0082]_gi|512047830|gb|EPD47505.1|hypotheticalproteinHM    -Q-----PK-AKYGIAFEKLMVNYFRTDPTLKAQFDE-VYRWSDWK---------YNG--
gi|881012697|ref|WP_048733406.1|damageinducibleprotein[Corynebacteriumurealyticum]/11670    -Q-----PK-AKYGIAFEKLMVNYFRTDPTLKAQFDE-VYRWSDWK---------YNG--
gi|755608478|ref|WP_042530519.1|damageinducibleprotein[Corynebacteriumsingulare]_gi|754340239|gb|AJI78730.1|putativehelicase[C/    -Q-----PK-AKYGIAFEKLMVNYFKTDPTLKNQFDE-VCRWTDWR---------YNG--
gi|491421796|ref|WP_005279593.1|helicase[Corynebacteriumaccolens]_gi|227077180|gb|EEI15143.1|helicaseCterminaldomainprotein[Co/    -Q-----PK-AKYGIAFEKLMVNYFQTDPTLKAQFDE-VSRWTDWR---------YNG--
gi|550752286|gb|ERS51451.1|hypotheticalproteinHMPREF1282_00033[Corynebacteriumsp.KPL1856]_gi|550759961|gb|ERS58964.1|hypothetic    -Q-----PK-AKYGIAFEKLMVNFFRTDPTLRTQFDE-VSRWVDWR---------YNG--
gi|497955053|ref|WP_010269209.1|helicase[Corynebacteriumbovis]/11599    -P---------DRGIAFEKLMVRYFGLHPTLRQEYDQ-VCRWTDWE---------YNG--
gi|860580040|ref|WP_048470569.1|hypotheticalprotein[Mycobacteriumchlorophenolicum]_gi|859098575|gb|KMO76871.1|typeIrestrictione    -P-----TN-VDRGTLFEKLMVRYFQLDPALTQQYDE-VWRWIDWP---------DRK--
gi|262085905|gb|ACY21873.1|typeIIIrestrictionproteinressubunit[GordoniabronchialisDSM43247]/11632    -P-----TN-SERGTKFEQLMVRYFELDPTMAQQYDG-VWRWIDWP---------GRQ--
gi|495996338|ref|WP_008720917.1|typeIIIrestrictionendonucleasesubunitR[Rhodococcussp.AW25M09]_gi|443412350|emb|CCQ18455.1|TypeI    -P-----SN-SERGTKFEQLMVRYFELDPTLSQQYEQ-VWRWIDWP---------DRQ--
gi|739284636|ref|WP_037147410.1|damageinducibleprotein[Rhodococcusfascians]/11641    -P-----SN-SERGTKFEQLMVRYFELDPTLAQQYEQ-VWRWIDWP---------DRQ--
gi|739328195|ref|WP_037190574.1|damageinducibleprotein[Rhodococcusfascians]/11643    -P-----SN-SERGTKFEQLMVRYFELDPTLSQQYEQ-VWRWIDWP---------DRQ--
gi|811698335|emb|CMN21041.1|helicase[Mycobacteriumtuberculosis]/11725    -P-----SN-AERGTKFEQLMVRYFELDPTMAQQYDA-VWRWIDWP---------ERR--
gi|801577898|emb|CFK23773.1|helicase[Mycobacteriumtuberculosis]/11675    -P-----SN-AERGTKFEQLMVRYFELDPTMAQQYDA-VWRWIDWP---------ERR--
MTU18II/11606    -P-----SN-AERGTKFEQLMVRYFELDPTMAQQYDA-VWRWIDWP---------ERR--
gi|887719858|emb|CMT27886.1|helicase[Mycobacteriumtuberculosis]/11667    -P-----SN-AERGTKFEQLMVRYFELDPTMAQQYDA-VWRWIDWP---------ERR--
gi|621694743|gb|KAS87156.1|hypotheticalproteinAL70_01801[MycobacteriumtuberculosisTKK_03_0115]/11604    -P-----SN-AERGTKFEQLMVRYFELDPTMAQQYDA-VWRWIDWP---------ERR--
gi|888128072|emb|CLQ84422.1|helicase[Mycobacteriumtuberculosis]_gi|889917090|emb|CLY86893.1|helicase[Mycobacteriumtuberculosis]    -P-----SN-AERGTKFEQLMVRYFELDPTMAQQYDA-VWRWIDWP---------ERR--
gi|884823424|emb|CKL96672.1|helicase[Mycobacteriumtuberculosis]/11466    -P-----SN-AERGTKFEQLMVRYFELDPTMAQQYDA-VWRWIDWP---------ERR--
gi|505103239|ref|WP_015290341.1|hypotheticalprotein[Mycobacteriumcanettii]_gi|432162733|emb|CCK60117.1|Conservedproteinofunknow    -P-----SN-AERGTKFEQLMVRYFELDPTMAQQYDA-VWRWIDWP---------DRR--
gi|505116250|ref|WP_015303352.1|hypotheticalprotein[Mycobacteriumcanettii]_gi|432158726|emb|CCK56026.1|Conservedproteinofunknow    -P-----SN-AERGTKFEQLMVRYFELDPTMAQQYDA-VWRWIDWP---------ERR--
gi|432154735|emb|CCK51974.1|Conservedproteinofunknownfunction(part1)[MycobacteriumcanettiiCIPT140060008]/11588    -P-----SN-AERGTKFEQLMVRYFELDPTMAQQYDA-VWRWIDWP---------ERR--
gi|809008027|ref|WP_046295375.1|hypotheticalprotein[Mycobacteriumsp.UM_Kg17]/11624    -P-----SN-SERGTKFEQLMVRYFELDPMLAQQYDA-VWRWIDWP---------GRD--
gi|808802633|ref|WP_046284139.1|hypotheticalprotein[Mycobacteriumsp.UM_NZ2]/11624    -P-----SN-SERGTKFEQLMVRYFELDPMLSQQYDA-VWRWIDWP---------GRD--
gi|750125774|ref|WP_040429852.1|damageinducibleprotein[Corynebacteriumnuruki]/11668    -R-----ST-SERGTQFEQLMVRWFELDPTLHEQYDK-VWRWPDWP---------GRH--
gi|602237614|gb|EYT60960.1|damageinducibleprotein[Dietziasp.UCDTHP]/11473    -P-----NT-VEMGTKFEQLMVRYFELDPTLSREYDA-VWRWMDWP---------GRD--
gi|665897412|ref|WP_031264618.1|damageinducibleprotein[Dietziasp.UCDTHP]_gi|602237599|gb|EYT60945.1|helicase[Dietziasp.UCDTH/11    -P-----NT-VEMGTKFEQLMVRYFELDPTLSREYDA-VWQWMDWP---------DRD--
```

```
gi|502903674|ref|WP_013138650.1|helicase[Segniliparusrotundus]_gi|296181291|gb|ADG98197.1|typeIIIrestrictionproteinressubunit[S    -P-----SN-SERGTKFEKLMVRYFALDPMLSQEYDA-VWRWIDWP---------DRA--
gi|780248306|ref|WP_045538930.1|hypotheticalprotein[Gordoniasp.no.9]_gi|778142514|gb|KJR06917.1|hypotheticalproteinUG54_12320[G    -P-----SN-SERGTKFEKLMVRYFELDPLLAQRFEE-VSLWIDWE---------GRE--
gi|665897368|ref|WP_031264574.1|damageinducibleprotein[Dietziasp.UCDTHP]_gi|602239137|gb|EYT62483.1|helicase[Dietziasp.UCDTH/11    -P-----TN-VERGTKFEQLMVRYFELDPMLSQQYDK-VWRWIDWP---------GRD--
gi|493372923|ref|WP_006329236.1|helicase[Gordoniarhizosphera]_gi|403207917|dbj|GAB88168.1|hypotheticalproteinGORHZ_006_00370[Go    -P-----SN-SERGTKFEKLMVRYFELDPTMSQQYDQ-VWRWIDWP---------DRA--
gi|764904870|ref|WP_044487821.1|hypotheticalprotein[Mycobacteriumtuberculosis]_gi|625005353|gb|KBZ57120.1|hypotheticalproteinK8    -P-----SN-SERGTKFEKLMVRYFDLDPLLSQQYDA-VWRWIDWP---------GRK--
gi|927068212|ref|WP_053778441.1|damageinducibleprotein[Gordoniasp.NB41Y]_gi|926660541|gb|EMP14526.2|damageinducibleprotein[G/11    -P-----SN-SERGTKFEKLMVRYFELDPMLSQQYDA-VWRWIDWP---------GRQ--
gi|935514169|ref|WP_054429894.1|damageinducibleprotein[Mycobacteriumimmunogenum]_gi|929801120|gb|KPG29247.1|damageinduciblepr/1    -P-----SN-SERGTKFEKLMVRYFELDPLLAQQYDA-VWRWIDWP---------DRK--
gi|941317728|ref|WP_055118574.1|hypotheticalprotein[Mycobacteriumperegrinum]/11657    -P-----SN-SERGTKFEKLMIRYFELDPLLSQQYDA-VWRWIDWP---------GRD--
gi|933683673|gb|ALG86377.1|hypotheticalproteinACH46_20120[Gordoniasp.QH11]/11636    -R-----SN-SERGTKFEELMVRYFELDPLLSQQYDA-VWRWIDWP---------DRD--
gi|873553947|emb|CRY84110.1|Predictedhelicase[Nocardiafarcinica]/11688    -P-----SN-SERGTKFEQLMVRYFELDPMLAQQYDK-VWRWTDWP---------DRA--
gi|499525749|ref|WP_011212389.1|helicase[Nocardiafarcinica]_gi|54019339|dbj|BAD60707.1|hypotheticalprotein[NocardiafarcinicaIFM    -P-----SN-SERGTKFEQLMVRYFELDPMLAQQYDK-VWRWTDWP---------DRA--
gi|640109609|ref|WP_024794187.1|damageinducibleprotein[Tomitellabiformata]/11653    -P-----SN-SERGTKFEHLMVRYFQLDPTLNQQYDQ-VWRWMDWP---------GRD--
gi|640109991|ref|WP_024794334.1|hypotheticalprotein[Tomitellabiformata]/11639    -P-----SN-SERGTKFEQLMVRYFQLDPTLGQQYDQ-VWRWMDWP---------GRD--
gi|939125496|ref|WP_054831438.1|damageinducibleprotein[Rhodococcuserythropolis]/11669    -P-----SN-SERGTKFEQLMVRYFELDPLLLQQYEK-VWRWIDWP---------GRK--
gi|910258550|ref|WP_050062711.1|hypotheticalprotein[Rhodococcussp.RD6.2]_gi|846871967|emb|CRK49225.1|conservedhypotheticalprote    -P-----SN-SERGTKFEQLMVRYFQLDPMLAQQYDQ-VWRWTDWP---------GRD--
gi|797148189|gb|KJY52358.1|putativeHelicase[Bifidobacteriumasteroides]/11801    -MAAQVEGGERARGTAFEQATKYFLTHDSAWAPQFSN-VWMWSEKGNPLLTNDAHGQPH-
gi|535476570|gb|EFG27357.2|hypotheticalproteinHMPREF9020_00999[ScardoviainopinataF0304]/11827    KLSSQGKRNQRVRGTAFEYAVRYFLMHDPAWASTFIN-VWMWGDEGNPLI----HGEYE-
gi|737482729|ref|WP_035462496.1|hypotheticalprotein[Alicyclobacillusmacrosporangiidus]/11512    ----------------------------MWVKTYQ--DWMIDTYP---------G----
gi|685434354|gb|KGA95679.1|helicase[BacillusalcalophilusATCC27647=CGMCC1.3604]/11575    ---DTKKHEQRDRGTLFELLVAVYLKKEPMYSRLFDK-VWKLSEVP---------SDY--
gi|945370253|gb|KQU27251.1|helicase[Bacillussp.Leaf13]/11557    ---DADYITQRDRGTMFELLSRTYFKNEPMYKRLFDE-VWTLNEVP---------VEY--
gi|880997015|ref|WP_048719116.1|helicase[Enterococcushirae]/11563    ---DLEEHVQRDRGTLFELLSKIYLSNEPMYSHRYDA-VWMLNEVP---------EQY--
gi|945356929|gb|KQU14100.1|helicase[Bacillussp.Leaf49]/11547    ---DGEYQTQRDRGTLFELLSKTYFENEPMYKRLFDK-VWMLNEVP---------ENY--
gi|763482876|gb|KIZ53217.1|helicase[Bacillussafensis]/11563    ---DTEKREQRDRGTLFEILVSAYLKNEPMYARLFDE-VWMLGSVP---------EEY--
gi|852223292|ref|WP_048309707.1|helicase[Anaerobacillusmacyae]_gi|850322910|gb|KMM38590.1|helicase[Anaerobacillusmacyae]/11573    ---EHKDMIQRDRGTFFELLVTAYLKNEPMYARLFDE-VWMLGNVP---------EEY--
gi|739708665|ref|WP_037562619.1|helicase[Sporolactobacillusterrae]/11573    ---ERDNMAQRDRGTLFELLVTAYLKNEPMYARLFDE-VWMLTDVP---------EEY--
gi|496687561|ref|WP_009329104.1|MULTISPECIES:helicase[Bacillus]_gi|317390867|gb|EFV71668.1|endonucleaseandmethylaseLlaGI[Bacill    ---EGKEMEQRDRGTLFELLVTAYLKKEPMYARLFDE-VWMLADVP---------EEY--
gi|930213599|gb|KPH74225.1|helicase[Oceanobacilluscaeni]/11569    ---ERKDMSQRDRGTLFELLVTAYLKKEPMYARLFDE-VWMLADVP---------EEY--
gi|516366003|ref|WP_017756036.1|helicase[Bacillussp.ZYK]/11571    ---EREDMSQRDRGTLFELLVTAYLKKEPMYARLFDE-VWMLKDVP---------EEY--
gi|488909977|ref|WP_002821052.1|helicase[Oenococcusoeni]_gi|399965230|gb|EJN99855.1|endonucleaseandmethylaseLlaGI[Oenococcusoen    ---QTAGGVQRDRGTLFERLVVTYLKNEPLYANKFDH-VWMLADVP---------DRY--
gi|495019967|ref|WP_007745780.1|helicase[Oenococcuskitaharae]_gi|366983682|gb|EHN59081.1|typeIIIrestrictionenzyme,ressubunit[Oe    ---QTAGDVQRDRGTLFERLVVTYLKNEPLYANKFDH-VWMLADVP---------DRY--
gi|489666119|ref|WP_003570406.1|typeIIIrestrictionendonuclease[Lactobacilluscasei]_gi|410527834|gb|EKQ02696.1|typeIIIrestrictio    ---ASANGVQRDRGTMFEEVVKSYLLNEPAYKNLYDN-VWMLREVP---------AEY--
gi|511447623|gb|EPC78110.1|superfamilyIIDNA/RNAhelicase[Lactobacillusparacaseisubsp.paracaseiLpp221}/11615    ---ANAKGVQRDRGTMFEEVVKSYLLNEPVYKHLYDE-VWMLNEVP---------AEY--
gi|497700411|ref|WP_010014595.1|helicase[Lactobacilluscoryniformis]/11585    ---ESANGVQRDRGTMFEEVVKSYLLNEPAYKNLYDD-VWLLSEVP---------AEY--
gi|489784048|ref|WP_003687939.1|helicase[Lactobacillusmali]_gi|394428539|gb|EJF01080.1|superfamilyIIDNA/RNAhelicase[Lactobacill    ---ASANGVQRDRGTMFEEVVKSYLLNEPAYKNLYTD-VWLLQEVP---------AEY--
gi|446780825|ref|WP_000858081.1|helicase[Staphylococcusaureus]_gi|374396604|gb|EHQ67835.1|typeIIIrestrictionenzyme,ressubunit[S    ---QSAGNNQRDRGTFFEELVKIYLQNEPVYKNLYSD-VWLLKEIP---------EKY--
gi|510796658|ref|WP_016171456.1|hypotheticalprotein[Enterococcusdispar]_gi|508167532|gb|EOT44083.1|hypotheticalproteinOMK_00227    ---DSAGSVQRDRGTYFEKLVEVYLQNEPTYKNLYSD-VWLLHEVP---------VEY--
gi|612613473|gb|EZS30411.1|hypotheticalproteinW603_02482[StaphylococcusaureusVET0353R]_gi|612881917|gb|EZU93992.1|hypotheticalp    ---ESAGSIQRDRGTYFEKLVEVYLKNEPVYKNLYSD-VWLLHEVP---------AEY--
gi|488332893|ref|WP_002402278.1|helicase[Enterococcusfaecalis]_gi|306513864|gb|EFM82466.1|helicaseCterminaldomainprotein[Enter/    ---EQANEVQRDKGTLFEKIAQIYFKNEPTYKNLFSD-VWLLNEVP---------EEY--
gi|921166029|ref|WP_053111806.1|helicase[Enterococcusdurans]_gi|915771660|gb|AKX85928.1|helicase[Enterococcusdurans]/11562    ---EQSSEIQRDKGTLFEKIAQIYFKNEPTYKNLFSD-VWLLNEVP---------EEY--
gi|498525358|ref|WP_010825030.1|hypotheticalprotein[Enterococcusfaecalis]_gi|487059631|gb|EOJ86727.1|hypotheticalproteinWOC_005    ---EQANEVQRDKGTLFEKIAQIYFKNEPTYKNLFSD-VWLLNEVP---------EEY--
gi|498412624|ref|WP_010722070.1|hypotheticalprotein[Enterococcusfaecium]_gi|486634654|gb|EOF75253.1|hypotheticalproteinSGC_0253    ---EQSSEIQRDKGTLFEKIAQIYFKNEPRYKNLFSD-VWLLNEVP---------EEY--
gi|498408534|ref|WP_010718364.1|hypotheticalprotein[Enterococcusfaecium]_gi|486609608|gb|EOF50873.1|hypotheticalproteinSCW_0257    ---EQSSEIQRDKGTLFEKIAQIYFKNEPTYKNLFSD-VWLLNEVP---------EEY--
gi|498407475|ref|WP_010717483.1|hypotheticalprotein[Enterococcusfaecalis]_gi|486594855|gb|EOF36348.1|hypotheticalproteinSCK_005    ---EQANEVQRDKGTLFEKIAQIYFQNEPTYKNLFSD-VWLLNEVP---------EEY--
gi|514885392|ref|WP_016615344.1|helicase[Enterococcusfaecalis]_gi|514505843|gb|EPH71676.1|helicaseprotein[Enterococcusfaecalis0    ---EQANEVQRDKGTLFEKIAQIYFKNEPTYKNLFSD-VWLLNEVP---------EEY--
gi|325157603|gb|EGC69759.1|helicaseCterminaldomainprotein[EnterococcuscasseliflavusATCC12755]/11561    ---EQSSENQRDKGTLFEKIAQIYFKNEPTYKNLFSD-VWLLNEVP---------EEY--
gi|895856206|ref|WP_048967668.1|helicase[Enterococcusfaecalis]/11551    ---EQSSEIQRDKGTLFEKIAQIYFKNEPTYKNLFSD-VWLLNEVP---------EEY--
gi|639458836|ref|WP_024636366.1|helicase[Enterococcusfaecium]_gi|658176502|gb|KEI51159.1|helicase[EnterococcusfaeciumUC7256]/1/    ---EQSSENQRDKGTLFEKIAQIYFKNEPTYKNLFSD-VWLLNEVP---------EEY--
gi|488266511|ref|WP_002337719.1|hypotheticalprotein[Enterococcusfaecium]_gi|430581409|gb|ELB19854.1|hypotheticalproteinOIQ_0351    ---EQSKEIQRDKGTLFEKIAQIYFKNEPTYKNLFSD-VWLLNEVP---------EEY--
gi|446353356|ref|WP_000431211.1|helicase[Staphylococcusaureus]_gi|253726329|gb|EES95058.1|helicaseCterminaldomainprotein[Staph/    ---ETAQDVQRERGTLFERLVLAYLKNEPTYKNLYKN-VWLLKDVP---------SEF--
gi|672712068|emb|CDM14336.1|DNAorRNAhelicaseofsuperfamilyII[StaphylococcusepidermidisPM221]/11464    ---KASNNIQREKGTLFEKLVLTYLKNEPTYKALYKN-VWELKDVP---------NEL--
gi|896298990|ref|WP_049279905.1|helicase[Staphylococcusaureus]/11571    ---DTSNNIQREKGTLFEKLVLAYLKNEPTYKALYKN-VWELRDVP---------NEL--
gi|541903432|gb|ERH36190.1|hypotheticalproteinSEQU_00085[StaphylococcusequorumUMCCNS924]/11578    ---DTSNNIQREKGTLFEKLVLAYLKNEPTYKALYKN-VWELRDVP---------NEF--
gi|488289747|ref|WP_002360955.1|helicase[Enterococcusfaecalis]_gi|256682216|gb|EEU21911.1|endonucleaseandmethylaseLlaGI[Enteroc    ---QTAGDIQRERGTLFEKLVLSYLRNEPTYQRLYKN-VWTLGDVP---------AEY--
gi|494392045|ref|WP_007207894.1|helicase[Enterococcusitalicus]_gi|315483798|gb|EFU74284.1|helicaseCterminaldomainprotein[Enter/    ---QTAGDIQRERGTLFEKLVLSYLRNEPTYQRLYKN-VWALGDVP---------AEY--
gi|446350692|ref|WP_000428547.1|helicase[Bacilluscereus]_gi|228727580|gb|EEL78721.1|RestrictionmodificationsystemLlaBIII[Bacil/    ---EVAGDIQRERGSLFEKLVLAYLKNEPTYQRLYKN-IWTLTEVP---------IEY--
gi|488255302|ref|WP_002326510.1|hypotheticalprotein[Enterococcusfaecium]_gi|430480276|gb|ELA57459.1|hypotheticalproteinOGE_0473    ---QTAGDIQRERGTLFEKLVLAYLKNEPTYQRLYKN-VWELSDVP---------TEY--
gi|843253770|ref|WP_047916698.1|helicase[Lactococcuspiscium]_gi|802124677|emb|CEN29502.1|EndonucleaseandmethylaseLlaGI,TypeISPr    ---TVSNDVQRERGTLFEKLTLAYLKNEPTYKALYQN-VWLLSEVP---------ESY--
LlaBIII/11578    ---TVSNDVQRERGTLFEKLTLAYLKNEPTYKALYQN-VWLLSEVP---------ESY--
gi|499320531|ref|WP_011011023.1|helicase[Listeriainnocua]_gi|16415805|emb|CAC42019.1|pli0021[ListeriainnocuaClip11262]/11569    ---TVSKDVQRERGTLFEKLTLAYLKNEPTYKALYQN-VWLLSEVP---------ESY--
gi|760026859|ref|WP_043709973.1|helicase[Weissellacibaria]_gi|758337165|gb|KIU20061.1|UvsWhelicase[Weissellacibaria]/11567    ---QSAGDVQRERGTLFEKLVLAYLKNEPTYKALYQN-VWLLADVP---------DEY--
LlaGI/11570    ---TVSKDVQRERGTLFEKLTLAYLKNEPTYKALYQN-VWLLSEVP---------ESY--
gi|499988414|ref|WP_011669132.1|helicase[Lactococcuslactis]_gi|116109019|gb|ABJ74139.1|DNAorRNAhelicaseofsuperfamilyII[Lactococ    ---TVSNDVQRERGTLFEKLTLAYLKNEPTYKALYQN-VWLLSEVP---------ESY--
gi|685938360|ref|WP_031670319.1|helicase[Listeriamonocytogenes]_gi|590403387|gb|EXL23261.1|hypotheticalproteinX846_1956[Listeri    ---TVSKDVQRERGTLFEKLTLAYLKNEPTYKALYQN-VWLLSEVP---------ESY--
gi|530793219|gb|EQC56240.1|helicase[Lactococcuslactissubsp.cremorisTIFN5]_gi|530793247|gb|EQC56263.1|helicase[Lactococcuslactis    ---TVSNDVQRERGTLFEKLTLAYLKNEPTYKALYQN-VWLLSEVP---------ESY--
gi|695270006|ref|WP_032492075.1|hypotheticalprotein[Listeriamonocytogenes]_gi|695212872|ref|YP_009081522.1|hypotheticalproteinp    ---TVSKDVQRERGTLFEKLTLAYLKNEPTYKALYQN-VWLLSEVP---------ESY--
gi|504101310|ref|WP_014335297.1|helicase[Leuconostocmesenteroides]_gi|859247905|gb|AKO63885.1|helicase(plasmid)[Leuconostocmese    ---QAAGDVQRERGTLFEKLVLAYLKHEPTYRALYQN-VWLLSDVP---------DNY--
gi|488905190|ref|WP_002816265.1|helicase[Leuconostocmesenteroides]_gi|227351522|gb|EEJ41777.1|helicaseCterminaldomainprotein[L/    ---QTAGDVQRERGTLFEKLVLAYLKHEPTYKALYQN-VWLLSDVP---------DKY--
```

```
gi|518121285|ref|WP_019291493.1|MULTISPECIES:hypotheticalprotein[Lactococcus]/11588    ---ENN-TQQRDRGTAFEYLVKAYLKNEPTYKNLFQE-VWMLSEVP---------ESE--
gi|754857213|ref|WP_042217902.1|helicase[Lactococcusgarvieae]/11549    ---DNN-TQQRDRGTAFEYLVQAYLKNEPTYKNLYSD-VWMLSEVP---------EEY--
gi|640048455|ref|WP_024784762.1|helicase[Streptococcusmutans]/11564    ---EVA-EAQRDRGTYFEYLVKAYLANEPTYKNEFKN-VWMLAEVP---------EEF--
gi|512695361|ref|WP_016476323.1|hypotheticalprotein[Veillonellasp.HPA0037]_gi|512079853|gb|EPD79018.1|hypotheticalproteinHMPREF    ---NIA-KTQRDRGTYFEYLVRAYFLNEPAYKNEFKN-VWMLSDVP---------DEY--
gi|636963514|ref|WP_024403747.1|helicase[Streptococcussuis]/11574    ---TAS-ETQRDRGTYFEYLARAYFQNEPTYKNEFKN-VWMLSDVP---------VEF--
gi|881115312|ref|WP_048802949.1|helicase[Staphylococcuspasteuri]/11573    ---NNA-DNQRDRGTYFEHLTKSYLKNEPTYQNEFKN-VWLINEVP---------EEY--
gi|446063996|ref|WP_000141851.1|helicase[Streptococcusmitis]_gi|307618725|gb|EFN97864.1|endonucleaseandmethylaseLlaGI[Streptoco    ---GLA-ETQRDRGTYFEYLARAYFQNEPTYQNEFKN-VWMLADVP---------EEF--
gi|488206609|ref|WP_002277817.1|restrictionmodificationsystemLlaBIII[Streptococcusmutans]_gi|449173033|gb|EMB75626.1|restricti/    ---YFA-KTQRDRGTYFEYLVRAYLQNEPTYQNEFKN-VWMLADVP---------EKF--
gi|640023290|ref|WP_024781621.1|helicase[Streptococcusmutans]/11566    ---YFA-KTQRDRGTYFEYLVRAYLQNEPTYQNEFKN-VWMLADVP---------EKF--
gi|814946581|ref|WP_046393781.1|helicase[Streptococcusuberis]_gi|810600885|gb|KKF57499.1|helicase[StreptococcusuberisB362]/1156    ---KLD-AAQRDRGTYFEYLVLAYFKNEPTYKNEFEN-VWMLSEVP---------SEY--
gi|765470762|ref|WP_044720336.1|helicase[Streptococcussuis]/11559    ---ESA-DTQRDRGTYFEYLVRAYLQHEPTYKNEFRN-VWMLSDVP---------AEY--
gi|654507127|ref|WP_027976443.1|helicase[Streptococcusdevriesei]/11558    ---KIA-ESQRDRGTYFEYLVRAYFQNEPTYQNEFKN-VWMLSEVP---------EEF--
gi|530812902|gb|EQC72481.1|DNAorRNAhelicaseofsuperfamilyII[Streptococcussp.HSISS2]/11570    ---GLA-ETQRDRGTYFEYLVRAYFQNEPTYQNEFKN-VWMLADVP---------AEF--
gi|489158846|ref|WP_003068504.1|helicase[Streptococcusintermedius]_gi|355367114|gb|EHG14827.1|hypotheticalproteinHMPREF9682_002    ---DLA-ETQRDRGTYFEYLVRAYLKNEPTYKNEFAE-VWMLADVP---------DSY--
RM.Lru27782ORF13200P/11620    ---NDM-TNQRDRGTAFEKLAVAYFKNEPAYRNKYSD-VWMLSDLP---------EEY--
gi|492676546|ref|WP_005919236.1|DNAphosphorothioationsystemrestrictionenzyme[Pediococcusacidilactici]_gi|482517924|gb|EOA08103.    ---NNL-DNQRDRGTAFEKMVLAYLKNEPTYHNKFSN-VWMLKDIP---------DKY--
gi|498183116|ref|WP_010497272.1|helicase[Lactobacillusacidipiscis]/11563    ---DNV-NVQRNRGTAFEKMVVSYFKNEPTYKNKYSD-VWMLNEVP---------EEY--
gi|822100485|ref|WP_046921865.1|helicase[Lactobacillusruminis]_gi|821684442|gb|KLA43321.1|restrictionmodificationsystemLlaBIII/    ---ENI-EQQRDRGTAFEKLAVAYLENEPAFKNKYSD-VWMLNEVP---------EEY--
gi|935565832|ref|WP_054450311.1|helicase[Lactobacilluskunkeei]/11573    ---EHL-TNDRDRGTAFEKMVVAYLKNEPIYKKKYSD-VWMLSDVP---------TNL--
gi|752296887|ref|WP_041152901.1|helicase[Lactobacilluskunkeei]_gi|751663520|gb|KIM18358.1|helicase[Lactobacilluskunkeei]/11577    ---EHL-TNDRDRGTAFEKMVVAYLKNEPIYKKKYSD-VWMLSDVP---------TNL--
gi|495745291|ref|WP_008469870.1|helicase[Lactobacillushominis]_gi|394484442|emb|CCI81207.1|RestrictionmodificationsystemLlaBII/    ---DNL-DKQRDRGTAFEKMVVAYLKNEPTYKQKYED-VWMLNEVP---------EEY--
gi|918672414|ref|WP_052543134.1|helicase[Lactobacillussp.]/11564    ---DNL-TKERDRGTAFEKMVVAYLKNEPAYKQKFQD-VWMLNEVP---------AEY--
gi|799141631|ref|WP_045927261.1|helicase[Lactobacilluskullabergensis]_gi|797155335|gb|KJY59264.1|putativerestrictionendonucleas    ---NNI-DNQRDRGTAFEKMVVAYLENEPAYKQKFQN-VWMLNEVP---------AEY--
gi|504379990|ref|WP_014567092.1|helicase[Lactobacillusjohnsonii]_gi|329666726|gb|AEB92674.1|putativerestrictionendonuclease[Lac    ---NNL-DKQRDRGTAFEKMVVAYLKNEPAYKQKFKD-VWMLNEVP---------EEY--
gi|489880179|ref|WP_003783646.1|DNAdamageinducibleprotein[Actinomycesnaeslundii]_gi|399901561|gb|EJN84440.1|typeIIIrestriction/    -AQ-----TQRGKGTAFERLVRQFLLTDPRHAERFDE-VWMWSDWP---------ER-G-
gi|496524497|ref|WP_009232780.1|DNAdamageinducibleprotein[Actinomycessp.oraltaxon849]_gi|365265413|gb|EHM95177.1|hypotheticalp/    -AK-----TQRGKGTSFERLVRQFLLTDPRYAERFDE-VWMWSDWP---------DR-G-
gi|915397782|ref|WP_050783167.1|damageinducibleprotein[Actinomycesoris]/11694    -TR-----TQRGKGTAFERLVRQFLLTDPRYAERFDE-VWMWSEWP---------ER-G-
gi|502925248|ref|WP_013160224.1|helicase[Propionibacteriumfreudenreichii]_gi|296921272|emb|CBL55824.1|helicase[Propionibacteriu    -AP-----SNGERGAKFERLMVEYFYTDPLLSVEYDE-VCTWPEWT---------HN-E-
gi|732164352|emb|CEG87481.1|helicase[Propionibacteriumfreudenreichii]/11618    -GS-----TMVDRGERFERLMVDYFTLDPTLSAEYDE-VQRWPDWD---------HR-E-
gi|739047402|ref|WP_036925058.1|damageinducibleprotein[Propionibacteriumthoenii]/11596    -EP-----SAVVRGTRFEELMAGYFSKDATLAATYDE-VWRWPEWP---------HN-E-
gi|739112839|ref|WP_036982533.1|damageinducibleprotein[Propionibacteriumjensenii]/11594    -EP-----SAVVRGTRFEELMAGYFAADATLAAAYDE-VFRWPEWS---------HN-E-
gi|918023558|ref|WP_052314121.1|damageinducibleprotein[Nocardiathailandica]/11612    -AL-----DERDKGDKFERLIKAYLLNEPEWTARFED-VWLWSEWP---------GR-G-
gi|498380271|ref|WP_010694427.1|DNAdamageinducibleprotein[Saccharopolysporaspinosa]/11605    -SL-----DERDKGDKFERLIAAYMRTDPEWQKRFSD-VWLWSEWP---------GR-N-
gi|749991459|ref|WP_040339809.1|damageinducibleprotein[CandidatusBlastococcusmassiliensis]/11597    -AL-----DERDKGSKFEQLIRAYLRTDPEWTARFSD-VWLWSEWP---------GR-Q-
gi|754177801|ref|WP_041796231.1|damageinducibleprotein[Modestobactermarinus]/11592    -SL-----DERDKGSKFERLIRAYLTSDPEWKSRFSD-VWLWSDWP---------ER-A-
gi|504188248|ref|WP_014375350.1|DNAdamageinducibleprotein[Blastococcussaxobsidens]_gi|378782790|emb|CCG02456.1|putativeHelicas/    -SL-----DERDKGSKFEQLIRAYLMTDPEWTSRFSD-VWLWSDWP---------GR-A-
gi|946476713|gb|KRF26798.1|damageinducibleprotein[Phycicoccussp.Soil803]/11609    -GE-----DKTGKGSAFERLMASYLRTAPEFADRFGE-VYLWDEWP---------DK-G-
gi|700176672|gb|KGN37509.1|DNAdamageinducibleprotein[KnoelliasubterraneaKCTC19937]/11633    -GE-----DKTGKGSAFERLMASYLRTAPEFSEQFSE-VYLWQDWP---------ER-G-
gi|700168841|gb|KGN29914.1|hypotheticalproteinN802_10215[KnoelliasinensisKCTC19936]/11631    -GD-----DKTGKGSAFERLMASYLRTAPEFSEQFSE-VYLWQDWP---------ER-G-
gi|946426241|gb|KRE76610.1|damageinducibleprotein[Arthrobactersp.Soil763]/11606    -AL-----SKVDQGTRFERLMKAFLTTDPVYADQFSE-VWLWSEWP---------GN-G-
gi|517318525|ref|WP_018500927.1|hypotheticalprotein[Frankiasp.BCU110501]/11592    -SI-----SETDKGAKFERLMKAYLKIDPVFADQFAD-VWLWSEYP---------DN-N-
gi|499072429|ref|WP_010851327.1|Helicase[Tetrasphaeraelongata]_gi|478756579|emb|CCH71501.1|putativeHelicase[Tetrasphaeraelongat    -AT-----SKADKGSKLERLMAQFLRTDPVYAEQFSD-VWLWQDWP---------DR-G-
gi|750080666|ref|WP_040385350.1|damageinducibleprotein[Demetriaterragena]/11625    -ST-----SEADKGSKFERLIEEFLRTDPVYANQFSQ-VWHWNDWP---------GR-E-
gi|752558292|ref|WP_041229523.1|damageinducibleprotein[Dermacoccusnishinomiyaensis]_gi|664689315|gb|AIF41904.1|damageinducibl/1    -ST-----SSSDQGTKFERLMEAFLRTDPTYAEQFSE-VYAWKEWP---------GN-G-
gi|829089213|ref|WP_047310701.1|damageinducibleprotein[Dermacoccussp.PE3]_gi|828384812|gb|KLO63770.1|damageinducibleprotein[D/1    -AT-----SERDKGSRFEQLMAAYLQTDPLFADQFSD-VWLWQDWP---------GR-D-
gi|748756635|ref|WP_040014750.1|damageinducibleprotein[Dermacoccussp.Ellin185]/11596    -AT-----SERDKGSRFEQLMAAYLQTDPLFADQFSD-VWLWQDWP---------GR-E-
gi|916534724|ref|WP_051141848.1|damageinducibleprotein[Actinomycesneuii]/11632    -SD-----SERLKGNYLEQLTRQYLLNDAQMKRQFCE-VWLWKDWP---------DR-AG
gi|921140030|ref|WP_053090276.1|damageinducibleprotein[Rothiamucilaginosa]/11616    -AL-----HTRDQGDRLERLTKEFLTKDSIQSRLYKK-VYLWKDWD---------GR-EN
gi|896406751|ref|WP_049343749.1|damageinducibleprotein[Rothiamucilaginosa]/11647    -AL-----HTRDQGDRLERLTKEFLTKDSIQSRLYKK-VYLWKDWD---------GR-EN
gi|723608234|gb|AIY01610.1|putativehelicase[Arthrobactersp.PAMC25486]/11613    -------------------MVERFLLTDGVHAPQYTD-VWLWSEWP---------DR-PS
gi|946394527|gb|KRE45160.1|damageinducibleprotein[Arthrobactersp.Soil736]/11616    -AD-----SERMKGSYFEQLIEQYLEHDGVQAPQYTN-VWLWRDWP---------GR-NG
gi|515765304|ref|WP_017197904.1|hypotheticalprotein[Arthrobactersp.M2012083]/11648    -AD-----SERMKGNYFEQLVGRYLEKDGVQAPQYRN-VWLWRDWP---------DR-DG
gi|945315587|gb|KQT73127.1|damageinducibleprotein[Microbacteriumsp.Leaf436]/11612    -AF-----DEYDKGAHFERLMQAFLRTEPQYAELYDE-VWLWSEYP---------ER-G-
gi|759908497|ref|WP_043594599.1|damageinducibleprotein[Frigoribacteriumsp.MEB024]_gi|758271359|gb|KIU03346.1|damageinduciblep/1    -AP-----DKRTKGLLLERLTRTFLTTDPKWTALYDE-VWLWQDWP---------GR-D-
gi|945072850|gb|KQR34237.1|hypotheticalproteinASF75_00055[Curtobacteriumsp.Leaf154]/11597    -AP-----DKRTKGRLFERLTRAYLTTDPKWTARFDE-VWLWQDWP---------DR-N-
gi|551263152|ref|WP_022883363.1|DNAdamageinducibleprotein[Glaciibactersuperstes]/11621    -AR-----DTREKGLLLERLARAYLTTDPVWTARFED-VWLWQDWP---------GR-N-
gi|945452341|gb|KQV08491.1|hypotheticalproteinASC63_10170[Leifsoniasp.Root112D2]/11623    -AR-----DTREKGLLFERLIAQFLRTDPLYSARFDA-VWLWQDWP---------GR-D-
gi|742759483|gb|KIC69912.1|damageinducibleprotein[Kocuriarhizophila]/11610    -AL-----DTRDRGDKFERLMRSYLLTDQAY--SFSK-VWLWNEYP---------ER-G-
gi|737919995|ref|WP_035885304.1|damageinducibleprotein[Kocuriarhizophila]/11611    -AL-----DTRDRGDKFERLMRSYLLTDQAY--SFSK-VWLWNEYP---------ER-G-
gi|737997701|ref|WP_035959325.1|damageinducibleprotein[Kocuriamarina]_gi|725823884|gb|KHE75603.1|damageinducibleprotein[Kocur/1    -AL-----DTRDRGDKFERLMRSYLLTDRAY--SFSK-VWLWNEYP---------ER-G-
gi|664521070|ref|WP_031037650.1|damageinducibleprotein[Streptomycessp.NRRLWC3774]/11624    -IG-----RNRDRGTRFEELILQYLSTDPQWTEQFSR-VWMWTDWP---------GA-EH
gi|500101034|ref|WP_011777041.1|DNAdamageinducibleprotein[Arthrobacteraurescens]_gi|119951547|gb|ABM10457.1|putativeHelicase[A/    -AS-----DERDKGSRFERLIRSYLQLDPQYAEQFSD-VWLWDEWP---------GR-D-
gi|916871953|ref|WP_051479009.1|damageinducibleprotein[Arthrobactersp.H5]/11588    -AP-----SEKEKGSRFERLMKSYLQTEPKYADAYSD-VWLWEEWP---------GR-A-
gi|612084649|gb|EZP43369.1|TypeIIIrestrictionenzyme,ressubunit[Micrococcusluteus]/11625    -EL-----SRQEQGRRFERLAKRFLEVEPKFADQFSD-VWPWNEWP---------GR-E-
gi|759739708|ref|WP_043450422.1|damageinducibleprotein[Arthrobacterphenanthrenivorans]_gi|742758264|gb|KIC68716.1|damageinduc/1    -AT-----DQHDKGTKFEHLMKRYLELEPKYADQFSD-VWLWSEWP---------GR-D-
gi|636845448|ref|WP_024366823.1|damageinducibleprotein[Arthrobactersp.TB26]/11605    -AK-----NERDKGTKFERLFKRYLQLEPKYSDQFSD-VWLWDEWP---------DR-K-
gi|500009025|ref|WP_011689743.1|DNAdamageinducibleprotein[Arthrobactersp.FB24]_gi|116613051|gb|ABK05760.1|typeIIIrestrictionen/    -AK-----NERDKGTKFERLFKRYLQLEPKYSDQFSD-VWLWDEWP---------DR-R-
```

```
gi|821249847|gb|KKZ12083.1|hypotheticalproteinTQ37_06090[CandidatusSynechococcusspongiarum15L]/11436   -AVQATFSRRRDMGTAFERLCTAFLRHDPVQAAQFST-VQTYGEWA---------RQ-R-
gi|739446902|ref|WP_037306885.1|damageinducibleprotein[Ruegeriahalocynthiae]/11592   -AV-----TEREKGTYFERLSLAYLRNDPVQAQQFSD-VWTYSDWA---------KD-N-
gi|640620081|ref|WP_025048131.1|damageinducibleprotein[Sulfitobactermediterraneus]_gi|751956650|gb|KIN77213.1|TypeIIrestricti/   -AV-----TEREKGTYFERLALAYLRNDPIQTQQFSE-VWTYGDWA---------KE-N-
gi|740247634|ref|WP_038088583.1|damageinducibleprotein[Thiobacillusprosperus]_gi|684049748|gb|KFZ90853.1|putativehelicase[Thio/   -SH-----DELHKGKLFERLIVQYLKTDPTYANTLAH-VWTWAEWP---------QR-PA
gi|518420321|ref|WP_019590528.1|hypotheticalprotein[Thioalkalivibriosp.ALE20]/11603   -AQ-----SEREKGGLFERLVANYLMTDPQYADLFDQ-VWLWDEWP---------AR-IG
gi|500156826|ref|WP_011831496.1|DNAdamageinducibleprotein[Methylibiumpetroleiphilum]_gi|124261892|gb|ABM96881.1|adeninespecifi/   -AR-----TNRDLGTSFERLFATYLVTDPQYSDRLSD-VWLWSEWP---------DR-WS
gi|769978537|ref|WP_045094522.1|damageinducibleprotein[Legionellafallonii]_gi|698841736|emb|CEG55682.1|Putativeproteinwithboxh/   -SR-----NNRDKGTQFERLMAKYLMTDPQYADRLED-VWLWSDWP---------DR-DG
gi|553752541|ref|WP_023085344.1|hypotheticalprotein[Pseudomonasaeruginosa]_gi|552201952|gb|ERU62653.1|hypotheticalproteinQ088_0   -AR-----SNREMGDKFERLIAAYLLADPQFSDRLAD-VWLWSEWP---------DR-SG
gi|880980418|ref|WP_048704461.1|damageinducibleprotein[Thauerahumireducens]_gi|875132785|gb|KMU70944.1|damageinducibleprotein/1   -AR-----SNREMGDRFERLIANYLLIDPQYADRLAE-VWLWSEWP---------DR-SG
gi|740135732|ref|WP_037982815.1|damageinducibleprotein[Thauerasp.27]/11603   -AR-----SNREMGDKFERLIANYLLTDPQYADRLAE-VWLWSEWP---------DR-SG
gi|738017371|ref|WP_035978332.1|damageinducibleprotein[Burkholderiaglumae]/11380   -AR-----TNRDKGTHFEYLIANYLMTDPQYADRLSD-VWLWSEWP---------DR-TG
gi|498506003|ref|WP_010806671.1|typeIIrestrictionenzyme,ressubunit[Pandoraeasp.SD62]_gi|490314561|gb|EON12879.1|typeIIrestri/   -AR-----TNRDKGTQFEHLIANYLVTDPQYADRLSD-VWLWSEWP---------DR-TG
gi|500117748|ref|WP_011793753.1|DNAdamageinducibleprotein[Acidovoraxcitrulli]_gi|120587742|gb|ABM31182.1|typeIIrestrictionenz/   -AH-----SNRGKGTQFEKLIANYLLTDPQYADRLAD-VWLWEEWP---------DR-WS
gi|651617328|ref|WP_026611444.1|damageinducibleprotein[Methylocaldumszegediense]/11601   -AQ-----SNHEKGFQFERLIANYLLTDPQYADRLSD-VWLWSEWP---------DR-WD
gi|737210208|ref|WP_035194994.1|damageinducibleprotein[Acidithiobacillusferrivorans]_gi|669953220|emb|CDQ11755.1|Adeninespecif/   -TV-----DQRGKGDAFERLMAAFLRLDPMFADQFAG-VWLWKDWP---------GL-QA
gi|935697386|ref|WP_054506180.1|damageinducibleprotein[Achromobactersp.]_gi|928716153|emb|CUK12836.1|Hefnuclease[Achromobacter/   -SA-----FNRDLGDRFERLMRAYLKLDPKYVGQYEN-VWLWKDWP---------QR-AE
gi|429502517|gb|ELA00827.1|adeninespecificDNAmethyltransferase[Cupriavidussp.HMR1]/11599   -ST-----FNRDLGDRFERLMRAYLKLDPKYVGQYED-VWMWKDWP---------HR-AD
gi|506356642|ref|WP_015876361.1|DNAdamageinducibleprotein[Burkholderiaglumae]_gi|237877158|gb|ACR29491.1|AdeninespecificDNAmet/   -AA-----FNRDLGDRFERLMRAFLKVDPQYMALYED-VWMWKDWP---------QR-ED
gi|696406787|ref|WP_032978003.1|damageinducibleprotein[Bordetellahinzii]_gi|625335076|gb|KCB49343.1|typeIIIrestrictionenzyme,r/   -SA-----FNRDLGDRFERLMRAYLKLDPQYLALFED-VWLWKDWP---------QR-EA
gi|817529125|gb|KKO72587.1|damageinducibleprotein[Kerstersiagyiorum]/11592   -SA-----FNRDLGDRFERLMRAYLKLDPQYLALFED-VWLWKDWP---------QR-EA
gi|737048910|ref|WP_035044118.1|damageinducibleprotein[Aquabacteriumsp.NJ1]_gi|699052896|gb|KGM38706.1|damageinducibleprotein/1   -SA-----FNRDLGDRFERLIRAYLKLDPQYVALFSD-VWLWKDWP---------ER-EA
gi|822492519|ref|WP_046933319.1|MULTISPECIES:damageinducibleprotein[Xanthomonas]_gi|821703525|gb|KLA61196.1|damageinduciblepr/1   -SA-----FNRDLGDRFERLIRAYLKLDPQYVALFSD-VWLWKDWP---------ER-EA
gi|751252861|ref|WP_040963740.1|damageinducibleprotein[Xanthomonasfuscans]_gi|749808363|gb|KIJ03887.1|damageinducibleprotein[/1   -SA-----FNRDLGDRFERLIRAYLKLDPQYVALFSD-VWMWKDWP---------ER-EA
gi|496774980|ref|WP_009368792.1|DNAdamageinducibleprotein[Bilophilasp.4_1_30]_gi|345039902|gb|EGW44205.1|hypotheticalproteinHM/   -QQ-----FNKDKGDAFERLVCAWLKCDPQYQSIFSD-VWLWKDWP---------QR-KE
gi|499241912|ref|WP_010939452.1|DNAdamageinducibleprotein[Desulfovibriovulgaris]_gi|46580580|ref|YP_011388.1|adeninespecificDN/   -QE-----YQRDKGDAFERLIRAYLKTDPQYQALFSD-VWLWKDWP---------ER-EA
gi|504274913|ref|WP_014462015.1|DNAdamageinducibleprotein[Bacillusmegaterium]_gi|345446919|gb|AEN91936.1|AdeninespecificDNAmet/   -SK-----SEREKGGRFEQLMVQYFKTDPLYKDKFNN-VWLWNDFP---------LR-E-
gi|504895966|ref|WP_015083068.1|typeIIrestrictionenzymeandmethyltransferasesubtypeG[Anabaenasp.90]_gi|413972424|gb|AFW96512.1|t   -ST-----SPRELGDKFERLMLAYLKTENFYKDHFSQ-VWLWMDFP---------KR-G-
gi|518327765|ref|WP_019497972.1|hypotheticalprotein[Calothrixsp.PCC7103]/11600   -AV-----SNRDLGDKFERLMLNYLKVDPLYQDLYSD-VWTWMDFP---------LR-G-
gi|652765503|ref|WP_027079917.1|damageinducibleprotein[Luteimonasmephitis]/11593   -AS-----DHRDKGDRFERLIRAYLTTDPLYVARFSH-VWLWTDWP---------DR-K-
gi|896539951|ref|WP_049442123.1|damageinducibleprotein[Stenotrophomonasmaltophilia]/11591   -AT-----DHRDKGGKFERLMKAYFLTDPLYVSRFSD-VWLWNEWP---------GR-K-
gi|500217900|ref|WP_011888043.1|DNAdamageinducibleprotein[Geobacillusthermodenitrificans]_gi|134267954|gb|ABO68149.1|Conserved/   -AR-----NKREQGELFERLMVSYLRTEPHYKQLFSN-VWRWMDWP---------GR-E-
gi|696570298|ref|WP_033102014.1|damageinducibleprotein[Thermoactinomycesdaqus]/11594   -AR-----NTREQGDLFERLMVSFLQTDPQYKQLFSN-VWRWMDWP---------SR-D-
gi|251772067|gb|EES52637.1|putativehelicase[Leptospirillumferrodiazotrophum]/11618   -AK-----SEREKGDLFERLVVAFLKTDPLFRDRFED-VWIWKEWP---------DL-YS
gi|738124101|ref|WP_036082108.1|damageinducibleprotein[Leptospirillumferriphilum]_gi|685243306|gb|KGA94177.1|putativehelicase[/   -AK-----SEREKGDLFERLVVAFLKTDPLFGDRFEG-VWIWKEWP---------DL-HN
gi|558699223|ref|WP_023524766.1|helicase[Leptospirillumsp.GroupII'CF1']_gi|206603764|gb|EDZ40244.1|Putativehelicase[Leptospiri/   -AK-----SEREKGDLFERLVVAFLKTDPLFRDRFED-VWIWKEWP---------DL-YL
gi|406774462|gb|AFS53887.1|putativehelicase[LeptospirillumferriphilumML04]/11610   -SQ-----SEREKGDRFEILMKAFFKADPTWADRFED-VWIWKEWP---------EL-AH
gi|740719463|ref|WP_038504749.1|damageinducibleprotein[Leptospirillumferriphilum]_gi|640838125|gb|AIA30173.1|damageinduciblep/1   -SR-----SEREKGDRFEILMKAFFKTDPTWVDRFED-VWIWKEWP---------DL-SR
gi|517810766|ref|WP_018980974.1|hypotheticalprotein[Saccharibacterfloricola]/11733   -SP-----TNRDRGTNFEQLCLTYLKNSPRWKALFQA-VKTYAQWA---------AEHAE
gi|502471675|ref|WP_012798533.1|helicase[Slackiaheliotrinireducens]_gi|256791761|gb|ACV22431.1|predictedhelicase[Slackiaheliotr   -AE-----SESDKGTKFERASRYYLKNDPLYSARFTD-VWMWKDAP---------TN---
gi|657871713|ref|WP_029576297.1|hypotheticalprotein[Bifidobacteriumthermacidophilum]_gi|673007521|gb|KFJ02535.1|helicase[Bifido   -AA-----DKRQRGTDFERLVKWFLQHDPSWQSKLQD-VWMWDEAP---------TN---
gi|673004757|gb|KFI99791.1|DNAhelicaserestrictionenzymetypeIIIRessubunit[Bifidobacteriumthermacidophilumsubsp.porcinum]/11682   -AA-----DKRQRGTDFERLVKWFLQHDPSWQSKLQD-VWMWDEAP---------TN---
gi|917316092|ref|WP_051922804.1|hypotheticalprotein[Bifidobacteriumstellenboschense]_gi|673003774|gb|KFI98812.1|helicase[Bifido   -SV-----DKREQGTRFEHAVEFFLEHDPSWLERITQ-VWLWDEAP---------TN---
gi|705415694|ref|WP_033502332.1|hypotheticalprotein[Bifidobacteriumpseudolongum]_gi|672982411|gb|KFI77685.1|DNAdamageinducible/   -SV-----DKREQGTRFERLVQWFLSNDPSWLERVSR-VWLWDDAP---------EE-L-
gi|916591418|ref|WP_051198509.1|hypotheticalprotein[Bifidobacteriumsp.AGR2158]/11704   -SV-----DTRQQGTWFEYTVKWFLENDPSWVERITE-VWMWDDAP---------EA-L-
gi|772242982|gb|AJW78255.1|hypotheticalproteinVO01_03165[Clavibactermichiganensissubsp.insidiosus]/11642   -AD-----SERMKGNYLEQLAKHFLTVEPLWADQLGT-VWLWKDWP---------GR---
gi|916649818|ref|WP_051256909.1|damageinducibleprotein[Actinomycesgraevenitzii]/11677   -AT-----SEREKGAMFEELTRQFLLHDARFAHQFKE-VYLWSEWP---------ER---
gi|496504578|ref|WP_009212861.1|helicase[Actinomycessp.oraltaxon180]_gi|315312695|gb|EFU60776.1|helicase[Actinomycessp.oraltaxo   -AK-----SEREKGTLFEELTRQFLLHDARFAHQFKE-IYLWSEWP---------DR---
gi|494862206|ref|WP_007588306.1|helicase[Actinomycessp.ICM39]_gi|398362799|gb|EJN46470.1|typeIIIrestrictionenzyme,ressubunit[Ac   -AK-----SEREKGTLFEELTRQFLLHDARFAHQFKE-IYLWGEWP---------ER---
gi|881057111|ref|WP_048774974.1|damageinducibleprotein[Sanguibacterkeddieii]/11697   -AK-----SEREKGTLFEELTRQFLLHDARFAHQFKE-IYLWSEWP---------EH---
gi|881020459|ref|WP_048740808.1|damageinducibleprotein[Kytococcussedentarius]/11704   -AR-----SEREKGTLFEELTRQFLLHDARFAHQFKE-IFLWGEWP---------ER---
gi|881052313|ref|WP_048770555.1|damageinducibleprotein[Cellulomonasflavigena]/11705   -AR-----SEREKGTLFEELTRQFLSHDARFAHQFKE-IYLWSEWP---------ER---
gi|490108682|ref|WP_004009424.1|helicase[Mobiluncuscurtisii]_gi|315490465|gb|EFU80089.1|helicaseCterminaldomainprotein[Mobilun/   -SD-----SERAKGNLFEQLILSYLRNDSQMSHQFGE-VYLWRDWP---------GG-GK
gi|490111935|ref|WP_004012603.1|helicase[Mobiluncusmulieris]_gi|227843812|gb|EEJ53982.1|helicaseCterminaldomainprotein[Mobilun/   -AR-----SQREKGNLFEQLVRAYLRLDSQMRLQFAR-VYAWRDWP---------GA---
gi|490114228|ref|WP_004014848.1|helicase[Mobiluncusmulieris]_gi|269934432|gb|EEZ90993.1|helicaseCterminaldomainprotein[Mobilun/   -AR-----SQREKGNLFEQLVRAYLRLDSQMRLQFAR-VYAWRDWP---------GA---
gi|493333551|ref|WP_006290636.1|helicase[Parascardoviadenticolens]_gi|315119487|gb|EFT82620.1|helicaseCterminaldomainprotein[P/   -AK-----TEHQKGMLWERVSRWYLRNDPEMQQFIGR-VWKATDPD---------CP-IA
gi|495817067|ref|WP_008541646.1|helicase,partial[Sutterellaparvirubra]_gi|378598583|gb|EHY31712.1|helicaseprotein,partial[Sutte   -SIEASSLSARDKGTRFETLIRDWLMKEPTYANLFTK-VQTWKDWS---------AENPG
gi|491572696|ref|WP_005430278.1|helicase[Sutterellawadsworthensis]_gi|319805116|gb|EFW01943.1|DNAhelicaserestrictionenzymeTypeI   -IKQASNNIKRDAGTRFETLIKDWLTQDQSYCDLFSK-VETFEEWA---------SSHPE
gi|748686321|ref|WP_039944445.1|hypotheticalprotein[Thermicanusaegyptius]/11573   -AT-----NTVEKGEIFERFVIAFLKNDPYYKEEFID-VWKWYDWP---------DRDK-
gi|916875698|ref|WP_051482754.1|hypotheticalprotein[Thioalkalivibriosp.HK1]/11366   -D------TLVMQGRLFERLMKRWLSVDPLYSGRFLE-VYSWGEWA---------SDRSD
gi|821248604|gb|KKZ10960.1|hypotheticalproteinTE42_09075[CandidatusSynechococcusspongiarumSP3]/11598   -AAQASFSRSRDMGTAFEDLCAAFLTHDPVQKAQFSM-VQSYGEWA---------RQ-R-
gi|737409843|ref|WP_035390956.1|hypotheticalprotein[Ferrimicrobiumacidiphilum]_gi|765544266|gb|KJE76241.1|typeIrestrictionenzym   -SA-----SFSQQGTAFERMMRTYLQTDPLYSDRFEK-VFSWMEWP---------DRPA-
gi|654906925|ref|WP_028358281.1|damageinducibleprotein[Brackiellaoedipodis]/11679   -PV-----TRRDQGTDFEHLCCVYLRNEPFYQDLYAE-VWTYGQWA---------AT-Q-
gi|502809901|ref|WP_013044877.1|hypotheticalprotein[CandidatusPuniceispirillummarinum]_gi|292663146|gb|ADE38247.1|hypotheticalp   -AR-----NTREQGTLFEDLMEAILPQLPD--AGFEE-VWSWKAWP---------DRHAQ
gi|251771370|gb|EES51950.1|typeIIrestrictionenzyme,ressubunit[Leptospirillumferrodiazotrophum]/11628   -AK-----DKRALGDAFERLIRSFLKTAPEYARRFEE-AWLWKDCP---------HL-AE
gi|498328480|ref|WP_010642636.1|DNAdamageinducibleprotein[Acidithiobacillusthiooxidans]/11627   -AK-----DAHHKGMLFERFMVAYLKTDPVYADTLEA-VWQYKDWP---------DRPDH
gi|647633864|ref|WP_025906698.1|damageinducibleprotein[Prochlorococcussp.scB245a_519B7]/11622   -AI-----NDKQLGDTFEQLAKVYLENDDIQIQEYSN-VWRYEDWA---------KENPT
gi|647619599|ref|WP_025893729.1|damageinducibleprotein[Prochlorococcussp.scB241_527N11]/11621   -AN-----SDTEMGLAFEKLSKVFLERDDTQKQQYSQ-VWHFADWA---------KERKE
```

```
gi|755034887|ref|WP_042387498.1|hypotheticalprotein[Clostridiumsp.CL6]/11606    -YK-----NQRDKGTSFEQLIKSYLLIEPKYSSYIEQ-VWTWKEFP---------YR-NA
gi|498139798|ref|WP_010453954.1|helicase[SuccinivibrionaceaebacteriumWG1]/11662    -SN-----STKEMGTKFERLIKNWFQTTYLYCNDIAE-IWLWNDFP---------YR-AQ
gi|527501386|gb|EPT34571.1|typeIIIrestrictionenzyme,ressubunit[Bacteroidetesbacteriumoraltaxon272str.F0290]/11789    -SA-----TELEKGHKFEQLIQRWLLSDPRYSNTVKR-VWLWNEFP---------AK-AE
gi|496047275|ref|WP_008771782.1|helicase[Bacteroidessp.2_1_33B]_gi|262296443|gb|EEY84373.1|helicaseCterminaldomainprotein[Bact/    -SF-----TEREKGAKFEKLMKRWFQTDPRYADKLQE-VWLWEEFP---------GK-KD
PDI8503III/11664    -SF-----TEREKGAKFEKLMKRWFQTDPRYADKLQE-VWLWEEFP---------GK-KD
gi|738719431|ref|WP_036613715.1|helicase[Parabacteroidesdistasonis]_gi|649542518|gb|KDS49387.1|restrictionendonucleasefamilypro    -SF-----TEREKGAKFEKLMKRWFQTDPRYADKLQE-VWLWEEFP---------GK-KD
gi|659417033|gb|KEJ86133.1|hypotheticalproteinHMPREF1002_00341[Porphyromonassp.31_2]/11656    -SF-----TEREKGAKFEKLMKRWFQTDPRYADKLQE-VWLWEEFP---------GK-KD
gi|567456807|gb|ETK11358.1|hypotheticalproteinT235_15845[Tannerellasp.oraltaxonBU063isolateCell8/11]/11433    -SF-----SEKYKGIRFERLMRAWLKTDPRYTDLFET-VGLWEEFP---------FR-DE
gi|545440279|ref|WP_021677877.1|helicase[Porphyromonasgingivalis]_gi|543985765|gb|ERJ89108.1|helicaseprotein[Porphyromonasgingi    -SF-----TEKNKGTQFERLMRAWLRTDARYANLFET-VWLWEEFP---------SR-SD
gi|497438056|ref|WP_009752254.1|helicase[Prevotellasp.oraltaxon473]_gi|429144866|gb|EKX87968.1|helicaseprotein[Prevotellasp.ora    -SF-----TEKEKGTQFERLMRAWLRTDPRYADRFEH-VWLWEDFP---------AR-QD
gi|490423474|ref|WP_004295683.1|MULTISPECIES:helicase[Bacteroides]_gi|156110098|gb|EDO11843.1|helicaseCterminaldomainprotein[B/    -SF-----TQKDKGTQFERLMRSWLLSDPRYSN-LTK-VWLWDDFP---------SR-AD
gi|941777229|ref|WP_055173506.1|helicase[Bacteroidescaccae]_gi|933263395|emb|CUQ00567.1|Predictedhelicase[Bacteroidescaccae]/1/    -SF-----TEKEKGTKFERLMRSWLLTDPRYNE-LES-VWLWEDFP---------GR-ND
gi|492413898|ref|WP_005836892.1|helicase[Bacteroidesuniformis]_gi|392678824|gb|EIY72229.1|hypotheticalproteinHMPREF1072_03583[B    -SF-----TEREKGAKFERLMRSWLLTDPRYNE-LES-VWLWEDFP---------GR-ND
gi|695455095|ref|WP_032566191.1|helicase[Bacteroidesfragilis]_gi|596095426|gb|EXZ59996.1|restrictionendonucleasefamilyprotein[B    -SF-----TEREKGTKFERLMRSWLLTDPRYNE-LES-VWLWEDFP---------GR-KD
gi|933227941|emb|CUQ02348.1|Predictedhelicase[Bacteroidesuniformis]/11658    -SF-----TEKEKGTKFERLMRSWLLTDPRYNE-LEK-VWLWEDFP---------GR-KD
gi|494823013|ref|WP_007558421.1|helicase[Bacteroidesplebeius]_gi|198272167|gb|EDY96436.1|helicaseCterminaldomainprotein[Bacter/    -SF-----TEKEKGTKFERLMRSWLLTDPRYNE-LES-VWLWEDFP---------GR-ND
gi|495427212|ref|WP_008151909.1|helicase[Parabacteroidesjohnsonii]_gi|218222381|gb|EEC95031.1|helicaseCterminaldomainprotein[P/    -SF-----TEKEKGTKFERLMRSWLLTDPRYNE-LEK-VWLWEDFP---------GR-KD
gi|933196855|emb|CUO94545.1|typeIrestrictionenzymeEcoKIsubunitR[Bacteroidesuniformis]_gi|933220098|emb|CUN86874.1|typeIrestrict    -SF-----TEKEKGTKFERLMRSWLLTDPRYNE-LEK-VWLWEEFP---------GR-KD
gi|823687748|ref|WP_047101121.1|helicase[Brachyspirahyodysenteriae]_gi|823334537|gb|KLI17932.1|helicase[Brachyspirahyodysenteri    -TI-----STVDKGHRFEDLIRNYLLTDTKY--NFKE-VYYWSEFP---------FK-ND
gi|496436691|ref|WP_009145538.1|helicase[Phascolarctobacteriumsuccinatutens]_gi|322414007|gb|EFY04839.1|helicaseCterminaldomai/    -AY-----TEHDKGTRFERLICNYLKTSKKYQILLKE-VWLWNEFP---------YR-KE
gi|503372880|ref|WP_013607541.1|helicase[Sphaerochaetaglobosa]_gi|324026933|gb|ADY13692.1|typeIIIrestrictionproteinressubunit[S    -SF-----DMRDQGSRFEKLMKAYLLTEPQYRGRFQE-VWLWNEFP---------SR-KD
gi|505821748|ref|WP_015707145.1|helicase[Treponemaprimitia]_gi|299483503|gb|ADJ19584.1|helicasedomaincontainingprotein[Trepone/    -AF-----SERDKGYRFEKLMQEYLKSDPLYAAQWSN-VWLWGDFP---------SR-KD
gi|505822741|ref|WP_015708134.1|helicase[Treponemaprimitia]_gi|333739639|gb|AEF85129.1|endonucleaseandmethylaseLlaGI[Treponemap    -AF-----SERDKGYRFEKLMQEYLKSDPLYAAQWSN-VWLWGDFP---------SR-ND
gi|488791994|ref|WP_002704400.1|helicase[Treponemasaccharophilum]_gi|380767691|gb|EIC01689.1|typeIIIrestrictionproteinressubuni    -SF-----SERDKGDRFEKLIKAYLLTKPEYKSQLSD-VWLWSEFP---------EK-SQ
gi|524671454|emb|CDD81750.1|putativeseptumsitedeterminingproteinMinC[Bacteroidessp.CAG:462]/11642    -AW-----SERDKGFRFERLMQAYLKTASLYAGRFEE-VWLWSEFP---------CH-DQ
gi|524573865|emb|CDC95766.1|putativeseptumsitedeterminingproteinMinC[Alistipessp.CAG:268]/11639    -AF-----SERDKGYRFERLMQAYLKTTTLYANLFEE-VWLWTEFP---------FH-DQ
gi|658617970|ref|WP_029759328.1|helicase[Fusobacteriumnucleatum]/11606    -SH-----SQRDKGTKFELLIKNWFLTTKLYSDNIKE-IWLWDEFP---------YR-NQ
gi|494412037|ref|WP_007216782.1|helicase[Bacteroidescellulosilyticus]_gi|392638921|gb|EIY32752.1|hypotheticalproteinHMPREF1062_    -SF-----SERDKGTRFERLMQSYLQTDPMYAPLFSH-VWLWEEFP---------SR-AD
gi|383107102|gb|AFG37435.1|putativehelicase[SpirochaetaafricanaDSM8902]/11616    -SF-----SERDKGERFERLMQAYLKTDPKYADRFKK-VWMWNEFP---------YR-KD
SAF8902III/11616    -SF-----SERDKGERFERLMQAYLKTDPKYADRFKK-VWMWNEFP---------YR-KD
gi|652538894|ref|WP_026932796.1|helicase[Gramellaechinicola]/11633    -SF-----SERDKGERFERLIQAYLQTDPKYSNQFKY-VWLWNEFP---------GK-KD
gi|639238223|ref|WP_024568839.1|helicase[Elizabethkingiaanophelis]/11628    -SF-----SERDKGDRFETLIQNYFLTDPKYSYNLKK-VWLWNEFP---------SK-TD
gi|652520381|ref|WP_026914582.1|helicase[Gramellaportivictoriae]/11623    -SF-----SERDKGERFEKLVHAYLQTDPAYSGILKY-VWMWNEFP---------SK-SD
gi|494922183|ref|WP_007648219.1|Helicase[Formosasp.AK20]_gi|469825489|gb|EMQ95550.1|Helicasedomainprotein[Formosasp.AK20]/11626    -SF-----SERDKGDRFERLMQAYLQTDPTYSNTLKY-VWMWNEFP---------GK-KD
gi|522083382|ref|WP_020594591.1|hypotheticalprotein[Spirosomapanaciterrae]/11637    -AF-----SEKDKGERFERLMKAFLLTYPKYADTFKK-VWLWNEFP---------GK-SD
gi|490455352|ref|WP_004326157.1|helicase[Bacteroidesovatus]_gi|392669277|gb|EIY62768.1|hypotheticalproteinHMPREF1070_03635[Bact    -AF-----SERDKGDKFEKLMQAYLQTDPKYATFFSK-VWLWNEFP---------FR-KD
gi|499516035|ref|WP_011202675.1|helicase[Bacteroidesfragilis]_gi|53713270|ref|YP_099262.1|helicasedomainprotein[Bacteroidesfrag    -AF-----SERDKGDKFEKLMQAYLQTDPKYATFFSK-VWLWNEFP---------FR-KD
gi|651338332|ref|WP_026461320.1|helicase[Adhaeribacteraquaticus]/11620    -AF-----SERDKGERFERLMQAYLKTDPKYAYLFKK-VWLWNEFP---------GK-SD
gi|806964642|gb|AKC17139.1|helicase[Flavobacteriumpsychrophilum]/11639    -SF-----SERDKGDRFERLIQEYLLTEPLYANRLKK-VWMWNDFP---------AK-ND
gi|752684374|ref|WP_041332322.1|helicase[Flavobacteriumpsychrophilum]_gi|666882889|gb|AIG30715.1|helicase[Flavobacteriumpsychro    -SF-----SERDKGDRFERLIQEYLLTEPLYANRLKK-VWMWNDFP---------AK-ND
gi|933196595|emb|CUP04894.1|DNAorRNAhelicasesofsuperfamilyII[Bacteroidesuniformis]/11631    -SY-----SEKDKGERFERLMQAYLLTDPQYAYKFKK-VWLWSEFH---------GK-DD
gi|670509109|ref|WP_031456918.1|helicase[Flavobacteriumchungangense]/11625    -SF-----SERDKGARFERLMQAYLLTDPQYAYRFKK-VWMWDEFP---------GK-ND

gi|490190857|ref|WP_004089451.1|hypotheticalprotein[Helicobacterbilis]_gi|476630638|gb|EMZ37196.1|hypotheticalproteinC826_02193    ----DKKKDTGIDIVIETK------------EN-------DFIAVQCKFYTKN--KLQLS
gi|488896133|ref|WP_002807242.1|MULTISPECIES:restrictionendonucleaseEco57I[Campylobacter]_gi|380541096|gb|EIA65377.1|hypothetic    -----NGQDCGIDLIIQTK------------DD-------DYIAVQCKFYESS--KVDLK
gi|746602240|ref|WP_039628365.1|restrictionendonucleaseEco57I[Campylobacterlari]_gi|744819395|gb|AJD03391.1|putativehelicase[Ca    -----DGQDRGIDLIIQTK------------DD-------DYIAVQCKFYENS--KVDFK
gi|727317135|ref|WP_033772558.1|restrictionendonucleaseEco57I[Helicobacterpylori]/11622    ----GNKSDCGIDMVIQTT------------SK-------EYIAVQCKFHQD---SVSLN
gi|727103490|ref|WP_033618954.1|restrictionendonucleaseEco57I[Helicobacterpylori]/11616    ----GNKSDCGIDMVIQTT------------SK-------EYIAVQCKFHQN---SVSLN
gi|727340205|ref|WP_033794408.1|restrictionendonucleaseEco57I[Helicobacterpylori]/11609    ----GNKSDCGIDMVIQTT------------SK-------EYIAVQCKFHQD---SVSLN
gi|727292407|ref|WP_033749975.1|restrictionendonucleaseEco57I[Helicobacterpylori]/11611    ----GNKNDCGIDMVIQTT------------SK-------EYIAVQCKFHQD---SVSLN
gi|727289795|ref|WP_033747591.1|restrictionendonucleaseEco57I[Helicobacterpylori]/11612    ----GKERDKGIDIVIQTT------------SK-------EYIAVQCKFHQN---SISYN
gi|727319676|ref|WP_033775047.1|restrictionendonucleaseEco57I[Helicobacterpylori]/11612    ----GNKSDCGIDMVIQTT------------SK-------EYIAVQCKFHQN---SVSLN
gi|447212910|ref|WP_001290166.1|restrictionendonucleaseEco57I[Helicobacterpylori]_gi|308063540|gb|ADO05427.1|hypotheticalprotei    ----GNEGDRGIDMVITTT------------SK-------EYIAVQCKFHQD---SVSLN
gi|447212901|ref|WP_001290157.1|restrictionendonucleaseEco57I[Helicobacterpylori]_gi|384557329|gb|AFH97797.1|hypotheticalprotei    ----GNEGDRGIDMVITTT------------SK-------EYIAVQCKFYQD---SVSLN
gi|515849671|ref|WP_017280424.1|hypotheticalprotein[Helicobacterpylori]/11615    ----GNERDHGIDMVITTA------------SK-------EYIAVQCKFHQD---SVSLN
gi|447212900|ref|WP_001290156.1|restrictionendonucleaseEco57I[Helicobacterpylori]_gi|317178970|dbj|BAJ56758.1|TypeIIGrestrictio    ----NNERDRGIDIVITTA------------SK-------EYIAVQCKFHQD---SVSLN
gi|447079018|ref|WP_001156274.1|hypotheticalprotein[Helicobacterpylori]/11614    ----GKERGGGIDMVITTA------------SK-------EYIAVQCKFHQN---SISLN
gi|447212800|ref|WP_001290056.1|hypotheticalprotein[Helicobacterpylori]_gi|532153248|gb|EQL67316.1|restrictionendonucleaseEco57    ----GKEGDRGIDMVVTTT------------SK-------EYIAVQCKYHQN---NISLN
gi|505457237|ref|WP_015644339.1|hypotheticalprotein[Helicobacterpylori]_gi|499061160|gb|AGL70074.1|restrictionendonucleaseEco57    ----GKEGDRGIDMVVTTT------------SK-------EYIAVQCKYHQN---NISLN
HPYUM037X/11591    ----GKEGDRGIDMVVTTT------------SK-------EYIAVQCKYHQN---NISLN
gi|920026297|ref|WP_052937176.1|restrictionendonuclease[Helicobacterpylori]_gi|912528678|gb|KNX48238.1|restrictionendonucleaseE    ----GKEGDRGIDMVVTTT------------SK-------EYIAVQCKYHQN---NISLN
gi|915539050|ref|WP_050841327.1|restrictionendonuclease[Helicobacterpylori]_gi|908890887|gb|KNE06183.1|restrictionendonucleaseE    ----GKERDRGIDMVITTA------------SK-------EYIAVQCKFHQN---SISLN
gi|727101397|ref|WP_033616957.1|restrictionendonucleaseEco57I[Helicobacterpylori]/11592    ----GNKGDRGIDMVITTA------------SK-------EYIAVQCKFHQD---SISLN
gi|562814875|emb|CDK13002.1|TypeISPRMsystem[Helicobacterpylori26695]/11588    ----GNERDKGIDIVITPS------------NK-------EYIAVQCKFHQN---SISYN
HPYAXVIII/11588    ----GNERDKGIDIVITPS------------NK-------EYIAVQCKFHQN---SISYN
gi|447079029|ref|WP_001156285.1|restrictionendonucleaseEco57I[Helicobacterpylori]_gi|393085956|gb|EJB86635.1|hypotheticalprotei    ----GKECDRGIDIVIQTA------------SK-------EYIAVQCKFHQN---SISYN
```

```
gi|489105953|ref|WP_003015814.1|restrictionendonuclease[Helicobacterpylori]_gi|477503388|gb|ENH59950.1|restrictionendonucleasef      ----NNEGDRGIDIVIQTA------------SK-------EYIAVQCKFHQN---SISYP
gi|447079006|ref|WP_001156262.1|restrictionendonucleaseEco57I[Helicobacterpylori]_gi|393067470|gb|EJB68280.1|hypotheticalprotei      ----GKERDKGIDIVIQTT------------SK-------EYIAVQCKYHQD---SISLN
gi|727314589|ref|WP_033770298.1|restrictionendonucleaseEco57I[Helicobacterpylori]/11586                                               ----GKERDRGIDMVITTA------------SK-------EYIAVQCKFHQN---SISLN
gi|447079019|ref|WP_001156275.1|restrictionendonucleaseEco57I[Helicobacterpylori]_gi|308065028|gb|ADO06920.1|hypotheticalprotei      ----GNEGDRGIDMVITTT------------SK-------EYIAVQCKFHQD---SVSLN
gi|447079028|ref|WP_001156284.1|restrictionendonucleaseEco57I[Helicobacterpylori]_gi|393076030|gb|EJB76784.1|hypotheticalprotei      ----GKERDKGIDIVITTS------------NK-------ECIAVQCKFHQN---SISYN
gi|393034009|gb|EJB35070.1|hypotheticalproteinHPNQ4053_0631[HelicobacterpyloriNQ4053]/11514                                         ----GKERDKGIDIVIQTT------------SK-------EYIAVQCKFHQN---SISYN
gi|447079004|ref|WP_001156260.1|restrictionendonucleaseEco57I[Helicobacterpylori]_gi|384552418|gb|AFI07366.1|hypotheticalprotei      ----GKERDKGIDIVITTS------------NK-------ECIAVQCKFHQN---SISYN
gi|746011306|ref|WP_039088215.1|restrictionendonucleaseEco57I[Helicobacterpylori]_gi|733488720|gb|KHL85979.1|restrictionendonuc      ----GKERDKGIDIVITTS------------NK-------EYIAVQCKFHQN---SISYN
gi|531081162|gb|EQD92166.1|restrictionendonucleaseEco57I[HelicobacterpyloriPZ5080]/11509                                            ----GKERDKGIDIVITTS------------NK-------EHIAVQCKFHQT---AISYN
gi|544760796|ref|WP_021187395.1|restrictionendonucleaseEco57I[Helicobacterpylori]_gi|531086508|gb|EQD96745.1|restrictionendonuc      ----GKERDKGIDIVITTS------------NK-------EYIAVQCKFHQN---SISYN
gi|447079012|ref|WP_001156268.1|restrictionendonucleaseEco57I[Helicobacterpylori]_gi|393028199|gb|EJB29286.1|hypotheticalprotei      ----GKERDKGIDIVIQTT------------SK-------EYIAVQCKFHQN---SISYN
gi|746008458|ref|WP_039085555.1|restrictionendonucleaseEco57I[Helicobacterpylori]_gi|733480358|gb|KHL78026.1|restrictionendonuc      ----GKERDRGIDIVIQTA------------SK-------EFIAVQCKFHQN---SISLN
gi|447079011|ref|WP_001156267.1|restrictionendonucleaseEco57I[Helicobacterpylori]_gi|393038534|gb|EJB39568.1|hypotheticalprotei      ----GKERDKGIDIVIQTA------------SK-------EYIAVQCKFHQN---SISLN
gi|393037163|gb|EJB38199.1|hypotheticalproteinHPNQ4161_0422[HelicobacterpyloriNQ4161]/11556                                         ----GNERDKGIDIVIQTT------------SK-------EYIAVQCKFHQN---SISYN
gi|447079007|ref|WP_001156263.1|restrictionendonucleaseEco57I[Helicobacterpylori]_gi|393051817|gb|EJB52768.1|hypotheticalprotei      ----GKERDKGIDIVITTS------------DK-------EYIAVQCKFHQN---SISYN
gi|487867094|ref|WP_001940560.1|helicase[Helicobacterpylori]_gi|459505657|gb|EMG98418.1|helicaseprotein[HelicobacterpyloriGAM21      ----GKERDKGIDIVIQTT------------SK-------EYIAVQCKFHQN---SVSYN
gi|487872094|ref|WP_001945560.1|helicase[Helicobacterpylori]_gi|459512104|gb|EMH04523.1|helicaseprotein[HelicobacterpyloriGAM24      ----GKERDKGIDIVITTS------------NE-------EYIAVQCKFHQN---SVSYN
gi|487880228|ref|WP_001953694.1|helicase[Helicobacterpylori]_gi|459529131|gb|EMH20734.1|helicaseprotein[HelicobacterpyloriGAM26      ----GKERDKGIDIVITTS------------NK-------EYIAVQCKFHQN---SVSLN
gi|487882593|ref|WP_001956059.1|helicase[Helicobacterpylori]_gi|459533462|gb|EMH24859.1|helicaseprotein[HelicobacterpyloriGAM26      ----GKERDKGIDIVITTS------------NK-------EYIAVQCKFHQN---SISYN
gi|487845430|ref|WP_001918896.1|helicase[Helicobacterpylori]_gi|410713871|gb|EKQ71363.1|helicaseprotein[HelicobacterpyloriGAM10      ----GKERDKGIDIVITTS------------NK-------EYIAVQCKFHQN---SISYN
gi|447079005|ref|WP_001156261.1|restrictionendonucleaseEco57I[Helicobacterpylori]_gi|393069830|gb|EJB70625.1|hypotheticalprotei      ----GKERDKGIDIVIQTT------------SK-------EYIAVQCKFHQN---SISYN
gi|459506808|gb|EMG99528.1|helicaseprotein[HelicobacterpyloriGAM121Aii]/11545                                                      ----GKERDKGIDIVITTS------------NK-------EYIAVQCKFHQN---SVSYN
gi|487825283|ref|WP_001898749.1|restrictionendonucleaseEco57I[Helicobacterpylori]_gi|393107526|gb|EJC08068.1|hypotheticalprotei      ----GKERDKGIDIVIQTT------------SK-------EYIAVQCKFHQN---SVSYN
gi|447079008|ref|WP_001156264.1|restrictionendonucleaseEco57I[Helicobacterpylori]_gi|393058636|gb|EJB59524.1|hypotheticalprotei      ----GKERDKGIDIVITTS------------NK-------EYIAVQCKFHQN---SISYN
gi|447079015|ref|WP_001156271.1|restrictionendonucleaseEco57I[Helicobacterpylori]_gi|307637357|gb|ADN79807.1|hypotheticalprotei      ----GKERDKGIDIVITTS------------NQ-------EYIAVQCKFHQN---SISYN
gi|487884861|ref|WP_001958327.1|helicase[Helicobacterpylori]_gi|459535535|gb|EMH26834.1|helicaseprotein[HelicobacterpyloriGAM26      ----GKERDKGIDIVIQTT------------SK-------EYIAVQCKFHQN---SVSLN
gi|487886365|ref|WP_001959831.1|helicase[Helicobacterpylori]_gi|459538727|gb|EMH29861.1|helicaseprotein[HelicobacterpyloriGAM27      ----GKERDKGIDIVITTS------------NK-------EYIAVQCKFHQN---SISYN
gi|545301637|ref|WP_021581412.1|helicase[Helicobacterpylori]_gi|535931889|gb|ERA61745.1|helicaseprotein[HelicobacterpyloriGAM11      ----GKERDKGIDIVIQTT------------SK-------EYIAVQCKFHQN---SVSYN
gi|447079010|ref|WP_001156266.1|restrictionendonucleaseEco57I[Helicobacterpylori]_gi|393095477|gb|EJB96081.1|hypotheticalprotei      ----GKERDKGIDIVIQTT------------SK-------EYIAVQCKFHQN---SISYN
gi|447079009|ref|WP_001156265.1|restrictionendonucleaseEco57I[Helicobacterpylori]_gi|393056294|gb|EJB57206.1|hypotheticalprotei      ----GKERDKGIDIVIQTT------------SK-------EYIAVQCKFHQN---SISYN
gi|471386985|gb|EMR58906.1|restrictionendonucleasefamilyprotein[HelicobacterpyloriHpH1]/11504                                       ----GKERDKGIDIVIQTT------------SK-------EYIAVQCKFHQN---SISYN
gi|487875679|ref|WP_001949145.1|helicase[Helicobacterpylori]_gi|459516928|gb|EMH09091.1|helicaseprotein[HelicobacterpyloriGAM25      ----GKERDKGIDIVITTS------------NQ-------EYIAVQCKFHQN---SISYN
gi|496130066|ref|WP_008854573.1|DNAdamageinducibleprotein[Commensalibacterintestini]_gi|353671689|gb|EHD13391.1|helicasedomain/     ALNIANKKDAGIDLVGVTL-D-----------G-------EYHAIQCKNYNPDE-TISKN
gi|924898894|ref|WP_053556546.1|damageinducibleprotein[Sphingopyxissp.113P3]_gi|924523996|gb|ALC14732.1|damageinducibleprotei/1     ES--GT--DTGIDLVAELA-DD---------PG-------RWCAIQCKFYAEGY-RLQRS
gi|780802950|gb|KJS08562.1|damageinducibleprotein[Hoefleasp.BRH_c9]/11633                                                          VS--GQ--DTGIDLVARVR-DD----------G-------GFCAIQCKFYREGH-RIQKG
gi|944671432|gb|KQN22886.1|damageinducibleprotein[Sphingomonassp.Leaf32]/11415                                                      WK--QT--DTGVDLVAKLR-NE----------D-------GFCAVQCKFYHADY-HVQKS
gi|944612953|gb|KQM64772.1|damageinducibleprotein[Sphingomonassp.Leaf16]/11726                                                      WK--QT--DTGVDLVAKLR-NE----------D-------GFCAVQCKFYHADY-HVQKS
gi|764353851|ref|WP_044331468.1|damageinducibleprotein[Sphingomonashengshuiensis]_gi|761897338|gb|AJP71707.1|damageinduciblep/1     VD--GR--DTGIDAVAKIR-GE----------E-------GFCAIQCKFYREGH-RIQKA
gi|945874472|gb|KQZ27973.1|damageinducibleprotein[Mesorhizobiumsp.Root552]/11651                                                   VD--GR--DTGIDAVAQVR-GE----------D-------AVCAIQCKFYREGH-RIQKA
gi|759408081|ref|WP_043132403.1|damageinducibleprotein[Paracoccussanguinis]_gi|694217086|gb|KGJ14281.1|damageinducibleprotein/1    WS--KA--DTGIDLVATLA-DG----------S-------GYAAIQCKFYAPNH-TIQKP
gi|654342671|ref|WP_027836116.1|damageinducibleprotein[Maritaleamyrionectae]/11632                                                 WA--SN--DTGIDLVATLA-DG----------S-------GYAAVQCKFYAPNY-SIQKP
gi|126717687|gb|EBA14409.1|hypotheticalproteinRSK20926_02032[Roseobactersp.SK20926]/11603                                          WS--NA--DTGIDLVATLA-DG----------S-------GYAAIQCKFYAPDH-SIQKP
gi|765301116|ref|WP_044628780.1|damageinducibleprotein[Halomonasmeridiana]_gi|764089679|gb|KJD19875.1|damageinducibleprotein[/1    WS--GA--DIGIDLVATLA-DG----------S-------GCAAIQCKFYAPNH-VIQKP
gi|518053006|ref|WP_019223214.1|DNAdamageinducibleprotein[Bartonellarattaustraliani]/11654                                         -D--GR--DIGIDLVAKIR-DE----------N-------GYAAIQCKFYETSH-CIKKE
gi|493637806|ref|WP_006589578.1|DNAdamageinducibleprotein[Bartonellabirtlesii]_gi|395410345|gb|EJF76900.1|hypotheticalproteinM/    WN--KN--DIGIDLVAKLR-HQ----------E-------GYVAIQCKFYKADH-QISKK
gi|501188603|ref|WP_012231622.1|DNAdamageinducibleprotein[Bartonellatribocorum]_gi|161017843|emb|CAK01401.1|helicase/methyltra/   WN--KN--DIGIDLVAKLR-HQ----------E-------GYVAIQCKFYQADH-QISKK
gi|651388616|ref|WP_026500615.1|damageinducibleprotein[Bartonellagrahamii]/11655                                                   WN--KN--DIGIDLVAKLR-NQ----------E-------GYVAIQCKFYKADH-QISKK
gi|501188669|ref|WP_012231687.1|DNAdamageinducibleprotein[Bartonellatribocorum]_gi|161017922|emb|CAK01480.1|helicase/methyltra/   WN--KN--DIGIDLVAKLR-NQ----------E-------GYVAIQCKFYQADH-QISKK
gi|518048832|ref|WP_019219040.1|DNAdamageinducibleprotein[Bartonellaflorencae]/11655                                               WN--KN--DIGIDLVAKLR-HQ----------E-------GYVAIQCKFYKADH-QISKK
gi|518053617|ref|WP_019223825.1|DNAdamageinducibleprotein[Bartonellarattaustraliani]/11607                                         -D--GR--DIGIDLVAKIR-DE----------E-------GYAAIQCKFYESSH-CIKKE
gi|506336449|ref|WP_015856168.1|DNAdamageinducibleprotein[Bartonellagrahamii]_gi|240267492|gb|ACS51080.1|helicase/methyltransf/    WN--KN--DIGIDLVAKLR-HQ----------E-------GYVAIQCKFYKADH-QISKK
gi|494588832|ref|WP_007347427.1|DNAdamageinducibleprotein[Bartonellarattimassiliensis]_gi|395419053|gb|EJF85361.1|hypothetical/  -D--GR--DIGIDLVATIR-DQ----------G-------GYAAIQCKCYDASH-IIKKE
gi|501188650|ref|WP_012231668.1|DNAdamageinducibleprotein[Bartonellatribocorum]_gi|161017902|emb|CAK01460.1|helicase/methyltra/   -D--GT--DIGIDLVATIR-DE----------G-------GYAAIQCKCYDASH-IIKKE
gi|501188624|ref|WP_012231643.1|DNAdamageinducibleprotein[Bartonellatribocorum]_gi|161017866|emb|CAK01424.1|helicase/methyltra/   WK--GSDTDTDIDLVAKIR-DQ----------D-------DYVAIRCQFYETNH-QITQD
gi|518053856|ref|WP_019224064.1|DNAdamageinducibleprotein[Bartonellarattaustraliani]/11625                                         WK--GSDTDTDIDLVAKIR-DQ----------D-------DYVAIRCKFYETNH-QITQD
gi|501188053|ref|WP_012231081.1|DNAdamageinducibleprotein[Bartonellatribocorum]_gi|161017434|emb|CAK00992.1|helicase/methyltra/   WK--GSDTDTDIDLVAKIR-DQ----------D-------DYVAIRCQFYETNH-QITQD
gi|651388630|ref|WP_026500626.1|damageinducibleprotein[Bartonellagrahamii]/11657                                                   WN--KN--DIGIDLVAKLR-HQ----------E-------GYVAIQCKCYKADH-QISKK
gi|518048855|ref|WP_019219063.1|DNAdamageinducibleprotein[Bartonellaflorencae]/11658                                               -D--GT--DIGIDLVATVR-DE----------G-------GYAAIQCKCYDASH-IIKKE
gi|490995101|ref|WP_004856829.1|DNAdamageinducibleprotein[Bartonelladoshiae]_gi|395413167|gb|EJF79645.1|hypotheticalproteinMCS/   -D--GR--DIGIDLVATIR-DQ----------G-------GYAAIQCKCYDTSH-IIKKE
gi|491002558|ref|WP_004864279.1|DNAdamageinducibleprotein[Bartonellavinsonii]_gi|395421392|gb|EJF87643.1|hypotheticalproteinME/    WD--GT--DIGIDLVAKIR-DE----------E-------GYAAIQCKFYSANH-QIKKE
gi|491003373|ref|WP_004865094.1|DNAdamageinducibleprotein[Bartonellavinsonii]_gi|395432864|gb|EJF98838.1|hypotheticalproteinME/    WD--GT--DIGIDLVAKIR-DE----------E-------GYAAIQCKFYSANH-QIKKE
gi|491002426|ref|WP_004864148.1|DNAdamageinducibleprotein[Bartonellavinsonii]_gi|395421767|gb|EJF88003.1|hypotheticalproteinME/    ED--GR--DIGIDLVAKIR-DE----------G-------SYAAIQCKCYEASH-CIKKE
gi|491004024|ref|WP_004865745.1|DNAdamageinducibleprotein[Bartonellavinsonii]_gi|395432542|gb|EJF98522.1|hypotheticalproteinME/    ED--GR--DIGIDLVAKIR-DE----------G-------GYAAIQCKCYDASH-CIKKE
gi|648396442|ref|WP_026088193.1|damageinducibleprotein[Bartonellarattaustraliani]/11593                                            ED--GR--DIGIDLVAKIR-DE----------D-------GYAAIQCKFYESSH-CIKKE
gi|748190174|ref|WP_039761290.1|damageinducibleprotein[Bartonellaqueenslandensis]/11658                                            ED--GR--DIGIDLVAKIR-DE----------E-------GYAAIQCKFYESSH-CIKKE
gi|494817599|ref|WP_007553007.1|DNAdamageinducibleprotein[Bartonellasp.DB56]_gi|395411312|gb|EJF77837.1|hypotheticalproteinMC/1    WD--GT--DIGIDLVAKIR-DE----------E-------GYAAIQCKFYSANH-QIKKE
gi|515763376|ref|WP_017195976.1|DNAdamageinducibleprotein[Bartonellabirtlesii]/11656                                               WD--GT--DIGIDLVAKIR-DE----------E-------GYAAIQCKFYSANH-QIKKE
```

```
gi|494589420|ref|WP_007348014.1|DNAdamageinducibleprotein[Bartonellarattimassiliensis]_gi|395416278|gb|EJF82667.1|hypothetical/    ED--GT--DIGIDLVATIR-DE----------G-------GYAAIQCKCYDASH-IIKKE
gi|506336469|ref|WP_015856188.1|DNAdamageinducibleprotein[Bartonellagrahamii]_gi|240267512|gb|ACS51100.1|helicase/methyltransf/    ED--GT--DIGIDLVATIR-DE----------G-------GYAAIQCKCYDASH-IIKKE
gi|749308087|ref|WP_040132227.1|damageinducibleprotein[Bartonellatribocorum]_gi|605051878|emb|CDO49988.1|helicase/methyltransf/    ED--GT--DIGIDLVATIR-DE----------G-------GYAAIQCKCYDASH-IIKKE
gi|515763800|ref|WP_017196400.1|DNAdamageinducibleprotein[Bartonellabirtlesii]/11658                                                ED--GR--DIGIDLVATIR-DQ----------G-------GYAAIQCKCYDASH-CIKKE
gi|492186994|ref|WP_005773380.1|DNAdamageinducibleprotein[Bartonellaelizabethae]_gi|395418361|gb|EJF84688.1|hypotheticalprotei/    ED--GR--DIGIDLVATIR-DQ----------G-------GYAAIQCKCYDAAH-IIKKE
gi|501188615|ref|WP_012231634.1|DNAdamageinducibleprotein[Bartonellatribocorum]_gi|161017856|emb|CAK01414.1|helicase/methyltra/    ED--GR--DIGIDLVATIR-DE----------G-------GYAAIQCKCYDASH-IIKKE
gi|501187470|ref|WP_012230499.1|DNAdamageinducibleprotein[Bartonellatribocorum]_gi|161017090|emb|CAK00648.1|helicase/methyltra/    ED--GR--DIGIDLVATIR-DQ----------G-------GYAAIQCKCYDASH-IIKKE
gi|501188636|ref|WP_012231655.1|DNAdamageinducibleprotein[Bartonellatribocorum]_gi|161017879|emb|CAK01437.1|helicase/methyltra/    ED--GR--DIGIDLVATIR-DQ----------G-------GYAAIQCKCYDASH-IIKKE
gi|930151941|emb|CTQ34818.1|UvsWhelicase[Jannaschiarubra]/11641                                                                    ED--AA--DSGIDLVATLA-DG----------S-------GFCAVQCKFYDETK-KVDKG
gi|928977106|ref|WP_054001461.1|damageinducibleprotein[Shimiasp.SK013]_gi|927698852|gb|KPA22698.1|typeIrestrictionenzymeEcoKIs/    WT--GK--DTGIDLVAKLA-DE----------D-------GYAAVQCKFYAANK-KIQIG
gi|738872465|ref|WP_036760884.1|damageinducibleprotein[Leisingeradaeponensis]/11643                                                 WD--GK--DVGIDLVAKLR-GH----------E-------GFAAIQCKFYNPTH-KIAKA
gi|518050881|ref|WP_019221089.1|DNAdamageinducibleprotein[Bartonellasenegalensis]/11652                                             WD--GC--DTGIDLVAKLR-HE----------E-------GFAAVQCKFYDAAY-RIKKA
gi|493980596|ref|WP_006923523.1|DNAdamageinducibleprotein[Bartonellawashoensis]_gi|395413322|gb|EJF79796.1|hypotheticalprotein/    WD--VR--DTGIDLVAKIR-NE----------E-------GFAAIQCKFYDAAY-RIRKA
gi|493983106|ref|WP_006925984.1|DNAdamageinducibleprotein[Bartonellawashoensis]_gi|395418127|gb|EJF84455.1|hypotheticalprotein/    WD--GR--DIGIDLVAKIR-DE----------E-------GFAAIQCKFYDAAY-RIRKA
gi|499494671|ref|WP_011181311.1|DNAdamageinducibleprotein[Bartonellahenselae]_gi|49239008|emb|CAF28308.1|hypotheticalproteinBH/    WD--GR--DTGIDLVAKLR-NE----------D-------GFAAIQCKFYDAAY-RIKKA
gi|651388426|ref|WP_026500441.1|damageinducibleprotein[Bartonellagrahamii]/11652                                                    WN--GH--DTGIDLVAKIR-NE----------E-------GFAAIQCKFYDAAY-RIRKA
gi|506337280|ref|WP_015856999.1|DNAdamageinducibleprotein[Bartonellagrahamii]_gi|240268402|gb|ACS51990.1|helicase/methyltransf/    WD--GH--DTGIDLVAKIR-NE----------E-------GFAAIQCKFYDAAY-RIRKA
gi|494817535|ref|WP_007552943.1|DNAdamageinducibleprotein[Bartonellasp.DB56]_gi|395412002|gb|EJF78518.1|hypotheticalproteinMC/1    WD--GR--DTGIDLVAKIR-DE----------E-------GFAAIQCKFYDAAY-RIKKA
gi|493638099|ref|WP_006589864.1|DNAdamageinducibleprotein[Bartonellabirtlesii]_gi|395409809|gb|EJF76394.1|hypotheticalproteinM/    WD--GR--DTGIDLVAKIR-DE----------E-------GFAAIQCKFYDAAY-RIKKA
gi|748189589|ref|WP_039760707.1|damageinducibleprotein[Bartonellaqueenslandensis]/11651                                             WD--GR--DIGIDLVAKLR-HE----------K-------GFAAIQCKFYDAEY-RIKKA
gi|501189483|ref|WP_012232501.1|DNAdamageinducibleprotein[Bartonellatribocorum]_gi|161018902|emb|CAK02460.1|helicase/methyltra/    WD--GR--DTGIDLVAKLR-HE----------D-------GFAAIQCKFYDESY-RIKKA
gi|518049181|ref|WP_019219389.1|DNAdamageinducibleprotein[Bartonellaflorencae]/11652                                                HD--GR--DTGIDLVAKLR-HE----------D-------GFAAIQCKFYDESY-RIRKA
gi|492193255|ref|WP_005774528.1|DNAdamageinducibleprotein[Bartonellaelizabethae]_gi|395416403|gb|EJF82779.1|hypotheticalprotei/    WD--GR--DTGIDLVAKLR-HE----------D-------GFAAIQCKFYDESY-RIKKA
gi|498387325|ref|WP_010701288.1|helicase/methyltransferase[Bartonellabovis]_gi|478418724|gb|ENN92426.1|helicase/methyltransfera    WD--AR--DTGIDLVAKLC-KE----------D-------GFAAIQCKFYDADY-RIRKR
gi|319408697|emb|CBI82352.1|conservedhypotheticalprotein[BartonellaschoenbuchensisR1]/11661                                         WD--AR--DTGIDLVAKLR-KE----------D-------GFAAIQCKFYDADY-RIRKR
gi|494741726|ref|WP_007477134.1|DNAdamageinducibleprotein[Bartonellamelophagi]_gi|395423925|gb|EJF90113.1|hypotheticalproteinM/    WN--SR--DTGIDLVAKLH-KE----------E-------GFAAIQCKFYDADY-RIKKK
gi|737261405|ref|WP_035245349.1|damageinducibleprotein[Alcanivoraxjadensis]_gi|686989709|gb|KGD62556.1|hypotheticalproteinT9A_/    LS--RK--DTGIDLVARLR-GA---------PE-------TFCAIQCKFYNRDY-KLHRK
gi|916687564|ref|WP_051294655.1|damageinducibleprotein[Gemmobacternectariphilus]/11641                                              WD--GR--DTGIDLVAKLK-DE----------D-------GFAAIQCKFFDANY-RIRKE
gi|740294127|ref|WP_038132495.1|damageinducibleprotein[Thioclavasp.DT234]_gi|660524826|gb|KEO54689.1|hypotheticalproteinDT23_/1    WS--GK--DIGIDLVAKLR-NE----------E-------GFAAIQCKFYAETH-RIQKA
gi|568331062|ref|WP_024099615.1|helicase[Phaeobactergallaeciensis]_gi|568232121|gb|AHD12007.1|putativehelicase[Phaeobactergalla    WD--GR--DTGIDLVAKLK-DE----------D-------GYAAIQCKFYDAAY-RIRKE
gi|518810459|ref|WP_019966413.1|DNAdamageinducibleprotein[Pannonibacterphragmitetus]/11635                                          RD--GK--DVGIDLVAKLR-NE----------D-------GFAAIQAKFYAADT-RIQKA
gi|495305760|ref|WP_008030510.1|DNAdamageinducibleprotein[Rhodobactersp.SW2]_gi|259021698|gb|EEW25000.1|typeIIIrestrictionprot/    RD--GK--DVGIDLVAKLR-NE----------A-------GFAAIQAKFYAADA-RIQKI
gi|506403303|ref|WP_015923022.1|DNAdamageinducibleprotein[Rhodobactersphaeroides]_gi|221162951|gb|ACM03922.1|HypotheticalProte/    RD--GK--DIGIDLVSKLR-NE----------D-------GFAAIQAKFYTADT-RIQKA
gi|738818386|ref|WP_036708790.1|damageinducibleprotein[Paracoccussanguinis]_gi|694226786|gb|KGJ22541.1|damageinducibleprotein/1    RD--GK--DIGIDLVSKLR-NE----------D-------GFAAIQAKFYTADT-RIQKA
gi|746096057|ref|WP_039162453.1|damageinducibleprotein[Gallibacteriumanatis]_gi|703617775|gb|KGQ63868.1|damageinducibleprotei/1    EG--TRKTDTGIDLVAIDF------------EG-------KFTAIQCKNYSPDH-RIQKS
gi|737716785|ref|WP_035685631.1|damageinducibleprotein[Avibacteriumparagallinarum]/11643                                            EG--TRKTDTGIDLVAIDF------------EG-------KFTAIQCKNYSPDH-RIQKS
gi|540072553|gb|ERF78672.1|hypotheticalproteinN561_04930[Gallibacteriumanatis12656/12}/11651                                        EG--TRKTDTGIDLVAIDF------------EG-------KFTAIQCKNYSPDH-RIQKS
gi|917509519|ref|WP_052115936.1|damageinducibleprotein[Gallibacteriumanatis]_gi|702414161|gb|KGQ42233.1|damageinducibleprotei/1    EG--TRKTDTGIDLVAIDF------------EG-------KFTAIQCKNYSPDH-RIQKS
gi|746082942|ref|WP_039150268.1|damageinducibleprotein[Gallibacteriumanatis]_gi|703617078|gb|KGQ63194.1|damageinducibleprotei/1    EG--TRKTDTGIDLVAIDF------------EG-------KFTAIQCKNYSPDH-RIQKS
gi|822522885|ref|WP_046952840.1|damageinducibleprotein[Haemophilushaemolyticus]_gi|821501762|gb|KKZ59267.1|damageinduciblepro/1    NL---PSNDTGIDLVAVTH------------DG-------ENHAIQCKNYSQYH-RISKA
gi|404381014|gb|EJZ77501.1|putativehelicase[Pasteurellamultocidasubsp.gallicidaP1059]/11681                                         NL---PSNDTGIDLVAITH------------SG-------ENHAIQCKFYSQSH-HISKK
gi|298281660|gb|EFI23149.1|helicaseCterminaldomainprotein[Neisseriasp.oraltaxon014str.F0314]/11493                                  ELDLGGATDAGIDLVATTF------------TG-------EHHAIQCKNYAPDH-TLQKK
gi|348013055|gb|EGY51980.1|helicasedomainprotein[Neisseriashayeganii871]/11481                                                      SLGLNSKTDTGIDLVATTQ------------AG-------EFHAIQCKNHAPEK-TIQKA
gi|496407140|ref|WP_009116004.1|DNAdamageinducibleprotein[Neisseriawadsworthii]_gi|349794756|gb|EGZ48566.1|helicasedomainprote/    SLGLSSKTDAGIDLVATTQ------------VG-------EFHAIQCKNYAPEK-TIQKA
gi|517216441|ref|WP_018405259.1|DNAdamageinducibleprotein[Marinobacterlipolyticus]/11656                                            ELGVSTKKDTGIDLVAITS------------DG-------QFHAIQCKNYSPDY-SIRKN
gi|738230264|ref|WP_036185691.1|damageinducibleprotein[Marinobactersp.ELB17]/11671                                                  ELGVSTKKDTGIDLVAITS------------DG-------QFHAIQCKNYSPDY-SIRKN
gi|447197741|ref|WP_001274997.1|DNAdamageinducibleprotein[Acinetobacterbaumannii]_gi|408512324|gb|EKK13969.1|typeIIIrestrictio/    KLNIADKRDYGIDLVAVTI------------DG-------EFHPIQCKNYNTT--KIQKK
gi|820775897|ref|WP_046739909.1|damageinducibleprotein[Acinetobactersp.AG1]_gi|819909758|gb|KKW75938.1|damageinducibleprotein/1    KLNITDKRDYGIDLVAVTI------------DG-------QFHPIQCKNYNTT--KIQKK
gi|584595163|ref|WP_024160846.1|helicase[Acinetobacterpittii]_gi|576702530|emb|CDI28049.1|helicasedomaincontainingprotein[Acin/    KLKVTDKRDYGIDLVAVTI------------EG-------EFHPIQCKNYNTT--KIQKK
gi|514963042|ref|WP_016651366.1|hypotheticalprotein[Acinetobactersp.NIPH2036]_gi|514162665|gb|EPG41391.1|hypotheticalproteinF90    KLKVTDKRDYGIDLIAVTI------------DG-------EFHPIQCKNYNTT--KIQKK
gi|515965564|ref|WP_017396147.1|DNAdamageinducibleprotein[Acinetobacterhaemolyticus]/11667                                          KLNITDKRDYGIDLVAVTM------------GG-------EFHPIQCKNYNTT--KIQKK
gi|491346810|ref|WP_005204747.1|MULTISPECIES:hypotheticalprotein[Acinetobacter]_gi|480335823|gb|ENX56033.1|hypotheticalproteinF    KLNITDKRDYGIDLVAVTM------------DG-------EFHPIQCKNYNTT--KIQKK
gi|693044472|ref|WP_032215268.1|damageinducibleprotein[Escherichiacoli]_gi|607725731|gb|EZE08230.1|damageinducibleprotein[Esc/1    IVGITTKKDAGIDLVAVTK------------TG-------EFHAIQCKNYNKT--KIAKK
gi|742382177|ref|WP_038861304.1|damageinducibleprotein[Cronobactermuytjensii]_gi|924333747|gb|ALB69766.1|damageinducibleprote/1    AVGIKTKKDAGIDLVAVTK------------TG-------EFHAIQCKNYNQT--KIAKK
gi|915912804|ref|WP_050927035.1|damageinducibleprotein[Yersiniaenterocolitica]_gi|899805802|emb|CQH32042.1|typeIrestrictionenz/    AVGITTKKDAGIDLVAVTK------------TG-------EFHAIQCKNYNQT--KIAKK
gi|921111327|gb|KON62781.1|typeIrestrictionenzymeEcoKIsubunitR[Komagataeibactereuropaeus]/11492                                     QEG-YVAKDTGIDLVARTR--G---------TG-------EYHAIQCKFYASSH-RISKQ
gi|582024278|gb|AHI27615.1|DNAhelicaserestrictionenzymeTypeIIIRsubunit[KomagataeibacterxylinusE25]/11636                           QEG-YVAKDTGIDLVARTR--G---------TG-------EYHAIQCKFYAPSH-SISKP
gi|347662673|dbj|BAK86128.1|DNAhelicaserestrictionenzymeTypeIIIRsubunit[KomagataeibactermedellinensisNBRC3288]/11737                EEG-YVAKDTGIDLVARTR--G---------TG-------EYHAIQCKFYASSH-RISKQ
gi|917445053|ref|WP_052051471.1|damageinducibleprotein[Acetobactertropicalis]/11429                                                 EEG-YPVKDTGIDLVARER--G---------TG-------NLHAIQCKFYAPDH-KITKK
gi|453329445|dbj|GAC88472.1|DNAhelicaserestrictionenzymetypeIIIRsubunit[GluconobacterthailandicusNBRC3255]_gi|530287981|dbj|GAD    ENG-HPVKDTGIDLVARER--G---------TG-------KLHAIQCKFYAPDH-KITKK
gi|889770922|ref|WP_048844278.1|damageinducibleprotein[Gluconobacterfrateurii]/11419                                                ENG-HPVKDTGIDLVARER--G---------TG-------KLHAIQCKFYAPDH-KITKK
gi|504886208|ref|WP_015073310.1|hypotheticalprotein[Gluconobacteroxydans]_gi|411027954|gb|AFW01209.1|hypotheticalproteinB932_16    ENG-HPVKDTGIDLVARER--G---------TG-------KLHAIQCKFYAPDH-KITKK
gi|480040211|gb|ENV79751.1|hypotheticalproteinF942_01641[AcinetobacterursingiiANC3649]/11432                                        RNGEDLGIDTGIDLVAKVR--D---------TE-------DYHAIQCKLYSEDY-NLQKK
gi|822498222|ref|WP_046936504.1|damageinducibleprotein[Xanthomonasgardneri]_gi|821751224|gb|KLB07223.1|damageinducibleprotein/1    AHG-FSAKDDGIDLVAEVA--G---------TG-------EIHAIQCKFYAADY-KLRKD
gi|654059559|ref|WP_027705556.1|damageinducibleprotein[Zymobacterpalmae]/11635                                                      LHG-FSAKDDGIDLIAQEA--G---------TG-------GFCAIQCKFYAPDY-RLKKT
gi|643535620|ref|WP_025224315.1|damageinducibleprotein[Methylophilaceaebacterium11]_gi|577704400|gb|EUJ10546.1|putativehelicas/    SQG-LARNDAGIDLVAQTQ--G---------TN-------EFHAIQCKFYAEDY-RVQKS
MBA11I/11635                                                                                                                        SQG-LARNDAGIDLVAQTQ--G---------TN-------EFHAIQCKFYAEDY-RVQKS
```

```
gi|655355857|ref|WP_028762287.1|damageinducibleprotein[Shewanellacolwelliana]/11632    EQG-IDGRDTGIDLVAQTD--A---------TN-------EYHAIQCKCYAEDY-RIQKA
gi|753204236|ref|WP_041510579.1|damageinducibleprotein[Shewanellasp.cp20]_gi|752457765|gb|KIO36326.1|damageinducibleprotein[S/1    EYD-RDGKDVGIDLVAKTR--D---------TN-------EYHAIQCKCYDESY-RLQKK
KPNNIH30III/11619    LNN-RDAKDVGIDLVAKTR--D---------TD-------EYHAIQCKCYDESY-RLQKS
gi|749295994|ref|WP_040120352.1|damageinducibleprotein[Klebsiellapneumoniae]_gi|723249300|gb|AIX81926.1|damageinducibleprotei/1    LNN-RDAKDVGIDLVAKTR--D---------TD-------EYHAIQCKCYDESY-RLQKS
gi|641639739|emb|CDQ54010.1|unnamedproteinproduct[Klebsiellapneumoniae]/11641    LNN-RDAKDVGIDLVAKTR--D---------TD-------EYHAIQCKCYDESY-RLQKS
gi|490797922|ref|WP_004660060.1|hypotheticalprotein[Acinetobactersp.NIPH236]_gi|479876779|gb|ENU27973.1|hypotheticalproteinF992    --QGLSGKDTGIDLVATTK------------DG-------EFHAIQCKLYDADR-KVSKA
gi|916750926|ref|WP_051357982.1|damageinducibleprotein[Acinetobactertjernbergiae]/11453    --QGLSGKDTGIDLVATTK------------DG-------EFYAIQCKLYDADR-KVTKA
gi|691098662|ref|WP_032025060.1|damageinducibleprotein[Acinetobacterbaumannii]_gi|593567521|gb|EXR50735.1|restrictionendonucle/    --KGLSGKDTGIDLVATTK------------DG-------DFHAIQCKLYDADR-KVTKA
gi|736669405|ref|WP_034676049.1|damageinducibleprotein[Acinetobacterbaumannii]/11643    --QGLSGKDTGIDLVATTK------------DG-------EFQAIQCKLYDADR-KVTKA
gi|491368928|ref|WP_005226826.1|hypotheticalprotein[Acinetobactersp.NIPH1847]_gi|480173259|gb|ENX09797.1|hypotheticalproteinF89    --QGLTGKDTGIDLVATTK------------DG-------EFHAIQCKLYDADS-KVPKS
gi|796853993|ref|WP_045795953.1|damageinducibleprotein[Acinetobacterindicus]_gi|787929761|gb|KJV44219.1|damageinducibleprotei/1    --QGLTGKDTGIDLVATTK------------EN-------KFHAIQCKLYDADS-TVSKG
gi|295797463|gb|ADG32253.1|typeIIIrestrictionproteinressubunit[ThiomonasintermediaK12]/11676    QGQSDPGADAGIDLVAVEDVAD---------NP-------RIFAIQAKFYAEDA-KIRKD
gi|499871179|ref|WP_011551913.1|DNAdamageinducibleprotein[Myxococcusxanthus]_gi|108467544|gb|ABF92729.1|putativerestriction/mo/    --HSLDKRDAGIDLVAESF------------SD-------EIHAIQCKLYAPDY-RVQKG
gi|495313852|ref|WP_008038600.1|DNAdamageinducibleprotein[Bartonellatamiae]_gi|395424181|gb|EJF90368.1|hypotheticalproteinME5_/    --HGKDGKDKGIDLVAQTK------------KG-------ELHAIQCKFYAPDH-KISKT
gi|501453335|ref|WP_012476784.1|DNAdamageinducibleprotein[Leptospirabiflexa]_gi|167777824|gb|ABZ96123.1|Helicase[Leptospirabif/    --EGKDARDVGIDIVAKTR--S---------TG-------EIHAIQCKFYDPDY-KIQKS
gi|738104613|ref|WP_036063193.1|damageinducibleprotein[Leptospiranoguchii]/11652    --QKIDKRDTGIDLVAKTQ--G---------TE-------EFHAIQCKFYSPEY-RIQKT
gi|410001039|gb|EKO51661.1|typeIIIrestrictionenzyme,ressubunit[Leptospirakirschneristr.200802841]/11651    --QKIDKRDTGIDLVAKTQ--G---------TE-------EYHAIQCKFYSPEH-RIQKS
gi|490901851|ref|WP_004763774.1|typeIIIrestrictionenzyme,ressubunit[Leptospirakirschneri]_gi|464209249|gb|EMN24014.1|typeIIIres    --QKIDKRDTGIDLVAKTQ--G---------TE-------EYHAIQCKFYSPEH-RIQKS
gi|464398131|gb|EMO65902.1|typeIIIrestrictionenzyme,ressubunit[Leptospirakirschneristr.200803703]/11648    --QKIDKRDTGIDLVAKTQ--G---------TE-------EYHAIQCKFYSPEH-RIQKS
gi|945029024|gb|KQQ84900.1|damageinducibleprotein[Xanthomonassp.Leaf131]/11423    --NGFSAKDDGIDLVAQA--AG---------TG-------EIHAIQCKFYSPDY-KLRK-
gi|896511314|ref|WP_049418183.1|damageinducibleprotein[Stenotrophomonasmaltophilia]/11639    --NGFSGKDDGIDLVAEV--AG---------TG-------EIHAIQCKFYAPDY-KLQK-
gi|896513082|ref|WP_049419616.1|damageinducibleprotein[Stenotrophomonasmaltophilia]/11638    --HGFSAKDDGIDLVAEE--AG---------TG-------AMHAIQCKFYDNTH-RLQK-
gi|896580616|ref|WP_049469425.1|damageinducibleprotein[Stenotrophomonasmaltophilia]/11641    --HGFSAKDDGIDLVAEE--AG---------TG-------AIHAIQCKFYDPDY-RLQK-
gi|648289350|ref|WP_026070499.1|damageinducibleprotein[Stenotrophomonasmaltophilia]/11639    --HGFSAKDDGIDLVAEE--AG---------TG-------AIHAIQCKFYDPDY-RLQK-
gi|656272786|ref|WP_029218442.1|damageinducibleprotein[Xanthomonascassavae]/11639    --HGFSAKDDGIDLVAEE--AG---------TV-------AIHAIQCKFYDPDY-RLQK-
gi|406976236|gb|EKD98747.1|hypotheticalproteinACD_23C00290G0002[unculturedbacterium]/11679    QGQPDPGVDAGIDLVAVEATKD---------NP-------RIFAVQAKFYAEDA-KISKA
gi|828145737|ref|WP_047238120.1|damageinducibleprotein[Chromobacteriumsubtsugae]/11645    -GHGWNKKDTGIDLVART--T----------EG-------EYHAIQCKFYAEDY-KVQK-
gi|654311174|ref|WP_027824327.1|damageinducibleprotein[Laribacterhongkongensis]/11641    -GNGWSKKDTGIDLVAKT--T----------DG-------DYHAIQCKFYAEDY-KVQK-
gi|757242173|gb|KIR15516.1|typeIrestrictionenzymeEcoKIsubunitR[Pseudomonasfluorescens]/11778    --QGLDGRDTGIDLVAKT--HG---------TG-------EFHAIQCKFYADNY-RIQK-
gi|653605161|ref|WP_027616682.1|damageinducibleprotein[Pseudomonassp.URHB0015]/11639    --QGLDGRDTGIDLVAKT--HG---------TG-------EIHAIQCKFYADNY-RIQK-
gi|520868025|ref|WP_020308060.1|helicase[Pseudomonassyringae]_gi|519159034|gb|EPM49935.1|helicasedomaincontainingprotein[Pseud/    --QGLDGRDTGIDLVAKT--HG---------TG-------EIHAIQCKFFADNY-RIQK-
gi|940156567|gb|KPX71668.1|Helicasedomaincontainingprotein[Pseudomonasamygdalipv.lachrymans]/11677    --HGLDGRDTGIDLVAKT--KG---------TG-------EFHAIQCKFYADNY-RVQR-
gi|505460508|ref|WP_015647610.1|helicase[Pseudomonassp.GLE121]_gi|499110212|ref|YP_007974374.1|helicasedomaincontainingprotein/    --QGLDGRDTGIDLVAKT--QG---------TG-------EYHAIQCKLYAEDY-RVQK-
gi|940235551|gb|KPY45311.1|hypotheticalproteinALO48_200154[Pseudomonassyringaepv.rhaphiolepidis]/11631    --QGFDGRDTGIDLVAKT--KG---------TG-------DYHAIQCKLYAENY-RVQK-
gi|520880808|ref|WP_020312235.1|helicase[Pseudomonassyringae]_gi|519172158|gb|EPM62027.1|helicasedomaincontainingprotein[Pseud/    --QGQDGRDTGIDLVAKT--QG---------TN-------EYHAIQCKLYAEDY-RVQK-
gi|829046479|ref|WP_047282259.1|damageinducibleprotein[Pseudomonasfluorescens]/11630    --HGQDGRDTGIDLVART--QG---------TG-------EYHAIQCKLYAEGY-RVQK-
gi|499415493|ref|WP_011102960.1|DNAdamageinducibleprotein[Pseudomonassyringaegroupgenomosp.3]_gi|28867279|ref|NP_789898.1|heli/    --QGLSGKDAGIDLVART--QG---------TG-------EYHAIQCKLYAEDY-KVQK-
gi|730238299|ref|WP_033942897.1|damageinducibleprotein[Pseudomonasaeruginosa]/11629    --QGLSGKDAGIDLVART--QG---------TG-------EYHAIQCKLYAEDY-KVQK-
gi|495310125|ref|WP_008034873.1|DNAdamageinducibleprotein[Pseudomonassp.GM60]_gi|398186685|gb|EJM74052.1|putativehelicase[Pseu/    --QGLHSRDTGIDLVART--QG---------TS-------EFHAIQCKLYAEDY-RVQK-
gi|779992653|ref|WP_045428690.1|damageinducibleprotein[Pseudomonassp.MT1]_gi|699967872|dbj|BAP80334.1|DNAhelicaserestrictione/1    --QGLDGRDTGIDLVART--QG---------TG-------EYHAIQCKLYAEDY-RVQK-
gi|786037289|ref|WP_045733274.1|damageinducibleprotein[Pseudomonaspseudoalcaligenes]_gi|782987904|gb|KJU79152.1|damageinducib/1    --RGLSGKDAGIDLVART--QG---------TG-------EYHAIQCKLYAEDY-RVQK-
gi|749666366|ref|WP_040265445.1|damageinducibleprotein[Pseudomonasrhodesiae]/11632    --QSLSGKDAGIDLVART--QG---------TG-------EYHAIQCKLYAEDY-KVQK-
gi|734902433|ref|WP_034133698.1|damageinducibleprotein[Pseudomonasfluorescens]/11635    --QSLSGKDAGIDLVART--QG---------TG-------EYHAIQCKLYAEDY-KVQK-
gi|490551031|ref|WP_004416146.1|typeIIrestrictionmodificationsystemrestriction/helicasesubunit[Pseudomonassyringae]_gi|4432823/    --QSLSGKDAGIDLVART--QG---------TG-------EYHAIQCKLYAEDY-KVQK-
gi|489199911|ref|WP_003109152.1|DNAdamageinducibleprotein[Pseudomonasaeruginosa]_gi|552648708|gb|ERY63184.1|hypotheticalprotei/    --QGLSGKDAGIDLVART--QG---------TG-------EYHAIQCKLYAEDY-KVQK-
gi|730641237|ref|WP_034061328.1|damageinducibleprotein[Pseudomonasaeruginosa]/11636    --QGLSGKDAGIDLVART--QG---------IG-------EYHAIQCKLYAEDY-KVQK-
gi|751304980|ref|WP_041013386.1|damageinducibleprotein[Pseudomonasxanthomarina]_gi|703407611|emb|CEG51014.1|Helicasedomaincon/1    --QGLSGKDAGIDLVART--QG---------TG-------EYHAIQCKLYAENY-RVQK-
gi|939335207|ref|WP_054881520.1|damageinducibleprotein[Pseudomonassp.NBRC111128]/11632    --QGLSGKDAGIDLVART--QG---------TG-------EYHAIQCKLYAVDY-RVQK-
gi|757693555|ref|WP_042923792.1|damageinducibleprotein[Pseudomonaschloritidismutans]_gi|780910610|gb|KJS78302.1|damageinducib/1    --QGLSGKDAGIDLVART--QG---------TG-------EYHAIQCKLYAEDY-RVQK-
gi|665953944|ref|WP_031319989.1|damageinducibleprotein[Pseudomonastolaasii]/11626    --QGLSGKDAGIDLVART--HG---------TG-------EYHAIQCKLYAEDY-KVQK-
gi|771600573|ref|WP_045213746.1|damageinducibleprotein[Pseudomonassp.21]_gi|770081123|gb|KJJ98634.1|damageinducibleprotein[Ps/1    --HVLDGRDTGIDLVART--QG---------TG-------EFHAIQCKLYAEDY-RVQK-
gi|740871396|ref|WP_038656644.1|damageinducibleprotein[Pseudomonasstutzeri]_gi|627772214|gb|AHY41437.1|damageinducibleprotein/1    --QGLDGRDTGIDLVART--QG---------TG-------EHHAIQCKLYAENY-RVQK-
gi|946242205|gb|KRC93782.1|damageinducibleprotein[Pseudomonassp.Root9]/11634    --QGLSGKDAGIDLVART--QG---------TG-------QYHAIQCKLYAEDY-KVQK-
gi|553777322|ref|WP_023109107.1|MULTISPECIES:hypotheticalprotein[Pseudomonas]_gi|552638674|gb|ERY53184.1|hypotheticalproteinQ05    --QSLSGKDAGIDLVART--QG---------TG-------EYHAIQCKLYAEDY-KVQK-
gi|896383370|ref|WP_049324618.1|damageinducibleprotein[Pseudomonasstutzeri]/11627    --QGLDGRDTGIDLVART--QG---------TG-------EYHAIQCKLYAENY-RVQK-
gi|696158510|gb|AIT41787.1|putativehelicase[Pseudomonassp.LM8]/11630    --QGLDGRDTGIDLVART--QG---------TG-------EYHAIQCKLYAEDY-RVQK-
gi|504634892|ref|WP_014821994.1|DNAdamageinducibleprotein[Pseudomonasstutzeri]_gi|390985249|gb|AFM35242.1|helicasedomainconta/1    --QGLDGRDTGIDLVART--QG---------TG-------EYHAIQCKLYSEDY-RVQK-
gi|544783974|ref|WP_021206729.1|DNAdamageinducibleprotein[Pseudomonasstutzeri]_gi|533224779|gb|EQM79688.1|DNAdamageinduciblep/1    --QGLDGRDTGIDLVART--QG---------TG-------EYHAIQCKLYAENY-RVQK-
gi|504567385|ref|WP_014754487.1|MULTISPECIES:DNAdamageinducibleprotein[Pseudomonas]_gi|388559493|gb|AFK68634.1|helicasedomain/1    --QGLSGKDAGIDLVART--QG---------TG-------QYHAIQCKLYAEDY-KVQK-
gi|654441469|ref|WP_027912052.1|damageinducibleprotein[Pseudomonassp.URIL14HWK12:I7]/11630    --QGLSGKDSGIDLVART--QG---------TG-------EYHAIQCKLYAEDY-KVQK-
gi|941470066|ref|WP_055127894.1|damageinducibleprotein[Pseudomonasmediterranea]/11631    --QSLSGKDAGIDLVART--QG---------TG-------EYHAIQCKLYAEDY-KVQK-
gi|557815599|ref|WP_023446184.1|DNAdamageinducibleprotein[Pseudomonaschloritidismutans]_gi|557364081|gb|ESQ98190.1|DNAdamagei/1    --QSLSGKDAGIDLVART--QG---------TG-------QYHAIQCKLYAEDY-KVQK-
gi|727814791|ref|WP_033898247.1|damageinducibleprotein[Pseudomonassp.FH1]_gi|567648005|gb|ETK23229.1|helicasedomaincontaining/1    --QSLSGKDAGIDLVART--QG---------TG-------EYHAIQCKLYAEDY-KVQK-
gi|503278702|ref|WP_013513363.1|DNAdamageinducibleprotein[Desulfovibrioaespoeensis]_gi|316942375|gb|ADU61426.1|typeIIrestrict/    --QGLAKRDTGIDLVAKT--RG---------TD-------EFHAIQCKLYGEDH-TIQK-
gi|505228796|ref|WP_015415898.1|typeIIrestrictionendonucleasesubunitR[Desulfovibriopiezophilus]_gi|451896976|emb|CCH49855.1|Ty    --EGLDKQDTGIDLVAKT--RG---------TE-------EFHAIQCKLYAESH-TIQK-
gi|517732543|ref|WP_018902751.1|hypotheticalprotein[Variovoraxparadoxus]/11675    --EGLDKRDTGIDLVAKT--HG---------TG-------EIHAIQCKLYAEDY-RLQK-
gi|550909475|ref|WP_022662570.1|DNAdamageinducibleprotein[Desulfovibriolongus]/11637    --QGLDKRDTGIDLVART--AG---------TG-------EFHAIQCKLYAEDY-TLQK-
```

```
gi|945494542|gb|KQV50024.1|damageinducibleprotein[Massiliasp.Root335]/11647    --QGLDKRDAGIDLVAKT--HG---------TG-------EIHAIQCKLYAEDY-RLQK-
gi|334196917|gb|AEG70102.1|helicasedomainprotein[RalstoniasolanacearumPo82]/11663    --QGLDKRDAGIDLVAKT--QG---------TG-------EYHAIQCKLFAPDH-KVQK-
gi|656006746|ref|WP_029048170.1|damageinducibleprotein[Cupriavidussp.amp6]/11661    --QGLDKRDAGIDLVAKT--QG---------TG-------EYHAIQCKLYAPDY-KVQK-
BPS2196ORF3054P/11659    --QGLDKRDTGIDLVAKT--RG---------TD-------EFHAVQCKLFAPGH-KVQK-
gi|740932312|ref|WP_038717332.1|damageinducibleprotein[Burkholderiapseudomallei]_gi|760211882|gb|KIX58314.1|damageinduciblepr/1    --QGLDKRDTGIDLVAKT--RG---------TD-------EFHAVQCKLFAPGH-KVQK-
gi|755810813|ref|WP_042583861.1|damageinducibleprotein[Burkholderiasp.MSHR3999]_gi|755174952|gb|KIP19790.1|DEAD/DEAHboxhelicas/    --QGLDKRDTGIDLVAKT--RG---------TD-------EFHAIQCKLFAPDH-KVQK-
gi|484586570|gb|AGK48195.1|DEAD/DEAHboxhelicasefamilyprotein[BurkholderiathailandensisMSMB121]/11646    --QGLDKRDTGIDLVAKT--RG---------TD-------EFHAIQCKLFAPDH-KVQK-
gi|737582935|ref|WP_035553941.1|damageinducibleprotein[Burkholderiasp.9120]/11658    --QGLDKRDAGIDLVAKT--RG---------TD-------EYHAIQCKMYAPDY-KVQK-
gi|740989872|ref|WP_038773225.1|damageinducibleprotein[Burkholderiapseudomallei]_gi|705645291|gb|KGS70691.1|DEAD/DEAHboxhelica/    --QGLDKRDTGIDLVAKT--RG---------TD-------EFHAIQCKLFAPGH-KVQK-
gi|654912525|ref|WP_028363424.1|damageinducibleprotein[Burkholderiasp.JPY366]/11659    --QGLDKRDTGIDLVANT--RG---------TN-------EFHAIQCKLFAADH-KVQK-
gi|740955879|ref|WP_038740512.1|damageinducibleprotein[Burkholderiapseudomallei]_gi|714684932|gb|KGW74438.1|DEAD/DEAHboxhelica/    --QGLDKRDTGIDLVAKT--RG---------TD-------EFHAIQCKLFAPGH-KVQK-
gi|919203714|ref|WP_052759215.1|damageinducibleprotein[Xanthomonasaxonopodis]/11388    ----G-KADTGIDLVAQER-D----------TG-------ALVSIQCKFYARSY-TLQKG
gi|522152381|ref|WP_020663589.1|hypotheticalprotein[Amycolatopsisbenzoatilytica]/11605    ----G-KPDTGIDIVAEER-E----------TG-------GFCAIQCKFYEPDH-YVQKS
gi|663196013|ref|WP_030233899.1|damageinducibleprotein[Streptomycessp.NRRLS455]/11641    ----GNQRDLGIDLVAQER-E----------TG-------ILWAIQCKFYESDH-TVQKS
gi|750429553|ref|WP_040711444.1|damageinducibleprotein[Nocardiopsispotens]/11622    ----HDKRDTGIDLVAQDR-E----------TG-------GFCAIQCKFYEPHH-TIRKE
gi|518656139|ref|WP_019819475.1|hypotheticalprotein[Saccharomonosporasaliphila]/11623    ----HDKRDTGIDLVAQDR-R----------TG-------EFCAIQCKFYDPDH-TVRKE
gi|759722933|ref|WP_043433758.1|damageinducibleprotein[Streptomycespluripotens]_gi|733404978|gb|KHK94361.1|damageinduciblepro/1    ----QDKRDTGIDLVAQDR-G----------TG-------EFCAIQCKFYEPHH-TVRKE
gi|663424167|ref|WP_030419030.1|damageinducibleprotein[Streptomycessp.NRRLF5065]/11627    ----QDKRDTGIDLVAQDR-E----------TG-------GFCAIQCKFYEPHH-TVRKE
gi|663412917|ref|WP_030408353.1|damageinducibleprotein[Streptomycessp.NRRLF5917]/11630    ----QDKRDTGIDLVAQDR-E----------TG-------GFCAIQCKFYEPHH-TVRKE
gi|671521483|ref|WP_031506489.1|damageinducibleprotein[Streptomycesmegasporus]/11630    ----QDKRDTGIDLVAQDR-E----------TG-------GFCAIQCKFYEPHH-TVRKE
gi|740053144|ref|WP_037902776.1|damageinducibleprotein[Streptomycessp.TAA204]/11626    ----HDKRDTGIDLVAQDR-E----------TG-------GFCAIQCKFYEPHH-TIRKE
gi|663409375|ref|WP_030404974.1|MULTISPECIES:damageinducibleprotein[Streptomyces]/11627    ----HDKRDTGIDLVAQDR-E----------TG-------GFCAIQCKFYEPQH-TIQKD
gi|746628608|ref|WP_039654277.1|damageinducibleprotein[Streptomycessp.MUSC125]_gi|745644381|gb|KIE25557.1|damageinducibleprot/1    ----HDKRDTGIDLVAQDR-E----------TG-------GFCAIQCKFYEPQH-TIQKD
gi|727322066|ref|WP_033777320.1|damageinducibleprotein[Salinisporapacifica]/11627    ----HDKRDVGIDLVAQDR-E----------TG-------GYCAIQCKFYEPHH-TVEKA
gi|653653242|ref|WP_027655726.1|damageinducibleprotein[Salinisporapacifica]/11618    ----HDRRDTGIDLVALDR-E----------TG-------GYCAIQCKFYEPHH-TVQKS
gi|648632543|ref|WP_026324294.1|damageinducibleprotein[Salinisporapacifica]/11621    ----HDRRDTGIDLVALDR-E----------TG-------GYCAIQCKFYEPHH-TVEKA
gi|881018002|ref|WP_048738463.1|damageinducibleprotein[Corynebacteriumfalsenii]/11676    ----N-QPDTGIDLVARRR-E----------DA-------NWVAIQCKFYKSSS-TISKS
gi|505465526|ref|WP_015652417.1|helicase[Corynebacteriumcallunae]_gi|459385704|gb|AGG67995.1|helicase[CorynebacteriumcallunaeDS    ----G-TMDTGIDLVAYNK-D----------DD-------AYTAIQCKFYLPTT-SLAKG
gi|41223062|emb|CAF19007.1|DNAorRNAhelicaseofsuperfamilyII[CorynebacteriumglutamicumATCC13032]/11646    ----G-TMDTGIDLVAYNK-D----------DD-------AYTAIQCKFYLPTT-SLAKG
CGL13032I/11643    ----G-TMDTGIDLVAYNK-D----------DD-------AYTAIQCKFYLPTT-SLAKG
gi|751257081|ref|WP_040967675.1|damageinducibleprotein[Corynebacteriumglutamicum]_gi|748811620|gb|AJE67931.1|damageinduciblep/1    ----G-KADTGIDLVARRR-E----------DG-------RWTAIQCKFYLPTT-SLQKQ
gi|749040539|ref|WP_040086524.1|damageinducibleprotein[Corynebacteriumhumireducens]/11663    ----N-LPDTGIDLVARHA-E----------SG-------RWAAIQCKFYAPTT-YLQKS
gi|491662899|ref|WP_005519615.1|helicase[Corynebacteriummatruchotii]_gi|224946756|gb|EEG27965.1|helicaseCterminaldomainprotein/    ----G-TSDQGIDLVARRR-E----------DG-------RWAAVQCKFYAEST-TLQKQ
gi|746717312|ref|WP_039676231.1|damageinducibleprotein[Corynebacteriumminutissimum]_gi|734991118|gb|KHO29219.1|damageinducibl/1    ----G-KQDTGIDLVARRA-D----------DD-------AWVAIQCKFYKPDT-YIQKR
gi|908654892|emb|CQD15529.1|SuperfamilyIIDNA/RNAhelicase(modularprotein)[Corynebacteriumstriatum]/11674    ----G-KQDTGIDLVARRA-D----------DD-------AWVAIQCKFYKPDT-YIQKR
gi|895994446|ref|WP_049064418.1|damageinducibleprotein[Corynebacteriumaurimucosum]/11667    ----G-KQDTGIDLVARRA-D----------DD-------AWVAIQCKFYKPDT-YIQKR
gi|896123331|ref|WP_049145390.1|damageinducibleprotein[Corynebacteriumaurimucosum]/11669    ----G-KQDTGIDLVARRA-D----------DD-------AWVAIQCKFYKPDT-YIQKR
gi|817681178|ref|WP_046645422.1|damageinducibleprotein[Corynebacteriumstriatum]_gi|817551679|gb|KKO79868.1|damageinduciblepro/1    ----G-KQDTGIDLVARRA-D----------DD-------AWVAIQCKFYKPDT-YIQKR
gi|896139239|ref|WP_049159146.1|damageinducibleprotein[Corynebacteriumaurimucosum]/11675    ----G-KQDTGIDLVARRA-D----------DD-------AWVAIQCKFYKPDT-YIQKR
gi|489943947|ref|WP_003847254.1|helicase[Corynebacteriumammoniagenes]_gi|295968218|gb|EFG81467.1|helicaseCterminaldomainprotei/    ----G-KSDTGIDLVAHRI-D----------DD-------SWVAIQAKFYKETT-SIQKS
gi|516653464|ref|WP_018022453.1|helicase[Corynebacteriumdoosanense]_gi|697778282|gb|AIT61018.1|damageinducibleprotein[Coryneba/    ----G-KADTGIDLVAHRI-E----------DD-------SWVAIQAKFYLPTT-SIQKS
gi|493894179|ref|WP_006840129.1|helicase[Corynebacteriumlipophiloflavum]_gi|227078819|gb|EEI16782.1|helicaseCterminaldomainpro/    ----G-NADTGIDLVARRR-E----------DG-------RWTAIQCKFYKEST-YLQKS
gi|740831472|ref|WP_038616755.1|damageinducibleprotein[Corynebacteriumpseudotuberculosis]_gi|666103058|gb|AIG06935.1|putativeh/    ----G-KPDTGIDIVAKRK-E----------DG-------SWVAIQAKFYQEST-SIQKR
gi|518023322|ref|WP_019193530.1|helicase[Corynebacteriumtimonense]/11655    ----G-LADTGIDIVAHRK-D----------DD-------TWVAIQAKFYKPNT-SIHKA
gi|552767697|ref|WP_023019559.1|MULTISPECIES:hypotheticalprotein[Corynebacterium]_gi|550739013|gb|ERS38609.1|hypotheticalprotei    ----G-QTDTGIDMVAHRV-A----------DD-------SWVAIQAKFYKPTT-SIQKG
gi|652659121|ref|WP_027017812.1|damageinducibleprotein[Corynebacteriumpseudodiphtheriticum]/11644    ----G-QTDTGIDMVAHRV-A----------DD-------SWVAIQAKFYKPTT-SIQKG
gi|749039309|ref|WP_040085390.1|damageinducibleprotein[Corynebacteriumhumireducens]/11657    ----G-QIDTGIDLVARRR-E----------DK-------TWTAIQCKFYLPTT-RIQKP
gi|740835591|ref|WP_038620874.1|damageinducibleprotein[Corynebacteriumulcerans]_gi|698973854|gb|AIU33664.1|HelicaseCterminald/1    ----G-KADTGIDLVARRK-E----------DS-------RWVAIQAKFYLPTT-SIQKR
gi|805307599|ref|WP_046096452.1|damageinducibleprotein[Corynebacteriumulcerans]_gi|803357323|gb|AKA97638.1|Damageinduciblepro/1    ----G-KADTGIDLVARRK-E----------DS-------RWVAIQAKFYLPTT-SIQKR
gi|504083555|ref|WP_014317549.1|helicase[Corynebacteriumdiphtheriae]_gi|371584249|gb|AEX47914.1|superfamilyIIDNA/RNAhelicase[Co    ----G-KVDTGIDLVAHRI-E----------DD-------SWTAIQCKFYKPTT-RIEKS
gi|896182193|ref|WP_049193580.1|damageinducibleprotein[Corynebacteriumjeikeium]/11645    ----G-KVDTGIDLVAHRI-E----------DD-------SWTAIQCKFYKPTT-RIDKS
gi|917636459|ref|WP_052205460.1|damageinducibleprotein[Corynebacteriumriegelii]_gi|914295811|gb|AKV59187.1|damageinduciblepro/1    ----G-QTDTGIDLVARRR-D----------DG-------KWVAIQAKFYKPTT-SIQKQ
gi|737013522|ref|WP_035009205.1|damageinducibleprotein[Corynebacteriumjeikeium]/11651    ----G-KTDTGIDLVARRR-D----------DG-------GWVAIQAKFYKQTT-SIQKQ
gi|737006291|ref|WP_035002094.1|damageinducibleprotein[Corynebacteriumjeikeium]/11658    ----G-KADTGIDLVARRA-D----------DV-------TWTAIQCKFYKPTT-SIQKA
gi|737010378|ref|WP_035006133.1|damageinducibleprotein[Corynebacteriumjeikeium]/11650    ----G-KADTGIDLVARRV-D----------DD-------SWTAIQCKFYKPTT-SISKA
gi|737010473|ref|WP_035006228.1|damageinducibleprotein[Corynebacteriumjeikeium]/11657    ----G-KADTGIDLVARRT-E----------DG-------AWTAIQCKFYLDST-PLPKS
gi|737006250|ref|WP_035002053.1|damageinducibleprotein[Corynebacteriumjeikeium]/11658    ----G-KADTGIDLVARRS-E----------DG-------AWTAIQCKFYLDST-PLPKS
gi|923018168|ref|WP_053412254.1|damageinducibleprotein[Corynebacteriumlactis]_gi|922845184|gb|ALA67504.1|helicase[Corynebacter/    ----G-KPDTGIDLVARRI-D----------DG-------SWTAIQCKFYLPTT-RIDKA
gi|923018885|ref|WP_053412970.1|damageinducibleprotein[Corynebacteriumlactis]_gi|922845845|gb|ALA68165.1|helicase[Corynebacter/    ----G-KPDTGIDLVARRI-D----------DG-------SWTAIQCKFYLPTT-RIDKA
gi|512464870|ref|WP_016422869.1|hypotheticalprotein[Corynebacteriumsp.HFH0082]_gi|512047830|gb|EPD47505.1|hypotheticalproteinHM    ----G-APDTGIDLVARRI-D----------DG-------TWVAIQCKFYMPTT-RLAKA
gi|881012697|ref|WP_048733406.1|damageinducibleprotein[Corynebacteriumurealyticum]/11670    ----G-APDTGIDLVARRV-D----------DG-------TWVAIQCKFYMPTT-RLAKA
gi|755608478|ref|WP_042530519.1|damageinducibleprotein[Corynebacteriumsingulare]_gi|754340239|gb|AJI78730.1|putativehelicase[C/    ----G-KADTGIDLVARRV-D----------DG-------TWTAIQCKFYDSKA-RIQKS
gi|491421796|ref|WP_005279593.1|helicase[Corynebacteriumaccolens]_gi|227077180|gb|EEI15143.1|helicaseCterminaldomainprotein[Co/    ----G-KADTGIDLVARRR-D----------DG-------TWTAIQCKFYASTA-SIQKS
gi|550752286|gb|ERS51451.1|hypotheticalproteinHMPREF1282_00033[Corynebacteriumsp.KPL1856]_gi|550759961|gb|ERS58964.1|hypothetic    ----G-KADTGIDLVARRV-D----------DG-------TWMAIQCKFYASNA-RIQKS
gi|497955053|ref|WP_010269209.1|helicase[Corynebacteriumbovis]/11599    ----G-RPDTGVDLVAHRR-E----------DD-------SWTAVQCKFYESTT-TLSKG
gi|860580040|ref|WP_048470569.1|hypotheticalprotein[Mycobacteriumchlorophenolicum]_gi|859098575|gb|KMO76871.1|typeIrestrictione    ----G-KPDTGIDLVARER-D----------TG-------EYTAIQCKFYEPTT-TLQKE
gi|262085905|gb|ACY21873.1|typeIIIrestrictionproteinressubunit[GordoniabronchialisDSM43247]/11632    ----G-KPDTGIDIVARER-D----------TG-------DYTAIQCKFYEPTH-TLAKG
```

```
gi|495996338|ref|WP_008720917.1|typeIIIrestrictionendonucleasesubunitR[Rhodococcussp.AW25M09]_gi|443412350|emb|CCQ18455.1|TypeI    ----G-KADTGIDLVARER-D----------TG-------DYAAIQCKFYEPTT-TLSKG
gi|739284636|ref|WP_037147410.1|damageinducibleprotein[Rhodococcusfascians]/11641    ----G-KADTGIDLVARER-D----------TG-------DYTAIQCKFYEPTT-TLSKG
gi|739328195|ref|WP_037190574.1|damageinducibleprotein[Rhodococcusfascians]/11643    ----G-KADTGIDLVARER-D----------TG-------DYAAIQCKFYEPTT-TLSKG
gi|811698335|emb|CMN21041.1|helicase[Mycobacteriumtuberculosis]/11725    ----G-RTDTGIDLVARER-D----------TG-------NYTAIQCKFYEPTH-TLAKG
gi|801577898|emb|CFK23773.1|helicase[Mycobacteriumtuberculosis]/11675    ----G-RTDTGIDLVARER-D----------TG-------NYTAIQCKFYEPTH-TLAKG
MTU18II/11606    ----G-RTDTGIDLVARER-D----------TG-------NYTAIQCKFYEPTH-TLAKG
gi|887719858|emb|CMT27886.1|helicase[Mycobacteriumtuberculosis]/11667    ----G-RTDTGIDLVARER-D----------TG-------NYTAIQCKFYEPTH-TLAKG
gi|621694743|gb|KAS87156.1|hypotheticalproteinAL70_01801[MycobacteriumtuberculosisTKK_03_0115]/11604    ----G-RTDTGIDLVARER-D----------TG-------NYTAIQCKFYEPTH-TLAKG
gi|888128072|emb|CLQ84422.1|helicase[Mycobacteriumtuberculosis]_gi|889917090|emb|CLY86893.1|helicase[Mycobacteriumtuberculosis]    ----G-RTDTGIDLVARER-D----------TG-------NYTAIQCKFYEPTH-TLAKG
gi|884823424|emb|CKL96672.1|helicase[Mycobacteriumtuberculosis]/11466    ----G-RTDTGIDLVARER-D----------TG-------NYTAIQCKFYEPTH-TLAKG
gi|505103239|ref|WP_015290341.1|hypotheticalprotein[Mycobacteriumcanettii]_gi|432162733|emb|CCK60117.1|Conservedproteinofunknow    ----G-RTDTGIDLVARER-D----------TG-------NYTAIQCKFYEPTH-TLAKG
gi|505116250|ref|WP_015303352.1|hypotheticalprotein[Mycobacteriumcanettii]_gi|432158726|emb|CCK56026.1|Conservedproteinofunknow    ----G-RTDTGIDLVARER-D----------TG-------NYTAIQCKFYEPTH-TLAKG
gi|432154735|emb|CCK51974.1|Conservedproteinofunknownfunction(part1)[MycobacteriumcanettiiCIPT140060008]/11588    ----G-RTDTGIDLVARER-D----------TG-------NYTAIQCKFYEPTH-TLAKG
gi|809008027|ref|WP_046295375.1|hypotheticalprotein[Mycobacteriumsp.UM_Kg17]/11624    ----G-KADTGIDLVARER-D----------TG-------DYTAIQCKFYEPTH-TLAKG
gi|808802633|ref|WP_046284139.1|hypotheticalprotein[Mycobacteriumsp.UM_NZ2]/11624    ----G-KADTGIDLVARER-D----------TG-------DLTAIQCKFYEPTH-TLAKG
gi|750125774|ref|WP_040429852.1|damageinducibleprotein[Corynebacteriumnuruki]/11668    ----N-HGDNGIDIVAHVR-E----------TGPDFGTDDEFAAVQCKFYDPEH-RLAKQ
gi|602237614|gb|EYT60960.1|damageinducibleprotein[Dietziasp.UCDTHP]/11473    ----G-KADMGIDLVARVR-D----------TH-------EYAAIQCKFYAPDH-HLSKA
gi|665897412|ref|WP_031264618.1|damageinducibleprotein[Dietziasp.UCDTHP]_gi|602237599|gb|EYT60945.1|helicase[Dietziasp.UCDTH/11    ----G-KPDNGIDLVARVR-D----------TG-------EYAAIQCKFYAPDH-TLTKA
gi|502903674|ref|WP_013138650.1|helicase[Segniliparusrotundus]_gi|296181291|gb|ADG98197.1|typeIIIrestrictionproteinressubunit[S    ----S-KADMGIDLVARSR-D----------TG-------ELTGVQCKFYEPEH-TLRKE
gi|780248306|ref|WP_045538930.1|hypotheticalprotein[Gordoniasp.no.9]_gi|778142514|gb|KJR06917.1|hypotheticalproteinUG54_12320[G    ----G-KPDTGIDLVARDR-V----------TG-------EWTAIQCKFYEPEH-HLSKK
gi|665897368|ref|WP_031264574.1|damageinducibleprotein[Dietziasp.UCDTHP]_gi|602239137|gb|EYT62483.1|helicase[Dietziasp.UCDTH/11    ----G-KPDTGIDLVARER-D----------TG-------EYTAVQCKFYEPEH-YLQKV
gi|493372923|ref|WP_006329236.1|helicase[Gordoniarhizosphera]_gi|403207917|dbj|GAB88168.1|hypotheticalproteinGORHZ_006_00370[Go    ----G-KPDTGIDLVARTR-D----------TG-------ALTAIQCKFYEPTT-TLAKD
gi|764904870|ref|WP_044487821.1|hypotheticalprotein[Mycobacteriumtuberculosis]_gi|625005353|gb|KBZ57120.1|hypotheticalproteinK8    ----G-KPDTGIDLVARER-D----------SG-------EFTAIQCKFYEPTH-QLRKE
gi|927068212|ref|WP_053778441.1|damageinducibleprotein[Gordoniasp.NB41Y]_gi|926660541|gb|EMP14526.2|damageinducibleprotein[G/11    ----G-KPDTGIDLVARER-D----------NG-------AYTAIQCKFYEPTH-TLRKE
gi|935514169|ref|WP_054429894.1|damageinducibleprotein[Mycobacteriumimmunogenum]_gi|929801120|gb|KPG29247.1|damageinduciblepr/1    ----G-KPDTGIDLVARER-D----------TG-------EYTAIQCKFYEPAH-TLRKE
gi|941317728|ref|WP_055118574.1|hypotheticalprotein[Mycobacteriumperegrinum]/11657    ----G-KPDTGIDLVARER-D----------SG-------NLTAIQCKFYEPTH-TLRKE
gi|933683673|gb|ALG86377.1|hypotheticalproteinACH46_20120[Gordoniasp.QH11]/11636    ----G-RPDTGIDLVARER-D----------TG-------ELTAIQCKFYEPTT-TLSKS
gi|873553947|emb|CRY84110.1|Predictedhelicase[Nocardiafarcinica]/11688    ----G-KPDTGIDLVARER-D----------TG-------ALTAIQCKFYEPTH-VLAKG
gi|499525749|ref|WP_011212389.1|helicase[Nocardiafarcinica]_gi|54019339|dbj|BAD60707.1|hypotheticalprotein[NocardiafarcinicaIFM    ----G-KPDTGIDLVARER-D----------TG-------ALTAIQCKFYEPTH-VLAKG
gi|640109609|ref|WP_024794187.1|damageinducibleprotein[Tomitellabiformata]/11653    ----G-KPDTGIDLVARER-D----------TG-------AYTAIQCKFYEPTS-TLSKG
gi|640109991|ref|WP_024794334.1|hypotheticalprotein[Tomitellabiformata]/11639    ----G-KPDTGIDLVARER-D----------TG-------AYTAIQCKFYEPTS-TLSKG
gi|939125496|ref|WP_054831438.1|damageinducibleprotein[Rhodococcuserythropolis]/11669    ----G-KTDTGIDLVARER-D----------TG-------EYTAIQCKFYEPTH-TLAKG
gi|910258550|ref|WP_050062711.1|hypotheticalprotein[Rhodococcussp.RD6.2]_gi|846871967|emb|CRK49225.1|conservedhypotheticalprote    ----G-KPDTGIDLVARER-D----------TG-------AYTAIQCKFYEPTH-TLAKG
gi|797148189|gb|KJY52358.1|putativeHelicase[Bifidobacteriumasteroides]/11801    -N-LP-RNDTGIDLVARGV------------DG-------SLWAIQCKYVETEH-VIDKA
gi|535476570|gb|EFG27357.2|hypotheticalproteinHMPREF9020_00999[ScardoviainopinataF0304]/11827    -D-FP-KNDTGIDLVAKDR------------SG-------ELWAIQCKYYADDR-KLDFS
gi|737482729|ref|WP_035462496.1|hypotheticalprotein[Alicyclobacillusmacrosporangiidus]/11512    ---YS-LQDEGIDLVAEDT------------DH-------NLWAIQSKDHRA---PVDWR
gi|685434354|gb|KGA95679.1|helicase[BacillusalcalophilusATCC27647=CGMCC1.3604]/11575    -N-IP-KKDTGVDLVARKR-E----------TG-------ELIAVQCKFYSKGT-TIRKE
gi|945370253|gb|KQU27251.1|helicase[Bacillussp.Leaf13]/11557    -G-IP-KTDTGVDLVAKER-E----------TG-------LLVAIQCKYYAEDT-IIQKQ
gi|880997015|ref|WP_048719116.1|helicase[Enterococcushirae]/11563    -H-IS-RKDTGVDLVARIR-D----------TG-------KLVAIQCKYYPKNK-KIDKK
gi|945356929|gb|KQU14100.1|helicase[Bacillussp.Leaf49]/11547    -G-IP-KTDTGVDLVARER-D----------TG-------DLVAIQCKYYSEGT-TIQKS
gi|763482876|gb|KIZ53217.1|helicase[Bacillussafensis]/11563    -G-VP-KKDTGVDLVARRR-E----------TG-------ELVAIQCKYYSMDT-TIQKS
gi|852223292|ref|WP_048309707.1|helicase[Anaerobacillusmacyae]_gi|850322910|gb|KMM38590.1|helicase[Anaerobacillusmacyae]/11573    -G-IP-KKDTGVDLVARYR-D----------TG-------ELVAIQCKYFSKDT-TIQKL
gi|739708665|ref|WP_037562619.1|helicase[Sporolactobacillusterrae]/11573    -G-IS-KKDTGVDLVARNR-E----------TG-------ELIAIQCKYYSKDT-TIQKS
gi|496687561|ref|WP_009329104.1|MULTISPECIES:helicase[Bacillus]_gi|317390867|gb|EFV71668.1|endonucleaseandmethylaseLlaGI[Bacill    -G-IP-KKDTGVDLVARKR-E----------TG-------ELIAIQCKYYSKDT-TIQKS
gi|930213599|gb|KPH74225.1|helicase[Oceanobacilluscaeni]/11569    -G-IP-KRDTGVDLVARNR-E----------TG-------ELFAIQCKYYSNDT-SIQKS
gi|516366003|ref|WP_017756036.1|helicase[Bacillussp.ZYK]/11571    -G-IP-KKDTGVDLVARNR-E----------TG-------ELVAIQCKYYAKET-TIQKS
gi|488909977|ref|WP_002821052.1|helicase[Oenococcusoeni]_gi|399965230|gb|EJN99855.1|endonucleaseandmethylaseLlaGI[Oenococcusoen    -G-IP-KKDTGVDIVASDK-L----------TG-------DLTAVQAKFYQG---KVGKA
gi|495019967|ref|WP_007745780.1|helicase[Oenococcuskitaharae]_gi|366983682|gb|EHN59081.1|typeIIIrestrictionenzyme,ressubunit[Oe    -G-IP-KKDTGVDIVASDK-L----------TG-------ELTAVQAKFYQG---KVGKA
gi|489666119|ref|WP_003570406.1|typeIIIrestrictionendonuclease[Lactobacilluscasei]_gi|410527834|gb|EKQ02696.1|typeIIIrestrictio    -Q-IP-RNDLGVDLVCRHR-G----------TG-------KLTAVQAKYYRG---KVGKD
gi|511447623|gb|EPC78110.1|superfamilyIIDNA/RNAhelicase[LactobacillusparacaseisubspparacaseiLpp221}/11615    -H-IP-KKDLGVDLVARYR-D----------SG-------ELTAVQAKYYHG---KVSKD
gi|497700411|ref|WP_010014595.1|helicase[Lactobacilluscoryniformis]/11585    -H-IP-KKDLGVDLVARHR-D----------SG-------ELTAVQAKYYRG---KVGKD
gi|489784048|ref|WP_003687939.1|helicase[Lactobacillusmali]_gi|394428539|gb|EJF01080.1|superfamilyIIDNA/RNAhelicase[Lactobacill    -H-IP-KKDLGVDLVARYR-D----------SG-------ALTAVQAKYYRG---KVGKD
gi|446780825|ref|WP_000858081.1|helicase[Staphylococcusaureus]_gi|374396604|gb|EHQ67835.1|typeIIIrestrictionenzyme,ressubunit[S    -G-IP-KQDTGVDLVAKNR-I----------TG-------KLTAIQAKFYQG---KIGKA
gi|510796658|ref|WP_016171456.1|hypotheticalprotein[Enterococcusdispar]_gi|508167532|gb|EOT44083.1|hypotheticalproteinOMK_00227    -G-IS-KKDTGVDLVARNK-N----------TG-------ELTAIQAKFYSG---KVGKA
gi|612613473|gb|EZS30411.1|hypotheticalproteinW603_02482[StaphylococcusaureusVET0353R]_gi|612881917|gb|EZU93992.1|hypotheticalp    -G-IS-KKDTGVDLVARNK-N----------TG-------ELTAIQAKFYSG---KVGKA
gi|488332893|ref|WP_002402278.1|helicase[Enterococcusfaecalis]_gi|306513864|gb|EFM82466.1|helicaseCterminaldomainprotein[Enter/    -V-IP-KKDTGVDLVARNE-A----------TG-------ELTAIQAKFYDN---KIYKR
gi|921166029|ref|WP_053111806.1|helicase[Enterococcusdurans]_gi|915771660|gb|AKX85928.1|helicase[Enterococcusdurans]/11562    -A-IP-KKDTGVDLVARNE-A----------TG-------ELTAIQAKFYDN---KIYKR
gi|498525358|ref|WP_010825030.1|hypotheticalprotein[Enterococcusfaecalis]_gi|487059631|gb|EOJ86727.1|hypotheticalproteinWOC_005    -V-IP-KKDTGVDLVARNE-A----------TG-------ELTAIQAKFYDN---KIYKR
gi|498412624|ref|WP_010722070.1|hypotheticalprotein[Enterococcusfaecium]_gi|486634654|gb|EOF75253.1|hypotheticalproteinSGC_0253    -A-IP-KKDTGVDLVARKE-T----------TG-------ELTAIQAKFYDN---KIYKR
gi|498408534|ref|WP_010718364.1|hypotheticalprotein[Enterococcusfaecium]_gi|486609608|gb|EOF50873.1|hypotheticalproteinSCW_0257    -A-IP-KKDTGVDLVARNE-A----------TG-------ELTAIQAKFYDN---KIYKR
gi|498407475|ref|WP_010717483.1|hypotheticalprotein[Enterococcusfaecalis]_gi|486594855|gb|EOF36348.1|hypotheticalproteinSCK_005    -V-IP-KKDTGVDLVARNE-A----------TG-------ELTAIQAKFYDN---KIYKR
gi|514885392|ref|WP_016615344.1|helicase[Enterococcusfaecalis]_gi|514505843|gb|EPH71676.1|helicaseprotein[Enterococcusfaecalis0    -V-IP-KKDTGVDLVARNE-A----------TG-------ELTAIQAKFYDN---KIYKR
gi|325157603|gb|EGC69759.1|helicaseCterminaldomainprotein[EnterococcuscasseliflavusATCC12755]/11561    -A-IP-KKDTGVDLVARNE-A----------TG-------ELTAIQAKFYDN---KIYKR
gi|895856206|ref|WP_048967668.1|helicase[Enterococcusfaecalis]/11551    -A-IP-KKDTGVDLVARNE-A----------TG-------ELTAIQAKFYDN---KIYKR
gi|639458836|ref|WP_024636366.1|helicase[Enterococcusfaecium]_gi|658176502|gb|KEI51159.1|helicase[EnterococcusfaeciumUC7256]/1/    -A-IP-KKDTGVDLVARNE-A----------TG-------ELTAIQAKFYDN---KIYKR
gi|488266511|ref|WP_002337719.1|hypotheticalprotein[Enterococcusfaecium]_gi|430581409|gb|ELB19854.1|hypotheticalproteinOIQ_0351    -P-IP-KKDTGVDLVARNE-A----------TG-------DLTAIQAKFYDN---KIYKR
gi|446353356|ref|WP_000431211.1|helicase[Staphylococcusaureus]_gi|253726329|gb|EES95058.1|helicaseCterminaldomainprotein[Staph/    -N-IS-KKDLGVDLVAEQ--Q----------NG-------DLVAIQAKFNKG---KIGKS
```

gi|672712068|emb|CDM14336.1|DNAorRNAhelicaseofsuperfamilyII[StaphylococcusepidermidisPM221]/11464	-G-LPDKKDKGVDLVAEQ--F----------NG-------DIIAIQAKFYKN---SIGKS
gi|896298990|ref|WP_049279905.1|helicase[Staphylococcusaureus]/11571	-G-LPDKKDKGVDLVAEQ--F----------NG-------DIIAIQAKFYKN---SIGKS
gi|541903432|gb|ERH36190.1|hypotheticalproteinSEQU_00085[StaphylococcusequorumUMCCNS924]/11578	-G-LPDKKDKGVDLVAEQ--F----------NG-------DIIAIQAKFYRN---TIGKS
gi|488289747|ref|WP_002360955.1|helicase[Enterococcusfaecalis]_gi|256682216|gb|EEU21911.1|endonucleaseandmethylaseLlaGI[Enteroc	-G-IP-KKDTGVDLVAEQ--F----------NG-------DLVAIQAKFYQD---KIGKN
gi|494392045|ref|WP_007207894.1|helicase[Enterococcusitalicus]_gi|315483798|gb|EFU74284.1|helicaseCterminaldomainprotein[Enter/	-G-IP-KKDTGVDLVAEQ--F----------NG-------ELVAIQAKFYQN---KVGKN
gi|446350692|ref|WP_000428547.1|helicase[Bacilluscereus]_gi|228727580|gb|EEL78721.1|RestrictionmodificationsystemLlaBIII[Bacil/	-G-IP-KKDTGVDLVAEQ--F----------NG-------DLVAIQAKFYKK---KIGKD
gi|488255302|ref|WP_002326510.1|hypotheticalprotein[Enterococcusfaecium]_gi|430480276|gb|ELA57459.1|hypotheticalproteinOGE_0473	-G-IP-KKDTGVDLVAEQ--F----------NG-------DLVAIQAKFYEN---KIGKS
gi|843253770|ref|WP_047916698.1|helicase[Lactococcuspiscium]_gi|802124677|emb|CEN29502.1|EndonucleaseandmethylaseLlaGI,TypeISPr	-G-IP-KKDTGVDLVAEQ--K----------NG-------DLVAIQAKFYTN---KVGKS
LlaBIII/11578	-G-IP-KKDTGVDLVAEQ--K----------NG-------DLVAIQAKFYTN---KVGKS
gi|499320531|ref|WP_011011023.1|helicase[Listeriainnocua]_gi|16415805|emb|CAC42019.1|pli0021[ListeriainnocuaClip11262]/11569	-G-IP-KKDTGVDLVAEQ--K----------NG-------DLVAIQAKFYTN---KVGKS
gi|760026859|ref|WP_043709973.1|helicase[Weissellacibaria]_gi|758337165|gb|KIU20061.1|UvsWhelicase[Weissellacibaria]/11567	-G-IS-KKDTGVDLVAQQ--K----------NG-------DLVAIQAKFYTH---KVGKD
LlaGI/11570	-G-IP-KKDTGVDLVAEQ--K----------NG-------DLVAIQAKFYTN---KVGKS
gi|499988414|ref|WP_011669132.1|helicase[Lactococcuslactis]_gi|116109019|gb|ABJ74139.1|DNAorRNAhelicaseofsuperfamilyII[Lactococ	-G-IP-KKDTGVDLVAEQ--K----------NG-------DLVAIQAKFYTN---KVGKS
gi|685938360|ref|WP_031670319.1|helicase[Listeriamonocytogenes]_gi|590403387|gb|EXL23261.1|hypotheticalproteinX846_1956[Listeri	-G-IP-KKDTGVDLVAEQ--K----------NG-------DLVAIQAKFYTN---KVGKS
gi|530793219|gb|EQC56240.1|helicase[Lactococcuslactissubsp.cremorisTIFN5]_gi|530793247|gb|EQC56263.1|helicase[Lactococcuslactis	-G-IP-KKDTGVDLVAEQ--K----------NG-------DLVAIQAKFYTN---KVGKS
gi|695270006|ref|WP_032492075.1|hypotheticalprotein[Listeriamonocytogenes]_gi|695212872|ref|YP_009081522.1|hypotheticalproteinp	-G-IP-KKDTGVDLVAEQ--K----------NG-------DLVAIQAKFYTN---KVGKS
gi|504101310|ref|WP_014335297.1|helicase[Leuconostocmesenteroides]_gi|859247905|gb|AKO63885.1|helicase(plasmid)[Leuconostocmese	-G-IS-KKDTGVDLVAEQ--K----------NG-------DLVAIQAKFYTH---KVGKD
gi|488905190|ref|WP_002816265.1|helicase[Leuconostocmesenteroides]_gi|227351522|gb|EEJ41777.1|helicaseCterminaldomainprotein[L/	-G-IS-KKDTGVDLVAEQ--K----------NG-------DLVAIQAKFYIH---KVGKD
gi|518121285|ref|WP_019291493.1|MULTISPECIES:hypotheticalprotein[Lactococcus]/11588	-D-IP-KKDTGVDLVAKKF------------TG-------ELVAVQAKFYDH---KLYKQ
gi|754857213|ref|WP_042217902.1|helicase[Lactococcusgarvieae]/11549	-G-IP-KNDIGVDLVAQKL------------TG-------ELVAVQAKFYDH---KIQKS
gi|640048455|ref|WP_024784762.1|helicase[Streptococcusmutans]/11564	-G-IP-NADIGVDLVAEKA------------TG-------ELVAIQAKFYNH---AIQKS
gi|512695361|ref|WP_016476323.1|hypotheticalprotein[Veillonellasp.HPA0037]_gi|512079853|gb|EPD79018.1|hypotheticalproteinHMPREF	-G-IP-KTDIGVDLVAEKY------------TG-------KLVAIQAKFYSH---SIQKS
gi|636963514|ref|WP_024403747.1|helicase[Streptococcussuis]/11574	-G-IP-KADIGVDLVAEKH------------TG-------ELVAIQAKFYNH---AIRKK
gi|881115312|ref|WP_048802949.1|helicase[Staphylococcuspasteuri]/11573	-G-IP-KYDIGVDLVAEKV------------TG-------GLVAVQAKFYKN---EIRKK
gi|446063996|ref|WP_000141851.1|helicase[Streptococcusmitis]_gi|307618725|gb|EFN97864.1|endonucleaseandmethylaseLlaGI[Streptoco	-G-IP-KVDLGVDLVAEKF------------TG-------ELVAIQAKFYNH---SIQKS
gi|488206609|ref|WP_002277817.1|restrictionmodificationsystemLlaBIII[Streptococcusmutans]_gi|449173033|gb|EMB75626.1|restricti/	-N-IP-KTDIGVDLVAEKF------------TG-------ELVAIQAKFYNH---AIQKS
gi|640023290|ref|WP_024781621.1|helicase[Streptococcusmutans]/11566	-N-IP-KTDIGVDLVAEKF------------TG-------ELVAIQAKFYNH---AIQKS
gi|814946581|ref|WP_046393781.1|helicase[Streptococcusuberis]_gi|810600885|gb|KKF57499.1|helicase[StreptococcusuberisB362]/1156	-G-IP-KTDIGVDLVAKAY------------TG-------ELVAIQAKFYRH---AIQKS
gi|765470762|ref|WP_044720336.1|helicase[Streptococcussuis]/11559	-G-IP-KTDLGVDLVAETI------------TG-------SLVAIQAKFYTQ---GIQKS
gi|654507127|ref|WP_027976443.1|helicase[Streptococcusdevriesei]/11558	-R-IP-KADIGVDLVAEKH------------TG-------ELVAIQAKFYNH---AIQKS
gi|530812902|gb|EQC72481.1|DNAorRNAhelicaseofsuperfamilyII[Streptococcussp.HSISS2]/11570	-G-IP-KADIGVDLVAEKF------------TG-------ELVAIQAKFYNH---AIQKA
gi|489158846|ref|WP_003068504.1|helicase[Streptococcusintermedius]_gi|355367114|gb|EHG14827.1|hypotheticalproteinHMPREF9682_002	-G-IP-KIDIGVDLVAKKH------------TG-------ELVAIQAKFYNH---SIQKS
RM.Lru27782ORF13200P/11620	-D-IP-HQDTGVDIVARDR-N----------TG-------KLTAIQAKFYKN---KVGKD
gi|492676546|ref|WP_005919236.1|DNAphosphorothioationsystemrestrictionenzyme[Pediococcusacidilactici]_gi|482517924|gb|EOA08103.	-G-IP-KKDTGVDLVAEDR-I----------TG-------ELTAIQAKFYRG---KVGKD
gi|498183116|ref|WP_010497272.1|helicase[Lactobacillusacidipiscis]/11563	-H-VS-RKDTGVDIVARDK-A----------TG-------KLTAIQAKYYKD---KVGKD
gi|822100485|ref|WP_046921865.1|helicase[Lactobacillusruminis]_gi|821684442|gb|KLA43321.1|restrictionmodificationsystemLlaBIII/	-H-IS-KQDTGVDIVARDR-A----------TG-------KLTAVQAKYYRS---KINKK
gi|935565832|ref|WP_054450311.1|helicase[Lactobacilluskunkeei]/11573	-G-IS-RIDTGVDIVAKDF------------NG-------QLTAVQAKFYKG---KVGKD
gi|752296887|ref|WP_041152901.1|helicase[Lactobacilluskunkeei]_gi|751663520|gb|KIM18358.1|helicase[Lactobacilluskunkeei]/11577	-G-IS-RIDTGVDIVAKDF------------NG-------QLTAVQAKFYKG---KVGKD
gi|495745291|ref|WP_008469870.1|helicase[Lactobacillushominis]_gi|394484442|emb|CCI81207.1|RestrictionmodificationsystemLlaBII/	-H-IS-KKDTGVDIVAKDY------------NG-------NLTAVQAKFYKG---KIGKS
gi|918672414|ref|WP_052543134.1|helicase[Lactobacillussp.]/11564	-H-IS-KKDTGVDIVAKDY------------DG-------NLTAVQAKFYKG---KVSKA
gi|799141631|ref|WP_045927261.1|helicase[Lactobacilluskullabergensis]_gi|797155335|gb|KJY59264.1|putativerestrictionendonucleas	-N-IS-KKDTGVDIVAKGY------------DD-------KLTAVQAKFYKG---KVDKK
gi|504379990|ref|WP_014567092.1|helicase[Lactobacillusjohnsonii]_gi|329666726|gb|AEB92674.1|putativerestrictionendonuclease[Lac	-H-IS-KKDTGVDIVAKDY------------DG-------NLTAVQAKFYKG---KVGKA
gi|489880179|ref|WP_003783646.1|DNAdamageinducibleprotein[Actinomycesnaeslundii]_gi|399901561|gb|EJN84440.1|typeIIIrestriction/	----A-RPDRGIDLVARER-E----------TG-------KLCAVQCKFYDPDTGVITKG
gi|496524497|ref|WP_009232780.1|DNAdamageinducibleprotein[Actinomycessp.oraltaxon849]_gi|365265413|gb|EHM95177.1|hypotheticalp/	----T-RPDHGIDLVARER-E----------TG-------ELCAVQCKFYDPQTGVITKN
gi|915397782|ref|WP_050783167.1|damageinducibleprotein[Actinomycesoris]/11694	----A-RPDRGIDLVARER-E----------TG-------ELCAVQCKFYDPQTGVITKN
gi|502925248|ref|WP_013160224.1|helicase[Propionibacteriumfreudenreichii]_gi|296921272|emb|CBL55824.1|helicase[Propionibacteriu	----H-THDSGIDLVARNR-E----------DG-------TWTAIQCKFYDPKH-YLQKG
gi|732164352|emb|CEG87481.1|helicase[Propionibacteriumfreudenreichii]/11618	----G-SHDSGIDLVARDK-V----------SG-------EWTAIQCKFYDPRH-SLQKQ
gi|739047402|ref|WP_036925058.1|damageinducibleprotein[Propionibacteriumthoenii]/11596	----G-THDTGIDLVARER-E----------SG-------RWAAIQCKFYDPRH-RLQKA
gi|739112839|ref|WP_036982533.1|damageinducibleprotein[Propionibacteriumjensenii]/11594	----G-THDTGIDLVARER-E----------SG-------EWTAIQCKFYDPRH-RLQKA
gi|918023558|ref|WP_052314121.1|damageinducibleprotein[Nocardiathailandica]/11612	----H-RTDTGIDLVAQRR-D----------GQ-------GLAAIQCKFYAADH-KVSKA
gi|498380271|ref|WP_010694427.1|DNAdamageinducibleprotein[Saccharopolysporaspinosa]/11605	----G-RPDTGIDLVARNR-D----------TG-------DYAAIQCKCYDSDA-TIAKG
gi|749991459|ref|WP_040339809.1|damageinducibleprotein[CandidatusBlastococcusmassiliensis]/11597	----G-RPDTGIDLVATNT-D----------DD-------GLTAIQCKFYAPGR-MVAKA
gi|754177801|ref|WP_041796231.1|damageinducibleprotein[Modestobactermarinus]/11592	----G-RPDTGIDLVAANA-D----------DG-------GLTAIQCKFYAPGR-TVAKA
gi|504188248|ref|WP_014375350.1|DNAdamageinducibleprotein[Blastococcussaxobsidens]_gi|378782790|emb|CCG02456.1|putativeHelicas/	----G-RPDTGIDLVALNA-D----------DG-------GLTAIQCKFYGSGR-TVAKA
gi|946476713|gb|KRF26798.1|damageinducibleprotein[Phycicoccussp.Soil803]/11609	----K-QHDHGIDIVARDI-H----------NG-------GWCAIQCKFYDPQH-HISKG
gi|700176672|gb|KGN37509.1|DNAdamageinducibleprotein[KnoelliasubterraneaKCTC19937]/11633	----N-AHDHGIDIVAKDE-I----------NG-------GWTAVQCKFYDPQS-YVSKK
gi|700168841|gb|KGN29914.1|hypotheticalproteinN802_10215[KnoelliasinensisKCTC19936]/11631	----N-AHDHGIDIVAKDE-I----------NG-------GWTAVQCKFYDPQH-YVSKK
gi|946426241|gb|KRE76610.1|damageinducibleprotein[Arthrobactersp.Soil763]/11606	----G-KHDTGIDLVARDR-V----------TD-------QYVAIQCKFYAPTS-TISKD
gi|517318525|ref|WP_018500927.1|hypotheticalprotein[Frankiasp.BCU110501]/11592	----G-QHDTGIDLVARDK-D----------TG-------HDVAVQCKFFAPST-YVTKP
gi|499072429|ref|WP_010851327.1|Helicase[Tetrasphaeraelongata]_gi|478756579|emb|CCH71501.1|putativeHelicase[Tetrasphaeraelongat	----G-KHDTGIDIVAVDR-L----------TG-------GNVAIQCKFFDPNS-TISKP
gi|750080666|ref|WP_040385350.1|damageinducibleprotein[Demetriaterragena]/11625	----G-KHDTGIDLVATNK-V----------SG-------GTVAIQCKFYAPES-TISKA
gi|752558292|ref|WP_041229523.1|damageinducibleprotein[Dermacoccusnishinomiyaensis]_gi|664689315|gb|AIF41904.1|damageinducibl/1	----G-KHDTGIDLVAVDA-L----------TG-------ENVAIQCKFYAEQR-HVSKR
gi|829089213|ref|WP_047310701.1|damageinducibleprotein[Dermacoccussp.PE3]_gi|828384812|gb|KLO63770.1|damageinducibleprotein[D/1	----G-KSDTGIDLVAVDR-V----------TG-------ENVAIQCKFYADNH-TVSKG
gi|748756635|ref|WP_040014750.1|damageinducibleprotein[Dermacoccussp.Ellin185]/11596	----G-KTDTGIDLVAVDR-V----------TG-------ENVAIQCKFYAENH-TVSKG
gi|916534724|ref|WP_051141848.1|damageinducibleprotein[Actinomycesneuii]/11632	------RPDCGIDLVAQGR-D-----------G-------SLAAVQCKFYAPGY-HIQKS
gi|921140030|ref|WP_053090276.1|damageinducibleprotein[Rothiamucilaginosa]/11616	------FGDIGIDLVAENV-D-----------G-------GVTAIQCKFYDEHS-TIAKE
gi|896406751|ref|WP_049343749.1|damageinducibleprotein[Rothiamucilaginosa]/11647	------FGDIGIDLVAENV-D-----------G-------GVTAIQCKFYDEHS-TIAKE

```
gi|723608234|gb|AIY01610.1|putativehelicase[Arthrobactersp.PAMC25486]/11613    -N-FS-NPDNGIDLVAARA-D-----------G-------GLTAIQAKFYAPGA-KIQKH
gi|946394527|gb|KRE45160.1|damageinducibleprotein[Arthrobactersp.Soil736]/11616    ------KPDTGIDLVAERA-D-----------G-------GVTAIQCKFYAETH-TMQKH
gi|515765304|ref|WP_017197904.1|hypotheticalprotein[Arthrobactersp.M2012083]/11648    ------KKDNGIDLVAERQ-D-----------G-------GFTAIQCKFYAEGH-RIQKQ
gi|945315587|gb|KQT73127.1|damageinducibleprotein[Microbacteriumsp.Leaf436]/11612    ----N-RTDTGVDLVARNR-D----------TG-------DLTAIQCKFFAPTT-TVTKA
gi|759908497|ref|WP_043594599.1|damageinducibleprotein[Frigoribacteriumsp.MEB024]_gi|758271359|gb|KIU03346.1|damageinduciblep/1    ----G-KPDTGIDLVARER-H----------TG-------DICAIQCKFYAPEH-TMQKS
gi|945072850|gb|KQR34237.1|hypotheticalproteinASF75_00055[Curtobacteriumsp.Leaf154]/11597    ----G-KTDTGIDLVARER-H----------GG-------GLCAIQCKFHDPEN-TIQKS
gi|551263152|ref|WP_022883363.1|DNAdamageinducibleprotein[Glaciibactersuperstes]/11621    ----G-KVDTGIDLVAKER-Y----------GD-------GVCAIQCKFYASSH-SIDRG
gi|945452341|gb|KQV08491.1|hypotheticalproteinASC63_10170[Leifsoniasp.Root112D2]/11623    ----G-KPDTGIDLVARES-Y----------TG-------KLCAIQCKFYASDH-YLQKS
gi|742759483|gb|KIC69912.1|damageinducibleprotein[Kocuriarhizophila]/11610    ----N-RSDIGVDLVGVRA-D----------TG-------EKVAIQCKFKDPDK-VIAKG
gi|737919995|ref|WP_035885304.1|damageinducibleprotein[Kocuriarhizophila]/11611    ----N-RSDIGVDLVGVRA-D----------TG-------EKVAIQCKFKDPDK-VIAKG
gi|737997701|ref|WP_035959325.1|damageinducibleprotein[Kocuriamarina]_gi|725823884|gb|KHE75603.1|damageinducibleprotein[Kocur/1    ----N-RSDIGVDLVGVRA-D----------TG-------EKVAIQCKFKDPDK-VIPKG
gi|664521070|ref|WP_031037650.1|damageinducibleprotein[Streptomycessp.NRRLWC3774]/11624    ----D-KRDTGIDLVAQDR-E----------TG-------GFCAIQCKFYEPQH-TIQKD
gi|500101034|ref|WP_011777041.1|DNAdamageinducibleprotein[Arthrobacteraurescens]_gi|119951547|gb|ABM10457.1|putativeHelicase[A/    ----G-HVDTGIDLVAAER-D----------TG-------DLVAIQCKFYDPAR-PLRKE
gi|916871953|ref|WP_051479009.1|damageinducibleprotein[Arthrobactersp.H5]/11588    ----G-KVDTGIDLVAREK-E----------TG-------ALVGVQCKFYDPAT-TLQKQ
gi|612084649|gb|EZP43369.1|TypeIIIrestrictionenzyme,ressubunit[Micrococcusluteus]/11625    ----N-RPDNGIDIVAKDR-Y----------TG-------ELTGVQVKFFDPEK-KLQKG
gi|759739708|ref|WP_043450422.1|damageinducibleprotein[Arthrobacterphenanthrenivorans]_gi|742758264|gb|KIC68716.1|damageinduc/1    ----G-HVDTGIDLVAKDR-Y----------TG-------ELTGIQCKFYDPAR-TLDKK
gi|636845448|ref|WP_024366823.1|damageinducibleprotein[Arthrobactersp.TB26]/11605    ----G-QVDTGIDLVAKDR-Y----------TG-------ELTAIQCKFYDPQR-TLDKK
gi|500009025|ref|WP_011689743.1|DNAdamageinducibleprotein[Arthrobactersp.FB24]_gi|116613051|gb|ABK05760.1|typeIIIrestrictionen/    ----G-QVDTGIDLVAKDR-Y----------TG-------ELTAIQCKFYDPQR-TLDKK
gi|821249847|gb|KKZ12083.1|hypotheticalproteinTQ37_06090[CandidatusSynechococcusspongiarum15L]/11436    -G-LS-SDDAGIDLVAELK-DE---------PG-------AYAAIQCKFRDIQG-SIAKA
gi|739446902|ref|WP_037306885.1|damageinducibleprotein[Ruegeriahalocynthiae]/11592    -G-WT-AKDTGIDLVAKLA-D----------ED-------GLAAVQCKFYSAQH-RVQKG
gi|640620081|ref|WP_025048131.1|damageinducibleprotein[Sulfitobactermediterraneus]_gi|751956650|gb|KIN77213.1|TypeIIIrestricti/    -G-WT-GKDTGIDLVAKLA-D----------ED-------GYAAVQCKFYAANK-KIQIG
gi|740247634|ref|WP_038088583.1|damageinducibleprotein[Thiobacillusprosperus]_gi|684049748|gb|KFZ90853.1|putativehelicase[Thio/    -D-WK-ADNTGIDLVGQ-T-H----------EG-------TFWAIQCKFYDAGS-TLDKA
gi|518420321|ref|WP_019590528.1|hypotheticalprotein[Thioalkalivibriosp.ALE20]/11603    -------ADAGIDLVAREQ-A----------TG-------DYWAIQCKFFDPDA-TIQKS
gi|500156826|ref|WP_011831496.1|DNAdamageinducibleprotein[Methylibiumpetroleiphilum]_gi|124261892|gb|ABM96881.1|adeninespecifi/    -------VDVGIDLVARER-G----------TG-------EYWAIQCKFLDPDT-YLQKA
gi|769978537|ref|WP_045094522.1|damageinducibleprotein[Legionellafallonii]_gi|698841736|emb|CEG55682.1|Putativeproteinwithboxh/    -------TDVGIDLVACEK-G----------TG-------EYWAIQCKFFDPHH-SLQKS
gi|553752541|ref|WP_023085344.1|hypotheticalprotein[Pseudomonasaeruginosa]_gi|552201952|gb|ERU62653.1|hypotheticalproteinQ088_0    -------MDVGIDLVARER-G----------TG-------DYWAIQCKFYDPGH-TLQKA
gi|880980418|ref|WP_048704461.1|damageinducibleprotein[Thauerahumireducens]_gi|875132785|gb|KMU70944.1|damageinducibleprotein/1    -------MDVGIDLVARER-G----------TG-------DYWAIQCKFYDPDY-ALQKA
gi|740135732|ref|WP_037982815.1|damageinducibleprotein[Thauerasp.27]/11603    -------MDVGIDLVARER-G----------TG-------DYWAIQCKFFDPGY-ALQKA
gi|738017371|ref|WP_035978332.1|damageinducibleprotein[Burkholderiaglumae]/11380    -------ADVGIDLVARER-G----------TG-------NYWAIQCKFYDPSH-TIQKS
gi|498506003|ref|WP_010806671.1|typeIIIrestrictionenzyme,ressubunit[Pandoraeasp.SD62]_gi|490314561|gb|EON12879.1|typeIIIrestri/    -------ADVGIDLVAKER-G----------TG-------NYWAIQCKFYDPSH-TIQKS
gi|500117748|ref|WP_011793753.1|DNAdamageinducibleprotein[Acidovoraxcitrulli]_gi|120587742|gb|ABM31182.1|typeIIIrestrictionenz/    -------TDVGIDLVARER-G----------TG-------EYWAIQCKFFDPDH-YLQKT
gi|651617328|ref|WP_026611444.1|damageinducibleprotein[Methylocaldumszegediense]/11601    -------TDVGIDLVARER-A----------TG-------EYWAIQCKFYDPTH-YLQKS
gi|737210208|ref|WP_035194994.1|damageinducibleprotein[Acidithiobacillusferrivorans]_gi|669953220|emb|CDQ11755.1|Adeninespecif/    HG-FS-AQDKGVDLVAEDR-D-----------G-------GFCAIQCKFYGEDQ-RISMD
gi|935697386|ref|WP_054506180.1|damageinducibleprotein[Achromobactersp.]_gi|928716153|emb|CUK12836.1|Hefnuclease[Achromobacter/    LG-YL-APDTGIDLVAKLR-D----------GQ-------GYCAIQCKFYE--T-PIQMS
gi|429502517|gb|ELA00827.1|adeninespecificDNAmethyltransferase[Cupriavidussp.HMR1]/11599    LG-YN-APDTGIDLVAKLR-D----------GE-------GFCAIQCKFYE--T-PIQMS
gi|506356642|ref|WP_015876361.1|DNAdamageinducibleprotein[Burkholderiaglumae]_gi|237877158|gb|ACR29491.1|AdeninespecificDNAmet/    LG-YK-APDTGIDLVAKLR-D----------DD-------GYCAIQCKFYD--S-SIQMG
gi|696406787|ref|WP_032978003.1|damageinducibleprotein[Bordetellahinzii]_gi|625335076|gb|KCB49343.1|typeIIIrestrictionenzyme,r/    LG-YK-LPDTGIDLVAKLR-D----------ED-------GYCAIQCKFYD--S-PIPLG
gi|817529125|gb|KKO72587.1|damageinducibleprotein[Kerstersiagyiorum]/11592    LG-YK-LPDTGIDLVAKLR-D----------ED-------GYCAIQCKFYD--S-PIPLG
gi|737048910|ref|WP_035044118.1|damageinducibleprotein[Aquabacteriumsp.NJ1]_gi|699052896|gb|KGM38706.1|damageinducibleprotein/1    LG-YK-APDTGIDLVAKLR-D----------EE-------GYCAIQCKFYD--S-PIPLG
gi|822492519|ref|WP_046933319.1|MULTISPECIES:damageinducibleprotein[Xanthomonas]_gi|821703525|gb|KLA61196.1|damageinduciblepr/1    YG-YK-APDTGIDLVAKLR-E----------DE-------GFCAIQCKFYE--T-PIPLG
gi|751252861|ref|WP_040963740.1|damageinducibleprotein[Xanthomonasfuscans]_gi|749808363|gb|KIJ03887.1|damageinducibleprotein[/1    YG-YK-APDTGIDLVAKLR-E----------DE-------GFCAIQCKFYD--T-PIPLG
gi|496774980|ref|WP_009368792.1|DNAdamageinducibleprotein[Bilophilasp.4_1_30]_gi|345039902|gb|EGW44205.1|hypotheticalproteinHM/    QG-YT-LPDTGIDLVAKFR-E----------GE-------TYCAIQCKFYD--S-PVAMS
gi|499241912|ref|WP_010939452.1|DNAdamageinducibleprotein[Desulfovibriovulgaris]_gi|46580580|ref|YP_011388.1|adeninespecificDN/    LG-YK-RPDTGIDLVARFR-D----------GE-------SYCAIQCKFYE--N-AISQG
gi|504274913|ref|WP_014462015.1|DNAdamageinducibleprotein[Bacillusmegaterium]_gi|345446919|gb|AEN91936.1|AdeninespecificDNAmet/    ----N-KPDTGIDLVAQEK-E----------NG-------EYCAIQCKFYGEGH-RIQKG
gi|504895966|ref|WP_015083068.1|typeIIrestrictionenzymeandmethyltransferasesubtypeG[Anabaenasp.90]_gi|413972424|gb|AFW96512.1|t    ----N-MPDTGIDLVAVER-Y----------TG-------DYWAIQCKCYSPDQ-TVEKS
gi|518327765|ref|WP_019497972.1|hypotheticalprotein[Calothrixsp.PCC7103]/11600    ----N-KPDTGIDLVAQER-A----------TG-------DYCAIQCKFYDPNQ-TLDKS
gi|652765503|ref|WP_027079917.1|damageinducibleprotein[Luteimonasmephitis]/11593    ----G-KPDTGIDLVAKER-D----------TG-------NLVAIQCKFYAPTY-TLQKG
gi|896539951|ref|WP_049442123.1|damageinducibleprotein[Stenotrophomonasmaltophilia]/11591    ----G-KADTGIDLVAQER-D----------SD-------ALVAIQCKFYAPSY-TLQKG
gi|500217900|ref|WP_011888043.1|DNAdamageinducibleprotein[Geobacillusthermodenitrificans]_gi|134267954|gb|ABO68149.1|Conserved/    ----G-RTDTGIDLVAEES-L----------TG-------DIWAIQCKFYDPDH-KIQKS
gi|696570298|ref|WP_033102014.1|damageinducibleprotein[Thermoactinomycesdaqus]/11594    ----G-RVDTGIDLVAKEV-G----------SG-------DIWAIQCKFYEHGH-KIQKS
gi|251772067|gb|EES52637.1|putativehelicase[Leptospirillumferrodiazotrophum]/11618    SG-FD-QRDTGIDLVARER-E-----------G-------GYCAIQCKFFDTNY-TIDKK
gi|738124101|ref|WP_036082108.1|damageinducibleprotein[Leptospirillumferriphilum]_gi|685243306|gb|KGA94177.1|putativehelicase[/    CG-FD-QRDTGIDLVARER-E-----------G-------GYCAIQCKFFDPNY-TIDKK
gi|558699223|ref|WP_023524766.1|helicase[Leptospirillumsp.GroupII'CF1']_gi|206603764|gb|EDZ40244.1|Putativehelicase[Leptospiri/    SG-FD-QRDTGIDLVAKER-E-----------G-------GSCAIQCKFFDPSY-TIDKK
gi|406774462|gb|AFS53887.1|putativehelicase[LeptospirillumferriphilumML04]/11610    YQ-FS-QQDTGIDLVAKEW-D-----------G-------GWCAIQCKCFDPHY-NVQKA
gi|740719463|ref|WP_038504749.1|damageinducibleprotein[Leptospirillumferriphilum]_gi|640838125|gb|AIA30173.1|damageinduciblep/1    YQ-FS-QQDTGIDLVARER-D-----------G-------GWCAIQCKCFDPHY-NVQKA
gi|517810766|ref|WP_018980974.1|hypotheticalprotein[Saccharibacterfloricola]/11733    HV-SD-GRDTGIDLVGVRH------------DG-------RAVAIQCKFFTSGA-RVSKK
gi|502471675|ref|WP_012798533.1|helicase[Slackiaheliotrinireducens]_gi|256791761|gb|ACV22431.1|predictedhelicase[Slackiaheliotr    ----N-GHDIGIDLVAQDA-E----------DG-------SYWAIQCKCYDEES-TLDYK
gi|657871713|ref|WP_029576297.1|hypotheticalprotein[Bifidobacteriumthermacidophilum]_gi|673007521|gb|KFJ02535.1|helicase[Bifido    ----D-GQDTGIDLVALD--V----------DG-------SYWAIQAKCYSK---SLSFE
gi|673004757|gb|KFI99791.1|DNAhelicaserestrictionenzymetypeIIIRessubunit[Bifidobacteriumthermacidophilumsubsp.porcinum]/11682    ----D-GQDTGIDLVALD--V----------DG-------SYWAIQAKCYSK---SLSFE
gi|917316092|ref|WP_051922804.1|hypotheticalprotein[Bifidobacteriumstellenboschense]_gi|673003774|gb|KFI98812.1|helicase[Bifido    ---SG-KMDTGIDLVAED--E----------DG-------SYWAIQAKCYSKP--KLAAG
gi|705415694|ref|WP_033502332.1|hypotheticalprotein[Bifidobacteriumpseudolongum]_gi|672982411|gb|KFI77685.1|DNAdamageinducible/    -R-KG-HADTGIDLVALD--E----------DG-------SYWAIQAKCYTGK--KLSER
gi|916591418|ref|WP_051198509.1|hypotheticalprotein[Bifidobacteriumsp.AGR2158]/11704    -R-RG-HTDTGIDLVALD--V----------DG-------SYWAIQAKCYRGK--KLNQD
gi|772242982|gb|AJW78255.1|hypotheticalproteinVO01_03165[Clavibactermichiganensissubsp.insidiosus]/11642    E---G-RPDTGIDLVAKIR------------GG-------GLLAVQVKFFDEMH-RIEKK
gi|916649818|ref|WP_051256909.1|damageinducibleprotein[Actinomycesgraevenitzii]/11677    ----Q-TGDTGIDLVAIPA-DE---------NA-------GLVAIQCKFYAPGH-TVHKR
gi|496504578|ref|WP_009212861.1|helicase[Actinomycessp.oraltaxon180]_gi|315312695|gb|EFU60776.1|helicase[Actinomycessp.oraltaxo    ----R-TGDTGIDLVAIPA-DP---------SA-------GPVAIQCKFYALGH-RIQKA
gi|494862206|ref|WP_007588306.1|helicase[Actinomycessp.ICM39]_gi|398362799|gb|EJN46470.1|typeIIIrestrictionenzyme,ressubunit[Ac    ----R-TGDTGIDLVAIPV-NP---------TA-------GPVAIQCKFYALGH-RIQKA
gi|881057111|ref|WP_048774974.1|damageinducibleprotein[Sanguibacterkeddieii]/11697    ----R-TGDTGIDLVAIPV-HD---------SE-------GPVAIQCKFYALGH-RIQKA
```

```
gi|881020459|ref|WP_048740808.1|damageinducibleprotein[Kytococcussedentarius]/11704    ----R-TGDTGIDLVAIPV-DE---------NA-------GPVAIQCKFYALGH-RIQKA
gi|881052313|ref|WP_048770555.1|damageinducibleprotein[Cellulomonasflavigena]/11705    ----R-TGDTGIDLVAIPV-RD---------GE-------GPVAIQCKFYALGH-RIQKA
gi|490108682|ref|WP_004009424.1|helicase[Mobiluncuscurtisii]_gi|315490465|gb|EFU80089.1|helicaseCterminaldomainprotein[Mobilun/    A---G-NPDTGIDLVAIDV-EDMPANGVVTTDT-------PAVAIQCKFYEAGH-TIQKA
gi|490111935|ref|WP_004012603.1|helicase[Mobiluncusmulieris]_gi|227843812|gb|EEJ53982.1|helicaseCterminaldomainprotein[Mobilun/    A---G-RPDTGIDLVAIEH-QDMPSDGEVTPDT-------PAVAVQCKFYAPQT-KIQKE
gi|490114228|ref|WP_004014848.1|helicase[Mobiluncusmulieris]_gi|269934432|gb|EEZ90993.1|helicaseCterminaldomainprotein[Mobilun/    A---G-RPDTGIDLVAIEH-QDMPSDGEVTPDT-------PAVAVQCKFYAPQT-KIQKE
gi|493333551|ref|WP_006290636.1|helicase[Parascardoviadenticolens]_gi|315119487|gb|EFT82620.1|helicaseCterminaldomainprotein[P/    ----Y-KTDTGVDIIAEDT-QN---------EG-------KYWAIQCKAYDVEH-EFTWE
gi|495817067|ref|WP_008541646.1|helicase,partial[Sutterellaparvirubra]_gi|378598583|gb|EHY31712.1|helicaseprotein,partial[Sutte    YA-VN-AKDYGIDLVATN--AD---------GD-------GFTAVQCKLYGREH-RVTKP
gi|491572696|ref|WP_005430278.1|helicase[Sutterellawadsworthensis]_gi|319805116|gb|EFW01943.1|DNAhelicaserestrictionenzymeTypeI    LE-QS-GKDIGIDLVATLE-DD---------PD-------AFAAIQCKFYDKDA-VVPKS
gi|748686321|ref|WP_039944445.1|hypotheticalprotein[Thermicanusaegyptius]/11573    ----D-EPDLGIDIVAKER--D---------TG-------KLWAIQAKSHEEKV---SYD
gi|916875698|ref|WP_051482754.1|hypotheticalprotein[Thioalkalivibriosp.HK1]/11366    F---D-ARDMGIDLVAKER--D---------SE-------GFCAIQCKFHSSKT-RISSA
gi|821248604|gb|KKZ10960.1|hypotheticalproteinTE42_09075[CandidatusSynechococcusspongiarumSP3]/11598    -G-LT-AGDHGIDLVAELR-DE---------PG-------AYAAIQCKFRATQG-SIAKV
gi|737409843|ref|WP_035390956.1|hypotheticalprotein[Ferrimicrobiumacidiphilum]_gi|765544266|gb|KJE76241.1|typeIrestrictionenzym    ----R-ERDTGIDLVGIER------------DG-------GVCAIQCKFIPAGQ-SVSKP
gi|654906925|ref|WP_028358281.1|damageinducibleprotein[Brackiellaoedipodis]/11679    -G-LS-KKDTGIDLVAQTR------------EG-------EVHAIQCKNYGPNY-SIKKA
gi|502809901|ref|WP_013044877.1|hypotheticalprotein[CandidatusPuniceispirillummarinum]_gi|292663146|gb|ADE38247.1|hypotheticalp    TG-MN-AQDVGIDLVGKRE--G---------ED-------GFCAIQCKFYDPDT-SIAES
gi|251771370|gb|EES51950.1|typeIIrestrictionenzyme,ressubunit[Leptospirillumferrodiazotrophum]/11628    WG-LT-AQDRGIDLVARDE-------------E-------GFCAIQCKFFSPDE-SVNQQ
gi|498328480|ref|WP_010642636.1|DNAdamageinducibleprotein[Acidithiobacillusthiooxidans]/11627    W---N-ADNLGFDLVARTI------------DG-------DYWAVQCKFYDAKS-RIHKE
gi|647633864|ref|WP_025906698.1|damageinducibleprotein[Prochlorococcussp.scB245a_519B7]/11622    Y---S-KDDIGIDLVAKLR--G---------ES-------GYCAIQCKFYKSDH-SISKS
gi|647619599|ref|WP_025893729.1|damageinducibleprotein[Prochlorococcussp.scB241_527N11]/11621    Y---S-KTDIGIDLVAKLK--E---------EE-------GYCAIQCKFYKPDH-SISKA
gi|755034887|ref|WP_042387498.1|hypotheticalprotein[Clostridiumsp.CL6]/11606    V------GDTGIDLVAKTY------------QG-------EFWAIQCKFYDEDY-SISKA
gi|498139798|ref|WP_010453954.1|helicase[SuccinivibrionaceaebacteriumWG1]/11662    I---G-SVDTGIDIVAQTV------------NG-------EFWAIQCKFYSDKT-SISKK
gi|527501386|gb|EPT34571.1|typeIIIrestrictionenzyme,ressubunit[Bacteroidetesbacteriumoraltaxon272str.F0290]/11789    F---G-THDLGIDLVAETD------------QK-------EFWAIQCKCYREDA-VIDMP
gi|496047275|ref|WP_008771782.1|helicase[Bacteroidessp.2_1_33B]_gi|262296443|gb|EEY84373.1|helicaseCterminaldomainprotein[Bact/    F---G-GKDLGIDLVAKTD------------LG-------DYWAIQCKCYDEKA-VISKA
PDI8503III/11664    F---G-GKDLGIDLVAKTD------------LG-------DYWAIQCKCYDEKA-VISKA
gi|738719431|ref|WP_036613715.1|helicase[Parabacteroidesdistasonis]_gi|649542518|gb|KDS49387.1|restrictionendonucleasefamilypro    F---G-GKDLGIDLVAKTD------------LG-------DYWAIQCKCYDEKA-IISKA
gi|659417033|gb|KEJ86133.1|hypotheticalproteinHMPREF1002_00341[Porphyromonassp.31_2]/11656    F---G-GKDLGIDLVAKTD------------LG-------DYWAIQCKCYDEKA-VISKA
gi|567456807|gb|ETK11358.1|hypotheticalproteinT235_15845[Tannerellasp.oraltaxonBU063isolateCell8/11]/11433    L---G-GTDTGIDLVARTY------------SG-------DYWAIQCKCYAEDT-VIDKS
gi|545440279|ref|WP_021677877.1|helicase[Porphyromonasgingivalis]_gi|543985765|gb|ERJ89108.1|helicaseprotein[Porphyromonasgingi    F---G-GKDTGIDLVAKTH------------IG-------DYWAIQCKCYAEDT-VIDKP
gi|497438056|ref|WP_009752254.1|helicase[Prevotellasp.oraltaxon473]_gi|429144866|gb|EKX87968.1|helicaseprotein[Prevotellasp.ora    F---G-GKDTGIDLVAKTH------------DG-------DYWAIQCKCYAENA-VIDKP
gi|490423474|ref|WP_004295683.1|MULTISPECIES:helicase[Bacteroides]_gi|156110098|gb|EDO11843.1|helicaseCterminaldomainprotein[B/    L---G-GKDTGIDLVARTE------------EG-------DYWAIQCKCYKEDS-VIDKP
gi|941777229|ref|WP_055173506.1|helicase[Bacteroidescaccae]_gi|933263395|emb|CUQ00567.1|Predictedhelicase[Bacteroidescaccae]/1/    F---G-GNDTGIDLVAKTE------------LG-------DYWAIQCKCYAENT-IIDKP
gi|492413898|ref|WP_005836892.1|helicase[Bacteroidesuniformis]_gi|392678824|gb|EIY72229.1|hypotheticalproteinHMPREF1072_03583[B    F---G-GNDTGIDLVAKTE------------LG-------DYWAIQCKCYAENT-IIDKP
gi|695455095|ref|WP_032566191.1|helicase[Bacteroidesfragilis]_gi|596095426|gb|EXZ59996.1|restrictionendonucleasefamilyprotein[B    F---G-GTDTGIDLVAKTE------------LG-------DYWAIQCKCYAENT-IIDKP
gi|933227941|emb|CUQ02348.1|Predictedhelicase[Bacteroidesuniformis]/11658    F---G-GTDTGIDLVAKTE------------LG-------DYWAIQCKCYAVDS-IIDKP
gi|494823013|ref|WP_007558421.1|helicase[Bacteroidesplebeius]_gi|198272167|gb|EDY96436.1|helicaseCterminaldomainprotein[Bacter/    F---G-GNDTGIDLVAKTE------------LG-------DYWAIQCKCYAENT-IIDKP
gi|495427212|ref|WP_008151909.1|helicase[Parabacteroidesjohnsonii]_gi|218222381|gb|EEC95031.1|helicaseCterminaldomainprotein[P/    F---G-GTDTGIDLVAKTE------------MG-------DYWAIQCKCYAEDA-VIDKP
gi|933196855|emb|CUO94545.1|typeIrestrictionenzymeEcoKIsubunitR[Bacteroidesuniformis]_gi|933220098|emb|CUN86874.1|typeIrestrict    F---G-GTDTGIDLVAKTE------------MG-------DYWAIQCKCYAEDA-TIDKP
gi|823687748|ref|WP_047101121.1|helicase[Brachyspirahyodysenteriae]_gi|823334537|gb|KLI17932.1|helicase[Brachyspirahyodysenteri    F---G-GHDIGIDLVALNK------------DD-------EYWAVQCKFHSENT-FIDKS
gi|496436691|ref|WP_009145538.1|helicase[Phascolarctobacteriumsuccinatutens]_gi|322414007|gb|EFY04839.1|helicaseCterminaldomai/    F---G-GSDTGIDLVALTK------------DG-------HYWAIQCKCYADDT-VINKA
gi|503372880|ref|WP_013607541.1|helicase[Sphaerochaetaglobosa]_gi|324026933|gb|ADY13692.1|typeIIrestrictionproteinressubunit[S    L---G-GKDLGIDLVAKTT------------LG-------EYWAIQCKFFKEDS-LITKP
gi|505821748|ref|WP_015707145.1|helicase[Treponemaprimitia]_gi|299483503|gb|ADJ19584.1|helicasedomaincontainingprotein[Trepone/    F---S-GKDTGIDLVARTI------------HG-------DYWAIQCKCFKEDT-RINKP
gi|505822741|ref|WP_015708134.1|helicase[Treponemaprimitia]_gi|333739639|gb|AEF85129.1|endonucleaseandmethylaseLlaGI[Treponemap    F---S-GKDTGIDLVARTI------------HG-------DYWAIQCKCFKEDT-RINKP
gi|488791994|ref|WP_002704400.1|helicase[Treponemasaccharophilum]_gi|380767691|gb|EIC01689.1|typeIIrestrictionproteinressubuni    FG-TG-GKDTGIDLVCRTL------------DG-------EYWAVQCKCYQKNK-KIDLA
gi|524671454|emb|CDD81750.1|putativeseptumsitedeterminingproteinMinC[Bacteroidessp.CAG:462]/11642    F---G-GKDIGIDLVAKTF------------SG-------EYWAVQCKCYADDK-YITKP
gi|524573865|emb|CDC95766.1|putativeseptumsitedeterminingproteinMinC[Alistipessp.CAG:268]/11639    F---G-GKDTGIDLVARTV------------EG-------EYWAIQCKCYAANA-FINKP
gi|658617970|ref|WP_029759328.1|helicase[Fusobacteriumnucleatum]/11606    F---G-GSDSGIDLIIHSL------------ED-------EYLAIQCKFYKENS-EISKS
gi|494412037|ref|WP_007216782.1|helicase[Bacteroidescellulosilyticus]_gi|392638921|gb|EIY32752.1|hypotheticalproteinHMPREF1062_    F---G-GKDTGIDLVAETH------------DG-------GYWAIQCKCYRRGA-RIDKP
gi|383107102|gb|AFG37435.1|putativehelicase[SpirochaetaafricanaDSM8902]/11616    L---G-GSDTGIDLVAQTV------------EG-------DYWAIQCKCYQESA-VINKP
SAF8902III/11616    L---G-GSDTGIDLVAQTV------------EG-------DYWAIQCKCYQESA-VINKP
gi|652538894|ref|WP_026932796.1|helicase[Gramellaechinicola]/11633    F---G-GKDTGIDLVGLTF------------EG-------DYWAIQCKCFQESS-TIDKN
gi|639238223|ref|WP_024568839.1|helicase[Elizabethkingiaanophelis]/11628    F---G-GKDIGIDLVALTI------------ND-------EYWAIQCKCFDENA-TIDKK
gi|652520381|ref|WP_026914582.1|helicase[Gramellaportivictoriae]/11623    F---G-GKDTGIDLVALTY------------EG-------DFWAIQCKCYAEKA-RIDKA
gi|494922183|ref|WP_007648219.1|Helicase[Formosasp.AK20]_gi|469825489|gb|EMQ95550.1|Helicasedomainprotein[Formosasp.AK20]/11626    F---G-GKDTGIDLVALTF------------EG-------DYWAIQCKCFAENG-RINKA
gi|522083382|ref|WP_020594591.1|hypotheticalprotein[Spirosomapanaciterrae]/11637    L---G-IFDTGIDLVALTH------------TG-------DYWAIQCKFFKEGG-VIDKP
gi|490455352|ref|WP_004326157.1|helicase[Bacteroidesovatus]_gi|392669277|gb|EIY62768.1|hypotheticalproteinHMPREF1070_03635[Bact    F---G-GKDTGVDLVAYTH------------NG-------DYWAIQCKCYAENA-TIDKP
gi|499516035|ref|WP_011202675.1|helicase[Bacteroidesfragilis]_gi|53713270|ref|YP_099262.1|helicasedomainprotein[Bacteroidesfrag    F---G-GKDTGVDLVAYTH------------NG-------DYWAIQCKCYAENA-TIDKP
gi|651338332|ref|WP_026461320.1|helicase[Adhaeribacteraquaticus]/11620    L---G-GSDTGIDLVALTI------------DG-------DYWAVQCKCYQENT-IIDKP
gi|806964642|gb|AKC17139.1|helicase[Flavobacteriumpsychrophilum]/11639    L---G-GKDTGIDLVALTY------------DG-------DYWAIQCKCYQETS-TIDKP
gi|752684374|ref|WP_041332322.1|helicase[Flavobacteriumpsychrophilum]_gi|666882889|gb|AIG30715.1|helicase[Flavobacteriumpsychro    L---G-GKDTGIDLVALTY------------DG-------DYWAIQCKCYQETS-TIDKP
gi|933196595|emb|CUP04894.1|DNAorRNAhelicasesofsuperfamilyII[Bacteroidesuniformis]/11631    L---G-GGDTGIDLVAITV------------EG-------DYWAIQCKCYQESS-IIDKP
gi|670509109|ref|WP_031456918.1|helicase[Flavobacteriumchungangense]/11625    L---G-SGDTGIDLVALTI------------DG-------DYWAIQCKCYQETS-LIDKP
                                                                                             .* :                           :: :

gi|490190857|ref|WP_004089451.1|hypotheticalprotein[Helicobacterbilis]_gi|476630638|gb|EMZ37196.1|hypotheticalproteinC826_02193    -DLESYFSLLQS------GVGD-I----YFKK-GILITT--T----DISSEVSNTIAQIS
gi|488896133|ref|WP_002807242.1|MULTISPECIES:restrictionendonucleaseEco57I[Campylobacter]_gi|380541096|gb|EIA65377.1|hypothetic    -DLSTYFTKLQS------GVGE-I----KFSK-GIIIST--S----DLTQNAKKEIEQIS
gi|746602240|ref|WP_039628365.1|restrictionendonucleaseEco57I[Campylobacterlari]_gi|744819395|gb|AJD03391.1|putativehelicase[Ca    -DLSTYFSKLQS------GIGE-I----KFSK-GIIISA--S----DLTQNAKKEMKQIL
gi|727317135|ref|WP_033772558.1|restrictionendonucleaseEco57I[Helicobacterpylori]/11622    -ELATFFTQLQS------GVGE-V----RFKK-GIIITT--S----NLTSNAQQAIEQIR
gi|727103490|ref|WP_033618954.1|restrictionendonucleaseEco57I[Helicobacterpylori]/11616    -DLSTFFTQLQS------GVGE-V----RFKK-GIIITT--S----NLSANAQQAIEQIR
```

gi|727340205|ref|WP_033794408.1|restrictionendonucleaseEco57I[Helicobacterpylori]/11609	-DLSTFFTQLQS------GVGE-V----RFKK-GIIITT--S----NLTSNAQQAIEQIR
gi|727292407|ref|WP_033749975.1|restrictionendonucleaseEco57I[Helicobacterpylori]/11611	-DIATFLAILQS------GVGE-V----RFKK-GIIITT--S----NLSANAQQAIEQIR
gi|727289795|ref|WP_033747591.1|restrictionendonucleaseEco57I[Helicobacterpylori]/11612	-DISPFLTQLLI------GVGE-V----KFKK-GIIIST--S----NLTSEAIKEIEQIR
gi|727319676|ref|WP_033775047.1|restrictionendonucleaseEco57I[Helicobacterpylori]/11612	-DLSTFFTQLQS------GVGE-V----RFKK-GIIITT--S----NLSANAQQAIEQIR
gi|447212910|ref|WP_001290166.1|restrictionendonucleaseEco57I[Helicobacterpylori]_gi|308063540|gb|ADO05427.1|hypotheticalprotei	-DIATFLSQLQS------GVKE-V----GFKK-GIIIST--S----NLSSNALKEIEQIR
gi|447212901|ref|WP_001290157.1|restrictionendonucleaseEco57I[Helicobacterpylori]_gi|384557329|gb|AFH97797.1|hypotheticalprotei	-DIATFLSQLQS------GVKE-I----GFKK-GIIIST--S----NLSSNALKEIEQIR
gi|515849671|ref|WP_017280424.1|hypotheticalprotein[Helicobacterpylori]/11615	-DIVTFLSKLQS------GVKE-V----KFKK-GIIIST--S----NLTSATLNEIEQIR
gi|447212900|ref|WP_001290156.1|restrictionendonucleaseEco57I[Helicobacterpylori]_gi|317178970|dbj|BAJ56758.1|TypeIIGrestrictio	-DLSTFFTKLQS------GVGE-V----RFKK-GIIIST--S----NLSSSTLEEIEQIR
gi|447079018|ref|WP_001156274.1|hypotheticalprotein[Helicobacterpylori]/11614	-DISTFLTQLQS------GVGE-V----RFKK-GIIIST--S----NLTSEALKAIEQIR
gi|447212800|ref|WP_001290056.1|hypotheticalprotein[Helicobacterpylori]_gi|532153248|gb|EQL67316.1|restrictionendonucleaseEco57	-DIATFLTQLQS------GVGE-V----RFKK-GIIIST--S----NLSSNALKAIEQIR
gi|505457237|ref|WP_015644339.1|hypotheticalprotein[Helicobacterpylori]_gi|499061160|gb|AGL70074.1|restrictionendonucleaseEco57	-DIATFLTQLQS------GVGE-V----RFKK-GIIIST--S----NLSSNALKAIEQIR
HPYUM037X/11591	-DIATFLTQLQS------GVGE-V----RFKK-GIIIST--S----NLSSNALKAIEQIR
gi|920026297|ref|WP_052937176.1|restrictionendonuclease[Helicobacterpylori]_gi|912528678|gb|KNX48238.1|restrictionendonucleaseE	-DIATFLTQLQS------GVGE-V----RFKK-GIIIST--S----NLSSNALKAIEQVR
gi|915539050|ref|WP_050841327.1|restrictionendonuclease[Helicobacterpylori]_gi|908890887|gb|KNE06183.1|restrictionendonucleaseE	-DISTFLTQLQS------GVGE-V----RFKK-GIIIST--S----HLTSNALKAIEQIR
gi|727101397|ref|WP_033616957.1|restrictionendonucleaseEco57I[Helicobacterpylori]/11592	-DISTFLSQLQA------GVGE-V----RFKK-GIIIST--S----HLTSTALEEIEQIR
gi|562814875|emb|CDK13002.1|TypeISPRMsystem[Helicobacterpylori26695]/11588	-DISPFLTQLQS------GVRE-V----RFKK-GIIIST--S----NLTSNALNAIEQIR
HPYAXVIII/11588	-DISPFLTQLQS------GVRE-V----RFKK-GIIIST--S----NLTSNALNAIEQIR
gi|447079029|ref|WP_001156285.1|restrictionendonucleaseEco57I[Helicobacterpylori]_gi|393085956|gb|EJB86635.1|hypotheticalprotei	-DIATFLHKLQA------GVGE-V----RFKK-GIIIST--S----NLTRAALEEIEQIR
gi|489105953|ref|WP_003015814.1|restrictionendonuclease[Helicobacterpylori]_gi|477503388|gb|ENH59950.1|restrictionendonucleasef	-DIATFLRKLHS------GVGE-V----RFKK-GIIIST--S----NLTRAALEEIEQTR
gi|447079006|ref|WP_001156262.1|restrictionendonucleaseEco57I[Helicobacterpylori]_gi|393067470|gb|EJB68280.1|hypotheticalprotei	-DISTFLTQLQS------GVGE-I----RFKK-GIIIST--S----HLTSEAIKAIEQIR
gi|727314589|ref|WP_033770298.1|restrictionendonucleaseEco57I[Helicobacterpylori]/11586	-DISTFLTQLQS------GVGE-V----RFKK-GIIISS--S----HLTSEALKAIEQIR
gi|447079019|ref|WP_001156275.1|restrictionendonucleaseEco57I[Helicobacterpylori]_gi|308065028|gb|ADO06920.1|hypotheticalprotei	-DIATFLTQLLS------GVGE-V----RFKK-GIIIST--S----HLTSEALKAIEQIR
gi|447079028|ref|WP_001156284.1|restrictionendonucleaseEco57I[Helicobacterpylori]_gi|393076030|gb|EJB76784.1|hypotheticalprotei	-DISPFLTQLQS------GVGE-V----RFKK-GIIIST--S----NLTSEALKAIEQIR
gi|393034009|gb|EJB35070.1|hypotheticalproteinHPNQ4053_0631[HelicobacterpyloriNQ4053]/11514	-DISPFLTQLLI------GVGE-V----RFKK-GIIIST--S----NLTSEALKAIEQIR
gi|447079004|ref|WP_001156260.1|restrictionendonucleaseEco57I[Helicobacterpylori]_gi|384552418|gb|AFI07366.1|hypotheticalprotei	-DISPFLTQLQS------GVGE-V----KFKK-GIIIST--S----NLTSEALKAIEQIR
gi|746011306|ref|WP_039088215.1|restrictionendonucleaseEco57I[Helicobacterpylori]_gi|733488720|gb|KHL85979.1|restrictionendonuc	-DISPFLTQLQS------GVGE-V----RFKK-GIIIST--S----NLTSEALKAIEQIR
gi|531081162|gb|EQD92166.1|restrictionendonucleaseEco57I[HelicobacterpyloriPZ5080]/11509	-DISPFLTQLQS------GVGE-V----RFKK-GIIIST--S----NLTSEALKAIEQIR
gi|544760796|ref|WP_021187395.1|restrictionendonucleaseEco57I[Helicobacterpylori]_gi|531086508|gb|EQD96745.1|restrictionendonuc	-DISPFLTQLLS------GVGE-V----RFKK-GIIIST--S----NLTSEALKAIEQIR
gi|447079012|ref|WP_001156268.1|restrictionendonucleaseEco57I[Helicobacterpylori]_gi|393028199|gb|EJB29286.1|hypotheticalprotei	-DISPFLTQLQS------GVGE-V----RFKK-GIIIST--S----NLTSEALKAIEQIR
gi|746008458|ref|WP_039085555.1|restrictionendonucleaseEco57I[Helicobacterpylori]_gi|733480358|gb|KHL78026.1|restrictionendonuc	-DISTFLTQLQS------GVGE-V----RFKK-GIIIST--S----NLTSEALKAIEQIR
gi|447079011|ref|WP_001156267.1|restrictionendonucleaseEco57I[Helicobacterpylori]_gi|393038534|gb|EJB39568.1|hypotheticalprotei	-DISTFLAQLQS------GVGE-V----RFKK-GIIIST--S----NLTSEALKAIEQIR
gi|393037163|gb|EJB38199.1|hypotheticalproteinHPNQ4161_0422[HelicobacterpyloriNQ4161]/11556	-DISPFLTQLLS------GVGE-V----RFKK-GIIIST--S----NLTSEALKAIEQIR
gi|447079007|ref|WP_001156263.1|restrictionendonucleaseEco57I[Helicobacterpylori]_gi|393051817|gb|EJB52768.1|hypotheticalprotei	-DISPFLTQLLS------GVGE-V----KFKK-GIIIST--S----NLTSEALKAIEQIR
gi|487867094|ref|WP_001940560.1|helicase[Helicobacterpylori]_gi|459505657|gb|EMG98418.1|helicaseprotein[HelicobacterpyloriGAM21	-DISPFLTQLLSGHKLQNGVGE-I----RFKK-GIIIST--S----NLTSEALKAIEQIR
gi|487872094|ref|WP_001945560.1|helicase[Helicobacterpylori]_gi|459512104|gb|EMH04523.1|helicaseprotein[HelicobacterpyloriGAM24	-DISPFLTQLLSGHKLQNGVGE-V----RFKK-GIIIST--S----NLTSEALKAIEQIR
gi|487880228|ref|WP_001953694.1|helicase[Helicobacterpylori]_gi|459529131|gb|EMH20734.1|helicaseprotein[HelicobacterpyloriGAM26	-DISTFLYKLQSGHKLQNGVGE-I----KFKK-GIIIST--S----NLTSEALKAIEQIR
gi|487882593|ref|WP_001956059.1|helicase[Helicobacterpylori]_gi|459533462|gb|EMH24859.1|helicaseprotein[HelicobacterpyloriGAM26	-DISPFLTQL------LSGVGE-V----KFKK-GIIIST--S----NLTSEALKAIEQIR
gi|487845430|ref|WP_001918896.1|helicase[Helicobacterpylori]_gi|410713871|gb|EKQ71363.1|helicaseprotein[HelicobacterpyloriGAM10	-DISPFLTQL------LSGVGE-V----KFKK-GIIIST--S----NLTSNALKAIEQIR
gi|447079005|ref|WP_001156261.1|restrictionendonucleaseEco57I[Helicobacterpylori]_gi|393069830|gb|EJB70625.1|hypotheticalprotei	-DISPFLTQLLS------GVGE-V----KFKK-GIIIST--S----NLTSEALKAIEQIR
gi|459506808|gb|EMG99528.1|helicaseprotein[HelicobacterpyloriGAM121Aii]/11545	-DISPFLTQLLSGHKLQNGVGE-I----KFKK-GIIIST--S----NLTSEALKEIEKIR
gi|487825283|ref|WP_001898749.1|restrictionendonucleaseEco57I[Helicobacterpylori]_gi|393107526|gb|EJC08068.1|hypotheticalprotei	-DISPFLTQLLSGHKLQNGVGE-V----KFKK-GIIIST--S----HLTSEALKAIEQIR
gi|447079008|ref|WP_001156264.1|restrictionendonucleaseEco57I[Helicobacterpylori]_gi|393058636|gb|EJB59524.1|hypotheticalprotei	-DISPFLTQLLS------GVGE-V----KFKK-GIIIST--S----NLTSEAIKAIEQIR
gi|447079015|ref|WP_001156271.1|restrictionendonucleaseEco57I[Helicobacterpylori]_gi|307637357|gb|ADN79807.1|hypotheticalprotei	-DISPFLTQLLS------GVGE-V----KFKK-GIIIST--S----NLTSEALKAIEQIR
gi|487884861|ref|WP_001958327.1|helicase[Helicobacterpylori]_gi|459535535|gb|EMH26834.1|helicaseprotein[HelicobacterpyloriGAM26	-DIATFLTQLQS------GVGE-I----KFKK-GIIIST--S----NLTSEALKAIEQIR
gi|487886365|ref|WP_001959831.1|helicase[Helicobacterpylori]_gi|459538727|gb|EMH29861.1|helicaseprotein[HelicobacterpyloriGAM27	-DISPFLTQLLS------GVGE-V----KFKK-GIIIST--S----NLTSEALKAIEQIR
gi|545301637|ref|WP_021581412.1|helicase[Helicobacterpylori]_gi|535931889|gb|ERA61745.1|helicaseprotein[HelicobacterpyloriGAM11	-DISPFLTQLLS------GVGS-V----KFKK-GIIIST--S----NLTSEALKAIEQIR
gi|447079010|ref|WP_001156266.1|restrictionendonucleaseEco57I[Helicobacterpylori]_gi|393095477|gb|EJB96081.1|hypotheticalprotei	-DISPFLTQLLS------GVGG-V----KFKK-GIIIST--S----NLTSEAIKAIEQIR
gi|447079009|ref|WP_001156265.1|restrictionendonucleaseEco57I[Helicobacterpylori]_gi|393056294|gb|EJB57206.1|hypotheticalprotei	-DISPFLTQLLS------GVGE-V----KFKK-GIIIST--S----NLTSEALKAIEQIR
gi|471386985|gb|EMR58906.1|restrictionendonucleasefamilyprotein[HelicobacterpyloriHpH1]/11504	-DISPFLTQLLS------GVGE-V----KFKK-GIIIST--S----NLTSEALKAIEQIR
gi|487875679|ref|WP_001949145.1|helicase[Helicobacterpylori]_gi|459516928|gb|EMH09091.1|helicaseprotein[HelicobacterpyloriGAM25	-DISPFLTQLLS------GVGG-V----KFKK-GIIIST--S----NLTSEALKEIEKIR
gi|496130066|ref|WP_008854573.1|DNAdamageinducibleprotein[Commensalibacterintestini]_gi|353671689|gb|EHD13391.1|helicasedomain/	-EIDSFFTASGK-----------K----YFTH-RIIVST--T---DKWTKNAEEALEN--
gi|924898894|ref|WP_053556546.1|damageinducibleprotein[Sphingopyxissp.113P3]_gi|924523996|gb|ALC14732.1|damageinducibleprotei/1	-DIDGFLAASAR-----------A----PFAR-RMFVDT--T--GVNWTDHAETTFRD--
gi|780802950|gb|KJS08562.1|damageinducibleprotein[Hoefleasp.BRH_c9]/11633	-DIDSFFTESGK-----------K----PFTE-RLIIDT--T--NAAWSEHAEAALRD--
gi|944671432|gb|KQN22886.1|damageinducibleprotein[Sphingomonassp.Leaf32]/11415	-DIDSFFTASGK-----------A----PFTR-RLIIDT--T--EAAWSKNAEDALDG--
gi|944612953|gb|KQM64772.1|damageinducibleprotein[Sphingomonassp.Leaf16]/11726	-DIDSFFTASGK-----------A----PFTR-RLIIDT--T--EAAWSKNAEDALDG--
gi|764353851|ref|WP_044331468.1|damageinducibleprotein[Sphingomonashengshuiensis]_gi|761897338|gb|AJP71707.1|damageinduciblep/1	-DIDSFFTASGK-----------R----HFSR-RLIIDT--T--DAPWSANAEDALDN--
gi|945874472|gb|KQZ27973.1|damageinducibleprotein[Mesorhizobiumsp.Root552]/11651	-DIDSFFTASGK-----------R----QFGR-RLIIDT--T--DAPWSANAEAALDD--
gi|759408081|ref|WP_043132403.1|damageinducibleprotein[Paracoccussanguinis]_gi|694217086|gb|KGJ14281.1|damageinducibleprotein/1	-DIDSFISAASN-----------D----LFTR-LIIADT--T--QKEFGKNASGTLDK--
gi|654342671|ref|WP_027836116.1|damageinducibleprotein[Maritaleamyrionectae]/11632	-DLDTFISASSN-----------D----VFSR-LIIADT--T--RKDFGRNANETLNN--
gi|126717687|gb|EBA14409.1|hypotheticalproteinRSK20926_02032[Roseobactersp.SK20926]/11603	-DIDSFISAASN-----------D----LFTR-LVIADT--T--HKEFGRNAKETLDK--
gi|765301116|ref|WP_044628780.1|damageinducibleprotein[Halomonasmeridiana]_gi|764089679|gb|KJD19875.1|damageinducibleprotein[/1	-DIDSFISAASN-----------D----SFTR-LIIADT--T--QKDFGRNTKETLDK--
gi|518053006|ref|WP_019223214.1|DNAdamageinducibleprotein[Bartonellarattaustraliani]/11654	-DIDSFIAASGK-----------D----PFTR-RLLVDS--T--ETDWSENVDLTCQG--
gi|493637806|ref|WP_006589578.1|DNAdamageinducibleprotein[Bartonellabirtlesii]_gi|395410345|gb|EJF76900.1|hypotheticalproteinM/	-DIDSFIAASGK-----------D----IFKY-RLLVDT--T--EVELSDNVNAMIKG--
gi|501188603|ref|WP_012231622.1|DNAdamageinducibleprotein[Bartonellatribocorum]_gi|161017843|emb|CAK01401.1|helicase/methyltra/	-DIDSFIAASGK-----------D----IFKY-RLLVDS--T--EVELSDNVNAMIKG--
gi|651388616|ref|WP_026500615.1|damageinducibleprotein[Bartonellagrahamii]/11655	-DIDSFIAASGK-----------D----IFKY-RLLVDS--T--EVELSDNVNAMIKG--
gi|501188669|ref|WP_012231687.1|DNAdamageinducibleprotein[Bartonellatribocorum]_gi|161017922|emb|CAK01480.1|helicase/methyltra/	-DIDSFIAASGK-----------D----IFKY-RLLVDS--T--EVELSDNVNAMIKG--
gi|518048832|ref|WP_019219040.1|DNAdamageinducibleprotein[Bartonellaflorencae]/11655	-DIDSFIAASGK-----------D----IFKY-RLLVDT--T--EVELSDNVNAMIKG--

```
gi|518053617|ref|WP_019223825.1|DNAdamageinducibleprotein[Bartonellarattaustraliani]/11607        -DIDSFIAASGK-----------D----PFTR-RLLVDS--T--ETDWSENVDLTCQG--
gi|506336449|ref|WP_015856168.1|DNAdamageinducibleprotein[Bartonellagrahamii]_gi|240267492|gb|ACS51080.1|helicase/methyltransf/        -DIDSFIAASGK-----------D----IFKY-RLLVDT--T--EVELSDNVNAMIKG--
gi|494588832|ref|WP_007347427.1|DNAdamageinducibleprotein[Bartonellarattimassiliensis]_gi|395419053|gb|EJF85361.1|hypothetical/        -DIDSFIAASGK-----------K----IFTR-RILVDS--T--ESNWSDNANNTCDG--
gi|501188650|ref|WP_012231668.1|DNAdamageinducibleprotein[Bartonellatribocorum]_gi|161017902|emb|CAK01460.1|helicase/methyltra/        -DIDSFIAASGK-----------K----IFTR-RILVDS--T--ETDWSDNVELTCEG--
gi|501188624|ref|WP_012231643.1|DNAdamageinducibleprotein[Bartonellatribocorum]_gi|161017866|emb|CAK01424.1|helicase/methyltra/        -DIESFIAISGK-----------K----RFKY-RLLIDS--T--ERDLSENANTMIEG--
gi|518053856|ref|WP_019224064.1|DNAdamageinducibleprotein[Bartonellarattaustraliani]/11625        -DIESFIAISSK-----------K----RFKY-RLLIDS--T--EKDLSDNAHTMIEG--
gi|501188053|ref|WP_012231081.1|DNAdamageinducibleprotein[Bartonellatribocorum]_gi|161017434|emb|CAK00992.1|helicase/methyltra/        -DIESFIAISGK-----------K----RFKY-RLLIDS--T--ERDLSENANTMIEG--
gi|651388630|ref|WP_026500626.1|damageinducibleprotein[Bartonellagrahamii]/11657        -DIDSFIAASGK-----------D----IFKY-RLLVDS--T--EVELSDNVNAMIKG--
gi|518048855|ref|WP_019219063.1|DNAdamageinducibleprotein[Bartonellaflorencae]/11658        -DIDSFIAASGK-----------K----IFTR-RILIDS--T--ETDWSDNVELTCEG--
gi|490995101|ref|WP_004856829.1|DNAdamageinducibleprotein[Bartonelladoshiae]_gi|395413167|gb|EJF79645.1|hypotheticalproteinMCS/        -DIDSFIAASGK-----------K----IFTR-RILVDS--T--ETDWSDNVELTCEG--
gi|491002558|ref|WP_004864279.1|DNAdamageinducibleprotein[Bartonellavinsonii]_gi|395421392|gb|EJF87643.1|hypotheticalproteinME/        -DIDSFIAASGK-----------A----PFTR-RILIDS--T--EINWSNNADLTCKG--
gi|491003373|ref|WP_004865094.1|DNAdamageinducibleprotein[Bartonellavinsonii]_gi|395432864|gb|EJF98838.1|hypotheticalproteinME/        -DIDSFIAASGK-----------A----PFTR-RILIDS--T--ESNWSNNADLTCKG--
gi|491002426|ref|WP_004864148.1|DNAdamageinducibleprotein[Bartonellavinsonii]_gi|395421767|gb|EJF88003.1|hypotheticalproteinME/        -DIDSFIAASGK-----------K----IFTR-RILIDS--T--ESDWSDNAFHTCEG--
gi|491004024|ref|WP_004865745.1|DNAdamageinducibleprotein[Bartonellavinsonii]_gi|395432542|gb|EJF98522.1|hypotheticalproteinME/        -DIDSFIAASGK-----------K----IFTR-RLLIDS--T--ESDWSDNASHTCEG--
gi|648396442|ref|WP_026088193.1|damageinducibleprotein[Bartonellarattaustraliani]/11593        -DIDSFIAASGR-----------D----PFTR-RLLVDS--T--ETDWSENVDLTCQG--
gi|748190174|ref|WP_039761290.1|damageinducibleprotein[Bartonellaqueenslandensis]/11658        -DIDSFIAASGK-----------D----PFTR-RLLVDS--T--ETDWSENVDLTCQG--
gi|494817599|ref|WP_007553007.1|DNAdamageinducibleprotein[Bartonellasp.DB56]_gi|395411312|gb|EJF77837.1|hypotheticalproteinMC/1        -DIDSFIAASGK-----------A----PFTR-RILIDS--T--EGNWSNNADLTCEG--
gi|515763376|ref|WP_017195976.1|DNAdamageinducibleprotein[Bartonellabirtlesii]/11656        -DIDSFIAASGK-----------A----PFTR-RILIDS--T--EGNWSNNADLTCEG--
gi|494589420|ref|WP_007348014.1|DNAdamageinducibleprotein[Bartonellarattimassiliensis]_gi|395416278|gb|EJF82667.1|hypothetical/        -DIDSFIAASGK-----------K----IFTR-RILIDS--T--ETDWSNNVELTCEG--
gi|506336469|ref|WP_015856188.1|DNAdamageinducibleprotein[Bartonellagrahamii]_gi|240267512|gb|ACS51100.1|helicase/methyltransf/        -DIDSFIAASGK-----------K----IFTR-RILIDS--T--ETDWSDNVELTCEG--
gi|749308087|ref|WP_040132227.1|damageinducibleprotein[Bartonellatribocorum]_gi|605051878|emb|CDO49988.1|helicase/methyltransf/        -DIDSFIAASGK-----------K----IFTR-RILVDS--T--ETDWSDNVELTCEG--
gi|515763800|ref|WP_017196400.1|DNAdamageinducibleprotein[Bartonellabirtlesii]/11658        -DIDSFIAASGK-----------K----IFTR-RILVDS--T--ETDWSDNVELTCEG--
gi|492186994|ref|WP_005773380.1|DNAdamageinducibleprotein[Bartonellaelizabethae]_gi|395418361|gb|EJF84688.1|hypotheticalprotei/        -DIDSFIAASGK-----------K----IFTR-RILVDS--T--ESNWSDNANNTCDG--
gi|501188615|ref|WP_012231634.1|DNAdamageinducibleprotein[Bartonellatribocorum]_gi|161017856|emb|CAK01414.1|helicase/methyltra/        -DIDSFIAASGK-----------K----IFTR-RILVDS--T--ESNWSDNANNTCDG--
gi|501187470|ref|WP_012230499.1|DNAdamageinducibleprotein[Bartonellatribocorum]_gi|161017090|emb|CAK00648.1|helicase/methyltra/        -DIDSFIAASGK-----------K----IFTR-RILVDS--T--ESNWSDNANNTCDG--
gi|501188636|ref|WP_012231655.1|DNAdamageinducibleprotein[Bartonellatribocorum]_gi|161017879|emb|CAK01437.1|helicase/methyltra/        -DIDSFIAASGK-----------K----IFTR-RILVDS--T--ESNWSDNANNTCDG--
gi|930151941|emb|CTQ34818.1|UvsWhelicase[Jannaschiarubra]/11641        -DIDSFFTASGK-----------E----GFVR-RLIVDT--S--DGNWTDRAEKALQG--
gi|928977106|ref|WP_054001461.1|damageinducibleprotein[Shimiasp.SK013]_gi|927698852|gb|KPA22698.1|typeIrestrictionenzymeEcoKIs/        -DIQTFITASGK-----------A----PFSQ-RLFIDT--T--EVDWGENAEDAIRG--
gi|738872465|ref|WP_036760884.1|damageinducibleprotein[Leisingeradaeponensis]/11643        -DIDSFLAASSK-----------P----PFAR-RIVIDA--T--EVEWSSNAAEMLHG--
gi|518050881|ref|WP_019221089.1|DNAdamageinducibleprotein[Bartonellasenegalensis]/11652        -DIDSFLSASGK-----------K----PFKR-RVVIDS--T--INAWSDNAEAMIQG--
gi|493980596|ref|WP_006923523.1|DNAdamageinducibleprotein[Bartonellawashoensis]_gi|395413322|gb|EJF79796.1|hypotheticalprotein/        -DIDSFISASGK-----------A----PFKR-RVIIDS--T--EGAWSDNAETMIQG--
gi|493983106|ref|WP_006925984.1|DNAdamageinducibleprotein[Bartonellawashoensis]_gi|395418127|gb|EJF84455.1|hypotheticalprotein/        -DIDSFISASGK-----------A----PFKR-RVIIDS--T--ENAWSDNAETMIRG--
gi|499494671|ref|WP_011181311.1|DNAdamageinducibleprotein[Bartonellahenselae]_gi|49239008|emb|CAF28308.1|hypotheticalproteinBH/        -DIDSFISASGK-----------A----PFKR-RVIIDS--T--KNAWSENAETMIRG--
gi|651388426|ref|WP_026500441.1|damageinducibleprotein[Bartonellagrahamii]/11652        -DIDSFISASGK-----------A----PFKR-RVIIDS--T--ESAWSDNAETMIRG--
gi|506337280|ref|WP_015856999.1|DNAdamageinducibleprotein[Bartonellagrahamii]_gi|240268402|gb|ACS51990.1|helicase/methyltransf/        -DIDSFISASGK-----------A----PFKR-RIIIDS--T--ESAWSDNAETMIRG--
gi|494817535|ref|WP_007552943.1|DNAdamageinducibleprotein[Bartonellasp.DB56]_gi|395412002|gb|EJF78518.1|hypotheticalproteinMC/1        -DIDSFISASGK-----------E----PFKR-RVIIDS--T--ESAWSDNAETMIRG--
gi|493638099|ref|WP_006589864.1|DNAdamageinducibleprotein[Bartonellabirtlesii]_gi|395409809|gb|EJF76394.1|hypotheticalproteinM/        -DIDSFISASGK-----------E----PFKR-RVIIDS--T--QNAWSDNAETMIRG--
gi|748189589|ref|WP_039760707.1|damageinducibleprotein[Bartonellaqueenslandensis]/11651        -DIDSFISASGK-----------E----PFKR-RVIIDS--T--INAWTDNAETMIRG--
gi|501189483|ref|WP_012232501.1|DNAdamageinducibleprotein[Bartonellatribocorum]_gi|161018902|emb|CAK02460.1|helicase/methyltra/        -DIDSFISASGK-----------E----PFKR-RVIIDS--T--IKAWTDNAETMIQG--
gi|518049181|ref|WP_019219389.1|DNAdamageinducibleprotein[Bartonellaflorencae]/11652        -DIDSFISASGK-----------A----PFKR-RVVIDS--T--INAWSDNAETMIRG--
gi|492193255|ref|WP_005774528.1|DNAdamageinducibleprotein[Bartonellaelizabethae]_gi|395416403|gb|EJF82779.1|hypotheticalprotei/        -DIDSFISASGK-----------E----PFKQ-RVIIDS--T--INAWTDNAETMIRG--
gi|498387325|ref|WP_010701288.1|helicase/methyltransferase[Bartonellabovis]_gi|478418724|gb|ENN92426.1|helicase/methyltransfera        -DIDSFISASGK-----------D----LFKR-RVVIDS--T--EKPWSDNAETMKEG--
gi|319408697|emb|CBI82352.1|conservedhypotheticalprotein[BartonellaschoenbuchensisR1]/11661        -DIDSFISASGK-----------S----PFKR-RVVIDS--T--EKPWSDNAETMKEG--
gi|494741726|ref|WP_007477134.1|DNAdamageinducibleprotein[Bartonellamelophagi]_gi|395423925|gb|EJF90113.1|hypotheticalproteinM/        -DIDSFISASGK-----------S----PFKR-RVVIDS--T--EKPWSDNAETMKEG--
gi|737261405|ref|WP_035245349.1|damageinducibleprotein[Alcanivoraxjadensis]_gi|686989709|gb|KGD62556.1|hypotheticalproteinT9A_/        -DLDGFFTASNR-----------K----SFTR-RIIVDT--T--ETELGTNVQALLDDAE
gi|916687564|ref|WP_051294655.1|damageinducibleprotein[Gemmobacternectariphilus]/11641        -DIDSFISASGK-----------E----PFKR-RIIVDS--T--ERPWSENAETMIRG--
gi|740294127|ref|WP_038132495.1|damageinducibleprotein[Thioclavasp.DT234]_gi|660524826|gb|KEO54689.1|hypotheticalproteinDT23_/1        -DIDSFISASGK-----------E----PFRR-RLVIDS--T--TVPWGSNAEEIISG--
gi|568331062|ref|WP_024099615.1|helicase[Phaeobactergallaeciensis]_gi|568232121|gb|AHD12007.1|putativehelicase[Phaeobactergalla        -DIDSFISASGK-----------E----PFKR-RVIIDT--T--ERTWSENAETMIRG--
gi|518810459|ref|WP_019966413.1|DNAdamageinducibleprotein[Pannonibacterphragmitetus]/11635        -HIDSFISASGK-----------E----PFRR-RVVLDT--T--EKEWGANAEEMIRD--
gi|495305760|ref|WP_008030510.1|DNAdamageinducibleprotein[Rhodobactersp.SW2]_gi|259021698|gb|EEW25000.1|typeIIIrestrictionprot/        -HIDSFISASGK-----------E----PFRR-RVVLDT--T--EKEWGTNAEEMIRD--
gi|506403303|ref|WP_015923022.1|DNAdamageinducibleprotein[Rhodobactersphaeroides]_gi|221162951|gb|ACM03922.1|HypotheticalProte/        -HIDSFISASGK-----------E----PFRR-RIVLDT--T--EHEWSANAEEMIRG--
gi|738818386|ref|WP_036708790.1|damageinducibleprotein[Paracoccussanguinis]_gi|694226786|gb|KGJ22541.1|damageinducibleprotein/1        -HIDSFISASGK-----------E----PFRR-RIVLDT--T--EHEWSANAEEMIRG--
gi|746096057|ref|WP_039162453.1|damageinducibleprotein[Gallibacteriumanatis]_gi|703617775|gb|KGQ63868.1|damageinducibleprotei/1        -DIDSFFTASGK-----------K----IFSH-RLIVTT--T---DHWSENATESLND--
gi|737716785|ref|WP_035685631.1|damageinducibleprotein[Avibacteriumparagallinarum]/11643        -DIDSFFTASGK-----------K----IFSH-RLIVTT--T---DHWSENAAESLND--
gi|540072553|gb|ERF78672.1|hypotheticalproteinN561_04930[Gallibacteriumanatis12656/12]/11651        -DIDSFFTASGK-----------K----IFSH-RLIVTT--T---DHWSENAAASLND--
gi|917509519|ref|WP_052115936.1|damageinducibleprotein[Gallibacteriumanatis]_gi|702414161|gb|KGQ42233.1|damageinducibleprotei/1        -DIDSFFTASGK-----------K----IFSH-RVIVTT--T---DHWSENAAASLND--
gi|746082942|ref|WP_039150268.1|damageinducibleprotein[Gallibacteriumanatis]_gi|703617078|gb|KGQ63194.1|damageinducibleprotei/1        -DIDSFFTASGK-----------K----IFSH-RLIVTT--T---DHWSENAAASLND--
gi|822522885|ref|WP_046952840.1|damageinducibleprotein[Haemophilushaemolyticus]_gi|821501762|gb|KKZ59267.1|damageinduciblepro/1        -DIDSFFTASGK-----------S----YFQL-RIIVTT--T---DHWTENAYDSLSH--
gi|404381014|gb|EJZ77501.1|putativehelicase[Pasteurellamultocidasubsp.gallicidaP1059]/11681        -DIDSFFTASGK-----------S----YFTH-RLIVAT--T---DHWTNNAYESLSQ--
gi|298281660|gb|EFI23149.1|helicaseCterminaldomainprotein[Neisseriasp.oraltaxon014str.F0314]/11493        -DIDSFFTASGK-----------T----HFSN-RIIVST--T---DKWSKNAEDALEG--
gi|348013055|gb|EGY51980.1|helicasedomainprotein[Neisseriashayeganii871]/11481        -DIDSFFTASGK-----------S----HFSY-RIIVTT--T---DKWSQNARAALPG--
gi|496407140|ref|WP_009116004.1|DNAdamageinducibleprotein[Neisseriawadsworthii]_gi|349794756|gb|EGZ48566.1|helicasedomainprote/        -DIDSFFTASGK-----------H----YFNH-RYIVTT--T---NKWSSNARAALID--
gi|517216441|ref|WP_018405259.1|DNAdamageinducibleprotein[Marinobacterlipolyticus]/11656        -DIDSFFTASGK-----------S----FFTY-RIIVTT--T---DQWTANAQNSLES--
gi|738230264|ref|WP_036185691.1|damageinducibleprotein[Marinobactersp.ELB17]/11671        -DIDSFFTASGK-----------S----FFTY-RIIVTT--T---DRWTANAQDALGS--
gi|447197741|ref|WP_001274997.1|DNAdamageinducibleprotein[Acinetobacterbaumannii]_gi|408512324|gb|EKK13969.1|typeIIIrestrictio/        -DIDSFLGGSGK-----------S----YFAY-RYIVAS--T---DDWTDNAKSSLVD--
gi|820775897|ref|WP_046739909.1|damageinducibleprotein[Acinetobactersp.AG1]_gi|819909758|gb|KKW75938.1|damageinducibleprotein/1        -DIDSFLGGSGK-----------S----YFTY-RYIVAS--T---DDWTDNAKSSLVD--
gi|584595163|ref|WP_024160846.1|helicase[Acinetobacterpittii]_gi|576702530|emb|CDI28049.1|helicasedomaincontainingprotein[Acin/        -DIDSFLGGSGK-----------S----YFAY-RYIVAS--T---DDWTDNAKSSLVD--
```

```
gi|514963042|ref|WP_016651366.1|hypotheticalprotein[Acinetobactersp.NIPH2036]_gi|514162665|gb|EPG41391.1|hypotheticalproteinF90    -DIDSFLGGSGK-----------S----YFAY-RYIVAS--T---DDWTDNAKSSLVD--
gi|515965564|ref|WP_017396147.1|DNAdamageinducibleprotein[Acinetobacterhaemolyticus]/11667    -DIDSFLGGSGK-----------S----YFAY-RYIVAS--T---DDWTDNAKSSLVD--
gi|491346810|ref|WP_005204747.1|MULTISPECIES:hypotheticalprotein[Acinetobacter]_gi|480335823|gb|ENX56033.1|hypotheticalproteinF    -DIDSFLGGSGK-----------S----YFSY-RYIVAS--T---DDWTDNAKSSLLD--
gi|693044472|ref|WP_032215268.1|damageinducibleprotein[Escherichiacoli]_gi|607725731|gb|EZE08230.1|damageinducibleprotein[Esc/1    -DIDSFLAASDK-----------T----YFTL-RYIVAS--T---DNWTEEAKEMLRD--
gi|742382177|ref|WP_038861304.1|damageinducibleprotein[Cronobactermuytjensii]_gi|924333747|gb|ALB69766.1|damageinducibleprote/1    -DIDSFLAASDK-----------T----YFTL-RYIVAS--T---DNWTEEAKDMLRD--
gi|915912804|ref|WP_050927035.1|damageinducibleprotein[Yersiniaenterocolitica]_gi|899805802|emb|CQH32042.1|typeIrestrictionenz/    -DIDSFLAASDK-----------T----YFTL-RYIVAS--T---DNWTEEAKNMLRD--
gi|921111327|gb|KON62781.1|typeIrestrictionenzymeEcoKIsubunitR[Komagataeibactereuropaeus]/11492    -DIDSFFTASGK-----------K----HFAR-RIIVAT--T---NHWTDNAEASLAD--
gi|582024278|gb|AHI27615.1|DNAhelicaserestrictionenzymeTypeIIIRsubunit[KomagataeibacterxylinusE25]/11636    -DIDSFFTASGK-----------K----HFAR-RIIVAT--T---NHWTDNAEASLAD--
gi|347662673|dbj|BAK86128.1|DNAhelicaserestrictionenzymeTypeIIIRsubunit[KomagataeibactermedellinensisNBRC3288]/11737    -DIDSFFTASGK-----------K----HFAR-RIIVAT--T---NHWTDNAEASLAD--
gi|917445053|ref|WP_052051471.1|damageinducibleprotein[Acetobactertropicalis]/11429    -DIDTFFSASGK-----------S----WFSH-RVIVAT--T---NYWNSNAEDTLAN--
gi|453329445|dbj|GAC88472.1|DNAhelicaserestrictionenzymetypeIIIRsubunit[GluconobacterthailandicusNBRC3255]_gi|530287981|dbj|GAD    -DIDTFFSASGK-----------S----WFSH-RVIVAT--T---NLWNSNAEATLAD--
gi|889770922|ref|WP_048844278.1|damageinducibleprotein[Gluconobacterfrateurii]/11419    -DIDTFFSASGK-----------S----WFSH-RVIVAT--T---NLWNSNAEATLAD--
gi|504886208|ref|WP_015073310.1|hypotheticalprotein[Gluconobacteroxydans]_gi|411027954|gb|AFW01209.1|hypotheticalproteinB932_16    -DIDTFFSASGK-----------S----WFSH-RVIVAT--T---NLWNSNAEATLAD--
gi|480040211|gb|ENV79751.1|hypotheticalproteinF942_01641[AcinetobacterursingiiANC3649]/11432    -DIDSFFTASGK-----------R----PFTH-RMIVST--T---NHWSKHAKDALKG--
gi|822498222|ref|WP_046936504.1|damageinducibleprotein[Xanthomonasgardneri]_gi|821751224|gb|KLB07223.1|damageinducibleprotein/1    -DIDSFFTASGR-----------K----PFAR-RIIFST--T---LDWSEHAENALVD--
gi|654059559|ref|WP_027705556.1|damageinducibleprotein[Zymobacterpalmae]/11635    -EIDSFFTASGR-----------K----PFTQ-RLIVST--T---LHWSEHADSALND--
gi|643535620|ref|WP_025224315.1|damageinducibleprotein[Methylophilaceaebacterium11]_gi|577704400|gb|EUJ10546.1|putativehelicas/    -DIDSFFTASGK-----------K----IFTH-RIIVST--T---NNWSANAEDALQN--
MBA11I/11635    -DIDSFFTASGK-----------K----IFTH-RIIVST--T---NNWSANAEDALQN--
gi|655355857|ref|WP_028762287.1|damageinducibleprotein[Shewanellacolwelliana]/11632    -DIDSFFTASGK-----------K----PFTH-RLIVTT--T---TNWSEHAENAMAD--
gi|753204236|ref|WP_041510579.1|damageinducibleprotein[Shewanellasp.cp20]_gi|752457765|gb|KIO36326.1|damageinducibleprotein[S/1    -DIDSFFTASGQ-----------A----PFSH-RIIVST--T---NNWSSNAEAALIG--
KPNNIH30III/11619    -DIDSFFTASGQ-----------A----PFSH-RVIIST--T---NNWSDNAEAALIG--
gi|749295994|ref|WP_040120352.1|damageinducibleprotein[Klebsiellapneumoniae]_gi|723249300|gb|AIX81926.1|damageinducibleprotei/1    -DIDSFFTASGQ-----------A----PFSH-RVIIST--T---NNWSDNAEAALIG--
gi|641639739|emb|CDQ54010.1|unnamedproteinproduct[Klebsiellapneumoniae]/11641    -DIDSFFTASGQ-----------A----PFGH-RVIIST--T---NNWSDNAEAALIG--
gi|490797922|ref|WP_004660060.1|hypotheticalprotein[Acinetobactersp.NIPH236]_gi|479876779|gb|ENU27973.1|hypotheticalproteinF992    -EIDSFLSAASR-----------T----YFKR-RYIVST--T---HAWSDNALATLEN--
gi|916750926|ref|WP_051357982.1|damageinducibleprotein[Acinetobactertjernbergiae]/11453    -EIDSFLSAASK-----------T----YFKR-RIIVST--T---HGWSDNALTTLEN--
gi|691098662|ref|WP_032025060.1|damageinducibleprotein[Acinetobacterbaumannii]_gi|593567521|gb|EXR50735.1|restrictionendonucle/    -EIDSFLAAASK-----------T----YFKR-RIIVST--T---HDWSDNALATLEN--
gi|736669405|ref|WP_034676049.1|damageinducibleprotein[Acinetobacterbaumannii]/11643    -EIDSFLSAASK-----------T----YFKR-RIIVST--T---HDWSDNALATLEN--
gi|491368928|ref|WP_005226826.1|hypotheticalprotein[Acinetobactersp.NIPH1847]_gi|480173259|gb|ENX09797.1|hypotheticalproteinF89    -EIDSFISAASK-----------T----YFTR-RIIVST--T---HAWSDNALATLEN--
gi|796853993|ref|WP_045795953.1|damageinducibleprotein[Acinetobacterindicus]_gi|787929761|gb|KJV44219.1|damageinducibleprotei/1    -EINSFLAAASK-----------T----YFER-RIIVST--T---HKWSDNALQTLEN--
gi|295797463|gb|ADG32253.1|typeIIIrestrictionproteinressubunit[ThiomonasintermediaK12]/11676    DGIDSFLSAMGK-----------K----PYTD-GLLFLT--S---YTASQHVNALVQQ--
gi|499871179|ref|WP_011551913.1|DNAdamageinducibleprotein[Myxococcusxanthus]_gi|108467544|gb|ABF92729.1|putativerestriction/mo/    -DIDSFFTASGK-----------K----PFTH-RIIVST--T---DLWSEHAEEALRD--
gi|495313852|ref|WP_008038600.1|DNAdamageinducibleprotein[Bartonellatamiae]_gi|395424181|gb|EJF90368.1|hypotheticalproteinME5_/    -DIDSFMSASGQ-----------R----PFVN-RFIVCT--T---NHWGDNAEAMLDN--
gi|501453335|ref|WP_012476784.1|DNAdamageinducibleprotein[Leptospirabiflexa]_gi|167777824|gb|ABZ96123.1|Helicase[Leptospirabif/    -DIDSFFTASGQ-----------K----PFVK-RIIVST--T---NHWSEHAENALLN--
gi|738104613|ref|WP_036063193.1|damageinducibleprotein[Leptospiranoguchii]/11652    -DIDSFFTASGK-----------K----PFTH-RIIVAT--T---NNWSEHAEDSLRG--
gi|410001039|gb|EKO51661.1|typeIIIrestrictionenzyme,ressubunit[Leptospirakirschneristr.200802841]/11651    -DIDSFFTASGK-----------M----PFTH-RIIVAT--T---NNWSEHAEDALRG--
gi|490901851|ref|WP_004763774.1|typeIIIrestrictionenzyme,ressubunit[Leptospirakirschneri]_gi|464209249|gb|EMN24014.1|typeIIIres    -DIDSFFTASGK-----------M----PFTH-RIIVAT--T---NNWSEHAEDALRG--
gi|464398131|gb|EMO65902.1|typeIIIrestrictionenzyme,ressubunit[Leptospirakirschneristr.200803703]/11648    -DIDSFFTASGK-----------M----PFTH-RIIVAT--T---NNWSEHAEDALRG--
gi|945029024|gb|KQQ84900.1|damageinducibleprotein[Xanthomonassp.Leaf131]/11423    EDIDSFFTASGR-----------K----PFAH-RVIVST--T---NHWSEHAENALVD--
gi|896511314|ref|WP_049418183.1|damageinducibleprotein[Stenotrophomonasmaltophilia]/11639    KDIDSFFTASGR-----------K----PFAR-RIIVAT--T---DHWSDNAENALVD--
gi|896513082|ref|WP_049419616.1|damageinducibleprotein[Stenotrophomonasmaltophilia]/11638    SEIDSFFTASGR-----------K----PFAH-RVIVST--T---NQWSDNAENALID--
gi|896580616|ref|WP_049469425.1|damageinducibleprotein[Stenotrophomonasmaltophilia]/11641    KDIDSFFTASGR-----------K----PFAH-RVIVSS--T---DHWSDNAENALID--
gi|648289350|ref|WP_026070499.1|damageinducibleprotein[Stenotrophomonasmaltophilia]/11639    KDIDSFFTASGR-----------K----PFTH-RVIVSS--T---NHWSDNAENALID--
gi|656272786|ref|WP_029218442.1|damageinducibleprotein[Xanthomonascassavae]/11639    KDIDSFFTASGR-----------K----PFTH-RVIVSS--T---NHWSDNAENALVD--
gi|406976236|gb|EKD98747.1|hypotheticalproteinACD_23C00290G0002[unculturedbacterium]/11679    AGIDSFLSAMGK-----------A----PFTN-GLLVLT--T---FKATEHVINLVQG--
gi|828145737|ref|WP_047238120.1|damageinducibleprotein[Chromobacteriumsubtsugae]/11645    SDIDSFFTASGK-----------Q----PFSR-RLIVTT--T---NNWGPNAEDALIG--
gi|654311174|ref|WP_027824327.1|damageinducibleprotein[Laribacterhongkongensis]/11641    ADIDSFFTASGK-----------Q----PFSR-RLIVTT--T---NNWGPNAEDALIG--
gi|757242173|gb|KIR15516.1|typeIrestrictionenzymeEcoKIsubunitR[Pseudomonasfluorescens]/11778    SDIDSFFTASGK-----------K----PFTH-RIIVTT--T---NNWNEHAADALQN--
gi|653605161|ref|WP_027616682.1|damageinducibleprotein[Pseudomonassp.URHB0015]/11639    GDIDSFFTASGK-----------K----PFTH-RVIVTT--T---NNWNEHAADALQN--
gi|520868025|ref|WP_020308060.1|helicase[Pseudomonassyringae]_gi|519159034|gb|EPM49935.1|helicasedomaincontainingprotein[Pseud/    SDIDSFFTASGK-----------K----PFTH-RVIVTT--T---NNWNEHAADALQN--
gi|940156567|gb|KPX71668.1|Helicasedomaincontainingprotein[Pseudomonasamygdalipv.lachrymans]/11677    SDVNSFLAASGQ-----------K----PFEH-RVIVTT--T---NNWNEHAADALQN--
gi|505460508|ref|WP_015647610.1|helicase[Pseudomonassp.GLE121]_gi|499110212|ref|YP_007974374.1|helicasedomaincontainingprotein/    KDIDSFFTASGK-----------K----PFTH-RIIVAT--T---NDWSEHANDALLD--
gi|940235551|gb|KPY45311.1|hypotheticalproteinALO48_200154[Pseudomonassyringaepv.rhaphiolepidis]/11631    KDIDSFFTASGK-----------K----PFTH-RIIVAT--T---NEWSEHANDALLD--
gi|520880808|ref|WP_020312235.1|helicase[Pseudomonassyringae]_gi|519172158|gb|EPM62027.1|helicasedomaincontainingprotein[Pseud/    KDIDSFFTASGK-----------K----PFSH-RVIVTT--T---NKWSEHANDALQD--
gi|829046479|ref|WP_047282259.1|damageinducibleprotein[Pseudomonasfluorescens]/11630    KDIDSFFTASGK-----------A----PFSH-RIIVTT--T---NNWSGHAEDALQG--
gi|499415493|ref|WP_011102960.1|DNAdamageinducibleprotein[Pseudomonassyringaegroupgenomosp.3]_gi|28867279|ref|NP_789898.1|heli/    KDIDSFFTASGK-----------A----PFTH-RIIVAT--T---NNWSEHAEDALQG--
gi|730238299|ref|WP_033942897.1|damageinducibleprotein[Pseudomonasaeruginosa]/11629    KDIDSFFTASGK-----------A----PFSH-RIIVTT--T---NNWSEHAEDALQG--
gi|495310125|ref|WP_008034873.1|DNAdamageinducibleprotein[Pseudomonassp.GM60]_gi|398186685|gb|EJM74052.1|putativehelicase[Pseu/    KDIDSFFTASGK-----------T----PFTH-RIIVAT--T---NNWSEHAEDALQD--
gi|779992653|ref|WP_045428690.1|damageinducibleprotein[Pseudomonassp.MT1]_gi|699967872|dbj|BAP80334.1|DNAhelicaserestrictione/1    KDIDSFFTASGK-----------A----PFSH-RIIVTT--T---NNWSEHAENALLG--
gi|786037289|ref|WP_045733274.1|damageinducibleprotein[Pseudomonaspseudoalcaligenes]_gi|782987904|gb|KJU79152.1|damageinducib/1    KDIDSFFTASGK-----------A----PFSH-RVIVTT--T---NNWSEHAEDALQG--
gi|749666366|ref|WP_040265445.1|damageinducibleprotein[Pseudomonasrhodesiae]/11632    KDIDSFFTASGK-----------A----PFSH-RVIVAT--T---NNWSEHAEDALQG--
gi|734902433|ref|WP_034133698.1|damageinducibleprotein[Pseudomonasfluorescens]/11635    KDIDSFFTASGK-----------A----PFSH-RVIVAT--T---NNWSEHAEDALQG--
gi|490551031|ref|WP_004416146.1|typeIIrestrictionmodificationsystemrestriction/helicasesubunit[Pseudomonassyringae]_gi|4432823/    KDIDSFFTASGK-----------A----PFSH-RVIVAT--T---NNWSEHAEDALQG--
gi|489199911|ref|WP_003109152.1|DNAdamageinducibleprotein[Pseudomonasaeruginosa]_gi|552648708|gb|ERY63184.1|hypotheticalprotei/    KDIDSFFTASGK-----------A----PFSH-RVIVTT--T---NNWSEHAEDALQG--
gi|730641237|ref|WP_034061328.1|damageinducibleprotein[Pseudomonasaeruginosa]/11636    KDIDSFFTASGK-----------A----PFSH-RIIVAT--T---NNWSEHAEDALQG--
gi|751304980|ref|WP_041013386.1|damageinducibleprotein[Pseudomonasxanthomarina]_gi|703407611|emb|CEG51014.1|Helicasedomaincon/1    KDIDSFFTASGK-----------A----PFSH-RVIVTT--T---NNWSEHAEDALQG--
gi|939335207|ref|WP_054881520.1|damageinducibleprotein[Pseudomonassp.NBRC111128]/11632    KDIDSFFTASGK-----------A----PFSH-RIIVAT--T---NNWSEHAEDALQG--
gi|757693555|ref|WP_042923792.1|damageinducibleprotein[Pseudomonaschloritidismutans]_gi|780910610|gb|KJS78302.1|damageinducib/1    KDIDSFFTASGK-----------A----PFSH-RIIVTT--T---NNWSEHAEDALQG--
```

```
gi|665953944|ref|WP_031319989.1|damageinducibleprotein[Pseudomonastolaasii]/11626    KDIDSFFTASGK-----------A----QFSH-RVIVTT--T---NNWSEHAEDALQG--
gi|771600573|ref|WP_045213746.1|damageinducibleprotein[Pseudomonassp.21]_gi|770081123|gb|KJJ98634.1|damageinducibleprotein[Ps/1    KDIDSFFTASGK-----------A----PFSH-RIIVAT--T---NNWSEHAEDALQG--
gi|740871396|ref|WP_038656644.1|damageinducibleprotein[Pseudomonasstutzeri]_gi|627772214|gb|AHY41437.1|damageinducibleprotein/1    KDIDSFFTASGK-----------A----PFSH-RVIVTT--T---NNWSEHAEDALQG--
gi|946242205|gb|KRC93782.1|damageinducibleprotein[Pseudomonassp.Root9]/11634    KDIDSFFTASGK-----------T----PFSH-RVIVTT--T---NNWSEHAEDALQG--
gi|553777322|ref|WP_023109107.1|MULTISPECIES:hypotheticalprotein[Pseudomonas]_gi|552638674|gb|ERY53184.1|hypotheticalproteinQ05    KDIDSFFTASGK-----------A----PFSH-RFIVAT--T---NNWSEHAEDALQG--
gi|896383370|ref|WP_049324618.1|damageinducibleprotein[Pseudomonasstutzeri]/11627    KDIDSFFTASGK-----------A----PFSH-RVIVTT--T---NNWSEHAEDALQG--
gi|696158510|gb|AIT41787.1|putativehelicase[Pseudomonassp.LM8]/11630    KDIDSFFTASGK-----------A----PFSH-RIIVTT--T---NNWSEHAEDALQG--
gi|504634892|ref|WP_014821994.1|DNAdamageinducibleprotein[Pseudomonasstutzeri]_gi|390985249|gb|AFM35242.1|helicasedomainconta/1    KDIDSFFTASGK-----------A----PFSH-RIIVTT--T---NNWSEHAEDALQG--
gi|544783974|ref|WP_021206729.1|DNAdamageinducibleprotein[Pseudomonasstutzeri]_gi|533224779|gb|EQM79688.1|DNAdamageinduciblep/1    KDIDSFFTASGK-----------A----PFSH-RIIVTT--T---NNWSEHAEDALQG--
gi|504567385|ref|WP_014754487.1|MULTISPECIES:DNAdamageinducibleprotein[Pseudomonas]_gi|388559493|gb|AFK68634.1|helicasedomain/1    KDIDSFFTASGK-----------T----PFSH-RVIVTT--T---NNWSEHAEDALQG--
gi|654441469|ref|WP_027912052.1|damageinducibleprotein[Pseudomonassp.URIL14HWK12:I7]/11630    KDIDSFFTASGK-----------A----PFSH-RVIVAT--T---NNWSEHAEDALQG--
gi|941470066|ref|WP_055127894.1|damageinducibleprotein[Pseudomonasmediterranea]/11631    KDIDSFFTASGK-----------A----PFSH-RVIVAT--T---NNWSEHAEDALQG--
gi|557815599|ref|WP_023446184.1|DNAdamageinducibleprotein[Pseudomonaschloritidismutans]_gi|557364081|gb|ESQ98190.1|DNAdamagei/1    KDIDSFFTASGK-----------T----PFSH-RVIVTT--T---NNWSEHAEDALQG--
gi|727814791|ref|WP_033898247.1|damageinducibleprotein[Pseudomonassp.FH1]_gi|567648005|gb|ETK23229.1|helicasedomaincontaining/1    KDIDSFFTASGK-----------A----PFSH-RVIVAT--T---NNWSEHAEDALQG--
gi|503278702|ref|WP_013513363.1|DNAdamageinducibleprotein[Desulfovibrioaespoeensis]_gi|316942375|gb|ADU61426.1|typeIIIrestrict/    SDIDSFFTASGK-----------K----YFTN-RIIVCT--T---NKWSKPAEDSLSD--
gi|505228796|ref|WP_015415898.1|typeIIIrestrictionendonucleasesubunitR[Desulfovibriopiezophilus]_gi|451896976|emb|CCH49855.1|Ty    SDIDSFFTASGK-----------K----YFTN-RIIVCT--T---NKWSGPAEKSLSD--
gi|517732543|ref|WP_018902751.1|hypotheticalprotein[Variovoraxparadoxus]/11675    SDIDSFFTASGK-----------K----PFAR-RLIIST--T---NHWSEHAEDALRD--
gi|550909475|ref|WP_022662570.1|DNAdamageinducibleprotein[Desulfovibriolongus]/11637    RDIDSFFTASGK-----------K----IFSH-RIIVST--T---NHWSEHAEEALRD--
gi|945494542|gb|KQV50024.1|damageinducibleprotein[Massiliasp.Root335]/11647    SDIDSFFTASGK-----------K----PFAH-RIIVST--T---NHWSTHAEEALRD--
gi|334196917|gb|AEG70102.1|helicasedomainprotein[RalstoniasolanacearumPo82]/11663    SDIDSFFTASGK-----------K----PFTR-RIIVAT--T---NHWSDHAEDALYD--
gi|656006746|ref|WP_029048170.1|damageinducibleprotein[Cupriavidussp.amp6]/11661    GDIDSFFTASGK-----------K----PFSR-RIIVAT--T---NHWSDHAEDALYD--
BPS2196ORF3054P/11659    SDIDSFFTASGK-----------K----PFTR-RIIVAT--T---NHWSEHAEDALLD--
gi|740932312|ref|WP_038717332.1|damageinducibleprotein[Burkholderiapseudomallei]_gi|760211882|gb|KIX58314.1|damageinduciblepr/1    SDIDSFFTASGK-----------K----PFTR-RIIVAT--T---NHWSEHAEDALLD--
gi|755810813|ref|WP_042583861.1|damageinducibleprotein[Burkholderiasp.MSHR3999]_gi|755174952|gb|KIP19790.1|DEAD/DEAHboxhelicas/    SDIDSFFTASGK-----------K----PFMR-RIIVAT--T---NHWSEHAEDALLN--
gi|484586570|gb|AGK48195.1|DEAD/DEAHboxhelicasefamilyprotein[BurkholderiathailandensisMSMB121]/11646    SDIDSFFTASGK-----------K----PFTR-RIIVAT--T---NHWSEHAEDALLN--
gi|737582935|ref|WP_035553941.1|damageinducibleprotein[Burkholderiasp.9120]/11658    GDIDSFFTASGK-----------K----PFTR-RIIVAT--T---NHWSEHAEDALLD--
gi|740989872|ref|WP_038773225.1|damageinducibleprotein[Burkholderiapseudomallei]_gi|705645291|gb|KGS70691.1|DEAD/DEAHboxhelica/    SDIDSFFTASGK-----------K----PFTR-RIIVAT--T---NHWSEHAEDALLD--
gi|654912525|ref|WP_028363424.1|damageinducibleprotein[Burkholderiasp.JPY366]/11659    SDIDSFFTASGK-----------K----PFTR-RIIVAT--T---NHWSEHAEDALLD--
gi|740955879|ref|WP_038740512.1|damageinducibleprotein[Burkholderiapseudomallei]_gi|714684932|gb|KGW74438.1|DEAD/DEAHboxhelica/    SDIDSFFTASGK-----------K----PFTR-RIIVAT--T---NHWSEHAEDALLD--
gi|919203714|ref|WP_052759215.1|damageinducibleprotein[Xanthomonasaxonopodis]/11388    -DIDSFFTASG---------------KLPFAS-RLIVST--T--D-KWSKHAEDALEA--
gi|522152381|ref|WP_020663589.1|hypotheticalprotein[Amycolatopsisbenzoatilytica]/11605    -DIDSFFTASG---------------KAPFTS-RIIIST--T--D-KWGQHAEDALDD--
gi|663196013|ref|WP_030233899.1|damageinducibleprotein[Streptomycessp.NRRLS455]/11641    -DIDSFFTASG---------------QGGFAR-RLIIST--T--D-KWGPNAEAALHD--
gi|750429553|ref|WP_040711444.1|damageinducibleprotein[Nocardiopsispotens]/11622    -DIDSFFTASG---------------KGGFTR-RMIIST--T--D-QWSEHAEAALED--
gi|518656139|ref|WP_019819475.1|hypotheticalprotein[Saccharomonosporasaliphila]/11623    -DIDSFFTASG---------------KGDFAR-RMIIST--T--D-KWSVHAESALTD--
gi|759722933|ref|WP_043433758.1|damageinducibleprotein[Streptomycespluripotens]_gi|733404978|gb|KHK94361.1|damageinduciblepro/1    -DIDSFFTASG---------------KGPFTR-RVIIST--T--D-KWSDNAEAALDG--
gi|663424167|ref|WP_030419030.1|damageinducibleprotein[Streptomycessp.NRRLF5065]/11627    -DIDSFFTASG---------------KGAFTR-RMIIST--T--D-KWSVHAEEALDD--
gi|663412917|ref|WP_030408353.1|damageinducibleprotein[Streptomycessp.NRRLF5917]/11630    -DIDSFFTASG---------------KGVFTR-RMIIST--T--D-KWSVHAEEALDD--
gi|671521483|ref|WP_031506489.1|damageinducibleprotein[Streptomycesmegasporus]/11630    -DIDSFFTASG---------------KGVFTR-RMIIST--T--D-KWSVHAEEALDD--
gi|740053144|ref|WP_037902776.1|damageinducibleprotein[Streptomycessp.TAA204]/11626    -DIDSFFTASG---------------KGGFTR-RMIIST--T--E-KWGSNAEAALAD--
gi|663409375|ref|WP_030404974.1|MULTISPECIES:damageinducibleprotein[Streptomyces]/11627    -DIDSFFTASG---------------KGGFTR-RMIVST--T--E-KWGVHAETALDD--
gi|746628608|ref|WP_039654277.1|damageinducibleprotein[Streptomycessp.MUSC125]_gi|745644381|gb|KIE25557.1|damageinducibleprot/1    -DIDSFFTASG---------------KGGFTR-RMIVST--T--E-KWGVHAEAALDD--
gi|727322066|ref|WP_033777320.1|damageinducibleprotein[Salinisporapacifica]/11627    -DIDSFFTASG---------------KGGFTR-RLIIST--T--D-KWNVHAEAALAD--
gi|653653242|ref|WP_027655726.1|damageinducibleprotein[Salinisporapacifica]/11618    -DIDSFFTASG---------------KGGFTR-RLIIST--T--D-KWNVHAEAALAD--
gi|648632543|ref|WP_026324294.1|damageinducibleprotein[Salinisporapacifica]/11621    -DIDSFFTASG---------------KGGFTR-RLIIST--T--E-KWNVHAEAALAD--
gi|881018002|ref|WP_048738463.1|damageinducibleprotein[Corynebacteriumfalsenii]/11676    -HLDSFFESSGRSFITD--RG-----RETFAQ-RIIIST--T--D-NWSSNAEKMLDN--
gi|505465526|ref|WP_015652417.1|helicase[Corynebacteriumcallunae]_gi|459385704|gb|AGG67995.1|helicase[CorynebacteriumcallunaeDS    -QLDSFFEASGRTFETP--EG-----TRSFSN-RLVIST--T--D-KWSSNAEKMLEN--
gi|41223062|emb|CAF19007.1|DNAorRNAhelicaseofsuperfamilyII[CorynebacteriumglutamicumATCC13032]/11646    -QLDSFFEASGRTFETP--EG-----TRSFSN-RLVIST--T--D-KWSSNAEKMLEN--
CGL13032I/11643    -QLDSFFEASGRTFETP--EG-----TSRFSN-RLVIST--T--D-KWSSNAEKMLEN--
gi|751257081|ref|WP_040967675.1|damageinducibleprotein[Corynebacteriumglutamicum]_gi|748811620|gb|AJE67931.1|damageinduciblep/1    -HLDSFFEASGRSFLTE--DG-----TESFAN-RLIIST--T--D-KWSSNAEAMLEN--
gi|749040539|ref|WP_040086524.1|damageinducibleprotein[Corynebacteriumhumireducens]/11663    -HLDTFFERSGRTFTTE--RG-----VETFAN-RIIVST--T--D-RWSRNAEEMLEN--
gi|491662899|ref|WP_005519615.1|helicase[Corynebacteriummatruchotii]_gi|224946756|gb|EEG27965.1|helicaseCterminaldomainprotein/    -HLDSFFEASGRTFVNS--DGNRGGGGAQFAH-RLIIST--T--D-KWSSNAESMLEN--
gi|746717312|ref|WP_039676231.1|damageinducibleprotein[Corynebacteriumminutissimum]_gi|734991118|gb|KHO29219.1|damageinducibl/1    -HLDSFFEASGHSFTTD--KG-----RESFAQ-RIIIST--S--E-RWSKNAEEMLEH--
gi|908654892|emb|CQD15529.1|SuperfamilyIIDNA/RNAhelicase(modularprotein)[Corynebacteriumstriatum]/11674    -HLDSFFEASGHSFTTD--KG-----TESFAQ-RIIIST--S--E-RWSKNAEAMLEH--
gi|895994446|ref|WP_049064418.1|damageinducibleprotein[Corynebacteriumaurimucosum]/11667    -HLDSFFEASGHSFTTD--KG-----TESFAQ-RIIIST--S--E-RWSKNAEAMLEH--
gi|896123331|ref|WP_049145390.1|damageinducibleprotein[Corynebacteriumaurimucosum]/11669    -HLDSFFEASGRSFTTD--KG-----TESFAQ-RIIIST--S--E-RWSKNAEAMLEH--
gi|817681178|ref|WP_046645422.1|damageinducibleprotein[Corynebacteriumstriatum]_gi|817551679|gb|KKO79868.1|damageinduciblepro/1    -HLDSFFEASGHSFTTD--KG-----TESFAQ-RIIIST--S--E-RWSKNAEAMLEH--
gi|896139239|ref|WP_049159146.1|damageinducibleprotein[Corynebacteriumaurimucosum]/11675    -HLDSFFEASGHSFTTD--KG-----TESFAQ-RIIIST--S--E-RWSKNAEAMLEH--
gi|489943947|ref|WP_003847254.1|helicase[Corynebacteriumammoniagenes]_gi|295968218|gb|EFG81467.1|helicaseCterminaldomainprotei/    -HIDSFFEASGHSFETE--KG-----KEHFSH-RYIIST--T--D-KWSKNAEDALEN--
gi|516653464|ref|WP_018022453.1|helicase[Corynebacteriumdoosanense]_gi|697778282|gb|AIT61018.1|damageinducibleprotein[Coryneba/    -HIDSFFEASGHSFQTE--RG-----REHFSH-RFIIST--T--D-KWSSNAESALEN--
gi|493894179|ref|WP_006840129.1|helicase[Corynebacteriumlipophiloflavum]_gi|227078819|gb|EEI16782.1|helicaseCterminaldomainpro/    -HLDSFFEASGHSFSAE--NG-----PESFAN-RIVIST--T--D-KWSANAEKALEN--
gi|740831472|ref|WP_038616755.1|damageinducibleprotein[Corynebacteriumpseudotuberculosis]_gi|666103058|gb|AIG06935.1|putativeh/    -HIDSFFEASGQSFETE--QG-----REHFSH-RYIIST--T--D-KWSKNAEEALAN--
gi|518023322|ref|WP_019193530.1|helicase[Corynebacteriumtimonense]/11655    -HIDSFFEASGQSFETE--KG-----REHFSH-RYIIAT--T--D-KWSKNAEAALAN--
gi|552767697|ref|WP_023019559.1|MULTISPECIES:hypotheticalprotein[Corynebacterium]_gi|550739013|gb|ERS38609.1|hypotheticalprotei    -HIDSFFEASGKSFETE--KG-----REHFSH-RYIIST--T--D-KWSSNAEAALAN--
gi|652659121|ref|WP_027017812.1|damageinducibleprotein[Corynebacteriumpseudodiphtheriticum]/11644    -HIDSFFEASGKSFETE--KG-----REHFSH-RYIIST--T--D-KWSSNAEAALAN--
gi|749039309|ref|WP_040085309.1|damageinducibleprotein[Corynebacteriumhumireducens]/11657    -HLDSFFEASGRTFQTD--KG-----TEAFAN-RLVIST--T--D-HWGSTAEAMLEN--
gi|740835591|ref|WP_038620874.1|damageinducibleprotein[Corynebacteriumulcerans]_gi|698973854|gb|AIU33664.1|HelicaseCterminald/1    -NLDSFFEASGHSFETE--NG-----KEHFAH-RIVIST--T--D-KWGKNAEEALEN--
gi|805307599|ref|WP_046096452.1|damageinducibleprotein[Corynebacteriumulcerans]_gi|803357323|gb|AKA97638.1|Damageinduciblepro/1    -NLDSFFEASGHSFETE--NG-----KEHFAH-RIVIST--T--D-KWGKNAEEALEN--
```

```
gi|504083555|ref|WP_014317549.1|helicase[Corynebacteriumdiphtheriae]_gi|371584249|gb|AEX47914.1|superfamilyIIDNA/RNAhelicase[Co      -NLDSFFEASGRAFQTE--KG-----SEHFKN-RLIIST--T--D-LWSSHAEEALAN--
gi|896182193|ref|WP_049193580.1|damageinducibleprotein[Corynebacteriumjeikeium]/11645      -NLDSFFEASGRSFDTE--KG-----PEHFKN-RLIIST--T--D-LWSSHAEEALAN--
gi|917636459|ref|WP_052205460.1|damageinducibleprotein[Corynebacteriumriegelii]_gi|914295811|gb|AKV59187.1|damageinduciblepro/1      -NIDSFFEASGRSFETE--SA-----VEHFAH-RYIIST--T--D-KWSKHAEAALEN--
gi|737013522|ref|WP_035009205.1|damageinducibleprotein[Corynebacteriumjeikeium]/11651      -NIDSFFEASGHSFETE--SK-----VEHFVH-RYIIST--T--D-KWSKNAEDALEN--
gi|737006291|ref|WP_035002094.1|damageinducibleprotein[Corynebacteriumjeikeium]/11658      -NLDSFFEASGRTFETE--DG-----VQHFGS-RLIIST--T--D-RWSSNAEEMLAN--
gi|737010378|ref|WP_035006133.1|damageinducibleprotein[Corynebacteriumjeikeium]/11650      -NLDSFFEASGRTFKTE--EG-----VEHFGS-RMIIST--T--D-RWSSNAEEMLNN--
gi|737010473|ref|WP_035006228.1|damageinducibleprotein[Corynebacteriumjeikeium]/11657      -HLDSFYEASGRSYIGE--SGE----PEYFAN-RLIIST--T--D-RWTKNAELSLSG--
gi|737006250|ref|WP_035002053.1|damageinducibleprotein[Corynebacteriumjeikeium]/11658      -HLDSFYEASGRSYIGE--SGE----PEYFAS-RLIIST--T--D-RWTKNAELSLSG--
gi|923018168|ref|WP_053412254.1|damageinducibleprotein[Corynebacteriumlactis]_gi|922845184|gb|ALA67504.1|helicase[Corynebacter/      -NLDSFFEASGHSFATE--DG-----PRHFAS-RMIIST--T--D-RWSSHAEAALAD--
gi|923018885|ref|WP_053412970.1|damageinducibleprotein[Corynebacteriumlactis]_gi|922845845|gb|ALA68165.1|helicase[Corynebacter/      -NLDSFFEASGHSFATE--DG-----PRHFAS-RMIIST--T--D-RWSSHAEAALAD--
gi|512464870|ref|WP_016422869.1|hypotheticalprotein[Corynebacteriumsp.HFH0082]_gi|512047830|gb|EPD47505.1|hypotheticalproteinHM      -DLDSFFEASGRSFTTE--SG-----PEHFGS-RIIIST--T--D-HWSSHAEEALAN--
gi|881012697|ref|WP_048733406.1|damageinducibleprotein[Corynebacteriumurealyticum]/11670      -DLDSFFEASGRSFNTE--SG-----PEHFGS-RIVIST--T--D-HWSSHAEEALAN--
gi|755608478|ref|WP_042530519.1|damageinducibleprotein[Corynebacteriumsingulare]_gi|754340239|gb|AJI78730.1|putativehelicase[C/      -HLDSFFEASGHSFETE--EG-----REHFGS-RLIIST--T--D-HWSSHAEDALAN--
gi|491421796|ref|WP_005279593.1|helicase[Corynebacteriumaccolens]_gi|227077180|gb|EEI15143.1|helicaseCterminaldomainprotein[Co/      -HLDSFFEASGHSFETE--HG-----REHFSS-RIIIST--T--D-RWSSHAEEALAN--
gi|550752286|gb|ERS51451.1|hypotheticalproteinHMPREF1282_00033[Corynebacteriumsp.KPL1856]_gi|550759961|gb|ERS58964.1|hypothetic      -HLDSFFEASGHSFETE--NG-----SEYFGS-RMIIST--T--D-YWSSHAEEALAN--
gi|497955053|ref|WP_010269209.1|helicase[Corynebacteriumbovis]/11599      -DLDSFFTASGQGFDTP--DG-----HRYFTN-RIIIST--T--D-RWGRNAEEAAIED--
gi|860580040|ref|WP_048470569.1|hypotheticalprotein[Mycobacteriumchlorophenolicum]_gi|859098575|gb|KMO76871.1|typeIrestrictione      -HIDSFFTASG---------------KKPFTN-RVIIST--T--D-KWGKNAEDALED--
gi|262085905|gb|ACY21873.1|typeIIIrestrictionproteinressubunit[GordoniabronchialisDSM43247]/11632      -DIDTFFTASG---------------KAGFTN-RVIIST--T--D-RWGRNAEDAITD--
gi|495996338|ref|WP_008720917.1|typeIIIrestrictionendonucleasesubunitR[Rhodococcussp.AW25M09]_gi|443412350|emb|CCQ18455.1|TypeI      -DLDSFFTASGQTFPTA--DG-----DRSFTN-RVIIST--T--D-RWGKNAEDAINH--
gi|739284636|ref|WP_037147410.1|damageinducibleprotein[Rhodococcusfascians]/11641      -DLDSFFTASGQTFPTA--DG-----DRSFTN-RVIIST--T--D-RWGKNAEDAINH--
gi|739328195|ref|WP_037190574.1|damageinducibleprotein[Rhodococcusfascians]/11643      -DLDSFFTASGQTFPTA--DG-----DRSFTN-RVIIST--T--D-RWGKNAEDAINH--
gi|811698335|emb|CMN21041.1|helicase[Mycobacteriumtuberculosis]/11725      -DIDSFFTASG---------------KTGFTN-RVIIST--T--D-RWGRNAEDALAD--
gi|801577898|emb|CFK23773.1|helicase[Mycobacteriumtuberculosis]/11675      -DIDSFFTASG---------------KTGFTN-RVIIST--T--D-RWGRNAEDALAD--
MTU18II/11606      -DIDSFFTASG---------------KTGFTN-RVIIST--T--D-RWGRNAEDALAD--
gi|887719858|emb|CMT27886.1|helicase[Mycobacteriumtuberculosis]/11667      -DIDSFFTASG---------------KTGFTN-RVIIST--T--D-RWGRNAEDALAD--
gi|621694743|gb|KAS87156.1|hypotheticalproteinAL70_01801[MycobacteriumtuberculosisTKK_03_0115]/11604      -DIDSFFTASG---------------KTGFTN-RVIIST--T--D-RWGRNAEDALAD--
gi|888128072|emb|CLQ84422.1|helicase[Mycobacteriumtuberculosis]_gi|889917090|emb|CLY86893.1|helicase[Mycobacteriumtuberculosis]      -DIDSFFTASG---------------KTGFTN-RVIIST--T--D-RWGRNAEDALAD--
gi|884823424|emb|CKL96672.1|helicase[Mycobacteriumtuberculosis]/11466      -DIDSFFTASG---------------KTGFTN-RVIIST--T--D-RWGRNAEDALAD--
gi|505103239|ref|WP_015290341.1|hypotheticalprotein[Mycobacteriumcanettii]_gi|432162733|emb|CCK60117.1|Conservedproteinofunknow      -DIDSFFTASG---------------KTGFTN-RVIIST--T--D-RWGRNAEDALAD--
gi|505116250|ref|WP_015303352.1|hypotheticalprotein[Mycobacteriumcanettii]_gi|432158726|emb|CCK56026.1|Conservedproteinofunknow      -DIDSFFTASG---------------KTGFTN-RVIIST--T--D-RWGRNAEDALAD--
gi|432154735|emb|CCK51974.1|Conservedproteinofunknownfunction(part1)[MycobacteriumcanettiiCIPT140060008]/11588      -DIDSFFTASG---------------KTGFTN-RVIIST--T--D-RWGRNAEDALAD--
gi|809008027|ref|WP_046295375.1|hypotheticalprotein[Mycobacteriumsp.UM_Kg17]/11624      -DIDSFFTASG---------------KTAFTN-RVIIST--T--D-RWGKNAEAALDD--
gi|808802633|ref|WP_046284139.1|hypotheticalprotein[Mycobacteriumsp.UM_NZ2]/11624      -DIDSFFTASG---------------KTAFTN-RVIIST--T--D-RWGKNAEAALDD--
gi|750125774|ref|WP_040429852.1|damageinducibleprotein[Corynebacteriumnuruki]/11668      -DIDSFFTESG---------------KAPFTR-RVIIST--T--D-KWGKNAEAALEG--
gi|602237614|gb|EYT60960.1|damageinducibleprotein[Dietziasp.UCDTHP]/11473      -NIDSFLAESG---------------KAPFTR-RIIIST--T--N-KWGKNALAALEG--
gi|665897412|ref|WP_031264618.1|damageinducibleprotein[Dietziasp.UCDTHP]_gi|602237599|gb|EYT60945.1|helicase[Dietziasp.UCDTH/11      -NIDSFFTESG---------------KAPFTR-RIIIST--T--N-KWGKNALAALEG--
gi|502903674|ref|WP_013138650.1|helicase[Segniliparusrotundus]_gi|296181291|gb|ADG98197.1|typeIIIrestrictionproteinressubunit[S      -DIDSFFTELG---------------KEPFVK-GIIIST--T--D-KWGKNAEDALVG--
gi|780248306|ref|WP_045538930.1|hypotheticalprotein[Gordoniasp.no.9]_gi|778142514|gb|KJR06917.1|hypotheticalproteinUG54_12320[G      -DIDSYFTALG---------------KAPFTN-GIIIST--T--D-RWGKNAEEALND--
gi|665897368|ref|WP_031264574.1|damageinducibleprotein[Dietziasp.UCDTHP]_gi|602239137|gb|EYT62483.1|helicase[Dietziasp.UCDTH/11      -DLDSFFTASG---------------KAPFTN-RVIIST--T--N-RWGKHAEDALDG--
gi|493372923|ref|WP_006329236.1|helicase[Gordoniarhizosphera]_gi|403207917|dbj|GAB88168.1|hypotheticalproteinGORHZ_006_00370[Go      -DIDSFFTASG---------------KKPFTN-RIIIST--T--D-KWGRNAEDALDG--
gi|764904870|ref|WP_044487821.1|hypotheticalprotein[Mycobacteriumtuberculosis]_gi|625005353|gb|KBZ57120.1|hypotheticalproteinK8      -DIDSFFTASG---------------KNPFTN-RIIIST--T--D-RWGGHAEDALED--
gi|927068212|ref|WP_053778441.1|damageinducibleprotein[Gordoniasp.NB41Y]_gi|926660541|gb|EMP14526.2|damageinducibleprotein[G/11      -DIDSFFTASG---------------KKPFTN-RIIIST--T--D-RWGKNAEDTLDD--
gi|935514169|ref|WP_054429894.1|damageinducibleprotein[Mycobacteriumimmunogenum]_gi|929801120|gb|KPG29247.1|damageinduciblepr/1      -DIDSFFTASG---------------KNPFTN-RIIIST--T--D-KWGKNAEEALED--
gi|941317728|ref|WP_055118574.1|hypotheticalprotein[Mycobacteriumperegrinum]/11657      -DIDSFFTASG---------------KNPFTN-RIIIST--T--D-KWGKNAEDALEG--
gi|933683673|gb|ALG86377.1|hypotheticalproteinACH46_20120[Gordoniasp.QH11]/11636      -DIDSFFTESG---------------KMPFTN-RVIIST--T--D-RWGRNAEEALDG--
gi|873553947|emb|CRY84110.1|Predictedhelicase[Nocardiafarcinica]/11688      -DIDSFFTASG---------------KKPFTN-RVIIST--T--D-RWGRNAEDALQD--
gi|499525749|ref|WP_011212389.1|helicase[Nocardiafarcinica]_gi|54019339|dbj|BAD60707.1|hypotheticalprotein[NocardiafarcinicaIFM      -DIDSFFTASG---------------KKPFTN-RVIIST--T--D-RWGRNAEDALQD--
gi|640109609|ref|WP_024794187.1|damageinducibleprotein[Tomitellabiformata]/11653      -DIDSFFTASG---------------KKPFSN-RLIIST--T--D-RWGSNAEAALAD--
gi|640109991|ref|WP_024794334.1|hypotheticalprotein[Tomitellabiformata]/11639      -DIDSFFTASG---------------KKPFSN-RLIIST--T--D-RWGSNAEAALAD--
gi|939125496|ref|WP_054831438.1|damageinducibleprotein[Rhodococcuserythropolis]/11669      -DIDSFFTASG---------------KTPFSN-RIIIST--T--D-RWGKNAEDALEG--
gi|910258550|ref|WP_050062711.1|hypotheticalprotein[Rhodococcussp.RD6.2]_gi|846871967|emb|CRK49225.1|conservedhypotheticalprote      -DIDSFFTASG---------------KKPFTN-RVIIST--T--D-RWGKNAEDALDG--
gi|797148189|gb|KJY52358.1|putativeHelicase[Bifidobacteriumasteroides]/11801      -DISTFFTAAFA-----------R----KVEPDHLIIVH--T-GA-GLTKTLYR--EA--
gi|535476570|gb|EFG27357.2|hypotheticalproteinHMPREF9020_00999[ScardoviainopinataF0304]/11827      -DVSTFFASSDT-----------R----GIKRGHFIIAH--T-GG-GLTQTLEK--EA--
gi|737482729|ref|WP_035462496.1|hypotheticalprotein[Alicyclobacillusmacrosporangiidus]/11512      -ELSTFVASATS-----------P----RFSFAKLLVVA----VG-GVTRTAEAR--C--
gi|685434354|gb|KGA95679.1|helicase[BacillusalcalophilusATCC27647=CGMCC1.3604]/11575      -HIDSFLNEVGK-----------Q----YYAE-GIIVTS----TD-KWSNNADDALKF--
gi|945370253|gb|KQU27251.1|helicase[Bacillussp.Leaf13]/11557      -HIDSFLNEVGK-----------K----FYAK-GIIITS----TD-RWGKNAEEALIG--
gi|880997015|ref|WP_048719116.1|helicase[Enterococcushirae]/11563      -DIDSFLNEVGK-----------R----QYSE-GIVFSS----TD-NWSSNAEDALSE--
gi|945356929|gb|KQU14100.1|helicase[Bacillussp.Leaf49]/11547      -HIDSYLNEVGK-----------S----YYSK-GIIIAS----TD-KWSKHAEDALKY--
gi|763482876|gb|KIZ53217.1|helicase[Bacillussafensis]/11563      -HIDSFLNEVGK-----------S----YYAE-GIIVTS----TD-KWSSNANNALLD--
gi|852223292|ref|WP_048309707.1|helicase[Anaerobacillusmacyae]_gi|850322910|gb|KMM38590.1|helicase[Anaerobacillusmacyae]/11573      -HIDSFLNEVGK-----------S----YYSE-GIIVTS----TD-KWSNNANDALLN--
gi|739708665|ref|WP_037562619.1|helicase[Sporolactobacillusterrae]/11573      -HIDSFLNEVGK-----------N----YYAE-GIIVTS----TD-KWSSNANDALLN--
gi|496687561|ref|WP_009329104.1|MULTISPECIES:helicase[Bacillus]_gi|317390867|gb|EFV71668.1|endonucleaseandmethylaseLlaGI[Bacill      -HIDSFLNEVGK-----------S----YYTE-GIVVTS----TD-KWSSNANDALLD--
gi|930213599|gb|KPH74225.1|helicase[Oceanobacilluscaeni]/11569      -QIDSFLNEVGK-----------S----YYAE-GIIVTS----TD-KWSNNADDALLV--
gi|516366003|ref|WP_017756036.1|helicase[Bacillussp.ZYK]/11571      -HIDSFLNEVGK-----------S----YYGE-GIIVTS----TD-KWSSNADDALLY--
gi|488909977|ref|WP_002821052.1|helicase[Oenococcusoeni]_gi|399965230|gb|EJN99855.1|endonucleaseandmethylaseLlaGI[Oenococcusoen      -TIDSFMAEMNK-----------S----YYAN-GLIVST----ID-DWNKNAEADFEN--
gi|495019967|ref|WP_007745780.1|helicase[Oenococcuskitaharae]_gi|366983682|gb|EHN59081.1|typeIIIrestrictionenzyme,ressubunit[Oe      -TIDSFMAEMNK-----------S----YYAT-GLIVST----ID-DWNKNAEADFEN--
gi|489666119|ref|WP_003570406.1|typeIIIrestrictionendonuclease[Lactobacilluscasei]_gi|410527834|gb|EKQ02696.1|typeIIIrestrictio      -TINSYVAELNK-----------N----YYAD-GLLVAT----TD-DWNKNAEAALLD--
```

```
gi|511447623|gb|EPC78110.1|superfamilyIIDNA/RNAhelicase[Lactobacillusparacaseisubsp.paracaseiLpp221}/11615               -TINSYVAELNK-----------N----YYTD-GLLVAT----TD-DWNKNAEAALDN--
gi|497700411|ref|WP_010014595.1|helicase[Lactobacilluscoryniformis]/11585                                                  -TINSYVAELNK-----------N----YYVD-GLLVAT----TD-DWNKNAEAALDD--
gi|489784048|ref|WP_003687939.1|helicase[Lactobacillusmali]_gi|394428539|gb|EJF01080.1|superfamilyIIDNA/RNAhelicase[Lactobacill -TINSYVAELNK-----------N----YYAD-GLLVTT----TD-DWNKNAEAALDD--
gi|446780825|ref|WP_000858081.1|helicase[Staphylococcusaureus]_gi|374396604|gb|EHQ67835.1|typeIIIrestrictionenzyme,ressubunit[S -EINSFIAELGK-----------S----YYSD-GLIVST----TD-EWNQNALNTIEK--
gi|510796658|ref|WP_016171456.1|hypotheticalprotein[Enterococcusdispar]_gi|508167532|gb|EOT44083.1|hypotheticalproteinOMK_00227 -EINSFVAELGK-----------D----YYSS-GMIVST----TD-EWNQNALTTVQE--
gi|612613473|gb|EZS30411.1|hypotheticalproteinW603_02482[StaphylococcusaureusVET0353R]_gi|612881917|gb|EZU93992.1|hypotheticalp -EINSFIAELGK-----------D----YYSS-GMIVST----TD-EWNQNALTTVQE--
gi|488332893|ref|WP_002402278.1|helicase[Enterococcusfaecalis]_gi|306513864|gb|EFM82466.1|helicaseCterminaldomainprotein[Enter/ -HIDSFLAELGK-----------S----YYSD-GIIVYS----SD-SLSSNADEAINQ--
gi|921166029|ref|WP_053111806.1|helicase[Enterococcusdurans]_gi|915771660|gb|AKX85928.1|helicase[Enterococcusdurans]/11562     -HIDSFLAELGK-----------S----YYSD-GIIVYS----LD-SLSSNADEAINQ--
gi|498525358|ref|WP_010825030.1|hypotheticalprotein[Enterococcusfaecalis]_gi|487059631|gb|EOJ86727.1|hypotheticalproteinWOC_005 -HIDSFLAELGK-----------S----YYSD-GIIVYS----LD-SLSSNADEAINQ--
gi|498412624|ref|WP_010722070.1|hypotheticalprotein[Enterococcusfaecium]_gi|486634654|gb|EOF75253.1|hypotheticalproteinSGC_0253 -HIDSFLAELGK-----------S----YYSD-GIIVYS----LD-SLSSNADEAINQ--
gi|498408534|ref|WP_010718364.1|hypotheticalprotein[Enterococcusfaecium]_gi|486609608|gb|EOF50873.1|hypotheticalproteinSCW_0257 -HIDSFLAELGK-----------S----YYSD-GIIVYS----LD-SLSSNADEAINQ--
gi|498407475|ref|WP_010717483.1|hypotheticalprotein[Enterococcusfaecalis]_gi|486594855|gb|EOF36348.1|hypotheticalproteinSCK_005 -HIDSFLAELGK-----------S----YYSD-GIIVYS----LD-SLSSNADEAINQ--
gi|514885392|ref|WP_016615344.1|helicase[Enterococcusfaecalis]_gi|514505843|gb|EPH71676.1|helicaseprotein[Enterococcusfaecalis0 -HIDSFLAELGK-----------S----YYSD-GIIVYS----SD-SLSSNADEAINQ--
gi|325157603|gb|EGC69759.1|helicaseCterminaldomainprotein[EnterococcuscasseliflavusATCC12755]/11561                         -HIDSFLAELGK-----------S----YYSD-GIIVYS----LD-SLSSNADEAINQ--
gi|895856206|ref|WP_048967668.1|helicase[Enterococcusfaecalis]/11551                                                       -HIDSFLAELGK-----------S----YYSD-GIIVYS----LD-SLSSNADEAINQ--
gi|639458836|ref|WP_024636366.1|helicase[Enterococcusfaecium]_gi|658176502|gb|KEI51159.1|helicase[EnterococcusfaeciumUC7256]/1/ -HIDSFLAELGK-----------S----YYSD-GIIVYS----LD-SLSSNADEAINQ--
gi|488266511|ref|WP_002337719.1|hypotheticalprotein[Enterococcusfaecium]_gi|430581409|gb|ELB19854.1|hypotheticalproteinOIQ_0351 -HIDSFLAELGK-----------S----YYFD-GIIVYS----LD-SLSSNADEAINQ--
gi|446353356|ref|WP_000431211.1|helicase[Staphylococcusaureus]_gi|253726329|gb|EES95058.1|helicaseCterminaldomainprotein[Staph/ -EINSFVAELGS-----------T----YYTR-GLIIAT----VD-EWNSNARATVDK--
gi|672712068|emb|CDM14336.1|DNAorRNAhelicaseofsuperfamilyII[StaphylococcusepidermidisPM221]/11464                          -EINSFVAETGS-----------K----YYQG-GLIVST----VD-EWNKNARSTIEN--
gi|896298990|ref|WP_049279905.1|helicase[Staphylococcusaureus]/11571                                                       -EINSFVAETGS-----------K----YYQG-GLIVST----VD-EWNKNARSTIEN--
gi|541903432|gb|ERH36190.1|hypotheticalproteinSEQU_00085[StaphylococcusequorumUMCCNS924]/11578                             -EINSFVAETGS-----------N----YYQG-GLIIST----VD-DWNKNAKSTIEN--
gi|488289747|ref|WP_002360955.1|helicase[Enterococcusfaecalis]_gi|256682216|gb|EEU21911.1|endonucleaseandmethylaseLlaGI[Enteroc -EINSFVAELGK-----------N----YYQH-GLIVST----VD-DWNKNARDTIDH--
gi|494392045|ref|WP_007207894.1|helicase[Enterococcusitalicus]_gi|315483798|gb|EFU74284.1|helicaseCterminaldomainprotein[Enter/ -EINSFVAELGK-----------S----YYQR-GLIVST----VD-DWNKNARDTIER--
gi|446350692|ref|WP_000428547.1|helicase[Bacilluscereus]_gi|228727580|gb|EEL78721.1|RestrictionmodificationsystemLlaBIII[Bacil/ -EINSFVAEMGK-----------S----YYQR-GLIIST----VD-DWNKNARETIEY--
gi|488255302|ref|WP_002326510.1|hypotheticalprotein[Enterococcusfaecium]_gi|430480276|gb|ELA57459.1|hypotheticalproteinOGE_0473 -EINSFVAELGK-----------S----YYQR-GLIVST----VD-DWNKNALETIEY--
gi|843253770|ref|WP_047916698.1|helicase[Lactococcuspiscium]_gi|802124677|emb|CEN29502.1|EndonucleaseandmethylaseLlaGI,TypeISPr -EINSFVAELGK-----------S----YYQR-GLIVST----MD-DWNSNARETIDQ--
LlaBIII/11578                                                                                                              -EINSFVAELGK-----------S----YYQR-GLIVST----MD-DWNSNARETIDQ--
gi|499320531|ref|WP_011011023.1|helicase[Listeriainnocua]_gi|16415805|emb|CAC42019.1|pli0021[ListeriainnocuaClip11262]/11569    -EINSFVAELGK-----------S----YYQR-GLIVST----MD-DWNSNARETIDQ--
gi|760026859|ref|WP_043709973.1|helicase[Weissellacibaria]_gi|758337165|gb|KIU20061.1|UvsWhelicase[Weissellacibaria]/11567      -AINSFVAELGK-----------S----YYQR-GLIIST----VD-EWNSNARETIEE--
LlaGI/11570                                                                                                                -EINSFVAELGK-----------S----YYQR-GLIVST----MD-DWNSNARETIDQ--
gi|499988414|ref|WP_011669132.1|helicase[Lactococcuslactis]_gi|116109019|gb|ABJ74139.1|DNAorRNAhelicaseofsuperfamilyII[Lactococ -AINSFVAELGK-----------S----YYQR-GLIVST----MD-DWNSNARETIDQ--
gi|685938360|ref|WP_031670319.1|helicase[Listeriamonocytogenes]_gi|590403387|gb|EXL23261.1|hypotheticalproteinX846_1956[Listeri -EINSFVAELGK-----------S----YYQR-GLIVST----MD-DWNSNARETIDQ--
gi|530793219|gb|EQC56240.1|helicase[Lactococcuslactissubsp.cremorisTIFN5]_gi|530793247|gb|EQC56263.1|helicase[Lactococcuslactis -EINSFVAELGK-----------S----YYQR-GLIVST----MD-DWNSNARETIDQ--
gi|695270006|ref|WP_032492075.1|hypotheticalprotein[Listeriamonocytogenes]_gi|695212872|ref|YP_009081522.1|hypotheticalproteinp -EINSFVAELGK-----------S----YYQR-GLIVST----MD-DWNSNARETIDQ--
gi|504101310|ref|WP_014335297.1|helicase[Leuconostocmesenteroides]_gi|859247905|gb|AKO63885.1|helicase(plasmid)[Leuconostocmese -AINSFVAELGK-----------S----YYQH-GLIVST----VD-EWNSNARETIEQ--
gi|488905190|ref|WP_002816265.1|helicase[Leuconostocmesenteroides]_gi|227351522|gb|EEJ41777.1|helicaseCterminaldomainprotein[L/ -AINSFVAELGK-----------N----YYQH-GLIIST----VD-EWNNNARETIEQ--
gi|518121285|ref|WP_019291493.1|MULTISPECIES:hypotheticalprotein[Lactococcus]/11588                                        -DIDSFLSEINK-----------D----YYAE-GLIVSS----TD-DWGANAEQAIEG--
gi|754857213|ref|WP_042217902.1|helicase[Lactococcusgarvieae]/11549                                                        -DIDSFLGELGK-----------D----YYSE-GIIVST----MD-EWGKNAEEAINN--
gi|640048455|ref|WP_024784762.1|helicase[Streptococcusmutans]/11564                                                        -NIDSFLSELGK-----------D----YYES-GIIVAS----TD-NWGKNAEKALAD--
gi|512695361|ref|WP_016476323.1|hypotheticalprotein[Veillonellasp.HPA0037]_gi|512079853|gb|EPD79018.1|hypotheticalproteinHMPREF -NIDSFLSELGK-----------D----FYES-GIIVAS----TD-KWGKNAEKALED--
gi|636963514|ref|WP_024403747.1|helicase[Streptococcussuis]/11574                                                          -DIDSFLGELGK-----------D----YYES-GIIVAS----TD-KWGANAEKALAD--
gi|881115312|ref|WP_048802949.1|helicase[Staphylococcuspasteuri]/11573                                                     -DIDSFLGELGK-----------D----YYDS-GIIVSS----TD-KWGKNAENALDS--
gi|446063996|ref|WP_000141851.1|helicase[Streptococcusmitis]_gi|307618725|gb|EFN97864.1|endonucleaseandmethylaseLlaGI[Streptoco -NIDSFLGELGK-----------D----YYES-GIIVAS----TD-KWGKNAEKALAD--
gi|488206609|ref|WP_002277817.1|restrictionmodificationsystemLlaBIII[Streptococcusmutans]_gi|449173033|gb|EMB75626.1|restricti/ -NIDSFLSELGK-----------D----YYES-GVIVAS----TD-KWGKNAEKALAD--
gi|640023290|ref|WP_024781621.1|helicase[Streptococcusmutans]/11566                                                        -NIDSFLSELGK-----------D----YYES-GVIVAS----TD-KWGKNAEKALAD--
gi|814946581|ref|WP_046393781.1|helicase[Streptococcusuberis]_gi|810600885|gb|KKF57499.1|helicase[StreptococcusuberisB362]/1156 -NIDSFLGEIGK-----------S----YYSS-GIIVSS----TD-KWGRNAEKALAD--
gi|765470762|ref|WP_044720336.1|helicase[Streptococcussuis]/11559                                                          -DIDSFLGELGK-----------N----YYES-GIIVAS----TD-KWGRNAEQALAD--
gi|654507127|ref|WP_027976443.1|helicase[Streptococcusdevriesei]/11558                                                     -NIDSFLSELGK-----------S----YYES-GIIVAS----TD-KWGKNAEKALAD--
gi|530812902|gb|EQC72481.1|DNAorRNAhelicaseofsuperfamilyII[Streptococcussp.HSISS2]/11570                                   -NIDSFLSELGK-----------N----YYES-GIIVAS----TD-KWGKNAENALSD--
gi|489158846|ref|WP_003068504.1|helicase[Streptococcusintermedius]_gi|355367114|gb|EHG14827.1|hypotheticalproteinHMPREF9682_002 -DIDSFLGELGK-----------D----YYDS-GIIVAS----TD-KWGTKAEEALVN--
RM.Lru27782ORF13200P/11620                                                                                                 -TINSFVAEAAK-----------D----YYAD-GIIIST----TD-EWNRNAERALAG--
gi|492676546|ref|WP_005919236.1|DNAphosphorothioationsystemrestrictionenzyme[Pediococcusacidilactici]_gi|482517924|gb|EOA08103. -TINSFIAEFSK-----------N----YYAA-GIIVST----TD-QWNRNAEQSLEG--
gi|498183116|ref|WP_010497272.1|helicase[Lactobacillusacidipiscis]/11563                                                   -TINSFIAEASK-----------D----YYTD-GIIVST----TD-KWNKNAEQALEG--
gi|822100485|ref|WP_046921865.1|helicase[Lactobacillusruminis]_gi|821684442|gb|KLA43321.1|restrictionmodificationsystemLlaBIII/ -NIDSFLAEVGK-----------D----YYSD-GIIVTS----TN-DWNKNAKNATEY--
gi|935565832|ref|WP_054450311.1|helicase[Lactobacilluskunkeei]/11573                                                       -AINSFVAETGK-----------D----CYSA-GIIVST----TD-EWNKNAESTLKN--
gi|752296887|ref|WP_041152901.1|helicase[Lactobacilluskunkeei]_gi|751663520|gb|KIM18358.1|helicase[Lactobacilluskunkeei]/11577 -AINSFVAETGK-----------D----CYSA-GIIVST----TD-EWNKNAESTLKN--
gi|495745291|ref|WP_008469870.1|helicase[Lactobacillushominis]_gi|394484442|emb|CCI81207.1|RestrictionmodificationsystemLlaBII/ -EIDSFVAETAK-----------D----AYSA-GMVVST----ID-EWNKNAQATLEN--
gi|918672414|ref|WP_052543134.1|helicase[Lactobacillussp.]/11564                                                           -GIDSFVAETGK-----------K----VYSA-GMLVSS----TD-DWNRNARKTLDD--
gi|799141631|ref|WP_045927261.1|helicase[Lactobacilluskullabergensis]_gi|797155335|gb|KJY59264.1|putativerestrictionendonucleas -EIDSFIAEAGK-----------N----YYDS-GMLVSS----TD-ELNKNVEETFAK--
gi|504379990|ref|WP_014567092.1|helicase[Lactobacillusjohnsonii]_gi|329666726|gb|AEB92674.1|putativerestrictionendonuclease[Lac -EIDSFVAEAGK-----------N----VYSA-GIIVSS----TD-KWNKNAKATLED--
gi|489880179|ref|WP_003783646.1|DNAdamageinducibleprotein[Actinomycesnaeslundii]_gi|399901561|gb|EJN84440.1|typeIIIrestriction/ -DVDSFLAESGT-----------G----EFTS-RLFVST--T--E-HWNSAAEETVSR--
gi|496524497|ref|WP_009232780.1|DNAdamageinducibleprotein[Actinomycessp.oraltaxon849]_gi|365265413|gb|EHM95177.1|hypotheticalp/ -DVDSFLAESGT-----------G----EFTS-RLFVST--T--E-RWNSAAEETVSR--
gi|915397782|ref|WP_050783167.1|damageinducibleprotein[Actinomycesoris]/11694                                              -DVDSFLAESGT-----------G----EFTS-RLFVST--T--E-RWNSAAEETVSR--
gi|502925248|ref|WP_013160224.1|helicase[Propionibacteriumfreudenreichii]_gi|296921272|emb|CBL55824.1|helicase[Propionibacteriu -DIDSFFTASGK-----------SWDGMRFDN-RIIIST--T--D-RWSGHAETALAN--
gi|732164352|emb|CEG87481.1|helicase[Propionibacteriumfreudenreichii]/11618                                                -DIDSFFTASGR-----------SWDGVQFAN-RIIIST--T--D-RWSHPAEEALEN--
gi|739047402|ref|WP_036925058.1|damageinducibleprotein[Propionibacteriumthoenii]/11596                                     -DIDSFFTASGR-----------SWDGVVFDN-RIIIST--T--D-RWSRNAEEALRH--
gi|739112839|ref|WP_036982533.1|damageinducibleprotein[Propionibacteriumjensenii]/11594                                    -DIDSFFTASGK-----------RWDGVAFTD-RIIIST--T--D-RWSRNAEDALRH--
```

```
gi|918023558|ref|WP_052314121.1|damageinducibleprotein[Nocardiathailandica]/11612        -DIDSFISASAK-----------S----EWTE-RYVFDT--A--A-GWSGNAEETLEG--
gi|498380271|ref|WP_010694427.1|DNAdamageinducibleprotein[Saccharopolysporaspinosa]/11605        -DIDSFVTTSSK-----------A----EFSL-RYLVDT--A--R-DWSKNAKDALDG--
gi|749991459|ref|WP_040339809.1|damageinducibleprotein[CandidatusBlastococcusmassiliensis]/11597        -DIDSFVSASAH-----------S----QFTR-RIVFDT--A--A-GWSANAEDTLAD--
gi|754177801|ref|WP_041796231.1|damageinducibleprotein[Modestobactermarinus]/11592        -DIDSFVSASAS-----------S----QFTR-RIVFDT--A--A-GWSVNAEETLAG--
gi|504188248|ref|WP_014375350.1|DNAdamageinducibleprotein[Blastococcussaxobsidens]_gi|378782790|emb|CCG02456.1|putativeHelicas/        -DIDSFVSASAS-----------A----QFTR-RIVFDT--A--A-GWSANAEDTLAD--
gi|946476713|gb|KRF26798.1|damageinducibleprotein[Phycicoccussp.Soil803]/11609        -DIDSFIAASAK-----------P----QYTG-RIMFST--A--K-DWGPTAERQLDG--
gi|700176672|gb|KGN37509.1|DNAdamageinducibleprotein[KnoelliasubterraneaKCTC19937]/11633        -DIDSFLAATAK-----------D----KFTG-RIVLST--A--R-GWGSNIEKQLDG--
gi|700168841|gb|KGN29914.1|hypotheticalproteinN802_10215[KnoelliasinensisKCTC19936]/11631        -DIDSFLAATAK-----------D----KFTG-RIVLST--A--K-GWGPTVEKQLDG--
gi|946426241|gb|KRE76610.1|damageinducibleprotein[Arthrobactersp.Soil763]/11606        -DIDTFLSESGK-----------E----GFVE-RILVST--T--D-KWNLHAENAIKN--
gi|517318525|ref|WP_018500927.1|hypotheticalprotein[Frankiasp.BCU110501]/11592        -MIDSFLAVSGH-----------A----KYGA-RIIIST--S--D-KWNPNAEDAIKN--
gi|499072429|ref|WP_010851327.1|Helicase[Tetrasphaeraelongata]_gi|478756579|emb|CCH71501.1|putativeHelicase[Tetrasphaeraelongat        -DIDTFLSASGK-----------E----GFTQ-RIIVST--T--D-KWNAHAEDAIQG--
gi|750080666|ref|WP_040385350.1|damageinducibleprotein[Demetriaterragena]/11625        -DIDTFLSASGK-----------A----GFTE-RMIVST--T--D-KWNQHAEDTIQG--
gi|752558292|ref|WP_041229523.1|damageinducibleprotein[Dermacoccusnishinomiyaensis]_gi|664689315|gb|AIF41904.1|damageinducibl/1        -DIDTFLSASGT-----------T----DFAQ-RIIIDT--T--S-DWGPNAEATLQK--
gi|829089213|ref|WP_047310701.1|damageinducibleprotein[Dermacoccussp.PE3]_gi|828384812|gb|KLO63770.1|damageinducibleprotein[D/1        -DIDSFLSASGT-----------S----KFGQ-RIIVDT--G--A-PWGRNAEDTIRD--
gi|748756635|ref|WP_040014750.1|damageinducibleprotein[Dermacoccussp.Ellin185]/11596        -DIDSFLSASGT-----------S----KFGQ-RVIVDT--G--A-PWGKNAEDTIRD--
gi|916534724|ref|WP_051141848.1|damageinducibleprotein[Actinomycesneuii]/11632        -DLDSFLSESGK-----------A----PFSR-RIYVET--T--GETWSANAESAIEG--
gi|921140030|ref|WP_053090276.1|damageinducibleprotein[Rothiamucilaginosa]/11616        -HIDSFISASGR-----------K----PFTH-RIIVST--T---DKWSKNAEKMLEE--
gi|896406751|ref|WP_049343749.1|damageinducibleprotein[Rothiamucilaginosa]/11647        -HIDSFISASGR-----------K----PFTH-RIIVST--T---DKWSKNAEKMLEE--
gi|723608234|gb|AIY01610.1|putativehelicase[Arthrobactersp.PAMC25486]/11613        -HIDSFLSASSK-----------K----PFTH-AVVVDT--T--EGEWSANATEMLDG--
gi|946394527|gb|KRE45160.1|damageinducibleprotein[Arthrobactersp.Soil736]/11616        -DIDSFISASGK-----------E----PFTH-RLIVST--T---SKWTQNAEEMLDS--
gi|515765304|ref|WP_017197904.1|hypotheticalprotein[Arthrobactersp.M2012083]/11648        -DVDSFISASGK-----------P----PFTH-RLIVDT--T--GRDWSANAEEMLDD--
gi|945315587|gb|KQT73127.1|damageinducibleprotein[Microbacteriumsp.Leaf436]/11612        -MLDSFLSASGKM--TE--NGA-P----EFTS-RLVIST--S--DK-WGKNAEDAISG--
gi|759908497|ref|WP_043594599.1|damageinducibleprotein[Frigoribacteriumsp.MEB024]_gi|758271359|gb|KIU03346.1|damageinduciblep/1        -DLDSFFTASGK-----------H----PFSS-RIFVST--S--SK-WGKHAEEALNG--
gi|945072850|gb|KQR34237.1|hypotheticalproteinASF75_00055[Curtobacteriumsp.Leaf154]/11597        -DLDSFFTASGK-----------A----AFSS-RLIVAT-----AP-LGKNALEALVD--
gi|551263152|ref|WP_022883363.1|DNAdamageinducibleprotein[Glaciibactersuperstes]/11621        -DIDSFFTASGK-----------H----PFTS-RMVIST--T--DK-WSKHAVDALVD--
gi|945452341|gb|KQV08491.1|hypotheticalproteinASC63_10170[Leifsoniasp.Root112D2]/11623        -DIDSFFTASGK-----------E----GFSS-RAIVST--T--SH-WSKHATDALLG--
gi|742759483|gb|KIC69912.1|damageinducibleprotein[Kocuriarhizophila]/11610        -DIDSFISASAT-----------A----EFGE-RLMVST--S--LKELGGNTRKTIDN--
gi|737919995|ref|WP_035885304.1|damageinducibleprotein[Kocuriarhizophila]/11611        -DIDSFISASAT-----------A----EFGE-RLMVST--S--LKELGGNTRKTIDN--
gi|737997701|ref|WP_035959325.1|damageinducibleprotein[Kocuriamarina]_gi|725823884|gb|KHE75603.1|damageinducibleprotein[Kocur/1        -DVDSFISASAT-----------A----EFGE-RLMVST--S--RKELGGNARKTIEN--
gi|664521070|ref|WP_031037650.1|damageinducibleprotein[Streptomycessp.NRRLWC3774]/11624        -DIDSFFTASGK-----------G----GFTR-RMIVST--T--EK-WGVHAEAALDD--
gi|500101034|ref|WP_011777041.1|DNAdamageinducibleprotein[Arthrobacteraurescens]_gi|119951547|gb|ABM10457.1|putativeHelicase[A/        -QIDSFFTAAGK-----------K----EFSR-GLIVST--S--DK-WSKHAEDALRG--
gi|916871953|ref|WP_051479009.1|damageinducibleprotein[Arthrobactersp.H5]/11588        -HIDSFFTAVGK-----------A----DFTY-GMIVTT--T--DK-WSKHAEDALRG--
gi|612084649|gb|EZP43369.1|TypeIIIrestrictionenzyme,ressubunit[Micrococcusluteus]/11625        -SIDSFFTELGK-----------V----DFAH-GMVIDT--A--LE-WSSSAEAARAG--
gi|759739708|ref|WP_043450422.1|damageinducibleprotein[Arthrobacterphenanthrenivorans]_gi|742758264|gb|KIC68716.1|damageinduc/1        -QIDSFFTAVGK-----------S----DFSY-GMVVST--T--DR-WSKHAEDALEG--
gi|636845448|ref|WP_024366823.1|damageinducibleprotein[Arthrobactersp.TB26]/11605        -QIDSFFTAAGK-----------V----DFSY-GLVVST--T--DK-WSKHAETALEG--
gi|500009025|ref|WP_011689743.1|DNAdamageinducibleprotein[Arthrobactersp.FB24]_gi|116613051|gb|ABK05760.1|typeIIIrestrictionen/        -HIDSFFTAAGK-----------V----DFSY-GLVVST--T--DK-WSKHAETALEG--
gi|821249847|gb|KKZ12083.1|hypotheticalproteinTQ37_06090[CandidatusSynechococcusspongiarum15L]/11436        -EIDSFLAASGR-----------P----EFRR-RIWMDT--T--GRAWSAKAEEILRQ--
gi|739446902|ref|WP_037306885.1|damageinducibleprotein[Ruegeriahalocynthiae]/11592        -DVDKFLSASSK-----------A----PFQR-RVFIDT--T--ERDWSENAEETIRG--
gi|640620081|ref|WP_025048131.1|damageinducibleprotein[Sulfitobactermediterraneus]_gi|751956650|gb|KIN77213.1|TypeIIIrestricti/        -DIQTFITASGK-----------A----PFSQ-RLFIDT--T--EVDWGENAEDAIRG--
gi|740247634|ref|WP_038088583.1|damageinducibleprotein[Thiobacillusprosperus]_gi|684049748|gb|KFZ90853.1|putativehelicase[Thio/        -HVSSFLADSAKVFTVD--GIE-Q----SFAY-RLFVST--T--A-RLGKGAEGVIAD--
gi|518420321|ref|WP_019590528.1|hypotheticalprotein[Thioalkalivibriosp.ALE20]/11603        -DIDSFFTASGKRFATN--EGER-----YFAR-RLIVTT--A--E-HWSKKAEEALAD--
gi|500156826|ref|WP_011831496.1|DNAdamageinducibleprotein[Methylibiumpetroleiphilum]_gi|124261892|gb|ABM96881.1|adeninespecifi/        -EIDSFFTASGKKFSTK--DGSR-----HFAH-RLIVST--T--D-KWSKHAEDALAN--
gi|769978537|ref|WP_045094522.1|damageinducibleprotein[Legionellafallonii]_gi|698841736|emb|CEG55682.1|Putativeproteinwithboxh/        -DIDSFFTASGKHFRTR--DGKRH----GFGQ-RVIIST--T--D-KWSKNAEEALKD--
gi|553752541|ref|WP_023085344.1|hypotheticalprotein[Pseudomonasaeruginosa]_gi|552201952|gb|ERU62653.1|hypotheticalproteinQ088_0        -DIDSFFTASGKRFATS--AGER-----FFSH-RLIIST--T--D-KWSSNAEKALEN--
gi|880980418|ref|WP_048704461.1|damageinducibleprotein[Thauerahumireducens]_gi|875132785|gb|KMU70944.1|damageinducibleprotein/1        -DIDSFFTASGKRFATS--QGER-----SFAH-RVIVST--T--D-KWSSNAEKALEN--
gi|740135732|ref|WP_037982815.1|damageinducibleprotein[Thauerasp.27]/11603        -DIDSFFTASGKRFTTN--QGER-----SFAH-RVIVST--T--D-KWSSNAEKALEN--
gi|738017371|ref|WP_035978332.1|damageinducibleprotein[Burkholderiaglumae]/11380        -DIDSFFTASGKRFATT--EGER-----GFSE-RVIVAT--T--D-KWSKHAEDALSD--
gi|498506003|ref|WP_010806671.1|typeIIIrestrictionenzyme,ressubunit[Pandoraeasp.SD62]_gi|490314561|gb|EON12879.1|typeIIIrestri/        -DIDSFFTASGKRFATT--DGER-----GFSE-RLIVAT--T--D-KWSKHAEDALSD--
gi|500117748|ref|WP_011793753.1|DNAdamageinducibleprotein[Acidovoraxcitrulli]_gi|120587742|gb|ABM31182.1|typeIIIrestrictionenz/        -DIDSFFTASGKKFATK--EGER-----SFAH-RIVVST--T--D-KWSKHADDALAN--
gi|651617328|ref|WP_026611444.1|damageinducibleprotein[Methylocaldumszegediense]/11601        -DIDSFFTASGKRFATK--EGER-----GFSH-RIVVST--T--D-KWSKHAEDALAN--
gi|737210208|ref|WP_035194994.1|damageinducibleprotein[Acidithiobacillusferrivorans]_gi|669953220|emb|CDQ11755.1|Adeninespecif/        -DLGTFFMLSGR-----------E----PFTS-RLIVST--T--D-HWGKNADEMLDT--
gi|935697386|ref|WP_054506180.1|damageinducibleprotein[Achromobactersp.]_gi|928716153|emb|CUK12836.1|Hefnuclease[Achromobacter/        -DLGNFFTLSGK-----------G----GFTQ-RMVIAT--A--S--LSKHAAAALDN--
gi|429502517|gb|ELA00827.1|adeninespecificDNAmethyltransferase[Cupriavidussp.HMR1]/11599        -DLGNFFTLSGK-----------G----GFTQ-RMVIAT--A--S--LSKHAAAALDN--
gi|506356642|ref|WP_015876361.1|DNAdamageinducibleprotein[Burkholderiaglumae]_gi|237877158|gb|ACR29491.1|AdeninespecificDNAmet/        -DLGNFFTLSGK-----------G----GFTE-RLIIAT--A--P--LSKHAADAMEN--
gi|696406787|ref|WP_032978003.1|damageinducibleprotein[Bordetellahinzii]_gi|625335076|gb|KCB49343.1|typeIIIrestrictionenzyme,r/        -DLGNFFTLSGK-----------G----GFTQ-RLIVAT--A--P--LSKHAADALEG--
gi|817529125|gb|KKO72587.1|damageinducibleprotein[Kerstersiagyiorum]/11592        -DLGNFFTLSGK-----------G----GFTQ-RLIVAT--A--P--LSKHAADALEG--
gi|737048910|ref|WP_035044118.1|damageinducibleprotein[Aquabacteriumsp.NJ1]_gi|699052896|gb|KGM38706.1|damageinducibleprotein/1        -DLGNFFTLSGK-----------G----GFTQ-RLIVAT--A--N--LSKHAADALEN--
gi|822492519|ref|WP_046933319.1|MULTISPECIES:damageinducibleprotein[Xanthomonas]_gi|821703525|gb|KLA61196.1|damageinduciblepr/1        -DLGNFFTLSGK-----------G----GFTQ-RLIVAT--A--N--LSKHAADALEN--
gi|751252861|ref|WP_040963740.1|damageinducibleprotein[Xanthomonasfuscans]_gi|749808363|gb|KIJ03887.1|damageinducibleprotein[/1        -DLGNFFTLSGK-----------G----GFTQ-RLIVAT--A--N--LSKHAADALEN--
gi|496774980|ref|WP_009368792.1|DNAdamageinducibleprotein[Bilophilasp.4_1_30]_gi|345039902|gb|EGW44205.1|hypotheticalproteinHM/        -DLGNFFTLSGK-----------G----GFSQ-RIIVAT--A--P--LTKHAADALEG--
gi|499241912|ref|WP_010939452.1|DNAdamageinducibleprotein[Desulfovibriovulgaris]_gi|46580580|ref|YP_011388.1|adeninespecificDN/        -DLGTFFTLSGK-----------G----GFSQ-RLIVAT--A--P--LSKNAADAIEH--
gi|504274913|ref|WP_014462015.1|DNAdamageinducibleprotein[Bacillusmegaterium]_gi|345446919|gb|AEN91936.1|AdeninespecificDNAmet/        -DIDSFFTASGK-----------N----IYSS-RIIVST--T--D-EWSKNAEEALED--
gi|504895966|ref|WP_015083068.1|typeIIrestrictionenzymeandmethyltransferasesubtypeG[Anabaenasp.90]_gi|413972424|gb|AFW96512.1|t        -DIDSFFTASGT-----------N----LFKQ-RMIIST--T--A-KWSKHALAALKD--
gi|518327765|ref|WP_019497972.1|hypotheticalprotein[Calothrixsp.PCC7103]/11600        -DIDSFFTASGK-----------A----PFQK-RIIVST--T--D-RWGKNAEDAIKN--
gi|652765503|ref|WP_027079917.1|damageinducibleprotein[Luteimonasmephitis]/11593        -DIDSFFTASGK-----------A----GFSS-RIVVST--T--D-RWSKHAEDALTD--
gi|896539951|ref|WP_049442123.1|damageinducibleprotein[Stenotrophomonasmaltophilia]/11591        -DIDSFFTASGK-----------S----PFAS-RLIVST--T--D-KWSKHAEDALEA--
gi|500217900|ref|WP_011888043.1|DNAdamageinducibleprotein[Geobacillusthermodenitrificans]_gi|134267954|gb|ABO68149.1|Conserved/        -DLDSFFTESGK-----------H----PFRK-RMIVVT-----A-PMSKHAYEACHD--
```

```
gi|696570298|ref|WP_033102014.1|damageinducibleprotein[Thermoactinomycesdaqus]/11594    -DLDSFFTESGK-----------H----PFRK-RMIIAT-----A-PMSKHAEDACQN--
gi|251772067|gb|EES52637.1|putativehelicase[Leptospirillumferrodiazotrophum]/11618    -DIDSFFTLSGK-----------E----PFTS-RLIVST--T--N-KWSVHAEEALKN--
gi|738124101|ref|WP_036082108.1|damageinducibleprotein[Leptospirillumferriphilum]_gi|685243306|gb|KGA94177.1|putativehelicase[/    -DIDSFFTLSGK-----------K----PFTS-RLIVSS--T--D-RWGVHAEEALKN--
gi|558699223|ref|WP_023524766.1|helicase[Leptospirillumsp.GroupII'CF1']_gi|206603764|gb|EDZ40244.1|Putativehelicase[Leptospiri/    -DIDSFFTLSGK-----------E----PFTS-RLIVST--T--D-KWSVHAEEALRN--
gi|406774462|gb|AFS53887.1|putativehelicase[LeptospirillumferriphilumML04]/11610    -DIDSFFTLSGK-----------K----PFSA-RLIVST--T--D-KWSVHAEEALAD--
gi|740719463|ref|WP_038504749.1|damageinducibleprotein[Leptospirillumferriphilum]_gi|640838125|gb|AIA30173.1|damageinduciblep/1    -DIDSFFTLSGK-----------K----PFSA-RLIVST--T--D-KWSVHAEEALAD--
gi|517810766|ref|WP_018980974.1|hypotheticalprotein[Saccharibacterfloricola]/11733    -EIDSFISASPL---------RES----SFSD-RLLITT-----A-PLGDNAQKTAYE--
gi|502471675|ref|WP_012798533.1|helicase[Slackiaheliotrinireducens]_gi|256791761|gb|ACV22431.1|predictedhelicase[Slackiaheliotr    -TVSTFFGAQGNN----------D----TYAH-NMLIST--T--E-NLTPNLDKVLTD--
gi|657871713|ref|WP_029576297.1|hypotheticalprotein[Bifidobacteriumthermacidophilum]_gi|673007521|gb|KFJ02535.1|helicase[Bifido    -DVSTFLAKAHMD----------K----RYQH-YMLADS--A--P-ALSGQLEKLIGE--
gi|673004757|gb|KFI99791.1|DNAhelicaserestrictionenzymetypeIIIRessubunit[Bifidobacteriumthermacidophilumsubsp.porcinum]/11682    -DVSTFLAKAHMD----------K----RYQH-YMLADS--A--P-ALSGQLKKLIGE--
gi|917316092|ref|WP_051922804.1|hypotheticalprotein[Bifidobacteriumstellenboschense]_gi|673003774|gb|KFI98812.1|helicase[Bifido    -DVSTFFMNALAD----------E----RYQH-YMIADT--T--S-GYTSVLEEFMLS--
gi|705415694|ref|WP_033502332.1|hypotheticalprotein[Bifidobacteriumpseudolongum]_gi|672982411|gb|KFI77685.1|DNAdamageinducible/    -DVSTFFMNALAD----------R----HYGH-YMIADT--A--P-SVTKTFENYMND--
gi|916591418|ref|WP_051198509.1|hypotheticalprotein[Bifidobacteriumsp.AGR2158]/11704    -KLASFFMNATDN----------P----LYGH-FMIADT--A--S-GYTSTLESFITQ--
gi|772242982|gb|AJW78255.1|hypotheticalproteinVO01_03165[Clavibactermichiganensissubsp.insidiosus]/11642    -DLDSFFEAVGK-----------E----PFTE-GLVIDT--T-VG-AWSTHAEEALRN--
gi|916649818|ref|WP_051256909.1|damageinducibleprotein[Actinomycesgraevenitzii]/11677    -DIDSFLTASGK-----------A----PFTR-RILVDT--S-GT-AWGKNSQDAIEG--
gi|496504578|ref|WP_009212861.1|helicase[Actinomycessp.oraltaxon180]_gi|315312695|gb|EFU60776.1|helicase[Actinomycessp.oraltaxo    -DIDSFLSASGK-----------E----PFGR-RIVVDT--S-GA-PWGKNAQDAIEG--
gi|494862206|ref|WP_007588306.1|helicase[Actinomycessp.ICM39]_gi|398362799|gb|EJN46470.1|typeIIIrestrictionenzyme,ressubunit[Ac    -DIDSFLSASGK-----------E----PFGR-RIVVDT--S-GA-PWGKNAQDAIEG--
gi|881057111|ref|WP_048774974.1|damageinducibleprotein[Sanguibacterkeddieii]/11697    -DIDSFLSASGK-----------E----PFGR-RIVVDT--S-GA-PWGKNAQDAIEG--
gi|881020459|ref|WP_048740808.1|damageinducibleprotein[Kytococcussedentarius]/11704    -DIDSFLSASGK-----------E----PFGR-RIVVDT--S-GA-PWGKNAQDAIEG--
gi|881052313|ref|WP_048770555.1|damageinducibleprotein[Cellulomonasflavigena]/11705    -DIDSFLSASGK-----------E----PFGR-RIVVDT--S-GA-PWGKNAQDAIEG--
gi|490108682|ref|WP_004009424.1|helicase[Mobiluncuscurtisii]_gi|315490465|gb|EFU80089.1|helicaseCterminaldomainprotein[Mobilun/    -QLDSFLSASGK-----------E----PFKR-RIFVET--T-GV-PWGSNAEDAIRN--
gi|490111935|ref|WP_004012603.1|helicase[Mobiluncusmulieris]_gi|227843812|gb|EEJ53982.1|helicaseCterminaldomainprotein[Mobilun/    -HLDSFLSESGK-----------E----PFKR-RIFVET--T-GV-AWSQNAEAAIQG--
gi|490114228|ref|WP_004014848.1|helicase[Mobiluncusmulieris]_gi|269934432|gb|EEZ90993.1|helicaseCterminaldomainprotein[Mobilun/    -HLDSFLSESGK-----------E----PFKR-RIFVET--T-GV-AWSQNAEAAIQG--
gi|493333551|ref|WP_006290636.1|helicase[Parascardoviadenticolens]_gi|315119487|gb|EFT82620.1|helicaseCterminaldomainprotein[P/    -ELSTFIASVATD----------S----RYTG-CMLLSTATNISN-QLAENFMRMRKN--
gi|495817067|ref|WP_008541646.1|helicase,partial[Sutterellaparvirubra]_gi|378598583|gb|EHY31712.1|helicaseprotein,partial[Sutte    -EIDSFLAASSK-----------A----HFTH-RLLVAT--N--E-KWSENVVKELHA--
gi|491572696|ref|WP_005430278.1|helicase[Sutterellawadsworthensis]_gi|319805116|gb|EFW01943.1|DNAhelicaserestrictionenzymeTypeI    -GVDSFIAASNR-----------D----YFKQ-RYIITT--N--E-NWSENALTEMQN--
gi|748686321|ref|WP_039944445.1|hypotheticalprotein[Thermicanusaegyptius]/11573    -DIKTFLAVSGR-----------K----EFSR-RMIVTA-----S-PLGQNAEKAIKG--
gi|916875698|ref|WP_051482754.1|hypotheticalprotein[Thioalkalivibriosp.HK1]/11366    -ALDSFLAASNR-----------A----PFTS-RMVIDT--G--D-EWGAMARKKIEG--
gi|821248604|gb|KKZ10960.1|hypotheticalproteinTE42_09075[CandidatusSynechococcusspongiarumSP3]/11598    -EIDSFLAASGR-----------P----EFRR-RIWMDT--T-GR-DWSVKAEETLRQ--
gi|737409843|ref|WP_035390956.1|hypotheticalprotein[Ferrimicrobiumacidiphilum]_gi|765544266|gb|KJE76241.1|typeIrestrictionenzym    -AIDSFLSASSR-----------E----PFTS-RLIIAT--T--D-HWNTNATNTLKD--
gi|654906925|ref|WP_028358281.1|damageinducibleprotein[Brackiellaoedipodis]/11679    -DIDSFMTASGK-----------K----PFSH-RLIITT--T--D-EWSDNAIDALDG--
gi|502809901|ref|WP_013044877.1|hypotheticalprotein[CandidatusPuniceispirillummarinum]_gi|292663146|gb|ADE38247.1|hypotheticalp    -DLGTFFTQSGK-----------D----AFSS-RLIITT--T--D-RWTKHAETAIRH--
gi|251771370|gb|EES51950.1|typeIIIrestrictionenzyme,ressubunit[Leptospirillumferrodiazotrophum]/11628    -ELGNFYSLSGKAYPGT----TRP----IFSS-RIVVST--T--E-KWSPHAEDLLSD--
gi|498328480|ref|WP_010642636.1|DNAdamageinducibleprotein[Acidithiobacillusthiooxidans]/11627    -AITNFTHDASRKFTVD---GEER----EFSF-KLFVDT--Q--N-DLGRQAEEALND--
gi|647633864|ref|WP_025906698.1|damageinducibleprotein[Prochlorococcussp.scB245a_519B7]/11622    -DLDSFISASST-----------D----DFKR-LVLIDT--S-NK-GLGKNATSVLDN--
gi|647619599|ref|WP_025893729.1|damageinducibleprotein[Prochlorococcussp.scB241_527N11]/11621    -DLDSFISASST-----------N----DFKR-LVLIDT--S-SQ-PIGKNAQIVFDN--
gi|755034887|ref|WP_042387498.1|hypotheticalprotein[Clostridiumsp.CL6]/11606    -DVDTFLATSSKSFNVD---GVKT----SYSF-RFIIST--T--D-KYSKNAEDVLID--
gi|498139798|ref|WP_010453954.1|helicase[SuccinivibrionaceaebacteriumWG1]/11662    -DVDTFIATSNRLFTVN---GQKV----GFNK-LFWVQSNPK--N-PWTSVAFDSIKN--
gi|527501386|gb|EPT34571.1|typeIIIrestrictionenzyme,ressubunit[Bacteroidetesbacteriumoraltaxon272str.F0290]/11789    -KVDSFISQATRTFTHP-DTKEKI----SFSE-MIWVST--T--S-KWNPHAEQKIQN--
gi|496047275|ref|WP_008771782.1|helicase[Bacteroidessp.2_1_33B]_gi|262296443|gb|EEY84373.1|helicaseCterminaldomainprotein[Bact/    -VVDSFISTAHRAFIDD-LTLKTT----YFSN-LIWVST--T--L-RWGANAEETLKG--
PDI8503III/11664    -VVDSFISTAHRAFIDD-LTLKTT----YFSN-LIWVST--T--L-RWGANAEETLKG--
gi|738719431|ref|WP_036613715.1|helicase[Parabacteroidesdistasonis]_gi|649542518|gb|KDS49387.1|restrictionendonucleasefamilypro    -VVDSFISTAHRAFIDD-LTLKTT----YFSN-LIWVST--T--L-RWGANAEETLKG--
gi|659417033|gb|KEJ86133.1|hypotheticalproteinHMPREF1002_00341[Porphyromonassp.31_2]/11656    -VVDSFISTAHRAFIDD-LTLKTT----YFSN-LIWVST--T--L-RWGANAEETLKG--
gi|567456807|gb|ETK11358.1|hypotheticalproteinT235_15845[Tannerellasp.oraltaxonBU063isolateCell8/11]/11433    -SVDSFLATSSRTFNH-----HQT----AFAH-RLWIAT--T--N-HWGSNAEDAIQN--
gi|545440279|ref|WP_021677877.1|helicase[Porphyromonasgingivalis]_gi|543985765|gb|ERJ89108.1|helicaseprotein[Porphyromonasgingi    -SVDSFLATSSRTFANE-ITFAQT----SFAH-RLWIAT--T--N-HWGPNAEEAIQN--
gi|497438056|ref|WP_009752254.1|helicase[Prevotellasp.oraltaxon473]_gi|429144866|gb|EKX87968.1|helicaseprotein[Prevotellasp.ora    -SVDSFLATSSRTFNNE-VTFQTT----SFAH-RLWIST--T--N-HWGPNAEEAIQN--
gi|490423474|ref|WP_004295683.1|MULTISPECIES:helicase[Bacteroides]_gi|156110098|gb|EDO11843.1|helicaseCterminaldomainprotein[B/    -AVDSFLATSSRQFKDP-ETLQTT----SFAK-RMWIST--T--N-HWGKNAEDAIQN--
gi|941777229|ref|WP_055173506.1|helicase[Bacteroidescaccae]_gi|933263395|emb|CUQ00567.1|Predictedhelicase[Bacteroidescaccae]/1/    -AVDSFLATSSRTFTNE-VTFQTV----RFSN-RIWIST--T--N-HWGTNAEEAIRN--
gi|492413898|ref|WP_005836892.1|helicase[Bacteroidesuniformis]_gi|392678824|gb|EIY72229.1|hypotheticalproteinHMPREF1072_03583[B    -AVDSFLATSSRTFTNE-VTFQTV----RFSN-RIWIST--T--N-HWGTNAEEAIRN--
gi|695455095|ref|WP_032566191.1|helicase[Bacteroidesfragilis]_gi|596095426|gb|EXZ59996.1|restrictionendonucleasefamilyprotein[B    -AVDSFLATSSRTFTNE-VTFQTT----RFSN-RIWIST--T--N-HWGANAEEAIRN--
gi|933227941|emb|CUQ02348.1|Predictedhelicase[Bacteroidesuniformis]/11658    -AVDSFLATSSRTFTNE-VTFQTT----RFSN-RIWIST--T--N-HWGTNAEEAIRN--
gi|494823013|ref|WP_007558421.1|helicase[Bacteroidesplebeius]_gi|198272167|gb|EDY96436.1|helicaseCterminaldomainprotein[Bacter/    -AVDSFLATSSRTFTNE-VTFQTV----RFSN-RIWIST--T--N-HWGTNAEEAIRN--
gi|495427212|ref|WP_008151909.1|helicase[Parabacteroidesjohnsonii]_gi|218222381|gb|EEC95031.1|helicaseCterminaldomainprotein[P/    -AVDSFLATSSRTFTNE-VTFQTT----RFAK-RIWIST--T--N-HWGSNAEEAIRH--
gi|933196855|emb|CUO94545.1|typeIrestrictionenzymeEcoKIsubunitR[Bacteroidesuniformis]_gi|933220098|emb|CUN86874.1|typeIrestrict    -AVDSFLATSSRTFTNE-VTFQTT----RFSN-RVWIST--T--S-HWGSNAEEAIRN--
gi|823687748|ref|WP_047101121.1|helicase[Brachyspirahyodysenteriae]_gi|823334537|gb|KLI17932.1|helicase[Brachyspirahyodysenteri    -GLDSFLATSSKTFNNG------I----KFSH-RLWVES--N--G-KWNSKAQEAIKN--
gi|496436691|ref|WP_009145538.1|helicase[Phascolarctobacteriumsuccinatutens]_gi|322414007|gb|EFY04839.1|helicaseCterminaldomai/    -AVDSFLSTSSRQFVDD--NFEQQ----SFSS-RLWFAT--N--N-KWGKNAKEAFEN--
gi|503372880|ref|WP_013607541.1|helicase[Sphaerochaetaglobosa]_gi|324026933|gb|ADY13692.1|typeIIIrestrictionproteinressubunit[S    -QVDTFISTSGKSFEDTEEIGKIV----HFSH-RLWIDT--T-SR-GFNPEAEQTVHN--
gi|505821748|ref|WP_015707145.1|helicase[Treponemaprimitia]_gi|299483503|gb|ADJ19584.1|helicasedomaincontainingprotein[Trepone/    -MVDTFLSTSGKSFYDVLEPGKKV----RFSC-RLWLDT--T-IA-GFNPEAENTIKG--
gi|505822741|ref|WP_015708134.1|helicase[Treponemaprimitia]_gi|333739639|gb|AEF85129.1|endonucleaseandmethylaseLlaGI[Treponemap    -MVDTFLSTSGKSFYDVLEPGKKV----RFSC-RLWLDT--T-IA-GFNPEAENVIKG--
gi|488791994|ref|WP_002704400.1|helicase[Treponemasaccharophilum]_gi|380767691|gb|EIC01689.1|typeIIIrestrictionproteinressubuni    -TVSTFITTSQFPFSVG---GENK----NFSH-RIWIDT--T-IN-GFNSEAQNAIDK--
gi|524671454|emb|CDD81750.1|putativeseptumsitedeterminingproteinMinC[Bacteroidessp.CAG:462]/11642    -DVDTFLSTSGKTFEVE---GVEH----AFAQ-RLWIST--T--N-KWNSVAELTIKK--
gi|524573865|emb|CDC95766.1|putativeseptumsitedeterminingproteinMinC[Alistipessp.CAG:268]/11639    -DVDTFLSTSGKRFETE---SGMT----GFVQ-RLWIST--T--N-KWNPTAEQTIRN--
gi|658617970|ref|WP_029759328.1|helicase[Fusobacteriumnucleatum]/11606    -DVDTFISTSSKLFEIN---GETK----KFSG-RIFIST--T--N-KWSKKASDLIEN--
gi|494412037|ref|WP_007216782.1|helicase[Bacteroidescellulosilyticus]_gi|392638921|gb|EIY32752.1|hypotheticalproteinHMPREF1062_    -AVDTFLSTSNKTFLNE--AGETA----CFTH-RLWIDT--T-EG-GFNSEALKTIQN--
gi|383107102|gb|AFG37435.1|putativehelicase[SpirochaetaafricanaDSM8902]/11616    -EVDSFLSSSSRQFKDE--NFQTT----GFTN-RLWIST--T--N-KWNRNAEQAIAN--
SAF8902III/11616    -EVDSFLSSSSRQFKDE--NFQTT----GFTN-RLWIST--T--N-KWNRNAEQAIAN--
gi|652538894|ref|WP_026932796.1|helicase[Gramellaechinicola]/11633    -AVDSFLSTSSRSFKNE--SFLTT----EFSN-RLWVST--T--N-KWGVNAEETIKN--
```

```
gi|639238223|ref|WP_024568839.1|helicase[Elizabethkingiaanophelis]/11628    -AVDSFLSTSSKTFKNE--QLQTV----GFSQ-RLWVST--T--N-KWGSNAEETIKN--
gi|652520381|ref|WP_026914582.1|helicase[Gramellaportivictoriae]/11623    -EVDSFLSTSSRHFKNE--KFESI----GFSR-RLWVST--T--N-NWSNNAEETLRN--
gi|494922183|ref|WP_007648219.1|Helicase[Formosasp.AK20]_gi|469825489|gb|EMQ95550.1|Helicasedomainprotein[Formosasp.AK20]/11626    -EVDSFLSTSSRDFKND--QLQTI----SFSK-RIWVST--T--N-NWSSNAEETIKN--
gi|522083382|ref|WP_020594591.1|hypotheticalprotein[Spirosomapanaciterrae]/11637    -AVDTFLSTSARSFKGE--DLKTH----HFAQ-RLWIDT--T-GK-PFGTNAAEATKN--
gi|490455352|ref|WP_004326157.1|helicase[Bacteroidesovatus]_gi|392669277|gb|EIY62768.1|hypotheticalproteinHMPREF1070_03635[Bact    -AVDSFLSTSSRSFTDE--SGNTS----TFAH-RLWIST--T--N-NWGINAEEAIRN--
gi|499516035|ref|WP_011202675.1|helicase[Bacteroidesfragilis]_gi|53713270|ref|YP_099262.1|helicasedomainprotein[Bacteroidesfrag    -AVDSFLSTSSRSFTDE--SGNTS----TFAH-RLWIST--T--N-NWGINAEEAIRN--
gi|651338332|ref|WP_026461320.1|helicase[Adhaeribacteraquaticus]/11620    -AVDSFLSTSSREFKNE--NLQTT----RFTQ-RLWIDT--T-GK-KWGSNAEEAIRN--
gi|806964642|gb|AKC17139.1|helicase[Flavobacteriumpsychrophilum]/11639    -AVDSFLATAGRSFKDENQNFKLT----HFSN-CLWVST--T--N-KWGTNAEEAIKN--
gi|752684374|ref|WP_041332322.1|helicase[Flavobacteriumpsychrophilum]_gi|666882889|gb|AIG30715.1|helicase[Flavobacteriumpsychro    -AVDSFLATAGRSFKDENQNFKLT----HFSN-CLWVST--T--N-KWGTNAEEAIKN--
gi|933196595|emb|CUP04894.1|DNAorRNAhelicasesofsuperfamilyII[Bacteroidesuniformis]/11631    -AVDSFLSTSSREFRNE--DIKTT----RFSH-RLWIST--T--N-KWGTNAEEAIRN--
gi|670509109|ref|WP_031456918.1|helicase[Flavobacteriumchungangense]/11625    -AVDSFLSTSSRTFKNE--DLKIV----GFSH-RLWIST--T--N-KWGPNATQAIKN--
  :  :

gi|490190857|ref|WP_004089451.1|hypotheticalprotein[Helicobacterbilis]_gi|476630638|gb|EMZ37196.1|hypotheticalproteinC826_02193    ---K-KIPIELITLQDFL-QSSIDWDKFDPTKN------K---LEIQ-AKKEPKP----H
gi|488896133|ref|WP_002807242.1|MULTISPECIES:restrictionendonucleaseEco57I[Campylobacter]_gi|380541096|gb|EIA65377.1|hypothetic    ---K-NIPIELITLEDFL-NSNISWDKFDPNNS-------L-ELPII-AKKKPRE----H
gi|746602240|ref|WP_039628365.1|restrictionendonucleaseEco57I[Campylobacterlari]_gi|744819395|gb|AJD03391.1|putativehelicase[Ca    ------TPIELITLEDFL-NSNISWDKFDPNNS-------L-ELPIT-AKKKPRE----H
gi|727317135|ref|WP_033772558.1|restrictionendonucleaseEco57I[Helicobacterpylori]/11622    STGM-GIDIDEINEEDFI-YSRIDWEKFDPTQT------QG-ELPLC-DKKRPRP----H
gi|727103490|ref|WP_033618954.1|restrictionendonucleaseEco57I[Helicobacterpylori]/11616    STGM-GIDIDEITEEDFI-YSRIDWEKFDPTKT------ED-EIPLC-DKKRSRP----H
gi|727340205|ref|WP_033794408.1|restrictionendonucleaseEco57I[Helicobacterpylori]/11609    STGM-GI--DEITEEDFI-HSGIDGEKFDPAQT------QG-ELPLC-DKKRQCP----H
gi|727292407|ref|WP_033749975.1|restrictionendonucleaseEco57I[Helicobacterpylori]/11611    STGM-GI--DEITEEDFI-HSGIDWEKFDPTQT------QG-ELPLC-DKKRPRP----H
gi|727289795|ref|WP_033747591.1|restrictionendonucleaseEco57I[Helicobacterpylori]/11612    SAGM-GIDIDEITEEDFI-YSRIDWEKFDPTKT------ED-EIPLC-DKKRPRP----H
gi|727319676|ref|WP_033775047.1|restrictionendonucleaseEco57I[Helicobacterpylori]/11612    STGM-GIDIDEITEEDFI-YSRIDWEKFDPTKT------ED-EIPLC-DKKRSRP----H
gi|447212910|ref|WP_001290166.1|restrictionendonucleaseEco57I[Helicobacterpylori]_gi|308063540|gb|ADO05427.1|hypotheticalprotei    K-SK-GIDIVEITEEDFI-YSQIDWEKFDPMQT------QG-ELPLC-DKKKPRP----H
gi|447212901|ref|WP_001290157.1|restrictionendonucleaseEco57I[Helicobacterpylori]_gi|384557329|gb|AFH97797.1|hypotheticalprotei    R-SK-GIDIVEITEEDFI-YSQIDWEKFDPMQT------QG-ELPLC-DKKKPRP----H
gi|515849671|ref|WP_017280424.1|hypotheticalprotein[Helicobacterpylori]/11615    K-SK-GIDIVEISEEDFI-HSQIDWEKFDPTQT------QG-EIPLC-DKKKPRP----H
gi|447212900|ref|WP_001290156.1|restrictionendonucleaseEco57I[Helicobacterpylori]_gi|317178970|dbj|BAJ56758.1|TypeIIGrestrictio    K-SK-GIDIVEISEEDFI-YSQIDWEKFDPTQT------QG-EIPLC-DKKKPRP----H
gi|447079018|ref|WP_001156274.1|hypotheticalprotein[Helicobacterpylori]/11614    STGM-GIDIDEITEEDFI-YSRIDWEKFDPTKT------ED-ELPLC-DKKKPCS----H
gi|447212800|ref|WP_001290056.1|hypotheticalprotein[Helicobacterpylori]_gi|532153248|gb|EQL67316.1|restrictionendonucleaseEco57    SNGM-GIDIDEITEEDFI-YSQIDWEKLDPTKT------QD-ELPLC-DKKKPRP----H
gi|505457237|ref|WP_015644339.1|hypotheticalprotein[Helicobacterpylori]_gi|499061160|gb|AGL70074.1|restrictionendonucleaseEco57    SNGM-GIDIDEITEEDFI-YSQIDWEKLDTT--------QS-ELPLC-DKKKPRS----H
HPYUM037X/11591    SNGM-GIDIDEITEEDFI-YSQIDWEKLDTT--------QS-ELPLC-DKKKPRS----H
gi|920026297|ref|WP_052937176.1|restrictionendonuclease[Helicobacterpylori]_gi|912528678|gb|KNX48238.1|restrictionendonucleaseE    SNGM-GIDIDEITEEDFI-YSQIDWEKFDSTKT------QD-ELPLC-DKKKPRP----H
gi|915539050|ref|WP_050841327.1|restrictionendonuclease[Helicobacterpylori]_gi|908890887|gb|KNE06183.1|restrictionendonucleaseE    SIGM-DIDIDIISEEDFI-YSRIDWEKFDPMQT------QG-ELPLC-DKKKPRT----H
gi|727101397|ref|WP_033616957.1|restrictionendonucleaseEco57I[Helicobacterpylori]/11592    STGM-DIDIDIITEEDFI-YSRIDWEKFDPTKT------QD-ELPLC-DKKKPRS----H
gi|562814875|emb|CDK13002.1|TypeISPRMsystem[Helicobacterpylori26695]/11588    STGM-GIDIDEITEEDFI-YSRIDWEKFDPTKT------QD-EIPLC-DKKKPRS----H
HPYAXVIII/11588    STGM-GIDIDEITEEDFI-YSRIDWEKFDPTKT------QD-EIPLC-DKKKPRS----H
gi|447079029|ref|WP_001156285.1|restrictionendonucleaseEco57I[Helicobacterpylori]_gi|393085956|gb|EJB86635.1|hypotheticalprotei    STGK-DIDIDIISEEDFI-YSRIDWEKFDPTKT------ED-ELPLC-DKKSPRP----H
gi|489105953|ref|WP_003015814.1|restrictionendonuclease[Helicobacterpylori]_gi|477503388|gb|ENH59950.1|restrictionendonucleasef    STGM-DIDIDIISEEDFI-YSQIDWEKFDPTKT------ED-ELPLC-DKKKPRP----H
gi|447079006|ref|WP_001156262.1|restrictionendonucleaseEco57I[Helicobacterpylori]_gi|393067470|gb|EJB68280.1|hypotheticalprotei    STGM-GIDIDEITEEDFI-YSRIDWEKFDPMKT------QD-ELPLC-DKKKPRS----H
gi|727314589|ref|WP_033770298.1|restrictionendonucleaseEco57I[Helicobacterpylori]/11586    STGM-GIDIDEITEEDFI-YSRIDWEKLDPTKT------QD-EIPLC-DKKKPRS----H
gi|447079019|ref|WP_001156275.1|restrictionendonucleaseEco57I[Helicobacterpylori]_gi|308065028|gb|ADO06920.1|hypotheticalprotei    STGM-GIDIDEITEEDFI-YSRIDWEKFDPTKT------ED-EIPLC-DKKKPRP----H
gi|447079028|ref|WP_001156284.1|restrictionendonucleaseEco57I[Helicobacterpylori]_gi|393076030|gb|EJB76784.1|hypotheticalprotei    STGM-GIDIDEITEEDFI-YSRIDWEKFDPTKT------ED-EIPLC-DKKRPCP----H
gi|393034009|gb|EJB35070.1|hypotheticalproteinHPNQ4053_0631[HelicobacterpyloriNQ4053]/11514    STGM-GIDIDEITEENFI-YSRIDWEKFDPTKT------ED-EIPLC-DKKRPRP----H
gi|447079004|ref|WP_001156260.1|restrictionendonucleaseEco57I[Helicobacterpylori]_gi|384552418|gb|AFI07366.1|hypotheticalprotei    STGM-GIDIDEITEEDFI-YSRIDWEKFDPTKT------ED-EIPLC-DKKRPRS----H
gi|746011306|ref|WP_039088215.1|restrictionendonucleaseEco57I[Helicobacterpylori]_gi|733488720|gb|KHL85979.1|restrictionendonuc    SIGM-GIDIDEITEEDFI-YSRIDWEKLDPMQT------QG-EIPLC-DKKKPRS----H
gi|531081162|gb|EQD92166.1|restrictionendonucleaseEco57I[HelicobacterpyloriPZ5080]/11509    STGM-GIDIDEITEEDFI-YSRIDWEKFDPTKT------ED-EIPLC-DKKKPHS----H
gi|544760796|ref|WP_021187395.1|restrictionendonucleaseEco57I[Helicobacterpylori]_gi|531086508|gb|EQD96745.1|restrictionendonuc    STGM-GIDIDEITEEDFI-YSRIDWEKFDPTKT------ED-EIPLC-DKKKPRS----H
gi|447079012|ref|WP_001156268.1|restrictionendonucleaseEco57I[Helicobacterpylori]_gi|393028199|gb|EJB29286.1|hypotheticalprotei    STGM-GIDIDEITEEDFI-YSRIDWEKFDPTKT------ED-EIPLC-DKKRPRP----H
gi|746008458|ref|WP_039085555.1|restrictionendonucleaseEco57I[Helicobacterpylori]_gi|733480358|gb|KHL78026.1|restrictionendonuc    STGM-GIDIDEITEEDFI-YSRIDWEKFDPTKT------ED-EIPLC-DKKRPRP----H
gi|447079011|ref|WP_001156267.1|restrictionendonucleaseEco57I[Helicobacterpylori]_gi|393038534|gb|EJB39568.1|hypotheticalprotei    STGM-GIDIDEITEEDFI-YSRIDWEKFDPTKT------ED-EIPLC-DKKKPRP----H
gi|393037163|gb|EJB38199.1|hypotheticalproteinHPNQ4161_0422[HelicobacterpyloriNQ4161]/11556    STGM-GIDIDEITEEDFI-YSRIDWEKFDPTKT------ED-EIPLC-DKKRPRS----H
gi|447079007|ref|WP_001156263.1|restrictionendonucleaseEco57I[Helicobacterpylori]_gi|393051817|gb|EJB52768.1|hypotheticalprotei    STGM-GIDIDEITEENFI-YSRIDWEKLDPTKT------ED-EIPLC-DKKRPRP----H
gi|487867094|ref|WP_001940560.1|helicase[Helicobacterpylori]_gi|459505657|gb|EMG98418.1|helicaseprotein[HelicobacterpyloriGAM21    STGM-GIDIDEITEEDFI-YSRIDWEKFDPTKT------ED-EIPLC-DKKRPRP----H
gi|487872094|ref|WP_001945560.1|helicase[Helicobacterpylori]_gi|459512104|gb|EMH04523.1|helicaseprotein[HelicobacterpyloriGAM24    STGM-GIDIDEITEEDFI-YSRIDWEKFDPTKT------ED-EIPLC-DKKRPRP----H
gi|487880228|ref|WP_001953694.1|helicase[Helicobacterpylori]_gi|459529131|gb|EMH20734.1|helicaseprotein[HelicobacterpyloriGAM26    SIGM-GIDIDEITEEDFI-YSRIDWEKFDPTKT------ED-EIPLC-DKKRPRP----H
gi|487882593|ref|WP_001956059.1|helicase[Helicobacterpylori]_gi|459533462|gb|EMH24859.1|helicaseprotein[HelicobacterpyloriGAM26    STGM-GIDIDEITEEDFI-YSRIDWEKFDPTKT------ED-EIPLC-DKKRPRP----H
gi|487845430|ref|WP_001918896.1|helicase[Helicobacterpylori]_gi|410713871|gb|EKQ71363.1|helicaseprotein[HelicobacterpyloriGAM10    STGM-GIDIDEITEEDFI-YSRIDWEKFDPTKT------ED-EIPLC-DKKRPRP----H
gi|447079005|ref|WP_001156261.1|restrictionendonucleaseEco57I[Helicobacterpylori]_gi|393069830|gb|EJB70625.1|hypotheticalprotei    STGM-GIDIDEITEEDFI-YSRIDWEKFDPTKT------ED-ELPLC-DKKRPRP----H
gi|459506808|gb|EMG99528.1|helicaseprotein[HelicobacterpyloriGAM121Aii]/11545    STGM-GIDIDEITEEDFI-YSRIDWEKFDPTKT------ED-EIPLC-DKKRPRP----H
gi|487825283|ref|WP_001898749.1|restrictionendonucleaseEco57I[Helicobacterpylori]_gi|393107526|gb|EJC08068.1|hypotheticalprotei    SAGM-GIDIDEITEEDFI-YSRIDWEKFDPTKT------ED-EIPLC-DKKRPRP----H
gi|447079008|ref|WP_001156264.1|restrictionendonucleaseEco57I[Helicobacterpylori]_gi|393058636|gb|EJB59524.1|hypotheticalprotei    STGM-GIDIDEITEEDFI-YSRIDWEKFDPTKT------ED-EIPLC-DKKRPRP----H
gi|447079015|ref|WP_001156271.1|restrictionendonucleaseEco57I[Helicobacterpylori]_gi|307637357|gb|ADN79807.1|hypotheticalprotei    STGM-GIDIDEITEEDFI-YSRIDWEKFDPTKT------ED-EIPLC-DKKRPRP----H
gi|487884861|ref|WP_001958327.1|helicase[Helicobacterpylori]_gi|459535535|gb|EMH26834.1|helicaseprotein[HelicobacterpyloriGAM26    STGM-GIDIDEITEEDFI-YSRIDWEKFDPTKT------ED-EIPLC-DKKRPRP----H
gi|487886365|ref|WP_001959831.1|helicase[Helicobacterpylori]_gi|459538727|gb|EMH29861.1|helicaseprotein[HelicobacterpyloriGAM27    STGM-GIDIDEITEEDFI-YSRIDWEKFDPTKT------ED-EIPLC-DKKRPRP----H
gi|545301637|ref|WP_021581412.1|helicase[Helicobacterpylori]_gi|535931889|gb|ERA61745.1|helicaseprotein[HelicobacterpyloriGAM11    STGM-GIDIDEITEEDFI-YSRIDWEKFDPTKT------ED-EIPLC-DKKRSRP----H
gi|447079010|ref|WP_001156266.1|restrictionendonucleaseEco57I[Helicobacterpylori]_gi|393095477|gb|EJB96081.1|hypotheticalprotei    STGM-GIDIDEITEEDFI-YSRIDWEKFDPTKT------ED-EIPLC-DKKRPRP----H
gi|447079009|ref|WP_001156265.1|restrictionendonucleaseEco57I[Helicobacterpylori]_gi|393056294|gb|EJB57206.1|hypotheticalprotei    STGM-GIDIDEITEEDFI-YSRIDWEKFDPTKT------ED-EIPLC-DKKRPRP----H
gi|471386985|gb|EMR58906.1|restrictionendonucleasefamilyprotein[HelicobacterpyloriHpH1]/11504    STGM-GIDIDEITEEDFI-YSRIDWEKFDPTKT------ED-ELPLC-DKKRPRP----H
```

```
gi|487875679|ref|WP_001949145.1|helicase[Helicobacterpylori]_gi|459516928|gb|EMH09091.1|helicaseprotein[HelicobacterpyloriGAM25      STGM-GIDIDEITEEDFI-YSRIDWEKFDPTKT------ED-EIPLC-DKKRPRP----H
gi|496130066|ref|WP_008854573.1|DNAdamageinducibleprotein[Commensalibacterintestini]_gi|353671689|gb|EHD13391.1|helicasedomain/      ---Q-NPPVSKIDLHYLE-QSVIDWSKYQDH--------TK-TILKPQ--KTLRD----H
gi|924898894|ref|WP_053556546.1|damageinducibleprotein[Sphingopyxissp.113P3]_gi|924523996|gb|ALC14732.1|damageinducibleprotei/1      ---Q-AIETQRVGLTELE-ESGIDWSAFAAD--------GT-VRLTDK--KRPRP----D
gi|780802950|gb|KJS08562.1|damageinducibleprotein[Hoefleasp.BRH_c9]/11633                                                           ---Q-NIGTLRIGMSDIE-QSPIDWSAYIRD--------DK-VVLASK--KELRP----H
gi|944671432|gb|KQN22886.1|damageinducibleprotein[Sphingomonassp.Leaf32]/11415                                                      ---Q-SIATTRISMDRLE-QSPIDWQAYLAE--------DR-IVLAPK--KEVRP----H
gi|944612953|gb|KQM64772.1|damageinducibleprotein[Sphingomonassp.Leaf16]/11726                                                      ---Q-SIATTRISMDRLE-QSPIDWQAYLAE--------DR-IVLAPK--KEVRP----H
gi|764353851|ref|WP_044331468.1|damageinducibleprotein[Sphingomonashengshuiensis]_gi|761897338|gb|AJP71707.1|damageinduciblep/1      ---Q-DKPISRIGLDRLE-DSPIDWAAYLLR--------DE-VKLAPP--KEIRP----H
gi|945874472|gb|KQZ27973.1|damageinducibleprotein[Mesorhizobiumsp.Root552]/11651                                                    ---Q-SKPTSRIGLDRLE-ESPIDWSAYLLR--------DE-IKVAAA--KQIRP----H
gi|759408081|ref|WP_043132403.1|damageinducibleprotein[Paracoccussanguinis]_gi|694217086|gb|KGJ14281.1|damageinducibleprotein/1      ---L-SKDWNRISISELE-ASRIDWSQFLRT--------GT-VSLALK--KELRP----H
gi|654342671|ref|WP_027836116.1|damageinducibleprotein[Maritaleamyrionectae]/11632                                                  ---L-SKDWNRIGIDELE-DSRIDWSQFLRT--------GN-ISLEPK--KQLRD----H
gi|126717687|gb|EBA14409.1|hypotheticalproteinRSK20926_02032[Roseobactersp.SK20926]/11603                                           ---L-SKEWNRIGINELE-ASRIDWSQFVRT--------GT-ISLAAK--KTLRD----H
gi|765301116|ref|WP_044628780.1|damageinducibleprotein[Halomonasmeridiana]_gi|764089679|gb|KJD19875.1|damageinducibleprotein[/1      ---L-SKDWNRISLEELE-ASRIDWSQFIRT--------GN-ISLAPK--KELKE----H
gi|518053006|ref|WP_019223214.1|DNAdamageinducibleprotein[Bartonellarattaustraliani]/11654                                          ---Q-KVPIQRINLFDLE-NSQIDWGMFANK--------GQ-ATLKKREVKQLRD----H
gi|493637806|ref|WP_006589578.1|DNAdamageinducibleprotein[Bartonellabirtlesii]_gi|395410345|gb|EJF76900.1|hypotheticalproteinM/      ---Q-AIPVYRIDLRHME-NSRIDWKIFATK--------KE-IVLKSI--KEPRP----H
gi|501188603|ref|WP_012231622.1|DNAdamageinducibleprotein[Bartonellatribocorum]_gi|161017843|emb|CAK01401.1|helicase/methyltra/      ---Q-AIPIYRIDLRHME-NSRIDWQIFATK--------KE-VVLKST--KKPRP----H
gi|651388616|ref|WP_026500615.1|damageinducibleprotein[Bartonellagrahamii]/11655                                                    ---Q-AIPVYRIDLRHME-NSRIDWQIYATK--------KE-VLLKST--KEPRP----H
gi|501188669|ref|WP_012231687.1|DNAdamageinducibleprotein[Bartonellatribocorum]_gi|161017922|emb|CAK01480.1|helicase/methyltra/      ---Q-AIPVYRIDLRHME-NSRIDWQIYATK--------KE-VLLKST--KKPRP----H
gi|518048832|ref|WP_019219040.1|DNAdamageinducibleprotein[Bartonellaflorencae]/11655                                                ---Q-AIPVYRIDLRHME-NSRIDWQIFATK--------KE-LVLKST--KEPRP----H
gi|518053617|ref|WP_019223825.1|DNAdamageinducibleprotein[Bartonellarattaustraliani]/11607                                          ---Q-KVPIQRINLFDLE-NSQIDWGAYKEQ--------GQ-AVLKEKPKKKLLD----H
gi|506336449|ref|WP_015856168.1|DNAdamageinducibleprotein[Bartonellagrahamii]_gi|240267492|gb|ACS51080.1|helicase/methyltransf/      ---Q-AIPVYRIDLRHME-NSRIDWQIFATK--------KE-IVLKAT--KKPRP----H
gi|494588832|ref|WP_007347427.1|DNAdamageinducibleprotein[Bartonellarattimassiliensis]_gi|395419053|gb|EJF85361.1|hypothetical/      ---Q-EVRIQQINLFDLE-NSQIDWDAYKEQ--------GQ-AVLKERPKKKLLD----H
gi|501188650|ref|WP_012231668.1|DNAdamageinducibleprotein[Bartonellatribocorum]_gi|161017902|emb|CAK01460.1|helicase/methyltra/      ---Q-EVRIQRINLFDLE-SSQIDWGAYKEQ--------GQ-AVLKEKPKKKLLD----H
gi|501188624|ref|WP_012231643.1|DNAdamageinducibleprotein[Bartonellatribocorum]_gi|161017866|emb|CAK01424.1|helicase/methyltra/      ---Q-AVPVYRINLFDMD-NSQIDWGIFDKT--------GN-IVLYEQKKKKLLD----H
gi|518053856|ref|WP_019224064.1|DNAdamageinducibleprotein[Bartonellarattaustraliani]/11625                                          ---Q-AVPVYRINLFDMD-HSPIDWGIFDKT--------GN-IVLYEQRKKKLLD----H
gi|501188053|ref|WP_012231081.1|DNAdamageinducibleprotein[Bartonellatribocorum]_gi|161017434|emb|CAK00992.1|helicase/methyltra/      ---Q-AVPVYRINLFDMD-NSQIDWGIFDKT--------GN-IVLYEQKKKKLLD----H
gi|651388630|ref|WP_026500626.1|damageinducibleprotein[Bartonellagrahamii]/11657                                                    ---Q-AIPVYRIDLRHME-NSRIDWQIFATK--------KE-IVLKST--KEPRP----H
gi|518048855|ref|WP_019219063.1|DNAdamageinducibleprotein[Bartonellaflorencae]/11658                                                ---Q-EVRIQRINLFDLE-NSQIDWGAYKEQ--------GQ-AVLKKQPKKKLLD----H
gi|490995101|ref|WP_004856829.1|DNAdamageinducibleprotein[Bartonelladoshiae]_gi|395413167|gb|EJF79645.1|hypotheticalproteinMCS/      ---Q-EVRIQRINLFDLE-NSQIDWGAYKEK--------GK-ATLKEQLKKKLLD----H
gi|491002558|ref|WP_004864279.1|DNAdamageinducibleprotein[Bartonellavinsonii]_gi|395421392|gb|EJF87643.1|hypotheticalproteinME/      ---Q-DVPIQRINLFDLE-NSQIDWSTFEAK--------GD-IVLKEQKKKKLLD----H
gi|491003373|ref|WP_004865094.1|DNAdamageinducibleprotein[Bartonellavinsonii]_gi|395432864|gb|EJF98838.1|hypotheticalproteinME/      ---Q-DVPIQRINLFDLE-NSQIDWSTFEAK--------GD-IVLKEQKKKKLLD----H
gi|491002426|ref|WP_004864148.1|DNAdamageinducibleprotein[Bartonellavinsonii]_gi|395421767|gb|EJF88003.1|hypotheticalproteinME/      ---Q-EVRIQQINLFDLE-KSQIDWSRFEGK--------ET-VVLKEQKKKKLLD----H
gi|491004024|ref|WP_004865745.1|DNAdamageinducibleprotein[Bartonellavinsonii]_gi|395432542|gb|EJF98522.1|hypotheticalproteinME/      ---Q-EVRIQQINLFDLE-NSQIDWSRFEGK--------ES-VVLKEQKKKKLLD----H
gi|648396442|ref|WP_026088193.1|damageinducibleprotein[Bartonellarattaustraliani]/11593                                             ---Q-KVPIQRINLFDLE-NSQIDWGMFANK--------GQ-ATLKKREVKQLRD----H
gi|748190174|ref|WP_039761290.1|damageinducibleprotein[Bartonellaqueenslandensis]/11658                                             ---Q-KVPIQRINLFDLE-NSQIDWGMFANK--------GQ-ATLKKREVKQLRD----H
gi|494817599|ref|WP_007553007.1|DNAdamageinducibleprotein[Bartonellasp.DB56]_gi|395411312|gb|EJF77837.1|hypotheticalproteinMC/1      ---Q-GVRIQRINLFDLE-NSQIDWATFESK--------GE-ATLKKKEPKKLLD----H
gi|515763376|ref|WP_017195976.1|DNAdamageinducibleprotein[Bartonellabirtlesii]/11656                                                ---Q-RIRIQRINLFDLE-NSQIDWASFESK--------GE-ATLKKKELKKLLD----H
gi|494589420|ref|WP_007348014.1|DNAdamageinducibleprotein[Bartonellarattimassiliensis]_gi|395416278|gb|EJF82667.1|hypothetical/      ---Q-EIRPQRINLFDLE-NSQIDWGAYKEQ--------GQ-AVLKEKPKRKLLD----H
gi|506336469|ref|WP_015856188.1|DNAdamageinducibleprotein[Bartonellagrahamii]_gi|240267512|gb|ACS51100.1|helicase/methyltransf/      ---Q-EVRIQRINLFDLE-NSQIDWGAYKEQ--------GQ-AVLKEQPQKKLLD----H
gi|749308087|ref|WP_040132227.1|damageinducibleprotein[Bartonellatribocorum]_gi|605051878|emb|CDO49988.1|helicase/methyltransf/      ---Q-EIRPQRINLFDLE-NSQIDWGAYKEQ--------GQ-AVLKEKPKKKLLD----H
gi|515763800|ref|WP_017196400.1|DNAdamageinducibleprotein[Bartonellabirtlesii]/11658                                                ---Q-EVRIQRINLFDLE-NSQIDWGAYKEK--------GE-ATLKEQSKKNLLD----H
gi|492186994|ref|WP_005773380.1|DNAdamageinducibleprotein[Bartonellaelizabethae]_gi|395418361|gb|EJF84688.1|hypotheticalprotei/      ---Q-EVRIQQINLFDLE-NSQIDWGAYKER--------GQ-AVLKEQPKKKLLD----H
gi|501188615|ref|WP_012231634.1|DNAdamageinducibleprotein[Bartonellatribocorum]_gi|161017856|emb|CAK01414.1|helicase/methyltra/      ---Q-EVRIQQINLFDLE-NSQIDWGAYKER--------GQ-AVLKEQPKKKLLD----H
gi|501187470|ref|WP_012230499.1|DNAdamageinducibleprotein[Bartonellatribocorum]_gi|161017090|emb|CAK00648.1|helicase/methyltra/      ---Q-EVRIQQINLFDLE-NSQIDWGAYKEQ--------GQ-AVLKEKSKKKLLD----H
gi|501188636|ref|WP_012231655.1|DNAdamageinducibleprotein[Bartonellatribocorum]_gi|161017879|emb|CAK01437.1|helicase/methyltra/      ---Q-EVRIQQINLFDLE-NSQIDWGAYKER--------GQ-AVLKEQPKKKLLD----H
gi|930151941|emb|CTQ34818.1|UvsWhelicase[Jannaschiarubra]/11641                                                                     ---Q-TIPTNRIGLSDLE-ASPILWSDFART--------GE-VRVSEA--KTLRP----H
gi|928977106|ref|WP_054001461.1|damageinducibleprotein[Shimiasp.SK013]_gi|927698852|gb|KPA22698.1|typeIrestrictionenzymeEcoKIs/      ---Q-SIPVVRVGLDALR-DSAIDWSVYEAS--------DK-VQLRNK--KQLHS----H
gi|738872465|ref|WP_036760884.1|damageinducibleprotein[Leisingeradaeponensis]/11643                                                 ---Q-SIPVIRIGLNDLR-NSPIDWSQFAAQ--------GK-IVLEAK--KELRK----H
gi|518050881|ref|WP_019221089.1|DNAdamageinducibleprotein[Bartonellasenegalensis]/11652                                             ---Q-SIPVIRIGLSDLQ-QSPIRWETFAAK--------GK-IVLEDK--KKLRP----H
gi|493980596|ref|WP_006923523.1|DNAdamageinducibleprotein[Bartonellawashoensis]_gi|395413322|gb|EJF79796.1|hypotheticalprotein/      ---Q-DIPVIRINLSDIQ-KSPIRWETFAAT--------GK-VVLEDK--KKIRS----H
gi|493983106|ref|WP_006925984.1|DNAdamageinducibleprotein[Bartonellawashoensis]_gi|395418127|gb|EJF84455.1|hypotheticalprotein/      ---Q-DIPVIRINLSNIQ-QSPIRWETFTAT--------GK-VVLENK--KKLRP----H
gi|499494671|ref|WP_011181311.1|DNAdamageinducibleprotein[Bartonellahenselae]_gi|49239008|emb|CAF28308.1|hypotheticalproteinBH/      ---Q-DVPVIRINLSDMQ-ESPIRWEAFTAK--------GK-IVLEDK--KKLRP----H
gi|651388426|ref|WP_026500441.1|damageinducibleprotein[Bartonellagrahamii]/11652                                                    ---Q-DIPVIRINLSDIQ-QSPIRWETFAAQ--------GK-IVLEDK--KKIRP----H
gi|506337280|ref|WP_015856999.1|DNAdamageinducibleprotein[Bartonellagrahamii]_gi|240268402|gb|ACS51990.1|helicase/methyltransf/      ---Q-DIPVIRINLSDIQ-QSPIRWETFAAQ--------GK-VVLEDK--KKIRP----H
gi|494817535|ref|WP_007552943.1|DNAdamageinducibleprotein[Bartonellasp.DB56]_gi|395412002|gb|EJF78518.1|hypotheticalproteinMC/1      ---Q-GIPVIRIGLSDMQ-QSPIRWETFAAK--------GK-VVLEDK--KTLRP----H
gi|493638099|ref|WP_006589864.1|DNAdamageinducibleprotein[Bartonellabirtlesii]_gi|395409809|gb|EJF76394.1|hypotheticalproteinM/      ---Q-SIPVIRIGLSNIR-QSPICWEIFAAK--------DK-IVLEDK--KTLRP----H
gi|748189589|ref|WP_039760707.1|damageinducibleprotein[Bartonellaqueenslandensis]/11651                                             ---Q-DIPVIRIGLSDLQ-ESPIRWEIFAAK--------DK-IVLEDK--KKLRS----H
gi|501189483|ref|WP_012232501.1|DNAdamageinducibleprotein[Bartonellatribocorum]_gi|161018902|emb|CAK02460.1|helicase/methyltra/      ---Q-KIPVTRINLSDLQ-KSPIRWETFAAK--------GK-IVLGDK--KKLRP----H
gi|518049181|ref|WP_019219389.1|DNAdamageinducibleprotein[Bartonellaflorencae]/11652                                                ---Q-DIPVNRISLSDLQ-KSPIRWETFAAK--------GT-IVLESK--KKIRP----H
gi|492193255|ref|WP_005774528.1|DNAdamageinducibleprotein[Bartonellaelizabethae]_gi|395416403|gb|EJF82779.1|hypotheticalprotei/      ---Q-KIPVIRINLSDMQ-KSPIRWETFAAK--------GI-IALEGK--KKLRP----H
gi|498387325|ref|WP_010701288.1|helicase/methyltransferase[Bartonellabovis]_gi|478418724|gb|ENN92426.1|helicase/methyltransfera      ---Q-SIPVIRINLSDMQ-ESPIRWETFAAK--------NQ-IVLAPK--KDLRK----H
gi|319408697|emb|CBI82352.1|conservedhypotheticalprotein[BartonellaschoenbuchensisR1]/11661                                         ---Q-SIPVIRINLSDMQ-QSPIRWEIFAAK--------NQ-IVLAPK--KDLRE----H
gi|494741726|ref|WP_007477134.1|DNAdamageinducibleprotein[Bartonellamelophagi]_gi|395423925|gb|EJF90113.1|hypotheticalproteinM/      ---Q-DIPIIRINLSDMQ-QSPIRWETFATN--------NQ-IVLAPK--KDLRK----H
gi|737261405|ref|WP_035245349.1|damageinducibleprotein[Alcanivoraxjadensis]_gi|686989709|gb|KGD62556.1|hypotheticalproteinT9A_/      ---Q-YVETYRISLRDLE-SSRIDWTLFSEK--------QE-IAYQAK--KQLRP----H
gi|916687564|ref|WP_051294655.1|damageinducibleprotein[Gemmobacternectariphilus]/11641                                              ---Q-AIPTIRIGLTDLQ-ESPIRWDAFAAR--------GE-IILADK--KKLRP----H
gi|740294127|ref|WP_038132495.1|damageinducibleprotein[Thioclavasp.DT234]_gi|660524826|gb|KEO54689.1|hypotheticalproteinDT23_/1      ---Q-TIPVVRISLTHLR-ESPIDWAIFGLR--------GE-AVLSEK--KKLRP----H
gi|568331062|ref|WP_024099615.1|helicase[Phaeobactergallaeciensis]_gi|568232121|gb|AHD12007.1|putativehelicase[Phaeobactergalla      ---Q-AIPTIRIGLNDLQ-ESPILWETFSDD--------GE-VVLAGK--KTLRT----H
gi|518810459|ref|WP_019966413.1|DNAdamageinducibleprotein[Pannonibacterphragmitetus]/11635                                          ---Q-TIPVVRIALTDLR-ESRIDWTIFEAR--------GE-IVLSAK--KSLLD----H
```

```
gi|495305760|ref|WP_008030510.1|DNAdamageinducibleprotein[Rhodobactersp.SW2]_gi|259021698|gb|EEW25000.1|typeIIIrestrictionprot/   ---Q-VIPVVRIGLTDLR-ESRIDWTIFEAR--------GE-IVLSAK--KSLLD----H
gi|506403303|ref|WP_015923022.1|DNAdamageinducibleprotein[Rhodobactersphaeroides]_gi|221162951|gb|ACM03922.1|HypotheticalProte/   ---Q-AIPVVRIGLTDLR-ESRIDWTIFEAR--------GE-IVLAAR--KTLMP----H
gi|738818386|ref|WP_036708790.1|damageinducibleprotein[Paracoccussanguinis]_gi|694226786|gb|KGJ22541.1|damageinducibleprotein/1   ---Q-AIPVVRIGLTDLR-ESRIDWTIFEAR--------GE-IVLAAR--KTLMP----H
gi|746096057|ref|WP_039162453.1|damageinducibleprotein[Gallibacteriumanatis]_gi|703617775|gb|KGQ63868.1|damageinducibleprotei/1   ---Q-QPPVTVIDRSALE-NSLIDWSSFQPS--------QE-VKTREK--KTLRE----H
gi|737716785|ref|WP_035685631.1|damageinducibleprotein[Avibacteriumparagallinarum]/11643                                          ---Q-QPPVTVIDRSALE-NSLIDWSSFQPS--------QE-VKTREK--KTLRE----H
gi|540072553|gb|ERF78672.1|hypotheticalproteinN561_04930[Gallibacteriumanatis12656/12}/11651                                     ---Q-QPPVTVIDRSALE-NSLIDWSSFQPS--------QE-VKTRKK--KTLRE----H
gi|917509519|ref|WP_052115936.1|damageinducibleprotein[Gallibacteriumanatis]_gi|702414161|gb|KGQ42233.1|damageinducibleprotei/1   ---Q-QPPVTVIDRSALE-NSLIDWSSFQPS--------QE-VKIRAK--KTLRE----H
gi|746082942|ref|WP_039150268.1|damageinducibleprotein[Gallibacteriumanatis]_gi|703617078|gb|KGQ63194.1|damageinducibleprotei/1   ---Q-QPPVTVIDRSALE-NSLIDWSSFQPS--------QE-VKIRAK--KTLRE----H
gi|822522885|ref|WP_046952840.1|damageinducibleprotein[Haemophilushaemolyticus]_gi|821501762|gb|KKZ59267.1|damageinduciblepro/1   ---Q-NPPVTVIDRSALE-NSAIDWDNFAFQ--------KE-LPLKPK--KSLRE----H
gi|404381014|gb|EJZ77501.1|putativehelicase[Pasteurellamultocidasubsp.gallicidaP1059]/11681                                      ---Q-NPPVTLINRSDLE-NSTIDWQRFVFN--------KP-LILKPK--KSLRE----H
gi|298281660|gb|EFI23149.1|helicaseCterminaldomainprotein[Neisseriasp.oraltaxon014str.F0314]/11493                               ---Q-HIPVSKITLSDLE-NSVIDWTQYHIG--------EP-PKRKNR--KSLRP----H
gi|348013055|gb|EGY51980.1|helicasedomainprotein[Neisseriashayeganii871]/11481                                                   ---Q-QIPVAVINLADLE-NSQIDWSRFSID--------EQ-PVLKAK--KGLRP----H
gi|496407140|ref|WP_009116004.1|DNAdamageinducibleprotein[Neisseriawadsworthii]_gi|349794756|gb|EGZ48566.1|helicasedomainprote/   ---Q-HIPVAVINLEDLE-NSRIDWTQYQID--------AS-PVLKAR--KDLRP----H
gi|517216441|ref|WP_018405259.1|DNAdamageinducibleprotein[Marinobacterlipolyticus]/11656                                         ---Q-NPPVSKIDLHNLE-NSVIDWSQYH--EN------TK-PVLKAQ--KTLRE----H
gi|738230264|ref|WP_036185691.1|damageinducibleprotein[Marinobactersp.ELB17]/11671                                               ---Q-NPPVFKIDLHSLE-NSVIDWSQYR--ED------TK-PVLRAQ--KTLRE----H
gi|447197741|ref|WP_001274997.1|DNAdamageinducibleprotein[Acinetobacterbaumannii]_gi|408512324|gb|EKK13969.1|typeIIrestrictio/   ---A-NPPVATISLLDLE-GSLIDWSQFNFDSN------VK-PIFRKK--NSLRD----H
gi|820775897|ref|WP_046739909.1|damageinducibleprotein[Acinetobactersp.AG1]_gi|819909758|gb|KKW75938.1|damageinducibleprotein/1   ---A-HPPVATISLLDLE-GSLIDWSQFNFDPN------VK-PIFRDK--KQLRK----H
gi|584595163|ref|WP_024160846.1|helicase[Acinetobacterpittii]_gi|576702530|emb|CDI28049.1|helicasedomaincontainingprotein[Acin/   ---A-NPPVATISLLDLE-GSLIDWSQFNFDSS------VK-PIFRKK--NSLRD----H
gi|514963042|ref|WP_016651366.1|hypotheticalprotein[Acinetobactersp.NIPH2036]_gi|514162665|gb|EPG41391.1|hypotheticalproteinF90   ---A-NPPVATISLLDLE-GSLIDWSQFNFDSN------VK-PIFRKK--NSLRD----H
gi|515965564|ref|WP_017396147.1|DNAdamageinducibleprotein[Acinetobacterhaemolyticus]/11667                                       ---A-NPPVATISLLDLE-GSLIDWSQFNFDSN------VK-PIFRKK--NSLRD----H
gi|491346810|ref|WP_005204747.1|MULTISPECIES:hypotheticalprotein[Acinetobacter]_gi|480335823|gb|ENX56033.1|hypotheticalproteinF   ---A-HPPVATISLLDLE-GSLIDWTQFDFDPN------VK-PIFRDK--KQLRK----H
gi|693044472|ref|WP_032215268.1|damageinducibleprotein[Escherichiacoli]_gi|607725731|gb|EZE08230.1|damageinducibleprotein[Esc/1   ---K-AVPVTALSLTDLE-QSALDWSQFYFDPA------YK-PVMKAK--KQLRP----H
gi|742382177|ref|WP_038861304.1|damageinducibleprotein[Cronobactermuytjensii]_gi|924333747|gb|ALB69766.1|damageinducibleprote/1   ---K-AVPVTALSLTDLE-QSALDWSQFDFDPA------YQ-PVMKAK--KQLRP----H
gi|915912804|ref|WP_050927035.1|damageinducibleprotein[Yersiniaenterocolitica]_gi|899805802|emb|CQH32042.1|typeIrestrictionenz/   ---K-AVPVTALSLTDLE-QSALDWSQFDFDPA------YK-PVMKAK--KQLRA----H
gi|921111327|gb|KON62781.1|typeIrestrictionenzymeEcoKIsubunitR[Komagataeibactereuropaeus]/11492                                   ---Q-HPPVSKIDLYDLE-TSLIDWSQYQPK--------IA-PILRAQ--KKARE----H
gi|582024278|gb|AHI27615.1|DNAhelicaserestrictionenzymeTypeIIIRsubunit[KomagataeibacterxylinusE25]/11636                         ---Q-HPPVSKIDLYDLE-TSLIDWSQYQPN--------IA-PVLRAQ--KKARE----H
gi|347662673|dbj|BAK86128.1|DNAhelicaserestrictionenzymeTypeIIIRsubunit[KomagataeibactermedellinensisNBRC3288]/11737             ---Q-HPPVSKIDLYDLE-TSLIDWSQYQPK--------IA-PILRAQ--KKARE----H
gi|917445053|ref|WP_052051471.1|damageinducibleprotein[Acetobactertropicalis]/11429                                              ---Q-HPPVSKIDLLDLE-TSIIDWSQYQPK--------QN-PVLREK--KKPRE----H
gi|453329445|dbj|GAC88472.1|DNAhelicaserestrictionenzymetypeIIIRsubunit[GluconobacterthailandicusNBRC3255]_gi|530287981|dbj|GAD   ---Q-HPPVSKIDLLDLE-TSVIDWSQYQPK--------QA-PVLRKK--KTPLP----H
gi|889770922|ref|WP_048844278.1|damageinducibleprotein[Gluconobacterfrateurii]/11419                                             ---Q-HPPVSKIDLLDLE-TSVIDWSQYQPK--------QA-PVLREK--KTPRE----H
gi|504886208|ref|WP_015073310.1|hypotheticalprotein[Gluconobacteroxydans]_gi|411027954|gb|AFW01209.1|hypotheticalproteinB932_16   ---Q-HPPVSKIDLLDLE-ASVIDWSQYQPK--------QA-PVLRKK--KTPLP----H
gi|480040211|gb|ENV79751.1|hypotheticalproteinF942_01641[AcinetobacterursingiiANC3649]/11432                                      ---Q-HLPVQKIDLYALE-HSVIDWEKYQPK--------QQ-LELKPK--KNLRS----H
gi|822498222|ref|WP_046936504.1|damageinducibleprotein[Xanthomonasgardneri]_gi|821751224|gb|KLB07223.1|damageinducibleprotein/1   ---Q-QPPVTKIDLQALE-DSQIDWSRFQPK--------QASVILKPK--KTLRP----H
gi|654059559|ref|WP_027705556.1|damageinducibleprotein[Zymobacterpalmae]/11635                                                  ---Q-QPPVTKIDLHDLE-SSLIDWSQFRVD--------EP-PALKPK--KSLRD----H
gi|643535620|ref|WP_025224315.1|damageinducibleprotein[Methylophilaceaebacterium11]_gi|577704400|gb|EUJ10546.1|putativehelicas/   ---Q-HPPVSKIDLNDLE-NSQIDWSKYQSK--------VT-PILKSK--KQLRE----H
MBA11I/11635                                                                                                                      ---Q-HPPVSKIDLNDLE-NSQIDWSKYQSK--------VT-PILKSK--KQLRE----H
gi|655355857|ref|WP_028762287.1|damageinducibleprotein[Shewanellacolwelliana]/11632                                              ---Q-QIPVSKIDLADLE-SSQIDWSKYESN--------EA-PVLKKK--FDPRP----H
gi|753204236|ref|WP_041510579.1|damageinducibleprotein[Shewanellasp.cp20]_gi|752457765|gb|KIO36326.1|damageinducibleprotein[S/1   ---Q-NPPVTRIDLQALE-DSVIDWSKYQPD--------TT-VQIKVK--NKPRP----H
KPNNIH30III/11619                                                                                                                 ---Q-NPPVTKIDLHALE-ESVIDWSKYQPK--------AE-VQCKVK--NKPRP----H
gi|749295994|ref|WP_040120352.1|damageinducibleprotein[Klebsiellapneumoniae]_gi|723249300|gb|AIX81926.1|damageinducibleprotei/1   ---Q-NPPVTKIDLHALE-ESVIDWSKYQPK--------AE-VQCKVK--NKPRP----H
gi|641639739|emb|CDQ54010.1|unnamedproteinproduct[Klebsiellapneumoniae]/11641                                                    ---Q-NPPVTKIDLHALE-ESVIDWSKYQPK--------AE-VQCKVK--NKPRP----H
gi|490797922|ref|WP_004660060.1|hypotheticalprotein[Acinetobactersp.NIPH236]_gi|479876779|gb|ENU27973.1|hypotheticalproteinF992   ---Q-DPPVTKIDLEILE-QSVIDWSKFAEK--------KQ-VVFKPK--KELRD----H
gi|916750926|ref|WP_051357982.1|damageinducibleprotein[Acinetobactertjernbergiae]/11453                                          ---Q-DPPVTKIDLDTLA-QSAIDWSLFAEK--------KE-VIFKKK--KYLRD----H
gi|691098662|ref|WP_032025060.1|damageinducibleprotein[Acinetobacterbaumannii]_gi|593567521|gb|EXR50735.1|restrictionendonucle/   ---Q-APPVTKIDLDTLA-QSAIDWSLFAEK--------KE-VVFKPK--KELRD----H
gi|736669405|ref|WP_034676049.1|damageinducibleprotein[Acinetobacterbaumannii]/11643                                             ---Q-DPPVTKIDLDTLE-QSAIDWSLFAEK--------KE-VVFKPK--KELRD----H
gi|491368928|ref|WP_005226826.1|hypotheticalprotein[Acinetobactersp.NIPH1847]_gi|480173259|gb|ENX09797.1|hypotheticalproteinF89   ---Q-DPPVTKIDLETLA-QSAIDWSQFAEK--------KE-VIFKKK--KELRD----H
gi|796853993|ref|WP_045795953.1|damageinducibleprotein[Acinetobacterindicus]_gi|787929761|gb|KJV44219.1|damageinducibleprotei/1   ---Q-DPPVTKIDLETLA-QSAIDWSKFAEK--------KE-VILKTK--KQLRD----H
gi|295797463|gb|ADG32253.1|typeIIIrestrictionproteinressubunit[ThiomonasintermediaK12]/11676                                     ---R-DKPVQIISLYDLE-ASQIDWARYQPD--------HA-PALKPK--KQLRP----H
gi|499871179|ref|WP_011551913.1|DNAdamageinducibleprotein[Myxococcusxanthus]_gi|108467544|gb|ABF92729.1|putativerestriction/mo/   ---Q-NTPVTKIDLAALE-KSAIDWSRFAPR--------KA-PVLRKK--KTPLP----H
gi|495313852|ref|WP_008038600.1|DNAdamageinducibleprotein[Bartonellatamiae]_gi|395424181|gb|EJF90368.1|hypotheticalproteinME5_/   ---Q-HVPVSKIDLAALE-QSVIDWEQYAPD--------KA-LVIHEK--KKLRP----H
gi|501453335|ref|WP_012476784.1|DNAdamageinducibleprotein[Leptospirabiflexa]_gi|167777824|gb|ABZ96123.1|Helicase[Leptospirabif/   ---Q-TPPVTKIDLYHLE-NSAIDWSKYQAK--------EK-VCFKES--KKLRD----H
gi|738104613|ref|WP_036063193.1|damageinducibleprotein[Leptospiranoguchii]/11652                                                 ---Q-TIPVTKIDLYDLE-KSSIDWNKFKPK--------EK-ITFKEK--KSLRP----H
gi|410001039|gb|EKO51661.1|typeIIIrestrictionenzyme,ressubunit[Leptospirakirschneristr.200802841]/11651                          ---Q-TIPVTKIDLFDLE-KSSIDWSRFKPK--------AK-VAFKEK--KSLRP----H
gi|490901851|ref|WP_004763774.1|typeIIIrestrictionenzyme,ressubunit[Leptospirakirschneri]_gi|464209249|gb|EMN24014.1|typeIIIres   ---Q-TIPVTKIDLFDLE-KSSIDWSRFKPK--------AK-VAFKEK--KSLRP----H
gi|464398131|gb|EMO65902.1|typeIIIrestrictionenzyme,ressubunit[Leptospirakirschneristr.200803703]/11648                          ---Q-TIPVTKIDLFDLE-KSSIDWSRFKPK--------AK-VAFKEK--KSLRP----H
gi|945029024|gb|KQQ84900.1|damageinducibleprotein[Xanthomonassp.Leaf131]/11423                                                  ---Q-QPPVSKIDLHDLE-GSQIDWSRYQSK--------V-VPVLKDK--KTLRP----H
gi|896511314|ref|WP_049418183.1|damageinducibleprotein[Stenotrophomonasmaltophilia]/11639                                        ---Q-QPPVSKITLHDLE-ASQIDWSRFQPS--------A-PVVLKPK--KALRP----H
gi|896513082|ref|WP_049419616.1|damageinducibleprotein[Stenotrophomonasmaltophilia]/11638                                        ---Q-QPPVSKITLHDLE-QSQIDWAKFQPR--------T-AITLKAK--KSLRP----H
gi|896580616|ref|WP_049469425.1|damageinducibleprotein[Stenotrophomonasmaltophilia]/11641                                        ---Q-QPPVSKITLHDLE-ASQIDWSRFQPN--------A-PVALKAK--KSLRP----H
gi|648289350|ref|WP_026070499.1|damageinducibleprotein[Stenotrophomonasmaltophilia]/11639                                        ---Q-QPPVSKITLHDLE-ESQIDWTKFQPK--------A-AVTLKAK--KSLRP----H
gi|656272786|ref|WP_029218442.1|damageinducibleprotein[Xanthomonascassavae]/11639                                                ---Q-QPPVSKITLHDLE-QSQIDWAKFQPK--------T-AITLKSK--KSLRP----H
gi|406976236|gb|EKD98747.1|hypotheticalproteinACD_23C00290G0002[unculturedbacterium]/11679                                       ---R-DKPVNIIAISDLE-ASLIDWSQYLPE--------VPAVTLRSK--KALRP----H
gi|828145737|ref|WP_047238120.1|damageinducibleprotein[Chromobacteriumsubtsugae]/11645                                           ---Q-QPPVTKLDLQALE-DSQIDWSQYASK--------Q-AVVLKPK--KQLRD----H
gi|654311174|ref|WP_027824327.1|damageinducibleprotein[Laribacterhongkongensis]/11641                                            ---Q-QPPVTKLDLQALE-DSQIDWNQYQPK--------Q-AAVLKPK--KQLRD----H
gi|757242173|gb|KIR15516.1|typeIrestrictionenzymeEcoKIsubunitR[Pseudomonasfluorescens]/11778                                    ---Q-QPPVSKIDRQDLE-KSQIDWARYQPN--------Q-AVAFKAK--KQLRD----H
gi|653605161|ref|WP_027616682.1|damageinducibleprotein[Pseudomonassp.URHB0015]/11639                                             ---Q-QPPVSKIDRQDLE-NSQIDWARYQPN--------Q-AVVLKAK--KQLRD----H
gi|520868025|ref|WP_020308060.1|helicase[Pseudomonassyringae]_gi|519159034|gb|EPM49935.1|helicasedomaincontainingprotein[Pseud/   ---Q-QPPVSKIDRQDLE-NSQIDWARYQPN--------Q-AVVLKAK--KQLRD----H
```

```
gi|940156567|gb|KPX71668.1|Helicasedomaincontainingprotein[Pseudomonasamygdalipv.lachrymans]/11677    ---Q-QPPVSKIDRQDLE-NSQIDWARYQPN--------Q-AVVFKAK--KELRD----H
gi|505460508|ref|WP_015647610.1|helicase[Pseudomonassp.GLE121]_gi|499110212|ref|YP_007974374.1|helicasedomaincontainingprotein/    ---Q-HQSINKIDLQALE-DSQIDWAKYQPN--------Q-APVLKEK--KQLRD----H
gi|940235551|gb|KPY45311.1|hypotheticalproteinALO48_200154[Pseudomonassyringaepv.rhaphiolepidis]/11631    ---Q-HQTINKIDLQALE-DSQIDWSKYQPN--------Q-AQVLKEK--KQLRD----H
gi|520880808|ref|WP_020312235.1|helicase[Pseudomonassyringae]_gi|519172158|gb|EPM62027.1|helicasedomaincontainingprotein[Pseud/    ---Q-HPPVSKIDLQALE-ESQIDWAKYQPS--------E-KPVLKAK--KSLEGEKYQH
gi|829046479|ref|WP_047282259.1|damageinducibleprotein[Pseudomonasfluorescens]/11630    ---Q-HQSINKIDLQALE-DSQIDWAKYQPN--------Q-PVALKAR--KQLRD----H
gi|499415493|ref|WP_011102960.1|DNAdamageinducibleprotein[Pseudomonassyringaegroupgenomosp.3]_gi|28867279|ref|NP_789898.1|heli/    ---Q-HQTINKIDLQALE-ESQIDWAKYQPH--------Q-AVVLKAR--KQLRD----H
gi|730238299|ref|WP_033942897.1|damageinducibleprotein[Pseudomonasaeruginosa]/11629    ---Q-QPPVSKIDLQALE-DSQIDWAKYQPN--------Q-AVVLKAR--KQLRD----H
gi|495310125|ref|WP_008034873.1|DNAdamageinducibleprotein[Pseudomonassp.GM60]_gi|398186685|gb|EJM74052.1|putativehelicase[Pseu/    ---Q-QLSINKIDMQALE-DSQIDWAKYQPK--------Q-AVALKSR--KQLRD----H
gi|779992653|ref|WP_045428690.1|damageinducibleprotein[Pseudomonassp.MT1]_gi|699967872|dbj|BAP80334.1|DNAhelicaserestrictione/1    ---N-HQTINKIDLQALE-DSQIDWAKYQPN--------Q-AVALKAK--KKLRD----H
gi|786037289|ref|WP_045733274.1|damageinducibleprotein[Pseudomonaspseudoalcaligenes]_gi|782987904|gb|KJU79152.1|damageinducib/1    ---Q-QPPVSKIDLQALE-DSQIDWAKYQPN--------Q-AVMLKAR--KQLRD----H
gi|749666366|ref|WP_040265445.1|damageinducibleprotein[Pseudomonasrhodesiae]/11632    ---Q-QPPVSKIDLQALE-DSQIDWAKYQPH--------Q-AVALKAK--KQLRE----H
gi|734902433|ref|WP_034133698.1|damageinducibleprotein[Pseudomonasfluorescens]/11635    ---Q-QPPVSKIDLQALE-DSQIDWAKYQPH--------Q-AVALKAK--KQLHE----H
gi|490551031|ref|WP_004416146.1|typeIIrestrictionmodificationsystemrestriction/helicasesubunit[Pseudomonassyringae]_gi|4432823/    ---Q-QPPVSKVDLQALE-DSQIDWAKYQPH--------Q-AVALKAK--KQLRE----H
gi|489199911|ref|WP_003109152.1|DNAdamageinducibleprotein[Pseudomonasaeruginosa]_gi|552648708|gb|ERY63184.1|hypotheticalprotei/    ---Q-QPPVSKIDLQALE-DSQVDWAKYQPN--------Q-AVVLKAR--KQLRD----H
gi|730641237|ref|WP_034061328.1|damageinducibleprotein[Pseudomonasaeruginosa]/11636    ---Q-QLSINKIDLQALE-ESQIDWTRYQPH--------E-KPILKAK--KSLEDEKYQH
gi|751304980|ref|WP_041013386.1|damageinducibleprotein[Pseudomonasxanthomarina]_gi|703407611|emb|CEG51014.1|Helicasedomaincon/1    ---Q-QPPVSKIDLQALE-DSQIDWAKYQPN--------Q-AVALKAK--KQLR----EH
gi|939335207|ref|WP_054881520.1|damageinducibleprotein[Pseudomonassp.NBRC111128]/11632    ---Q-QPPVSKIDLQALE-DSQIDWAKYQPN--------Q-AVALKAK--KQLR----EH
gi|757693555|ref|WP_042923792.1|damageinducibleprotein[Pseudomonaschloritidismutans]_gi|780910610|gb|KJS78302.1|damageinducib/1    ---Q-QPPVSKIDLQALE-DSQIDWAKYQPN--------Q-TVALKAK--KTLRD----H
gi|665953944|ref|WP_031319989.1|damageinducibleprotein[Pseudomonastolaasii]/11626    ---Q-QPPVSKIDLQALE-DSQIDWVKYQSS--------K-AVTLKTK--KQLRE----H
gi|771600573|ref|WP_045213746.1|damageinducibleprotein[Pseudomonassp.21]_gi|770081123|gb|KJJ98634.1|damageinducibleprotein[Ps/1    ---Q-QLSINKIDLQALE-ESQIDWAKYQPN--------Q-AVAFKAR--KQLRD----H
gi|740871396|ref|WP_038656644.1|damageinducibleprotein[Pseudomonasstutzeri]_gi|627772214|gb|AHY41437.1|damageinducibleprotein/1    ---Q-QPPVSKIDLQALE-DSQIDWAKYQPN--------Q-AVALKAK--KQLRE----H
gi|946242205|gb|KRC93782.1|damageinducibleprotein[Pseudomonassp.Root9]/11634    ---Q-QPPVSKIDLQALE-DSQIDWAKYQPN--------Q-AVALKAK--KQLRD----H
gi|553777322|ref|WP_023109107.1|MULTISPECIES:hypotheticalprotein[Pseudomonas]_gi|552638674|gb|ERY53184.1|hypotheticalproteinQ05    ---Q-QPPVSKIDLQALE-DSQIDWAKYQPN--------Q-AVALKAK--KQLRE----H
gi|896383370|ref|WP_049324618.1|damageinducibleprotein[Pseudomonasstutzeri]/11627    ---Q-QPPVSKIDLQALE-DSQIDWAKYQPN--------Q-AVAFKAK--KQLRE----H
gi|696158510|gb|AIT41787.1|putativehelicase[Pseudomonassp.LM8]/11630    ---Q-QPPVSKIDLQALE-DSQIDWAKYQPN--------Q-AVALKAK--KQLRE----H
gi|504634892|ref|WP_014821994.1|DNAdamageinducibleprotein[Pseudomonasstutzeri]_gi|390985249|gb|AFM35242.1|helicasedomainconta/1    ---Q-QPPVSKIDLQALE-DSQIDWAKYQPN--------Q-AVALKAK--KQLRE----H
gi|544783974|ref|WP_021206729.1|DNAdamageinducibleprotein[Pseudomonasstutzeri]_gi|533224779|gb|EQM79688.1|DNAdamageinduciblep/1    ---Q-QPPVSKIDLQALE-DSQIDWAKYQPN--------Q-AVALKAK--KQLRE----H
gi|504567385|ref|WP_014754487.1|MULTISPECIES:DNAdamageinducibleprotein[Pseudomonas]_gi|388559493|gb|AFK68634.1|helicasedomain/1    ---Q-QPPVSKIDLQALE-DSQIDWAKYQPN--------Q-AVALKAK--KQLRD----H
gi|654441469|ref|WP_027912052.1|damageinducibleprotein[Pseudomonassp.URIL14HWK12:I7]/11630    ---Q-QPPVSKIDLQALE-DSQIDWAKYQPN--------Q-AVALKAK--KQLRE----H
gi|941470066|ref|WP_055127894.1|damageinducibleprotein[Pseudomonasmediterranea]/11631    ---Q-QPPVSKIDLQALE-DSQIDWAKYQPH--------Q-AVALKAK--KQLRE----H
gi|557815599|ref|WP_023446184.1|DNAdamageinducibleprotein[Pseudomonaschloritidismutans]_gi|557364081|gb|ESQ98190.1|DNAdamagei/1    ---Q-QPPVSKIDLQALE-DSQIDWAKYQPN--------Q-AVALKTK--KQLRD----H
gi|727814791|ref|WP_033898247.1|damageinducibleprotein[Pseudomonassp.FH1]_gi|567648005|gb|ETK23229.1|helicasedomaincontaining/1    ---Q-QPPVSKIDLQALE-DSQIDWAKYQPN--------Q-AVALKAK--KQLRA----H
gi|503278702|ref|WP_013513363.1|DNAdamageinducibleprotein[Desulfovibrioaespoeensis]_gi|316942375|gb|ADU61426.1|typeIIIrestrict/    ---Q-FPPVNKIDLHDLE-NSQIDWAQYQPK--------A-EPVLKPK--KVLRP----H
gi|505228796|ref|WP_015415898.1|typeIIIrestrictionendonucleasesubunitR[Desulfovibriopiezophilus]_gi|451896976|emb|CCH49855.1|Ty    ---Q-FPPVNKIDLHDLE-NSQIDWSQYQPD--------T-EPVLKEK--YGLRD----H
gi|517732543|ref|WP_018902751.1|hypotheticalprotein[Variovoraxparadoxus]/11675    ---Q-QPPVNKIDLYDLE-NSQIDWAKYQSR--------A-PAVLKPQ--KVIRP----H
gi|550909475|ref|WP_022662570.1|DNAdamageinducibleprotein[Desulfovibriolongus]/11637    ---Q-QPPVSKIDLFDLE-NSQIDWASYQPQ--------Q-QPTLRAK--KHPLD----H
gi|945494542|gb|KQV50024.1|damageinducibleprotein[Massiliasp.Root335]/11647    ---Q-QPPVSKIDLNDLE-NSQIDWGKYQPK--------A-VPVLKVK--KQLRL----H
gi|334196917|gb|AEG70102.1|helicasedomainprotein[RalstoniasolanacearumPo82]/11663    ---Q-QPPVSKIDLTALE-ESQIDWSQYQPK--------APTPVIKAK--KQLRD----H
gi|656006746|ref|WP_029048170.1|damageinducibleprotein[Cupriavidussp.amp6]/11661    ---Q-QPPVSKIDLTALE-ESQIDWSRYKPK--------A-APVIKAK--KRLRD----H
BPS2196ORF3054P/11659    ---Q-QPPVSKIDLTALE-ESQIEWGHYQPK--------A-VPVIKAK--KQLRQ----H
gi|740932312|ref|WP_038717332.1|damageinducibleprotein[Burkholderiapseudomallei]_gi|760211882|gb|KIX58314.1|damageinduciblepr/1    ---Q-QPPVSKIDLTALE-ESQIEWGHYQPK--------A-VPVIKAK--KQLRQ----H
gi|755810813|ref|WP_042583861.1|damageinducibleprotein[Burkholderiasp.MSHR3999]_gi|755174952|gb|KIP19790.1|DEAD/DEAHboxhelicas/    ---Q-QPPVSKIDLTALA-ESQIDWQKYQPK--------A-APVIKAK--KQLRP----H
gi|484586570|gb|AGK48195.1|DEAD/DEAHboxhelicasefamilyprotein[BurkholderiathailandensisMSMB121]/11646    ---Q-QPPVSKIDLTALE-ESQIDWSQYEPK--------T-APVIKAK--KQLRP----H
gi|737582935|ref|WP_035553941.1|damageinducibleprotein[Burkholderiasp.9120]/11658    ---Q-QPPVSKIDLTALE-ESQIDWGQYQAK--------A-TPVIKSK--KQLRP----H
gi|740989872|ref|WP_038773225.1|damageinducibleprotein[Burkholderiapseudomallei]_gi|705645291|gb|KGS70691.1|DEAD/DEAHboxhelica/    ---Q-QPPVSKIDLTALE-ESQIDWGKYQPN--------A-APVIKAK--KQLRQ----H
gi|654912525|ref|WP_028363424.1|damageinducibleprotein[Burkholderiasp.JPY366]/11659    ---Q-QPPVSKIDLTALE-ESQIDWGQYQPK--------A-APVIKAK--KELRP----H
gi|740955879|ref|WP_038740512.1|damageinducibleprotein[Burkholderiapseudomallei]_gi|714684932|gb|KGW74438.1|DEAD/DEAHboxhelica/    ---Q-QPPVSKIDLSALE-ESQIDWGQYQPK--------A-APVIKAK--KQLRQ----H
gi|919203714|ref|WP_052759215.1|damageinducibleprotein[Xanthomonasaxonopodis]/11388    ---Q-QIPCARLRVADLE-DSAVDWSKFSLA-------KPD-HV-VLRTKNKLFK----H
gi|522152381|ref|WP_020663589.1|hypotheticalprotein[Amycolatopsisbenzoatilytica]/11605    ---Q-RIPVSRVGLADIG-QSPIRWDLAWPRPG-----IDI-EL-RLEHKKTLRP----H
gi|663196013|ref|WP_030233899.1|damageinducibleprotein[Streptomycessp.NRRLS455]/11641    ---Q-QIPVQRLGLSDIA-ASRVEWRLPASGDA-----AQV-EL-SLKGKKTPRS----H
gi|750429553|ref|WP_040711444.1|damageinducibleprotein[Nocardiopsispotens]/11622    ---Q-QIPVTRIGLADIA-DSPVEWHLPAPGSP-----TGI-KA-SLQAKKTLRP----H
gi|518656139|ref|WP_019819475.1|hypotheticalprotein[Saccharomonosporasaliphila]/11623    ---Q-QIPVTRVGLAEIA-FSPVQWHVPAPGAR-----SEI-GM-TLRARKQPLP----H
gi|759722933|ref|WP_043433758.1|damageinducibleprotein[Streptomycespluripotens]_gi|733404978|gb|KHK94361.1|damageinduciblepro/1    ---Q-QIPVTRIGLADIA-NSAVEWHLPAEGID-----HEV-QM-TLRDKKSPRP----H
gi|663424167|ref|WP_030419030.1|damageinducibleprotein[Streptomycessp.NRRLF5065]/11627    ---Q-QILVTRIGLADIA-NSAVEWHLPAEGID-----HEV-QM-TLRDKKSPRP----H
gi|663412917|ref|WP_030408353.1|damageinducibleprotein[Streptomycessp.NRRLF5917]/11630    ---Q-QIPVTRIGLADIA-NSPVEWHLPAEGID-----HEV-QM-TLREKKTPRP----H
gi|671521483|ref|WP_031506489.1|damageinducibleprotein[Streptomycesmegasporus]/11630    ---Q-QIPVTRIGLSDIA-NSPVEWHLPAEGID-----HEV-QM-TLREKKSPRP----H
gi|740053144|ref|WP_037902776.1|damageinducibleprotein[Streptomycessp.TAA204]/11626    ---Q-QIPVTRLGLSDIA-NSPVEWHLPAQD-------APF-EL-SLQDKKAPRR----H
gi|663409375|ref|WP_030404974.1|MULTISPECIES:damageinducibleprotein[Streptomyces]/11627    ---Q-QIPVTRLGLSDIA-NSPIEWRMPAVD-------EPF-EL-ALQEKKAPRK----H
gi|746628608|ref|WP_039654277.1|damageinducibleprotein[Streptomycessp.MUSC125]_gi|745644381|gb|KIE25557.1|damageinducibleprot/1    ---Q-QIPVTRLGLSDIA-NSPVEWRLPAVD-------EPI-EL-TLQKKKEPRK----H
gi|727322066|ref|WP_033777320.1|damageinducibleprotein[Salinisporapacifica]/11627    ---Q-QVPVTRIGLDEIA-QSPVQWQLPGVD-------RRI-EM-TLRAKKSPLK----H
gi|653653242|ref|WP_027655726.1|damageinducibleprotein[Salinisporapacifica]/11618    ---Q-QIPVTRIGLVEIS-ESPVEWYMPTYG-------HRV-DL-RLRAKKAPRP----H
gi|648632543|ref|WP_026324294.1|damageinducibleprotein[Salinisporapacifica]/11621    ---Q-QIPVTRVGLVEIS-QSPVEWHIPSYG-------HQP-DL-TLRAKKTPRP----H
gi|881018002|ref|WP_048738463.1|damageinducibleprotein[Corynebacteriumfalsenii]/11676    ---Q-LIPTNRIGLADMQ-ASPINWELTFPADS-----LEI-HL-TRRETFEPRE----H
gi|505465526|ref|WP_015652417.1|helicase[Corynebacteriumcallunae]_gi|459385704|gb|AGG67995.1|helicase[CorynebacteriumcallunaeDS    ---Q-TIPTNRIGLSAIA-ESPIDWDIAYPGSE-----LTI-NL-QLKEPYSPRP----H
gi|41223062|emb|CAF19007.1|DNAorRNAhelicaseofsuperfamilyII[CorynebacteriumglutamicumATCC13032]/11646    ---Q-TIPTNRIGLSAIA-ESPIDWDIAYPGSE-----LTI-NL-QLKEPYSPRP----H
CGL13032I/11643    ---Q-TIPTNRIGLSAIA-ESPIDWDIAYPGSE-----LTI-NL-QLKEPYSPRP----H
gi|751257081|ref|WP_040967675.1|damageinducibleprotein[Corynebacteriumglutamicum]_gi|748811620|gb|AJE67931.1|damageinduciblep/1    ---Q-VIPTNRIGLGAIA-ESPINWDIAYPGSE-----LTI-NL-ERKETFEPRP----H
```

```
gi|749040539|ref|WP_040086524.1|damageinducibleprotein[Corynebacteriumhumireducens]/11663    ---Q-PIPVSRIGIGAMA-ESPINWDIAYPNSE-----LTI-NL-SRRETFKPRP----H
gi|491662899|ref|WP_005519615.1|helicase[Corynebacteriummatruchotii]_gi|224946756|gb|EEG27965.1|helicaseCterminaldomainprotein/    ---Q-LIPTSRIGLSTIA-ESPIDWDIVYPGSE-----ISF-NL-TLRETYEPRP----H
gi|746717312|ref|WP_039676231.1|damageinducibleprotein[Corynebacteriumminutissimum]_gi|734991118|gb|KHO29219.1|damageinducibl/1    ---Q-LIPTNRIGTAAIA-ESPINWDVTFPGSE-----IEI-NL-TRREVFSSRP----H
gi|908654892|emb|CQD15529.1|SuperfamilyIIDNA/RNAhelicase(modularprotein)[Corynebacteriumstriatum]/11674    ---Q-LIPTNRISTAAIA-ESPINWDVTFPGSE-----IEI-NL-TRREVFSPRP----H
gi|895994446|ref|WP_049064418.1|damageinducibleprotein[Corynebacteriumaurimucosum]/11667    ---Q-LIPTNRIGTAAIA-ESPIDWDVTFPGSE-----IEI-NL-TRREVFSPRP----H
gi|896123331|ref|WP_049145390.1|damageinducibleprotein[Corynebacteriumaurimucosum]/11669    ---Q-LIPTNRIGTAAIA-ESPIDWDVTFPGSE-----IEI-NL-TRREVFSPRP----H
gi|817681178|ref|WP_046645422.1|damageinducibleprotein[Corynebacteriumstriatum]_gi|817551679|gb|KKO79868.1|damageinduciblepro/1    ---Q-LIPTNRIGTAAIA-ESPIDWDVTFPGSE-----IEI-NL-TRREVFSPRP----H
gi|896139239|ref|WP_049159146.1|damageinducibleprotein[Corynebacteriumaurimucosum]/11675    ---Q-LIPTNRIGTAAIA-ESPIDWDVTFPGSE-----IEI-NL-TRREVFSPRP----H
gi|489943947|ref|WP_003847254.1|helicase[Corynebacteriumammoniagenes]_gi|295968218|gb|EFG81467.1|helicaseCterminaldomainprotei/    ---Q-VIETSRIGMSDIA-TAPVNWDVAFPGSE-----IQI-NL-SRKEAFAPRK----H
gi|516653464|ref|WP_018022453.1|helicase[Corynebacteriumdoosanense]_gi|697778282|gb|AIT61018.1|damageinducibleprotein[Coryneba/    ---Q-MIETSRIGMADIA-SAPVNWDVAFPGSE-----IQI-NL-TKKQAFDPRP----H
gi|493894179|ref|WP_006840129.1|helicase[Corynebacteriumlipophiloflavum]_gi|227078819|gb|EEI16782.1|helicaseCterminaldomainpro/    ---Q-VIPTSRIGLADIA-QSPIDWDVAFPGSE-----IQV-NL-SRKETFSPRP----H
gi|740831472|ref|WP_038616755.1|damageinducibleprotein[Corynebacteriumpseudotuberculosis]_gi|666103058|gb|AIG06935.1|putativeh/    ---Q-MIPTSRIGRADIS-GAPVDWDVVFPGSE-----IEV-KL-TRKEPFKPRK----H
gi|518023322|ref|WP_019193530.1|helicase[Corynebacteriumtimonense]/11655    ---Q-VIETSRIGMADIA-AAPVNWDVVFPGSE-----MQI-NL-TRKEAFMPRK----H
gi|552767697|ref|WP_023019559.1|MULTISPECIES:hypotheticalprotein[Corynebacterium]_gi|550739013|gb|ERS38609.1|hypotheticalprotei    ---Q-IIETSRIGIGDIA-SAPVNWDVAFPGSE-----IQV-KL-ERKQAFQPRK----H
gi|652659121|ref|WP_027017812.1|damageinducibleprotein[Corynebacteriumpseudodiphtheriticum]/11644    ---Q-IIETSRIGIADIA-SAPVNWDVAFPGSE-----IQV-KL-ERKQAFQPRK----H
gi|749039309|ref|WP_040085390.1|damageinducibleprotein[Corynebacteriumhumireducens]/11657    ---Q-LIPTNRIGMYSIS-KSPINWDLTFPNSE-----IEI-NL-SLREVYKPLP----H
gi|740835591|ref|WP_038620874.1|damageinducibleprotein[Corynebacteriumulcerans]_gi|698973854|gb|AIU33664.1|HelicaseCterminald/1    ---Q-QIPASRIGYSDIA-RSPINWEIVFPGSE-----INV-HL-TRREAFAPRK----H
gi|805307599|ref|WP_046096452.1|damageinducibleprotein[Corynebacteriumulcerans]_gi|803357323|gb|AKA97638.1|Damageinduciblepro/1    ---Q-QIPASRIGHSDIA-RSPINWEIVFPGSE-----INV-HL-TRREAFAPRK----H
gi|504083555|ref|WP_014317549.1|helicase[Corynebacteriumdiphtheriae]_gi|371584249|gb|AEX47914.1|superfamilyIIDNA/RNAhelicase[Co    ---Q-LIATNRIGMASIA-ESPINWDVVFPGS-----EIQI-NL-SRREIFKPRT----H
gi|896182193|ref|WP_049193580.1|damageinducibleprotein[Corynebacteriumjeikeium]/11645    ---Q-LIATNRIGMASIA-ESPINWDVAFPGS-----EIQI-NL-SRHEIFKPRP----H
gi|917636459|ref|WP_052205460.1|damageinducibleprotein[Corynebacteriumriegelii]_gi|914295811|gb|AKV59187.1|damageinduciblepro/1    ---Q-QIPTSRIGYSQIA-ESPVNWDVVFPGS-----EIHF-NL-SRRETFEPRP----H
gi|737013522|ref|WP_035009205.1|damageinducibleprotein[Corynebacteriumjeikeium]/11651    ---Q-QIPTSRIGFSQIA-ESPVNWDVVFPGS-----EIQV-HL-TRRETFEPRP----H
gi|737006291|ref|WP_035002094.1|damageinducibleprotein[Corynebacteriumjeikeium]/11658    ---Q-VIPTSRIGVAAIS-ESPINWDIAFPGAAMGDSAIQI-NL-SRRETFEPRP----H
gi|737010378|ref|WP_035006133.1|damageinducibleprotein[Corynebacteriumjeikeium]/11650    ---Q-VIPTSRIGVSAIS-ESPINWDLVFPGAAMGDSAMQI-YL-SRRETFEPRP----H
gi|737010473|ref|WP_035006228.1|damageinducibleprotein[Corynebacteriumjeikeium]/11657    ---Q-SIPTGRIGMSTIA-DSPIDWDIAFPGSD-----IEV-NL-TKREAFSPRP----H
gi|737006250|ref|WP_035002053.1|damageinducibleprotein[Corynebacteriumjeikeium]/11658    ---Q-SIPTGRIGMSTIA-DSPIDWDIAFPGSD-----IEV-NL-TKREAFSPRP----H
gi|923018168|ref|WP_053412254.1|damageinducibleprotein[Corynebacteriumlactis]_gi|922845184|gb|ALA67504.1|helicase[Corynebacter/    ---Q-IIPTSRIGVAAIA-ESPINWDVAFPGSE-----IQI-NL-SQRETFSPRP----H
gi|923018885|ref|WP_053412970.1|damageinducibleprotein[Corynebacteriumlactis]_gi|922845845|gb|ALA68165.1|helicase[Corynebacter/    ---Q-IIPTSRIGVAAIA-ESPINWDVAFPGSE-----IQI-NL-SQRETFSPRP----H
gi|512464870|ref|WP_016422869.1|hypotheticalprotein[Corynebacteriumsp.HFH0082]_gi|512047830|gb|EPD47505.1|hypotheticalproteinHM    ---Q-LIPTSRIGTAAIA-ESPINWDVAFPGSE-----IQI-NL-SQRETFEPRP----H
gi|881012697|ref|WP_048733406.1|damageinducibleprotein[Corynebacteriumurealyticum]/11670    ---Q-LIPTSRIGTAAIA-ESPINWDVAFPGSE-----IQI-NL-SQRETFEPRP----H
gi|755608478|ref|WP_042530519.1|damageinducibleprotein[Corynebacteriumsingulare]_gi|754340239|gb|AJI78730.1|putativehelicase[C/    ---Q-IIPTSRIGISAIA-ESPINWDVAFPGSEVRDKTIQI-NL-SQRETFEPRP----H
gi|491421796|ref|WP_005279593.1|helicase[Corynebacteriumaccolens]_gi|227077180|gb|EEI15143.1|helicaseCterminaldomainprotein[Co/    ---Q-IIPTSRIGISSIA-ESPINWDVAFPGSEVQDKNIQI-NL-SQRETFEPRP----H
gi|550752286|gb|ERS51451.1|hypotheticalproteinHMPREF1282_00033[Corynebacteriumsp.KPL1856]_gi|550759961|gb|ERS58964.1|hypothetic    ---Q-MIPTSRIGISTIA-ESPINWDVAFPGSEVRDKNIQI-NL-SQRETFEPRP----H
gi|497955053|ref|WP_010269209.1|helicase[Corynebacteriumbovis]/11599    ---Q-AVPVQRFGLAEIA-EAPIDWDIAYPGSG---APVVV-DM-RERQRFALRP----H
gi|860580040|ref|WP_048470569.1|hypotheticalprotein[Mycobacteriumchlorophenolicum]_gi|859098575|gb|KMO76871.1|typeIrestrictione    ---Q-STPVQRINMADIA-ESPINWDIAWPQ-G----NLTI-DL-APAERKEPRP----H
gi|262085905|gb|ACY21873.1|typeIIIrestrictionproteinressubunit[GordoniabronchialisDSM43247]/11632    ---Q-QIPVQRIGLAEIA-ESPIDWDIAWPQ-G----ELQV-EL-TPAVRHQPRP----H
gi|495996338|ref|WP_008720917.1|typeIIIrestrictionendonucleasesubunitR[Rhodococcussp.AW25M09]_gi|443412350|emb|CCQ18455.1|TypeI    ---Q-TIPVQRIGLAEIA-EAPIDWDIAWPQ-G----ELTV-EL-SEAVRNEPRP----H
gi|739284636|ref|WP_037147410.1|damageinducibleprotein[Rhodococcusfascians]/11641    ---Q-TIPVQRIGLAEIA-EAPIDWDIAWPQ-G----ELTV-EL-SEAIRNEPRP----H
gi|739328195|ref|WP_037190574.1|damageinducibleprotein[Rhodococcusfascians]/11643    ---Q-TIPVQRIGLAEIA-EAPIDWDIAWPQ-G----ELTV-EL-SEAVRNEPRP----H
gi|811698335|emb|CMN21041.1|helicase[Mycobacteriumtuberculosis]/11725    ---Q-LVPVQRIGMAEIA-ESPIDWDIAWPA-G----DLQV-NL-TPAKRHELRP----H
gi|801577898|emb|CFK23773.1|helicase[Mycobacteriumtuberculosis]/11675    ---Q-LVPVQRIGMAEIA-ESPIDWDIAWPA-G----DLQV-NL-TPAKRHELRP----H
MTU18II/11606    ---Q-LVPVQRIGMAEIA-ESPIDWDIAWPA-G----DLQV-NL-TPAKRHELRP----H
gi|887719858|emb|CMT27886.1|helicase[Mycobacteriumtuberculosis]/11667    ---Q-LVPVQRIGMAEIA-ESPIDWDIAWPA-G----DLQV-NL-TPAKRHELRP----H
gi|621694743|gb|KAS87156.1|hypotheticalproteinAL70_01801[MycobacteriumtuberculosisTKK_03_0115]/11604    ---Q-LVPVQRIGMAEIA-ESPIDWDIAWPA-G----DLQV-NL-TPAKRHELRP----H
gi|888128072|emb|CLQ84422.1|helicase[Mycobacteriumtuberculosis]_gi|889917090|emb|CLY86893.1|helicase[Mycobacteriumtuberculosis]    ---Q-LVPVQRIGMAEIA-ESPIDWDIAWPA-G----DLQV-NL-TPAKRHELRP----H
gi|884823424|emb|CKL96672.1|helicase[Mycobacteriumtuberculosis]/11466    ---Q-LVPVQRIGMAEIA-ESPIDWDIAWPA-G----DLQV-NL-TPAKRHELRP----H
gi|505103239|ref|WP_015290341.1|hypotheticalprotein[Mycobacteriumcanettii]_gi|432162733|emb|CCK60117.1|Conservedproteinofunknow    ---Q-LIPVQRIGMAEIA-ESPIDWDIVWPA-G----DLQV-NL-TPAKRHELRP----H
gi|505116250|ref|WP_015303352.1|hypotheticalprotein[Mycobacteriumcanettii]_gi|432158726|emb|CCK56026.1|Conservedproteinofunknow    ---Q-LVPVQRIGMAEIA-ESPIDWDIAWPA-G----DLQV-NL-TPAKRHELRP----H
gi|432154735|emb|CCK51974.1|Conservedproteinofunknownfunction(part1)[MycobacteriumcanettiiCIPT140060008]/11588    ---Q-LVPVQRIGMAEIA-ESPIDWDIAWPA-G----DLQV-NL-TPAKRHELRP----H
gi|809008027|ref|WP_046295375.1|hypotheticalprotein[Mycobacteriumsp.UM_Kg17]/11624    ---Q-TKPVQRIGMAEIA-ESPIDWDIAWPA-G----DLHV-DL-SPALKHQPRP----H
gi|808802633|ref|WP_046284139.1|hypotheticalprotein[Mycobacteriumsp.UM_NZ2]/11624    ---Q-TKPVQRIGMAEIA-ESPIDWDIAWPPGG----DLHV-EL-SPALRHQPRP----H
gi|750125774|ref|WP_040429852.1|damageinducibleprotein[Corynebacteriumnuruki]/11668    ---Q-QIPVTRISMKDLE-DSPVSWDIAWPQ-N----SLQV-DL-TPKKPHELRP----H
gi|602237614|gb|EYT60960.1|damageinducibleprotein[Dietziasp.UCDTHP]/11473    ---L-QVSVQRIGTEHLE-AAPIDWDIAWPK-G----ELSI-DL-SPSTPHELRP----H
gi|665897412|ref|WP_031264618.1|damageinducibleprotein[Dietziasp.UCDTHP]_gi|602237599|gb|EYT60945.1|helicase[Dietziasp.UCDTH/11    ---Q-TVSVQRIGTEHLE-AAPIDWDIAWPK-G----ELSI-DL-SPSTPHELRP----H
gi|502903674|ref|WP_013138650.1|helicase[Segniliparusrotundus]_gi|296181291|gb|ADG98197.1|typeIIIrestrictionproteinressubunit[S    ---Q-SKPVGRLSLAELD-GSPIDWRVEWAG-E----DFEV-AI-REAARHNARP----H
gi|780248306|ref|WP_045538930.1|hypotheticalprotein[Gordoniasp.no.9]_gi|778142514|gb|KJR06917.1|hypotheticalproteinUG54_12320[G    ---Q-SKPVQRIGLDVLA-QSPIDWQVEWAD-D----DFTV-GV-EKVTRHSPRE----H
gi|665897368|ref|WP_031264574.1|damageinducibleprotein[Dietziasp.UCDTHP]_gi|602239137|gb|EYT62483.1|helicase[Dietziasp.UCDTH/11    ---Q-TIPVQRISLEDLA-ESAIDWDIAWPQ-G----ELQV-DL-APSAPYTLRP----H
gi|493372923|ref|WP_006329236.1|helicase[Gordoniarhizosphera]_gi|403207917|dbj|GAB88168.1|hypotheticalproteinGORHZ_006_00370[Go    ---Q-TIPVQRIGMAEIA-ESPIDWDIAWPQ-G----ELQV-DL-SEAKRHEPRP----H
gi|764904870|ref|WP_044487821.1|hypotheticalprotein[Mycobacteriumtuberculosis]_gi|625005353|gb|KBZ57120.1|hypotheticalproteinK8    ---Q-TVPVQRIGLAEIA-DSPIDWDIAWVD-G----DLRI-DV-SEATRHEPRP----H
gi|927068212|ref|WP_053778441.1|damageinducibleprotein[Gordoniasp.NB41Y]_gi|926660541|gb|EMP14526.2|damageinducibleprotein[G/11    ---Q-TIPVQRIGLAEIA-DSPIDWDVSLVD-G----EVEV-HL-AEATRHEPRP----H
gi|935514169|ref|WP_054429894.1|damageinducibleprotein[Mycobacteriumimmunogenum]_gi|929801120|gb|KPG29247.1|damageinduciblepr/1    ---Q-TIPVQRIGLAEIA-DSPIDWDVSMVD-G----DLQI-DV-SEASRHEPRQ----H
gi|941317728|ref|WP_055118574.1|hypotheticalprotein[Mycobacteriumperegrinum]/11657    ---Q-TIPVQRIGLAEIA-DSPIDWDVSMVD-G----DLQI-DV-SEATRHEPRQ----H
gi|933683673|gb|ALG86377.1|hypotheticalproteinACH46_20120[Gordoniasp.QH11]/11636    ---Q-RIPVQRIGLTEIA-ESPIDWDIAWPD-G----HLEV-SM-SEATRHEPRP----H
gi|873553947|emb|CRY84110.1|Predictedhelicase[Nocardiafarcinica]/11688    ---Q-TIPVQRIGLAEIA-ESPIDWDIAWPA-G----ELQV-SL-SEAKRHEPRL----H
gi|499525749|ref|WP_011212389.1|helicase[Nocardiafarcinica]_gi|54019339|dbj|BAD60707.1|hypotheticalprotein[NocardiafarcinicaIFM    ---Q-TIPVQRIGLAEIA-ESPIDWDIAWPA-G----ELQV-SL-SEAKRHEPRP----H
gi|640109609|ref|WP_024794187.1|damageinducibleprotein[Tomitellabiformata]/11653    ---Q-VIPVQRIGLAEIA-ESPINWDIARPD-E----DFHV-TL-TEAVKHEPRP----H
gi|640109991|ref|WP_024794334.1|hypotheticalprotein[Tomitellabiformata]/11639    ---Q-VIPVQRIGLAEIA-ESPINWDIARPD-A----DFQV-TL-TEAVRHEPRT----H
```

```
gi|939125496|ref|WP_054831438.1|damageinducibleprotein[Rhodococcuserythropolis]/11669    ---Q-SIPVQRIGLAEIA-ESPIDWDIAWPQ-G----DLQV-NL-SEAKRHEPRL----H
gi|910258550|ref|WP_050062711.1|hypotheticalprotein[Rhodococcussp.RD6.2]_gi|846871967|emb|CRK49225.1|conservedhypotheticalprote    ---Q-SIPVQRIGLAEIA-ESPIDWDIAWPA-G----ELQV-DL-SEAKRHEPRT----H
gi|797148189|gb|KJY52358.1|putativeHelicase[Bifidobacteriumasteroides]/11801    ---E-QFGATIIDSTALD-GDFVDWTPFLPSAT-------------V-RKFSPRP----Y
gi|535476570|gb|EFG27357.2|hypotheticalproteinHMPREF9020_00999[ScardoviainopinataF0304]/11827    ---S-PRNVVVLDSEQLN-NEFVDWSAFLPESH------Y----HNL-KKFEPRP----Y
gi|737482729|ref|WP_035462496.1|hypotheticalprotein[Alicyclobacillusmacrosporangiidus]/11512    ---Q-ERGIAVWTGDDFE-QADVNWERFTWRAS------D--KLVRH-APAVLRP----Y
gi|685434354|gb|KGA95679.1|helicase[BacillusalcalophilusATCC27647=CGMCC1.3604]/11575    ---R-DKPIVRIALSDLQ-ESKIDWSSYSFNNP------D--KVKLQ-DKKTPRP----H
gi|945370253|gb|KQU27251.1|helicase[Bacillussp.Leaf13]/11557    ---R-DKEITRISLTQLK-DSQIDWSEFIFAKP------E--QVKVQ-QKKTPRT----H
gi|880997015|ref|WP_048719116.1|helicase[Enterococcushirae]/11563    ---R-SKPITRIGLVELQ-NSKIDWSKFSFTKP------M--EVKLH-AKKTPRN----H
gi|945356929|gb|KQU14100.1|helicase[Bacillussp.Leaf49]/11547    ---R-DKDIQRISLEQLK-DSQVDWSTFCPEQP------E--EVKLE-DKKTPRP----H
gi|763482876|gb|KIZ53217.1|helicase[Bacillussafensis]/11563    ---R-DKKIVRIGLSQLR-DSEIDWSNFSIDDP------K--TVELK-AVKKPRK----H
gi|852223292|ref|WP_048309707.1|helicase[Anaerobacillusmacyae]_gi|850322910|gb|KMM38590.1|helicase[Anaerobacillusmacyae]/11573    ---R-DKSIARIGLSQLR-DSEIDWSKFSLEKP------K--TVKLK-SSKQPRP----H
gi|739708665|ref|WP_037562619.1|helicase[Sporolactobacillusterrae]/11573    ---R-DKKIARIGLSQLR-DSEIDWSQFSFDKP------L--KIELK-SPKQPRS----H
gi|496687561|ref|WP_009329104.1|MULTISPECIES:helicase[Bacillus]_gi|317390867|gb|EFV71668.1|endonucleaseandmethylaseLlaGI[Bacill    ---R-DKNIARIGLSQLR-ESEIDWSQFSFEKP------Q--TIELK-SPKQPRT----H
gi|930213599|gb|KPH74225.1|helicase[Oceanobacilluscaeni]/11569    ---R-DKNIARIGLSQLR-ESEIDWSQFTFEKP------E--KVALK-SPKEPRT----H
gi|516366003|ref|WP_017756036.1|helicase[Bacillussp.ZYK]/11571    ---R-DKNIARIGLSQLR-DSEIDWSQFSFDKP------H--TIKLK-SPKQPRS----H
gi|488909977|ref|WP_002821052.1|helicase[Oenococcusoeni]_gi|399965230|gb|EJN99855.1|endonucleaseandmethylaseLlaGI[Oenococcusoen    ---T-TKPISRIGLTDLK-NASFDWSQFSFAKT------E--NPIQK-THKAIRH----Y
gi|495019967|ref|WP_007745780.1|helicase[Oenococcuskitaharae]_gi|366983682|gb|EHN59081.1|typeIIIrestrictionenzyme,ressubunit[Oe    ---T-TKPISRIGLADLK-NAHFDWSQFSFAKN------E--NPIQK-THKAIRH----Y
gi|489666119|ref|WP_003570406.1|typeIIIrestrictionendonuclease[Lactobacilluscasei]_gi|410527834|gb|EKQ02696.1|typeIIIrestrictio    ---G-TKEINRIGLSDLR-HSGFDWSQFNFGDS------G--KAIQA-SHKQPRA----Y
gi|511447623|gb|EPC78110.1|superfamilyIIDNA/RNAhelicase[Lactobacillusparacaseisubsp.paracaseiLpp221}/11615    ---G-SKDINRIGLSDLR-HSSFDWSKFSFGDA------T--KAIQA-SHKQPRA----Y
gi|497700411|ref|WP_010014595.1|helicase[Lactobacilluscoryniformis]/11585    ---G-SKDINRIGLSDLK-HSSFDWSQFSFGNA------E--KAITA-SHKQPRE----Y
gi|489784048|ref|WP_003687939.1|helicase[Lactobacillusmali]_gi|394428539|gb|EJF01080.1|superfamilyIIDNA/RNAhelicase[Lactobacill    ---G-SKHITRIGLSDLK-HSSFDWSQFHFGNA------T--KSIQV-SHKQPRG----Y
gi|446780825|ref|WP_000858081.1|helicase[Staphylococcusaureus]_gi|374396604|gb|EHQ67835.1|typeIIIrestrictionenzyme,ressubunit[S    ---Q-TKQVQRIGLSNLR-HSSIDWSIFDFEKP------K--EVVVD-SPKKLRD----Y
gi|510796658|ref|WP_016171456.1|hypotheticalprotein[Enterococcusdispar]_gi|508167532|gb|EOT44083.1|hypotheticalproteinOMK_00227    ---Q-TKQVQLIGLSNLR-HSDIDWSSFNFEKP------K--EVIVK-SHKGLRD----Y
gi|612613473|gb|EZS30411.1|hypotheticalproteinW603_02482[StaphylococcusaureusVET0353R]_gi|612881917|gb|EZU93992.1|hypotheticalp    ---Q-TKQVQLIGLSDLL-HSKIDWSSVNFEKP------K--ETIVK-SHKGIRD----Y
gi|488332893|ref|WP_002402278.1|helicase[Enterococcusfaecalis]_gi|306513864|gb|EFM82466.1|helicaseCterminaldomainprotein[Enter/    ---L-SKPVAQIGLSDLR-NSQVDWESFIPSRP------N--EVKVK-NTKKTRP----Y
gi|921166029|ref|WP_053111806.1|helicase[Enterococcusdurans]_gi|915771660|gb|AKX85928.1|helicase[Enterococcusdurans]/11562    ---L-SKPVAQIGLSDLR-NSQVDWESFIPSRP------N--EVKVK-NVKKTRP----Y
gi|498525358|ref|WP_010825030.1|hypotheticalprotein[Enterococcusfaecalis]_gi|487059631|gb|EOJ86727.1|hypotheticalproteinWOC_005    ---L-SKPVAQIGLSDLR-NSQVDWESFIPSRP------N--EVKVK-NVKKTRP----Y
gi|498412624|ref|WP_010722070.1|hypotheticalprotein[Enterococcusfaecium]_gi|486634654|gb|EOF75253.1|hypotheticalproteinSGC_0253    ---L-SKSVQQIGLSDLR-NSQVDWESFIPSRP------N--EVKVK-NVKKTRP----Y
gi|498408534|ref|WP_010718364.1|hypotheticalprotein[Enterococcusfaecium]_gi|486609608|gb|EOF50873.1|hypotheticalproteinSCW_0257    ---L-SKPVAQIGLSDLR-NSQVDWESFIPSRP------N--EVKVK-NVKKTRP----Y
gi|498407475|ref|WP_010717483.1|hypotheticalprotein[Enterococcusfaecalis]_gi|486594855|gb|EOF36348.1|hypotheticalproteinSCK_005    ---L-SKPVAQIGLSDLR-NSQVDWESFIPSRP------N--EVKVK-NVKKTRP----Y
gi|514885392|ref|WP_016615344.1|helicase[Enterococcusfaecalis]_gi|514505843|gb|EPH71676.1|helicaseprotein[Enterococcusfaecalis0    ---L-SKPVAQIGLSDLR-NSQVDWESFIPSRP------N--EVKVK-NTKKTRP----Y
gi|325157603|gb|EGC69759.1|helicaseCterminaldomainprotein[EnterococcuscasseliflavusATCC12755]/11561    ---L-SKPVAQIGLSDLR-NSQVDWESFIPSRP------N--EVKVK-NVKKTRP----Y
gi|895856206|ref|WP_048967668.1|helicase[Enterococcusfaecalis]/11551    ---L-SKPVAQIGLSDLR-NSQVDWESFIPSRP------N--EVKVK-NVKKTRP----Y
gi|639458836|ref|WP_024636366.1|helicase[Enterococcusfaecium]_gi|658176502|gb|KEI51159.1|helicase[EnterococcusfaeciumUC7256]/1/    ---L-SKPVAQIGLSDLR-NSQVDWESFIPSRP------N--EVKVK-NVKKTRP----Y
gi|488266511|ref|WP_002337719.1|hypotheticalprotein[Enterococcusfaecium]_gi|430581409|gb|ELB19854.1|hypotheticalproteinOIQ_0351    ---L-SKPVAQIGLSDLR-NSQVDWESFIPSRP------N--EVKVK-NVKKTRP----Y
gi|446353356|ref|WP_000431211.1|helicase[Staphylococcusaureus]_gi|253726329|gb|EES95058.1|helicaseCterminaldomainprotein[Staph/    ---N-EKGIEIIGLSDLR-NSQINWADFSFERP------E--QTTIK-QPKSLRP----Y
gi|672712068|emb|CDM14336.1|DNAorRNAhelicaseofsuperfamilyII[StaphylococcusepidermidisPM221]/11464    ---N-QKNIEILGLSDLR-NSQINWSDFNFQRP------E--ELTIK-KPKELRF----Y
gi|896298990|ref|WP_049279905.1|helicase[Staphylococcusaureus]/11571    ---N-QKNIEILGLSDLR-NSQINWSDFNFERP------E--ELTIK-KPKELRF----Y
gi|541903432|gb|ERH36190.1|hypotheticalproteinSEQU_00085[StaphylococcusequorumUMCCNS924]/11578    ---N-QKNIEILGLSDLR-NSQINWSDFNFERP------E--ELTIK-EPKELRF----Y
gi|488289747|ref|WP_002360955.1|helicase[Enterococcusfaecalis]_gi|256682216|gb|EEU21911.1|endonucleaseandmethylaseLlaGI[Enteroc    ---N-EKGIEIIGLSDLR-NSQIDWTKYSFERP------E--AVAVK-EPKKLRS----Y
gi|494392045|ref|WP_007207894.1|helicase[Enterococcusitalicus]_gi|315483798|gb|EFU74284.1|helicaseCterminaldomainprotein[Enter/    ---N-EKGIEIIGLSDLR-NSQIDWTKYRFERP------E--VVTIK-EPKKLRP----Y
gi|446350692|ref|WP_000428547.1|helicase[Bacilluscereus]_gi|228727580|gb|EEL78721.1|RestrictionmodificationsystemLlaBIII[Bacil/    ---N-EKGIEIIGLSDLR-NSQIDWTKFSFKRP------E--VITVK-SPKKLRG----Y
gi|488255302|ref|WP_002326510.1|hypotheticalprotein[Enterococcusfaecium]_gi|430480276|gb|ELA57459.1|hypotheticalproteinOGE_0473    ---N-EKGIELIGLSDLR-NSQIDWTKFSFERP------E--IISIK-SPKKLRS----Y
gi|843253770|ref|WP_047916698.1|helicase[Lactococcuspiscium]_gi|802124677|emb|CEN29502.1|EndonucleaseandmethylaseLlaGI,TypeISPr    ---N-EKGIEIIGLSDLR-NSQIDWSQFNFERP------E--NVVVK-KPKKLRD----Y
LlaBIII/11578    ---N-EKGIEIIGLSDLR-NSQIDWSQFNFERP------E--NVVVK-KPKKLRD----Y
gi|499320531|ref|WP_011011023.1|helicase[Listeriainnocua]_gi|16415805|emb|CAC42019.1|pli0021[ListeriainnocuaClip11262]/11569    ---N-EKGIEIIGLSDLR-NSQIDWSQFNFERP------E--NVVVK-KPKKLRE----Y
gi|760026859|ref|WP_043709973.1|helicase[Weissellacibaria]_gi|758337165|gb|KIU20061.1|UvsWhelicase[Weissellacibaria]/11567    ---N-EKGIEIIGLSDLR-NSQIDWAQFNFERP------E--KVAIK-KTKQLRE----Y
LlaGI/11570    ---N-EKGIEIIGLSDLR-NSQIDWSQFNFERP------E--NVVVK-KPKKLRD----Y
gi|499988414|ref|WP_011669132.1|helicase[Lactococcuslactis]_gi|116109019|gb|ABJ74139.1|DNAorRNAhelicaseofsuperfamilyII[Lactococ    ---N-EKGIEIIGLSDLR-NSQIDWSQFNFERP------E--NVVVK-KPKKLRE----Y
gi|685938360|ref|WP_031670319.1|helicase[Listeriamonocytogenes]_gi|590403387|gb|EXL23261.1|hypotheticalproteinX846_1956[Listeri    ---N-EKGIEIIGLSDLR-NSQIDWSQFNFERP------E--NVVVK-KPKKLRD----Y
gi|530793219|gb|EQC56240.1|helicase[Lactococcuslactissubsp.cremorisTIFN5]_gi|530793247|gb|EQC56263.1|helicase[Lactococcuslactis    ---N-EKGIEIIGLSDLR-NSQIDWSQFNFERP------E--NVVVK-KPKKLRD----Y
gi|695270006|ref|WP_032492075.1|hypotheticalprotein[Listeriamonocytogenes]_gi|695212872|ref|YP_009081522.1|hypotheticalproteinp    ---N-EKGIEIIGLSDLR-NSQIDWSQFNFERP------E--NVVVK-KPKKLRD----Y
gi|504101310|ref|WP_014335297.1|helicase[Leuconostocmesenteroides]_gi|859247905|gb|AKO63885.1|helicase(plasmid)[Leuconostocmese    ---N-EKGIEIIGLSDLR-NSQIDWSQFNFERP------E--KVTIK-KPKQLRG----Y
gi|488905190|ref|WP_002816265.1|helicase[Leuconostocmesenteroides]_gi|227351522|gb|EEJ41777.1|helicaseCterminaldomainprotein[L/    ---N-EKGIEIIGLSDLR-NSQIDWAQFSFERP------E--KVSTK-KQKQLRG----Y
gi|518121285|ref|WP_019291493.1|MULTISPECIES:hypotheticalprotein[Lactococcus]/11588    ---L-SKSISRIGLSDLR-NSQIDWDKFSFVSS------E--KIQVK-QSKKLRD----Y
gi|754857213|ref|WP_042217902.1|helicase[Lactococcusgarvieae]/11549    ---R-S-DVVRIGLSDLR-HSQINWEQWSFAEP------D--KVQVK-SIKDPHN----Y
gi|640048455|ref|WP_024784762.1|helicase[Streptococcusmutans]/11564    ---R-S-DVIRIGLSDLR-HSQIDWEQFSFDRP------E--SVVIK-EKKQPRY----Y
gi|512695361|ref|WP_016476323.1|hypotheticalprotein[Veillonellasp.HPA0037]_gi|512079853|gb|EPD79018.1|hypotheticalproteinHMPREF    ---R-S-DVIRIGISDLK-NSRVDWNKFSFDNP------M--IVSVK-NKKKLRY----Y
gi|636963514|ref|WP_024403747.1|helicase[Streptococcussuis]/11574    ---R-S-DVVRIGLSELR-NSRIDWSKFSFDRP------E--VVTVK-AKKQPRY----Y
gi|881115312|ref|WP_048802949.1|helicase[Staphylococcuspasteuri]/11573    ---R-S-DVVRIGLSDLK-KSRIEWEKYSFDKP------E--EVVIK-AKKKTRS----Y
gi|446063996|ref|WP_000141851.1|helicase[Streptococcusmitis]_gi|307618725|gb|EFN97864.1|endonucleaseandmethylaseLlaGI[Streptoco    ---R-S-DVIRIGLSDLR-NSQIDWSKFSFESP------E--VVAVK-AKKKLRY----Y
gi|488206609|ref|WP_002277817.1|restrictionmodificationsystemLlaBIII[Streptococcusmutans]_gi|449173033|gb|EMB75626.1|restricti/    ---R-S-DVVRIGLSDLR-NSRIDWEQFSFEHP------E--EVKIK-AKKQPRY----Y
gi|640023290|ref|WP_024781621.1|helicase[Streptococcusmutans]/11566    ---R-S-DVVRIGLSDLR-NSRIDWEQFSFERP------E--EVKIK-AKKQPRY----Y
gi|814946581|ref|WP_046393781.1|helicase[Streptococcusuberis]_gi|810600885|gb|KKF57499.1|helicase[StreptococcusuberisB362]/1156    ---R-S-DVIRIGLSDLR-NSCIDWTKFSFEKP------E--VVAVK-AKKEPRY----Y
gi|765470762|ref|WP_044720336.1|helicase[Streptococcussuis]/11559    ---R-H-DVVRIGLSDLR-NSQIDWTQFSFERP------E--QVSVK-AKKSPRY----Y
gi|654507127|ref|WP_027976443.1|helicase[Streptococcusdevriesei]/11558    ---R-S-DIVRVGLSDLR-NSRIDWTKFSFERP------D--EVQVK-AKKQPRY----Y
gi|530812902|gb|EQC72481.1|DNAorRNAhelicaseofsuperfamilyII[Streptococcussp.HSISS2]/11570    ---R-S-DVIRIGLSDLR-SSRIDWTKFSFDRP------E--EVTVK-AKKKPRY----Y
```

```
gi|489158846|ref|WP_003068504.1|helicase[Streptococcusintermedius]_gi|355367114|gb|EHG14827.1|hypotheticalproteinHMPREF9682_002      ---R-S-DVIRIGLSDLR-NSRIDWEQFTFERP------D--EVKTK-AKKQPRY----Y
RM.Lru27782ORF13200P/11620                                                                                                          ---L-SKPIARIGLTDLE-NADIDWGMFDFGSA------DA-ID-KK-PAKKLRD----Y
gi|492676546|ref|WP_005919236.1|DNAphosphorothioationsystemrestrictionenzyme[Pediococcusacidilactici]_gi|482517924|gb|EOA08103.     ---L-SKPMTRIGLAQLE-HANIDWELFSFDQN------ND-HL-RK-RAKKLRD----Y
gi|498183116|ref|WP_010497272.1|helicase[Lactobacillusacidipiscis]/11563                                                            ---L-SKPITRIGLSQLQ-NADVNWELFDFDHE------NK-DL-GK-GAKKLRD----Y
gi|822100485|ref|WP_046921865.1|helicase[Lactobacillusruminis]_gi|821684442|gb|KLA43321.1|restrictionmodificationsystemLlaBIII/     ---L-TKEISVVGLSQLQ-NANFDWQLFDFNSR------N--NL-TM-KPKKLRD----Y
gi|935565832|ref|WP_054450311.1|helicase[Lactobacilluskunkeei]/11573                                                                ---T-SKPFSIIGLTQLK-NSSVDWGKFSFSED-------N-DLSIK-KPKKLRG----Y
gi|752296887|ref|WP_041152901.1|helicase[Lactobacilluskunkeei]_gi|751663520|gb|KIM18358.1|helicase[Lactobacilluskunkeei]/11577      ---T-SKPFSIIGLTQLK-NSSVDWSKFSFSED-------N-DLSIK-KSKKLRG----Y
gi|495745291|ref|WP_008469870.1|helicase[Lactobacillushominis]_gi|394484442|emb|CCI81207.1|RestrictionmodificationsystemLlaBII/     ---T-TKPFTRIGLNQLK-HANFDWEKFSFAKE------NK-NLSVK-KPKKLRP----Y
gi|918672414|ref|WP_052543134.1|helicase[Lactobacillussp.]/11564                                                                    ---T-TKPFTVIGLSQLR-HADFDWQKFSFAKE------NT-NLSKK-KPKKLRP----Y
gi|799141631|ref|WP_045927261.1|helicase[Lactobacilluskullabergensis]_gi|797155335|gb|KJY59264.1|putativerestrictionendonucleas     ---Q-TKPFTTIGLSQLR-NAHFQWQKFSFSKE------NK-NLSTK-KKNQPRD----Y
gi|504379990|ref|WP_014567092.1|helicase[Lactobacillusjohnsonii]_gi|329666726|gb|AEB92674.1|putativerestrictionendonuclease[Lac     ---T-TKPFSIIGLSQLR-HAHFSWQKFNFAKE------NT-DLSNK-VIKKIRD----Y
gi|489880179|ref|WP_003783646.1|DNAdamageinducibleprotein[Actinomycesnaeslundii]_gi|399901561|gb|EJN84440.1|typeIIrestriction/     ---Q-AIPVSRIGLEDLF-NSTIDWDSFSLETP------EV--MVRT-GGKSLRE----H
gi|496524497|ref|WP_009232780.1|DNAdamageinducibleprotein[Actinomycessp.oraltaxon849]_gi|365265413|gb|EHM95177.1|hypotheticalp/     ---Q-AISVSRVGLEDLF-NSTIDWDSFSLETP------EV--MVRT-GGKSLRE----H
gi|915397782|ref|WP_050783167.1|damageinducibleprotein[Actinomycesoris]/11694                                                       ---Q-AIPVSRVGLEDLF-NSTIDWDSFSLETP------EV--MVRT-GGKSLRE----H
gi|502925248|ref|WP_013160224.1|helicase[Propionibacteriumfreudenreichii]_gi|296921272|emb|CBL55824.1|helicase[Propionibacteriu     ---Q-SIPVQRISLADIA-ESPVDWMQQSKGSL------DF-EPQKA-TRYGLRP----H
gi|732164352|emb|CEG87481.1|helicase[Propionibacteriumfreudenreichii]/11618                                                         ---Q-QIPVERIGLADIA-ESPIDWMAERPREL------VF-RGRRA-VRYSLRP----H
gi|739047402|ref|WP_036925058.1|damageinducibleprotein[Propionibacteriumthoenii]/11596                                              ---Q-SVPVQRIGMADIA-ESRVDWMFHSDDPL------GF-TARKA-VRFGLRP----H
gi|739112839|ref|WP_036982533.1|damageinducibleprotein[Propionibacteriumjensenii]/11594                                             ---Q-SVPVQRIGMADIA-ESRVDWMFHADDPL------GF-TPRKA-VRFGLRP----H
gi|918023558|ref|WP_052314121.1|damageinducibleprotein[Nocardiathailandica]/11612                                                   -------VAQRVDISCLD-DARVDWSRYSWSKP------DA--VELA-TKKQPHP----Y
gi|498380271|ref|WP_010694427.1|DNAdamageinducibleprotein[Saccharopolysporaspinosa]/11605                                          ---L-SVPVQRIDIRYLD-DANIDWSQFSWSTP------EV--VVST-GKKSLRP----H
gi|749991459|ref|WP_040339809.1|damageinducibleprotein[CandidatusBlastococcusmassiliensis]/11597                                    ---G---VAQRVDIGYLA-DAAIDWTQFSWTTP------EV--VVPT-GKKALRP----H
gi|754177801|ref|WP_041796231.1|damageinducibleprotein[Modestobactermarinus]/11592                                                  ---G---VAQRVDIGYLA-DAAIDWDQFSWTTP------EI--VVPT-GKKALRP----H
gi|504188248|ref|WP_014375350.1|DNAdamageinducibleprotein[Blastococcussaxobsidens]_gi|378782790|emb|CCG02456.1|putativeHelicas/     ---G---VAQRVDIGYLA-DAAIDWGQFSWTTP------EI--VVPT-GKKALRP----H
gi|946476713|gb|KRF26798.1|damageinducibleprotein[Phycicoccussp.Soil803]/11609                                                     ---L-TIPVTRIGVNDLL-ESKVDWSQIDWQEP------QT-YLPLT-GKKQLRP----H
gi|700176672|gb|KGN37509.1|DNAdamageinducibleprotein[KnoelliasubterraneaKCTC19937]/11633                                           ---L-TVPVTRIGINDLL-ESKVDWSQIDWQAP------QT-YLPLT-GKNQLRP----H
gi|700168841|gb|KGN29914.1|hypotheticalproteinN802_10215[KnoelliasinensisKCTC19936]/11631                                          ---L-TVPVTRIGINDLL-ESKVDWSQIDWQEP------QT-YLPLS-GKKQLRP----H
gi|946426241|gb|KRE76610.1|damageinducibleprotein[Arthrobactersp.Soil763]/11606                                                    ---Q-QVPVRRIGMSDLE-ASPVDWEQFSIETP------EV--LVTR-GRKSPKL----H
gi|517318525|ref|WP_018500927.1|hypotheticalprotein[Frankiasp.BCU110501]/11592                                                     ---Q-LIPVRRIGLADLE-ASRVDWSIFSLDKP------DQ--MTLA-SQKAPRK----H
gi|499072429|ref|WP_010851327.1|Helicase[Tetrasphaeraelongata]_gi|478756579|emb|CCH71501.1|putativeHelicase[Tetrasphaeraelongat     ---Q-QVPVRRIGLADLE-ASTIDWDAFDWATP------EA--LPVT-DKKKLRA----H
gi|750080666|ref|WP_040385350.1|damageinducibleprotein[Demetriaterragena]/11625                                                     ---Q-QVPVTRVGMADLE-HSVVNWDDFDWKLP------QV--LSKL-AKKKLRP----H
gi|752558292|ref|WP_041229523.1|damageinducibleprotein[Dermacoccusnishinomiyaensis]_gi|664689315|gb|AIF41904.1|damageinducibl/1     ---Q-AIPVRRIGLSDLA-NSTIDWTQFSWSQP------EV--LPVG-DKKQPRK----H
gi|829089213|ref|WP_047310701.1|damageinducibleprotein[Dermacoccussp.PE3]_gi|828384812|gb|KLO63770.1|damageinducibleprotein[D/1     ---Q-AVAVRRISLADLQ-ESNVDWSKFSWSAP------GT--LPTV-GKKNPRP----H
gi|748756635|ref|WP_040014750.1|damageinducibleprotein[Dermacoccussp.Ellin185]/11596                                                ---Q-TVKVRRISLADLQ-NSNVDWSKFTWSAP------DS--LPTV-GKKTPRP----H
gi|916534724|ref|WP_051141848.1|damageinducibleprotein[Actinomycesneuii]/11632                                                      ---Q-QIPVTRVGLTDFR-TSNIDWSTFQKSDP------SA-GV-AVLPQKTLRN----H
gi|921140030|ref|WP_053090276.1|damageinducibleprotein[Rothiamucilaginosa]/11616                                                    ---Q-RFPVQRMGLQTFR-DSNIDWSTYSLDDP------GE-QQVGYKPHKELRP----H
gi|896406751|ref|WP_049343749.1|damageinducibleprotein[Rothiamucilaginosa]/11647                                                    ---Q-RFPVQRMGLQTFR-DSNIDWSTYSLDDP------GE-QQVGYKPHKELRP----H
gi|723608234|gb|AIY01610.1|putativehelicase[Arthrobactersp.PAMC25486]/11613                                                        ---R-YVPVQRVGLSDFR-HSNIDWATYDLERP------EE--L-KVFDKKTLRT----H
gi|946394527|gb|KRE45160.1|damageinducibleprotein[Arthrobactersp.Soil736]/11616                                                    ---Q-HIPVQRIGLSDFR-HSNIDWSTYELTDP------ST-AP-KLHEKKQLRP----H
gi|515765304|ref|WP_017197904.1|hypotheticalprotein[Arthrobactersp.M2012083]/11648                                                  ---Q-HIPIQRIGLTDFR-NSNIDWATYELTDP------SK-AP-KLHDKKQLRT----H
gi|945315587|gb|KQT73127.1|damageinducibleprotein[Microbacteriumsp.Leaf436]/11612                                                  ---Q-SIPVERLRVQDLD-DSSIDWSQFVWNEP------TK-LE-KK-RLKTPFD----Y
gi|759908497|ref|WP_043594599.1|damageinducibleprotein[Frigoribacteriumsp.MEB024]_gi|758271359|gb|KIU03346.1|damageinduciblep/1     ---Q-QITTLRLGVDDFD-QSDIDWSTYSFSKP------DE-VA-AP-KIKTLRK----H
gi|945072850|gb|KQR34237.1|hypotheticalproteinASF75_00055[Curtobacteriumsp.Leaf154]/11597                                          ---Q-QLPTAQIPIEEFE-HSGIDWSEYSFDHP------DS-LA-AP-VQKSLRP----H
gi|551263152|ref|WP_022883363.1|DNAdamageinducibleprotein[Glaciibactersuperstes]/11621                                             ---Q-RVKTWRVGIDELA-QSDIDWSRYSLAKP------DE-LV-AP-TLKQLRP----H
gi|945452341|gb|KQV08491.1|hypotheticalproteinASC63_10170[Leifsoniasp.Root112D2]/11623                                             ---Q-QIDTFRIGIDQLE-ASDIDWNRFRLDEP------EE-MR-RE-PLKDLRE----H
gi|742759483|gb|KIC69912.1|damageinducibleprotein[Kocuriarhizophila]/11610                                                         ---L-DKPWSTVGLAQLA-DAPIDWSQFNLDQP------DH-MV-RTDEGKSPRP----Y
gi|737919995|ref|WP_035885304.1|damageinducibleprotein[Kocuriarhizophila]/11611                                                     ---L-DKPWSTVGLAQLA-DAPIDWSQFNLDQP------DH-MV-RTDEGKSPRP----H
gi|737997701|ref|WP_035959325.1|damageinducibleprotein[Kocuriamarina]_gi|725823884|gb|KHE75603.1|damageinducibleprotein[Kocur/1     ---L-DKPWSTVGLDQLA-NAPIDWSQFNLDQP------EH-MV-RTDEGKSPRP----H
gi|664521070|ref|WP_031037650.1|damageinducibleprotein[Streptomycessp.NRRLWC3774]/11624                                             ---Q-QIPVTRLGLSDIA-NSPVEWRLPAVDEP------IE-LT-LQ-KKKEPRK----H
gi|500101034|ref|WP_011777041.1|DNAdamageinducibleprotein[Arthrobacteraurescens]_gi|119951547|gb|ABM10457.1|putativeHelicase[A/     ---Q-SKPVSRLRFQDLE-DSTIDWSTFDLDQP------EE-ME-RK-DKKRPRP----H
gi|916871953|ref|WP_051479009.1|damageinducibleprotein[Arthrobactersp.H5]/11588                                                     ---Q-SKAINRLRFGELE-QSTIDWNTFDLDQP------EE-ME-RK-DKKRPRP----H
gi|612084649|gb|EZP43369.1|TypeIIIrestrictionenzyme,ressubunit[Micrococcusluteus]/11625                                            ---Q-SKDVTRVGIQTLV-DSTVDWSEFTFDRP------EE-LT-QR-PRKEPRR----Y
gi|759739708|ref|WP_043450422.1|damageinducibleprotein[Arthrobacterphenanthrenivorans]_gi|742758264|gb|KIC68716.1|damageinduc/1     ---Q-SKPMTRLRFADLA-DSTIDWAEFSIDAP------EQ-MK-QL-GGKEPRK----Y
gi|636845448|ref|WP_024366823.1|damageinducibleprotein[Arthrobactersp.TB26]/11605                                                  ---Q-SKPMTRLRLQDLA-DSTIDWAEFDLDRP------EE-MR-QI-DHKELRK----Y
gi|500009025|ref|WP_011689743.1|DNAdamageinducibleprotein[Arthrobactersp.FB24]_gi|116613051|gb|ABK05760.1|typeIIrestrictionen/     ---Q-SKPMTRLRLQDLA-DSTIDWAEFDLDRP------EE-MR-QI-DRKEPRK----Y
gi|821249847|gb|KKZ12083.1|hypotheticalproteinTQ37_06090[CandidatusSynechococcusspongiarum15L]/11436                               ---Q-EKPVQRLGLHDLK-ASPVCWAEFVQSG--------G-IS-ERKPPRTPRP----H
gi|739446902|ref|WP_037306885.1|damageinducibleprotein[Ruegeriahalocynthiae]/11592                                                  ---Q-SIPVIRVGLDALR-ESAIDWSVFEAS--------EE-IK-LH-DKKQLRS----H
gi|640620081|ref|WP_025048131.1|damageinducibleprotein[Sulfitobactermediterraneus]_gi|751956650|gb|KIN77213.1|TypeIIIrestricti/     ---Q-SIPVVRVGLDALR-DSAIDWSVYEAS--------DK-VQ-LR-NKKQLHS----H
gi|740247634|ref|WP_038088583.1|damageinducibleprotein[Thiobacillusprosperus]_gi|684049748|gb|KFZ90853.1|putativehelicase[Thio/     ---Q-P-NFGILYREDLA-EAPVDWSELDVLKP------EQ-MK-RR-TRKHLRE----H
gi|518420321|ref|WP_019590528.1|hypotheticalprotein[Thioalkalivibriosp.ALE20]/11603                                                ---Q-TIPVTRLFFKDLE-ESPIDWTVFQLDRP------QD-LR-LQ-VKNEVRP----H
gi|500156826|ref|WP_011831496.1|DNAdamageinducibleprotein[Methylibiumpetroleiphilum]_gi|124261892|gb|ABM96881.1|adeninespecifi/     ---Q-VTPVSRLWFKDLA-NSPIDWAKLDLARP------DQ-LR-LV-ARHETRP----H
gi|769978537|ref|WP_045094522.1|damageinducibleprotein[Legionellafallonii]_gi|698841736|emb|CEG55682.1|Putativeproteinwithboxh/     ---Q-SIPVARIWFKDLE-KSPIAWEKFELSKI------KD-LH-LK-PKKTLLE----H
gi|553752541|ref|WP_023085344.1|hypotheticalprotein[Pseudomonasaeruginosa]_gi|552201952|gb|ERU62653.1|hypotheticalproteinQ088_0     ---Q-TIPVSRLWFKDLA-DSPVDWSLFDLARV------SD-IR-LQ-KKKDLMP----H
gi|880980418|ref|WP_048704461.1|damageinducibleprotein[Thauerahumireducens]_gi|875132785|gb|KMU70944.1|damageinducibleprotein/1     ---Q-TIPVSRLWFKDLA-ESPIDWSQFSLERI------QD-IR-LR-EKKTLMP----H
gi|740135732|ref|WP_037982815.1|damageinducibleprotein[Thauerasp.27]/11603                                                         ---Q-TIPVSRLWFKDLA-ESPIDWRQFSLERI------QD-IR-LR-DKKTLLP----H
gi|738017371|ref|WP_035978332.1|damageinducibleprotein[Burkholderiaglumae]/11380                                                    ---Q-TIPVTRLWFKDLA-GSPIDWAQFSLSNI------KD-IR-LK-RRNDLRD----Y
gi|498506003|ref|WP_010806671.1|typeIIIrestrictionenzyme,ressubunit[Pandoraeasp.SD62]_gi|490314561|gb|EON12879.1|typeIIIrestri/     ---Q-TIPVTRLWFKDLA-DSPIDWAQFSLSNI------KN-IK-LK-KQKDLRD----Y
gi|500117748|ref|WP_011793753.1|DNAdamageinducibleprotein[Acidovoraxcitrulli]_gi|120587742|gb|ABM31182.1|typeIIrestrictionenz/      ---Q-VISVSRLWFKELA-ESPIDWSQFSLSNI------KD-IK-LK-KKKQPRE----H
```

```
gi|651617328|ref|WP_026611444.1|damageinducibleprotein[Methylocaldumszegediense]/11601    ---Q-SIPVSRLWFKDLA-DSPIDWSQFSLSNI------KD-IR-LK-PKKELRE----H
gi|737210208|ref|WP_035194994.1|damageinducibleprotein[Acidithiobacillusferrivorans]_gi|669953220|emb|CDQ11755.1|Adeninespecif/    ---Q-QIRVRRIGLADLE-NSVVDWSAFDVEKP------ES-LR-RK-TGKQIRP----H
gi|935697386|ref|WP_054506180.1|damageinducibleprotein[Achromobactersp.]_gi|928716153|emb|CUK12836.1|Hefnuclease[Achromobacter/    ---Q-TIPAELLTLEDLD-DAPIDWSQFSLEKP------EL-LR-KR-PRKTPLQ----H
gi|429502517|gb|ELA00827.1|adeninespecificDNAmethyltransferase[Cupriavidussp.HMR1]/11599    ---Q-TIPAELLTLEDLD-EAPIDWSQFSLDKP------EQ-LR-KR-PKKTPLP----H
gi|506356642|ref|WP_015876361.1|DNAdamageinducibleprotein[Burkholderiaglumae]_gi|237877158|gb|ACR29491.1|AdeninespecificDNAmet/    ---Q-TIPANLLSLEDLE-ASPIDWTQFSLEKP------DQ-LR-KL-PRKTPLP----H
gi|696406787|ref|WP_032978003.1|damageinducibleprotein[Bordetellahinzii]_gi|625335076|gb|KCB49343.1|typeIIIrestrictionenzyme,r/    ---Q-TIPVELMTLEDLE-ASPIDWSQFSLDKP------DQ-LR-KL-PKKAPRP----H
gi|817529125|gb|KKO72587.1|damageinducibleprotein[Kerstersiagyiorum]/11592    ---Q-TIPVELMTLEDLE-ASPIDWSQFSLDKP------DQ-LR-KL-PKKAPRP----H
gi|737048910|ref|WP_035044118.1|damageinducibleprotein[Aquabacteriumsp.NJ1]_gi|699052896|gb|KGM38706.1|damageinducibleprotein/1    ---Q-TIPVEMMTLEDLE-ASPIDWSHFSIEKP------EH-LR-KL-PKKGPRD----H
gi|822492519|ref|WP_046933319.1|MULTISPECIES:damageinducibleprotein[Xanthomonas]_gi|821703525|gb|KLA61196.1|damageinduciblepr/1    ---Q-TIPVEMMTLEDLD-ASPIDWSQFSLDKP------DQ-LR-KL-PKKDPRE----H
gi|751252861|ref|WP_040963740.1|damageinducibleprotein[Xanthomonasfuscans]_gi|749808363|gb|KIJ03887.1|damageinducibleprotein[/1    ---Q-TIPVEMMTLEDLE-ASPIDWSQFSIEKP------DQ-LR-KL-PKKDPRE----H
gi|496774980|ref|WP_009368792.1|DNAdamageinducibleprotein[Bilophilasp.4_1_30]_gi|345039902|gb|EGW44205.1|hypotheticalproteinHM/    ---Q-TIPVSLITLEDME-EAPIDWTAFSLDKP------DE-LK-CSTVKKTPRP----H
gi|499241912|ref|WP_010939452.1|DNAdamageinducibleprotein[Desulfovibriovulgaris]_gi|46580580|ref|YP_011388.1|adeninespecificDN/    ---Q-TIPVALLTLEDLA-SAPIDWTQCTIAAP------DT-LK-PI-EHKKPRP----H
gi|504274913|ref|WP_014462015.1|DNAdamageinducibleprotein[Bacillusmegaterium]_gi|345446919|gb|AEN91936.1|AdeninespecificDNAmet/    ---Q-SKPITRIRLADLA-KSPIDWSKFSFSNL------EN-MK-LK-PKKVLRK----H
gi|504895966|ref|WP_015083068.1|typeIIrestrictionenzymeandmethyltransferasesubtypeG[Anabaenasp.90]_gi|413972424|gb|AFW96512.1|t    ---Q-QIPVILATIHDLA-NSPIDWDKFSFKNP------DH-LE-LK-PKKQIRP----H
gi|518327765|ref|WP_019497972.1|hypotheticalprotein[Calothrixsp.PCC7103]/11600    ---Q-HIPVARLRVQDLA-QSPVDWSQFNLKRP------QD-IK-LK-PKKQIRP----H
gi|652765503|ref|WP_027079917.1|damageinducibleprotein[Luteimonasmephitis]/11593    ---Q-QLPVTRLRVQDLD-ESSVDWSKFSLAKP------DV-VK-LR-TKNKLRK----H
gi|896539951|ref|WP_049442123.1|damageinducibleprotein[Stenotrophomonasmaltophilia]/11591    ---Q-QIPCARLRVADLE-DSAVDWSKFSLAKP------DQ-VV-LR-TKKKPFK----H
gi|500217900|ref|WP_011888043.1|DNAdamageinducibleprotein[Geobacillusthermodenitrificans]_gi|134267954|gb|ABO68149.1|Conserved/    ---Q-QIETTILDMDKLE-QSTIDWGLFSWNRP------DA-LP-VR-EKKELRE----H
gi|696570298|ref|WP_033102014.1|damageinducibleprotein[Thermoactinomycesdaqus]/11594    ---Q-QIPVTILDMEALE-KSSIDWSRFSWNSP------QN-LQ-PR-KKKKLRP----H
gi|251772067|gb|EES52637.1|putativehelicase[Leptospirillumferrodiazotrophum]/11618    ---Q-KIKVNRIGLSGLE-ASLIDWSRIVPEEP------ER-LF-FR-PKKFLLS----H
gi|738124101|ref|WP_036082108.1|damageinducibleprotein[Leptospirillumferriphilum]_gi|685243306|gb|KGA94177.1|putativehelicase[/    ---Q-KIPVNRIGLGDLE-ASLIDWNRIVPEEP------ER-MF-FR-PKKSLFP----H
gi|558699223|ref|WP_023524766.1|helicase[Leptospirillumsp.GroupII'CF1']_gi|206603764|gb|EDZ40244.1|Putativehelicase[Leptospiri/    ---Q-KIKVNRIGLADLE-SSLIDWTRIVPTEP------ER-LF-FR-KKKSLLP----H
gi|406774462|gb|AFS53887.1|putativehelicase[LeptospirillumferriphilumML04]/11610    ---Q-QIPINRIGLSDLA-DSRIDWSRVVPDHY------DI-LS-LH-SRKSLLS----H
gi|740719463|ref|WP_038504749.1|damageinducibleprotein[Leptospirillumferriphilum]_gi|640838125|gb|AIA30173.1|damageinduciblep/1    ---Q-QIPTNRIGLSDLA-DSRIDWSRVVPDHY------DI-LP-LR-SRKSLLS----H
gi|517810766|ref|WP_018980974.1|hypotheticalprotein[Saccharibacterfloricola]/11733    ---N---RLIIVDQQGLE-ESGFDWAHYVPEEA------LL-T-EGAREPLSLRA----Y
gi|502471675|ref|WP_012798533.1|helicase[Slackiaheliotrinireducens]_gi|256791761|gb|ACV22431.1|predictedhelicase[Slackiaheliotr    ---W---STVRLFPSEMD-QAEIDWQPFIEGKE------I-----AERSVYDPLP----H
gi|657871713|ref|WP_029576297.1|hypotheticalprotein[Bifidobacteriumthermacidophilum]_gi|673007521|gb|KFJ02535.1|helicase[Bifido    -----DTELTRVDYETMR-DANVDWDAFIHNKQ------A-----VHRTTFDPRP----H
gi|673004757|gb|KFI99791.1|DNAhelicaserestrictionenzymetypeIIIRessubunit[Bifidobacteriumthermacidophilumsubsp.porcinum]/11682    -----DTELTRVDYETMR-DANVDWDAFIHNKQ------A-----AHRTTFDPRP----H
gi|917316092|ref|WP_051922804.1|hypotheticalprotein[Bifidobacteriumstellenboschense]_gi|673003774|gb|KFI98812.1|helicase[Bifido    ---HPGMDMVRLDLDTMR-ESNLDWGAFIDGTE------S-----STRVTYDPRP----H
gi|705415694|ref|WP_033502332.1|hypotheticalprotein[Bifidobacteriumpseudolongum]_gi|672982411|gb|KFI77685.1|DNAdamageinducible/    ---YPDRDIVRLDLDTMR-HANLDWDAFEAGSR------S-----GARRTYDPRP----H
gi|916591418|ref|WP_051198509.1|hypotheticalprotein[Bifidobacteriumsp.AGR2158]/11704    ---SP-KDIVRLDLDTMK-DSNLDWHAFLRGEK------P-----NERETFDPRP----H
gi|772242982|gb|AJW78255.1|hypotheticalproteinVO01_03165[Clavibactermichiganensissubsp.insidiosus]/11642    ---R-TKPVRRVNLEELR-HCAVDWSSYELKYP------AK-A-PARHARKTERA----H
gi|916649818|ref|WP_051256909.1|damageinducibleprotein[Actinomycesgraevenitzii]/11677    ---Q-HIPVSRITLADLR-DSDIDWRTYSLGSR-------Q-A-PTTRERKVPRD----H
gi|496504578|ref|WP_009212861.1|helicase[Actinomycessp.oraltaxon180]_gi|315312695|gb|EFU60776.1|helicase[Actinomycessp.oraltaxo    ---Q-QIPVSRITLADLR-DSDIDWRTYSLGST-------Q-A-PKTRERKVPRD----H
gi|494862206|ref|WP_007588306.1|helicase[Actinomycessp.ICM39]_gi|398362799|gb|EJN46470.1|typeIIIrestrictionenzyme,ressubunit[Ac    ---Q-QIPVSRITLADLR-DSDIDWRTYSLGST-------Q-A-PKTRERKVPRD----H
gi|881057111|ref|WP_048774974.1|damageinducibleprotein[Sanguibacterkeddieii]/11697    ---Q-QIPVSRITLADLR-DSDIDWRTYSLGST-------Q-A-PKTRERKVPRD----H
gi|881020459|ref|WP_048740808.1|damageinducibleprotein[Kytococcussedentarius]/11704    ---Q-QIPVSRITLADLR-DSDIDWRTYSLGST-------Q-A-PKTRERKVPRD----H
gi|881052313|ref|WP_048770555.1|damageinducibleprotein[Cellulomonasflavigena]/11705    ---Q-QIPVSRITLADLR-DSDIDWRTYSLGST-------Q-A-PKTRERKVPRD----H
gi|490108682|ref|WP_004009424.1|helicase[Mobiluncuscurtisii]_gi|315490465|gb|EFU80089.1|helicaseCterminaldomainprotein[Mobilun/    ---Q-TKPVCRIGLTDLR-NSDIDWSTYSFEKP------QQ-A-PKTKSRKTLRD----H
gi|490111935|ref|WP_004012603.1|helicase[Mobiluncusmulieris]_gi|227843812|gb|EEJ53982.1|helicaseCterminaldomainprotein[Mobilun/    ---Q-SKPVTRIGLTDLR-ASNIDWKTYDFATP------EL-S-PTLQAHKRTLA----H
gi|490114228|ref|WP_004014848.1|helicase[Mobiluncusmulieris]_gi|269934432|gb|EEZ90993.1|helicaseCterminaldomainprotein[Mobilun/    ---Q-SKPVTRIGLTDLR-ASNIDWKTYDFATP------EL-S-PTLQAHKRTLA----H
gi|493333551|ref|WP_006290636.1|helicase[Parascardoviadenticolens]_gi|315119487|gb|EFT82620.1|helicaseCterminaldomainprotein[P/    ---K-GLETHIIGLQTMS-ESNLDWSNLFTDRK------AE-T-----QTFDPRP----H
gi|495817067|ref|WP_008541646.1|helicase,partial[Sutterellaparvirubra]_gi|378598583|gb|EHY31712.1|helicaseprotein,partial[Sutte    ---Q-AIPVTLITRRDLA-ASVIDWSVYAE--S------GK-V-ETRP-KRVPRK----Y
gi|491572696|ref|WP_005430278.1|helicase[Sutterellawadsworthensis]_gi|319805116|gb|EFW01943.1|DNAhelicaserestrictionenzymeTypeI    ---V-TPPIQLVRRSMLA-SSRINWSVYLN--T------GK-V-VQQK-KRTPRK----Y
gi|748686321|ref|WP_039944445.1|hypotheticalprotein[Thermicanusaegyptius]/11573    ---Q-EKEVTVITLYDIVNNYGIDWESFQWHNP------EN-I-TYEK-KKNLRP----Y
gi|916875698|ref|WP_051482754.1|hypotheticalprotein[Thioalkalivibriosp.HK1]/11366    ---V-QPPCTVIDYTTLA-NSQVRWPDLTQDEP------ES-IIADSAAKHRLRA----H
gi|821248604|gb|KKZ10960.1|hypotheticalproteinTE42_09075[CandidatusSynechococcusspongiarumSP3]/11598    ---Q-EKPVQRMGLHDLK-ASPVCWAEFVQ--S------GA-I-SGREPAKTLRP----H
gi|737409843|ref|WP_035390956.1|hypotheticalprotein[Ferrimicrobiumacidiphilum]_gi|765544266|gb|KJE76241.1|typeIrestrictionenzym    ---Q-RPPVQRLGLSDLL-ESPIDWSKFDPTIP------DR-L-LKLS-VRTLLP----H
gi|654906925|ref|WP_028358281.1|damageinducibleprotein[Brackiellaoedipodis]/11679    ---Q-NTPVSMISYTDLE-RSVIDWRQFSPNQE------E--V-VCKP-KFELRS----H
gi|502809901|ref|WP_013044877.1|hypotheticalprotein[CandidatusPuniceispirillummarinum]_gi|292663146|gb|ADE38247.1|hypotheticalp    ---Q-DKPTNRMRMADLA-ELAIDWDIHKPKQ----------T-KLDITKYTLNG----R
gi|251771370|gb|EES51950.1|typeIIIrestrictionenzyme,ressubunit[Leptospirillumferrodiazotrophum]/11628    ---Q-SIPSTRINLVNLE-ESGVDWGQFVSP-D------AP-L-PLLP-KKSLRP----Y
gi|498328480|ref|WP_010642636.1|DNAdamageinducibleprotein[Acidithiobacillusthiooxidans]/11627    ---I--DDLKVLPLRNME-AAPIDWEKFSWGAP------EA-L-TRTP-GKSLRE----H
gi|647633864|ref|WP_025906698.1|damageinducibleprotein[Prochlorococcussp.scB245a_519B7]/11622    ---I-NKDYLRIRTSDLD-GSRIDWLKYVK--E------NK-I-SFHK-KKDKFD----H
gi|647619599|ref|WP_025893729.1|damageinducibleprotein[Prochlorococcussp.scB241_527N11]/11621    ---L-SQDYIRIQSSEIE-KSRIDWYSYIN--D------GS-V-NFSS-KKELKD----H
gi|755034887|ref|WP_042387498.1|hypotheticalprotein[Clostridiumsp.CL6]/11606    ---Q-NPPVRRIGMVDLL-ESEIDWKKFSI--N------NE-I-QLKE-KYKPKP----H
gi|498139798|ref|WP_010453954.1|helicase[SuccinivibrionaceaebacteriumWG1]/11662    ---Q-RIDCHYIDINDLE-ASAVDWEAILDGHF------GE-K-ARLS-PKTLMP----H
gi|527501386|gb|EPT34571.1|typeIIIrestrictionenzyme,ressubunit[Bacteroidetesbacteriumoraltaxon272str.F0290]/11789    ---Q-LIPFTRINQFELR-NAAVDWEKLNSGQQ------GQ-N-AVLP-GKSPRA----H
gi|496047275|ref|WP_008771782.1|helicase[Bacteroidessp.2_1_33B]_gi|262296443|gb|EEY84373.1|helicaseCterminaldomainprotein[Bact/    ---Q-DISVTRINMHELE-ASPVDWDKLLKGDT------GK-A-ALRE-GKQPRK----H
PDI8503III/11664    ---Q-DISVTRINMHELE-ASPVDWDKLLKGDT------GK-A-ALRE-GKQPRK----H
gi|738719431|ref|WP_036613715.1|helicase[Parabacteroidesdistasonis]_gi|649542518|gb|KDS49387.1|restrictionendonucleasefamilypro    ---Q-DIPVTRINMHELE-ASPVDWDKLLKGDT------GK-A-ALRE-GKQPRK----H
gi|659417033|gb|KEJ86133.1|hypotheticalproteinHMPREF1002_00341[Porphyromonassp.31_2]/11656    ---Q-DISVTRINMHELE-ASPVDWDKLLKGDT------GK-A-ALRE-GKQPRK----H
gi|567456807|gb|ETK11358.1|hypotheticalproteinT235_15845[Tannerellasp.oraltaxonBU063isolateCell8/11]/11433    ---Q-NPPVSRVNLYDLD-HSSVDWEKLFQGIE------GR-E-ALSA-KKQLRE----H
gi|545440279|ref|WP_021677877.1|helicase[Porphyromonasgingivalis]_gi|543985765|gb|ERJ89108.1|helicaseprotein[Porphyromonasgingi    ---Q-NPPVSRVNLYDLD-HSSVDWNKLFQGLE------GK-A-ALSA-KKQLRE----H
gi|497438056|ref|WP_009752254.1|helicase[Prevotellasp.oraltaxon473]_gi|429144866|gb|EKX87968.1|helicaseprotein[Prevotellasp.ora    ---Q-NPPVSRVNLYDLD-HSSVDWGKLFEGLE------GK-A-ALAG-KKQLRA----H
gi|490423474|ref|WP_004295683.1|MULTISPECIES:helicase[Bacteroides]_gi|156110098|gb|EDO11843.1|helicaseCterminaldomainprotein[B/    ---Q-NPPFNRVGLVDLQ-NSPVDWQLLIDGLK------GK-E-AMLP-GKQPRE----H
gi|941777229|ref|WP_055173506.1|helicase[Bacteroidescaccae]_gi|933263395|emb|CUQ00567.1|Predictedhelicase[Bacteroidescaccae]/1/    ---Q-EPPVTRIGMADLD-SSPVDWQKLMDGLT------GN-S-ALVE-GKKPRE----H
gi|492413898|ref|WP_005836892.1|helicase[Bacteroidesuniformis]_gi|392678824|gb|EIY72229.1|hypotheticalproteinHMPREF1072_03583[B    ---Q-EPPVTRIGMADLD-SSPVDWQKLMDGLT------GN-S-ALVE-GKKPRE----H
```

```
gi|695455095|ref|WP_032566191.1|helicase[Bacteroidesfragilis]_gi|596095426|gb|EXZ59996.1|restrictionendonucleasefamilyprotein[B      ---Q-EPPVTRIGMADLD-SSPVDWQKLMDGLT------GN-S-ALVE-GKKPRE----H
gi|933227941|emb|CUQ02348.1|Predictedhelicase[Bacteroidesuniformis]/11658                                                             ---Q-EPPVTRIGMADLE-SSPVDWQKLLDGLT------GN-S-ALVE-GKKPRE----H
gi|494823013|ref|WP_007558421.1|helicase[Bacteroidesplebeius]_gi|198272167|gb|EDY96436.1|helicaseCterminaldomainprotein[Bacter/      ---Q-EPPVTRIGMADLD-SSPVDWQKLMDGLT------GN-S-ALVE-GKKPRE----H
gi|495427212|ref|WP_008151909.1|helicase[Parabacteroidesjohnsonii]_gi|218222381|gb|EEC95031.1|helicaseCterminaldomainprotein[P/      ---Q-DPPFSRVGLIDLN-SSCVDWQKLMDGLT------GN-S-ALVE-GKKPRK----H
gi|933196855|emb|CUO94545.1|typeIrestrictionenzymeEcoKIsubunitR[Bacteroidesuniformis]_gi|933220098|emb|CUN86874.1|typeIrestrict      ---Q-EPPVTRVGMADLN-SSPVDWQKLLDGLT------GN-S-ALIE-GKKPRK----H
gi|823687748|ref|WP_047101121.1|helicase[Brachyspirahyodysenteriae]_gi|823334537|gb|KLI17932.1|helicase[Brachyspirahyodysenteri      ---Q-VPPVSRITKTDLE-NSGIDWEKIFKGIY------GQ-K-ALKT-KKSLFP----H
gi|496436691|ref|WP_009145538.1|helicase[Phascolarctobacteriumsuccinatutens]_gi|322414007|gb|EFY04839.1|helicaseCterminaldomai/      ---Q-NPPAYIINSWDVADDETVDWDELDAGLF------GS-T-AVKKRKISPKK----H
gi|503372880|ref|WP_013607541.1|helicase[Sphaerochaetaglobosa]_gi|324026933|gb|ADY13692.1|typeIIIrestrictionproteinressubunit[S      ---Q-TPPVSRLNLYELM-DSEVDWDKIEAGLS------GA-N-ATVT-KKTPRE----H
gi|505821748|ref|WP_015707145.1|helicase[Treponemaprimitia]_gi|299483503|gb|ADJ19584.1|helicasedomaincontainingprotein[Trepone/      ---Q-TPEVIRRGYLDLV-DAPVDWAKLDKGSS------GE-Q-AVKK-RYSPKP----H
gi|505822741|ref|WP_015708134.1|helicase[Treponemaprimitia]_gi|333739639|gb|AEF85129.1|endonucleaseandmethylaseLlaGI[Treponemap      ---Q-SPEVKRRGYYDLV-DAPVDWAKLDKGSS------GE-Q-AVKK-RYSPKP----H
gi|488791994|ref|WP_002704400.1|helicase[Treponemasaccharophilum]_gi|380767691|gb|EIC01689.1|typeIIIrestrictionproteinressubuni      ------LHVARLGLIDLK-NDSVDWEKLDAGLY------GD-K-AASV-KYDLRE----H
gi|524671454|emb|CDD81750.1|putativeseptumsitedeterminingproteinMinC[Bacteroidessp.CAG:462]/11642                                  ---Q-TPQVTRLNLIDLE-ADDVDWEKLEQGLY------GK-A-SRPK-PFAIME----H
gi|524573865|emb|CDC95766.1|putativeseptumsitedeterminingproteinMinC[Alistipessp.CAG:268]/11639                                   ---Q-NPPVTRLNLIDLE-NDDVDWNSLEQGIF------GM-A-SRSK-PFTIRE----H
gi|658617970|ref|WP_029759328.1|helicase[Fusobacteriumnucleatum]/11606                                                             ---Q-EISVIRISLNELE-NSDVDWSKIYLGKN------GK-E-AKKA-SKTIRN----H
gi|494412037|ref|WP_007216782.1|helicase[Bacteroidescellulosilyticus]_gi|392638921|gb|EIY32752.1|hypotheticalproteinHMPREF1062_      ---Q-QPPVSRIGLLQLE-QAPVDWQKMEVGLA------GG-E-AQTR-KKQPRD----H
gi|383107102|gb|AFG37435.1|putativehelicase[SpirochaetaafricanaDSM8902]/11616                                                      ---Q-NPPVSRINLFVLR-DAPVDWEKIEQGIS------GV-E-ARTE-AKTLRD----H
SAF8902III/11616                                                                                                                    ---Q-NPPVSRINLFVLR-DAPVDWEKIEQGIS------GV-E-ARTE-AKTLRD----H
gi|652538894|ref|WP_026932796.1|helicase[Gramellaechinicola]/11633                                                                 ---Q-NPPVTRINLNELQ-EAPVDWEKLENGIS------GD-L-ARKE-KHTLRT----H
gi|639238223|ref|WP_024568839.1|helicase[Elizabethkingiaanophelis]/11628                                                           ---Q-NPPVTRINLSDLQ-QAPVDWGKIEDGIS------GG-L-ARNE-KKFLRL----H
gi|652520381|ref|WP_026914582.1|helicase[Gramellaportivictoriae]/11623                                                             ---Q-NPPVTRINLSELE-QSPVDWELLQKGIT------GD-L-ARTE-KKVLRL----H
gi|494922183|ref|WP_007648219.1|Helicase[Formosasp.AK20]_gi|469825489|gb|EMQ95550.1|Helicasedomainprotein[Formosasp.AK20]/11626    ---Q-NPPVTRINLTDLQ-QAPVEWERLQKGIT------GD-L-ARTD-KKTLFK----H
gi|522083382|ref|WP_020594591.1|hypotheticalprotein[Spirosomapanaciterrae]/11637                                                  ---Q-HIPFLRLSLLELA-EAPLDWEKLDNGIS------GE-G-ARTT-KKQLRP----H
gi|490455352|ref|WP_004326157.1|helicase[Bacteroidesovatus]_gi|392669277|gb|EIY62768.1|hypotheticalproteinHMPREF1070_03635[Bact     ---Q-QPAVSRIGLFHLE-SAPVDWCKLEEGLS------GE-A-SRLV-KKELFP----H
gi|499516035|ref|WP_011202675.1|helicase[Bacteroidesfragilis]_gi|53713270|ref|YP_099262.1|helicasedomainprotein[Bacteroidesfrag      ---Q-QPAVSRIGLFHLE-SAPVDWCKLEEGLS------GE-A-SRLV-KKELFP----H
gi|651338332|ref|WP_026461320.1|helicase[Adhaeribacteraquaticus]/11620                                                             ---Q-HPPVTRLNLYELR-EAPVDWEKLEQGIH------GE-S-ARTP-KKNLRG----H
gi|806964642|gb|AKC17139.1|helicase[Flavobacteriumpsychrophilum]/11639                                                             ---Q-KPPVQRISLTDLQ-NASVDWEKLENGIH------GE-Q-ARTN-LKSIRK----H
gi|752684374|ref|WP_041332322.1|helicase[Flavobacteriumpsychrophilum]_gi|666882889|gb|AIG30715.1|helicase[Flavobacteriumpsychro      ---Q-KPPVQRISLTDLQ-NASVDWEKLENGIH------GE-Q-ARTN-LKSIRK----H
gi|933196595|emb|CUP04894.1|DNAorRNAhelicasesofsuperfamilyII[Bacteroidesuniformis]/11631                                          ---Q-NPPVTRLNLYDLQ-HAPVDWEKLEMGIH------GE-A-SRNP-KKVLKP----H
gi|670509109|ref|WP_031456918.1|helicase[Flavobacteriumchungangense]/11625                                                         ---Q-HPPVSRIHLHDLQ-DAPVDWAQMESGVH------GE-I-ARIT-GRDLRD----H
                                                                                                                                                     .      .

gi|490190857|ref|WP_004089451.1|hypotheticalprotein[Helicobacterbilis]_gi|476630638|gb|EMZ37196.1|hypotheticalproteinC826_02193      QQEALNAVKEYFNNP-------SNTRGKLIMACGTGKTFTSLKITETLTDS---------
gi|488896133|ref|WP_002807242.1|MULTISPECIES:restrictionendonucleaseEco57I[Campylobacter]_gi|380541096|gb|EIA65377.1|hypothetic     QIQAINNTKEYFANK-------NNTRGKLIMACGTGKTFTSLKIIEEI-TP---------
gi|746602240|ref|WP_039628365.1|restrictionendonucleaseEco57I[Campylobacterlari]_gi|744819395|gb|AJD03391.1|putativehelicase[Ca     QIQAINNTKEYFANK-------NNTRGKLIMACGTGKTFTSLKIIEEI-TP---------
gi|727317135|ref|WP_033772558.1|restrictionendonucleaseEco57I[Helicobacterpylori]/11622                                           QTEAIEKTKEYFSDP-------KNVRGKLIMACGTGKTYTSLKIMESL-EP---------
gi|727103490|ref|WP_033618954.1|restrictionendonucleaseEco57I[Helicobacterpylori]/11616                                           QTEAIEKTKEYFSDP-------KNARGKLIMACGTGKTYTSLKIMEAL-DP---------
gi|727340205|ref|WP_033794408.1|restrictionendonucleaseEco57I[Helicobacterpylori]/11609                                           QQEAINATKEYFSNP-------KNARGKLIMACGTGKTYTSLKIMESL-EP---------
gi|727292407|ref|WP_033749975.1|restrictionendonucleaseEco57I[Helicobacterpylori]/11611                                           QQEAINATKEYFSNP-------KNARGKLIMACGTGKTYTSLKIMESL-EP---------
gi|727289795|ref|WP_033747591.1|restrictionendonucleaseEco57I[Helicobacterpylori]/11612                                           QTEAIEKTKEYFSDP-------KNARGKLIMACGTGKTYTSLKIMEAL-DP---------
gi|727319676|ref|WP_033775047.1|restrictionendonucleaseEco57I[Helicobacterpylori]/11612                                           QTEAIEKTKEYFSDP-------KNARGKLIMACGTGKTYTSLKIMEAL-DP---------
gi|447212910|ref|WP_001290166.1|restrictionendonucleaseEco57I[Helicobacterpylori]_gi|308063540|gb|ADO05427.1|hypotheticalprotei    QIEAIKATKEYFSDP-------KNTRGKLIMACGTGKTYTSLKIMEAL-DP---------
gi|447212901|ref|WP_001290157.1|restrictionendonucleaseEco57I[Helicobacterpylori]_gi|384557329|gb|AFH97797.1|hypotheticalprotei    QIEAIKATKEYFSDP-------KNTRGKLIMACGTGKTYTSLKIMEAL-EP---------
gi|515849671|ref|WP_017280424.1|hypotheticalprotein[Helicobacterpylori]/11615                                                      QIEAIKATKEYFSNP-------KNTRGKLIMACGTGKTYTSLKIMEAL-DP---------
gi|447212900|ref|WP_001290156.1|restrictionendonucleaseEco57I[Helicobacterpylori]_gi|317178970|dbj|BAJ56758.1|TypeIIGrestrictio   QIEAIKATKEYFSNP-------KNTRGKLIMACGTGKTYTSLKIMEAL-EP---------
gi|447079018|ref|WP_001156274.1|hypotheticalprotein[Helicobacterpylori]/11614                                                      QTEAIKATKEYFSSP-------KNTRGKLIMACGTGKTYTSLKIMEAL-DP---------
gi|447212800|ref|WP_001290056.1|hypotheticalprotein[Helicobacterpylori]_gi|532153248|gb|EQL67316.1|restrictionendonucleaseEco57    QTEAIKATKEYFSDP-------KNTRGKLIMACGTGKTYTSLKIMEAL-DP---------
gi|505457237|ref|WP_015644339.1|hypotheticalprotein[Helicobacterpylori]_gi|499061160|gb|AGL70074.1|restrictionendonucleaseEco57    QVEAINATKKYFSSP-------KNTRGKLIMACGTGKTYTSLKIMETL-DP---------
HPYUM037X/11591                                                                                                                     QVEAINATKKYFSSP-------KNTRGKLIMACGTGKTYTSLKIMETL-DP---------
gi|920026297|ref|WP_052937176.1|restrictionendonuclease[Helicobacterpylori]_gi|912528678|gb|KNX48238.1|restrictionendonucleaseE    QVEAINATKEYFSDL-------KNTRGKLIMACGTGKTYTSLKIMEAL-DP---------
gi|915539050|ref|WP_050841327.1|restrictionendonuclease[Helicobacterpylori]_gi|908890887|gb|KNE06183.1|restrictionendonucleaseE    QTEAIKATKEYFSDP-------KNARGKLIMACGTGKTYTSLKIMEAL-DP---------
gi|727101397|ref|WP_033616957.1|restrictionendonucleaseEco57I[Helicobacterpylori]/11592                                           QTEAINATKEFFLNP-------KNTRGKLIMACGTGKTYTSLKIMEAL-DP---------
gi|562814875|emb|CDK13002.1|TypeISPRMsystem[Helicobacterpylori26695]/11588                                                        QTEAINATKEYFSDP-------KNARGKLIMACGTGKTYTSLKIMEAL-DS---------
HPYAXVIII/11588                                                                                                                     QTEAINATKEYFSDP-------KNARGKLIMACGTGKTYTSLKIMEAL-DS---------
gi|447079029|ref|WP_001156285.1|restrictionendonucleaseEco57I[Helicobacterpylori]_gi|393085956|gb|EJB86635.1|hypotheticalprotei    QTEAINATKEYFSSP-------KNTRGKLIMACGTGKTYTSLKIMEAL-DP---------
gi|489105953|ref|WP_003015814.1|restrictionendonuclease[Helicobacterpylori]_gi|477503388|gb|ENH59950.1|restrictionendonucleasef    QTEAIKATKEYFSSP-------KNARGKLIMACGTGKTYTSLKIMETL-EP---------
gi|447079006|ref|WP_001156262.1|restrictionendonucleaseEco57I[Helicobacterpylori]_gi|393067470|gb|EJB68280.1|hypotheticalprotei    QTEAINATKEYFSSP-------KNARGKLIMACGTGKTYTSLKIMEAL-GP---------
gi|727314589|ref|WP_033770298.1|restrictionendonucleaseEco57I[Helicobacterpylori]/11586                                           QQEAINATKEYFSDP-------KNARGKLIMACGTGKTYTSLKIMEAL-DP---------
gi|447079019|ref|WP_001156275.1|restrictionendonucleaseEco57I[Helicobacterpylori]_gi|308065028|gb|ADO06920.1|hypotheticalprotei    QTEAIEKTKEYFSNP-------KNARGKLIMACGTGKTYTSLKIMESL-DP---------
gi|447079028|ref|WP_001156284.1|restrictionendonucleaseEco57I[Helicobacterpylori]_gi|393076030|gb|EJB76784.1|hypotheticalprotei    QIEAIEKTKEYFSDP-------KNARGKLIMACGTGKTYTSLKIMETL-DP---------
gi|393034009|gb|EJB35070.1|hypotheticalproteinHPNQ4053_0631[HelicobacterpyloriNQ4053]/11514                                       QTEAINATKEYFSDP-------KNARGKLIMACGTGKTYTSLKIMEAL-DP---------
gi|447079004|ref|WP_001156260.1|restrictionendonucleaseEco57I[Helicobacterpylori]_gi|384552418|gb|AFI07366.1|hypotheticalprotei    QTEAIEETKKYFSDP-------KNARGKLIMACGTGKTYTSLKIMEAL-DP---------
gi|746011306|ref|WP_039088215.1|restrictionendonucleaseEco57I[Helicobacterpylori]_gi|733488720|gb|KHL85979.1|restrictionendonuc    QTEAIEKTKEYFSDP-------KNARGKLIMACGTGKTYTSLKIMEAL-DP---------
gi|531081162|gb|EQD92166.1|restrictionendonucleaseEco57I[HelicobacterpyloriPZ5080]/11509                                          QIEAIEKTKEYFSNP-------KNARGKLIMACGTGKTYTSLKIMEAL-DP---------
gi|544760796|ref|WP_021187395.1|restrictionendonucleaseEco57I[Helicobacterpylori]_gi|531086508|gb|EQD96745.1|restrictionendonuc    QTEAINATKEYFSDP-------KNTRGKLIMACGTGKTYTSLKIMEAL-DP---------
gi|447079012|ref|WP_001156268.1|restrictionendonucleaseEco57I[Helicobacterpylori]_gi|393028199|gb|EJB29286.1|hypotheticalprotei    QTEAIEKTKEYFSDP-------KNARGKLIMACGTGKTYTSLKIMETL-DP---------
gi|746008458|ref|WP_039085555.1|restrictionendonucleaseEco57I[Helicobacterpylori]_gi|733480358|gb|KHL78026.1|restrictionendonuc    QTEAIEKTKEYFSSP-------KNTRGKLIMACGTGKTYTSLKIMEAL-DP---------
gi|447079011|ref|WP_001156267.1|restrictionendonucleaseEco57I[Helicobacterpylori]_gi|393038534|gb|EJB39568.1|hypotheticalprotei    QTEAIKATKEYFSDP-------KNARGKLIMACGTGKTYTSLKIMETL-EP---------
```

```
gi|393037163|gb|EJB38199.1|hypotheticalproteinHPNQ4161_0422[HelicobacterpyloriNQ4161]/11556          QTEAIEKTKEYFSDP-------KNARGKLIMACGTGKTYTSLKIMEAL-DP---------
gi|447079007|ref|WP_001156263.1|restrictionendonucleaseEco57I[Helicobacterpylori]_gi|393051817|gb|EJB52768.1|hypotheticalprotei          QTEAIEKTKEYFSNP-------KNARGKLIMACGTGKTYTSLKIMEAL-DP---------
gi|487867094|ref|WP_001940560.1|helicase[Helicobacterpylori]_gi|459505657|gb|EMG98418.1|helicaseprotein[HelicobacterpyloriGAM21          QTEAIEETKKYFSNP-------KNARGKLIMACGTGKTYTSLKIMEAL-DP---------
gi|487872094|ref|WP_001945560.1|helicase[Helicobacterpylori]_gi|459512104|gb|EMH04523.1|helicaseprotein[HelicobacterpyloriGAM24          QKEAIEKTKEYFSDP-------KNARGKLIMACGTGKTYTSLKIMEAL-DP---------
gi|487880228|ref|WP_001953694.1|helicase[Helicobacterpylori]_gi|459529131|gb|EMH20734.1|helicaseprotein[HelicobacterpyloriGAM26          QTEAIEKTKEYFSDP-------KNARGKLIMACGTGKTYTSLKIMEAL-DP---------
gi|487882593|ref|WP_001956059.1|helicase[Helicobacterpylori]_gi|459533462|gb|EMH24859.1|helicaseprotein[HelicobacterpyloriGAM26          QKEAIEETKKYFSDP-------KNARGKLIMACGTGKTYTSLKIMEAL-DP---------
gi|487845430|ref|WP_001918896.1|helicase[Helicobacterpylori]_gi|410713871|gb|EKQ71363.1|helicaseprotein[HelicobacterpyloriGAM10          QTEAIEKTKEYFSDP-------KNARGKLIMACGTGKTYTSLKIMEAL-DP---------
gi|447079005|ref|WP_001156261.1|restrictionendonucleaseEco57I[Helicobacterpylori]_gi|393069830|gb|EJB70625.1|hypotheticalprotei          QKEAIEKTKEYFSNP-------KNARGKLIMACGTGKTYTSLKIMEAL-DP---------
gi|459506808|gb|EMG99528.1|helicaseprotein[HelicobacterpyloriGAM121Aii]/11545          QTEAINATKEYFSDP-------KNARGKLIMACGTGKTYTSLKIMEAL-DP---------
gi|487825283|ref|WP_001898749.1|restrictionendonucleaseEco57I[Helicobacterpylori]_gi|393107526|gb|EJC08068.1|hypotheticalprotei          QTEAIEKTKEYFSDP-------KNARGKLIMACGTGKTYTSLKIMEAL-DP---------
gi|447079008|ref|WP_001156264.1|restrictionendonucleaseEco57I[Helicobacterpylori]_gi|393058636|gb|EJB59524.1|hypotheticalprotei          QTEAIEKTKEYFSNP-------KNARGKLIMACGTGKTYTSLKIMEAL-DP---------
gi|447079015|ref|WP_001156271.1|restrictionendonucleaseEco57I[Helicobacterpylori]_gi|307637357|gb|ADN79807.1|hypotheticalprotei          QTEAIEETKKYFSNP-------KNARGKLIMACGTGKTYTSLKIMEAL-DP---------
gi|487884861|ref|WP_001958327.1|helicase[Helicobacterpylori]_gi|459535535|gb|EMH26834.1|helicaseprotein[HelicobacterpyloriGAM26          QTEAIEKTKKYFSDP-------KNARGKLIMACGTGKTYTSLKIMEAL-DP---------
gi|487886365|ref|WP_001959831.1|helicase[Helicobacterpylori]_gi|459538727|gb|EMH29861.1|helicaseprotein[HelicobacterpyloriGAM27          QTEAIEKTKEYFSDP-------KNARGKLIMACGTGKTYTSLKIMEAL-DP---------
gi|545301637|ref|WP_021581412.1|helicase[Helicobacterpylori]_gi|535931889|gb|ERA61745.1|helicaseprotein[HelicobacterpyloriGAM11          QTEAIEKTKEYFSDP-------KNARGKLIMACGTGKTYTSLKIMEAL-DP---------
gi|447079010|ref|WP_001156266.1|restrictionendonucleaseEco57I[Helicobacterpylori]_gi|393095477|gb|EJB96081.1|hypotheticalprotei          QTEAIEKTKKYFSDP-------KNARGKLIMACGTGKTYTSLKIMEAL-DP---------
gi|447079009|ref|WP_001156265.1|restrictionendonucleaseEco57I[Helicobacterpylori]_gi|393056294|gb|EJB57206.1|hypotheticalprotei          QTEAIEKTKEYFSDP-------KNARGKLIMACGTGKTYTSLKIMEAL-DP---------
gi|471386985|gb|EMR58906.1|restrictionendonucleasefamilyprotein[HelicobacterpyloriHpH1]/11504          QKEAIEKTKEYFSNP-------KNARGKLIMACGTGKTYTSLKIMEAL-DP---------
gi|487875679|ref|WP_001949145.1|helicase[Helicobacterpylori]_gi|459516928|gb|EMH09091.1|helicaseprotein[HelicobacterpyloriGAM25          QTEAIEETKKYFSDP-------KNARGKLIMACGTGKTYTSLKIMESL-DP---------
gi|496130066|ref|WP_008854573.1|DNAdamageinducibleprotein[Commensalibacterintestini]_gi|353671689|gb|EHD13391.1|helicasedomain/          QQTALDAVQHGLKT---------ADRGKLIMACGTGKTFTSLKIAEAMAGK---------
gi|924898894|ref|WP_053556546.1|damageinducibleprotein[Sphingopyxissp.113P3]_gi|924523996|gb|ALC14732.1|damageinducibleprotei/1          QIEAIDCVTDKLAT---------ADRGKLIMACGTGKTFTGLKIAEAMAGR---------
gi|780802950|gb|KJS08562.1|damageinducibleprotein[Hoefleasp.BRH_c9]/11633          QREAVEAVREGFTK---------ADRGKLIMACGTGKTFTSLKIAEDLAGV---------
gi|944671432|gb|KQN22886.1|damageinducibleprotein[Sphingomonassp.Leaf32]/11415          QRDALAAARHGLAS---------ADRGKLIMACGTGKTFTGLKIAEDLAGP---------
gi|944612953|gb|KQM64772.1|damageinducibleprotein[Sphingomonassp.Leaf16]/11726          QRDALAAARHGLAS---------ADRGKLIMACGTGKTFTGLKIAEDLAGP---------
gi|764353851|ref|WP_044331468.1|damageinducibleprotein[Sphingomonashengshuiensis]_gi|761897338|gb|AJP71707.1|damageinduciblep/1          QRDALEAVRDGLAK---------ADRGKLIMACGTGKTFTGLKIAEDLAGA---------
gi|945874472|gb|KQZ27973.1|damageinducibleprotein[Mesorhizobiumsp.Root552]/11651          QQDALAAVRAGLAE---------DDRGKLIMACGTGKTFTGLKIAEDMAGP---------
gi|759408081|ref|WP_043132403.1|damageinducibleprotein[Paracoccussanguinis]_gi|694217086|gb|KGJ14281.1|damageinducibleprotein/1          QRDALKAVTEGLAQ---------ADRGKLIMACGTGKTFTGLRIAEALAGP---------
gi|654342671|ref|WP_027836116.1|damageinducibleprotein[Maritaleamyrionectae]/11632          QREALELVRSGLQE---------ADRGKLIMACGTGKTFTALRIAEELAGK---------
gi|126717687|gb|EBA14409.1|hypotheticalproteinRSK20926_02032[Roseobactersp.SK20926]/11603          QRDALNAVASGFET---------ADRGKLIMACGTGKTFTGLRIAETLAGQ---------
gi|765301116|ref|WP_044628780.1|damageinducibleprotein[Halomonasmeridiana]_gi|764089679|gb|KJD19875.1|damageinducibleprotein[/1          QREALKAVSEGLEK---------ADRGKLIMACGTGKTFTGLRIAEAIGGP---------
gi|518053006|ref|WP_019223214.1|DNAdamageinducibleprotein[Bartonellarattaustraliani]/11654          QAEALKAVCEGLKA---------ADRGKLIMACGTGKTFTSLKIAEQIAGK---------
gi|493637806|ref|WP_006589578.1|DNAdamageinducibleprotein[Bartonellabirtlesii]_gi|395410345|gb|EJF76900.1|hypotheticalproteinM/          QEEAIKKVCEGLKE---------ANRGKLIMACGTGKTFTSLKIAETIAGK---------
gi|501188603|ref|WP_012231622.1|DNAdamageinducibleprotein[Bartonellatribocorum]_gi|161017843|emb|CAK01401.1|helicase/methyltra/          QEEAIKKVCEGLKE---------ADRGKLIMACGTGKTFTSLKIAETLAGK---------
gi|651388616|ref|WP_026500615.1|damageinducibleprotein[Bartonellagrahamii]/11655          QEEAIKKVCEGLKE---------ADRGKLIMACGTGKTFTSLKIAEHIAGK---------
gi|501188669|ref|WP_012231687.1|DNAdamageinducibleprotein[Bartonellatribocorum]_gi|161017922|emb|CAK01480.1|helicase/methyltra/          QEEAIKKVCEGLKE---------ADRGKLIMACGTGKTFTSLKIAETLAGK---------
gi|518048832|ref|WP_019219040.1|DNAdamageinducibleprotein[Bartonellaflorencae]/11655          QEEAIKKVCEGLKE---------ADRGKLIMACGTGKTFTSLKIAETIAGK---------
gi|518053617|ref|WP_019223825.1|DNAdamageinducibleprotein[Bartonellarattaustraliani]/11607          QKEALKAVCEGLKE---------ADRGKLIMACGTGKTFTSLKIAEQIAGK---------
gi|506336449|ref|WP_015856168.1|DNAdamageinducibleprotein[Bartonellagrahamii]_gi|240267492|gb|ACS51080.1|helicase/methyltransf/          QEEAIKKVCEGLKE---------ANRGKLIMACGTGKTFTSLKIAETIAGK---------
gi|494588832|ref|WP_007347427.1|DNAdamageinducibleprotein[Bartonellarattimassiliensis]_gi|395419053|gb|EJF85361.1|hypothetical/          QIEALEKVCEGLQE---------ADRGKLIMACGTGKTFTSLKIAERLAGK---------
gi|501188650|ref|WP_012231668.1|DNAdamageinducibleprotein[Bartonellatribocorum]_gi|161017902|emb|CAK01460.1|helicase/methyltra/          QKEALEKVCEGLQE---------ADRGKLIMACGTGKTFTSLKIAETIAGQ---------
gi|501188624|ref|WP_012231643.1|DNAdamageinducibleprotein[Bartonellatribocorum]_gi|161017866|emb|CAK01424.1|helicase/methyltra/          QKEALKAVCEGLQE---------ADRGKLIMACGTGKTFTSLKIAEHIAGT---------
gi|518053856|ref|WP_019224064.1|DNAdamageinducibleprotein[Bartonellarattaustraliani]/11625          QKAALKAVCEGLKE---------ADRGKLIMACGTGKTFTSLKIAEHIAGT---------
gi|501188053|ref|WP_012231081.1|DNAdamageinducibleprotein[Bartonellatribocorum]_gi|161017434|emb|CAK00992.1|helicase/methyltra/          QKEALKAVCEGLQE---------ADRGKLIMACGTGKTFTSLKIAEHIAGT---------
gi|651388630|ref|WP_026500626.1|damageinducibleprotein[Bartonellagrahamii]/11657          QEEAIKKVCEGLKE---------ADRGKLIMACGTGKTFTSLKIAETIAGK---------
gi|518048855|ref|WP_019219063.1|DNAdamageinducibleprotein[Bartonellaflorencae]/11658          QKEALEKVCEGLKE---------ADRGKLIMACGTGKTFTSLKIAEHIAGK---------
gi|490995101|ref|WP_004856829.1|DNAdamageinducibleprotein[Bartonelladoshiae]_gi|395413167|gb|EJF79645.1|hypotheticalproteinMCS/          QKEALKDVCEGLKE---------ADRGKLIMACGTGKTFTSLKIAETIAGK---------
gi|491002558|ref|WP_004864279.1|DNAdamageinducibleprotein[Bartonellavinsonii]_gi|395421392|gb|EJF87643.1|hypotheticalproteinME/          QKEALEAVCEGFKE---------ADRGKLIMACGTGKTFTSLKIAETLAGQ---------
gi|491003373|ref|WP_004865094.1|DNAdamageinducibleprotein[Bartonellavinsonii]_gi|395432864|gb|EJF98838.1|hypotheticalproteinME/          QKEALKAVCEGLQE---------ADRGKLIMACGTGKTFTSLKIAEQIAGT---------
gi|491002426|ref|WP_004864148.1|DNAdamageinducibleprotein[Bartonellavinsonii]_gi|395421767|gb|EJF88003.1|hypotheticalproteinME/          QKEALEAVCKGLRE---------ADRGKLIMACGTGKTFTSLKIAETLAGQ---------
gi|491004024|ref|WP_004865745.1|DNAdamageinducibleprotein[Bartonellavinsonii]_gi|395432542|gb|EJF98522.1|hypotheticalproteinME/          QKEALEAVCEGLKE---------ADRGKLIMACGTGKTFTSLKIAETLAGQ---------
gi|648396442|ref|WP_026088193.1|damageinducibleprotein[Bartonellarattaustraliani]/11593          QAEALNAVCEGLKA---------ADRGKLIMACGTGKTFTSFKIAEQIAGR---------
gi|748190174|ref|WP_039761290.1|damageinducibleprotein[Bartonellaqueenslandensis]/11658          QKEALKAVCEGLEK---------ADRGKLIMACGTGKTFTSLKIAEQIAGK---------
gi|494817599|ref|WP_007553007.1|DNAdamageinducibleprotein[Bartonellasp.DB56]_gi|395411312|gb|EJF77837.1|hypotheticalproteinMC/1          QQEALKAVCEGLQE---------ADRGKLIMACGTGKTFTSLKIAETLAGQ---------
gi|515763376|ref|WP_017195976.1|DNAdamageinducibleprotein[Bartonellabirtlesii]/11656          QQEALKAVCESLQE---------ADRGKLIMACGTGKTFTSLKIAETLAGQ---------
gi|494589420|ref|WP_007348014.1|DNAdamageinducibleprotein[Bartonellarattimassiliensis]_gi|395416278|gb|EJF82667.1|hypothetical/          QKEALEKVCEGLKE---------ADRGKLIMACGTGKTFTSLKIAERLAGK---------
gi|506336469|ref|WP_015856188.1|DNAdamageinducibleprotein[Bartonellagrahamii]_gi|240267512|gb|ACS51100.1|helicase/methyltransf/          QKEALEKVCEGLKE---------ADRGKLIMACGTGKTFTSLKIAEHIAGK---------
gi|749308087|ref|WP_040132227.1|damageinducibleprotein[Bartonellatribocorum]_gi|605051878|emb|CDO49988.1|helicase/methyltransf/          QKEALEKVCEGLKE---------ADRGKLIMACGTGKTFTSLKIAEQIAGQ---------
gi|515763800|ref|WP_017196400.1|DNAdamageinducibleprotein[Bartonellabirtlesii]/11658          QIEALKAVCEGLQE---------ADRGKLIMACGTGKTFTSLKIAETIAGK---------
gi|492186994|ref|WP_005773380.1|DNAdamageinducibleprotein[Bartonellaelizabethae]_gi|395418361|gb|EJF84688.1|hypotheticalprotei/          QKEALKEVCEGLQE---------ADRGKLIMACGTGKTFTSLKIAETIAGQ---------
gi|501188615|ref|WP_012231634.1|DNAdamageinducibleprotein[Bartonellatribocorum]_gi|161017856|emb|CAK01414.1|helicase/methyltra/          QIEALERVCEGLQE---------ADRGKLIMACGTGKTFTSLKIAEAIAGK---------
gi|501187470|ref|WP_012230499.1|DNAdamageinducibleprotein[Bartonellatribocorum]_gi|161017090|emb|CAK00648.1|helicase/methyltra/          QIEALERVCEGLQE---------ADRGKLIMACGTGKTFTSLKIAETIAGQ---------
gi|501188636|ref|WP_012231655.1|DNAdamageinducibleprotein[Bartonellatribocorum]_gi|161017879|emb|CAK01437.1|helicase/methyltra/          QIEALERVCEGLQE---------ADRGKLIMACGTGKTFTSLKIAETIAGK---------
gi|930151941|emb|CTQ34818.1|UvsWhelicase[Jannaschiarubra]/11641          QKEALEAVRAGLEE---------ADRGKLILACGTGKTFTSLRIAEDLVGA---------
gi|928977106|ref|WP_054001461.1|damageinducibleprotein[Shimiasp.SK013]_gi|927698852|gb|KPA22698.1|typeIrestrictionenzymeEcoKIs/          QKLALEHVGKGFEE---------GDRGKLIMACGTGKTFTALRIAEAVAGK---------
gi|738872465|ref|WP_036760884.1|damageinducibleprotein[Leisingeradaeponensis]/11643          QSDALDAVRKGLAD---------HDRGKIIMACGTGKTFTSLKIAEDLVGA---------
gi|518050881|ref|WP_019221089.1|DNAdamageinducibleprotein[Bartonellasenegalensis]/11652          QVEALRFVRAGLME---------ADRGKLIMACGTGKTFTSLKIAEDLAGR---------
gi|493980596|ref|WP_006923523.1|DNAdamageinducibleprotein[Bartonellawashoensis]_gi|395413322|gb|EJF79796.1|hypotheticalprotein/          QLEALHFVRAGLTE---------ADRGKLIMACGTGKTFTSLKIAEDLAGK---------
```

gi|493983106|ref|WP_006925984.1|DNAdamageinducibleprotein[Bartonellawashoensis]_gi|395418127|gb|EJF84455.1|hypotheticalprotein/	QLEALHFVRAGLTE---------ADRGKLIMACGTGKTFTSLKIAEDLAGE---------
gi|499494671|ref|WP_011181311.1|DNAdamageinducibleprotein[Bartonellahenselae]_gi|49239008|emb|CAF28308.1|hypotheticalproteinBH/	QVEALHFVRAGLTK---------ADRGKLIMACGTGKTFTSLKIAEDLAGE---------
gi|651388426|ref|WP_026500441.1|damageinducibleprotein[Bartonellagrahamii]/11652	QQDALRDVRAGFAQ---------ADRGKLIMACGTGKTFTSLKIAEDLAGE---------
gi|506337280|ref|WP_015856999.1|DNAdamageinducibleprotein[Bartonellagrahamii]_gi|240268402|gb|ACS51990.1|helicase/methyltransf/	QQDALRDVRAGFAQ---------ADRGKLIMACGTGKTFTSLKIAEDLAGE---------
gi|494817535|ref|WP_007552943.1|DNAdamageinducibleprotein[Bartonellasp.DB56]_gi|395412002|gb|EJF78518.1|hypotheticalproteinMC/1	QLEALRDVRAGLAE---------ADRGKLIMACGTGKTFTSLKIAEDLAGE---------
gi|493638099|ref|WP_006589864.1|DNAdamageinducibleprotein[Bartonellabirtlesii]_gi|395409809|gb|EJF76394.1|hypotheticalproteinM/	QVEALRDVRAGLME---------ADRGKLIMACGTGKTFTSLKIAEDLAGE---------
gi|748189589|ref|WP_039760707.1|damageinducibleprotein[Bartonellaqueenslandensis]/11651	QAEALRFVRAGLEQ---------ADRGKLIMACGTGKTFTSLKIAENLAGE---------
gi|501189483|ref|WP_012232501.1|DNAdamageinducibleprotein[Bartonellatribocorum]_gi|161018902|emb|CAK02460.1|helicase/methyltra/	QQEALRFVRAGLAQ---------ADRGKLIMACGTGKTFTSLKIAEDLVGE---------
gi|518049181|ref|WP_019219389.1|DNAdamageinducibleprotein[Bartonellaflorencae]/11652	QGEALRFVRAGLAE---------ADRGKLIMACGTGKTFTSLKIAEDLAGK---------
gi|492193255|ref|WP_005774528.1|DNAdamageinducibleprotein[Bartonellaelizabethae]_gi|395416403|gb|EJF82779.1|hypotheticalprotei/	QGEALRFVRAGLAQ---------ADRGKLIMACGTGKTFTSLKIAEDLAGQ---------
gi|498387325|ref|WP_010701288.1|helicase/methyltransferase[Bartonellabovis]_gi|478418724|gb|ENN92426.1|helicase/methyltransfera	QEEALHAVRAGLTE---------ADRGKLIMACGTGKTFTSLKIAEDLAGK---------
gi|319408697|emb|CBI82352.1|conservedhypotheticalprotein[BartonellaschoenbuchensisR1]/11661	QKEALHAVRSGLAE---------ADRGKLIMACGTGKTFTSLKIAEDLAGK---------
gi|494741726|ref|WP_007477134.1|DNAdamageinducibleprotein[Bartonellamelophagi]_gi|395423925|gb|EJF90113.1|hypotheticalproteinM/	QKEALHAVRAGLAE---------ADRGKLIMACGTGKTFTSLKIAEDLAGK---------
gi|737261405|ref|WP_035245349.1|damageinducibleprotein[Alcanivoraxjadensis]_gi|686989709|gb|KGD62556.1|hypotheticalproteinT9A_/	QEEALADVRAGLGK---------ADRGKLIMACGTGKTFTSLKIAEDLAGP---------
gi|916687564|ref|WP_051294655.1|damageinducibleprotein[Gemmobacternectariphilus]/11641	QRDALEAVRAGLEE---------GDRGKLIMACGTGKTFTALKIAEDLAGP---------
gi|740294127|ref|WP_038132495.1|damageinducibleprotein[Thioclavasp.DT234]_gi|660524826|gb|KEO54689.1|hypotheticalproteinDT23_/1	QIEALEAVRTGLAT---------DDRGKLIMACGTGKTFTSLKIAEDLVGK---------
gi|568331062|ref|WP_024099615.1|helicase[Phaeobactergallaeciensis]_gi|568232121|gb|AHD12007.1|putativehelicase[Phaeobactergalla	QKEALRDVRAGLTE---------ADRGKMIMACGTGKTFTSLKIAEDLAGQ---------
gi|518810459|ref|WP_019966413.1|DNAdamageinducibleprotein[Pannonibacterphragmitetus]/11635	QKDALADVSKGLAA---------ADRGKMIMACGTGKTFTSLKIAEAIAGK---------
gi|495305760|ref|WP_008030510.1|DNAdamageinducibleprotein[Rhodobactersp.SW2]_gi|259021698|gb|EEW25000.1|typeIIIrestrictionprot/	QKDALADVSKGLAA---------ADRGKMIMACGTGKTFTSLKIAEAIAGK---------
gi|506403303|ref|WP_015923022.1|DNAdamageinducibleprotein[Rhodobactersphaeroides]_gi|221162951|gb|ACM03922.1|HypotheticalProte/	QRDALAHVRDGLAK---------ADRGKMIMACGTGKTFTALKIAEDLAGK---------
gi|738818386|ref|WP_036708790.1|damageinducibleprotein[Paracoccussanguinis]_gi|694226786|gb|KGJ22541.1|damageinducibleprotein/1	QRDALAHVRDGLAK---------ADRGKMIMACGTGKTFTALKIAEDLAGK---------
gi|746096057|ref|WP_039162453.1|damageinducibleprotein[Gallibacteriumanatis]_gi|703617775|gb|KGQ63868.1|damageinducibleprotei/1	QISALNAVKDGL---Q-----T-ADRGKLIMACGTGKTFTSLKIAEEIAGK---------
gi|737716785|ref|WP_035685631.1|damageinducibleprotein[Avibacteriumparagallinarum]/11643	QISALNAVKDGL---Q-----T-ADRGKLIMACGTGKTFTSLKIAEEIAGK---------
gi|540072553|gb|ERF78672.1|hypotheticalproteinN561_04930[Gallibacteriumanatis12656/12}/11651	QISALNAVKAGL---Q-----T-ANRGKLIMACGTGKTFTSLKIAEEIAGK---------
gi|917509519|ref|WP_052115936.1|damageinducibleprotein[Gallibacteriumanatis]_gi|702414161|gb|KGQ42233.1|damageinducibleprotei/1	QISALNAVKAGL---Q-----T-ANRGKLIMACGTGKTFTSLKIAEEIAGK---------
gi|746082942|ref|WP_039150268.1|damageinducibleprotein[Gallibacteriumanatis]_gi|703617078|gb|KGQ63194.1|damageinducibleprotei/1	QISALNAVKAGL---Q-----T-ANRGKLIMACGTGKTFTSLKIAEEIAGK---------
gi|822522885|ref|WP_046952840.1|damageinducibleprotein[Haemophilushaemolyticus]_gi|821501762|gb|KKZ59267.1|damageinduciblepro/1	QKSALSAVRNGL---K-----N-ADRGKLIMACGTGKTFTSLKIAENIAGN---------
gi|404381014|gb|EJZ77501.1|putativehelicase[Pasteurellamultocidasubsp.gallicidaP1059]/11681	QKTALHKVKAGL---S-----T-ADRGKLIMACGTGKTFTSLKIAEEMAGK---------
gi|298281660|gb|EFI23149.1|helicaseCterminaldomainprotein[Neisseriasp.oraltaxon014str.F0314]/11493	QQSALTAVSNGF---A-----RGETRGKLIMACGTGKTFTSLRIAEHLAGK---------
gi|348013055|gb|EGY51980.1|helicasedomainprotein[Neisseriashayeganii871]/11481	QQRALKSVQTRF---N-----GGEERGKLIMACGTGKTFTALKIAETMAGT---------
gi|496407140|ref|WP_009116004.1|DNAdamageinducibleprotein[Neisseriawadsworthii]_gi|349794756|gb|EGZ48566.1|helicasedomainprote/	QQQALKSVQTRF---D-----GGETRGKLIMACGTGKTFTGLKIAENIAGV---------
gi|517216441|ref|WP_018405259.1|DNAdamageinducibleprotein[Marinobacterlipolyticus]/11656	QVDALNGAVSGL---K-----T-ADRGKLIMACGTGKTFTSLKIAEDIAGK---------
gi|738230264|ref|WP_036185691.1|damageinducibleprotein[Marinobactersp.ELB17]/11671	QVDALNGALSGL---K-----T-ADRGKLIMACGTGKTFTSLKIAEDIAGK---------
gi|447197741|ref|WP_001274997.1|DNAdamageinducibleprotein[Acinetobacterbaumannii]_gi|408512324|gb|EKK13969.1|typeIIIrestrictio/	QRPALSAVKYGL---A-----E-ADRGKLIMACGTGKTFTSLRIAEELAGR---------
gi|820775897|ref|WP_046739909.1|damageinducibleprotein[Acinetobactersp.AG1]_gi|819909758|gb|KKW75938.1|damageinducibleprotein/1	QRPALQAVKHGL---A-----T-ADRGKLIMACGTGKTFTSLRIAEELAGR---------
gi|584595163|ref|WP_024160846.1|helicase[Acinetobacterpittii]_gi|576702530|emb|CDI28049.1|helicasedomaincontainingprotein[Acin/	QRPALSAVKYGL---A-----E-ADRGKLIMACGTGKTFTSLRIAEELAGC---------
gi|514963042|ref|WP_016651366.1|hypotheticalprotein[Acinetobactersp.NIPH2036]_gi|514162665|gb|EPG41391.1|hypotheticalproteinF90	QRPALSAVKYGL---A-----E-ADRGKLIMACGTGKTFTSLRIAEELVGR---------
gi|515965564|ref|WP_017396147.1|DNAdamageinducibleprotein[Acinetobacterhaemolyticus]/11667	QRPALSAVKYGL---A-----E-ADRGKLIMACGTGKTFTSLRIAEELAGR---------
gi|491346810|ref|WP_005204747.1|MULTISPECIES:hypotheticalprotein[Acinetobacter]_gi|480335823|gb|ENX56033.1|hypotheticalproteinF	QRPALQAVKHGL---A-----A-ADRGKLIMACGTGKTFTSLRIAEELAGR---------
gi|693044472|ref|WP_032215268.1|damageinducibleprotein[Escherichiacoli]_gi|607725731|gb|EZE08230.1|damageinducibleprotein[Esc/1	QTPALEAVKRGL---S-----I-SDRGKLIMACGTGKTFTSLRIAEAIAGR---------
gi|742382177|ref|WP_038861304.1|damageinducibleprotein[Cronobactermuytjensii]_gi|924333747|gb|ALB69766.1|damageinducibleprote/1	QTPALEAVKRGL---T-----T-ADRGKLIMACGTGKTFTSLRIAEAVAGR---------
gi|915912804|ref|WP_050927035.1|damageinducibleprotein[Yersiniaenterocolitica]_gi|899805802|emb|CQH32042.1|typeIrestrictionenz/	QIPALESVKRGL---T-----T-ADRGKLIMACGTGKTFTSLRIAEAVAGR---------
gi|921111327|gb|KON62781.1|typeIrestrictionenzymeEcoKIsubunitR[Komagataeibactereuropaeus]/11492	QETAIRRVLAGF---K-----C-HDRGRLIMACGTGKTFTSLKIAEQQVGT---------
gi|582024278|gb|AHI27615.1|DNAhelicaserestrictionenzymeTypeIIIRsubunit[KomagataeibacterxylinusE25]/11636	QETAIRRVLAGF---K-----N-HNRGRLIMACGTGKTFTSLKIAEQQVGA---------
gi|347662673|dbj|BAK86128.1|DNAhelicaserestrictionenzymeTypeIIIRsubunit[KomagataeibactermedellinensisNBRC3288]/11737	QETAIRRVLAGF---K-----C-HDRGRLIMACGTGKTFTSLKIAEQQVGA---------
gi|917445053|ref|WP_052051471.1|damageinducibleprotein[Acetobactertropicalis]/11429	QKTAIGNVLAGF---K-----N-HNRGRLIMACGTGKTFTSLKLAEELVGA---------
gi|453329445|dbj|GAC88472.1|DNAhelicaserestrictionenzymetypeIIIRsubunit[GluconobacterthailandicusNBRC3255]_gi|530287981|dbj|GAD	QDTAIKNVLAGF---I-----A-SDRGRLIMACGTGKTFTSLKLAEQQVGA---------
gi|889770922|ref|WP_048844278.1|damageinducibleprotein[Gluconobacterfrateurii]/11419	QGTAIKNVLAGF---K-----T-DDRGRLIMACGTGKTFTSLKLAEQQVGA---------
gi|504886208|ref|WP_015073310.1|hypotheticalprotein[Gluconobacteroxydans]_gi|411027954|gb|AFW01209.1|hypotheticalproteinB932_16	QDTAIKNVLAGF---I-----A-SDRGRLIMACGTGKTFTSLKLAEQQVGA---------
gi|480040211|gb|ENV79751.1|hypotheticalproteinF942_01641[AcinetobacterursingiiANC3649]/11432	QKQALQAVTEQLNY-P-----N-ISRGKLIMACGTGKTFTSLKIAEAMAGR---------
gi|822498222|ref|WP_046936504.1|damageinducibleprotein[Xanthomonasgardneri]_gi|821751224|gb|KLB07223.1|damageinducibleprotein/1	QRAALQDVRAGL---Q-----Q-ADRGKLIMACGTGKTFTSLKIAEDIAGK---------
gi|654059559|ref|WP_027705556.1|damageinducibleprotein[Zymobacterpalmae]/11635	QRQALNAVTAGF---A-----T-AERGKLIMACGTGKTFTSLKIAEAMAGR---------
gi|643535620|ref|WP_025224315.1|damageinducibleprotein[Methylophilaceaebacterium11]_gi|577704400|gb|EUJ10546.1|putativehelicas/	QQTALNAVVESF---K-----S-AERGKLIMACGTGKTFTSLKIAEAYAGK---------
MBA11I/11635	QQTALNAVVESF---K-----S-AERGKLIMACGTGKTFTSLKIAEAYAGK---------
gi|655355857|ref|WP_028762287.1|damageinducibleprotein[Shewanellacolwelliana]/11632	QKTAIDNVVREF---K-----T-TDRGKLIMACGTGKTFTSLRLAEQQAGK---------
gi|753204236|ref|WP_041510579.1|damageinducibleprotein[Shewanellasp.cp20]_gi|752457765|gb|KIO36326.1|damageinducibleprotein[S/1	QQTAITKVKEGL---A-----K-AERGTLLMACGTGKTFTSLKIAEELAGA---------
KPNNIH30III/11619	QQNAINAVKEGL---A-----N-AERGTLLMACGTGKTFTSLKIAEELAGA---------
gi|749295994|ref|WP_040120352.1|damageinducibleprotein[Klebsiellapneumoniae]_gi|723249300|gb|AIX81926.1|damageinducibleprotei/1	QQNAINAVKEGL---A-----N-AERGTLLMACGTGKTFTSLKIAEELAGA---------
gi|641639739|emb|CDQ54010.1|unnamedproteinproduct[Klebsiellapneumoniae]/11641	QQNAINAVKEGL---A-----N-AERGTLLMACGTGKTFTSLKIAEELAGA---------
gi|490797922|ref|WP_004660060.1|hypotheticalprotein[Acinetobactersp.NIPH236]_gi|479876779|gb|ENU27973.1|hypotheticalproteinF992	QKAALSSVKIGLYE-Q-----K-LDRGKLIMACGTGKTFTSLKIAEACAGA---------
gi|916750926|ref|WP_051357982.1|damageinducibleprotein[Acinetobactertjernbergiae]/11453	QKAALTSTRIGLYD-R-----Q-LERGKLIMACGTGKTFTSLKIAEECAGK---------
gi|691098662|ref|WP_032025060.1|damageinducibleprotein[Acinetobacterbaumannii]_gi|593567521|gb|EXR50735.1|restrictionendonucle/	QKAALANVKAGLYE-Q-----K-LERGKLIMACGTGKTFTSLKIAEACAGK---------
gi|736669405|ref|WP_034676049.1|damageinducibleprotein[Acinetobacterbaumannii]/11643	QKAALVNVKAGLYD-Q-----K-LERGKLIMACGTGKTFTSLKIAEECAGK---------
gi|491368928|ref|WP_005226826.1|hypotheticalprotein[Acinetobactersp.NIPH1847]_gi|480173259|gb|ENX09797.1|hypotheticalproteinF89	QKAALTSVKLGLYE-Q-----K-LERGKLIMACGTGKTFTSLKIAEACAGK---------
gi|796853993|ref|WP_045795953.1|damageinducibleprotein[Acinetobacterindicus]_gi|787929761|gb|KJV44219.1|damageinducibleprotei/1	QKAALTNVKIGLYE-Q-----K-LERGKLIMACGTGKTFTSLKIAEECAGK---------
gi|295797463|gb|ADG32253.1|typeIIIrestrictionproteinressubunit[ThiomonasintermediaK12]/11676	QKTALEKVTAGLAD---------ADRGKLIMACGTGKTYTALKIAEAVAGA---------
gi|499871179|ref|WP_011551913.1|DNAdamageinducibleprotein[Myxococcusxanthus]_gi|108467544|gb|ABF92729.1|putativerestriction/mo/	QKTALKAVLEGLNA---------SERGKLIMACGTGKTFTSLKIAEHLAGE---------

gi|495313852|ref|WP_008038600.1|DNAdamageinducibleprotein[Bartonellatamiae]_gi|395424181|gb|EJF90368.1|hypotheticalproteinME5_/	QEIASKNVLKGLEL---------ADRGKLIMACGTGKTFTSLKIAEAQAGK---------
gi|501453335|ref|WP_012476784.1|DNAdamageinducibleprotein[Leptospirabiflexa]_gi|167777824|gb|ABZ96123.1|Helicase[Leptospirabif/	QKEALVSVVGGLKT---------ADRGKLIMACGTGKTFTSLKIAESVAGR---------
gi|738104613|ref|WP_036063193.1|damageinducibleprotein[Leptospiranoguchii]/11652	QKSAVEQVISGLTS---------NDRGKLIMACGTGKTFTSLKIAERIAGK---------
gi|410001039|gb|EKO51661.1|typeIIIrestrictionenzyme,ressubunit[Leptospirakirschneristr.200802841]/11651	QKTAVEQVVSGLSV---------DDRGKLIMACGTGKTFTSLKIAERMTGK---------
gi|490901851|ref|WP_004763774.1|typeIIIrestrictionenzyme,ressubunit[Leptospirakirschneri]_gi|464209249|gb|EMN24014.1|typeIIIres	QKTAVEQVVSGLSV---------DDRGKLIMACGTGKTFTSLKIAERMTGK---------
gi|464398131|gb|EMO65902.1|typeIIIrestrictionenzyme,ressubunit[Leptospirakirschneristr.200803703]/11648	QKTAVEQVVSGLSV---------DDRGKLIMACGTGKTFTSLKIAERMTGK---------
gi|945029024|gb|KQQ84900.1|damageinducibleprotein[Xanthomonassp.Leaf131]/11423	QQTALRAVMVGLE--------S-ADRGKLIMACGTGKTFTSLKIAEAIAGK---------
gi|896511314|ref|WP_049418183.1|damageinducibleprotein[Stenotrophomonasmaltophilia]/11639	QENALKATMVGLA--------E-ADRGKLIMACGTGKTFTSLKIAESMAGK---------
gi|896513082|ref|WP_049419616.1|damageinducibleprotein[Stenotrophomonasmaltophilia]/11638	QESALKAVTAGLQ--------D-ADRGKLIMACGTGKTFTSLKIAEQLAGK---------
gi|896580616|ref|WP_049469425.1|damageinducibleprotein[Stenotrophomonasmaltophilia]/11641	QQSALKAVTAGLQ--------D-ADRGKLIMACGTGKTFTSLKIAESMAGK---------
gi|648289350|ref|WP_026070499.1|damageinducibleprotein[Stenotrophomonasmaltophilia]/11639	QESALKAVTAGLK--------D-ADRGKLIMACGTGKTFTSLKIAEALAGK---------
gi|656272786|ref|WP_029218442.1|damageinducibleprotein[Xanthomonascassavae]/11639	QESALKAVTAGLQ--------D-ADRGKLIMACGTGKTFTSLKIAEQLAGK---------
gi|406976236|gb|EKD98747.1|hypotheticalproteinACD_23C00290G0002[unculturedbacterium]/11679	QQNALNATLAGFQ--------R-ESRGKLIMACGTGKTFTSLKIAEAYAGA---------
gi|828145737|ref|WP_047238120.1|damageinducibleprotein[Chromobacteriumsubtsugae]/11645	QIRALARVEVGLA--------T-ADRGKLIMACGTGKTFTSLKIAEKLVGA---------
gi|654311174|ref|WP_027824327.1|damageinducibleprotein[Laribacterhongkongensis]/11641	QIRALARVEAGLA--------N-AERGKLIMACGTGKTFTSLKIAEKLVGA---------
gi|757242173|gb|KIR15516.1|typeIrestrictionenzymeEcoKIsubunitR[Pseudomonasfluorescens]/11778	QKRALLSVSSGLQ--------N-ADRGKLIMACGTGKTFTSLKIAEQLAGA---------
gi|653605161|ref|WP_027616682.1|damageinducibleprotein[Pseudomonassp.URHB0015]/11639	QKRALLSVSSGLQ--------S-ADRGKLIMACGTGKTFTSLKIAEQLAGA---------
gi|520868025|ref|WP_020308060.1|helicase[Pseudomonassyringae]_gi|519159034|gb|EPM49935.1|helicasedomaincontainingprotein[Pseud/	QKRALLSVASGLQ--------T-ADRGKLIMACGTGKTFTSLKIAEQLAGA---------
gi|940156567|gb|KPX71668.1|Helicasedomaincontainingprotein[Pseudomonasamygdalipv.lachrymans]/11677	QKRALLSVANGLQ--------N-AERGKLIMACGTGKTFTSLRIAEQLAGA---------
gi|505460508|ref|WP_015647610.1|helicase[Pseudomonassp.GLE121]_gi|499110212|ref|YP_007974374.1|helicasedomaincontainingprotein/	QKTALKATEIGLK--------T-AERGKLIMACGTGKTFTSLKIAEQLAGR---------
gi|940235551|gb|KPY45311.1|hypotheticalproteinALO48_200154[Pseudomonassyringaepv.rhaphiolepidis]/11631	QRTALNATEAGLK--------T-AERGKLIMACGTGKTFTSLKIAEQLAGR---------
gi|520880808|ref|WP_020312235.1|helicase[Pseudomonassyringae]_gi|519172158|gb|EPM62027.1|helicasedomaincontainingprotein[Pseud/	QKRALLAVTSGLQ--------T-EARGKLIMACGTGKTFTSLKIAEKIAGK---------
gi|829046479|ref|WP_047282259.1|damageinducibleprotein[Pseudomonasfluorescens]/11630	QQTALNATAAGLK--------D-AERGKLIMACGTGKTFTSLKIAEKLAGK---------
gi|499415493|ref|WP_011102960.1|DNAdamageinducibleprotein[Pseudomonassyringaegroupgenomosp.3]_gi|28867279|ref|NP_789898.1|heli/	QQTALNATAAGLN--------D-AERGKLIMACGTGKTFTSLKIAERLAGK---------
gi|730238299|ref|WP_033942897.1|damageinducibleprotein[Pseudomonasaeruginosa]/11629	QQTALNAVAAGLR--------D-AERGKLIMACGTGKTFTSLKIAEQQAGK---------
gi|495310125|ref|WP_008034873.1|DNAdamageinducibleprotein[Pseudomonassp.GM60]_gi|398186685|gb|EJM74052.1|putativehelicase[Pseu/	QKTALNAVAAGLK--------N-AERGKLIMACGTGKTFTSLKIAEQQAGK---------
gi|779992653|ref|WP_045428690.1|damageinducibleprotein[Pseudomonassp.MT1]_gi|699967872|dbj|BAP80334.1|DNAhelicaserestrictione/1	QQTALNAVMAGLK--------D-AERGKLIMACGTGKTFTSLKIAERLAGK---------
gi|786037289|ref|WP_045733274.1|damageinducibleprotein[Pseudomonaspseudoalcaligenes]_gi|782987904|gb|KJU79152.1|damageinducib/1	QQTALNAVAAGLK--------E-AERGKLIMACGTGKTFTSLKIAEQQAGK---------
gi|749666366|ref|WP_040265445.1|damageinducibleprotein[Pseudomonasrhodesiae]/11632	QQTALNAVAAGLK--------D-AERGKLIMACGTGKTFTSLKIAEQQAGK---------
gi|734902433|ref|WP_034133698.1|damageinducibleprotein[Pseudomonasfluorescens]/11635	QQTALNAVAAGLK--------E-AERGKLIMACGTGKTFTSLKIAEQQAGK---------
gi|490551031|ref|WP_004416146.1|typeIIrestrictionmodificationsystemrestriction/helicasesubunit[Pseudomonassyringae]_gi|4432823/	QQTALNAVAAGLK--------D-AERGKLIMACGTGKTFTSLKIAEQQAGK---------
gi|489199911|ref|WP_003109152.1|DNAdamageinducibleprotein[Pseudomonasaeruginosa]_gi|552648708|gb|ERY63184.1|hypotheticalprotei/	QQTALNAVVAGLK--------D-AERGKLIMACGTGKTFTSLKIAEQQAGK---------
gi|730641237|ref|WP_034061328.1|damageinducibleprotein[Pseudomonasaeruginosa]/11636	QKTALNAVAAGLK--------N-AERGKLIMACGTGKTFTSLKIAERLAGK---------
gi|751304980|ref|WP_041013386.1|damageinducibleprotein[Pseudomonasxanthomarina]_gi|703407611|emb|CEG51014.1|Helicasedomaincon/1	QQTALNAVAAGLK--------D-AERGKLIMACGTGKTFTSLKIAERLAGK---------
gi|939335207|ref|WP_054881520.1|damageinducibleprotein[Pseudomonassp.NBRC111128]/11632	QQTALNAVAAGLK--------D-AERGKLIMACGTGKTFTSLKIAEQQAGK---------
gi|757693555|ref|WP_042923792.1|damageinducibleprotein[Pseudomonaschloritidismutans]_gi|780910610|gb|KJS78302.1|damageinducib/1	QKTALNAVAAGLK--------T-AERGKLIMACGTGKTFTSLKIAERLAGK---------
gi|665953944|ref|WP_031319989.1|damageinducibleprotein[Pseudomonastolaasii]/11626	QQTALNAVEAGLK--------D-VERGKLIMACGTGKTFTSLKIAERLAGK---------
gi|771600573|ref|WP_045213746.1|damageinducibleprotein[Pseudomonassp.21]_gi|770081123|gb|KJJ98634.1|damageinducibleprotein[Ps/1	QKTALNAVAAGLK--------D-AERGKLIMACGTGKTFTSLKIAEQQAGK---------
gi|740871396|ref|WP_038656644.1|damageinducibleprotein[Pseudomonasstutzeri]_gi|627772214|gb|AHY41437.1|damageinducibleprotein/1	QQTALNAVAAGLK--------D-AERGKLIMACGTGKTFTSLKIAERLAGK---------
gi|946242205|gb|KRC93782.1|damageinducibleprotein[Pseudomonassp.Root9]/11634	QQTALNAVAAGLK--------D-AERGKLIMACGTGKTFTSLKIAEQQAGK---------
gi|553777322|ref|WP_023109107.1|MULTISPECIES:hypotheticalprotein[Pseudomonas]_gi|552638674|gb|ERY53184.1|hypotheticalproteinQ05	QQTALNAVAAGLK--------D-AERGKLIMACGTGKTFTSLKIAEQQAGK---------
gi|896383370|ref|WP_049324618.1|damageinducibleprotein[Pseudomonasstutzeri]/11627	QQTALNAVSAGLK--------G-AERGKLIMACGTGKTFTSLKIAERLAGK---------
gi|696158510|gb|AIT41787.1|putativehelicase[Pseudomonassp.LM8]/11630	QQSALNAVAAGLK--------D-ADRGKLIMACGTGKTFTGLKIAERLAGK---------
gi|504634892|ref|WP_014821994.1|DNAdamageinducibleprotein[Pseudomonasstutzeri]_gi|390985249|gb|AFM35242.1|helicasedomainconta/1	QQSALNAVAAGLK--------D-ADRGKLIMACGTGKTFTGLKIAERLAGK---------
gi|544783974|ref|WP_021206729.1|DNAdamageinducibleprotein[Pseudomonasstutzeri]_gi|533224779|gb|EQM79688.1|DNAdamageinduciblep/1	QQSALNAVAAGLK--------A-ADRGKLIMACGTGKTFTGLKIAERLAGK---------
gi|504567385|ref|WP_014754487.1|MULTISPECIES:DNAdamageinducibleprotein[Pseudomonas]_gi|388559493|gb|AFK68634.1|helicasedomain/1	QQTALNAVAAGLK--------D-AERGKLIMACGTGKTFTSLKIAEQQTGK---------
gi|654441469|ref|WP_027912052.1|damageinducibleprotein[Pseudomonassp.URIL14HWK12:I7]/11630	QQTALNAVAAGLK--------D-AERGKLIMACGTGKTFTSLKIAEQQAGK---------
gi|941470066|ref|WP_055127894.1|damageinducibleprotein[Pseudomonasmediterranea]/11631	QQTALNAVAAGLK--------D-AERGKLIMACGTGKTFTSLKIAEQQAGK---------
gi|557815599|ref|WP_023446184.1|DNAdamageinducibleprotein[Pseudomonaschloritidismutans]_gi|557364081|gb|ESQ98190.1|DNAdamagei/1	QQTALNAVAAGLK--------E-AERGKLIMACGTGKTFTSLKIAEQQAGK---------
gi|727814791|ref|WP_033898247.1|damageinducibleprotein[Pseudomonassp.FH1]_gi|567648005|gb|ETK23229.1|helicasedomaincontaining/1	QQTALNAVAAGLK--------D-AERGKLIMACGTGKTFTSLKIAEQQAGK---------
gi|503278702|ref|WP_013513363.1|DNAdamageinducibleprotein[Desulfovibrioaespoeensis]_gi|316942375|gb|ADU61426.1|typeIIIrestrict/	QQDAYTAVEKGLK--------T-ADRGKLIMACGTGKTLTSLKIAEGLAGK---------
gi|505228796|ref|WP_015415898.1|typeIIIrestrictionendonucleasesubunitR[Desulfovibriopiezophilus]_gi|451896976|emb|CCH49855.1|Ty	QQAAYDAVEKGLK--------T-ADRGKLIMACGTGKTFTSLKIAEGLAGK---------
gi|517732543|ref|WP_018902751.1|hypotheticalprotein[Variovoraxparadoxus]/11675	QATALHAVLAGLQ--------S-ADRGKLIMACGTGKTFASLKIAEALAGK---------
gi|550909475|ref|WP_022662570.1|DNAdamageinducibleprotein[Desulfovibriolongus]/11637	QQKALDAVREGLE--------S-ADRGKLIMACGTGKTYASLKIAEVLAGK---------
gi|945494542|gb|KQV50024.1|damageinducibleprotein[Massiliasp.Root335]/11647	QQQALDAALSGLQ--------T-ADRGKLIMACGTGKTFTSLKIAEALAGR---------
gi|334196917|gb|AEG70102.1|helicasedomainprotein[RalstoniasolanacearumPo82]/11663	QKSALNGAIHGLA--------G-ADRGKLIMACGTGKTFTSLKIAETLAGA---------
gi|656006746|ref|WP_029048170.1|damageinducibleprotein[Cupriavidussp.amp6]/11661	QTNALNAAVHGLA--------N-ADRGKLIMACGTGKTFTSLKIAESLAGA---------
BPS2196ORF3054P/11659	QTNALNAVVHGLA--------D-ADRGKLIMACGTGKTFTSLKIAEAMAGA---------
gi|740932312|ref|WP_038717332.1|damageinducibleprotein[Burkholderiapseudomallei]_gi|760211882|gb|KIX58314.1|damageinduciblepr/1	QTNALNAVVHGLA--------D-ADRGKLIMACGTGKTFTSLKIAEAMAGA---------
gi|755810813|ref|WP_042583861.1|damageinducibleprotein[Burkholderiasp.MSHR3999]_gi|755174952|gb|KIP19790.1|DEAD/DEAHboxhelicas/	QASALKAVIHGLA--------D-ADRGKLIMACGTGKTMTALKIAETLAGA---------
gi|484586570|gb|AGK48195.1|DEAD/DEAHboxhelicasefamilyprotein[BurkholderiathailandensisMSMB121]/11646	QTSALKAVIHGLA--------D-ADRGKLIMACGTGKTMTALKIAETLAGA---------
gi|737582935|ref|WP_035553941.1|damageinducibleprotein[Burkholderiasp.9120]/11658	QTSALNAVVHGLA--------E-ADRGKLIMACGTGKTFTSLKIAETLAGA---------
gi|740989872|ref|WP_038773225.1|damageinducibleprotein[Burkholderiapseudomallei]_gi|705645291|gb|KGS70691.1|DEAD/DEAHboxhelica/	QTNALNAVAHGLA--------D-ADRGKLIMACGTGKTFTSLKIAETLAGA---------
gi|654912525|ref|WP_028363424.1|damageinducibleprotein[Burkholderiasp.JPY366]/11659	QTNALNAVVHGLA--------D-ADRGKLIMACGTGKTFTSLKIAEALAGA---------
gi|740955879|ref|WP_038740512.1|damageinducibleprotein[Burkholderiapseudomallei]_gi|714684932|gb|KGW74438.1|DEAD/DEAHboxhelica/	QTNALNAVVHGLA--------D-ADRGKLIMACGTGKTFTSLKIAETLAGP---------
gi|919203714|ref|WP_052759215.1|damageinducibleprotein[Xanthomonasaxonopodis]/11388	QQAALKAVREGFQK---------HERGKLIMACGTGKTFTSLKIVEQET-----------
gi|522152381|ref|WP_020663589.1|hypotheticalprotein[Amycolatopsisbenzoatilytica]/11605	QQEAVDAVFDGFAA---------HDRGKLIMACGTGKTFTSLKVAERLAAER--------

```
gi|663196013|ref|WP_030233899.1|damageinducibleprotein[Streptomycessp.NRRLS455]/11641    QLEAIDAVFRGFEE---------HERGKLIMACGTGKTFTGLKIVERLAAERTA------
gi|750429553|ref|WP_040711444.1|damageinducibleprotein[Nocardiopsispotens]/11622    QRDAIDDVFAGFAG---------HERGQLIMACGTGKTFTSLKIAERLHRERVE------
gi|518656139|ref|WP_019819475.1|hypotheticalprotein[Saccharomonosporasaliphila]/11623    QRAAIDEVFAGFAK---------HDRGKLIMACGTGKTFTSLKIAERLHRERAE------
gi|759722933|ref|WP_043433758.1|damageinducibleprotein[Streptomycespluripotens]_gi|733404978|gb|KHK94361.1|damageinduciblepro/1    QKAAIDDVFAGFVE---------RDRGKLIMACGTGKTFTSLKIAERLQQERAE------
gi|663424167|ref|WP_030419030.1|damageinducibleprotein[Streptomycessp.NRRLF5065]/11627    QKAAIDDVFAGFAE---------HDRGKLIMACGTGKTFTSLKIAERHQQERAE------
gi|663412917|ref|WP_030408353.1|damageinducibleprotein[Streptomycessp.NRRLF5917]/11630    QKAAIDDVFAGFAK---------HDRGKLIMACGTGKTFTSLKIAERLQQERAE------
gi|671521483|ref|WP_031506489.1|damageinducibleprotein[Streptomycesmegasporus]/11630    QKAAIDDVFAGFTK---------QNRGKLIMACGTGKTFTSLKIAERLQQERAE------
gi|740053144|ref|WP_037902776.1|damageinducibleprotein[Streptomycessp.TAA204]/11626    QVAAIDDVFAGFAG---------HNRGKLIMACGTGKTFTGLKIAERLQQERAQ------
gi|663409375|ref|WP_030404974.1|MULTISPECIES:damageinducibleprotein[Streptomyces]/11627    QRAAIDDVFAGFTE---------HKRGKLIMACGTGKTFTGLKIVERLQKERAA------
gi|746628608|ref|WP_039654277.1|damageinducibleprotein[Streptomycessp.MUSC125]_gi|745644381|gb|KIE25557.1|damageinducibleprot/1    QRAAIDDVFAGFAD---------HNRGKLIMACGTGKTFTGLKIVERLQKERAA------
gi|727322066|ref|WP_033777320.1|damageinducibleprotein[Salinisporapacifica]/11627    QREAIDDVFARFAE---------HDRGKLIMACGTGKTFTSLKIAERLQRERAV------
gi|653653242|ref|WP_027655726.1|damageinducibleprotein[Salinisporapacifica]/11618    QREAIENAFTGFAQ---------HSRGKLIMACGTGKTFTSLKIAERLQRERAA------
gi|648632543|ref|WP_026324294.1|damageinducibleprotein[Salinisporapacifica]/11621    QREAVEDVFAGLTR---------HDRGKLIMACGTGKTFTSLKIAERLQSERAA------
gi|881018002|ref|WP_048738463.1|damageinducibleprotein[Corynebacteriumfalsenii]/11676    QATAIAKALEGFGT---------HDRGKLIMACGTGKTFTSLRLAEKFTEDK--------
gi|505465526|ref|WP_015652417.1|helicase[Corynebacteriumcallunae]_gi|459385704|gb|AGG67995.1|helicase[CorynebacteriumcallunaeDS    QQTAIEKAIEGFQT---------HDRGKLIMACGTGKTFTALRLSEEVARF---------
gi|41223062|emb|CAF19007.1|DNAorRNAhelicaseofsuperfamilyII[CorynebacteriumglutamicumATCC13032]/11646    QQTAIEKAIEGFQT---------HDRGKLIMACGTGKTFTALRLSEEVARL---------
CGL13032I/11643    QQTAIEKAIEGFQT---------HDRGKLIMACGTGKTFTALRLSEEVARL---------
gi|751257081|ref|WP_040967675.1|damageinducibleprotein[Corynebacteriumglutamicum]_gi|748811620|gb|AJE67931.1|damageinduciblep/1    QDTAITKAIEGFET---------HDRGKLIMACGTGKTFTALRLAERVADS---------
gi|749040539|ref|WP_040086524.1|damageinducibleprotein[Corynebacteriumhumireducens]/11663    QHTAIEKTLAGFDT---------HDRGKLIMACGTGKTFTALRLAEQVAIRNAPPTATKE
gi|491662899|ref|WP_005519615.1|helicase[Corynebacteriummatruchotii]_gi|224946756|gb|EEG27965.1|helicaseCterminaldomainprotein/    QVTAIDKTLAGFQI---------HDRGKLIMACGTGKTFTALRLAEQFAEKK--------
gi|746717312|ref|WP_039676231.1|damageinducibleprotein[Corynebacteriumminutissimum]_gi|734991118|gb|KHO29219.1|damageinducibl/1    QQEAISKAIAGFEA---------HDRGKLIMACGTGKTFTALRLVEQYAENN--------
gi|908654892|emb|CQD15529.1|SuperfamilyIIDNA/RNAhelicase(modularprotein)[Corynebacteriumstriatum]/11674    QQEAISKAIAGFET---------HDRGKLIMACGTGKTFTALRLAEQYAENN--------
gi|895994446|ref|WP_049064418.1|damageinducibleprotein[Corynebacteriumaurimucosum]/11667    QQEAISKAIAGFET---------HDRGKLIMACGTGKTFTALRLAEQFAENN--------
gi|896123331|ref|WP_049145390.1|damageinducibleprotein[Corynebacteriumaurimucosum]/11669    QQEAISKAIAGFET---------HDRGKLIMACGTGKTFTALRLAEQYAENN--------
gi|817681178|ref|WP_046645422.1|damageinducibleprotein[Corynebacteriumstriatum]_gi|817551679|gb|KKO79868.1|damageinduciblepro/1    QQEAISKAIAGFET---------HDRGKLIMACGTGKTFTALRLAEQFAENN--------
gi|896139239|ref|WP_049159146.1|damageinducibleprotein[Corynebacteriumaurimucosum]/11675    QQEAISKAIAGFET---------HDRGKLIMACGTGKTFTALRLAEQFAENN--------
gi|489943947|ref|WP_003847254.1|helicase[Corynebacteriumammoniagenes]_gi|295968218|gb|EFG81467.1|helicaseCterminaldomainprotei/    QVEAIDMVMTGFGN---------NDRGKLIMACGTGKTFTSLRLAEQYAKEN--------
gi|516653464|ref|WP_018022453.1|helicase[Corynebacteriumdoosanense]_gi|697778282|gb|AIT61018.1|damageinducibleprotein[Coryneba/    QAEAIAKAMAGFET---------HDRGKLIMACGTGKTFTSLRLAEQVAEAN--------
gi|493894179|ref|WP_006840129.1|helicase[Corynebacteriumlipophiloflavum]_gi|227078819|gb|EEI16782.1|helicaseCterminaldomainpro/    QQTAIDKAIEGFET---------ADRGKLIMACGTGKTFTALRLAEQIADRN--------
gi|740831472|ref|WP_038616755.1|damageinducibleprotein[Corynebacteriumpseudotuberculosis]_gi|666103058|gb|AIG06935.1|putativeh/    QAEAIEKALEGFNA---------HDRGKLIMACGTGKTFTSLRLAEQFAHKN--------
gi|518023322|ref|WP_019193530.1|helicase[Corynebacteriumtimonense]/11655    QAEAIESVVGGFEA---------HDRGKLIMACGTGKTFTALRLAEQMAENN--------
gi|552767697|ref|WP_023019559.1|MULTISPECIES:hypotheticalprotein[Corynebacterium]_gi|550739013|gb|ERS38609.1|hypotheticalprotei    QSEAIEKALAGFAT---------HDRGKLIMACGTGKTFTSLRLAEQFAQQR--------
gi|652659121|ref|WP_027017812.1|damageinducibleprotein[Corynebacteriumpseudodiphtheriticum]/11644    QSEAIEKALAGFAT---------HDRGKLIMACGTGKTFTSLRLAEQFAREN--------
gi|749039309|ref|WP_040085390.1|damageinducibleprotein[Corynebacteriumhumireducens]/11657    QQTAIEKVLEGFST---------HDRGKLIMACGTGKTFTALRLAERVAKNN--------
gi|740835591|ref|WP_038620874.1|damageinducibleprotein[Corynebacteriumulcerans]_gi|698973854|gb|AIU33664.1|HelicaseCterminald/1    QQQAIDKVIEGFET---------HDRGKLIMACGTGKTFTSLRLAEQIATNN--------
gi|805307599|ref|WP_046096452.1|damageinducibleprotein[Corynebacteriumulcerans]_gi|803357323|gb|AKA97638.1|Damageinduciblepro/1    QQQAIDKVIEGFET---------HDRGKLIMACGTGKTFTSLRLAEQVAANN--------
gi|504083555|ref|WP_014317549.1|helicase[Corynebacteriumdiphtheriae]_gi|371584249|gb|AEX47914.1|superfamilyIIDNA/RNAhelicase[Co    QQTAIEKTLQGFAT---------HDRGKLIMACGTGKTFTSLRLAEKVAENN--------
gi|896182193|ref|WP_049193580.1|damageinducibleprotein[Corynebacteriumjeikeium]/11645    QQTAIEKTLQGFTT---------YDRGKLIMACGTGKTFTALRLAEKVAENN--------
gi|917636459|ref|WP_052205460.1|damageinducibleprotein[Corynebacteriumriegelii]_gi|914295811|gb|AKV59187.1|damageinduciblepro/1    QQEAIDKVMDGFRT---------HDRGKLIMACGTGKTFTALRLAERVAEDN--------
gi|737013522|ref|WP_035009205.1|damageinducibleprotein[Corynebacteriumjeikeium]/11651    QQQAIENVMAGFNT---------HDRGKLIMACGTGKTFTSLRLAEQVAEQN--------
gi|737006291|ref|WP_035002094.1|damageinducibleprotein[Corynebacteriumjeikeium]/11658    QQEAIDRAIAGFST---------HDRGKLIMACGTGKTFTALRLAEKVAENN--------
gi|737010378|ref|WP_035006133.1|damageinducibleprotein[Corynebacteriumjeikeium]/11650    QQEAIDRAIEGFST---------HDRGKLIMACGTGKTFTALRLAEQVAENN--------
gi|737010473|ref|WP_035006228.1|damageinducibleprotein[Corynebacteriumjeikeium]/11657    QEAAISKVLEGFAT---------HDRGKLIMACGTGKTFTALRLAERVAEKN--------
gi|737006250|ref|WP_035002053.1|damageinducibleprotein[Corynebacteriumjeikeium]/11658    QETAISKVLEGFAT---------HDRGKLIMACGTGKTFTALRLAERVAEKN--------
gi|923018168|ref|WP_053412254.1|damageinducibleprotein[Corynebacteriumlactis]_gi|922845184|gb|ALA67504.1|helicase[Corynebacter/    QQAAIDKAMEGFAT---------HDRGKLIMACGTGKTFTALRLAERVAEER--------
gi|923018885|ref|WP_053412970.1|damageinducibleprotein[Corynebacteriumlactis]_gi|922845845|gb|ALA68165.1|helicase[Corynebacter/    QQAAIDKAMEGFAT---------QTRGKLIMACGTGKTFTALRLAERVAEDR--------
gi|512464870|ref|WP_016422869.1|hypotheticalprotein[Corynebacteriumsp.HFH0082]_gi|512047830|gb|EPD47505.1|hypotheticalproteinHM    QQEAIDSAMAGFVT---------HDRGKLIMACGTGKTFTALRLAERVAEEN--------
gi|881012697|ref|WP_048733406.1|damageinducibleprotein[Corynebacteriumurealyticum]/11670    QQEAIDSAMAGFAT---------RERGKLIMACGTGKTFTALRLAERVAEAN--------
gi|755608478|ref|WP_042530519.1|damageinducibleprotein[Corynebacteriumsingulare]_gi|754340239|gb|AJI78730.1|putativehelicase[C/    QQAAIDKAIEGFKT---------HDRGKLIMACGTGKTFTALRLAERVAEDN--------
gi|491421796|ref|WP_005279593.1|helicase[Corynebacteriumaccolens]_gi|227077180|gb|EEI15143.1|helicaseCterminaldomainprotein[Co/    QQAAIDKAIEGFDT---------HDRGKLIMACGTGKTFTALRLAERVAENN--------
gi|550752286|gb|ERS51451.1|hypotheticalproteinHMPREF1282_00033[Corynebacteriumsp.KPL1856]_gi|550759961|gb|ERS58964.1|hypothetic    QQTAIDKAIEGFKT---------HDRGKLIMACGTGKTFTALRLAERVAEEN--------
gi|497955053|ref|WP_010269209.1|helicase[Corynebacteriumbovis]/11599    QREAVDKVLAGFRT---------HDRGQLIMACGTGKTFTALRLAEEWAENN--------
gi|860580040|ref|WP_048470569.1|hypotheticalprotein[Mycobacteriumchlorophenolicum]_gi|859098575|gb|KMO76871.1|typeIrestrictione    QVDAINAVMKGFEVG--------NDRGKLIMACGTGKTFTALKLAERIAGDA--------
gi|262085905|gb|ACY21873.1|typeIIIrestrictionproteinressubunit[GordoniabronchialisDSM43247]/11632    QQQAIDAVFDGFAAG--------NDRGKLIMACGTGKTFTALKIAERTAAEN--------
gi|495996338|ref|WP_008720917.1|typeIIIrestrictionendonucleasesubunitR[Rhodococcussp.AW25M09]_gi|443412350|emb|CCQ18455.1|TypeI    QVEAIDAVFAGFSAG--------NDRGKLIMACGTGKTFTALKIAERTAAEA--------
gi|739284636|ref|WP_037147410.1|damageinducibleprotein[Rhodococcusfascians]/11641    QVEAIDAVFAGFSAG--------NDRGKLIMACGTGKTFTALKIAERTAAEA--------
gi|739328195|ref|WP_037190574.1|damageinducibleprotein[Rhodococcusfascians]/11643    QVEAIDAVFAGFSAG--------NDRGKLIMACGTGKTFTALKIAERTAAEA--------
gi|811698335|emb|CMN21041.1|helicase[Mycobacteriumtuberculosis]/11725    QQQAIDAVFRGFAVG--------NDRGKLIMACGTGKTFTALKIAERIAADN--------
gi|801577898|emb|CFK23773.1|helicase[Mycobacteriumtuberculosis]/11675    QQQAIDAVFRGFAVG--------NDRGKLIMACGTGKTFTALKIAERIAADN--------
MTU18II/11606    QQQAIDAVFRGFAVG--------NDRGKLIMACGTGKTFTALKIAERIAADN--------
gi|887719858|emb|CMT27886.1|helicase[Mycobacteriumtuberculosis]/11667    QQQAIDAVFRGFAVG--------NDRGKLIMACGTGKTFTALKIAERIAADN--------
gi|621694743|gb|KAS87156.1|hypotheticalproteinAL70_01801[MycobacteriumtuberculosisTKK_03_0115]/11604    QQQAIDAVFRGFAVG--------NDRGKLIMACGTGKTFTALKIAERIAADN--------
gi|888128072|emb|CLQ84422.1|helicase[Mycobacteriumtuberculosis]_gi|889917090|emb|CLY86893.1|helicase[Mycobacteriumtuberculosis]    QQQAIDAVFRGFAVG--------NDRGKLIMACGTGKTFTALKIAERIAADN--------
gi|884823424|emb|CKL96672.1|helicase[Mycobacteriumtuberculosis]/11466    QQQAIDAVFRGFAVG--------NDRGKLIMACGTGKTFTALKIAERIAADN--------
gi|505103239|ref|WP_015290341.1|hypotheticalprotein[Mycobacteriumcanettii]_gi|432162733|emb|CCK60117.1|Conservedproteinofunknow    QQQAIDAVFRGFAVG--------NDRGKLIMACGTGKTFTALKIAERTAAEN--------
gi|505116250|ref|WP_015303352.1|hypotheticalprotein[Mycobacteriumcanettii]_gi|432158726|emb|CCK56026.1|Conservedproteinofunknow    QQQAIDAVFRGFAVG--------NDRGKLIMACGTGKTFTALKIAERIAAEN--------
gi|432154735|emb|CCK51974.1|Conservedproteinofunknownfunction(part1)[MycobacteriumcanettiiCIPT140060008]/11588    QQQAIDAVFRGFAVG--------NDRGKLIMACGTGKTFTALKIAERIAAEN--------
```

```
gi|809008027|ref|WP_046295375.1|hypotheticalprotein[Mycobacteriumsp.UM_Kg17]/11624    QDQAIDAVFRGFAAG--------NERGKLIMACGTGKTFTALKIAERTATDN--------
gi|808802633|ref|WP_046284139.1|hypotheticalprotein[Mycobacteriumsp.UM_NZ2]/11624    QEQAIDAVFRGFAAG--------NDRGKLIMACGTGKTFTALKIAERTAAEN--------
gi|750125774|ref|WP_040429852.1|damageinducibleprotein[Corynebacteriumnuruki]/11668    QSEAVDAVFQGWAEG----DGTTTDRGKLIMACGTGKTFTSLKIAERLAGDL--------
gi|602237614|gb|EYT60960.1|damageinducibleprotein[Dietziasp.UCDTHP]/11473    QQTAVDKVFTGFDEG--------NDRGKLVMACGTGKTFTALKIAERTAAGN--------
gi|665897412|ref|WP_031264618.1|damageinducibleprotein[Dietziasp.UCDTHP]_gi|602237599|gb|EYT60945.1|helicase[Dietziasp.UCDTH/11    QQTAVDKVFTGFDEG--------NDRGKLVMACGTGKTFTALKIAERTAADN--------
gi|502903674|ref|WP_013138650.1|helicase[Segniliparusrotundus]_gi|296181291|gb|ADG98197.1|typeIIIrestrictionproteinressubunit[S    QECAVEKVFEGFGLG--------NERGKLVMACGTGKTFTSLRIAERAAAEN--------
gi|780248306|ref|WP_045538930.1|hypotheticalprotein[Gordoniasp.no.9]_gi|778142514|gb|KJR06917.1|hypotheticalproteinUG54_12320[G    QQLAIDKVFTGFAAG--------NDRGKLIMACGTGKTFTALQIAERTADEN--------
gi|665897368|ref|WP_031264574.1|damageinducibleprotein[Dietziasp.UCDTHP]_gi|602239137|gb|EYT62483.1|helicase[Dietziasp.UCDTH/11    QQEALDAVFRGFAEG--------NERGKLIMACGTGKTFTSLKIAEQTALDN--------
gi|493372923|ref|WP_006329236.1|helicase[Gordoniarhizosphera]_gi|403207917|dbj|GAB88168.1|hypotheticalproteinGORHZ_006_00370[Go    QQAAIDDVFAEFGRG--------LDRGQMIMACGTGKTFTALKIAERTVEDN--------
gi|764904870|ref|WP_044487821.1|hypotheticalprotein[Mycobacteriumtuberculosis]_gi|625005353|gb|KBZ57120.1|hypotheticalproteinK8    QATAIEEVFSGFAAG--------NDRGKLIMACGTGKTFTALKIAERTVEEH--------
gi|927068212|ref|WP_053778441.1|damageinducibleprotein[Gordoniasp.NB41Y]_gi|926660541|gb|EMP14526.2|damageinducibleprotein[G/11    QITAIEKVFAGFGKG--------DDRGKLIMACGTGKTFTALKIAERTAAEN--------
gi|935514169|ref|WP_054429894.1|damageinducibleprotein[Mycobacteriumimmunogenum]_gi|929801120|gb|KPG29247.1|damageinduciblepr/1    QATAIEKVFDGFAKG--------NDRGKLIMACGTGKTFTALKIAERAASEN--------
gi|941317728|ref|WP_055118574.1|hypotheticalprotein[Mycobacteriumperegrinum]/11657    QATAIDKVFDGFAKG--------NDRGKLIMACGTGKTFTALKIAERAAAEN--------
gi|933683673|gb|ALG86377.1|hypotheticalproteinACH46_20120[Gordoniasp.QH11]/11636    QREAIDAVFDGFATG--------ADRGKLIMACGTGKTFTALKIAERTAADQ--------
gi|873553947|emb|CRY84110.1|Predictedhelicase[Nocardiafarcinica]/11688    QAQAIDAVFNGFAAG--------HDRGKLIMACGTGKTFTALKIAERTAVEN--------
gi|499525749|ref|WP_011212389.1|helicase[Nocardiafarcinica]_gi|54019339|dbj|BAD60707.1|hypotheticalprotein[NocardiafarcinicaIFM    QAQAIDAVFNGFAAG--------HDRGKLIMACGTGKTFTALKIAERTAVEN--------
gi|640109609|ref|WP_024794187.1|damageinducibleprotein[Tomitellabiformata]/11653    QQEAMDAVFGGFGAG--------NDRGKLIMACGTGKTFTALKIAERTAAEN--------
gi|640109991|ref|WP_024794334.1|hypotheticalprotein[Tomitellabiformata]/11639    QQEAMDAVFGGFNAG--------NDRGKLIMACGTGKTFTALKIAERTAAEN--------
gi|939125496|ref|WP_054831438.1|damageinducibleprotein[Rhodococcuserythropolis]/11669    QQAAIDDVFAGFEKG--------SDRGKLIMACGTGKTFTALKIAERAALEN--------
gi|910258550|ref|WP_050062711.1|hypotheticalprotein[Rhodococcussp.RD6.2]_gi|846871967|emb|CRK49225.1|conservedhypotheticalprote    QQDAIDAVFAGFEAG--------NDRGKLIMACGTGKTFTAMKIAERTAAEN--------
gi|797148189|gb|KJY52358.1|putativeHelicase[Bifidobacteriumasteroides]/11801    QRKAIDDCLRGFET---------HDRGKLIMACGTGKTLTALRLAEEMKSR---------
gi|535476570|gb|EFG27357.2|hypotheticalproteinHMPREF9020_00999[ScardoviainopinataF0304]/11827    QNEAIQDCIDGFKT---------DDRGKLIMACGTGKTLTALRLAEELREE---------
gi|737482729|ref|WP_035462496.1|hypotheticalprotein[Alicyclobacillusmacrosporangiidus]/11512    QERAIEAILSGWQE---------NDRGKCIMPPGTGKTLVALRIVERVACL---------
gi|685434354|gb|KGA95679.1|helicase[BacillusalcalophilusATCC27647=CGMCC1.3604]/11575    QIPAIDAVVKGFKS---------VDRGKLIMAPGTGKTYTSMAIAEKLADE---------
gi|945370253|gb|KQU27251.1|helicase[Bacillussp.Leaf13]/11557    QIPAIEAVVKGFET---------VDRGKLIMAPGTGKTYTSMAIAEELAKK---------
gi|880997015|ref|WP_048719116.1|helicase[Enterococcushirae]/11563    QIPAIKAVVEGFEV---------ANRGKLIMAPGTGKTYTSMVIAEELAKK---------
gi|945356929|gb|KQU14100.1|helicase[Bacillussp.Leaf49]/11547    QKPAIEAVVKGLEK---------EDRGKLIMAPGTGKTFTSMAIAEELAKK---------
gi|763482876|gb|KIZ53217.1|helicase[Bacillussafensis]/11563    QIPAIEKVVNGFET---------VDRGKLIMAPGTGKTYTSLVIAEEMAEK---------
gi|852223292|ref|WP_048309707.1|helicase[Anaerobacillusmacyae]_gi|850322910|gb|KMM38590.1|helicase[Anaerobacillusmacyae]/11573    QIPAIEAVVNGFDN---------VDRGKLIMAPGTGKTYTSMVIAEKMAEK---------
gi|739708665|ref|WP_037562619.1|helicase[Sporolactobacillusterrae]/11573    QIPAIDAVVNGFEK---------TDRGKLIMAPGTGKTYTSMVIAEKMAEK---------
gi|496687561|ref|WP_009329104.1|MULTISPECIES:helicase[Bacillus]_gi|317390867|gb|EFV71668.1|endonucleaseandmethylaseLlaGI[Bacill    QIPAIEAVVNGFET---------VDRGKLIMAPGTGKTYTSMVIAERMAEK---------
gi|930213599|gb|KPH74225.1|helicase[Oceanobacilluscaeni]/11569    QTPAIEKVLDGFKT---------ADRGKLIMAPGTGKTYTSMVIAERMAEK---------
gi|516366003|ref|WP_017756036.1|helicase[Bacillussp.ZYK]/11571    QIPAIEAVVNGFET---------ADRGKLIMAPGTGKTYTSMVIAEKMAEK---------
gi|488909977|ref|WP_002821052.1|helicase[Oenococcusoeni]_gi|399965230|gb|EJN99855.1|endonucleaseandmethylaseLlaGI[Oenococcusoen    QQEAIDESLAYFKD---------HDRGQLIMAPGTGKTFTSLKITESLMSQ---------
gi|495019967|ref|WP_007745780.1|helicase[Oenococcuskitaharae]_gi|366983682|gb|EHN59081.1|typeIIIrestrictionenzyme,ressubunit[Oe    QQEAIDKSLAYFKN---------HDRGQLIMAPGTGKTFTSLKIAESLMSQ---------
gi|489666119|ref|WP_003570406.1|typeIIIrestrictionendonuclease[Lactobacilluscasei]_gi|410527834|gb|EKQ02696.1|typeIIIrestrictio    QKIAIEKTVAYFDT---------HERGKLIMAPGTGKTFTSLKIAEALAKH---------
gi|511447623|gb|EPC78110.1|superfamilyIIDNA/RNAhelicase[Lactobacillusparacaseisubsp.paracaseiLpp221}/11615    QKEAIDKTVAYFET---------HERGKLIMAPGTGKTFTSLKIAEAMAKH---------
gi|497700411|ref|WP_010014595.1|helicase[Lactobacilluscoryniformis]/11585    QKEAITKTVAYFAN---------HERGKLIMAPGTGKTFTSLKIAEAMAAH---------
gi|489784048|ref|WP_003687939.1|helicase[Lactobacillusmali]_gi|394428539|gb|EJF01080.1|superfamilyIIDNA/RNAhelicase[Lactobacill    QKDAIAKAVAYFDA---------HERGKLIMAPGTGKTFTSLKITEAMAQH---------
gi|446780825|ref|WP_000858081.1|helicase[Staphylococcusaureus]_gi|374396604|gb|EHQ67835.1|typeIIIrestrictionenzyme,ressubunit[S    QEEAIRLAKKYYKN---------NDRGMLVMAPGTGKTFTSLKIAESFAKD---------
gi|510796658|ref|WP_016171456.1|hypotheticalprotein[Enterococcusdispar]_gi|508167532|gb|EOT44083.1|hypotheticalproteinOMK_00227    QKQAIEKASEYYKT---------NDRGMLIMAPGTGKTFTSLKMVEQFAKD---------
gi|612613473|gb|EZS30411.1|hypotheticalproteinW603_02482[StaphylococcusaureusVET0353R]_gi|612881917|gb|EZU93992.1|hypotheticalp    QKQAIEKAKEYFTT---------HDRGMLVMAPGTGKTFTSLKMVEQFAKD---------
gi|488332893|ref|WP_002402278.1|helicase[Enterococcusfaecalis]_gi|306513864|gb|EFM82466.1|helicaseCterminaldomainprotein[Enter/    QNDAIDLTIEYFKE---------NDRGQLIMAPGTGKTFTSLKLVEKMAKQ---------
gi|921166029|ref|WP_053111806.1|helicase[Enterococcusdurans]_gi|915771660|gb|AKX85928.1|helicase[Enterococcusdurans]/11562    QNEAIDLTINYFQE---------HDRGQLIMAPGTGKTFTSLKLVEKMAKQ---------
gi|498525358|ref|WP_010825030.1|hypotheticalprotein[Enterococcusfaecalis]_gi|487059631|gb|EOJ86727.1|hypotheticalproteinWOC_005    QNEAIDLTIEYFQE---------HDRGQLIMAPGTGKTFTSLKLVEKMAKQ---------
gi|498412624|ref|WP_010722070.1|hypotheticalprotein[Enterococcusfaecium]_gi|486634654|gb|EOF75253.1|hypotheticalproteinSGC_0253    QNDAINLTIEYFKE---------NDRGQLIMAPGTGKTFTSLKLVEKMAKQ---------
gi|498408534|ref|WP_010718364.1|hypotheticalprotein[Enterococcusfaecium]_gi|486609608|gb|EOF50873.1|hypotheticalproteinSCW_0257    QNDAINLTMDYFKE---------NDRGQLIMAPGTGKTFTSLKLVEKMAKQ---------
gi|498407475|ref|WP_010717483.1|hypotheticalprotein[Enterococcusfaecalis]_gi|486594855|gb|EOF36348.1|hypotheticalproteinSCK_005    QNEAIDLTIEYFQE---------HDRGQLIMAPGTGKTFTSLKLVEKMAKQ---------
gi|514885392|ref|WP_016615344.1|helicase[Enterococcusfaecalis]_gi|514505843|gb|EPH71676.1|helicaseprotein[Enterococcusfaecalis0    QNDAIDLTIEYFKE---------NDRGQLIMAPGTGKTFTSLKLVEKMAKQ---------
gi|325157603|gb|EGC69759.1|helicaseCterminaldomainprotein[EnterococcuscasseliflavusATCC12755]/11561    QNEAIDLTIDYFKE---------NDRGQLIMAPGTGKTFTSLKLVEKMAKQ---------
gi|895856206|ref|WP_048967668.1|helicase[Enterococcusfaecalis]/11551    QNEAINLTMDYFKE---------NDRGQLIMAPGTGKTFTSLKLVEKMAKQ---------
gi|639458836|ref|WP_024636366.1|helicase[Enterococcusfaecium]_gi|658176502|gb|KEI51159.1|helicase[EnterococcusfaeciumUC7256]/1/    QNEAIDLTIDYFKE---------NDRGQLIMAPGTGKTFTSLKLVEKMAKQ---------
gi|488266511|ref|WP_002337719.1|hypotheticalprotein[Enterococcusfaecium]_gi|430581409|gb|ELB19854.1|hypotheticalproteinOIQ_0351    QNEAIDLTIEYFKE---------NDRGQLIMAPGTGKTFTSLKLVEKMAKQ---------
gi|446353356|ref|WP_000431211.1|helicase[Staphylococcusaureus]_gi|253726329|gb|EES95058.1|helicaseCterminaldomainprotein[Staph/    QLDALDSAISYFKT---------KDRGQLIMAPGTGKTFTSLKIAEALSKQ---------
gi|672712068|emb|CDM14336.1|DNAorRNAhelicaseofsuperfamilyII[StaphylococcusepidermidisPM221]/11464    QKTALENAINHFEK---------YDRGQLIMAPGTGKTFTSLKISEALSKK---------
gi|896298990|ref|WP_049279905.1|helicase[Staphylococcusaureus]/11571    QKTALENAINHFEK---------NERGQLIMAPGTGKTFTSLKISEALSKK---------
gi|541903432|gb|ERH36190.1|hypotheticalproteinSEQU_00085[StaphylococcusequorumUMCCNS924]/11578    QKTALENAINHFKK---------YDRGQLIMAPGTGKTFTSLKISEALSKK---------
gi|488289747|ref|WP_002360955.1|helicase[Enterococcusfaecalis]_gi|256682216|gb|EEU21911.1|endonucleaseandmethylaseLlaGI[Enteroc    QETALEYALEHFTT---------KDRGQMIMAPGTGKTFTSLKIAEAFSKK---------
gi|494392045|ref|WP_007207894.1|helicase[Enterococcusitalicus]_gi|315483798|gb|EFU74284.1|helicaseCterminaldomainprotein[Enter/    QEIALEYALKHFLT---------KDRGQLIMAPGTGKTFTSLKIAEAFVKK---------
gi|446350692|ref|WP_000428547.1|helicase[Bacilluscereus]_gi|228727580|gb|EEL78721.1|RestrictionmodificationsystemLlaBIII[Bacil/    QEAALGYAMEHFNK---------YDRGQLIMAPGTGKTFTSLRISEALAKK---------
gi|488255302|ref|WP_002326510.1|hypotheticalprotein[Enterococcusfaecium]_gi|430480276|gb|ELA57459.1|hypotheticalproteinOGE_0473    QELALEHALEHYEE---------NDRGQLIMAPGTGKTFTSLKIAEAFSKK---------
gi|843253770|ref|WP_047916698.1|helicase[Lactococcuspiscium]_gi|802124677|emb|CEN29502.1|EndonucleaseandmethylaseLlaGI,TypeISPr    QQTAKENALAHFKE---------NDRGQLIMAPGTGKTFTSLKISEALSKD---------
LlaBIII/11578    QQTAKENALAHFKE---------NDRGQLIMAPGTGKTFTSLKISEALSKD---------
gi|499320531|ref|WP_011011023.1|helicase[Listeriainnocua]_gi|16415805|emb|CAC42019.1|pli0021[ListeriainnocuaClip11262]/11569    QQTAKDNALSHFKE---------NERGQLIMAPGTGKTFTSLKISEALAKD---------
gi|760026859|ref|WP_043709973.1|helicase[Weissellacibaria]_gi|758337165|gb|KIU20061.1|UvsWhelicase[Weissellacibaria]/11567    QQTAKENALNHFKE---------HERGQLIMAPGTGKTFTSLKIAEALAAE---------
LlaGI/11570    QQTAKENALAHFKE---------NDRGQLIMAPGTGKTFTSLKISEALSKD---------
gi|499988414|ref|WP_011669132.1|helicase[Lactococcuslactis]_gi|116109019|gb|ABJ74139.1|DNAorRNAhelicaseofsuperfamilyII[Lactococ    QQTAKDNALSHFKE---------NERGQLIMAPGTGKTFTSLKISEALAKD---------
```

```
gi|685938360|ref|WP_031670319.1|helicase[Listeriamonocytogenes]_gi|590403387|gb|EXL23261.1|hypotheticalproteinX846_1956[Listeri    QQTAKENALAHFKE---------NERGQLIMAPGTGKTFTSLKISEALSKD---------
gi|530793219|gb|EQC56240.1|helicase[Lactococcuslactissubsp.cremorisTIFN5]_gi|530793247|gb|EQC56263.1|helicase[Lactococcuslactis    QQTAKENALAHFKE---------NDRGQLIMAPGTGKTFTSLKISEALSKD---------
gi|695270006|ref|WP_032492075.1|hypotheticalprotein[Listeriamonocytogenes]_gi|695212872|ref|YP_009081522.1|hypotheticalproteinp    QQTAKENALAHFKE---------NERGQLIMAPGTGKTFTSLKISEALSKD---------
gi|504101310|ref|WP_014335297.1|helicase[Leuconostocmesenteroides]_gi|859247905|gb|AKO63885.1|helicase(plasmid)[Leuconostocmese    QQTAKENALNHFKE---------RDRGQLIMAPGTGKTFTSLKIAEALAKA---------
gi|488905190|ref|WP_002816265.1|helicase[Leuconostocmesenteroides]_gi|227351522|gb|EEJ41777.1|helicaseCterminaldomainprotein[L/    QKTAKENALNHFHE---------HDRGQLIMAPGTGKTFTSLKIAEALAKE---------
gi|518121285|ref|WP_019291493.1|MULTISPECIES:hypotheticalprotein[Lactococcus]/11588    QEKAISNSLEYFKK---------YDRGQLIMAPGTGKTFTSLKIAEAMANE---------
gi|754857213|ref|WP_042217902.1|helicase[Lactococcusgarvieae]/11549    QKEVIAKSLEYFKE---------NDRGQLIMAPGTGKTFTSLKVAEALAKA---------
gi|640048455|ref|WP_024784762.1|helicase[Streptococcusmutans]/11564    QKDVIKNALEHFKE---------HDRGQLIMAPGTGKTFTSLKVTEALAKK---------
gi|512695361|ref|WP_016476323.1|hypotheticalprotein[Veillonellasp.HPA0037]_gi|512079853|gb|EPD79018.1|hypotheticalproteinHMPREF    QEHVIKAAITHFEE---------NSRGQLIMAPGTGKTFTSLKIAESLAKL---------
gi|636963514|ref|WP_024403747.1|helicase[Streptococcussuis]/11574    QNNVITSALEYFKE---------HDRGQLIMAPGTGKTFTSLKVAEAMAKE---------
gi|881115312|ref|WP_048802949.1|helicase[Staphylococcuspasteuri]/11573    QEQAIQAAVTHFEK---------YDRGQLIMAPGTGKTFTSLKLTERMAEI---------
gi|446063996|ref|WP_000141851.1|helicase[Streptococcusmitis]_gi|307618725|gb|EFN97864.1|endonucleaseandmethylaseLlaGI[Streptoco    QESAIQSALNHFTE---------NDRGQLIMAPGTGKTFTSLKIAEAMARD---------
gi|488206609|ref|WP_002277817.1|restrictionmodificationsystemLlaBIII[Streptococcusmutans]_gi|449173033|gb|EMB75626.1|restricti/    QKEAISSALEYFKN---------HDRGQLIMAPGTGKTFTSLKVAESMAQE---------
gi|640023290|ref|WP_024781621.1|helicase[Streptococcusmutans]/11566    QKEAISSALEYFKN---------HDRGQLIMAPGTGKTFTSLKVAESMAQE---------
gi|814946581|ref|WP_046393781.1|helicase[Streptococcusuberis]_gi|810600885|gb|KKF57499.1|helicase[StreptococcusuberisB362]/1156    QKEVISSALNYYQS---------NDRGQLIMAPGTGKTFTSLKVAEAMAKE---------
gi|765470762|ref|WP_044720336.1|helicase[Streptococcussuis]/11559    QREVIKAALEYFKN---------HDRGQLIMAPGTGKTFTSLKIAEAMASE---------
gi|654507127|ref|WP_027976443.1|helicase[Streptococcusdevriesei]/11558    QRDVIKSALEYFKG---------NDRGQLIMAPGTGKTFTSLKVAEAMAKE---------
gi|530812902|gb|EQC72481.1|DNAorRNAhelicaseofsuperfamilyII[Streptococcussp.HSISS2]/11570    QREVIKSALEHFKE---------NDRGQLIMAPGTGKTFTSLKVAEAMAKE---------
gi|489158846|ref|WP_003068504.1|helicase[Streptococcusintermedius]_gi|355367114|gb|EHG14827.1|hypotheticalproteinHMPREF9682_002    QRDVIKSALAHFKD---------HDRGQLIMAPGTGKTFTSLKVAEAMAKE---------
RM.Lru27782ORF13200P/11620    QKEAIEKSVEYFRD---------NERGKLIMAPGTGKTFTSLKIAEALYRE---------
gi|492676546|ref|WP_005919236.1|DNAphosphorothioationsystemrestrictionenzyme[Pediococcusacidilactici]_gi|482517924|gb|EOA08103.    QQEAIDKSLSYFKD---------HDRGKLIMAPGTGKTFTSLKIAEALMKD---------
gi|498183116|ref|WP_010497272.1|helicase[Lactobacillusacidipiscis]/11563    QIEAIEKSLAYFKE---------HDRGKLIMAPGTGKTFTSLKIAEALMED---------
gi|822100485|ref|WP_046921865.1|helicase[Lactobacillusruminis]_gi|821684442|gb|KLA43321.1|restrictionmodificationsystemLlaBIII/    QKEAIKKSLDYFKT---------NSRGKLVMAPGTGKTFTSLKIAEALFNE---------
gi|935565832|ref|WP_054450311.1|helicase[Lactobacilluskunkeei]/11573    QEEAIKNAVEYFKS-N----EDYHDRGKLIMAPGTGKTFTSLKITEALMKD---------
gi|752296887|ref|WP_041152901.1|helicase[Lactobacilluskunkeei]_gi|751663520|gb|KIM18358.1|helicase[Lactobacilluskunkeei]/11577    QEEAIKNAVEYFKS-N----EEYHDRGKLIMAPGTGKTFTSLKITEALMKD---------
gi|495745291|ref|WP_008469870.1|helicase[Lactobacillushominis]_gi|394484442|emb|CCI81207.1|RestrictionmodificationsystemLlaBII/    QEKAINNAVNYFED---------RDRGKLIMAPGTGKTFTSLKIAEAEMRK---------
gi|918672414|ref|WP_052543134.1|helicase[Lactobacillussp.]/11564    QETAINNSLKYFKE---------YDRGKLIMAPGTGKTFTSLKIAEALMRD---------
gi|799141631|ref|WP_045927261.1|helicase[Lactobacilluskullabergensis]_gi|797155335|gb|KJY59264.1|putativerestrictionendonucleas    QNEAIARAVKYFKT---------NSRGKLIMAPGTGKTFTSLKIAEALMND---------
gi|504379990|ref|WP_014567092.1|helicase[Lactobacillusjohnsonii]_gi|329666726|gb|AEB92674.1|putativerestrictionendonuclease[Lac    QNIAINKSLEYFKE---------HNRGKLIMAPGTGKTFTSLKIAEALMKK---------
gi|489880179|ref|WP_003783646.1|DNAdamageinducibleprotein[Actinomycesnaeslundii]_gi|399901561|gb|EJN84440.1|typeIIrestriction/    QKRALDAVSAGLST-A--------DRGKLIMACGTGKTFTSLRIAEAMAGP---------
gi|496524497|ref|WP_009232780.1|DNAdamageinducibleprotein[Actinomycessp.oraltaxon849]_gi|365265413|gb|EHM95177.1|hypotheticalp/    QKRALDAVSAGLST-A--------DRGKLIMACGTGKTFTSLRIAEAMAGP---------
gi|915397782|ref|WP_050783167.1|damageinducibleprotein[Actinomycesoris]/11694    QKRALDAVSAGLST-A--------DRGKLIMACGTGKTFTSLRIAEAMVGP---------
gi|502925248|ref|WP_013160224.1|helicase[Propionibacteriumfreudenreichii]_gi|296921272|emb|CBL55824.1|helicase[Propionibacteriu    QKEAIAKIQEGFQT-R--------DRGQWISACGTGKTFTSLKLAEERCKN---------
gi|732164352|emb|CEG87481.1|helicase[Propionibacteriumfreudenreichii]/11618    QKLAIGKILEGFRA-H--------DRGKWISACGTGKTFTSLRLAEQMCAQ---------
gi|739047402|ref|WP_036925058.1|damageinducibleprotein[Propionibacteriumthoenii]/11596    QKEAIDAIEAGFAS-H--------DRGQWISACGTGKTFTSLKLAERRCAE---------
gi|739112839|ref|WP_036982533.1|damageinducibleprotein[Propionibacteriumjensenii]/11594    QREAIDAIQAGFES-H--------DRGQWISACGTGKTFTSLKLAERRCAE---------
gi|918023558|ref|WP_052314121.1|damageinducibleprotein[Nocardiathailandica]/11612    QKTAIDAVRRGLAE-Q--------DRGKLIMACGTGKTFTSLKIAEDLVGT---------
gi|498380271|ref|WP_010694427.1|DNAdamageinducibleprotein[Saccharopolysporaspinosa]/11605    QQKALDAVRDGFAE-R--------DRGKLVMACGTGKTYTSLKIAEDQVGA---------
gi|749991459|ref|WP_040339809.1|damageinducibleprotein[CandidatusBlastococcusmassiliensis]/11597    QQKALDDVRAGLAE-H--------DRGQLVMACGTGKTFTSLRIAEELVGA---------
gi|754177801|ref|WP_041796231.1|damageinducibleprotein[Modestobactermarinus]/11592    QQEALDDVRAGLAE-R--------DRGQLVMACGTGKTFTSLRIAEELVGA---------
gi|504188248|ref|WP_014375350.1|DNAdamageinducibleprotein[Blastococcussaxobsidens]_gi|378782790|emb|CCG02456.1|putativeHelicas/    QQKALADVRAGLAV-H--------DRGQLVMACGTGKTFTSLRIAEELAGA---------
gi|946476713|gb|KRF26798.1|damageinducibleprotein[Phycicoccussp.Soil803]/11609    QKIAKDAVISGLAT-Q--------DRGQLIMACGTGKTFTSLKIAEQLATD---------
gi|700176672|gb|KGN37509.1|DNAdamageinducibleprotein[KnoelliasubterraneaKCTC19937]/11633    QKQAKDAVINGLAT-H--------DRGQLIMACGTGKTFTSLKIAEQLATD---------
gi|700168841|gb|KGN29914.1|hypotheticalproteinN802_10215[KnoelliasinensisKCTC19936]/11631    QKVAKDAAITGLAT-H--------DRGQLIMACGTGKTFTSLKIAEQLATD---------
gi|946426241|gb|KRE76610.1|damageinducibleprotein[Arthrobactersp.Soil763]/11606    QVDAIAAATAKFGE-H--------DRGKLIMACGTGKTFTSLRLAEQMVGP---------
gi|517318525|ref|WP_018500927.1|hypotheticalprotein[Frankiasp.BCU110501]/11592    QQVAIEKIEAGFGE-H--------DRGKMIMACGTGKTFTSLKIAERNVGA---------
gi|499072429|ref|WP_010851327.1|Helicase[Tetrasphaeraelongata]_gi|478756579|emb|CCH71501.1|putativeHelicase[Tetrasphaeraelongat    QRKAIEDVCRGLAS-A--------DRGKLIMACGTGKTFTSLRLAEQLVGA---------
gi|750080666|ref|WP_040385350.1|damageinducibleprotein[Demetriaterragena]/11625    QTEAVEKVTAGFVA-H--------DRGKLIMACGTGKTFTSLRLAEQYAGP---------
gi|752558292|ref|WP_041229523.1|damageinducibleprotein[Dermacoccusnishinomiyaensis]_gi|664689315|gb|AIF41904.1|damageinducibl/1    QREAIDTVLGKFTD-H--------DRGKLVMACGTGKTFTSLRLAERQVGA---------
gi|829089213|ref|WP_047310701.1|damageinducibleprotein[Dermacoccussp.PE3]_gi|828384812|gb|KLO63770.1|damageinducibleprotein[D/1    QRTAIDKVIDGLKT-H--------DRGKLVMACGTGKTFTSLRLAEQQVGA---------
gi|748756635|ref|WP_040014750.1|damageinducibleprotein[Dermacoccussp.Ellin185]/11596    QRTAIDKVLDGLKT-H--------DRGKLIMACGTGKTFTSLRLAEEQVGA---------
gi|916534724|ref|WP_051141848.1|damageinducibleprotein[Actinomycesneuii]/11632    QARALADVFKGFET-H--------DRGTLVMACGTGKTFTSLKIAEHLVEL---------
gi|921140030|ref|WP_053090276.1|damageinducibleprotein[Rothiamucilaginosa]/11616    QDIAITRTLKGLQK-D--------DRGQLIMACGTGKTFTAIRLAEKFAEQ---------
gi|896406751|ref|WP_049343749.1|damageinducibleprotein[Rothiamucilaginosa]/11647    QDIAITRTLKGLQK-D--------DRGQLIMACGTGKTFTAIRLAEKFAEQ---------
gi|723608234|gb|AIY01610.1|putativehelicase[Arthrobactersp.PAMC25486]/11613    QHEAVEATLKGFTT-S--------DRGKLIMACGTGKTFTALRLAERFAED---------
gi|946394527|gb|KRE45160.1|damageinducibleprotein[Arthrobactersp.Soil736]/11616    QHDAVAATMKGFID-N--------NRGKLIMACGTGKTFTALKVAERFAEM---------
gi|515765304|ref|WP_017197904.1|hypotheticalprotein[Arthrobactersp.M2012083]/11648    QHEAVNAAMKGFET-H--------HRGKLIMACGTGKTFTALKIAERFAEQ---------
gi|945315587|gb|KQT73127.1|damageinducibleprotein[Microbacteriumsp.Leaf436]/11612    QSVAIDDVVAGFTD-R--------DRGKLIMACGTGKTFTSLRLAEKVVPL---------
gi|759908497|ref|WP_043594599.1|damageinducibleprotein[Frigoribacteriumsp.MEB024]_gi|758271359|gb|KIU03346.1|damageinduciblep/1    QVQALAAVQAGFAD-N--------DRGKLIMACGTGKTFTSLRIAEEIAGR---------
gi|945072850|gb|KQR34237.1|hypotheticalproteinASF75_00055[Curtobacteriumsp.Leaf154]/11597    QVEALNAVQAGLES-T--------DRGKLIMACGTGKTFTGLRIAEAVAGR---------
gi|551263152|ref|WP_022883363.1|DNAdamageinducibleprotein[Glaciibactersuperstes]/11621    QEQAVADVAKGFKD-S--------PRGKLIMACGTGKTFTSLRAAETIVGS---------
gi|945452341|gb|KQV08491.1|hypotheticalproteinASC63_10170[Leifsoniasp.Root112D2]/11623    QLEALRAVKQGFTE-H--------DRGKLIMACGTGKTFTSLRIAERTVAR---------
gi|742759483|gb|KIC69912.1|damageinducibleprotein[Kocuriarhizophila]/11610    QLQAMAAVKEGFKS-H--------DRGKLIMACGTGKTYTSLQIMQEQTPE---------
gi|737919995|ref|WP_035885304.1|damageinducibleprotein[Kocuriarhizophila]/11611    QLQAMAAVKEGFES-H--------DRGKLIMACGTGKTYTSLQIMQEQTPE---------
gi|737997701|ref|WP_035959325.1|damageinducibleprotein[Kocuriamarina]_gi|725823884|gb|KHE75603.1|damageinducibleprotein[Kocur/1    QLQAMAAVKEGFES-H--------DRGKLIMACGTGKTYTSLQIMQEQTPR---------
gi|664521070|ref|WP_031037650.1|damageinducibleprotein[Streptomycessp.NRRLWC3774]/11624    QRAAIDDVFTGFAD-H--------NRGKLIMACGTGKTFTGLKIVERLQKERAA------
gi|500101034|ref|WP_011777041.1|DNAdamageinducibleprotein[Arthrobacteraurescens]_gi|119951547|gb|ABM10457.1|putativeHelicase[A/    QRAAIEKVREGFQA-H--------DRGKLIMACGTGKTYTSLRIVEDMVPV---------
```

```
gi|916871953|ref|WP_051479009.1|damageinducibleprotein[Arthrobactersp.H5]/11588                                     QRTAIEKVREGFQT-M--------DRGKLIMACGTGKTYTSLRIVEDLVPV---------
gi|612084649|gb|EZP43369.1|TypeIIIrestrictionenzyme,ressubunit[Micrococcusluteus]/11625                             QREAIDDVVAGFET-K--------DRGRLIMACGTGKTFTSLKLVEQLVPV---------
gi|759739708|ref|WP_043450422.1|damageinducibleprotein[Arthrobacterphenanthrenivorans]_gi|742758264|gb|KIC68716.1|damageinduc/1  QREAIEDVKKGFTL-S--------DRGKLIMACGTGKTYTSLKMVEEIVPI---------
gi|636845448|ref|WP_024366823.1|damageinducibleprotein[Arthrobactersp.TB26]/11605                                   QREAITDVITGFHG-S--------DRGKLIMACGTGKTYTSLKIVEEMVPV---------
gi|500009025|ref|WP_011689743.1|DNAdamageinducibleprotein[Arthrobactersp.FB24]_gi|116613051|gb|ABK05760.1|typeIIIrestrictionen/  QRDAIDDVITGFQT-S--------DRGKLIMACGTGKTYTSLKIVEEMVPV---------
gi|821249847|gb|KKZ12083.1|hypotheticalproteinTQ37_06090[CandidatusSynechococcusspongiarum15L]/11436              QQEAINSALAHLEAPG--------TRGTLLMACGTGKTLVGLRVAEAVAG----------
gi|739446902|ref|WP_037306885.1|damageinducibleprotein[Ruegeriahalocynthiae]/11592                                  QQQALDAVKSGFEE-H--------DRGKLIMACGTGKTFTALKIAEALAG----------
gi|640620081|ref|WP_025048131.1|damageinducibleprotein[Sulfitobactermediterraneus]_gi|751956650|gb|KIN77213.1|TypeIIIrestricti/  QKLALEHVGKGFEQ-G--------DRGKLIMACGTGKTFTALRIAEAVAG----------
gi|740247634|ref|WP_038088583.1|damageinducibleprotein[Thiobacillusprosperus]_gi|684049748|gb|KFZ90853.1|putativehelicase[Thio/  QEEAIRETVDGFAG-L--------DRGKLIMACGTGKTFTALRLNETVTP----------
gi|518420321|ref|WP_019590528.1|hypotheticalprotein[Thioalkalivibriosp.ALE20]/11603                                 QKEALEDCLRGFGQ-H--------ERGKLIMACGTGKTFTSLRLAEEMVP----------
gi|500156826|ref|WP_011831496.1|DNAdamageinducibleprotein[Methylibiumpetroleiphilum]_gi|124261892|gb|ABM96881.1|adeninespecifi/  QDEAIDSSLAGFKS-V--------DRGKLIMACGTGKTFTALRLAERQTP----------
gi|769978537|ref|WP_045094522.1|damageinducibleprotein[Legionellafallonii]_gi|698841736|emb|CEG55682.1|Putativeproteinwithboxh/  QDEAISKALYALKD-Y--------DRGKLVMACGTGKTFTSLRLMEQMLP----------
gi|553752541|ref|WP_023085344.1|hypotheticalprotein[Pseudomonasaeruginosa]_gi|552201952|gb|ERU62653.1|hypotheticalproteinQ088_0  QATAIESVLSGFES-V--------DRGKLIMACGTGKTFTSLRLMEQATP----------
gi|880980418|ref|WP_048704461.1|damageinducibleprotein[Thauerahumireducens]_gi|875132785|gb|KMU70944.1|damageinducibleprotein/1  QEEAVGNILEGFKE-V--------DRGKLIMACGTGKTFTSLRLMEQATP----------
gi|740135732|ref|WP_037982815.1|damageinducibleprotein[Thauerasp.27]/11603                                          QKEAVAKIIEGFQE-L--------DRGKLIMACGTGKTFTSLRLMEQVTP----------
gi|738017371|ref|WP_035978332.1|damageinducibleprotein[Burkholderiaglumae]/11380                                    QRDAIAKVVEGFQS-A--------DRGKLIMACGTGKTFTSLRLVEQITP----------
gi|498506003|ref|WP_010806671.1|typeIIIrestrictionenzyme,ressubunit[Pandoraeasp.SD62]_gi|490314561|gb|EON12879.1|typeIIIrestri/  QRDAIAKVVEGFQS-A--------DRGKLIMACGTGKTFTSLRLVEQITP----------
gi|500117748|ref|WP_011793153.1|DNAdamageinducibleprotein[Acidovoraxcitrulli]_gi|120587742|gb|ABM31182.1|typeIIIrestrictionenz/  QQEAIAAVAAGFTE-A--------DRGKLIMACGTGKTFTALRMMENEVA----------
gi|651617328|ref|WP_026611444.1|damageinducibleprotein[Methylocaldumszegediense]/11601                               QKEAIEAVLQGFQS-A--------DRGKLIMACGTGKTFTSLRLMETLVP----------
gi|737210208|ref|WP_035194994.1|damageinducibleprotein[Acidithiobacillusferrivorans]_gi|669953220|emb|CDQ11755.1|Adeninespecif/  QESAREDVLIGFQD-H--------DRGKLIMACGTGKTYTSLSIMEYMVQ----------
gi|935697386|ref|WP_054506180.1|damageinducibleprotein[Achromobactersp.]_gi|928716153|emb|CUK12836.1|Hefnuclease[Achromobacter/  QEEALTDVIKGFAS-N--------DRGKLIMACGTGKTYTSLAITEKMIK----------
gi|429502517|gb|ELA00827.1|adeninespecificDNAmethyltransferase[Cupriavidussp.HMR1]/11599                             QNEALADVVKGFAS-N--------DRGKLIMACGTGKTYTSLAIAEKMIK----------
gi|506356642|ref|WP_015876361.1|DNAdamageinducibleprotein[Burkholderiaglumae]_gi|237877158|gb|ACR29491.1|AdeninespecificDNAmet/  QKEALADVMKGFKT-S--------ERGKLIMACGTGKTYTSLAVTEELIT----------
gi|696406787|ref|WP_032978003.1|damageinducibleprotein[Bordetellahinzii]_gi|625335076|gb|KCB49343.1|typeIIIrestrictionenzyme,r/  QSEALANVTKGFQA-H--------DRGKLIMACGTGKTYTSLIIAEQMVK----------
gi|817529125|gb|KKO72587.1|damageinducibleprotein[Kerstersiagyiorum]/11592                                         QSEALANVTKGFQA-H--------DRGKLIMACGTGKTYTSLIIAEQMVK----------
gi|737048910|ref|WP_035044118.1|damageinducibleprotein[Aquabacteriumsp.NJ1]_gi|699052896|gb|KGM38706.1|damageinducibleprotein/1  QREALANVLKGFKS-S--------DRGKLIMACGTGKTYTSLIISEEMVR----------
gi|822492519|ref|WP_046933319.1|MULTISPECIES:damageinducibleprotein[Xanthomonas]_gi|821703525|gb|KLA61196.1|damageinduciblepr/1  QREALVNVTKGFKT-S--------NRGKLIMACGTGKTYTSLIVAEEMVK----------
gi|751252861|ref|WP_040963740.1|damageinducibleprotein[Xanthomonasfuscans]_gi|749808363|gb|KIJ03887.1|damageinducibleprotein[/1  QSEALVDVLKGFKT-S--------DRGKLIMACGTGKTYTSLIVAQEMVK----------
gi|496774980|ref|WP_009368792.1|DNAdamageinducibleprotein[Bilophilasp.4_1_30]_gi|345039902|gb|EGW44205.1|hypotheticalproteinHM/  QEEALEAVLKGFEA-H--------DRGKLIMACGTGKTYTSLVIAQKMVK----------
gi|499241912|ref|WP_010939452.1|DNAdamageinducibleprotein[Desulfovibriovulgaris]_gi|46580580|ref|YP_011388.1|adeninespecificDN/  QNEALEAVRKGFSA-H--------DRGKLIMACGTGKTYASLLIAEDMVA----------
gi|504274913|ref|WP_014462015.1|DNAdamageinducibleprotein[Bacillusmegaterium]_gi|345446919|gb|AEN91936.1|AdeninespecificDNAmet/  QCEALENVMRGFEK-A--------DRGQMVMACGTGKTFTSLKIAEEFVKG---------
gi|504895966|ref|WP_015083068.1|typeIIrestrictionenzymeandmethyltransferasesubtypeG[Anabaenasp.90]_gi|413972424|gb|AFW96512.1|t  QQTALEKVLAGFKT-G--------DRGKLIMACGTGKTFTALKIAENFPR----------
gi|518327765|ref|WP_019497972.1|hypotheticalprotein[Calothrixsp.PCC7103]/11600                                      QTTALQKVMDGFKG-S--------ERGKLIMACGTGKTFTALKIAEQFAQD---------
gi|652765503|ref|WP_027079917.1|damageinducibleprotein[Luteimonasmephitis]/11593                                    QAEALKKVSAGFEE-H--------DRGKLIMACGTGKTFTSLKIVEHLVP----------
gi|896539951|ref|WP_049442123.1|damageinducibleprotein[Stenotrophomonasmaltophilia]/11591                           QQAALKAVHEGFQK-H--------ERGKLIMACGTGKTFTSLKIVEQQTP----------
gi|500217900|ref|WP_011888043.1|DNAdamageinducibleprotein[Geobacillusthermodenitrificans]_gi|134267954|gb|ABO68149.1|Conserved/  QKEAIADVLEGFKQ-H--------DRGKLIMACGTGKTFTALKLTEKMVD----------
gi|696570298|ref|WP_033102014.1|damageinducibleprotein[Thermoactinomycesdaqus]/11594                                 QEAAIANVVAGFQT-Y--------DRGKLIMACGTGKTFTSLKLTERLVP----------
gi|251772067|gb|EES52637.1|putativehelicase[Leptospirillumferrodiazotrophum]/11618                                  QKIAVEKVIEGFKV-H--------DRGKLIMACGTGKTFTSLRIAESLVP----------
gi|738124101|ref|WP_036082108.1|damageinducibleprotein[Leptospirillumferriphilum]_gi|685243306|gb|KGA94177.1|putativehelicase[/  QNAAVNNVIEGFQE-H--------DRGKLIMACGTGKTFTSLKIAESVVP----------
gi|558699223|ref|WP_023524766.1|helicase[Leptospirillumsp.GroupII'CF1']_gi|206603764|gb|EDZ40244.1|Putativehelicase[Leptospiri/  QKVAVEKVIEGFTN-H--------DRGKLIMACGTGKTFTSLKIAETLVP----------
gi|406774462|gb|AFS53887.1|putativehelicase[LeptospirillumferriphilumML04]/11610                                   QQEAVSKVLSGFQT-S--------DRGKLVMACGTGKTFTSLKIAEAIVP----------
gi|740719463|ref|WP_038504749.1|damageinducibleprotein[Leptospirillumferriphilum]_gi|640838125|gb|AIA30173.1|damageinduciblep/1  QQEAVSKVLAGFQS-S--------DRGKLVMACGTGKTFTSLKIAESLVS----------
gi|517810766|ref|WP_018980974.1|hypotheticalprotein[Saccharibacterfloricola]/11733                                  QVSAVAACHEAFCE-Q------SKERAQLHMACGTGKTLTSLAIAEEVAG----------
gi|502471675|ref|WP_012798533.1|helicase[Slackiaheliotrinireducens]_gi|256791761|gb|ACV22431.1|predictedhelicase[Slackiaheliotr  QERAIEDCIRGFSI-S--------DRGKLIMACGTGKTLTSLRLTEKLVG----------
gi|657871713|ref|WP_029576297.1|hypotheticalprotein[Bifidobacteriumthermacidophilum]_gi|673007521|gb|KFJ02535.1|helicase[Bifido  QREAIDSIKKTFET-H--------DRCSLVMACGTGKTLTSLRLCEEMLG----------
gi|673004757|gb|KFI99791.1|DNAhelicaserestrictionenzymetypeIIIRessubunit[Bifidobacteriumthermacidophilumsubsp.porcinum]/11682  QREAIDSIKKTFET-H--------DRCSLVMACGTGKTLTSLRLCEEMLG----------
gi|917316092|ref|WP_051922804.1|hypotheticalprotein[Bifidobacteriumstellenboschense]_gi|673003774|gb|KFI98812.1|helicase[Bifido  QREAIDAVKTELTE-H--------DRCSLVMACGTGKTLTSLRLVEEQVG----------
gi|705415694|ref|WP_033502332.1|hypotheticalprotein[Bifidobacteriumpseudolongum]_gi|672982411|gb|KFI77685.1|DNAdamageinducible/  QVEAIDAVKRELAD-H--------DRCSLVMACGTGKTLTALRLCEQYPG----------
gi|916591418|ref|WP_051198509.1|hypotheticalprotein[Bifidobacteriumsp.AGR2158]/11704                                QVEAIEAVRKELDE-H--------DRCTLVMACGTGKTLTALRYAEQVPG----------
gi|772242982|gb|AJW78255.1|hypotheticalproteinVO01_03165[Clavibactermichiganensissubsp.insidiosus]/11642             QRRAISAVIEGFRG-T--SKRPGVDRGKMIMACGTGKTFTALKLAERWTRE---------
gi|916649818|ref|WP_051256909.1|damageinducibleprotein[Actinomycesgraevenitzii]/11677                                QVRARSAVMAGFDT---------SDRGQLIMACGTGKTFTSLIIAREFMER---------
gi|496504578|ref|WP_009212861.1|helicase[Actinomycessp.oraltaxon180]_gi|315312695|gb|EFU60776.1|helicase[Actinomycessp.oraltaxo  QVRARSAVMSGFEE---------HDRGTMVMACGTGKTFTALTIAREFVEK---------
gi|494862206|ref|WP_007588306.1|helicase[Actinomycessp.ICM39]_gi|398362799|gb|EJN46470.1|typeIIIrestrictionenzyme,ressubunit[Ac  QVRARSAVMSGFEE---------HDRGTMVMACGTGKTFTALTIAREFVEK---------
gi|881057111|ref|WP_048774974.1|damageinducibleprotein[Sanguibacterkeddieii]/11697                                  QVRARSAVMAGFEE---------HDRGTMVMACGTGKTFTALTIAREFVEK---------
gi|881020459|ref|WP_048740808.1|damageinducibleprotein[Kytococcussedentarius]/11704                                 QVRARSAVMAGFEE---------HDRGTMVMACGTGKTFTALTIARGFVEK---------
gi|881052313|ref|WP_048770555.1|damageinducibleprotein[Cellulomonasflavigena]/11705                                 QVRARSAVMAGFEE---------HDRGTMVMACGTGKTFTALTIAREFVEK---------
gi|490108682|ref|WP_004009424.1|helicase[Mobiluncuscurtisii]_gi|315490465|gb|EFU80089.1|helicaseCterminaldomainprotein[Mobilun/  QVDAINDVFEGFES---------HNRGILVMACGTGKTFTSLKIAEQLTNE---------
gi|490111935|ref|WP_004012603.1|helicase[Mobiluncusmulieris]_gi|227843812|gb|EEJ53982.1|helicaseCterminaldomainprotein[Mobilun/  QTKAINDVMTGFET---------HDRGTLVMACGTGKTFTSLQIAQKFAER---------
gi|490114228|ref|WP_004014848.1|helicase[Mobiluncusmulieris]_gi|269934432|gb|EEZ90993.1|helicaseCterminaldomainprotein[Mobilun/  QTKAINDVMTGFET---------HDRGTLVMACGTGKTFTSLQIAQKFAER---------
gi|493333551|ref|WP_006290636.1|helicase[Parascardoviadenticolens]_gi|315119487|gb|EFT82620.1|helicaseCterminaldomainprotein[P/  QIKAINQINAAFEK-H--------DRCKAIMACGTGKTLMSLRLAEERAK----------
gi|495817067|ref|WP_008541646.1|helicase,partial[Sutterellaparvirubra]_gi|378598583|gb|EHY31712.1|helicaseprotein,partial[Sutte  QEYAIKDVLTGFQT-S--------DRGKLIMACGTGKTFTSMQIAEAMEG----------
gi|491572696|ref|WP_005430278.1|helicase[Sutterellawadsworthensis]_gi|319805116|gb|EFW01943.1|DNAhelicaserestrictionenzymeTypeI  QEEAIQRVIEGFKT-N--------NRGKLIMACGTGKTFTSMKIAEKMEG----------
gi|748686321|ref|WP_039944445.1|hypotheticalprotein[Thermicanusaegyptius]/11573                                     QEQAIKAVTEGFVE-A--------DRGKLIMPPGSGKTLVALKIAEKQVG----------
gi|916875698|ref|WP_051482754.1|hypotheticalprotein[Thioalkalivibriosp.HK1]/11366                                   QSQAIADIRKGFAS-H--------DRGRLIMACGTGKTFTALRLAEEMAG----------
gi|821248604|gb|KKZ10960.1|hypotheticalproteinTE42_09075[CandidatusSynechococcusspongiarumSP3]/11598                 QRAAINSALAHLKA-R--------GARGKLLMACGTGKTLVGLRVAEGIAG---------
gi|737409843|ref|WP_035390956.1|hypotheticalprotein[Ferrimicrobiumacidiphilum]_gi|765544266|gb|KJE76241.1|typeIrestrictionenzym  QRAAIDDVKQGFRE-A--------DRGKLIMACGTGKTFTSLRLAEEVVG----------
gi|654906925|ref|WP_028358281.1|damageinducibleprotein[Brackiellaoedipodis]/11679                                   QKDAYKDVEAGFAE-Q--------DRGKLIMACGTGKTFTALRIAEGLTG----------
```

```
gi|502809901|ref|WP_013044877.1|hypotheticalprotein[CandidatusPuniceispirillummarinum]_gi|292663146|gb|ADE38247.1|hypotheticalp      QKEARDAVITGLKD-N--------DRGKLIMACGTGKTLTSLHIAEMMIE----------
gi|251771370|gb|EES51950.1|typeIIIrestrictionenzyme,ressubunit[Leptospirillumferrodiazotrophum]/11628      QENARTDVLEGLKT-H--------SRGKLIMACGTGKTLTGLRIVEEIVP----------
gi|498328480|ref|WP_010642636.1|DNAdamageinducibleprotein[Acidithiobacillusthiooxidans]/11627      QREALQATMKGFET-Q--------DRGKLIMACGTGKTFTSLNIMERIAP----------
gi|647633864|ref|WP_025906698.1|damageinducibleprotein[Prochlorococcussp.scB245a_519B7]/11622      QIEAVEEVRKGLLE-N--------DRGKLIMACGTGKTFTSLCIAEEIFG----------
gi|647619599|ref|WP_025893729.1|damageinducibleprotein[Prochlorococcussp.scB241_527N11]/11621      QIKALEAVREGLKK-D--------DRGKMIMACGTGKTFTSLRIAEELAG----------
gi|755034887|ref|WP_042387498.1|hypotheticalprotein[Clostridiumsp.CL6]/11606      QRKAIDDVIEGFKN-N--------DRGKLIMACGTGKTFTSLRIVEELLN----------
gi|498139798|ref|WP_010453954.1|helicase[SuccinivibrionaceaebacteriumWG1]/11662      QQNALNHVHKYFID-D------KNERGKLVMACGTGKTFTSLRIAEDLLG----------
gi|527501386|gb|EPT34571.1|typeIIIrestrictionenzyme,ressubunit[Bacteroidetesbacteriumoraltaxon272str.F0290]/11789      QIKAMECAQNHF---M------EHDRGKLIMACGTGKTYTSLKIVEQETN----------
gi|496047275|ref|WP_008771782.1|helicase[Bacteroidessp.2_1_33B]_gi|262296443|gb|EEY84373.1|helicaseCterminaldomainprotein[Bact/      QLEAMKAAHEYF---R------VHDRGKLIMACGTGKTYTSLEIIEQETG----------
PDI8503III/11664      QLEAMKAAHEYF---R------VHDRGKLIMACGTGKTYTSLEIIEQETG----------
gi|738719431|ref|WP_036613715.1|helicase[Parabacteroidesdistasonis]_gi|649542518|gb|KDS49387.1|restrictionendonucleasefamilypro      QLEAMKAAHEYF---R------VHDRGKLIMACGTGKTYTSLEIIEQETG----------
gi|659417033|gb|KEJ86133.1|hypotheticalproteinHMPREF1002_00341[Porphyromonassp.31_2]/11656      QLEAMKAAHEYF---R------VHDRGKLIMACGTGKTYTSLEIIEQETG----------
gi|567456807|gb|ETK11358.1|hypotheticalproteinT235_15845[Tannerellasp.oraltaxonBU063isolateCell8/11]/11433      QREALKAAHEHY---Q------NSDRGKLVMACGTGKTFTSLKMVEQELS----------
gi|545440279|ref|WP_021677877.1|helicase[Porphyromonasgingivalis]_gi|543985765|gb|ERJ89108.1|helicaseprotein[Porphyromonasgingi      QREALRTAHKHF---S------SHDRGKLVMACGTGKTFTSLKLIEQELG----------
gi|497438056|ref|WP_009752254.1|helicase[Prevotellasp.oraltaxon473]_gi|429144866|gb|EKX87968.1|helicaseprotein[Prevotellasp.ora      QREALEAAHLHF---Q------NHDRGKLVMACGTGKTFTSLKLIEQELQ----------
gi|490423474|ref|WP_004295683.1|MULTISPECIES:helicase[Bacteroides]_gi|156110098|gb|EDO11843.1|helicaseCterminaldomainprotein[B/      QLRAMSAAHAY--F-Q------EHDRGKLIMACGTGKTYTALKIAEDLLN----------
gi|941777229|ref|WP_055173506.1|helicase[Bacteroidescaccae]_gi|933263395|emb|CUQ00567.1|Predictedhelicase[Bacteroidescaccae]/1/      QLNAISKAYVHYMA-D------GNERGKPIMACGTGKTYTSLLIAEQLFD----------
gi|492413898|ref|WP_005836892.1|helicase[Bacteroidesuniformis]_gi|392678824|gb|EIY72229.1|hypotheticalproteinHMPREF1072_03583[B      QLNAITKAHIHYMA-D------GNDRGKLIMACGTGKTYTSLLIAEQLFG----------
gi|695455095|ref|WP_032566191.1|helicase[Bacteroidesfragilis]_gi|596095426|gb|EXZ59996.1|restrictionendonucleasefamilyprotein[B      QLNAISKAYVHYMS-D------NNDRGKLIMACGTGKTYTSLLIAEQLFG----------
gi|933227941|emb|CUQ02348.1|Predictedhelicase[Bacteroidesuniformis]/11658      QLNAISKAYVHYVT-D------GNDRGKLIMACGTGKTYTSLLIAEQLFD----------
gi|494823013|ref|WP_007558421.1|helicase[Bacteroidesplebeius]_gi|198272167|gb|EDY96436.1|helicaseCterminaldomainprotein[Bacter/      QLNAISKAYVHYMA-D------GNERGKLIMACGTGKTYTSLLIAEQLFD----------
gi|495427212|ref|WP_008151909.1|helicase[Parabacteroidesjohnsonii]_gi|218222381|gb|EEC95031.1|helicaseCterminaldomainprotein[P/      QLDAISKAYTHYII-D------GNDRGKLIMACGTGKTYTSLLITEQLLG----------
gi|933196855|emb|CUO94545.1|typeIrestrictionenzymeEcoKIsubunitR[Bacteroidesuniformis]_gi|933220098|emb|CUN86874.1|typeIrestrict      QLDAISKAYMHYIT-E------GNDRGKLIMACGTGKTYTSLLIAEQLLN----------
gi|823687748|ref|WP_047101121.1|helicase[Brachyspirahyodysenteriae]_gi|823334537|gb|KLI17932.1|helicase[Brachyspirahyodysenteri      QNEALENAHKYFKT-K--------DRGKLIMACGTGKTLTALKIAEKETN----------
gi|496436691|ref|WP_009145538.1|helicase[Phascolarctobacteriumsuccinatutens]_gi|322414007|gb|EFY04839.1|helicaseCterminaldomai/      QETALANAAKYYKT-H--------DRGKLIMACGTGKTYTSLCLVEQETQ----------
gi|503372880|ref|WP_013607541.1|helicase[Sphaerochaetaglobosa]_gi|324026933|gb|ADY13692.1|typeIIIrestrictionproteinressubunit[S      QKTAIRKCNEHFQS-N--------DRGKLIMACGTGKTFTSLKIAENIAGN---------
gi|505821748|ref|WP_015707145.1|helicase[Treponemaprimitia]_gi|299483503|gb|ADJ19584.1|helicasedomaincontainingprotein[Trepone/      QQTAIDATHNYLKT-S--------DRGKLIMACGTGKTFTSLRILENETG----------
gi|505822741|ref|WP_015708134.1|helicase[Treponemaprimitia]_gi|333739639|gb|AEF85129.1|endonucleaseandmethylaseLlaGI[Treponemap      QQTAIDATHNYLKT-S--------DRGKLIMACGTGKTFTSLRIAENETG----------
gi|488791994|ref|WP_002704400.1|helicase[Treponemasaccharophilum]_gi|380767691|gb|EIC01689.1|typeIIIrestrictionproteinressubuni      QQKAYDSTIKYFEN-H--------NRGKLIMACGTGKTFTSLRISEGLAR----------
gi|524671454|emb|CDD81750.1|putativeseptumsitedeterminingproteinMinC[Bacteroidessp.CAG:462]/11642      QQKAINRVHEYFKT-S--------DRGKLIMSCGTGKTFTSLKIAENETQ----------
gi|524573865|emb|CDC95766.1|putativeseptumsitedeterminingproteinMinC[Alistipessp.CAG:268]/11639      QQQAIDQTHAYFKI-DEATGQPAHTRGKLIMACGTGKTFTSLRIAENETG----------
gi|658617970|ref|WP_029759328.1|helicase[Fusobacteriumnucleatum]/11606      QKEARDSANKYFKE-N--------DRGKLIMACGTGKTFTSLKIAENETN----------
gi|494412037|ref|WP_007216782.1|helicase[Bacteroidescellulosilyticus]_gi|392638921|gb|EIY32752.1|hypotheticalproteinHMPREF1062_      QKQAIETAHTYFLT-R--------DRGKLIMACGTGKTYTSLCIAERETS----------
gi|383107102|gb|AFG37435.1|putativehelicase[SpirochaetaafricanaDSM8902]/11616      QVKAVQAAHDYYQE-A--------ERGKMIMACGTGKTYTSLKIAENETG----------
SAF8902III/11616      QVKAVQAAHDYYQE-A--------ERGKMIMACGTGKTYTSLKIAENETG----------
gi|652538894|ref|WP_026932796.1|helicase[Gramellaechinicola]/11633      QTQALEKTHEHFKE-N--------DRGKLIMACGTGKTFTSLKIAENETK----------
gi|639238223|ref|WP_024568839.1|helicase[Elizabethkingiaanophelis]/11628      QTNALEKTNEYLKE-H--------DRGKLIMACGTGKTFTSLRIAENETN----------
gi|652520381|ref|WP_026914582.1|helicase[Gramellaportivictoriae]/11623      QTEALEKTQDYFNE-N--------DRGKLIMACGTGKTFTSLRIAENETR----------
gi|494922183|ref|WP_007648219.1|Helicase[Formosasp.AK20]_gi|469825489|gb|EMQ95550.1|Helicasedomainprotein[Formosasp.AK20]/11626      QEEALNKTHEHFKE-N--------DRGKLIMACGTGKTFTSLRIAENETN----------
gi|522083382|ref|WP_020594591.1|hypotheticalprotein[Spirosomapanaciterrae]/11637      QRQALIQTHEHFKT-H--------DRGKLIMACGTGKTFNSLRIAENETN----------
gi|490455352|ref|WP_004326157.1|helicase[Bacteroidesovatus]_gi|392669277|gb|EIY62768.1|hypotheticalproteinHMPREF1070_03635[Bact      QKTALTKAHHYFKD-N--------DRGKLIMACGTGKTFTSLRIAENETN----------
gi|499516035|ref|WP_011202675.1|helicase[Bacteroidesfragilis]_gi|53713270|ref|YP_099262.1|helicasedomainprotein[Bacteroidesfrag      QKTALTEAHHYFKD-N--------DRGKLIMACGTGKTFTSLRIAENETN----------
gi|651338332|ref|WP_026461320.1|helicase[Adhaeribacteraquaticus]/11620      QKEALTKTHEHFKT-D--------ERGKIIMACGTGKTFTALRIAENETN----------
gi|806964642|gb|AKC17139.1|helicase[Flavobacteriumpsychrophilum]/11639      QKEAMDKTHLHFTT-S--------DRGKLIMACGTGKTYTALQIAENETN----------
gi|752684374|ref|WP_041332322.1|helicase[Flavobacteriumpsychrophilum]_gi|666882889|gb|AIG30715.1|helicase[Flavobacteriumpsychro      QKEAMDKTHLHFTT-S--------DRGKLIMACGTGKTYTALQIAENETN----------
gi|933196595|emb|CUP04894.1|DNAorRNAhelicasesofsuperfamilyII[Bacteroidesuniformis]/11631      QKEALEETNKHFKS-E--------DRGKLIMACGTGKTFTSLRIAENETN----------
gi|670509109|ref|WP_031456918.1|helicase[Flavobacteriumchungangense]/11625      QKEALDKTHLNFQS-T--------DRGKLIMACGTGKTFTSLRIAENETN----------
                                                                                                          *   .                      *        *:***  .:    .

gi|490190857|ref|WP_004089451.1|hypotheticalprotein[Helicobacterbilis]_gi|476630638|gb|EMZ37196.1|hypotheticalproteinC826_02193      ------KNS-----------IILFLAPSIALVGQTFREYAIQKS--ENFIACIVCSDSTA
gi|488896133|ref|WP_002807242.1|MULTISPECIES:restrictionendonucleaseEco57I[Campylobacter]_gi|380541096|gb|EIA65377.1|hypothetic      ------KNS-----------IVLFLAPSIALVGQTFREYCKEKT--DDFVASIVCSDSKS
gi|746602240|ref|WP_039628365.1|restrictionendonucleaseEco57I[Campylobacterlari]_gi|744819395|gb|AJD03391.1|putativehelicase[Ca      ------KNS-----------IVLFLAPSIALVGQTFREYCKEKT--DDFVASIVCSDSKS
gi|727317135|ref|WP_033772558.1|restrictionendonucleaseEco57I[Helicobacterpylori]/11622      ------K-------------IMLFLAPSIALLSQTFREYAQEKS--DPFYASIVCSDDKT
gi|727103490|ref|WP_033618954.1|restrictionendonucleaseEco57I[Helicobacterpylori]/11616      ------K-------------ITLFLAPSIALLSQTFREYAQEKS--DPFYASIVCSDDKT
gi|727340205|ref|WP_033794408.1|restrictionendonucleaseEco57I[Helicobacterpylori]/11609      ------K-------------IMLFLAPSIALLSQTFREYAQEKS--EPFYASIVCSDDKV
gi|727292407|ref|WP_033749975.1|restrictionendonucleaseEco57I[Helicobacterpylori]/11611      ------K-------------ITLFLAPSIALLSQTFREYAQEKS--EPFYASIVCSDDKV
gi|727289795|ref|WP_033747591.1|restrictionendonucleaseEco57I[Helicobacterpylori]/11612      ------K-------------ITLFLAPSIALLSQTFREYAQEKS--DPFYASIVCSDDKT
gi|727319676|ref|WP_033775047.1|restrictionendonucleaseEco57I[Helicobacterpylori]/11612      ------K-------------ITLFLAPSIALLSQTFREYAQEKS--DPFYASIVCSDDKT
gi|447212910|ref|WP_001290166.1|restrictionendonucleaseEco57I[Helicobacterpylori]_gi|308063540|gb|ADO05427.1|hypotheticalprotei      ------K-------------ITLFLAPSIALLSQTFREYAQEKS--EPFYASIVCSDDKV
gi|447212901|ref|WP_001290157.1|restrictionendonucleaseEco57I[Helicobacterpylori]_gi|384557329|gb|AFH97797.1|hypotheticalprotei      ------K-------------ITLFLAPSIALLSQTFREYAQEKN--EPFYASIVCSDDKV
gi|515849671|ref|WP_017280424.1|hypotheticalprotein[Helicobacterpylori]/11615      ------K-------------ITLFLAPSIALLSQTFREYAQEKS--DPFYASIVCSDDKV
gi|447212900|ref|WP_001290156.1|restrictionendonucleaseEco57I[Helicobacterpylori]_gi|317178970|dbj|BAJ56758.1|TypeIIGrestrictio      ------K-------------ITLFLAPSIALLSQTFREYAQEKS--DPFYASIVCSDDKV
gi|447079018|ref|WP_001156274.1|hypotheticalprotein[Helicobacterpylori]/11614      ------K-------------ITLFLAPSIALLSQTFREYAQEKS--EPFYASIVCSDDKV
gi|447212800|ref|WP_001290056.1|hypotheticalprotein[Helicobacterpylori]_gi|532153248|gb|EQL67316.1|restrictionendonucleaseEco57      ------K-------------ITLFLAPSIALLSQTFREYAQEKS--EPFYASIVCSDDKV
gi|505457237|ref|WP_015644339.1|hypotheticalprotein[Helicobacterpylori]_gi|499061160|gb|AGL70074.1|restrictionendonucleaseEco57      ------K-------------ITLFLAPSIALLSQTFREYAQEKS--DPFYASIVCSDDKV
HPYUM037X/11591      ------K-------------ITLFLAPSIALLSQTFREYAQEKS--DPFYASIVCSDDKV
gi|920026297|ref|WP_052937176.1|restrictionendonuclease[Helicobacterpylori]_gi|912528678|gb|KNX48238.1|restrictionendonucleaseE      ------K-------------ITLFLAPSIALLSQTFREYAQEKS--EPFYASIVCSDDKV
```

```
gi|915539050|ref|WP_050841327.1|restrictionendonuclease[Helicobacterpylori]_gi|908890887|gb|KNE06183.1|restrictionendonucleaseE      ------K-------------ITLFLEPSIALLSQTFREYVQEKS--EPFYASIVCSDDKV
gi|727101397|ref|WP_033616957.1|restrictionendonucleaseEco57I[Helicobacterpylori]/11592                                              ------K-------------ITLFLAPSIALLSQTFREYAQEKS--DPFYASIVCSDDKV
gi|562814875|emb|CDK13002.1|TypeISPRMsystem[Helicobacterpylori26695]/11588                                                           ------K-------------ITLFLAPSIALLSQTFREYAQEKS--EPFYASIVCSDDKV
HPYAXVIII/11588                                                                                                                      ------K-------------ITLFLAPSIALLSQTFREYAQEKS--EPFYASIVCSDDKV
gi|447079029|ref|WP_001156285.1|restrictionendonucleaseEco57I[Helicobacterpylori]_gi|393085956|gb|EJB86635.1|hypotheticalprotei      ------K-------------IMLFLAPSIALLSQTFREYAQEKS--EPFYASIVCSDDKV
gi|489105953|ref|WP_003015814.1|restrictionendonuclease[Helicobacterpylori]_gi|477503388|gb|ENH59950.1|restrictionendonucleasef      ------K-------------ITLFLAPSIALLSQTFREYAQEKS--EPFYASIVCSDDKV
gi|447079006|ref|WP_001156262.1|restrictionendonucleaseEco57I[Helicobacterpylori]_gi|393067470|gb|EJB68280.1|hypotheticalprotei      ------K-------------ITLFLAPSIALLSQTFREYVQEKS--EPFYASIVCSDDKV
gi|727314589|ref|WP_033770298.1|restrictionendonucleaseEco57I[Helicobacterpylori]/11586                                              ------K-------------ITLFLAPSIALLSQTFREYAQEKS--EPFYASIVCSDDKV
gi|447079019|ref|WP_001156275.1|restrictionendonucleaseEco57I[Helicobacterpylori]_gi|308065028|gb|ADO06920.1|hypotheticalprotei      ------K-------------ITLFLAPSIALLSQTFREYAQEKS--EPFYASIVCSDDKT
gi|447079028|ref|WP_001156284.1|restrictionendonucleaseEco57I[Helicobacterpylori]_gi|393076030|gb|EJB76784.1|hypotheticalprotei      ------K-------------ITLFLAPSIALLSQTFREYAQEKS--EPFYASIVCSDDKV
gi|393034009|gb|EJB35070.1|hypotheticalproteinHPNQ4053_0631[HelicobacterpyloriNQ4053]/11514                                         ------K-------------IMLFLAPSIALLSQTFREYAQEKS--EPFYASIVCSDDKT
gi|447079004|ref|WP_001156260.1|restrictionendonucleaseEco57I[Helicobacterpylori]_gi|384552418|gb|AFI07366.1|hypotheticalprotei      ------K-------------ITLFLAPSIALLSQTFREYAQEKS--EPFYASIVCSDNKT
gi|746011306|ref|WP_039088215.1|restrictionendonucleaseEco57I[Helicobacterpylori]_gi|733488720|gb|KHL85979.1|restrictionendonuc      ------K-------------ITLFLAPSIALLSQTFREYAQEKS--DPFYASIVCSDDKT
gi|531081162|gb|EQD92166.1|restrictionendonucleaseEco57I[HelicobacterpyloriPZ5080]/11509                                             ------K-------------ITLFLAPSIALLSQTFREYAQEKS--EPFYASIVCSDDKT
gi|544760796|ref|WP_021187395.1|restrictionendonucleaseEco57I[Helicobacterpylori]_gi|531086508|gb|EQD96745.1|restrictionendonuc      ------K-------------ITLFLAPSIALLSQTFREYAQEKS--EPFYASIVCSDDKT
gi|447079012|ref|WP_001156268.1|restrictionendonucleaseEco57I[Helicobacterpylori]_gi|393028199|gb|EJB29286.1|hypotheticalprotei      ------K-------------ITLFLAPSIALLSQTFREYAQEKS--EPFYASIVCSDDKV
gi|746008458|ref|WP_039085555.1|restrictionendonucleaseEco57I[Helicobacterpylori]_gi|733480358|gb|KHL78026.1|restrictionendonuc      ------K-------------IMLFLAPSIALLSQTFREYAQEKS--EPFYASIVCSDDKT
gi|447079011|ref|WP_001156267.1|restrictionendonucleaseEco57I[Helicobacterpylori]_gi|393038534|gb|EJB39568.1|hypotheticalprotei      ------K-------------ITLFLAPSIALLSQTFREYAQEKS--DPFYASIVCSDDKT
gi|393037163|gb|EJB38199.1|hypotheticalproteinHPNQ4161_0422[HelicobacterpyloriNQ4161]/11556                                         ------K-------------ITLFLAPSIALLSQTFREYAQEKS--DPFYASIVCSDDKT
gi|447079007|ref|WP_001156263.1|restrictionendonucleaseEco57I[Helicobacterpylori]_gi|393051817|gb|EJB52768.1|hypotheticalprotei      ------K-------------ITLFLAPSIALLSQTFREYAQEKS--EPFYASIVCSDDKT
gi|487867094|ref|WP_001940560.1|helicase[Helicobacterpylori]_gi|459505657|gb|EMG98418.1|helicaseprotein[HelicobacterpyloriGAM21      ------K-------------ITLFLAPSIALLSQTFREYAQEKS--EPFYASIVCSDDKT
gi|487872094|ref|WP_001945560.1|helicase[Helicobacterpylori]_gi|459512104|gb|EMH04523.1|helicaseprotein[HelicobacterpyloriGAM24      ------K-------------ITLFLAPSIALLSQTFREYAQEKS--EPFYASIVCSDDKT
gi|487880228|ref|WP_001953694.1|helicase[Helicobacterpylori]_gi|459529131|gb|EMH20734.1|helicaseprotein[HelicobacterpyloriGAM26      ------K-------------ITLFLVPSIALLSQTFREYAQEKS--EPFYASIVCSDDKT
gi|487882593|ref|WP_001956059.1|helicase[Helicobacterpylori]_gi|459533462|gb|EMH24859.1|helicaseprotein[HelicobacterpyloriGAM26      ------K-------------ITLFLAPSIALLSQTFREYAQEKS--DPFYASIVCSDDKT
gi|487845430|ref|WP_001918896.1|helicase[Helicobacterpylori]_gi|410713871|gb|EKQ71363.1|helicaseprotein[HelicobacterpyloriGAM10      ------K-------------ITLFLAPSIALLSQTFREYAQEKS--EPFYASIVCSDDKT
gi|447079005|ref|WP_001156261.1|restrictionendonucleaseEco57I[Helicobacterpylori]_gi|393069830|gb|EJB70625.1|hypotheticalprotei      ------K-------------ITLFLAPSIALLSQTFREYAQEKS--EPFYASIVCSDDKT
gi|459506808|gb|EMG99528.1|helicaseprotein[HelicobacterpyloriGAM121Aii]/11545                                                       ------K-------------IVLFLAPSIALLSQTFREYAQEKS--EPFYASIVCSDDKT
gi|487825283|ref|WP_001898749.1|restrictionendonucleaseEco57I[Helicobacterpylori]_gi|393107526|gb|EJC08068.1|hypotheticalprotei      ------K-------------ITLFLAPSIALLSQTFREYAQEKS--EPFYASIVCSDDKT
gi|447079008|ref|WP_001156264.1|restrictionendonucleaseEco57I[Helicobacterpylori]_gi|393058636|gb|EJB59524.1|hypotheticalprotei      ------K-------------ITLFLAPSIALLSQTFREYAQEKS--EPFYASIVCSDNKT
gi|447079015|ref|WP_001156271.1|restrictionendonucleaseEco57I[Helicobacterpylori]_gi|307637357|gb|ADN79807.1|hypotheticalprotei      ------K-------------ITLFLAPSIALLSQTFREYAQEKS--DPFYASIVCSDDKT
gi|487884861|ref|WP_001958327.1|helicase[Helicobacterpylori]_gi|459535535|gb|EMH26834.1|helicaseprotein[HelicobacterpyloriGAM26      ------K-------------ITLFLAPSIALLSQTFREYAQEKS--EPFYASIVCSDDKT
gi|487886365|ref|WP_001959831.1|helicase[Helicobacterpylori]_gi|459538727|gb|EMH29861.1|helicaseprotein[HelicobacterpyloriGAM27      ------K-------------ITLFLAPSIALLSQTFREYAQEKS--EPFYASIVCSDDKT
gi|545301637|ref|WP_021581412.1|helicase[Helicobacterpylori]_gi|535931889|gb|ERA61745.1|helicaseprotein[HelicobacterpyloriGAM11      ------K-------------VTLFLVPSIALLSQTFREYAQEKS--EPFYASIVCSDDKT
gi|447079010|ref|WP_001156266.1|restrictionendonucleaseEco57I[Helicobacterpylori]_gi|393095477|gb|EJB96081.1|hypotheticalprotei      ------K-------------ITLFLAPSIALLSQTFREYAQEKS--EPFYASIVCSDDKT
gi|447079009|ref|WP_001156265.1|restrictionendonucleaseEco57I[Helicobacterpylori]_gi|393056294|gb|EJB57206.1|hypotheticalprotei      ------K-------------ITLFLAPSIALLSQTFREYAQEKS--EPFYASIVCSDDKT
gi|471386985|gb|EMR58906.1|restrictionendonucleasefamilyprotein[HelicobacterpyloriHpH1]/11504                                       ------K-------------ITLFLAPSIALLSQTFREYAQEKS--EPFYASIVCSDDKT
gi|487875679|ref|WP_001949145.1|helicase[Helicobacterpylori]_gi|459516928|gb|EMH09091.1|helicaseprotein[HelicobacterpyloriGAM25      ------K-------------ITLFLAPSIALLSQTFREYAQEKS--EPFYASIVCSDNKT
gi|496130066|ref|WP_008854573.1|DNAdamageinducibleprotein[Commensalibacterintestini]_gi|353671689|gb|EHD13391.1|helicasedomain/      -------GK-----------MVLFLVPSLSLLSQSLTEWTQESA--IEINSFAVCSDSDV
gi|924898894|ref|WP_053556546.1|damageinducibleprotein[Sphingopyxissp.113P3]_gi|924523996|gb|ALC14732.1|damageinducibleprotei/1      -------GG-----------RVLFLVPSLALMQQTVREWSIDSE--APLRSFAVCSDAQV
gi|780802950|gb|KJS08562.1|damageinducibleprotein[Hoefleasp.BRH_c9]/11633                                                           -------GK-----------MVLYLVPSLALMSQSVREWSIDTE--TPLRSFAVCSDVQV
gi|944671432|gb|KQN22886.1|damageinducibleprotein[Sphingomonassp.Leaf32]/11415                                                      -------GK-----------FVLFLVPSLALMSQTVREWSLDST--TPLRAFAVCSDAQV
gi|944612953|gb|KQM64772.1|damageinducibleprotein[Sphingomonassp.Leaf16]/11726                                                      -------GK-----------FVLFLVPSLALMSQTVREWSLDST--TPLRAFAVCSDAQV
gi|764353851|ref|WP_044331468.1|damageinducibleprotein[Sphingomonashengshuiensis]_gi|761897338|gb|AJP71707.1|damageinduciblep/1      -------GK-----------TVLFLVPSLALMSQTIREWTIDTA--TPLRAFAVCSDVQV
gi|945874472|gb|KQZ27973.1|damageinducibleprotein[Mesorhizobiumsp.Root552]/11651                                                    -------GR-----------MVLFLVPSLALMSQTIREWTIDAV--VPLRAFAVCSDVQV
gi|759408081|ref|WP_043132403.1|damageinducibleprotein[Paracoccussanguinis]_gi|694217086|gb|KGJ14281.1|damageinducibleprotein/1      -------GK-----------RVLFMVPSLALMSQTVREWSNDRQ--QEFTAFSACSDVRI
gi|654342671|ref|WP_027836116.1|damageinducibleprotein[Maritaleamyrionectae]/11632                                                  -------GK-----------RVLFMVPSLALMSQTVREWKNDCQ--EDFTAFSACSDKKV
gi|126717687|gb|EBA14409.1|hypotheticalproteinRSK20926_02032[Roseobactersp.SK20926]/11603                                           -------GK-----------RVLFMVPSLALMSQTVREWKNDCQ--EEFTAFSACSDTKV
gi|765301116|ref|WP_044628780.1|damageinducibleprotein[Halomonasmeridiana]_gi|764089679|gb|KJD19875.1|damageinducibleprotein[/1      -------GK-----------RVLFMVPSLALMSQTVREWKNDAQ--ADFTAFSACSDKKV
gi|518053006|ref|WP_019223214.1|DNAdamageinducibleprotein[Bartonellarattaustraliani]/11654                                          -------GK-----------RVLFLVPSLALMSQTIREWTTDAE--VPLRSFAVCSDIQV
gi|493637806|ref|WP_006589578.1|DNAdamageinducibleprotein[Bartonellabirtlesii]_gi|395410345|gb|EJF76900.1|hypotheticalproteinM/      -------GK-----------RVLFLVPSLALVSQTIREWTADAQ--VPLRSFAVCSDTKV
gi|501188603|ref|WP_012231622.1|DNAdamageinducibleprotein[Bartonellatribocorum]_gi|161017843|emb|CAK01401.1|helicase/methyltra/      -------GK-----------RVLFLVPSLALVSQTIREWTADAQ--ISLRSFAVCSDTKV
gi|651388616|ref|WP_026500615.1|damageinducibleprotein[Bartonellagrahamii]/11655                                                    -------GK-----------RVLFLVPSLALVSQTIREWTADAQ--VPLRSFAVCSDTKV
gi|501188669|ref|WP_012231687.1|DNAdamageinducibleprotein[Bartonellatribocorum]_gi|161017922|emb|CAK01480.1|helicase/methyltra/      -------GK-----------RVLFLVPSLALVSQTIREWTTDAQ--VPLRSFAVCSDTKV
gi|518048832|ref|WP_019219040.1|DNAdamageinducibleprotein[Bartonellaflorencae]/11655                                                -------GK-----------RILFLVPSLALVSQTIREWTADAQ--VPLRSFAVCSDTKV
gi|518053617|ref|WP_019223825.1|DNAdamageinducibleprotein[Bartonellarattaustraliani]/11607                                          -------GK-----------RVLFLVPSLALMSQTIREWTTDAE--VPLRSFAVCSDTQV
gi|506336449|ref|WP_015856168.1|DNAdamageinducibleprotein[Bartonellagrahamii]_gi|240267492|gb|ACS51080.1|helicase/methyltransf/      -------GK-----------RVLFLVPSLALVSQTIREWTADAQ--LPLRSFAVCSDTKV
gi|494588832|ref|WP_007347427.1|DNAdamageinducibleprotein[Bartonellarattimassiliensis]_gi|395419053|gb|EJF85361.1|hypothetical/      -------GK-----------RVLFLVPSLALISQTIREWAEDTQ--VPLRSFAVCSDTQV
gi|501188650|ref|WP_012231668.1|DNAdamageinducibleprotein[Bartonellatribocorum]_gi|161017902|emb|CAK01460.1|helicase/methyltra/      -------GK-----------RVLFLVPSLALISQTIREWTEDTE--VPLRSFAVCSDTQV
gi|501188624|ref|WP_012231643.1|DNAdamageinducibleprotein[Bartonellatribocorum]_gi|161017866|emb|CAK01424.1|helicase/methyltra/      -------GK-----------HVLFLVPSLALMSQSIREWTADAQ--VPLRCFAVCSDKQI
gi|518053856|ref|WP_019224064.1|DNAdamageinducibleprotein[Bartonellarattaustraliani]/11625                                          -------GK-----------RVLFLVPSLALMSQSIREWTADAQ--VPLHCFAVCSDKQI
gi|501188053|ref|WP_012231081.1|DNAdamageinducibleprotein[Bartonellatribocorum]_gi|161017434|emb|CAK00992.1|helicase/methyltra/      -------GK-----------HVLFLVPSLALMSQSIREWTADAQ--VPLRCFAVCSDKQI
gi|651388630|ref|WP_026500626.1|damageinducibleprotein[Bartonellagrahamii]/11657                                                    -------GK-----------RVLFLVPSLALVSQTIREWTADAQ--VPLRSFAVCSDTQV
gi|518048855|ref|WP_019219063.1|DNAdamageinducibleprotein[Bartonellaflorencae]/11658                                                -------GK-----------RVLFLVPSLALISQTIREWTLDTE--VPLRSFAVCSDTQV
gi|490995101|ref|WP_004856829.1|DNAdamageinducibleprotein[Bartonelladoshiae]_gi|395413167|gb|EJF79645.1|hypotheticalproteinMCS/      -------GK-----------RVLFLVPSLALMSQTIREWTLDTE--VPLRSFAVCSDTQV
gi|491002558|ref|WP_004864279.1|DNAdamageinducibleprotein[Bartonellavinsonii]_gi|395421392|gb|EJF87643.1|hypotheticalproteinME/      -------GK-----------RVLFLVPSLALMSQTIREWTSDTE--VPLRSFAVCSDTQV
gi|491003373|ref|WP_004865094.1|DNAdamageinducibleprotein[Bartonellavinsonii]_gi|395432864|gb|EJF98838.1|hypotheticalproteinME/      -------GK-----------RVLFLVPSLALMSQTIREWTADTE--IALRSFAVCSDTQV
gi|491002426|ref|WP_004864148.1|DNAdamageinducibleprotein[Bartonellavinsonii]_gi|395421767|gb|EJF88003.1|hypotheticalproteinME/      -------GK-----------RVLFLVPSLALMSQTIREWTADTE--IPLRSFAVCSDLHI
```

```
gi|491004024|ref|WP_004865745.1|DNAdamageinducibleprotein[Bartonellavinsonii]_gi|395432542|gb|EJF98522.1|hypotheticalproteinME/      -------GK-----------RVLFLVPSLALMSQTIREWTADTE--IALRSFAVCSDTQV
gi|648396442|ref|WP_026088193.1|damageinducibleprotein[Bartonellarattaustraliani]/11593                                               -------GK-----------RVLFLVPSLALMSQTIREWTTDTE--VPLRSFAVCSDTQV
gi|748190174|ref|WP_039761290.1|damageinducibleprotein[Bartonellaqueenslandensis]/11658                                               -------GK-----------HVLFLVPSLALMSQTIREWTTDAE--VPLRSFAVCSDTQV
gi|494817599|ref|WP_007553007.1|DNAdamageinducibleprotein[Bartonellasp.DB56]_gi|395411312|gb|EJF77837.1|hypotheticalproteinMC/1      -------GK-----------RVLFLVPSLSLMSQTIREWTTDTE--IPLRSFAVCSDTQV
gi|515763376|ref|WP_017195976.1|DNAdamageinducibleprotein[Bartonellabirtlesii]/11656                                                 -------GK-----------RVLFLVPSLSLMSQTIREWTTDTE--IPLRSFAVCSDTQV
gi|494589420|ref|WP_007348014.1|DNAdamageinducibleprotein[Bartonellarattimassiliensis]_gi|395416278|gb|EJF82667.1|hypothetical/      -------GK-----------RVLFLVPSLALISQTIREWTEDTK--VPLRSFAVCSDTQV
gi|506336469|ref|WP_015856188.1|DNAdamageinducibleprotein[Bartonellagrahamii]_gi|240267512|gb|ACS51100.1|helicase/methyltransf/      -------GK-----------RVLFLVPSLALISQTIREWTEDTE--VPLRSFAVCSDTQV
gi|749308087|ref|WP_040132227.1|damageinducibleprotein[Bartonellatribocorum]_gi|605051878|emb|CDO49988.1|helicase/methyltransf/      -------GK-----------RVLFLVPSLALISQTIREWTEDTE--VPLRSFAVCSDTQV
gi|515763800|ref|WP_017196400.1|DNAdamageinducibleprotein[Bartonellabirtlesii]/11658                                                 -------GK-----------RVLFLVPSLALMSQTIREWTLDTE--VPLRSFAVCSDTQV
gi|492186994|ref|WP_005773380.1|DNAdamageinducibleprotein[Bartonellaelizabethae]_gi|395418361|gb|EJF84688.1|hypotheticalprotei/      -------GK-----------RVLFLVPSLALMSQTIREWTLDTE--IPLRSFAVCSDTQV
gi|501188615|ref|WP_012231634.1|DNAdamageinducibleprotein[Bartonellatribocorum]_gi|161017856|emb|CAK01414.1|helicase/methyltra/      -------GK-----------RVLFLVPSLALMSQTIREWTLDTE--IPLRSFAVCSDTQV
gi|501187470|ref|WP_012230499.1|DNAdamageinducibleprotein[Bartonellatribocorum]_gi|161017090|emb|CAK00648.1|helicase/methyltra/      -------GK-----------RVLFLVPSLALMSQTIREWTLDTE--IPLRSFAVCSDTQV
gi|501188636|ref|WP_012231655.1|DNAdamageinducibleprotein[Bartonellatribocorum]_gi|161017879|emb|CAK01437.1|helicase/methyltra/      -------GK-----------RVLFLVPSLALMSQTIREWTLDTE--IPLRSFAVCSDTQV
gi|930151941|emb|CTQ34818.1|UvsWhelicase[Jannaschiarubra]/11641                                                                      -------GG-----------TVLFMVPSLALMSQAVREWTNDAA--VPLRSFAVCSDAKV
gi|928977106|ref|WP_054001461.1|damageinducibleprotein[Shimiasp.SK013]_gi|927698852|gb|KPA22698.1|typeIrestrictionenzymeEcoKIs/      -------GK-----------RVLFLVPSLALIAQTVREWTYDTE--LELRSFAVCSDAQV
gi|738872465|ref|WP_036760884.1|damageinducibleprotein[Leisingeradaeponensis]/11643                                                   -------DG-----------TVLFLVPSLALMAQTVREWTTDAD--VPLRSFAVCSDTQV
gi|518050881|ref|WP_019221089.1|DNAdamageinducibleprotein[Bartonellasenegalensis]/11652                                              -------GK-----------FVLFLVPSLALMSQTVREWTTDTE--IGLRSFAVCSDTQV
gi|493980596|ref|WP_006923523.1|DNAdamageinducibleprotein[Bartonellawashoensis]_gi|395413322|gb|EJF79796.1|hypotheticalprotein/      -------GK-----------FVLFLVPSLALMSQTVREWTTDAE--IGLRSFAVCSDTQV
gi|493983106|ref|WP_006925984.1|DNAdamageinducibleprotein[Bartonellawashoensis]_gi|395418127|gb|EJF84455.1|hypotheticalprotein/      -------GK-----------FVLFLVPSLALMSQTVREWTTDAE--IGLRSFAVCSDTQV
gi|499494671|ref|WP_011181311.1|DNAdamageinducibleprotein[Bartonellahenselae]_gi|49239008|emb|CAF28308.1|hypotheticalproteinBH/      -------GK-----------FVLFLVPSLALMSQTVREWTTDTE--IGLRSFAVCSDTQV
gi|651388426|ref|WP_026500441.1|damageinducibleprotein[Bartonellagrahamii]/11652                                                     -------GK-----------FVLFLVPSLALISQTVREWTTDAE--IGLRSFAVCSDTQV
gi|506337280|ref|WP_015856999.1|DNAdamageinducibleprotein[Bartonellagrahamii]_gi|240268402|gb|ACS51990.1|helicase/methyltransf/      -------GK-----------FVLFLVPSLALMSQTVREWTTDAE--IGLRSFAVCSDTQV
gi|494817535|ref|WP_007552943.1|DNAdamageinducibleprotein[Bartonellasp.DB56]_gi|395412002|gb|EJF78518.1|hypotheticalproteinMC/1      -------AK-----------FVLFLVPSLALMSQTVREWTTDAE--MGLRSFAVCSDTQV
gi|493638099|ref|WP_006589864.1|DNAdamageinducibleprotein[Bartonellabirtlesii]_gi|395409809|gb|EJF76394.1|hypotheticalproteinM/      -------GK-----------FVLFLVPSLALMSQTVREWTTDTE--MGLRSFAVCSDTQV
gi|748189589|ref|WP_039760707.1|damageinducibleprotein[Bartonellaqueenslandensis]/11651                                               -------GK-----------FVLFLVPSLALMSQTVREWTTDVE--IGLRSFAVCSDTQV
gi|501189483|ref|WP_012232501.1|DNAdamageinducibleprotein[Bartonellatribocorum]_gi|161018902|emb|CAK02460.1|helicase/methyltra/      -------GK-----------FVLFLVPSLALMSQTVREWTTDAE--IGLCSFAVCSDTQV
gi|518049181|ref|WP_019219389.1|DNAdamageinducibleprotein[Bartonellaflorencae]/11652                                                 -------GK-----------FVLFLVPSLALMSQTVREWTTDAE--IGLRSFAVCSDTQV
gi|492193255|ref|WP_005774528.1|DNAdamageinducibleprotein[Bartonellaelizabethae]_gi|395416403|gb|EJF82779.1|hypotheticalprotei/      -------GK-----------FVLFLVPSLALMSQTVREWTTDAE--IDLRSFAVCSDTQV
gi|498387325|ref|WP_010701288.1|helicase/methyltransferase[Bartonellabovis]_gi|478418724|gb|ENN92426.1|helicase/methyltransfera      -------GK-----------RVLFLVPSLALMSQTVREWANDTQ--IYLRSFAVCSDTQV
gi|319408697|emb|CBI82352.1|conservedhypotheticalprotein[BartonellaschoenbuchensisR1]/11661                                          -------GK-----------RVLFLVPSLALMSQTVREWATDAQ--IHLRSFAVCSDTQV
gi|494741726|ref|WP_007477134.1|DNAdamageinducibleprotein[Bartonellamelophagi]_gi|395423925|gb|EJF90113.1|hypotheticalproteinM/      -------GK-----------RVLFLVPSLALMSQTIHEWANDAQ--ICLRSFAVCSDTQV
gi|737261405|ref|WP_035245349.1|damageinducibleprotein[Alcanivoraxjadensis]_gi|686989709|gb|KGD62556.1|hypotheticalproteinT9A_/      -------GK-----------RVLFMVPSLALMSQTVTEWSNDAT--VPLRSFAVCSDAQV
gi|916687564|ref|WP_051294655.1|damageinducibleprotein[Gemmobacternectariphilus]/11641                                               -------GK-----------LVLFLVPSLALMAQTVREWTADSE--TPLHSFAVCSDTQV
gi|740294127|ref|WP_038132495.1|damageinducibleprotein[Thioclavasp.DT234]_gi|660524826|gb|KEO54689.1|hypotheticalproteinDT23_/1      -------GG-----------RVLFMVPSLALMSQTVREWTNDTE--TPLRSFAVCSDAQV
gi|568331062|ref|WP_024099615.1|helicase[Phaeobactergallaeciensis]_gi|568232121|gb|AHD12007.1|putativehelicase[Phaeobactergalla      -------GK-----------LVLFLVPSLALMAQTVREWTGDTA--TPLRSFAVCSDTQV
gi|518810459|ref|WP_019966413.1|DNAdamageinducibleprotein[Pannonibacterphragmitetus]/11635                                           -------GK-----------RVLFMVPSLALMSQTVREWTNDTE--TPIRAFAVCSDAHV
gi|495305760|ref|WP_008030510.1|DNAdamageinducibleprotein[Rhodobactersp.SW2]_gi|259021698|gb|EEW25000.1|typeIIIrestrictionprot/      -------GK-----------RVLFMVPSLALMSQTVREWTNDTE--TPIRAFAVCSDAHV
gi|506403303|ref|WP_015923022.1|DNAdamageinducibleprotein[Rhodobactersphaeroides]_gi|221162951|gb|ACM03922.1|HypotheticalProte/      -------GK-----------RVLFMVPSLALMSQTVREWTNDTE--TPIRAFAVCSDAHV
gi|738818386|ref|WP_036708790.1|damageinducibleprotein[Paracoccussanguinis]_gi|694226786|gb|KGJ22541.1|damageinducibleprotein/1      -------GK-----------RVLFMVPSLALMSQTVREWTNDTE--TPIRAFAVCSDAHV
gi|746096057|ref|WP_039162453.1|damageinducibleprotein[Gallibacteriumanatis]_gi|703617775|gb|KGQ63868.1|damageinducibleprotei/1      -------GK-----------SVLFLVPSLALLSQSLTEWTQEAD--IPLHSFAVCSDSDV
gi|737716785|ref|WP_035685631.1|damageinducibleprotein[Avibacteriumparagallinarum]/11643                                              -------GK-----------SVLFLVPSLALLSQSLTEWTQEAD--IPLHSFAVCSDSDV
gi|540072553|gb|ERF78672.1|hypotheticalproteinN561_04930[Gallibacteriumanatis12656/12}/11651                                        -------GK-----------NVLFLVPSLALLSQSLTEWTQEAA--IPLHSFAVCSDSDV
gi|917509519|ref|WP_052115936.1|damageinducibleprotein[Gallibacteriumanatis]_gi|702414161|gb|KGQ42233.1|damageinducibleprotei/1      -------GK-----------NVLFLVPSLALLSQSLTEWTQEAA--IPLHSFAVCSDSDV
gi|746082942|ref|WP_039150268.1|damageinducibleprotein[Gallibacteriumanatis]_gi|703617078|gb|KGQ63194.1|damageinducibleprotei/1      -------GK-----------NVLFLVPSLALLSQSLTEWTQEAA--IPLHSFAVCSDSDV
gi|822522885|ref|WP_046952840.1|damageinducibleprotein[Haemophilushaemolyticus]_gi|821501762|gb|KKZ59267.1|damageinduciblepro/1      -------GK-----------KVLFLVPSLALLSQSLTEWTQESE--YPLHCFAVCSDSDV
gi|404381014|gb|EJZ77501.1|putativehelicase[Pasteurellamultocidasubsp.gallicidaP1059]/11681                                          -------GK-----------KVLFLVPSLALLSQSLTEWTQEST--TSLHCFAVCSDSDV
gi|298281660|gb|EFI23149.1|helicaseCterminaldomainprotein[Neisseriasp.oraltaxon014str.F0314]/11493                                   -------GK-----------RVLFLVPSLSLLSQTLREWTQDAD--LPLRNFAVCSDSEV
gi|348013055|gb|EGY51980.1|helicasedomainprotein[Neisseriashayeganii871]/11481                                                       -------GK-----------RVLFLVPSLSLLSQTLREWTQDSE--TPLNSFAVCSDSDV
gi|496407140|ref|WP_009116004.1|DNAdamageinducibleprotein[Neisseriawadsworthii]_gi|349794756|gb|EGZ48566.1|helicasedomainprote/      -------GK-----------RVLFLVPGLSLLSQTLREWTQDSE--IALNSFAVCSDSDV
gi|517216441|ref|WP_018405259.1|DNAdamageinducibleprotein[Marinobacterlipolyticus]/11656                                             -------GK-----------RVLFLVPSLSLMGQTLTEWTQESA--VPLKSFAVCSDSDV
gi|738230264|ref|WP_036185691.1|damageinducibleprotein[Marinobactersp.ELB17]/11671                                                   -------GK-----------RVLFLVPSLSLMGQTLTEWTQESA--VPLKSFAVCSDSDV
gi|447197741|ref|WP_001274997.1|DNAdamageinducibleprotein[Acinetobacterbaumannii]_gi|408512324|gb|EKK13969.1|typeIIIrestrictio/      -------GK-----------NVLFLVPSLALLSQTLDEWTQQTS--LGIRSFAVCSDSDV
gi|820775897|ref|WP_046739909.1|damageinducibleprotein[Acinetobactersp.AG1]_gi|819909758|gb|KKW75938.1|damageinducibleprotein/1      -------GK-----------NVLFLVPSLALLSQTLDEWTQDTM--IGIRSFAVCSDSDV
gi|584595163|ref|WP_024160846.1|helicase[Acinetobacterpittii]_gi|576702530|emb|CDI28049.1|helicasedomaincontainingprotein[Acin/      -------GK-----------NVLFLVPSLALLSQTLDEWTQQTS--FGIRSFAVCSDSDV
gi|514963042|ref|WP_016651366.1|hypotheticalprotein[Acinetobactersp.NIPH2036]_gi|514162665|gb|EPG41391.1|hypotheticalproteinF90      -------GK-----------NVLFLVPSLALLSQTLDEWTQQTS--LGIRSFAVCSDSDV
gi|515965564|ref|WP_017396147.1|DNAdamageinducibleprotein[Acinetobacterhaemolyticus]/11667                                           -------GK-----------NVLFLVPSLALLSQTLDEWTQQTS--LGIRSFAVCSDSDV
gi|491346810|ref|WP_005204747.1|MULTISPECIES:hypotheticalprotein[Acinetobacter]_gi|480335823|gb|ENX56033.1|hypotheticalproteinF      -------GK-----------NVLFLVPSLALLSQTLDEWTQDTM--IGIRSFAVCSDSDV
gi|693044472|ref|WP_032215268.1|damageinducibleprotein[Escherichiacoli]_gi|607725731|gb|EZE08230.1|damageinducibleprotein[Esc/1      -------GK-----------TVLFLVPSLALLSQTLDEWTQDTL--IDLRCFAVCSDSDV
gi|742382177|ref|WP_038861304.1|damageinducibleprotein[Cronobactermuytjensii]_gi|924333747|gb|ALB69766.1|damageinducibleprote/1      -------GK-----------TVLFLVPSLALLSQTLDEWTQDTL--IDLRCFAVCSDSDV
gi|915912804|ref|WP_050927035.1|damageinducibleprotein[Yersiniaenterocolitica]_gi|899805802|emb|CQH32042.1|typeIrestrictionenz/      -------SK-----------TVLFLVPSLALLSQTLDEWTQDTL--VDLRCFAVCSDSDV
gi|921111327|gb|KON62781.1|typeIrestrictionenzymeEcoKIsubunitR[Komagataeibactereuropaeus]/11492                                     -------GG-----------RVLFLVPSLSLLSQTLTEWTQESQ--VPLHSFAVCSDSDV
gi|582024278|gb|AHI27615.1|DNAhelicaserestrictionenzymeTypeIIIRsubunit[KomagataeibacterxylinusE25]/11636                             -------GG-----------RVLFLVPSLSLLSQTLTEWTQESQ--VPLHSFAVCSDSAV
gi|347662673|dbj|BAK86128.1|DNAhelicaserestrictionenzymeTypeIIIRsubunit[KomagataeibactermedellinensisNBRC3288]/11737                 -------GG-----------RVLFLVPSLSLLSQTLTEWTQESQ--VPLHSFAVCSDSDV
gi|917445053|ref|WP_052051471.1|damageinducibleprotein[Acetobactertropicalis]/11429                                                  -------GG-----------RVLFLVPSLSLLSQSLTEWTQESQ--IPLHSFAVCSDSDV
gi|453329445|dbj|GAC88472.1|DNAhelicaserestrictionenzymetypeIIIRsubunit[GluconobacterthailandicusNBRC3255]_gi|530287981|dbj|GAD      -------GG-----------RVLFLVPSLSLLSQSLTEWTQESQ--VPLHSFAVCSDSDV
gi|889770922|ref|WP_048844278.1|damageinducibleprotein[Gluconobacterfrateurii]/11419                                                 -------GG-----------RVLFLVPSLSLLSQSLTEWTQESQ--VPLHSFAVCSDSDV
gi|504886208|ref|WP_015073310.1|hypotheticalprotein[Gluconobacteroxydans]_gi|411027954|gb|AFW01209.1|hypotheticalproteinB932_16      -------GG-----------RVLFLVPSLSLLSQSLTEWTQESQ--VPLHSFAVCSDSDV
```

gi|480040211|gb|ENV79751.1|hypotheticalproteinF942_01641[AcinetobacterursingiiANC3649]/11432 -------GK-----------RVLFLVPSLSLLSQTLTEWTQESS--TPLCSFAVCSDSDI
gi|822498222|ref|WP_046936504.1|damageinducibleprotein[Xanthomonasgardneri]_gi|821751224|gb|KLB07223.1|damageinducibleprotein/1 -------GK-----------RVLFLVPSLSLLSQSLTEWTQESV--TPLHSFAVCSDSEV
gi|654059559|ref|WP_027705556.1|damageinducibleprotein[Zymobacterpalmae]/11635 -------GK-----------AVLFLVPSLSLLSQTLTEWTQESD--VPLHSFAVCSDHDV
gi|643535620|ref|WP_025224315.1|damageinducibleprotein[Methylophilaceaebacterium11]_gi|577704400|gb|EUJ10546.1|putativehelicas/ -------GK-----------HVLFLVPSLSLLSQTLTEWTQESS--TPLHSFAVCSDSDV
MBA11I/11635 -------GK-----------HVLFLVPSLSLLSQTLTEWTQESS--TPLHSFAVCSDSDV
gi|655355857|ref|WP_028762287.1|damageinducibleprotein[Shewanellacolwelliana]/11632 -------GK-----------RVLFLVPSLSLLSQTLTEWTQQSE--IPLHSFAVCSDSDV
gi|753204236|ref|WP_041510579.1|damageinducibleprotein[Shewanellasp.cp20]_gi|752457765|gb|KIO36326.1|damageinducibleprotein[S/1 -------GK-----------RVLFLVPSLSLLSQTLTEWTQHSE--VPLHSFAVCSDSDV
KPNNIH30III/11619 -------GK-----------RVLFLVPSLSLLSQTLTEWTQHSE--VPLHSFAVCSDSDV
gi|749295994|ref|WP_040120352.1|damageinducibleprotein[Klebsiellapneumoniae]_gi|723249300|gb|AIX81926.1|damageinducibleprotei/1 -------GK-----------RVLFLVPSLSLLSQTLTEWTQHSE--VPLHSFAVCSDSDV
gi|641639739|emb|CDQ54010.1|unnamedproteinproduct[Klebsiellapneumoniae]/11641 -------GK-----------RVLFLVPSLSLLSQTLTEWTQHSE--VPLHSFAVCSDSDV
gi|490797922|ref|WP_004660060.1|hypotheticalprotein[Acinetobactersp.NIPH236]_gi|479876779|gb|ENU27973.1|hypotheticalproteinF992 -------GK-----------RVLFLVPSLSLLSQTLTEWTQEST--TPLHSYAVCSDTEI
gi|916750926|ref|WP_051357982.1|damageinducibleprotein[Acinetobactertjernbergiae]/11453 -------GK-----------RVLFLVPSLSLLSQTLTEWTQEST--TPLHSYAVCSDNEI
gi|691098662|ref|WP_032025060.1|damageinducibleprotein[Acinetobacterbaumannii]_gi|593567521|gb|EXR50735.1|restrictionendonucle/ -------GK-----------RVLFLVPSLSLLSQTLTEWTQEST--TPLHSYAVCSDTEV
gi|736669405|ref|WP_034676049.1|damageinducibleprotein[Acinetobacterbaumannii]/11643 -------GK-----------RVLFLVPSLSLLSQTLTEWTQESK--TPLHSYAVCSDTEV
gi|491368928|ref|WP_005226826.1|hypotheticalprotein[Acinetobactersp.NIPH1847]_gi|480173259|gb|ENX09797.1|hypotheticalproteinF89 -------GK-----------RVLFLVPSLSLLSQTLTEWTQEST--TPLHSYAVCSDTEV
gi|796853993|ref|WP_045795953.1|damageinducibleprotein[Acinetobacterindicus]_gi|787929761|gb|KJV44219.1|damageinducibleprotei/1 -------GK-----------RVLFLVPSLSLLSQTLTEWTQEST--TPLHSYAVCSDTEI
gi|295797463|gb|ADG32253.1|typeIIIrestrictionproteinressubunit[ThiomonasintermediaK12]/11676 -------GK-----------RVLFLVPSLNLLSQTLTEWTQESA--VPLHSYAVCSDEDV
gi|499871179|ref|WP_011551913.1|DNAdamageinducibleprotein[Myxococcusxanthus]_gi|108467544|gb|ABF92729.1|putativerestriction/mo/ -------GK-----------RVLFLVPSLALLSQTLTEWTQESE--ARIQSFAVCSDSDV
gi|495313852|ref|WP_008038600.1|DNAdamageinducibleprotein[Bartonellatamiae]_gi|395424181|gb|EJF90368.1|hypotheticalproteinME5_/ -------GK-----------RVLFLVPSLALLSQTLTEWTQQSE--TKLHSFAVCSDAEV
gi|501453335|ref|WP_012476784.1|DNAdamageinducibleprotein[Leptospirabiflexa]_gi|167777824|gb|ABZ96123.1|Helicase[Leptospirabif/ -------GK-----------RVLFLVPSLSLLSQSLTEWTQESE--IPLHSFAVCSDSEV
gi|738104613|ref|WP_036063193.1|damageinducibleprotein[Leptospiranoguchii]/11652 -------GK-----------RILFLVPSLSLLSQALSEWTQESE--ISLHSFAVCSDSEI
gi|410001039|gb|EKO51661.1|typeIIIrestrictionenzyme,ressubunit[Leptospirakirschneristr.200802841]/11651 -------GK-----------RILFLVPSLSLLSQALSEWTQESE--ISLHSFAVCSDSEI
gi|490901851|ref|WP_004763774.1|typeIIIrestrictionenzyme,ressubunit[Leptospirakirschneri]_gi|464209249|gb|EMN24014.1|typeIIIres -------GK-----------RILFLVPSLSLLSQALSEWTQESE--ISLHSFAVCSDSEI
gi|464398131|gb|EMO65902.1|typeIIIrestrictionenzyme,ressubunit[Leptospirakirschneristr.200803703]/11648 -------GK-----------RILFLVPSLSLLSQALSEWTQESE--ISLHSFAVCSDSEI
gi|945029024|gb|KQQ84900.1|damageinducibleprotein[Xanthomonassp.Leaf131]/11423 -------GR-----------RVLFLVPSLSLLSQSLTEWTQESA--IPLHSFAVCSDSEV
gi|896511314|ref|WP_049418183.1|damageinducibleprotein[Stenotrophomonasmaltophilia]/11639 -------GK-----------RVLFLVPSLSLLSQTLTEWTQESA--IPLHSFAVCSDSDV
gi|896513082|ref|WP_049419616.1|damageinducibleprotein[Stenotrophomonasmaltophilia]/11638 -------GK-----------RVLFLVPSLSLLQQTLTEWTQESA--IPLHSFAVCSDSDV
gi|896580616|ref|WP_049469425.1|damageinducibleprotein[Stenotrophomonasmaltophilia]/11641 -------GG-----------RVLFLVPSLSLLQQTLTEWTQESA--IPLHSFAVCSDSDV
gi|648289350|ref|WP_026070499.1|damageinducibleprotein[Stenotrophomonasmaltophilia]/11639 -------GK-----------RVLFLVPSLSLLQQTLTEWTQEST--IPLHSFAVCSDSDV
gi|656272786|ref|WP_029218442.1|damageinducibleprotein[Xanthomonascassavae]/11639 -------GK-----------RVLFLVPSLSLLQQTLTEWTQESA--IPLHSFAVCSDSDV
gi|406976236|gb|EKD98747.1|hypotheticalproteinACD_23C00290G0002[unculturedbacterium]/11679 -------GK-----------RVLFLVPSLSLLSQTLTEWTQESA--TLLHSFAVCSDSDV
gi|828145737|ref|WP_047238120.1|damageinducibleprotein[Chromobacteriumsubtsugae]/11645 -------GK-----------RVLFLVPSLSLLSQTLTEWTQESD--TPLHSFAVCSDSDV
gi|654311174|ref|WP_027824327.1|damageinducibleprotein[Laribacterhongkongensis]/11641 -------GK-----------RVLFLVPSLSLLSQTLTEWTQESA--TPLHSFAVCSDSDV
gi|757242173|gb|KIR15516.1|typeIrestrictionenzymeEcoKIsubunitR[Pseudomonasfluorescens]/11778 -------GK-----------RVLFLVPSLSLLSQSLTEWTQESE--TLLHSFAVCSDSDV
gi|653605161|ref|WP_027616682.1|damageinducibleprotein[Pseudomonassp.URHB0015]/11639 -------GK-----------RVLFLVPSLSLLSQTLTEWTQESE--TPLHSFAVCSDSDV
gi|520868025|ref|WP_020308060.1|helicase[Pseudomonassyringae]_gi|519159034|gb|EPM49935.1|helicasedomaincontainingprotein[Pseud/ -------GK-----------RVLFLVPSLSLLSQTLTEWTQESE--TPLHSFAVCSDSDV
gi|940156567|gb|KPX71668.1|Helicasedomaincontainingprotein[Pseudomonasamygdalipv.lachrymans]/11677 -------GK-----------RVLFLVPSLSLLSQTLTEWTQESE--TPLHSFAVCSDSDV
gi|505460508|ref|WP_015647610.1|helicase[Pseudomonassp.GLE121]_gi|499110212|ref|YP_007974374.1|helicasedomaincontainingprotein/ -------GQ-----------RVLFLVPSLSLLSQTLTEWTQESA--IPLHSFAVCSDSDV
gi|940235551|gb|KPY45311.1|hypotheticalproteinALO48_200154[Pseudomonassyringaepv.rhaphiolepidis]/11631 -------SQ-----------RILFLVPSLSLLSQTLTEWTQESA--IPLHSFAVCSDSDV
gi|520880808|ref|WP_020312235.1|helicase[Pseudomonassyringae]_gi|519172158|gb|EPM62027.1|helicasedomaincontainingprotein[Pseud/ -------GK-----------RVLFLVPSLSLLSQTLTEWTQESE--TPLHSFAVCSDSDV
gi|829046479|ref|WP_047282259.1|damageinducibleprotein[Pseudomonasfluorescens]/11630 -------GK-----------RVLFLVPSLSLLSQTLTEWTQESE--TPLHSFAVCSDSDV
gi|499415493|ref|WP_011102960.1|DNAdamageinducibleprotein[Pseudomonassyringaegroupgenomosp.3]_gi|28867279|ref|NP_789898.1|heli/ -------GK-----------RVLFLVPSLSLLSQTLTEWTQESE--TPLHSFAVCSDSDV
gi|730238299|ref|WP_033942897.1|damageinducibleprotein[Pseudomonasaeruginosa]/11629 -------GK-----------RVLFLVPSLSLLSQTLTEWTQESA--TPLHSFAVCSDSDV
gi|495310125|ref|WP_008034873.1|DNAdamageinducibleprotein[Pseudomonassp.GM60]_gi|398186685|gb|EJM74052.1|putativehelicase[Pseu/ -------GM-----------RVLFLVPSLSLLSQTLTEWTQESE--TPLHSFAVCSDSDV
gi|779992653|ref|WP_045428690.1|damageinducibleprotein[Pseudomonassp.MT1]_gi|699967872|dbj|BAP80334.1|DNAhelicaserestrictione/1 -------GK-----------RVLFLVPSLSLLSQTLTEWTQESE--TPLHSFAVCSDSDV
gi|786037289|ref|WP_045733274.1|damageinducibleprotein[Pseudomonaspseudoalcaligenes]_gi|782987904|gb|KJU79152.1|damageinducib/1 -------GK-----------RVLFLVPSLSLLSQTLTEWTQESA--TPLHSFAVCSDSDV
gi|749666366|ref|WP_040265445.1|damageinducibleprotein[Pseudomonasrhodesiae]/11632 -------GK-----------RVLFLVPSLSLLSQTLTEWTQESA--TPLHSFAVCSDSDV
gi|734902433|ref|WP_034133698.1|damageinducibleprotein[Pseudomonasfluorescens]/11635 -------GK-----------RVLFLVPSLSLLSQTLTEWTQESA--TPLHSFAVCSDSDV
gi|490551031|ref|WP_004416146.1|typeIIrestrictionmodificationsystemrestriction/helicasesubunit[Pseudomonassyringae]_gi|4432823/ -------GK-----------RVLFLVPSLSLLSQTLTEWTQESA--TPLHSFAVCSDSDV
gi|489199911|ref|WP_003109152.1|DNAdamageinducibleprotein[Pseudomonasaeruginosa]_gi|552648708|gb|ERY63184.1|hypotheticalprotei/ -------GK-----------RVLFLVPSLSLLSQTLTEWTQESA--TLLHSFAVCSDSDV
gi|730641237|ref|WP_034061328.1|damageinducibleprotein[Pseudomonasaeruginosa]/11636 -------GK-----------RVLFLVPSLSLLSQTLTEWTQESE--TPLHSFAVCSDSDV
gi|751304980|ref|WP_041013386.1|damageinducibleprotein[Pseudomonasxanthomarina]_gi|703407611|emb|CEG51014.1|Helicasedomaincon/1 -------GK-----------RVLFLVPSLSLLSQTLTEWTQESE--TPLHSFAVCSDSDV
gi|939335207|ref|WP_054881520.1|damageinducibleprotein[Pseudomonassp.NBRC111128]/11632 -------GK-----------RVLFLVPSLSLLSQTLTEWTQESA--TPLHSFAVCSDSDV
gi|757693555|ref|WP_042923792.1|damageinducibleprotein[Pseudomonaschloritidismutans]_gi|780910610|gb|KJS78302.1|damageinducib/1 -------GK-----------RVLFLVPSLSLLSQTLTEWTQESE--TPLHSFAVCSDSDV
gi|665953944|ref|WP_031319989.1|damageinducibleprotein[Pseudomonastolaasii]/11626 -------GK-----------RVLFLVPSLSLLSQTLTEWTQETG--TPLHSFAVCSDSDV
gi|771600573|ref|WP_045213746.1|damageinducibleprotein[Pseudomonassp.21]_gi|770081123|gb|KJJ98634.1|damageinducibleprotein[Ps/1 -------GK-----------RVLFLVPSLSLLSQTLTEWTQESE--TPLHNFAVCSDSDV
gi|740871396|ref|WP_038656644.1|damageinducibleprotein[Pseudomonasstutzeri]_gi|627772214|gb|AHY41437.1|damageinducibleprotein/1 -------GK-----------RVLFLVPSLSLLSQTLTEWTQESE--TPLHSFAVCSDSDV
gi|946242205|gb|KRC93782.1|damageinducibleprotein[Pseudomonassp.Root9]/11634 -------GK-----------RVLFLVPSLSLLSQTLTEWTQEST--TPLHSFAVCSDSDV
gi|553777322|ref|WP_023109107.1|MULTISPECIES:hypotheticalprotein[Pseudomonas]_gi|552638674|gb|ERY53184.1|hypotheticalproteinQ05 -------GK-----------RVLFLVPSLSLLSQTLTEWTQESA--TPLHSFAVCSDSDV
gi|896383370|ref|WP_049324618.1|damageinducibleprotein[Pseudomonasstutzeri]/11627 -------GK-----------RVLFLVPSLSLLSQTLTEWTQESE--TPLHSFAVCSDSDV
gi|696158510|gb|AIT41787.1|putativehelicase[Pseudomonassp.LM8]/11630 -------GK-----------RVLFLVPSLSLLSQTLTEWTQESE--IPLHSFAVCSDSDV
gi|504634892|ref|WP_014821994.1|DNAdamageinducibleprotein[Pseudomonasstutzeri]_gi|390985249|gb|AFM35242.1|helicasedomainconta/1 -------GK-----------RVLFLVPSLSLLSQTLTEWTQESE--IPLHSFAVCSDSDV
gi|544783974|ref|WP_021206729.1|DNAdamageinducibleprotein[Pseudomonasstutzeri]_gi|533224779|gb|EQM79688.1|DNAdamageinduciblep/1 -------GK-----------RVLFLVPSLSLLSQTLTEWTQESE--IPLHSFAVCSDSDV
gi|504567385|ref|WP_014754487.1|MULTISPECIES:DNAdamageinducibleprotein[Pseudomonas]_gi|388559493|gb|AFK68634.1|helicasedomain/1 -------GK-----------RVLFLVPSLSLLSQTLTEWTQEST--TPLHSFAVCSDSDV
gi|654441469|ref|WP_027912052.1|damageinducibleprotein[Pseudomonassp.URIL14HWK12:I7]/11630 -------GK-----------RVLFLVPSLSLLSQTLTEWTQESA--TPLHSFAVCSDSDV
gi|941470066|ref|WP_055127894.1|damageinducibleprotein[Pseudomonasmediterranea]/11631 -------SK-----------RVLFLVPSLSLLSQTLTEWTQESA--TPLHSFAVCSDSDV
gi|557815599|ref|WP_023446184.1|DNAdamageinducibleprotein[Pseudomonaschloritidismutans]_gi|557364081|gb|ESQ98190.1|DNAdamagei/1 -------GK-----------RVLFLVPSLSLLSQTLTEWTQESA--TPLHSFAVCSDSDV

```
gi|727814791|ref|WP_033898247.1|damageinducibleprotein[Pseudomonassp.FH1]_gi|567648005|gb|ETK23229.1|helicasedomaincontaining/1      -------GK-----------RVLFLVPSLSLLSQTLTEWTQESA--TPLHSFAVCSDSDV
gi|503278702|ref|WP_013513363.1|DNAdamageinducibleprotein[Desulfovibrioaespoeensis]_gi|316942375|gb|ADU61426.1|typeIIIrestrict/      -------GK-----------RVLFMVPSLALLSQTLTEWTQESA--TPLHSFAVCSDSDV
gi|505228796|ref|WP_015415898.1|typeIIIrestrictionendonucleasesubunitR[Desulfovibriopiezophilus]_gi|451896976|emb|CCH49855.1|Ty      -------GK-----------RVLFMVPSLALLSQTLTEWTQQSV--TPLHSFAVCSDSDV
gi|517732543|ref|WP_018902751.1|hypotheticalprotein[Variovoraxparadoxus]/11675                                                      -------GK-----------RVLFLVPSLSLLSQTLTEWTQESA--VPLHSFAVCSDSDV
gi|550909475|ref|WP_022662570.1|DNAdamageinducibleprotein[Desulfovibriolongus]/11637                                                -------GK-----------RVLFLVPSLALLSQTLTEWTQESA--TPLHSFAVCSDSDV
gi|945494542|gb|KQV50024.1|damageinducibleprotein[Massiliasp.Root335]/11647                                                         -------GK-----------RVLFLVPSLSLLSQTLTEWTQESA--TPLHSFAVCSDSDV
gi|334196917|gb|AEG70102.1|helicasedomainprotein[RalstoniasolanacearumPo82]/11663                                                   -------GK-----------RVLFLVPSLSLLSQTLTEWTQESE--TPLHSFAVCSDSDV
gi|656006746|ref|WP_029048170.1|damageinducibleprotein[Cupriavidussp.amp6]/11661                                                    -------GK-----------RVLFLVPSLSLLSQTLTEWTQESE--TPLHSFAVCSDSDV
BPS2196ORF3054P/11659                                                                                                               -------GK-----------RVLFLVPSLSLLSQTLTEWTQESE--TPLHSFAVCSDSDV
gi|740932312|ref|WP_038717332.1|damageinducibleprotein[Burkholderiapseudomallei]_gi|760211882|gb|KIX58314.1|damageinduciblepr/1     -------GK-----------RVLFLVPSLSLLSQTLTEWTQESE--TPLHSFAVCSDSDV
gi|755810813|ref|WP_042583861.1|damageinducibleprotein[Burkholderiasp.MSHR3999]_gi|755174952|gb|KIP19790.1|DEAD/DEAHboxhelicas/     -------GK-----------RVLFLVPSLSLLSQTLTEWTQESE--TPLHSFAVCSDSDV
gi|484586570|gb|AGK48195.1|DEAD/DEAHboxhelicasefamilyprotein[BurkholderiathailandensisMSMB121]/11646                                -------GK-----------RVLFLVPSLSLLSQTLTEWTQESE--TPLHSFAVCSDSDV
gi|737582935|ref|WP_035553941.1|damageinducibleprotein[Burkholderiasp.9120]/11658                                                   -------GK-----------RVLFLVPSLSLLSQTLTEWTQESE--TPLHSFAVCSDSDV
gi|740989872|ref|WP_038773225.1|damageinducibleprotein[Burkholderiapseudomallei]_gi|705645291|gb|KGS70691.1|DEAD/DEAHboxhelica/     -------GK-----------RVLFLVPSLSLLSQSLTEWTQESE--TPLHSFAVCSDSDV
gi|654912525|ref|WP_028363424.1|damageinducibleprotein[Burkholderiasp.JPY366]/11659                                                 -------GK-----------RVLFLVPSLSLLSQSLTEWTQESE--TPLHSFAVCSDSDV
gi|740955879|ref|WP_038740512.1|damageinducibleprotein[Burkholderiapseudomallei]_gi|714684932|gb|KGW74438.1|DEAD/DEAHboxhelica/     -------GK-----------RVLFLVPSLSLLSQTLTEWTQESE--TPLHSFAVCSDSDV
gi|919203714|ref|WP_052759215.1|damageinducibleprotein[Xanthomonasaxonopodis]/11388                                                 -----PSNV-----------LVLFLVPSISLLSQTLKEWTAQA--TDEIRAFAVCSDTKV
gi|522152381|ref|WP_020663589.1|hypotheticalprotein[Amycolatopsisbenzoatilytica]/11605                                              ----DGQPT-----------NVLFLVPSISLLSQTLREWTAQT--QTPMRTFAVCSDTKV
gi|663196013|ref|WP_030233899.1|damageinducibleprotein[Streptomycessp.NRRLS455]/11641                                               --GGQGEHT-----------TVLFLVPSIALLSQTLREWRYET--RTPMRAFAVCSDVKV
gi|750429553|ref|WP_040711444.1|damageinducibleprotein[Nocardiopsispotens]/11622                                                    --AGQGEHT-----------TVLFLVPSIALLSQTLREWTQEA--EVPMRPFAVCSDTKV
gi|518656139|ref|WP_019819475.1|hypotheticalprotein[Saccharomonosporasaliphila]/11623                                               --AGDGEQT-----------TALFLVPSIALLSQALREWSYEA--EVEIRSFAVCSDAQV
gi|759722933|ref|WP_043433758.1|damageinducibleprotein[Streptomycespluripotens]_gi|733404978|gb|KHK94361.1|damageinduciblepro/1     --AGQGEHT-----------TVLFLVPSIALLSQSLREWSYES--EVPLRAFAVCSDAQV
gi|663424167|ref|WP_030419030.1|damageinducibleprotein[Streptomycessp.NRRLF5065]/11627                                              --AGQGEHT-----------TVLFLVPSIALLSQSLREWSYEA--EVPLRAFAVCSDAQV
gi|663412917|ref|WP_030408353.1|damageinducibleprotein[Streptomycessp.NRRLF5917]/11630                                              --AGQGEHT-----------TVLFLVPSIALLSQSLREWSYEA--EVPLRAFAVCSDAQV
gi|671521483|ref|WP_031506489.1|damageinducibleprotein[Streptomycesmegasporus]/11630                                                --AGQGEHT-----------TVLFLVPSIALLSQSLREWSYEA--EVPLRAFAVCSDAQV
gi|740053144|ref|WP_037902776.1|damageinducibleprotein[Streptomycessp.TAA204]/11626                                                 --AGEGEHT-----------TVLFLVPSIALLSQTLREWSFEA--EVPLRSFAVCSDTKV
gi|663409375|ref|WP_030404974.1|MULTISPECIES:damageinducibleprotein[Streptomyces]/11627                                             --ASEGEHT-----------SVLFLVPSIALLSQTLREWSFEA--EVPLRSFAVCSDTKV
gi|746628608|ref|WP_039654277.1|damageinducibleprotein[Streptomycessp.MUSC125]_gi|745644381|gb|KIE25557.1|damageinducibleprot/1     --AGEGEHT-----------SVLFLVPSIALLSQTLREWSFEA--EVSLRPFAVCSDTKV
gi|727322066|ref|WP_033777320.1|damageinducibleprotein[Salinisporapacifica]/11627                                                   --AGEAEHT-----------TVLFLVPSIALLSQSLREWSYES--DVTLRAFAVCSDTKV
gi|653653242|ref|WP_027655726.1|damageinducibleprotein[Salinisporapacifica]/11618                                                   --AGEGEHT-----------NVLFLVPSIALLSQSLREWSYES--EVPLRAFAVCSDAKV
gi|648632543|ref|WP_026324294.1|damageinducibleprotein[Salinisporapacifica]/11621                                                   --AGAGEHT-----------NVLFLVPSIALLSQSLREWSYES--EVPLRAFAVCSDVQV
gi|881018002|ref|WP_048738463.1|damageinducibleprotein[Corynebacteriumfalsenii]/11676                                               -----GGKA-----------RILFLVPSIALLSQTLKEWTAQA--REEIHPYAVCSDQKV
gi|505465526|ref|WP_015652417.1|helicase[Corynebacteriumcallunae]_gi|459385704|gb|AGG67995.1|helicase[CorynebacteriumcallunaeDS      ----NGNKA-----------RILFLVPSISLLSQTLKEWTAQK--TMDLRPVAVCSDSKV
gi|41223062|emb|CAF19007.1|DNAorRNAhelicaseofsuperfamilyII[CorynebacteriumglutamicumATCC13032]/11646                                ----NGNKA-----------RILFLVPSISLLSQTLKEWTAQK--TMDLRPVAVCSDSKV
CGL13032I/11643                                                                                                                     ----NGNKA-----------RILFLVPSISLLSQTLKEWTAQK--TMDLRPVAVCSDSKV
gi|751257081|ref|WP_040967675.1|damageinducibleprotein[Corynebacteriumglutamicum]_gi|748811620|gb|AJE67931.1|damageinduciblep/1     ----SGGRA-----------RVLFLVPSIALLSQSLKEWTAQT--QVDLRSYAVCSDTKV
gi|749040539|ref|WP_040086524.1|damageinducibleprotein[Corynebacteriumhumireducens]/11663                                            EDRKNRANA-----------RVLFLVPSISLLSQTLREWTAQT--ELDLRAYAVCSDSKV
gi|491662899|ref|WP_005519615.1|helicase[Corynebacteriummatruchotii]_gi|224946756|gb|EEG27965.1|helicaseCterminaldomainprotein/     -----GGKA-----------RILFLVPSISLLSQTLKEWTAQA--RIDMRSYAVCSDNKV
gi|746717312|ref|WP_039676231.1|damageinducibleprotein[Corynebacteriumminutissimum]_gi|734991118|gb|KHO29219.1|damageinducibl/1     -----GGRA-----------RVLFLVPSISLLSQTLKEWTAQNQEHLPLKSYAVCSDTKV
gi|908654892|emb|CQD15529.1|SuperfamilyIIDNA/RNAhelicase(modularprotein)[Corynebacteriumstriatum]/11674                             -----GGRA-----------RVLFLVPSISLLSQTLKEWTAQNQEHLPLKSYAVCSDTKV
gi|895994446|ref|WP_049064418.1|damageinducibleprotein[Corynebacteriumaurimucosum]/11667                                            -----GGRA-----------RVLFLVPSISLLSQTLKEWTAQNQEHLPLKSYAVCSDTKV
gi|896123331|ref|WP_049145390.1|damageinducibleprotein[Corynebacteriumaurimucosum]/11669                                            -----GGRA-----------RVLFLVPSISLLSQTLKEWTAQNQEHLPLKSYAVCSDTKV
gi|817681178|ref|WP_046645422.1|damageinducibleprotein[Corynebacteriumstriatum]_gi|817551679|gb|KKO79868.1|damageinduciblepro/1     -----GGRA-----------RVLFLVPSISLLSQTLKEWTAQNQEHLPLKSYAVCSDTKV
gi|896139239|ref|WP_049159146.1|damageinducibleprotein[Corynebacteriumaurimucosum]/11675                                            -----GGRA-----------RVLFLVPSISLLSQTLKEWTAQNQEHLPLKSYAVCSDTKV
gi|489943947|ref|WP_003847254.1|helicase[Corynebacteriumammoniagenes]_gi|295968218|gb|EFG81467.1|helicaseCterminaldomainprotei/     -----GNKA-----------RVLFLVPSIALLSQTLREWTAQS--TMDLRCFAVCSDTKV
gi|516653464|ref|WP_018022453.1|helicase[Corynebacteriumdoosanense]_gi|697778282|gb|AIT61018.1|damageinducibleprotein[Coryneba/     -----GNKA-----------RILFLVPSIALLSQTLREWTAQS--TTELRSFAVCSDTKA
gi|493894179|ref|WP_006840129.1|helicase[Corynebacteriumlipophiloflavum]_gi|227078819|gb|EEI16782.1|helicaseCterminaldomainpro/     -----GGKA-----------RVLFLVPSISLLSQTLKEWTAQA--CVDLRSIAVCSDTKV
gi|740831472|ref|WP_038616755.1|damageinducibleprotein[Corynebacteriumpseudotuberculosis]_gi|666103058|gb|AIG06935.1|putativeh/     -----GGRA-----------RVLFLVPSIALLSQTLREWTAQA--TVSMRSFAVCSDTKA
gi|518023322|ref|WP_019193530.1|helicase[Corynebacteriumtimonense]/11655                                                            -----GGKA-----------RVLFLVPSIALLSQTLREWTAQA--TMEMRSFAVCSDTKA
gi|552767697|ref|WP_023019559.1|MULTISPECIES:hypotheticalprotein[Corynebacterium]_gi|550739013|gb|ERS38609.1|hypotheticalprotei      -----GGRA-----------RVLFLVPSIALLSQTLREWTAQA--TVDMRSFAVCSDTKV
gi|652659121|ref|WP_027017812.1|damageinducibleprotein[Corynebacteriumpseudodiphtheriticum]/11644                                   ----GGGKA-----------RVLFLVPSIALLSQTLREWTAQA--TVDMRSFAVCSDTKV
gi|749039309|ref|WP_040085390.1|damageinducibleprotein[Corynebacteriumhumireducens]/11657                                            -----GGRA-----------RVLFLVPSIALLSQTLKEWTAQT--EVDLRPYAVCSDTKV
gi|740835591|ref|WP_038620874.1|damageinducibleprotein[Corynebacteriumulcerans]_gi|698973854|gb|AIU33664.1|HelicaseCterminald/1     -----GGKA-----------RVLFLVPSIALLSQTLREWTAQT--TMDLKTFAVCSDSKA
gi|805307599|ref|WP_046096452.1|damageinducibleprotein[Corynebacteriumulcerans]_gi|803357323|gb|AKA97638.1|Damageinduciblepro/1     -----GGKA-----------RILFLVPSIALLSQTLREWTAQT--TMDLKTFAVCSDSKA
gi|504083555|ref|WP_014317549.1|helicase[Corynebacteriumdiphtheriae]_gi|371584249|gb|AEX47914.1|superfamilyIIDNA/RNAhelicase[Co     -----GGKA-----------RILFLVPSISLLSQTLREWTAQA--GADLRSFAVCSDSKV
gi|896182193|ref|WP_049193580.1|damageinducibleprotein[Corynebacteriumjeikeium]/11645                                               -----GGKA-----------RILFLVPSISLLSQTLKEWTAQA--GVDLRSFAVCSDSKV
gi|917636459|ref|WP_052205460.1|damageinducibleprotein[Corynebacteriumriegelii]_gi|914295811|gb|AKV59187.1|damageinduciblepro/1     -----GGRA-----------RVLFLVPSIALLAQTLREWTAQA--HLDLKSFAVCSDNKA
gi|737013522|ref|WP_035009205.1|damageinducibleprotein[Corynebacteriumjeikeium]/11651                                               -----GGRA-----------RVLFLVPSIALLAQTLREWTAQA--RMDLKSFAVCSDNKA
gi|737006291|ref|WP_035002094.1|damageinducibleprotein[Corynebacteriumjeikeium]/11658                                               -----GGKA-----------RVLFLVPSISLLSQTLKEWTAQA--RLDMRTFAVCSDSKV
gi|737010378|ref|WP_035006133.1|damageinducibleprotein[Corynebacteriumjeikeium]/11650                                               -----GGKA-----------RVLFLVPSISLLSQTLKEWTAQG--RMDMRTFAVCSDAKV
gi|737010473|ref|WP_035006228.1|damageinducibleprotein[Corynebacteriumjeikeium]/11657                                               -----GGRA-----------RVLFLVPSISLLSQTLKEWTAQA--TVSLRSYAVCSDAKV
gi|737006250|ref|WP_035002053.1|damageinducibleprotein[Corynebacteriumjeikeium]/11658                                               -----GGRA-----------RVLFLVPSISLLSQTLKEWTAQT--TVSLRSYAVCSDAKV
gi|923018168|ref|WP_053412254.1|damageinducibleprotein[Corynebacteriumlactis]_gi|922845184|gb|ALA67504.1|helicase[Corynebacter/     -----GGKA-----------RILFLVPSISLLSQTLKEWTAQA--RLDMRTFAVCSDSKV
gi|923018885|ref|WP_053412970.1|damageinducibleprotein[Corynebacteriumlactis]_gi|922845845|gb|ALA68165.1|helicase[Corynebacter/     -----GGKA-----------RILFLVPSISLLSQTLKEWTAQA--RLDMRTFAVCSDSKV
gi|512464870|ref|WP_016422869.1|hypotheticalprotein[Corynebacteriumsp.HFH0082]_gi|512047830|gb|EPD47505.1|hypotheticalproteinHM     -----GGKA-----------RILFLVPSISLLSQTLKEWTAQG--RLDMRTFAVCSDSKV
gi|881012697|ref|WP_048733406.1|damageinducibleprotein[Corynebacteriumurealyticum]/11670                                            -----GGKA-----------RVLFLVPSISLLSQTLKEWTAQG--RLDMRTFAVCSDSKV
gi|755608478|ref|WP_042530519.1|damageinducibleprotein[Corynebacteriumsingulare]_gi|754340239|gb|AJI78730.1|putativehelicase[C/     -----GGKA-----------RILFLVPSISLLSQTLKEWTAQG--RLDMRAFAVCSDSKV
```

gi|491421796|ref|WP_005279593.1|helicase[Corynebacteriumaccolens]_gi|227077180|gb|EEI15143.1|helicaseCterminaldomainprotein[Co/	-----GGKA-----------RILFLVPSISLLSQTLKEWTAQG--RLDMRTFAVCSDSKV
gi|550752286|gb|ERS51451.1|hypotheticalproteinHMPREF1282_00033[Corynebacteriumsp.KPL1856]_gi|550759961|gb|ERS58964.1|hypothetic	-----GGKA-----------RILFLVPSISLLSQTLKEWTAQG--RLDMRAFAVCSDSKV
gi|497955053|ref|WP_010269209.1|helicase[Corynebacteriumbovis]/11599	-----GGRA-----------RVLFCVPSLSLLAQSMREWTTHA--ELDLRCFAVCSDTKV
gi|860580040|ref|WP_048470569.1|hypotheticalprotein[Mycobacteriumchlorophenolicum]_gi|859098575|gb|KMO76871.1|typeIrestrictione	-----GGSA-----------RILFLVPSISLLSQSLREWTAQC--QLDMRAFAVCSDTKV
gi|262085905|gb|ACY21873.1|typeIIIrestrictionproteinressubunit[GordoniabronchialisDSM43247]/11632	-----GGSA-----------RILFAVPSISLLSQTLREWTAQT--ETDIRAFAVCSDTKV
gi|495996338|ref|WP_008720917.1|typeIIIrestrictionendonucleasesubunitR[Rhodococcussp.AW25M09]_gi|443412350|emb|CCQ18455.1|TypeI	-----GGSA-----------RILFAVPSISLLSQTLREWTAQT--QLDIRAFAVCSDSKV
gi|739284636|ref|WP_037147410.1|damageinducibleprotein[Rhodococcusfascians]/11641	-----GGSA-----------RILFAVPSISLLSQTLREWTAQT--QLDIRAFAVCSDSKV
gi|739328195|ref|WP_037190574.1|damageinducibleprotein[Rhodococcusfascians]/11643	-----GGSA-----------RILFAVPSISLLSQTLREWTAQT--QLDIRAFAVCSDSKV
gi|811698335|emb|CMN21041.1|helicase[Mycobacteriumtuberculosis]/11725	-----GGSA-----------RILLLVPSISLLSQTLREWTAQS--ELDVRAFAVCSDTKV
gi|801577898|emb|CFK23773.1|helicase[Mycobacteriumtuberculosis]/11675	-----GGSA-----------RILLLVPSISLLSQTLREWTAQS--ELDVRAFAVCSDTKV
MTU18II/11606	-----GGSA-----------RILLLVPSISLLSQTLREWTAQS--ELDVRAFAVCSDTKV
gi|887719858|emb|CMT27886.1|helicase[Mycobacteriumtuberculosis]/11667	-----GGSA-----------RILLLVPSISLLSQTLREWTAQS--ELDVRAFAVCSDTKV
gi|621694743|gb|KAS87156.1|hypotheticalproteinAL70_01801[MycobacteriumtuberculosisTKK_03_0115]/11604	-----GGSA-----------RILLLVPSISLLSQTLREWTAQS--ELDVRAFAVCSDTKV
gi|888128072|emb|CLQ84422.1|helicase[Mycobacteriumtuberculosis]_gi|889917090|emb|CLY86893.1|helicase[Mycobacteriumtuberculosis]	-----GGSA-----------RILLLVPSISLLSQTLREWTAQS--ELDVRAFAVCSDTKV
gi|884823424|emb|CKL96672.1|helicase[Mycobacteriumtuberculosis]/11466	-----GGSA-----------RILLLVPSISLLSQTLREWTAQS--ELDVRAFAVCSDTKV
gi|505103239|ref|WP_015290341.1|hypotheticalprotein[Mycobacteriumcanettii]_gi|432162733|emb|CCK60117.1|Conservedproteinofunknow	-----GGSA-----------RILFLVPSISLLSQTLREWTAQT--ELDVRAFAVCSDTKV
gi|505116250|ref|WP_015303352.1|hypotheticalprotein[Mycobacteriumcanettii]_gi|432158726|emb|CCK56026.1|Conservedproteinofunknow	-----GGSA-----------RILLLVPSISLLSQTLREWTAQS--ELDVRAFAVCSDTKV
gi|432154735|emb|CCK51974.1|Conservedproteinofunknownfunction(part1)[MycobacteriumcanettiiCIPT140060008]/11588	-----GGSA-----------RILLLVPSISLLSQTLREWTAQS--ELDVRAFAVCSDTKV
gi|809008027|ref|WP_046295375.1|hypotheticalprotein[Mycobacteriumsp.UM_Kg17]/11624	-----GGAA-----------RILFLVPSISLLSQTLRAWTAQT--ELDLRAFAVCSDTKV
gi|808802633|ref|WP_046284139.1|hypotheticalprotein[Mycobacteriumsp.UM_NZ2]/11624	-----GGAA-----------RILFLVPSISLLSQTLRGWTAQT--ELDLRAFAVCSDTKV
gi|750125774|ref|WP_040429852.1|damageinducibleprotein[Corynebacteriumnuruki]/11668	-----GGKA-----------RILFAVPSISLLSQTLREWNAQS--TVDMRSFAVCSDNKV
gi|602237614|gb|EYT60960.1|damageinducibleprotein[Dietziasp.UCDTHP]/11473	-----GGRA-----------RILFAVPSISLLSQTLREWSAQC--QLDMRSFAVCSDNKV
gi|665897412|ref|WP_031264618.1|damageinducibleprotein[Dietziasp.UCDTHP]_gi|602237599|gb|EYT60945.1|helicase[Dietziasp.UCDTH/11	-----GGRA-----------RILFAVPSISLLSQTLREWSAQC--QLDMRSFAVCSDNKV
gi|502903674|ref|WP_013138650.1|helicase[Segniliparusrotundus]_gi|296181291|gb|ADG98197.1|typeIIIrestrictionproteinressubunit[S	-----SGYV-----------RILFCVPSISLLSQTLREWTAQT--ATPLRAFAVCSDSKV
gi|780248306|ref|WP_045538930.1|hypotheticalprotein[Gordoniasp.no.9]_gi|778142514|gb|KJR06917.1|hypotheticalproteinUG54_12320[G	-----GGRA-----------RIVFCVPSISLLSQTLREWTAQT--SMPLRAFAVCSDQKV
gi|665897368|ref|WP_031264574.1|damageinducibleprotein[Dietziasp.UCDTHP]_gi|602239137|gb|EYT62483.1|helicase[Dietziasp.UCDTH/11	-----GGRA-----------RILFAVPSIALLSQTLREWTARA--ELDIRAFAVCSDTKV
gi|493372923|ref|WP_006329236.1|helicase[Gordoniarhizosphera]_gi|403207917|dbj|GAB88168.1|hypotheticalproteinGORHZ_006_00370[Go	-----HGSA-----------RILFCVPSISLLSQTLREWTAQT--QLDLRAFGVCSDTKV
gi|764904870|ref|WP_044487821.1|hypotheticalprotein[Mycobacteriumtuberculosis]_gi|625005353|gb|KBZ57120.1|hypotheticalproteinK8	-----GGNA-----------RVLFCVPSISLLSQTLREWTAQT--QLDLRAFAVCSDTKV
gi|927068212|ref|WP_053778441.1|damageinducibleprotein[Gordoniasp.NB41Y]_gi|926660541|gb|EMP14526.2|damageinducibleprotein[G/11	-----AGNA-----------RVLFCVPSISLLSQTLREWTAQT--QLDLRAFAVCSDTKV
gi|935514169|ref|WP_054429894.1|damageinducibleprotein[Mycobacteriumimmunogenum]_gi|929801120|gb|KPG29247.1|damageinduciblepr/1	-----GGNA-----------RILFAVPSISLLSQTLREWTAQT--QLDLRAFAVCSDTKV
gi|941317728|ref|WP_055118574.1|hypotheticalprotein[Mycobacteriumperegrinum]/11657	-----GGSA-----------RILFAVPSISLLSQTLREWTAQT--QLNLRAFAVCSDTRV
gi|933683673|gb|ALG86377.1|hypotheticalproteinACH46_20120[Gordoniasp.QH11]/11636	-----GGSA-----------RILFAVPSISLLSQTLREWTAQT--QLDLRAFAVCSDTKV
gi|873553947|emb|CRY84110.1|Predictedhelicase[Nocardiafarcinica]/11688	-----GGNA-----------RILFAVPSISLLSQTLREWTAQT--QLDLRAFAVCSDNKV
gi|499525749|ref|WP_011212389.1|helicase[Nocardiafarcinica]_gi|54019339|dbj|BAD60707.1|hypotheticalprotein[NocardiafarcinicaIFM	-----GGNA-----------RILFAVPSISLLSQTLREWTAQT--QLDLRAFAVCSDNKV
gi|640109609|ref|WP_024794187.1|damageinducibleprotein[Tomitellabiformata]/11653	-----GGSA-----------RILFAVPSISLLSQTLREWTAQT--KLDLRAFAVCSDQKV
gi|640109991|ref|WP_024794334.1|hypotheticalprotein[Tomitellabiformata]/11639	-----GGSA-----------RILFAVPSISLLSQTLREWTAQT--KLDLRAFAVCSDQKV
gi|939125496|ref|WP_054831438.1|damageinducibleprotein[Rhodococcuserythropolis]/11669	-----GGNA-----------RILFAVPSISLLSQTLREWTAQT--QLDLRAFAVCSDTKV
gi|910258550|ref|WP_050062711.1|hypotheticalprotein[Rhodococcussp.RD6.2]_gi|846871967|emb|CRK49225.1|conservedhypotheticalprote	-----GGSA-----------KVLFAVPSISLLSQTLREWTAQT--QLELRAFAVCSDTKV
gi|797148189|gb|KJY52358.1|putativeHelicase[Bifidobacteriumasteroides]/11801	-------RDERYPDLKNAPFRVLFLAPSIALVSQTFQYWTHQA--MDRMAGFVVCSDETA
gi|535476570|gb|EFG27357.2|hypotheticalproteinHMPREF9020_00999[ScardoviainopinataF0304]/11827	-------RDNEYSESKNSPFRVLFLAPSIALVSQTFNYWTHQS--KEQISSFVVCSDTTA
gi|737482729|ref|WP_035462496.1|hypotheticalprotein[Alicyclobacillusmacrosporangiidus]/11512	-------GD-----------LVLFCAPSIALVNQTIRAWKRDT--TLPLRFVAVTSDRGV
gi|685434354|gb|KGA95679.1|helicase[BacillusalcalophilusATCC27647=CGMCC1.3604]/11575	-------KK--------GIFRVLYLVPSIQLLSQTLRGWTADTKF--DMDSIAVCSDRKV
gi|945370253|gb|KQU27251.1|helicase[Bacillussp.Leaf13]/11557	-------KN--------DVFRVLYLVPSIQLLSQTLRGWTADTNY--NMDAIAVCSDRKV
gi|880997015|ref|WP_048719116.1|helicase[Enterococcushirae]/11563	-------KG--------DIFRVLYLVPSIQLLSQTLRGWTGDTNY--EMDTIAVCSDRKV
gi|945356929|gb|KQU14100.1|helicase[Bacillussp.Leaf49]/11547	-------KQ--------GVFKVLYLVPSIQLLSQTLKSWTSDTNF--IMDSIAVCSDTKV
gi|763482876|gb|KIZ53217.1|helicase[Bacillussafensis]/11563	-------KD--------GTFRVLYLVPSIQLLSQSLRGWTADSKYRKDMDTFAVCSDRKV
gi|852223292|ref|WP_048309707.1|helicase[Anaerobacillusmacyae]_gi|850322910|gb|KMM38590.1|helicase[Anaerobacillusmacyae]/11573	-------KD--------GIFRVLYLVPSIQLLSQSLRGWTADSKYNEHMDTFAVCSDRKV
gi|739708665|ref|WP_037562619.1|helicase[Sporolactobacillusterrae]/11573	-------KT--------EPFRVLYFVPSIQLLSQSLRGWTADSKYREDMDTFAVCSDRKV
gi|496687561|ref|WP_009329104.1|MULTISPECIES:helicase[Bacillus]_gi|317390867|gb|EFV71668.1|endonucleaseandmethylaseLlaGI[Bacill	-------KN--------GTFRVLYLVPSIQLLSQSLRGWTADSKYRENMDTFAVCSDRKV
gi|930213599|gb|KPH74225.1|helicase[Oceanobacilluscaeni]/11569	-------KN--------GPFRVLYLVPSIQLLSQSLRGWTADSKFRHDMDTFAVCSDRKV
gi|516366003|ref|WP_017756036.1|helicase[Bacillussp.ZYK]/11571	-------KD--------GTFRVLYFVPSIQLLSQSLRGWTADSNYRENMDTFAVCSDRKV
gi|488909977|ref|WP_002821052.1|helicase[Oenococcusoeni]_gi|399965230|gb|EJN99855.1|endonucleaseandmethylaseLlaGI[Oenococcusoen	-------QG-------KQTFYLLYLVPSIQLLTQTLFSWNADVSDELELVSFAVTSDNKA
gi|495019967|ref|WP_007745780.1|helicase[Oenococcuskitaharae]_gi|366983682|gb|EHN59081.1|typeIIIrestrictionenzyme,ressubunit[Oe	-------EE-------KQTFYLLYLVPSIQLLTQTLFSWNADVSDELELVSFAVTSDNKA
gi|489666119|ref|WP_003570406.1|typeIIIrestrictionendonuclease[Lactobacilluscasei]_gi|410527834|gb|EKQ02696.1|typeIIIrestrictio	-------ER-------KDNYFVLYLVPSIQLLSQTLFNWNSDVTPETDLVSFAVTSDRNA
gi|511447623|gb|EPC78110.1|superfamilyIIDNA/RNAhelicase[Lactobacillusparacaseisubsp.paracaseiLpp221}/11615	-------EQ-------KKDYFVLYLVPSIQLLSQTLFSWNSDITPTTNLVSFAVTSDRNA
gi|497700411|ref|WP_010014595.1|helicase[Lactobacilluscoryniformis]/11585	-------EQ-------KQDYFVLYLVPSIQLLSQTLFSWNSDVTTATNLVSFAVTSDRNA
gi|489784048|ref|WP_003687939.1|helicase[Lactobacillusmali]_gi|394428539|gb|EJF01080.1|superfamilyIIDNA/RNAhelicase[Lactobacill	-------EY-------KQDYFVLYLIPSIQLLSQTLFSWNSDVNPANELISFAVTSDRNA
gi|446780825|ref|WP_000858081.1|helicase[Staphylococcusaureus]_gi|374396604|gb|EHQ67835.1|typeIIIrestrictionenzyme,ressubunit[S	-------SG-------KKIYNILYLVPSIQLLTQTLFSWNTDKSINFNINSFAVTSDRKA
gi|510796658|ref|WP_016171456.1|hypotheticalprotein[Enterococcusdispar]_gi|508167532|gb|EOT44083.1|hypotheticalproteinOMK_00227	-------SG-------QKVYNVLYLVPSIQLLTQTLFSWNSDVSDDFELNSFAVTSDRKA
gi|612613473|gb|EZS30411.1|hypotheticalproteinW603_02482[StaphylococcusaureusVET0353R]_gi|612881917|gb|EZU93992.1|hypotheticalp	-------SG-------QKVYNVLYLVPSIQLLTQTLFSWNSDVVDDFELNSFAVTSDRKA
gi|488332893|ref|WP_002402278.1|helicase[Enterococcusfaecalis]_gi|306513864|gb|EFM82466.1|helicaseCterminaldomainprotein[Enter/	-------TS-------KETFNVLYLVPSIQLLSQTLIGWNNDT--ELSMHSFAVTSDRNA
gi|921166029|ref|WP_053111806.1|helicase[Enterococcusdurans]_gi|915771660|gb|AKX85928.1|helicase[Enterococcusdurans]/11562	-------TN-------KETFNVLYLVPSIQLLSQTLIGWNNDT--ELSMHSFAVTSDRNA
gi|498525358|ref|WP_010825030.1|hypotheticalprotein[Enterococcusfaecalis]_gi|487059631|gb|EOJ86727.1|hypotheticalproteinWOC_005	-------TS-------KETFNVLYLVPSIQLLSQTLIGWNNDT--ELSMHSFAVTSDRNA
gi|498412624|ref|WP_010722070.1|hypotheticalprotein[Enterococcusfaecium]_gi|486634654|gb|EOF75253.1|hypotheticalproteinSGC_0253	-------TN-------KETFNVLYLVPSIQLLSQTLIGWNNDT--ELSMHSFAVTSDRNA
gi|498408534|ref|WP_010718364.1|hypotheticalprotein[Enterococcusfaecium]_gi|486609608|gb|EOF50873.1|hypotheticalproteinSCW_0257	-------TN-------KETFNVLYLVPSIQLLSQTLIGWNNDT--ELSMHSFAVTSDRNA
gi|498407475|ref|WP_010717483.1|hypotheticalprotein[Enterococcusfaecalis]_gi|486594855|gb|EOF36348.1|hypotheticalproteinSCK_005	-------TS-------KETFNVLYLVPSIQLLSQTLIGWNNDT--ELSMHSFAVTSDRNA
gi|514885392|ref|WP_016615344.1|helicase[Enterococcusfaecalis]_gi|514505843|gb|EPH71676.1|helicaseprotein[Enterococcusfaecalis0	-------TS-------KETFNVLYLVPSIQLLSQTLIGWNNDT--ELSMHSFAVTSDRNA

```
gi|325157603|gb|EGC69759.1|helicaseCterminaldomainprotein[EnterococcuscasseliflavusATCC12755]/11561    -------TN-------KETFYVLYLVPSIQLLSQTLIGWNNDT--ELSMHSFAVTSDRNA
gi|895856206|ref|WP_048967668.1|helicase[Enterococcusfaecalis]/11551    -------TN-------KETFNVLYLVPSIQLLSQTLIGWNNDT--ELSMHSFAVTSDRNA
gi|639458836|ref|WP_024636366.1|helicase[Enterococcusfaecium]_gi|658176502|gb|KEI51159.1|helicase[EnterococcusfaeciumUC7256]/1/    -------TN-------KATFNVLYLVPSIQLLSQTLIGWNNDT--ELSMHSFAVTSDRNA
gi|488266511|ref|WP_002337719.1|hypotheticalprotein[Enterococcusfaecium]_gi|430581409|gb|ELB19854.1|hypotheticalproteinOIQ_0351    -------TN-------KETFNVLYLVPSIQLLSQTLIGWNNDT--ELSMHSFAVTSDRNA
gi|446353356|ref|WP_000431211.1|helicase[Staphylococcusaureus]_gi|253726329|gb|EES95058.1|helicaseCterminaldomainprotein[Staph/    -------SN--------RQFKVLYLVPSIQLLTQTLRGWNNDT--EYTMTSMAVTSDRDA
gi|672712068|emb|CDM14336.1|DNAorRNAhelicaseofsuperfamilyII[StaphylococcusepidermidisPM221]/11464    -------AN--------NQFKVLYLVPSIQLLTQTLRGWNNDT--ELKIASMAVTSDRDA
gi|896298990|ref|WP_049279905.1|helicase[Staphylococcusaureus]/11571    -------AS--------NQFKVLYLVPSIQLLTQTLRGWNNDT--ELKIASMAVTSDRDA
gi|541903432|gb|ERH36190.1|hypotheticalproteinSEQU_00085[StaphylococcusequorumUMCCNS924]/11578    -------AN--------NQFKVLYLVPSIQLLTQTLRGWNNDT--ELKIASMAVTSDRDA
gi|488289747|ref|WP_002360955.1|helicase[Enterococcusfaecalis]_gi|256682216|gb|EEU21911.1|endonucleaseandmethylaseLlaGI[Enteroc    -------EN--------KQFKVLYLVPSIQLLTQTLRGWNNDT--ELKITSMAVTSDRDA
gi|494392045|ref|WP_007207894.1|helicase[Enterococcusitalicus]_gi|315483798|gb|EFU74284.1|helicaseCterminaldomainprotein[Enter/    -------EN--------KQLKVLYLVPSIQLLTQTLRGWNNDT--ELKITSMAVTSDRDA
gi|446350692|ref|WP_000428547.1|helicase[Bacilluscereus]_gi|228727580|gb|EEL78721.1|RestrictionmodificationsystemLlaBIII[Bacil/    -------AN--------KQFKVLYLVPSIQLLTQTLRGWNNDT--ELNIASMAVTSDRDA
gi|488255302|ref|WP_002326510.1|hypotheticalprotein[Enterococcusfaecium]_gi|430480276|gb|ELA57459.1|hypotheticalproteinOGE_0473    -------QA--------GQFKVLYLVPSIQLLTQTLRGWNNDT--EMIMTSMAVTSDRDA
gi|843253770|ref|WP_047916698.1|helicase[Lactococcuspiscium]_gi|802124677|emb|CEN29502.1|EndonucleaseandmethylaseLlaGI,TypeISPr    -------KD--------GPFKVLYLVPSIQLLTQTLRGWNNDT--ELTMTSMAVTSDRDA
LlaBIII/11578    -------KD--------GPFKVLYLVPSIQLLTQTLRGWNNDT--ELTMTSMAVTSDRDA
gi|499320531|ref|WP_011011023.1|helicase[Listeriainnocua]_gi|16415805|emb|CAC42019.1|pli0021[ListeriainnocuaClip11262]/11569    -------KA--------GPFKVLYLVPSIQLLTQTLRGWNNDT--ELTITSMAVTSDRDA
gi|760026859|ref|WP_043709973.1|helicase[Weissellacibaria]_gi|758337165|gb|KIU20061.1|UvsWhelicase[Weissellacibaria]/11567    -------QE--------TPFKVLYLVPSIQLLTQTLRGWNNDT--ELTMTSMAVTSDRDA
LlaGI/11570    -------KN--------GPFKVLYLVPSIQLLTQTLRGWNNDT--ELTITSMAVTSDRDA
gi|499988414|ref|WP_011669132.1|helicase[Lactococcuslactis]_gi|116109019|gb|ABJ74139.1|DNAorRNAhelicaseofsuperfamilyII[Lactococ    -------KN--------GPFKVLYLVPSIQLLTQTLRGWNNDT--ELTITSMAVTYDRDA
gi|685938360|ref|WP_031670319.1|helicase[Listeriamonocytogenes]_gi|590403387|gb|EXL23261.1|hypotheticalproteinX846_1956[Listeri    -------KA--------GPFKVLYLVPSIQLLTQTLRGWNNDT--ELTITSMAVTSDRDA
gi|530793219|gb|EQC56240.1|helicase[Lactococcuslactissubsp.cremorisTIFN5]_gi|530793247|gb|EQC56263.1|helicase[Lactococcuslactis    -------KN--------GPFKVLYLVPSIQLLTQTLRGWNNDT--ELTITSMAVTSDRDA
gi|695270006|ref|WP_032492075.1|hypotheticalprotein[Listeriamonocytogenes]_gi|695212872|ref|YP_009081522.1|hypotheticalproteinp    -------KA--------GPFKVLYLVPSIQLLTQTLRGWNNDT--ELTITSMAVTSDRDA
gi|504101310|ref|WP_014335297.1|helicase[Leuconostocmesenteroides]_gi|859247905|gb|AKO63885.1|helicase(plasmid)[Leuconostocmese    -------QS--------TPFKVLYLVPSIQLLTQTLRGWNNDT--ELTMTSMAVTSDRDA
gi|488905190|ref|WP_002816265.1|helicase[Leuconostocmesenteroides]_gi|227351522|gb|EEJ41777.1|helicaseCterminaldomainprotein[L/    -------QS--------TPFKVLYLVPSIQLLTQTLRGWNNDT--ELTMTSMAVTSDRDA
gi|518121285|ref|WP_019291493.1|MULTISPECIES:hypotheticalprotein[Lactococcus]/11588    -------SD-------NETFSILYLVPSIQLLTQTLRAWNNDT--DLSISSMAVTSDRNA
gi|754857213|ref|WP_042217902.1|helicase[Lactococcusgarvieae]/11549    -------SD-------KENYKVLYLVPSIQLLTQTLRAWNNDT--QMSMSSMAVTSDRNA
gi|640048455|ref|WP_024784762.1|helicase[Streptococcusmutans]/11564    -------SG-------QDQFVVLYLVPSIQLLTQTLRGWNNDT--EMTMSSMAVTSDRNA
gi|512695361|ref|WP_016476323.1|hypotheticalprotein[Veillonellasp.HPA0037]_gi|512079853|gb|EPD79018.1|hypotheticalproteinHMPREF    -------SS-------EDQYKILYLVPSIQLLTQTLRGWNSDT--DMTISSMAVTSDRNA
gi|636963514|ref|WP_024403747.1|helicase[Streptococcussuis]/11574    -------AG-------KEQYVVLYLVPSIQLLTQTLRGWNNDT--DMSMSSMAVTSDRNA
gi|881115312|ref|WP_048802949.1|helicase[Staphylococcuspasteuri]/11573    -------AN-------KEQFVVLYLVPSIQLLTQTLRAWNNDT--YMSISSMAVTSDRNA
gi|446063996|ref|WP_000141851.1|helicase[Streptococcusmitis]_gi|307618725|gb|EFN97864.1|endonucleaseandmethylaseLlaGI[Streptoco    -------AS-------KEQYTILYLVPSIQLLTQTLRGWNNDT--EMTMSSMAVTSDRNA
gi|488206609|ref|WP_002277817.1|restrictionmodificationsystemLlaBIII[Streptococcusmutans]_gi|449173033|gb|EMB75626.1|restricti/    -------AG-------KEQYVILYLVPSIQLLTQTLRGWNNDT--QMTMSSMAVTSDRNA
gi|640023290|ref|WP_024781621.1|helicase[Streptococcusmutans]/11566    -------AG-------KEQYVILYLVPSIQLLTQTLRGWNNDT--QMTMSSMAVTSDRNA
gi|814946581|ref|WP_046393781.1|helicase[Streptococcusuberis]_gi|810600885|gb|KKF57499.1|helicase[StreptococcusuberisB362]/1156    -------AN-------KDQFVVLYLVPSIQLLTQTLRGWNNDS--ELSISSMAVTSDRNA
gi|765470762|ref|WP_044720336.1|helicase[Streptococcussuis]/11559    -------AK-------KDQYVVLYLVPSIQLLTQTLRGWNNDT--DMTMSSMAVTSDRNA
gi|654507127|ref|WP_027976443.1|helicase[Streptococcusdevriesei]/11558    -------AG-------KEQFTVLYLVPSIQLLTQTLRGWNNDT--DMSMSSMAVTSDRNA
gi|530812902|gb|EQC72481.1|DNAorRNAhelicaseofsuperfamilyII[Streptococcussp.HSISS2]/11570    -------AE-------QEQYTVLYLVPSIQLLTQTLRGWNNDT--ELTMSSMAVTSDRNA
gi|489158846|ref|WP_003068504.1|helicase[Streptococcusintermedius]_gi|355367114|gb|EHG14827.1|hypotheticalproteinHMPREF9682_002    -------AG-------KEQYTVLYLVPSIQLLTQTLRGWNNDT--DMTMSSMAVTSDRNA
RM.Lru27782ORF13200P/11620    -------KG-------GSNYNILYLVPSIQLLSQTLFNWNSDVSDDINMISFSVVSDRKA
gi|492676546|ref|WP_005919236.1|DNAphosphorothioationsystemrestrictionenzyme[Pediococcusacidilactici]_gi|482517924|gb|EOA08103.    -------QK-------KGNFNVLYLVPSIQLLSQTLFNWNDDHAESIYMNSFSVVSDRKA
gi|498183116|ref|WP_010497272.1|helicase[Lactobacillusacidipiscis]/11563    -------SG-------IETYNVLYLVPSIQLLSQTLFNWNNDIAADIEMTSFSVVSDRKA
gi|822100485|ref|WP_046921865.1|helicase[Lactobacillusruminis]_gi|821684442|gb|KLA43321.1|restrictionmodificationsystemLlaBIII/    -------KG-------GNNYNVLYLVPSIQLLSQTLFNWNSDKSNEIEMVSFSVVSDKKA
gi|935565832|ref|WP_054450311.1|helicase[Lactobacilluskunkeei]/11573    -------QV-------KSKFNVLYLVPSIQLLSQTLFNWNDDVSEDIHMTSFSVVSDRKA
gi|752296887|ref|WP_041152901.1|helicase[Lactobacilluskunkeei]_gi|751663520|gb|KIM18358.1|helicase[Lactobacilluskunkeei]/11577    -------QV-------KSKFNVLYLVPSIQLLSQTLFNWNDDVSEDIHMTSFSVVSDRKA
gi|495745291|ref|WP_008469870.1|helicase[Lactobacillushominis]_gi|394484442|emb|CCI81207.1|RestrictionmodificationsystemLlaBII/    -------QK-------KKDFKVLYLVPSIQLLSQTLFNWNDDVSEDIHMTSLSVVSDRKA
gi|918672414|ref|WP_052543134.1|helicase[Lactobacillussp.]/11564    -------QK-------KKQFNVLYLVPSIQLLSQTLFSWNSDISDDIHMTSLSVVSDTKA
gi|799141631|ref|WP_045927261.1|helicase[Lactobacilluskullabergensis]_gi|797155335|gb|KJY59264.1|putativerestrictionendonucleas    -------QH-------KQEFNVLYLVPSIQLLSQTLFSWNDNVSDDIHMTSFSVVSDTKA
gi|504379990|ref|WP_014567092.1|helicase[Lactobacillusjohnsonii]_gi|329666726|gb|AEB92674.1|putativerestrictionendonuclease[Lac    -------QG-------KKQFNVLYLVPSIQLLSQTLFGWNADVSEDIHMTSLSVVSDTKA
gi|489880179|ref|WP_003783646.1|DNAdamageinducibleprotein[Actinomycesnaeslundii]_gi|399901561|gb|EJN84440.1|typeIIIrestriction/    -------GG-----------RVLFLVPSIALLSQTLMEWSAESE--VPLRSFAVCSDTKV
gi|496524497|ref|WP_009232780.1|DNAdamageinducibleprotein[Actinomycessp.oraltaxon849]_gi|365265413|gb|EHM95177.1|hypotheticalp/    -------GG-----------RVLFLVPSIALLSQTLMEWSAESE--VPLRSFAVCSDAKV
gi|915397782|ref|WP_050783167.1|damageinducibleprotein[Actinomycesoris]/11694    -------GG-----------RVLFLVPSIALLSQTLMEWSAESE--VPLRSFAVCSDAKV
gi|502925248|ref|WP_013160224.1|helicase[Propionibacteriumfreudenreichii]_gi|296921272|emb|CBL55824.1|helicase[Propionibacteriu    ---NG-GQL-----------KVLFLAPSISLVSQTLREWMAQTQ--TDIRPFVVCSDTKA
gi|732164352|emb|CEG87481.1|helicase[Propionibacteriumfreudenreichii]/11618    ---AG-GHL-----------KVLFLAPSISLVSQTLREWMGQSQ--TLIRPFVVCSDTKA
gi|739047402|ref|WP_036925058.1|damageinducibleprotein[Propionibacteriumthoenii]/11596    ---NE-GHL-----------TVLFLAPSISLVSQSLREWLAQSQ--TPIRPLVVCSDTKA
gi|739112839|ref|WP_036982533.1|damageinducibleprotein[Propionibacteriumjensenii]/11594    ---NE-GHL-----------TVLFLAPSISLVSQSLREWLAQSQ--TPIRPFVVCSDTKA
gi|918023558|ref|WP_052314121.1|damageinducibleprotein[Nocardiathailandica]/11612    -------GG-----------RVLFLVPSIQLLSQTLREWMAQSE--VDIRPFAVCSDVRV
gi|498380271|ref|WP_010694427.1|DNAdamageinducibleprotein[Saccharopolysporaspinosa]/11605    -------GG-----------SVLFLVPSIQLLSQTLREWMANSE--VDIRPFAVCSDVRV
gi|749991459|ref|WP_040339809.1|damageinducibleprotein[CandidatusBlastococcusmassiliensis]/11597    -------GG-----------TVLFLVPSIQLLSQSLREWMANAE--VDIAPYAVCSDVRV
gi|754177801|ref|WP_041796231.1|damageinducibleprotein[Modestobactermarinus]/11592    -------GG-----------TVLFLVPSIQLLSQSLREWMANAE--VDIRALAVCSDVRV
gi|504188248|ref|WP_014375350.1|DNAdamageinducibleprotein[Blastococcussaxobsidens]_gi|378782790|emb|CCG02456.1|putativeHelicas/    -------GR-----------NVLFLVPSIQLLSQSLREWMANAE--VDIRAMAVCSDVRV
gi|946476713|gb|KRF26798.1|damageinducibleprotein[Phycicoccussp.Soil803]/11609    ---NKAKAT-----------TVLFLVPSIQLVNQTFREWAQETE--LPFRAFAVCSDVKV
gi|700176672|gb|KGN37509.1|DNAdamageinducibleprotein[KnoelliasubterraneaKCTC19937]/11633    ---SKAKAT-----------TVLFLVPSIQLVNQTFREWAQETE--LPFRAFAVCSDVKV
gi|700168841|gb|KGN29914.1|hypotheticalproteinN802_10215[KnoelliasinensisKCTC19936]/11631    ---NKAKAT-----------TVLFLVPSIQLVNQTFREWAQETE--LPFRAFAVCSDAKV
gi|946426241|gb|KRE76610.1|damageinducibleprotein[Arthrobactersp.Soil763]/11606    -------GG-----------SVLFLVPSISLLSQTLREWAVESE--TRLHPLAVCSDRKS
gi|517318525|ref|WP_018500927.1|hypotheticalprotein[Frankiasp.BCU110501]/11592    -------GG-----------KVLFLVPSINLLSQTVREWIDNSE--LQIRALAVCSDAKS
gi|499072429|ref|WP_010851327.1|Helicase[Tetrasphaeraelongata]_gi|478756579|emb|CCH71501.1|putativeHelicase[Tetrasphaeraelongat    -------GG-----------SVLFLVPSISLLSQTLREWATEAE--VPLASLAVCSDRKA
gi|750080666|ref|WP_040385350.1|damageinducibleprotein[Demetriaterragena]/11625    -------GK-----------SVLFLVPSISLMSQTLTEWAAEAH--APMTALAVCSDRKV
gi|752558292|ref|WP_041229523.1|damageinducibleprotein[Dermacoccusnishinomiyaensis]_gi|664689315|gb|AIF41904.1|damageinducibl/1    -------GG-----------SVLFLVPSISLLSQTVREWATESE--VSLRMLAVCSDAKS
```

```
gi|829089213|ref|WP_047310701.1|damageinducibleprotein[Dermacoccussp.PE3]_gi|828384812|gb|KLO63770.1|damageinducibleprotein[D/1    -------GG-----------SVLFLVPSISLLSQTVREWAANAE--VPLDMIAICSDPKS
gi|748756635|ref|WP_040014750.1|damageinducibleprotein[Dermacoccussp.Ellin185]/11596                                                -------GG-----------SVLFLVPSISLLSQTVREWSANKL--IPQDAIAICSDPKS
gi|916534724|ref|WP_051141848.1|damageinducibleprotein[Actinomycesneuii]/11632                                                       ----NGGTA-----------KVLFMVPSLALMTQTLSEWSAECS--EMLHSWAVCSDTKV
gi|921140030|ref|WP_053090276.1|damageinducibleprotein[Rothiamucilaginosa]/11616                                                     ----NGGHA-----------RVLFMVPSLALMSQSISEFSNEIE--GPFHAYAVCSDSKV
gi|896406751|ref|WP_049343749.1|damageinducibleprotein[Rothiamucilaginosa]/11647                                                     ----NGGHA-----------RVLFMVPSLALMSQSISEFSNEIE--GPFHAYAVCSDSKV
gi|723608234|gb|AIY01610.1|putativehelicase[Arthrobactersp.PAMC25486]/11613                                                          ----L-GHA-----------WILVMVPSLALISQTLKEWSDEAD--QPMQAYAVCSDTKV
gi|946394527|gb|KRE45160.1|damageinducibleprotein[Arthrobactersp.Soil736]/11616                                                      ----E-GHA-----------RILFLVPSLALMSQSLKEWSDETE--QPMHAYAVCSDVKV
gi|515765304|ref|WP_017197904.1|hypotheticalprotein[Arthrobactersp.M2012083]/11648                                                   ----E-SHV-----------RVLFLVPSLALMSQSLKEWSDETT--QTMHAYAVCSDTKV
gi|945315587|gb|KQT73127.1|damageinducibleprotein[Microbacteriumsp.Leaf436]/11612                                                    -------GG-----------TVLFLMPSIALLSQTLKEWTIQAE--VPMRSFAVCSDVSV
gi|759908497|ref|WP_043594599.1|damageinducibleprotein[Frigoribacteriumsp.MEB024]_gi|758271359|gb|KIU03346.1|damageinduciblep/1      -------GG-----------KVLFLVPSIALLSQTLREWSQERR--MDLRAFAICSDTKV
gi|945072850|gb|KQR34237.1|hypotheticalproteinASF75_00055[Curtobacteriumsp.Leaf154]/11597                                            -------GG-----------KVLFLVPSIALLSQTLREWSQERA--MPLRAFAVCSDSKV
gi|551263152|ref|WP_022883363.1|DNAdamageinducibleprotein[Glaciibactersuperstes]/11621                                               -------GG-----------SVLFLVPSIALLSQTLKEWAAERN--MPLRAFAICSDSKV
gi|945452341|gb|KQV08491.1|hypotheticalproteinASC63_10170[Leifsoniasp.Root112D2]/11623                                               -------GG-----------SVLFLVPSIALLSQTLREWAAQQE--IPQRAFAICSDTKV
gi|742759483|gb|KIC69912.1|damageinducibleprotein[Kocuriarhizophila]/11610                                                           -------NA-----------TVLFLVPSISLLSQTLTEWKRDAS--EEFRAFAVCSDVKV
gi|737919995|ref|WP_035885304.1|damageinducibleprotein[Kocuriarhizophila]/11611                                                      -------NA-----------TVLFLVPSISLLSQTLTEWKRDAS--EEFRAFAVCSDVKV
gi|737997701|ref|WP_035959325.1|damageinducibleprotein[Kocuriamarina]_gi|725823884|gb|KHE75603.1|damageinducibleprotein[Kocur/1      -------DA-----------TVLFLVPSISLLSQTLTEWKRDAS--EEFRAFAVCSDVKV
gi|664521070|ref|WP_031037650.1|damageinducibleprotein[Streptomycessp.NRRLWC3774]/11624                                              --AGEGEHT-----------SVLFLVPSIALLSQTLREWSFEAE--VSLRAFAVCSDTKV
gi|500101034|ref|WP_011777041.1|DNAdamageinducibleprotein[Arthrobacteraurescens]_gi|119951547|gb|ABM10457.1|putativeHelicase[A/     -------GG-----------SVLFLVPSISLLQQTLTEWTAESE--VPLRPLAVCSDTKV
gi|916871953|ref|WP_051479009.1|damageinducibleprotein[Arthrobactersp.H5]/11588                                                      -------GG-----------SVLFLVPSISLLQQTLSEWTAEAE--MPLRPLAVCSDTKV
gi|612084649|gb|EZP43369.1|TypeIIIrestrictionenzyme,ressubunit[Micrococcusluteus]/11625                                              -------GG-----------SVLFLVPSIALLQQTLNEWTANAD--VPLRPFAVCSDASV
gi|759739708|ref|WP_043450422.1|damageinducibleprotein[Arthrobacterphenanthrenivorans]_gi|742758264|gb|KIC68716.1|damageinduc/1      -------GG-----------SVLFLVPSIALLQQTLNEWTAQAT--VPLRPLAVCSDSKV
gi|636845448|ref|WP_024366823.1|damageinducibleprotein[Arthrobactersp.TB26]/11605                                                    -------GG-----------TALFLVPSIALLQQTLNEWTAQAT--VPLRPLAVCSDTKV
gi|500009025|ref|WP_011689743.1|DNAdamageinducibleprotein[Arthrobactersp.FB24]_gi|116613051|gb|ABK05760.1|typeIIIrestrictionen/     -------GG-----------TALFLVPSIALLQQTLNEWTAQAT--VPLRPLAVCSDTKV
gi|821249847|gb|KKZ12083.1|hypotheticalproteinTQ37_06090[CandidatusSynechococcusspongiarum15L]/11436                                 ------AGG-----------RVLVLAPSLALLSQTLRAWLDDAC--LPIRAFAVCSDNQT
gi|739446902|ref|WP_037306885.1|damageinducibleprotein[Ruegeriahalocynthiae]/11592                                                   ------KGK-----------RVLFLVPSLALIAQTVREWTFDTE--TELRSFAVCSDAQV
gi|640620081|ref|WP_025048131.1|damageinducibleprotein[Sulfitobactermediterraneus]_gi|751956650|gb|KIN77213.1|TypeIIIrestricti/     ------KGK-----------RVLFLVPSLALIAQTVREWTYDTE--LELRSFAVCSDAQV
gi|740247634|ref|WP_038088583.1|damageinducibleprotein[Thiobacillusprosperus]_gi|684049748|gb|KFZ90853.1|putativehelicase[Thio/     ------DTG-----------LTLFLAPSITLVSQTLREWSAEAE--QPFDAFVVCSDSRV
gi|518420321|ref|WP_019590528.1|hypotheticalprotein[Thioalkalivibriosp.ALE20]/11603                                                  ------EGG-----------RILFLAPSISLVGQTLREWTAQAH--APLRALVACSDTKV
gi|500156826|ref|WP_011831496.1|DNAdamageinducibleprotein[Methylibiumpetroleiphilum]_gi|124261892|gb|ABM96881.1|adeninespecifi/     ------KAG-----------RILFLAPSISLVSQTLREWTAQAQ--EPFHAFVVCSDSKV
gi|769978537|ref|WP_045094522.1|damageinducibleprotein[Legionellafallonii]_gi|698841736|emb|CEG55682.1|Putativeproteinwithboxh/      ------TAG-----------RVLFLAPSISLVSQSLREWTAEAI--KPIHAFVVCSDTKV
gi|553752541|ref|WP_023085344.1|hypotheticalprotein[Pseudomonasaeruginosa]_gi|552201952|gb|ERU62653.1|hypotheticalproteinQ088_0     ------ENG-----------LILFLAPSISLVGQSLREWSAQAE--KPFHAFVVCSDSKV
gi|880980418|ref|WP_048704461.1|damageinducibleprotein[Thauerahumireducens]_gi|875132785|gb|KMU70944.1|damageinducibleprotein/1      ------ATG-----------RVLFLAPSISLVGQTLREWSSEAI--DPFHAFVVCSDTKV
gi|740135732|ref|WP_037982815.1|damageinducibleprotein[Thauerasp.27]/11603                                                           ------APG-----------RVLFLAPSISLVGQTLREWSSEAI--DPFHAFVVCSDTKV
gi|738017371|ref|WP_035978332.1|damageinducibleprotein[Burkholderiaglumae]/11380                                                     ------HNG-----------RVLFLAPSISLVSQSMREWTAQAS--EPIHAFVVCSDSKV
gi|498506003|ref|WP_010806671.1|typeIIIrestrictionenzyme,ressubunit[Pandoraeasp.SD62]_gi|490314561|gb|EON12879.1|typeIIIrestri/     ------VNG-----------RVLFLAPSISLVSQSMREWTAQAA--DPIHAFVVCSDSKV
gi|500117748|ref|WP_011793753.1|DNAdamageinducibleprotein[Acidovoraxcitrulli]_gi|120587742|gb|ABM31182.1|typeIIIrestrictionenz/     ------ADG-----------RVLFLAPSISLVAQSLREWTAESL--EPFHAFVVCSDSKV
gi|651617328|ref|WP_026611444.1|damageinducibleprotein[Methylocaldumszegediense]/11601                                               ------AGG-----------KVLFLAPSISLVAQTLREWSAESH--TPFHAFVVCSDSKV
gi|737210208|ref|WP_035194994.1|damageinducibleprotein[Acidithiobacillusferrivorans]_gi|669953220|emb|CDQ11755.1|Adeninespecif/     ------PGG-----------HVLFLVPSLALLAQTLREWTAEAH--APLRCYAVCSDSKV
gi|935697386|ref|WP_054506180.1|damageinducibleprotein[Achromobactersp.]_gi|928716153|emb|CUK12836.1|Hefnuclease[Achromobacter/     ------PGQ-----------NVLFLVPSIALLSQTLRAWTADAS--VPLRCFAVCSDSAA
gi|429502517|gb|ELA00827.1|adeninespecificDNAmethyltransferase[Cupriavidussp.HMR1]/11599                                             ------PGQ-----------NVLFLVPSIALLSQTLRAWTADTS--VPLRCFAVCSDSAA
gi|506356642|ref|WP_015876361.1|DNAdamageinducibleprotein[Burkholderiaglumae]_gi|237877158|gb|ACR29491.1|AdeninespecificDNAmet/     ------PGQ-----------NVLFLVPSIALLSQTLRAWTSDSS--VPLRCFAVCSDSKA
gi|696406787|ref|WP_032978003.1|damageinducibleprotein[Bordetellahinzii]_gi|625335076|gb|KCB49343.1|typeIIIrestrictionenzyme,r/     ------PGQ-----------NILFLVPSIALLSQTLRAWTADAS--VPLRCFAVCSDSKA
gi|817529125|gb|KKO72587.1|damageinducibleprotein[Kerstersiagyiorum]/11592                                                          ------PGQ-----------NILFLVPSIALLSQTLRAWTADAS--VPLRCFAVCSDSKA
gi|737048910|ref|WP_035044118.1|damageinducibleprotein[Aquabacteriumsp.NJ1]_gi|699052896|gb|KGM38706.1|damageinducibleprotein/1     ------PGQ-----------YVLFLVPSIALLSQTLRAWTADAA--VPLRCFAVCSDSKA
gi|822492519|ref|WP_046933319.1|MULTISPECIES:damageinducibleprotein[Xanthomonas]_gi|821703525|gb|KLA61196.1|damageinduciblepr/1     ------PGQ-----------TILFLVPSIALLSQTLRAWTADAS--VPLRCFAVCSDSKA
gi|751252861|ref|WP_040963740.1|damageinducibleprotein[Xanthomonasfuscans]_gi|749808363|gb|KIJ03887.1|damageinducibleprotein[/1     ------PGQ-----------TILFLVPSIALLSQTLRAWTADAS--VPLRCFAVCSDSKA
gi|496774980|ref|WP_009368792.1|DNAdamageinducibleprotein[Bilophilasp.4_1_30]_gi|345039902|gb|EGW44205.1|hypotheticalproteinHM/     ------PGG-----------TVLFLVPSISLLSQTLRAWTADAS--VPLRCFAVCSDSKA
gi|499241912|ref|WP_010939452.1|DNAdamageinducibleprotein[Desulfovibriovulgaris]_gi|46580580|ref|YP_011388.1|adeninespecificDN/     ------PGG-----------TVLFMVPSIALLSQTLRAWTADST--KPLRCFAVCSDSKV
gi|504274913|ref|WP_014462015.1|DNAdamageinducibleprotein[Bacillusmegaterium]_gi|345446919|gb|AEN91936.1|AdeninespecificDNAmet/     ---NEEKPT-----------KILYLVPSISLLSQTLREWNNDSE--ISFNTFAVCSDKKV
gi|504895966|ref|WP_015083068.1|typeIIrestrictionenzymeandmethyltransferasesubtypeG[Anabaenasp.90]_gi|413972424|gb|AFW96512.1|t     ------ENN-----------LILFLVPSISLLSQTLREWTAESD--INFHSIAVCSDVNV
gi|518327765|ref|WP_019497972.1|hypotheticalprotein[Calothrixsp.PCC7103]/11600                                                      ------KQA-----------QVLFLVPSISLLSQTLREWTAEAE--LSLHSIAVCSDTKV
gi|652765503|ref|WP_027079917.1|damageinducibleprotein[Luteimonasmephitis]/11593                                                     ------KDG-----------LVLFLVPSISLLSQTLKEWTAQAA--MDFRSFAVCSDTKV
gi|896539951|ref|WP_049442123.1|damageinducibleprotein[Stenotrophomonasmaltophilia]/11591                                            ------SNG-----------LVLFLVPSISLLSQTLKEWTAQAT--DEIRAFAVCSDTKV
gi|500217900|ref|WP_011888043.1|DNAdamageinducibleprotein[Geobacillusthermodenitrificans]_gi|134267954|gb|ABO68149.1|Conserved/     ------RGG-----------LVLFLVPSIALLSQTLREWTAEAE--IPLRAFAVCSDTKV
gi|696570298|ref|WP_033102014.1|damageinducibleprotein[Thermoactinomycesdaqus]/11594                                                 ------SGG-----------LVLFLVPSIALLSQTLREWTAEAE--IPLRSFAVCSDKKV
gi|251772067|gb|EES52637.1|putativehelicase[Leptospirillumferrodiazotrophum]/11618                                                   ------DGG-----------LILYLVPSIALVSQTLSEWLRESE--GLLHSFVVCSDTTV
gi|738124101|ref|WP_036082108.1|damageinducibleprotein[Leptospirillumferriphilum]_gi|685243306|gb|KGA94177.1|putativehelicase[/     ------ENG-----------LILYLVPSIALVSQTLSEWVRESN--GLLHSFVVCSDTTV
gi|558699223|ref|WP_023524766.1|helicase[Leptospirillumsp.GroupII'CF1']_gi|206603764|gb|EDZ40244.1|Putativehelicase[Leptospiri/     ------ENG-----------LILYLVPSIALVSQTLSEWTRESE--GFLHSFVVCSDTTV
gi|406774462|gb|AFS53887.1|putativehelicase[LeptospirillumferriphilumML04]/11610                                                     ------QGG-----------AILFLSPSISLVSQSLTEWTREAE--APFRAFAVCSDSKV
gi|740719463|ref|WP_038504749.1|damageinducibleprotein[Leptospirillumferriphilum]_gi|640838125|gb|AIA30173.1|damageinduciblep/1     ------TEG-----------TILFLSPSISLVSQSLTEWTREAE--APFRAFAVCSDSKV
gi|517810766|ref|WP_018980974.1|hypotheticalprotein[Saccharibacterfloricola]/11733                                                   ------AGK-----------TVLFLAPSLGLVQQTLDAWTNKAS--TAITPHVVCSDSDI
gi|502471675|ref|WP_012798533.1|helicase[Slackiaheliotrinireducens]_gi|256791761|gb|ACV22431.1|predictedhelicase[Slackiaheliotr     ------KGG-----------FILFLAPSISLVGQSMRAWINQSKL--PMRVAVVCSDEKA
gi|657871713|ref|WP_029576297.1|hypotheticalprotein[Bifidobacteriumthermacidophilum]_gi|673007521|gb|KFJ02535.1|helicase[Bifido     ------MGG-----------TVLFLAPSISLISQTMRAWVNQIRDNEKINLYVVCSDPKA
gi|673004757|gb|KFI99791.1|DNAhelicaserestrictionenzymetypeIIIRessubunit[Bifidobacteriumthermacidophilumsubsp.porcinum]/11682       ------MGG-----------TVLFLAPSISLISQTMRAWVNQIRDNEKINLYVVCSDPKA
gi|917316092|ref|WP_051922804.1|hypotheticalprotein[Bifidobacteriumstellenboschense]_gi|673003774|gb|KFI98812.1|helicase[Bifido     ------EGG-----------TVLFLAPSISLVSQSMRDWVNQTRC--RINVYVVCSDGKA
gi|705415694|ref|WP_033502332.1|hypotheticalprotein[Bifidobacteriumpseudolongum]_gi|672982411|gb|KFI77685.1|DNAdamageinducible/     ------TGG-----------TVLFLAPSISLVSQSMRDWVNQTRG--RINVYVVCSDAKA
gi|916591418|ref|WP_051198509.1|hypotheticalprotein[Bifidobacteriumsp.AGR2158]/11704                                                 ------MGG-----------TVLFLAPSISLVSQTMRNWVNQVRG--RINLYVCCSSPKA
```

```
gi|772242982|gb|AJW78255.1|hypotheticalproteinVO01_03165[Clavibactermichiganensissubsp.insidiosus]/11642    ---HSSGSS-----------SVLFMVPSLALLQQSLAEWSAEHDPELGFRAFVVGSDMNI
gi|916649818|ref|WP_051256909.1|damageinducibleprotein[Actinomycesgraevenitzii]/11677    ----EGGSA-----------RILFAVPSLALLKQTLDDWSVEVDSA--FTAWAVCSDTKV
gi|496504578|ref|WP_009212861.1|helicase[Actinomycessp.oraltaxon180]_gi|315312695|gb|EFU60776.1|helicase[Actinomycessp.oraltaxo    ----EGGTA-----------RILFAVPSLALLKQTLDDWAAEADGA--FTAWAVCSDTKV
gi|494862206|ref|WP_007588306.1|helicase[Actinomycessp.ICM39]_gi|398362799|gb|EJN46470.1|typeIIIrestrictionenzyme,ressubunit[Ac    ----EGGTA-----------RILFAVPSLALLKQTLDDWAAEADGA--FTAWAVCSDTKV
gi|881057111|ref|WP_048774974.1|damageinducibleprotein[Sanguibacterkeddieii]/11697    ----EGGTA-----------RILFAVPSLALLKQTLDDWAAEADGA--FTAWAVCSDTKV
gi|881020459|ref|WP_048740808.1|damageinducibleprotein[Kytococcussedentarius]/11704    ----EGGNA-----------RILFAVPSLALLKQTLDDWAAEADGA--FTAWAVCSDTKV
gi|881052313|ref|WP_048770555.1|damageinducibleprotein[Cellulomonasflavigena]/11705    ----EGGTA-----------RILFAVPSLALLKQTLDDWAAEADGA--FTAWAVCSDTKV
gi|490108682|ref|WP_004009424.1|helicase[Mobiluncuscurtisii]_gi|315490465|gb|EFU80089.1|helicaseCterminaldomainprotein[Mobilun/    ----LGGNA-----------RIMFMVPSLALMSQTLGEWAAEVQVP--FSAWSVCSDAKV
gi|490111935|ref|WP_004012603.1|helicase[Mobiluncusmulieris]_gi|227843812|gb|EEJ53982.1|helicaseCterminaldomainprotein[Mobilun/    ---GDSAGA-----------RILFMVPSLALMSQTMHEWAAEVSVP--FTAWSVCSDTKV
gi|490114228|ref|WP_004014848.1|helicase[Mobiluncusmulieris]_gi|269934432|gb|EEZ90993.1|helicaseCterminaldomainprotein[Mobilun/    ---GDSAGA-----------RILFMVPSLALMSQTMHEWAAEVSVP--FTAWSVCSDTKV
gi|493333551|ref|WP_006290636.1|helicase[Parascardoviadenticolens]_gi|315119487|gb|EFT82620.1|helicaseCterminaldomainprotein[P/    -------GG-----------LILFAAPSIALVSQAMREWTNQAR--CKMRALVVCSDAKA
gi|495817067|ref|WP_008541646.1|helicase,partial[Sutterellaparvirubra]_gi|378598583|gb|EHY31712.1|helicaseprotein,partial[Sutte    ------EGG-----------FVMFLVPSLSLLSQTLSDWKQQCE--FEINAFAVCSDTTT
gi|491572696|ref|WP_005430278.1|helicase[Sutterellawadsworthensis]_gi|319805116|gb|EFW01943.1|DNAhelicaserestrictionenzymeTypeI    ------NNG-----------FVMFLVPSLALLSQTLTDWKRQCA--VPIHAFAVCSDATT
gi|748686321|ref|WP_039944445.1|hypotheticalprotein[Thermicanusaegyptius]/11573    ------QGG-----------HVLFLAPSIALVEQTLRAWLVDAD--IPLRVFAVTSDRTV
gi|916875698|ref|WP_051482754.1|hypotheticalprotein[Thioalkalivibriosp.HK1]/11366    ------IGA-----------RVLYLVPSISLMQQSMRAFAEQKS--IEHRYIGICSDTKA
gi|821248604|gb|KKZ10960.1|hypotheticalproteinTE42_09075[CandidatusSynechococcusspongiarumSP3]/11598    ------PGG-----------RVLVLVPSLALLSQTLRAWLDDAR--LPVRAFAVCSDNQT
gi|737409843|ref|WP_035390956.1|hypotheticalprotein[Ferrimicrobiumacidiphilum]_gi|765544266|gb|KJE76241.1|typeIrestrictionenzym    ------RGG-----------HVLFLVPSIALLSQALKEWTRESE--IPLQCLAVCSDTEV
gi|654906925|ref|WP_028358281.1|damageinducibleprotein[Brackiellaoedipodis]/11679    ------PGK-----------RVLFMVPSLALLAQTVTEWTQQSK--VPLHNYAVCSDSEV
gi|502809901|ref|WP_013044877.1|hypotheticalprotein[CandidatusPuniceispirillummarinum]_gi|292663146|gb|ADE38247.1|hypotheticalp    ------GTG-----------HVLFMVPSISLLAQSLHEWSFQRR--KDHRYIAVCSDTKV
gi|251771370|gb|EES51950.1|typeIIIrestrictionenzyme,ressubunit[Leptospirillumferrodiazotrophum]/11628    ------RGG-----------TVLFAVPSITLLSQTLREWARESL--VPMRFFAVCSDTRI
gi|498328480|ref|WP_010642636.1|DNAdamageinducibleprotein[Acidithiobacillusthiooxidans]/11627    ------EDG-----------LTLFLAPSITLVSQTLREWADQAQ--KPFDAFVVCSDSKV
gi|647633864|ref|WP_025906698.1|damageinducibleprotein[Prochlorococcussp.scB245a_519B7]/11622    ------VGN-----------KILYLVPSLALMSQTIRDWKNDSK--NDFIAFSVCSDAKV
gi|647619599|ref|WP_025893729.1|damageinducibleprotein[Prochlorococcussp.scB241_527N11]/11621    ------ANK-----------MVLYMVPSLALMSQTVREWKNDAH--EDFLAFSVCSDQKV
gi|755034887|ref|WP_042387498.1|hypotheticalprotein[Clostridiumsp.CL6]/11606    ------GKG-----------TVLFLVPSIALINQTMSEWTAQCN--YTYDALVVCSDNKA
gi|498139798|ref|WP_010453954.1|helicase[SuccinivibrionaceaebacteriumWG1]/11662    ------TKG-----------LVLFLVPSLALMNQSLVAWKADAI--NDFNAVCVCSDRNV
gi|527501386|gb|EPT34571.1|typeIIIrestrictionenzyme,ressubunit[Bacteroidetesbacteriumoraltaxon272str.F0290]/11789    ------KKG-----------LVLFLVPSIALLGQGLNNWMTDTS--AVIKPICICSDSKA
gi|496047275|ref|WP_008771782.1|helicase[Bacteroidessp.2_1_33B]_gi|262296443|gb|EEY84373.1|helicaseCterminaldomainprotein[Bact/    ------GKG-----------LILFMVPSIALLGQSLNAWMTDTK--YRMKAVCICSDSKA
PDI8503III/11664    ------GKG-----------LILFMVPSIALLGQSLNAWMTDTK--YRMKAVCICSDSKA
gi|738719431|ref|WP_036613715.1|helicase[Parabacteroidesdistasonis]_gi|649542518|gb|KDS49387.1|restrictionendonucleasefamilypro    ------GKG-----------LILFMVPSIALLGQSLNAWMTDTK--YRMKAVCICSDSKA
gi|659417033|gb|KEJ86133.1|hypotheticalproteinHMPREF1002_00341[Porphyromonassp.31_2]/11656    ------GKG-----------LILFMVPSIALLGQSLNAWMTDTK--YRMKAVCICSDSKA
gi|567456807|gb|ETK11358.1|hypotheticalproteinT235_15845[Tannerellasp.oraltaxonBU063isolateCell8/11]/11433    ------GKG-----------LVLFLVPSIALLGQSLNDWMVDAA--YPIKAICICSDSRA
gi|545440279|ref|WP_021677877.1|helicase[Porphyromonasgingivalis]_gi|543985765|gb|ERJ89108.1|helicaseprotein[Porphyromonasgingi    ------DKG-----------LVLFLVPSIALLGQSLNDWMGDAD--HPIKAVCICSDGRA
gi|497438056|ref|WP_009752254.1|helicase[Prevotellasp.oraltaxon473]_gi|429144866|gb|EKX87968.1|helicaseprotein[Prevotellasp.ora    ------GKG-----------LVLFLVPSIALLGQSLNDWMGDAE--TPIKAICICSDSRA
gi|490423474|ref|WP_004295683.1|MULTISPECIES:helicase[Bacteroides]_gi|156110098|gb|EDO11843.1|helicaseCterminaldomainprotein[B/    ------NKG-----------LVLFMVPSISLLGQSLNAWCADAV--NPIKGICICSDSRA
gi|941777229|ref|WP_055173506.1|helicase[Bacteroidescaccae]_gi|933263395|emb|CUQ00567.1|Predictedhelicase[Bacteroidescaccae]/1/    ------NKG-----------LVLFMVPSIALLGQSLNAWSADAK--KPIKAVCICSDSKA
gi|492413898|ref|WP_005836892.1|helicase[Bacteroidesuniformis]_gi|392678824|gb|EIY72229.1|hypotheticalproteinHMPREF1072_03583[B    ------NNG-----------LVLFMVPSIALLGQSLNAWSADAK--KPIKAVCICSDSKA
gi|695455095|ref|WP_032566191.1|helicase[Bacteroidesfragilis]_gi|596095426|gb|EXZ59996.1|restrictionendonucleasefamilyprotein[B    ------NKG-----------LVLFMVPSIALLGQSLNAWSADAK--NPIKAVCICSDSKA
gi|933227941|emb|CUQ02348.1|Predictedhelicase[Bacteroidesuniformis]/11658    ------NKG-----------LVLFMVPSIALLGQSLNAWAADAK--KPIKAVCICSDSKA
gi|494823013|ref|WP_007558421.1|helicase[Bacteroidesplebeius]_gi|198272167|gb|EDY96436.1|helicaseCterminaldomainprotein[Bacter/    ------NKG-----------LVLFMVPSIALLGQSLNAWSADAK--KPIKAVCICSDSKA
gi|495427212|ref|WP_008151909.1|helicase[Parabacteroidesjohnsonii]_gi|218222381|gb|EEC95031.1|helicaseCterminaldomainprotein[P/    ------GKG-----------LVLFMVPSIALLGQSLNAWSADAK--KPIKAVCICSDSKA
gi|933196855|emb|CUO94545.1|typeIrestrictionenzymeEcoKIsubunitR[Bacteroidesuniformis]_gi|933220098|emb|CUN86874.1|typeIrestrict    ------GKG-----------LVLFMVPSIALLGQSLNAWSADAG--KTIKAVCICSDSKA
gi|823687748|ref|WP_047101121.1|helicase[Brachyspirahyodysenteriae]_gi|823334537|gb|KLI17932.1|helicase[Brachyspirahyodysenteri    ------NNG-----------LVLFLVPSIALLGQTLNEWHSQSE--KPIYSICICSDSKV
gi|496436691|ref|WP_009145538.1|helicase[Phascolarctobacteriumsuccinatutens]_gi|322414007|gb|EFY04839.1|helicaseCterminaldomai/    ------NKG-----------LILVLVPSIALINQTLNEWQSCAK--HDIYPICVCSDSTA
gi|503372880|ref|WP_013607541.1|helicase[Sphaerochaetaglobosa]_gi|324026933|gb|ADY13692.1|typeIIIrestrictionproteinressubunit[S    ------GSG-----------FVLFLVPSIALLGQTLKEWASQSD--NPLHAICVCSDPKV
gi|505821748|ref|WP_015707145.1|helicase[Treponemaprimitia]_gi|299483503|gb|ADJ19584.1|helicasedomaincontainingprotein[Trepone/    ------GEG-----------FALFLVPSIALLGQTLREWSAQAQ--EPLYPICICSDAQV
gi|505822741|ref|WP_015708134.1|helicase[Treponemaprimitia]_gi|333739639|gb|AEF85129.1|endonucleaseandmethylaseLlaGI[Treponemap    ------GKG-----------FVLFLVPSIALLGQTLREWSAQAQ--EPIYPICICSDAQV
gi|488791994|ref|WP_002704400.1|helicase[Treponemasaccharophilum]_gi|380767691|gb|EIC01689.1|typeIIIrestrictionproteinressubuni    ------DSH-----------LVLFLVPSIALLSQTLKEWSAQCK--QNIYPICICSDAKA
gi|524671454|emb|CDD81750.1|putativeseptumsitedeterminingproteinMinC[Bacteroidessp.CAG:462]/11642    ------SNG-----------FVLFLVPSIALLGQTLRAWSAQAD--IPIHAVCICSDGQV
gi|524573865|emb|CDC95766.1|putativeseptumsitedeterminingproteinMinC[Alistipessp.CAG:268]/11639    ------GRG-----------LVLFLVPSIALLGQTLRSWLQQAM--EPMMAVCICSDPQV
gi|658617970|ref|WP_029759328.1|helicase[Fusobacteriumnucleatum]/11606    ------KNG-----------FILFLVPSIALLGQTLREWTNDIDKDTKLYPICICSDSKI
gi|494412037|ref|WP_007216782.1|helicase[Bacteroidescellulosilyticus]_gi|392638921|gb|EIY32752.1|hypotheticalproteinHMPREF1062_    ------GRG-----------LVLFLVPSIALLGQTLREWTADAS--EAIQAVCICSDAEV
gi|383107102|gb|AFG37435.1|putativehelicase[SpirochaetaafricanaDSM8902]/11616    ------GSG-----------LVLFLVPSIALLGQTLNEWTADAQ--EPIKPICICSDARV
SAF8902III/11616    ------GSG-----------LVLFLVPSIALLGQTLNEWTADAQ--EPIKPICICSDARV
gi|652538894|ref|WP_026932796.1|helicase[Gramellaechinicola]/11633    ------GEG-----------LVLFLVPSIALLGQTLSEWSANSE--YAINPICICSDSRI
gi|639238223|ref|WP_024568839.1|helicase[Elizabethkingiaanophelis]/11628    ------GKG-----------LVLFLVPSIALLGQTLREWTANAL--EPITSICICSDTKI
gi|652520381|ref|WP_026914582.1|helicase[Gramellaportivictoriae]/11623    ------NKG-----------LVLFLVPSIALLGQTLREWSSDAE--KPIKPIAICSDSRI
gi|494922183|ref|WP_007648219.1|Helicase[Formosasp.AK20]_gi|469825489|gb|EMQ95550.1|Helicasedomainprotein[Formosasp.AK20]/11626    ------NSG-----------LVLFLVPSIALLGQTLREWTSDAN--EPIKPICICSDSRI
gi|522083382|ref|WP_020594591.1|hypotheticalprotein[Spirosomapanaciterrae]/11637    ------GKG-----------FVLFLVPSIALLGQTLNEWYADAV--EKINAICICSDPEI
gi|490455352|ref|WP_004326157.1|helicase[Bacteroidesovatus]_gi|392669277|gb|EIY62768.1|hypotheticalproteinHMPREF1070_03635[Bact    ------GKG-----------FILFLVPSIALLGQTLEEWYAEAD--EPIKAICICSDAEV
gi|499516035|ref|WP_011202675.1|helicase[Bacteroidesfragilis]_gi|53713270|ref|YP_099262.1|helicasedomainprotein[Bacteroidesfrag    ------GKG-----------FILFLVPSIALLGQTLEEWYAEAD--EPIKAICICSDAEV
gi|651338332|ref|WP_026461320.1|helicase[Adhaeribacteraquaticus]/11620    ------GQG-----------LILFLVPSISLLGQTLREWSADAL--EPINPICICSDPEI
gi|806964642|gb|AKC17139.1|helicase[Flavobacteriumpsychrophilum]/11639    ------ANG-----------FILFLVPSIALLGQTLREWCNDAK--NPINAICICSDPEI
gi|752684374|ref|WP_041332322.1|helicase[Flavobacteriumpsychrophilum]_gi|666882889|gb|AIG30715.1|helicase[Flavobacteriumpsychro    ------ANG-----------FILFLVPSIALLGQTLREWCNDAK--NPINAICICSDPEI
gi|933196595|emb|CUP04894.1|DNAorRNAhelicasesofsuperfamilyII[Bacteroidesuniformis]/11631    ------GKG-----------LILFLVPSIALLGQTLREWSADAQ--EPINAICICSDPEI
gi|670509109|ref|WP_031456918.1|helicase[Flavobacteriumchungangense]/11625    ------GSG-----------MILFLVPSISLLGQTLREWSTFAK--EPINAICICSDPDI
                          :   *.: *: * .  :                 .
```

```
gi|490190857|ref|WP_004089451.1|hypotheticalprotein[Helicobacterbilis]_gi|476630638|gb|EMZ37196.1|hypotheticalproteinC826_02193      AKG-----------EDDLDIKELPKKPSTNPQDIIEAYKKAQ-------ENNERFIIFST
gi|488896133|ref|WP_002807242.1|MULTISPECIES:restrictionendonucleaseEco57I[Campylobacter]_gi|380541096|gb|EIA65377.1|hypothetic      GKS----------VEDDIDILELPIPASTDTQSIKKAYDIAK-------KDNKRFIIFST
gi|746602240|ref|WP_039628365.1|restrictionendonucleaseEco57I[Campylobacterlari]_gi|744819395|gb|AJD03391.1|putativehelicase[Ca      GKS----------IEDDIDILELPIPASTDTQSIKKAYDIAK-------KDNKRFIIFST
gi|727317135|ref|WP_033772558.1|restrictionendonucleaseEco57I[Helicobacterpylori]/11622                                              GQSKKNK--SKNEDNDDIKFSELPLKPSTRLEDILSVYEKAK-------QENKRFIIFST
gi|727103490|ref|WP_033618954.1|restrictionendonucleaseEco57I[Helicobacterpylori]/11616                                              GQSKKNK--SKNEDNDDIKFSELPLKPSTRLEDILSVYEKAK-------QENKRFIIFST
gi|727340205|ref|WP_033794408.1|restrictionendonucleaseEco57I[Helicobacterpylori]/11609                                              GK-------SKNEDNDDINFSELPLKPSTRLEDILSVYEKAK-------KENKRFIIFST
gi|727292407|ref|WP_033749975.1|restrictionendonucleaseEco57I[Helicobacterpylori]/11611                                              GK-------SKNEDNDDINFSELPLKPSTRLEDILSVYEKAK-------QENKRFIIFST
gi|727289795|ref|WP_033747591.1|restrictionendonucleaseEco57I[Helicobacterpylori]/11612                                              GQSKKNK--SKNEDNDDIKFSELPLKPSTRLEDILSTYEKAQ-------KENKRFIIFST
gi|727319676|ref|WP_033775047.1|restrictionendonucleaseEco57I[Helicobacterpylori]/11612                                              GQSKKNK--SKNEDNDDIKFSELPLKPSTRLEDILSVYEKAK-------QENKRFIIFST
gi|447212910|ref|WP_001290166.1|restrictionendonucleaseEco57I[Helicobacterpylori]_gi|308063540|gb|ADO05427.1|hypotheticalprotei      GKGKKNK--N-DDGTDDINFSELPLKPSTRPEDILSVHEKAQ-------KENKRFIIFST
gi|447212901|ref|WP_001290157.1|restrictionendonucleaseEco57I[Helicobacterpylori]_gi|384557329|gb|AFH97797.1|hypotheticalprotei      GKGKKNK--N-DDDADDIHFSELPLKPSTRLEDILSVHKKAQ-------KENKRFIIFST
gi|515849671|ref|WP_017280424.1|hypotheticalprotein[Helicobacterpylori]/11615                                                        GKGKKNK--N-DDDTDDINFSELPNKPSTRPEDILSVCELAQ-------KENKRFIIFST
gi|447212900|ref|WP_001290156.1|restrictionendonucleaseEco57I[Helicobacterpylori]_gi|317178970|dbj|BAJ56758.1|TypeIIGrestrictio      GKGKKNK--N-DDDTDDINFSELPLKPSTRPEDILSVCELVQ-------KENKRFIIFST
gi|447079018|ref|WP_001156274.1|hypotheticalprotein[Helicobacterpylori]/11614                                                        GKGKKNK--S-DDGIDDINFSELPLKPSTRLEDILSVYEKAQ-------KENKRFIIFST
gi|447212800|ref|WP_001290056.1|hypotheticalprotein[Helicobacterpylori]_gi|532153248|gb|EQL67316.1|restrictionendonucleaseEco57      GKGKKNK--S-DDNSDDIHFSELPLKPSTRLEDILSVHEKAK-------KENKRFIIFST
gi|505457237|ref|WP_015644339.1|hypotheticalprotein[Helicobacterpylori]_gi|499061160|gb|AGL70074.1|restrictionendonucleaseEco57      GKGKKNK--N-DDNTDDIKFSELPLKPSTRLEDILSVYEKAQ-------KENKRFIIFST
HPYUM037X/11591                                                                                                                      GKGKKNK--N-DDNTDDIKFSELPLKPSTRLEDILSVYEKAQ-------KENKRFIIFST
gi|920026297|ref|WP_052937176.1|restrictionendonuclease[Helicobacterpylori]_gi|912528678|gb|KNX48238.1|restrictionendonucleaseE      GKGKKSK--S-DDNSDDINFSELPLKPSTRLEDILSVHKKAQ-------KENKRFIIFST
gi|915539050|ref|WP_050841327.1|restrictionendonuclease[Helicobacterpylori]_gi|908890887|gb|KNE06183.1|restrictionendonucleaseE      GKS-------KDEDNDDINFSELPLKPSTRLEDILSVYEKAQ-------KENKRFIIFST
gi|727101397|ref|WP_033616957.1|restrictionendonucleaseEco57I[Helicobacterpylori]/11592                                              GKS-------KDEDNDDIKFSELPIKPSTRLEDILSVHKKAQ-------KENKRFIIFST
gi|562814875|emb|CDK13002.1|TypeISPRMsystem[Helicobacterpylori26695]/11588                                                          GKS-------KDEDNDDIKFSELPLKPSTRLEDILSVRKKAQ-------KENKRFIIFST
HPYAXVIII/11588                                                                                                                      GKS-------KDEDNDDIKFSELPLKPSTRLEDILSVRKKAQ-------KENKRFIIFST
gi|447079029|ref|WP_001156285.1|restrictionendonucleaseEco57I[Helicobacterpylori]_gi|393085956|gb|EJB86635.1|hypotheticalprotei      GQS-------KNEDNDDIKFSELPIKPSTRLEDILSVYEKAQ-------KENKRFIIFST
gi|489105953|ref|WP_003015814.1|restrictionendonuclease[Helicobacterpylori]_gi|477503388|gb|ENH59950.1|restrictionendonucleasef      GQN-------KNEDNDDIKFSELPIKASTRLEDILSVYEKAQ-------KENKRFIIFST
gi|447079006|ref|WP_001156262.1|restrictionendonucleaseEco57I[Helicobacterpylori]_gi|393067470|gb|EJB68280.1|hypotheticalprotei      GKS-------KDEDNDDIKFSELPIKSSTRLEDILSVHKKAQ-------KENKRFIIFST
gi|727314589|ref|WP_033770298.1|restrictionendonucleaseEco57I[Helicobacterpylori]/11586                                              GKS-------KNEDNDDIKFSELPIKPSTRLEDILSVYEKAQ-------KENKRFIIFST
gi|447079019|ref|WP_001156275.1|restrictionendonucleaseEco57I[Helicobacterpylori]_gi|308065028|gb|ADO06920.1|hypotheticalprotei      AQS-------KNEDNDDIKFSELPLKPSTRLEDILSVHKKAQ-------KENKRFIIFST
gi|447079028|ref|WP_001156284.1|restrictionendonucleaseEco57I[Helicobacterpylori]_gi|393076030|gb|EJB76784.1|hypotheticalprotei      GKS-------KDEDNDDINFSELPLKPSTRLEDILSVYQKAQ-------KENKRFIIFST
gi|393034009|gb|EJB35070.1|hypotheticalproteinHPNQ4053_0631[HelicobacterpyloriNQ4053]/11514                                         GES-------KNEDNDDINFSELPIKASTRLEDILSAYEKAQ-------KENKRFIIFST
gi|447079004|ref|WP_001156260.1|restrictionendonucleaseEco57I[Helicobacterpylori]_gi|384552418|gb|AFI07366.1|hypotheticalprotei      GQS-------KNEDNDDIKFSELPLKPSTRLEDILSTYEKAQ-------KENKRFIIFST
gi|746011306|ref|WP_039088215.1|restrictionendonucleaseEco57I[Helicobacterpylori]_gi|733488720|gb|KHL85979.1|restrictionendonuc      GQS-------KNEDNDDINFSELPIKPSTRLEDILSVHEKAQ-------KENKRFIIFST
gi|531081162|gb|EQD92166.1|restrictionendonucleaseEco57I[HelicobacterpyloriPZ5080]/11509                                             AQS-------KNEDNDDIKFSELPLKASTRLEDILSVYEKAQ-------KENKRFIIFST
gi|544760796|ref|WP_021187395.1|restrictionendonucleaseEco57I[Helicobacterpylori]_gi|531086508|gb|EQD96745.1|restrictionendonuc      GQS-------KNEDNDDIKFSELPVKPSTRLEDILSVYEKAQ-------KENKRFIIFST
gi|447079012|ref|WP_001156268.1|restrictionendonucleaseEco57I[Helicobacterpylori]_gi|393028199|gb|EJB29286.1|hypotheticalprotei      GKS-------KNEDNDNIKFSELPIKPSTRLEDILSVYEKAQ-------KENKRFIIFST
gi|746008458|ref|WP_039085555.1|restrictionendonucleaseEco57I[Helicobacterpylori]_gi|733480358|gb|KHL78026.1|restrictionendonuc      GQS-------KNEDNDDIKFSELPLKPSTRLEDILSVYEKAQ-------KENKRFIIFST
gi|447079011|ref|WP_001156267.1|restrictionendonucleaseEco57I[Helicobacterpylori]_gi|393038534|gb|EJB39568.1|hypotheticalprotei      GQS-------KNEDNDDIKFSELPIKASTRLEDILSVHEKAQ-------KENKRFIIFST
gi|393037163|gb|EJB38199.1|hypotheticalproteinHPNQ4161_0422[HelicobacterpyloriNQ4161]/11556                                         GQSKKNK--SKNEDNDDIKFSELPIKPSTRLEDILNVYEKAQ-------KENKRFIIFST
gi|447079007|ref|WP_001156263.1|restrictionendonucleaseEco57I[Helicobacterpylori]_gi|393051817|gb|EJB52768.1|hypotheticalprotei      GQS-------KNEDNDDIKFSELPIKPSTRLEDILSVRKKAQ-------KENKRFIIFST
gi|487867094|ref|WP_001940560.1|helicase[Helicobacterpylori]_gi|459505657|gb|EMG98418.1|helicaseprotein[HelicobacterpyloriGAM21      GQS-------KNEDNDDIKFSELPLKPSTRLEDILSTYEKAQ-------KENKCFIIFST
gi|487872094|ref|WP_001945560.1|helicase[Helicobacterpylori]_gi|459512104|gb|EMH04523.1|helicaseprotein[HelicobacterpyloriGAM24      GQSKKNK--SKNEDNDDIKFSELPLKPSTRLEDILSVYEKAQ-------KENKRFIIFST
gi|487880228|ref|WP_001953694.1|helicase[Helicobacterpylori]_gi|459529131|gb|EMH20734.1|helicaseprotein[HelicobacterpyloriGAM26      GQSKKNK--SKNEDNDDIKFSELPIKASTRLEDILSVYERAQ-------KENKRFIIFST
gi|487882593|ref|WP_001956059.1|helicase[Helicobacterpylori]_gi|459533462|gb|EMH24859.1|helicaseprotein[HelicobacterpyloriGAM26      GQSKKNK--SKNEDNDDIKFSELPIKASTRLEDILSVYEKAQ-------KENKRFIIFST
gi|487845430|ref|WP_001918896.1|helicase[Helicobacterpylori]_gi|410713871|gb|EKQ71363.1|helicaseprotein[HelicobacterpyloriGAM10      GQSKKNK--SKNEDNDDIKFSELPIKASTRLEDILSVYERAQ-------KENKRFIIFST
gi|447079005|ref|WP_001156261.1|restrictionendonucleaseEco57I[Helicobacterpylori]_gi|393069830|gb|EJB70625.1|hypotheticalprotei      GQSKKNK--SKNEDNDDIKFSELPIKPSTRLKDILSVYEKAQ-------KENKRFIIFST
gi|459506808|gb|EMG99528.1|helicaseprotein[HelicobacterpyloriGAM121Aii]/11545                                                        GQSKKNK--SKNEDNDDIKFSELPLKASTRLEDILSVYEKAQ-------KENKRFIIFST
gi|487825283|ref|WP_001898749.1|restrictionendonucleaseEco57I[Helicobacterpylori]_gi|393107526|gb|EJC08068.1|hypotheticalprotei      GQSKKNK--SKNEDNDDIKFSELPLKPSTRLEDILSTYEKAQ-------KENKRFIIFST
gi|447079008|ref|WP_001156264.1|restrictionendonucleaseEco57I[Helicobacterpylori]_gi|393058636|gb|EJB59524.1|hypotheticalprotei      GQSKKNK--SKNEDNDDINFSELPIKPSTRLEDILSTYEKAQ-------KENKRFIIFST
gi|447079015|ref|WP_001156271.1|restrictionendonucleaseEco57I[Helicobacterpylori]_gi|307637357|gb|ADN79807.1|hypotheticalprotei      GQSKKNK--SKNEDNDDIKFSELPIKPSTRLEDILSTYEKAQ-------KENKRFIIFST
gi|487884861|ref|WP_001958327.1|helicase[Helicobacterpylori]_gi|459535535|gb|EMH26834.1|helicaseprotein[HelicobacterpyloriGAM26      GQSKKNK--SKNEDNDDIKFSELPIKPSTRLEDILSAYEKAQ-------KENKRFIIFST
gi|487886365|ref|WP_001959831.1|helicase[Helicobacterpylori]_gi|459538727|gb|EMH29861.1|helicaseprotein[HelicobacterpyloriGAM27      GQSKKNK--SKNEDNDDIKFSELPIKASTRLEDILSVYEKAQ-------KENKRFIIFST
gi|545301637|ref|WP_021581412.1|helicase[Helicobacterpylori]_gi|535931889|gb|ERA61745.1|helicaseprotein[HelicobacterpyloriGAM11      GQS-------KNEDNDDIKFSELPLKPSTRLEDILSTYEKAQ-------KENKRFIIFST
gi|447079010|ref|WP_001156266.1|restrictionendonucleaseEco57I[Helicobacterpylori]_gi|393095477|gb|EJB96081.1|hypotheticalprotei      GQSKKNK--SKNEDNDDIKFSELPIKASTRLEDILSTYEKAQ-------KENKRFIIFST
gi|447079009|ref|WP_001156265.1|restrictionendonucleaseEco57I[Helicobacterpylori]_gi|393056294|gb|EJB57206.1|hypotheticalprotei      GQSKKNK--SKNEDNDDIKFSELPLKPSTRLEDILSIYEKAQ-------KENKRFIIFST
gi|471386985|gb|EMR58906.1|restrictionendonucleasefamilyprotein[HelicobacterpyloriHpH1]/11504                                       GQSKKNK--SKNEDNDDIKFSELPIKASTRLEDILSTYEKAQ-------KENKRFIIFST
gi|487875679|ref|WP_001949145.1|helicase[Helicobacterpylori]_gi|459516928|gb|EMH09091.1|helicaseprotein[HelicobacterpyloriGAM25      GQS-------KNEDNDDIKFSELPLKASTRLEDILSTYEKAQ-------KENKRFIIFST
gi|496130066|ref|WP_008854573.1|DNAdamageinducibleprotein[Commensalibacterintestini]_gi|353671689|gb|EHD13391.1|helicasedomain/      GKKHAK----E-DDSVIVGISDLQFPATTNATSLKREIAIA-----RQQRPDAMTVVFST
gi|924898894|ref|WP_053556546.1|damageinducibleprotein[Sphingopyxissp.113P3]_gi|924523996|gb|ALC14732.1|damageinducibleprotei/1      GVRRG-----R-DDTTDIDAHDLEIPATTNASVLAAKA--------TIDVPERMTVIFST
gi|780802950|gb|KJS08562.1|damageinducibleprotein[Hoefleasp.BRH_c9]/11633                                                           GKRKS-----G-DDLADIDIHDLAYPATTDARTLASKI--------TAHPDLRMTVVFST
gi|944671432|gb|KQN22886.1|damageinducibleprotein[Sphingomonassp.Leaf32]/11415                                                      GKRNRG----S-DDIGDIDVHDLAHPATTDPAKLAAKA--------KVPTPDEMTVVFST
gi|944612953|gb|KQM64772.1|damageinducibleprotein[Sphingomonassp.Leaf16]/11726                                                      GKRNRG----S-DDIGDIDVHDLAHPATTDPAKLAAKA--------KVPTPDEMTVVFST
gi|764353851|ref|WP_044331468.1|damageinducibleprotein[Sphingomonashengshuiensis]_gi|761897338|gb|AJP71707.1|damageinduciblep/1      GKRRKS----N-SDVAEIETHDLDYPATTNAAKLAQKA--------SHPADDRMTVVFST
gi|945874472|gb|KQZ27973.1|damageinducibleprotein[Mesorhizobiumsp.Root552]/11651                                                    GKRRKA----D-NDIAEIETHDLDYPATTDAAKLAQKA--------TQPAADRMTVVFST
gi|759408081|ref|WP_043132403.1|damageinducibleprotein[Paracoccussanguinis]_gi|694217086|gb|KGJ14281.1|damageinducibleprotein/1      GRNAD-------ADSLDLNVHDLAFPATTDPQKLAQQV--------AAAPPDRMTVVFST
gi|654342671|ref|WP_027836116.1|damageinducibleprotein[Maritaleamyrionectae]/11632                                                  GRNED-------ADTLDLNVHDLAFPATTSAEKLSHQV--------QTAPNEKMTVVFST
gi|126717687|gb|EBA14409.1|hypotheticalproteinRSK20926_02032[Roseobactersp.SK20926]/11603                                           GRRAD-------ADSLDLNVHDLAFPATTDPEKIARQV--------TDAPADQMTVVFST
gi|765301116|ref|WP_044628780.1|damageinducibleprotein[Halomonasmeridiana]_gi|764089679|gb|KJD19875.1|damageinducibleprotein[/1      GRQTD-------ADSLDLNIHDLAFPATTDAGKLATQV--------LNADGEKMTVVFST
gi|518053006|ref|WP_019223214.1|DNAdamageinducibleprotein[Bartonellarattaustraliani]/11654                                          GKRRKV----Q-DDAVAMDSSDLVLPATTDAKELASKA--------HKTSPNVMTVIFST
```

```
gi|493637806|ref|WP_006589578.1|DNAdamageinducibleprotein[Bartonellabirtlesii]_gi|395410345|gb|EJF76900.1|hypotheticalproteinM/    GKRRKN----Q-DDIVGMETSDLVLPATTDAKALAKEA--------CENLTDAMTVIFST
gi|501188603|ref|WP_012231622.1|DNAdamageinducibleprotein[Bartonellatribocorum]_gi|161017843|emb|CAK01401.1|helicase/methyltra/    GKRRKN----Q-EDIVGMETSDLVLPATTDAQALAKEA--------CENLADAMTVVFST
gi|651388616|ref|WP_026500615.1|damageinducibleprotein[Bartonellagrahamii]/11655                                                   GKRRKN----Q-DDIVGMETSDLVLPATTDAQALAKEA--------RENLTDAMTVVFST
gi|501188669|ref|WP_012231687.1|DNAdamageinducibleprotein[Bartonellatribocorum]_gi|161017922|emb|CAK01480.1|helicase/methyltra/    GKRRKN----Q-EDIVGMETSDLVLPATTDAQALAKEA--------CENLEDAMTVVFST
gi|518048832|ref|WP_019219040.1|DNAdamageinducibleprotein[Bartonellaflorencae]/11655                                               GKRRKN----Q-DDIVGMETSDLVLPATTDAKALAKEA--------YENLADAMTVIFST
gi|518053617|ref|WP_019223825.1|DNAdamageinducibleprotein[Bartonellarattaustraliani]/11607                                         GKRRKV----Q-DDAIAMDTSDLVLPATTDAKELASKA--------HKTSPDVMTVIFST
gi|506336449|ref|WP_015856168.1|DNAdamageinducibleprotein[Bartonellagrahamii]_gi|240267492|gb|ACS51080.1|helicase/methyltransf/     GKRRKN----Q-DDIVGMETSDLVLPATTDAKALAKEA--------CENLTDAMTVIFST
gi|494588832|ref|WP_007347427.1|DNAdamageinducibleprotein[Bartonellarattimassiliensis]_gi|395419053|gb|EJF85361.1|hypothetical/    GKRRKN----KNDDEAGLDASDLVLPATTDSQELARKA--------NKTSLDVMTVVFST
gi|501188650|ref|WP_012231668.1|DNAdamageinducibleprotein[Bartonellatribocorum]_gi|161017902|emb|CAK01460.1|helicase/methyltra/    GKRRKN----KNDDEAGLDASDLVLPATTDAKELARKA--------NKTSLDVMTVVFST
gi|501188624|ref|WP_012231643.1|DNAdamageinducibleprotein[Bartonellatribocorum]_gi|161017866|emb|CAK01424.1|helicase/methyltra/    GKRRKN----Q-EDDGEISASDLALPATTDASRLVEKV--------GNSSPHAMSVIFAT
gi|518053856|ref|WP_019224064.1|DNAdamageinducibleprotein[Bartonellarattaustraliani]/11625                                         GKRRKK----Q-GDDGEISASDLALPATTDASKLFEKV--------GNSSPHVMRVIFAT
gi|501188053|ref|WP_012231081.1|DNAdamageinducibleprotein[Bartonellatribocorum]_gi|161017434|emb|CAK00992.1|helicase/methyltra/    GKRRKN----Q-EDDGEISASDLALPATTDASRLVEKV--------GNASPHAMSVIFAT
gi|651388630|ref|WP_026500626.1|damageinducibleprotein[Bartonellagrahamii]/11657                                                   GKRRKN----Q-DDIVGMETSDLVLPATTDAKALAKEA--------CENLADAMTVIFST
gi|518048855|ref|WP_019219063.1|DNAdamageinducibleprotein[Bartonellaflorencae]/11658                                               GKRRKG----KHDDEASLDASDLVLPATTDAQALARKA--------NKTSLDVMTVVFST
gi|490995101|ref|WP_004856829.1|DNAdamageinducibleprotein[Bartonelladoshiae]_gi|395413167|gb|EJF79645.1|hypotheticalproteinMCS/    GKRRKG----KHDDEASLDASDLVLPATTDAQALARKA--------NKTSLDVMTVVFST
gi|491002558|ref|WP_004864279.1|DNAdamageinducibleprotein[Bartonellavinsonii]_gi|395421392|gb|EJF87643.1|hypotheticalproteinME/    GKRRKN----Q-DDIAELDVSDLALPATTDPQELASKA--------NKISPDVMTVVFST
gi|491003373|ref|WP_004865094.1|DNAdamageinducibleprotein[Bartonellavinsonii]_gi|395432864|gb|EJF98838.1|hypotheticalproteinME/    GKRRKN----Q-DDIAELDVSDLALPATTDPQELASKA--------NKTSPDVMTVVFST
gi|491002426|ref|WP_004864148.1|DNAdamageinducibleprotein[Bartonellavinsonii]_gi|395421767|gb|EJF88003.1|hypotheticalproteinME/    GKRRKN----Q-DDDAGLDSSDLALPATTDAKQLASKA--------NKASPNVMTVIFST
gi|491004024|ref|WP_004865745.1|DNAdamageinducibleprotein[Bartonellavinsonii]_gi|395432542|gb|EJF98522.1|hypotheticalproteinME/    GKRRKN----Q-DDDAGLDTSDLALPATTDAKQLASKA--------NKTSPDVMTVIFST
gi|648396442|ref|WP_026088193.1|damageinducibleprotein[Bartonellarattaustraliani]/11593                                            GKRRKA----Q-DDAIAMDTSDLVLPATTDAKELASKA--------HKTSLDVMTVIFST
gi|748190174|ref|WP_039761290.1|damageinducibleprotein[Bartonellaqueenslandensis]/11658                                            GKRRKT----K-DDAIAMDSSDLVLPATTDAKELASKA--------HKTSLDVMTVVFST
gi|494817599|ref|WP_007553007.1|DNAdamageinducibleprotein[Bartonellasp.DB56]_gi|395411312|gb|EJF77837.1|hypotheticalproteinMC/1    GKRRKA----Q-DDAAELDVSDLALPATTDAQKLGEKA--------NKTSPDAMTVVFAT
gi|515763376|ref|WP_017195976.1|DNAdamageinducibleprotein[Bartonellabirtlesii]/11656                                               GKRRKA----Q-DDAAELDISDLALPATTDAQKLGEKA--------NKTSPDAMTVVFAT
gi|494589420|ref|WP_007348014.1|DNAdamageinducibleprotein[Bartonellarattimassiliensis]_gi|395416278|gb|EJF82667.1|hypothetical/    GKRRKN----NKNDALEFDTSDLALPATTDAKELASKA--------GKVSHDLMTVVFST
gi|506336469|ref|WP_015856188.1|DNAdamageinducibleprotein[Bartonellagrahamii]_gi|240267512|gb|ACS51100.1|helicase/methyltransf/     GKRRKN----NKNDALEFDSSDLALPATTDAKELASKA--------GKVSHDLMTVVFST
gi|749308087|ref|WP_040132227.1|damageinducibleprotein[Bartonellatribocorum]_gi|605051878|emb|CDO49988.1|helicase/methyltransf/    GKRRKN----NKNDALEFDSSDLALPATTDAKELASKA--------GKVSHDLMTVVFST
gi|515763800|ref|WP_017196400.1|DNAdamageinducibleprotein[Bartonellabirtlesii]/11658                                               GKRRKS----KHDDEAGLDTSDLALPATTDAKELARKA--------NKTSLDVMTVVFST
gi|492186994|ref|WP_005773380.1|DNAdamageinducibleprotein[Bartonellaelizabethae]_gi|395418361|gb|EJF84688.1|hypotheticalprotei/    GKRRKG----KHDDESGLDASDLVLPATTDAEELARKA--------NKTSLDVMTVVFST
gi|501188615|ref|WP_012231634.1|DNAdamageinducibleprotein[Bartonellatribocorum]_gi|161017856|emb|CAK01414.1|helicase/methyltra/    GKRRKG----KHDDESGLDASDLVLPATTDSQELARKA--------NKTSLDVMTVVFST
gi|501187470|ref|WP_012230499.1|DNAdamageinducibleprotein[Bartonellatribocorum]_gi|161017090|emb|CAK00648.1|helicase/methyltra/    GKRRKG----KHDDESGLDASDLVLPATTDSQELARKA--------NKTSLDVMTVVFST
gi|501188636|ref|WP_012231655.1|DNAdamageinducibleprotein[Bartonellatribocorum]_gi|161017879|emb|CAK01437.1|helicase/methyltra/    GKRRKG----KHDDESGLDASDLVLPATTDSQELARKA--------NKTSLDVMTVVFST
gi|930151941|emb|CTQ34818.1|UvsWhelicase[Jannaschiarubra]/11641                                                                    GKRQG-----R-QDTADIAIHDLAYPATTNGTALADKM--------ADAAPEAMTAVFAT
gi|928977106|ref|WP_054001461.1|damageinducibleprotein[Shimiasp.SK013]_gi|927698852|gb|KPA22698.1|typeIrestrictionenzymeEcoKIs/    GKRRKD----R-GDVAEIEVHELEFPATTQPGKLVEKA--------GVDDAERMTVVFST
gi|738872465|ref|WP_036760884.1|damageinducibleprotein[Leisingeradaeponensis]/11643                                                GKRRQF----K-DDVAEIDILDLAFPATTDPAKLAAGA--------ADEAQGRMTVIFAT
gi|518050881|ref|WP_019221089.1|DNAdamageinducibleprotein[Bartonellasenegalensis]/11652                                            GKRRKN----T-HDIAEIDVFDLAFPATTDAAKLAERA--------TNSVADEMTVVFAT
gi|493980596|ref|WP_006923523.1|DNAdamageinducibleprotein[Bartonellawashoensis]_gi|395413322|gb|EJF79796.1|hypotheticalprotein/    GKRRKN----S-DDIAEIDVFDLAFPATTNAAKLAECV--------SDGVVDKMTVVFAT
gi|493983106|ref|WP_006925984.1|DNAdamageinducibleprotein[Bartonellawashoensis]_gi|395418127|gb|EJF84455.1|hypotheticalprotein/    GKRRKN----S-DDIAEIDVFDLAFPATTNAAKLAECV--------SDGVVDKMTVVFAT
gi|499494671|ref|WP_011181311.1|DNAdamageinducibleprotein[Bartonellahenselae]_gi|49239008|emb|CAF28308.1|hypotheticalproteinBH/    GKRRKN----T-HDIAEIDVFDLAFPATTDAAKLAKQA--------KNSVADKMTVVFAT
gi|651388426|ref|WP_026500441.1|damageinducibleprotein[Bartonellagrahamii]/11652                                                   GKRRKN----S-DDVAEIDVFDLAFPATTDAAKLAEQA--------GKSVADRMSVVFAT
gi|506337280|ref|WP_015856999.1|DNAdamageinducibleprotein[Bartonellagrahamii]_gi|240268402|gb|ACS51990.1|helicase/methyltransf/     GKRRKN----S-DDVAEIDVFDLAFPATTDAAKLAEQA--------GKSVADRMSVVFAT
gi|494817535|ref|WP_007552943.1|DNAdamageinducibleprotein[Bartonellasp.DB56]_gi|395412002|gb|EJF78518.1|hypotheticalproteinMC/1    GKRRKN----S-DDVAEIDVFDLAFPATTDAAKLAEQA--------GKSVADRMTVVFAT
gi|493638099|ref|WP_006589864.1|DNAdamageinducibleprotein[Bartonellabirtlesii]_gi|395409809|gb|EJF76394.1|hypotheticalproteinM/    GKRRKS----S-DDVAEIDVFDLAFPATTDAAKLAERA--------SGDVADRMTVVFAT
gi|748189589|ref|WP_039760707.1|damageinducibleprotein[Bartonellaqueenslandensis]/11651                                            GKRRKN----S-DDVAEIDVFDLAFPATTDAAKLAEQA--------GDGVLDKMSVVFAT
gi|501189483|ref|WP_012232501.1|DNAdamageinducibleprotein[Bartonellatribocorum]_gi|161018902|emb|CAK02460.1|helicase/methyltra/    GKRRKN----S-DDVAEIDVCDLAFPATTYAAKLAEQA--------GTSVADEMSVVFAT
gi|518049181|ref|WP_019219389.1|DNAdamageinducibleprotein[Bartonellaflorencae]/11652                                               GKRRKN----S-DDVAEIDVCDLAFPATTDAAKLAEQA--------GKSVADEMSVVFAT
gi|492193255|ref|WP_005774528.1|DNAdamageinducibleprotein[Bartonellaelizabethae]_gi|395416403|gb|EJF82779.1|hypotheticalprotei/    GKRRKN----S-DDVAEIDVCDLAFPATTDAAKLAEQV--------GKTVADEMSVVFAT
gi|498387325|ref|WP_010701288.1|helicase/methyltransferase[Bartonellabovis]_gi|478418724|gb|ENN92426.1|helicase/methyltransfera    GKRRKS----N-DDIAEIDVSDLVFPATTDAAQLAKNA--------SDVVADEMTVVFAT
gi|319408697|emb|CBI82352.1|conservedhypotheticalprotein[BartonellaschoenbuchensisR1]/11661                                       GKRRKN----N-DDIAEIDVSDLVFPATTNAAQLAKNA--------SDVVADEMTVVFAT
gi|494741726|ref|WP_007477134.1|DNAdamageinducibleprotein[Bartonellamelophagi]_gi|395423925|gb|EJF90113.1|hypotheticalproteinM/    GKRRKS----N-DDIAEIDVSDLVVPATTNAVQLAKNA--------NDVVADEMTVVFAT
gi|737261405|ref|WP_035245349.1|damageinducibleprotein[Alcanivoraxjadensis]_gi|686989709|gb|KGD62556.1|hypotheticalproteinT9A_/    GKRKK-----G-DDVADLQQHDLAFPATTDPAKLAAEA--------GQADPDAMTVVFST
gi|916687564|ref|WP_051294655.1|damageinducibleprotein[Gemmobacternectariphilus]/11641                                             GKRRQS----K-DDIAEIDVLDLAFPATTDAVKLSLGA--------GSPEPEKMRVVFAT
gi|740294127|ref|WP_038132495.1|damageinducibleprotein[Thioclavasp.DT234]_gi|660524826|gb|KEO54689.1|hypotheticalproteinDT23_/1    GKRRVG----A-DDAAEIDAHDLAFPATTNPERLVEKA--------AIDAADRMTVVFST
gi|568331062|ref|WP_024099615.1|helicase[Phaeobactergallaeciensis]_gi|568232121|gb|AHD12007.1|putativehelicase[Phaeobactergalla    GKRRAS----N-NDIAEIDILDLAFPASTDAAKIAEGV--------NHQATDKMTVVFAT
gi|518810459|ref|WP_019966413.1|DNAdamageinducibleprotein[Pannonibacterphragmitetus]/11635                                         GKRRKN----S-DDIAEIEIHDLAFPATTDPVKVAEKA--------GDDDAERMTVIFST
gi|495305760|ref|WP_008030510.1|DNAdamageinducibleprotein[Rhodobactersp.SW2]_gi|259021698|gb|EEW25000.1|typeIIIrestrictionprot/    GKRRTS----T-DDIAEIEIHDLAFPATTDPVKVAEKA--------GEDDPERMTVIFST
gi|506403303|ref|WP_015923022.1|DNAdamageinducibleprotein[Rhodobactersphaeroides]_gi|221162951|gb|ACM03922.1|HypotheticalProte/    GKRRKN----T-DDIAEIEIHDLAFPATTDPQKVAEKA--------GDDDPERMTVIFST
gi|738818386|ref|WP_036708790.1|damageinducibleprotein[Paracoccussanguinis]_gi|694226786|gb|KGJ22541.1|damageinducibleprotein/1    GKRRKN----T-DDIAEIEIHDLAFPATTDPQKVAEKA--------GDDDPERMTVIFST
gi|746096057|ref|WP_039162453.1|damageinducibleprotein[Gallibacteriumanatis]_gi|703617775|gb|KGQ63868.1|damageinducibleprotei/1    GKQNKD-----NNDLVQTQIHELQYPATTNAASLFKAVTFR-------HNDEAMSVVFST
gi|737716785|ref|WP_035685631.1|damageinducibleprotein[Avibacteriumparagallinarum]/11643                                           GKQNKD-----NNDLVQTQIHELQYPATTNAASLFKAVTFR-------HNDEAMSVVFST
gi|540072553|gb|ERF78672.1|hypotheticalproteinN561_04930[Gallibacteriumanatis12656/12}/11651                                      GKQHKN-----NDDLVQTQIHELQYPATTNADSLLKAVTIR-------HNDEAMTVVFST
gi|917509519|ref|WP_052115936.1|damageinducibleprotein[Gallibacteriumanatis]_gi|702414161|gb|KGQ42233.1|damageinducibleprotei/1    GKQHKN-----NDDLVQTQIHELQYPATTNADSLLKAVTIR-------HNDEAMTVVFST
gi|746082942|ref|WP_039150268.1|damageinducibleprotein[Gallibacteriumanatis]_gi|703617078|gb|KGQ63194.1|damageinducibleprotei/1    GKQHKN-----NDDLVQTQIHELQYPATTNADSLLKAVTIR-------HNDEAMTVVFST
gi|822522885|ref|WP_046952840.1|damageinducibleprotein[Haemophilushaemolyticus]_gi|821501762|gb|KKZ59267.1|damageinduciblepro/1    GKQGKIK--N-EDDRVQQLNHELQYPATTNAKSLIKAVNAQ-------SRDLAMTVVFST
gi|404381014|gb|EJZ77501.1|putativehelicase[Pasteurellamultocidasubsp.gallicidaP1059]/11681                                       GKQGKNK--GKDDDRVQLLNHELQYPATTNATALVKAFNAQ-------SRQLAMNVVFST
gi|298281660|gb|EFI23149.1|helicaseCterminaldomainprotein[Neisseriasp.oraltaxon014str.F0314]/11493                                GKYKK------DDDRTFVRPSDLNYPATTNAKSLYQAASVL----H---DAEHMTVVYST
gi|348013055|gb|EGY51980.1|helicasedomainprotein[Neisseriashayeganii871]/11481                                                     GKKDKK----SEDDRVLVTTSDLQFPATTQPKSLAQALTVT----ERRNGGDAMTVVFST
gi|496407140|ref|WP_009116004.1|DNAdamageinducibleprotein[Neisseriawadsworthii]_gi|349794756|gb|EGZ48566.1|helicasedomainprote/    GKKGKK----DEDDRVLVTTSDLQFPATTHAKSLAQAISVA----EAKNNRQAMTVVYAT
```

```
gi|517216441|ref|WP_018405259.1|DNAdamageinducibleprotein[Marinobacterlipolyticus]/11656    GKRQGK-----DDDRVISGISDLQYPATTKALSLQKQMA-------DHHADDSMTVVFST
gi|738230264|ref|WP_036185691.1|damageinducibleprotein[Marinobactersp.ELB17]/11671    GKRQGK-----DDDRVISGISDLQYPATTNALSLQKQMA-------THHAEDAMTVVFST
gi|447197741|ref|WP_001274997.1|DNAdamageinducibleprotein[Acinetobacterbaumannii]_gi|408512324|gb|EKK13969.1|typeIIrestrictio/    GKK--K-----DDDNIVVGISDLKFPATTNPKSLVKAFKARDL-FKPNEANEDMNVVFST
gi|820775897|ref|WP_046739909.1|damageinducibleprotein[Acinetobactersp.AG1]_gi|819909758|gb|KKW75938.1|damageinducibleprotein/1    GKK--K-----DDDNIVVGISDLKFPATTNPQSLVKAFKARDL-FKPNEASEDMNVVFST
gi|584595163|ref|WP_024160846.1|helicase[Acinetobacterpittii]_gi|576702530|emb|CDI28049.1|helicasedomaincontainingprotein[Acin/    GKK--K-----DDDNIVVGISDLKFPATTNPQSLVKAFKARDL-FKPNEANEDMNVVFST
gi|514963042|ref|WP_016651366.1|hypotheticalprotein[Acinetobactersp.NIPH2036]_gi|514162665|gb|EPG41391.1|hypotheticalproteinF90    GKK--K-----DDDNIVVGISDLKFPATTNPQSLVKAFKARDL-FKPNEANEDMNVVFST
gi|515965564|ref|WP_017396147.1|DNAdamageinducibleprotein[Acinetobacterhaemolyticus]/11667    GKK--K-----DDDNIVVGISDLKFPATTNPQSLVKAFKARDL-FKPNEANEDMNVVFST
gi|491346810|ref|WP_005204747.1|MULTISPECIES:hypotheticalprotein[Acinetobacter]_gi|480335823|gb|ENX56033.1|hypotheticalproteinF    GKK--K-----DDDNIVVGISDLKFPATTNPQSLVKAFKARDL-FKPNEANEDMNVVFST
gi|693044472|ref|WP_032215268.1|damageinducibleprotein[Escherichiacoli]_gi|607725731|gb|EZE08230.1|damageinducibleprotein[Esc/1    GKK--N-----HDDNVVVGISDLKYPATTNATSLFKAFNQPDI-FGGD-RPTYMNVVFST
gi|742382177|ref|WP_038861304.1|damageinducibleprotein[Cronobactermuytjensii]_gi|924333747|gb|ALB69766.1|damageinducibleprote/1    GKK--N-----HDDNVVVGISDLKYPATTNANSLVKAFNQPDI-FGSE-KPPYMNVVFST
gi|915912804|ref|WP_050927035.1|damageinducibleprotein[Yersiniaenterocolitica]_gi|899805802|emb|CQH32042.1|typeIrestrictionenz/    GKR--N-----HDDNVVVGISDLKYPATTNANSLVKAFNQPDI-FGSD-KPPYMNVVFST
gi|921111327|gb|KON62781.1|typeIrestrictionenzymeEcoKIsubunitR[Komagataeibactereuropaeus]/11492    GKKKAR----ND-DEIKVNIHEIRYPATTSPGRLATEHAKR-------HDDAHMTVVFAT
gi|582024278|gb|AHI27615.1|DNAhelicaserestrictionenzymeTypeIIIRsubunit[KomagataeibacterxylinusE25]/11636    GNQQAE----NN-DEIRINIHELRYPATTDANRLSSEFIKR-------CDNTHMNVVFAT
gi|347662673|dbj|BAK86128.1|DNAhelicaserestrictionenzymeTypeIIIRsubunit[KomagataeibactermedellinensisNBRC3288]/11737    GKKKAK----ND-DEIKVNIHEIRYPATTSPGRLATEHAKR-------HDDAHMTVVFAT
gi|917445053|ref|WP_052051471.1|damageinducibleprotein[Acetobactertropicalis]/11429    GKKKAS----ND-DEIKVKVHELRYPATTNPERLATEYQKR-------HDATHMTVVFST
gi|453329445|dbj|GAC88472.1|DNAhelicaserestrictionenzymetypeIIIRsubunit[GluconobacterthailandicusNBRC3255]_gi|530287981|dbj|GAD    GKKKAS----QD-DEIKVKVHELRYPATTNPERLASEYQKR-------HDATHMTVVFST
gi|889770922|ref|WP_048844278.1|damageinducibleprotein[Gluconobacterfrateurii]/11419    GKKKAS----QD-DEIKVKVHELRYPATTNPERLASEYQKR-------HDATHMTVVFST
gi|504886208|ref|WP_015073310.1|hypotheticalprotein[Gluconobacteroxydans]_gi|411027954|gb|AFW01209.1|hypotheticalproteinB932_16    GKKKAS----QD-DEIKVKVHELRYPATTNPERLASEYQKR-------HDATHMTVVFST
gi|480040211|gb|ENV79751.1|hypotheticalproteinF942_01641[AcinetobacterursingiiANC3649]/11432    GKKRQA----DE-DAVNLLVHELDYPATTNAKRLAEEMQSR-------HKEHKMSVVFST
gi|822498222|ref|WP_046936504.1|damageinducibleprotein[Xanthomonasgardneri]_gi|821751224|gb|KLB07223.1|damageinducibleprotein/1    GKKKGR----KEDDALQTFAHELRYPATTSGKTLASSMAAR-------HDADHMTVVFST
gi|654059559|ref|WP_027705556.1|damageinducibleprotein[Zymobacterpalmae]/11635    GKKRKR----REDDAVQTFVHELRYPATTAADKLAYEVSQR-------ADADHMTVVFGT
gi|643535620|ref|WP_025224315.1|damageinducibleprotein[Methylophilaceaebacterium11]_gi|577704400|gb|EUJ10546.1|putativehelicas/    GKKRKK----DE-ETVQTFIHELRYPATTEPARLAIEMEKR-------HDAHHMSVVFST
MBA11I/11635    GKKRKK----DE-ETVQTFIHELRYPATTEPARLAIEMEKR-------HDAHHMSVVFST
gi|655355857|ref|WP_028762287.1|damageinducibleprotein[Shewanellacolwelliana]/11632    GKNRDT----NEDDVVQTLVHELRYPATTDAYKLAKEFELR-------HDDEHMSVVYST
gi|753204236|ref|WP_041510579.1|damageinducibleprotein[Shewanellasp.cp20]_gi|752457765|gb|KIO36326.1|damageinducibleprotein[S/1    GKKRNA----NE-DTVQTFTHELRYPATTNAKRLAEEVAKR-------QDSSHMSVVFST
KPNNIH30III/11619    GKKRKA----ND-DVVQTFTHELRYPATTNAKRLAEEVAKR-------SDSTHMNVVFST
gi|749295994|ref|WP_040120352.1|damageinducibleprotein[Klebsiellapneumoniae]_gi|723249300|gb|AIX81926.1|damageinducibleprotei/1    GKKRKA----ND-DVVQTFTHELRYPATTNAKRLAEEVAKR-------SDSTHMNVVFST
gi|641639739|emb|CDQ54010.1|unnamedproteinproduct[Klebsiellapneumoniae]/11641    GKKRKA----ND-DVVQTFTHELRYPATTNAKRLAEEVAKR-------SDSTHMNVVFST
gi|490797922|ref|WP_004660060.1|hypotheticalprotein[Acinetobactersp.NIPH236]_gi|479876779|gb|ENU27973.1|hypotheticalproteinF992    GKKKDA----TAIDAVTTLEHELQYPATTNPEKLAENVEHH-------HDADHMTVVFST
gi|916750926|ref|WP_051357982.1|damageinducibleprotein[Acinetobactertjernbergiae]/11453    GKKKNS----TAIDAVTTLEHELQYPATTDAARLAENVEKH-------HDSEHMTVVFST
gi|691098662|ref|WP_032025060.1|damageinducibleprotein[Acinetobacterbaumannii]_gi|593567521|gb|EXR50735.1|restrictionendonucle/    GKKKNA----TAIDAVTTLEHELQYPATTDAKKLAENVEKH-------HDDEHMTVVFST
gi|736669405|ref|WP_034676049.1|damageinducibleprotein[Acinetobacterbaumannii]/11643    GKKKNA----TAIDAVTTLQHELQYPATTNAQKLADNVEKH-------HDDDHMTVVFST
gi|491368928|ref|WP_005226826.1|hypotheticalprotein[Acinetobactersp.NIPH1847]_gi|480173259|gb|ENX09797.1|hypotheticalproteinF89    GKKKNA----TAIDAVTTLEHELQYPATTDAKKLAENVEKH-------HDDEHMTVVFST
gi|796853993|ref|WP_045795953.1|damageinducibleprotein[Acinetobacterindicus]_gi|787929761|gb|KJV44219.1|damageinducibleprotei/1    GKKKNH----SVIDAVTTLAHELQYPATTNAKKLAENVESN-------HDDEHMTVVFST
gi|295797463|gb|ADG32253.1|typeIIIrestrictionproteinressubunit[ThiomonasintermediaK12]/11676    GKKRQD------GDDYQTLIHELRYPATTDAARLAQAIAAR-------HDTSHMTVVFST
gi|499871179|ref|WP_011551913.1|DNAdamageinducibleprotein[Myxococcusxanthus]_gi|108467544|gb|ABF92729.1|putativerestriction/mo/    GKRRKK-----DDDTVQTFVHELQYPATTNAARLAAAMKKR-------HDAEHMSVVYAT
gi|495313852|ref|WP_008038600.1|DNAdamageinducibleprotein[Bartonellatamiae]_gi|395424181|gb|EJF90368.1|hypotheticalproteinME5_/    GKKRDK----KDDDSVQTFVHELQYPATTDAEKLAEEMTAW-------HEDDAMSVIFST
gi|501453335|ref|WP_012476784.1|DNAdamageinducibleprotein[Leptospirabiflexa]_gi|167777824|gb|ABZ96123.1|Helicase[Leptospirabif/    GKKKDK-----NEDVVETIEHELQYPATTDAKRLSEEFQKI-------HDDSHMSVVFST
gi|738104613|ref|WP_036063193.1|damageinducibleprotein[Leptospiranoguchii]/11652    GKKRKK-----DDDAVETFAHELQYPATTQPLRLASEMKKR-------HDDEHMSVVFST
gi|410001039|gb|EKO51661.1|typeIIIrestrictionenzyme,ressubunit[Leptospirakirschneristr.200802841]/11651    GKKRKK-----DDDAVETFAHELQYPATTQPERLAIEMKKR-------HDDEHMSVVFST
gi|490901851|ref|WP_004763774.1|typeIIIrestrictionenzyme,ressubunit[Leptospirakirschneri]_gi|464209249|gb|EMN24014.1|typeIIIres    GKKRKK-----DDDAVETFAHELQYPATTQPERLAIEMKKR-------HDDEHMSVVFST
gi|464398131|gb|EMO65902.1|typeIIIrestrictionenzyme,ressubunit[Leptospirakirschneristr.200803703]/11648    GKKRKK-----DDDAVETFAHELQYPATTQPERLAIEMKKR-------HDDEHMSVVFST
gi|945029024|gb|KQQ84900.1|damageinducibleprotein[Xanthomonassp.Leaf131]/11423    GKKRKR----KEDDAIQTFAHELRYPATTDAKRLAQSMSMR-------EDEEHMTVVFST
gi|896511314|ref|WP_049418183.1|damageinducibleprotein[Stenotrophomonasmaltophilia]/11639    GKKKGR----KEDDAVQTFAHELRYPATTDAKRLAQGVAAR-------HDAEHMTVVFST
gi|896513082|ref|WP_049419616.1|damageinducibleprotein[Stenotrophomonasmaltophilia]/11638    GKKKGR----KEDDAVQTLAHELRYPATTNAKALAKGVAVR-------SDGEHMTVVFST
gi|896580616|ref|WP_049469425.1|damageinducibleprotein[Stenotrophomonasmaltophilia]/11641    GKKKGR----KEDDAVQTLAHELRYPATTDARALAKGVAAR-------SDNAHMTVVFST
gi|648289350|ref|WP_026070499.1|damageinducibleprotein[Stenotrophomonasmaltophilia]/11639    GKKKGR----KEDDAVQTLAHELRYPATTDARALAKSVAAR-------SDDAHMTVVFST
gi|656272786|ref|WP_029218442.1|damageinducibleprotein[Xanthomonascassavae]/11639    GKKKGR----KEDDAVQTLAHELRYPATTNAKALAKGVAVR-------SDGEHMTVVFST
gi|406976236|gb|EKD98747.1|hypotheticalproteinACD_23C00290G0002[unculturedbacterium]/11679    GKKK-G----VDDFE--MQVHELRYPATTDPARLALEMNKR-------HDSEHMSVVFST
gi|828145737|ref|WP_047238120.1|damageinducibleprotein[Chromobacteriumsubtsugae]/11645    GKRR-K----KDDDVVQTLAHELRYPATTDAARLATEMAKR-------HDNEHMSVVFST
gi|654311174|ref|WP_027824327.1|damageinducibleprotein[Laribacterhongkongensis]/11641    GKKR-K----KDDDVVQTLAHELRYPATTDAARLAAEMAKR-------HDGEHMSVVFST
gi|757242173|gb|KIR15516.1|typeIrestrictionenzymeEcoKIsubunitR[Pseudomonasfluorescens]/11778    GKKR-K----KDDDAVQTFAHELRYPATTEPGKLAAEMLKR-------HDDQHMSVVFST
gi|653605161|ref|WP_027616682.1|damageinducibleprotein[Pseudomonassp.URHB0015]/11639    GKKR-K----KDDDAVQTFTHELRYPATTEPGKLAAEMLKR-------HDDQHMSVVFST
gi|520868025|ref|WP_020308060.1|helicase[Pseudomonassyringae]_gi|519159034|gb|EPM49935.1|helicasedomaincontainingprotein[Pseud/    GKKR-K----KDDDAVQTFAHELRYPATTAPRKLAAEMLKR-------HDDQHMSVVFST
gi|940156567|gb|KPX71668.1|Helicasedomaincontainingprotein[Pseudomonasamygdalipv.lachrymans]/11677    GKKR-K----KDDDAVQTFTHELRYPATTEPAKLAAEMLKR-------HDDQHMSVVFST
gi|505460508|ref|WP_015647610.1|helicase[Pseudomonassp.GLE121]_gi|499110212|ref|YP_007974374.1|helicasedomaincontainingprotein/    GKKR-K----ADDDVVQTFTHELRYPATTEPQRLASEMSKR-------YDDQHMSVVFST
gi|940235551|gb|KPY45311.1|hypotheticalproteinALO48_200154[Pseudomonassyringaepv.rhaphiolepidis]/11631    GKKR-K----ADDDVVQTFTHELRYPATTEPQRLASEMSKR-------YDDQHMSVVFST
gi|520880808|ref|WP_020312235.1|helicase[Pseudomonassyringae]_gi|519172158|gb|EPM62027.1|helicasedomaincontainingprotein[Pseud/    GKKR-K----QDDDVVQTFTHELRFPATTEPHRLASEMAKR-------HDDQHMSVVFST
gi|829046479|ref|WP_047282259.1|damageinducibleprotein[Pseudomonasfluorescens]/11630    GKKR-K----ADEDTVQVFTHELRYPATTKADRLAAEMLKR-------HDGEHMSVVFST
gi|499415493|ref|WP_011102960.1|DNAdamageinducibleprotein[Pseudomonassyringaegroupgenomosp.3]_gi|28867279|ref|NP_789898.1|heli/    GKKR-K----ADEDTVQVFTHELRYPATTKADRLAAEMLKR-------HDAEHMSVVFST
gi|730238299|ref|WP_033942897.1|damageinducibleprotein[Pseudomonasaeruginosa]/11629    GKKR-K----AEDDAVQVFTHELRYPATTKADRLAAEMLKR-------HDAEHMSVVFST
gi|495310125|ref|WP_008034873.1|DNAdamageinducibleprotein[Pseudomonassp.GM60]_gi|398186685|gb|EJM74052.1|putativehelicase[Pseu/    GKKR-K----AGDDAPQVFAHELRYPATTKADRLAAEMLKR-------HDAEHMSVVFST
gi|779992653|ref|WP_045428690.1|damageinducibleprotein[Pseudomonassp.MT1]_gi|699967872|dbj|BAP80334.1|DNAhelicaserestrictione/1    GKKR-K----VEDDAVQVFTHELRYPATTKADRLAAEMPKR-------HDAEHMSVVFST
gi|786037289|ref|WP_045733274.1|damageinducibleprotein[Pseudomonaspseudoalcaligenes]_gi|782987904|gb|KJU79152.1|damageinducib/1    GKKR-K----AEDDAVQVFTHELRYPATTKADRLAAEMLKR-------HDADHMSVVFST
gi|749666366|ref|WP_040265445.1|damageinducibleprotein[Pseudomonasrhodesiae]/11632    GKKR-K----AEDDAVQVFTHELRYPATTKADRLAAEMLKR-------HDAEHMSVVFST
gi|734902433|ref|WP_034133698.1|damageinducibleprotein[Pseudomonasfluorescens]/11635    GKKR-K----AEDDAVQVFTHELRYPATTKADRLAAEMLKR-------HDAEHMSVVFST
gi|490551031|ref|WP_004416146.1|typeIIrestrictionmodificationsystemrestriction/helicasesubunit[Pseudomonassyringae]_gi|4432823/    GKKR-K----AEDDAVQVFTHELRYPATTKADRLAAEMLKR-------HDAEHMSVVFST
```

```
gi|489199911|ref|WP_003109152.1|DNAdamageinducibleprotein[Pseudomonasaeruginosa]_gi|552648708|gb|ERY63184.1|hypotheticalprotei/    GKKR-K----AEDDAVQVFTHELRYPATTKADRLASEMLKR-------HDAEHMSVVFST
gi|730641237|ref|WP_034061328.1|damageinducibleprotein[Pseudomonasaeruginosa]/11636    GKKR-K----ADEDTPQVFAHELRYPATTKADRLAAEMLKR-------HDAEHMSVVFST
gi|751304980|ref|WP_041013386.1|damageinducibleprotein[Pseudomonasxanthomarina]_gi|703407611|emb|CEG51014.1|Helicasedomaincon/1    GKKR-K----VEDDAVQVFTHELRYPATTKADRLAAEMPKR-------HDAEHMSVVFST
gi|939335207|ref|WP_054881520.1|damageinducibleprotein[Pseudomonassp.NBRC111128]/11632    GKKR-K----SEDDAVQVFTHELRYPATTKADRLAAEMLKR-------HDAEHMSVVFST
gi|757693555|ref|WP_042923792.1|damageinducibleprotein[Pseudomonaschloritidismutans]_gi|780910610|gb|KJS78302.1|damageinducib/1    GKKR-K----ADEDTPQVFAHELRYPATTKADRLAAEMLKR-------HDDEHMSVVFST
gi|665953944|ref|WP_031319989.1|damageinducibleprotein[Pseudomonastolaasii]/11626    GKKR-K----AEDDAVQVFTHELRYPATTKADRLAAEMLKR-------HDAEHMSVVFST
gi|771600573|ref|WP_045213746.1|damageinducibleprotein[Pseudomonassp.21]_gi|770081123|gb|KJJ98634.1|damageinducibleprotein[Ps/1    GKKR-K----ADEDTPQVFAHELRYPATTKADRLAAEMLKR-------HDAEHMSVVFST
gi|740871396|ref|WP_038656644.1|damageinducibleprotein[Pseudomonasstutzeri]_gi|627772214|gb|AHY41437.1|damageinducibleprotein/1    GKKR-K----VEDDAVQVFTHELRYPATTKADRLAAEMPKR-------HDAEHMSVVFST
gi|946242205|gb|KRC93782.1|damageinducibleprotein[Pseudomonassp.Root9]/11634    GKKR-K----AEDDAVQVFTHELRYPATTQADRLAAEMLKR-------HDVEHMSVVFST
gi|553777322|ref|WP_023109107.1|MULTISPECIES:hypotheticalprotein[Pseudomonas]_gi|552638674|gb|ERY53184.1|hypotheticalproteinQ05    GKKR-K----AEDDAVQVFTHELRYPATTKADRLAAEMLKR-------HDAEHMSVVFST
gi|896383370|ref|WP_049324618.1|damageinducibleprotein[Pseudomonasstutzeri]/11627    GKKR-K----VEDDAVQVFTHELRYPATTKADRLAAEMSKR-------HDDEHMSVVFST
gi|696158510|gb|AIT41787.1|putativehelicase[Pseudomonassp.LM8]/11630    GKKR-K----ADEDTVQVFTHELRYPATTKADRLAAEMLKR-------HDAEHMSVVFST
gi|504634892|ref|WP_014821994.1|DNAdamageinducibleprotein[Pseudomonasstutzeri]_gi|390985249|gb|AFM35242.1|helicasedomainconta/1    GKKR-K----ADEDTVQVFTHELRYPATTKADRLAAEMLKR-------HDDEHMSVVFST
gi|544783974|ref|WP_021206729.1|DNAdamageinducibleprotein[Pseudomonasstutzeri]_gi|533224779|gb|EQM79688.1|DNAdamageinduciblep/1    GKKR-K----ADEDTVQVFTHELRYPATTKADRLAAEMFKR-------HDDQHMSVVFST
gi|504567385|ref|WP_014754487.1|MULTISPECIES:DNAdamageinducibleprotein[Pseudomonas]_gi|388559493|gb|AFK68634.1|helicasedomain/1    GKKR-K----AEDDAVQVFTHELRYPATTQADRLAAEMLKR-------HDAEHMSVVFST
gi|654441469|ref|WP_027912052.1|damageinducibleprotein[Pseudomonassp.URIL14HWK12:I7]/11630    GKKR-K----AEDDAVQVFTHELRYPATTKADRLAAEMLKR-------HDAEHMSVVFST
gi|941470066|ref|WP_055127894.1|damageinducibleprotein[Pseudomonasmediterranea]/11631    GKKR-K----AEDDAVQVFTHELRYPATTKADRLAAEMLKR-------HDAEHMSVVFST
gi|557815599|ref|WP_023446184.1|DNAdamageinducibleprotein[Pseudomonaschloritidismutans]_gi|557364081|gb|ESQ98190.1|DNAdamagei/1    GKKR-K----AEDDAVQVFTHELRYPATTKADRLAAEMLKR-------HDAEHMSVVFST
gi|727814791|ref|WP_033898247.1|damageinducibleprotein[Pseudomonassp.FH1]_gi|567648005|gb|ETK23229.1|helicasedomaincontaining/1    GKKR-K----AEDDAVQVFTHELRYPATTKADRLAAEMLKR-------HDSEHMSVVFST
gi|503278702|ref|WP_013513363.1|DNAdamageinducibleprotein[Desulfovibrioaespoeensis]_gi|316942375|gb|ADU61426.1|typeIIrestrict/    GKKR-Q----TDDDSVQVFTHELRYPATTQPDKLAKEMVKR-------HDSQHMSVVFST
gi|505228796|ref|WP_015415898.1|typeIIIrestrictionendonucleasesubunitR[Desulfovibriopiezophilus]_gi|451896976|emb|CCH49855.1|Ty    GKKR-K----KDDDSVQVFTHELRYPATTQPDKLAQEMDKR-------HDSQHMSVVFST
gi|517732543|ref|WP_018902751.1|hypotheticalprotein[Variovoraxparadoxus]/11675    GKKR-L----KEDDSVQTFTHELRYPATTQPARLAAEMTRR-------HDDTHMSVVFGT
gi|550909475|ref|WP_022662570.1|DNAdamageinducibleprotein[Desulfovibriolongus]/11637    GKKR-K----KDDDAVQTFTHELRYPATTEPARLAGEMTKR-------HDAQHMSVVFST
gi|945494542|gb|KQV50024.1|damageinducibleprotein[Massiliasp.Root335]/11647    GKKR-Q----KDDDSVQTFTHELRYPATTHPARLASEMAKR-------HDAAHMSVVFST
gi|334196917|gb|AEG70102.1|helicasedomainprotein[RalstoniasolanacearumPo82]/11663    GKKR-K----KDDDAVQTFVHELRYPATTDSARLAAEMAKR-------HDSTHMSVVFST
gi|656006746|ref|WP_029048170.1|damageinducibleprotein[Cupriavidussp.amp6]/11661    GKKR-K----KEDDAVQTFVHELRYPATTDAARLAAEMAKR-------HDASHMSVVFST
BPS2196ORF3054P/11659    GKKR-K----KEDDMVQTFVHELRYPATTNSARLAEEMSKR-------HDASHMSVVFST
gi|740932312|ref|WP_038717332.1|damageinducibleprotein[Burkholderiapseudomallei]_gi|760211882|gb|KIX58314.1|damageinduciblepr/1    GKKR-K----KEDDMVQTFVHELRYPATTNSARLAEEMSKR-------HDASHMSVVFST
gi|755810813|ref|WP_042583861.1|damageinducibleprotein[Burkholderiasp.MSHR3999]_gi|755174952|gb|KIP19790.1|DEAD/DEAHboxhelicas/    GKKR-K----KEDDEVKTFIHELRYPATTDSARLAAEMAKR-------HDASHMSAVFST
gi|484586570|gb|AGK48195.1|DEAD/DEAHboxhelicasefamilyprotein[BurkholderiathailandensisMSMB121]/11646    GKKR-K----KEDDEVKTFIHELRYPATTDSARLAAEMAKR-------HDASHMSAVFST
gi|737582935|ref|WP_035553941.1|damageinducibleprotein[Burkholderiasp.9120]/11658    GKKR-K----KEDDMVQTFVHELRYPATTEPARLAAEMTKR-------HDASHMSVVFST
gi|740989872|ref|WP_038773225.1|damageinducibleprotein[Burkholderiapseudomallei]_gi|705645291|gb|KGS70691.1|DEAD/DEAHboxhelica/    GKKR-K----KEDDMVQTFVHELRYPATTDSARLAAEMEKR-------HDASHMSVVFST
gi|654912525|ref|WP_028363424.1|damageinducibleprotein[Burkholderiasp.JPY366]/11659    GKKR-K----KQDDMVQTFVHELRYPATTDSARLAAEMAKR-------HDASHMSVVFST
gi|740955879|ref|WP_038740512.1|damageinducibleprotein[Burkholderiapseudomallei]_gi|714684932|gb|KGW74438.1|DEAD/DEAHboxhelica/    GKKR-K----KEDDMVQTFVHELRYPATTDSARLAAEMTKR-------HDASHMSVVFST
gi|919203714|ref|WP_052759215.1|damageinducibleprotein[Xanthomonasaxonopodis]/11388    GKHS-----------EDIAVHDLAFPATTDAKRLGQVMVAR------PQGDTRRTVIFST
gi|522152381|ref|WP_020663589.1|hypotheticalprotein[Amycolatopsisbenzoatilytica]/11605    GKRSE---------SEDIGVHDLAFPATTDTAKLVAQVART-------DGATPLTVIFST
gi|663196013|ref|WP_030233899.1|damageinducibleprotein[Streptomycessp.NRRLS455]/11641    SRGRV------DADDLDMSTHDLAMPATTDPARLLAQMSGDG-----GPVPAGLTVIFST
gi|750429553|ref|WP_040711444.1|damageinducibleprotein[Nocardiopsispotens]/11622    GKQKA------QDDDKDMATHDLALPATTSPDRLIEQMNRV-------ETEPGLTVVFST
gi|518656139|ref|WP_019819475.1|hypotheticalprotein[Saccharomonosporasaliphila]/11623    SKQKA------QGDDKDMATHELALPATTDPDRLVTQIARV-------EAMPGLTVVFST
gi|759722933|ref|WP_043433758.1|damageinducibleprotein[Streptomycespluripotens]_gi|733404978|gb|KHK94361.1|damageinduciblepro/1    GKQKA------QGDDKDMSTHDLALPATTSPDRLIAQMASV-------EAVPGLTVVFST
gi|663424167|ref|WP_030419030.1|damageinducibleprotein[Streptomycessp.NRRLF5065]/11627    GKQKA------QGDDKDMSTHDLALPATTSPDRLMAQMASV-------EAAPGLTVVFST
gi|663412917|ref|WP_030408353.1|damageinducibleprotein[Streptomycessp.NRRLF5917]/11630    GKQKA------QGDDKDMSTHDLALPATTDPDRLVAQMASV-------EAVPGLTVVFST
gi|671521483|ref|WP_031506489.1|damageinducibleprotein[Streptomycesmegasporus]/11630    GKQKA------QGDDKDMSTHDLALPATTDPDRLVAQMASV-------EAVPGLTVVFST
gi|740053144|ref|WP_037902776.1|damageinducibleprotein[Streptomycessp.TAA204]/11626    GKQQT------RGDDEDMAAHDLALPATTDPDQLIKQTARV-------EAAPGLTVVFST
gi|663409375|ref|WP_030404974.1|MULTISPECIES:damageinducibleprotein[Streptomyces]/11627    GKQQA------KGDDKDMATHDLALPATTDPDRLIQQMASV-------EAAPGLTVVFST
gi|746628608|ref|WP_039654277.1|damageinducibleprotein[Streptomycessp.MUSC125]_gi|745644381|gb|KIE25557.1|damageinducibleprot/1    GKQQP------KGDDKDMAAHDLALPATTDPDRLIQQMASV-------EAAPGLTVVFST
gi|727322066|ref|WP_033777320.1|damageinducibleprotein[Salinisporapacifica]/11627    GKQRAA-----TGDDNDMATYDLALPATTDPDRLIEQMDRV-------EAEPGLTVVFST
gi|653653242|ref|WP_027655726.1|damageinducibleprotein[Salinisporapacifica]/11618    GKQQP------VGDDKDMATYDLALPATTDPDRLIEQMARA-------EVEPGLTVVFST
gi|648632543|ref|WP_026324294.1|damageinducibleprotein[Salinisporapacifica]/11621    GKQQS------AADDKDMATYDLALPATTDPARLIEQMTSV-------AEHPGLTVVFST
gi|881018002|ref|WP_048738463.1|damageinducibleprotein[Corynebacteriumfalsenii]/11676    TK--K---------AEDIAPHDLQVGVSTNGAAIAERFA-H------SRRAKGMHVIFST
gi|505465526|ref|WP_015652417.1|helicase[Corynebacteriumcallunae]_gi|459385704|gb|AGG67995.1|helicase[CorynebacteriumcallunaeDS    SK--A---------AEDIAAYDLEVPVSTDGALIAEKLE-H------RKRAAGLTVVFST
gi|41223062|emb|CAF19007.1|DNAorRNAhelicaseofsuperfamilyII[CorynebacteriumglutamicumATCC13032]/11646    SK--A---------AEDIAAYDLEVPVSTDGALIAEKLE-H------RKRAAGLTVVFST
CGL13032I/11643    SK--A---------AEDIAAYDLEVPVSTDGALIAEKLE-H------RKRAAGLTVVFST
gi|751257081|ref|WP_040967675.1|damageinducibleprotein[Corynebacteriumglutamicum]_gi|748811620|gb|AJE67931.1|damageinduciblep/1    SR--G---------AEDIASYDLEVPVSTNGHDIAERLG-H------RRRAAGLTVVFST
gi|749040539|ref|WP_040086524.1|damageinducibleprotein[Corynebacteriumhumireducens]/11663    SK--A---------AEDIATYDLEVPVSTNGRDIAARLA-Q------RKRAAGLTVVFST
gi|491662899|ref|WP_005519615.1|helicase[Corynebacteriummatruchotii]_gi|224946756|gb|EEG27965.1|helicaseCterminaldomainprotein/    AK--K---------AEDIATYDLEVPVSTNGEDIYKRLS-H------SKRAKGLTVVFST
gi|746717312|ref|WP_039676231.1|damageinducibleprotein[Corynebacteriumminutissimum]_gi|734991118|gb|KHO29219.1|damageinducibl/1    SK--K---------AEDIASYDLEVPVSTNGKDIAERTA-Q------GKRRAGINVIFST
gi|908654892|emb|CQD15529.1|SuperfamilyIIDNA/RNAhelicase(modularprotein)[Corynebacteriumstriatum]/11674    SK--K---------AEDIASYDLEVPVSTNGKNIAERTA-Q------GKRRAGINVIFST
gi|895994446|ref|WP_049064418.1|damageinducibleprotein[Corynebacteriumaurimucosum]/11667    SK--K---------AEDIASYDLEVPVSTNGKNIAERTA-Q------GKRRTGINVIFST
gi|896123331|ref|WP_049145390.1|damageinducibleprotein[Corynebacteriumaurimucosum]/11669    SK--K---------AEDIASYDLEVPVSTNGKNIAERTA-Q------GKRRAGINVIFST
gi|817681178|ref|WP_046645422.1|damageinducibleprotein[Corynebacteriumstriatum]_gi|817551679|gb|KKO79868.1|damageinduciblepro/1    SK--K---------AEDIASYDLEVPVSTNGKNIAERTA-Q------GKRRAGINVVFST
gi|896139239|ref|WP_049159146.1|damageinducibleprotein[Corynebacteriumaurimucosum]/11675    SK--K---------AEDIASYDLEVPVSTNGKNIAERTA-Q------GKRRTGINVIFST
gi|489943947|ref|WP_003847254.1|helicase[Corynebacteriumammoniagenes]_gi|295968218|gb|EFG81467.1|helicaseCterminaldomainprotei/    GR--A---------AEDIAPHDLEIPVSTNGEMISKTFA-S------GKRAQGLHVVFST
gi|516653464|ref|WP_018022453.1|helicase[Corynebacteriumdoosanense]_gi|697778282|gb|AIT61018.1|damageinducibleprotein[Coryneba/    GK--A---------AEDIAPHDLEVPVSTDGKTIAAAFGAG------GKRAQGLHVVFST
gi|493894179|ref|WP_006840129.1|helicase[Corynebacteriumlipophiloflavum]_gi|227078819|gb|EEI16782.1|helicaseCterminaldomainpro/    SK--K---------AEDIASYDLEVPVSTNGAEIASRMS-S------GKRSSGLHVIFST
gi|740831472|ref|WP_038616755.1|damageinducibleprotein[Corynebacteriumpseudotuberculosis]_gi|666103058|gb|AIG06935.1|putativeh/    GR--Q---------AEDIASYDVEIPVSTDGKTLAEAFE-T------GKRSKGLHVVFST
gi|518023322|ref|WP_019193530.1|helicase[Corynebacteriumtimonense]/11655    SR--A---------AEDIAPYDLEVPVSTSGGKLAEIFA-S------GKHAKGLHVVFST
```

```
gi|552767697|ref|WP_023019559.1|MULTISPECIES:hypotheticalprotein[Corynebacterium]_gi|550739013|gb|ERS38609.1|hypotheticalprotei      GK--G---------AEDIAPYDLEVPVSTDGTRLAQIFN-T------GKHGAGLHVVFST
gi|652659121|ref|WP_027017812.1|damageinducibleprotein[Corynebacteriumpseudodiphtheriticum]/11644                                    GK--G---------AEDIAPYDLEVPVSTNGKRLAEVFN-T------GKHSAGLHVVFST
gi|749039309|ref|WP_040085390.1|damageinducibleprotein[Corynebacteriumhumireducens]/11657                                            NK--R---------AEDIASYDLEIPVSTDGTAIARRFE-R------GRRRAGLDVVFST
gi|740835591|ref|WP_038620874.1|damageinducibleprotein[Corynebacteriumulcerans]_gi|698973854|gb|AIU33664.1|HelicaseCterminald/1      GK--Q---------AEDIAIHDLEVPVSTTGADIAARFQ-H------GKRAAGLNVVFST
gi|805307599|ref|WP_046096452.1|damageinducibleprotein[Corynebacteriumulcerans]_gi|803357323|gb|AKA97638.1|Damageinduciblepro/1      GK--Q---------AEDIAIHDLEVPVSTTGADIAARFQ-H------GKRAAGLNVVFST
gi|504083555|ref|WP_014317549.1|helicase[Corynebacteriumdiphtheriae]_gi|371584249|gb|AEX47914.1|superfamilyIIDNA/RNAhelicase[Co      SK-KK---------TEDISSYDLEVPVSTNGADIAERFS-V------GKRAKGLNVVFST
gi|896182193|ref|WP_049193580.1|damageinducibleprotein[Corynebacteriumjeikeium]/11645                                                SK--K---------AEDISSYDLEVPVSTNGADIAERFS-V------GKRAKGLNVVFST
gi|917636459|ref|WP_052205460.1|damageinducibleprotein[Corynebacteriumriegelii]_gi|914295811|gb|AKV59187.1|damageinduciblepro/1      SR--Q---------AEDIASYDLEVPVSTNGKDIADRFA-S------GRRAKGLNVVFST
gi|737013522|ref|WP_035009205.1|damageinducibleprotein[Corynebacteriumjeikeium]/11651                                                SR--Q---------AEDIASYDLEVPVSTEGKDIAARFA-Q------GKRRDGLHVVFST
gi|737006291|ref|WP_035002094.1|damageinducibleprotein[Corynebacteriumjeikeium]/11658                                                SR--S---------AEDIATYDLEVPVSTDGAQIAERFA-S------GKRAKGLHVVFST
gi|737010378|ref|WP_035006133.1|damageinducibleprotein[Corynebacteriumjeikeium]/11650                                                SR--S---------AEDIATYDLEVPVSTEGSQIAERFA-S------GKRAKGLHVVFST
gi|737010473|ref|WP_035006228.1|damageinducibleprotein[Corynebacteriumjeikeium]/11657                                                SK--K---------AEDIATYDLEVPVSTQGADIAERLLRG------GKRAAGLHVVFST
gi|737006250|ref|WP_035002053.1|damageinducibleprotein[Corynebacteriumjeikeium]/11658                                                SK--K---------AEDIATYDLEVPVSTQGADIAERLLRG------GKRAAGLHVVFST
gi|923018168|ref|WP_053412254.1|damageinducibleprotein[Corynebacteriumlactis]_gi|922845184|gb|ALA67504.1|helicase[Corynebacter/      SR--K---------AEDIATYDLEVPVSTDGVEIAQRFA-S------GKRAKGLNIVFST
gi|923018885|ref|WP_053412970.1|damageinducibleprotein[Corynebacteriumlactis]_gi|922845845|gb|ALA68165.1|helicase[Corynebacter/      SR--K---------AEDIATYDLEVPVSTDGAEIAKRFA-S------GKRAKGLNIVFST
gi|512464870|ref|WP_016422869.1|hypotheticalprotein[Corynebacteriumsp.HFH0082]_gi|512047830|gb|EPD47505.1|hypotheticalproteinHM      SR--K---------AEDIATYDLEVPVSTNGAEIAKRFE-C------GKRAKGLHIVFST
gi|881012697|ref|WP_048733406.1|damageinducibleprotein[Corynebacteriumurealyticum]/11670                                             SR--K---------GEDIATYDLEVPVSTNGVEIAKRFE-S------GKRAKGLHIVFST
gi|755608478|ref|WP_042530519.1|damageinducibleprotein[Corynebacteriumsingulare]_gi|754340239|gb|AJI78730.1|putativehelicase[C/      SK--K---------AEDIAVYDLEVPVSTEGKDITTRFE-S------GKRAKGLNIVFST
gi|491421796|ref|WP_005279593.1|helicase[Corynebacteriumaccolens]_gi|227077180|gb|EEI15143.1|helicaseCterminaldomainprotein[Co/      SK--K---------AEDIAVYDLEVPVSTDGADIAKRFA-S------GKRAKGLNIVFST
gi|550752286|gb|ERS51451.1|hypotheticalproteinHMPREF1282_00033[Corynebacteriumsp.KPL1856]_gi|550759961|gb|ERS58964.1|hypothetic      SK--K---------AEDIAVYDLEVPVSTDGEEITQRFA-S------GKRAKGLNIVFST
gi|497955053|ref|WP_010269209.1|helicase[Corynebacteriumbovis]/11599                                                                 TR--A---------AEDIATYDLEIPVTTDGATLAERL-RT------RRRAAGLSVVFAT
gi|860580040|ref|WP_048470569.1|hypotheticalprotein[Mycobacteriumchlorophenolicum]_gi|859098575|gb|KMO76871.1|typeIrestrictione      GRLRN---------AEDFNVYDVPIPVTTNAAKLVDEM-SY------RKRAKGLTIVFST
gi|262085905|gb|ACY21873.1|typeIIIrestrictionproteinressubunit[GordoniabronchialisDSM43247]/11632                                    SR--A---------AEDVSVVDVAIPVTTDGPTLAAHM-EH------RKRARGLTVVFTT
gi|495996338|ref|WP_008720917.1|typeIIIrestrictionendonucleasesubunitR[Rhodococcussp.AW25M09]_gi|443412350|emb|CCQ18455.1|TypeI      SR--S---------AEDYSTVDVAIPVTTDPDNLASHM-AH------RKRSKGLTVVFTT
gi|739284636|ref|WP_037147410.1|damageinducibleprotein[Rhodococcusfascians]/11641                                                    SR--S---------AEDYSTVDVAIPVTTDPDNLASHM-AH------RKRSKGLTVVFTT
gi|739328195|ref|WP_037190574.1|damageinducibleprotein[Rhodococcusfascians]/11643                                                    SR--S---------AEDYSTVDVAIPVTTDPDNLASHM-AH------RKRSKGLTVVFTT
gi|811698335|emb|CMN21041.1|helicase[Mycobacteriumtuberculosis]/11725                                                               SR--S---------AEDYHVHDVPIPVTTDARVLLHEM-AH------RRRAQGLTVVFCT
gi|801577898|emb|CFK23773.1|helicase[Mycobacteriumtuberculosis]/11675                                                               SR--S---------AEDYHVHDVPIPVTTDARVLLHEM-AH------RRRAQGLTVVFCT
MTU18II/11606                                                                                                                        SR--S---------AEDYHVHDVPIPVTTDARVLLHEM-AH------RRRAQGLTVVFCT
gi|887719858|emb|CMT27886.1|helicase[Mycobacteriumtuberculosis]/11667                                                               SR--S---------AEDYHVHDVPIPVTTDARVLLHEM-AH------RRRAQGLTVVFCT
gi|621694743|gb|KAS87156.1|hypotheticalproteinAL70_01801[MycobacteriumtuberculosisTKK_03_0115]/11604                                 SR--S---------AEDYHVHDVPIPVTTDARVLLHEM-AH------RRRAQGLTVVFCT
gi|888128072|emb|CLQ84422.1|helicase[Mycobacteriumtuberculosis]_gi|889917090|emb|CLY86893.1|helicase[Mycobacteriumtuberculosis]      SR--S---------AEDYHVHDVPIPVTTDARVLLHEM-AH------RRRAQGLTVVFCT
gi|884823424|emb|CKL96672.1|helicase[Mycobacteriumtuberculosis]/11466                                                               SR--S---------AEDYHVHDVPIPVTTDARVLLHEM-AH------RRRAQGLTVVFCT
gi|505103239|ref|WP_015290341.1|hypotheticalprotein[Mycobacteriumcanettii]_gi|432162733|emb|CCK60117.1|Conservedproteinofunknow      SR--S---------AEDYHVHDVPIPVTTDARVLLHEM-QH------RRRAQGLTVVFST
gi|505116250|ref|WP_015303352.1|hypotheticalprotein[Mycobacteriumcanettii]_gi|432158726|emb|CCK56026.1|Conservedproteinofunknow      SR--S---------AEDYHVHDVPIPVTTDARVLLHEM-AH------RRRAQGLTVVFCT
gi|432154735|emb|CCK51974.1|Conservedproteinofunknownfunction(part1)[MycobacteriumcanettiiCIPT140060008]/11588                      SR--S---------AEDYHVHDVPIPVTTDARVLLHEM-AH------RRRAQGLTVVFCT
gi|809008027|ref|WP_046295375.1|hypotheticalprotein[Mycobacteriumsp.UM_Kg17]/11624                                                  SR--T---------VEDYNVHDVPIPVTTDPQRLADEM-AH------RKRAKGLTVVFST
gi|808802633|ref|WP_046284139.1|hypotheticalprotein[Mycobacteriumsp.UM_NZ2]/11624                                                   SR--T---------VEDYNVHDVPIPVTTDPARLADEM-AH------RKRAKGLTVVFST
gi|750125774|ref|WP_040429852.1|damageinducibleprotein[Corynebacteriumnuruki]/11668                                                  SR--A---------TEDMSTEDVIIPVTTDGTVLAEKLGGH------RRRAKGLTVVFTT
gi|602237614|gb|EYT60960.1|damageinducibleprotein[Dietziasp.UCDTHP]/11473                                                          SR--A---------AEDYATEDVAIPVTTDPAVLAERM-GK------GKRAAGLTVVFTT
gi|665897412|ref|WP_031264618.1|damageinducibleprotein[Dietziasp.UCDTHP]_gi|602237599|gb|EYT60945.1|helicase[Dietziasp.UCDTH/11      SR--A---------AEDYATEDVAIPVTTNPAVLAERM-GK------GKRAAGLTVVFTT
gi|502903674|ref|WP_013138650.1|helicase[Segniliparusrotundus]_gi|296181291|gb|ADG98197.1|typeIIIrestrictionproteinressubunit[S      SR--A---------AEDSKAHDVAIPVTTKPDQLAAAI-AA------HGESPGLTVVFTT
gi|780248306|ref|WP_045538930.1|hypotheticalprotein[Gordoniasp.no.9]_gi|778142514|gb|KJR06917.1|hypotheticalproteinUG54_12320[G      SR--A---------AEDSRTYDVAIPVTTDPAKLAESL-AT------ADDSDGLTVVFTT
gi|665897368|ref|WP_031264574.1|damageinducibleprotein[Dietziasp.UCDTHP]_gi|602239137|gb|EYT62483.1|helicase[Dietziasp.UCDTH/11      SL--A---------AEDYDTSDVAIPVTTNPEQLAEKM-SH------RKRAAGMTVVFTT
gi|493372923|ref|WP_006329236.1|helicase[Gordoniarhizosphera]_gi|403207917|dbj|GAB88168.1|hypotheticalproteinGORHZ_006_00370[Go      AR--A---------AEDVAAHDLAIPVTTDGATLAAEL-AH------RKRAKGLTVVFTT
gi|764904870|ref|WP_044487821.1|hypotheticalprotein[Mycobacteriumtuberculosis]_gi|625005353|gb|KBZ57120.1|hypotheticalproteinK8      SR--A---------AEDINTYEVAIPVTTNAAKLVHEM-EH------RKRAKGLTVVFTT
gi|927068212|ref|WP_053778441.1|damageinducibleprotein[Gordoniasp.NB41Y]_gi|926660541|gb|EMP14526.2|damageinducibleprotein[G/11      SR--A---------AEDISSYDVAVPVTTNADRLVGEM-SH------RKRAKGLTVVFTT
gi|935514169|ref|WP_054429894.1|damageinducibleprotein[Mycobacteriumimmunogenum]_gi|929801120|gb|KPG29247.1|damageinduciblepr/1      SR--A---------AEDINVYDVAIPVTTNAAKLADEM-EH------RKRAKGLTVVFTT
gi|941317728|ref|WP_055118574.1|hypotheticalprotein[Mycobacteriumperegrinum]/11657                                                  SR--A---------AEDINVYDVAIPVTTNAAKLADEM-EH------RKRAKGLTVVFTT
gi|933683673|gb|ALG86377.1|hypotheticalproteinACH46_20120[Gordoniasp.QH11]/11636                                                    SR--S---------AEDIGSVDVAIPVTTNPDTLAGEM-GH------RKRAKGLTVVFTT
gi|873553947|emb|CRY84110.1|Predictedhelicase[Nocardiafarcinica]/11688                                                              SR--S---------AEDVSVHDVAIPVTTSSAVLAAEM-ER------RRHAAGLTVVFTT
gi|499525749|ref|WP_011212389.1|helicase[Nocardiafarcinica]_gi|54019339|dbj|BAD60707.1|hypotheticalprotein[NocardiafarcinicaIFM      SR--S---------AEDVSVHDVAIPVTTSSAVLAAEM-ER------RRHAAGLTVVFTT
gi|640109609|ref|WP_024794187.1|damageinducibleprotein[Tomitellabiformata]/11653                                                    SR--S---------AEDINAYDVAIPVTTDPATLTVEM-GH------RKRAKGLTVVFTT
gi|640109991|ref|WP_024794334.1|hypotheticalprotein[Tomitellabiformata]/11639                                                       SR--S---------AEDINAYDVAIPVTTDPIKLVAEM-GH------RKRAKGLTVVFTT
gi|939125496|ref|WP_054831438.1|damageinducibleprotein[Rhodococcuserythropolis]/11669                                                SR--S---------AEDVNIHDVAIPVTTDPVKLAAEM-AH------RKRAKGLTVVFTT
gi|910258550|ref|WP_050062711.1|hypotheticalprotein[Rhodococcussp.RD6.2]_gi|846871967|emb|CRK49225.1|conservedhypotheticalprote      SR--S---------AEDVAVHDVAIPVTTDAATLVAEM-GH------RKRAKGLTVVFTT
gi|797148189|gb|KJY52358.1|putativeHelicase[Bifidobacteriumasteroides]/11801                                                        NKPRGED-----NDAWSSSVLDVPFPTTTNAATLKRNVEI-------GSIKAGLSVVFST
gi|535476570|gb|EFG27357.2|hypotheticalproteinHMPREF9020_00999[ScardoviainopinataF0304]/11827                                        NK---RD-----NDEWDGSVLDVPFPTTTDPRLLADHIKI-------GEQSTGLSVVFST
gi|737482729|ref|WP_035462496.1|hypotheticalprotein[Alicyclobacillusmacrosporangiidus]/11512                                         GRNDE------------TGDISLVIPPTTNADMLARAAKP---------VLDAITVVVST
gi|685434354|gb|KGA95679.1|helicase[BacillusalcalophilusATCC27647=CGMCC1.3604]/11575                                                 TKA------NGDNELEDIASADLGYPATTDNKKLLDYQVQIE----NSVLAGDFLTVFST
gi|945370253|gb|KQU27251.1|helicase[Bacillussp.Leaf13]/11557                                                                         TKKE------SNDDVADINVADIGYPATTNKNKLLEYQSRIE----NSNNKGDFLTVFST
gi|880997015|ref|WP_048719116.1|helicase[Enterococcushirae]/11563                                                                    TKK----I-TGENELEDIAAADLGYPATTSHERLLDYQKEID----EQTDKTQFLAVFST
gi|945356929|gb|KQU14100.1|helicase[Bacillussp.Leaf49]/11547                                                                         TKTETKKQ-SEDMDVADIAVADIGFPATTNYEKLLSYQEIIE----NSTEKGNFLTVFST
gi|763482876|gb|KIZ53217.1|helicase[Bacillussafensis]/11563                                                                          TKK----T-SGENEFEDIAAADLGYPATTDYQKLLDRQNVID----LSDNPSKFLVVFST
gi|852223292|ref|WP_048309707.1|helicase[Anaerobacillusmacyae]_gi|850322910|gb|KMM38590.1|helicase[Anaerobacillusmacyae]/11573      TKK----V-KGENEMEDIAAADLGYPATTDYNKLLERQKEID----AADNQSAFLVVFST
gi|739708665|ref|WP_037562619.1|helicase[Sporolactobacillusterrae]/11573                                                             TKK----V-KGENEFEDIAAADLGYPATTDYHKLLEHQKVID----SSDSQSKFLVVFST
gi|496687561|ref|WP_009329104.1|MULTISPECIES:helicase[Bacillus]_gi|317390867|gb|EFV71668.1|endonucleaseandmethylaseLlaGI[Bacill      TKK----A-KGENEFEDIAAADLGYPATTDYHKLLERQRVID----SADTQSKFLVVFST
```

```
gi|930213599|gb|KPH74225.1|helicase[Oceanobacilluscaeni]/11569      TKK----A-KGESEIEDIAAADLGYPATTDYQKLLERQKVID----SANNQSKFLVVFST
gi|516366003|ref|WP_017756036.1|helicase[Bacillussp.ZYK]/11571      TKK----V-KGENEFEDIAAADLGYPATTDYQKLLQRQKDID----TADSQSKFLVVFST
gi|488909977|ref|WP_002821052.1|helicase[Oenococcusoeni]_gi|399965230|gb|EJN99855.1|endonucleaseandmethylaseLlaGI[Oenococcusoen      TKHK--------NEDEDLAPEDIGFPATTDDRQLLKNLHSLK----TS-SQKRLLVVFST
gi|495019967|ref|WP_007745780.1|helicase[Oenococcuskitaharae]_gi|366983682|gb|EHN59081.1|typeIIIrestrictionenzyme,ressubunit[Oe      TKLK--------NDDEDLAPEDIGFPATTDDQQLLKNLHSLK----AS-SQKRLLVVFST
gi|489666119|ref|WP_003570406.1|typeIIIrestrictionendonuclease[Lactobacilluscasei]_gi|410527834|gb|EKQ02696.1|typeIIIrestrictio      SKKRTYSN---SD-DSDMNVQDIGFPASTSAEKLMANYHALQ----TD-AGSRITVIFST
gi|511447623|gb|EPC78110.1|superfamilyIIDNA/RNAhelicase[Lactobacillusparacaseisubsp.paracaseiLpp221}/11615      SKKRTYNN---ND-DSDMNIQDIGFPASTSADKLMSNFSMLK----TI-ADPRITVIFST
gi|497700411|ref|WP_010014595.1|helicase[Lactobacilluscoryniformis]/11585      SKKRTYNN---ND-DSDINIQDIGFPASTNADKLMANFKALT----PT-SGQRITVIFST
gi|489784048|ref|WP_003687939.1|helicase[Lactobacillusmali]_gi|394428539|gb|EJF01080.1|superfamilyIIDNA/RNAhelicase[Lactobacill      SKKRTYSN---NDDNSDMNIQDIGFPASTNADKLMANFAALK----PT-DGQRITVIFST
gi|446780825|ref|WP_000858081.1|helicase[Staphylococcusaureus]_gi|374396604|gb|EHQ67835.1|typeIIIrestrictionenzyme,ressubunit[S      TKKKT------DSDDLDVLAIDIGFPATTNEKEILSNYNSIK----YDNEKITMNVIFST
gi|510796658|ref|WP_016171456.1|hypotheticalprotein[Enterococcusdispar]_gi|508167532|gb|EOT44083.1|hypotheticalproteinOMK_00227      TKKKG------NDDDADIQATDIGFPATTRATDIVKNYESSL----STNEKIRLNVVFST
gi|612613473|gb|EZS30411.1|hypotheticalproteinW603_02482[StaphylococcusaureusVET0353R]_gi|612881917|gb|EZU93992.1|hypotheticalp      TKKKA------N-DDADIQATDIGFPATTKAMDIVKNYKSLP----ETSNKIRLNVVFST
gi|488332893|ref|WP_002402278.1|helicase[Enterococcusfaecalis]_gi|306513864|gb|EFM82466.1|helicaseCterminaldomainprotein[Enter/      SKKKN------A---DELSAKDIGFPATTDSEKLLSNYKKIE----N--NQRDLTVVYST
gi|921166029|ref|WP_053111806.1|helicase[Enterococcusdurans]_gi|915771660|gb|AKX85928.1|helicase[Enterococcusdurans]/11562      SKKKN------A---DELSAKDIGFPATTDSEKLLSNYKKIE----N--NQRDLTVVYST
gi|498525358|ref|WP_010825030.1|hypotheticalprotein[Enterococcusfaecalis]_gi|487059631|gb|EOJ86727.1|hypotheticalproteinWOC_005      SKKKN------A---DELSAKDIGFPATTDSEKLLSNYKKIE----N--NQRDLTVVYST
gi|498412624|ref|WP_010722070.1|hypotheticalprotein[Enterococcusfaecium]_gi|486634654|gb|EOF75253.1|hypotheticalproteinSGC_0253      SKKKN------D---DELSAKDIGFPATTDSEKLLSNYKKIE----N--NQRDLMVVYST
gi|498408534|ref|WP_010718364.1|hypotheticalprotein[Enterococcusfaecium]_gi|486609608|gb|EOF50873.1|hypotheticalproteinSCW_0257      SKKKN------A---DELSAKDIGFPATTDSEKLLSNYKKIE----N--NQRDLTVVYST
gi|498407475|ref|WP_010717483.1|hypotheticalprotein[Enterococcusfaecalis]_gi|486594855|gb|EOF36348.1|hypotheticalproteinSCK_005      SKKKN------A---DELSAKDIGFPATTDSEKLLSNYKKIE----N--NQRDLTVVYST
gi|514885392|ref|WP_016615344.1|helicase[Enterococcusfaecalis]_gi|514505843|gb|EPH71676.1|helicaseprotein[Enterococcusfaecalis0      SKKKN------A---DELSAKDIGFPATTDSEKLLSNYKKIE----N--NQRDLTVVYST
gi|325157603|gb|EGC69759.1|helicaseCterminaldomainprotein[EnterococcuscasseliflavusATCC12755]/11561      SKKKN------A---DELSAKDIGFPATTDSDKLLSNYKKIE----N--NQRDLTVVYST
gi|895856206|ref|WP_048967668.1|helicase[Enterococcusfaecalis]/11551      SKKKN------D---DELSAKDIGFPATTDSEKLLSNYKKIE----N--NQRDLTVVYST
gi|639458836|ref|WP_024636366.1|helicase[Enterococcusfaecium]_gi|658176502|gb|KEI51159.1|helicase[EnterococcusfaeciumUC7256]/1/      SKKKN------A---DELSAKDIGFPATTDSDKLLSNYKKIE----N--NQRDLTVVYST
gi|488266511|ref|WP_002337719.1|hypotheticalprotein[Enterococcusfaecium]_gi|430581409|gb|ELB19854.1|hypotheticalproteinOIQ_0351      SKKKN------A---DELSAKDIGFPATTDSDKLLSNYKKIE----N--NQRDLTVVYST
gi|446353356|ref|WP_000431211.1|helicase[Staphylococcusaureus]_gi|253726329|gb|EES95058.1|helicaseCterminaldomainprotein[Staph/      SRGDD------G--TEDIKATDIGYPATTSKDQLLKNWNDVQ----KKESNTDLFVVFST
gi|672712068|emb|CDM14336.1|DNAorRNAhelicaseofsuperfamilyII[StaphylococcusepidermidisPM221]/11464      SRGSD------G--TEDIKATDIGYPATTSKDQLLKNWSDIK----ETQSDRDMTVVFST
gi|896298990|ref|WP_049279905.1|helicase[Staphylococcusaureus]/11571      SRGSD------G--TEDIKATDIGYPVTTSKDQLLKNWNDIK----ETQSDRDMTVVFST
gi|541903432|gb|ERH36190.1|hypotheticalproteinSEQU_00085[StaphylococcusequorumUMCCNS924]/11578      SRGRD------G--TEDIKATDIGYPATTSKDQLLKNWNDVK----ETQSDRDMTVVFST
gi|488289747|ref|WP_002360955.1|helicase[Enterococcusfaecalis]_gi|256682216|gb|EEU21911.1|endonucleaseandmethylaseLlaGI[Enteroc      SRGED------G--TEDIKASDIGYPATTSTEKLLKNWEDMK----KTQQTADMIVVFST
gi|494392045|ref|WP_007207894.1|helicase[Enterococcusitalicus]_gi|315483798|gb|EFU74284.1|helicaseCterminaldomainprotein[Enter/      SRGED------G--TEDIKASDIGYPATTSKEKLLKNWEDVK----KTQQTADMVVVFST
gi|446350692|ref|WP_000428547.1|helicase[Bacilluscereus]_gi|228727580|gb|EEL78721.1|RestrictionmodificationsystemLlaBIII[Bacil/      SRGTN------G--TEDIKANDIGYPATTSKEQLLKNWKDIE----EVQQNADMVVVFST
gi|488255302|ref|WP_002326510.1|hypotheticalprotein[Enterococcusfaecium]_gi|430480276|gb|ELA57459.1|hypotheticalproteinOGE_0473      SRGND------G--TEDIKATDIGYPATTSKEQLLKNWHDVE----ETQKDSDIFVVFST
gi|843253770|ref|WP_047916698.1|helicase[Lactococcuspiscium]_gi|802124677|emb|CEN29502.1|EndonucleaseandmethylaseLlaGI,TypeISPr      SRGTD------G--TEDIKASDIGYPATTSSKKILQNWHDFE----SLPKPTDMLVVFST
LlaBIII/11578      SRGTD------G--TEDIKASDIGYPATTSSKKILQNWHDFE----SLPKQTDMLVVFST
gi|499320531|ref|WP_011011023.1|helicase[Listeriainnocua]_gi|16415805|emb|CAC42019.1|pli0021[ListeriainnocuaClip11262]/11569      SRGTD------G--TEDIKASDIGYPATTSSKKILQNWHDFE----SLPKPTDMLVVFST
gi|760026859|ref|WP_043709973.1|helicase[Weissellacibaria]_gi|758337165|gb|KIU20061.1|UvsWhelicase[Weissellacibaria]/11567      SRGTD------G--NEDIKAADIGYPATTSSQKILQNWHDFE----K-GNPTDLVVIFST
LlaGI/11570      SRGTD------G--TEDIKASDIGYPATTSSKKILQNWHDFE----SLPKQTDMLVVFST
gi|499988414|ref|WP_011669132.1|helicase[Lactococcuslactis]_gi|116109019|gb|ABJ74139.1|DNAorRNAhelicaseofsuperfamilyII[Lactococ      SRGTD------G--TEDIKASDIGYPATTSSKKILQNWHDFE----SLPKPTDMLVVFST
gi|685938360|ref|WP_031670319.1|helicase[Listeriamonocytogenes]_gi|590403387|gb|EXL23261.1|hypotheticalproteinX846_1956[Listeri      SRGTD------G--TEDIKASDIGYPATTSSKKILQNWHDFE----SLSKPTDMLVVFST
gi|530793219|gb|EQC56240.1|helicase[Lactococcuslactissubsp.cremorisTIFN5]_gi|530793247|gb|EQC56263.1|helicase[Lactococcuslactis      SRGTD------G--TEDIKASDIGYPATTSSKKILQNWHDFE----SLPKQTDMLVVFST
gi|695270006|ref|WP_032492075.1|hypotheticalprotein[Listeriamonocytogenes]_gi|695212872|ref|YP_009081522.1|hypotheticalproteinp      SRGTD------G--TEDIKASDIGYPATTSSKKILQNWHDFE----SLSKPTDMLVVFST
gi|504101310|ref|WP_014335297.1|helicase[Leuconostocmesenteroides]_gi|859247905|gb|AKO63885.1|helicase(plasmid)[Leuconostocmese      SRGTD------G--NEDIKAADIGYPATTSSQKILQNWRDFE----K-GQSTDMVVIFST
gi|488905190|ref|WP_002816265.1|helicase[Leuconostocmesenteroides]_gi|227351522|gb|EEJ41777.1|helicaseCterminaldomainprotein[L/      SRGTD------E--NEDIKAADIGYPATTSSQKILHNWRDFE----K-GQSTDMVVIFST
gi|518121285|ref|WP_019291493.1|MULTISPECIES:hypotheticalprotein[Lactococcus]/11588      SRQSLDE----EEKNLFVKAQDIGFPATTSTAQVLKNYEEIK----KA-EPTNLLVVFST
gi|754857213|ref|WP_042217902.1|helicase[Lactococcusgarvieae]/11549      SRGTVKQ----DESNLFVKASDIGFPATTSTEKVLENYKELE----NS-APTELLVVYST
gi|640048455|ref|WP_024784762.1|helicase[Streptococcusmutans]/11564      TRDSVKQ----DESNVFVKASDIGYPATTSAQKVVKNYEELM----KQ-PKKELLVVFST
gi|512695361|ref|WP_016476323.1|hypotheticalprotein[Veillonellasp.HPA0037]_gi|512079853|gb|EPD79018.1|hypotheticalproteinHMPREF      SRGSIKQ----DESNITVKASDIGYPATTSSKKVVENYQALM----KQ-PKKDLLVVFGT
gi|636963514|ref|WP_024403747.1|helicase[Streptococcussuis]/11574      SRGSVKKKDKDEETYIQIKASDIGYPATTSAKKVVENYEELM----TQ-PKKELLVVFGT
gi|881115312|ref|WP_048802949.1|helicase[Staphylococcuspasteuri]/11573      SRGKVKD----DESNIKFKASDIGYPATTSVEQVVKNYNELV----GQ-PKKDLLVVFST
gi|446063996|ref|WP_000141851.1|helicase[Streptococcusmitis]_gi|307618725|gb|EFN97864.1|endonucleaseandmethylaseLlaGI[Streptoco      SRGSVKQ----DESNLTIKASDIGYPATTSSKKVVENYLELM----TR-PKKELLVVFGT
gi|488206609|ref|WP_002277817.1|restrictionmodificationsystemLlaBIII[Streptococcusmutans]_gi|449173033|gb|EMB75626.1|restricti/      SRESVKQ----DESNIQIKASDIGYPATTSAQKVLENYKELM----TK-PKKELLVVFGT
gi|640023290|ref|WP_024781621.1|helicase[Streptococcusmutans]/11566      SRESVKQ----DESNIQIKASDIGYPATTSAQKVLENYKELM----TK-PKKELLVVFGT
gi|814946581|ref|WP_046393781.1|helicase[Streptococcusuberis]_gi|810600885|gb|KKF57499.1|helicase[StreptococcusuberisB362]/1156      SRGSVKQ----DESNFVIKASDIGYPATTSAKKVVDNYQELM----QL-PKKDLLVVFGT
gi|765470762|ref|WP_044720336.1|helicase[Streptococcussuis]/11559      SRGSVKN----DESNIQIKASDIGYPATTSSKKVVENYEELM----KQ-PKKELLVVFGT
gi|654507127|ref|WP_027976443.1|helicase[Streptococcusdevriesei]/11558      SRGSVKQ----DESNITIKASDIGYPATTSSKKVVENYQELM----EH-PKKELLVVFGT
gi|530812902|gb|EQC72481.1|DNAorRNAhelicaseofsuperfamilyII[Streptococcussp.HSISS2]/11570      SRGTVKQ----DESNLTIKASDIGYPATTSAEKVVENYQELM----EY-PKKEMLVVFGT
gi|489158846|ref|WP_003068504.1|helicase[Streptococcusintermedius]_gi|355367114|gb|EHG14827.1|hypotheticalproteinHMPREF9682_002      SRRSIKKQDKDEETYIQVKATDIGYPATTSAKKVVENYQELM----GR-PKKELLVIFGT
RM.Lru27782ORF13200P/11620      TK-KT-G-------DDNLTAKDVGFPATTNVENLMENYETID----TS-KGRDMTVIFST
gi|492676546|ref|WP_005919236.1|DNAphosphorothioationsystemrestrictionenzyme[Pediococcusacidilactici]_gi|482517924|gb|EOA08103.      TKQKH-G-------KDDLSAKDIGFPATTNVDDLLSNFQKIK----QN-ENYQMNIIFST
gi|498183116|ref|WP_010497272.1|helicase[Lactobacillusacidipiscis]/11563      TKKKT-G-------DEDLSAKDIGFPATTNVAELMENYSAIT----RH-TQKKMNVVFST
gi|822100485|ref|WP_046921865.1|helicase[Lactobacillusruminis]_gi|821684442|gb|KLA43321.1|restrictionmodificationsystemLlaBIII/      TKKKQ-G-------EDDLSARDVGFPATTNVNELMANYNSIR----ET-SSKTMTVVFST
gi|935565832|ref|WP_054450311.1|helicase[Lactobacilluskunkeei]/11573      TKKGKES-------DDDLSSKDIGFPATTDVNELIKNYEYVK----ELDTGREMTVIFST
gi|752296887|ref|WP_041152901.1|helicase[Lactobacilluskunkeei]_gi|751663520|gb|KIM18358.1|helicase[Lactobacilluskunkeei]/11577      TKKGKES-------EDDLSSKDIGFPATTDVNELIKNYEYVK----ELDTGREMTVVFST
gi|495745291|ref|WP_008469870.1|helicase[Lactobacillushominis]_gi|394484442|emb|CCI81207.1|RestrictionmodificationsystemLlaBII/      NKKKSTN-------DDDLGAIDIGFPEPTTNVEDLVNHYKLIQ----EHKLPNNMTVVFST
gi|918672414|ref|WP_052543134.1|helicase[Lactobacillussp.]/11564      NKQKDKN-------DDDLGAREIGFPEPTTNVQDLVDHYKLIE----EHNLPNDMRVVFST
gi|799141631|ref|WP_045927261.1|helicase[Lactobacilluskullabergensis]_gi|797155335|gb|KJY59264.1|putativerestrictionendonucleas      NKKKQKD-------DDNLGAQDIGFEPTTNVESLVNNYKFSE----AHKLNNDMRVVFST
gi|504379990|ref|WP_014567092.1|helicase[Lactobacillusjohnsonii]_gi|329666726|gb|AEB92674.1|putativerestrictionendonuclease[Lac      NKKKNKD-------DDDLGAREIGFEPTTKVEDLINHYKLIE----SNNLPNDMRVVFST
gi|489880179|ref|WP_003783646.1|DNAdamageinducibleprotein[Actinomycesnaeslundii]_gi|399901561|gb|EJN84440.1|typeIIrestriction/      GKGR-----KRTSLSEDISVVDLAIPATTDGAALADRLTRRPA---PVNGTAPMTVVFST
gi|496524497|ref|WP_009232780.1|DNAdamageinducibleprotein[Actinomycessp.oraltaxon849]_gi|365265413|gb|EHM95177.1|hypotheticalp/      GKGR-----KRTSLSEDISVVDLAIPATTDGAALANRLTRRPA---PVNGTAPMTVVFST
```

gi|915397782|ref|WP_050783167.1|damageinducibleprotein[Actinomycesoris]/11694 GKGR-----KRTSLSEDISVVDLAIPATTDGAALANRLTRRPA---PVNGTAPMTVLFST
gi|502925248|ref|WP_013160224.1|helicase[Propionibacteriumfreudenreichii]_gi|296921272|emb|CBL55824.1|helicase[Propionibacteriu SKQA-----------EDISVHDIPLPT-TDAAKLASLMAAG------GRRGKQMTVVFST
gi|732164352|emb|CEG87481.1|helicase[Propionibacteriumfreudenreichii]/11618 GRQA-----------EDITTHDIPLPT-TDATRLAEGLAGV------GRRGRAMTVVFST
gi|739047402|ref|WP_036925058.1|damageinducibleprotein[Propionibacteriumthoenii]/11596 SRQA-----------EDITVHDIPLPT-TDPDRLVAEMAHG------GRRGKQMTVIFST
gi|739112839|ref|WP_036982533.1|damageinducibleprotein[Propionibacteriumjensenii]/11594 SRQA-----------EDITVHDIPLPT-TDPDRLVAEMARG------GRRGRQMTVIFST
gi|918023558|ref|WP_052314121.1|damageinducibleprotein[Nocardiathailandica]/11612 GRRTS----T--ADTGDISTIDLTEPATTDAQTLVKRVNGG------TEAADRMTVVFST
gi|498380271|ref|WP_010694427.1|DNAdamageinducibleprotein[Saccharopolysporaspinosa]/11605 GRKTV----D--DD-SGMSTIDLTEPATTDAATLAARMRSA------PNVDGRMTVVFAT
gi|749991459|ref|WP_040339809.1|damageinducibleprotein[CandidatusBlastococcusmassiliensis]/11597 GRRTP----S--DD-ADQLPIDLTEPASTDPQTLLSRFEAN------TNADGQMRVVFST
gi|754177801|ref|WP_041796231.1|damageinducibleprotein[Modestobactermarinus]/11592 GRRAP----S--DD-ADQLPIDLTEPASTDPQTLLSRFEAS------ANGDGRMRVVFST
gi|504188248|ref|WP_014375350.1|DNAdamageinducibleprotein[Blastococcussaxobsidens]_gi|378782790|emb|CCG02456.1|putativeHelicas/ GRQAQ----S--DD-ADQLPIDLTEPASTDPETLLARFEQG------TNAAGRMRVIFST
gi|946476713|gb|KRF26798.1|damageinducibleprotein[Phycicoccussp.Soil803]/11609 GKGGS----KPADDPEDIAVTDLIIPATTDTKALAAKVKEA------H-ENDRFTVIFST
gi|700176672|gb|KGN37509.1|DNAdamageinducibleprotein[KnoelliasubterraneaKCTC19937]/11633 GKGGP----KPKDDPEDIAVHDLIIPATTDTKALAAKIKEA------H-EHDKFTVIFST
gi|700168841|gb|KGN29914.1|hypotheticalproteinN802_10215[KnoelliasinensisKCTC19936]/11631 GKGGP----KPKDDPEDISVSDLIIPATTDTKALAAKVKEA------H-ENDRFTVIFST
gi|946426241|gb|KRE76610.1|damageinducibleprotein[Arthrobactersp.Soil763]/11606 TKRS-----ANA-DVEDIATVDLALPATTNIPILAERLRQA------KRDSSSMTVVFST
gi|517318525|ref|WP_018500927.1|hypotheticalprotein[Frankiasp.BCU110501]/11592 TARK-----KKDDAYEDISTIDFALPATTNILELKKRIKDA------EAETNSMTVVFST
gi|499072429|ref|WP_010851327.1|Helicase[Tetrasphaeraelongata]_gi|478756579|emb|CCH71501.1|putativeHelicase[Tetrasphaeraelongat TARS-----K--ATNEDISAVDLALPSTTNVAVLESRWTQA------VGETDSMTVVFAT
gi|750080666|ref|WP_040385350.1|damageinducibleprotein[Demetriaterragena]/11625 TTRS-----K--AVNEDISAVDLPLPATTDVERLVPQLKNA------FAEDDSMTVVFST
gi|752558292|ref|WP_041229523.1|damageinducibleprotein[Dermacoccusnishinomiyaensis]_gi|664689315|gb|AIF41904.1|damageinducibl/1 TRRS-----D--A-NEDISAVDVALPATTNLAVLTERWSKA------IADADSMTVVFST
gi|829089213|ref|WP_047310701.1|damageinducibleprotein[Dermacoccussp.PE3]_gi|828384812|gb|KLO63770.1|damageinducibleprotein[D/1 TSRS-----N--A-VEDISAVDVALPASTNNAVVQERWARA------TERSESMTVVFST
gi|748756635|ref|WP_040014750.1|damageinducibleprotein[Dermacoccussp.Ellin185]/11596 TSRS-----N--A-VEDISAVDVALPASTNNAVVQERWAKA------AERPESMTVILST
gi|916534724|ref|WP_051141848.1|damageinducibleprotein[Actinomycesneuii]/11632 NRRK-------TDDLTDIAAVDLKIPPTTNGADLAKSAAA-------ASTEDGLHVVFAT
gi|921140030|ref|WP_053090276.1|damageinducibleprotein[Rothiamucilaginosa]/11616 GREKKCG--GANADLADLRVEDLQLPATTDGAELAAAMNA-------NPLDEGLEVVFST
gi|896406751|ref|WP_049343749.1|damageinducibleprotein[Rothiamucilaginosa]/11647 GREKKRG--GANADLADLRVEDLQLPATTDGAELAAAMNA-------NPLDEGLEVVFST
gi|723608234|gb|AIY01610.1|putativehelicase[Arthrobactersp.PAMC25486]/11613 GRSK-------DTDMTDVAIHDLQIPATTDGRRIIEAMGT-------RTMDEGMTVVFST
gi|946394527|gb|KRE45160.1|damageinducibleprotein[Arthrobactersp.Soil736]/11616 GRQK-------NSDLTDVAIHDLQIPATTDGKTLVEAMGK-------KELDEGMTVVFST
gi|515765304|ref|WP_017197904.1|hypotheticalprotein[Arthrobactersp.M2012083]/11648 GRQK-------NSDITDVAIHDLQIPATTDGATLLEAMGT-------REPDEGMTVVFST
gi|945315587|gb|KQT73127.1|damageinducibleprotein[Microbacteriumsp.Leaf436]/11612 GKRRT---------EEDIPVTDLAFPATTNASALVKQFSQ-------GDGSDKLTVIFST
gi|759908497|ref|WP_043594599.1|damageinducibleprotein[Frigoribacteriumsp.MEB024]_gi|758271359|gb|KIU03346.1|damageinduciblep/1 GKS-----------NEDFTVSDLAYPATTSTPQLLDQVAK-------ASTPDGLTVFFST
gi|945072850|gb|KQR34237.1|hypotheticalproteinASF75_00055[Curtobacteriumsp.Leaf154]/11597 GRV-----------SEDFTVADLAYPATTNTQQLLKEVAK-------ADEPDGLTVFFST
gi|551263152|ref|WP_022883363.1|DNAdamageinducibleprotein[Glaciibactersuperstes]/11621 GKY-----------SEDYSVSDLAYPATTSTAQLLNEVAK-------AETPDGLTVYFST
gi|945452341|gb|KQV08491.1|hypotheticalproteinASC63_10170[Leifsoniasp.Root112D2]/11623 GKK-----------AEDYSVSDLAYPATTNAPSLLKAALG-------SGPDDGITVYFST
gi|742759483|gb|KIC69912.1|damageinducibleprotein[Kocuriarhizophila]/11610 GKST----------ADDISATDLALPATTSAERLITQLNR-------FPDYAGRTVVFST
gi|737919995|ref|WP_035885304.1|damageinducibleprotein[Kocuriarhizophila]/11611 GKST----------ADDISSTDLALPATTSAERLITQLNR-------FPDYAGRTVVFST
gi|737997701|ref|WP_035959325.1|damageinducibleprotein[Kocuriamarina]_gi|725823884|gb|KHE75603.1|damageinducibleprotein[Kocur/1 GKNT----------ADDISSTDLALPATTSAERLITQLNR-------FPDYRGRTVVFST
gi|664521070|ref|WP_031037650.1|damageinducibleprotein[Streptomycessp.NRRLWC3774]/11624 GKQQP------KGDDKDMATHDLALPATTDPDRLIQQMAS-------VEAAPGLTVVFST
gi|500101034|ref|WP_011777041.1|DNAdamageinducibleprotein[Arthrobacteraurescens]_gi|119951547|gb|ABM10457.1|putativeHelicase[A/ GKN-----------HEDLSAHDLAFPASTDSQKLYSRSRK-------STGQAAVTVVFST
gi|916871953|ref|WP_051479009.1|damageinducibleprotein[Arthrobactersp.H5]/11588 GAK-----------HEDMSLHDLAFPASTDPQKLYARSQI-------STGQDAVTVVFST
gi|612084649|gb|EZP43369.1|TypeIIIrestrictionenzyme,ressubunit[Micrococcusluteus]/11625 GRRRA---------SEDIAASDLGFPASTDPEKLAARAAV-------STGDEAVTVVFST
gi|759739708|ref|WP_043450422.1|damageinducibleprotein[Arthrobacterphenanthrenivorans]_gi|742758264|gb|KIC68716.1|damageinduc/1 GRKE----------HEDVSVHDLAFPATTDPAKLLHRAKI-------STGQEAITVVFST
gi|636845448|ref|WP_024366823.1|damageinducibleprotein[Arthrobactersp.TB26]/11605 GRRE----------HEDVSVHDLAFPATTDPQKLFYRTSI-------STGQEAVTVVFST
gi|500009025|ref|WP_011689743.1|DNAdamageinducibleprotein[Arthrobactersp.FB24]_gi|116613051|gb|ABK05760.1|typeIIIrestrictionen/ GRRE----------HEDVSVHDLAFPATTDPQKLFYRTSI-------STGQEAVTVVFST
gi|821249847|gb|KKZ12083.1|hypotheticalproteinTQ37_06090[CandidatusSynechococcusspongiarum15L]/11436 GRPRR-----NNADTADMDVLDLAYPATTDATALASQAVP--------AAPNAMTVVFAT
gi|739446902|ref|WP_037306885.1|damageinducibleprotein[Ruegeriahalocynthiae]/11592 GKRRK-----DNDDIAEIEVHELEFPATTQPAKIVEKAGA--------DDAERMTVVFST
gi|640620081|ref|WP_025048131.1|damageinducibleprotein[Sulfitobactermediterraneus]_gi|751956650|gb|KIN77213.1|TypeIIIrestricti/ GKRRK-----DRDDVAEIEVHELEFPATTQPEKLVEKAGV--------DDTERMTVVFST
gi|740247634|ref|WP_038088583.1|damageinducibleprotein[Thiobacillusprosperus]_gi|684049748|gb|KFZ90853.1|putativehelicase[Thio/ GREE-----------EDIKTAELAYPATTDARRLGRAIVQ------AGQRHTRRRVVFST
gi|518420321|ref|WP_019590528.1|hypotheticalprotein[Thioalkalivibriosp.ALE20]/11603 SKDA-----------EDLPVHDLAYPATTDPRKLALHGSQ--------ETTDRRTVVFAT
gi|500156826|ref|WP_011831496.1|DNAdamageinducibleprotein[Methylibiumpetroleiphilum]_gi|124261892|gb|ABM96881.1|adeninespecifi/ GNDD-----------EDLKTHDLAYPATTDHKALAAAAKL--------LSKNRRTVVFST
gi|769978537|ref|WP_045094522.1|damageinducibleprotein[Legionellafallonii]_gi|698841736|emb|CEG55682.1|Putativeproteinwithboxh/ GKEE-----------EDIPLHDLAYPATTNPEKLVQASKM--------RPQDRPTVIFST
gi|553752541|ref|WP_023085344.1|hypotheticalprotein[Pseudomonasaeruginosa]_gi|552201952|gb|ERU62653.1|hypotheticalproteinQ088_0 GKDE-----------EDISAHDLAYPATTDTRKIIRAASV--------LSKDRRTVIFST
gi|880980418|ref|WP_048704461.1|damageinducibleprotein[Thauerahumireducens]_gi|875132785|gb|KMU70944.1|damageinducibleprotein/1 GKDE-----------EDISTHDLAYPATTDARKVAQAASV--------LSPDRRTVVFST
gi|740135732|ref|WP_037982815.1|damageinducibleprotein[Thauerasp.27]/11603 GKDE-----------EDISTHDLAYPATTDARKVAQAASV--------LSPDRRTVVFST
gi|738017371|ref|WP_035978332.1|damageinducibleprotein[Burkholderiaglumae]/11380 GKEH-----------EDISTHDLAYPATTDARKLAAAVSL--------SSDDRRTVVFST
gi|498506003|ref|WP_010806671.1|typeIIIrestrictionenzyme,ressubunit[Pandoraeasp.SD62]_gi|490314561|gb|EON12879.1|typeIIIrestri/ GKEH-----------EDISTHDLAYPATTDARKLAAAVSV--------SSKDRRTVVFST
gi|500117748|ref|WP_011793753.1|DNAdamageinducibleprotein[Acidovoraxcitrulli]_gi|120587742|gb|ABM31182.1|typeIIIrestrictionenz/ GKDE-----------EDLNTHDLAYPATTDAKRLSKAAAM--------LPKGRRTVVFST
gi|651617328|ref|WP_026611444.1|damageinducibleprotein[Methylocaldumszegediense]/11601 GRDS-----------EDISTHDLAYPATTDAKKVALVAGK--------LSPNRRTVVFST
gi|737210208|ref|WP_035194994.1|damageinducibleprotein[Acidithiobacillusferrivorans]_gi|669953220|emb|CDQ11755.1|Adeninespecif/ GKEE-----------EDIRVHDLAYPATTDFQKLAMHLNH-------TSDDGRITVIFST
gi|935697386|ref|WP_054506180.1|damageinducibleprotein[Achromobactersp.]_gi|928716153|emb|CUK12836.1|Hefnuclease[Achromobacter/ GRNE-----------EDLRIYELAFPATTNATKLAKSWHD-------THNGDAVTVIFST
gi|429502517|gb|ELA00827.1|adeninespecificDNAmethyltransferase[Cupriavidussp.HMR1]/11599 GRNE-----------EDMRIYELAYPATTNATKLAKSWQD-------THNDDAVTVIFST
gi|506356642|ref|WP_015876361.1|DNAdamageinducibleprotein[Burkholderiaglumae]_gi|237877158|gb|ACR29491.1|AdeninespecificDNAmet/ SRNE-----------EDMRIYELAYPATTNATKLAQSWKD-------KHDDSAVTVIFST
gi|696406787|ref|WP_032978003.1|damageinducibleprotein[Bordetellahinzii]_gi|625335076|gb|KCB49343.1|typeIIIrestrictionenzyme,r/ SRNE-----------EDMRIYELAYPATTNANKLAASLSA-------TQDASAITVIFST
gi|817529125|gb|KKO72587.1|damageinducibleprotein[Kerstersiagyiorum]/11592 SRNE-----------EDMRIYELAYPATTNANKLAASLSA-------TQDESAITVIFST
gi|737048910|ref|WP_035044118.1|damageinducibleprotein[Aquabacteriumsp.NJ1]_gi|699052896|gb|KGM38706.1|damageinducibleprotein/1 SRNE-----------EDMRIYELAYPATTNATKLAKSLTE-------TKDDSALTVIFST
gi|822492519|ref|WP_046933319.1|MULTISPECIES:damageinducibleprotein[Xanthomonas]_gi|821703525|gb|KLA61196.1|damageinduciblepr/1 SRNE-----------EDMRIYELAYPATTNATKLAKSLTE-------TKDDSAVTVIFST
gi|751252861|ref|WP_040963740.1|damageinducibleprotein[Xanthomonasfuscans]_gi|749808363|gb|KIJ03887.1|damageinducibleprotein[/1 SRNE-----------EDMRIYELAYPATTNAAKLAKSMTE-------TQDSTAVTVVFST
gi|496774980|ref|WP_009368792.1|DNAdamageinducibleprotein[Bilophilasp.4_1_30]_gi|345039902|gb|EGW44205.1|hypotheticalproteinHM/ SRNE-----------EDMRISEMAYPSTTNPHKLAKAFES-------TQDNTALTIIFST
gi|499241912|ref|WP_010939452.1|DNAdamageinducibleprotein[Desulfovibriovulgaris]_gi|46580580|ref|YP_011388.1|adeninespecificDN/ SRDD-----------EDMRIAEMAYPATTNATKLAAAFNA-------TQDTSRLTVVFAT
gi|504274913|ref|WP_014462015.1|DNAdamageinducibleprotein[Bacillusmegaterium]_gi|345446919|gb|AEN91936.1|AdeninespecificDNAmet/ TKN---------S--EDINSHDLGFPSTTKPEILVQQMNS-------VDNDKEMSVVFST

```
gi|504895966|ref|WP_015083068.1|typeIIrestrictionenzymeandmethyltransferasesubtypeG[Anabaenasp.90]_gi|413972424|gb|AFW96512.1|t    GKNKKK---SKNDDLADITINDLAFPPTTNAEDIIKSYQSI-----QSKNQKDLTVIFST
gi|518327765|ref|WP_019497972.1|hypotheticalprotein[Calothrixsp.PCC7103]/11600    SKK---------SEVEDISVSDLILPATTDAQTIVSRVQA-------LEGQRQLTVIFST
gi|652765503|ref|WP_027079917.1|damageinducibleprotein[Luteimonasmephitis]/11593    GKH---------N--EDSSAHDLAFPATTDTKRLIQAITA------RPKGDKRVTVIFST
gi|896539951|ref|WP_049442123.1|damageinducibleprotein[Stenotrophomonasmaltophilia]/11591    GKH---------S--EDIAVHDLAFPATTDAKRLGQVMAA------RPAADTRRTVIFST
gi|500217900|ref|WP_011888043.1|DNAdamageinducibleprotein[Geobacillusthermodenitrificans]_gi|134267954|gb|ABO68149.1|Conserved/    GKGDS----------EDLRVTDLAYPATTNAETLANQVNK------EL--GKKTTVIFST
gi|696570298|ref|WP_033102014.1|damageinducibleprotein[Thermoactinomycesdaqus]/11594    GKKEG----------EDLRVSDLAYPATTNAKHLVDEIKK------GL--GDSLTIIFST
gi|251772067|gb|EES52637.1|putativehelicase[Leptospirillumferrodiazotrophum]/11618    GKKKN-------DWTEDIRVHDLAYPATTDPEKLSKHVLR------HQRSVTGRAVVFST
gi|738124101|ref|WP_036082108.1|damageinducibleprotein[Leptospirillumferriphilum]_gi|685243306|gb|KGA94177.1|putativehelicase[/    GKK-N-------DWTEDLRVHDLAYPATTDPEKLTKHVLG------YKRSGTGRTVVFST
gi|558699223|ref|WP_023524766.1|helicase[Leptospirillumsp.GroupII'CF1']_gi|206603764|gb|EDZ40244.1|Putativehelicase[Leptospiri/    GKKKD-------NWPEDLRVHDLAYPATTDPEKLSKHVLR------HQREGIGRTVVFST
gi|406774462|gb|AFS53887.1|putativehelicase[LeptospirillumferriphilumML04]/11610    GKRKD-------PF-EDIRAHDLAYPATTDPVKLAQAVQA------HA--SARRKVIFST
gi|740719463|ref|WP_038504749.1|damageinducibleprotein[Leptospirillumferriphilum]_gi|640838125|gb|AIA30173.1|damageinduciblep/1    GKRKD-------PF-EDIRTHDLAYPATTDAKKLAQAVRA------QD--SGQRTVIFST
gi|517810766|ref|WP_018980974.1|hypotheticalprotein[Saccharibacterfloricola]/11733    GKRVVD------GEGARFVIQTLSYPATTEPASLVKSFQKGQ-----AAEKLSMTVVFAT
gi|502471675|ref|WP_012798533.1|helicase[Slackiaheliotrinireducens]_gi|256791761|gb|ACV22431.1|predictedhelicase[Slackiaheliotr    SSVEGD--------TWDTSLKDLPYPATTNPDLLFAQMNRKI-------ASDGLTVVFST
gi|657871713|ref|WP_029576297.1|hypotheticalprotein[Bifidobacteriumthermacidophilum]_gi|673007521|gb|KFJ02535.1|helicase[Bifido    SKVGKTK--Q-EEDEAYGRLTDLPYPATTNPITIAHRFR--K-------HGDALNVVFST
gi|673004757|gb|KFI99791.1|DNAhelicaserestrictionenzymetypeIIIRessubunit[Bifidobacteriumthermacidophilumsubsp.porcinum]/11682    SKIGKTK--Q-EEDEAYGRLSDLPYPATTNPVTIAHRFR--K-------HSDALNVIFST
gi|917316092|ref|WP_051922804.1|hypotheticalprotein[Bifidobacteriumstellenboschense]_gi|673003774|gb|KFI98812.1|helicase[Bifido    SKLSKS------DRESYGQLTDIPFPATTNPETVANRFK--P-------REDALNVVFST
gi|705415694|ref|WP_033502332.1|hypotheticalprotein[Bifidobacteriumpseudolongum]_gi|672982411|gb|KFI77685.1|DNAdamageinducible/    SKV---------SEEAYGQLSDIPFPATTNAQTVANRFR--P-------KADALNVVFST
gi|916591418|ref|WP_051198509.1|hypotheticalprotein[Bifidobacteriumsp.AGR2158]/11704    SHT---------TAESYGQLSDIPFPATTNAQTIANRFK--P-------KKDALNVIFST
gi|772242982|gb|AJW78255.1|hypotheticalproteinVO01_03165[Clavibactermichiganensissubsp.insidiosus]/11642    G--RTK-----NDDLTSVMLEDLGAPATTDGRRLAELLDEQE-------DAEGMVVVFST
gi|916649818|ref|WP_051256909.1|damageinducibleprotein[Actinomycesgraevenitzii]/11677    S----A-----RSDAPADSTVDLPIPATTDAQRLALSLGANN-------DAEGLQVVFAT
gi|496504578|ref|WP_009212861.1|helicase[Actinomycessp.oraltaxon180]_gi|315312695|gb|EFU60776.1|helicase[Actinomycessp.oraltaxo    SSS--A-----RNDTAEESAVDLPIPATTDGQRLADSLNANN-------TTEGLQVVFAT
gi|494862206|ref|WP_007588306.1|helicase[Actinomycessp.ICM39]_gi|398362799|gb|EJN46470.1|typeIIrestrictionenzyme,ressubunit[Ac    SSS--A-----RNDTAEESAVDLPIPATTDGQRLADSLNANN-------ATEGLQVVFAT
gi|881057111|ref|WP_048774974.1|damageinducibleprotein[Sanguibacterkeddieii]/11697    SSS--A-----RNDTAEESAVDLPIPATTDGQRLADSLNANN-------ATEGLQVVFAT
gi|881020459|ref|WP_048740808.1|damageinducibleprotein[Kytococcussedentarius]/11704    SSS--A-----RNDTAEESAVDLPIPATTDGQRLADSLNANN-------ATEGMQVVFAT
gi|881052313|ref|WP_048770555.1|damageinducibleprotein[Cellulomonasflavigena]/11705    SSS--A-----RSDTAEESAVDLPIPATTDGQRLADSLNANN-------ATEGLQVIFAT
gi|490108682|ref|WP_004009424.1|helicase[Mobiluncuscurtisii]_gi|315490465|gb|EFU80089.1|helicaseCterminaldomainprotein[Mobilun/    NRKRQQ-----RDDLADIATVDLKTPPTTDAAKLAQSLTSHL-------DAEGLQVVFCT
gi|490111935|ref|WP_004012603.1|helicase[Mobiluncusmulieris]_gi|227843812|gb|EEJ53982.1|helicaseCterminaldomainprotein[Mobilun/    NRKRAD-----RDDIADIATMDLQIPPTTDAASLADSLTQAR-------PNEGLQVVFAT
gi|490114228|ref|WP_004014848.1|helicase[Mobiluncusmulieris]_gi|269934432|gb|EEZ90993.1|helicaseCterminaldomainprotein[Mobilun/    NRKRAD-----RDDIADIATMDLQIPPTTDAASLADSLTQAR-------PNEGLQVVFAT
gi|493333551|ref|WP_006290636.1|helicase[Parascardoviadenticolens]_gi|315119487|gb|EFT82620.1|helicaseCterminaldomainprotein[P/    SKQAKD------NDDILDSLLDLNYPANTDAQSLLQRYQTIR-----RKSPDSTVVVFVT
gi|495817067|ref|WP_008541646.1|helicase,partial[Sutterellaparvirubra]_gi|378598583|gb|EHY31712.1|helicaseprotein,partial[Sutte    GKADLE------DIESLTVGSELSYPATTEPEKLAAEVKKAR-------ERGGMTVIFST
gi|491572696|ref|WP_005430278.1|helicase[Sutterellawadsworthensis]_gi|319805116|gb|EFW01943.1|DNAhelicaserestrictionenzymeTypeI    GKADLK------NVDEITSASDLSYPATTKADRLAEEVKKAR-------SKTAMTVIFST
gi|748686321|ref|WP_039944445.1|hypotheticalprotein[Thermicanusaegyptius]/11573    GKDQDS----------IDRLTVLTIPPTTNAEDLVKAIQQE--------KTDSMTVVVST
gi|916875698|ref|WP_051482754.1|hypotheticalprotein[Thioalkalivibriosp.HK1]/11366    GRND-----------EDASLVELEIPVTTDAMTIQRSLIQP--------RPDAMRVVFCT
gi|821248604|gb|KKZ10960.1|hypotheticalproteinTE42_09075[CandidatusSynechococcusspongiarumSP3]/11598    GRPRR----N-NADTADMDVLDLAWPATTDATALASQATPA--------APNAMTVVFAT
gi|737409843|ref|WP_035390956.1|hypotheticalprotein[Ferrimicrobiumacidiphilum]_gi|765544266|gb|KJE76241.1|typeIrestrictionenzym    TRDS-----------EDSRTFDIALPATTKPERLGAHLETARQRALSQGDHEPMYVTFST
gi|654906925|ref|WP_028358281.1|damageinducibleprotein[Brackiellaoedipodis]/11679    GKHKNY-----SADLAVATISDLQYPATTNAHSLAQQVQQRH-------DPDHLSVVYAT
gi|502809901|ref|WP_013044877.1|hypotheticalprotein[CandidatusPuniceispirillummarinum]_gi|292663146|gb|ADE38247.1|hypotheticalp    DRTS-----------EDSAVEDLIFPASTNAHSVAHALRQ---------KANRMTVVFCT
gi|251771370|gb|EES51950.1|typeIIrestrictionenzyme,ressubunit[Leptospirillumferrodiazotrophum]/11628    GKDE-----------EDLRVYDLAYPATTDPQKLARHASKS--------YPKGITAILTT
gi|498328480|ref|WP_010642636.1|DNAdamageinducibleprotein[Acidithiobacillusthiooxidans]/11627    GQED-----------EDMRTAELAYPATTNADRLANAIQKT--------KNNRRKVIFST
gi|647633864|ref|WP_025906698.1|damageinducibleprotein[Prochlorococcussp.scB245a_519B7]/11622    GKKKLS------EDSIDIQINELAFPATTNPAKLSDQIKQA--------DQSKMIVIFST
gi|647619599|ref|WP_025893729.1|damageinducibleprotein[Prochlorococcussp.scB241_527N11]/11621    GKIKTS------DDQIRISLNDLAFPATTDASKLSEKIKRA--------DKSKMKVIFST
gi|755034887|ref|WP_042387498.1|hypotheticalprotein[Clostridiumsp.CL6]/11606    SKGE--------------NAVDLGIPSTTDVNRLYNYVNNIE------NIGNNMHFIFST
gi|498139798|ref|WP_010453954.1|helicase[SuccinivibrionaceaebacteriumWG1]/11662    SKKVSR-----DDDTNSESIEELALPACTDEKSTAHQIENYL---T--GSNDKLTVVFST
gi|527501386|gb|EPT34571.1|typeIIrestrictionenzyme,ressubunit[Bacteroidetesbacteriumoraltaxon272str.F0290]/11789    SKL------N-E--DIDVSIVDMPYPATTNIPTTVKHLLQYR-------KSDALIVVFST
gi|496047275|ref|WP_008771782.1|helicase[Bacteroidessp.2_1_33B]_gi|262296443|gb|EEY84373.1|helicaseCterminaldomainprotein[Bact/    SKR------N-DFDNDETSIIDNPLPATTNINSIKRQLLGYK-------DTDGLVVVFST
PDI8503III/11664    SKR------N-DFDNDETSIIDNPLPATTNINSIKRQLLGYK-------DTDGLVVVFST
gi|738719431|ref|WP_036613715.1|helicase[Parabacteroidesdistasonis]_gi|649542518|gb|KDS49387.1|restrictionendonucleasefamilypro    SKR------N-DFDNDETSIIDTPLPATTNINSIKRQLLGYK-------DTDGLVVVFST
gi|659417033|gb|KEJ86133.1|hypotheticalproteinHMPREF1002_00341[Porphyromonassp.31_2]/11656    SKR------N-DFDNDETSIIDNPLPATTNINSIKRQLLGYK-------DTDGLVVVFST
gi|567456807|gb|ETK11358.1|hypotheticalproteinT235_15845[Tannerellasp.oraltaxonBU063isolateCell8/11]/11433    SRKIKRG--N-DNDDLENSVVDLALPASTNTTSIVRQLRACK-------AHNALTVVFST
gi|545440279|ref|WP_021677877.1|helicase[Porphyromonasgingivalis]_gi|543985765|gb|ERJ89108.1|helicaseprotein[Porphyromonasgingi    SRKIKRG--N-DNDELENSVVDLALPASTNTKSIVRQLQACQ-------EHDGLTVVFST
gi|497438056|ref|WP_009752254.1|helicase[Prevotellasp.oraltaxon473]_gi|429144866|gb|EKX87968.1|helicaseprotein[Prevotellasp.ora    SRKIKRG--N-ASDELDDSTVDLALPASTNVNSIVRQLDACR-------SHQGLTVVFST
gi|490423474|ref|WP_004295683.1|MULTISPECIES:helicase[Bacteroides]_gi|156110098|gb|EDO11843.1|helicaseCterminaldomainprotein[B/    SRKIKK-----DFDDTQDSVVDLAVPATTNPKSIAKQLKLYR-------NHNGLTVVFST
gi|941777229|ref|WP_055173506.1|helicase[Bacteroidescaccae]_gi|933263395|emb|CUQ00567.1|Predictedhelicase[Bacteroidescaccae]/1/    SRKTTK-----GSDDTDDSVVDLAVPASTNPQSIASQLKKYR-------DHDGLVVVFST
gi|492413898|ref|WP_005836892.1|helicase[Bacteroidesuniformis]_gi|392678824|gb|EIY72229.1|hypotheticalproteinHMPREF1072_03583[B    SRKTTK-----ESDDTDDSVVDLAVPASTNPQSIASQLKKYR-------DHDGLVVVFST
gi|695455095|ref|WP_032566191.1|helicase[Bacteroidesfragilis]_gi|596095426|gb|EXZ59996.1|restrictionendonucleasefamilyprotein[B    SRKTTK-----ESDDTDDSVVDLAVPASTNPQSIASQLKKYW-------NHDGLVVVFST
gi|933227941|emb|CUQ02348.1|Predictedhelicase[Bacteroidesuniformis]/11658    SRKTNK-----DSDDTDDSVVDLAVPASTNPQSIASQLKKYR-------NHDGLVVVFST
gi|494823013|ref|WP_007558421.1|helicase[Bacteroidesplebeius]_gi|198272167|gb|EDY96436.1|helicaseCterminaldomainprotein[Bacter/    SRKTTK-----GSDDTDDSVVDLAVPASTNPQSIASQLKKYR-------DHDGLVVVFST
gi|495427212|ref|WP_008151909.1|helicase[Parabacteroidesjohnsonii]_gi|218222381|gb|EEC95031.1|helicaseCterminaldomainprotein[P/    SRKIQ----N-KYDDMDDSVVDLAVPASTNPKSIASQLKKYC-------SHDGLVVVFST
gi|933196855|emb|CUO94545.1|typeIrestrictionenzymeEcoKIsubunitR[Bacteroidesuniformis]_gi|933220098|emb|CUN86874.1|typeIrestrict    SRKIQK---N-KYDDADDSVVDLAVPASTNPKSIASQLKKYR-------SHDGLVVVFST
gi|823687748|ref|WP_047101121.1|helicase[Brachyspirahyodysenteriae]_gi|823334537|gb|KLI17932.1|helicase[Brachyspirahyodysenteri    TGSTVK---E------DNDLFDLPLPATTEIKSIKNRLEKAL---K--NKNRGMIVVFST
gi|496436691|ref|WP_009145538.1|helicase[Phascolarctobacteriumsuccinatutens]_gi|322414007|gb|EFY04839.1|helicaseCterminaldomai/    SRLK--------DNDNADNPVDLAMPASTNYYSVAQQLVNAK---KQMEGKEGLIVVYST
gi|503372880|ref|WP_013607541.1|helicase[Sphaerochaetaglobosa]_gi|324026933|gb|ADY13692.1|typeIIrestrictionproteinressubunit[S    SKKNID------DDPTLLSTVDLALPATTDSRSVAAQFMDAT---LLKQRDNGLIVIFAT
gi|505821748|ref|WP_015707145.1|helicase[Treponemaprimitia]_gi|299483503|gb|ADJ19584.1|helicasedomaincontainingprotein[Trepone/    SKTKDD------D-----SVVDLALPASTNIKNITQQYDRAI---ASQKKSGGLVVVFST
gi|505822741|ref|WP_015708134.1|helicase[Treponemaprimitia]_gi|333739639|gb|AEF85129.1|endonucleaseandmethylaseLlaGI[Treponemap    SKTKDD------D-----SVVDLALPASTSIKNITQQYDRAI---ASQKKSGGLVVVFST
gi|488791994|ref|WP_002704400.1|helicase[Treponemasaccharophilum]_gi|380767691|gb|EIC01689.1|typeIIrestrictionproteinressubuni    SKAN------------DDTISNLVLPATTDTNKVRWQFLNFK---RRQAKEGGMIVVFST
gi|524671454|emb|CDD81750.1|putativeseptumsitedeterminingproteinMinC[Bacteroidessp.CAG:462]/11642    SQQKIK------NDEEGVSVIDLALPASTNTDYILYQLEVIR---H--EGRNGMTVVFST
gi|524573865|emb|CDC95766.1|putativeseptumsitedeterminingproteinMinC[Alistipessp.CAG:268]/11639    SKQTEK------NDNDTTSVVDLALPASTDVPSIIKQLQHAR---R--HNAEGLTVVFST
```

gi|658617970|ref|WP_029759328.1|helicase[Fusobacteriumnucleatum]/11606	SSKKST------NDDNITSVVDLALPATTDIDKIVNQLEKI-------KDKNGMKVVFST
gi|494412037|ref|WP_007216782.1|helicase[Bacteroidescellulosilyticus]_gi|392638921|gb|EIY32752.1|hypotheticalproteinHMPREF1062_	TKVKRK---K-EEDADGFTVEDLALPASTFVPSIVSQLRRLH------GRGEGMTVVFST
gi|383107102|gb|AFG37435.1|putativehelicase[SpirochaetaafricanaDSM8902]/11616	SRKT-K---K-GDDSDEISVLDLALPATTDTKKIVKQLRQMR---S--SDSSGLTVVFST
SAF8902III/11616	SRKT-K---K-GDDSDEISVLDLALPATTDTKKIVKQLRQMR---S--SDSSGLTVVFST
gi|652538894|ref|WP_026932796.1|helicase[Gramellaechinicola]/11633	TKKH-S---K-ADDSTIMSVVDLALPASTNVPTIVKQFKGLE---A--SNKKGMTVVFST
gi|639238223|ref|WP_024568839.1|helicase[Elizabethkingiaanophelis]/11628	TKQK-T---N--DDLGDTSLVDLPLPATTDVKSVIKQFKNIN---R--TNNTGMTVVFST
gi|652520381|ref|WP_026914582.1|helicase[Gramellaportivictoriae]/11623	TKQR-G---N-NSDSEDLSEVDLAFPASTDVKSVIKQFRNIN---K--NDEDGMTVVFST
gi|494922183|ref|WP_007648219.1|Helicase[Formosasp.AK20]_gi|469825489|gb|EMQ95550.1|Helicasedomainprotein[Formosasp.AK20]/11626	TRQR-G---N-NDDDSDFSVVDLAYPASTDPNSVIKQFKAID---R--TTKKGMTVVFST
gi|522083382|ref|WP_020594591.1|hypotheticalprotein[Spirosomapanaciterrae]/11637	TKNR-K---Q-LDDIDTSSVVDLAFPASTNVENILHQFSGLE---T--KANAGLTVVFST
gi|490455352|ref|WP_004326157.1|helicase[Bacteroidesovatus]_gi|392669277|gb|EIY62768.1|hypotheticalproteinHMPREF1070_03635[Bact	SKQK-T---K-SEDLDGYSTVDLALPASTDVESIICQLKSN----S--QNDCGMTVVFST
gi|499516035|ref|WP_011202675.1|helicase[Bacteroidesfragilis]_gi|53713270|ref|YP_099262.1|helicasedomainprotein[Bacteroidesfrag	SKQK-T---K-SEDLDGYSTVDLALPASTDVESIICQLKSN----S--QNDCGMTVVFST
gi|651338332|ref|WP_026461320.1|helicase[Adhaeribacteraquaticus]/11620	SRKK-T---K-NEDTDSFSVVDLALPASTNVKDILKQFRHIK---E--NQKPGMTVVFST
gi|806964642|gb|AKC17139.1|helicase[Flavobacteriumpsychrophilum]/11639	SKKK-T---K-IEDSESFNTIDLALPASTNVPQILSQFASLE---L--NAKEGMTVVFST
gi|752684374|ref|WP_041332322.1|helicase[Flavobacteriumpsychrophilum]_gi|666882889|gb|AIG30715.1|helicase[Flavobacteriumpsychro	SKKK-T---K-IEDSESFNTIDLALPASTNVPQILSQFASLE---L--NAKEGMTVVFST
gi|933196595|emb|CUP04894.1|DNAorRNAhelicasesofsuperfamilyII[Bacteroidesuniformis]/11631	SRKK-T---K-NEDNDNFSVVDLALPASTNTAEILRQFNHIK---T--NSNSGMTVVFST
gi|670509109|ref|WP_031456918.1|helicase[Flavobacteriumchungangense]/11625	SKKK-T---K-DEDTDTFSTVDLALPASTDPEEILKQFNLFK---S--TNKSGMTVVFST
	* *

gi|490190857|ref|WP_004089451.1|hypotheticalprotein[Helicobacterbilis]_gi|476630638|gb|EMZ37196.1|hypotheticalproteinC826_02193	YQSIEQIKKAQ---QLDSK-----------------DKLPQIDLIICDEAHRSVGSLYTT
gi|488896133|ref|WP_002807242.1|MULTISPECIES:restrictionendonucleaseEco57I[Campylobacter]_gi|380541096|gb|EIA65377.1|hypothetic	YQSVEKIMQAQ---K---Q-----------------NDLDEIDLIICDEAHRSVGNLYSG
gi|746602240|ref|WP_039628365.1|restrictionendonucleaseEco57I[Campylobacterlari]_gi|744819395|gb|AJD03391.1|putativehelicase[Ca	YQSVEKIMQAQ---K---Q-----------------NDLDEINLIICDEAHRSVGNLYSG
gi|727317135|ref|WP_033772558.1|restrictionendonucleaseEco57I[Helicobacterpylori]/11622	YQSALRIKEAQ-------E-----------------RGLDKIDLMICDEAHRTVGAMFST
gi|727103490|ref|WP_033618954.1|restrictionendonucleaseEco57I[Helicobacterpylori]/11616	YQSALRIKEAQ-------E-----------------RGLDKIDLMICDEAHRTVGAMFST
gi|727340205|ref|WP_033794408.1|restrictionendonucleaseEco57I[Helicobacterpylori]/11609	YQSALRIKEAQ-------E-----------------RGLDKIDLMVCDEAHRTVGAMFST
gi|727292407|ref|WP_033749975.1|restrictionendonucleaseEco57I[Helicobacterpylori]/11611	YQSALRIKEAQ-------E-----------------RGLDKIDLMVCDEAHRTVGAMFST
gi|727289795|ref|WP_033747591.1|restrictionendonucleaseEco57I[Helicobacterpylori]/11612	YQSALRIKEAQ-------E-----------------AGLNGIDLIICDEAHRTVGAMYST
gi|727319676|ref|WP_033775047.1|restrictionendonucleaseEco57I[Helicobacterpylori]/11612	YQSALRIKEAQ-------E-----------------RGLDKIDLMICDEAHRTVGAMFST
gi|447212910|ref|WP_001290166.1|restrictionendonucleaseEco57I[Helicobacterpylori]_gi|308063540|gb|ADO05427.1|hypotheticalprotei	YQSALRIQEAQ-------E-----------------VGLGEMDLIICDEAHRTVGALYSS
gi|447212901|ref|WP_001290157.1|restrictionendonucleaseEco57I[Helicobacterpylori]_gi|384557329|gb|AFH97797.1|hypotheticalprotei	YQSALRIQEAQ-------E-----------------VGLGEMDLIICDEAHRTVGAMYSS
gi|515849671|ref|WP_017280424.1|hypotheticalprotein[Helicobacterpylori]/11615	YQSALRIQEAQ-------E-----------------VGLGEIDLIVCDEAHRTVGAMYSS
gi|447212900|ref|WP_001290156.1|restrictionendonucleaseEco57I[Helicobacterpylori]_gi|317178970|dbj|BAJ56758.1|TypeIIGrestrictio	YQSALRIKEAQ-------E-----------------VGLGEIDLVICDEAHRTVGAMYSS
gi|447079018|ref|WP_001156274.1|hypotheticalprotein[Helicobacterpylori]/11614	YQSALRIKEAQ-------E-----------------AGLNGIDLIICDEAHRTVGAMYSS
gi|447212800|ref|WP_001290056.1|hypotheticalprotein[Helicobacterpylori]_gi|532153248|gb|EQL67316.1|restrictionendonucleaseEco57	YQSALRIQEAQ-------R-----------------MGLNEIDLIIGDEAHRTVGAMYSS
gi|505457237|ref|WP_015644339.1|hypotheticalprotein[Helicobacterpylori]_gi|499061160|gb|AGL70074.1|restrictionendonucleaseEco57	YQSALRIQEAQ-------E-----------------VGLGKIDLIICDEAHRTVGAMYSS
HPYUM037X/11591	YQSALRIQEAQ-------E-----------------VGLGKIDLIICDEAHRTVGAMYSS
gi|920026297|ref|WP_052937176.1|restrictionendonuclease[Helicobacterpylori]_gi|912528678|gb|KNX48238.1|restrictionendonucleaseE	YQSALRIQEAQ-------R-----------------MGLDGIDLMICDEAHRTVGAMYSS
gi|915539050|ref|WP_050841327.1|restrictionendonuclease[Helicobacterpylori]_gi|908890887|gb|KNE06183.1|restrictionendonucleaseE	YQSALRIKEVQ-------E-----------------AGLGEIDLIICDEAHRTVGAMYSS
gi|727101397|ref|WP_033616957.1|restrictionendonucleaseEco57I[Helicobacterpylori]/11592	YQSALRIKEAQ-------E-----------------AGLNEIDLIICDEAHRTVGAMYSS
gi|562814875|emb|CDK13002.1|TypeISPRMsystem[Helicobacterpylori26695]/11588	YQSALRIKEAQ-------E-----------------AGLGGIDLIICDEAHRTVGAMYSS
HPYAXVIII/11588	YQSALRIKEAQ-------E-----------------AGLGGIDLIICDEAHRTVGAMYSS
gi|447079029|ref|WP_001156285.1|restrictionendonucleaseEco57I[Helicobacterpylori]_gi|393085956|gb|EJB86635.1|hypotheticalprotei	YQSALHIKEAQ-------E-----------------AGLNGIDLIICDEAHRTVGAMYST
gi|489105953|ref|WP_003015814.1|restrictionendonuclease[Helicobacterpylori]_gi|477503388|gb|ENH59950.1|restrictionendonucleasef	YQSALRIKEAQ-------E-----------------AGLGGIDLIICDEAHRTVGAMYSS
gi|447079006|ref|WP_001156262.1|restrictionendonucleaseEco57I[Helicobacterpylori]_gi|393067470|gb|EJB68280.1|hypotheticalprotei	YQSALRIKEAQ-------E-----------------AGLGGIDLIICDEAHRTVGAMYSS
gi|727314589|ref|WP_033770298.1|restrictionendonucleaseEco57I[Helicobacterpylori]/11586	YQSALRIKEAQ-------E-----------------AGLNGIDLIICDEAHRTVGAMYSS
gi|447079019|ref|WP_001156275.1|restrictionendonucleaseEco57I[Helicobacterpylori]_gi|308065028|gb|ADO06920.1|hypotheticalprotei	YQSALRIKEAQ-------E-----------------AGLGEIDLIICDEAHRTVGAMYSS
gi|447079028|ref|WP_001156284.1|restrictionendonucleaseEco57I[Helicobacterpylori]_gi|393076030|gb|EJB76784.1|hypotheticalprotei	YQSVLRIKEAQ-------E-----------------AGLNGIDLIICDEAHRTVGAMYST
gi|393034009|gb|EJB35070.1|hypotheticalproteinHPNQ4053_0631[HelicobacterpyloriNQ4053]/11514	YQSALRIKEAQ-------E-----------------AGLGEIDLIICDEAHRTVGAMYSS
gi|447079004|ref|WP_001156260.1|restrictionendonucleaseEco57I[Helicobacterpylori]_gi|384552418|gb|AFI07366.1|hypotheticalprotei	YQSALRIKEAQ-------E-----------------AGLKEIDLIICDEAHRTVGAMYSS
gi|746011306|ref|WP_039088215.1|restrictionendonucleaseEco57I[Helicobacterpylori]_gi|733488720|gb|KHL85979.1|restrictionendonuc	YQSALRIKEAQ-------E-----------------AGLGGIDLIICDEAHRTVGAMYSS
gi|531081162|gb|EQD92166.1|restrictionendonucleaseEco57I[HelicobacterpyloriPZ5080]/11509	YQSALRIKEAQ-------E-----------------AGLNGIDLIICDEAHRTVGAMYSS
gi|544760796|ref|WP_021187395.1|restrictionendonucleaseEco57I[Helicobacterpylori]_gi|531086508|gb|EQD96745.1|restrictionendonuc	YQSALRIKEAQ-------E-----------------AGLNGIDLIICDEAHRTVGALYSS
gi|447079012|ref|WP_001156268.1|restrictionendonucleaseEco57I[Helicobacterpylori]_gi|393028199|gb|EJB29286.1|hypotheticalprotei	YQSALRIKEAQ-------E-----------------AGLNEIDLIICDEAHRTVGAMYSS
gi|746008458|ref|WP_039085555.1|restrictionendonucleaseEco57I[Helicobacterpylori]_gi|733480358|gb|KHL78026.1|restrictionendonuc	YQSALRIKEAQ-------E-----------------AGLNGIDLIICDEAHRTVGAMYSS
gi|447079011|ref|WP_001156267.1|restrictionendonucleaseEco57I[Helicobacterpylori]_gi|393038534|gb|EJB39568.1|hypotheticalprotei	YQSALRIKEAQ-------E-----------------AGLNGIDLIICDEAHRTVGAMYSS
gi|393037163|gb|EJB38199.1|hypotheticalproteinHPNQ4161_0422[HelicobacterpyloriNQ4161]/11556	YQSALRIKEAQ-------E-----------------VGLKGIDLIICDEAHRTVGAMYST
gi|447079007|ref|WP_001156263.1|restrictionendonucleaseEco57I[Helicobacterpylori]_gi|393051817|gb|EJB52768.1|hypotheticalprotei	YQSALRIKEAQ-------E-----------------AGLNGIDLIICDEAHRTVGAMYSS
gi|487867094|ref|WP_001940560.1|helicase[Helicobacterpylori]_gi|459505657|gb|EMG98418.1|helicaseprotein[HelicobacterpyloriGAM21	YQSALRIKEAQ-------E-----------------AGLNGIDLIICDEAHRTVGAMYST
gi|487872094|ref|WP_001945560.1|helicase[Helicobacterpylori]_gi|459512104|gb|EMH04523.1|helicaseprotein[HelicobacterpyloriGAM24	YQSALRIKEAQ-------E-----------------AGLNGIDLIICDEAHRTVGAMYST
gi|487880228|ref|WP_001953694.1|helicase[Helicobacterpylori]_gi|459529131|gb|EMH20734.1|helicaseprotein[HelicobacterpyloriGAM26	YQSALRIKEAQ-------E-----------------AGLNGIDLIICDEAHRTVGAMYST
gi|487882593|ref|WP_001956059.1|helicase[Helicobacterpylori]_gi|459533462|gb|EMH24859.1|helicaseprotein[HelicobacterpyloriGAM26	YQSALRIKEAQ-------E-----------------AGLNGIDLIICDEAHRTVGAMYST
gi|487845430|ref|WP_001918896.1|helicase[Helicobacterpylori]_gi|410713871|gb|EKQ71363.1|helicaseprotein[HelicobacterpyloriGAM10	YQSALRIKEAQ-------E-----------------AGLNGIDLIICDEAHRTVGAMYST
gi|447079005|ref|WP_001156261.1|restrictionendonucleaseEco57I[Helicobacterpylori]_gi|393069830|gb|EJB70625.1|hypotheticalprotei	YQSALRIKEAQ-------E-----------------AGLNGIDLIICDEAHRTVGAMYST
gi|459506808|gb|EMG99528.1|helicaseprotein[HelicobacterpyloriGAM121Aii]/11545	YQSALRIKEAQ-------E-----------------AGLKGIDLIICDEAHRTVGAMYST
gi|487825283|ref|WP_001898749.1|restrictionendonucleaseEco57I[Helicobacterpylori]_gi|393107526|gb|EJC08068.1|hypotheticalprotei	YQSALRIKEAQ-------E-----------------AGLNGIDLIICDEAHRTVGAMYST
gi|447079008|ref|WP_001156264.1|restrictionendonucleaseEco57I[Helicobacterpylori]_gi|393058636|gb|EJB59524.1|hypotheticalprotei	YQSALRIKEAQ-------E-----------------AGLNEIDLIICDEAHRTVGAMYST
gi|447079015|ref|WP_001156271.1|restrictionendonucleaseEco57I[Helicobacterpylori]_gi|307637357|gb|ADN79807.1|hypotheticalprotei	YQSALRIKEAQ-------E-----------------AGLNEIDLIICDEAHRTVGAMYST
gi|487884861|ref|WP_001958327.1|helicase[Helicobacterpylori]_gi|459535535|gb|EMH26834.1|helicaseprotein[HelicobacterpyloriGAM26	YQSALRIKEAQ-------E-----------------AGLNGIDLIICDEAHRTVGAMYST

```
gi|487886365|ref|WP_001959831.1|helicase[Helicobacterpylori]_gi|459538727|gb|EMH29861.1|helicaseprotein[HelicobacterpyloriGAM27        YQSALRIKEVQ-------E-----------------AGLNGIDLIICDEAHRTVGAMYST
gi|545301637|ref|WP_021581412.1|helicase[Helicobacterpylori]_gi|535931889|gb|ERA61745.1|helicaseprotein[HelicobacterpyloriGAM11        YQSALRIKEVQ-------E-----------------AGLKEIDLIICDEAHRTVGAMYST
gi|447079010|ref|WP_001156266.1|restrictionendonucleaseEco57I[Helicobacterpylori]_gi|393095477|gb|EJB96081.1|hypotheticalprotei        YQSALRIKEAQ-------E-----------------AGLNEIDLIICDEAHRTVGAMYST
gi|447079009|ref|WP_001156265.1|restrictionendonucleaseEco57I[Helicobacterpylori]_gi|393056294|gb|EJB57206.1|hypotheticalprotei        YQSALRIKEAQ-------E-----------------SGLNGIDLIICDEAHRTVGAMYST
gi|471386985|gb|EMR58906.1|restrictionendonucleasefamilyprotein[HelicobacterpyloriHpH1]/11504        YQSALRIKEAQ-------E-----------------AGLNGIDLIICDEAHRTVGAMYST
gi|487875679|ref|WP_001949145.1|helicase[Helicobacterpylori]_gi|459516928|gb|EMH09091.1|helicaseprotein[HelicobacterpyloriGAM25        YQSALRIKEVQ-------E-----------------AGLNGIDLIICDEAHRTVGAMYST
gi|496130066|ref|WP_008854573.1|DNAdamageinducibleprotein[Commensalibacterintestini]_gi|353671689|gb|EHD13391.1|helicasedomain/        YHSIAVLHDAQ--QGEH--------------------ALSAFDLIICDEAHRTTGATYED
gi|924898894|ref|WP_053556546.1|damageinducibleprotein[Sphingopyxissp.113P3]_gi|924523996|gb|ALC14732.1|damageinducibleprotei/1        YQSLPTIAVAQ---EKF--------------------GLPAFDLIICDEAHRTTGVTLSG
gi|780802950|gb|KJS08562.1|damageinducibleprotein[Hoefleasp.BRH_c9]/11633        YQSIQTISDAQ---KKF--------------------GLPEFDLIICDEAHRTTGAKFED
gi|944671432|gb|KQN22886.1|damageinducibleprotein[Sphingomonassp.Leaf32]/11415        YQSIQVISNAQ---KQH--------------------GFPEFDLIICDEAHRTTGATLDG
gi|944612953|gb|KQM64772.1|damageinducibleprotein[Sphingomonassp.Leaf16]/11726        YQSIQVISNAQ---KQH--------------------GFPEFDLIICDEAHRTTGATLDG
gi|764353851|ref|WP_044331468.1|damageinducibleprotein[Sphingomonashengshuiensis]_gi|761897338|gb|AJP71707.1|damageinduciblep/1        YQSIQVISDAQ---RLH--------------------GLPEFDLIICDEAHRTTGATLDG
gi|945874472|gb|KQZ27973.1|damageinducibleprotein[Mesorhizobiumsp.Root552]/11651        YQSIQVISDAQ---QKH--------------------GLREFDLIICDEAHRTTGATLDG
gi|759408081|ref|WP_043132403.1|damageinducibleprotein[Paracoccussanguinis]_gi|694217086|gb|KGJ14281.1|damageinducibleprotein/1        YHSIDVLTRAQ---KSH--------------------GLPEFDLVICDEAHRTTGVTLKD
gi|654342671|ref|WP_027836116.1|damageinducibleprotein[Maritaleamyrionectae]/11632        YHSIDVLSRAQ---KDF--------------------GLPEFDLVVCDEAHRTTGVTLKD
gi|126717687|gb|EBA14409.1|hypotheticalproteinRSK20926_02032[Roseobactersp.SK20926]/11603        YHSIDVLTRAQ---KQH--------------------GLPEFDLVICDEAHRTTGVTLKD
gi|765301116|ref|WP_044628780.1|damageinducibleprotein[Halomonasmeridiana]_gi|764089679|gb|KJD19875.1|damageinducibleprotein[/1        YHSIDVLTQAQ---QNH--------------------GLPEFDLVICDEAHRTTGVTLKD
gi|518053006|ref|WP_019223214.1|DNAdamageinducibleprotein[Bartonellarattaustraliani]/11654        YHSIQVIAGAQ---KDH--------------------GLPEFDLIICDEAHRTTGVVLGT
gi|493637806|ref|WP_006589578.1|DNAdamageinducibleprotein[Bartonellabirtlesii]_gi|395410345|gb|EJF76900.1|hypotheticalproteinM/        YHSIQVISDAQ---KDH--------------------GLPEFDLIICDEAHRTTGASLGS
gi|501188603|ref|WP_012231622.1|DNAdamageinducibleprotein[Bartonellatribocorum]_gi|161017843|emb|CAK01401.1|helicase/methyltra/        YHSIQVISDAQ---KDH--------------------GLPEFDLIICDEAHRTTGASLGN
gi|651388616|ref|WP_026500615.1|damageinducibleprotein[Bartonellagrahamii]/11655        YHSIQVISDAQ---KEH--------------------GLPEFDLIICDEAHRTTGASLGS
gi|501188669|ref|WP_012231687.1|DNAdamageinducibleprotein[Bartonellatribocorum]_gi|161017922|emb|CAK01480.1|helicase/methyltra/        YHSIQVISDAQ---KEH--------------------GLPEFDLIICDEAHRTTGASLGS
gi|518048832|ref|WP_019219040.1|DNAdamageinducibleprotein[Bartonellaflorencae]/11655        YHSIQVISDAQ---KEH--------------------GLPEFDLIICDEAHRTTGVVLGT
gi|518053617|ref|WP_019223825.1|DNAdamageinducibleprotein[Bartonellarattaustraliani]/11607        YHSIQVIADAQ---KEY--------------------NLPEFDLIICDEAHRTTGVVLGT
gi|506336449|ref|WP_015856168.1|DNAdamageinducibleprotein[Bartonellagrahamii]_gi|240267492|gb|ACS51080.1|helicase/methyltransf/        YHSIQVISDAQ---NTH--------------------GLPEFDLIICDEAHRTTGASLGS
gi|494588832|ref|WP_007347427.1|DNAdamageinducibleprotein[Bartonellarattimassiliensis]_gi|395419053|gb|EJF85361.1|hypothetical/        YHSIQVISDAQ---KEH--------------------DLPEFDLIICDEAHRTTGAVLGT
gi|501188650|ref|WP_012231668.1|DNAdamageinducibleprotein[Bartonellatribocorum]_gi|161017902|emb|CAK01460.1|helicase/methyltra/        YHSIQVISDAQ---KKY--------------------DLPEFDLIICDEAHRTTGAVLGT
gi|501188624|ref|WP_012231643.1|DNAdamageinducibleprotein[Bartonellatribocorum]_gi|161017866|emb|CAK01424.1|helicase/methyltra/        YQSIQVIVDAQ---KDH--------------------GLAAFDLIICDEAHRTTGASLGT
gi|518053856|ref|WP_019224064.1|DNAdamageinducibleprotein[Bartonellarattaustraliani]/11625        YQSIQVIVDAQ---KDH--------------------GLAAFDFIICDEAHRTTGASLGT
gi|501188053|ref|WP_012231081.1|DNAdamageinducibleprotein[Bartonellatribocorum]_gi|161017434|emb|CAK00992.1|helicase/methyltra/        YQSIQVIVDAQ---KDH--------------------GLAAFDLIICDEAHRTTGASLGT
gi|651388630|ref|WP_026500626.1|damageinducibleprotein[Bartonellagrahamii]/11657        YHSIQVISDAQ---KDH--------------------GLPEFDLIICDEAHRTTGAVLGT
gi|518048855|ref|WP_019219063.1|DNAdamageinducibleprotein[Bartonellaflorencae]/11658        YHSIQVISDAQ---KDH--------------------GLPEFDLIICDEAHRTTGVVLGT
gi|490995101|ref|WP_004856829.1|DNAdamageinducibleprotein[Bartonelladoshiae]_gi|395413167|gb|EJF79645.1|hypotheticalproteinMCS/        YHSIQVISDAQ---KDH--------------------GLPEFDLIICDEAHRTTGVVLGT
gi|491002558|ref|WP_004864279.1|DNAdamageinducibleprotein[Bartonellavinsonii]_gi|395421392|gb|EJF87643.1|hypotheticalproteinME/        YHSIQVIADAQ---KDH--------------------GLPEFDLIICDEAHRTTGVSLGT
gi|491003373|ref|WP_004865094.1|DNAdamageinducibleprotein[Bartonellavinsonii]_gi|395432864|gb|EJF98838.1|hypotheticalproteinME/        YHSIQVIADAQ---KNH--------------------GLPEFDLIICDEAHRTTGVSLGT
gi|491002426|ref|WP_004864148.1|DNAdamageinducibleprotein[Bartonellavinsonii]_gi|395421767|gb|EJF88003.1|hypotheticalproteinME/        YHSIQVISDAQ---RKY--------------------ALPEFDLIICDEAHRTTGAVLGT
gi|491004024|ref|WP_004865745.1|DNAdamageinducibleprotein[Bartonellavinsonii]_gi|395432542|gb|EJF98522.1|hypotheticalproteinME/        YHSIQVISDAQ---NKY--------------------ALPEFDLIICDEAHRTTGAVLGT
gi|648396442|ref|WP_026088193.1|damageinducibleprotein[Bartonellarattaustraliani]/11593        YHSIQVIAGAQ---KDH--------------------DLPEFDLIICDEAHRTTGVVLGT
gi|748190174|ref|WP_039761290.1|damageinducibleprotein[Bartonellaqueenslandensis]/11658        YHSIQVIADAQ---KDH--------------------DLPEFDLIICDEAHRTTGVVLGT
gi|494817599|ref|WP_007553007.1|DNAdamageinducibleprotein[Bartonellasp.DB56]_gi|395411312|gb|EJF77837.1|hypotheticalproteinMC/1        YHSIQVISDAQ---KEH--------------------GLPEFDLIICDEAHRTTGAVLGT
gi|515763376|ref|WP_017195976.1|DNAdamageinducibleprotein[Bartonellabirtlesii]/11656        YHSIQVISDAQ---KRY--------------------ALPEFDLIICDEAHRTTGAVLGT
gi|494589420|ref|WP_007348014.1|DNAdamageinducibleprotein[Bartonellarattimassiliensis]_gi|395416278|gb|EJF82667.1|hypothetical/        YHSIQVISDAQ---KEY--------------------DLPEFDLIICDEAHRTTGVSLGT
gi|506336469|ref|WP_015856188.1|DNAdamageinducibleprotein[Bartonellagrahamii]_gi|240267512|gb|ACS51100.1|helicase/methyltransf/        YHSIQVISDAQ---KDH--------------------SLPEFDLIICDEAHRTTGASLGT
gi|749308087|ref|WP_040132227.1|damageinducibleprotein[Bartonellatribocorum]_gi|605051878|emb|CDO49988.1|helicase/methyltransf/        YHSIQVISDAQ---KKY--------------------DLPEFDLIICDEAHRTTGASLGT
gi|515763800|ref|WP_017196400.1|DNAdamageinducibleprotein[Bartonellabirtlesii]/11658        YHSIQVISDAQ---KDH--------------------DLPEFDLIICDEAHRTTGVVLGT
gi|492186994|ref|WP_005773380.1|DNAdamageinducibleprotein[Bartonellaelizabethae]_gi|395418361|gb|EJF84688.1|hypotheticalprotei/        YHSIQVISDAQ---KEH--------------------DLPEFDLIICDEAHRTTGVVLGT
gi|501188615|ref|WP_012231634.1|DNAdamageinducibleprotein[Bartonellatribocorum]_gi|161017856|emb|CAK01414.1|helicase/methyltra/        YHSIQVISDAQ---KEY--------------------DLPEFDLIICDEAHRTTGVVLGT
gi|501187470|ref|WP_012230499.1|DNAdamageinducibleprotein[Bartonellatribocorum]_gi|161017090|emb|CAK00648.1|helicase/methyltra/        YHSIQVISDAQ---KEY--------------------DLPEFDLIICDEAHRTTGVVLGT
gi|501188636|ref|WP_012231655.1|DNAdamageinducibleprotein[Bartonellatribocorum]_gi|161017879|emb|CAK01437.1|helicase/methyltra/        YHSIQVISDAQ---KEY--------------------DLPEFDLIICDEAHRTTGVVLGT
gi|930151941|emb|CTQ34818.1|UvsWhelicase[Jannaschiarubra]/11641        YQSIQVISAAQ----EA--------------------GLEAFDLIVCDEAHRTTGVTTGE
gi|928977106|ref|WP_054001461.1|damageinducibleprotein[Shimiasp.SK013]_gi|927698852|gb|KPA22698.1|typeIrestrictionenzymeEcoKIs/        YQSIPVITAAQ----EQ--------------------GLQQFDLIICDEAHRTTGATIDG
gi|738872465|ref|WP_036760884.1|damageinducibleprotein[Leisingeradaeponensis]/11643        YQSIQVIADAQ---ADH--------------------GLPPFDLIICDEAHRTTGATLDG
gi|518050881|ref|WP_019221089.1|DNAdamageinducibleprotein[Bartonellasenegalensis]/11652        YQSLQVIVDAQ---KQY--------------------GFPTFDLIICDEAHRTTGATLVG
gi|493980596|ref|WP_006923523.1|DNAdamageinducibleprotein[Bartonellawashoensis]_gi|395413322|gb|EJF79796.1|hypotheticalprotein/        YQSIQVIADAQ---KKY--------------------GLPTFDLIICDEAHRTTGATLVG
gi|493983106|ref|WP_006925984.1|DNAdamageinducibleprotein[Bartonellawashoensis]_gi|395418127|gb|EJF84455.1|hypotheticalprotein/        YQSIQVIADAQ---KKY--------------------GLPTFDLIICDEAHRTTGATLVG
gi|499494671|ref|WP_011181311.1|DNAdamageinducibleprotein[Bartonellahenselae]_gi|49239008|emb|CAF28308.1|hypotheticalproteinBH/        YQSIQVIADAQ---KKY--------------------GLPTFDLIICDEAHRTTGATLVG
gi|651388426|ref|WP_026500441.1|damageinducibleprotein[Bartonellagrahamii]/11652        YQSIQVVADAQ---KKY--------------------GLPIFDLIICDEAHRTTGATLVG
gi|506337280|ref|WP_015856999.1|DNAdamageinducibleprotein[Bartonellagrahamii]_gi|240268402|gb|ACS51990.1|helicase/methyltransf/        YQSIQVVADAQ---KKY--------------------GLPIFDLIICDEAHRTTGATLVG
gi|494817535|ref|WP_007552943.1|DNAdamageinducibleprotein[Bartonellasp.DB56]_gi|395412002|gb|EJF78518.1|hypotheticalproteinMC/1        YHSIQVVADAQ---KNY--------------------GLPTFDLIICDEAHRTTGATLVG
gi|493638099|ref|WP_006589864.1|DNAdamageinducibleprotein[Bartonellabirtlesii]_gi|395409809|gb|EJF76394.1|hypotheticalproteinM/        YQSIQVVSDAQ---KKY--------------------GLPTFDLIICDEAHRTTGATLVG
gi|748189589|ref|WP_039760707.1|damageinducibleprotein[Bartonellaqueenslandensis]/11651        YQSIQVIADAQ---KNY--------------------GLPTFDLIICDEAHRTTGATLVG
gi|501189483|ref|WP_012232501.1|DNAdamageinducibleprotein[Bartonellatribocorum]_gi|161018902|emb|CAK02460.1|helicase/methyltra/        YQSIQVIADAQ---KNH--------------------GFPTFDLIICDEAHRTTGATLVG
gi|518049181|ref|WP_019219389.1|DNAdamageinducibleprotein[Bartonellaflorencae]/11652        YQSIQVIADAQ---KNY--------------------GFPTFDLIICDEAHRTTGATLEG
gi|492193255|ref|WP_005774528.1|DNAdamageinducibleprotein[Bartonellaelizabethae]_gi|395416403|gb|EJF82779.1|hypotheticalprotei/        YQSIQVIADAQ---KNY--------------------GFPTFDLIICDEAHRTTGATLEG
gi|498387325|ref|WP_010701288.1|helicase/methyltransferase[Bartonellabovis]_gi|478418724|gb|ENN92426.1|helicase/methyltransfera        YQSIQVISDAQ---NKY--------------------GLPVFDLIICDEAHRTTGATLSG
gi|319408697|emb|CBI82352.1|conservedhypotheticalprotein[BartonellaschoenbuchensisR1]/11661        YQSIQVVSDAQ---NEH--------------------GLPAFDLVICDEAHRTTGATVSG
gi|494741726|ref|WP_007477134.1|DNAdamageinducibleprotein[Bartonellamelophagi]_gi|395423925|gb|EJF90113.1|hypotheticalproteinM/        YQSIQVVSDAQ---NEY--------------------GLPAFDLVICDEAHRTTGATLSG
```

gi|737261405|ref|WP_035245349.1|damageinducibleprotein[Alcanivoraxjadensis]_gi|686989709|gb|KGD62556.1|hypotheticalproteinT9A_/	YQSIQAISQAQ---QEH--------------------SLPEFDLVICDEAHRTTGVTLSG
gi|916687564|ref|WP_051294655.1|damageinducibleprotein[Gemmobacternectariphilus]/11641	YQSIQVIADAQ---SQH--------------------GLPEFDLVICDEAHRTTGATLAG
gi|740294127|ref|WP_038132495.1|damageinducibleprotein[Thioclavasp.DT234]_gi|660524826|gb|KEO54689.1|hypotheticalproteinDT23_/1	YQSIGVLDAAQ----KA--------------------GLPAFDLIICDEAHRTTGATLAG
gi|568331062|ref|WP_024099615.1|helicase[Phaeobactergallaeciensis]_gi|568232121|gb|AHD12007.1|putativehelicase[Phaeobactergalla	YQSIQVIADAQ---KQH--------------------DFPEFDLIICDEAHRTTGATLAG
gi|518810459|ref|WP_019966413.1|DNAdamageinducibleprotein[Pannonibacterphragmitetus]/11635	YQSIVTLTRAQ----EA--------------------GLPEFDLIICDEAHRTTGATLDG
gi|495305760|ref|WP_008030510.1|DNAdamageinducibleprotein[Rhodobactersp.SW2]_gi|259021698|gb|EEW25000.1|typeIIIrestrictionprot/	YQSIVTLTRAQ----EA--------------------GLPEFDLIICDEAHRTTGATLDG
gi|506403303|ref|WP_015923022.1|DNAdamageinducibleprotein[Rhodobactersphaeroides]_gi|221162951|gb|ACM03922.1|HypotheticalProte/	YQSIGTLSRAQ----EA--------------------GLPAFDLIICDEAHRTTGATLDK
gi|738818386|ref|WP_036708790.1|damageinducibleprotein[Paracoccussanguinis]_gi|694226786|gb|KGJ22541.1|damageinducibleprotein/1	YQSIGTLSRAQ----EA--------------------GLPAFDLIICDEAHRTTGATLDK
gi|746096057|ref|WP_039162453.1|damageinducibleprotein[Gallibacteriumanatis]_gi|703617775|gb|KGQ63868.1|damageinducibleprotei/1	YHSIDVIHQAQ---QKG---------------------LPPFDLVICDEAHRTTGASFDD
gi|737716785|ref|WP_035685631.1|damageinducibleprotein[Avibacteriumparagallinarum]/11643	YHSIDVIHQAQ---QKG---------------------LPPFDLVICDEAHRTTGASFDD
gi|540072553|gb|ERF78672.1|hypotheticalproteinN561_04930[Gallibacteriumanatis12656/12}/11651	YHSIDVIHQAQ---QKG---------------------LPPFDLVICDEAHRTTGASFDD
gi|917509519|ref|WP_052115936.1|damageinducibleprotein[Gallibacteriumanatis]_gi|702414161|gb|KGQ42233.1|damageinducibleprotei/1	YHSIDVIHQAQ---QKG---------------------LPPFDLVICDEAHRTTGASFDD
gi|746082942|ref|WP_039150268.1|damageinducibleprotein[Gallibacteriumanatis]_gi|703617078|gb|KGQ63194.1|damageinducibleprotei/1	YHSIDVIHQAQ---QKG---------------------LPPFDLVICDEAHRTTGASFDD
gi|822522885|ref|WP_046952840.1|damageinducibleprotein[Haemophilushaemolyticus]_gi|821501762|gb|KKZ59267.1|damageinduciblepro/1	YHSIDVIHQAQ---QQG---------------------LGEFDLIICDEAHRTTGASFDD
gi|404381014|gb|EJZ77501.1|putativehelicase[Pasteurellamultocidasubsp.gallicidaP1059]/11681	YHSIDVIHQAQ---KQG---------------------LGEFDLIICDEAHRTTGASIEG
gi|298281660|gb|EFI23149.1|helicaseCterminaldomainprotein[Neisseriasp.oraltaxon014str.F0314]/11493	YHSIDVIHQAQ---AQY--------------------GLPEFDLIICDEAHRTTGATFDG
gi|348013055|gb|EGY51980.1|helicasedomainprotein[Neisseriashayeganii871]/11481	YHSIDVIHQAQ---QQY--------------------GLPEFDLIICDEAHRTTGATWEG
gi|496407140|ref|WP_009116004.1|DNAdamageinducibleprotein[Neisseriawadsworthii]_gi|349794756|gb|EGZ48566.1|helicasedomainprote/	YHSIDVIHQAQ---KQF--------------------GLPEFDLIICDEAHRTTGATFDG
gi|517216441|ref|WP_018405259.1|DNAdamageinducibleprotein[Marinobacterlipolyticus]/11656	YHSIGVINEAQ---TTGSQ------------------PIPGFDLIICDEAHRTTGATFEG
gi|738230264|ref|WP_036185691.1|damageinducibleprotein[Marinobactersp.ELB17]/11671	YHSIGRINEAQ---TTGSQ------------------PIPEFDLIICDEAHRTTGATFEN
gi|447197741|ref|WP_001274997.1|DNAdamageinducibleprotein[Acinetobacterbaumannii]_gi|408512324|gb|EKK13969.1|typeIIIrestrictio/	YHSIDVIAEAQ---KVG---------------------FPEFDLIICDEAHRTTGATFEG
gi|820775897|ref|WP_046739909.1|damageinducibleprotein[Acinetobactersp.AG1]_gi|819909758|gb|KKW75938.1|damageinducibleprotein/1	YHSIDVIAEAQ---KAG---------------------FPEFDLIICDEAHRTTGATFEG
gi|584595163|ref|WP_024160846.1|helicase[Acinetobacterpittii]_gi|576702530|emb|CDI28049.1|helicasedomaincontainingprotein[Acin/	YHSIDVIAEAQ---KVG---------------------FPEFDLIICDEAHRTTGATFEG
gi|514963042|ref|WP_016651366.1|hypotheticalprotein[Acinetobactersp.NIPH2036]_gi|514162665|gb|EPG41391.1|hypotheticalproteinF90	YHSIDVIAEAQ---KAG---------------------FPEFDLIICDEAHRTTGATFEG
gi|515965564|ref|WP_017396147.1|DNAdamageinducibleprotein[Acinetobacterhaemolyticus]/11667	YHSIDVIAEAQ---KAG---------------------FPEFDLIICDEAHRTTGATFEG
gi|491346810|ref|WP_005204747.1|MULTISPECIES:hypotheticalprotein[Acinetobacter]_gi|480335823|gb|ENX56033.1|hypotheticalproteinF	YHSIDVIAEAQ---KAG---------------------FPEFDLIICDEAHRTTGATFEG
gi|693044472|ref|WP_032215268.1|damageinducibleprotein[Escherichiacoli]_gi|607725731|gb|EZE08230.1|damageinducibleprotein[Esc/1	YHSVDVIHQAQ---KLG---------------------FPTFDLIICDEAHRTTGATFEG
gi|742382177|ref|WP_038861304.1|damageinducibleprotein[Cronobactermuytjensii]_gi|924333747|gb|ALB69766.1|damageinducibleprote/1	YHSVEVIHQAQ---KLG---------------------FPEFDFIICDEAHRTTGATFDG
gi|915912804|ref|WP_050927035.1|damageinducibleprotein[Yersiniaenterocolitica]_gi|899805802|emb|CQH32042.1|typeIrestrictionenz/	YHSVEVIHQAQ---KLG---------------------FPAFDFIICDEAHRTTGATFEG
gi|921111327|gb|KON62781.1|typeIrestrictionenzymeEcoKIsubunitR[Komagataeibactereuropaeus]/11492	YHSIDVISRAQ---KEH--------------------DFPEFDLIVCDEAHRTTGVTFGG
gi|582024278|gb|AHI27615.1|DNAhelicaserestrictionenzymeTypeIIIRsubunit[KomagataeibacterxylinusE25]/11636	YHSIDVISRAQ---KEH--------------------DFPEFDLIVCDEAHRTTGVTFGG
gi|347662673|dbj|BAK86128.1|DNAhelicaserestrictionenzymeTypeIIIRsubunit[KomagataeibactermedellinensisNBRC3288]/11737	YHSIDVISRAQ---KEH--------------------DLPEFDLIVCDEAHRTTGVTFGG
gi|917445053|ref|WP_052051471.1|damageinducibleprotein[Acetobactertropicalis]/11429	YHSIDVISQAQ---KEH--------------------GFPEFNLIVCDEAHRTTGVTFGG
gi|453329445|dbj|GAC88472.1|DNAhelicaserestrictionenzymetypeIIIRsubunit[GluconobacterthailandicusNBRC3255]_gi|530287981|dbj|GAD	YHSIDVISQAQ---KEF--------------------GFPEFDLIVCDEAHRTTGVTFGG
gi|889770922|ref|WP_048844278.1|damageinducibleprotein[Gluconobacterfrateurii]/11419	YHSIDVISQAQ---KEF--------------------GFPEFDLIVCDEAHRTTGVTFGG
gi|504886208|ref|WP_015073310.1|hypotheticalprotein[Gluconobacteroxydans]_gi|411027954|gb|AFW01209.1|hypotheticalproteinB932_16	YHSIDVISQAQ---KEF--------------------GFPEFDLIVCDEAHRTTGVTFGG
gi|480040211|gb|ENV79751.1|hypotheticalproteinF942_01641[AcinetobacterursingiiANC3649]/11432	YHSINVISEAQ---NSH--------------------LLPEFDFVICDEAHRTTGATFDG
gi|822498222|ref|WP_046936504.1|damageinducibleprotein[Xanthomonasgardneri]_gi|821751224|gb|KLB07223.1|damageinducibleprotein/1	YHSIDVIHHAQ---HDH--------------------GLAEFDLIVCDEAHRTTGATFES
gi|654059559|ref|WP_027705556.1|damageinducibleprotein[Zymobacterpalmae]/11635	YHSIDVISQAQ---HQY--------------------GLGEFNLVVCDEAHRTTGATYDG
gi|643535620|ref|WP_025224315.1|damageinducibleprotein[Methylophilaceaebacterium11]_gi|577704400|gb|EUJ10546.1|putativehelicas/	YHSIDVISKAQ---FQN--------------------KLADFDLIICDEAHRTTGATFDD
MBA11I/11635	YHSIDVISKAQ---FQN--------------------KLADFDLIICDEAHRTTGATFDD
gi|655355857|ref|WP_028762287.1|damageinducibleprotein[Shewanellacolwelliana]/11632	YHSITVINRAQ---KQH--------------------GLADFDLIICDEAHRTTGATFAD
gi|753204236|ref|WP_041510579.1|damageinducibleprotein[Shewanellasp.cp20]_gi|752457765|gb|KIO36326.1|damageinducibleprotein[S/1	YHSIEVIANAQ---NEF--------------------GLAPFDLIICDEAHRTTGATFAG
KPNNIH30III/11619	YHSIEVIGDAQ---NEH--------------------SMPAFDLIICDEAHRTTGATFAG
gi|749295994|ref|WP_040120352.1|damageinducibleprotein[Klebsiellapneumoniae]_gi|723249300|gb|AIX81926.1|damageinducibleprotei/1	YHSIEVIGDAQ---NEH--------------------SMPAFDLIICDEAHRTTGATFAG
gi|641639739|emb|CDQ54010.1|unnamedproteinproduct[Klebsiellapneumoniae]/11641	YHSIEVIGDAQ---NEH--------------------SMPAFDLIICDEAHRTTGATFAG
gi|490797922|ref|WP_004660060.1|hypotheticalprotein[Acinetobactersp.NIPH236]_gi|479876779|gb|ENU27973.1|hypotheticalproteinF992	YHSIQTVSDAQ---NNE--------------------GMGEFDLIICDEAHRTTGSTHDS
gi|916750926|ref|WP_051357982.1|damageinducibleprotein[Acinetobactertjernbergiae]/11453	YHSINTVSDAQ---NDY--------------------QMAEFDLIICDEAHRTTGSTHDS
gi|691098662|ref|WP_032025060.1|damageinducibleprotein[Acinetobacterbaumannii]_gi|593567521|gb|EXR50735.1|restrictionendonucle/	YHSINTVSDAQ---NEH--------------------QMHEFDLIICDEAHRTTGATHDS
gi|736669405|ref|WP_034676049.1|damageinducibleprotein[Acinetobacterbaumannii]/11643	YHSIQTVSDAQ---NDH--------------------QMHEFDLIICDEAHRTTGSTHDT
gi|491368928|ref|WP_005226826.1|hypotheticalprotein[Acinetobactersp.NIPH1847]_gi|480173259|gb|ENX09797.1|hypotheticalproteinF89	YHSINTVSDAQ---NDH--------------------QMAEFDLIICDEAHRTTGSTHDS
gi|796853993|ref|WP_045795953.1|damageinducibleprotein[Acinetobacterindicus]_gi|787929761|gb|KJV44219.1|damageinducibleprotei/1	YHSINTVSDAQ---NDE--------------------GMGEFDLIICDEAHRTTGSTHDS
gi|295797463|gb|ADG32253.1|typeIIIrestrictionproteinressubunit[ThiomonasintermediaK12]/11676	YHSIDVLHRAQ---HLF--------------------GLEAFDLVVCDEAHRTTGATFDD
gi|499871179|ref|WP_011551913.1|DNAdamageinducibleprotein[Myxococcusxanthus]_gi|108467544|gb|ABF92729.1|putativerestriction/mo/	YHSVEVIHQAQ---KKQ--------------------GLPEFDLIICDEAHRTTGARFDG
gi|495313852|ref|WP_008038600.1|DNAdamageinducibleprotein[Bartonellatamiae]_gi|395424181|gb|EJF90368.1|hypotheticalproteinME5_/	YHSIDVIAKAQ---KEY--------------------GLPEFDLVICDEAHRTTGQTYDG
gi|501453335|ref|WP_012476784.1|DNAdamageinducibleprotein[Leptospirabiflexa]_gi|167777824|gb|ABZ96123.1|Helicase[Leptospirabif/	YHSIDVLSEAQ---KKY--------------------KLGEFDLVICDEAHRTTGATFES
gi|738104613|ref|WP_036063193.1|damageinducibleprotein[Leptospiranoguchii]/11652	YHSIEVIRLAQ---KNH--------------------KLEKFDLVICDEAHRTTGATFEG
gi|410001039|gb|EKO51661.1|typeIIIrestrictionenzyme,ressubunit[Leptospirakirschneristr.200802841]/11651	YHSIDVISLAQ---KNH--------------------ELEKFDLIICDEAHRTTGATFEG
gi|490901851|ref|WP_004763774.1|typeIIIrestrictionenzyme,ressubunit[Leptospirakirschneri]_gi|464209249|gb|EMN24014.1|typeIIIres	YHSIDVISLAQ---KNH--------------------ELEKFDLIICDEAHRTTGATFEG
gi|464398131|gb|EMO65902.1|typeIIIrestrictionenzyme,ressubunit[Leptospirakirschneristr.200803703]/11648	YHSIDVISLAQ---KNH--------------------ELEKFDLIICDEAHRTTGATFEG
gi|945029024|gb|KQQ84900.1|damageinducibleprotein[Xanthomonassp.Leaf131]/11423	YHSIDVVHRSQ---HDY--------------------GLADFDLIICDEAHRTTGATFEG
gi|896511314|ref|WP_049418183.1|damageinducibleprotein[Stenotrophomonasmaltophilia]/11639	YHSIEVLHDAQ---HAH--------------------GLDDFDLIVCDEAHRTTGATFEG
gi|896513082|ref|WP_049419616.1|damageinducibleprotein[Stenotrophomonasmaltophilia]/11638	YHSIEVIHDAQ---LHY--------------------GLGQFDLIVCDEAHRTTGATFEG
gi|896580616|ref|WP_049469425.1|damageinducibleprotein[Stenotrophomonasmaltophilia]/11641	YHSIEVIHDAQ---Q-Q--------------------GLADFDLIVCDEAHRTTGATFEG
gi|648289350|ref|WP_026070499.1|damageinducibleprotein[Stenotrophomonasmaltophilia]/11639	YHSIEVIHDAQ---Q-Q--------------------GLADFDLIVCDEAHRTTGATFEG
gi|656272786|ref|WP_029218442.1|damageinducibleprotein[Xanthomonascassavae]/11639	YHSIEVIHDAQ---Q-Q--------------------GLADFDLIVCDEAHRTTGATFEG
gi|406976236|gb|EKD98747.1|hypotheticalproteinACD_23C00290G0002[unculturedbacterium]/11679	YHSIDTISQAQ---QQH--------------------GLPAFDLIVCDEAHRTTGATFGD

```
gi|828145737|ref|WP_047238120.1|damageinducibleprotein[Chromobacteriumsubtsugae]/11645    YHSIDVISRAQ---LEH--------------------GLADFDLIICDEAHRTTGATFDG
gi|654311174|ref|WP_027824327.1|damageinducibleprotein[Laribacterhongkongensis]/11641    YHSIDVISRAQ---LEH--------------------GLADFDLIICDEAHRTTGATFDG
gi|757242173|gb|KIR15516.1|typeIrestrictionenzymeEcoKIsubunitR[Pseudomonasfluorescens]/11778    YHSIAVISDAQ---HQH--------------------SLADFDLIICDEAHRTTGATFGD
gi|653605161|ref|WP_027616682.1|damageinducibleprotein[Pseudomonassp.URHB0015]/11639    YHSIDVINDAQ---HLH--------------------GLGDFDLIICDEAHRTTGATFGD
gi|520868025|ref|WP_020308060.1|helicase[Pseudomonassyringae]_gi|519159034|gb|EPM49935.1|helicasedomaincontainingprotein[Pseud/    YHSIAVISDAQ---TQH--------------------GLADFDLIICDEAHRTTGATFGD
gi|940156567|gb|KPX71668.1|Helicasedomaincontainingprotein[Pseudomonasamygdalipv.lachrymans]/11677    YHSIGVINDAQ---HLY--------------------GFADFDLIICDEAHRTTGATFGD
gi|505460508|ref|WP_015647610.1|helicase[Pseudomonassp.GLE121]_gi|499110212|ref|YP_007974374.1|helicasedomaincontainingprotein/    YHSIDVISRSQ---KQY--------------------GLEPFDLVICDEAHRTTGATFGD
gi|940235551|gb|KPY45311.1|hypotheticalproteinALO48_200154[Pseudomonassyringaepv.rhaphiolepidis]/11631    YHSIDVISRAQ---KEY--------------------GLEQFDLVICDEAHRTTGATFGD
gi|520880808|ref|WP_020312235.1|helicase[Pseudomonassyringae]_gi|519172158|gb|EPM62027.1|helicasedomaincontainingprotein[Pseud/    YHSIDVISRAQ---LQH--------------------SLAEFDLVICDEAHRTTGATFDE
gi|829046479|ref|WP_047282259.1|damageinducibleprotein[Pseudomonasfluorescens]/11630    YHSIDVISRAQ---HEY--------------------DLAAFDLVVCDEAHRTTGATFGE
gi|499415493|ref|WP_011102960.1|DNAdamageinducibleprotein[Pseudomonassyringaegroupgenomosp.3]_gi|28867279|ref|NP_789898.1|heli/    YHSIDVISRAQ---HEY--------------------DLAAFDLVICDEAHRTTGATFDD
gi|730238299|ref|WP_033942897.1|damageinducibleprotein[Pseudomonasaeruginosa]/11629    YHSIDVISRAQ---ADH--------------------GLPPFDLVICDEAHRTTGATFGD
gi|495310125|ref|WP_008034873.1|DNAdamageinducibleprotein[Pseudomonassp.GM60]_gi|398186685|gb|EJM74052.1|putativehelicase[Pseu/    YHSIDVISRAQ---HEH--------------------GLATFDLIICDEAHRTTGATFDD
gi|779992653|ref|WP_045428690.1|damageinducibleprotein[Pseudomonassp.MT1]_gi|699967872|dbj|BAP80334.1|DNAhelicaserestrictione/1    YHSIDVISRAQ---LDH--------------------GLPAFDLVICDEAHRTTGATFGE
gi|786037289|ref|WP_045733274.1|damageinducibleprotein[Pseudomonaspseudoalcaligenes]_gi|782987904|gb|KJU79152.1|damageinducib/1    YHSIDVISRAQ---ADH--------------------GLPAFDLVICDEAHRTTGATFGD
gi|749666366|ref|WP_040265445.1|damageinducibleprotein[Pseudomonasrhodesiae]/11632    YHSIDVISRAQ---ADH--------------------GLAAFDLVICDEAHRTTGATFDD
gi|734902433|ref|WP_034133698.1|damageinducibleprotein[Pseudomonasfluorescens]/11635    YHSIDVISRAQ---NDY--------------------GLAAFDLVICDEAHRTTGATFDD
gi|490551031|ref|WP_004416146.1|typeIIrestrictionmodificationsystemrestriction/helicasesubunit[Pseudomonassyringae]_gi|4432823/    YHSIGVISRAQ---ADH--------------------GLAAFDLVICDEAHRTTGATFGD
gi|489199911|ref|WP_003109152.1|DNAdamageinducibleprotein[Pseudomonasaeruginosa]_gi|552648708|gb|ERY63184.1|hypotheticalprotei/    YHSIDVISRAQ---ADH--------------------GLPAFDLVICDEAHRTTGATFGD
gi|730641237|ref|WP_034061328.1|damageinducibleprotein[Pseudomonasaeruginosa]/11636    YHSIDVISRAQ---HEY--------------------DLAAFDLVICDEAHRTTGATFDD
gi|751304980|ref|WP_041013386.1|damageinducibleprotein[Pseudomonasxanthomarina]_gi|703407611|emb|CEG51014.1|Helicasedomaincon/1    YHSIDVISRAQ---LEY--------------------GLDAFDLVICDEAHRTTGATFGE
gi|939335207|ref|WP_054881520.1|damageinducibleprotein[Pseudomonassp.NBRC111128]/11632    YHSIDVISRAQ---ADH--------------------GLAAFDLVICDEAHRTTGATFGD
gi|757693555|ref|WP_042923792.1|damageinducibleprotein[Pseudomonaschloritidismutans]_gi|780910610|gb|KJS78302.1|damageinducib/1    YHSIDVISRAQ---HEY--------------------DLAAFDLVICDEAHRTTGATFGE
gi|665953944|ref|WP_031319989.1|damageinducibleprotein[Pseudomonastolaasii]/11626    YHSIDVISRAQ---HEH--------------------GLADFDLVICDEAHRTTGATFGD
gi|771600573|ref|WP_045213746.1|damageinducibleprotein[Pseudomonassp.21]_gi|770081123|gb|KJJ98634.1|damageinducibleprotein[Ps/1    YHSIDVISRAQ---HEH--------------------GLPAFDLIICDEAHRTTGATFDD
gi|740871396|ref|WP_038656644.1|damageinducibleprotein[Pseudomonasstutzeri]_gi|627772214|gb|AHY41437.1|damageinducibleprotein/1    YHSIDVISRAQ---HDH--------------------GLEAFDLVICDEAHRTTGATFGE
gi|946242205|gb|KRC93782.1|damageinducibleprotein[Pseudomonassp.Root9]/11634    YHSIDVISRAQ---ADH--------------------GLAAFDLVICDEAHRTTGATFGD
gi|553777322|ref|WP_023109107.1|MULTISPECIES:hypotheticalprotein[Pseudomonas]_gi|552638674|gb|ERY53184.1|hypotheticalproteinQ05    YHSIDVISRAQ---ADH--------------------GLAAFDLVICDEAHRTTGATFGD
gi|896383370|ref|WP_049324618.1|damageinducibleprotein[Pseudomonasstutzeri]/11627    YHSIDVISRAQ---HEF--------------------DLAAFDLVICDEAHRTTGATFGE
gi|696158510|gb|AIT41787.1|putativehelicase[Pseudomonassp.LM8]/11630    YHSIDVISRAQ---HDY--------------------ELAAFDLVICDEAHRTTGATFGE
gi|504634892|ref|WP_014821994.1|DNAdamageinducibleprotein[Pseudomonasstutzeri]_gi|390985249|gb|AFM35242.1|helicasedomainconta/1    YHSIDVISRAQ---HDH--------------------GLDAFDLVICDEAHRTTGATFGE
gi|544783974|ref|WP_021206729.1|DNAdamageinducibleprotein[Pseudomonasstutzeri]_gi|533224779|gb|EQM79688.1|DNAdamageinduciblep/1    YHSIDVISRAQ---HDH--------------------DLAAFDLVICDEAHRTTGATFGE
gi|504567385|ref|WP_014754487.1|MULTISPECIES:DNAdamageinducibleprotein[Pseudomonas]_gi|388559493|gb|AFK68634.1|helicasedomain/1    YHSIDVISRAQ---HEH--------------------DMATFDLVICDEAHRTTGATFDD
gi|654441469|ref|WP_027912052.1|damageinducibleprotein[Pseudomonassp.URIL14HWK12:I7]/11630    YHSIDVISRAQ---ADH--------------------GLAAFDLVICDEAHRTTGATFGD
gi|941470066|ref|WP_055127894.1|damageinducibleprotein[Pseudomonasmediterranea]/11631    YHSIDVISRAQ---ADH--------------------GLAAFDLVICDEAHRTTGATFGD
gi|557815599|ref|WP_023446184.1|DNAdamageinducibleprotein[Pseudomonaschloritidismutans]_gi|557364081|gb|ESQ98190.1|DNAdamagei/1    YHSIDVISRAQ---ADH--------------------GLAAFDLVICDEAHRTTGATFGD
gi|727814791|ref|WP_033898247.1|damageinducibleprotein[Pseudomonassp.FH1]_gi|567648005|gb|ETK23229.1|helicasedomaincontaining/1    YHSIDVISRAQ---ADH--------------------GLAAFDLVICDEAHRTTGATFDD
gi|503278702|ref|WP_013513363.1|DNAdamageinducibleprotein[Desulfovibrioaespoeensis]_gi|316942375|gb|ADU61426.1|typeIIrestrict/    YHSIDVLSQAQ---YDH--------------------DLPQFDLIICDEAHRTTGATFAG
gi|505228796|ref|WP_015415898.1|typeIIrestrictionendonucleasesubunitR[Desulfovibriopiezophilus]_gi|451896976|emb|CCH49855.1|Ty    YHSIDVLNAAQ---YEH--------------------DLPEFDLIICDEAHRTTGATFAN
gi|517732543|ref|WP_018902751.1|hypotheticalprotein[Variovoraxparadoxus]/11675    YHSIDVISNAQ---HAF--------------------GLADFDLIVCDEAHRTTGATFDG
gi|550909475|ref|WP_022662570.1|DNAdamageinducibleprotein[Desulfovibriolongus]/11637    YHSIDVVSRAQ---HEH--------------------GLADFDLIICDEAHRTTGATFGD
gi|945494542|gb|KQV50024.1|damageinducibleprotein[Massiliasp.Root335]/11647    YHSIDVISRAQ---HEH--------------------GLAEFDLIVCDEAHRTTGATFGD
gi|334196917|gb|AEG70102.1|helicasedomainprotein[RalstoniasolanacearumPo82]/11663    YHSIDVISCAQ---KEF--------------------GLTAFDLIVCDEAHRTTGATFGD
gi|656006746|ref|WP_029048170.1|damageinducibleprotein[Cupriavidussp.amp6]/11661    YHSIDVISRAQ---KEF--------------------RLADFDLVVCDEAHRTTGATFGD
BPS2196ORF3054P/11659    YHSIDVISRAQ---KQF--------------------GLADFDLIVCDEAHRTTGATFGD
gi|740932312|ref|WP_038717332.1|damageinducibleprotein[Burkholderiapseudomallei]_gi|760211882|gb|KIX58314.1|damageinduciblepr/1    YHSIDVISRAQ---KQF--------------------GLADFDLIVCDEAHRTTGATFGD
gi|755810813|ref|WP_042583861.1|damageinducibleprotein[Burkholderiasp.MSHR3999]_gi|755174952|gb|KIP19790.1|DEAD/DEAHboxhelicas/    YHSIDVISRAQ---KQF--------------------GLADFDLIVCDEAHRTTGATFGD
gi|484586570|gb|AGK48195.1|DEAD/DEAHboxhelicasefamilyprotein[BurkholderiathailandensisMSMB121]/11646    YHSIDVISRAQ---KQF--------------------GLADFDLIVCDEAHRTTGATFGD
gi|737582935|ref|WP_035553941.1|damageinducibleprotein[Burkholderiasp.9120]/11658    YHSIDVISRAQ---KQF--------------------GLGDFDLIVCDEAHRTTGATFGN
gi|740989872|ref|WP_038773225.1|damageinducibleprotein[Burkholderiapseudomallei]_gi|705645291|gb|KGS70691.1|DEAD/DEAHboxhelica/    YHSIDVISRAQ---KQF--------------------GLTDFDLIVCDEAHRTTGATFGD
gi|654912525|ref|WP_028363424.1|damageinducibleprotein[Burkholderiasp.JPY366]/11659    YHSIDVISRAQ---KQF--------------------GLADFDLIVCDEAHRTTGATFGD
gi|740955879|ref|WP_038740512.1|damageinducibleprotein[Burkholderiapseudomallei]_gi|714684932|gb|KGW74438.1|DEAD/DEAHboxhelica/    YHSIDVISRAQ---KQF--------------------GLADFDLIVCDEAHRTTGVSYGG
gi|919203714|ref|WP_052759215.1|damageinducibleprotein[Xanthomonasaxonopodis]/11388    YQSIDVISQAQ--KKH---------------------GVGEFDLIVCDEAHRTTGVTLSG
gi|522152381|ref|WP_020663589.1|hypotheticalprotein[Amycolatopsisbenzoatilytica]/11605    YQSITTVSAAQ--Q-A---------------------GFPDFDLVICDEAHRTTGVTLAG
gi|663196013|ref|WP_030233899.1|damageinducibleprotein[Streptomycessp.NRRLS455]/11641    YQSIGTIAKAQ--E-M---------------------GLDAFDLVLCDEAHRTTGVTLAG
gi|750429553|ref|WP_040711444.1|damageinducibleprotein[Nocardiopsispotens]/11622    YQSITTVAAAQ--Q-Q---------------------GLARFDLVLCDEAHRTTGVTLSG
gi|518656139|ref|WP_019819475.1|hypotheticalprotein[Saccharomonosporasaliphila]/11623    YQSIATVAAAQ--K-K---------------------GLPRFDLVLCDEAHRTTGVTLSG
gi|759722933|ref|WP_043433758.1|damageinducibleprotein[Streptomycespluripotens]_gi|733404978|gb|KHK94361.1|damageinduciblepro/1    YQSIATISAAQ--K-Q---------------------GLPRFDLILCDEAHRTTGVTLSG
gi|663424167|ref|WP_030419030.1|damageinducibleprotein[Streptomycessp.NRRLF5065]/11627    YQSIATISAAQ--K-Q---------------------GMPRFDLILCDEAHRTTGVTLSG
gi|663412917|ref|WP_030408353.1|damageinducibleprotein[Streptomycessp.NRRLF5917]/11630    YQSIATVSAAQ--K-K---------------------GLPRFDLILCDEAHRTTGVTLSG
gi|671521483|ref|WP_031506489.1|damageinducibleprotein[Streptomycesmegasporus]/11630    YQSIATVSAAQ--K-K---------------------GLPRFDLILCDEAHRTTGVTLSG
gi|740053144|ref|WP_037902776.1|damageinducibleprotein[Streptomycessp.TAA204]/11626    YHSIAAVADAQ--K-K---------------------ALPRFDLILCDEAHRTTGVTLVG
gi|663409375|ref|WP_030404974.1|MULTISPECIES:damageinducibleprotein[Streptomyces]/11627    YQSIATVSEAQ--K-K---------------------GLPRFDLILCDEAHRTTGVTLAG
gi|746628608|ref|WP_039654277.1|damageinducibleprotein[Streptomycessp.MUSC125]_gi|745644381|gb|KIE25557.1|damageinducibleprot/1    YQSIATVSEAQ--K-K---------------------GLPRFDLILCDEAHRTTGVTLAG
gi|727322066|ref|WP_033777320.1|damageinducibleprotein[Salinisporapacifica]/11627    YQSIGTVAAAQ--R-K---------------------GLSRFDLILCDEAHRTTGVTLAG
gi|653653242|ref|WP_027655726.1|damageinducibleprotein[Salinisporapacifica]/11618    YQSIGTVAAAQ--R-Q---------------------GLSRFDLILCDEAHRTTGVTLAG
gi|648632543|ref|WP_026324294.1|damageinducibleprotein[Salinisporapacifica]/11621    YQSIATVAAAQ--Q-Q---------------------GLPRFDLILCDEAHRTTGVTLAG
```

gi|881018002|ref|WP_048738463.1|damageinducibleprotein[Corynebacteriumfalsenii]/11676 YQSLPAVHDAQ---QQ---------------------GLEDFDLVICDEAHRTTGITLAG
gi|505465526|ref|WP_015652417.1|helicase[Corynebacteriumcallunae]_gi|459385704|gb|AGG67995.1|helicase[CorynebacteriumcallunaeDS YQSLPAVHAAQ---EA---------------------GAEPFDLVICDEAHRTTGITLAG
gi|41223062|emb|CAF19007.1|DNAorRNAhelicaseofsuperfamilyII[CorynebacteriumglutamicumATCC13032]/11646 YQSLPAVHAAQ---EA---------------------GAEPFDLVICDEAHRTTGITLAG
CGL13032I/11643 YQSLPAVHAAQ---EA---------------------GAEPFDLVICDEAHRTTGITLAG
gi|751257081|ref|WP_040967675.1|damageinducibleprotein[Corynebacteriumglutamicum]_gi|748811620|gb|AJE67931.1|damageinduciblep/1 YQSLPAIHEAQ---QQ---------------------GLEDFDLVICDEAHRTTGVTLAG
gi|749040539|ref|WP_040086524.1|damageinducibleprotein[Corynebacteriumhumireducens]/11663 YQSLPAIHDAQ---QE---------------------GLADFDLVICDEAHRTTGVTLAG
gi|491662899|ref|WP_005519615.1|helicase[Corynebacteriummatruchotii]_gi|224946756|gb|EEG27965.1|helicaseCterminaldomainprotein/ YQSLAAIHDAQ---QH---------------------GFEPFDLIICDEAHRTTGATLLG
gi|746717312|ref|WP_039676231.1|damageinducibleprotein[Corynebacteriumminutissimum]_gi|734991118|gb|KHO29219.1|damageinducibl/1 YQSIEAIHEAQ--AEY---------------------GMDDFDLVICDEAHRTTGVTLAG
gi|908654892|emb|CQD15529.1|SuperfamilyIIDNA/RNAhelicase(modularprotein)[Corynebacteriumstriatum]/11674 YQSIEAIHEAQ--AEY---------------------GMDDFDLVICDEAHRTTGVTLVG
gi|895994446|ref|WP_049064418.1|damageinducibleprotein[Corynebacteriumaurimucosum]/11667 YQSIEAIHEAQ--AEY---------------------GMDDFDLVICDEAHRTTGVTLAG
gi|896123331|ref|WP_049145390.1|damageinducibleprotein[Corynebacteriumaurimucosum]/11669 YQSIEAIHEAQ--AEY---------------------GMDDFDLVICDEAHRTTGVTLAG
gi|817681178|ref|WP_046645422.1|damageinducibleprotein[Corynebacteriumstriatum]_gi|817551679|gb|KKO79868.1|damageinduciblepro/1 YQSIEAIHEAQ--AEY---------------------GMDDFDLVICDEAHRTTGVTLAG
gi|896139239|ref|WP_049159146.1|damageinducibleprotein[Corynebacteriumaurimucosum]/11675 YQSIEAIHEAQ--AEY---------------------GMDDFDLVICDEAHRTTGVTLAG
gi|489943947|ref|WP_003847254.1|helicase[Corynebacteriumammoniagenes]_gi|295968218|gb|EFG81467.1|helicaseCterminaldomainprotei/ YQSLPAVHDAQMTEGG---------------------GIENFDLIICDEAHRTTGVTLAG
gi|516653464|ref|WP_018022453.1|helicase[Corynebacteriumdoosanense]_gi|697778282|gb|AIT61018.1|damageinducibleprotein[Coryneba/ YQSLPAVHNAQ---ED---------------------GMAPFDLVICDEAHRTTGVTLAG
gi|493894179|ref|WP_006840129.1|helicase[Corynebacteriumlipophiloflavum]_gi|227078819|gb|EEI16782.1|helicaseCterminaldomainpro/ YQSLPAIHNAQ---TN---------------------GLDEFDIVICDEAHRTTGVTLAG
gi|740831472|ref|WP_038616755.1|damageinducibleprotein[Corynebacteriumpseudotuberculosis]_gi|666103058|gb|AIG06935.1|putativeh/ YQSLPAVHEAQ---QQ---------------------GLADFDLVICDEAHRTTGVTLAG
gi|518023322|ref|WP_019193530.1|helicase[Corynebacteriumtimonense]/11655 YQSLPAVHDAQ---QE---------------------GLEAFDLVICDEAHRTTGVTLAG
gi|552767697|ref|WP_023019559.1|MULTISPECIES:hypotheticalprotein[Corynebacterium]_gi|550739013|gb|ERS38609.1|hypotheticalprotei YQSLPTVHDAQ---QH---------------------GLDDFDLVICDEAHRTTGVTLAG
gi|652659121|ref|WP_027017812.1|damageinducibleprotein[Corynebacteriumpseudodiphtheriticum]/11644 YQSLPTVHDAQ---HN---------------------GLDDFDLVICDEAHRTTGVTLAG
gi|749039309|ref|WP_040085390.1|damageinducibleprotein[Corynebacteriumhumireducens]/11657 YQSLPAIHEAQ---QE---------------------GLADFDLVICDEAHRTTGVTLAG
gi|740835591|ref|WP_038620874.1|damageinducibleprotein[Corynebacteriumulcerans]_gi|698973854|gb|AIU33664.1|HelicaseCterminald/1 YQSIQAVHEAQ---QA---------------------GLEEFDLVICDEAHRTTGVTLAN
gi|805307599|ref|WP_046096452.1|damageinducibleprotein[Corynebacteriumulcerans]_gi|803357323|gb|AKA97638.1|Damageinduciblepro/1 YQSIQAVHEAQ---QA---------------------GLEEFDLVICDEAHRTTGVTLAN
gi|504083555|ref|WP_014317549.1|helicase[Corynebacteriumdiphtheriae]_gi|371584249|gb|AEX47914.1|superfamilyIIDNA/RNAhelicase[Co YQSLPAVHEAQ---AH---------------------GLEDFDLVICDEAHRTTGVTLAG
gi|896182193|ref|WP_049193580.1|damageinducibleprotein[Corynebacteriumjeikeium]/11645 YQSLPAVHEAQ---AH---------------------GLEEFDLVICDEAHRTTGVTLAG
gi|917636459|ref|WP_052205460.1|damageinducibleprotein[Corynebacteriumriegelii]_gi|914295811|gb|AKV59187.1|damageinduciblepro/1 YQSLPAVHDAL---AE---------------------GLDDFDLVICDEAHRTTGVTLAG
gi|737013522|ref|WP_035009205.1|damageinducibleprotein[Corynebacteriumjeikeium]/11651 YQSLPAVHDAQ---EN---------------------GLDDFDIVICDEAHRTTGVTLAG
gi|737006291|ref|WP_035002094.1|damageinducibleprotein[Corynebacteriumjeikeium]/11658 YQSLPAVHDAQ---QQ---------------------GLDDFDLVICDEAHRTTGVTLAG
gi|737010378|ref|WP_035006133.1|damageinducibleprotein[Corynebacteriumjeikeium]/11650 YQSLPAVHDAQ---QE---------------------GLDDFDLVICDEAHRTTGVTLAG
gi|737010473|ref|WP_035006228.1|damageinducibleprotein[Corynebacteriumjeikeium]/11657 YQSLPAVHDAQ---EN---------------------GLDAFDLVICDEAHRTTGLTLAG
gi|737006250|ref|WP_035002053.1|damageinducibleprotein[Corynebacteriumjeikeium]/11658 YQSVPAVHDAQ---EN---------------------GLDAFDLVICDEAHRTTGVTLAG
gi|923018168|ref|WP_053412254.1|damageinducibleprotein[Corynebacteriumlactis]_gi|922845184|gb|ALA67504.1|helicase[Corynebacter/ YQSLPAVHAAQ---QA---------------------GLDEFDLVICDEAHRTTGVTLAG
gi|923018885|ref|WP_053412970.1|damageinducibleprotein[Corynebacteriumlactis]_gi|922845845|gb|ALA68165.1|helicase[Corynebacter/ YQSLSAVHAAQ---QV---------------------GLDEFDLVICDEAHRTTGVTLAG
gi|512464870|ref|WP_016422869.1|hypotheticalprotein[Corynebacteriumsp.HFH0082]_gi|512047830|gb|EPD47505.1|hypotheticalproteinHM YQSLPAVHEAQ---QQ---------------------GLDDFDLVICDEAHRTTGITLAG
gi|881012697|ref|WP_048733406.1|damageinducibleprotein[Corynebacteriumurealyticum]/11670 YQSLPAVHEAQ---QQ---------------------GLDDFDLVICDEAHRTTGITLAG
gi|755608478|ref|WP_042530519.1|damageinducibleprotein[Corynebacteriumsingulare]_gi|754340239|gb|AJI78730.1|putativehelicase[C/ YQSIQAVHEAQ---QV---------------------GLDDFDLVICDEAHRTTGITLAS
gi|491421796|ref|WP_005279593.1|helicase[Corynebacteriumaccolens]_gi|227077180|gb|EEI15143.1|helicaseCterminaldomainprotein[Co/ YQSLPAVHDAQ---QQ---------------------GLDDFDLVICDEAHRTTGITLAG
gi|550752286|gb|ERS51451.1|hypotheticalproteinHMPREF1282_00033[Corynebacteriumsp.KPL1856]_gi|550759961|gb|ERS58964.1|hypothetic YQSLPAVHEAQ---QQ---------------------GLDDFDLVICDEAHRTTGITLAG
gi|497955053|ref|WP_010269209.1|helicase[Corynebacteriumbovis]/11599 YQSLQAVHGAQ---EL---------------------GADDFDLVLCDEAHRTTGVTLAG
gi|860580040|ref|WP_048470569.1|hypotheticalprotein[Mycobacteriumchlorophenolicum]_gi|859098575|gb|KMO76871.1|typeIrestrictione YQSLPTVADAQ---SL---------------------GVDTFDLVICDEAHRTTGAILSD
gi|262085905|gb|ACY21873.1|typeIIIrestrictionproteinressubunit[GordoniabronchialisDSM43247]/11632 YQSLPAVADAQ---NR---------------------GVDPFDLVICDEAHRTTGVTVSG
gi|495996338|ref|WP_008720917.1|typeIIIrestrictionendonucleasesubunitR[Rhodococcussp.AW25M09]_gi|443412350|emb|CCQ18455.1|TypeI YQSLPTVAAAQ---GM---------------------GVDAFDLVICDEAHRTTGVTVAG
gi|739284636|ref|WP_037147410.1|damageinducibleprotein[Rhodococcusfascians]/11641 YQSLPTVAAAQ---GL---------------------GVDAFDLVICDEAHRTTGVTVAG
gi|739328195|ref|WP_037190574.1|damageinducibleprotein[Rhodococcusfascians]/11643 YQSLPTVAAAQ---GM---------------------GVDAFDLVICDEAHRTTGVTVAG
gi|811698335|emb|CMN21041.1|helicase[Mycobacteriumtuberculosis]/11725 YQSLPTVAKAQ---RL---------------------GVDEFDLVMCDEAHRTTGVTLAG
gi|801577898|emb|CFK23773.1|helicase[Mycobacteriumtuberculosis]/11675 YQSLPTVAKAQ---RL---------------------GVDEFDLVMCDEAHRTTGVTLAG
MTU18II/11606 YQSLPTVAKAQ---RL---------------------GVDEFDLVMCDEAHRTTGVTLAG
gi|887719858|emb|CMT27886.1|helicase[Mycobacteriumtuberculosis]/11667 YQSLPTVAKAQ---RL---------------------GVDEFDLVMCDEAHRTTGVTLAG
gi|621694743|gb|KAS87156.1|hypotheticalproteinAL70_01801[MycobacteriumtuberculosisTKK_03_0115]/11604 YQSLPTVAKAQ---RL---------------------GVDEFDLVMCDEAHRTTGVTLAG
gi|888128072|emb|CLQ84422.1|helicase[Mycobacteriumtuberculosis]_gi|889917090|emb|CLY86893.1|helicase[Mycobacteriumtuberculosis] YQSLPTVAKAQ---RL---------------------GVDEFDLVMCDEAHRTTGVTLAG
gi|884823424|emb|CKL96672.1|helicase[Mycobacteriumtuberculosis]/11466 YQSLPTVAKAQ---RL---------------------GVDEFDLVMCDEAHRTTGVTLAG
gi|505103239|ref|WP_015290341.1|hypotheticalprotein[Mycobacteriumcanettii]_gi|432162733|emb|CCK60117.1|Conservedproteinofunknow YQSLPTVAKAQ---RL---------------------GVDEFDLVMCDEAHRTTGVTLAG
gi|505116250|ref|WP_015303352.1|hypotheticalprotein[Mycobacteriumcanettii]_gi|432158726|emb|CCK56026.1|Conservedproteinofunknow YQSLPTVAKAQ---RL---------------------GVDEFDLVMCDEAHRTTGVTLAG
gi|432154735|emb|CCK51974.1|Conservedproteinofunknownfunction(part1)[MycobacteriumcanettiiCIPT140060008]/11588 YQSLPTVAKAQ---RL---------------------GVDEFDLVMCDEAHRTTGVTLAG
gi|809008027|ref|WP_046295375.1|hypotheticalprotein[Mycobacteriumsp.UM_Kg17]/11624 YQSLPVVAEAQ---KL---------------------GVDEFDLVICDEAHRTTGVTLAG
gi|808802633|ref|WP_046284139.1|hypotheticalprotein[Mycobacteriumsp.UM_NZ2]/11624 YQSLPAVAEAQ---KL---------------------GVDEFDLVICDEAHRTTGVTLAG
gi|750125774|ref|WP_040429852.1|damageinducibleprotein[Corynebacteriumnuruki]/11668 YQSLPAIHEAQ---QH---------------------GADAFDLIICDEAHRTTGVTLDG
gi|602237614|gb|EYT60960.1|damageinducibleprotein[Dietziasp.UCDTHP]/11473 YQSLPAVAAAQ--SEH---------------------GVDPFDLVICDEAHRTTGVTLAG
gi|665897412|ref|WP_031264618.1|damageinducibleprotein[Dietziasp.UCDTHP]_gi|602237599|gb|EYT60945.1|helicase[Dietziasp.UCDTH/11 YQSLPSVAAAQ--SEH---------------------GVDPFDLVICDEAHRTTGVTLAG
gi|502903674|ref|WP_013138650.1|helicase[Segniliparusrotundus]_gi|296181291|gb|ADG98197.1|typeIIIrestrictionproteinressubunit[S YQSLPVVADAQ---KL---------------------GVPEFDLVLCDEAHRTTGVTLFG
gi|780248306|ref|WP_045538930.1|hypotheticalprotein[Gordoniasp.no.9]_gi|778142514|gb|KJR06917.1|hypotheticalproteinUG54_12320[G YQSLPVVSQAQ---AQ---------------------GLSAFDLVLCDEAHRTTGVTLTG
gi|665897368|ref|WP_031264574.1|damageinducibleprotein[Dietziasp.UCDTHP]_gi|602239137|gb|EYT62483.1|helicase[Dietziasp.UCDTH/11 YQSLPTVSKAQ---SL---------------------GVDPFDLVICDEAHRTTGITLVG
gi|493372923|ref|WP_006329236.1|helicase[Gordoniarhizosphera]_gi|403207917|dbj|GAB88168.1|hypotheticalproteinGORHZ_006_00370[Go YQSLPAIAKAQ---EL---------------------GVDPFDLIICDEAHRTTGVTLAG
gi|764904870|ref|WP_044487821.1|hypotheticalprotein[Mycobacteriumtuberculosis]_gi|625005353|gb|KBZ57120.1|hypotheticalproteinK8 YQSLPVVADAQ---QL---------------------GVAPFDLVICDEAHRTTGVTLED
gi|927068212|ref|WP_053778441.1|damageinducibleprotein[Gordoniasp.NB41Y]_gi|926660541|gb|EMP14526.2|damageinducibleprotein[G/11 YQSLPVVAQAQ---RD---------------------GVDPFDLVICDEAHRTTGVTLEG
gi|935514169|ref|WP_054429894.1|damageinducibleprotein[Mycobacteriumimmunogenum]_gi|929801120|gb|KPG29247.1|damageinduciblepr/1 YQSLPVVADAQ---KL---------------------GVAPFDMVICDEAHRTTGVTLED
gi|941317728|ref|WP_055118574.1|hypotheticalprotein[Mycobacteriumperegrinum]/11657 YQSLPVVADAQ---KL---------------------GVAPFDMAICDEAHRTTGVTLED

gi|933683673|gb|ALG86377.1|hypotheticalproteinACH46_20120[Gordoniasp.QH11]/11636 YQSLPVVAAAQ--AEH---------------------GVDPFDLVICDEAHRTTGVTLAG
gi|873553947|emb|CRY84110.1|Predictedhelicase[Nocardiafarcinica]/11688 YQSLPAVAGAQ--AEH---------------------GVEPFDLVICDEAHRTTGVTLVG
gi|499525749|ref|WP_011212389.1|helicase[Nocardiafarcinica]_gi|54019339|dbj|BAD60707.1|hypotheticalprotein[NocardiafarcinicaIFM YQSLPAVAGAQ--AEH---------------------GVEPFDLVICDEAHRTTGVTLVG
gi|640109609|ref|WP_024794187.1|damageinducibleprotein[Tomitellabiformata]/11653 YQSLPVIAAAQ---TQ---------------------GVDPFDLVICDEAHRTTGVTLFG
gi|640109991|ref|WP_024794334.1|hypotheticalprotein[Tomitellabiformata]/11639 YQSLPVVAAAQ---TQ---------------------GVDPFDLVICDEAHRTTGVTLFG
gi|939125496|ref|WP_054831438.1|damageinducibleprotein[Rhodococcuserythropolis]/11669 YQSLPAVAKAQ---EL---------------------GVDPFDLVICDEAHRTTGVTLFG
gi|910258550|ref|WP_050062711.1|hypotheticalprotein[Rhodococcussp.RD6.2]_gi|846871967|emb|CRK49225.1|conservedhypotheticalprote YQSLPVVAAAQ---TQ---------------------GVDPFDIVICDEAHRTTGVTLFG
gi|797148189|gb|KJY52358.1|putativeHelicase[Bifidobacteriumasteroides]/11801 YQSIQTTIDAQ---RM---------------------GMPEFDLVVCDEAHRTAGTSDNR
gi|535476570|gb|EFG27357.2|hypotheticalproteinHMPREF9020_00999[ScardoviainopinataF0304]/11827 YQSIDTIIAAQ--AQE---------------------GLPEFDLVICDEAHRTAGVADNV
gi|737482729|ref|WP_035462496.1|hypotheticalprotein[Alicyclobacillusmacrosporangiidus]/11512 YQSLQVVADAQ---QQ---------------------GLPVFHVAIADEAHRTTGVTREE
gi|685434354|gb|KGA95679.1|helicase[BacillusalcalophilusATCC27647=CGMCC1.3604]/11575 YQSIDVIIEAQ---KN---------------------GFYEFDLVICDEAHRTTGATESG
gi|945370253|gb|KQU27251.1|helicase[Bacillussp.Leaf13]/11557 YQSIDVIIEAQ---KQ---------------------GFYEFDLVVCDEAHRTTGATELG
gi|880997015|ref|WP_048719116.1|helicase[Enterococcushirae]/11563 YQSIDVIIEAQ---KN---------------------GFYEFDLVICDEAHRTTGKTELG
gi|945356929|gb|KQU14100.1|helicase[Bacillussp.Leaf49]/11547 YQSIDVISEAQ---KH---------------------GFYEFDLIICDEAHRTTGATELG
gi|763482876|gb|KIZ53217.1|helicase[Bacillussafensis]/11563 YQSIDVIIEAQ---KN---------------------GFYEFDLVVCDEAHRTTGATESG
gi|852223292|ref|WP_048309707.1|helicase[Anaerobacillusmacyae]_gi|850322910|gb|KMM38590.1|helicase[Anaerobacillusmacyae]/11573 YQSIDVIMDAQ---KN---------------------GFYEFDLVVCDEAHRTTGATEVG
gi|739708665|ref|WP_037562619.1|helicase[Sporolactobacillusterrae]/11573 YQSIDVIIEAQ---KN---------------------GFYEFDLIICDEAHRTTGATEIG
gi|496687561|ref|WP_009329104.1|MULTISPECIES:helicase[Bacillus]_gi|317390867|gb|EFV71668.1|endonucleaseandmethylaseLlaGI[Bacill YQSIDVIIDAQ---KN---------------------GFYEFDLVVCDEAHRTTGATEAG
gi|930213599|gb|KPH74225.1|helicase[Oceanobacilluscaeni]/11569 YQSIDVIIDAQ---KN---------------------GFYEFDLVVCDEAHRTTGATEAG
gi|516366003|ref|WP_017756036.1|helicase[Bacillussp.ZYK]/11571 YQSIDVIIEAQ---KK---------------------GFYEFDLVICDEAHRTTGATETG
gi|488909977|ref|WP_002821052.1|helicase[Oenococcusoeni]_gi|399965230|gb|EJN99855.1|endonucleaseandmethylaseLlaGI[Oenococcusoen YQSIDVIHRAQ---KE---------------------GFPDFDLIVADEAHRTTGAHALG
gi|495019967|ref|WP_007745780.1|helicase[Oenococcuskitaharae]_gi|366983682|gb|EHN59081.1|typeIIIrestrictionenzyme,ressubunit[Oe YQSIDVIHRAQ---KQ---------------------GFPDFDLIVADEAHRTTGAHALG
gi|489666119|ref|WP_003570406.1|typeIIIrestrictionendonuclease[Lactobacilluscasei]_gi|410527834|gb|EKQ02696.1|typeIIIrestrictio YQSIDVIQRAQ---QQ---------------------GYPEFDLIIADEAHRTTGSHAAN
gi|511447623|gb|EPC78110.1|superfamilyIIDNA/RNAhelicase[Lactobacillusparacaseisubsp.paracaseiLpp221}/11615 YQSIDVIHRAQ---EM---------------------GYPEFDLIIADEAHRTTGSHAAN
gi|497700411|ref|WP_010014595.1|helicase[Lactobacilluscoryniformis]/11585 YQSIDVIHQAQ---AL---------------------GYPEFDLIIADEAHRTTGSHAAN
gi|489784048|ref|WP_003687939.1|helicase[Lactobacillusmali]_gi|394428539|gb|EJF01080.1|superfamilyIIDNA/RNAhelicase[Lactobacill YQSIDVIHQAQ---EQ---------------------GYPEFDLIIADEAHRTTGAHAAN
gi|446780825|ref|WP_000858081.1|helicase[Staphylococcusaureus]_gi|374396604|gb|EHQ67835.1|typeIIIrestrictionenzyme,ressubunit[S YQSIDVVKKAQ---DL---------------------GYPKFDFIICDEAHRTTGITEEG
gi|510796658|ref|WP_016171456.1|hypotheticalprotein[Enterococcusdispar]_gi|508167532|gb|EOT44083.1|hypotheticalproteinOMK_00227 YQSIGVMHEAQ---EN---------------------GFPEFDFIISDEAHRTTGFHEQN
gi|612613473|gb|EZS30411.1|hypotheticalproteinW603_02482[StaphylococcusaureusVET0353R]_gi|612881917|gb|EZU93992.1|hypotheticalp YQSIDVIHKAQ---EN---------------------GFPTFDFIISDEAHRTTGFHEQN
gi|488332893|ref|WP_002402278.1|helicase[Enterococcusfaecalis]_gi|306513864|gb|EFM82466.1|helicaseCterminaldomainprotein[Enter/ YQSIEVLHEAQ---EK---------------------GFPEFDIVICDEAHRTTGAKALG
gi|921166029|ref|WP_053111806.1|helicase[Enterococcusdurans]_gi|915771660|gb|AKX85928.1|helicase[Enterococcusdurans]/11562 YQSIEVLHEAQ---EK---------------------GFPEFDIIICDEAHRTTGAKALG
gi|498525358|ref|WP_010825030.1|hypotheticalprotein[Enterococcusfaecalis]_gi|487059631|gb|EOJ86727.1|hypotheticalproteinWOC_005 YQSIEVLHEAQ---KK---------------------GFPEFDIIICDEAHRTTGAKALG
gi|498412624|ref|WP_010722070.1|hypotheticalprotein[Enterococcusfaecium]_gi|486634654|gb|EOF75253.1|hypotheticalproteinSGC_0253 YQSIEVLHEAQ---EK---------------------GFPNFDIIICDEAHRTTGAKALG
gi|498408534|ref|WP_010718364.1|hypotheticalprotein[Enterococcusfaecium]_gi|486609608|gb|EOF50873.1|hypotheticalproteinSCW_0257 YQSIEVLHEAQ---EK---------------------GFPEFDIIICDEAHRTTGAKALG
gi|498407475|ref|WP_010717483.1|hypotheticalprotein[Enterococcusfaecalis]_gi|486594855|gb|EOF36348.1|hypotheticalproteinSCK_005 YQSIEVLHEAQ---EK---------------------GFPEFDIVICDEAHRTTGAKALG
gi|514885392|ref|WP_016615344.1|helicase[Enterococcusfaecalis]_gi|514505843|gb|EPH71676.1|helicaseprotein[Enterococcusfaecalis0 YQSIEVLHEAQ---EK---------------------GFPEFDIVICDEAHRTTGAKALG
gi|325157603|gb|EGC69759.1|helicaseCterminaldomainprotein[EnterococcuscasseliflavusATCC12755]/11561 YQSIEVLHEAQ---EK---------------------GFPEFDIVICDEAHRTTGAKALG
gi|895856206|ref|WP_048967668.1|helicase[Enterococcusfaecalis]/11551 YQSIEVLHEAQ---EK---------------------GFPEFDIIICDEAHRTTGAKALG
gi|639458836|ref|WP_024636366.1|helicase[Enterococcusfaecium]_gi|658176502|gb|KEI51159.1|helicase[EnterococcusfaeciumUC7256]/1/ YQSIKILHEAQ---EK---------------------GFPEFDIVICDEAHRTTGAKALG
gi|488266511|ref|WP_002337719.1|hypotheticalprotein[Enterococcusfaecium]_gi|430581409|gb|ELB19854.1|hypotheticalproteinOIQ_0351 YQSIGVLHEAQ---EK---------------------GFPEFDIVICDEAHRTTGAKALG
gi|446353356|ref|WP_000431211.1|helicase[Staphylococcusaureus]_gi|253726329|gb|EES95058.1|helicaseCterminaldomainprotein[Staph/ YQSIDVIGKAQ---KE---------------------GFPEFDLIISDEAHRTTGAHEMN
gi|672712068|emb|CDM14336.1|DNAorRNAhelicaseofsuperfamilyII[StaphylococcusepidermidisPM221]/11464 YQSIDVIGDAQ---KE---------------------GFPEFDLIISDEAHRTTGAHEMN
gi|896298990|ref|WP_049279905.1|helicase[Staphylococcusaureus]/11571 YQSIDVIGDAQ---KE---------------------GFPEFDLIISDEAHRTTGAHEMN
gi|541903432|gb|ERH36190.1|hypotheticalproteinSEQU_00085[StaphylococcusequorumUMCCNS924]/11578 YQSIDVIGDAQ---KE---------------------EFPEFDLIISDEAHRTTGAHEMN
gi|488289747|ref|WP_002360955.1|helicase[Enterococcusfaecalis]_gi|256682216|gb|EEU21911.1|endonucleaseandmethylaseLlaGI[Enteroc YQSIDVIGNAQ---KE---------------------GFPEFDLIISDEAHRTTGAHESS
gi|494392045|ref|WP_007207894.1|helicase[Enterococcusitalicus]_gi|315483798|gb|EFU74284.1|helicaseCterminaldomainprotein[Enter/ YQSIDVIGGAQ---KD---------------------GFPEFDLIISDEAHRTTGAHESS
gi|446350692|ref|WP_000428547.1|helicase[Bacilluscereus]_gi|228727580|gb|EEL78721.1|RestrictionmodificationsystemLlaBIII[Bacil/ YQSIDVIGKAQ---KE---------------------GFPEFDLIISDEAHRTTGAYEAN
gi|488255302|ref|WP_002326510.1|hypotheticalprotein[Enterococcusfaecium]_gi|430480276|gb|ELA57459.1|hypotheticalproteinOGE_0473 YQSIEVIGNAQ---KE---------------------GFPEFDLIISDEAHRTTGAHEAS
gi|843253770|ref|WP_047916698.1|helicase[Lactococcuspiscium]_gi|802124677|emb|CEN29502.1|EndonucleaseandmethylaseLlaGI,TypeISPr YQSIEVIGEAQ---KE---------------------GFPEFDFIISDEAHRTTGAHEAA
LlaBIII/11578 YQSIEVIGEAQ---KE---------------------GFPEFDFIISDEAHRTTGAHEAA
gi|499320531|ref|WP_011011023.1|helicase[Listeriainnocua]_gi|16415805|emb|CAC42019.1|pli0021[ListeriainnocuaClip11262]/11569 YQSIEVIGEAQ---KE---------------------GFPEFDFIISDEAHRTTGAHEAT
gi|760026859|ref|WP_043709973.1|helicase[Weissellacibaria]_gi|758337165|gb|KIU20061.1|UvsWhelicase[Weissellacibaria]/11567 YQSIDVIGEAQ---KQ---------------------GLPEFDFIISDEAHRTTGAHEST
LlaGI/11570 YQSIEVIGEAQ---KE---------------------GFPEFDFIISDEAHRTTGAHEAA
gi|499988414|ref|WP_011669132.1|helicase[Lactococcuslactis]_gi|116109019|gb|ABJ74139.1|DNAorRNAhelicaseofsuperfamilyII[Lactococ YQSIEVIGEAQ---KE---------------------GFPEFDFIISDEAHRTTGAHEAT
gi|685938360|ref|WP_031670319.1|helicase[Listeriamonocytogenes]_gi|590403387|gb|EXL23261.1|hypotheticalproteinX846_1956[Listeri YQSIEVIGEAQ---KE---------------------GFPEFDFIISDEAHRTTGAHEAA
gi|530793219|gb|EQC56240.1|helicase[Lactococcuslactissubsp.cremorisTIFN5]_gi|530793247|gb|EQC56263.1|helicase[Lactococcuslactis YQSIEVIGEAQ---KE---------------------GFPEFDFIISDEAHRTTGAHEAA
gi|695270006|ref|WP_032492075.1|hypotheticalprotein[Listeriamonocytogenes]_gi|695212872|ref|YP_009081522.1|hypotheticalproteinp YQSIEVIGEAQ---KE---------------------GFPEFDFIISDEAHRTTGAHEAA
gi|504101310|ref|WP_014335297.1|helicase[Leuconostocmesenteroides]_gi|859247905|gb|AKO63885.1|helicase(plasmid)[Leuconostocmese YQSIDVIGEAQ---KH---------------------GLPEFDFIISDEAHRTTGAHEST
gi|488905190|ref|WP_002816265.1|helicase[Leuconostocmesenteroides]_gi|227351522|gb|EEJ41777.1|helicaseCterminaldomainprotein[L/ YQSIDVIGEAQ---KH---------------------GLPEFDFIIADEAHRTTGAHEST
gi|518121285|ref|WP_019291493.1|MULTISPECIES:hypotheticalprotein[Lactococcus]/11588 YQSIDVLGQAQ---KE---------------------GFPEFDLIIADEAHRTTGARLED
gi|754857213|ref|WP_042217902.1|helicase[Lactococcusgarvieae]/11549 YQSIDVLGNAQ---KA---------------------GFPDFDLIIADEAHRTTGTTALG
gi|640048455|ref|WP_024784762.1|helicase[Streptococcusmutans]/11564 YQSIDVLGKAQ---IN---------------------GFPEFDLIIGDEAHRTTGAKALG
gi|512695361|ref|WP_016476323.1|hypotheticalprotein[Veillonellasp.HPA0037]_gi|512079853|gb|EPD79018.1|hypotheticalproteinHMPREF YQSIDVLGEAQ---KN---------------------GFPEFDLIIADEAHRTTGAKALG
gi|636963514|ref|WP_024403747.1|helicase[Streptococcussuis]/11574 YQSIEVLGKAQ---KN---------------------GFPDFDLIIADEAHRTTGAKALG
gi|881115312|ref|WP_048802949.1|helicase[Staphylococcuspasteuri]/11573 YQSIEVLGAAQ---KE---------------------GFPEFNLIISDEAHRTTGAKSLN
gi|446063996|ref|WP_000141851.1|helicase[Streptococcusmitis]_gi|307618725|gb|EFN97864.1|endonucleaseandmethylaseLlaGI[Streptoco YQSIDVLGEAQ---KE---------------------GFPEFDLIIADEAHRTTGAKAFG
gi|488206609|ref|WP_002277817.1|restrictionmodificationsystemLlaBIII[Streptococcusmutans]_gi|449173033|gb|EMB75626.1|restricti/ YQSIAVLDKAQ---KD---------------------GFPEFDLIIADEAHRTTGAKALG

gi|640023290|ref|WP_024781621.1|helicase[Streptococcusmutans]/11566	YQSIAVLGKAQ---KD---------------------GFPEFDLIIADEAHRTTGAKALG
gi|814946581|ref|WP_046393781.1|helicase[Streptococcusuberis]_gi|810600885|gb|KKF57499.1|helicase[StreptococcusuberisB362]/1156	YQSIDVLGKAQ---KL---------------------GFPEFDLIIADEAHRTTGAKALG
gi|765470762|ref|WP_044720336.1|helicase[Streptococcussuis]/11559	YQSIDVLGKAQ---KN---------------------GFPDFDLIIADEAHRTTGAKSFG
gi|654507127|ref|WP_027976443.1|helicase[Streptococcusdevriesei]/11558	YQSIDVLGKAQ---QN---------------------GFPDFDLIIADEAHRTTGAKALG
gi|530812902|gb|EQC72481.1|DNAorRNAhelicaseofsuperfamilyII[Streptococcussp.HSISS2]/11570	YQSIDVLGEAQ---QN---------------------GFPEFDLIIADEAHRTTGAKALG
gi|489158846|ref|WP_003068504.1|helicase[Streptococcusintermedius]_gi|355367114|gb|EHG14827.1|hypotheticalproteinHMPREF9682_002	YQSIDVLGRAQ---KN---------------------GFPEFDLIIADEAHRTTGAKALG
RM.Lru27782ORF13200P/11620	YQSIDVIHQAQ---EA---------------------GYPEFDLIIADEAHRTTGSSKL-
gi|492676546|ref|WP_005919236.1|DNAphosphorothioationsystemrestrictionenzyme[Pediococcusacidilactici]_gi|482517924|gb|EOA08103.	YQSIDVVHKAQ---NS---------------------GLPEFDLIVADEAHRTTGANEM-
gi|498183116|ref|WP_010497272.1|helicase[Lactobacillusacidipiscis]/11563	YQSIDVIHKAQ---AQ---------------------GYPEFDLIIADEAHRTTGASQL-
gi|822100485|ref|WP_046921865.1|helicase[Lactobacillusruminis]_gi|821684442|gb|KLA43321.1|restrictionmodificationsystemLlaBIII/	YQSIDVIHDAQ---EA---------------------GYPEFDLIIADEAHRTTGAAKL-
gi|935565832|ref|WP_054450311.1|helicase[Lactobacilluskunkeei]/11573	YQSIDVIKQAQ---EM---------------------GYPEFDLIIADEAHRTTGANKL-
gi|752296887|ref|WP_041152901.1|helicase[Lactobacilluskunkeei]_gi|751663520|gb|KIM18358.1|helicase[Lactobacilluskunkeei]/11577	YQSIDVIKQAQ---EM---------------------GYPEFDLIIADEAHRTTGANKL-
gi|495745291|ref|WP_008469870.1|helicase[Lactobacillushominis]_gi|394484442|emb|CCI81207.1|RestrictionmodificationsystemLlaBII/	YQSIDVIKEAQ---KN---------------------GFPEFDLIVADEAHRTASSYAEK
gi|918672414|ref|WP_052543134.1|helicase[Lactobacillussp.]/11564	YQSIDVIKQAQ---KD---------------------GYPEFDLIIADEAHRTTGAIAEK
gi|799141631|ref|WP_045927261.1|helicase[Lactobacilluskullabergensis]_gi|797155335|gb|KJY59264.1|putativerestrictionendonucleas	YQSIDVIKKAQ---EA---------------------GYPEFDLIVADEAHRTTGAQAL-
gi|504379990|ref|WP_014567092.1|helicase[Lactobacillusjohnsonii]_gi|329666726|gb|AEB92674.1|putativerestrictionendonuclease[Lac	YQSIDVLKQAQ---KD---------------------GFPEFDLIIADEAHRTTGAIAER
gi|489880179|ref|WP_003783646.1|DNAdamageinducibleprotein[Actinomycesnaeslundii]_gi|399901561|gb|EJN84440.1|typeIIIrestriction/	YQSIDAVAQAQ--E-L---------------------GAPGFDLVICDEAHRTTGATVAG
gi|496524497|ref|WP_009232780.1|DNAdamageinducibleprotein[Actinomycessp.oraltaxon849]_gi|365265413|gb|EHM95177.1|hypotheticalp/	YQSIDAVAQAQ--E-L---------------------GAPGFDLVICDEAHRTTGATPLG
gi|915397782|ref|WP_050783167.1|damageinducibleprotein[Actinomycesoris]/11694	YQSIDAVAQAQ--E-L---------------------GAPGFDLVICDEAHRTTGATVSG
gi|502925248|ref|WP_013160224.1|helicase[Propionibacteriumfreudenreichii]_gi|296921272|emb|CBL55824.1|helicase[Propionibacteriu	YQSIDVVAKAQ--RES----------------------GQQFDLILCDEAHRTTGVTLTG
gi|732164352|emb|CEG87481.1|helicase[Propionibacteriumfreudenreichii]/11618	YQSIDVVAAAQ--RE----------------------SAEQFDLVLCDEAHRTTGVTLSG
gi|739047402|ref|WP_036925058.1|damageinducibleprotein[Propionibacteriumthoenii]/11596	YQSIDVVARAQ--KQS---------------------GAGPFDLVLCDEAHRTTGVTLAG
gi|739112839|ref|WP_036982533.1|damageinducibleprotein[Propionibacteriumjensenii]/11594	YQSIDVVARAL--KQS---------------------GAGPFDLVLCDEAHRTTGVTLAG
gi|918023558|ref|WP_052314121.1|damageinducibleprotein[Nocardiathailandica]/11612	YQSIDVISQAQ--L-L---------------------GLGDFDLVICDEAHRTTGVTLAG
gi|498380271|ref|WP_010694427.1|DNAdamageinducibleprotein[Saccharopolysporaspinosa]/11605	YQSIDVVAQAQ--Q-L---------------------DVPAFDLVICDEAHRTTGVTLAG
gi|749991459|ref|WP_040339809.1|damageinducibleprotein[CandidatusBlastococcusmassiliensis]/11597	YQSIDVVTRAQ--E-L---------------------GLPGFDLVICDEAHRTTGVTLAE
gi|754177801|ref|WP_041796231.1|damageinducibleprotein[Modestobactermarinus]/11592	YQSIDVVTRAQ--E-L---------------------GLPAFDLVVCDEAHRTTGVTLAD
gi|504188248|ref|WP_014375350.1|DNAdamageinducibleprotein[Blastococcussaxobsidens]_gi|378782790|emb|CCG02456.1|putativeHelicas/	YQSIDVVTRAQ--E-L---------------------GLPDFDLVICDEAHRTTGVTLAD
gi|946476713|gb|KRF26798.1|damageinducibleprotein[Phycicoccussp.Soil803]/11609	YQSIDVVAQAQ--R-D---------------------GVPEFDLVIADEAHRTTGVTLAD
gi|700176672|gb|KGN37509.1|DNAdamageinducibleprotein[KnoelliasubterraneaKCTC19937]/11633	YQSIDVVAQAQ--K-D---------------------GVPDFDLIIADEAHRTTGVTLAD
gi|700168841|gb|KGN29914.1|hypotheticalproteinN802_10215[KnoelliasinensisKCTC19936]/11631	YQSIDVVAQAQ--K-D---------------------GVPDFDLVIADEAHRTTGVTLAD
gi|946426241|gb|KRE76610.1|damageinducibleprotein[Arthrobactersp.Soil763]/11606	YQSIDVVAKAQ--REA---------------------GLEPFDLIICDEAHRTTGVTLAD
gi|517318525|ref|WP_018500927.1|hypotheticalprotein[Frankiasp.BCU110501]/11592	YQSIDVVAQAQ----Q---------------------GLREFDLIVADEAHRTTGTTLAG
gi|499072429|ref|WP_010851327.1|Helicase[Tetrasphaeraelongata]_gi|478756579|emb|CCH71501.1|putativeHelicase[Tetrasphaeraelongat	YQSIDVVAQAQ--QQG---------------------DLPAFDLIVCDEAHRTTGTTLAG
gi|750080666|ref|WP_040385350.1|damageinducibleprotein[Demetriaterragena]/11625	YQSIDVVSNAQ--KAA---------------------GVGAFDLIICDEAHRTTGVTIAG
gi|752558292|ref|WP_041229523.1|damageinducibleprotein[Dermacoccusnishinomiyaensis]_gi|664689315|gb|AIF41904.1|damageinducibl/1	YQSLEVVAAAQ--KAG---------------------GFDAFDLIICDEAHRTTGAKAAG
gi|829089213|ref|WP_047310701.1|damageinducibleprotein[Dermacoccussp.PE3]_gi|828384812|gb|KLO63770.1|damageinducibleprotein[D/1	YQSIDVVAAAQ--KHG---------------------GFEAFDLIVCDEAHRTTGVSVAG
gi|748756635|ref|WP_040014750.1|damageinducibleprotein[Dermacoccussp.Ellin185]/11596	YQSIDVVAGAQ--AQG---------------------GFGPFDLIICDEAHRTTGVSVAG
gi|916534724|ref|WP_051141848.1|damageinducibleprotein[Actinomycesneuii]/11632	YQSIDVVTAAQ---KA---------------------GMDDFDLIICDEAHRTTGVTLAG
gi|921140030|ref|WP_053090276.1|damageinducibleprotein[Rothiamucilaginosa]/11616	YHSIDAVSEAQ---AA---------------------GVGEFDLIICDEAHRTTGVTLAD
gi|896406751|ref|WP_049343749.1|damageinducibleprotein[Rothiamucilaginosa]/11647	YHSIDAVSAAQ---AA---------------------GVGEFDLIICDEAHRTTGVTLAD
gi|723608234|gb|AIY01610.1|putativehelicase[Arthrobactersp.PAMC25486]/11613	YQSIEAVRDAQ---QA---------------------GLPDFDLVICDEAHRTTGATLVD
gi|946394527|gb|KRE45160.1|damageinducibleprotein[Arthrobactersp.Soil736]/11616	YQSIDAVAQAQ---QA---------------------GLPDFDLIICDEAHRTTGATLSG
gi|515765304|ref|WP_017197904.1|hypotheticalprotein[Arthrobactersp.M2012083]/11648	YQSIDAVAQAQ---KA---------------------GLPDFDLIICDEAHRTTGATLAG
gi|945315587|gb|KQT73127.1|damageinducibleprotein[Microbacteriumsp.Leaf436]/11612	YQSLPVISKAQ---GM---------------------GLPEFDLIVSDEAHRTTGAAMSD
gi|759908497|ref|WP_043594599.1|damageinducibleprotein[Frigoribacteriumsp.MEB024]_gi|758271359|gb|KIU03346.1|damageinduciblep/1	YQSIDVVAKAQ---AE---------------------GLAEFDLVICDEAHRTAGFTLKD
gi|945072850|gb|KQR34237.1|hypotheticalproteinASF75_00055[Curtobacteriumsp.Leaf154]/11597	YQSIDVVAKAQ---AE---------------------GLADFDLVICDEAHRTAGFSRQG
gi|551263152|ref|WP_022883363.1|DNAdamageinducibleprotein[Glaciibactersuperstes]/11621	YQSIDVVAKAQ---AA---------------------GLAEFDLVICDEAHRTAGFTLAS
gi|945452341|gb|KQV08491.1|hypotheticalproteinASC63_10170[Leifsoniasp.Root112D2]/11623	YQSIAVVAEAQ---AA---------------------GLPEFDLVVCDEAHRTTGVTLSG
gi|742759483|gb|KIC69912.1|damageinducibleprotein[Kocuriarhizophila]/11610	YQSIDVLHQAQ---QM---------------------GFPDFDLIVCDEAHRTTGATLAG
gi|737919995|ref|WP_035885304.1|damageinducibleprotein[Kocuriarhizophila]/11611	YQSIDVLHQAQ---QM---------------------GFPDFDLIVCDEAHRTTGATLAG
gi|737997701|ref|WP_035959325.1|damageinducibleprotein[Kocuriamarina]_gi|725823884|gb|KHE75603.1|damageinducibleprotein[Kocur/1	YQSIDVLHEAQ---QM---------------------GFPDFDLIVCDEAHRTTGATLAG
gi|664521070|ref|WP_031037650.1|damageinducibleprotein[Streptomycessp.NRRLWC3774]/11624	YQSIATVSEAQ---KR---------------------GLPRFDLILCDEAHRTTGVTLAG
gi|500101034|ref|WP_011777041.1|DNAdamageinducibleprotein[Arthrobacteraurescens]_gi|119951547|gb|ABM10457.1|putativeHelicase[A/	YQSIDVVAQAQ---GL---------------------GLGEFDLIVCDEAHRTTGLTQGD
gi|916871953|ref|WP_051479009.1|damageinducibleprotein[Arthrobactersp.H5]/11588	YQSIDVVAQAQ---AL---------------------GLPEFDLIVCDEAHRTTGLTQGD
gi|612084649|gb|EZP43369.1|TypeIIIrestrictionenzyme,ressubunit[Micrococcusluteus]/11625	YQSIDVVRQAQ---DL---------------------GLAEFDLVVCDEAHRTTGVTEQG
gi|759739708|ref|WP_043450422.1|damageinducibleprotein[Arthrobacterphenanthrenivorans]_gi|742758264|gb|KIC68716.1|damageinduc/1	YQSIDVIHQAQ---KA---------------------GLPEFDLIVCDEAHRTTGITEAD
gi|636845448|ref|WP_024366823.1|damageinducibleprotein[Arthrobactersp.TB26]/11605	YQSIDVIAQAQ---AL---------------------GLPDFDVILCDEAHRTTGITEAE
gi|500009025|ref|WP_011689743.1|DNAdamageinducibleprotein[Arthrobactersp.FB24]_gi|116613051|gb|ABK05760.1|typeIIIrestrictionen/	YQSIDVIAQAQ---AL---------------------GLPDFDVILCDEAHRTTGITEAE
gi|821249847|gb|KKZ12083.1|hypotheticalproteinTQ37_06090[CandidatusSynechococcusspongiarum15L]/11436	YQSSPMIGAAQ--QNH---------------------GLPDFDLAVADEAHRTAGALVPG
gi|739446902|ref|WP_037306885.1|damageinducibleprotein[Ruegeriahalocynthiae]/11592	YQSLPVITAAQ---EQ---------------------GLQQFDLIICDEAHRTTGATIDG
gi|640620081|ref|WP_025048131.1|damageinducibleprotein[Sulfitobactermediterraneus]_gi|751956650|gb|KIN77213.1|TypeIIIrestricti/	YQSIPVITAAQ---EQ---------------------GLQEFDLVICDEAHRTTGATIDG
gi|740247634|ref|WP_038088583.1|damageinducibleprotein[Thiobacillusprosperus]_gi|684049748|gb|KFZ90853.1|putativehelicase[Thio/	YQSIQVVIDAQ--RSG---------------------GLPAFDLVICDEAHRTTGLTLPK
gi|518420321|ref|WP_019590528.1|hypotheticalprotein[Thioalkalivibriosp.ALE20]/11603	YQSIQVVADAQ--S-Q---------------------GLGTFDLVICDEAHRTTGLTLPG
gi|500156826|ref|WP_011831496.1|DNAdamageinducibleprotein[Methylibiumpetroleiphilum]_gi|124261892|gb|ABM96881.1|adeninespecifi/	YQSIQVVADAQ--K-L---------------------GLGEFDLVVSDEAHRTTGLTLAG
gi|769978537|ref|WP_045094522.1|damageinducibleprotein[Legionellafallonii]_gi|698841736|emb|CEG55682.1|Putativeproteinwithboxh/	YQSIQVIADAQ--K-L---------------------DLGEFDLIICDEAHRTTGLTMLD
gi|553752541|ref|WP_023085344.1|hypotheticalprotein[Pseudomonasaeruginosa]_gi|552201952|gb|ERU62653.1|hypotheticalproteinQ088_0	YQSIQVVADAQ--K-Q---------------------GLGRFDLIVCDEAHRTTGLTLPG

```
gi|880980418|ref|WP_048704461.1|damageinducibleprotein[Thauerahumireducens]_gi|875132785|gb|KMU70944.1|damageinducibleprotein/1    YQSIQVVADAQ--R-Q---------------------GLGSFDLIICDEAHRTTGLTLPG
gi|740135732|ref|WP_037982815.1|damageinducibleprotein[Thauerasp.27]/11603    YQSIQVVADAQ--R-Q---------------------GLGAFDLIICDEAHRTTGLTLPG
gi|738017371|ref|WP_035978332.1|damageinducibleprotein[Burkholderiaglumae]/11380    YQSIQVVADAQ--K-R---------------------GLGEFDLIICDEAHRTTGLTLPD
gi|498506003|ref|WP_010806671.1|typeIIIrestrictionenzyme,ressubunit[Pandoraeasp.SD62]_gi|490314561|gb|EON12879.1|typeIIIrestri/    YQSIQVVADAQ--K-Q---------------------GLWDFDLIICDEAHRTTGLTLPD
gi|500117748|ref|WP_011793753.1|DNAdamageinducibleprotein[Acidovoraxcitrulli]_gi|120587742|gb|ABM31182.1|typeIIIrestrictionenz/    YQSIQVVADAQ--K-G---------------------GLGEFDLIVCDEAHRTTGLTLPS
gi|651617328|ref|WP_026611444.1|damageinducibleprotein[Methylocaldumszegediense]/11601    YQSIQVVADAQ--Q-Q---------------------GLGEFDLIVCDEAHRTTGLTLPG
gi|737210208|ref|WP_035194994.1|damageinducibleprotein[Acidithiobacillusferrivorans]_gi|669953220|emb|CDQ11755.1|Adeninespecif/    YQSIEVVHEAQ---KA---------------------GCPAFDLVICDEAHRTAGVAQFD
gi|935697386|ref|WP_054506180.1|damageinducibleprotein[Achromobactersp.]_gi|928716153|emb|CUK12836.1|Hefnuclease[Achromobacter/    YQSIEVVRLAQ---EK---------------------TGMVFNLVICDEAHRTAGYTAPG
gi|429502517|gb|ELA00827.1|adeninespecificDNAmethyltransferase[Cupriavidussp.HMR1]/11599    YQSIEVVHRAQ---QK---------------------TGMVFDLVICDEAHRTAGYTAPG
gi|506356642|ref|WP_015876361.1|DNAdamageinducibleprotein[Burkholderiaglumae]_gi|237877158|gb|ACR29491.1|AdeninespecificDNAmet/    YQSIDVVHRAQ---EK---------------------TGLLFDLVICDEAHRTAGYTAPK
gi|696406787|ref|WP_032978003.1|damageinducibleprotein[Bordetellahinzii]_gi|625335076|gb|KCB49343.1|typeIIIrestrictionenzyme,r/    YQSIEVVHQAQ---QK---------------------TGMVFDLVVCDEAHRTAGYTAPG
gi|817529125|gb|KKO72587.1|damageinducibleprotein[Kerstersiagyiorum]/11592    YQSIEVVHQAQ---QK---------------------TGMVFDLVVCDEAHRTAGYTAPG
gi|737048910|ref|WP_035044118.1|damageinducibleprotein[Aquabacteriumsp.NJ1]_gi|699052896|gb|KGM38706.1|damageinducibleprotein/1    YQSIDVVHQAQ---QK---------------------TGIEFDLVVCDEAHRTAGYTVPG
gi|822492519|ref|WP_046933319.1|MULTISPECIES:damageinducibleprotein[Xanthomonas]_gi|821703525|gb|KLA61196.1|damageinduciblepr/1    YQSIEVVHQAQ---QK---------------------TGIEFDLVVCDEAHRTAGYTAPG
gi|751252861|ref|WP_040963740.1|damageinducibleprotein[Xanthomonasfuscans]_gi|749808363|gb|KIJ03887.1|damageinducibleprotein[/1    YQSIEVVHQAQ---QK---------------------TGIEFDLVVCDEAHRTAGYTAPG
gi|496774980|ref|WP_009368792.1|DNAdamageinducibleprotein[Bilophilasp.4_1_30]_gi|345039902|gb|EGW44205.1|hypotheticalproteinHM/    YQSIDVVHQAQ---AA---------------------AGFTFDLIICDEAHRTAGYTAKD
gi|499241912|ref|WP_010939452.1|DNAdamageinducibleprotein[Desulfovibriovulgaris]_gi|46580580|ref|YP_011388.1|adeninespecificDN/    YQSIAVVHEAQ---QQ---------------------AGFTFDLIVCDEAHRTAGYTPKG
gi|504274913|ref|WP_014462015.1|DNAdamageinducibleprotein[Bacillusmegaterium]_gi|345446919|gb|AEN91936.1|AdeninespecificDNAmet/    YQSIEVISEAQ---KI---------------------GLGEFDLIIGDEAHRTTGVTLAG
gi|504895966|ref|WP_015083068.1|typeIIrestrictionenzymeandmethyltransferasesubtypeG[Anabaenasp.90]_gi|413972424|gb|AFW96512.1|t    YQSIQAISDAQ---KK---------------------GLPEFDLIICDEAHRTTGVTISG
gi|518327765|ref|WP_019497972.1|hypotheticalprotein[Calothrixsp.PCC7103]/11600    YQSIQSVADAQ---AK---------------------GLPEFDLIICDEAHRTTGVTLQG
gi|652765503|ref|WP_027079917.1|damageinducibleprotein[Luteimonasmephitis]/11593    YQSIEVVAGAQ---KK---------------------GLGDFDLVVCDEAHRTTGVTLAD
gi|896539951|ref|WP_049442123.1|damageinducibleprotein[Stenotrophomonasmaltophilia]/11591    YQSIDVISQAQ--KKH---------------------GVGEFDLIVCDEAHRTTGVTLSG
gi|500217900|ref|WP_011888043.1|DNAdamageinducibleprotein[Geobacillusthermodenitrificans]_gi|134267954|gb|ABO68149.1|Conserved/    YQSIDVICNAQ---KA---------------------GMPAFDLVICDEAHRTTGVVQ-G
gi|696570298|ref|WP_033102014.1|damageinducibleprotein[Thermoactinomycesdaqus]/11594    YQSIDVVSEAQ---KA---------------------GLPAFDLVICDESHRTTGVVR-G
gi|251772067|gb|EES52637.1|putativehelicase[Leptospirillumferrodiazotrophum]/11618    YQSIQVIHDAQ--EKF---------------------SLPSFDLAICDEAHRTTGVTAQG
gi|738124101|ref|WP_036082108.1|damageinducibleprotein[Leptospirillumferriphilum]_gi|685243306|gb|KGA94177.1|putativehelicase[/    YQSIQVIHDSQ--EKF---------------------GLPPFDLTICDEAHRTTGVTAQG
gi|558699223|ref|WP_023524766.1|helicase[Leptospirillumsp.GroupII'CF1']_gi|206603764|gb|EDZ40244.1|Putativehelicase[Leptospiri/    YQSIQVIHDAQ--EKF---------------------SLPSFDLAICDEAHRTTGVTAQG
gi|406774462|gb|AFS53887.1|putativehelicase[LeptospirillumferriphilumML04]/11610    YQSIEVLHEAQ--IHH---------------------GLPEFDLVICDEAHRTTGATQSG
gi|740719463|ref|WP_038504749.1|damageinducibleprotein[Leptospirillumferriphilum]_gi|640838125|gb|AIA30173.1|damageinduciblep/1    YQSIEVLHEAQ--IHH---------------------GLPEFDLVICDEAHRTTGVTPSG
gi|517810766|ref|WP_018980974.1|hypotheticalprotein[Saccharibacterfloricola]/11733    YQSIATVHEAQ--KEAY--------------------GLPEFDLVICDEAHRTTGLVEQG
gi|502471675|ref|WP_012798533.1|helicase[Slackiaheliotrinireducens]_gi|256791761|gb|ACV22431.1|predictedhelicase[Slackiaheliotr    YQSIQVVSDAQ--AMG----------------------LPEFDLVICDEAHRTTGAKEAS
gi|657871713|ref|WP_029576297.1|hypotheticalprotein[Bifidobacteriumthermacidophilum]_gi|673007521|gb|KFJ02535.1|helicase[Bifido    YQSIDVIHQAQ--QNG----------------------LFDFDLTICDEAHRTTGVMDGE
gi|673004757|gb|KFI99791.1|DNAhelicaserestrictionenzymetypeIIIRessubunit[Bifidobacteriumthermacidophilumsubsp.porcinum]/11682    YQSIDVIHQAQ--QNG----------------------LFDFDLTICDEAHRTTGVMDGE
gi|917316092|ref|WP_051922804.1|hypotheticalprotein[Bifidobacteriumstellenboschense]_gi|673003774|gb|KFI98812.1|helicase[Bifido    YQSIQVIHDAQ--ELG----------------------LPAFDLTICDEAHRTTGVMDGE
gi|705415694|ref|WP_033502332.1|hypotheticalprotein[Bifidobacteriumpseudolongum]_gi|672982411|gb|KFI77685.1|DNAdamageinducible/    YQSIDVIHQAQ--ELG----------------------LSGFDLIICDEAHRTTGVMDGE
gi|916591418|ref|WP_051198509.1|hypotheticalprotein[Bifidobacteriumsp.AGR2158]/11704    YQSIDVIHEAQ--ELG----------------------LCDFDLIVCDEAHRTTGTMDDD
gi|772242982|gb|AJW78255.1|hypotheticalproteinVO01_03165[Clavibactermichiganensissubsp.insidiosus]/11642    YQSIDAIATAQ--QLG-------------------AP---AFDLIICDEAHRTTGATLAG
gi|916649818|ref|WP_051256909.1|damageinducibleprotein[Actinomycesgraevenitzii]/11677    YQSIEVIHQAQ--ELA-------------------GETWRDFDLVICDEAHRTTGAKLTT
gi|496504578|ref|WP_009212861.1|helicase[Actinomycessp.oraltaxon180]_gi|315312695|gb|EFU60776.1|helicase[Actinomycessp.oraltaxo    YQSIEVIHRAQ--ELA-------------------GDEWRDFDLVICDEAHRTTGATLTG
gi|494862206|ref|WP_007588306.1|helicase[Actinomycessp.ICM39]_gi|398362799|gb|EJN46470.1|typeIIrestrictionenzyme,ressubunit[Ac    YQSIEVIHRAQ--EIA-------------------GDEWRDFDLIICDEAHRTTGATLTG
gi|881057111|ref|WP_048774974.1|damageinducibleprotein[Sanguibacterkeddieii]/11697    YQSIEVIHRAQ--EIA-------------------GDEWRDFDLIICDEAHRTTGATLTG
gi|881020459|ref|WP_048740808.1|damageinducibleprotein[Kytococcussedentarius]/11704    YQSIDVIHRAQ--EIA-------------------GDDWRDFDLIICDEAHRTTGATLTG
gi|881052313|ref|WP_048770555.1|damageinducibleprotein[Cellulomonasflavigena]/11705    YQSIDVIHRAQ--EIA-------------------GDDWRDFDLIICDEAHRTTGATLTG
gi|490108682|ref|WP_004009424.1|helicase[Mobiluncuscurtisii]_gi|315490465|gb|EFU80089.1|helicaseCterminaldomainprotein[Mobilun/    YQSIDIVHQAQ--ELA-------------------GKHWRDFDLIICDEAHRTTGVKLSD
gi|490111935|ref|WP_004012603.1|helicase[Mobiluncusmulieris]_gi|227843812|gb|EEJ53982.1|helicaseCterminaldomainprotein[Mobilun/    YQSIGVIHEAQ--VLA-------------------GEAWRDFDLVICDEAHRTTGAKLAN
gi|490114228|ref|WP_004014848.1|helicase[Mobiluncusmulieris]_gi|269934432|gb|EEZ90993.1|helicaseCterminaldomainprotein[Mobilun/    YQSIGVIHEAQ--EVA-------------------GDLWRDFDLVICDEAHRTTGAKLAN
gi|493333551|ref|WP_006290636.1|helicase[Parascardoviadenticolens]_gi|315119487|gb|EFT82620.1|helicaseCterminaldomainprotein[P/    YQSMQVIQDAQ--DLG----------------------LPEFDLAICDEAHRTTGIRTVD
gi|495817067|ref|WP_008541646.1|helicase,partial[Sutterellaparvirubra]_gi|378598583|gb|EHY31712.1|helicaseprotein,partial[Sutte    YQSIEVVSQAQ--KQY---------------------GMDEIGLIICDEAHRTAGGHHVD
gi|491572696|ref|WP_005430278.1|helicase[Sutterellawadsworthensis]_gi|319805116|gb|EFW01943.1|DNAhelicaserestrictionenzymeTypeI    YQSIEVVSEAQ--HKF---------------------GMEEIGLIICDEAHRTAGGHYQD
gi|748686321|ref|WP_039944445.1|hypotheticalprotein[Thermicanusaegyptius]/11573    YHSIDVIKEAQ--NNG----------------------FPEFDLIIADEAHRTTGVVKNG
gi|916875698|ref|WP_051482754.1|hypotheticalprotein[Thioalkalivibriosp.HK1]/11366    YQSLELIACAQ--DKG----------------------APEFDLILCDEAHRTTGVSASG
gi|821248604|gb|KKZ10960.1|hypotheticalproteinTE42_09075[CandidatusSynechococcusspongiarumSP3]/11598    YQSSPMIGAAQ--QEH---------------------GLPDFDLAVADEAHRTAGAFIPG
gi|737409843|ref|WP_035390956.1|hypotheticalprotein[Ferrimicrobiumacidiphilum]_gi|765544266|gb|KJE76241.1|typeIrestrictionenzym    YQSLRVIEEAQ--RDF---------------------GFPEFDLIICDEAHRTAGVATET
gi|654906925|ref|WP_028358281.1|damageinducibleprotein[Brackiellaoedipodis]/11679    YHSVDVIAQAQ--QQY---------------------GLGEFDLIICDEAHRTTGVTYKD
gi|502809901|ref|WP_013044877.1|hypotheticalprotein[CandidatusPuniceispirillummarinum]_gi|292663146|gb|ADE38247.1|hypotheticalp    YQSIDIIHQAQ--QMG----------------------APAFDLVICDEAHRTTGIDRAN
gi|251771370|gb|EES51950.1|typeIIIrestrictionenzyme,ressubunit[Leptospirillumferrodiazotrophum]/11628    YQSMEVIREAQ--GMG----------------------LPSFDFVLCDEAHRTTGIELSY
gi|498328480|ref|WP_010642636.1|DNAdamageinducibleprotein[Acidithiobacillusthiooxidans]/11627    YQSIDVVIKAQ--KIG---------------------NLPDFDLVICDEAHRTTGLTLKN
gi|647633864|ref|WP_025906698.1|damageinducibleprotein[Prochlorococcussp.scB245a_519B7]/11622    YQSIDVISKAQ--YDFN---------------------VDSFDLIICDEAHRTTGATFAG
gi|647619599|ref|WP_025893729.1|damageinducibleprotein[Prochlorococcussp.scB241_527N11]/11621    YQSIEVISKSQ--YEFE---------------------LDEFDLIICDEAHRTTGATLSG
gi|755034887|ref|WP_042387498.1|hypotheticalprotein[Clostridiumsp.CL6]/11606    YQSIDVVSELQ--KRS----------------------KIKFDIVVCDEAHRTTGVTLGD
gi|498139798|ref|WP_010453954.1|helicase[SuccinivibrionaceaebacteriumWG1]/11662    YQSIEVIIKAQ--AILLS-----------------KGSLGVFDLVVCDEAHRTTGVKLPN
gi|527501386|gb|EPT34571.1|typeIIIrestrictionenzyme,ressubunit[Bacteroidetesbacteriumoraltaxon272str.F0290]/11789    YQSIDVVAEAQ--RQILEET---------------NGTFGVFDMIVCDEAHRTTGAKSKY
gi|496047275|ref|WP_008771782.1|helicase[Bacteroidessp.2_1_33B]_gi|262296443|gb|EEY84373.1|helicaseCterminaldomainprotein[Bact/    YQSIDVLAEAQ--RALLE-A---------------DPSYGIFDYIVCDEAHRTTGFKQKG
PDI8503III/11664    YQSIDVLAEAQ--RALLE-A---------------DPSYGIFDYIVCDEAHRTTGFKQKG
gi|738719431|ref|WP_036613715.1|helicase[Parabacteroidesdistasonis]_gi|649542518|gb|KDS49387.1|restrictionendonucleasefamilypro    YQSIDVLAEAQ--RALLE-A---------------DPSYGIFDYIVCDEAHRTTGFKQKG
gi|659417033|gb|KEJ86133.1|hypotheticalproteinHMPREF1002_00341[Porphyromonassp.31_2]/11656    YQSIDVLAEAQ--RALLE-A---------------DPSYGIFDYIVCDEAHRTTGFKQKG
gi|567456807|gb|ETK11358.1|hypotheticalproteinT235_15845[Tannerellasp.oraltaxonBU063isolateCell8/11]/11433    YQSIDIVAEAQ--AAMLKET---------------KGEFGIFDFIVCDEAHRTTGVKLSS
```

```
gi|545440279|ref|WP_021677877.1|helicase[Porphyromonasgingivalis]_gi|543985765|gb|ERJ89108.1|helicaseprotein[Porphyromonasgingi      YQSIDVIAEAQ--TAILKET---------------KGTFGVFDFIVCDEAHRTTGVKVSS
gi|497438056|ref|WP_009752254.1|helicase[Prevotellasp.oraltaxon473]_gi|429144866|gb|EKX87968.1|helicaseprotein[Prevotellasp.ora      YQSIEVIATAQ--ALLLEQT---------------HGAFGTFDFVVCDEAHRTTGLKIAK
gi|490423474|ref|WP_004295683.1|MULTISPECIES:helicase[Bacteroides]_gi|156110098|gb|EDO11843.1|helicaseCterminaldomainprotein[B/      YQSIEAIHAAQ--HEILKET---------------AGTYGKFDLIVCDEAHRTTGVKLSD
gi|941777229|ref|WP_055173506.1|helicase[Bacteroidescaccae]_gi|933263395|emb|CUQ00567.1|Predictedhelicase[Bacteroidescaccae]/1/      YQSIDAVSAAQ--QEILSET---------------NGEYGVFDFIICDEAHRTTGVKIAD
gi|492413898|ref|WP_005836892.1|helicase[Bacteroidesuniformis]_gi|392678824|gb|EIY72229.1|hypotheticalproteinHMPREF1072_03583[B      YQSIDAISAAQ--QEILSET---------------NGEYGVFDFIICDEAHRTTGVKIAD
gi|695455095|ref|WP_032566191.1|helicase[Bacteroidesfragilis]_gi|596095426|gb|EXZ59996.1|restrictionendonucleasefamilyprotein[B      YQSIDAVSAAQ--REILSKT---------------NGEYGVFDFIICDEAHRTTGVKIAE
gi|933227941|emb|CUQ02348.1|Predictedhelicase[Bacteroidesuniformis]/11658                                                            YQSIDAVSAAQ--QEILSET---------------NGEFGVFDFIICDEAHRTTGVKIAE
gi|494823013|ref|WP_007558421.1|helicase[Bacteroidesplebeius]_gi|198272167|gb|EDY96436.1|helicaseCterminaldomainprotein[Bacter/      YQSIDAVSAAQ--QEILSET---------------NGEYGVFDFIICDEAHRTTGVKIAD
gi|495427212|ref|WP_008151909.1|helicase[Parabacteroidesjohnsonii]_gi|218222381|gb|EEC95031.1|helicaseCterminaldomainprotein[P/      YQSIDAVSAAQ--QEILSET---------------DGEYGVFDFIICDEAHRTTGVKLSD
gi|933196855|emb|CUO94545.1|typeIrestrictionenzymeEcoKIsubunitR[Bacteroidesuniformis]_gi|933220098|emb|CUN86874.1|typeIrestrict      YQSIDAVSEAQ--QEILSET---------------NGEYGVFDFIICDEAHRTTGVKLSD
gi|823687748|ref|WP_047101121.1|helicase[Brachyspirahyodysenteriae]_gi|823334537|gb|KLI17932.1|helicase[Brachyspirahyodysenteri      YQSITVISEAQ--KKI---------------------DNFEFDLIICDEAHRTTGVSLAE
gi|496436691|ref|WP_009145538.1|helicase[Phascolarctobacteriumsuccinatutens]_gi|322414007|gb|EFY04839.1|helicaseCterminaldomai/      YQSIDVIGKAQ--RFLRGEKVEDDSQQMLFDDFIPEEHDFTFDYIVCDEAHRTTGVIING
gi|503372880|ref|WP_013607541.1|helicase[Sphaerochaetaglobosa]_gi|324026933|gb|ADY13692.1|typeIIIrestrictionproteinressubunit[S      YQSIDAVKNAQ--NYLLSQ----------------G-KQAVFDLIVCDEAHRTTGVTLKD
gi|505821748|ref|WP_015707145.1|helicase[Treponemaprimitia]_gi|299483503|gb|ADJ19584.1|helicasedomaincontainingprotein[Trepone/      YQSIDVISQVQ--KSINKQ----------------KQGSFLFDLIICDEAHRTTGVTISG
gi|505822741|ref|WP_015708134.1|helicase[Treponemaprimitia]_gi|333739639|gb|AEF85129.1|endonucleaseandmethylaseLlaGI[Treponemap      YQSIDVISQVQ--KSINKQ----------------KQGSFLFDLIICDEAHRTTGVTISG
gi|488791994|ref|WP_002704400.1|helicase[Treponemasaccharophilum]_gi|380767691|gb|EIC01689.1|typeIIIrestrictionproteinressubuni      YQSIDVISKVQ--KAINLS----------------ENDSFIFDLIVCDEAHRTTGYTPNG
gi|524671454|emb|CDD81750.1|putativeseptumsitedeterminingproteinMinC[Bacteroidessp.CAG:462]/11642                                    YQSIEVISRAQ--QELLKRT---------------KGEYGIFDLIICDEAHRTTGISLKG
gi|524573865|emb|CDC95766.1|putativeseptumsitedeterminingproteinMinC[Alistipessp.CAG:268]/11639                                       YQSIDVISRAQ--QQLLKET---------------GDAFGTFDLIICDEAHRTTGVTLKD
gi|658617970|ref|WP_029759328.1|helicase[Fusobacteriumnucleatum]/11606                                                                YQSIDVIAKAQ--EKILAQ----------------DKNFGEFDLIICDEAHRTTGVTLKN
gi|494412037|ref|WP_007216782.1|helicase[Bacteroidescellulosilyticus]_gi|392638921|gb|EIY32752.1|hypotheticalproteinHMPREF1062_      YQSIGVVAEAQ--RQIGAE--------------------AEFDLIVCDEAHRTTGVTLKD
gi|383107102|gb|AFG37435.1|putativehelicase[SpirochaetaafricanaDSM8902]/11616                                                        YQSIDVIATAQ--KVYMSELG-------------QDKSNAIFDMVICDEAHRTTGVAITA
SAF8902III/11616                                                                                                                      YQSIDVIATAQ--KVYMSELG-------------QDKSNAIFDMVICDEAHRTTGVAITA
gi|652538894|ref|WP_026932796.1|helicase[Gramellaechinicola]/11633                                                                   YQSIDVIAAAQ--KELKQQ----------------HKINAEFDLIICDEAHRTTGTALTA
gi|639238223|ref|WP_024568839.1|helicase[Elizabethkingiaanophelis]/11628                                                             YQSIEVIATAQ--QNLLKE----------------GNENAVFDLIICDEAHRTTGVSLTK
gi|652520381|ref|WP_026914582.1|helicase[Gramellaportivictoriae]/11623                                                               YQSIEVIANAQ--RHLLNE----------------DNGNAVFDLIICDEAHRTTGVTLSG
gi|494922183|ref|WP_007648219.1|Helicase[Formosasp.AK20]_gi|469825489|gb|EMQ95550.1|Helicasedomainprotein[Formosasp.AK20]/11626      YQSIDVIANAQ--RHLLNE----------------NNNNAIFDLIICDEAHRTTGVTLAG
gi|522083382|ref|WP_020594591.1|hypotheticalprotein[Spirosomapanaciterrae]/11637                                                     YQSIDVIAKAQ--KALATT----------------LPKYAEFDLIICDEAHRTTGVSLAG
gi|490455352|ref|WP_004326157.1|helicase[Bacteroidesovatus]_gi|392669277|gb|EIY62768.1|hypotheticalproteinHMPREF1070_03635[Bact      YQSIEVIAKAQ--KAYKQE----------------IGDKAIFDLIVCDEAHRTTGVTLAK
gi|499516035|ref|WP_011202675.1|helicase[Bacteroidesfragilis]_gi|53713270|ref|YP_099262.1|helicasedomainprotein[Bacteroidesfrag      YQSIEVIAKAQ--KAYKQE----------------IGDKAIFDLIVCDEAHRTTGVTLAK
gi|651338332|ref|WP_026461320.1|helicase[Adhaeribacteraquaticus]/11620                                                               YQSIEVIAQAQ--KQLLQS----------------DIGFGVFDLIICDEAHRTTGVALAG
gi|806964642|gb|AKC17139.1|helicase[Flavobacteriumpsychrophilum]/11639                                                               YQSIEVIAKAQ--QQLEKQ----------------KSKFAQFDLIICDEAHRTTGVTIAG
gi|752684374|ref|WP_041332322.1|helicase[Flavobacteriumpsychrophilum]_gi|666882889|gb|AIG30715.1|helicase[Flavobacteriumpsychro      YQSIEVIAKAQ--QQLEKQ----------------KSKFAQFDLIICDEAHRTTGVTIAG
gi|933196595|emb|CUP04894.1|DNAorRNAhelicasesofsuperfamilyII[Bacteroidesuniformis]/11631                                             YQSIDVIARAQ--KELI------------------KKGFSEFDLIICDEAHRTTGVSLAG
gi|670509109|ref|WP_031456918.1|helicase[Flavobacteriumchungangense]/11625                                                           YQSIDVIAKAQ--NVIL------------------KNGFSEFDIIICDEAHRTTGLKIAG
                                                                                                                                      *:*                                      :   : **:**:..

gi|490190857|ref|WP_004089451.1|hypotheticalprotein[Helicobacterbilis]_gi|476630638|gb|EMZ37196.1|hypotheticalproteinC826_02193      KIK------------------GQDLQDTQKNAFILCHDDTHVNAKRRIYMTATPKIYSDR
gi|488896133|ref|WP_002807242.1|MULTISPECIES:restrictionendonucleaseEco57I[Campylobacter]_gi|380541096|gb|EIA65377.1|hypothetic      KEK------------------------DKLNTFTLCHSNENINAKKRIYMSATPKIYSSS
gi|746602240|ref|WP_039628365.1|restrictionendonucleaseEco57I[Campylobacterlari]_gi|744819395|gb|AJD03391.1|putativehelicase[Ca      KEK------------------------DKLNTFTLCHSDENIKAKKRIYMSATPKIYSSS
gi|727317135|ref|WP_033772558.1|restrictionendonucleaseEco57I[Helicobacterpylori]/11622                                              NER------------------------DDKNAFTLCHSDGNIKAKQRLYMTATPKVYSED
gi|727103490|ref|WP_033618954.1|restrictionendonucleaseEco57I[Helicobacterpylori]/11616                                              NER------------------------DDKNAFTLCHSDENIKAKQRLYMTATPKVYSED
gi|727340205|ref|WP_033794408.1|restrictionendonucleaseEco57I[Helicobacterpylori]/11609                                              NER------------------------DEKNAFTLCHSDGNIKAKQRLYMTATPKVYSED
gi|727292407|ref|WP_033749975.1|restrictionendonucleaseEco57I[Helicobacterpylori]/11611                                              NER------------------------DDKNAFTLCHSDENIKAKQRLYMTATPKVYSED
gi|727289795|ref|WP_033747591.1|restrictionendonucleaseEco57I[Helicobacterpylori]/11612                                              NER------------------------DDKNAFTLCHSDENIKAKQRLYMTATPKVYSES
gi|727319676|ref|WP_033775047.1|restrictionendonucleaseEco57I[Helicobacterpylori]/11612                                              NER------------------------DDKNAFTLCHSDENIKAKQRLYMTATPKVYSED
gi|447212910|ref|WP_001290166.1|restrictionendonucleaseEco57I[Helicobacterpylori]_gi|308063540|gb|ADO05427.1|hypotheticalprotei      NER------------------------DDKNAFTLCHSDEHIKAKKRLYMTATPKVYSES
gi|447212901|ref|WP_001290157.1|restrictionendonucleaseEco57I[Helicobacterpylori]_gi|384557329|gb|AFH97797.1|hypotheticalprotei      NER------------------------DDKNAFTLCHSDEHIKAKKRLYMTATPKVYSES
gi|515849671|ref|WP_017280424.1|hypotheticalprotein[Helicobacterpylori]/11615                                                        NER------------------------DDKNAFTLCHSDGNIKAKKRLYMTATPKVYSES
gi|447212900|ref|WP_001290156.1|restrictionendonucleaseEco57I[Helicobacterpylori]_gi|317178970|dbj|BAJ56758.1|TypeIIGrestrictio      NER------------------------DDKNAFTLCHSDGNIKAKKRLYMTATPKVYSES
gi|447079018|ref|WP_001156274.1|hypotheticalprotein[Helicobacterpylori]/11614                                                        NER------------------------DDKNAFTLCHSDENIKAKKRLYMTATPKVYSES
gi|447212800|ref|WP_001290056.1|hypotheticalprotein[Helicobacterpylori]_gi|532153248|gb|EQL67316.1|restrictionendonucleaseEco57      NER------------------------DDKNAFTLCHSDEHIKAKKRLYMTATPKVYSES
gi|505457237|ref|WP_015644339.1|hypotheticalprotein[Helicobacterpylori]_gi|499061160|gb|AGL70074.1|restrictionendonucleaseEco57      NER------------------------DDKNAFTLCHSDEHIKAKKRLYMTATPKVYSES
HPYUM037X/11591                                                                                                                       NER------------------------DDKNAFTLCHSDEHIKAKKRLYMTATPKVYSES
gi|920026297|ref|WP_052937176.1|restrictionendonuclease[Helicobacterpylori]_gi|912528678|gb|KNX48238.1|restrictionendonucleaseE      NER------------------------DDKNAFTLCHSDANIKAKKRLYMTATPKVYSES
gi|915539050|ref|WP_050841327.1|restrictionendonuclease[Helicobacterpylori]_gi|908890887|gb|KNE06183.1|restrictionendonucleaseE      NER------------------------DDKNAFTLCHSDEHIKAKKRLYMTATPKVYSES
gi|727101397|ref|WP_033616957.1|restrictionendonucleaseEco57I[Helicobacterpylori]/11592                                              NER------------------------DDKNAFTLCHSDGHIKAKKRLYMTATPKVYSES
gi|562814875|emb|CDK13002.1|TypeISPRMsystem[Helicobacterpylori26695]/11588                                                           NER------------------------DDKNAFTLCHSDKNIKAKKRLYMTATPKVYSES
HPYAXVIII/11588                                                                                                                       NER------------------------DDKNAFTLCHSDKNIKAKKRLYMTATPKVYSES
gi|447079029|ref|WP_001156285.1|restrictionendonucleaseEco57I[Helicobacterpylori]_gi|393085956|gb|EJB86635.1|hypotheticalprotei      NER------------------------DDKNAFTLCHSDKNIKAKKRLYMTATPKVYSES
gi|489105953|ref|WP_003015814.1|restrictionendonuclease[Helicobacterpylori]_gi|477503388|gb|ENH59950.1|restrictionendonucleasef      NER------------------------DDKNAFTLCHSDENIKAKKRLYMTATPKVYSES
gi|447079006|ref|WP_001156262.1|restrictionendonucleaseEco57I[Helicobacterpylori]_gi|393067470|gb|EJB68280.1|hypotheticalprotei      NER------------------------DDKNAFTLCHSDENIKAKKRLYMTATPKVYSES
gi|727314589|ref|WP_033770298.1|restrictionendonucleaseEco57I[Helicobacterpylori]/11586                                              NER------------------------DDKNAFTLCHSDENIKAKQRLYMTATPKVYSES
gi|447079019|ref|WP_001156275.1|restrictionendonucleaseEco57I[Helicobacterpylori]_gi|308065028|gb|ADO06920.1|hypotheticalprotei      NER------------------------DDKNAFTLCHSDENIKATKRLYMTATPKVYSES
gi|447079028|ref|WP_001156284.1|restrictionendonucleaseEco57I[Helicobacterpylori]_gi|393076030|gb|EJB76784.1|hypotheticalprotei      NER------------------------DDKNAFTLCHSDENIKATKRLYMTATPKVYSEN
gi|393034009|gb|EJB35070.1|hypotheticalproteinHPNQ4053_0631[HelicobacterpyloriNQ4053]/11514                                          NER------------------------DDKNAFTLCHSDENIKAKKRLYMTATPKVYSES
gi|447079004|ref|WP_001156260.1|restrictionendonucleaseEco57I[Helicobacterpylori]_gi|384552418|gb|AFI07366.1|hypotheticalprotei      NER------------------------DDKNAFTLCHSDENIKAKKRLYMTATPKIYSES
gi|746011306|ref|WP_039088215.1|restrictionendonucleaseEco57I[Helicobacterpylori]_gi|733488720|gb|KHL85979.1|restrictionendonuc      NER------------------------DDKNAFTLCHSDENIKAKKRLYMTATPKVYSES
```

```
gi|531081162|gb|EQD92166.1|restrictionendonucleaseEco57I[HelicobacterpyloriPZ5080]/11509    NER------------------------DDKNAFTLCHSDENIKATKRLYMTATPKVYSES
gi|544760796|ref|WP_021187395.1|restrictionendonucleaseEco57I[Helicobacterpylori]_gi|531086508|gb|EQD96745.1|restrictionendonuc    NER------------------------DDKNAFTLCHSDENIKATKRLYMTATPKVYSES
gi|447079012|ref|WP_001156268.1|restrictionendonucleaseEco57I[Helicobacterpylori]_gi|393028199|gb|EJB29286.1|hypotheticalprotei    NER------------------------DDKNAFTLCHSDKNIKATKRLYMTATPKVYSES
gi|746008458|ref|WP_039085555.1|restrictionendonucleaseEco57I[Helicobacterpylori]_gi|733480358|gb|KHL78026.1|restrictionendonuc    NER------------------------DDKNAFTLCHSDENIKATKRLYMTATPKVYSES
gi|447079011|ref|WP_001156267.1|restrictionendonucleaseEco57I[Helicobacterpylori]_gi|393038534|gb|EJB39568.1|hypotheticalprotei    NER------------------------DDKNAFTLCHSDENIKATKRLYMTATPKVYSES
gi|393037163|gb|EJB38199.1|hypotheticalproteinHPNQ4161_0422[HelicobacterpyloriNQ4161]/11556    NER------------------------DDKNAFTLCHSDENIKATKRLYMTATPKIYSES
gi|447079007|ref|WP_001156263.1|restrictionendonucleaseEco57I[Helicobacterpylori]_gi|393051817|gb|EJB52768.1|hypotheticalprotei    NER------------------------DDKNAFTLCHSDENIKAKQRLYMTATPKVYSES
gi|487867094|ref|WP_001940560.1|helicase[Helicobacterpylori]_gi|459505657|gb|EMG98418.1|helicaseprotein[HelicobacterpyloriGAM21    NER------------------------DDKNAFTLCHSDGNIKATKRLYMTATPKVYSES
gi|487872094|ref|WP_001945560.1|helicase[Helicobacterpylori]_gi|459512104|gb|EMH04523.1|helicaseprotein[HelicobacterpyloriGAM24    NER------------------------DDKNAFTLCHSDENIKATKRLYMTATPKVYSES
gi|487880228|ref|WP_001953694.1|helicase[Helicobacterpylori]_gi|459529131|gb|EMH20734.1|helicaseprotein[HelicobacterpyloriGAM26    NER------------------------DDKNAFTLCHSDENIKATKRLYMTATPKVYSES
gi|487882593|ref|WP_001956059.1|helicase[Helicobacterpylori]_gi|459533462|gb|EMH24859.1|helicaseprotein[HelicobacterpyloriGAM26    NER------------------------DDKNAFTLCHSDENIKATKRLYMTATPKVYSES
gi|487845430|ref|WP_001918896.1|helicase[Helicobacterpylori]_gi|410713871|gb|EKQ71363.1|helicaseprotein[HelicobacterpyloriGAM10    NER------------------------DDKNAFTLCHSDGNIKATKRLYMTATPKVYSES
gi|447079005|ref|WP_001156261.1|restrictionendonucleaseEco57I[Helicobacterpylori]_gi|393069830|gb|EJB70625.1|hypotheticalprotei    NER------------------------DDKNAFTLCHSDGNIKAKKRLYMTATPKVYSES
gi|459506808|gb|EMG99528.1|helicaseprotein[HelicobacterpyloriGAM121Aii]/11545    NER------------------------DDKNAFTLCHSDENIKATKRLYMTATPKVYSES
gi|487825283|ref|WP_001898749.1|restrictionendonucleaseEco57I[Helicobacterpylori]_gi|393107526|gb|EJC08068.1|hypotheticalprotei    NER------------------------DDKNAFTLCHSDENIKATKRLYMTATPKVYSES
gi|447079008|ref|WP_001156264.1|restrictionendonucleaseEco57I[Helicobacterpylori]_gi|393058636|gb|EJB59524.1|hypotheticalprotei    NER------------------------DDKNAFTLCHSDENIKATKRLYMTATPKVYSES
gi|447079015|ref|WP_001156271.1|restrictionendonucleaseEco57I[Helicobacterpylori]_gi|307637357|gb|ADN79807.1|hypotheticalprotei    NER------------------------DDKNAFTLCHSDENIKAKKRLYMTATPKVYSES
gi|487884861|ref|WP_001958327.1|helicase[Helicobacterpylori]_gi|459535535|gb|EMH26834.1|helicaseprotein[HelicobacterpyloriGAM26    NER------------------------DDKNAFTLCHSDENIKATKRLYMTATPKVYSES
gi|487886365|ref|WP_001959831.1|helicase[Helicobacterpylori]_gi|459538727|gb|EMH29861.1|helicaseprotein[HelicobacterpyloriGAM27    NER------------------------DDKNAFTLCHSDENIKATKRLYMTATPKVYSES
gi|545301637|ref|WP_021581412.1|helicase[Helicobacterpylori]_gi|535931889|gb|ERA61745.1|helicaseprotein[HelicobacterpyloriGAM11    NER------------------------DDKNAFTLCHSDENIKAKKRLYMTATPKVYSES
gi|447079010|ref|WP_001156266.1|restrictionendonucleaseEco57I[Helicobacterpylori]_gi|393095477|gb|EJB96081.1|hypotheticalprotei    NER------------------------DDKNAFTLCHSDENIKATKRLYMTATPKVYSES
gi|447079009|ref|WP_001156265.1|restrictionendonucleaseEco57I[Helicobacterpylori]_gi|393056294|gb|EJB57206.1|hypotheticalprotei    NER------------------------DDKNAFTLCHSDENIKATKRLYMTATPKVYSES
gi|471386985|gb|EMR58906.1|restrictionendonucleasefamilyprotein[HelicobacterpyloriHpH1]/11504    NER------------------------DDKNAFTLCHSDENIKAKKRLYMTATPKVYSES
gi|487875679|ref|WP_001949145.1|helicase[Helicobacterpylori]_gi|459516928|gb|EMH09091.1|helicaseprotein[HelicobacterpyloriGAM25    NER------------------------DDKNAFTLCHSDGNIKAKKRLYMTATPKVYSES
gi|496130066|ref|WP_008854573.1|DNAdamageinducibleprotein[Commensalibacterintestini]_gi|353671689|gb|EHD13391.1|helicasedomain/    E---------------------------EESAFVRVHDNGFIKGNKRLYMTATPRIYAED
gi|924898894|ref|WP_053556546.1|damageinducibleprotein[Sphingopyxissp.113P3]_gi|924523996|gb|ALC14732.1|damageinducibleprotei/1    E---------------------------DESNFVRVHDATYIRAAKRLYMTATPRVYGEA
gi|780802950|gb|KJS08562.1|damageinducibleprotein[Hoefleasp.BRH_c9]/11633    E---------------------------EESNFIKVHSQDFIKGNHRLYMTATPRIYGDG
gi|944671432|gb|KQN22886.1|damageinducibleprotein[Sphingomonassp.Leaf32]/11415    E---------------------------DESNFVRIHNQDFIAGRKRLYMTATPRIFGDA
gi|944612953|gb|KQM64772.1|damageinducibleprotein[Sphingomonassp.Leaf16]/11726    E---------------------------DESNFVRIHNQDFIAGRKRLYMTATPRIFGDA
gi|764353851|ref|WP_044331468.1|damageinducibleprotein[Sphingomonashengshuiensis]_gi|761897338|gb|AJP71707.1|damageinduciblep/1    E---------------------------DESNFVKIHNQDFIAGKKRLYMTATPRVFGDA
gi|945874472|gb|KQZ27973.1|damageinducibleprotein[Mesorhizobiumsp.Root552]/11651    E---------------------------DESNFVKIHNQQFIAGRKRLYMTATPRVFGDA
gi|759408081|ref|WP_043132403.1|damageinducibleprotein[Paracoccussanguinis]_gi|694217086|gb|KGJ14281.1|damageinducibleprotein/1    E---------------------------DDSAFVRIHSNDHVAAKKRLYMTATPRIFGDA
gi|654342671|ref|WP_027836116.1|damageinducibleprotein[Maritaleamyrionectae]/11632    Q---------------------------DDSNFVRIHSNDHVAAKKRLYMTATPRIFGDA
gi|126717687|gb|EBA14409.1|hypotheticalproteinRSK20926_02032[Roseobactersp.SK20926]/11603    E---------------------------DDSNFVRIHDNEFVAAKKRLYMTATPRIFADT
gi|765301116|ref|WP_044628780.1|damageinducibleprotein[Halomonasmeridiana]_gi|764089679|gb|KJD19875.1|damageinducibleprotein[/1    E---------------------------DDSNFVRIHNNQYVNAKKRLYMTATPRIFGDA
gi|518053006|ref|WP_019223214.1|DNAdamageinducibleprotein[Bartonellarattaustraliani]/11654    DK--------------------------SESEFIKVHDNSMIQGKKRLYMTATPKIFTNN
gi|493637806|ref|WP_006589578.1|DNAdamageinducibleprotein[Bartonellabirtlesii]_gi|395410345|gb|EJF76900.1|hypotheticalproteinM/    ED--------------------------NESEFIKVHDNSIIRGKKRLYMTATPRIFSDT
gi|501188603|ref|WP_012231622.1|DNAdamageinducibleprotein[Bartonellatribocorum]_gi|161017843|emb|CAK01401.1|helicase/methyltra/    ED--------------------------NESEFIKVHDNSIIRGKKRLYMTATPRIFSDT
gi|651388616|ref|WP_026500615.1|damageinducibleprotein[Bartonellagrahamii]/11655    ED--------------------------NESEFIKVHDNSIIRGKKRLYMTATPRIFSDT
gi|501188669|ref|WP_012231687.1|DNAdamageinducibleprotein[Bartonellatribocorum]_gi|161017922|emb|CAK01480.1|helicase/methyltra/    ED--------------------------NESEFIKVHDNSIIRGKKRLYMTATPRIFSDT
gi|518048832|ref|WP_019219040.1|DNAdamageinducibleprotein[Bartonellaflorencae]/11655    DK--------------------------YESEFIKVHDNSIIRGKKRLYMTATPKIFTDK
gi|518053617|ref|WP_019223825.1|DNAdamageinducibleprotein[Bartonellarattaustraliani]/11607    DD--------------------------SESEFVKVHDNSIIRGKKRLYMTATPKIFTDK
gi|506336449|ref|WP_015856168.1|DNAdamageinducibleprotein[Bartonellagrahamii]_gi|240267492|gb|ACS51080.1|helicase/methyltransf/    ED--------------------------NESEFIKVHDNSIIRGKKRLYMTATPKIFTDK
gi|494588832|ref|WP_007347427.1|DNAdamageinducibleprotein[Bartonellarattimassiliensis]_gi|395419053|gb|EJF85361.1|hypothetical/    DK--------------------------RESEFIKVHDNSIIRGKKRLYMTATPKIFTDK
gi|501188650|ref|WP_012231668.1|DNAdamageinducibleprotein[Bartonellatribocorum]_gi|161017902|emb|CAK01460.1|helicase/methyltra/    DK--------------------------RESEFIKVHDNSIIFGKKRLYMTATPKIFTDK
gi|501188624|ref|WP_012231643.1|DNAdamageinducibleprotein[Bartonellatribocorum]_gi|161017866|emb|CAK01424.1|helicase/methyltra/    ED--------------------------NESDFIKVHDNSLIRGKKRLYMTATPRIFSNH
gi|518053856|ref|WP_019224064.1|DNAdamageinducibleprotein[Bartonellarattaustraliani]/11625    ED--------------------------SESDFIKVHDNSLIRGKKRLYMTATPRIFSDH
gi|501188053|ref|WP_012231081.1|DNAdamageinducibleprotein[Bartonellatribocorum]_gi|161017434|emb|CAK00992.1|helicase/methyltra/    ED--------------------------NESDFIKVHDNTLIRGKKRLYMTATPRIFSNH
gi|651388630|ref|WP_026500626.1|damageinducibleprotein[Bartonellagrahamii]/11657    DK--------------------------CESEFIKVHDNSIIRGKKRLYMTATPKIFSDK
gi|518048855|ref|WP_019219063.1|DNAdamageinducibleprotein[Bartonellaflorencae]/11658    DK--------------------------HESEFIKVHDNSIIRGKKRLYMTATPKIFSDK
gi|490995101|ref|WP_004856829.1|DNAdamageinducibleprotein[Bartonelladoshiae]_gi|395413167|gb|EJF79645.1|hypotheticalproteinMCS/    DK--------------------------HESEFIKVHDNNIIRGKKRLYMTATPKIFSDK
gi|491002558|ref|WP_004864279.1|DNAdamageinducibleprotein[Bartonellavinsonii]_gi|395421392|gb|EJF87643.1|hypotheticalproteinME/    DN--------------------------SESKFVKIHDNSIIQGKKRLYMTATPKIFADD
gi|491003373|ref|WP_004865094.1|DNAdamageinducibleprotein[Bartonellavinsonii]_gi|395432864|gb|EJF98838.1|hypotheticalproteinME/    DN--------------------------SESKFVKVHDNSIVQGKKRLYMTATPKIFADD
gi|491002426|ref|WP_004864148.1|DNAdamageinducibleprotein[Bartonellavinsonii]_gi|395421767|gb|EJF88003.1|hypotheticalproteinME/    DN--------------------------SESEFVKVHDNSIIQGKKRLYMTATPKIFTDN
gi|491004024|ref|WP_004865745.1|DNAdamageinducibleprotein[Bartonellavinsonii]_gi|395432542|gb|EJF98522.1|hypotheticalproteinME/    DN--------------------------SESEFVKVHDNSIIQGKKRLYMTATPKIFTDN
gi|648396442|ref|WP_026088193.1|damageinducibleprotein[Bartonellarattaustraliani]/11593    DK--------------------------SESEFIKVHGNSIIRGKKRLYMTATPKIFTNN
gi|748190174|ref|WP_039761290.1|damageinducibleprotein[Bartonellaqueenslandensis]/11658    DK--------------------------SESEFIKVHDNSMIQGKKRLYMTATPKIFTNN
gi|494817599|ref|WP_007553007.1|DNAdamageinducibleprotein[Bartonellasp.DB56]_gi|395411312|gb|EJF77837.1|hypotheticalproteinMC/1    DK--------------------------SESEFIKVHDNSLIRGKKRLYMTATPRIFSDN
gi|515763376|ref|WP_017195976.1|DNAdamageinducibleprotein[Bartonellabirtlesii]/11656    DK--------------------------SESEFIKVHDNSIIRGKKRLYMTATPRIFSDN
gi|494589420|ref|WP_007348014.1|DNAdamageinducibleprotein[Bartonellarattimassiliensis]_gi|395416278|gb|EJF82667.1|hypothetical/    DN--------------------------NESDFIKVHDNSIIQGKKRLYMTATPKIFTDD
gi|506336469|ref|WP_015856188.1|DNAdamageinducibleprotein[Bartonellagrahamii]_gi|240267512|gb|ACS51100.1|helicase/methyltransf/    ED--------------------------NESDFIKVHDNSIIQGKKRLYMTATPKIFTDD
gi|749308087|ref|WP_040132227.1|damageinducibleprotein[Bartonellatribocorum]_gi|605051878|emb|CDO49988.1|helicase/methyltransf/    ED--------------------------NESDFIKVHDNSIIQGKKRLYMTATPKIFTDD
gi|515763800|ref|WP_017196400.1|DNAdamageinducibleprotein[Bartonellabirtlesii]/11658    DK--------------------------HESEFIKVHDNSIIRGKKRLYMTATPKIFADH
gi|492186994|ref|WP_005773380.1|DNAdamageinducibleprotein[Bartonellaelizabethae]_gi|395418361|gb|EJF84688.1|hypotheticalprotei/    DK--------------------------HESEFIKVHDNSIIQGKKRLYMTATPKIFADH
gi|501188615|ref|WP_012231634.1|DNAdamageinducibleprotein[Bartonellatribocorum]_gi|161017856|emb|CAK01414.1|helicase/methyltra/    DK--------------------------HESEFIKVHDNSIIRGKKRLYMTATPKIFADS
gi|501187470|ref|WP_012230499.1|DNAdamageinducibleprotein[Bartonellatribocorum]_gi|161017090|emb|CAK00648.1|helicase/methyltra/    DK--------------------------HESEFIKVHDNSIIRGKKRLYMTATPKIFADS
gi|501188636|ref|WP_012231655.1|DNAdamageinducibleprotein[Bartonellatribocorum]_gi|161017879|emb|CAK01437.1|helicase/methyltra/    DK--------------------------HESEFIKVHDNSIIRGKKRLYMTATPKIFADS
```

```
gi|930151941|emb|CTQ34818.1|UvsWhelicase[Jannaschiarubra]/11641    E---------------------------DESNFVRIHSDEHVRAAKRLYMTATPRIYGEA
gi|928977106|ref|WP_054001461.1|damageinducibleprotein[Shimiasp.SK013]_gi|927698852|gb|KPA22698.1|typeIrestrictionenzymeEcoKIs/    E---------------------------DESNFVRVHDNKYVAGAKRLYMTATPRIFGDN
gi|738872465|ref|WP_036760884.1|damageinducibleprotein[Leisingeradaeponensis]/11643    Q---------------------------PESNFVRVHNNETIQAKKRLYMTATPRIFGDG
gi|518050881|ref|WP_019221089.1|DNAdamageinducibleprotein[Bartonellasenegalensis]/11652    E---------------------------GESNFVKVHFNDIIRAKKRLYMTATPRIFGDN
gi|493980596|ref|WP_006923523.1|DNAdamageinducibleprotein[Bartonellawashoensis]_gi|395413322|gb|EJF79796.1|hypotheticalprotein/    E---------------------------DESNFVKVHSNDVIRAKKRLYMTATPRIFGDN
gi|493983106|ref|WP_006925984.1|DNAdamageinducibleprotein[Bartonellawashoensis]_gi|395418127|gb|EJF84455.1|hypotheticalprotein/    E---------------------------DESNFVKVHSNDVIRAKKRLYMTATPRIFGDN
gi|499494671|ref|WP_011181311.1|DNAdamageinducibleprotein[Bartonellahenselae]_gi|49239008|emb|CAF28308.1|hypotheticalproteinBH/    E---------------------------DESNFVKVHSNDVIRAKKRLYMTATPRIFGDN
gi|651388426|ref|WP_026500441.1|damageinducibleprotein[Bartonellagrahamii]/11652    E---------------------------DESNFVKVHSNDVIRAKKRLYMTATPRIFGDN
gi|506337280|ref|WP_015856999.1|DNAdamageinducibleprotein[Bartonellagrahamii]_gi|240268402|gb|ACS51990.1|helicase/methyltransf/    E---------------------------DESNFVKVHSNDVIRAKKRLYMTATPRIFGDN
gi|494817535|ref|WP_007552943.1|DNAdamageinducibleprotein[Bartonellasp.DB56]_gi|395412002|gb|EJF78518.1|hypotheticalproteinMC/1    E---------------------------DESNFVKVHSNDVIRAKKRLYMTATPRIFGDN
gi|493638099|ref|WP_006589864.1|DNAdamageinducibleprotein[Bartonellabirtlesii]_gi|395409809|gb|EJF76394.1|hypotheticalproteinM/    E---------------------------DESNFVKVHSNDVICAKKRLYMTATPRIFGDN
gi|748189589|ref|WP_039760707.1|damageinducibleprotein[Bartonellaqueenslandensis]/11651    E---------------------------DESNFVKVHSNDVIRAKKRLYMTATPRVFGDN
gi|501189483|ref|WP_012232501.1|DNAdamageinducibleprotein[Bartonellatribocorum]_gi|161018902|emb|CAK02460.1|helicase/methyltra/    A---------------------------DESNFVKVHSNDIIRAKKRLYMTATPRIFGDN
gi|518049181|ref|WP_019219389.1|DNAdamageinducibleprotein[Bartonellaflorencae]/11652    E---------------------------DESNFVKVHSNDVIRAKKRLYMTATPRIFGDN
gi|492193255|ref|WP_005774528.1|DNAdamageinducibleprotein[Bartonellaelizabethae]_gi|395416403|gb|EJF82779.1|hypotheticalprotei/    E---------------------------DESNFVKVHSNDAIRAKKRLYMTATPRIFGDN
gi|498387325|ref|WP_010701288.1|helicase/methyltransferase[Bartonellabovis]_gi|478418724|gb|ENN92426.1|helicase/methyltransfera    E---------------------------NESNFVKVHFNECIRAKKRLYMTATPRIFGDN
gi|319408697|emb|CBI82352.1|conservedhypotheticalprotein[BartonellaschoenbuchensisR1]/11661    K---------------------------SESNFIKVHSNEFIRAKKRLYMTATPRIFGDN
gi|494741726|ref|WP_007477134.1|DNAdamageinducibleprotein[Bartonellamelophagi]_gi|395423925|gb|EJF90113.1|hypotheticalproteinM/    A---------------------------NESNFVKVHFNEFIRAKKRLYMTATPRIFGDN
gi|737261405|ref|WP_035245349.1|damageinducibleprotein[Alcanivoraxjadensis]_gi|686989709|gb|KGD62556.1|hypotheticalproteinT9A_/    E---------------------------DESNFVRIHDNGHVMAKKRLYMTATPRVFGDN
gi|916687564|ref|WP_051294655.1|damageinducibleprotein[Gemmobacternectariphilus]/11641    E---------------------------DESNFVKVHDNAVIKARKRLYMTATPRIFGDI
gi|740294127|ref|WP_038132495.1|damageinducibleprotein[Thioclavasp.DT234]_gi|660524826|gb|KEO54689.1|hypotheticalproteinDT23_/1    E---------------------------EDSNFVRIHDDAYVAGRKRLYMTATPRIFGDA
gi|568331062|ref|WP_024099615.1|helicase[Phaeobactergallaeciensis]_gi|568232121|gb|AHD12007.1|putativehelicase[Phaeobactergalla    E---------------------------DESNFVKVHDNDVIQGNKRLYMTATPRIFGDN
gi|518810459|ref|WP_019966413.1|DNAdamageinducibleprotein[Pannonibacterphragmitetus]/11635    E---------------------------EESNFVKIHSNDHVKGRKRLYMTATPRIFGDN
gi|495305760|ref|WP_008030510.1|DNAdamageinducibleprotein[Rhodobactersp.SW2]_gi|259021698|gb|EEW25000.1|typeIIIrestrictionprot/    D---------------------------EESNFVKIHSNDNVKARKRLYMTATPRIFGDN
gi|506403303|ref|WP_015923022.1|DNAdamageinducibleprotein[Rhodobactersphaeroides]_gi|221162951|gb|ACM03922.1|HypotheticalProte/    E---------------------------DESNFVKIHSDDHVAGRKRLYMTATPRIFGDN
gi|738818386|ref|WP_036708790.1|damageinducibleprotein[Paracoccussanguinis]_gi|694226786|gb|KGJ22541.1|damageinducibleprotein/1    E---------------------------DESNFVKIHSDEHVAGRKRLYMTATPRIFGDN
gi|746096057|ref|WP_039162453.1|damageinducibleprotein[Gallibacteriumanatis]_gi|703617775|gb|KGQ63868.1|damageinducibleprotei/1    DE--------------------------NESAFVRIHNNDYINAHKRLYMTATPRIYTED
gi|737716785|ref|WP_035685631.1|damageinducibleprotein[Avibacteriumparagallinarum]/11643    DE--------------------------NESAFVRIHNNDYINAHKRLYMTATPRIYTED
gi|540072553|gb|ERF78672.1|hypotheticalproteinN561_04930[Gallibacteriumanatis12656/12}/11651    DE--------------------------NESAFVRIHHNDYINAHKRLYMTATPRIYTED
gi|917509519|ref|WP_052115936.1|damageinducibleprotein[Gallibacteriumanatis]_gi|702414161|gb|KGQ42233.1|damageinducibleprotei/1    DE--------------------------NESAFVRIHHNDYINAHKRLYMTATPRIYTED
gi|746082942|ref|WP_039150268.1|damageinducibleprotein[Gallibacteriumanatis]_gi|703617078|gb|KGQ63194.1|damageinducibleprotei/1    DE--------------------------NESAFVRIHNNDYINAHKRLYMTATPRIYTED
gi|822522885|ref|WP_046952840.1|damageinducibleprotein[Haemophilushaemolyticus]_gi|821501762|gb|KKZ59267.1|damageinduciblepro/1    K---------------------------EESAFIRVHNNDYIKAQKRLYMTATPRIYTED
gi|404381014|gb|EJZ77501.1|putativehelicase[Pasteurellamultocidasubsp.gallicidaP1059]/11681    NE--------------------------QESAFVRIHHNNYIQAKKRLYMTATPRIYTED
gi|298281660|gb|EFI23149.1|helicaseCterminaldomainprotein[Neisseriasp.oraltaxon014str.F0314]/11493    D---------------------------DESAFVRVHDAGYIKGAKRLYMTATPRIYVDD
gi|348013055|gb|EGY51980.1|helicasedomainprotein[Neisseriashayeganii871]/11481    D---------------------------DESAFVRIHDADYIKGAKRLYMTATPRIFGEA
gi|496407140|ref|WP_009116004.1|DNAdamageinducibleprotein[Neisseriawadsworthii]_gi|349794756|gb|EGZ48566.1|helicasedomainprote/    D---------------------------DESAFVRIHDADYIKGAKRLYMTATPRIYGET
gi|517216441|ref|WP_018405259.1|DNAdamageinducibleprotein[Marinobacterlipolyticus]/11656    E---------------------------EESAFVRVHDNDYIQSAKRVYMTATPRIYGED
gi|738230264|ref|WP_036185691.1|damageinducibleprotein[Marinobactersp.ELB17]/11671    E---------------------------IESAFVKIHYNSHIQGAKRIYMTATPRIYGED
gi|447197741|ref|WP_001274997.1|DNAdamageinducibleprotein[Acinetobacterbaumannii]_gi|408512324|gb|EKK13969.1|typeIIIrestrictio/    D---------------------------DESKFVRIHNNDYVQGKKRLYMTATPRIYGDA
gi|820775897|ref|WP_046739909.1|damageinducibleprotein[Acinetobactersp.AG1]_gi|819909758|gb|KKW75938.1|damageinducibleprotein/1    D---------------------------DESKFVRIHNNDYVQGKKRLYMTATPRIYGED
gi|584595163|ref|WP_024160846.1|helicase[Acinetobacterpittii]_gi|576702530|emb|CDI28049.1|helicasedomaincontainingprotein[Acin/    D---------------------------DESKFVRIHNNDYVQGKKRLYMTATPRIYGET
gi|514963042|ref|WP_016651366.1|hypotheticalprotein[Acinetobactersp.NIPH2036]_gi|514162665|gb|EPG41391.1|hypotheticalproteinF90    D---------------------------DESKFVRIHNNDYVQGKKRLYMTATPRIYGDT
gi|515965564|ref|WP_017396147.1|DNAdamageinducibleprotein[Acinetobacterhaemolyticus]/11667    D---------------------------DESKFVRIHNNDYVQGKKRLYMTATPRIYGDT
gi|491346810|ref|WP_005204747.1|MULTISPECIES:hypotheticalprotein[Acinetobacter]_gi|480335823|gb|ENX56033.1|hypotheticalproteinF    D---------------------------DESKFVRIHDNSYVQGKKRLYMTATPRIYGDT
gi|693044472|ref|WP_032215268.1|damageinducibleprotein[Escherichiacoli]_gi|607725731|gb|EZE08230.1|damageinducibleprotein[Esc/1    D---------------------------DESAFVRIHDNAYINGRKRLYMTATPRIFGDD
gi|742382177|ref|WP_038861304.1|damageinducibleprotein[Cronobactermuytjensii]_gi|924333747|gb|ALB69766.1|damageinducibleprote/1    D---------------------------DESAFVRIHDNAYVAGKKRLYMTATPRIFGED
gi|915912804|ref|WP_050927035.1|damageinducibleprotein[Yersiniaenterocolitica]_gi|899805802|emb|CQH32042.1|typeIrestrictionenz/    D---------------------------DESAFVRIHDNAYIAGKKRLYMTATPRIYSDD
gi|921111327|gb|KON62781.1|typeIrestrictionenzymeEcoKIsubunitR[Komagataeibactereuropaeus]/11492    EE--------------------------NDSAFVRVHNQDYLAGRHRLYMTATPRIYGDV
gi|582024278|gb|AHI27615.1|DNAhelicaserestrictionenzymeTypeIIIRsubunit[KomagataeibacterxylinusE25]/11636    EE--------------------------NDSAFVRVHNQDYLAGRHRLYMTATPSIYGDV
gi|347662673|dbj|BAK86128.1|DNAhelicaserestrictionenzymeTypeIIIRsubunit[KomagataeibactermedellinensisNBRC3288]/11737    EE--------------------------NDSAFVRVHNQDYLAGSHRLYMTATPRIYGDV
gi|917445053|ref|WP_052051471.1|damageinducibleprotein[Acetobactertropicalis]/11429    EE--------------------------NDSAFVRVHNQDYLHGQRRLYMTATPRIYGDV
gi|453329445|dbj|GAC88472.1|DNAhelicaserestrictionenzymetypeIIIRsubunit[GluconobacterthailandicusNBRC3255]_gi|530287981|dbj|GAD    EE--------------------------NDSAFVRVHNQTYLQGQKRLYMTATPRIYGDV
gi|889770922|ref|WP_048844278.1|damageinducibleprotein[Gluconobacterfrateurii]/11419    EE--------------------------NDSAFVRVHNQTYLQGQKRLYMTATPRIYGDV
gi|504886208|ref|WP_015073310.1|hypotheticalprotein[Gluconobacteroxydans]_gi|411027954|gb|AFW01209.1|hypotheticalproteinB932_16    EE--------------------------NDSAFVRVHNQTYLQGQKRLYMTATPRIYGDV
gi|480040211|gb|ENV79751.1|hypotheticalproteinF942_01641[AcinetobacterursingiiANC3649]/11432    E---------------------------DDSAFTSVHDEEFIHAKKRLYMTATPRIYSED
gi|822498222|ref|WP_046936504.1|damageinducibleprotein[Xanthomonasgardneri]_gi|821751224|gb|KLB07223.1|damageinducibleprotein/1    D---------------------------SESAFVRVHDGQIIRAAKRLYMTATPRIYGNE
gi|654059559|ref|WP_027705556.1|damageinducibleprotein[Zymobacterpalmae]/11635    E---------------------------DDSAFVRVHDNDYLYGRKRLYMTATPRIYGDD
gi|643535620|ref|WP_025224315.1|damageinducibleprotein[Methylophilaceaebacterium11]_gi|577704400|gb|EUJ10546.1|putativehelicas/    D---------------------------DESKFVKVHDAAFIKAHKRLYMTATPRIYGEM
MBA11I/11635    D---------------------------DESKFVKVHDAAFIKAHKRLYMTATPRIYGEM
gi|655355857|ref|WP_028762287.1|damageinducibleprotein[Shewanellacolwelliana]/11632    E---------------------------DESAFVRVHDQNFIRADKRLYMTATPRLYGNS
gi|753204236|ref|WP_041510579.1|damageinducibleprotein[Shewanellasp.cp20]_gi|752457765|gb|KIO36326.1|damageinducibleprotein[S/1    E---------------------------DESAFVRVHDKKIIHGIKRLYMTATPRIYGEA
KPNNIH30III/11619    E---------------------------DESAFVRVHDGCFIHGQKRLYMTATPRIYGES
gi|749295994|ref|WP_040120352.1|damageinducibleprotein[Klebsiellapneumoniae]_gi|723249300|gb|AIX81926.1|damageinducibleprotei/1    E---------------------------DESAFVRVHDGCFIHGQKRLYMTATPRIYGES
gi|641639739|emb|CDQ54010.1|unnamedproteinproduct[Klebsiellapneumoniae]/11641    E---------------------------DESAFVRVHDGCFIHGQKRLYMTATPRIYGES
gi|490797922|ref|WP_004660060.1|hypotheticalprotein[Acinetobactersp.NIPH236]_gi|479876779|gb|ENU27973.1|hypotheticalproteinF992    E---------------------------NDSNFVKIHDAGFILGKKRLYMTATPRIFSDD
gi|916750926|ref|WP_051357982.1|damageinducibleprotein[Acinetobactertjernbergiae]/11453    E---------------------------DDSNFVKIHDAGFILGKKRLYMTATPRIFSDD
gi|691098662|ref|WP_032025060.1|damageinducibleprotein[Acinetobacterbaumannii]_gi|593567521|gb|EXR50735.1|restrictionendonucle/    E---------------------------DDSNFVKIHDGGFILGKKRLYMTATPRIFSDD
```

```
gi|736669405|ref|WP_034676049.1|damageinducibleprotein[Acinetobacterbaumannii]/11643    E---------------------------DDSNFVKIHDAGFILGKKRLYMTATPRIFSDD
gi|491368928|ref|WP_005226826.1|hypotheticalprotein[Acinetobactersp.NIPH1847]_gi|480173259|gb|ENX09797.1|hypotheticalproteinF89    E---------------------------DDSNFVKIHDGGFILGKKRLYMTATPRIFSDE
gi|796853993|ref|WP_045795953.1|damageinducibleprotein[Acinetobacterindicus]_gi|787929761|gb|KJV44219.1|damageinducibleprotei/1    E---------------------------DDSNFVKIHDAGFILGKKRLYMTATPRIFSDD
gi|295797463|gb|ADG32253.1|typeIIIrestrictionproteinressubunit[ThiomonasintermediaK12]/11676    D---------------------------RESAFVRVHDADYLRAAKRLYMTATPRIYGEV
gi|499871179|ref|WP_011551913.1|DNAdamageinducibleprotein[Myxococcusxanthus]_gi|108467544|gb|ABF92729.1|putativerestriction/mo/    E---------------------------EESHFVRVHDGDYIRGKKRLYMTATPRIFGDA
gi|495313852|ref|WP_008038600.1|DNAdamageinducibleprotein[Bartonellatamiae]_gi|395424181|gb|EJF90368.1|hypotheticalproteinME5_/    D---------------------------EESAFVRIHNNDFINAAKRLYMTATPRIYRDD
gi|501453335|ref|WP_012476784.1|DNAdamageinducibleprotein[Leptospirabiflexa]_gi|167777824|gb|ABZ96123.1|Helicase[Leptospirabif/    Q---------------------------EESHFVKVHDDKFLKATKRLYMTATPRIFGDI
gi|738104613|ref|WP_036063193.1|damageinducibleprotein[Leptospiranoguchii]/11652    E---------------------------DESAFVKIHDAKFIIADKRLYMTATPRIYGDI
gi|410001039|gb|EKO51661.1|typeIIIrestrictionenzyme,ressubunit[Leptospirakirschneristr.200802841]/11651    E---------------------------DESAFVKVHDAKFIKADKRLYMTATPRIYGDI
gi|490901851|ref|WP_004763774.1|typeIIIrestrictionenzyme,ressubunit[Leptospirakirschneri]_gi|464209249|gb|EMN24014.1|typeIIIres    E---------------------------DESAFVKVHDAKFIKADKRLYMTATPRIYGDI
gi|464398131|gb|EMO65902.1|typeIIIrestrictionenzyme,ressubunit[Leptospirakirschneristr.200803703]/11648    E---------------------------DESAFVKVHDAKFIKADKRLYMTATPRIYGDI
gi|945029024|gb|KQQ84900.1|damageinducibleprotein[Xanthomonassp.Leaf131]/11423    -D--------------------------DESAFVRVHDADYLRASKRLYMTATPRIYGNE
gi|896511314|ref|WP_049418183.1|damageinducibleprotein[Stenotrophomonasmaltophilia]/11639    -D--------------------------DESAFVRVHDAAYLRAAKRLYMTATPRIYGNE
gi|896513082|ref|WP_049419616.1|damageinducibleprotein[Stenotrophomonasmaltophilia]/11638    -D--------------------------NESAFVRVHDPAYIRAAKRLYMTATPRIYGNE
gi|896580616|ref|WP_049469425.1|damageinducibleprotein[Stenotrophomonasmaltophilia]/11641    -D--------------------------DESAFVRVHDAAYIRAGKRLYMTATPRIYGNE
gi|648289350|ref|WP_026070499.1|damageinducibleprotein[Stenotrophomonasmaltophilia]/11639    -D--------------------------DESAFVRVHDATYIRAAKRLYMTATPRIYGNE
gi|656272786|ref|WP_029218442.1|damageinducibleprotein[Xanthomonascassavae]/11639    -D--------------------------GESAFVRVHDAAYIRAAKRLYMTATPRIYGNE
gi|406976236|gb|EKD98747.1|hypotheticalproteinACD_23C00290G0002[unculturedbacterium]/11679    -E--------------------------DESNFVKVHDAAFIQATKRLYMTATPRIYGDN
gi|828145737|ref|WP_047238120.1|damageinducibleprotein[Chromobacteriumsubtsugae]/11645    -E--------------------------EESAFVRVHDGAYIRAAKRLYMTATPRIYGDS
gi|654311174|ref|WP_027824327.1|damageinducibleprotein[Laribacterhongkongensis]/11641    -E--------------------------EESAFVRVHDGDYIRAAKRLYMTATPRIYGDG
gi|757242173|gb|KIR15516.1|typeIrestrictionenzymeEcoKIsubunitR[Pseudomonasfluorescens]/11778    -D--------------------------DESAFVKVHNANYIRSAKRLYMTATPRIYSDM
gi|653605161|ref|WP_027616682.1|damageinducibleprotein[Pseudomonassp.URHB0015]/11639    -D--------------------------DESAFVKVHSADFIRSSKRLYMTATPRIYGEV
gi|520868025|ref|WP_020308060.1|helicase[Pseudomonassyringae]_gi|519159034|gb|EPM49935.1|helicasedomaincontainingprotein[Pseud/    -D--------------------------DESAFVKVHIADYIRSTKRLYMTATPRIYGEL
gi|940156567|gb|KPX71668.1|Helicasedomaincontainingprotein[Pseudomonasamygdalipv.lachrymans]/11677    -D--------------------------DESAFVKVHSADFIRSSKRLYMTATPRIYGEM
gi|505460508|ref|WP_015647610.1|helicase[Pseudomonassp.GLE121]_gi|499110212|ref|YP_007974374.1|helicasedomaincontainingprotein/    -D--------------------------DESNFVKIHDADYIRSVKRLYMTATPRIYGDN
gi|940235551|gb|KPY45311.1|hypotheticalproteinALO48_200154[Pseudomonassyringaepv.rhaphiolepidis]/11631    -D--------------------------DESNFVKIHDSNYILSARRLYMTATPRIYGDN
gi|520880808|ref|WP_020312235.1|helicase[Pseudomonassyringae]_gi|519172158|gb|EPM62027.1|helicasedomaincontainingprotein[Pseud/    -E--------------------------DESNFVRIHDANYIRAAKRLYMTATPRIYGDS
gi|829046479|ref|WP_047282259.1|damageinducibleprotein[Pseudomonasfluorescens]/11630    -D--------------------------DESTFVRIHDADYIRAAKRLYMTATPRIYGDN
gi|499415493|ref|WP_011102960.1|DNAdamageinducibleprotein[Pseudomonassyringaegroupgenomosp.3]_gi|28867279|ref|NP_789898.1|heli/    -D--------------------------DESTFVRIHDVDYIRAIKRLYMTATPRIYGDN
gi|730238299|ref|WP_033942897.1|damageinducibleprotein[Pseudomonasaeruginosa]/11629    -D--------------------------DESNFVRVHDGDYIRAAKRLYMTATPRIYGDS
gi|495310125|ref|WP_008034873.1|DNAdamageinducibleprotein[Pseudomonassp.GM60]_gi|398186685|gb|EJM74052.1|putativehelicase[Pseu/    -D--------------------------DESTFVRVHDANYIHAAKRLYMTATPRIYGDN
gi|779992653|ref|WP_045428690.1|damageinducibleprotein[Pseudomonassp.MT1]_gi|699967872|dbj|BAP80334.1|DNAhelicaserestrictione/1    -D--------------------------DESTFVRIHDADYIRAAKRLYMTATPRIYGDN
gi|786037289|ref|WP_045733274.1|damageinducibleprotein[Pseudomonaspseudoalcaligenes]_gi|782987904|gb|KJU79152.1|damageinducib/1    -D--------------------------DESNFVRVHDGDYIRAAKRLYMTATPRIYGDN
gi|749666366|ref|WP_040265445.1|damageinducibleprotein[Pseudomonasrhodesiae]/11632    -D--------------------------DESTFVRIHDVDYIRATKRLYMTATPRIYGDN
gi|734902433|ref|WP_034133698.1|damageinducibleprotein[Pseudomonasfluorescens]/11635    -D--------------------------DESTFVRIHDADYIRATKRLYMTATPRIYGDN
gi|490551031|ref|WP_004416146.1|typeIIrestrictionmodificationsystemrestriction/helicasesubunit[Pseudomonassyringae]_gi|4432823/    -D--------------------------DESNFVRVHDADYIRATKRLYMTATPRIYGDN
gi|489199911|ref|WP_003109152.1|DNAdamageinducibleprotein[Pseudomonasaeruginosa]_gi|552648708|gb|ERY63184.1|hypotheticalprotei/    -D--------------------------DESNFVRIHDGDYIRAAKRLYMTATPRIYGDS
gi|730641237|ref|WP_034061328.1|damageinducibleprotein[Pseudomonasaeruginosa]/11636    -D--------------------------DESSFVRIHDADYIRSAKRLYMTATPRIYGDN
gi|751304980|ref|WP_041013386.1|damageinducibleprotein[Pseudomonasxanthomarina]_gi|703407611|emb|CEG51014.1|Helicasedomaincon/1    -D--------------------------DESTFVRIHDADYIRTAKRLYMTATPRIYGDN
gi|939335207|ref|WP_054881520.1|damageinducibleprotein[Pseudomonassp.NBRC111128]/11632    -D--------------------------DESNFVRVHDADYIRATKRLYMTATPRIYGDN
gi|757693555|ref|WP_042923792.1|damageinducibleprotein[Pseudomonaschloritidismutans]_gi|780910610|gb|KJS78302.1|damageinducib/1    -D--------------------------DESTFVRIHDADYIRAAKRLYMTATPRIYGDN
gi|665953944|ref|WP_031319989.1|damageinducibleprotein[Pseudomonastolaasii]/11626    -D--------------------------DESNFVRIHDGADIRAAKRLYMTATPRIYGDS
gi|771600573|ref|WP_045213746.1|damageinducibleprotein[Pseudomonassp.21]_gi|770081123|gb|KJJ98634.1|damageinducibleprotein[Ps/1    -D--------------------------DESAFVRVHDSDYIRAIKRLYMTATPRIYGDS
gi|740871396|ref|WP_038656644.1|damageinducibleprotein[Pseudomonasstutzeri]_gi|627772214|gb|AHY41437.1|damageinducibleprotein/1    -D--------------------------DESKFVRIHDADYIRAAKRLYMTATPRIYGDN
gi|946242205|gb|KRC93782.1|damageinducibleprotein[Pseudomonassp.Root9]/11634    -D--------------------------DESNFVRVHDADYIRATKRLYMTATPRIYGDN
gi|553777322|ref|WP_023109107.1|MULTISPECIES:hypotheticalprotein[Pseudomonas]_gi|552638674|gb|ERY53184.1|hypotheticalproteinQ05    -D--------------------------DESNFVRVHDADYIRATKRLYMTATPRIYGDN
gi|896383370|ref|WP_049324618.1|damageinducibleprotein[Pseudomonasstutzeri]/11627    -D--------------------------DESTFVRVHDADYLRAAKRLYMTATPRIYGDA
gi|696158510|gb|AIT41787.1|putativehelicase[Pseudomonassp.LM8]/11630    -D--------------------------DESTFVRVHDADYLRAAKRLYMTATPRIYGDA
gi|504634892|ref|WP_014821994.1|DNAdamageinducibleprotein[Pseudomonasstutzeri]_gi|390985249|gb|AFM35242.1|helicasedomainconta/1    -D--------------------------DESTFVRIHDANYIRAAKRLYMTATPRIYGDN
gi|544783974|ref|WP_021206729.1|DNAdamageinducibleprotein[Pseudomonasstutzeri]_gi|533224779|gb|EQM79688.1|DNAdamageinduciblep/1    -D--------------------------DESTFVRVHDADYLRAAKRLYMTATPRIYGDA
gi|504567385|ref|WP_014754487.1|MULTISPECIES:DNAdamageinducibleprotein[Pseudomonas]_gi|388559493|gb|AFK68634.1|helicasedomain/1    -D--------------------------DESTFVRIHDADYIRASKRLYMTATPRIYGDN
gi|654441469|ref|WP_027912052.1|damageinducibleprotein[Pseudomonassp.URIL14HWK12:I7]/11630    -D--------------------------DESNFVRVHDADYIRATKRLYMTATPRIYGDN
gi|941470066|ref|WP_055127894.1|damageinducibleprotein[Pseudomonasmediterranea]/11631    -D--------------------------DESNFVRVHDADYIRATKRLYMTATPRIYGDN
gi|557815599|ref|WP_023446184.1|DNAdamageinducibleprotein[Pseudomonaschloritidismutans]_gi|557364081|gb|ESQ98190.1|DNAdamagei/1    -D--------------------------DESNFVRVHDADYIRATKRLYMTATPRIYGDN
gi|727814791|ref|WP_033898247.1|damageinducibleprotein[Pseudomonassp.FH1]_gi|567648005|gb|ETK23229.1|helicasedomaincontaining/1    -D--------------------------DESTFVRIHDVDYIRATKRLYMTATPRIYGDN
gi|503278702|ref|WP_013513363.1|DNAdamageinducibleprotein[Desulfovibrioaespoeensis]_gi|316942375|gb|ADU61426.1|typeIIIrestrict/    -D--------------------------DESNFVKVHENRFIQAAKRIYMTATPRIYGDT
gi|505228796|ref|WP_015415898.1|typeIIIrestrictionendonucleasesubunitR[Desulfovibriopiezophilus]_gi|451896976|emb|CCH49855.1|Ty    -D--------------------------DESNFVKVHENRFVKAAKRIYMTATPRLYGDT
gi|517732543|ref|WP_018902751.1|hypotheticalprotein[Variovoraxparadoxus]/11675    -D--------------------------DESNFVKVHDADFLRSVKRLYMTATPRIYGNA
gi|550909475|ref|WP_022662570.1|DNAdamageinducibleprotein[Desulfovibriolongus]/11637    -D--------------------------DESNFVKVHDSAFIRSAKRLYMTATPRIYGDT
gi|945494542|gb|KQV50024.1|damageinducibleprotein[Massiliasp.Root335]/11647    -D--------------------------DESNFVKVHDSAFIRSAKRLYMTATPRIYGDS
gi|334196917|gb|AEG70102.1|helicasedomainprotein[RalstoniasolanacearumPo82]/11663    -D--------------------------DESTFVRVHDAKYIRSAKRLYMTATPRIYGDT
gi|656006746|ref|WP_029048170.1|damageinducibleprotein[Cupriavidussp.amp6]/11661    -D--------------------------DESTFVRVHDADYIRSAKRLYMTATPRIYGDS
BPS2196ORF3054P/11659    -D--------------------------DESAFVRVHDADYIRSAKRLYMTATPRIYGDS
gi|740932312|ref|WP_038717332.1|damageinducibleprotein[Burkholderiapseudomallei]_gi|760211882|gb|KIX58314.1|damageinduciblepr/1    -D--------------------------DESAFVRVHDADYIRSAKRLYMTATPRIYGDS
gi|755810813|ref|WP_042583861.1|damageinducibleprotein[Burkholderiasp.MSHR3999]_gi|755174952|gb|KIP19790.1|DEAD/DEAHboxhelicas/    -D--------------------------DESAFVRVHDGDYIRAAKRLYMTATPRIYGDS
gi|484586570|gb|AGK48195.1|DEAD/DEAHboxhelicasefamilyprotein[BurkholderiathailandensisMSMB121]/11646    -D--------------------------DESAFVRVHDGDYIRAAKRLYMTATPRIYGDS
gi|737582935|ref|WP_035553941.1|damageinducibleprotein[Burkholderiasp.9120]/11658    -D--------------------------DESTFVRVHDADYIRAAKRIYMTATPRIYGDS
```

```
gi|740989872|ref|WP_038773225.1|damageinducibleprotein[Burkholderiapseudomallei]_gi|705645291|gb|KGS70691.1|DEAD/DEAHboxhelica/      -D--------------------------DESAFVRVHDADYIRSAKRLYMTATPRIYGDS
gi|654912525|ref|WP_028363424.1|damageinducibleprotein[Burkholderiasp.JPY366]/11659      -D--------------------------DESAFVRVHDADYIRSAKRLYMTATPRIYGDL
gi|740955879|ref|WP_038740512.1|damageinducibleprotein[Burkholderiapseudomallei]_gi|714684932|gb|KGW74438.1|DEAD/DEAHboxhelica/      DD--------------------------DESHFVKVHDGDFIKAAKRLYMTATPRIYSDT
gi|919203714|ref|WP_052759215.1|damageinducibleprotein[Xanthomonasaxonopodis]/11388      ---------------------------DDESAFVRVHKPDFLVAKRRLYMTATPKIYADE
gi|522152381|ref|WP_020663589.1|hypotheticalprotein[Amycolatopsisbenzoatilytica]/11605      ---------------------------QDESQFVKVHDNTVIKATKRLYMTATPRLFDDN
gi|663196013|ref|WP_030233899.1|damageinducibleprotein[Streptomycessp.NRRLS455]/11641      ---------------------------HDESNFVRVHDNDVIRAARRLYMTATPRIYNED
gi|750429553|ref|WP_040711444.1|damageinducibleprotein[Nocardiopsispotens]/11622      ---------------------------ADESAFVRVHDDGFIGADRRLYMTATPRVYEEK
gi|518656139|ref|WP_019819475.1|hypotheticalprotein[Saccharomonosporasaliphila]/11623      ---------------------------HDESTFVRVHDNNFISADRRLYMTATPRVYSDD
gi|759722933|ref|WP_043433758.1|damageinducibleprotein[Streptomycespluripotens]_gi|733404978|gb|KHK94361.1|damageinduciblepro/1      ---------------------------HDESAFVRVHDDNFIGADRRLYMTATPRVYSDD
gi|663424167|ref|WP_030419030.1|damageinducibleprotein[Streptomycessp.NRRLF5065]/11627      ---------------------------HDESAFVRVHDDSFIGADRRLYMTATPRVYSDD
gi|663412917|ref|WP_030408353.1|damageinducibleprotein[Streptomycessp.NRRLF5917]/11630      ---------------------------HDESAFVRVHDDSFIGADRRLYMTATPRVYSDD
gi|671521483|ref|WP_031506489.1|damageinducibleprotein[Streptomycesmegasporus]/11630      ---------------------------HDESAFVRVHDDSFIGADRRLYMTATPRVYSDD
gi|740053144|ref|WP_037902776.1|damageinducibleprotein[Streptomycessp.TAA204]/11626      ---------------------------HDESAFVRVHNDDYLGAERRLYMTATPRIYNED
gi|663409375|ref|WP_030404974.1|MULTISPECIES:damageinducibleprotein[Streptomyces]/11627      ---------------------------HDESAFVRVHDDSYLGADRRLYMTATPRIYNED
gi|746628608|ref|WP_039654277.1|damageinducibleprotein[Streptomycessp.MUSC125]_gi|745644381|gb|KIE25557.1|damageinducibleprot/1      ---------------------------HDESAFVRVHDDAYLGADRRLYMTATPRIYNED
gi|727322066|ref|WP_033777320.1|damageinducibleprotein[Salinisporapacifica]/11627      ---------------------------HDESSFVRVHDDTYLGGDRRLYMTATPRIYNED
gi|653653242|ref|WP_027655726.1|damageinducibleprotein[Salinisporapacifica]/11618      ---------------------------HDESSFVRVHDDTYLSGDRRLYMTATPRIYNED
gi|648632543|ref|WP_026324294.1|damageinducibleprotein[Salinisporapacifica]/11621      ---------------------------QDESAFVRIHDEPYIGGDRRLYMTATPRLYNES
gi|881018002|ref|WP_048738463.1|damageinducibleprotein[Corynebacteriumfalsenii]/11676      ---------------------------EAESNFVRIHDSDYIRASKRLYMTATPRLFDDA
gi|505465526|ref|WP_015652417.1|helicase[Corynebacteriumcallunae]_gi|459385704|gb|AGG67995.1|helicase[CorynebacteriumcallunaeDS      ---------------------------EDPSNFTRIHDASYIKAAKRLYMTATPRLFDDS
gi|41223062|emb|CAF19007.1|DNAorRNAhelicaseofsuperfamilyII[CorynebacteriumglutamicumATCC13032]/11646      ---------------------------EDPSNFTRIHDASYIKAAKRLYMTATPRLFDDS
CGL13032I/11643      ---------------------------EDPSNFTRIHDASYIKAAKRLYMTATPRLFDDS
gi|751257081|ref|WP_040967675.1|damageinducibleprotein[Corynebacteriumglutamicum]_gi|748811620|gb|AJE67931.1|damageinduciblep/1      ---------------------------EDSSHFVRVHDADYIKAAKRLYMTATPRLFDDQ
gi|749040539|ref|WP_040086524.1|damageinducibleprotein[Corynebacteriumhumireducens]/11663      ---------------------------EDASNFVRIHDNNYVKAAKRLYMTATPRLFDDA
gi|491662899|ref|WP_005519615.1|helicase[Corynebacteriummatruchotii]_gi|224946756|gb|EEG27965.1|helicaseCterminaldomainprotein/      ---------------------------DEPSVFSRIHDANYIAGTKRLYMTATPRLFDDN
gi|746717312|ref|WP_039676231.1|damageinducibleprotein[Corynebacteriumminutissimum]_gi|734991118|gb|KHO29219.1|damageinducibl/1      ---------------------------EDASTFVKIHDEKYIKASKRLYMTATPRLFDDN
gi|908654892|emb|CQD15529.1|SuperfamilyIIDNA/RNAhelicase(modularprotein)[Corynebacteriumstriatum]/11674      ---------------------------EDASNFVKIHDEKYIKASKRLYMTATPRLFDDN
gi|895994446|ref|WP_049064418.1|damageinducibleprotein[Corynebacteriumaurimucosum]/11667      ---------------------------EDASNFVKIHDEKYIKASKRLYMTATPRLFDDN
gi|896123331|ref|WP_049145390.1|damageinducibleprotein[Corynebacteriumaurimucosum]/11669      ---------------------------EDASNFVKIHDEKYIKASKRLYMTATPRLFDDN
gi|817681178|ref|WP_046645422.1|damageinducibleprotein[Corynebacteriumstriatum]_gi|817551679|gb|KKO79868.1|damageinduciblepro/1      ---------------------------EDASNFVKIHDEKYIKASKRLYMTATPRLFDDN
gi|896139239|ref|WP_049159146.1|damageinducibleprotein[Corynebacteriumaurimucosum]/11675      ---------------------------EDASNFVKIHDEKYIKASKRLYMTATPRLFDDN
gi|489943947|ref|WP_003847254.1|helicase[Corynebacteriumammoniagenes]_gi|295968218|gb|EFG81467.1|helicaseCterminaldomainprotei/      ---------------------------EDASNFVRVHDEDYIKADKRLYMTATPRLFDDS
gi|516653464|ref|WP_018022453.1|helicase[Corynebacteriumdoosanense]_gi|697778282|gb|AIT61018.1|damageinducibleprotein[Coryneba/      ---------------------------EDASNFVRVHDADYIKSHKRLYMTATPRLFDDT
gi|493894179|ref|WP_006840129.1|helicase[Corynebacteriumlipophiloflavum]_gi|227078819|gb|EEI16782.1|helicaseCterminaldomainpro/      ---------------------------EEASNFTKVHDADYIRAAKRLYMTATPRLFDDA
gi|740831472|ref|WP_038616755.1|damageinducibleprotein[Corynebacteriumpseudotuberculosis]_gi|666103058|gb|AIG06935.1|putativeh/      ---------------------------QDASNFVKVHDPAYIKAEKRLYMTATPRLFDDT
gi|518023322|ref|WP_019193530.1|helicase[Corynebacteriumtimonense]/11655      ---------------------------EDASNFVRVHDGAYIEATKRLYMTATPRLFDDA
gi|552767697|ref|WP_023019559.1|MULTISPECIES:hypotheticalprotein[Corynebacterium]_gi|550739013|gb|ERS38609.1|hypotheticalprotei      ---------------------------EDASNFVRIHEGDYIKADKRLYMTATPKLFDDD
gi|652659121|ref|WP_027017812.1|damageinducibleprotein[Corynebacteriumpseudodiphtheriticum]/11644      ---------------------------EDASNFVRIHEGDYIKADKRLYMTATPKLFDDD
gi|749039309|ref|WP_040085390.1|damageinducibleprotein[Corynebacteriumhumireducens]/11657      ---------------------------EDASNFVRIHDNNYIKATKRLYMTATPRLFDDA
gi|740835591|ref|WP_038620874.1|damageinducibleprotein[Corynebacteriumulcerans]_gi|698973854|gb|AIU33664.1|HelicaseCterminald/1      ---------------------------QDASNFVRVHDAHYIKAAKRLYMTATPRLYDDA
gi|805307599|ref|WP_046096452.1|damageinducibleprotein[Corynebacteriumulcerans]_gi|803357323|gb|AKA97638.1|Damageinduciblepro/1      ---------------------------QDASNFVRVHDAHYIKAAKRLYMTATPRLYDDA
gi|504083555|ref|WP_014317549.1|helicase[Corynebacteriumdiphtheriae]_gi|371584249|gb|AEX47914.1|superfamilyIIDNA/RNAhelicase[Co      ---------------------------EEASNFTKIHDANYIKVSKRLYMTATPRLFDEA
gi|896182193|ref|WP_049193580.1|damageinducibleprotein[Corynebacteriumjeikeium]/11645      ---------------------------EEASNFTKIHDATYIKAAKRLYMTATPRLFDET
gi|917636459|ref|WP_052205460.1|damageinducibleprotein[Corynebacteriumriegelii]_gi|914295811|gb|AKV59187.1|damageinduciblepro/1      ---------------------------ADESNFVRIHDADYIQAARRLYMTATPRLFDDT
gi|737013522|ref|WP_035009205.1|damageinducibleprotein[Corynebacteriumjeikeium]/11651      ---------------------------ADESNFVRIHDEDYIKSARRLYMTATPRLFDDS
gi|737006291|ref|WP_035002094.1|damageinducibleprotein[Corynebacteriumjeikeium]/11658      ---------------------------EDASNFVRIHDADYIRSAKRLYMTATPRLFDDQ
gi|737010378|ref|WP_035006133.1|damageinducibleprotein[Corynebacteriumjeikeium]/11650      ---------------------------EDASNFVRIHDADYIRSAKRLYMTATPRLFDDQ
gi|737010473|ref|WP_035006228.1|damageinducibleprotein[Corynebacteriumjeikeium]/11657      ---------------------------EDSSTFVKIHDDSYIKTAKRLYMTATPRLYDDT
gi|737006250|ref|WP_035002053.1|damageinducibleprotein[Corynebacteriumjeikeium]/11658      ---------------------------EDSSNFVKIHDDSYIKTAKRLYMTATPRLYDDT
gi|923018168|ref|WP_053412254.1|damageinducibleprotein[Corynebacteriumlactis]_gi|922845184|gb|ALA67504.1|helicase[Corynebacter/      ---------------------------EEASNFTKIHDAAYIKAAKRLYMTATPRLFDEA
gi|923018885|ref|WP_053412970.1|damageinducibleprotein[Corynebacteriumlactis]_gi|922845845|gb|ALA68165.1|helicase[Corynebacter/      ---------------------------EEASNFTKIHDAAYIKAAKRLYMTATPRLFDEA
gi|512464870|ref|WP_016422869.1|hypotheticalprotein[Corynebacteriumsp.HFH0082]_gi|512047830|gb|EPD47505.1|hypotheticalproteinHM      ---------------------------EDASNFVRIHEADYIKATKRLYMTATPRLYDDS
gi|881012697|ref|WP_048733406.1|damageinducibleprotein[Corynebacteriumurealyticum]/11670      ---------------------------EDASNFVRIHDADYIKTKKRLYMTATPRLYDDS
gi|755608478|ref|WP_042530519.1|damageinducibleprotein[Corynebacteriumsingulare]_gi|754340239|gb|AJI78730.1|putativehelicase[C/      ---------------------------EDASNFVRVHDADYIKASKRLYMTATPRLYDEA
gi|491421796|ref|WP_005279593.1|helicase[Corynebacteriumaccolens]_gi|227077180|gb|EEI15143.1|helicaseCterminaldomainprotein[Co/      ---------------------------EDSSNFVRVHDTDYIKAAKRLYMTATPRLFDDA
gi|550752286|gb|ERS51451.1|hypotheticalproteinHMPREF1282_00033[Corynebacteriumsp.KPL1856]_gi|550759961|gb|ERS58964.1|hypothetic      ---------------------------EDASNFVRIHDANYIRASKRLYMTATPRLYDDA
gi|497955053|ref|WP_010269209.1|helicase[Corynebacteriumbovis]/11599      ---------------------------EDPGQFVRVHDPEYIRARHRLYMTATPRLYDDE
gi|860580040|ref|WP_048470569.1|hypotheticalprotein[Mycobacteriumchlorophenolicum]_gi|859098575|gb|KMO76871.1|typeIrestrictione      ---------------------------GDESNFVRIHDNDYLRADRRLYMTATPRIYSDA
gi|262085905|gb|ACY21873.1|typeIIIrestrictionproteinressubunit[GordoniabronchialisDSM43247]/11632      ---------------------------ADESNFVKVHDDDFLHAHRRLYMTATPRIFADT
gi|495996338|ref|WP_008720917.1|typeIIIrestrictionendonucleasesubunitR[Rhodococcussp.AW25M09]_gi|443412350|emb|CCQ18455.1|TypeI      ---------------------------ADESNFVRVHDAEFLKASRRLYMTATPRIFDDA
gi|739284636|ref|WP_037147410.1|damageinducibleprotein[Rhodococcusfascians]/11641      ---------------------------ADESNFVRVHDAKFLKASRRLYMTATPRIFDDA
gi|739328195|ref|WP_037190574.1|damageinducibleprotein[Rhodococcusfascians]/11643      ---------------------------ADESNFVRVHDAEFLKASRRLYMTATPRIFDDA
gi|811698335|emb|CMN21041.1|helicase[Mycobacteriumtuberculosis]/11725      ---------------------------DDESNFVRVHDGQYLKAARRLYMTATPRIFTES
gi|801577898|emb|CFK23773.1|helicase[Mycobacteriumtuberculosis]/11675      ---------------------------DDESNFVRVHDGQYLKAARRLYMTATPRIFTES
MTU18II/11606      ---------------------------DDESNFVRVHDGQYLKAARRLYMTATPRIFTES
gi|887719858|emb|CMT27886.1|helicase[Mycobacteriumtuberculosis]/11667      ---------------------------DDESNFVRVHDGQYLKAARRLYMTATPRIFTES
gi|621694743|gb|KAS87156.1|hypotheticalproteinAL70_01801[MycobacteriumtuberculosisTKK_03_0115]/11604      ---------------------------DDESNFVRVHDGQYLKAARRLYMTATPRIFTES
```

gi|888128072|emb|CLQ84422.1|helicase[Mycobacteriumtuberculosis]_gi|889917090|emb|CLY86893.1|helicase[Mycobacteriumtuberculosis] ----------------------------DDESNFVRVHDGQYLKAARRLYMTATPRIFTES
gi|884823424|emb|CKL96672.1|helicase[Mycobacteriumtuberculosis]/11466 ----------------------------DDESNFVRVHDGQYLKAARRLYMTATPRIFTES
gi|505103239|ref|WP_015290341.1|hypotheticalprotein[Mycobacteriumcanettii]_gi|432162733|emb|CCK60117.1|Conservedproteinofunknow ----------------------------EDESNFVRVHDGQYLKAARRLYMTATPRIFTES
gi|505116250|ref|WP_015303352.1|hypotheticalprotein[Mycobacteriumcanettii]_gi|432158726|emb|CCK56026.1|Conservedproteinofunknow ----------------------------DDESNFVRVHDGQYLKAARRLYMTATPRIFTES
gi|432154735|emb|CCK51974.1|Conservedproteinofunknownfunction(part1)[MycobacteriumcanettiiCIPT140060008]/11588 ----------------------------DDESNFVRVHDGQYLKAARRLYMTATPRIFTES
gi|809008027|ref|WP_046295375.1|hypotheticalprotein[Mycobacteriumsp.UM_Kg17]/11624 ----------------------------DDESNFVRIHDADYLKAQRRLYMTATPRIFTDS
gi|808802633|ref|WP_046284139.1|hypotheticalprotein[Mycobacteriumsp.UM_NZ2]/11624 ----------------------------DDESNFVRIHNPDYLKAQRRLYMTATPRIFTDA
gi|750125774|ref|WP_040429852.1|damageinducibleprotein[Corynebacteriumnuruki]/11668 ----------------------------EDASNFVKVHDPDFIRSAKRLYMTATPRLFDDT
gi|602237614|gb|EYT60960.1|damageinducibleprotein[Dietziasp.UCDTHP]/11473 ----------------------------DDPSNFTRVHDADFLRAHRRLYMTATPRLFDDT
gi|665897412|ref|WP_031264618.1|damageinducibleprotein[Dietziasp.UCDTHP]_gi|602237599|gb|EYT60945.1|helicase[Dietziasp.UCDTH/11 ----------------------------DDPSNFTRVHDADFLRAHRRLYMTATPRLFDDT
gi|502903674|ref|WP_013138650.1|helicase[Segniliparusrotundus]_gi|296181291|gb|ADG98197.1|typeIIIrestrictionproteinressubunit[S ----------------------------EDESNFVRIHDGDYLRAKRRLYMTATPRIYDEK
gi|780248306|ref|WP_045538930.1|hypotheticalprotein[Gordoniasp.no.9]_gi|778142514|gb|KJR06917.1|hypotheticalproteinUG54_12320[G ----------------------------ADESNFVKVHDPDFLKSARRLYLTATPRIYDQK
gi|665897368|ref|WP_031264574.1|damageinducibleprotein[Dietziasp.UCDTHP]_gi|602239137|gb|EYT62483.1|helicase[Dietziasp.UCDTH/11 ----------------------------EDASNFVAVHDPDFLKASRRLYMTATPRLFNDE
gi|493372923|ref|WP_006329236.1|helicase[Gordoniarhizosphera]_gi|403207917|dbj|GAB88168.1|hypotheticalproteinGORHZ_006_00370[Go ----------------------------EDASNFVLVHNNDTIKGHRRLYMTATPRLYSED
gi|764904870|ref|WP_044487821.1|hypotheticalprotein[Mycobacteriumtuberculosis]_gi|625005353|gb|KBZ57120.1|hypotheticalproteinK8 ----------------------------TRESNFVKVHDANYVHADRRLYMTATPRIYDET
gi|927068212|ref|WP_053778441.1|damageinducibleprotein[Gordoniasp.NB41Y]_gi|926660541|gb|EMP14526.2|damageinducibleprotein[G/11 ----------------------------GDESNFVRVHDMSDYIKSARRLYMTATPRIFDES
gi|935514169|ref|WP_054429894.1|damageinducibleprotein[Mycobacteriumimmunogenum]_gi|929801120|gb|KPG29247.1|damageinduciblepr/1 ----------------------------AAESNFVRIHDADYIKAARRLYMTATPRIFDEA
gi|941317728|ref|WP_055118574.1|hypotheticalprotein[Mycobacteriumperegrinum]/11657 ----------------------------ADESNFVKVHDEDYLHATRRLYMTATPRIFDQA
gi|933683673|gb|ALG86377.1|hypotheticalproteinACH46_20120[Gordoniasp.QH11]/11636 ----------------------------EDESNFVRVHDADYLKATRRLYMTATPRIYDDN
gi|873553947|emb|CRY84110.1|Predictedhelicase[Nocardiafarcinica]/11688 ----------------------------DDESNFVRVHDGDYLKAQRRLYMTATPRIFDET
gi|499525749|ref|WP_011212389.1|helicase[Nocardiafarcinica]_gi|54019339|dbj|BAD60707.1|hypotheticalprotein[NocardiafarcinicaIFM ----------------------------DDESNFVRVHDGDYLKAQRRLYMTATPRIFDET
gi|640109609|ref|WP_024794187.1|damageinducibleprotein[Tomitellabiformata]/11653 ----------------------------NDESNFVRVHDGDFLKSTRRLYMTATPRIFDDN
gi|640109991|ref|WP_024794334.1|hypotheticalprotein[Tomitellabiformata]/11639 ----------------------------NDESNFVRVHDGDFLKASRRLYMTATPRIFDDN
gi|939125496|ref|WP_054831438.1|damageinducibleprotein[Rhodococcuserythropolis]/11669 ----------------------------EDDSNFVRVHDAEFLKATRRLYMTATPRIFDDT
gi|910258550|ref|WP_050062711.1|hypotheticalprotein[Rhodococcussp.RD6.2]_gi|846871967|emb|CRK49225.1|conservedhypotheticalprote ----------------------------DDESNFVRVHDADYLKSERRLYMTATPRIFDET
gi|797148189|gb|KJY52358.1|putativeHelicase[Bifidobacteriumasteroides]/11801 ----------------------------GKTSPFVLVHSAELLKSARRLYMTATPRVYADA
gi|535476570|gb|EFG27357.2|hypotheticalproteinHMPREF9020_00999[ScardoviainopinataF0304]/11827 ----------------------------GVKSAFVKVHDNSLVHAKKRLYMTATPRVYTQA
gi|737482729|ref|WP_035462496.1|hypotheticalprotein[Alicyclobacillusmacrosporangiidus]/11512 ----------------------------QDPSGFLMFHDDGKIQAKRRLYLTATPRIFTET
gi|685434354|gb|KGA95679.1|helicase[BacillusalcalophilusATCC27647=CGMCC1.3604]/11575 ----------------------------KEASAFTKVHSDKNIKTAKRLYQTATPRVYGED
gi|945370253|gb|KQU27251.1|helicase[Bacillussp.Leaf13]/11557 ----------------------------NEDSSFTKVHYDENIKTAKRLYQTATPRVYGDA
gi|880997015|ref|WP_048719116.1|helicase[Enterococcushirae]/11563 ----------------------------GEATAFTKVHSNNNIRAKKRLYQTATPRVYGES
gi|945356929|gb|KQU14100.1|helicase[Bacillussp.Leaf49]/11547 ----------------------------NELSHFTKVHYDDYIKAAKRLYQTATPRVYGDK
gi|763482876|gb|KIZ53217.1|helicase[Bacillussafensis]/11563 ----------------------------TEASAFTKVHDDNNIKTAKRLYQTATPRVYGED
gi|852223292|ref|WP_048309707.1|helicase[Anaerobacillusmacyae]_gi|850322910|gb|KMM38590.1|helicase[Anaerobacillusmacyae]/11573 ----------------------------KDDSAFVKVHNDNNIKAKKRLYQTATPRIYGEN
gi|739708665|ref|WP_037562619.1|helicase[Sporolactobacillusterrae]/11573 ----------------------------KEASAFVKVHSNSNIKAKKRLYQTATPRIYGED
gi|496687561|ref|WP_009329104.1|MULTISPECIES:helicase[Bacillus]_gi|317390867|gb|EFV71668.1|endonucleaseandmethylaseLlaGI[Bacill ----------------------------KEASAFVKVHSDNNIKAKKRLYQTATPRIYGED
gi|930213599|gb|KPH74225.1|helicase[Oceanobacilluscaeni]/11569 ----------------------------KESSAFVKVHSDSNIKAKKRLYQTATPRIYGED
gi|516366003|ref|WP_017756036.1|helicase[Bacillussp.ZYK]/11571 ----------------------------KEASAFVKVHSNNNIKAKKRLYQTATPRIYGED
gi|488909977|ref|WP_002821052.1|helicase[Oenococcusoeni]_gi|399965230|gb|EJN99855.1|endonucleaseandmethylaseLlaGI[Oenococcusoen ----------------------------EEA-SFTKVHDNQNVSAKLRLYQTATPKIYGPD
gi|495019967|ref|WP_007745780.1|helicase[Oenococcuskitaharae]_gi|366983682|gb|EHN59081.1|typeIIIrestrictionenzyme,ressubunit[Oe ----------------------------EQA-SFTKVHDNANVSAKLRLYQTATPKIYGPD
gi|489666119|ref|WP_003570406.1|typeIIIrestrictionendonuclease[Lactobacilluscasei]_gi|410527834|gb|EKQ02696.1|typeIIIrestrictio ----------------------------EEAGMFTKVHSNSIIKAHHRLYQTATPKIYSQD
gi|511447623|gb|EPC78110.1|superfamilyIIDNA/RNAhelicase[Lactobacillusparacaseisubsp.paracaseiLpp221}/11615 ----------------------------TEAGIFTKVHNNRIVRARHRLYQTATPKIYSQE
gi|497700411|ref|WP_010014595.1|helicase[Lactobacilluscoryniformis]/11585 ----------------------------EEAGIFTKVHNNKIVQARHRLYQTATPKIYSQD
gi|489784048|ref|WP_003687939.1|helicase[Lactobacillusmali]_gi|394428539|gb|EJF01080.1|superfamilyIIDNA/RNAhelicase[Lactobacill ----------------------------TEAGMFTKVHSNNTVKAKHRLYQTATPKIYSQD
gi|446780825|ref|WP_000858081.1|helicase[Staphylococcusaureus]_gi|374396604|gb|EHQ67835.1|typeIIIrestrictionenzyme,ressubunit[S ----------------------------KKSSHFTKVHNNNYINGEIRLYQTATPKIYSDD
gi|510796658|ref|WP_016171456.1|hypotheticalprotein[Enterococcusdispar]_gi|508167532|gb|EOT44083.1|hypotheticalproteinOMK_00227 ----------------------------QEESPFAKVHSNINIKGKLRLYQTATPKIYSDE
gi|612613473|gb|EZS30411.1|hypotheticalproteinW603_02482[StaphylococcusaureusVET0353R]_gi|612881917|gb|EZU93992.1|hypotheticalp ----------------------------EEQSPFAKVHSNNNVKGKLRLYQTATPKIYSDD
gi|488332893|ref|WP_002402278.1|helicase[Enterococcusfaecalis]_gi|306513864|gb|EFM82466.1|helicaseCterminaldomainprotein[Enter/ ----------------------------EEASVFTRVHNNNYIKASKRLYQTATPKIYGTD
gi|921166029|ref|WP_053111806.1|helicase[Enterococcusdurans]_gi|915771660|gb|AKX85928.1|helicase[Enterococcusdurans]/11562 ----------------------------EEASVFTRVHNNNYIKANKRLYQTATPKIYGTD
gi|498525358|ref|WP_010825030.1|hypotheticalprotein[Enterococcusfaecalis]_gi|487059631|gb|EOJ86727.1|hypotheticalproteinWOC_005 ----------------------------EEASVFTRVHNNNYVKANKRLYQTATPKIYGTD
gi|498412624|ref|WP_010722070.1|hypotheticalprotein[Enterococcusfaecium]_gi|486634654|gb|EOF75253.1|hypotheticalproteinSGC_0253 ----------------------------EEASVFTRVHNNNYIKASKRLYQTATPKIYGTD
gi|498408534|ref|WP_010718364.1|hypotheticalprotein[Enterococcusfaecium]_gi|486609608|gb|EOF50873.1|hypotheticalproteinSCW_0257 ----------------------------EEASVFTRVHNNNYIKASKRLYQTATPKIYGTD
gi|498407475|ref|WP_010717483.1|hypotheticalprotein[Enterococcusfaecalis]_gi|486594855|gb|EOF36348.1|hypotheticalproteinSCK_005 ----------------------------EEASVFTRVHNNNYIKASKRLYQTATPKIYGTD
gi|514885392|ref|WP_016615344.1|helicase[Enterococcusfaecalis]_gi|514505843|gb|EPH71676.1|helicaseprotein[Enterococcusfaecalis0 ----------------------------EEASVFTRVHNNNYIKASKRLYQTATPKIYGTD
gi|325157603|gb|EGC69759.1|helicaseCterminaldomainprotein[EnterococcuscasseliflavusATCC12755]/11561 ----------------------------EEASVFTRVHNNNYIKANKRLYQTATPKIYGTD
gi|895856206|ref|WP_048967668.1|helicase[Enterococcusfaecalis]/11551 ----------------------------EEASVFTRVHNNNYVKANKRLYQTATPKIYGTD
gi|639458836|ref|WP_024636366.1|helicase[Enterococcusfaecium]_gi|658176502|gb|KEI51159.1|helicase[EnterococcusfaeciumUC7256]/1/ ----------------------------EEASVFTRVHNNNYVKANKRLYQTATPKIYGTD
gi|488266511|ref|WP_002337719.1|hypotheticalprotein[Enterococcusfaecium]_gi|430581409|gb|ELB19854.1|hypotheticalproteinOIQ_0351 ----------------------------EEASVFTRVHNNNYVKANKRLYQTATPKIYGTD
gi|446353356|ref|WP_000431211.1|helicase[Staphylococcusaureus]_gi|253726329|gb|EES95058.1|helicaseCterminaldomainprotein[Staph/ ----------------------------KDASVFTKVHDNENVKGKLRMYQTATPKIYGDN
gi|672712068|emb|CDM14336.1|DNAorRNAhelicaseofsuperfamilyII[StaphylococcusepidermidisPM221]/11464 ----------------------------KDASAFTKVHNNENVKGKLRMYQTATPKIYGDN
gi|896298990|ref|WP_049279905.1|helicase[Staphylococcusaureus]/11571 ----------------------------KDASVFTKVHDNEKVKGKLRMYQTATPKIYGDN
gi|541903432|gb|ERH36190.1|hypotheticalproteinSEQU_00085[StaphylococcusequorumUMCCNS924]/11578 ----------------------------KDASAFTKVHDNENVRGKLRMYQTATPKIYGDN
gi|488289747|ref|WP_002360955.1|helicase[Enterococcusfaecalis]_gi|256682216|gb|EEU21911.1|endonucleaseandmethylaseLlaGI[Enteroc ----------------------------KEASVFSKVHSDIYVQGRKRIYQTATPKIYGES
gi|494392045|ref|WP_007207894.1|helicase[Enterococcusitalicus]_gi|315483798|gb|EFU74284.1|helicaseCterminaldomainprotein[Enter/ ----------------------------KEASIFSRVHSNTYVQGRKRIYQTATPKIYGES
gi|446350692|ref|WP_000428547.1|helicase[Bacilluscereus]_gi|228727580|gb|EEL78721.1|RestrictionmodificationsystemLlaBIII[Bacil/ ----------------------------KQESVFSKVHSNTNVQGIKRMYQTATPKIYADS
gi|488255302|ref|WP_002326510.1|hypotheticalprotein[Enterococcusfaecium]_gi|430480276|gb|ELA57459.1|hypotheticalproteinOGE_0473 ----------------------------KEASAFTKVHSNNNVQGKKRMYQTATPKIYGEG
gi|843253770|ref|WP_047916698.1|helicase[Lactococcuspiscium]_gi|802124677|emb|CEN29502.1|EndonucleaseandmethylaseLlaGI,TypeISPr ----------------------------KEASAFSKVHSNNNVKGLKRMYQTATPKIYGES

```
LlaBIII/11578                                                                                                    ---------------------------KEASAFSKVHSNNNVKGLKRMYQTATPKIYGES
gi|499320531|ref|WP_011011023.1|helicase[Listeriainnocua]_gi|16415805|emb|CAC42019.1|pli0021[ListeriainnocuaClip11262]/11569      ---------------------------KEASAFSKVHSNTNVKGLKRMYQTATPKIYGES
gi|760026859|ref|WP_043709973.1|helicase[Weissellacibaria]_gi|758337165|gb|KIU20061.1|UvsWhelicase[Weissellacibaria]/11567       ---------------------------KEASIFAKVHSNNNVQGRKRMYQTATPKIFGES
LlaGI/11570                                                                                                      ---------------------------KEASAFSKVHSNNNVKGLKRMYQTATPKIYGES
gi|499988414|ref|WP_011669132.1|helicase[Lactococcuslactis]_gi|116109019|gb|ABJ74139.1|DNAorRNAhelicaseofsuperfamilyII[Lactococ  ---------------------------KEASAFSKVHSNTNVKGLKRMYQTATPKIYGES
gi|685938360|ref|WP_031670319.1|helicase[Listeriamonocytogenes]_gi|590403387|gb|EXL23261.1|hypotheticalproteinX846_1956[Listeri  ---------------------------KEASAFSKVHSNNNVKGLKRMYQTATPKIYGES
gi|530793219|gb|EQC56240.1|helicase[Lactococcuslactissubsp.cremorisTIFN5]_gi|530793247|gb|EQC56263.1|helicase[Lactococcuslactis  ---------------------------KEASAFSKVHSNNNVKGLKRMYQTATPKIYGES
gi|695270006|ref|WP_032492075.1|hypotheticalprotein[Listeriamonocytogenes]_gi|695212872|ref|YP_009081522.1|hypotheticalproteinp  ---------------------------KEASAFSKVHSNNNVKGLKRMYQTATPKIYGES
gi|504101310|ref|WP_014335297.1|helicase[Leuconostocmesenteroides]_gi|859247905|gb|AKO63885.1|helicase(plasmid)[Leuconostocmese  ---------------------------KEASIFAKVHSNSNVQGLKRMYQTATPKIFGES
gi|488905190|ref|WP_002816265.1|helicase[Leuconostocmesenteroides]_gi|227351522|gb|EEJ41777.1|helicaseCterminaldomainprotein[L/  ---------------------------KEASIFAKVHSNNNVQGLKRMYQTATPKIFGES
gi|518121285|ref|WP_019291493.1|MULTISPECIES:hypotheticalprotein[Lactococcus]/11588                              ---------------------------QDDSAFTKVHSNDNIKGKLRLYQTATPKVYGQE
gi|754857213|ref|WP_042217902.1|helicase[Lactococcusgarvieae]/11549                                              ---------------------------EEDSAFVKVHDNNHIKGSKRLYQTATPKIYGAE
gi|640048455|ref|WP_024784762.1|helicase[Streptococcusmutans]/11564                                              ---------------------------EDASVFTKVHSNLNIKGKKRLYQTATPKLYGAE
gi|512695361|ref|WP_016476323.1|hypotheticalprotein[Veillonellasp.HPA0037]_gi|512079853|gb|EPD79018.1|hypotheticalproteinHMPREF  ---------------------------EDASVFTKVHDNVNVKAKKRMYQTATPKLYGSD
gi|636963514|ref|WP_024403747.1|helicase[Streptococcussuis]/11574                                                ---------------------------EEDSSFTKVHSNLNVKGLKRLYQTATPKLYGLD
gi|881115312|ref|WP_048802949.1|helicase[Staphylococcuspasteuri]/11573                                           ---------------------------EEASVFTKVHSDENVKGKKRLYQTATPKLYGPE
gi|446063996|ref|WP_000141851.1|helicase[Streptococcusmitis]_gi|307618725|gb|EFN97864.1|endonucleaseandmethylaseLlaGI[Streptoco  ---------------------------DEASSFTKVHSDLNVKGIKRLYQTATPKLYGIE
gi|488206609|ref|WP_002277817.1|restrictionmodificationsystemLlaBIII[Streptococcusmutans]_gi|449173033|gb|EMB75626.1|restricti/  ---------------------------DEDSSFIKVHSDLNVKGHKRLYQTATPKLYGTE
gi|640023290|ref|WP_024781621.1|helicase[Streptococcusmutans]/11566                                              ---------------------------DEDSSFTKVHSDLNVKGHKRLYQTATPKLYGTE
gi|814946581|ref|WP_046393781.1|helicase[Streptococcusuberis]_gi|810600885|gb|KKF57499.1|helicase[StreptococcusuberisB362]/1156  ---------------------------DDASVFTKVHSDLNVKGIKRLYQTATPKLYGVD
gi|765470762|ref|WP_044720336.1|helicase[Streptococcussuis]/11559                                                ---------------------------EEASVFTKVHSDVNVKGMKRLYQTATPKLYGAD
gi|654507127|ref|WP_027976443.1|helicase[Streptococcusdevriesei]/11558                                           ---------------------------DDDSSFTKVHRNSNVKGHKRLYQTATPKLYGTE
gi|530812902|gb|EQC72481.1|DNAorRNAhelicaseofsuperfamilyII[Streptococcussp.HSISS2]/11570                         ---------------------------EEASVFTKVHSNLNVRGVKRLYQTATPKLYGVD
gi|489158846|ref|WP_003068504.1|helicase[Streptococcusintermedius]_gi|355367114|gb|EHG14827.1|hypotheticalproteinHMPREF9682_002  ---------------------------EEASAFTKVHSDLNVKGVKRLYQTATPKLYGAD
RM.Lru27782ORF13200P/11620                                                                                       ---------------------------GEDSMFTKVHGNDNVKGRLRLYQTATPKVYGPD
gi|492676546|ref|WP_005919236.1|DNAphosphorothioationsystemrestrictionenzyme[Pediococcusacidilactici]_gi|482517924|gb|EOA08103.  ---------------------------GDPSVFTKVHSNKNVKSKLRLYQTATPKVYGVE
gi|498183116|ref|WP_010497272.1|helicase[Lactobacillusacidipiscis]/11563                                         ---------------------------GESSMFTKVHSNENIKAKLRLYQTATPKIYGTE
gi|822100485|ref|WP_046921865.1|helicase[Lactobacillusruminis]_gi|821684442|gb|KLA43321.1|restrictionmodificationsystemLlaBIII/  ---------------------------GEDSMFTKVHSDKNVAGNLRLYQTATPKVYGPD
gi|935565832|ref|WP_054450311.1|helicase[Lactobacilluskunkeei]/11573                                             ---------------------------NEDSMFTEVHSNSNVKGKLRLYQTATPKIYDQN
gi|752296887|ref|WP_041152901.1|helicase[Lactobacilluskunkeei]_gi|751663520|gb|KIM18358.1|helicase[Lactobacilluskunkeei]/11577   ---------------------------NEDSMFTEVHSNSNVKGKIRLYQTATPKIYDQN
gi|495745291|ref|WP_008469870.1|helicase[Lactobacillushominis]_gi|394484442|emb|CCI81207.1|RestrictionmodificationsystemLlaBII/  ---------------------------EGDSPFTEIHSNNHVKGKLRLYQTATPKIYDQS
gi|918672414|ref|WP_052543134.1|helicase[Lactobacillussp.]/11564                                                 ---------------------------EGDTTFTEVHSNTNVKGKLRLYQTATPKIYDQN
gi|799141631|ref|WP_045927261.1|helicase[Lactobacilluskullabergensis]_gi|797155335|gb|KJY59264.1|putativerestrictionendonucleas  ---------------------------NEDSIFTEVHSNNNVKSKLRLYQTATPKIYDQN
gi|504379990|ref|WP_014567092.1|helicase[Lactobacillusjohnsonii]_gi|329666726|gb|AEB92674.1|putativerestrictionendonuclease[Lac  ---------------------------EGDSTFTEVHSNHNIKGKIRLYQTATPKIYDQN
gi|489880179|ref|WP_003783646.1|DNAdamageinducibleprotein[Actinomycesnaeslundii]_gi|399901561|gb|EJN84440.1|typeIIrestriction/   -T--------------------------DESAFVRVHNNGYIHASKRLYMTATPRIYDDS
gi|496524497|ref|WP_009232780.1|DNAdamageinducibleprotein[Actinomycessp.oraltaxon849]_gi|365265413|gb|EHM95177.1|hypotheticalp/  -T--------------------------DESAFVRVHNNAYIHASKRLYMTATPRIYDDT
gi|915397782|ref|WP_050783167.1|damageinducibleprotein[Actinomycesoris]/11694                                    -M--------------------------DESAFVRVHNDDYLHASKRLYMTATPRIYDDT
gi|502925248|ref|WP_013160224.1|helicase[Propionibacteriumfreudenreichii]_gi|296921272|emb|CBL55824.1|helicase[Propionibacteriu  DE--------------------------GESAFVKVHNNTYLPATKRLYMTATPRIYGED
gi|732164352|emb|CEG87481.1|helicase[Propionibacteriumfreudenreichii]/11618                                      DT--------------------------SESSFVKVHDNSFLPAGKRLYMTATPRVYGEG
gi|739047402|ref|WP_036925058.1|damageinducibleprotein[Propionibacteriumthoenii]/11596                            DT--------------------------SESAFIKVHDNAYLPAAKRLYMTATPRIYGTD
gi|739112839|ref|WP_036982533.1|damageinducibleprotein[Propionibacteriumjensenii]/11594                           DT--------------------------GESAFIKVHDNSFLPALKRLYMTATPRIYGTD
gi|918023558|ref|WP_052314121.1|damageinducibleprotein[Nocardiathailandica]/11612                                -Q--------------------------DESHFVKVHDNRFIQASKRLYMTATPKVFDDA
gi|498380271|ref|WP_010694427.1|DNAdamageinducibleprotein[Saccharopolysporaspinosa]/11605                        -Q--------------------------DESAFVRVHDGEFLKARRRLYMTATPRVFGDD
gi|749991459|ref|WP_040339809.1|damageinducibleprotein[CandidatusBlastococcusmassiliensis]/11597                 -G--------------------------DESAFVRVHDADALKAAKRLYMTATPKVFGEE
gi|754177801|ref|WP_041796231.1|damageinducibleprotein[Modestobactermarinus]/11592                               -K--------------------------DESAFVRIHDADALKAAKRLYMTATPRVFGEE
gi|504188248|ref|WP_014375350.1|DNAdamageinducibleprotein[Blastococcussaxobsidens]_gi|378782790|emb|CCG02456.1|putativeHelicas/  -K--------------------------DESAFVRVHDAGALKAAKRLYMTATPRVFGEE
gi|946476713|gb|KRF26798.1|damageinducibleprotein[Phycicoccussp.Soil803]/11609                                   -T--------------------------VESQFVRVHDNSYIDAAKRLYMTATPRLFDPK
gi|700176672|gb|KGN37509.1|DNAdamageinducibleprotein[KnoelliasubterraneaKCTC19937]/11633                         -T--------------------------VESQFVRVHDNSYIKAAKRLYMTATPRLFDPK
gi|700168841|gb|KGN29914.1|hypotheticalproteinN802_10215[KnoelliasinensisKCTC19936]/11631                        -T--------------------------VESQFVRVHDNSYIKAAKRLYMTATPRLFDPK
gi|946426241|gb|KRE76610.1|damageinducibleprotein[Arthrobactersp.Soil763]/11606                                  -E--------------------------DESPFVRVHDDTYIQGEKRLYMTATPRIYDDA
gi|517318525|ref|WP_018500927.1|hypotheticalprotein[Frankiasp.BCU110501]/11592                                   -E--------------------------NESTFVKVHNDAFLPAGKRLYMTATPRIYDDS
gi|499072429|ref|WP_010851327.1|Helicase[Tetrasphaeraelongata]_gi|478756579|emb|CCH71501.1|putativeHelicase[Tetrasphaeraelongat  -E--------------------------NESAFVRIHDASYIRGDKRLYMTATPRIYDDA
gi|750080666|ref|WP_040385350.1|damageinducibleprotein[Demetriaterragena]/11625                                  -A--------------------------DESSFVKVHDNDFLPGTKRLYMTATPRLFGED
gi|752558292|ref|WP_041229523.1|damageinducibleprotein[Dermacoccusnishinomiyaensis]_gi|664689315|gb|AIF41904.1|damageinducibl/1  -S--------------------------ESSFMKVHDDDAVIAGRKRLYMTATPRIFGED
gi|829089213|ref|WP_047310701.1|damageinducibleprotein[Dermacoccussp.PE3]_gi|828384812|gb|KLO63770.1|damageinducibleprotein[D/1  -Q--------------------------DESAFVRVHNDDVIKGVKRLYMTATPRIFGED
gi|748756635|ref|WP_040014750.1|damageinducibleprotein[Dermacoccussp.Ellin185]/11596                             -Q--------------------------NESAFVRVHNNDVIQGTKRLYMTATPRIFGED
gi|916534724|ref|WP_051141848.1|damageinducibleprotein[Actinomycesneuii]/11632                                   -D--------------------------NESHFVKIHSNNNVRSDRRLYMTATPRIFNEN
gi|921140030|ref|WP_053090276.1|damageinducibleprotein[Rothiamucilaginosa]/11616                                 -E--------------------------SESNFVKIHDDNWVAGRKRVYMTATPKIFNED
gi|896406751|ref|WP_049343749.1|damageinducibleprotein[Rothiamucilaginosa]/11647                                 -E--------------------------SESNFVKIHDDKWVAGRKRVYMTATPKIFNED
gi|723608234|gb|AIY01610.1|putativehelicase[Arthrobactersp.PAMC25486]/11613                                      -G--------------------------DQSNFVKIHDNSIISANKRVYMTATPRIFNDD
gi|946394527|gb|KRE45160.1|damageinducibleprotein[Arthrobactersp.Soil736]/11616                                  -T--------------------------EESHFVKVHRNDVIAGGKRLYMTATPRLFNDD
gi|515765304|ref|WP_017197904.1|hypotheticalprotein[Arthrobactersp.M2012083]/11648                               -S--------------------------EDSHFVKVHRNDVVAGAKRLYMTATPRLFNDD
gi|945315587|gb|KQT73127.1|damageinducibleprotein[Microbacteriumsp.Leaf436]/11612                                -D--------------------------DESAFMKVHDQSFVQGKKRLYMTATPRIFSDG
gi|759908497|ref|WP_043594599.1|damageinducibleprotein[Frigoribacteriumsp.MEB024]_gi|758271359|gb|KIU03346.1|damageinduciblep/1  -G--------------------------NESAFLRVHSDEAIKTKRRLYMTATPKIYAES
gi|945072850|gb|KQR34237.1|hypotheticalproteinASF75_00055[Curtobacteriumsp.Leaf154]/11597                        -A--------------------------DESAFLRVHSDDAIKTSARVYMTATPKIYAES
gi|551263152|ref|WP_022883363.1|DNAdamageinducibleprotein[Glaciibactersuperstes]/11621                           -S--------------------------AESAFLRVHDEGAIKAKKRLYMTATPKIYGEA
gi|945452341|gb|KQV08491.1|hypotheticalproteinASC63_10170[Leifsoniasp.Root112D2]/11623                           -D--------------------------NESAFVRVHDASYIRAKKRLYMTATPKLYGEA
```

```
gi|742759483|gb|KIC69912.1|damageinducibleprotein[Kocuriarhizophila]/11610        -Q--------------------------EDSAFVRVHDQSFIRGTKRLYMTATPRVFDEG
gi|737919995|ref|WP_035885304.1|damageinducibleprotein[Kocuriarhizophila]/11611        -Q--------------------------EDSAFVRVHDQSFIRGTKRLYMTATPRVFDEG
gi|737997701|ref|WP_035959325.1|damageinducibleprotein[Kocuriamarina]_gi|725823884|gb|KHE75603.1|damageinducibleprotein[Kocur/1        -Q--------------------------EDSAFVRVHDQSFICGAKRLYMTATPRVFDEG
gi|664521070|ref|WP_031037650.1|damageinducibleprotein[Streptomycessp.NRRLWC3774]/11624        -H--------------------------DESAFVRVHDDAYLGADRRLYMTATPRIYNED
gi|500101034|ref|WP_011777041.1|DNAdamageinducibleprotein[Arthrobacteraurescens]_gi|119951547|gb|ABM10457.1|putativeHelicase[A/        -D--------------------------DDSAFVRVHDQDFIKAKKRLYMTATPRIYVQE
gi|916871953|ref|WP_051479009.1|damageinducibleprotein[Arthrobactersp.H5]/11588        -E--------------------------DDSAFVRVHDQDFLKAAKRLYMTATPRIYVQE
gi|612084649|gb|EZP43369.1|TypeIIIrestrictionenzyme,ressubunit[Micrococcusluteus]/11625        -Q--------------------------DGSAFTMVHDAAKIRAAKRLYMTATPRIYVQE
gi|759739708|ref|WP_043450422.1|damageinducibleprotein[Arthrobacterphenanthrenivorans]_gi|742758264|gb|KIC68716.1|damageinduc/1        -H--------------------------NDSAFVKVHDNTYIESGKRLYMTATPRIYVQE
gi|636845448|ref|WP_024366823.1|damageinducibleprotein[Arthrobactersp.TB26]/11605        -H--------------------------DDSAFVRVHDQEYLRAKKRLYMTATPRIYVQD
gi|500009025|ref|WP_011689743.1|DNAdamageinducibleprotein[Arthrobactersp.FB24]_gi|116613051|gb|ABK05760.1|typeIIIrestrictionen/        -H--------------------------DDSAFVRVHDQAYLRAKKRLYMTATPRIYVQD
gi|821249847|gb|KKZ12083.1|hypotheticalproteinTQ37_06090[CandidatusSynechococcusspongiarum15L]/11436        -E--------------------------DPSPFVLVHDNTRLRADRRLYMTATPKVYAER
gi|739446902|ref|WP_037306885.1|damageinducibleprotein[Ruegeriahalocynthiae]/11592        -E--------------------------EASNFVKIHDNAYVNGAKRLYMTATPRIFGDS
gi|640620081|ref|WP_025048131.1|damageinducibleprotein[Sulfitobactermediterraneus]_gi|751956650|gb|KIN77213.1|TypeIIIrestricti/        -E--------------------------DESNFVRVHDNKYVAGAKRLYMTATPRIFGDN
gi|740247634|ref|WP_038088583.1|damageinducibleprotein[Thiobacillusprosperus]_gi|684049748|gb|KFZ90853.1|putativehelicase[Thio/        -E--------------------------DASDFVKVHDNAVLRASKRLYMTATPRIFAEA
gi|518420321|ref|WP_019590528.1|hypotheticalprotein[Thioalkalivibriosp.ALE20]/11603        -E--------------------------DPSDFVKVHDNDKLTAHRRLYMTATPRIFAEQ
gi|500156826|ref|WP_011831496.1|DNAdamageinducibleprotein[Methylibiumpetroleiphilum]_gi|124261892|gb|ABM96881.1|adeninespecifi/        -D--------------------------DASEFVKVHNPALIKAKKRLYMTATPRIYGEA
gi|769978537|ref|WP_045094522.1|damageinducibleprotein[Legionellafallonii]_gi|698841736|emb|CEG55682.1|Putativeproteinwithboxh/        -K--------------------------DRSDFVKVHDNDIIRGKKRIYMTATPRIYCGS
gi|553752541|ref|WP_023085344.1|hypotheticalprotein[Pseudomonasaeruginosa]_gi|552201952|gb|ERU62653.1|hypotheticalproteinQ088_0        -E--------------------------SPSEFVKVHDHENVSGAKRLYMTATPRIFADA
gi|880980418|ref|WP_048704461.1|damageinducibleprotein[Thauerahumireducens]_gi|875132785|gb|KMU70944.1|damageinducibleprotein/1        -E--------------------------SPSEFVKVHHNENIVGAKRLYMTATPRIYAEA
gi|740135732|ref|WP_037982815.1|damageinducibleprotein[Thauerasp.27]/11603        -E--------------------------SPSEFVKVHHNENIIGAKRLYMTATPRIYAEA
gi|738017371|ref|WP_035978332.1|damageinducibleprotein[Burkholderiaglumae]/11380        -D--------------------------PAPSEFVKVHDNNVVHGKKRLYMTATPRIFGDA
gi|498506003|ref|WP_010806671.1|typeIIIrestrictionenzyme,ressubunit[Pandoraeasp.SD62]_gi|490314561|gb|EON12879.1|typeIIIrestri/        -D--------------------------PAPSEFVKVHDNGVVRGKRRIYMTATPRIFGDV
gi|500117748|ref|WP_011793753.1|DNAdamageinducibleprotein[Acidovoraxcitrulli]_gi|120587742|gb|ABM31182.1|typeIIIrestrictionenz/        -E--------------------------DPSEFVKVHNNAIVKAKKRLYMTATPRIFADK
gi|651617328|ref|WP_026611444.1|damageinducibleprotein[Methylocaldumszegediense]/11601        -E--------------------------SPSEFVKVHNNSIVRGKKRLYMTATPRIFGDS
gi|737210208|ref|WP_035194994.1|damageinducibleprotein[Acidithiobacillusferrivorans]_gi|669953220|emb|CDQ11755.1|Adeninespecif/        -N---------------------EKNTLEYSHFVRVHDNDYIHAQKRLYMTATPKIYKDD
gi|935697386|ref|WP_054506180.1|damageinducibleprotein[Achromobactersp.]_gi|928716153|emb|CUK12836.1|Hefnuclease[Achromobacter/        -D--------------------------EPSAFVSVHNKDYIRGHKRLYMTATPKIYAEA
gi|429502517|gb|ELA00827.1|adeninespecificDNAmethyltransferase[Cupriavidussp.HMR1]/11599        -D--------------------------EPSAFVSVHNKDYIQAHKRLYMTATPKIYAEA
gi|506356642|ref|WP_015876361.1|DNAdamageinducibleprotein[Burkholderiaglumae]_gi|237877158|gb|ACR29491.1|AdeninespecificDNAmet/        -D--------------------------PPSAFVSVHDKDYIRAKKRLYMTATPRIYAEA
gi|696406787|ref|WP_032978003.1|damageinducibleprotein[Bordetellahinzii]_gi|625335076|gb|KCB49343.1|typeIIIrestrictionenzyme,r/        -E--------------------------DHSAFVRVHDNAYIKANKRLYMTATPRIYAEA
gi|817529125|gb|KKO72587.1|damageinducibleprotein[Kerstersiagyiorum]/11592        -E--------------------------DHSAFVRVHDNAYIKANKRLYMTATPRIYAEA
gi|737048910|ref|WP_035044118.1|damageinducibleprotein[Aquabacteriumsp.NJ1]_gi|699052896|gb|KGM38706.1|damageinducibleprotein/1        -E--------------------------DPSAFIRVHDNAYLKGKKRLYMTATPRIYAEA
gi|822492519|ref|WP_046933319.1|MULTISPECIES:damageinducibleprotein[Xanthomonas]_gi|821703525|gb|KLA61196.1|damageinducibleprotein/1        -D--------------------------DPSAFVRVHDNTFIKGKKRLYMTATPRIYAEA
gi|751252861|ref|WP_040963740.1|damageinducibleprotein[Xanthomonasfuscans]_gi|749808363|gb|KIJ03887.1|damageinducibleprotein[/1        -D--------------------------DPSAFVRVHDNAFIKGKKRLYMTATPRIYAEA
gi|496774980|ref|WP_009368792.1|DNAdamageinducibleprotein[Bilophilasp.4_1_30]_gi|345039902|gb|EGW44205.1|hypotheticalproteinHM/        -E--------------------------DHSAFVKVHDNSHIRGKKRLYMTATPRIYAEA
gi|499241912|ref|WP_010939452.1|DNAdamageinducibleprotein[Desulfovibriovulgaris]_gi|46580580|ref|YP_011388.1|adeninespecificDN/        -E--------------------------DHSAFVRIHDADYIRGQKRLYMTATPRLYAEQ
gi|504274913|ref|WP_014462015.1|DNAdamageinducibleprotein[Bacillusmegaterium]_gi|345446919|gb|AEN91936.1|AdeninespecificDNAmet/        -D--------------------------NESHFIRIHDQNFIRGKKRLYQTATPKIFAEE
gi|504895966|ref|WP_015083068.1|typeIIrestrictionenzymeandmethyltransferasesubtypeG[Anabaenasp.90]_gi|413972424|gb|AFW96512.1|t        -E--------------------------NESYFVRVHNQDFIKAKKRLYMTATPKVYHPD
gi|518327765|ref|WP_019497972.1|hypotheticalprotein[Calothrixsp.PCC7103]/11600        -E--------------------------EESAFVRVHNQEFIKGKKRLYMTATPKLYTDG
gi|652765503|ref|WP_027079917.1|damageinducibleprotein[Luteimonasmephitis]/11593        -D--------------------------DESAFVRVHDQKFLKAAKRLYMTATPRIYADE
gi|896539951|ref|WP_049442123.1|damageinducibleprotein[Stenotrophomonasmaltophilia]/11591        -E--------------------------DESAFVRVHKPDFLVAKRRLYMTATPKIYADE
gi|500217900|ref|WP_011888043.1|DNAdamageinducibleprotein[Geobacillusthermodenitrificans]_gi|134267954|gb|ABO68149.1|Conserved/        -D--------------------------EASYFTKVHEEKFLKAKKRLYMTATPRIYADE
gi|696570298|ref|WP_033102014.1|damageinducibleprotein[Thermoactinomycesdaqus]/11594        -N--------------------------EASYFTKVHEERFLHAKKRLYMTATPRIYADA
gi|251772067|gb|EES52637.1|putativehelicase[Leptospirillumferrodiazotrophum]/11618        -D--------------------------TDSAFVAVHRKEYITAKKRLYMTATPRIYTDS
gi|738124101|ref|WP_036082108.1|damageinducibleprotein[Leptospirillumferriphilum]_gi|685243306|gb|KGA94177.1|putativehelicase[/        -E--------------------------ADSAFVAVHRKDYILAHKRLYMTATPRIYTDS
gi|558699223|ref|WP_023524766.1|helicase[Leptospirillumsp.GroupII'CF1']_gi|206603764|gb|EDZ40244.1|Putativehelicase[Leptospiri/        -E--------------------------ADSAFVAVHRKDYIGAKKRLYMTATPRIYTDS
gi|406774462|gb|AFS53887.1|putativehelicase[LeptospirillumferriphilumML04]/11610        -E--------------------------ADSAFVAVHRLDYLLARKRLFMTATPRIYADS
gi|740719463|ref|WP_038504749.1|damageinducibleprotein[Leptospirillumferriphilum]_gi|640838125|gb|AIA30173.1|damageinduciblep/1        -E--------------------------SDSAFVAVHRPDYLLSKKRLFMTATPRIYADS
gi|517810766|ref|WP_018980974.1|hypotheticalprotein[Saccharibacterfloricola]/11733        ----------------------------DDPGMFQAVHDGAYLQAKHRLYMTATPRIYAGE
gi|502471675|ref|WP_012798533.1|helicase[Slackiaheliotrinireducens]_gi|256791761|gb|ACV22431.1|predictedhelicase[Slackiaheliotr        AA------------------------KEDISQYVKVHDNSIIIAKKRLYMTATPRIYGDN
gi|657871713|ref|WP_029576297.1|hypotheticalprotein[Bifidobacteriumthermacidophilum]_gi|673007521|gb|KFJ02535.1|helicase[Bifido        ------------------------------SAFQRVHDQEYLRSDKRLYMTATPRIYGED
gi|673004757|gb|KFI99791.1|DNAhelicaserestrictionenzymetypeIIIressubunit[Bifidobacteriumthermacidophilumsubsp.porcinum]/11682        ------------------------------SAFQRVHDQEYLRSDKRLYMTATPRIYGED
gi|917316092|ref|WP_051922804.1|hypotheticalprotein[Bifidobacteriumstellenboschense]_gi|673003774|gb|KFI98812.1|helicase[Bifido        -----------------------------AAFQKIHDAGFVHSEKRLYMTATPRIYGEG
gi|705415694|ref|WP_033502332.1|hypotheticalprotein[Bifidobacteriumpseudolongum]_gi|672982411|gb|KFI77685.1|DNAdamageinducible/        ------------------------------GAFQRVHDNDFIHGAKRLYMTATPRIYGEG
gi|916591418|ref|WP_051198509.1|hypotheticalprotein[Bifidobacteriumsp.AGR2158]/11704        -----------------------------VSFRKVHSNKNVRGDKRLYMTATPRIYGED
gi|772242982|gb|AJW78255.1|hypotheticalproteinVO01_03165[Clavibactermichiganensissubsp.insidiosus]/11642        ---------------------------AEESHFTRIHDDAYVAGSKRVYMTATPRIFAPD
gi|916649818|ref|WP_051256909.1|damageinducibleprotein[Actinomycesgraevenitzii]/11677        ---------------------------ANESAFTKIHDDAFIRRSKTLYMTATPRIFAEN
gi|496504578|ref|WP_009212861.1|helicase[Actinomycessp.oraltaxon180]_gi|315312695|gb|EFU60776.1|helicase[Actinomycessp.oraltaxo        ---------------------------EDESAFTKIHSDEFIRRAKTLYMTATPRIFADK
gi|494862206|ref|WP_007588306.1|helicase[Actinomycessp.ICM39]_gi|398362799|gb|EJN46470.1|typeIIIrestrictionenzyme,ressubunit[Ac        ---------------------------EDESAFTKIHSDEFIRRAKTLYMTATPRIFAEN
gi|881057111|ref|WP_048774974.1|damageinducibleprotein[Sanguibacterkeddieii]/11697        ---------------------------EDESAFTKIHSDEFIRRAKTLYMTATPRIFAEN
gi|881020459|ref|WP_048740808.1|damageinducibleprotein[Kytococcussedentarius]/11704        ---------------------------EDESAFTKIHSDEFIRRAKTLYMTATPRIFAEN
gi|881052313|ref|WP_048770555.1|damageinducibleprotein[Cellulomonasflavigena]/11705        ---------------------------EDESAFTKIHSDEFIRRAKTLYMTATPRIFAEN
gi|490108682|ref|WP_004009424.1|helicase[Mobiluncuscurtisii]_gi|315490465|gb|EFU80089.1|helicaseCterminaldomainprotein[Mobilun/        ---------------------------EDESAFTRVHDNAYVRAEKRLYMTATPRIFQPN
gi|490111935|ref|WP_004012603.1|helicase[Mobiluncusmulieris]_gi|227843812|gb|EEJ53982.1|helicaseCterminaldomainprotein[Mobilun/        ---------------------------EDESAFTRIHDNTYIRADKRLYMTATPRIFNPA
gi|490114228|ref|WP_004014848.1|helicase[Mobiluncusmulieris]_gi|269934432|gb|EEZ90993.1|helicaseCterminaldomainprotein[Mobilun/        ---------------------------EDESAFTRIHDNTYIRADKRLYMTATPRIFNPA
gi|493333551|ref|WP_006290636.1|helicase[Parascardoviadenticolens]_gi|315119487|gb|EFT82620.1|helicaseCterminaldomainprotein[P/        MR-------------------------QSDVSAFQIVNGDRIRASKRLYMTATPKIYGES
gi|495817067|ref|WP_008541646.1|helicase,partial[Sutterellaparvirubra]_gi|378598583|gb|EHY31712.1|helicaseprotein,partial[Sutte        ---------------------------DEDAPFQRIHDQNFIRGKKRLYMTATPRIFGGD
gi|491572696|ref|WP_005430278.1|helicase[Sutterellawadsworthensis]_gi|319805116|gb|EFW01943.1|DNAhelicaserestrictionenzymeTypeI        ---------------------------ESDAPFQRIHSDDFIKGKKRLYMTATPRIYGDL
```

```
gi|748686321|ref|WP_039944445.1|hypotheticalprotein[Thermicanusaegyptius]/11573                                                ----------------------------EESYYTKVHNNQNVNGKKRLYMTATPRIYVPK
gi|916875698|ref|WP_051482754.1|hypotheticalprotein[Thioalkalivibriosp.HK1]/11366                                              VK-----------------------DKTRVSPFVLVHDQERIRGVKRLYMTATPRIYTEA
gi|821248604|gb|KKZ10960.1|hypotheticalproteinTE42_09075[CandidatusSynechococcusspongiarumSP3]/11598                            ---------------------------EDPSPFVLIHDNTRLRAERRLYMTATPKVYAQS
gi|737409843|ref|WP_035390956.1|hypotheticalprotein[Ferrimicrobiumacidiphilum]_gi|765544266|gb|KJE76241.1|typeIrestrictionenzym   ----------------------------HDSAFVLVHDNERIKATKRIYMTATPKVYGDR
gi|654906925|ref|WP_028358281.1|damageinducibleprotein[Brackiellaoedipodis]/11679                                               DK---------------------RNSTQEESTFVRIHDNDYVRGCKRLYMTATPRVYSES
gi|502809901|ref|WP_013044877.1|hypotheticalprotein[CandidatusPuniceispirillummarinum]_gi|292663146|gb|ADE38247.1|hypotheticalp  LA------------------------KGKTSPFVRVHDGDYLKAAKRLYMTATPRIYTDH
gi|251771370|gb|EES51950.1|typeIIIrestrictionenzyme,ressubunit[Leptospirillumferrodiazotrophum]/11628                            EAKESVIARKKRKAENAGGSYTPQSEAGELSSFMMVHEDSYVQARKRLYMTATPRIFTEK
gi|498328480|ref|WP_010642636.1|DNAdamageinducibleprotein[Acidithiobacillusthiooxidans]/11627                                   ES-------------------------TKDSDFVKVHQNDLLHARKRLYMTATPRIYTEK
gi|647633864|ref|WP_025906698.1|damageinducibleprotein[Prochlorococcussp.scB245a_519B7]/11622                                   ---------------------------ENDSNFVKIHLNKYVKGKKRIYMTATPRIYGES
gi|647619599|ref|WP_025893729.1|damageinducibleprotein[Prochlorococcussp.scB241_527N11]/11621                                   ---------------------------EDESNFVKIHSNENIKGKKRLYMTATPKIYGET
gi|755034887|ref|WP_042387498.1|hypotheticalprotein[Clostridiumsp.CL6]/11606                                                    ---------------------------SDESYFVKVHDNNFIQADKRLYMTATPRIFGDN
gi|498139798|ref|WP_010453954.1|helicase[SuccinivibrionaceaebacteriumWG1]/11662                                                 -S-------------------------ADESYFTKVHDNNKIKALRRLYMTATPKLYGED
gi|527501386|gb|EPT34571.1|typeIIIrestrictionenzyme,ressubunit[Bacteroidetesbacteriumoraltaxon272str.F0290]/11789               ---------------------------KEESNFTKIHDNNFIQGRKRMYMTATPRYYNAN
gi|496047275|ref|WP_008771782.1|helicase[Bacteroidessp.2_1_33B]_gi|262296443|gb|EEY84373.1|helicaseCterminaldomainprotein[Bact/  ---------------------------RDESHFTKIHDNDLIRGKKRLYMTATPRYYNDN
PDI8503III/11664                                                                                                                ---------------------------RDESHFTKIHDNDLIRGKKRLYMTATPRYYNDN
gi|738719431|ref|WP_036613715.1|helicase[Parabacteroidesdistasonis]_gi|649542518|gb|KDS49387.1|restrictionendonucleasefamilypro  ---------------------------RDESHFTKIHNNDLIRGKKRLYMTATPRYYNDN
gi|659417033|gb|KEJ86133.1|hypotheticalproteinHMPREF1002_00341[Porphyromonassp.31_2]/11656                                     ---------------------------RDESHFTKIHDNDLIRGKKRLYMTATPRYYNDN
gi|567456807|gb|ETK11358.1|hypotheticalproteinT235_15845[Tannerellasp.oraltaxonBU063isolateCell8/11]/11433                      ---------------------------KDESSFTKIHNNDFIQGHKRLYMTATPRLYGES
gi|545440279|ref|WP_021677877.1|helicase[Porphyromonasgingivalis]_gi|543985765|gb|ERJ89108.1|helicaseprotein[Porphyromonasgingi  ---------------------------KDESNFTKVHNNDFIQGHKRLYMTATPRLYGES
gi|497438056|ref|WP_009752254.1|helicase[Prevotellasp.oraltaxon473]_gi|429144866|gb|EKX87968.1|helicaseprotein[Prevotellasp.ora  ---------------------------QDESNFIKIHNDDFIRGKKRLYMTATPRLYTDD
gi|490423474|ref|WP_004295683.1|MULTISPECIES:helicase[Bacteroides]_gi|156110098|gb|EDO11843.1|helicaseCterminaldomainprotein[B/  ---------------------------RDESNFTKIHDAEYIKGNKRLYMTATPRLYGQS
gi|941777229|ref|WP_055173506.1|helicase[Bacteroidescaccae]_gi|933263395|emb|CUQ00567.1|Predictedhelicase[Bacteroidescaccae]/1/  ---------------------------KDESNFIKIHSNENVQGRKRLYMTATPRLYGES
gi|492413898|ref|WP_005836892.1|helicase[Bacteroidesuniformis]_gi|392678824|gb|EIY72229.1|hypotheticalproteinHMPREF1072_03583[B  ---------------------------KDESNFIKIHSNENVQGQKRLYMTATPRLYGES
gi|695455095|ref|WP_032566191.1|helicase[Bacteroidesfragilis]_gi|596095426|gb|EXZ59996.1|restrictionendonucleasefamilyprotein[B  ---------------------------KDESNFIKIHSNENVQGRKRLYMTATPRLYGES
gi|933227941|emb|CUQ02348.1|Predictedhelicase[Bacteroidesuniformis]/11658                                                      ---------------------------SDESNFIKIHSNYNVQGRKRLYMTATPRLYGES
gi|494823013|ref|WP_007558421.1|helicase[Bacteroidesplebeius]_gi|198272167|gb|EDY96436.1|helicaseCterminaldomainprotein[Bacter/  ---------------------------KDESNFIKIHSNENVQGRKRLYMTATPRLYGES
gi|495427212|ref|WP_008151909.1|helicase[Parabacteroidesjohnsonii]_gi|218222381|gb|EEC95031.1|helicaseCterminaldomainprotein[P/  ---------------------------KDESNFTKIHSDDNVQGRKRLYMTATPRLYGES
gi|933196855|emb|CUO94545.1|typeIrestrictionenzymeEcoKIsubunitR[Bacteroidesuniformis]_gi|933220098|emb|CUN86874.1|typeIrestrict  ---------------------------KDESNFTKIHSDDNVKGRKRLYMTATPRLYGES
gi|823687748|ref|WP_047101121.1|helicase[Brachyspirahyodysenteriae]_gi|823334537|gb|KLI17932.1|helicase[Brachyspirahyodysenteri  ---------------------------EDESSFIKVHNNDFIKSKKRLYMTATPRIYSDD
gi|496436691|ref|WP_009145538.1|helicase[Phascolarctobacteriumsuccinatutens]_gi|322414007|gb|EFY04839.1|helicaseCterminaldomai/  ---------------------------KDESAFTKVHNNSNVAGKHRLYMTATPRLYADN
gi|503372880|ref|WP_013607541.1|helicase[Sphaerochaetaglobosa]_gi|324026933|gb|ADY13692.1|typeIIIrestrictionproteinressubunit[S  ---------------------------EEESAFVRVHDPDFIKANKRLYMTATPRLYSED
gi|505821748|ref|WP_015707145.1|helicase[Treponemaprimitia]_gi|299483503|gb|ADJ19584.1|helicasedomaincontainingprotein[Trepone/  ---------------------------QDESAFVKVHDDKFLKSKKRIYMTATPRLYSES
gi|505822741|ref|WP_015708134.1|helicase[Treponemaprimitia]_gi|333739639|gb|AEF85129.1|endonucleaseandmethylaseLlaGI[Treponemap  ---------------------------QDESAFVKVHDDKFLKSKKRIYMTATPRLYSES
gi|488791994|ref|WP_002704400.1|helicase[Treponemasaccharophilum]_gi|380767691|gb|EIC01689.1|typeIIIrestrictionproteinressubuni  ---------------------------REDSAFVKVHSDSNVKAKKRLYMTATPKLYDTN
gi|524671454|emb|CDD81750.1|putativeseptumsitedeterminingproteinMinC[Bacteroidessp.CAG:462]/11642                              ---------------------------TEESAFIKVHDNDFIQAKRRLYMTATPRLYTDE
gi|524573865|emb|CDC95766.1|putativeseptumsitedeterminingproteinMinC[Alistipessp.CAG:268]/11639                                 ---------------------------ETESAFVRVHNNDFLRAVRRIYMTATPRLYTDE
gi|658617970|ref|WP_029759328.1|helicase[Fusobacteriumnucleatum]/11606                                                          ---------------------------EDESNFVKVHKNDFLKAKKRLYMTATPRLYDDN
gi|494412037|ref|WP_007216782.1|helicase[Bacteroidescellulosilyticus]_gi|392638921|gb|EIY32752.1|hypotheticalproteinHMPREF1062_  ---------------------------AEESAFVRVHDSRFLCAKKRLYMTATPRLYSDT
gi|383107102|gb|AFG37435.1|putativehelicase[SpirochaetaafricanaDSM8902]/11616                                                   ---------------------------DDQSSFIKVHDDDFLKAKKRLYMTATPRIYSDD
SAF8902III/11616                                                                                                                ---------------------------DDQSSFIKVHDDDFLKAKKRLYMTATPRIYSDD
gi|652538894|ref|WP_026932796.1|helicase[Gramellaechinicola]/11633                                                              ---------------------------EDSSHFIKVHDNEFLEGKKRLYMTATPRLYSDD
gi|639238223|ref|WP_024568839.1|helicase[Elizabethkingiaanophelis]/11628                                                        ---------------------------EDSSSFIKVHDNDFIKAQKRLYMTATPRLYSED
gi|652520381|ref|WP_026914582.1|helicase[Gramellaportivictoriae]/11623                                                          ---------------------------NDESAFVKVHNNEFIKASKRLYMTATPRLYSDD
gi|494922183|ref|WP_007648219.1|Helicase[Formosasp.AK20]_gi|469825489|gb|EMQ95550.1|Helicasedomainprotein[Formosasp.AK20]/11626  ---------------------------NDESAFVKVHDNEFLQANKRLYMTATPRLFSDD
gi|522083382|ref|WP_020594591.1|hypotheticalprotein[Spirosomapanaciterrae]/11637                                                ---------------------------EDESAFTKVHNNDFIKAKKRLYMTATPRLYDDT
gi|490455352|ref|WP_004326157.1|helicase[Bacteroidesovatus]_gi|392669277|gb|EIY62768.1|hypotheticalproteinHMPREF1070_03635[Bact  ---------------------------EDESAFVKVHNNDFIEAKKRLYMTATPRLYSDD
gi|499516035|ref|WP_011202675.1|helicase[Bacteroidesfragilis]_gi|53713270|ref|YP_099262.1|helicasedomainprotein[Bacteroidesfrag  ---------------------------EDESAFVKVHNNDFIEAKKRLYMTATPRLYSDD
gi|651338332|ref|WP_026461320.1|helicase[Adhaeribacteraquaticus]/11620                                                          ---------------------------EDESAFTKVHDNSFLQAKKRLYMTATPRLYSDD
gi|806964642|gb|AKC17139.1|helicase[Flavobacteriumpsychrophilum]/11639                                                          ---------------------------EDESAFTKVHNNDFLKAKKRLYMTATPRLYDQD
gi|752684374|ref|WP_041332322.1|helicase[Flavobacteriumpsychrophilum]_gi|666882889|gb|AIG30715.1|helicase[Flavobacteriumpsychro  ---------------------------EDESAFTKVHNNDFLKAKKRLYMTATPRLYDQD
gi|933196595|emb|CUP04894.1|DNAorRNAhelicasesofsuperfamilyII[Bacteroidesuniformis]/11631                                       ---------------------------EDESAFIKVHKNDFIKAKKRLYMTATPRLYSDD
gi|670509109|ref|WP_031456918.1|helicase[Flavobacteriumchungangense]/11625                                                      ---------------------------FDESAFTKVHDNDFIKAKKRLYMTATPRLYNDE
                                                                                                                                                                :   .    :    :: :***   :

gi|490190857|ref|WP_004089451.1|hypotheticalprotein[Helicobacterbilis]_gi|476630638|gb|EMZ37196.1|hypotheticalproteinC826_02193  QKAKASEKDNE-VFSMDDEAIFGKTIYEIRFDKAVKEGLLTDYKVIILTLKKETYAQVAN
gi|488896133|ref|WP_002807242.1|MULTISPECIES:restrictionendonucleaseEco57I[Campylobacter]_gi|380541096|gb|EIA65377.1|hypothetic  QKSKALENDNE-VFSMDDEEIFGNEIYSINFREAVEKDLLTDYKVIILAIKQEGIANIAN
gi|746602240|ref|WP_039628365.1|restrictionendonucleaseEco57I[Campylobacterlari]_gi|744819395|gb|AJD03391.1|putativehelicase[Ca  QKSKALESDNE-VFSMDDEEIFGDEIYSINFREAVEKGLLTDYKVIILAIKQEGIANIAN
gi|727317135|ref|WP_033772558.1|restrictionendonucleaseEco57I[Helicobacterpylori]/11622                                        SQTKAKESGNL-VYSMDDAEIFGEEIYTLNFEKAIALDLLTDYKVIILAVRSENLSGVTN
gi|727103490|ref|WP_033618954.1|restrictionendonucleaseEco57I[Helicobacterpylori]/11616                                        SQTKAKESGNL-VYSMDDAEIFGEEIYTLNFEKAIALDLLTDYKVIILVVRSENLSGVTN
gi|727340205|ref|WP_033794408.1|restrictionendonucleaseEco57I[Helicobacterpylori]/11609                                        SQTKAKESGNL-VYSMDDAEIFGEEIYTLNFEKAIALDLLTDYKVIILAVRSENLSGVTN
gi|727292407|ref|WP_033749975.1|restrictionendonucleaseEco57I[Helicobacterpylori]/11611                                        SQTKAKESGNL-VYSMDDADIFGEEIYTLNFEKAIALDLLTDYKVIILAVRSENLSGVTN
gi|727289795|ref|WP_033747591.1|restrictionendonucleaseEco57I[Helicobacterpylori]/11612                                        SKAKAKEKDNV-IYSMDDAEIFGEEIYTLNFEKAIALDLLTDYKVIILAVRSENLSGVTN
gi|727319676|ref|WP_033775047.1|restrictionendonucleaseEco57I[Helicobacterpylori]/11612                                        SQTKAKESGNL-VYSMDDAQIFGEEIYTLNFEKAIALDLLTDYKVIILAVRSENLSGVTN
gi|447212910|ref|WP_001290166.1|restrictionendonucleaseEco57I[Helicobacterpylori]_gi|308063540|gb|ADO05427.1|hypotheticalprotei  SKAKAKESDNA-IYSMDDAEIFGEEIYTLNFERAIALDLLTDYKVMILAVRKENLSGVTN
gi|447212901|ref|WP_001290157.1|restrictionendonucleaseEco57I[Helicobacterpylori]_gi|384557329|gb|AFH97797.1|hypotheticalprotei  SKAKAKESDNA-IYSMDDAQIFGEEIYTLNFERAIALDLLTDYKVMILAVRKENLSGVTN
gi|515849671|ref|WP_017280424.1|hypotheticalprotein[Helicobacterpylori]/11615                                                   SKARAKESDNA-IYSMDDEGIFGEEIYTLNFTRAIALDLLTDYKVMILAVRKENLSGVTN
gi|447212900|ref|WP_001290156.1|restrictionendonucleaseEco57I[Helicobacterpylori]_gi|317178970|dbj|BAJ56758.1|TypeIIGrestrictio  SKARAKESDNA-IYSMDDAEIFGEEIYTLNFERAIALDLLTDYKVMILAVRKENLSGVTN
```

```
gi|447079018|ref|WP_001156274.1|hypotheticalprotein[Helicobacterpylori]/11614    SKAKAKESDNV-IYSMDDEEIFGEEIYTLNFERAIALDLLTDYKVIILAVRKENLSGVTN
gi|447212800|ref|WP_001290056.1|hypotheticalprotein[Helicobacterpylori]_gi|532153248|gb|EQL67316.1|restrictionendonucleaseEco57    SKAKAKEKDNV-IYSMDDEEIFGQEIYTLNFERAIALDLLTDYKVIILAVRSKNLSGVTN
gi|505457237|ref|WP_015644339.1|hypotheticalprotein[Helicobacterpylori]_gi|499061160|gb|AGL70074.1|restrictionendonucleaseEco57    SKAKAKESDNV-IYSMDDEEIFGGEIYTLNFERAIALDLLTDYKVIILAVRKENLSGVTN
HPYUM037X/11591    SKAKAKESDNV-IYSMDDEEIFGGEIYTLNFERAIALDLLTDYKVIILAVRKENLSGVTN
gi|920026297|ref|WP_052937176.1|restrictionendonuclease[Helicobacterpylori]_gi|912528678|gb|KNX48238.1|restrictionendonucleaseE    SKAKAKEKDNV-IYSMDDEEIFGGEIYTLNFSKAIALDLLTDYKVIILAVRKENLSGVTN
gi|915539050|ref|WP_050841327.1|restrictionendonuclease[Helicobacterpylori]_gi|908890887|gb|KNE06183.1|restrictionendonucleaseE    SKAKAKEKDNV-IYSMDDEEIFGEEIYTLNFSKAIALDLLTDYKVIILAVRKENLSGVTN
gi|727101397|ref|WP_033616957.1|restrictionendonucleaseEco57I[Helicobacterpylori]/11592    SKAKAKESDNV-IYSMDDEEIFGEEIYTLNFSKAIALDLLTDYKVIILAVRKENLSGVTN
gi|562814875|emb|CDK13002.1|TypeISPRMsystem[Helicobacterpylori26695]/11588    SKAKAKESDNV-IYSMDDAEIFGEEIYTLNFSKAIALDLLTDYKVIILAVRKENLSGVTN
HPYAXVIII/11588    SKAKAKESDNV-IYSMDDAEIFGEEIYTLNFSKAIALDLLTDYKVIILAVRKENLSGVTN
gi|447079029|ref|WP_001156285.1|restrictionendonucleaseEco57I[Helicobacterpylori]_gi|393085956|gb|EJB86635.1|hypotheticalprotei    SKAKAKEKDNV-IYSMDDEEIFGEEIYTLNFSKAIALDLLTDYKVIILAVRKENLSGVTN
gi|489105953|ref|WP_003015814.1|restrictionendonuclease[Helicobacterpylori]_gi|477503388|gb|ENH59950.1|restrictionendonucleasef    SKAKAKGSDNV-IYSMDDGEIFGEEIYTLNFSKAIALDLLTDYKVIILAVRKENLSGVTN
gi|447079006|ref|WP_001156262.1|restrictionendonucleaseEco57I[Helicobacterpylori]_gi|393067470|gb|EJB68280.1|hypotheticalprotei    SKAKAKESDNV-IYSMDDAEVFGEEIYTLNFSQAIALDLLTDYKVIILAVRKENLSGVTN
gi|727314589|ref|WP_033770298.1|restrictionendonucleaseEco57I[Helicobacterpylori]/11586    SKAKAKESDNV-IYSMDDEEIFGEEIYTLNFSQAIALDLLTDYKVIILAVRKENLSGVTN
gi|447079019|ref|WP_001156275.1|restrictionendonucleaseEco57I[Helicobacterpylori]_gi|308065028|gb|ADO06920.1|hypotheticalprotei    SKAKAKESDNV-IYSMDDADTFGEEIYTLNFEKAIALDLLTDYKVIILAVRSENLSGVTN
gi|447079028|ref|WP_001156284.1|restrictionendonucleaseEco57I[Helicobacterpylori]_gi|393076030|gb|EJB76784.1|hypotheticalprotei    SKAKAKESDNV-IYSMDDAKVFGEEIYTLNFERAIALDLLTDYKVIILAVRSENLSGVTN
gi|393034009|gb|EJB35070.1|hypotheticalproteinHPNQ4053_0631[HelicobacterpyloriNQ4053]/11514    SKAKAKEKDNV-IYSMDDAQTFGEEIYTLNFSKAIALDLLTDYKVIILAVRKENLSGVTN
gi|447079004|ref|WP_001156260.1|restrictionendonucleaseEco57I[Helicobacterpylori]_gi|384552418|gb|AFI07366.1|hypotheticalprotei    SKAKAKESDNV-IYSMDDVQTFGEEIYTLNFERAIALDLLTDYKVIILAVRSENLSGVTN
gi|746011306|ref|WP_039088215.1|restrictionendonucleaseEco57I[Helicobacterpylori]_gi|733488720|gb|KHL85979.1|restrictionendonuc    SKAKAKESDNV-IYSMDDEEIFGEEIYTLNFERAIALDLLTDYKVIILAVRSENLSGVTN
gi|531081162|gb|EQD92166.1|restrictionendonucleaseEco57I[HelicobacterpyloriPZ5080]/11509    SKAKAKESDNV-IYSMDDEEVFGEEIYTLNFERAIALDLLTDYKVIILAVRSKNLSGVTN
gi|544760796|ref|WP_021187395.1|restrictionendonucleaseEco57I[Helicobacterpylori]_gi|531086508|gb|EQD96745.1|restrictionendonuc    SKAKAKESDNV-IYSMDDEEVFGEEIYTLNFSKAIALDLLTDYKVIILAVRSENLSGVTN
gi|447079012|ref|WP_001156268.1|restrictionendonucleaseEco57I[Helicobacterpylori]_gi|393028199|gb|EJB29286.1|hypotheticalprotei    SKAKAKESDNV-IYSMDDAEIFGEEIHTLNFSKAIALDLLTDYKVIILAVRKENLSGVTN
gi|746008458|ref|WP_039085555.1|restrictionendonucleaseEco57I[Helicobacterpylori]_gi|733480358|gb|KHL78026.1|restrictionendonuc    SKAKAKESDNI-IYSMDDADTFGEEIYTLNFERAIALDLLTDYKVIILAVRSENLSGVTN
gi|447079011|ref|WP_001156267.1|restrictionendonucleaseEco57I[Helicobacterpylori]_gi|393038534|gb|EJB39568.1|hypotheticalprotei    SKAKAKEKDNV-IYSMDDAKTFGEEIYTLNFERAIALDLLTDYKVIILAVRSENLSSVTN
gi|393037163|gb|EJB38199.1|hypotheticalproteinHPNQ4161_0422[HelicobacterpyloriNQ4161]/11556    SKAKAKEKDNV-IYSMDDADIFGEEIYTLNFERAIALDLLTDYKVIILAVRSENLSGVTN
gi|447079007|ref|WP_001156263.1|restrictionendonucleaseEco57I[Helicobacterpylori]_gi|393051817|gb|EJB52768.1|hypotheticalprotei    SKTKAKEKDNV-IYSMDDEEIFGEEIYTLNFSKAIALDLLTDYKVIILAVRKENLSGVTN
gi|487867094|ref|WP_001940560.1|helicase[Helicobacterpylori]_gi|459505657|gb|EMG98418.1|helicaseprotein[HelicobacterpyloriGAM21    SKARAKESDNV-IYSMDDAQTFGEEIYTLNFERAIALDLLTDYKVLILAVRSENLSGVTN
gi|487872094|ref|WP_001945560.1|helicase[Helicobacterpylori]_gi|459512104|gb|EMH04523.1|helicaseprotein[HelicobacterpyloriGAM24    SKAKAKESDNV-IYSMDDAETFGEEIYTLNFERAIALDLLTDYKVIILAVRSENLSGVTN
gi|487880228|ref|WP_001953694.1|helicase[Helicobacterpylori]_gi|459529131|gb|EMH20734.1|helicaseprotein[HelicobacterpyloriGAM26    SKAKAKESDNV-IYSMDDAKVFGDEIYTLNFERAIALDLLTDYKVLILAVRSENLSGVTN
gi|487882593|ref|WP_001956059.1|helicase[Helicobacterpylori]_gi|459533462|gb|EMH24859.1|helicaseprotein[HelicobacterpyloriGAM26    SKAKAKESDNV-IYSMDDAKVFGDEIYTLNFERAIALDLLTDYKVIILAVRSENLSGVTN
gi|487845430|ref|WP_001918896.1|helicase[Helicobacterpylori]_gi|410713871|gb|EKQ71363.1|helicaseprotein[HelicobacterpyloriGAM10    SKAKAKESDNV-IYSMDDTETFGEEIYTLNFERAIALDLLTDYKVIILAVRSENLSGVTN
gi|447079005|ref|WP_001156261.1|restrictionendonucleaseEco57I[Helicobacterpylori]_gi|393069830|gb|EJB70625.1|hypotheticalprotei    SKAKAKEKDNV-IYSMDDADTFGEEIYTLNFERAIALDLLTDYKVIILAVRSENLSGVTN
gi|459506808|gb|EMG99528.1|helicaseprotein[HelicobacterpyloriGAM121Aii]/11545    SKAKAKESDNV-IYSMDDAKVFGDEIYTLNFERAIALDLLTDYKVLILAVRSENLSGVTN
gi|487825283|ref|WP_001898749.1|restrictionendonucleaseEco57I[Helicobacterpylori]_gi|393107526|gb|EJC08068.1|hypotheticalprotei    SKAKAKESDNV-IYSMDDAEVFGEEIYTLNFERAIALDLLTDYKVIILAVRSENLSGVTN
gi|447079008|ref|WP_001156264.1|restrictionendonucleaseEco57I[Helicobacterpylori]_gi|393058636|gb|EJB59524.1|hypotheticalprotei    SKAKAKEKDNV-IYSMDDAQTFGEEIYTLNFERAIALDLLTDYKVIILAVRSENLSGVTN
gi|447079015|ref|WP_001156271.1|restrictionendonucleaseEco57I[Helicobacterpylori]_gi|307637357|gb|ADN79807.1|hypotheticalprotei    SKAKAKEKDNV-IYSMDDAQTFGEEIYTLNFERAIALDLLTDYKVIILAVRSENLSGVTN
gi|487884861|ref|WP_001958327.1|helicase[Helicobacterpylori]_gi|459535535|gb|EMH26834.1|helicaseprotein[HelicobacterpyloriGAM26    SKAKAKESDNV-IYSMDDTKVFGDEIYTLNFERAIALDLLTDYKVIILAVRSENLSGVTN
gi|487886365|ref|WP_001959831.1|helicase[Helicobacterpylori]_gi|459538727|gb|EMH29861.1|helicaseprotein[HelicobacterpyloriGAM27    SKAKAKEKDNV-IYSMDDAQTFGEEIYTLNFERAIALDLLTDYKVLILAVRSENLSGVTN
gi|545301637|ref|WP_021581412.1|helicase[Helicobacterpylori]_gi|535931889|gb|ERA61745.1|helicaseprotein[HelicobacterpyloriGAM11    SKAKAKESDNV-IYSMDDADIFGEEIYTLNFERAIALDLLTDYKVLILAVRSENLSGVTN
gi|447079010|ref|WP_001156266.1|restrictionendonucleaseEco57I[Helicobacterpylori]_gi|393095477|gb|EJB96081.1|hypotheticalprotei    SKAKAKEKDNV-IYSMDDAETFGEEIYTLNFERAIALDLLTDYKVIILAVRSENLSGVTN
gi|447079009|ref|WP_001156265.1|restrictionendonucleaseEco57I[Helicobacterpylori]_gi|393056294|gb|EJB57206.1|hypotheticalprotei    SKAKAKEKDNV-IYSMDDAQTFGEEIYTLNFERAIALDLLTDYKVIILAVRSENLSGVTN
gi|471386985|gb|EMR58906.1|restrictionendonucleasefamilyprotein[HelicobacterpyloriHpH1]/11504    SKAKAKEKDNV-IYSMDDAQTFGEEIYTLNFERAIALDLLTDYKVIILAVRSENLSGVTN
gi|487875679|ref|WP_001949145.1|helicase[Helicobacterpylori]_gi|459516928|gb|EMH09091.1|helicaseprotein[HelicobacterpyloriGAM25    SKAKAKESDNV-IYSMDDAEVFGEEIYTLNFERAIALDLLTDYKVIILAVRSENLSGVTN
gi|496130066|ref|WP_008854573.1|DNAdamageinducibleprotein[Commensalibacterintestini]_gi|353671689|gb|EHD13391.1|helicasedomain/    SKKKAKDDNIV-VYSMDDVPTYGQELYVITFSQAVARDLLVDYKVIVLAVEESH---INR
gi|924898894|ref|WP_053556546.1|damageinducibleprotein[Sphingopyxissp.113P3]_gi|924523996|gb|ALC14732.1|damageinducibleprotei/1    VRDVARDENAA-LVSMDDPALYGETLFTRSFGWAVQNDLLTDYKVIVLAINEAD---VAA
gi|780802950|gb|KJS08562.1|damageinducibleprotein[Hoefleasp.BRH_c9]/11633    VKTKAAESSVE-LCSMDDPDLYGEVFFQRGFSWAVQNGLLTDYKVIVLAVDEAV---VSA
gi|944671432|gb|KQN22886.1|damageinducibleprotein[Sphingomonassp.Leaf32]/11415    VRSKAKEVSAE-LCSMDDPALYGDTLFQRGFGWAVQNDLLTDYKVIVLAVDEAM---VST
gi|944612953|gb|KQM64772.1|damageinducibleprotein[Sphingomonassp.Leaf16]/11726    VRSKAKEVSAE-LCSMDDPALYGDTLFQRGFGWAVQNDLLTDYKVIVLAVDEAM---VST
gi|764353851|ref|WP_044331468.1|damageinducibleprotein[Sphingomonashengshuiensis]_gi|761897338|gb|AJP71707.1|damageinduciblep/1    VKTKANEASAV-LCSMDDESLYGETLFARGFGWAVENGLLTDYKVLVLAVDEGM---VSG
gi|945874472|gb|KQZ27973.1|damageinducibleprotein[Mesorhizobiumsp.Root552]/11651    IKSKANEASAV-LCSMDDEALYGKVLFARGFGWAVENGLLTDYKVLVLAVDEQM---VSG
gi|759408081|ref|WP_043132403.1|damageinducibleprotein[Paracoccussanguinis]_gi|694217086|gb|KGJ14281.1|damageinducibleprotein/1    ARRKADDHDAE-LASMDDEDKFGKDLFHRGFGWAVENNLLTDYKVVVLAVDEGL---IST
gi|654342671|ref|WP_027836116.1|damageinducibleprotein[Maritaleamyrionectae]/11632    AKRKADDHDAE-LASMDDESKFGKDLFHRGFGWAVERELLTDYKVVVLAVDEGL---VSS
gi|126717687|gb|EBA14409.1|hypotheticalproteinRSK20926_02032[Roseobactersp.SK20926]/11603    AKRKADDHDAK-LASMDDEEKFGKDLFHRGFGWAVENELLTDYKVVVLAVDEGL---ISQ
gi|765301116|ref|WP_044628780.1|damageinducibleprotein[Halomonasmeridiana]_gi|764089679|gb|KJD19875.1|damageinducibleprotein[/1    ARRKADDHDAE-LASMDDEAKFGKDLFHRGFGWAVENEQLTDYKVVVLAVDEGL---VST
gi|518053006|ref|WP_019223214.1|DNAdamageinducibleprotein[Bartonellarattaustraliani]/11654    AKRRADGMNAV-LASMDDETIYGKQLHHYTFTEAIENGLLTPYKIVVLGVNENF---LTP
gi|493637806|ref|WP_006589578.1|DNAdamageinducibleprotein[Bartonellabirtlesii]_gi|395410345|gb|EJF76900.1|hypotheticalproteinM/    AKRRADEINAV-LASMDDEALYGKQLHHYSFAEAVDNELLTPYKVVVLGIDETY---ITP
gi|501188603|ref|WP_012231622.1|DNAdamageinducibleprotein[Bartonellatribocorum]_gi|161017843|emb|CAK01401.1|helicase/methyltra/    AKRRADEINAV-LASMDDETLFGKQLHHYTFAEAVDNELLTPYKIVVLGINETY---ITP
gi|651388616|ref|WP_026500615.1|damageinducibleprotein[Bartonellagrahamii]/11655    AKRRADEINAV-LASMDDETLYGKQLHHYTFAEAVDNELLTPYKIVVLGINETY---ITP
gi|501188669|ref|WP_012231687.1|DNAdamageinducibleprotein[Bartonellatribocorum]_gi|161017922|emb|CAK01480.1|helicase/methyltra/    AKRRADEINAV-LASMDDETLFGKQLHHYTFAEAVDNELLTPYKIVVLGINETY---ITP
gi|518048832|ref|WP_019219040.1|DNAdamageinducibleprotein[Bartonellaflorencae]/11655    LKKKADVSDAV-LASMDDKELYGKNLYTYTFSKALKDKLLTPYKVIVLAVDEAI---INA
gi|518053617|ref|WP_019223825.1|DNAdamageinducibleprotein[Bartonellarattaustraliani]/11607    LKKKADVSDAV-LASMDDEKIYGKNLYTYTFSKALKDKLLTPYKVIVLAVDEAI---INA
gi|506336449|ref|WP_015856168.1|DNAdamageinducibleprotein[Bartonellagrahamii]_gi|240267492|gb|ACS51080.1|helicase/methyltransf/    LKKKADVSDAV-LASMDDKELYGKNLYTYTFSKALKDKLLTPYKVIVLAVDEAI---INA
gi|494588832|ref|WP_007347427.1|DNAdamageinducibleprotein[Bartonellarattimassiliensis]_gi|395419053|gb|EJF85361.1|hypothetical/    LKKKADASDAV-LASMDDEELYGKNLYTYTFSKALKDKLLTPYKVIVLAVDEAI---INA
gi|501188650|ref|WP_012231668.1|DNAdamageinducibleprotein[Bartonellatribocorum]_gi|161017902|emb|CAK01460.1|helicase/methyltra/    LKKKADASDAV-LASMDDEKLYGKNLYTYTFSKALKDKLLTPYKVIVLAVDEAI---INA
gi|501188624|ref|WP_012231643.1|DNAdamageinducibleprotein[Bartonellatribocorum]_gi|161017866|emb|CAK01424.1|helicase/methyltra/    AKRRADEVDAV-LASMDNETLYGKVLYTYSFTDAVKNELLTPYKIIVLGVDEGK---ISK
gi|518053856|ref|WP_019224064.1|DNAdamageinducibleprotein[Bartonellarattaustraliani]/11625    AKRRADEVDAV-LASMDNETIYGKVLYTYSFTDAVKNELLTPYKIIVLGVDEGE---ISK
gi|501188053|ref|WP_012231081.1|DNAdamageinducibleprotein[Bartonellatribocorum]_gi|161017434|emb|CAK00992.1|helicase/methyltra/    AKRRADEVDAV-LASMDNEAIYGKALYTYSFTDAVKNELLTPYKIIVLGVDEGK---ISE
gi|651388630|ref|WP_026500626.1|damageinducibleprotein[Bartonellagrahamii]/11657    LKRNAVLSNNV-LVSMDNEKLYGKQLYTYNFSRALEDKLLSPCKVIILVVNEKE---VSQ
```

```
gi|518048855|ref|WP_019219063.1|DNAdamageinducibleprotein[Bartonellaflorencae]/11658    LKRNAVLSNNV-LVSMDNEKLYGKQLYTYNFSRALEDKLLSPCKVIILVVNEEE---VSQ
gi|490995101|ref|WP_004856829.1|DNAdamageinducibleprotein[Bartonelladoshiae]_gi|395413167|gb|EJF79645.1|hypotheticalproteinMCS/    LKRNAVLSNNV-LVSMDNEKLYGKQLYTYNFSRALEDKLLSPCKVIILVVNEGE---VSQ
gi|491002558|ref|WP_004864279.1|DNAdamageinducibleprotein[Bartonellavinsonii]_gi|395421392|gb|EJF87643.1|hypotheticalproteinME/    AKKQANEINAV-LASMDDEDLYGKTLYTYTFSKAVQNELLAPYKIIVLGVNEEA---VAQ
gi|491003373|ref|WP_004865094.1|DNAdamageinducibleprotein[Bartonellavinsonii]_gi|395432864|gb|EJF98838.1|hypotheticalproteinME/    AKKKANEINAV-LASMDDEDLYGKTLYTYTFSKAVQNELLAPYKIIVLGVNEEA---VAQ
gi|491002426|ref|WP_004864148.1|DNAdamageinducibleprotein[Bartonellavinsonii]_gi|395421767|gb|EJF88003.1|hypotheticalproteinME/    AKKQANEINAV-LASMDDEELYGKNLYTYTFSKAVQNELLAPYKIIVLGVNEEA---VAQ
gi|491004024|ref|WP_004865745.1|DNAdamageinducibleprotein[Bartonellavinsonii]_gi|395432542|gb|EJF98522.1|hypotheticalproteinME/    AKKQANEINAV-LASMDDEELYGKNLYTYTFSKAVQNDLLAPYKIIVLGVNEEA---VAQ
gi|648396442|ref|WP_026088193.1|damageinducibleprotein[Bartonellarattaustraliani]/11593    AKRRANEINAV-LASMDDEELYGKELYTYSFSKAVKNELLSPYKVIVLGVDEEE---VSQ
gi|748190174|ref|WP_039761290.1|damageinducibleprotein[Bartonellaqueenslandensis]/11658    AKRRADEINTV-LASMDDEELYGKDLYTYSFSKAVKNEQLSPYKVIVLGVDEEE---VSQ
gi|494817599|ref|WP_007553007.1|DNAdamageinducibleprotein[Bartonellasp.DB56]_gi|395411312|gb|EJF77837.1|hypotheticalproteinMC/1    AKKQANEIDAV-LASMDDEELYGKILYSYSFSNAVKNGLLTPYKIIVLGVDEGE---VSK
gi|515763376|ref|WP_017195976.1|DNAdamageinducibleprotein[Bartonellabirtlesii]/11656    AKKQANEIDAV-LASMDDEELYGKILYSYSFSHAVKNGLLTPYKIIVLGVDEGE---VSK
gi|494589420|ref|WP_007348014.1|DNAdamageinducibleprotein[Bartonellarattimassiliensis]_gi|395416278|gb|EJF82667.1|hypothetical/    AKKKANEINAV-LASMDDEELYGKELYIYTFSKAVQNELLAPYKIIVLGVNEEE---VSA
gi|506336469|ref|WP_015856188.1|DNAdamageinducibleprotein[Bartonellagrahamii]_gi|240267512|gb|ACS51100.1|helicase/methyltransf/    AKKKADEINAV-LASMDDEELYGKELYTYTFSKAVQNELLAPYKIIVLGVNEEE---VSQ
gi|749308087|ref|WP_040132227.1|damageinducibleprotein[Bartonellatribocorum]_gi|605051878|emb|CDO49988.1|helicase/methyltransf/    AKKNADEINAV-LASMDDEELYGKELYTYTFSKAVQNELLAPYKIIVLGINEEE---VSE
gi|515763800|ref|WP_017196400.1|DNAdamageinducibleprotein[Bartonellabirtlesii]/11658    AKKQAHEMNGI-LASMDDEALYGKNLYTYTFSKAVKNELLVPYKIIILGVNEEE---VSE
gi|492186994|ref|WP_005773380.1|DNAdamageinducibleprotein[Bartonellaelizabethae]_gi|395418361|gb|EJF84688.1|hypotheticalprotei/    AKKQAHEMNGI-LASMDDEALYGKELYTYTFSKAVKNELLVPYKIIVLGVNEEE---VSE
gi|501188615|ref|WP_012231634.1|DNAdamageinducibleprotein[Bartonellatribocorum]_gi|161017856|emb|CAK01414.1|helicase/methyltra/    AKKQAHEMNGI-LASMDDEALYGKNLYTYTFSKAVKNELLVPYKIIILGVNEEE---VSE
gi|501187470|ref|WP_012230499.1|DNAdamageinducibleprotein[Bartonellatribocorum]_gi|161017090|emb|CAK00648.1|helicase/methyltra/    AKKQAHEMNGI-LASMDDEALYGKNLYTYTFSKAVKNELLVPYKIIILGVNEEE---VSE
gi|501188636|ref|WP_012231655.1|DNAdamageinducibleprotein[Bartonellatribocorum]_gi|161017879|emb|CAK01437.1|helicase/methyltra/    AKKQAHEMNGI-LASMDDEALYGKNLYTYTFSKAVKNELLVPYKIIILGVNEEE---VSE
gi|930151941|emb|CTQ34818.1|UvsWhelicase[Jannaschiarubra]/11641    ARQRAKEQSLT-LAQMDDPATYGETLFERGFSWAVEKGLLVDYKVIVLAVDEGK---VAR
gi|928977106|ref|WP_054001461.1|damageinducibleprotein[Shimiasp.SK013]_gi|927698852|gb|KPA22698.1|typeIrestrictionenzymeEcoKIs/    ARTKAAEAAVT-LCDMDDEEIYGPTFFTRGFSWAVENGMLTDYKVIVLAMDETL---VSR
gi|738872465|ref|WP_036760884.1|damageinducibleprotein[Leisingeradaeponensis]/11643    AKSKADDLGIT-LATMDNEDMFGPTLFYRGFGWAVQEGLLTDYKVIVLAMDEKL---VAK
gi|518050881|ref|WP_019221089.1|DNAdamageinducibleprotein[Bartonellasenegalensis]/11652    AKSRANEANVV-LASMDDEKLFGKTLFYRGFSWAVQNDLLTDYKVIVLAMDEKL---VSS
gi|493980596|ref|WP_006923523.1|DNAdamageinducibleprotein[Bartonellawashoensis]_gi|395413322|gb|EJF79796.1|hypotheticalprotein/    VKSRAHEVNAV-LASMDDEKLFGKTLFYRGFSWAVKNDLLTDYKVIVLAVDEKL---VSS
gi|493983106|ref|WP_006925984.1|DNAdamageinducibleprotein[Bartonellawashoensis]_gi|395418127|gb|EJF84455.1|hypotheticalprotein/    AKSRAHETNAV-LASMDDKKLFGKTLFYRGFSWAVKNDLLTDYKVIVLAMDEKL---VSS
gi|499494671|ref|WP_011181311.1|DNAdamageinducibleprotein[Bartonellahenselae]_gi|49239008|emb|CAF28308.1|hypotheticalproteinBH/    AKSRANEANVV-LASMDDEKLFGKTLFYRGFSWAVQNNLLTDYKVIVLAMDEKL---VSS
gi|651388426|ref|WP_026500441.1|damageinducibleprotein[Bartonellagrahamii]/11652    AKSRANEANVV-LASMDDEKLFGKTLFYRGFSWAVQNDLLTDYKVIVLAMDEKL---VSS
gi|506337280|ref|WP_015856999.1|DNAdamageinducibleprotein[Bartonellagrahamii]_gi|240268402|gb|ACS51990.1|helicase/methyltransf/    AKSRANEANVV-LASMDDEKLFGKTLFYRGFSWAVQNDLLTDYKVIVLAMDEKL---VSS
gi|494817535|ref|WP_007552943.1|DNAdamageinducibleprotein[Bartonellasp.DB56]_gi|395412002|gb|EJF78518.1|hypotheticalproteinMC/1    VKSRAHEVDAV-LASMDDEKLFGKTVFYRGFSWAVQHDLLTDYKVIVLAVDEKL---VSS
gi|493638099|ref|WP_006589864.1|DNAdamageinducibleprotein[Bartonellabirtlesii]_gi|395409809|gb|EJF76394.1|hypotheticalproteinM/    VKSRAHEVNAV-LASMDDEKLFGKTVFYRGFSWAVQHDLLTDYKVIVLAVDEQL---VSS
gi|748189589|ref|WP_039760707.1|damageinducibleprotein[Bartonellaqueenslandensis]/11651    AKSRANEVDAV-LASMDDEKLFGKTLFYRGFSWAVRNNLLTDYKVIVLAMDEKL---VSS
gi|501189483|ref|WP_012232501.1|DNAdamageinducibleprotein[Bartonellatribocorum]_gi|161018902|emb|CAK02460.1|helicase/methyltra/    AKSRANEANAV-LASMDDEKLFGKTLFYRGFSWAVQHDLLTDYKVIVLAMDEKL---ISS
gi|518049181|ref|WP_019219389.1|DNAdamageinducibleprotein[Bartonellaflorencae]/11652    AKSRANEANAV-LASMDDEKLFGKTLFYRGFSWAVQHDLLTDYKVIVLAMDEKL---VSS
gi|492193255|ref|WP_005774528.1|DNAdamageinducibleprotein[Bartonellaelizabethae]_gi|395416403|gb|EJF82779.1|hypotheticalprotei/    AKSRAHEANAV-LASMDDEKLFGKTLFYRGFSWAVQHDLLTDYKVIVLAVDEKL---VSS
gi|498387325|ref|WP_010701288.1|helicase/methyltransferase[Bartonellabovis]_gi|478418724|gb|ENN92426.1|helicase/methyltransfera    AKSRANEVDAV-LASMDDEKLFGKTLFYRGFSWAVQNGLLTDYKVIVLAMDEGL---VSS
gi|319408697|emb|CBI82352.1|conservedhypotheticalprotein[BartonellaschoenbuchensisR1]/11661    VKSQANAVGAV-LASMDDEQLFGKTLFYRGFSWAVQNGLLTDYKVIVLTMDEKL---VSS
gi|494741726|ref|WP_007477134.1|DNAdamageinducibleprotein[Bartonellamelophagi]_gi|395423925|gb|EJF90113.1|hypotheticalproteinM/    AKSRANEVGAV-VASMDDEELFGKTLFYRGFSWAVQEGLLTDYKVIVLAMDEGL---VSS
gi|737261405|ref|WP_035245349.1|damageinducibleprotein[Alcanivoraxjadensis]_gi|686989709|gb|KGD62556.1|hypotheticalproteinT9A_/    AKGKADESGHE-LCSMDDKLKFGEVLFYRGFSWAVENDLLTDYKVIILAMDEGL---VGR
gi|916687564|ref|WP_051294655.1|damageinducibleprotein[Gemmobacternectariphilus]/11641    VKSRAEEAEAV-LVSMDDEALFGKTLFYRGFSWAVQEGLLTDYKVIVLAMDEGL---VSS
gi|740294127|ref|WP_038132495.1|damageinducibleprotein[Thioclavasp.DT234]_gi|660524826|gb|KEO54689.1|hypotheticalproteinDT23_/1    VKSKADEEAAV-LSSMDDESLYGKTLLHKGFGWAVQKGLLTDYKVIVLAMDEGL---VSA
gi|568331062|ref|WP_024099615.1|helicase[Phaeobactergallaeciensis]_gi|568232121|gb|AHD12007.1|putativehelicase[Phaeobactergalla    VKAKADEADAV-LASMDDEDLFGKTLFYRGFSWAVQNNLLTDYKVIVLAMDEGL---IST
gi|518810459|ref|WP_019966413.1|DNAdamageinducibleprotein[Pannonibacterphragmitetus]/11635    VRSKADEVGAE-LASMDNPALFGETLFYRGFGWAVQNGLLADYKVIVLAMDEGL---VST
gi|495305760|ref|WP_008030510.1|DNAdamageinducibleprotein[Rhodobactersp.SW2]_gi|259021698|gb|EEW25000.1|typeIIIrestrictionprot/    VRSKADEVGAE-LASMDNPALFGETLFYRGFGWAVQNGLLTDYKVIVLAMDEGL---VSA
gi|506403303|ref|WP_015923022.1|DNAdamageinducibleprotein[Rhodobactersphaeroides]_gi|221162951|gb|ACM03922.1|HypotheticalProte/    VRSKADEVGAE-LASMDDETLFGKTLFYRGFGWAVQNGLLTDYKVIVLAMDEGL---VSA
gi|738818386|ref|WP_036708790.1|damageinducibleprotein[Paracoccussanguinis]_gi|694226786|gb|KGJ22541.1|damageinducibleprotein/1    VRSKADEVGAE-LASMDDPALFGETLFYRGFGWAVQNGLLTDYKVIVLAMDEGL---VSA
gi|746096057|ref|WP_039162453.1|damageinducibleprotein[Gallibacteriumanatis]_gi|703617775|gb|KGQ63868.1|damageinducibleprotei/1    AKK---TEGVV-VYSMDDKNLFGDELYTINFSSAVKRGLLVDYKVLVLAVEESH---INR
gi|737716785|ref|WP_035685631.1|damageinducibleprotein[Avibacteriumparagallinarum]/11643    AKK---TEGVV-VYSMDDKNLFGDELYTINFSSAVKRGLLVDYKVLVLAVEESH---INR
gi|540072553|gb|ERF78672.1|hypotheticalproteinN561_04930[Gallibacteriumanatis12656/12]/11651    AKK---TEGVV-VYSMDDKNLFGDELYTINFSSAVKRGLLVDYKVLVLAVEESH---INR
gi|917509519|ref|WP_052115936.1|damageinducibleprotein[Gallibacteriumanatis]_gi|702414161|gb|KGQ42233.1|damageinducibleprotei/1    AKK---TEGVV-VYSMDDKNLFGDELYTINFSSAVKRGLLVDYKVLVLAVEESH---INR
gi|746082942|ref|WP_039150268.1|damageinducibleprotein[Gallibacteriumanatis]_gi|703617078|gb|KGQ63194.1|damageinducibleprotei/1    AKK---TEGVV-VYSMDDKNLFGDELYTINFSSAVKRGLLVDYKVLVLAVEESH---INR
gi|822522885|ref|WP_046952840.1|damageinducibleprotein[Haemophilushaemolyticus]_gi|821501762|gb|KKZ59267.1|damageinduciblepro/1    AKK---TEGVT-VYSMDDETKFGQDLYTISFSSAVKQNLLVDYKVLVLAVEESH---IHQ
gi|404381014|gb|EJZ77501.1|putativehelicase[Pasteurellamultocidasubsp.gallicidaP1059]/11681    AKK---TEGVT-VYSMDDKELFGEELHVISFSSAVKQGLLVDYKVLVLAVEESH---IHQ
gi|298281660|gb|EFI23149.1|helicaseCterminaldomainprotein[Neisseriasp.oraltaxon014str.F0314]/11493    AKA---ADGVS-VYSMDDETHYGKEFFVLTFSQAVSQKLLVDYKVIVLAIDQAH---VER
gi|348013055|gb|EGY51980.1|helicasedomainprotein[Neisseriashayeganii871]/11481    AKA---TEGVA-LYSMDDETHYGKEFFVLTFSEAVSDGLLVDYKVIVLAIEEAH---INR
gi|496407140|ref|WP_009116004.1|DNAdamageinducibleprotein[Neisseriawadsworthii]_gi|349794756|gb|EGZ48566.1|helicasedomainprote/    AKA---AEGVV-LYSMDDETHYGKEFFVLTFSQAVSDGLLVDYKVIVLAIEESH---VNR
gi|517216441|ref|WP_018405259.1|DNAdamageinducibleprotein[Marinobacterlipolyticus]/11656    AKQ---TENVT-LCSMDDKALYGEELYVITFSEAVARKLLVDYKVIVLAVEEAH---VNR
gi|738230264|ref|WP_036185691.1|damageinducibleprotein[Marinobactersp.ELB17]/11671    AKQ---TENVT-LCSMDDKTLYGEELYVITFSEAVARKLLVDYKVIVLAVEEAH---VNR
gi|447197741|ref|WP_001274997.1|DNAdamageinducibleprotein[Acinetobacterbaumannii]_gi|408512324|gb|EKK13969.1|typeIIIrestrictio/    AKE---TEGVT-LCSMDDKSLYGEDLYVITFSQAVKMGILCDYKVIVLAVEESH---INR
gi|820775897|ref|WP_046739909.1|damageinducibleprotein[Acinetobactersp.AG1]_gi|819909758|gb|KKW75938.1|damageinducibleprotein/1    ALKDEEENKVK-LYPMNRVEIFGEDLYVITFSQAVKMDILCDYKVIVLAVEESH---INR
gi|584595163|ref|WP_024160846.1|helicase[Acinetobacterpittii]_gi|576702530|emb|CDI28049.1|helicasedomaincontainingprotein[Acin/    AKE---TEGVT-LCSMDDKSLYGEDLYVITFSQAVKMDILCDYKVIVLAVEESH---INR
gi|514963042|ref|WP_016651366.1|hypotheticalprotein[Acinetobactersp.NIPH2036]_gi|514162665|gb|EPG41391.1|hypotheticalproteinF90    AKE---TEGVT-LCSMDDKSLYGEDLYVITFSQAVKMGILCDYKVIVLAVEESH---INR
gi|515965564|ref|WP_017396147.1|DNAdamageinducibleprotein[Acinetobacterhaemolyticus]/11667    AKE---TEGVT-LCSMDDKSLYGEDLYVITFSQAVKMGILCDYKVIVLAVEESH---INR
gi|491346810|ref|WP_005204747.1|MULTISPECIES:hypotheticalprotein[Acinetobacter]_gi|480335823|gb|ENX56033.1|hypotheticalproteinF    AKE---TEGVT-LCSMDDKSLYGDDLYVITFSQAVKMDILCDYKVIVLAVEESH---INR
gi|693044472|ref|WP_032215268.1|damageinducibleprotein[Escherichiacoli]_gi|607725731|gb|EZE08230.1|damageinducibleprotein[Esc/1    AKE---TEGVT-LCSMDDKSLYGDDLYVITFSKAVQLGILCDYKVIVLAVEEKH---VSR
gi|742382177|ref|WP_038861304.1|damageinducibleprotein[Cronobactermuytjensii]_gi|924333747|gb|ALB69766.1|damageinducibleprote/1    AKE---TEGVT-LCSMDDKNLYGEDLYVITFSKAVQLGILCDYKVIVLAVEESH---ISR
gi|915912804|ref|WP_050927035.1|damageinducibleprotein[Yersiniaenterocolitica]_gi|899805802|emb|CQH32042.1|typeIrestrictionenz/    AEN---TEGVENIYSMNNEKWFGEDLYVITFSKAVQLGILCDYKVIVLAVEQKH---VSR
gi|921111327|gb|KON62781.1|typeIrestrictionenzymeEcoKIsubunitR[Komagataeibactereuropaeus]/11492    AREKAEKEGAI-VYGMNNVAIYGPEFHVITFSEAVRRKLLVDYKVIVLAVDEGT---VSR
gi|582024278|gb|AHI27615.1|DNAhelicaserestrictionenzymeTypeIIIRsubunit[KomagataeibacterxylinusE25]/11636    AQEKAEKEGAI-VYGMNNEAIYGPEFHVITFSEAVRRKLLVDYKVIVLAVDEGT---VSR
```

gi|347662673|dbj|BAK86128.1|DNAhelicaserestrictionenzymeTypeIIIRsubunit[KomagataeibactermedellinensisNBRC3288]/11737	AQEKAEKEGAT-VYGMNNVAIYGPEFHVITFSEAVRRKLLVDYKVIVLAVDEGT---VSR
gi|917445053|ref|WP_052051471.1|damageinducibleprotein[Acetobactertropicalis]/11429	AQEKAEKEGAI-VYGMNNAEIFGPEFHVITFSEAVRRELLVDYKVIVLAVDEGT---VSA
gi|453329445|dbj|GAC88472.1|DNAhelicaserestrictionenzymetypeIIIRsubunit[GluconobacterthailandicusNBRC3255]_gi|530287981|dbj|GAD	AQEKAENEGAI-VYGMNNAEIFGPEFHVITFSEAVRRKLLVDYKVIVLAVDEGT---VSA
gi|889770922|ref|WP_048844278.1|damageinducibleprotein[Gluconobacterfrateurii]/11419	AQEKAENEGAI-VYGMNNAEIFGPEFHVITFSEAVRRKLLVDYKVIVLAVDEGT---VSA
gi|504886208|ref|WP_015073310.1|hypotheticalprotein[Gluconobacteroxydans]_gi|411027954|gb|AFW01209.1|hypotheticalproteinB932_16	AQEKAENEGAI-VYGMNNAEIFGPEFHVITFSEAVRRKLLVDYKVIVLAVDEGT---VSA
gi|480040211|gb|ENV79751.1|hypotheticalproteinF942_01641[AcinetobacterursingiiANC3649]/11432	VEINAERDKAK-LYGMNNEAIYGKELFVINFSEAVQRGLLVDYKVIVLAVDESH---VNK
gi|822498222|ref|WP_046936504.1|damageinducibleprotein[Xanthomonasgardneri]_gi|821751224|gb|KLB07223.1|damageinducibleprotein/1	AKAVAERDNIA-LYSMDNEDWYGKTLFTINFSEAVKRGLLVDYKVFVLAVEESH---INR
gi|654059559|ref|WP_027705556.1|damageinducibleprotein[Zymobacterpalmae]/11635	SKTTAERNSVT-LYSMDNEAWFGKELFVINFSTAVQKGLLVDYKVIVLAVEEYH---INR
gi|643535620|ref|WP_025224315.1|damageinducibleprotein[Methylophilaceaebacterium11]_gi|577704400|gb|EUJ10546.1|putativehelicas/	AKASADKDAVT-LCSMDDVALYGKELYVITFSEAVKRDLLVDYKVLVLAVDEAH---VSR
MBA11I/11635	AKASADKDAVT-LCSMDDVALYGKELYVITFSEAVKRDLLVDYKVLVLAVDEAH---VSR
gi|655355857|ref|WP_028762287.1|damageinducibleprotein[Shewanellacolwelliana]/11632	AKANAEKDSVT-LCSMDDPALYGRDLHTITFSEAVRRDLLCDYKVIVLTVDEAY---ISK
gi|753204236|ref|WP_041510579.1|damageinducibleprotein[Shewanellasp.cp20]_gi|752457765|gb|KIO36326.1|damageinducibleprotein[S/1	AKASADKGDVA-LASMDDESLFGKNLHTLTFSEAVSRGLLCDYKVIVLTMDEHH---VTR
KPNNIH30III/11619	AKASADKGDVT-LASMDDESLFGKNLHILTFSEAVSRGLLCDYKVIVLTMDEHH---INR
gi|749295994|ref|WP_040120352.1|damageinducibleprotein[Klebsiellapneumoniae]_gi|723249300|gb|AIX81926.1|damageinducibleprotei/1	AKASADKGDVT-LASMDDESLFGKNLHILTFSEAVSRGLLCDYKVIVLTMDEHH---INR
gi|641639739|emb|CDQ54010.1|unnamedproteinproduct[Klebsiellapneumoniae]/11641	AKASADKGDVT-LASMDDESLFGKNLHILTFSEAVSRGLLCDYKVIVLTMDEHH---INR
gi|490797922|ref|WP_004660060.1|hypotheticalprotein[Acinetobactersp.NIPH236]_gi|479876779|gb|ENU27973.1|hypotheticalproteinF992	VKNNA--SDYT-LFSMDNEKLFGETLYTINFSEAVKRGLLVDYKVIVLTVDSDT---IIN
gi|916750926|ref|WP_051357982.1|damageinducibleprotein[Acinetobactertjernbergiae]/11453	VKNNA--TDYT-LFSMDNEKLFGETLYTINFSEAVKRGLLVDYKVIVLTVDSDT---IIE
gi|691098662|ref|WP_032025060.1|damageinducibleprotein[Acinetobacterbaumannii]_gi|593567521|gb|EXR50735.1|restrictionendonucle/	VKDKA--TDFT-LYSMDNEKLFGETLYTINFSEAVKRGLLVDYKVIVLTVDSDT---IIN
gi|736669405|ref|WP_034676049.1|damageinducibleprotein[Acinetobacterbaumannii]/11643	VKNNA--TDYT-LYSMDNEKLFGETLYTINFSEAVKRGLLVDYKVIVLTVDSDT---IIK
gi|491368928|ref|WP_005226826.1|hypotheticalprotein[Acinetobactersp.NIPH1847]_gi|480173259|gb|ENX09797.1|hypotheticalproteinF89	VKNNA--TDYT-LFSMDNEKLFGETLYTINFSEAVKRGLLVDYKVIVLTVDSDT---IIN
gi|796853993|ref|WP_045795953.1|damageinducibleprotein[Acinetobacterindicus]_gi|787929761|gb|KJV44219.1|damageinducibleprotei/1	VKNNA--TDFT-LFSMDDEKLFGETLYTVNFSEAVKRSLLVDYKVIVLTVDSDT---IIK
gi|295797463|gb|ADG32253.1|typeIIIrestrictionproteinressubunit[ThiomonasintermediaK12]/11676	AKSKADRENIV-LCSMDDEALYGRELHVITFSEAVSRGLLVDYKVIVLAVEEAH---INR
gi|499871179|ref|WP_011551913.1|DNAdamageinducibleprotein[Myxococcusxanthus]_gi|108467544|gb|ABF92729.1|putativerestriction/mo/	AKATAERDNVA-LCSMDDERLYGPALHVLTFSEAVKRGLLVDYKVIVLAIEESH---VSR
gi|495313852|ref|WP_008038600.1|DNAdamageinducibleprotein[Bartonellatamiae]_gi|395424181|gb|EJF90368.1|hypotheticalproteinME5_/	AKLKAEGGDVV-LYSMDDETIYGKDLHVLTFSEAVQRGLLVDYKVIVLTVEESQ---INR
gi|501453335|ref|WP_012476784.1|DNAdamageinducibleprotein[Leptospirabiflexa]_gi|167777824|gb|ABZ96123.1|Helicase[Leptospirabif/	AKASAEKDNVT-LCSMDDETLFGKELYVINFSTAVSRELLVDYKVIVLAVDEDH---VNA
gi|738104613|ref|WP_036063193.1|damageinducibleprotein[Leptospiranoguchii]/11652	AQAKAQQENIT-LASMDDESLFGKALYVINFSDAVQRGLLVDYKVIVLAIEEDH---INS
gi|410001039|gb|EKO51661.1|typeIIIrestrictionenzyme,ressubunit[Leptospirakirschneristr.200802841]/11651	AQAKAQQENIT-LASMDDESLFGKALHIINFSDAVQRGLLVDYKVIVLAVEEDH---INN
gi|490901851|ref|WP_004763774.1|typeIIIrestrictionenzyme,ressubunit[Leptospirakirschneri]_gi|464209249|gb|EMN24014.1|typeIIIres	AQAKAQQENIT-LASMDDESLFGKALHIINFSDAVQRGLLVDYKVIVLAVEEDH---INN
gi|464398131|gb|EMO65902.1|typeIIIrestrictionenzyme,ressubunit[Leptospirakirschneristr.200803703]/11648	AQAKAQQENIT-LASMDDESLFGKALHIINFSDAVQRGLLVDYKVIVLAVEEDH---INN
gi|945029024|gb|KQQ84900.1|damageinducibleprotein[Xanthomonassp.Leaf131]/11423	AKAVAERDNVA-LYSMDNDDWYGKSLFTINFSEAVKRGLLVDYKVIVLAVEESH---INR
gi|896511314|ref|WP_049418183.1|damageinducibleprotein[Stenotrophomonasmaltophilia]/11639	AKAIAERDNAT-LYSMDNEDWYGKQLFVITFSEAVKRGLLVDYKVIVLAVEEAH---VNR
gi|896513082|ref|WP_049419616.1|damageinducibleprotein[Stenotrophomonasmaltophilia]/11638	AKAVAERDSVA-LYSMDNEAWYGKQLFVITFSEAVKRGLLVDYKVIVLAVEEAH---INR
gi|896580616|ref|WP_049469425.1|damageinducibleprotein[Stenotrophomonasmaltophilia]/11641	AKAVAERDNVA-LYSMDNEAWYGKQLFVITFSEAVKRGLLVDYKVIVLAVEESH---INR
gi|648289350|ref|WP_026070499.1|damageinducibleprotein[Stenotrophomonasmaltophilia]/11639	AKAVAERDNVA-LYSMDNEDWYGKQLFVITFSEAVKRGLLVDYKVIVLAVEESH---INR
gi|656272786|ref|WP_029218442.1|damageinducibleprotein[Xanthomonascassavae]/11639	AKAVAERDNVA-LYSMDNEAWYGRQLFVITFSEAVKRGLLVDYKVIVLAVEESH---INR
gi|406976236|gb|EKD98747.1|hypotheticalproteinACD_23C00290G0002[unculturedbacterium]/11679	AKAKAETDNVA-LCSMDDPLLYGPELYVINFSEAVRLGLLCDYKVVVLAIEESH---VSR
gi|828145737|ref|WP_047238120.1|damageinducibleprotein[Chromobacteriumsubtsugae]/11645	AKVTAEKGDVT-LCSMDDETLFGKELFVITFSEAVKAGLLVDYKVLVLAMDEAH---ISR
gi|654311174|ref|WP_027824327.1|damageinducibleprotein[Laribacterhongkongensis]/11641	AKAQEEKGGVA-LCSMDDESLFGKELFVITFSEAVKAGLLVDYKVLVLAMDEAH---ISR
gi|757242173|gb|KIR15516.1|typeIrestrictionenzymeEcoKIsubunitR[Pseudomonasfluorescens]/11778	AEAKAEQENAK-LYGMNQEEWYGKELFVITFSEAVKAELLVDYKVLVLAMDEKH---ISS
gi|653605161|ref|WP_027616682.1|damageinducibleprotein[Pseudomonassp.URHB0015]/11639	AKASAEKDNVA-LCSMDDIVLYGKELFVITFSEAVKAELLVDYKVLVLAMDEKH---ISS
gi|520868025|ref|WP_020308060.1|helicase[Pseudomonassyringae]_gi|519159034|gb|EPM49935.1|helicasedomaincontainingprotein[Pseud/	AKASAEKDDVA-LCSMDDGSLYGKELFVITFSEAVKAELLVDYKVLVLAMDEKH---ISS
gi|940156567|gb|KPX71668.1|Helicasedomaincontainingprotein[Pseudomonasamygdalipv.lachrymans]/11677	AKASAEKDNVA-LCSMDDAVLYGKELFVITFSEAVKAELLVDYKVLVLAMDEKH---ISS
gi|505460508|ref|WP_015647610.1|helicase[Pseudomonassp.GLE121]_gi|499110212|ref|YP_007974374.1|helicasedomaincontainingprotein/	AKVKAETNNVA-LCSMDDEALYGKELYTITFSEAVKRGLLVDYKVLVLAVEETH---VNR
gi|940235551|gb|KPY45311.1|hypotheticalproteinALO48_200154[Pseudomonassyringaepv.rhaphiolepidis]/11631	AKVKAETDNVT-LCSMDNEALYGKELYTITFSDAVKRGLLVDYKVLVLAVEETH---VNR
gi|520880808|ref|WP_020312235.1|helicase[Pseudomonassyringae]_gi|519172158|gb|EPM62027.1|helicasedomaincontainingprotein[Pseud/	AKVKAESGDVT-LCSMDDEALYGKELFIINFSEAVKRGLLVDYKVLVLTVEESV---ISR
gi|829046479|ref|WP_047282259.1|damageinducibleprotein[Pseudomonasfluorescens]/11630	AKIKAESGEVT-LCSMDDEALYGKELFVINFSEAVQRGLLTDYKVLVLTVEESA---ISR
gi|499415493|ref|WP_011102960.1|DNAdamageinducibleprotein[Pseudomonassyringaegroupgenomosp.3]_gi|28867279|ref|NP_789898.1|heli/	AKIKAESGEVT-LCSMDDEALYGKELFVINFSEAVQRGLLTDYKVLVLTVEESI---VNR
gi|730238299|ref|WP_033942897.1|damageinducibleprotein[Pseudomonasaeruginosa]/11629	AKVKAESGEVT-LCSMDDEALYGKELFVINFSEAVQRGLLTDYKVLVLTVEEST---ISR
gi|495310125|ref|WP_008034873.1|DNAdamageinducibleprotein[Pseudomonassp.GM60]_gi|398186685|gb|EJM74052.1|putativehelicase[Pseu/	AKVKAESGEVT-LCSMDDETLYGKELFVINFSEAVQRGLLTDYKVLVLTVEESA---ISR
gi|779992653|ref|WP_045428690.1|damageinducibleprotein[Pseudomonassp.MT1]_gi|699967872|dbj|BAP80334.1|DNAhelicaserestrictione/1	AKVKAESGDVT-LCSMDDETLYGKELFVINFSEAVQRGLLTDYKVLVLTVEESV---INR
gi|786037289|ref|WP_045733274.1|damageinducibleprotein[Pseudomonaspseudoalcaligenes]_gi|782987904|gb|KJU79152.1|damageinducib/1	AKIKAESGEVT-LCSMDDEALYGKELFVINFSEAVQRGLLTDYKVLVLTVEESV---INR
gi|749666366|ref|WP_040265445.1|damageinducibleprotein[Pseudomonasrhodesiae]/11632	AKIKAESGEVT-LCSMDDEALYGKELFVINFSEAVQRGLLTDYKVLVLTVEESV---INR
gi|734902433|ref|WP_034133698.1|damageinducibleprotein[Pseudomonasfluorescens]/11635	AKIKAESGEVT-LCSMDDEALYGKELFVINFSEAVQRGLLTDYKVLVLTVEESV---INR
gi|490551031|ref|WP_004416146.1|typeIIrestrictionmodificationsystemrestriction/helicasesubunit[Pseudomonassyringae]_gi|4432823/	AKLKAESGEVT-LCSMDDEALYGKELFVINFSEAVQRGLLTDYKVLVLTVEEST---ISR
gi|489199911|ref|WP_003109152.1|DNAdamageinducibleprotein[Pseudomonasaeruginosa]_gi|552648708|gb|ERY63184.1|hypotheticalprotei/	AKIKAESGEVT-LCSMDDEALYGKELFVINFSEAVQRGLLTDYKVLVLTVEEST---ISR
gi|730641237|ref|WP_034061328.1|damageinducibleprotein[Pseudomonasaeruginosa]/11636	AKLKAESGEVT-LCSMDDEALYGKELFVINFSEAVQRGLLTDYKVLVLTVEEST---ISR
gi|751304980|ref|WP_041013386.1|damageinducibleprotein[Pseudomonasxanthomarina]_gi|703407611|emb|CEG51014.1|Helicasedomaincon/1	AKIKAESGEVT-LCSMDDEALYGKELFVINFSEAVQRGLLTDYKVLVLTVEESV---INR
gi|939335207|ref|WP_054881520.1|damageinducibleprotein[Pseudomonassp.NBRC111128]/11632	AKLKAESGEVT-LCSMDDEALYGKELFVINFSEAVQRGLLTDYKVLVLTVEEST---ISR
gi|757693555|ref|WP_042923792.1|damageinducibleprotein[Pseudomonaschloritidismutans]_gi|780910610|gb|KJS78302.1|damageinducib/1	AKLKAESGEVT-LCSMDDEALYGKELFVINFSEAVQRGLLTDYKVLVLTVEQSV---ISR
gi|665953944|ref|WP_031319989.1|damageinducibleprotein[Pseudomonastolaasii]/11626	AKVKAESGEVT-LCSMDDEALYGKELFVINFSEAVQRGLLTDYKVLVLTVEEST---ISR
gi|771600573|ref|WP_045213746.1|damageinducibleprotein[Pseudomonassp.21]_gi|770081123|gb|KJJ98634.1|damageinducibleprotein[Ps/1	AKVKAESGEVT-LCSMDDVALYGKELFVINFSEAVQRGLLTDYKVLVLTVEESA---ISR
gi|740871396|ref|WP_038656644.1|damageinducibleprotein[Pseudomonasstutzeri]_gi|627772214|gb|AHY41437.1|damageinducibleprotein/1	AKIKAESGEVT-LCSMDDEALYGKELFVINFSEAVQRGLLTDYKVLVLTVEESV---INR
gi|946242205|gb|KRC93782.1|damageinducibleprotein[Pseudomonassp.Root9]/11634	AKLKAESGEVT-LCSMDDEALYGKELFVINFSEAVQRGLLTDYKVLVLTVEEST---ISR
gi|553777322|ref|WP_023109107.1|MULTISPECIES:hypotheticalprotein[Pseudomonas]_gi|552638674|gb|ERY53184.1|hypotheticalproteinQ05	AKLKAESGEVT-LCSMDDDALYGKELFVINFSEAVQRGLLTDYKVLVLTVEEST---ISR
gi|896383370|ref|WP_049324618.1|damageinducibleprotein[Pseudomonasstutzeri]/11627	AKVKAESGEVA-LCSMDDEALYGKELFVINFSEAVQRGLLTDYKVLVLTVEESV---INR
gi|696158510|gb|AIT41787.1|putativehelicase[Pseudomonassp.LM8]/11630	AKVKAESGEVA-LCSMDDEALYGKELFVINFSEAVQRGLLTDYKVLVLTVEESV---INR
gi|504634892|ref|WP_014821994.1|DNAdamageinducibleprotein[Pseudomonasstutzeri]_gi|390985249|gb|AFM35242.1|helicasedomainconta/1	AKIKAESGEVT-LCSMDDEALYGKELFVINFSEAVQRGLLTDYKVLVLTVEESV---INR

gi|544783974|ref|WP_021206729.1|DNAdamageinducibleprotein[Pseudomonasstutzeri]_gi|533224779|gb|EQM79688.1|DNAdamageinduciblep/1 AKVKAESGEVA-LCSMDDEALYGKELFVINFSEAVQRGLLTDYKVLVLTVEESV---ISR
gi|504567385|ref|WP_014754487.1|MULTISPECIES:DNAdamageinducibleprotein[Pseudomonas]_gi|388559493|gb|AFK68634.1|helicasedomain/1 AKLKAESGEVT-LCSMDDEALYGKELFVINFSEAVQRGLLTDYKVLVLTVEEST---ISR
gi|654441469|ref|WP_027912052.1|damageinducibleprotein[Pseudomonassp.URIL14HWK12:I7]/11630 AKLKAESGEVT-LCSMDDEALYGKELFVINFSEAVQRGLLTDYKVLVLTVEEST---ISR
gi|941470066|ref|WP_055127894.1|damageinducibleprotein[Pseudomonasmediterranea]/11631 AKLKAESGEVT-LCSMDDEALYGKELFVINFSEAVQRGLLTDYKVLVLTVEEST---ISR
gi|557815599|ref|WP_023446184.1|DNAdamageinducibleprotein[Pseudomonaschloritidismutans]_gi|557364081|gb|ESQ98190.1|DNAdamagei/1 AKLKAESGEVT-LCSMDDEALYGKELFVINFSEAVQRGLLTDYKVLVLTVEEST---ISR
gi|727814791|ref|WP_033898247.1|damageinducibleprotein[Pseudomonassp.FH1]_gi|567648005|gb|ETK23229.1|helicasedomaincontaining/1 AKIKAESGEVT-LCSMDDEALYGKELFVINFSEAVQRGLLTDYKVLVLTVEESV---INR
gi|503278702|ref|WP_013513363.1|DNAdamageinducibleprotein[Desulfovibrioaespoeensis]_gi|316942375|gb|ADU61426.1|typeIIIrestrict/ AKATAERDDIV-LCSMDDEERYGKDLYVINFSEAVKRGLLCDYKVLVLSVDEGH---VSR
gi|505228796|ref|WP_015415898.1|typeIIIrestrictionendonucleasesubunitR[Desulfovibriopiezophilus]_gi|451896976|emb|CCH49855.1|Ty AKATAERDDIV-LCSMDDEKQYGKELYTINFSEAVKLGLLCDYKVLVLSVDETH---VSR
gi|517732543|ref|WP_018902751.1|hypotheticalprotein[Variovoraxparadoxus]/11675 AKASAEKDNVA-LYSMDDKALYGDELHVITFSQAVANKLLVDYKVIVLAVEETH---VNR
gi|550909475|ref|WP_022662570.1|DNAdamageinducibleprotein[Desulfovibriolongus]/11637 AKATAEKDNVA-LCSMDDEKLYGKELYVLSFSESVKRQLLVDYKVLVLAVEESA---INR
gi|945494542|gb|KQV50024.1|damageinducibleprotein[Massiliasp.Root335]/11647 AKAKAELDSVA-LCSMDDPTLYGPELHVITFSEAVRRGLLVDYKVIVLSVEESH---VNR
gi|334196917|gb|AEG70102.1|helicasedomainprotein[RalstoniasolanacearumPo82]/11663 AKASAERDNVA-LCSMDDESLYGKELFVITFSEAVKRQLLVDYKVIVLAVEETH---VNR
gi|656006746|ref|WP_029048170.1|damageinducibleprotein[Cupriavidussp.amp6]/11661 AKATAERDNVA-LCSMDDKALYGEELFVITFSEAVKRELLVDYKVIVLAVEEAH---VSR
BPS2196ORF3054P/11659 AKATAERDNVA-LCSMDDKALYGDELFVITFSEAVKRGLLVDYKVIVLAVEETH---VNR
gi|740932312|ref|WP_038717332.1|damageinducibleprotein[Burkholderiapseudomallei]_gi|760211882|gb|KIX58314.1|damageinduciblepr/1 AKATAERDNVA-LCSMDDKALYGDELFVITFSEAVKRGLLVDYKVIVLAVEETH---VNR
gi|755810813|ref|WP_042583861.1|damageinducibleprotein[Burkholderiasp.MSHR3999]_gi|755174952|gb|KIP19790.1|DEAD/DEAHboxhelicas/ AKATAERDNVA-ICSMDDKALYGNELFVITFSEAVKRGLLVDYKVIVLAVEETH---VNR
gi|484586570|gb|AGK48195.1|DEAD/DEAHboxhelicasefamilyprotein[BurkholderiathailandensisMSMB121]/11646 AKATAERDNVA-LCSMDDKALYGNELFVITFSEAVKRGLLVDYKVIVLAVEETH---VNR
gi|737582935|ref|WP_035553941.1|damageinducibleprotein[Burkholderiasp.9120]/11658 AKATAIRDDVA-LCSMDDKALYGDELFVITFSEAVKRGLLVDYKVIVLAVEEKH---VNR
gi|740989872|ref|WP_038773225.1|damageinducibleprotein[Burkholderiapseudomallei]_gi|705645291|gb|KGS70691.1|DEAD/DEAHboxhelica/ AKATAERDNVA-LCSMDDRALYGDELFVITFSEAVKRGLLVDYKVIVLAVEETH---VNR
gi|654912525|ref|WP_028363424.1|damageinducibleprotein[Burkholderiasp.JPY366]/11659 AKATAERDNVA-LCSMDDKALYGDELFVITFSEAVKRGLLVDYKVIVLAVEETH---VNR
gi|740955879|ref|WP_038740512.1|damageinducibleprotein[Burkholderiapseudomallei]_gi|714684932|gb|KGW74438.1|DEAD/DEAHboxhelica/ AEAQAERDNLR-LYGMNKEEWYGKELFVITFSEAVKRGLLVDYKVIVLAVEETH---VNR
gi|919203714|ref|WP_052759215.1|damageinducibleprotein[Xanthomonasaxonopodis]/11388 SKTKAEEAGAE-IASMDDETKFGPEFHRLGFGEAVSQGLLADYKVMVLAVDEKS---VTK
gi|522152381|ref|WP_020663589.1|hypotheticalprotein[Amycolatopsisbenzoatilytica]/11605 TKTSAQQNSAE-LCSMDDEALYGPEFHRLGFGKAVAEGLLTDYKVLILTVDEKY---IAT
gi|663196013|ref|WP_030233899.1|damageinducibleprotein[Streptomycessp.NRRLS455]/11641 TKADAKDASAV-LASMDDESLYGPEFHRLGFGKAVEQGLLTDYRVLILTVDQNV---VAK
gi|750429553|ref|WP_040711444.1|damageinducibleprotein[Nocardiopsispotens]/11622 AKQDAKNADAV-LTSMGDESVYGPEFHRLGFGKAVERGLLTDYRVLVLTVDEGA---VAK
gi|518656139|ref|WP_019819475.1|hypotheticalprotein[Saccharomonosporasaliphila]/11623 TKQEAKGAAAA-VASMDDETTYGPEFHRLGFGKAVQKGLLTDYKVLILTVDEGV---VAK
gi|759722933|ref|WP_043433758.1|damageinducibleprotein[Streptomycespluripotens]_gi|733404978|gb|KHK94361.1|damageinduciblepro/1 TKQEAKGASAA-VASMDDETKYGPEFHRLGFGKAVGQGLLTDYKVLILTIDEGV---VAK
gi|663424167|ref|WP_030419030.1|damageinducibleprotein[Streptomycessp.NRRLF5065]/11627 TKQEAKGASAA-VASMDNETQYGPEFHRLGFGKAVEQGLLTDYKVLILTIDEGV---VAK
gi|663412917|ref|WP_030408353.1|damageinducibleprotein[Streptomycessp.NRRLF5917]/11630 TKQDAKGAAAA-VASMDDETKYGPEFHRLGFGKAVEQGLLTDYKVLILTIDEGV---VAK
gi|671521483|ref|WP_031506489.1|damageinducibleprotein[Streptomycesmegasporus]/11630 TKQEAKGASAA-VASMDDETKYGPEFHRLGFGKAVEQGLLTDYKVLILTIDEGV---VAK
gi|740053144|ref|WP_037902776.1|damageinducibleprotein[Streptomycessp.TAA204]/11626 AKADAKNADAV-LASMDNEQMFGPEFHRLGFGQAVEQGLLTDYKVLILTIDEGV---VAE
gi|663409375|ref|WP_030404974.1|MULTISPECIES:damageinducibleprotein[Streptomyces]/11627 TKADAKNAEAV-LASMDNEQMFGPEFHRLGFGQAVEQGLLTDYKVLILTIDEGV---VAK
gi|746628608|ref|WP_039654277.1|damageinducibleprotein[Streptomycessp.MUSC125]_gi|745644381|gb|KIE25557.1|damageinducibleprot/1 TKADAKNADAV-LASMDNQQMFGPEFHRLGFGQAVEQGLLTDYKVLILTIDEGV---VAK
gi|727322066|ref|WP_033777320.1|damageinducibleprotein[Salinisporapacifica]/11627 TKSEARNADAL-LASMDDEATYGPEFHRLGFGKAVEQGLLTDYKVLILTVDEGR---VAK
gi|653653242|ref|WP_027655726.1|damageinducibleprotein[Salinisporapacifica]/11618 TKAEARNADAL-LASMDDEATYGPEFHRLGFGKAVEQGLLTDYKVLILTVDEGR---VAK
gi|648632543|ref|WP_026324294.1|damageinducibleprotein[Salinisporapacifica]/11621 TKAEARNADAL-LASMDDEATYGPEFHRLGFGKAVERGLLTDYKVLILTVDEGR---VAK
gi|881018002|ref|WP_048738463.1|damageinducibleprotein[Corynebacteriumfalsenii]/11676 VKGKAADHSAE-VASMDDEGIFGPEFHHLGFGEAVAKNLLTDYRVLVMTVDEEA---GAS
gi|505465526|ref|WP_015652417.1|helicase[Corynebacteriumcallunae]_gi|459385704|gb|AGG67995.1|helicase[CorynebacteriumcallunaeDS VKGKAADHSAE-VSSMDDEAIYGPEFHRLGFGEAVEKGLLTDYKVVVMTVDEQV---AAN
gi|41223062|emb|CAF19007.1|DNAorRNAhelicaseofsuperfamilyII[CorynebacteriumglutamicumATCC13032]/11646 VKGKAADHSAE-VSSMDDEAIYGPEFHRLGFGEAVEKGLLTDYKVVVMTVDEQV---AAS
CGL13032I/11643 VKGKAADHSAE-VSSMDDEAIYGPEFHRLGFGEAVEKGLLTDYKVVVMTVDEQV---AAS
gi|751257081|ref|WP_040967675.1|damageinducibleprotein[Corynebacteriumglutamicum]_gi|748811620|gb|AJE67931.1|damageinduciblep/1 IKGKAAEHSAE-LVSMDDEAIYGPEFHRLGFGEAVERGLLTDYKVLVMTVDEQL---ASD
gi|749040539|ref|WP_040086524.1|damageinducibleprotein[Corynebacteriumhumireducens]/11663 VKGKAAEHSAE-LASMDDESVYGPEFHRLGFGEAVEKGLLTDYKVLVMTVDEQL---ASD
gi|491662899|ref|WP_005519615.1|helicase[Corynebacteriummatruchotii]_gi|224946756|gb|EEG27965.1|helicaseCterminaldomainprotein/ VKGKAAEHSAE-LYSMDDEAIYGPEFHRLGFGDAVEMGLLTDYKVLVMTVDESV---AAD
gi|746717312|ref|WP_039676231.1|damageinducibleprotein[Corynebacteriumminutissimum]_gi|734991118|gb|KHO29219.1|damageinducibl/1 VKGKAEEHSAE-LASMDDEAIYGPEFYRLGFGEAVDKGLLTDYKVLVMTVEEDI---AAD
gi|908654892|emb|CQD15529.1|SuperfamilyIIDNA/RNAhelicase(modularprotein)[Corynebacteriumstriatum]/11674 VKGKAEEHSAE-LASMDDEAIYGPEFYRLGFGEAVDKGLLTDYKVLVMTVEEDI---AAD
gi|895994446|ref|WP_049064418.1|damageinducibleprotein[Corynebacteriumaurimucosum]/11667 VKGKAEEHSAE-LASMDDEAIYGPEFYRLGFGEAVDKGLLTDYKVLVMTVEEDI---AAD
gi|896123331|ref|WP_049145390.1|damageinducibleprotein[Corynebacteriumaurimucosum]/11669 VKGKAEEHSAE-LASMDDEAIYGPEFYRLGFGEAVDKGLLTDYKVLVMTVEEDI---AAD
gi|817681178|ref|WP_046645422.1|damageinducibleprotein[Corynebacteriumstriatum]_gi|817551679|gb|KKO79868.1|damageinduciblepro/1 VKGKAEEHSAE-LASMDDEAIYGPEFYRLGFGEAVDKGLLTDYKVLVMTVEEDI---AAD
gi|896139239|ref|WP_049159146.1|damageinducibleprotein[Corynebacteriumaurimucosum]/11675 VKGKAEEHSAE-LASMDDEAIYGPEFYRLGFGEAVDKGLLTDYKVLVMTVEEDI---AAD
gi|489943947|ref|WP_003847254.1|helicase[Corynebacteriumammoniagenes]_gi|295968218|gb|EFG81467.1|helicaseCterminaldomainprotei/ IKGKAAEHSAE-LASMDDEAIYGPEFHRLGFGEAVERGLLTDYKVLVMTVDEDV---AAE
gi|516653464|ref|WP_018022453.1|helicase[Corynebacteriumdoosanense]_gi|697778282|gb|AIT61018.1|damageinducibleprotein[Coryneba/ VKGKAAEHSAE-LASMDDEAVFGPEFHRLGFGEAVEKGLLTDYKVLVMTVDEEV---AAD
gi|493894179|ref|WP_006840129.1|helicase[Corynebacteriumlipophiloflavum]_gi|227078819|gb|EEI16782.1|helicaseCterminaldomainpro/ VKGKAAEHSAE-VASMDDEAIFGPEFHRLGFGEAVEAGLLTDYKVLVMTVDEDI---AAD
gi|740831472|ref|WP_038616755.1|damageinducibleprotein[Corynebacteriumpseudotuberculosis]_gi|666103058|gb|AIG06935.1|putativeh/ VKGKAADHFAE-LASMDDEAIYGPEFHRLGFGEAVERGLLTDYKVLVMTVDESV---AAE
gi|518023322|ref|WP_019193530.1|helicase[Corynebacteriumtimonense]/11655 VKGKAADHSAE-LASMDDESIYGPEFHRLGFGEAVERGLLTDYKVLVMTVDEDV---AAE
gi|552767697|ref|WP_023019559.1|MULTISPECIES:hypotheticalprotein[Corynebacterium]_gi|550739013|gb|ERS38609.1|hypotheticalprotei VKDKAADHSAE-LASMDDEGVYGPEFHRLGFGEAVERGLLTDYKVLVMTVDEDV---AAE
gi|652659121|ref|WP_027017812.1|damageinducibleprotein[Corynebacteriumpseudodiphtheriticum]/11644 VKDKAADHSAE-LASMDDEGVYGPEFHRLGFGEAVERGLLTDYKVLVMTVDEDV---AAE
gi|749039309|ref|WP_040085390.1|damageinducibleprotein[Corynebacteriumhumireducens]/11657 VKGKAAEHSAE-LASMDDEAVYGPEFHRLGFGEAVERGLLTDYKVLVMTVDEDV---AAE
gi|740835591|ref|WP_038620874.1|damageinducibleprotein[Corynebacteriumulcerans]_gi|698973854|gb|AIU33664.1|HelicaseCterminald/1 TKGKAKDHSAE-VVSMDDEAIYGPELHRLGFGEAVDKGLLTDYRVLVMTVDEDV---AAE
gi|805307599|ref|WP_046096452.1|damageinducibleprotein[Corynebacteriumulcerans]_gi|803357323|gb|AKA97638.1|Damageinduciblepro/1 TKGKAKDHSAE-VVSMDDEAIYGPELHRLGFGEAVDKGLLTDYRVLVMTVDEDV---AAE
gi|504083555|ref|WP_014317549.1|helicase[Corynebacteriumdiphtheriae]_gi|371584249|gb|AEX47914.1|superfamilyIIDNA/RNAhelicase[Co VKGKAAEHSAE-LASMDDEAIYGPEFHRLGFGEAVEKGLLTDYKVLIMTVDESV---AAE
gi|896182193|ref|WP_049193580.1|damageinducibleprotein[Corynebacteriumjeikeium]/11645 VKGKAAEHSAE-LASMDDEAIYGPEFHRLGFGEAVEKGLLTDYKVLVMTVDESV---AAE
gi|917636459|ref|WP_052205460.1|damageinducibleprotein[Corynebacteriumriegelii]_gi|914295811|gb|AKV59187.1|damageinduciblepro/1 VKTKAEEHSAE-IASMDDESLYGPEFHRLGFGEAVDKGLLTDYKVLVMTVDEDV---AAE
gi|737013522|ref|WP_035009205.1|damageinducibleprotein[Corynebacteriumjeikeium]/11651 VKTKAEEHSAE-LASMDDEAIYGPELHRLGFGEAVDKGLLTDYRVLVMTVDEDV---AAE
gi|737006291|ref|WP_035002094.1|damageinducibleprotein[Corynebacteriumjeikeium]/11658 VKGKAAEHSAE-LASMDDEAVYGPEFYRLGFGEAVDKGLLTDYRVLVMTVDESV---AAD
gi|737010378|ref|WP_035006133.1|damageinducibleprotein[Corynebacteriumjeikeium]/11650 VKGKAAEHSAE-LASMDDEAVYGPEFYRLGFGEAVDKGLLTDYRVLVMTVDESV---AAE
gi|737010473|ref|WP_035006228.1|damageinducibleprotein[Corynebacteriumjeikeium]/11657 IKSKAEERSAE-LASMDDESVYGPEFHRLGFGEAVEKGLLTDYKVLVMTVDEEV---AAD
gi|737006250|ref|WP_035002053.1|damageinducibleprotein[Corynebacteriumjeikeium]/11658 IKNKAEERSAE-LASMDDESVYGPEFHRLGFGEAVEKGLLTDYKVLVMTVDEEV---AAD

gi|923018168|ref|WP_053412254.1|damageinducibleprotein[Corynebacteriumlactis]_gi|922845184|gb|ALA67504.1|helicase[Corynebacter/	VKGKAAEHSAE-LASMDDEGIYGSEFYRLGFGEAVDKGLLTDYKVLVMTVDESV---AAE
gi|923018885|ref|WP_053412970.1|damageinducibleprotein[Corynebacteriumlactis]_gi|922845845|gb|ALA68165.1|helicase[Corynebacter/	VKGKAAEHSAE-LASMDDEAVYGPEFYRLGFGEAVDKGLLTDYKVLVMTVDESV---AAE
gi|512464870|ref|WP_016422869.1|hypotheticalprotein[Corynebacteriumsp.HFH0082]_gi|512047830|gb|EPD47505.1|hypotheticalproteinHM	VKGKAQDHSAE-LASMDDEAIYGPEFYRLGFGEAVEKGLLTDYKVLVMTVDESV---AAD
gi|881012697|ref|WP_048733406.1|damageinducibleprotein[Corynebacteriumurealyticum]/11670	VKGKALDHSAE-LASMDDEAIYGPEFYRLGFGEAVEKGLLTDYKVLVMTVDESV---AAD
gi|755608478|ref|WP_042530519.1|damageinducibleprotein[Corynebacteriumsingulare]_gi|754340239|gb|AJI78730.1|putativehelicase[C/	VKGKAEEHSAE-LASMDDEAIYGPEFYRLGFGEAVDKGLLTDYKVLVMTVDESV---AAQ
gi|491421796|ref|WP_005279593.1|helicase[Corynebacteriumaccolens]_gi|227077180|gb|EEI15143.1|helicaseCterminaldomainprotein[Co/	VKGKAAEHSAE-LTSMDDEGIYGPEFYRLGFGEAVDKGLLTDYKVLVMTVDESV---AAN
gi|550752286|gb|ERS51451.1|hypotheticalproteinHMPREF1282_00033[Corynebacteriumsp.KPL1856]_gi|550759961|gb|ERS58964.1|hypothetic	VKGKAAEHSAE-LTSMDDEGIYGPEFYRLGFGEAVDKDLLTDYKVLVMTVDESV---AAE
gi|497955053|ref|WP_010269209.1|helicase[Corynebacteriumbovis]/11599	VKGAAAEHSAE-LASMDDEAVFGPEFHRLGFGTAVESGLLTDYRVLVLTVDQEL---AAA
gi|860580040|ref|WP_048470569.1|hypotheticalprotein[Mycobacteriumchlorophenolicum]_gi|859098575|gb|KMO76871.1|typeIrestrictione	VKDKAEQHSAE-LVSMDDELRYGPEFHNLSFGDAVERGLLTDYKVLVLTIDESV---IAA
gi|262085905|gb|ACY21873.1|typeIIIrestrictionproteinressubunit[GordoniabronchialisDSM43247]/11632	VREKAEEHSAE-LTSMDDMDIYGPEFHRLSFGDAVDRGLLTDYKVIVLTVDEGL---VAA
gi|495996338|ref|WP_008720917.1|typeIIIrestrictionendonucleasesubunitR[Rhodococcussp.AW25M09]_gi|443412350|emb|CCQ18455.1|TypeI	VKEKAADHSAE-LSSMDDVDIFGPELHRLSFGDAVERGLLTDYKVIVLTVDEDL---VAA
gi|739284636|ref|WP_037147410.1|damageinducibleprotein[Rhodococcusfascians]/11641	VKEKAADHSAE-LSSMDDIDIFGPEMHRLSFGDAVERGLLTDYKVIVLTVDEDL---VAA
gi|739328195|ref|WP_037190574.1|damageinducibleprotein[Rhodococcusfascians]/11643	VKEKAADHSAE-LSSMDDVDIFGPELHRLSFGDAVERGLLTDYKVIVLTVDEDL---VAA
gi|811698335|emb|CMN21041.1|helicase[Mycobacteriumtuberculosis]/11725	IKDRADQHSAE-LVSMDDELTFGPEFHRLSFGEAVERGLLTDYKVMVLTVDQGV---IAP
gi|801577898|emb|CFK23773.1|helicase[Mycobacteriumtuberculosis]/11675	IKDRADQHSAE-LVSMDDELTFGPEFHRLSFGEAVERGLLTDYKVMVLTVDQGV---IAP
MTU18II/11606	IKDRADQHSAE-LVSMDDELTFGPEFHRLSFGEAVERGLLTDYKVMVLTVDQGV---IAP
gi|887719858|emb|CMT27886.1|helicase[Mycobacteriumtuberculosis]/11667	IKDRADQHSAE-LVSMDDELTFGPEFHRLSFGEAVERGLLTDYKVMVLTVDQGV---IAP
gi|621694743|gb|KAS87156.1|hypotheticalproteinAL70_01801[MycobacteriumtuberculosisTKK_03_0115]/11604	IKDRADQHSAE-LVSMDDELTFGPEFHRLSFGEAVERGLLTDYKVMVLTVDQGV---IAP
gi|888128072|emb|CLQ84422.1|helicase[Mycobacteriumtuberculosis]_gi|889917090|emb|CLY86893.1|helicase[Mycobacteriumtuberculosis]	IKDRADQHSAE-LVSMDDELTFGPEFHRLSFGEAVERGLLTDYKVMVLTVDQGV---IAP
gi|884823424|emb|CKL96672.1|helicase[Mycobacteriumtuberculosis]/11466	IKDRADQHSAE-LVSMDDELTFGPEFHRLSFGEAGERGLLTDYKVMVLTVDQGV---IAP
gi|505103239|ref|WP_015290341.1|hypotheticalprotein[Mycobacteriumcanettii]_gi|432162733|emb|CCK60117.1|Conservedproteinofunknow	IKDKADQHSAE-LVSMDDELTFGPEFHRLSFGEAVERGLLTDYKVMVLTVDQGV---IAP
gi|505116250|ref|WP_015303352.1|hypotheticalprotein[Mycobacteriumcanettii]_gi|432158726|emb|CCK56026.1|Conservedproteinofunknow	IKDRADQHSAE-LVSMDDELTFGPEFHRLSFGEAVERGLLTDYKVMVLTVDQGV---IAP
gi|432154735|emb|CCK51974.1|Conservedproteinofunknownfunction(part1)[MycobacteriumcanettiiCIPT140060008]/11588	IKDRADQHSAE-LVSMDDELTFGPEFHRLSFGEAVERGLLTDYKVMVLTVDQGV---IAP
gi|809008027|ref|WP_046295375.1|hypotheticalprotein[Mycobacteriumsp.UM_Kg17]/11624	VKDKAAAHSAE-LATMDSEEIYGPEFHRLSFGDAVERGLLTDYKVLVLTIDESV---IAP
gi|808802633|ref|WP_046284139.1|hypotheticalprotein[Mycobacteriumsp.UM_NZ2]/11624	VRDKAASHSAE-LSSMDDEATYGPEFHRLSFGDAVERGLLTDYKVLVLTIDESV---IAP
gi|750125774|ref|WP_040429852.1|damageinducibleprotein[Corynebacteriumnuruki]/11668	TKSKAEEHSAE-IVSMDDLDTFGPEFHRLSFGEAVDRGLLTDYKVLVLTVDDSV---MAG
gi|602237614|gb|EYT60960.1|damageinducibleprotein[Dietziasp.UCDTHP]/11473	TKAKAEEHSAE-ISSMDDLEIFGPEFHRLGFGEAVDKGLLTDYKVLVLTVDESV---MAP
gi|665897412|ref|WP_031264618.1|damageinducibleprotein[Dietziasp.UCDTHP]_gi|602237599|gb|EYT60945.1|helicase[Dietziasp.UCDTH/11	TKAKAEDHSAE-ISSMDDLEIFGPEFHRLGFGEAVDKGLLTDYKVLVLTVDESV---MAP
gi|502903674|ref|WP_013138650.1|helicase[Segniliparusrotundus]_gi|296181291|gb|ADG98197.1|typeIIIrestrictionproteinressubunit[S	AKGKADEHSAE-IASMDDETIFGPEFYHLPFGEAVDKGLLTDYKVLVLTVDQSL---VAG
gi|780248306|ref|WP_045538930.1|hypotheticalprotein[Gordoniasp.no.9]_gi|778142514|gb|KJR06917.1|hypotheticalproteinUG54_12320[G	LKDKAEEHSAE-VASMDNEELFGPEFHHLGFGEAVERGLLTDYKVLVLTVDQSL---VAG
gi|665897368|ref|WP_031264574.1|damageinducibleprotein[Dietziasp.UCDTHP]_gi|602239137|gb|EYT62483.1|helicase[Dietziasp.UCDTH/11	TKDKAEEHSAE-IASMDDLETYGPEFHRLSFGEAVDRGLLTDYKVLVLTVDESV---MAS
gi|493372923|ref|WP_006329236.1|helicase[Gordoniarhizosphera]_gi|403207917|dbj|GAB88168.1|hypotheticalproteinGORHZ_006_00370[Go	TKTHAAEHSAE-LASMDDEMVFGPVFHRLTFGQAVDQGLLTDYRVLVLTVDESV---MSG
gi|764904870|ref|WP_044487821.1|hypotheticalprotein[Mycobacteriumtuberculosis]_gi|625005353|gb|KBZ57120.1|hypotheticalproteinK8	VKAKADEHSAE-ITSMDDETKYGPEFHRLSFGEAVERGLLTDYKVLVLTVDEEM---VAA
gi|927068212|ref|WP_053778441.1|damageinducibleprotein[Gordoniasp.NB41Y]_gi|926660541|gb|EMP14526.2|damageinducibleprotein[G/11	VKTKADEHSAE-ITSMDDEGEYGPEFHRLSFGEAVERGLLTDYKVLVLTVDEEM---VAA
gi|935514169|ref|WP_054429894.1|damageinducibleprotein[Mycobacteriumimmunogenum]_gi|929801120|gb|KPG29247.1|damageinduciblepr/1	VKAKADEHSAE-ITSMDDESKYGPEFHRLSFGEAVERGLLTDYKVLVLTVDEEM---VAA
gi|941317728|ref|WP_055118574.1|hypotheticalprotein[Mycobacteriumperegrinum]/11657	VKAKADEHSAE-ITSMDDESKYGPEFHRLSFGEAVERGLLTDYKVLVLTVDEEM---VAA
gi|933683673|gb|ALG86377.1|hypotheticalproteinACH46_20120[Gordoniasp.QH11]/11636	VKDKAAEHAAE-LASMDDEDVFGPELHRLSFGTAVERGLLTDYKVIVLTVDEDL---VAA
gi|873553947|emb|CRY84110.1|Predictedhelicase[Nocardiafarcinica]/11688	VKDKAAEHSAE-LSSMDDETRYGPEFHRLSFGEAVERGLLTDYKVIVLTVDEDL---VAA
gi|499525749|ref|WP_011212389.1|helicase[Nocardiafarcinica]_gi|54019339|dbj|BAD60707.1|hypotheticalprotein[NocardiafarcinicaIFM	VKDKAAEHSAE-LSSMDDETRYGPEFHRLSFGAAVERGLLTDYKVIVLTVDEDL---VAA
gi|640109609|ref|WP_024794187.1|damageinducibleprotein[Tomitellabiformata]/11653	VRAKADEHSAE-ITSMDDEDTYGPEFHRLSFGEAVDRGLLTDYKVLVLTVDESV---MAS
gi|640109991|ref|WP_024794334.1|hypotheticalprotein[Tomitellabiformata]/11639	VRAKADEHSAE-ITSMDDEDTYGPEFHRLSFGEAVDRGLLTDYKVLVLTVDESV---MAS
gi|939125496|ref|WP_054831438.1|damageinducibleprotein[Rhodococcuserythropolis]/11669	VKDKAEEHSAE-LTSMDDESVYGPEFHRLSFGAAVDRGLLTDYKVIVLTVDESL---MAA
gi|910258550|ref|WP_050062711.1|hypotheticalprotein[Rhodococcussp.RD6.2]_gi|846871967|emb|CRK49225.1|conservedhypotheticalprote	VKDKADEHSAE-LSSMDDETIYGPEFHRLSFGDAVERGLLTDYKVLVLTVDEDL---VAS
gi|797148189|gb|KJY52358.1|putativeHelicase[Bifidobacteriumasteroides]/11801	AKDQAKRADYT-AYSMDNEAVFGPEFHRLKFGSAVEQGILSDYRVLVLGVAQGQASDLQE
gi|535476570|gb|EFG27357.2|hypotheticalproteinHMPREF9020_00999[ScardoviainopinataF0304]/11827	AQTKAVKNDFT-TFSMDDEDTFGREFHRLKFGDAVAKGILTDYRVLVLGIAESQGTNFAK
gi|737482729|ref|WP_035462496.1|hypotheticalprotein[Alicyclobacillusmacrosporangiidus]/11512	LRNRLSGEGVQ-TYSMDDVEVFGPEFFRYSFRDGVEDGYLADYRIRVVIFNEQN---VQQ
gi|685434354|gb|KGA95679.1|helicase[BacillusalcalophilusATCC27647=CGMCC1.3604]/11575	AKQKADEMSVV-IADMNDSEIYGEELYRIGFGEAIRKGILTDYKVMVLAVDEEV---VAR
gi|945370253|gb|KQU27251.1|helicase[Bacillussp.Leaf13]/11557	AKQKADELSVV-IADMDNTDLYGEEFYRIGFGDAIRQGILTDYKVMVLAVDEEM---IAR
gi|880997015|ref|WP_048719116.1|helicase[Enterococcushirae]/11563	AKEKAEEMSVM-IADMDDESLYGKEFYRLGFGEAVNKGILTDYKVMVLAVDETM---VAR
gi|945356929|gb|KQU14100.1|helicase[Bacillussp.Leaf49]/11547	AMQRAEELSVV-IADMDNPELYGEEFYRIGFGEAIRQGVLTDYKVMVLAVDEEM---ISR
gi|763482876|gb|KIZ53217.1|helicase[Bacillussafensis]/11563	AKKKAEEMSVL-ISDMGNSEIYGEEFYRIGFGDAIKKDILTDYKVMVLAVDEEV---IAR
gi|852223292|ref|WP_048309707.1|helicase[Anaerobacillusmacyae]_gi|850322910|gb|KMM38590.1|helicase[Anaerobacillusmacyae]/11573	AKQKADEMSIV-ISDMNNEDIYGKEFYHIGFGDAVNKGILSDYKVMVLAVDEKE---IAR
gi|739708665|ref|WP_037562619.1|helicase[Sporolactobacillusterrae]/11573	AKKKADEMSVV-IADMSDKDIYGEEFYRIGFGDAVNKGILTDYKVMILAVDEKM---IAR
gi|496687561|ref|WP_009329104.1|MULTISPECIES:helicase[Bacillus]_gi|317390867|gb|EFV71668.1|endonucleaseandmethylaseLlaGI[Bacill	AKQKADEMSVV-IADMNDETIYGEEFYRIGFGDAVNNGILTDYKVMVLAVDEEM---IAR
gi|930213599|gb|KPH74225.1|helicase[Oceanobacilluscaeni]/11569	AKQKADEMSVV-IADMNDESIYGEEFYRIGFGDAVNKGILTDYKVMVLAVDEEM---IAR
gi|516366003|ref|WP_017756036.1|helicase[Bacillussp.ZYK]/11571	AKQKADEMSVV-IADMNDKDIYGEEFYRIGFGDAVNKGILTDYKVMVLAVDEEM---IAR
gi|488909977|ref|WP_002821052.1|helicase[Oenococcusoeni]_gi|399965230|gb|EJN99855.1|endonucleaseandmethylaseLlaGI[Oenococcusoen	AKKKGADSSIE-ISSMDDEALYGQEIYRLGFGTAVSQGILTDYKVMVLTVSEQA---IQK
gi|495019967|ref|WP_007745780.1|helicase[Oenococcuskitaharae]_gi|366983682|gb|EHN59081.1|typeIIIrestrictionenzyme,ressubunit[Oe	AKKKGADSSIE-ISSMDDEALYGQEIYRLGFGTAVSQGILTDYKVMVLTVSEQA---IQK
gi|489666119|ref|WP_003570406.1|typeIIIrestrictionendonuclease[Lactobacilluscasei]_gi|410527834|gb|EKQ02696.1|typeIIIrestrictio	TKKKGEDENIV-IASMDDQDIYGDEIYRLGFGDAVAKGILTDYKVEVLAVDESV---IQR
gi|511447623|gb|EPC78110.1|superfamilyIIDNA/RNAhelicase[Lactobacillusparacaseisubsp.paracaseiLpp221}/11615	TKKKGQEENIV-IASMDDKDLYGDEIYRLGFGDAVSQGILTDYKVEVLAVDEAV---VQR
gi|497700411|ref|WP_010014595.1|helicase[Lactobacilluscoryniformis]/11585	TKKKGQEENIV-IASMDDVDMYGDEIFRLGFGDAVAKGILTDYKVEVLAVDEAV---IQR
gi|489784048|ref|WP_003687939.1|helicase[Lactobacillusmali]_gi|394428539|gb|EJF01080.1|superfamilyIIDNA/RNAhelicase[Lactobacill	TKKKGEDNIV-IASMDDKNIYGEELYRLGFGDAVAQGILTDYKVEVLAVDESV---IQR
gi|446780825|ref|WP_000858081.1|helicase[Staphylococcusaureus]_gi|374396604|gb|EHQ67835.1|typeIIIrestrictionenzyme,ressubunit[S	AKSKKNENSII-ISSMDNEEIYGKEIFRLGFGEAVARGYLTDYKVMVLTVEEEA---MTR
gi|510796658|ref|WP_016171456.1|hypotheticalprotein[Enterococcusdispar]_gi|508167532|gb|EOT44083.1|hypotheticalproteinOMK_00227	AKSKGIEKSVV-IASMDDDSIFGTEIFRLGFGEAVARGYLTDYKVMVLTVEEEA---MTK
gi|612613473|gb|EZS30411.1|hypotheticalproteinW603_02482[StaphylococcusaureusVET0353R]_gi|612881917|gb|EZU93992.1|hypotheticalp	SKSKGIEKSVV-IASMDDESIFGTEIFRLGFGEAVARGYLTDYKVMVLTVEEEA---MTK
gi|488332893|ref|WP_002402278.1|helicase[Enterococcusfaecalis]_gi|306513864|gb|EFM82466.1|helicaseCterminaldomainprotein[Enter/	AKQKAEDSSII-LSSMDNKDIYGEEIFRLGFGQAVSNDILTDYKVMVLAVDEKV---IQK
gi|921166029|ref|WP_053111806.1|helicase[Enterococcusdurans]_gi|915771660|gb|AKX85928.1|helicase[Enterococcusdurans]/11562	AKQKAEDSSIV-LSSMDNKDIYGEEIFRLGFGQAVSNDILTDYKVMVLAVDEKV---IQK

gi|498525358|ref|WP_010825030.1|hypotheticalprotein[Enterococcusfaecalis]_gi|487059631|gb|EOJ86727.1|hypotheticalproteinWOC_005	AKQKAEDSSIV-LSSMDNKDIYGEEIFRLGFGQAVSNDILTDYKVMVLAVDEKV---IQK
gi|498412624|ref|WP_010722070.1|hypotheticalprotein[Enterococcusfaecium]_gi|486634654|gb|EOF75253.1|hypotheticalproteinSGC_0253	AKQKAEDSSIV-LSSMDNKDIYGEEIFRLGFGQAVSNDILTDYKVMVLAVDEKV---IQK
gi|498408534|ref|WP_010718364.1|hypotheticalprotein[Enterococcusfaecium]_gi|486609608|gb|EOF50873.1|hypotheticalproteinSCW_0257	AKQKAEDSSIV-LSSMDNKDIYGEEIFRLGFGQAVSNDILTDYKVMVLAVDEKV---IQK
gi|498407475|ref|WP_010717483.1|hypotheticalprotein[Enterococcusfaecalis]_gi|486594855|gb|EOF36348.1|hypotheticalproteinSCK_005	AKQKAEDSSIV-LSSMDNKDIYGEEIFRLGFGQAVSNDILTDYKVMVLAVDEKV---IQK
gi|514885392|ref|WP_016615344.1|helicase[Enterococcusfaecalis]_gi|514505843|gb|EPH71676.1|helicaseprotein[Enterococcusfaecalis0	AKQKAEDSSII-LSSMDNKDIYGEEIFRLGFGQAVSNDILTDYKVMVLAVDEKV---IQK
gi|325157603|gb|EGC69759.1|helicaseCterminaldomainprotein[EnterococcuscasseliflavusATCC12755]/11561	AKQKAEDSSIV-LSSMDNKDIYGEEIFRLGFGQAVSNDILTDYKVMVLAVDEKV---IQK
gi|895856206|ref|WP_048967668.1|helicase[Enterococcusfaecalis]/11551	AKQKAEDSSIV-LSSMDNKDIYGEEIFRLGFGQAVSNDILTDYKVMVLAVDEKV---IQK
gi|639458836|ref|WP_024636366.1|helicase[Enterococcusfaecium]_gi|658176502|gb|KEI51159.1|helicase[EnterococcusfaeciumUC7256]/1/	AKQKAEDSSIV-LSSMDNKDIYGEEIFRLGFGQAVLNDILTDYKVMVLAVDETA---IKK
gi|488266511|ref|WP_002337719.1|hypotheticalprotein[Enterococcusfaecium]_gi|430581409|gb|ELB19854.1|hypotheticalproteinOIQ_0351	AKQKAEDSSIV-LSSMDNKDIYGDEIFRLGFGQAVSNDILTDYKVMVLAVDEKV---IQK
gi|446353356|ref|WP_000431211.1|helicase[Staphylococcusaureus]_gi|253726329|gb|EES95058.1|helicaseCterminaldomainprotein[Staph/	AKKNAKDKSIL-LSSMDDESKYGEVIYRMGFGQAVSRGILTDYKVMVLAVDETA---IQR
gi|672712068|emb|CDM14336.1|DNAorRNAhelicaseofsuperfamilyII[StaphylococcusepidermidisPM221]/11464	AKKNAKDKSIL-LSSMDDETKYGEVFYRMGFGQAVSHGILTDYKVMVLAVDETA---IQK
gi|896298990|ref|WP_049279905.1|helicase[Staphylococcusaureus]/11571	AKKNAKDKSIL-LSSMDDETKYGEVFYRMGFGQAVSHGILTDYKVMVLAVDETA---IQK
gi|541903432|gb|ERH36190.1|hypotheticalproteinSEQU_00085[StaphylococcusequorumUMCCNS924]/11578	AKKNAKDKSIL-LSSMDDELKYGEVIYRMGFGQAVSHGILTDYKVMVLAVDETA---IQK
gi|488289747|ref|WP_002360955.1|helicase[Enterococcusfaecalis]_gi|256682216|gb|EEU21911.1|endonucleaseandmethylaseLlaGI[Enteroc	AKKNAKEKSIL-ISSMDDENKYGEVFYRMGFGQAVSHGILTDYKVMVLAVDETA---IQK
gi|494392045|ref|WP_007207894.1|helicase[Enterococcusitalicus]_gi|315483798|gb|EFU74284.1|helicaseCterminaldomainprotein[Enter/	AKKNAKEKSIL-ISSMDDESKYGEVFYRMGFGQAVSHGILTDYKVMVLAVDETA---IQK
gi|446350692|ref|WP_000428547.1|helicase[Bacilluscereus]_gi|228727580|gb|EEL78721.1|RestrictionmodificationsystemLlaBIII[Bacil/	AKKNAKDKSIL-ISSMDDESKYGKVFYRMGFGQAVSHGILTEYKVMVLAIDEAA---IQK
gi|488255302|ref|WP_002326510.1|hypotheticalprotein[Enterococcusfaecium]_gi|430480276|gb|ELA57459.1|hypotheticalproteinOGE_0473	AKKSAKDKSIL-LSSMDDVSKFGEVFYRMGFGQAVSHGILTDYKVMVLAVDEAA---IQK
gi|843253770|ref|WP_047916698.1|helicase[Lactococcuspiscium]_gi|802124677|emb|CEN29502.1|EndonucleaseandmethylaseLlaGI,TypeISPr	AKKNAKDKSIL-LSSMDDESKYGEVFFRMGFGQAVSRDILTDYKVMVLAVDEAA---IQK
LlaBIII/11578	AKKNAKDKSIL-LSSMDDESKYGEVFFRMGFGQAVSRDILTDYKVMVLAVDEAA---IQK
gi|499320531|ref|WP_011011023.1|helicase[Listeriainnocua]_gi|16415805|emb|CAC42019.1|pli0021[ListeriainnocuaClip11262]/11569	AKKNAKDKSIL-LSSMDDESKYGEVFFRMGFGQAVSRDILTDYKVMVLAVDEAA---IQK
gi|760026859|ref|WP_043709973.1|helicase[Weissellacibaria]_gi|758337165|gb|KIU20061.1|UvsWhelicase[Weissellacibaria]/11567	AKKNAKEKSIL-LASMDDESKYGEVFFRMGFGQAVSRDILTDYKVMVLAVDEAA---IQK
LlaGI/11570	AKKNAKDKSIL-LSSMDDESKYGEVFFRMGFGQAVSRDILTDYKVMVLAVDEAA---IQK
gi|499988414|ref|WP_011669132.1|helicase[Lactococcuslactis]_gi|116109019|gb|ABJ74139.1|DNAorRNAhelicaseofsuperfamilyII[Lactococ	AKKNAKDKSIL-LSSMDDESKYGEVFFRMGFGQAVSRDILTDYKVMVLAVDEAA---IQK
gi|685938360|ref|WP_031670319.1|helicase[Listeriamonocytogenes]_gi|590403387|gb|EXL23261.1|hypotheticalproteinX846_1956[Listeri	AKKNAKDKSIL-LSSMDDESKYGEVFFRMGFGQAVSRDILTDYKVMVLAVDEAA---IQK
gi|530793219|gb|EQC56240.1|helicase[Lactococcuslactissubsp.cremorisTIFN5]_gi|530793247|gb|EQC56263.1|helicase[Lactococcuslactis	AKKNAKDKSIL-LSSMDDESKYGEVFFRMGFGQAVSRDILTDYKVMVLAVDEAA---IQK
gi|695270006|ref|WP_032492075.1|hypotheticalprotein[Listeriamonocytogenes]_gi|695212872|ref|YP_009081522.1|hypotheticalproteinp	AKKNAKDKSIL-LSSMDDESKYGEVFFRMGFGQAVSRDTILTDYKVMVLAVDEAA---IQK
gi|504101310|ref|WP_014335297.1|helicase[Leuconostocmesenteroides]_gi|859247905|gb|AKO63885.1|helicase(plasmid)[Leuconostocmese	AKKNAQEKSIL-LSSMDDESKYGEVFFRMGFGQAVSHDILTDYKVMVLAVDEAA---IQK
gi|488905190|ref|WP_002816265.1|helicase[Leuconostocmesenteroides]_gi|227351522|gb|EEJ41777.1|helicaseCterminaldomainprotein[L/	AKKNAQEKSIL-LASMDDESKYGEVFFRMGFGQAVSHDILTDYKVMVLAVDEAA---IQK
gi|518121285|ref|WP_019291493.1|MULTISPECIES:hypotheticalprotein[Lactococcus]/11588	AKNTANEQSVV-AASMDDESIYGTQIFRLGFGDAIGRGILTDYKLMVLAVNESG---IRK
gi|754857213|ref|WP_042217902.1|helicase[Lactococcusgarvieae]/11549	AKSKGSEMSVA-LSSMDDEELYGQVIYRLGFGDAITRAVLTDYKLMVLAVDEKT---IQK
gi|640048455|ref|WP_024784762.1|helicase[Streptococcusmutans]/11564	AKKKADDLSVV-ISSMDDESLYGKVFYRLGFGDAISQDILTDYKLMVLAVDESV---VQR
gi|512695361|ref|WP_016476323.1|hypotheticalprotein[Veillonellasp.HPA0037]_gi|512079853|gb|EPD79018.1|hypotheticalproteinHMPREF	AKKKAVANSIV-ISSMDDESLYGKVFYRLGFGDAISQDILTDYKLMVLAVDESV---VQR
gi|636963514|ref|WP_024403747.1|helicase[Streptococcussuis]/11574	AKKKAQDNSIV-ISSMDDEELYGKVFYRLGFGDAISHDILTDYKLMVLAVDETV---VQK
gi|881115312|ref|WP_048802949.1|helicase[Staphylococcuspasteuri]/11573	AKKKAETNSIV-ISSMDDESLYGKEFFRLGFGDAIVNGILTDYKLMVLAVDESV---VQK
gi|446063996|ref|WP_000141851.1|helicase[Streptococcusmitis]_gi|307618725|gb|EFN97864.1|endonucleaseandmethylaseLlaGI[Streptoco	AKKKAEENSSV-ISSMDDENLYGSVFYRLGFGDAISQDILTDYKLMVLAVDETV---VQK
gi|488206609|ref|WP_002277817.1|restrictionmodificationsystemLlaBIII[Streptococcusmutans]_gi|449173033|gb|EMB75626.1|restricti/	AKKKAESNSIV-VSSMDDESLYGKVFYRLGFGDAISHDILTDYKLMVLAVDETV---VQK
gi|640023290|ref|WP_024781621.1|helicase[Streptococcusmutans]/11566	AKKKAESNSIV-VSSMDDESLYGKVFYRLGFGDAISHDILTDYKLMVLAVDETV---VQK
gi|814946581|ref|WP_046393781.1|helicase[Streptococcusuberis]_gi|810600885|gb|KKF57499.1|helicase[StreptococcusuberisB362]/1156	AKKKAEANSIV-VSSMDDERLYGTVFYRLGFGDAISQDILTDYKLMVLAVDESV---VQK
gi|765470762|ref|WP_044720336.1|helicase[Streptococcussuis]/11559	AKKKAEDNSIV-ISSMDDESLYGEVFYRLGFGDAISHDILTDYKLMVLAVDETV---VQK
gi|654507127|ref|WP_027976443.1|helicase[Streptococcusdevriesei]/11558	AKKKAESNSIL-ISSMDDESLYGKVFYRLGFGDAISHDILTDYKLMVLAVDEKV---VQK
gi|530812902|gb|EQC72481.1|DNAorRNAhelicaseofsuperfamilyII[Streptococcussp.HSISS2]/11570	AKKKAEDNSIV-ISSMDDESLYGNVFYRLGFGDAISHDILTDYKLMVLAVDETV---VQK
gi|489158846|ref|WP_003068504.1|helicase[Streptococcusintermedius]_gi|355367114|gb|EHG14827.1|hypotheticalproteinHMPREF9682_002	AKKKAQDNSIV-ISSMDDESLYGKVFYRLGFGDAISHDILTDYKLMVLAVDETV---VQK
RM.Lru27782ORF13200P/11620	AKKKAESNSVE-LASMDDESIYGREIFRLGFGEAVNRGYLTDYKVSVLAVNEKY---VDR
gi|492676546|ref|WP_005919236.1|DNAphosphorothioationsystemrestrictionenzyme[Pediococcusacidilactici]_gi|482517924|gb|EOA08103.	AKKKAHEQSVV-ISSMDDEKLYGQVIFHLGFGDAVNMGYLTDYKVSVLAVSEQY---VNK
gi|498183116|ref|WP_010497272.1|helicase[Lactobacillusacidipiscis]/11563	AKKKAESNSIE-VSSMDDEEKFGKEIFRLGFGDAVSRGYLTDYKVSVLAVSEHY---INK
gi|822100485|ref|WP_046921865.1|helicase[Lactobacillusruminis]_gi|821684442|gb|KLA43321.1|restrictionmodificationsystemLlaBIII/	AKKKAESNSIE-LSSMDDEEKYGKEIFRLGFGDAVSRGYLTDYKVSVLAVNENY---VNK
gi|935565832|ref|WP_054450311.1|helicase[Lactobacilluskunkeei]/11573	AKKKAEENSIV-VSSMDDPEKYGHEIFRLGFGDAVAQGYLTDYKVSVLAVSESY---INK
gi|752296887|ref|WP_041152901.1|helicase[Lactobacilluskunkeei]_gi|751663520|gb|KIM18358.1|helicase[Lactobacilluskunkeei]/11577	AKKKAEENSIV-VSSMDDPAKYGHEIFRLGFGDAVAQGYLTDYKVSVLAVSESY---INK
gi|495745291|ref|WP_008469870.1|helicase[Lactobacillushominis]_gi|394484442|emb|CCI81207.1|RestrictionmodificationsystemLlaBII/	SKKKAKENSIV-VASMDDKDIYGEEIFRLGFGDAVAQGYLTDYKVSVLAVDESY---INK
gi|918672414|ref|WP_052543134.1|helicase[Lactobacillussp.]/11564	AKKKAKENSIV-VSSMDDKSIYGDEIFRLGFGDAVAQGYLTDYKVSVLAVSESY---INK
gi|799141631|ref|WP_045927261.1|helicase[Lactobacilluskullabergensis]_gi|797155335|gb|KJY59264.1|putativerestrictionendonucleas	AKKKAKEKSIV-VSSMDDKDIYGEEIYRLGFGDAVSKGILTDYKVSVLAISESY---INK
gi|504379990|ref|WP_014567092.1|helicase[Lactobacillusjohnsonii]_gi|329666726|gb|AEB92674.1|putativerestrictionendonuclease[Lac	AKKKAKENSIV-VSSMDDKSIYGEEIYRLGFGKAVERGILTDYKVSVLAVSESY---INK
gi|489880179|ref|WP_003783646.1|DNAdamageinducibleprotein[Actinomycesnaeslundii]_gi|399901561|gb|EJN84440.1|typeIIrestriction/	TKARAGQKNAI-LASMDDVETYGEELFRLGFGEAVSRSLLTDYKVLVLTVSEDQ---IAD
gi|496524497|ref|WP_009232780.1|DNAdamageinducibleprotein[Actinomycessp.oraltaxon849]_gi|365265413|gb|EHM95177.1|hypotheticalp/	TKARAGQKNAI-LASMDDVETYGEELFRLGFGEAVSRSLLTDYKVLVLTVSEDQ---IAD
gi|915397782|ref|WP_050783167.1|damageinducibleprotein[Actinomycesoris]/11694	TKARAGQKNAI-LASMDDVETYGEELFRLGFGEAVSRSLLTDYKVLVLTVSEDQ---IAD
gi|502925248|ref|WP_013160224.1|helicase[Propionibacteriumfreudenreichii]_gi|296921272|emb|CBL55824.1|helicase[Propionibacteriu	VKKKADEHSAL-LTSMDDEATFGPEFHRLGFGQAVEKGLLTDYKVMILCVQNDA---IAD
gi|732164352|emb|CEG87481.1|helicase[Propionibacteriumfreudenreichii]/11618	VKQKADEASAV-LTSMDDEAVFGPEFWRLGFGQAVEENLLSDYKVMVLVVENDA---IAE
gi|739047402|ref|WP_036925058.1|damageinducibleprotein[Propionibacteriumthoenii]/11596	VKKKADDAAAV-LTSMDDEAMFGPVFHRLGFGQAVERGLLSDYKVMILAVANDA---IDP
gi|739112839|ref|WP_036982533.1|damageinducibleprotein[Propionibacteriumjensenii]/11594	VKKKADDAAAV-LTSMDDQAMFGPVFHRLGFGQAVERGLLSDYKVMILAVANDA---IDP
gi|918023558|ref|WP_052314121.1|damageinducibleprotein[Nocardiathailandica]/11612	VTKKAIENDAV-LADMNREDQFGPELHRLGFGEAVEKGLLTDYKVLVLSVEEEY---VAA
gi|498380271|ref|WP_010694427.1|DNAdamageinducibleprotein[Saccharopolysporaspinosa]/11605	ARRKADDANAA-LADMDKEALFGPELHRLGFGEAVEERLLTDYKVVVLAVDEQY---VSE
gi|749991459|ref|WP_040339809.1|damageinducibleprotein[CandidatusBlastococcusmassiliensis]/11597	VRRKATDADAV-LADMGDQDTFGPELHRLGFGQAVEADLLTDYKVLVLAVDEKY---VAE
gi|754177801|ref|WP_041796231.1|damageinducibleprotein[Modestobactermarinus]/11592	VRRKATDADAV-LADMGDEGVFGPELHRLGFGQAVEADLLTDYKVLVLAVDEKY---VAE
gi|504188248|ref|WP_014375350.1|DNAdamageinducibleprotein[Blastococcussaxobsidens]_gi|378782790|emb|CCG02456.1|putativeHelicas/	VKRKATDADAV-LADMGNADVFGPELHRLGFGQAVEADLLTDYKVLVLAVDEKY---VAE
gi|946476713|gb|KRF26798.1|damageinducibleprotein[Phycicoccussp.Soil803]/11609	VKEKATTNDAV-LVSMDDESLYGPVLHRLGFGEAVEKDLLTDYKVLVLKVDESF---VAE
gi|700176672|gb|KGN37509.1|DNAdamageinducibleprotein[KnoelliasubterraneaKCTC19937]/11633	VKEKADNAAAV-LVSMDDESLYGPVLHRLGFGEAVERDLLTDYKVLVLKVDESF---VAE
gi|700168841|gb|KGN29914.1|hypotheticalproteinN802_10215[KnoelliasinensisKCTC19936]/11631	VKEKATNNEAV-LVSMDDESLYGPVLHRLGFGEAVEKDLLTDYKVLVLKVDESF---VAE

gi|946426241|gb|KRE76610.1|damageinducibleprotein[Arthrobactersp.Soil763]/11606	TKTKAGEAAAV-LASMDNEETFGPEFYRLGFGDAVAKGLLTDYKVLVLAVDGKT---VAS
gi|517318525|ref|WP_018500927.1|hypotheticalprotein[Frankiasp.BCU110501]/11592	SKTKAGDANAV-LASMDDEELFGPEFHRLGFGEAVEQGLLADYKVLVLTVDEES---VAR
gi|499072429|ref|WP_010851327.1|Helicase[Tetrasphaeraelongata]_gi|478756579|emb|CCH71501.1|putativeHelicase[Tetrasphaeraelongat	SKAKAGEASAV-LASMDNEDLFGEELHRLGFGEAVAQDLLADYKVLVLAVDETA---VSR
gi|750080666|ref|WP_040385350.1|damageinducibleprotein[Demetriaterragena]/11625	TKKKAAEGAAQ-IASMDDEEKFGPELYRLGFGEAVEKNLLTDYRVLILTVDEKY---VTS
gi|752558292|ref|WP_041229523.1|damageinducibleprotein[Dermacoccusnishinomiyaensis]_gi|664689315|gb|AIF41904.1|damageinducibl/1	AKAKARDGAAV-IASMDDESIFGPEFHRLSFGQAVSGDLLTDYRVVILSVDEAY---VAK
gi|829089213|ref|WP_047310701.1|damageinducibleprotein[Dermacoccussp.PE3]_gi|828384812|gb|KLO63770.1|damageinducibleprotein[D/1	SKKKALDGAAE-LASMDDEAKFGPELHRLGFGEAVEKNLLTDYRVLILAVDEEY---VSK
gi|748756635|ref|WP_040014750.1|damageinducibleprotein[Dermacoccussp.Ellin185]/11596	SKKKAFDGAAE-LASMDDESKFGPELHRLGFGEAVEKNLLTDYRVLILAVDEEY---VTR
gi|916534724|ref|WP_051141848.1|damageinducibleprotein[Actinomycesneuii]/11632	TRQKATEKDAV-LASMDDQTIYGPIFHRLSFGQAVGEGLLTDYKVVVLAISEDQ---VSR
gi|921140030|ref|WP_053090276.1|damageinducibleprotein[Rothiamucilaginosa]/11616	TKFQASEKAAV-LCSMDDMDTFGPVFYRIGFGEAVEKGLLTDYKVLVLGVSESQ---IAT
gi|896406751|ref|WP_049343749.1|damageinducibleprotein[Rothiamucilaginosa]/11647	TKFQASEKAAV-LCSMDDVDTFGVPFYRIGFGEAVEKGLLTDYKVLVLGVSESQ---IAT
gi|723608234|gb|AIY01610.1|putativehelicase[Arthrobactersp.PAMC25486]/11613	TKDAAKERDAE-LCSMDDLEIFGPVFYRIGFGEAVTKGLLTDYKVLVLGVDEHQ---VAG
gi|946394527|gb|KRE45160.1|damageinducibleprotein[Arthrobactersp.Soil736]/11616	TKNAAMEKDAI-LCSMDDETLYGPVFYRIGFGEAVTKKLLADYKVLVLGVEETQ---VVS
gi|515765304|ref|WP_017197904.1|hypotheticalprotein[Arthrobactersp.M2012083]/11648	TKNKALERDAI-LCSMDDETMYGPVFYRIGFGEAVTKKLLTDYKVLVLGVDETQ---VVS
gi|945315587|gb|KQT73127.1|damageinducibleprotein[Microbacteriumsp.Leaf436]/11612	TRKKAGEIGAK-LADMDDEAVYGPEFHRLGFGQAVSMGRLTDYKVLVLAVDETF---VAR
gi|759908497|ref|WP_043594599.1|damageinducibleprotein[Frigoribacteriumsp.MEB024]_gi|758271359|gb|KIU03346.1|damageinduciblep/1	VRMEADEKAAV-LVSMDDEATYGPVFHRLGFGEAVERDLLTDYRVLVLTIDEDS---ISS
gi|945072850|gb|KQR34237.1|hypotheticalproteinASF75_00055[Curtobacteriumsp.Leaf154]/11597	AKNQADENSAV-IYSMDNESVFGPVFHRLGFGEAVERDLLSDYRVLVLTIDEDS---IGS
gi|551263152|ref|WP_022883363.1|DNAdamageinducibleprotein[Glaciibactersuperstes]/11621	VRTKASEKNAV-LVSMDDESTFGPTFHRLGFGEAVERDLLTDYRVLVLAVDEET---IGA
gi|945452341|gb|KQV08491.1|hypotheticalproteinASC63_10170[Leifsoniasp.Root112D2]/11623	AKTKASEKLAV-VVSMDDESRFGPEFHRLGFGAAVERGLLSDYRVLVLAVDEGN---VNA
gi|742759483|gb|KIC69912.1|damageinducibleprotein[Kocuriarhizophila]/11610	TRKKAEENSVV-VASMDDAALFGPEFHRLGFGEAVEGGLLSDYKVLVLAVDEAA---ITR
gi|737919995|ref|WP_035885304.1|damageinducibleprotein[Kocuriarhizophila]/11611	TRKKAEENSVV-VASMDDEALFGPEFHRLGFGEAVEGGLLSDYKVLVLAVDEAA---ITR
gi|737997701|ref|WP_035959325.1|damageinducibleprotein[Kocuriamarina]_gi|725823884|gb|KHE75603.1|damageinducibleprotein[Kocur/1	TRKKAEENSVV-VASMDDEALFGPEFHRLGFGEAVEGGLLSDYKVLVLAVDEAA---ITR
gi|664521070|ref|WP_031037650.1|damageinducibleprotein[Streptomycessp.NRRLWC3774]/11624	TKTDAKNAEAV-LASMDNQQMFGPEFHRLGFGQAVEQGLLTDYKVLILTIDEGV---VAK
gi|500101034|ref|WP_011777041.1|DNAdamageinducibleprotein[Arthrobacteraurescens]_gi|119951547|gb|ABM10457.1|putativeHelicase[A/	SKTKAAENDVK-VFSMDDEAAYGPEFHHLGFGEAVERNLLSDYKVLVLAVNEES---VNK
gi|916871953|ref|WP_051479009.1|damageinducibleprotein[Arthrobactersp.H5]/11588	SKTKAAENDVK-VFSMDDEVTYGPEFHHLGFGEAVEKDLLADYKVLVLAVNEES---VNK
gi|612084649|gb|EZP43369.1|TypeIIIrestrictionenzyme,ressubunit[Micrococcusluteus]/11625	AKAKAAQDQVT-VYSMDDESIYGPEFHRLGFGKAVEQGHLTDYKVLILAVDTNA---IGA
gi|759739708|ref|WP_043450422.1|damageinducibleprotein[Arthrobacterphenanthrenivorans]_gi|742758264|gb|KIC68716.1|damageinduc/1	AKAKAGQDDVK-MYSMDDEAVYGPEFHHLGFGKAVEMGHLADYKVLVLAVDEEA---VSK
gi|636845448|ref|WP_024366823.1|damageinducibleprotein[Arthrobactersp.TB26]/11605	SKAKAAENDVA-VYSMDDVAVYGPEFHHLGFGKAVEMGHLADYKVLVLAVDQDA---VSR
gi|500009025|ref|WP_011689743.1|DNAdamageinducibleprotein[Arthrobactersp.FB24]_gi|116613051|gb|ABK05760.1|typeIIIrestrictionen/	SKAKAAENDVA-VYSMDDVAVYGPEFHHLGFGKAVEMGHLSDYKVLVLAVNEEA---VSR
gi|821249847|gb|KKZ12083.1|hypotheticalproteinTQ37_06090[CandidatusSynechococcusspongiarum15L]/11436	ARNRAGECAAM-LCSMDDEERYGPMLHETRFGDAVDRGLLSDYRVIVLTVPESL---AAR
gi|739446902|ref|WP_037306885.1|damageinducibleprotein[Ruegeriahalocynthiae]/11592	ARAKAADAAVE-LCDMDDEALYGPTFFTRGFSWAVENGMLTDYKVIVLAMDETV---VSR
gi|640620081|ref|WP_025048131.1|damageinducibleprotein[Sulfitobactermediterraneus]_gi|751956650|gb|KIN77213.1|TypeIIIrestricti/	ARTKAAEAAVT-LCDMDDEEIYGPTFFTRGFSWAVENGMLTDYKVIVLAMDETL---VSR
gi|740247634|ref|WP_038088583.1|damageinducibleprotein[Thiobacillusprosperus]_gi|684049748|gb|KFZ90853.1|putativehelicase[Thio/	SKTKAGEKNAV-LYSMDDEATFGTEFYRLSFGQAVQRDLLAEYKVLIVAVDEDR---MAS
gi|518420321|ref|WP_019590528.1|hypotheticalprotein[Thioalkalivibriosp.ALE20]/11603	SKSKAEEKDAE-IFSMDDEAAYGPEFHRLSFGKAVERDLLTDYKVLIVAVQEET---MAS
gi|500156826|ref|WP_011831496.1|DNAdamageinducibleprotein[Methylibiumpetroleiphilum]_gi|124261892|gb|ABM96881.1|adeninespecifi/	SKTKAGEREAV-LFSMDDEETFGPEFYRLGFGKAVSLDLLSEYKVLIVAVEEEA---MAG
gi|769978537|ref|WP_045094522.1|damageinducibleprotein[Legionellafallonii]_gi|698841736|emb|CEG55682.1|Putativeproteinwithboxh/	SKTRANEANAS-LASMDDEALFGKELYRLGFGKAVEHNLLTDYKVLIVAVKESE---MAN
gi|553752541|ref|WP_023085344.1|hypotheticalprotein[Pseudomonasaeruginosa]_gi|552201952|gb|ERU62653.1|hypotheticalproteinQ088_0	SKTKANNADAA-LFSMDDEATFGPEFYRLGFGKAVELDLLSEYKVLIVAVREAE---MAK
gi|880980418|ref|WP_048704461.1|damageinducibleprotein[Thauerahumireducens]_gi|875132785|gb|KMU70944.1|damageinducibleprotein/1	SKTKANNSDAV-LFSMDDEETFGPAFYRLGFGKAVELDLLSEYKVLIVAVRENE---MAK
gi|740135732|ref|WP_037982815.1|damageinducibleprotein[Thauerasp.27]/11603	SKTKANNSDAV-LFSMDDEETFGPAFHRLGFGKAVELDLLSEYKVLIVAVRENE---MAK
gi|738017371|ref|WP_035978332.1|damageinducibleprotein[Burkholderiaglumae]/11380	SKTKANKADAV-LYSMDDEAVYGQEFYRIGFGKAVELGCLSEYKVLIVAVKETE---MAN
gi|498506003|ref|WP_010806671.1|typeIIIrestrictionenzyme,ressubunit[Pandoraeasp.SD62]_gi|490314561|gb|EON12879.1|typeIIIrestri/	SKTKANKADAV-LYSMDDEDIYGQEFYRIGFGKAVELGCLSEYKVLIVAVKETE---MAN
gi|500117748|ref|WP_011793753.1|DNAdamageinducibleprotein[Acidovoraxcitrulli]_gi|120587742|gb|ABM31182.1|typeIIIrestrictionenz/	SKTKANEADAV-LFSMDDVETYGQEFYRLGFGKAVTRDLLTEYKVLIVAVKEAE---MAK
gi|651617328|ref|WP_026611444.1|damageinducibleprotein[Methylocaldumszegediense]/11601	SKTKANEAEAV-LYSMDDEATYGAELYRLGFGKAVEKGLLTEYKVLIVAVKENE---MAK
gi|737210208|ref|WP_035194994.1|damageinducibleprotein[Acidithiobacillusferrivorans]_gi|669953220|emb|CDQ11755.1|Adeninespecif/	ASRKAEEKGHL-VYSMSNEAHFGPVFHRLSFAEAVDRDLLSDYKVLILAVDQES---ILQ
gi|935697386|ref|WP_054506180.1|damageinducibleprotein[Achromobactersp.]_gi|928716153|emb|CUK12836.1|Hefnuclease[Achromobacter/	SKAKAEESEIV-VYSMDDQATFGPVFHRLRFDEAVRRDLLSDYKVLIIAVDELH---VNE
gi|429502517|gb|ELA00827.1|adeninespecificDNAmethyltransferase[Cupriavidussp.HMR1]/11599	SKAKAEESEIV-VYSMDDLATFGPVFHRLRFDEAVRRDLLSDYKVLIIAVDELH---VNE
gi|506356642|ref|WP_015876361.1|DNAdamageinducibleprotein[Burkholderiaglumae]_gi|237877158|gb|ACR29491.1|AdeninespecificDNAmet/	SKTKAEESDIQ-VFSMDDAATYGPVFHRLRFDEAVKRDLLSDYKVLVIAVDELH---VNQ
gi|696406787|ref|WP_032978003.1|damageinducibleprotein[Bordetellahinzii]_gi|625335076|gb|KCB49343.1|typeIIIrestrictionenzyme,r/	SKSKAEENDVQ-VFSMDDVATFGPVFHQLRFDEAVRRDLLSDYKVLVIAVDELH---VSR
gi|817529125|gb|KKO72587.1|damageinducibleprotein[Kerstersiagyiorum]/11592	SKSKAEENDVQ-VFSMDDVATFGPVFHQLRFDEAVRRDLLSDYKVLVIAVDELH---VSR
gi|737048910|ref|WP_035044118.1|damageinducibleprotein[Aquabacteriumsp.NJ1]_gi|699052896|gb|KGM38706.1|damageinducibleprotein/1	SKSKAEESNVQ-VFSMDDISTFGPEFHRLRFDEAVKRDLLSDYKVLVIAVDELH---VSQ
gi|822492519|ref|WP_046933319.1|MULTISPECIES:damageinducibleprotein[Xanthomonas]_gi|821703525|gb|KLA61196.1|damageinduciblepr/1	SKSKAEESNVQ-VYSMDDVATFGPEFHRLRFDEAVKRDLLSDYKVLVIAVDELH---VSQ
gi|751252861|ref|WP_040963740.1|damageinducibleprotein[Xanthomonasfuscans]_gi|749808363|gb|KIJ03887.1|damageinducibleprotein[/1	SKTKAEESNVQ-VYSMDDVSTFGPVFHRLRFDEAVRRDLLSDYKVLVIAVDELH---VSQ
gi|496774980|ref|WP_009368792.1|DNAdamageinducibleprotein[Bilophilasp.4_1_30]_gi|345039902|gb|EGW44205.1|hypotheticalproteinHM/	SKDRAKDLGTE-VFSMDDPQKFGPLFHRLRFDEAVRRDLLSDYKVLIIAVDERH---VNL
gi|499241912|ref|WP_010939452.1|DNAdamageinducibleprotein[Desulfovibriovulgaris]_gi|46580580|ref|YP_011388.1|adeninespecificDN/	SKSKAEKRDIA-VFSMDDEATFGKEFHRLRFDEAVRRDLLSDYKVLIIAVEEKH---VTL
gi|504274913|ref|WP_014462015.1|DNAdamageinducibleprotein[Bacillusmegaterium]_gi|345446919|gb|AEN91936.1|AdeninespecificDNAmet/	TKTKADEHNAL-LCSMDNREIFGEEFHYLGFGEAVNRGLLTDYKVMVLAVDETY---IHS
gi|504895966|ref|WP_015083068.1|typeIIrestrictionenzymeandmethyltransferasesubtypeG[Anabaenasp.90]_gi|413972424|gb|AFW96512.1|t	AKNQAQENDVL-LCSMDDEDIYGKEFHRLGFGEAVSADLLSDYKVMVLAVDKNF---VNA
gi|518327765|ref|WP_019497972.1|hypotheticalprotein[Calothrixsp.PCC7103]/11600	AKTQAQENDAT-LCSMDDESIYGTEFHRLGFSEAVSSGLLTDYKVMVLAVDEKY---VST
gi|652765503|ref|WP_027079917.1|damageinducibleprotein[Luteimonasmephitis]/11593	SKSKAEEGGAE-IASMDDLNLFGPEFHRLGFGEAVGLDLLSDYKVMVLAVDEKS---VSK
gi|896539951|ref|WP_049442123.1|damageinducibleprotein[Stenotrophomonasmaltophilia]/11591	SKTKADEAGAE-IASMDDETKFGPEFHRLGFGEAVSQGLLADYKVMVLAVDEKS---VTK
gi|500217900|ref|WP_011888043.1|DNAdamageinducibleprotein[Geobacillusthermodenitrificans]_gi|134267954|gb|ABO68149.1|Conserved/	SKTKARDANVQ-LYSMDDETVYGPEFHRLNFSKAVELGILSDYKVVILAVDEKH---VLR
gi|696570298|ref|WP_033102014.1|damageinducibleprotein[Thermoactinomycesdaqus]/11594	SKSKAKEANVE-LYSMDDESIYGPEFHRLNFSKAVELGILSDYKVIILAVDEQH---VLD
gi|251772067|gb|EES52637.1|putativehelicase[Leptospirillumferrodiazotrophum]/11618	AKSKARQSEFQ-LYSMDDPEVYGPEFHRLGFGEAVKEDLLSDYKVLVLMVDQED---ISR
gi|738124101|ref|WP_036082108.1|damageinducibleprotein[Leptospirillumferriphilum]_gi|685243306|gb|KGA94177.1|putativehelicase[/	AKSKARQSEIQ-MYSMDDPEVYGPEFHRLGFGEAVKDDLLTDYKVLVLVVDQED---ISK
gi|558699223|ref|WP_023524766.1|helicase[Leptospirillumsp.GroupII'CF1']_gi|206603764|gb|EDZ40244.1|Putativehelicase[Leptospiri/	AKSKARQSEFQ-LYSMDDPEIYGPEFHRLGFGEAVKENLLSDYKVLVLMVDQED---ISR
gi|406774462|gb|AFS53887.1|putativehelicase[LeptospirillumferriphilumML04]/11610	AKSKAKENDIQ-VYSMDDPAIYGPEFHRLGFGEAVKKGLLSDYKVLVLVMDQQD---ISR
gi|740719463|ref|WP_038504749.1|damageinducibleprotein[Leptospirillumferriphilum]_gi|640838125|gb|AIA30173.1|damageinduciblep/1	AKSKARENDIQ-VYSMDDETVYGPEFHRLGFGEAVKRGLLSDYKVLVLVMDQQD---ISR
gi|517810766|ref|WP_018980974.1|hypotheticalprotein[Saccharibacterfloricola]/11733	TKQKASQKAMV-LASMDDEKTYGPVVFAYSFRQAVDDGYLCDYRLVVLRIQQSD---VRK
gi|502471675|ref|WP_012798533.1|helicase[Slackiaheliotrinireducens]_gi|256791761|gb|ACV22431.1|predictedhelicase[Slackiaheliotr	IKKVAKADDYV-VASMDDETVYGPEFHRLTFGQAVDEQLLTDYKVIALTVSESM---VDA

```
gi|657871713|ref|WP_029576297.1|hypotheticalprotein[Bifidobacteriumthermacidophilum]_gi|673007521|gb|KFJ02535.1|helicase[Bifido    AKRKAREGVVP-IASMDDEKTYGPIAYRLGFGQAVERGLLTDYKIVVMGVTANM---VRV
gi|673004757|gb|KFI99791.1|DNAhelicaserestrictionenzymetypeIIIRessubunit[Bifidobacteriumthermacidophilumsubsp.porcinum]/11682    AKRKAREGVVP-IASMDDEKTYGPIAYRLGFGQAVERGLLTDYKIVVMGVTANM---VRV
gi|917316092|ref|WP_051922804.1|hypotheticalprotein[Bifidobacteriumstellenboschense]_gi|673003774|gb|KFI98812.1|helicase[Bifido    AKSKAKEGAIE-IASMDDENVYGRIAYRLSFGKAVDLGLLTDYKIVVMQVAESM---MGS
gi|705415694|ref|WP_033502332.1|hypotheticalprotein[Bifidobacteriumpseudolongum]_gi|672982411|gb|KFI77685.1|DNAdamageinducible/    AKNKAADGAVE-IASMDDERVYGRVAYRLTFGKAVAAGLLTDYKIVIMQVGEDQ---MGR
gi|916591418|ref|WP_051198509.1|hypotheticalprotein[Bifidobacteriumsp.AGR2158]/11704    AKNKAKAGAVE-IASMDNVDVYGRVAYTLSFGKAVQLGLLTDYKIVVMQVAEDQ---MSR
gi|772242982|gb|AJW78255.1|hypotheticalproteinVO01_03165[Clavibactermichiganensissubsp.insidiosus]/11642    VKNLARQKEAE-LISMDDESLFGPELYRIGFDEAVRAQLLTDYKVIVLGVSEDQ---IVE
gi|916649818|ref|WP_051256909.1|damageinducibleprotein[Actinomycesgraevenitzii]/11677    AKSKASQKDVI-LTSMDDKDTYGPVFFRLGFGQAVKENLLTDYKVIILTVSEEE---VSQ
gi|496504578|ref|WP_009212861.1|helicase[Actinomycessp.oraltaxon180]_gi|315312695|gb|EFU60776.1|helicase[Actinomycessp.oraltaxo    TKNTASEKDVI-LTSMDDQETYGPVFFRLGFGRAVKENLLTDYKVIILTVSEEE---VSG
gi|494862206|ref|WP_007588306.1|helicase[Actinomycessp.ICM39]_gi|398362799|gb|EJN46470.1|typeIIrestrictionenzyme,ressubunit[Ac    AKNRASEKDAI-LTSMDDQETYGPVFFRLGFGQAVKENLLTDYKVIILTVSEEE---VSQ
gi|881057111|ref|WP_048774974.1|damageinducibleprotein[Sanguibacterkeddieii]/11697    AKNRASEKDAI-LTSMDDQETYGPVFFRLGFGQAVKENLLTDYKVIILTVSEEE---VSQ
gi|881020459|ref|WP_048740808.1|damageinducibleprotein[Kytococcussedentarius]/11704    AKNRASEKDAI-LTSMDDQETYGPVFFRLGFGQAVKENLLTDYKVIILTVSEEE---VSQ
gi|881052313|ref|WP_048770555.1|damageinducibleprotein[Cellulomonasflavigena]/11705    AKNRASEKDAI-LTSMDDQETYGPVFFRLGFGQAVKENLLTDYKVIILTVSEEE---VSQ
gi|490108682|ref|WP_004009424.1|helicase[Mobiluncuscurtisii]_gi|315490465|gb|EFU80089.1|helicaseCterminaldomainprotein[Mobilun/    VKNIAKERDAV-LTSMDDETIYGPVFHRLGFGRAVSLGLLTDYKVVVLAVPEDQ---ITR
gi|490111935|ref|WP_004012603.1|helicase[Mobiluncusmulieris]_gi|227843812|gb|EEJ53982.1|helicaseCterminaldomainprotein[Mobilun/    TKKAAREKDAV-LSSMDDQAIYGPVFHRLGFGQAVAGGLLTDYKVVVLQVPEDQ---ITS
gi|490114228|ref|WP_004014848.1|helicase[Mobiluncusmulieris]_gi|269934432|gb|EEZ90993.1|helicaseCterminaldomainprotein[Mobilun/    IKKAAREKDAV-LSSMDDQAIYGPVFHRLGFGQAVAGGLLTDYKVVVLQVPEDQ---ITS
gi|493333551|ref|WP_006290636.1|helicase[Parascardoviadenticolens]_gi|315119487|gb|EFT82620.1|helicaseCterminaldomainprotein[P/    VKQKAKAAEAV-LCDMDDESVYGPVAASISFATAVEQKLLCDYRVVVLAVNEDG---IPS
gi|495817067|ref|WP_008541646.1|helicase,partial[Sutterellaparvirubra]_gi|378598583|gb|EHY31712.1|helicaseprotein,partial[Sutte    AKKQSEEGDVV-LYSMDDEAVFGKTFHTISFSEAVRLKSLVDYKVIVLAVDESV---VKN
gi|491572696|ref|WP_005430278.1|helicase[Sutterellawadsworthensis]_gi|319805116|gb|EFW01943.1|DNAhelicaserestrictionenzymeTypeI    VKEQKNNGEVV-LYSMDDEQIYGPTFHTITFSQAVALGSLVDYKVIVLSVEENI---LKE
gi|748686321|ref|WP_039944445.1|hypotheticalprotein[Thermicanusaegyptius]/11573    LKEKLEEMEIE-YFSMDNETIYGKEFFRYGFGQAVDDGHLCDYKVIVFAVSENE---IQT
gi|916875698|ref|WP_051482754.1|hypotheticalprotein[Thioalkalivibriosp.HK1]/11366    TKTKAGENEVR-LFSMDDPKDYGPEFHRLSFSDALNQGLLSDYRVVVLAFSEAA---ATR
gi|821248604|gb|KKZ10960.1|hypotheticalproteinTE42_09075[CandidatusSynechococcusspongiarumSP3]/11598    ARNRAGEFAAA-LCSMDDEERYGLMLHETRFGDAVERGLLSDYRVIVLTVPESL---AAG
gi|737409843|ref|WP_035390956.1|hypotheticalprotein[Ferrimicrobiumacidiphilum]_gi|765544266|gb|KJE76241.1|typeIrestrictionenzym    AKSKATEAEVI-LYSMDNEAHFGKEFHRLGFGEAVRRELLSDYQVVILAIDEAY---AAQ
gi|654906925|ref|WP_028358281.1|damageinducibleprotein[Brackiellaoedipodis]/11679    SKQ--SESIQV-LYSMDDKEHFGETFHVVSFNDAVQQGLLVDYKVIVLAIDEEH---ISG
gi|502809901|ref|WP_013044877.1|hypotheticalprotein[CandidatusPuniceispirillummarinum]_gi|292663146|gb|ADE38247.1|hypotheticalp    SKQKADDQNIG-IYSMDDEAEFGTVLYRLSFSDAITEGLLSDYRVIVLNMSESH---VSQ
gi|251771370|gb|EES51950.1|typeIIIrestrictionenzyme,ressubunit[Leptospirillumferrodiazotrophum]/11628    TKKRASENEAL-VYSMDDESRFGPTLHTLSFGNAVQQGILSDFKVIIVAMTDER---MQE
gi|498328480|ref|WP_010642636.1|DNAdamageinducibleprotein[Acidithiobacillusthiooxidans]/11627    TRKKADEKEAV-LYSMDDEAIYGPEFFNLSFGEATSRGILAEYKVIIVAMREED---MAD
gi|647633864|ref|WP_025906698.1|damageinducibleprotein[Prochlorococcussp.scB245a_519B7]/11622    AIKKEEEGKVN-LFPMDKEEIYGKTLFYRSFGWAVENNLLTDYKVVVLNLNEQL---VSD
gi|647619599|ref|WP_025893729.1|damageinducibleprotein[Prochlorococcussp.scB241_527N11]/11621    AKRREDEGEVN-LASMDDEKKFGKNLFYRSFGWAVENNLLTDYKVVVLMMDEQI---VSD
gi|755034887|ref|WP_042387498.1|hypotheticalprotein[Clostridiumsp.CL6]/11606    VKNKAKDINAE-LCSMDNEEKYGPEFHNLTFSDAVADNLLTDYKVLVLAIDQGY---VEL
gi|498139798|ref|WP_010453954.1|helicase[SuccinivibrionaceaebacteriumWG1]/11662    VKNKASLNDCV-LCSMDDENIYGKEIYHVGFSYAVEHNLLTDYKVLVLTVSEDD---LPG
gi|527501386|gb|EPT34571.1|typeIIIrestrictionenzyme,ressubunit[Bacteroidetesbacteriumoraltaxon272str.F0290]/11789    IKETAKEKDML-LWSMDNEDFFGKEFYRIGFGEAVDLKLLTDYKVLVLTLSEED---LSE
gi|496047275|ref|WP_008771782.1|helicase[Bacteroidessp.2_1_33B]_gi|262296443|gb|EEY84373.1|helicaseCterminaldomainprotein[Bact/    AKATAKDKDLV-LWSMNNPDYYGEEFFRIGFGRAVREGLLTDYKVLVLTISEDD---IPD
PDI8503III/11664    AKATAKDKDLV-LWSMNNPDYYGEEFFRIGFGRAVREGLLTDYKVLVLTISEDD---IPD
gi|738719431|ref|WP_036613715.1|helicase[Parabacteroidesdistasonis]_gi|649542518|gb|KDS49387.1|restrictionendonucleasefamilypro    AKATAKDKDLV-LWSMNNPDYYGEEFFRIGFGRAVREGLLTDYKVLVLTISEDD---IPD
gi|659417033|gb|KEJ86133.1|hypotheticalproteinHMPREF1002_00341[Porphyromonassp.31_2]/11656    AKATAKDKDLV-LWSMNNPDYYGEEFFRIGFGRAVREGLLTDYKVLVLTISEDD---IPD
gi|567456807|gb|ETK11358.1|hypotheticalproteinT235_15845[Tannerellasp.oraltaxonBU063isolateCell8/11]/11433    AKIKASKNDCI-LCSMDDEAIYGKEFHRVNFSYAVQNGILTDYKVLVLTVSEDM---IPE
gi|545440279|ref|WP_021677877.1|helicase[Porphyromonasgingivalis]_gi|543985765|gb|ERJ89108.1|helicaseprotein[Porphyromonasgingi    AKIKASKNDCI-LCSMDNEEIYGKEFYRVNFSYAVQNGILTDYKVLVLTVSENM---IPS
gi|497438056|ref|WP_009752254.1|helicase[Prevotellasp.oraltaxon473]_gi|429144866|gb|EKX87968.1|helicaseprotein[Prevotellasp.ora    AKLRANKADAT-LCSMDDEKIYGQEFFRVNFSYAVRHGILTDYKVLVLTVSEDM---IPD
gi|490423474|ref|WP_004295683.1|MULTISPECIES:helicase[Bacteroides]_gi|156110098|gb|EDO11843.1|helicaseCterminaldomainprotein[B/    AKIKASEKDAI-LCSMDDPKLYGEEFFRVNFSYAVEHGLLTDYKVLVLTVNEDD---LPD
gi|941777229|ref|WP_055173506.1|helicase[Bacteroidescaccae]_gi|933263395|emb|CUQ00567.1|Predictedhelicase[Bacteroidescaccae]/1/    AKIKASEKDCI-LCSMDDKAIYGEEFYRVNFSYAVQNGLLTDYKVLVLTVSEDD---VPA
gi|492413898|ref|WP_005836892.1|helicase[Bacteroidesuniformis]_gi|392678824|gb|EIY72229.1|hypotheticalproteinHMPREF1072_03583[B    AKIKASEKDCI-LCSMDDKAIYGEEFYRVNFSYAVQNGLLTDYKVLVLTVSEDD---VPA
gi|695455095|ref|WP_032566191.1|helicase[Bacteroidesfragilis]_gi|596095426|gb|EXZ59996.1|restrictionendonucleasefamilyprotein[B    AKIKASEKDCI-LCSMDDKAIYGEEFYRVNFSYAVQNGLLTDYKVLVLTVSEND---VPA
gi|933227941|emb|CUQ02348.1|Predictedhelicase[Bacteroidesuniformis]/11658    AKIKASEKDCI-LCSMDDQSLYGGEFYRVNFSYAVQNGLLTDYKVLVLTVSEDD---VPD
gi|494823013|ref|WP_007558421.1|helicase[Bacteroidesplebeius]_gi|198272167|gb|EDY96436.1|helicaseCterminaldomainprotein[Bacter/    AKIKASEKDCI-LCSMDDKAIYGEEFYRVNFSYAVQNGLLTDYKVLVLTVSEDD---VPD
gi|495427212|ref|WP_008151909.1|helicase[Parabacteroidesjohnsonii]_gi|218222381|gb|EEC95031.1|helicaseCterminaldomainprotein[P/    AKIKASEKDCI-LCSMDDKALYGEEFYRVNFSYAVQNGLLTDYKVLVLTVGEDD---VPE
gi|933196855|emb|CUO94545.1|typeIrestrictionenzymeEcoKIsubunitR[Bacteroidesuniformis]_gi|933220098|emb|CUN86874.1|typeIrestrict    AKVKASEKDCI-LCSMDDKALYGEEFYRVNFSYAVQNGLLTDYKVLVLTVGEND---VPD
gi|823687748|ref|WP_047101121.1|helicase[Brachyspirahyodysenteriae]_gi|823334537|gb|KLI17932.1|helicase[Brachyspirahyodysenteri    SKVKAEKNEMT-LCSMDDEKLYGEEIYRIGFGQAVENGLLSDYKVLILTISDNN---INE
gi|496436691|ref|WP_009145538.1|helicase[Phascolarctobacteriumsuccinatutens]_gi|322414007|gb|EFY04839.1|helicaseCterminaldomai/    AKKKAEENSVV-LCSMDDKNIYGEEIYRIGFGEAVEKDLLSDYKVLILTVRENT--QLPA
gi|503372880|ref|WP_013607541.1|helicase[Sphaerochaetaglobosa]_gi|324026933|gb|ADY13692.1|typeIIIrestrictionproteinressubunit[S    SQKKAKDKEAI-LCSMDDTAIYGEEFYRLGFGEAVEKQLLSDYKVIVLTIDEDQ---LSA
gi|505821748|ref|WP_015707145.1|helicase[Treponemaprimitia]_gi|299483503|gb|ADJ19584.1|helicasedomaincontainingprotein[Trepone/    AQKKAKEADAL-LCSMDDTKLYGEEMYRIGFGEAVDKELLSDYKVIVLTIETEQ---LNE
gi|505822741|ref|WP_015708134.1|helicase[Treponemaprimitia]_gi|333739639|gb|AEF85129.1|endonucleaseandmethylaseLlaGI[Treponemap    AQKKAKEADAL-LCSMDDTKLYGEEMYRIGFGEAVDKELLSDYKVIVLTIETEQ---LNE
gi|488791994|ref|WP_002704400.1|helicase[Treponemasaccharophilum]_gi|380767691|gb|EIC01689.1|typeIIIrestrictionproteinressubuni    TQTKAKETETT-LWSMDDAEIYGEEIYRIGFGEAVEKKLLSDYKVVVLTLDENS---VDP
gi|524671454|emb|CDD81750.1|putativeseptumsitedeterminingproteinMinC[Bacteroidessp.CAG:462]/11642    ARKKAEESDVV-LCSMDDVSLYGDEIYRIGFGEAVKKQLLTDYKVLILAVGDKD---ITP
gi|524573865|emb|CDC95766.1|putativeseptumsitedeterminingproteinMinC[Alistipessp.CAG:268]/11639    TKKRAEENSAV-LCSMDDRSMYGDEIYRIGFGEAVKQELLSDYKVLILAVGEKD---ITP
gi|658617970|ref|WP_029759328.1|helicase[Fusobacteriumnucleatum]/11606    SKSKAKESDTY-LCSMDDKKLYGEEIYRIGFGKAVENNLLADYKVLILTLNSSQ---IPR
gi|494412037|ref|WP_007216782.1|helicase[Bacteroidescellulosilyticus]_gi|392638921|gb|EIY32752.1|hypotheticalproteinHMPREF1062_    AQSKARESEAT-LCSMDDAALFGDEIYRIGFGEAVEKQLLADYKVLVLTLGEDQ---VTP
gi|383107102|gb|AFG37435.1|putativehelicase[SpirochaetaafricanaDSM8902]/11616    AQSKAAESDAI-LCSMDDESLYGKEFYRIGFGEAVSNDLLTDYKVLILTLTEND---VSA
SAF8902III/11616    AQSKAAESDAI-LCSMDDESLYGKEFYRIGFGEAVSNDLLTDYKVLILTLTEND---VSA
gi|652538894|ref|WP_026932796.1|helicase[Gramellaechinicola]/11633    SKSKAAQNDAI-ICSMDDTSLYGEEIYRIGFGEAVQNDLLSDYKVLILTLSDAD---VPP
gi|639238223|ref|WP_024568839.1|helicase[Elizabethkingiaanophelis]/11628    SISKAQQNDAI-ICSMDDVNTYGEEIYRIGFGEAVEKDLLTDYKVLILTLNEKD---IPA
gi|652520381|ref|WP_026914582.1|helicase[Gramellaportivictoriae]/11623    SKSKAALNETE-LCSMDDESKYGKEIYRIGFGEAVDRGLLTDYKVLVLTLSEND---IPQ
gi|494922183|ref|WP_007648219.1|Helicase[Formosasp.AK20]_gi|469825489|gb|EMQ95550.1|Helicasedomainprotein[Formosasp.AK20]/11626    SKSKADMNDAV-ICSMDDETKYGIEMYRIGFGEAVERGLLTDYKVLVLTLSEND---IPS
gi|522083382|ref|WP_020594591.1|hypotheticalprotein[Spirosomapanaciterrae]/11637    TKSKAAQAEAL-LCSMDDENLYGGEIYRIGFGEAVEKDLLTDYKVLILTLNEND---VPP
gi|490455352|ref|WP_004326157.1|helicase[Bacteroidesovatus]_gi|392669277|gb|EIY62768.1|hypotheticalproteinHMPREF1070_03635[Bact    SKSKAAQGDAI-LCSMDDERIYGGEIYRIGFGEAVNKGMLSDYKVLILTVSEND---MPV
gi|499516035|ref|WP_011202675.1|helicase[Bacteroidesfragilis]_gi|53713270|ref|YP_099262.1|helicasedomainprotein[Bacteroidesfrag    SKSKAAQGDAI-LCSMDDERIYGGEIYRIGFGEAVNKGMLSDYKVLILTVSEND---MPV
gi|651338332|ref|WP_026461320.1|helicase[Adhaeribacteraquaticus]/11620    TKSKAAQADAV-LCSMDDAKLYGDEMYRIGFGEAVSRDLLTDYKVLILTLSDQD---VPP
gi|806964642|gb|AKC17139.1|helicase[Flavobacteriumpsychrophilum]/11639    TKSKAAQAEAI-LCSMDEPELYGEEIYRIGFGEAVDLQLLTDYKVLILTLNEAD---VPP
```

```
gi|752684374|ref|WP_041332322.1|helicase[Flavobacteriumpsychrophilum]_gi|666882889|gb|AIG30715.1|helicase[Flavobacteriumpsychro      TKSKAAQAEAI-LCSMDEPELYGEEIYRIGFGEAVDLQLLTDYKVLILTLNEAD---VPP
gi|933196595|emb|CUP04894.1|DNAorRNAhelicasesofsuperfamilyII[Bacteroidesuniformis]/11631                                          TKSKAAQADAF-LCSMDDVALYGEEFYRIGFGEAVDKGLLADYKVLILTLNDRD---VPP
gi|670509109|ref|WP_031456918.1|helicase[Flavobacteriumchungangense]/11625                                                         VKSKAAQAEAV-LCSMDDVNLYGEEIYRIGFGEAVEKDLLTDYKVLILTLNDHD---IPP
                                                                                                                                                  *     :*       *  .     *   ::  . .

gi|490190857|ref|WP_004089451.1|hypotheticalprotein[Helicobacterbilis]_gi|476630638|gb|EMZ37196.1|hypotheticalproteinC826_02193      QAIA----KL------------------KEK--G---MNRLNEKLVD-----LDFVCKII
gi|488896133|ref|WP_002807242.1|MULTISPECIES:restrictionendonucleaseEco57I[Campylobacter]_gi|380541096|gb|EIA65377.1|hypothetic      TAIA-------K---------------LK-D--E----KNVDEKYVD-----IDFVSKII
gi|746602240|ref|WP_039628365.1|restrictionendonucleaseEco57I[Campylobacterlari]_gi|744819395|gb|AJD03391.1|putativehelicase[Ca      TAIA-------K---------------LK-D--E----KNVDEKYVD-----IDFVSKII
gi|727317135|ref|WP_033772558.1|restrictionendonucleaseEco57I[Helicobacterpylori]/11622                                            SVNK----KISQ---------------LNAE--G----TKLDKKLIN-----NEFVCKII
gi|727103490|ref|WP_033618954.1|restrictionendonucleaseEco57I[Helicobacterpylori]/11616                                            SVNK----KISQ---------------LNAE--G----TKLDKKLIN-----NEFVCKII
gi|727340205|ref|WP_033794408.1|restrictionendonucleaseEco57I[Helicobacterpylori]/11609                                            SVNK----KISQ---------------LNAE--G----TKLDKKLIN-----NEFVCKII
gi|727292407|ref|WP_033749975.1|restrictionendonucleaseEco57I[Helicobacterpylori]/11611                                            SVNK----KISQ---------------LNAE--G----TKLDKKLIN-----NEFVCKII
gi|727289795|ref|WP_033747591.1|restrictionendonucleaseEco57I[Helicobacterpylori]/11612                                            SVNK----KISQ---------------LNAE--G----TKLDKKLIN-----NEFVCKII
gi|727319676|ref|WP_033775047.1|restrictionendonucleaseEco57I[Helicobacterpylori]/11612                                            SVNK----KISQ---------------LNAE--G----TKLDKKLIN-----NEFVCKII
gi|447212910|ref|WP_001290166.1|restrictionendonucleaseEco57I[Helicobacterpylori]_gi|308063540|gb|ADO05427.1|hypotheticalprotei      SVNK----KISR---------------LEAE--G----TKLDKKLIN-----NEFVCKII
gi|447212901|ref|WP_001290157.1|restrictionendonucleaseEco57I[Helicobacterpylori]_gi|384557329|gb|AFH97797.1|hypotheticalprotei      SVNK----KISQ---------------LEAE--G----TKLDKKLIN-----NEFVCKII
gi|515849671|ref|WP_017280424.1|hypotheticalprotein[Helicobacterpylori]/11615                                                      SVNK----KISR---------------LEAK--G----TKLDKKLID-----NEFVCKII
gi|447212900|ref|WP_001290156.1|restrictionendonucleaseEco57I[Helicobacterpylori]_gi|317178970|dbj|BAJ56758.1|TypeIIGrestrictio      SVNQ----KISR---------------LEAK--G----TKLDKKLID-----NEFVCKII
gi|447079018|ref|WP_001156274.1|hypotheticalprotein[Helicobacterpylori]/11614                                                      SVNK----KISQ---------------LEAK--G----TKLDKKLIN-----NEFVCKII
gi|447212800|ref|WP_001290056.1|hypotheticalprotein[Helicobacterpylori]_gi|532153248|gb|EQL67316.1|restrictionendonucleaseEco57      SVNK----KISQ---------------LEAK--G----TKLDKKLIN-----NEFVCKII
gi|505457237|ref|WP_015644339.1|hypotheticalprotein[Helicobacterpylori]_gi|499061160|gb|AGL70074.1|restrictionendonucleaseEco57      SVNK----KISQ---------------LEAK--G----TKLDKKLIN-----NEFVCKII
HPYUM037X/11591                                                                                                                    SVNK----KISQ---------------LEAK--G----TKLDKKLIN-----NEFVCKII
gi|920026297|ref|WP_052937176.1|restrictionendonuclease[Helicobacterpylori]_gi|912528678|gb|KNX48238.1|restrictionendonucleaseE      SVNK----KISQ---------------LKAE--G----TKLDKKLIN-----NEFVCKII
gi|915539050|ref|WP_050841327.1|restrictionendonuclease[Helicobacterpylori]_gi|908890887|gb|KNE06183.1|restrictionendonucleaseE      SVNK----KISQ---------------LKAE--G----TKLDKKLIN-----NEFVCKIV
gi|727101397|ref|WP_033616957.1|restrictionendonucleaseEco57I[Helicobacterpylori]/11592                                            SVNK----KISQ---------------LKAE--G----TKLDKKLIN-----NEFVCKIV
gi|562814875|emb|CDK13002.1|TypeISPRMsystem[Helicobacterpylori26695]/11588                                                         SVNK----KISQ---------------LKAE--G----TKLDKKLIN-----NEFVCKII
HPYAXVIII/11588                                                                                                                    SVNK----KISQ---------------LKAE--G----TKLDKKLIN-----NEFVCKII
gi|447079029|ref|WP_001156285.1|restrictionendonucleaseEco57I[Helicobacterpylori]_gi|393085956|gb|EJB86635.1|hypotheticalprotei      SVNK----KISQ---------------LEAK--G----TKLDKKLIN-----NEFVCKIV
gi|489105953|ref|WP_003015814.1|restrictionendonuclease[Helicobacterpylori]_gi|477503388|gb|ENH59950.1|restrictionendonucleasef      SVNK----KISQ---------------LKAE--G----TKLDKKLIN-----NEFVCKIV
gi|447079006|ref|WP_001156262.1|restrictionendonucleaseEco57I[Helicobacterpylori]_gi|393067470|gb|EJB68280.1|hypotheticalprotei      SVNK----KISQ---------------LEAK--G----TKLDKKLIN-----NEFVCKII
gi|727314589|ref|WP_033770298.1|restrictionendonucleaseEco57I[Helicobacterpylori]/11586                                            SVNQ----KISQ---------------LKAE--G----TKLDKKLIN-----NEFVCKII
gi|447079019|ref|WP_001156275.1|restrictionendonucleaseEco57I[Helicobacterpylori]_gi|308065028|gb|ADO06920.1|hypotheticalprotei      SVNK----KISQ---------------LKAE--G----TKLDKKLID-----NEFVCKIV
gi|447079028|ref|WP_001156284.1|restrictionendonucleaseEco57I[Helicobacterpylori]_gi|393076030|gb|EJB76784.1|hypotheticalprotei      SVNK----KISQ---------------LKAE--G----TKLDKKLIN-----NEFVCKII
gi|393034009|gb|EJB35070.1|hypotheticalproteinHPNQ4053_0631[HelicobacterpyloriNQ4053]/11514                                       SVNK----KISQ---------------LKAE--G----TKLDKKLIN-----NEFVCKIV
gi|447079004|ref|WP_001156260.1|restrictionendonucleaseEco57I[Helicobacterpylori]_gi|384552418|gb|AFI07366.1|hypotheticalprotei      SVNK----KISQ---------------LKAE--G----TKLDKKLIN-----NEFVCKIV
gi|746011306|ref|WP_039088215.1|restrictionendonucleaseEco57I[Helicobacterpylori]_gi|733488720|gb|KHL85979.1|restrictionendonuc      SVNK----KISQ---------------LEAK--G----TKLDKKLIN-----NEFVCKII
gi|531081162|gb|EQD92166.1|restrictionendonucleaseEco57I[HelicobacterpyloriPZ5080]/11509                                           SVSK----KISQ---------------LEAK--G----TKLDKKLIN-----NEFVCKIV
gi|544760796|ref|WP_021187395.1|restrictionendonucleaseEco57I[Helicobacterpylori]_gi|531086508|gb|EQD96745.1|restrictionendonuc      SVNK----KISQ---------------LEAK--G----TKLDKKLIN-----NEFVCKIV
gi|447079012|ref|WP_001156268.1|restrictionendonucleaseEco57I[Helicobacterpylori]_gi|393028199|gb|EJB29286.1|hypotheticalprotei      SVNK----KISQ---------------LKAE--G----TKLDKKLIN-----NEFVCKIV
gi|746008458|ref|WP_039085555.1|restrictionendonucleaseEco57I[Helicobacterpylori]_gi|733480358|gb|KHL78026.1|restrictionendonuc      SVNK----KISQ---------------LKAK--G----TKLDKKLIN-----NEFVCKIV
gi|447079011|ref|WP_001156267.1|restrictionendonucleaseEco57I[Helicobacterpylori]_gi|393038534|gb|EJB39568.1|hypotheticalprotei      SVNK----KISQ---------------LEAK--G----TKLDKKLIN-----NEFVCKIV
gi|393037163|gb|EJB38199.1|hypotheticalproteinHPNQ4161_0422[HelicobacterpyloriNQ4161]/11556                                       SVNK----KISQ---------------LEAK--G----TKLDKKLIN-----NEFVCKIV
gi|447079007|ref|WP_001156263.1|restrictionendonucleaseEco57I[Helicobacterpylori]_gi|393051817|gb|EJB52768.1|hypotheticalprotei      SVNK----KISQ---------------LKAE--G----TKLDKKLTN-----NEFVCKIV
gi|487867094|ref|WP_001940560.1|helicase[Helicobacterpylori]_gi|459505657|gb|EMG98418.1|helicaseprotein[HelicobacterpyloriGAM21      SVNK----KISQ---------------LEAK--G----TKLDKKLIN-----NEFVCKIV
gi|487872094|ref|WP_001945560.1|helicase[Helicobacterpylori]_gi|459512104|gb|EMH04523.1|helicaseprotein[HelicobacterpyloriGAM24      SVNK----KISQ---------------LEAK--G----TKLDKKLIN-----NEFVCKIV
gi|487880228|ref|WP_001953694.1|helicase[Helicobacterpylori]_gi|459529131|gb|EMH20734.1|helicaseprotein[HelicobacterpyloriGAM26      SVNK----KISQ---------------LEAK--G----TKLDKKLIN-----NEFVCKIV
gi|487882593|ref|WP_001956059.1|helicase[Helicobacterpylori]_gi|459533462|gb|EMH24859.1|helicaseprotein[HelicobacterpyloriGAM26      SVNK----KISQ---------------LKAK--G----TKLDKKLIN-----NEFVCKIV
gi|487845430|ref|WP_001918896.1|helicase[Helicobacterpylori]_gi|410713871|gb|EKQ71363.1|helicaseprotein[HelicobacterpyloriGAM10      SVNK----KISQ---------------LEAK--G----TKLDKKLIN-----NEFVCKIV
gi|447079005|ref|WP_001156261.1|restrictionendonucleaseEco57I[Helicobacterpylori]_gi|393069830|gb|EJB70625.1|hypotheticalprotei      SVNK----KISQ---------------LEAK--G----TKLDKKLIN-----NEFVCKIV
gi|459506808|gb|EMG99528.1|helicaseprotein[HelicobacterpyloriGAM121Aii]/11545                                                      SVNK----KISQ---------------LKAE--G----TKLDKKLIN-----NEFVCKIV
gi|487825283|ref|WP_001898749.1|restrictionendonucleaseEco57I[Helicobacterpylori]_gi|393107526|gb|EJC08068.1|hypotheticalprotei      SVNK----KISQ---------------LKAE--G----TKLDKKLIN-----NEFVCKIV
gi|447079008|ref|WP_001156264.1|restrictionendonucleaseEco57I[Helicobacterpylori]_gi|393058636|gb|EJB59524.1|hypotheticalprotei      SVNK----KISQ---------------LEAK--G----TKLDKKLIN-----NEFVCKIV
gi|447079015|ref|WP_001156271.1|restrictionendonucleaseEco57I[Helicobacterpylori]_gi|307637357|gb|ADN79807.1|hypotheticalprotei      SVNK----KISQ---------------LEAK--G----TKLDKKLIN-----NEFVCKIV
gi|487884861|ref|WP_001958327.1|helicase[Helicobacterpylori]_gi|459535535|gb|EMH26834.1|helicaseprotein[HelicobacterpyloriGAM26      SVNK----KISQ---------------LKAE--G----TKLDKKLIN-----NEFVCKIV
gi|487886365|ref|WP_001959831.1|helicase[Helicobacterpylori]_gi|459538727|gb|EMH29861.1|helicaseprotein[HelicobacterpyloriGAM27      SVNK----KISQ---------------LKAE--G----TKLDKKLIN-----NEFVCKIV
gi|545301637|ref|WP_021581412.1|helicase[Helicobacterpylori]_gi|535931889|gb|ERA61745.1|helicaseprotein[HelicobacterpyloriGAM11      SVNK----KISQ---------------LEAK--G----TTLDKKLIN-----NEFVCKIV
gi|447079010|ref|WP_001156266.1|restrictionendonucleaseEco57I[Helicobacterpylori]_gi|393095477|gb|EJB96081.1|hypotheticalprotei      SVNK----KISQ---------------LEAK--G----TKLDKKLIN-----NEFVCKIV
gi|447079009|ref|WP_001156265.1|restrictionendonucleaseEco57I[Helicobacterpylori]_gi|393056294|gb|EJB57206.1|hypotheticalprotei      SVNK----KISQ---------------LEAK--G----TKLDKKLIN-----NEFVCKIV
gi|471386985|gb|EMR58906.1|restrictionendonucleasefamilyprotein[HelicobacterpyloriHpH1]/11504                                     SVNK----KISQ---------------LEAK--G----TKLDKKLIN-----NEFVCKIV
gi|487875679|ref|WP_001949145.1|helicase[Helicobacterpylori]_gi|459516928|gb|EMH09091.1|helicaseprotein[HelicobacterpyloriGAM25      SVNK----KISQ---------------LEAK--G----TKLDKKLIN-----NEFVCKIV
gi|496130066|ref|WP_008854573.1|DNAdamageinducibleprotein[Commensalibacterintestini]_gi|353671689|gb|EHD13391.1|helicasedomain/      RLQN----LLTD-----------------------------NNNSLK-----VDDAAKIV
gi|924898894|ref|WP_053556546.1|damageinducibleprotein[Sphingopyxissp.113P3]_gi|924523996|gb|ALC14732.1|damageinducibleprotei/1      GLQA----RLAD-----------------------------DDSALV-----LDDATKLV
gi|780802950|gb|KJS08562.1|damageinducibleprotein[Hoefleasp.BRH_c9]/11633                                                         GVQH----RLAD-----------------------------ANSELL-----LDDATKII
gi|944671432|gb|KQN22886.1|damageinducibleprotein[Sphingomonassp.Leaf32]/11415                                                    SIQN----RLKD-----------------------------ADNELK-----LDDATKIV
gi|944612953|gb|KQM64772.1|damageinducibleprotein[Sphingomonassp.Leaf16]/11726                                                    SIQN----RLKD-----------------------------ADNELK-----LDDATKIV
gi|764353851|ref|WP_044331468.1|damageinducibleprotein[Sphingomonashengshuiensis]_gi|761897338|gb|AJP71707.1|damageinduciblep/1      GVQN----RLAD-----------------------------GTSELK-----LDDATKII
gi|945874472|gb|KQZ27973.1|damageinducibleprotein[Mesorhizobiumsp.Root552]/11651                                                  GVQK----RLAD-----------------------------GSSELK-----LDDATKII
```

```
gi|759408081|ref|WP_043132403.1|damageinducibleprotein[Paracoccussanguinis]_gi|694217086|gb|KGJ14281.1|damageinducibleprotein/1    TIQN----RLKD-----------------------------GPELT-----LDDATKII
gi|654342671|ref|WP_027836116.1|damageinducibleprotein[Maritaleamyrionectae]/11632    TIQT----RLKE-----------------------------GAELS-----LDDATKIV
gi|126717687|gb|EBA14409.1|hypotheticalproteinRSK20926_02032[Roseobactersp.SK20926]/11603    TIQN----RLKD-----------------------------GPELT-----LDDATKII
gi|765301116|ref|WP_044628780.1|damageinducibleprotein[Halomonasmeridiana]_gi|764089679|gb|KJD19875.1|damageinducibleprotein[/1    TIQG----RLKD-----------------------------GAELT-----LDDATKII
gi|518053006|ref|WP_019223214.1|DNAdamageinducibleprotein[Bartonellarattaustraliani]/11654    EIQH----IITN-----------------------------KDCAID-----LGDVAKII
gi|493637806|ref|WP_006589578.1|DNAdamageinducibleprotein[Bartonellabirtlesii]_gi|395410345|gb|EJF76900.1|hypotheticalproteinM/    AIQE----IIAN-----------------------------ENREID-----LDDAAKII
gi|501188603|ref|WP_012231622.1|DNAdamageinducibleprotein[Bartonellatribocorum]_gi|161017843|emb|CAK01401.1|helicase/methyltra/    AIQE----IIAN-----------------------------ENREID-----LDDAAKII
gi|651388616|ref|WP_026500615.1|damageinducibleprotein[Bartonellagrahamii]/11655    AIQE----IIAN-----------------------------ENREID-----LDDAAKII
gi|501188669|ref|WP_012231687.1|DNAdamageinducibleprotein[Bartonellatribocorum]_gi|161017922|emb|CAK01480.1|helicase/methyltra/    AIQE----IIAN-----------------------------ENREID-----LDDAAKII
gi|518048832|ref|WP_019219040.1|DNAdamageinducibleprotein[Bartonellaflorencae]/11655    TIQK----RLKD-----------------------------KNSELI-----LDDTTKII
gi|518053617|ref|WP_019223825.1|DNAdamageinducibleprotein[Bartonellarattaustraliani]/11607    TIQK----RLKD-----------------------------KNSELI-----LDDTTKII
gi|506336449|ref|WP_015856168.1|DNAdamageinducibleprotein[Bartonellagrahamii]_gi|240267492|gb|ACS51080.1|helicase/methyltransf/    TIQK----RLKD-----------------------------KNSELI-----LDDTTKII
gi|494588832|ref|WP_007347427.1|DNAdamageinducibleprotein[Bartonellarattimassiliensis]_gi|395419053|gb|EJF85361.1|hypothetical/    TIQK----RLKD-----------------------------KNSELI-----LDDTTKII
gi|501188650|ref|WP_012231668.1|DNAdamageinducibleprotein[Bartonellatribocorum]_gi|161017902|emb|CAK01460.1|helicase/methyltra/    TIQK----RLKD-----------------------------KNSELI-----LDDTTKII
gi|501188624|ref|WP_012231643.1|DNAdamageinducibleprotein[Bartonellatribocorum]_gi|161017866|emb|CAK01424.1|helicase/methyltra/    TMEM----PTTS-----------------------------KDYELL-----LDYRTKIV
gi|518053856|ref|WP_019224064.1|DNAdamageinducibleprotein[Bartonellarattaustraliani]/11625    SMEM----PTTN-----------------------------NDYELL-----LDDRTKIV
gi|501188053|ref|WP_012231081.1|DNAdamageinducibleprotein[Bartonellatribocorum]_gi|161017434|emb|CAK00992.1|helicase/methyltra/    TMEM----PTTS-----------------------------KDYELI-----LDYRTKIV
gi|651388630|ref|WP_026500626.1|damageinducibleprotein[Bartonellagrahamii]/11657    SIQH----LITD-----------------------------KDYELI-----LDDRTKIN
gi|518048855|ref|WP_019219063.1|DNAdamageinducibleprotein[Bartonellaflorencae]/11658    SIQH----LITD-----------------------------KNYELI-----LDDRTKIN
gi|490995101|ref|WP_004856829.1|DNAdamageinducibleprotein[Bartonelladoshiae]_gi|395413167|gb|EJF79645.1|hypotheticalproteinMCS/    SIQH----LITD-----------------------------KNYELI-----LDDRTKIN
gi|491002558|ref|WP_004864279.1|DNAdamageinducibleprotein[Bartonellavinsonii]_gi|395421392|gb|EJF87643.1|hypotheticalproteinME/    SIQH----LMTD-----------------------------DNYELI-----LDDKTKIV
gi|491003373|ref|WP_004865094.1|DNAdamageinducibleprotein[Bartonellavinsonii]_gi|395432864|gb|EJF98838.1|hypotheticalproteinME/    SIQH----LMTD-----------------------------DNYELI-----LDDKTKIV
gi|491002426|ref|WP_004864148.1|DNAdamageinducibleprotein[Bartonellavinsonii]_gi|395421767|gb|EJF88003.1|hypotheticalproteinME/    SIQH----LMTD-----------------------------DNYELT-----LDDETKLV
gi|491004024|ref|WP_004865745.1|DNAdamageinducibleprotein[Bartonellavinsonii]_gi|395432542|gb|EJF98522.1|hypotheticalproteinME/    SIQH----LMTD-----------------------------DNYELT-----LDDETKLV
gi|648396442|ref|WP_026088193.1|damageinducibleprotein[Bartonellarattaustraliani]/11593    SLQQ----LMTD-----------------------------ENYELT-----LDDKTKII
gi|748190174|ref|WP_039761290.1|damageinducibleprotein[Bartonellaqueenslandensis]/11658    SLQQ----LMTD-----------------------------ENYELT-----LDDKTKII
gi|494817599|ref|WP_007553007.1|DNAdamageinducibleprotein[Bartonellasp.DB56]_gi|395411312|gb|EJF77837.1|hypotheticalproteinMC/1    SIQH----LMTD-----------------------------ENYELI-----LDDRTKIM
gi|515763376|ref|WP_017195976.1|DNAdamageinducibleprotein[Bartonellabirtlesii]/11656    SIQH----LMTD-----------------------------ENYELI-----LDDRTKIV
gi|494589420|ref|WP_007348014.1|DNAdamageinducibleprotein[Bartonellarattimassiliensis]_gi|395416278|gb|EJF82667.1|hypothetical/    SIQH----LMTD-----------------------------ENYELI-----LDDKTKII
gi|506336469|ref|WP_015856188.1|DNAdamageinducibleprotein[Bartonellagrahamii]_gi|240267512|gb|ACS51100.1|helicase/methyltransf/    SIQH----LMTD-----------------------------ENYELI-----LDDKTKII
gi|749308087|ref|WP_040132227.1|damageinducibleprotein[Bartonellatribocorum]_gi|605051878|emb|CDO49988.1|helicase/methyltransf/    SIQH----LMTD-----------------------------ENYELI-----LDDKTKII
gi|515763800|ref|WP_017196400.1|DNAdamageinducibleprotein[Bartonellabirtlesii]/11658    SIQH----LMID-----------------------------ENYELT-----LDDKTKII
gi|492186994|ref|WP_005773380.1|DNAdamageinducibleprotein[Bartonellaelizabethae]_gi|395418361|gb|EJF84688.1|hypotheticalprotei/    SIQH----LMTD-----------------------------ENYELI-----LDDKTKII
gi|501188615|ref|WP_012231634.1|DNAdamageinducibleprotein[Bartonellatribocorum]_gi|161017856|emb|CAK01414.1|helicase/methyltra/    SIQH----LMTD-----------------------------ENYELT-----LDDKTKII
gi|501187470|ref|WP_012230499.1|DNAdamageinducibleprotein[Bartonellatribocorum]_gi|161017090|emb|CAK00648.1|helicase/methyltra/    SIQH----LMTD-----------------------------ENYELT-----LDDKTKII
gi|501188636|ref|WP_012231655.1|DNAdamageinducibleprotein[Bartonellatribocorum]_gi|161017879|emb|CAK01437.1|helicase/methyltra/    SIQH----LMTD-----------------------------ENYELT-----LDDKTKII
gi|930151941|emb|CTQ34818.1|UvsWhelicase[Jannaschiarubra]/11641    SVQR----RLET-----------------------------SDSDLA-----LDDATKIV
gi|928977106|ref|WP_054001461.1|damageinducibleprotein[Shimiasp.SK013]_gi|927698852|gb|KPA22698.1|typeIrestrictionenzymeEcoKIs/    GVQG----RIAA------------------------------DNELQ-----LDDATKII
gi|738872465|ref|WP_036760884.1|damageinducibleprotein[Leisingeradaeponensis]/11643    SVQS----RIED-----------------------------PTSGLI-----LDDATRIL
gi|518050881|ref|WP_019221089.1|DNAdamageinducibleprotein[Bartonellasenegalensis]/11652    AVQK----RLSD-----------------------------SESTLV-----LDDATKII
gi|493980596|ref|WP_006923523.1|DNAdamageinducibleprotein[Bartonellawashoensis]_gi|395413322|gb|EJF79796.1|hypotheticalprotein/    AVQK----RLSD-----------------------------GQSELI-----LDDATKII
gi|493983106|ref|WP_006925984.1|DNAdamageinducibleprotein[Bartonellawashoensis]_gi|395418127|gb|EJF84455.1|hypotheticalprotein/    AVQK----RLSD-----------------------------GQSELV-----LDDVTKII
gi|499494671|ref|WP_011181311.1|DNAdamageinducibleprotein[Bartonellahenselae]_gi|49239008|emb|CAF28308.1|hypotheticalproteinBH/    AVQK----RLSD-----------------------------DQSELV-----LDDATKII
gi|651388426|ref|WP_026500441.1|damageinducibleprotein[Bartonellagrahamii]/11652    AVQK----RLSD-----------------------------DQSSLV-----LDDATKII
gi|506337280|ref|WP_015856999.1|DNAdamageinducibleprotein[Bartonellagrahamii]_gi|240268402|gb|ACS51990.1|helicase/methyltransf/    AVQK----RLSD-----------------------------DQSSLV-----LDDATKII
gi|494817535|ref|WP_007552943.1|DNAdamageinducibleprotein[Bartonellasp.DB56]_gi|395412002|gb|EJF78518.1|hypotheticalproteinMC/1    AVQK----RLSD-----------------------------GKSELV-----LDDATKII
gi|493638099|ref|WP_006589864.1|DNAdamageinducibleprotein[Bartonellabirtlesii]_gi|395409809|gb|EJF76394.1|hypotheticalproteinM/    AVQK----RLSD-----------------------------GKSELV-----LDDATKII
gi|748189589|ref|WP_039760707.1|damageinducibleprotein[Bartonellaqueenslandensis]/11651    AVQK----RLSD-----------------------------SDSELI-----LDDATKII
gi|501189483|ref|WP_012232501.1|DNAdamageinducibleprotein[Bartonellatribocorum]_gi|161018902|emb|CAK02460.1|helicase/methyltra/    AVQK----RLSD-----------------------------RESALI-----LDDATKII
gi|518049181|ref|WP_019219389.1|DNAdamageinducibleprotein[Bartonellaflorencae]/11652    AVQK----RLSD-----------------------------SESGLI-----LDDATKII
gi|492193255|ref|WP_005774528.1|DNAdamageinducibleprotein[Bartonellaelizabethae]_gi|395416403|gb|EJF82779.1|hypotheticalprotei/    AVQK----RLSD-----------------------------SESALI-----LDDATKII
gi|498387325|ref|WP_010701288.1|helicase/methyltransferase[Bartonellabovis]_gi|478418724|gb|ENN92426.1|helicase/methyltransfera    AVQK----RLSD-----------------------------SESELI-----LDDATKII
gi|319408697|emb|CBI82352.1|conservedhypotheticalprotein[BartonellaschoenbuchensisR1]/11661    AVQK----RLSD-----------------------------SDSKLI-----LDDATKII
gi|494741726|ref|WP_007477134.1|DNAdamageinducibleprotein[Bartonellamelophagi]_gi|395423925|gb|EJF90113.1|hypotheticalproteinM/    AVQK----RLSD-----------------------------SK-SLI-----LDDATKII
gi|737261405|ref|WP_035245349.1|damageinducibleprotein[Alcanivoraxjadensis]_gi|686989709|gb|KGD62556.1|hypotheticalproteinT9A_/    GVQR----ALSS-----------------------------GDNELQ-----LDDASRIV
gi|916687564|ref|WP_051294655.1|damageinducibleprotein[Gemmobacternectariphilus]/11641    AVQK----RLGD-----------------------------GASELV-----LDDATKIV
gi|740294127|ref|WP_038132495.1|damageinducibleprotein[Thioclavasp.DT234]_gi|660524826|gb|KEO54689.1|hypotheticalproteinDT23_/1    NVQK----RLAD-----------------------------ENSELD-----LDDATKII
gi|568331062|ref|WP_024099615.1|helicase[Phaeobactergallaeciensis]_gi|568232121|gb|AHD12007.1|putativehelicase[Phaeobactergalla    GVQK----RLAD-----------------------------SASELV-----LDDATKIV
gi|518810459|ref|WP_019966413.1|DNAdamageinducibleprotein[Pannonibacterphragmitetus]/11635    AVQK----RLGD-----------------------------AGSELV-----LDDATKII
gi|495305760|ref|WP_008030510.1|DNAdamageinducibleprotein[Rhodobactersp.SW2]_gi|259021698|gb|EEW25000.1|typeIIIrestrictionprot/    AVQK----RLGD-----------------------------AGSELV-----LDDATKII
gi|506403303|ref|WP_015923022.1|DNAdamageinducibleprotein[Rhodobactersphaeroides]_gi|221162951|gb|ACM03922.1|HypotheticalProte/    AVQK----RLGD-----------------------------AGSELV-----LDDATKII
gi|738818386|ref|WP_036708790.1|damageinducibleprotein[Paracoccussanguinis]_gi|694226786|gb|KGJ22541.1|damageinducibleprotein/1    AVQK----RLGD-----------------------------AGSELV-----LDDATKII
gi|746096057|ref|WP_039162453.1|damageinducibleprotein[Gallibacteriumanatis]_gi|703617775|gb|KGQ63868.1|damageinducibleprotei/1    RLEY----LLNR-----------------------------ENNEIQ-----VDDAAKIV
gi|737716785|ref|WP_035685631.1|damageinducibleprotein[Avibacteriumparagallinarum]/11643    RLEY----LLNR-----------------------------ENNEIQ-----VDDAAKIV
gi|540072553|gb|ERF78672.1|hypotheticalproteinN561_04930[Gallibacteriumanatis12656/12]/11651    RLEY----LLNR-----------------------------ENNEIQ-----VDDAAKIV
gi|917509519|ref|WP_052115936.1|damageinducibleprotein[Gallibacteriumanatis]_gi|702414161|gb|KGQ42233.1|damageinducibleprotei/1    RLEY----LLNR-----------------------------ENNEIQ-----VDDAAKIV
gi|746082942|ref|WP_039150268.1|damageinducibleprotein[Gallibacteriumanatis]_gi|703617078|gb|KGQ63194.1|damageinducibleprotei/1    RLEY----LLNR-----------------------------ENNEIQ-----VDDAAKIV
```

```
gi|822522885|ref|WP_046952840.1|damageinducibleprotein[Haemophilushaemolyticus]_gi|821501762|gb|KKZ59267.1|damageinduciblepro/1    RLEK----LF-------------------------------EGTEIQ-----VDDAAKIV
gi|404381014|gb|EJZ77501.1|putativehelicase[Pasteurellamultocidasubsp.gallicidaP1059]/11681                                          RLEK----LF-------------------------------EGTEIQ-----VDDAAKIV
gi|298281660|gb|EFI23149.1|helicaseCterminaldomainprotein[Neisseriasp.oraltaxon014str.F0314]/11493                                  RLQE----LLRDD----------------------------TGSELK-----IDDAAKIV
gi|348013055|gb|EGY51980.1|helicasedomainprotein[Neisseriashayeganii871]/11481                                                      RLQG----LLAG-----------------------------GNNEIK-----VDDAAKIV
gi|496407140|ref|WP_009116004.1|DNAdamageinducibleprotein[Neisseriawadsworthii]_gi|349794756|gb|EGZ48566.1|helicasedomainprote/    RLQN----LLTD-----------------------------SNNELK-----VDDAAKIV
gi|517216441|ref|WP_018405259.1|DNAdamageinducibleprotein[Marinobacterlipolyticus]/11656                                             RLQS----LLKD-----------------------------ADNSLK-----VDDAAKIV
gi|738230264|ref|WP_036185691.1|damageinducibleprotein[Marinobactersp.ELB17]/11671                                                  RLQS----LLKD-----------------------------ADNSLK-----VDDAAKIV
gi|447197741|ref|WP_001274997.1|DNAdamageinducibleprotein[Acinetobacterbaumannii]_gi|408512324|gb|EKK13969.1|typeIIIrestrictio/    QLQK----MLSD-----------------------------ESNSLK-----VDDAAKIV
gi|820775897|ref|WP_046739909.1|damageinducibleprotein[Acinetobactersp.AG1]_gi|819909758|gb|KKW75938.1|damageinducibleprotein/1    QLQG----LLKD-----------------------------ENNSLK-----VDDAAKIV
gi|584595163|ref|WP_024160846.1|helicase[Acinetobacterpittii]_gi|576702530|emb|CDI28049.1|helicasedomaincontainingprotein[Acin/    QLQG----LLKD-----------------------------ENNSLK-----VDDAAKIV
gi|514963042|ref|WP_016651366.1|hypotheticalprotein[Acinetobactersp.NIPH2036]_gi|514162665|gb|EPG41391.1|hypotheticalproteinF90    QLQG----LLKD-----------------------------ENNSLK-----VDDAAKIV
gi|515965564|ref|WP_017396147.1|DNAdamageinducibleprotein[Acinetobacterhaemolyticus]/11667                                          QLQG----LLKD-----------------------------ENNSLK-----VDDAAKIV
gi|491346810|ref|WP_005204747.1|MULTISPECIES:hypotheticalprotein[Acinetobacter]_gi|480335823|gb|ENX56033.1|hypotheticalproteinF    QLQS----LLKA-----------------------------ENNSLK-----VDDAAKIV
gi|693044472|ref|WP_032215268.1|damageinducibleprotein[Escherichiacoli]_gi|607725731|gb|EZE08230.1|damageinducibleprotein[Esc/1    RIQS----LLRD-----------------------------ENNQLK-----VDDAAKIV
gi|742382177|ref|WP_038861304.1|damageinducibleprotein[Cronobactermuytjensii]_gi|924333747|gb|ALB69766.1|damageinducibleprote/1    RIQS----LLRD-----------------------------ENNQLK-----VDDAAKIV
gi|915912804|ref|WP_050927035.1|damageinducibleprotein[Yersiniaenterocolitica]_gi|899805802|emb|CQH32042.1|typeIrestrictionenz/    RIQS----LLRD-----------------------------ENNQLK-----VDDAAKIV
gi|921111327|gb|KON62781.1|typeIrestrictionenzymeEcoKIsubunitR[Komagataeibactereuropaeus]/11492                                     SVQK----LLDD-----------------------------PDNGLT-----VSDASKIV
gi|582024278|gb|AHI27615.1|DNAhelicaserestrictionenzymeTypeIIIRsubunit[KomagataeibacterxylinusE25]/11636                            SVQK----LLDD-----------------------------PDNGLT-----VSDASKIV
gi|347662673|dbj|BAK86128.1|DNAhelicaserestrictionenzymeTypeIIIRsubunit[KomagataeibactermedellinensisNBRC3288]/11737               SVQK----LLDD-----------------------------PDNGLT-----VSDASKIV
gi|917445053|ref|WP_052051471.1|damageinducibleprotein[Acetobactertropicalis]/11429                                                 RLQK----LLDD-----------------------------PENGLK-----VDDAAKII
gi|453329445|dbj|GAC88472.1|DNAhelicaserestrictionenzymetypeIIIRsubunit[GluconobacterthailandicusNBRC3255]_gi|530287981|dbj|GAD    RLQK----LLDD-----------------------------PDNGLK-----VDDAAKIV
gi|889770922|ref|WP_048844278.1|damageinducibleprotein[Gluconobacterfrateurii]/11419                                                RLQK----LLDD-----------------------------PDNGLK-----VDDAAKIV
gi|504886208|ref|WP_015073310.1|hypotheticalprotein[Gluconobacteroxydans]_gi|411027954|gb|AFW01209.1|hypotheticalproteinB932_16    RLQK----LLDD-----------------------------PDNGLK-----VDDAAKIV
gi|480040211|gb|ENV79751.1|hypotheticalproteinF942_01641[AcinetobacterursingiiANC3649]/11432                                        RLQD----LLAD-----------------------------ENNSLK-----VDDAAKII
gi|822498222|ref|WP_046936504.1|damageinducibleprotein[Xanthomonasgardneri]_gi|821751224|gb|KLB07223.1|damageinducibleprotein/1    KLQS----LLKD-----------------------------ENNSLR-----VDDAAKII
gi|654059559|ref|WP_027705556.1|damageinducibleprotein[Zymobacterpalmae]/11635                                                      RLQD----LLRD-----------------------------DENQLR-----VDDAAKIV
gi|643535620|ref|WP_025224315.1|damageinducibleprotein[Methylophilaceaebacterium11]_gi|577704400|gb|EUJ10546.1|putativehelicas/    NLQK----LLAD-----------------------------DNNQLK-----VDDAAKII
MBA11I/11635                                                                                                                         NLQK----LLAD-----------------------------DNNQLK-----VDDAAKII
gi|655355857|ref|WP_028762287.1|damageinducibleprotein[Shewanellacolwelliana]/11632                                                 KIQS----LLKD-----------------------------ENNELK-----VDDAAKII
gi|753204236|ref|WP_041510579.1|damageinducibleprotein[Shewanellasp.cp20]_gi|752457765|gb|KIO36326.1|damageinducibleprotein[S/1    RIQS----LLKD-----------------------------ENNTIK-----VDDAAKII
KPNNIH30III/11619                                                                                                                    RLQN----LLKD-----------------------------ESNTLK-----VGDAAKII
gi|749295994|ref|WP_040120352.1|damageinducibleprotein[Klebsiellapneumoniae]_gi|723249300|gb|AIX81926.1|damageinducibleprotei/1    RLQN----LLKD-----------------------------ESNTLK-----VGDAAKII
gi|641639739|emb|CDQ54010.1|unnamedproteinproduct[Klebsiellapneumoniae]/11641                                                       RLQN----LLKD-----------------------------ESNTLK-----VGDAAKII
gi|490797922|ref|WP_004660060.1|hypotheticalprotein[Acinetobactersp.NIPH236]_gi|479876779|gb|ENU27973.1|hypotheticalproteinF992    KIGS----TIT------------------------------ENSEIV-----VDDAARIV
gi|916750926|ref|WP_051357982.1|damageinducibleprotein[Acinetobactertjernbergiae]/11453                                             KIGA----TIT------------------------------DNKDIV-----VDDAARIV
gi|691098662|ref|WP_032025060.1|damageinducibleprotein[Acinetobacterbaumannii]_gi|593567521|gb|EXR50735.1|restrictionendonucle/    KIGS----TIT------------------------------ENSEIV-----VDDAARIV
gi|736669405|ref|WP_034676049.1|damageinducibleprotein[Acinetobacterbaumannii]/11643                                                KIGN----TIT------------------------------ENSEIV-----VDDAARIV
gi|491368928|ref|WP_005226826.1|hypotheticalprotein[Acinetobactersp.NIPH1847]_gi|480173259|gb|ENX09797.1|hypotheticalproteinF89    KIGS----TIT------------------------------ENSEIV-----VDDAARIV
gi|796853993|ref|WP_045795953.1|damageinducibleprotein[Acinetobacterindicus]_gi|787929761|gb|KJV44219.1|damageinducibleprotei/1    KIGS----TIT------------------------------ENSEIV-----VDDAARIV
gi|295797463|gb|ADG32253.1|typeIIIrestrictionproteinressubunit[ThiomonasintermediaK12]/11676                                        RLQT----LLAD-----------------------------ESNSLK-----VDDAARIV
gi|499871179|ref|WP_011551913.1|DNAdamageinducibleprotein[Myxococcusxanthus]_gi|108467544|gb|ABF92729.1|putativerestriction/mo/    RIQS----LLKD-----------------------------ANNELR-----VDDAAKIV
gi|495313852|ref|WP_008038600.1|DNAdamageinducibleprotein[Bartonellatamiae]_gi|395424181|gb|EJF90368.1|hypotheticalproteinME5_/    RLQN----LLKD-----------------------------ENNEVR-----VDDAAKIV
gi|501453335|ref|WP_012476784.1|DNAdamageinducibleprotein[Leptospirabiflexa]_gi|167777824|gb|ABZ96123.1|Helicase[Leptospirabif/    RLQK----LLAD-----------------------------EDKQIK-----VDDAAKII
gi|738104613|ref|WP_036063193.1|damageinducibleprotein[Leptospiranoguchii]/11652                                                    RLQK----LLAD-----------------------------EDNQLR-----MDDAAKIV
gi|410001039|gb|EKO51661.1|typeIIIrestrictionenzyme,ressubunit[Leptospirakirschneristr.200802841]/11651                             RLQK----LLAD-----------------------------EDNQLR-----MDDAAKIV
gi|490901851|ref|WP_004763774.1|typeIIIrestrictionenzyme,ressubunit[Leptospirakirschneri]_gi|464209249|gb|EMN24014.1|typeIIIres    RLQK----LLAD-----------------------------EDNQLR-----MDDAAKIV
gi|464398131|gb|EMO65902.1|typeIIIrestrictionenzyme,ressubunit[Leptospirakirschneristr.200803703]/11648                             RLQK----LLAD-----------------------------EDNQLR-----MDDAAKIV
gi|945029024|gb|KQQ84900.1|damageinducibleprotein[Xanthomonassp.Leaf131]/11423                                                      KLQA----LLRD-----------------------------ENNSLR-----VDDAAKIV
gi|896511314|ref|WP_049418183.1|damageinducibleprotein[Stenotrophomonasmaltophilia]/11639                                           RLQT----LLKD-----------------------------ESNTLK-----VDDAAKIV
gi|896513082|ref|WP_049419616.1|damageinducibleprotein[Stenotrophomonasmaltophilia]/11638                                           RLQE----LLKD-----------------------------ENNSLR-----VDDAAKIV
gi|896580616|ref|WP_049469425.1|damageinducibleprotein[Stenotrophomonasmaltophilia]/11641                                           RLQT----LLKD-----------------------------ENNSLR-----VDDAAKIV
gi|648289350|ref|WP_026070499.1|damageinducibleprotein[Stenotrophomonasmaltophilia]/11639                                           RLQT----LLKD-----------------------------ENNSLR-----VDDAAKIV
gi|656272786|ref|WP_029218442.1|damageinducibleprotein[Xanthomonascassavae]/11639                                                   RLQT----LLKD-----------------------------ENNSLR-----VDDAAKIV
gi|406976236|gb|EKD98747.1|hypotheticalproteinACD_23C00290G0002[unculturedbacterium]/11679                                          RIQD----LLKS-----------------------------EHNELR-----VDDAAKII
gi|828145737|ref|WP_047238120.1|damageinducibleprotein[Chromobacteriumsubtsugae]/11645                                              SLQN----LLKD-----------------------------DDNQIK-----VEDAAKIV
gi|654311174|ref|WP_027824327.1|damageinducibleprotein[Laribacterhongkongensis]/11641                                               SLQN----LLKD-----------------------------DNNQIK-----VEDAAKIV
gi|757242173|gb|KIR15516.1|typeIrestrictionenzymeEcoKIsubunitR[Pseudomonasfluorescens]/11778                                        RLQK----LLAD-----------------------------DDNQIK-----LEDAAKII
gi|653605161|ref|WP_027616682.1|damageinducibleprotein[Pseudomonassp.URHB0015]/11639                                                RLQK----LLAD-----------------------------DDNQIK-----LEDAAKII
gi|520868025|ref|WP_020308060.1|helicase[Pseudomonassyringae]_gi|519159034|gb|EPM49935.1|helicasedomaincontainingprotein[Pseud/    RLQK----LLAD-----------------------------DDNQIK-----LEDAAKII
gi|940156567|gb|KPX71668.1|Helicasedomaincontainingprotein[Pseudomonasamygdalipv.lachrymans]/11677                                  RLQK----LLAD-----------------------------DDNQIK-----LEDAAKII
gi|505460508|ref|WP_015647610.1|helicase[Pseudomonassp.GLE121]_gi|499110212|ref|YP_007974374.1|helicasedomaincontainingprotein/    RLQE----LFKD-----------------------------SDNQLK-----VDDAAKIV
gi|940235551|gb|KPY45311.1|hypotheticalproteinALO48_200154[Pseudomonassyringaepv.rhaphiolepidis]/11631                              RLQE----LFKD-----------------------------SDNQLK-----VDDAAKII
gi|520880808|ref|WP_020312235.1|helicase[Pseudomonassyringae]_gi|519172158|gb|EPM62027.1|helicasedomaincontainingprotein[Pseud/    RLQE----LLKD-----------------------------EDNQLK-----VDDAAKIV
gi|829046479|ref|WP_047282259.1|damageinducibleprotein[Pseudomonasfluorescens]/11630                                                RLQD----LLKD-----------------------------EDNQLK-----VDDAAKIV
gi|499415493|ref|WP_011102960.1|DNAdamageinducibleprotein[Pseudomonassyringaegroupgenomosp.3]_gi|28867279|ref|NP_789898.1|heli/    RLQE----LLKD-----------------------------EDNQLK-----VDDAAKIV
gi|730238299|ref|WP_033942897.1|damageinducibleprotein[Pseudomonasaeruginosa]/11629                                                 RLQD----MLKD-----------------------------ENNQLK-----VDDAAKIV
gi|495310125|ref|WP_008034873.1|DNAdamageinducibleprotein[Pseudomonassp.GM60]_gi|398186685|gb|EJM74052.1|putativehelicase[Pseu/    RLQD----LLKD-----------------------------ENNQLK-----VDDAAKIV
```

```
gi|779992653|ref|WP_045428690.1|damageinducibleprotein[Pseudomonassp.MT1]_gi|699967872|dbj|BAP80334.1|DNAhelicaserestrictione/1        RLQE----LLKD-----------------------------EDNQLK-----VDDAAKIV
gi|786037289|ref|WP_045733274.1|damageinducibleprotein[Pseudomonaspseudoalcaligenes]_gi|782987904|gb|KJU79152.1|damageinducib/1        RLQE----LLKD-----------------------------EDNQLK-----VDDAAKIV
gi|749666366|ref|WP_040265445.1|damageinducibleprotein[Pseudomonasrhodesiae]/11632        RLQE----LLKD-----------------------------EDNQLK-----VDDAAKIV
gi|734902433|ref|WP_034133698.1|damageinducibleprotein[Pseudomonasfluorescens]/11635        RLQE----LLKD-----------------------------EDNQLK-----VDDAAKIV
gi|490551031|ref|WP_004416146.1|typeIIrestrictionmodificationsystemrestriction/helicasesubunit[Pseudomonassyringae]_gi|4432823/        RLQE----MLKD-----------------------------ENNQLK-----VDDAAKIV
gi|489199911|ref|WP_003109152.1|DNAdamageinducibleprotein[Pseudomonasaeruginosa]_gi|552648708|gb|ERY63184.1|hypotheticalprotei/        RLQD----MLKD-----------------------------ENNQLK-----VDDAAKIV
gi|730641237|ref|WP_034061328.1|damageinducibleprotein[Pseudomonasaeruginosa]/11636        RLQD----MLKD-----------------------------ENNQLK-----VDDAAKIV
gi|751304980|ref|WP_041013386.1|damageinducibleprotein[Pseudomonasxanthomarina]_gi|703407611|emb|CEG51014.1|Helicasedomaincon/1        RLQE----LLKD-----------------------------DDNQLK-----VDDAAKIV
gi|939335207|ref|WP_054881520.1|damageinducibleprotein[Pseudomonassp.NBRC111128]/11632        RLQE----MLKD-----------------------------ENNQLK-----VDDAAKIV
gi|757693555|ref|WP_042923792.1|damageinducibleprotein[Pseudomonaschloritidismutans]_gi|780910610|gb|KJS78302.1|damageinducib/1        RLQE----LLRD-----------------------------DDNQLK-----VDDAAKII
gi|665953944|ref|WP_031319989.1|damageinducibleprotein[Pseudomonastolaasii]/11626        RLQD----MLKD-----------------------------ENNQLK-----VDDAAKIV
gi|771600573|ref|WP_045213746.1|damageinducibleprotein[Pseudomonassp.21]_gi|770081123|gb|KJJ98634.1|damageinducibleprotein[Ps/1        RLQD----LLKD-----------------------------ENNQLK-----VDDAAKIV
gi|740871396|ref|WP_038656644.1|damageinducibleprotein[Pseudomonasstutzeri]_gi|627772214|gb|AHY41437.1|damageinducibleprotein/1        RLQD----LLKD-----------------------------EDNQLK-----VDDAAKIV
gi|946242205|gb|KRC93782.1|damageinducibleprotein[Pseudomonassp.Root9]/11634        RLQE----MLKD-----------------------------ENNQLK-----VDDAAKIV
gi|553777322|ref|WP_023109107.1|MULTISPECIES:hypotheticalprotein[Pseudomonas]_gi|552638674|gb|ERY53184.1|hypotheticalproteinQ05        RLQE----MLKD-----------------------------ENNQLK-----VDDAAKIV
gi|896383370|ref|WP_049324618.1|damageinducibleprotein[Pseudomonasstutzeri]/11627        RLQE----LLKD-----------------------------EDNQLK-----VDDAAKIV
gi|696158510|gb|AIT41787.1|putativehelicase[Pseudomonassp.LM8]/11630        RLQE----LLKD-----------------------------EDNQLK-----VDDAAKIV
gi|504634892|ref|WP_014821994.1|DNAdamageinducibleprotein[Pseudomonasstutzeri]_gi|390985249|gb|AFM35242.1|helicasedomainconta/1        RLQD----LLKD-----------------------------EDNQLK-----VDDAAKIV
gi|544783974|ref|WP_021206729.1|DNAdamageinducibleprotein[Pseudomonasstutzeri]_gi|533224779|gb|EQM79688.1|DNAdamageinduciblep/1        RLQE----LLKD-----------------------------EDNQLK-----VDDAAKIV
gi|504567385|ref|WP_014754487.1|MULTISPECIES:DNAdamageinducibleprotein[Pseudomonas]_gi|388559493|gb|AFK68634.1|helicasedomain/1        RLQD----MLKD-----------------------------ENNQLK-----VDDAAKIV
gi|654441469|ref|WP_027912052.1|damageinducibleprotein[Pseudomonassp.URIL14HWK12:I7]/11630        RLQE----MLKD-----------------------------ENNQLK-----VDDAAKIV
gi|941470066|ref|WP_055127894.1|damageinducibleprotein[Pseudomonasmediterranea]/11631        RLQE----MLKD-----------------------------ENNQLK-----VDDAAKIV
gi|557815599|ref|WP_023446184.1|DNAdamageinducibleprotein[Pseudomonaschloritidismutans]_gi|557364081|gb|ESQ98190.1|DNAdamagei/1        RLQE----MLKD-----------------------------ENNQLK-----VDDAAKIV
gi|727814791|ref|WP_033898247.1|damageinducibleprotein[Pseudomonassp.FH1]_gi|567648005|gb|ETK23229.1|helicasedomaincontaining/1        RLQE----LLKD-----------------------------EDNQLK-----VDDAAKIV
gi|503278702|ref|WP_013513363.1|DNAdamageinducibleprotein[Desulfovibrioaespoeensis]_gi|316942375|gb|ADU61426.1|typeIIIrestrict/        RIQD----LLKS-----------------------------GDNQLK-----VDDAAKII
gi|505228796|ref|WP_015415898.1|typeIIIrestrictionendonucleasesubunitR[Desulfovibriopiezophilus]_gi|451896976|emb|CCH49855.1|Ty        RIQD----LLKS-----------------------------KDNQLK-----VDDAAKII
gi|517732543|ref|WP_018902751.1|hypotheticalprotein[Variovoraxparadoxus]/11675        RLQS----LLRD-----------------------------ENNQLK-----VDDAAKIV
gi|550909475|ref|WP_022662570.1|DNAdamageinducibleprotein[Desulfovibriolongus]/11637        RLQD----LLKD-----------------------------ENNQLK-----VDDAAKIV
gi|945494542|gb|KQV50024.1|damageinducibleprotein[Massiliasp.Root335]/11647        RIQS----LLKD-----------------------------ENNQLK-----VDDAAKII
gi|334196917|gb|AEG70102.1|helicasedomainprotein[RalstoniasolanacearumPo82]/11663        RLQD----LLRD-----------------------------DNNQLK-----VDDAARIV
gi|656006746|ref|WP_029048170.1|damageinducibleprotein[Cupriavidussp.amp6]/11661        RLQD----LLRD-----------------------------DNNQLK-----VDDAARIV
BPS2196ORF3054P/11659        RLQD----LLRD-----------------------------ENNQLK-----VDDAAKIV
gi|740932312|ref|WP_038717332.1|damageinducibleprotein[Burkholderiapseudomallei]_gi|760211882|gb|KIX58314.1|damageinduciblepr/1        RLQD----LLRD-----------------------------ENNQLK-----VDDAAKIV
gi|755810813|ref|WP_042583861.1|damageinducibleprotein[Burkholderiasp.MSHR3999]_gi|755174952|gb|KIP19790.1|DEAD/DEAHboxhelicas/        RLQD----LLRD-----------------------------ENNQLK-----VDDAAKIV
gi|484586570|gb|AGK48195.1|DEAD/DEAHboxhelicasefamilyprotein[BurkholderiathailandensisMSMB121]/11646        RLQD----LLRD-----------------------------DNNQLK-----VDDAAKIV
gi|737582935|ref|WP_035553941.1|damageinducibleprotein[Burkholderiasp.9120]/11658        RLQG----LLRD-----------------------------DNNQLK-----VDDAAKIV
gi|740989872|ref|WP_038773225.1|damageinducibleprotein[Burkholderiapseudomallei]_gi|705645291|gb|KGS70691.1|DEAD/DEAHboxhelica/        RLQD----LLRD-----------------------------ENNQLK-----VDDAAKIV
gi|654912525|ref|WP_028363424.1|damageinducibleprotein[Burkholderiasp.JPY366]/11659        RLQD----LLRD-----------------------------ENNQLK-----VDDAAKIV
gi|740955879|ref|WP_038740512.1|damageinducibleprotein[Burkholderiapseudomallei]_gi|714684932|gb|KGW74438.1|DEAD/DEAHboxhelica/        RLQD----LLRD-----------------------------ENNQLK-----VDDAAKIV
gi|919203714|ref|WP_052759215.1|damageinducibleprotein[Xanthomonasaxonopodis]/11388        TFQS----QLAD-------------------------K----NKELN-----LDDVVKIV
gi|522152381|ref|WP_020663589.1|hypotheticalprotein[Amycolatopsisbenzoatilytica]/11605        SLQS----QVAD-------------------------E----NNEIN-----LDDAVKIV
gi|663196013|ref|WP_030233899.1|damageinducibleprotein[Streptomycessp.NRRLS455]/11641        SYQQ----GLAA-------------------------G----GSELN-----LDDAAKII
gi|750429553|ref|WP_040711444.1|damageinducibleprotein[Nocardiopsispotens]/11622        TLQK----GLAS-------------------------G----DSELN-----LDDAAKII
gi|518656139|ref|WP_019819475.1|hypotheticalprotein[Saccharomonosporasaliphila]/11623        TLQQ----GFTG-------------------------G----DSELN-----LDDAAKII
gi|759722933|ref|WP_043433758.1|damageinducibleprotein[Streptomycespluripotens]_gi|733404978|gb|KHK94361.1|damageinduciblepro/1        TLQE----GFAG-------------------------G----DSELR-----LDDAAKII
gi|663424167|ref|WP_030419030.1|damageinducibleprotein[Streptomycessp.NRRLF5065]/11627        TLQE----GFAG-------------------------G----DSELR-----IDDAAKII
gi|663412917|ref|WP_030408353.1|damageinducibleprotein[Streptomycessp.NRRLF5917]/11630        TIQE----GFAG-------------------------G----DSELR-----LDDAAKII
gi|671521483|ref|WP_031506489.1|damageinducibleprotein[Streptomycesmegasporus]/11630        TLQE----GFAG-------------------------G----DSELR-----LDDAAKII
gi|740053144|ref|WP_037902776.1|damageinducibleprotein[Streptomycessp.TAA204]/11626        TLQQ----GIAS-------------------------G----SSELN-----LNDAAKII
gi|663409375|ref|WP_030404974.1|MULTISPECIES:damageinducibleprotein[Streptomyces]/11627        TLQQ----GIAG-------------------------G----SSELN-----LDDAAKII
gi|746628608|ref|WP_039654277.1|damageinducibleprotein[Streptomycessp.MUSC125]_gi|745644381|gb|KIE25557.1|damageinducibleprot/1        TLQQ----GLAG-------------------------G----SSELD-----LGDAAKII
gi|727322066|ref|WP_033777320.1|damageinducibleprotein[Salinisporapacifica]/11627        TLQQ----GLAG-------------------------G----DGELR-----LDDAAKII
gi|653653242|ref|WP_027655726.1|damageinducibleprotein[Salinisporapacifica]/11618        TLQQ----GLAG-------------------------G----DAELR-----LDDAAKII
gi|648632543|ref|WP_026324294.1|damageinducibleprotein[Salinisporapacifica]/11621        TLQQ----GLAG-------------------------G----DSELR-----LDDAAKII
gi|881018002|ref|WP_048738463.1|damageinducibleprotein[Corynebacteriumfalsenii]/11676        ALAA----S----------------------------P----GQNVN-----LNLAASMI
gi|505465526|ref|WP_015652417.1|helicase[Corynebacteriumcallunae]_gi|459385704|gb|AGG67995.1|helicase[CorynebacteriumcallunaeDS        ALTV----LGST-------------------------P----GEELT-----LDMTSAII
gi|41223062|emb|CAF19007.1|DNAorRNAhelicaseofsuperfamilyII[CorynebacteriumglutamicumATCC13032]/11646        ALTV----LGST-------------------------P----GEELT-----LDMTSAII
CGL13032I/11643        ALTV----LGST-------------------------P----GEELT-----LDMTSAII
gi|751257081|ref|WP_040967675.1|damageinducibleprotein[Corynebacteriumglutamicum]_gi|748811620|gb|AJE67931.1|damageinduciblep/1        TLAR----FTPQ-------------------------P----GQELT-----LDLASAMI
gi|749040539|ref|WP_040086524.1|damageinducibleprotein[Corynebacteriumhumireducens]/11663        TMAR----FTPQ-------------------------P----GQELT-----LDMASAMI
gi|491662899|ref|WP_005519615.1|helicase[Corynebacteriummatruchotii]_gi|224946756|gb|EEG27965.1|helicaseCterminaldomainprotein/        AMAR----FTGG-------------------------S----GQELT-----LSLASAMI
gi|746717312|ref|WP_039676231.1|damageinducibleprotein[Corynebacteriumminutissimum]_gi|734991118|gb|KHO29219.1|damageinducibl/1        VLAA----N----------------------------P----TNEIN-----LTTASAMI
gi|908654892|emb|CQD15529.1|SuperfamilyIIDNA/RNAhelicase(modularprotein)[Corynebacteriumstriatum]/11674        VLAA----N----------------------------P----TNEIN-----LTTASAMI
gi|895994446|ref|WP_049064418.1|damageinducibleprotein[Corynebacteriumaurimucosum]/11667        VLAA----N----------------------------P----TNEIN-----LTTASAMI
gi|896123331|ref|WP_049145390.1|damageinducibleprotein[Corynebacteriumaurimucosum]/11669        VLAA----N----------------------------P----TNEIN-----LTTASAMI
gi|817681178|ref|WP_046645422.1|damageinducibleprotein[Corynebacteriumstriatum]_gi|817551679|gb|KKO79868.1|damageinduciblepro/1        VLAA----N----------------------------P----TNEIN-----LTTASAMI
gi|896139239|ref|WP_049159146.1|damageinducibleprotein[Corynebacteriumaurimucosum]/11675        VLAA----N----------------------------P----TNEIN-----LTTASAMI
```

```
gi|489943947|ref|WP_003847254.1|helicase[Corynebacteriumammoniagenes]_gi|295968218|gb|EFG81467.1|helicaseCterminaldomainprotei/  ALAS----A----------------------------P----SPDIN-----LTTASAMI
gi|516653464|ref|WP_018022453.1|helicase[Corynebacteriumdoosanense]_gi|697778282|gb|AIT61018.1|damageinducibleprotein[Coryneba/  AMAF----A----------------------------P----GQDVN-----LTTASAMI
gi|493894179|ref|WP_006840129.1|helicase[Corynebacteriumlipophiloflavum]_gi|227078819|gb|EEI16782.1|helicaseCterminaldomainpro/  TLAR----G----------------------------D-----QEIN-----LSLASAMI
gi|740831472|ref|WP_038616755.1|damageinducibleprotein[Corynebacteriumpseudotuberculosis]_gi|666103058|gb|AIG06935.1|putativeh/  ALAH----S----------------------------P----SELLN-----LTTASAII
gi|518023322|ref|WP_019193530.1|helicase[Corynebacteriumtimonense]/11655  ALAH----S----------------------------P----NDLVN-----LTTASAII
gi|552767697|ref|WP_023019559.1|MULTISPECIES:hypotheticalprotein[Corynebacterium]_gi|550739013|gb|ERS38609.1|hypotheticalprotei  ALAN----A----------------------------P----GQLVN-----LTTASAII
gi|652659121|ref|WP_027017812.1|damageinducibleprotein[Corynebacteriumpseudodiphtheriticum]/11644  ALAN----A----------------------------P----GQLVN-----LTTASAII
gi|749039309|ref|WP_040085390.1|damageinducibleprotein[Corynebacteriumhumireducens]/11657  AMAR----G----------------------------P----GQSVN-----LSLASAMI
gi|740835591|ref|WP_038620874.1|damageinducibleprotein[Corynebacteriumulcerans]_gi|698973854|gb|AIU33664.1|HelicaseCterminald/1  AMTR----H----------------------------T----QEGMN-----LTTASAMI
gi|805307599|ref|WP_046096452.1|damageinducibleprotein[Corynebacteriumulcerans]_gi|803357323|gb|AKA97638.1|Damageinduciblepro/1  AMAH----H----------------------------T----QEGMN-----LTTASAMI
gi|504083555|ref|WP_014317549.1|helicase[Corynebacteriumdiphtheriae]_gi|371584249|gb|AEX47914.1|superfamilyIIDNA/RNAhelicase[Co  AMAR----G----------------------------A----GQEIN-----LTLASAMI
gi|896182193|ref|WP_049193580.1|damageinducibleprotein[Corynebacteriumjeikeium]/11645  AMAR----G----------------------------A----GQEIN-----LTLASAMI
gi|917636459|ref|WP_052205460.1|damageinducibleprotein[Corynebacteriumriegelii]_gi|914295811|gb|AKV59187.1|damageinduciblepro/1  AMAR----N----------------------------T----QEGMN-----LTTASAMI
gi|737013522|ref|WP_035009205.1|damageinducibleprotein[Corynebacteriumjeikeium]/11651  AMAR----S----------------------------T----QEGMN-----LTTASAMI
gi|737006291|ref|WP_035002094.1|damageinducibleprotein[Corynebacteriumjeikeium]/11658  AMAR----G----------------------------E----GGQLN-----LSLASAMI
gi|737010378|ref|WP_035006133.1|damageinducibleprotein[Corynebacteriumjeikeium]/11650  AMAR----G----------------------------E----DGQLN-----LTLASAMI
gi|737010473|ref|WP_035006228.1|damageinducibleprotein[Corynebacteriumjeikeium]/11657  ALSR----S----------------------------Q----GSGVN-----LTLASAMI
gi|737006250|ref|WP_035002053.1|damageinducibleprotein[Corynebacteriumjeikeium]/11658  ALSR----S----------------------------Q----GSGVN-----LTLASAMI
gi|923018168|ref|WP_053412254.1|damageinducibleprotein[Corynebacteriumlactis]_gi|922845184|gb|ALA67504.1|helicase[Corynebacter/  AMAR----S----------------------------E----NNQIN-----LTLASAMI
gi|923018885|ref|WP_053412970.1|damageinducibleprotein[Corynebacteriumlactis]_gi|922845845|gb|ALA68165.1|helicase[Corynebacter/  AMAR----S----------------------------E----NNQIN-----LTLASAMI
gi|512464870|ref|WP_016422869.1|hypotheticalprotein[Corynebacteriumsp.HFH0082]_gi|512047830|gb|EPD47505.1|hypotheticalproteinHM  AINR----S----------------------------Q----NSQIN-----LTLASAMI
gi|881012697|ref|WP_048733406.1|damageinducibleprotein[Corynebacteriumurealyticum]/11670  AINR----S----------------------------Q----NSQIN-----LTLASAMI
gi|755608478|ref|WP_042530519.1|damageinducibleprotein[Corynebacteriumsingulare]_gi|754340239|gb|AJI78730.1|putativehelicase[C/  AMAQ----S----------------------------E----NNQVN-----LSLASAMI
gi|491421796|ref|WP_005279593.1|helicase[Corynebacteriumaccolens]_gi|227077180|gb|EEI15143.1|helicaseCterminaldomainprotein[Co/  AMAH----S----------------------------E----NNQIN-----LTLASAMI
gi|550752286|gb|ERS51451.1|hypotheticalproteinHMPREF1282_00033[Corynebacteriumsp.KPL1856]_gi|550759961|gb|ERS58964.1|hypothetic  AMAR----S----------------------------D----DNQIN-----LTTASAMI
gi|497955053|ref|WP_010269209.1|helicase[Corynebacteriumbovis]/11599  ATQD----LVED-------------------------D----GTEIR-----LDDVARLI
gi|860580040|ref|WP_048470569.1|hypotheticalprotein[Mycobacteriumchlorophenolicum]_gi|859098575|gb|KMO76871.1|typeIrestrictione  PMQQ----QLA--------------------------T----LGELP-----LDDASKIV
gi|262085905|gb|ACY21873.1|typeIIIrestrictionproteinressubunit[GordoniabronchialisDSM43247]/11632  PMQK----QLAG-------------------------E----FAELQ-----LDDASRIV
gi|495996338|ref|WP_008720917.1|typeIIIrestrictionendonucleasesubunitR[Rhodococcussp.AW25M09]_gi|443412350|emb|CCQ18455.1|TypeI  PMQK----QLAG-------------------------T----YSELQ-----LDDASKIV
gi|739284636|ref|WP_037147410.1|damageinducibleprotein[Rhodococcusfascians]/11641  PMQK----QLAG-------------------------N----FAELQ-----LDDASKIV
gi|739328195|ref|WP_037190574.1|damageinducibleprotein[Rhodococcusfascians]/11643  PMQK----QLAG-------------------------T----YSELQ-----LDDASKIV
gi|811698335|emb|CMN21041.1|helicase[Mycobacteriumtuberculosis]/11725  RLQQ----ELSG-------------------------V----SGELM-----LDDASKIV
gi|801577898|emb|CFK23773.1|helicase[Mycobacteriumtuberculosis]/11675  RLQQ----ELSG-------------------------V----SGELM-----LDDASKIV
MTU18II/11606  RLQQ----ELSG-------------------------V----SGELM-----LDDASKIV
gi|887719858|emb|CMT27886.1|helicase[Mycobacteriumtuberculosis]/11667  RLQQ----ELSG-------------------------V----SGELM-----LDDASKIV
gi|621694743|gb|KAS87156.1|hypotheticalproteinAL70_01801[MycobacteriumtuberculosisTKK_03_0115]/11604  RLQQ----ELSG-------------------------V----SGELM-----LDDASKIV
gi|888128072|emb|CLQ84422.1|helicase[Mycobacteriumtuberculosis]_gi|889917090|emb|CLY86893.1|helicase[Mycobacteriumtuberculosis]  RLQQ----ELSG-------------------------V----SGELM-----LDDASKIV
gi|884823424|emb|CKL96672.1|helicase[Mycobacteriumtuberculosis]/11466  RLQQ----ELSG-------------------------V----SGELM-----LDDASKIV
gi|505103239|ref|WP_015290341.1|hypotheticalprotein[Mycobacteriumcanettii]_gi|432162733|emb|CCK60117.1|Conservedproteinofunknow  RLQQ----ELSG-------------------------V----FGELM-----LDDASKIV
gi|505116250|ref|WP_015303352.1|hypotheticalprotein[Mycobacteriumcanettii]_gi|432158726|emb|CCK56026.1|Conservedproteinofunknow  RLQQ----ELSG-------------------------V----SGELM-----LDDASKIV
gi|432154735|emb|CCK51974.1|Conservedproteinofunknownfunction(part1)[MycobacteriumcanettiiCIPT140060008]/11588  RLQQ----ELSG-------------------------V----SGELM-----LDDASKIV
gi|809008027|ref|WP_046295375.1|hypotheticalprotein[Mycobacteriumsp.UM_Kg17]/11624  GLQR----QLSA-------------------------I----DGELT-----LDDASKLV
gi|808802633|ref|WP_046284139.1|hypotheticalprotein[Mycobacteriumsp.UM_NZ2]/11624  GLQQ----QVAA-------------------------H----GSELT-----LDDASKLV
gi|750125774|ref|WP_040429852.1|damageinducibleprotein[Corynebacteriumnuruki]/11668  KLQG----SLA--------------------------T----DGELN-----LDDASKIV
gi|602237614|gb|EYT60960.1|damageinducibleprotein[Dietziasp.UCDTHP]/11473  RMQD----QFA--------------------------V----DGELS-----LDDASKIV
gi|665897412|ref|WP_031264618.1|damageinducibleprotein[Dietziasp.UCDTHP]_gi|602237599|gb|EYT60945.1|helicase[Dietziasp.UCDTH/11  RMQD----QFA--------------------------V----DGELS-----LDDASKIV
gi|502903674|ref|WP_013138650.1|helicase[Segniliparusrotundus]_gi|296181291|gb|ADG98197.1|typeIIIrestrictionproteinressubunit[S  PMQT----QLAG-------------------------Q----NHELN-----LDDATRIV
gi|780248306|ref|WP_045538930.1|hypotheticalprotein[Gordoniasp.no.9]_gi|778142514|gb|KJR06917.1|hypotheticalproteinUG54_12320[G  PMQE----QLAG-------------------------P----DNELS-----LDDATRIV
gi|665897368|ref|WP_031264574.1|damageinducibleprotein[Dietziasp.UCDTHP]_gi|602239137|gb|EYT62483.1|helicase[Dietziasp.UCDTH/11  RLQS----DLA--------------------------D----AGELK-----LDDASKLV
gi|493372923|ref|WP_006329236.1|helicase[Gordoniarhizosphera]_gi|403207917|dbj|GAB88168.1|hypotheticalproteinGORHZ_006_00370[Go  RLQK----ELAG-------------------------D----FNELD-----LDDASKII
gi|764904870|ref|WP_044487821.1|hypotheticalprotein[Mycobacteriumtuberculosis]_gi|625005353|gb|KBZ57120.1|hypotheticalproteinK8  PLQT----QLAG-------------------------G----DGELR-----LDDATKIV
gi|927068212|ref|WP_053778441.1|damageinducibleprotein[Gordoniasp.NB41Y]_gi|926660541|gb|EMP14526.2|damageinducibleprotein[G/11  PLQT----QLAG-------------------------P----DGDLR-----LDDATKIV
gi|935514169|ref|WP_054429894.1|damageinducibleprotein[Mycobacteriumimmunogenum]_gi|929801120|gb|KPG29247.1|damageinduciblepr/1  PLQG----QLAG-------------------------G----EGELR-----LDDATKIV
gi|941317728|ref|WP_055118574.1|hypotheticalprotein[Mycobacteriumperegrinum]/11657  PLQG----QLAG-------------------------G----EGELR-----LDDATKIV
gi|933683673|gb|ALG86377.1|hypotheticalproteinACH46_20120[Gordoniasp.QH11]/11636  PMQK----QLAG-------------------------E----FSELK-----LDDASKIV
gi|873553947|emb|CRY84110.1|Predictedhelicase[Nocardiafarcinica]/11688  PLQQ----QLAG-------------------------Q----FSELR-----LDDASKIV
gi|499525749|ref|WP_011212389.1|helicase[Nocardiafarcinica]_gi|54019339|dbj|BAD60707.1|hypotheticalprotein[NocardiafarcinicaIFM  PLQQ----QLAG-------------------------R----FSELR-----LDDASKIV
gi|640109609|ref|WP_024794187.1|damageinducibleprotein[Tomitellabiformata]/11653  PLQG----QLAG-------------------------D----YGELR-----LDDASKIV
gi|640109991|ref|WP_024794334.1|hypotheticalprotein[Tomitellabiformata]/11639  PLQG----QLAG-------------------------N----VGELR-----LDDASKIV
gi|939125496|ref|WP_054831438.1|damageinducibleprotein[Rhodococcuserythropolis]/11669  PLQD----QLAG-------------------------V----HSELR-----LDDASKII
gi|910258550|ref|WP_050062711.1|hypotheticalprotein[Rhodococcussp.RD6.2]_gi|846871967|emb|CRK49225.1|conservedhypotheticalprote  PLQQ----QLAG-------------------------E----FSELR-----LDDASKIV
gi|797148189|gb|KJY52358.1|putativeHelicase[Bifidobacteriumasteroides]/11801  QMTS----TQTMRD------EYDKIEAQRRRSRKADRDTLAETRKAKIAEQAQSFAGKIV
gi|535476570|gb|EFG27357.2|hypotheticalproteinHMPREF9020_00999[ScardoviainopinataF0304]/11827  RIQE----TQTFQE------QIDSIEQLERRSRAKDKAEKAAQDKLKIRTQAESLSAKVL
gi|737482729|ref|WP_035462496.1|hypotheticalprotein[Alicyclobacillusmacrosporangiidus]/11512  QFVE----WLQE------------------------------SDAPS-----IPHLVRVH
gi|685434354|gb|KGA95679.1|helicase[BacillusalcalophilusATCC27647=CGMCC1.3604]/11575  RFQQ----MLA------------------------------RNDELE-----FDDVTKII
gi|945370253|gb|KQU27251.1|helicase[Bacillussp.Leaf13]/11557  RFQQ----MLAN-----------------------------KDSELE-----FDDVTKII
gi|880997015|ref|WP_048719116.1|helicase[Enterococcushirae]/11563  RFQN----VFSD-----------------------------DNGELK-----FDDVTKII
```

gi|945356929|gb|KQU14100.1|helicase[Bacillussp.Leaf49]/11547 RFQY----MLAK-----------------------------DNSELE-----FDDITKIV
gi|763482876|gb|KIZ53217.1|helicase[Bacillussafensis]/11563 RFQQ----MLA------------------------------RESELE-----FDDVTKII
gi|852223292|ref|WP_048309707.1|helicase[Anaerobacillusmacyae]_gi|850322910|gb|KMM38590.1|helicase[Anaerobacillusmacyae]/11573 RFQT----MLANN----------------------------QDTELE-----FDDVTKII
gi|739708665|ref|WP_037562619.1|helicase[Sporolactobacillusterrae]/11573 HFQT----MLANK----------------------------RDTELE-----FDDVTKII
gi|496687561|ref|WP_009329104.1|MULTISPECIES:helicase[Bacillus]_gi|317390867|gb|EFV71668.1|endonucleaseandmethylaseLlaGI[Bacill SFQN----MLANK----------------------------RDTELE-----FDDVTKII
gi|930213599|gb|KPH74225.1|helicase[Oceanobacilluscaeni]/11569 SFQN----MLANK----------------------------RDTELE-----FDDVTKII
gi|516366003|ref|WP_017756036.1|helicase[Bacillussp.ZYK]/11571 RFQT----LLANK----------------------------RDTELE-----FDDVTKII
gi|488909977|ref|WP_002821052.1|helicase[Oenococcusoeni]_gi|399965230|gb|EJN99855.1|endonucleaseandmethylaseLlaGI[Oenococcusoen DMQQ----SLAD-----------------------------AENGLN-----IDDIGRII
gi|495019967|ref|WP_007745780.1|helicase[Oenococcuskitaharae]_gi|366983682|gb|EHN59081.1|typeIIrestrictionenzyme,ressubunit[Oe DMQQ----SLSD-----------------------------AENGLN-----IDDIGRII
gi|489666119|ref|WP_003570406.1|typeIIrestrictionendonuclease[Lactobacilluscasei]_gi|410527834|gb|EKQ02696.1|typeIIrestrictio DLQS----SLS------------------------------TENGLG-----INDIGKII
gi|511447623|gb|EPC78110.1|superfamilyIIDNA/RNAhelicase[Lactobacillusparacaseisubsp.paracaseiLpp221]/11615 DMQD----SLA------------------------------TENGLN-----IDDIGKII
gi|497700411|ref|WP_010014595.1|helicase[Lactobacilluscoryniformis]/11585 NLQS----SLA------------------------------TENGLN-----IDDIGKII
gi|489784048|ref|WP_003687939.1|helicase[Lactobacillusmali]_gi|394428539|gb|EJF01080.1|superfamilyIIDNA/RNAhelicase[Lactobacill DMQN----SLS------------------------------TENGLN-----IDDIGKII
gi|446780825|ref|WP_000858081.1|helicase[Staphylococcusaureus]_gi|374396604|gb|EHQ67835.1|typeIIIrestrictionenzyme,ressubunit[S NLQQ----TLAD------------------------------ENGLR-----MNDIGKII
gi|510796658|ref|WP_016171456.1|hypotheticalprotein[Enterococcusdispar]_gi|508167532|gb|EOT44083.1|hypotheticalproteinOMK_00227 NLQQ----TLAD-----------------------------PENGLN-----IDDVGRIV
gi|612613473|gb|EZS30411.1|hypotheticalproteinW603_02482[StaphylococcusaureusVET0353R]_gi|612881917|gb|EZU93992.1|hypotheticalp NLQQ----TLSD-----------------------------PENGLN-----IADIGRIV
gi|488332893|ref|WP_002402278.1|helicase[Enterococcusfaecalis]_gi|306513864|gb|EFM82466.1|helicaseCterminaldomainprotein[Enter/ DMQK----VLSD-----------------------------SENGLD-----VDDVSKLI
gi|921166029|ref|WP_053111806.1|helicase[Enterococcusdurans]_gi|915771660|gb|AKX85928.1|helicase[Enterococcusdurans]/11562 DMQK----VLSD-----------------------------SENGLD-----VDDVSKLI
gi|498525358|ref|WP_010825030.1|hypotheticalprotein[Enterococcusfaecalis]_gi|487059631|gb|EOJ86727.1|hypotheticalproteinWOC_005 DMQK----VLSD-----------------------------SENGLD-----VDDVSKLI
gi|498412624|ref|WP_010722070.1|hypotheticalprotein[Enterococcusfaecium]_gi|486634654|gb|EOF75253.1|hypotheticalproteinSGC_0253 DMQK----VLSD-----------------------------SENGLD-----VDDVSKLI
gi|498408534|ref|WP_010718364.1|hypotheticalprotein[Enterococcusfaecium]_gi|486609608|gb|EOF50873.1|hypotheticalproteinSCW_0257 DMQK----VLSD-----------------------------SENGLD-----VDDVSKLI
gi|498407475|ref|WP_010717483.1|hypotheticalprotein[Enterococcusfaecalis]_gi|486594855|gb|EOF36348.1|hypotheticalproteinSCK_005 DMQR----VLSD-----------------------------SENGLD-----VDDVSKLI
gi|514885392|ref|WP_016615344.1|helicase[Enterococcusfaecalis]_gi|514505843|gb|EPH71676.1|helicaseprotein[Enterococcusfaecalis0 DMQK----VLSD-----------------------------SENGLD-----VDDVSKLI
gi|325157603|gb|EGC69759.1|helicaseCterminaldomainprotein[EnterococcuscasseliflavusATCC12755]/11561 DMQK----VLSD-----------------------------SENGLD-----VDDVSKLI
gi|895856206|ref|WP_048967668.1|helicase[Enterococcusfaecalis]/11551 DMQR----VLSD-----------------------------SENGLD-----IDDVSKLI
gi|639458836|ref|WP_024636366.1|helicase[Enterococcusfaecium]_gi|658176502|gb|KEI51159.1|helicase[EnterococcusfaeciumUC7256]/1/ DMQK----ILNI-----------------------------EENGLG-----VDDVSKLI
gi|488266511|ref|WP_002337719.1|hypotheticalprotein[Enterococcusfaecium]_gi|430581409|gb|ELB19854.1|hypotheticalproteinOIQ_0351 DMQK----VLAD-----------------------------SENGLD-----VDDVSKLI
gi|446353356|ref|WP_000431211.1|helicase[Staphylococcusaureus]_gi|253726329|gb|EES95058.1|helicaseCterminaldomainprotein[Staph/ DMQR----TLAD-----------------------------SENGLN-----IDDVGRIV
gi|672712068|emb|CDM14336.1|DNAorRNAhelicaseofsuperfamilyII[StaphylococcusepidermidisPM221]/11464 DMQK----TLAD-----------------------------PENGLN-----IDDVGRIV
gi|896298990|ref|WP_049279905.1|helicase[Staphylococcusaureus]/11571 DMQK----TLAD-----------------------------PENGLN-----IDDVGRIV
gi|541903432|gb|ERH36190.1|hypotheticalproteinSEQU_00085[StaphylococcusequorumUMCCNS924]/11578 DMQK----TLAD-----------------------------PENGLN-----IDDVGRIV
gi|488289747|ref|WP_002360955.1|helicase[Enterococcusfaecalis]_gi|256682216|gb|EEU21911.1|endonucleaseandmethylaseLlaGI[Enteroc DMQR----TLAD-----------------------------PENGLN-----IDDVGRIV
gi|494392045|ref|WP_007207894.1|helicase[Enterococcusitalicus]_gi|315483798|gb|EFU74284.1|helicaseCterminaldomainprotein[Enter/ DMQR----TLAD-----------------------------PENGLN-----IDDVGRIV
gi|446350692|ref|WP_000428547.1|helicase[Bacilluscereus]_gi|228727580|gb|EEL78721.1|RestrictionmodificationsystemLlaBIII[Bacil/ DMQK----TLAD-----------------------------PENGLN-----IDDVGRIV
gi|488255302|ref|WP_002326510.1|hypotheticalprotein[Enterococcusfaecium]_gi|430480276|gb|ELA57459.1|hypotheticalproteinOGE_0473 DMQR----TLSD-----------------------------PENGLN-----IDDVGRIV
gi|843253770|ref|WP_047916698.1|helicase[Lactococcuspiscium]_gi|802124677|emb|CEN29502.1|EndonucleaseandmethylaseLlaGI,TypeISPr DMQR----TLAD-----------------------------PENGLN-----IDDVGRIV
LlaBIII/11578 DMQR----TLAD-----------------------------PENGLN-----IDDVGRIV
gi|499320531|ref|WP_011011023.1|helicase[Listeriainnocua]_gi|16415805|emb|CAC42019.1|pli0021[ListeriainnocuaClip11262]/11569 DMQR----TLAD-----------------------------PENGLN-----IDDVGRIV
gi|760026859|ref|WP_043709973.1|helicase[Weissellacibaria]_gi|758337165|gb|KIU20061.1|UvsWhelicase[Weissellacibaria]/11567 DMQR----TLAD-----------------------------PENGLN-----IDDVGRIV
LlaGI/11570 DMQR----TLAD-----------------------------PENGLN-----IDDVGRIV
gi|499988414|ref|WP_011669132.1|helicase[Lactococcuslactis]_gi|116109019|gb|ABJ74139.1|DNAorRNAhelicaseofsuperfamilyII[Lactococ DMQR----TLAD-----------------------------PENGLN-----IDDVGRIV
gi|685938360|ref|WP_031670319.1|helicase[Listeriamonocytogenes]_gi|590403387|gb|EXL23261.1|hypotheticalproteinX846_1956[Listeri DMQR----TLAD-----------------------------PENGLN-----IDDVGRIV
gi|530793219|gb|EQC56240.1|helicase[Lactococcuslactissubsp.cremorisTIFN5]_gi|530793247|gb|EQC56263.1|helicase[Lactococcuslactis DMQR----TLAD-----------------------------PENGLN-----IDDVGRIV
gi|695270006|ref|WP_032492075.1|hypotheticalprotein[Listeriamonocytogenes]_gi|695212872|ref|YP_009081522.1|hypotheticalproteinp DMQR----TLAD-----------------------------PENGLN-----IDDVGRIV
gi|504101310|ref|WP_014335297.1|helicase[Leuconostocmesenteroides]_gi|859247905|gb|AKO63885.1|helicase(plasmid)[Leuconostocmese DMQR----TLAD-----------------------------PENGLN-----IDDVGRIV
gi|488905190|ref|WP_002816265.1|helicase[Leuconostocmesenteroides]_gi|227351522|gb|EEJ41777.1|helicaseCterminaldomainprotein[L/ DMQR----TLAD-----------------------------PENGLN-----IDDVGRIV
gi|518121285|ref|WP_019291493.1|MULTISPECIES:hypotheticalprotein[Lactococcus]/11588 ELQQ----TMQ------------------------------GDNGLN-----LDDIGRII
gi|754857213|ref|WP_042217902.1|helicase[Lactococcusgarvieae]/11549 DMQK----SLSD-----------------------------PENGLN-----LDDVGRII
gi|640048455|ref|WP_024784762.1|helicase[Streptococcusmutans]/11564 DMQK----SLSD-----------------------------PENGLN-----IDDVGRII
gi|512695361|ref|WP_016476323.1|hypotheticalprotein[Veillonellasp.HPA0037]_gi|512079853|gb|EPD79018.1|hypotheticalproteinHMPREF DMQK----ALSD-----------------------------SENGLN-----IDDVGRII
gi|636963514|ref|WP_024403747.1|helicase[Streptococcussuis]/11574 DMQK----SLSD-----------------------------PENGLN-----IDDVGRII
gi|881115312|ref|WP_048802949.1|helicase[Staphylococcuspasteuri]/11573 DMQK----SLSD-----------------------------PENGLN-----IDDVGRII
gi|446063996|ref|WP_000141851.1|helicase[Streptococcusmitis]_gi|307618725|gb|EFN97864.1|endonucleaseandmethylaseLlaGI[Streptoco DMQK----ALAD-----------------------------SENGLN-----IDDVGRII
gi|488206609|ref|WP_002277817.1|restrictionmodificationsystemLlaBIII[Streptococcusmutans]_gi|449173033|gb|EMB75626.1|restricti/ DMQK----SLAD-----------------------------AENGLN-----IDDVGRII
gi|640023290|ref|WP_024781621.1|helicase[Streptococcusmutans]/11566 DMQK----SLAD-----------------------------AENGLN-----IDDVGRII
gi|814946581|ref|WP_046393781.1|helicase[Streptococcusuberis]_gi|810600885|gb|KKF57499.1|helicase[StreptococcusuberisB362]/1156 DMQK----SLSN-----------------------------PENGLD-----IDDVGRII
gi|765470762|ref|WP_044720336.1|helicase[Streptococcussuis]/11559 DMQK----SLSD-----------------------------PENGLN-----IDDVGRII
gi|654507127|ref|WP_027976443.1|helicase[Streptococcusdevriesei]/11558 DMQK----SLSD-----------------------------PENGLN-----IDDVGRII
gi|530812902|gb|EQC72481.1|DNAorRNAhelicaseofsuperfamilyII[Streptococcussp.HSISS2]/11570 DMQK----SLSD-----------------------------SENGLN-----IDDVGRII
gi|489158846|ref|WP_003068504.1|helicase[Streptococcusintermedius]_gi|355367114|gb|EHG14827.1|hypotheticalproteinHMPREF9682_002 DMQK----SLSD-----------------------------SENGLN-----IDDVGRII
RM.Lru27782ORF13200P/11620 NMQN----IMAS-----------------------------EDNELN-----TSDIGKII
gi|492676546|ref|WP_005919236.1|DNAphosphorothioationsystemrestrictionenzyme[Pediococcusacidilactici]_gi|482517924|gb|EOA08103. TLQA----TLSS------------------------------DNELK-----TDDPGKII
gi|498183116|ref|WP_010497272.1|helicase[Lactobacillusacidipiscis]/11563 NMQA----MMSS------------------------------EHELN-----TTDIGKII
gi|822100485|ref|WP_046921865.1|helicase[Lactobacillusruminis]_gi|821684442|gb|KLA43321.1|restrictionmodificationsystemLlaBIII/ NMQK----IMSS-----------------------------DENELK-----TSDIGKII
gi|935565832|ref|WP_054450311.1|helicase[Lactobacilluskunkeei]/11573 DMQQ----IMAA------------------------------DNQLK-----VDDIGKII
gi|752296887|ref|WP_041152901.1|helicase[Lactobacilluskunkeei]_gi|751663520|gb|KIM18358.1|helicase[Lactobacilluskunkeei]/11577 DMQQ----IMAA------------------------------DNQLK-----VDDIGKII
gi|495745291|ref|WP_008469870.1|helicase[Lactobacillushominis]_gi|394484442|emb|CCI81207.1|RestrictionmodificationsystemLlaBII/ DMQR----LLSA------------------------------DNDLK-----VDDIGKII

```
gi|918672414|ref|WP_052543134.1|helicase[Lactobacillussp.]/11564    DMQK----IMSA------------------------------DNQLK-----VDDIGKII
gi|799141631|ref|WP_045927261.1|helicase[Lactobacilluskullabergensis]_gi|797155335|gb|KJY59264.1|putativerestrictionendonucleas    DMQK----IMSA------------------------------DNQLK-----VDDIGKII
gi|504379990|ref|WP_014567092.1|helicase[Lactobacillusjohnsonii]_gi|329666726|gb|AEB92674.1|putativerestrictionendonuclease[Lac    DMQT----LLSS------------------------------DNQLK-----VDDIGKII
gi|489880179|ref|WP_003783646.1|DNAdamageinducibleprotein[Actinomycesnaeslundii]_gi|399901561|gb|EJN84440.1|typeIIIrestriction/    QMQS----SLAQ-------------------------G----G-DLG-----LDDAARIV
gi|496524497|ref|WP_009232780.1|DNAdamageinducibleprotein[Actinomycessp.oraltaxon849]_gi|365265413|gb|EHM95177.1|hypotheticalp/    QMQS----SLAQ-------------------------D----G-QLT-----LDDAARIV
gi|915397782|ref|WP_050783167.1|damageinducibleprotein[Actinomycesoris]/11694    QMQS----SLAQ-------------------------D----G-QLT-----LDDAARIV
gi|502925248|ref|WP_013160224.1|helicase[Propionibacteriumfreudenreichii]_gi|296921272|emb|CBL55824.1|helicase[Propionibacteriu    SMQG----AIAN-------------------------S----DGEIS-----LDDASKII
gi|732164352|emb|CEG87481.1|helicase[Propionibacteriumfreudenreichii]/11618    PLQE----SLAG-------------------------A----GAELS-----LDDMAKLV
gi|739047402|ref|WP_036925058.1|damageinducibleprotein[Propionibacteriumthoenii]/11596    GMQA----CLAG-------------------------E----GHELS-----LDDAARIV
gi|739112839|ref|WP_036982533.1|damageinducibleprotein[Propionibacteriumjensenii]/11594    GMQA----CLAG-------------------------E----GHELS-----LDDAARIV
gi|918023558|ref|WP_052314121.1|damageinducibleprotein[Nocardiathailandica]/11612    HFQK----AMAE-------------------------S----G-EIQ-----LGDASKIL
gi|498380271|ref|WP_010694427.1|DNAdamageinducibleprotein[Saccharopolysporaspinosa]/11605    NFQN----ALAQ-------------------------S----G-EIK-----LDEAARLI
gi|749991459|ref|WP_040339809.1|damageinducibleprotein[CandidatusBlastococcusmassiliensis]/11597    NFQQ----AMAN-------------------------S----G-EIQ-----LDQAAKLI
gi|754177801|ref|WP_041796231.1|damageinducibleprotein[Modestobactermarinus]/11592    NFQQ----AMAN-------------------------S----G-EIQ-----LDQAAKLI
gi|504188248|ref|WP_014375350.1|DNAdamageinducibleprotein[Blastococcussaxobsidens]_gi|378782790|emb|CCG02456.1|putativeHelicas/    NFQQ----AMAN-------------------------S----G-EIQ-----LDQAAKLI
gi|946476713|gb|KRF26798.1|damageinducibleprotein[Phycicoccussp.Soil803]/11609    SFQS----ELAQ-------------------------S----G-EIK-----LEDAAKLI
gi|700176672|gb|KGN37509.1|DNAdamageinducibleprotein[KnoelliasubterraneaKCTC19937]/11633    SFQS----ELAQ-------------------------S----G-EIK-----LEDAAKLI
gi|700168841|gb|KGN29914.1|hypotheticalproteinN802_10215[KnoelliasinensisKCTC19936]/11631    SFQQ----ELAQ-------------------------S----G-EIK-----LEDAAKLI
gi|946426241|gb|KRE76610.1|damageinducibleprotein[Arthrobactersp.Soil763]/11606    TFQA----QLAD-------------------------G----SGELQ-----LDDVAKIV
gi|517318525|ref|WP_018500927.1|hypotheticalprotein[Frankiasp.BCU110501]/11592    TFQM----QLSD-------------------------D----GNELK-----LDDAAKIV
gi|499072429|ref|WP_010851327.1|Helicase[Tetrasphaeraelongata]_gi|478756579|emb|CCH71501.1|putativeHelicase[Tetrasphaeraelongat    TFQL----QLAD-------------------------E----GNELR-----LDDAAKIV
gi|750080666|ref|WP_040385350.1|damageinducibleprotein[Demetriaterragena]/11625    QFQQ----ELAE-------------------------L----G-EIP-----LGDAARMV
gi|752558292|ref|WP_041229523.1|damageinducibleprotein[Dermacoccusnishinomiyaensis]_gi|664689315|gb|AIF41904.1|damageinducibl/1    NFQQ----ELSE-------------------------D----G-EIQ-----LGDAARMV
gi|829089213|ref|WP_047310701.1|damageinducibleprotein[Dermacoccussp.PE3]_gi|828384812|gb|KLO63770.1|damageinducibleprotein[D/1    NFQS----ELAQ-------------------------D----G-EIQ-----LGDAARMV
gi|748756635|ref|WP_040014750.1|damageinducibleprotein[Dermacoccussp.Ellin185]/11596    NFQT----ELAQ-------------------------D----G-EIQ-----LGDAARMV
gi|916534724|ref|WP_051141848.1|damageinducibleprotein[Actinomycesneuii]/11632    IYQS----V-AN-------------------------D----EGELP-----LTDMAKLV
gi|921140030|ref|WP_053090276.1|damageinducibleprotein[Rothiamucilaginosa]/11616    SFQN----LLAD-------------------------E----SNELA-----IDDVAKLV
gi|896406751|ref|WP_049343749.1|damageinducibleprotein[Rothiamucilaginosa]/11647    SFQN----LLAD-------------------------E----SNELA-----IDDVAKLV
gi|723608234|gb|AIY01610.1|putativehelicase[Arthrobactersp.PAMC25486]/11613    TFQD----QLAD-------------------------S----NSELN-----IDDIAKLV
gi|946394527|gb|KRE45160.1|damageinducibleprotein[Arthrobactersp.Soil736]/11616    SFQD----QLAD-------------------------S----NMELN-----IDDVAKLI
gi|515765304|ref|WP_017197904.1|hypotheticalprotein[Arthrobactersp.M2012083]/11648    SFQD----QLAD-------------------------S----NMELQ-----IDDVAKLI
gi|945315587|gb|KQT73127.1|damageinducibleprotein[Microbacteriumsp.Leaf436]/11612    EFQS----LMSQ-------------------------D----GYELA-----MEDTAKIV
gi|759908497|ref|WP_043594599.1|damageinducibleprotein[Frigoribacteriumsp.MEB024]_gi|758271359|gb|KIU03346.1|damageinduciblep/1    AFQE----EFAQ-------------------------N----G-ELN-----IPDAARIV
gi|945072850|gb|KQR34237.1|hypotheticalproteinASF75_00055[Curtobacteriumsp.Leaf154]/11597    AFQE----TFAA-------------------------D----G-ELN-----IPDAARIV
gi|551263152|ref|WP_022883363.1|DNAdamageinducibleprotein[Glaciibactersuperstes]/11621    GFQQ----QFAS-------------------------D----G-ELN-----IPDAARIV
gi|945452341|gb|KQV08491.1|hypotheticalproteinASC63_10170[Leifsoniasp.Root112D2]/11623    NFQQ----EFAQ-------------------------Q----G-ELS-----IPDAARIV
gi|742759483|gb|KIC69912.1|damageinducibleprotein[Kocuriarhizophila]/11610    QFQE----LLTD-------------------------E----DGQLS-----IDDVARMV
gi|737919995|ref|WP_035885304.1|damageinducibleprotein[Kocuriarhizophila]/11611    QFQE----LLTD-------------------------E----DGQLS-----IDDVARMV
gi|737997701|ref|WP_035959325.1|damageinducibleprotein[Kocuriamarina]_gi|725823884|gb|KHE75603.1|damageinducibleprotein[Kocur/1    QFQE----LLTD-------------------------E----DGQLS-----IGDVARMV
gi|664521070|ref|WP_031037650.1|damageinducibleprotein[Streptomycessp.NRRLWC3774]/11624    TLQQ----GIAG-------------------------G----SSELN-----LDDAAKII
gi|500101034|ref|WP_011777041.1|DNAdamageinducibleprotein[Arthrobacteraurescens]_gi|119951547|gb|ABM10457.1|putativeHelicase[A/    TFQQ----LLTD-------------------------D----NNELN-----LDDVAKIV
gi|916871953|ref|WP_051479009.1|damageinducibleprotein[Arthrobactersp.H5]/11588    TFQQ----LLTD-------------------------E----DNELS-----LDDVAKIV
gi|612084649|gb|EZP43369.1|TypeIIIrestrictionenzyme,ressubunit[Micrococcusluteus]/11625    SFQQ----MLA--------------------------D----TEGLD-----LDDAARIV
gi|759739708|ref|WP_043450422.1|damageinducibleprotein[Arthrobacterphenanthrenivorans]_gi|742758264|gb|KIC68716.1|damageinduc/1    SFQG----LFE--------------------------E----NGDLN-----LDDAARIV
gi|636845448|ref|WP_024366823.1|damageinducibleprotein[Arthrobactersp.TB26]/11605    SFQG----LFQ--------------------------E----NGDLS-----LDDAARIV
gi|500009025|ref|WP_011689743.1|DNAdamageinducibleprotein[Arthrobactersp.FB24]_gi|116613051|gb|ABK05760.1|typeIIIrestrictionen/    SFQG----LFQ--------------------------E----NGDLS-----LDDAARIV
gi|821249847|gb|KKZ12083.1|hypotheticalproteinTQ37_06090[CandidatusSynechococcusspongiarum15L]/11436    IKI-----RNLA-------------------------E----GKTLT-----IDEQGKMI
gi|739446902|ref|WP_037306885.1|damageinducibleprotein[Ruegeriahalocynthiae]/11592    SVQS----R-LA-------------------------E----DNELK-----LDDATKII
gi|640620081|ref|WP_025048131.1|damageinducibleprotein[Sulfitobactermediterraneus]_gi|751956650|gb|KIN77213.1|TypeIIIrestricti/    GVQG----R-IA-------------------------A----DNELQ-----LDDATKII
gi|740247634|ref|WP_038088583.1|damageinducibleprotein[Thiobacillusprosperus]_gi|684049748|gb|KFZ90853.1|putativehelicase[Thio/    VANSFNNAYKID-------------------------E----KNAVD-----IDFATRII
gi|518420321|ref|WP_019590528.1|hypotheticalprotein[Thioalkalivibriosp.ALE20]/11603    LANAYNQTYKLD-------------------------D----KKAVD-----INFATRMI
gi|500156826|ref|WP_011831496.1|DNAdamageinducibleprotein[Methylibiumpetroleiphilum]_gi|124261892|gb|ABM96881.1|adeninespecifi/    LANAYNNAYKLD-------------------------D----KRALD-----IRFATKII
gi|769978537|ref|WP_045094522.1|damageinducibleprotein[Legionellafallonii]_gi|698841736|emb|CEG55682.1|Putativeproteinwithboxh/    LTNSF-NAYKFD-------------------------E----KKAID-----VNFATKII
gi|553752541|ref|WP_023085344.1|hypotheticalprotein[Pseudomonasaeruginosa]_gi|552201952|gb|ERU62653.1|hypotheticalproteinQ088_0    LANNFNNAFRLD-------------------------E----KKAID-----INFATKII
gi|880980418|ref|WP_048704461.1|damageinducibleprotein[Thauerahumireducens]_gi|875132785|gb|KMU70944.1|damageinducibleprotein/1    LANNFNNAYRLD-------------------------E----KKAID-----INFATKII
gi|740135732|ref|WP_037982815.1|damageinducibleprotein[Thauerasp.27]/11603    LANNFNNAYRLD-------------------------E----KKAID-----INFATKII
gi|738017371|ref|WP_035978332.1|damageinducibleprotein[Burkholderiaglumae]/11380    LANNYNNAYKLD-------------------------D----KKAID-----IRFATKIV
gi|498506003|ref|WP_010806671.1|typeIIIrestrictionenzyme,ressubunit[Pandoraeasp.SD62]_gi|490314561|gb|EON12879.1|typeIIIrestri/    LANNYNNAYKLD-------------------------D----KKAID-----IRFATKIV
gi|500117748|ref|WP_011793753.1|DNAdamageinducibleprotein[Acidovoraxcitrulli]_gi|120587742|gb|ABM31182.1|typeIIIrestrictionenz/    LANNYNNAYKID-------------------------D----KKAID-----INFATKII
gi|651617328|ref|WP_026611444.1|damageinducibleprotein[Methylocaldumszegediense]/11601    LANNYNNAFKID-------------------------A----KKAID-----IKLATKII
gi|737210208|ref|WP_035194994.1|damageinducibleprotein[Acidithiobacillusferrivorans]_gi|669953220|emb|CDQ11755.1|Adeninespecif/    ELED----RIGD-------------------------S----GDSLK-----LDDAVKIV
gi|935697386|ref|WP_054506180.1|damageinducibleprotein[Achromobactersp.]_gi|928716153|emb|CUK12836.1|Hefnuclease[Achromobacter/    VLNR----RIAD-------------------------S----GDELK-----LDDAVKIV
gi|429502517|gb|ELA00827.1|adeninespecificDNAmethyltransferase[Cupriavidussp.HMR1]/11599    VLNR----RIAD-------------------------S----GDELK-----LDDAVKIV
gi|506356642|ref|WP_015876361.1|DNAdamageinducibleprotein[Burkholderiaglumae]_gi|237877158|gb|ACR29491.1|AdeninespecificDNAmet/    VLNR----RIAD-------------------------S----GDELK-----LDDAVKIV
gi|696406787|ref|WP_032978003.1|damageinducibleprotein[Bordetellahinzii]_gi|625335076|gb|KCB49343.1|typeIIIrestrictionenzyme,r/    VLNK----RIAD-------------------------S----GDELK-----LDDAVKIV
gi|817529125|gb|KKO72587.1|damageinducibleprotein[Kerstersiagyiorum]/11592    VLNK----RIAD-------------------------S----GDELK-----LDDAVKIV
gi|737048910|ref|WP_035044118.1|damageinducibleprotein[Aquabacteriumsp.NJ1]_gi|699052896|gb|KGM38706.1|damageinducibleprotein/1    VLNQ----RIGD-------------------------R----GDELK-----LDDAVKIV
```

```
gi|822492519|ref|WP_046933319.1|MULTISPECIES:damageinducibleprotein[Xanthomonas]_gi|821703525|gb|KLA61196.1|damageinduciblepr/1    VLNQ----RIAD-------------------------S----GDELK-----LDDAVKIV
gi|751252861|ref|WP_040963740.1|damageinducibleprotein[Xanthomonasfuscans]_gi|749808363|gb|KIJ03887.1|damageinducibleprotein[/1    VLNQ----RIAD-------------------------S----GDELK-----LDDAVKVV
gi|496774980|ref|WP_009368792.1|DNAdamageinducibleprotein[Bilophilasp.4_1_30]_gi|345039902|gb|EGW44205.1|hypotheticalproteinHM/    SLQS----RIGD-------------------------S----GDELN-----LDDAVKIV
gi|499241912|ref|WP_010939452.1|DNAdamageinducibleprotein[Desulfovibriovulgaris]_gi|46580580|ref|YP_011388.1|adeninespecificDN/    ALQS----RIED-------------------------N----SDELD-----LDDAVKIV
gi|504274913|ref|WP_014462015.1|DNAdamageinducibleprotein[Bacillusmegaterium]_gi|345446919|gb|AEN91936.1|AdeninespecificDNAmet/    AFQT----ELAN-------------------------E----NNELT-----LNDVVKIS
gi|504895966|ref|WP_015083068.1|typeIIrestrictionenzymeandmethyltransferasesubtypeG[Anabaenasp.90]_gi|413972424|gb|AFW96512.1|t    TFQQ----QISD-------------------------G----KYVLN-----LDDKAKII
gi|518327765|ref|WP_019497972.1|hypotheticalprotein[Calothrixsp.PCC7103]/11600    AFQK----QLAD-------------------------A----NNELN-----LEDAVKIT
gi|652765503|ref|WP_027079917.1|damageinducibleprotein[Luteimonasmephitis]/11593    TFQS----QLAD-------------------------A----NNELT-----LDDAVKIV
gi|896539951|ref|WP_049442123.1|damageinducibleprotein[Stenotrophomonasmaltophilia]/11591    TFQN----QLAD-------------------------K----NKELN-----LDDVVKIV
gi|500217900|ref|WP_011888043.1|DNAdamageinducibleprotein[Geobacillusthermodenitrificans]_gi|134267954|gb|ABO68149.1|Conserved/    RLGY----RIHD-------------------------E----KDQVK-----LDDVAKIV
gi|696570298|ref|WP_033102014.1|damageinducibleprotein[Thermoactinomycesdaqus]/11594    HLGN----KIHA-------------------------N----EENIT-----LDDAAKIV
gi|251772067|gb|EES52637.1|putativehelicase[Leptospirillumferrodiazotrophum]/11618    RFPY----LTDV-------------------------S----GKEIP-----LPDAAKII
gi|738124101|ref|WP_036082108.1|damageinducibleprotein[Leptospirillumferriphilum]_gi|685243306|gb|KGA94177.1|putativehelicase[/    RFPY----LTDV-------------------------S----GKEIP-----LPDAAKII
gi|558699223|ref|WP_023524766.1|helicase[Leptospirillumsp.GroupII'CF1']_gi|206603764|gb|EDZ40244.1|Putativehelicase[Leptospiri/    RFPY----LTDI-------------------------S----GKEIP-----LPDAAKII
gi|406774462|gb|AFS53887.1|putativehelicase[LeptospirillumferriphilumML04]/11610    RFPY----LLSQ-------------------------E----GEELK-----LPDVAKII
gi|740719463|ref|WP_038504749.1|damageinducibleprotein[Leptospirillumferriphilum]_gi|640838125|gb|AIA30173.1|damageinduciblep/1    RFPF----LLGH-------------------------E----GEELR-----LPDVAKII
gi|517810766|ref|WP_018980974.1|hypotheticalprotein[Saccharibacterfloricola]/11733    ILAN----SSHEDDRQAAEANMEEVPQKTGRKRGGKLKDEAEQARVN-----LDDASRII
gi|502471675|ref|WP_012798533.1|helicase[Slackiaheliotrinireducens]_gi|256791761|gb|ACV22431.1|predictedhelicase[Slackiaheliotr    VYQM----AM-A-------------------------G----DTGFE-----IPDAAKII
gi|657871713|ref|WP_029576297.1|hypotheticalprotein[Bifidobacteriumthermacidophilum]_gi|673007521|gb|KFJ02535.1|helicase[Bifido    PFQ-----SITD-------------------------A----GMKLQ-----IEDTAKFI
gi|673004757|gb|KFI99791.1|DNAhelicaserestrictionenzymetypeIIIRessubunit[Bifidobacteriumthermacidophilumsubsp.porcinum]/11682    PFQ-----SITD-------------------------A----GMKLQ-----IEDTAKFI
gi|917316092|ref|WP_051922804.1|hypotheticalprotein[Bifidobacteriumstellenboschense]_gi|673003774|gb|KFI98812.1|helicase[Bifido    VMQK----QYGA-------------------------K----DSEIP-----LDDAAKFV
gi|705415694|ref|WP_033502332.1|hypotheticalprotein[Bifidobacteriumpseudolongum]_gi|672982411|gb|KFI77685.1|DNAdamageinducible/    AMQE----AYSR-------------------------E----NAPIP-----LSDAAKFV
gi|916591418|ref|WP_051198509.1|hypotheticalprotein[Bifidobacteriumsp.AGR2158]/11704    MMQE----RYSE-------------------------A----KSQIP-----LSDAAKFV
gi|772242982|gb|AJW78255.1|hypotheticalproteinVO01_03165[Clavibactermichiganensissubsp.insidiosus]/11642    GFQR----ELAD-------------------------D----GHELQ-----IGDVAKLI
gi|916649818|ref|WP_051256909.1|damageinducibleprotein[Actinomycesgraevenitzii]/11677    KYQL-----IAE-------------------------M----DGELN-----LDTAAKLT
gi|496504578|ref|WP_009212861.1|helicase[Actinomycessp.oraltaxon180]_gi|315312695|gb|EFU60776.1|helicase[Actinomycessp.oraltaxo    QYQA-----IAE-------------------------M----GGELN-----LDTAAKLT
gi|494862206|ref|WP_007588306.1|helicase[Actinomycessp.ICM39]_gi|398362799|gb|EJN46470.1|typeIIIrestrictionenzyme,ressubunit[Ac    HYQA-----IAE-------------------------M----GGELN-----LDTAAKLT
gi|881057111|ref|WP_048774974.1|damageinducibleprotein[Sanguibacterkeddieii]/11697    HYQA-----IAE-------------------------M----GGELN-----LDTAAKLT
gi|881020459|ref|WP_048740808.1|damageinducibleprotein[Kytococcussedentarius]/11704    HYQA-----IAE-------------------------M----GGELN-----LDTAAKLT
gi|881052313|ref|WP_048770555.1|damageinducibleprotein[Cellulomonasflavigena]/11705    HYQA-----IAE-------------------------M----GGELN-----LDTAAKLT
gi|490108682|ref|WP_004009424.1|helicase[Mobiluncuscurtisii]_gi|315490465|gb|EFU80089.1|helicaseCterminaldomainprotein[Mobilun/    IYQQ-----SA--------------------------V----DGELT-----IPETAKLV
gi|490111935|ref|WP_004012603.1|helicase[Mobiluncusmulieris]_gi|227843812|gb|EEJ53982.1|helicaseCterminaldomainprotein[Mobilun/    IFQQ-----GD--------------------------E----YGELS-----IPEAAKLA
gi|490114228|ref|WP_004014848.1|helicase[Mobiluncusmulieris]_gi|269934432|gb|EEZ90993.1|helicaseCterminaldomainprotein[Mobilun/    IFQQ-----GD--------------------------E----YGELS-----IPEAAKLA
gi|493333551|ref|WP_006290636.1|helicase[Parascardoviadenticolens]_gi|315119487|gb|EFT82620.1|helicaseCterminaldomainprotein[P/    SIQQ----IISN-------------------------G-----GELP-----LDDAAKII
gi|495817067|ref|WP_008541646.1|helicase,partial[Sutterellaparvirubra]_gi|378598583|gb|EHY31712.1|helicaseprotein,partial[Sutte    ---Q----GMDD-------------------------YTLVEAGGLT-----VKHAAKVI
gi|491572696|ref|WP_005430278.1|helicase[Sutterellawadsworthensis]_gi|319805116|gb|EFW01943.1|DNAhelicaserestrictionenzymeTypeI    ---R----ALSD-------------------------YELVQAGGLP-----VKHAAKVI
gi|748686321|ref|WP_039944445.1|hypotheticalprotein[Thermicanusaegyptius]/11573    ELFE----YLNQ-------------------------P-----DTPE-----VVDATRII
gi|916875698|ref|WP_051482754.1|hypotheticalprotein[Thioalkalivibriosp.HK1]/11366    TLQE----HLAD-------------------------E----GGDIK-----MSDAARIV
gi|821248604|gb|KKZ10960.1|hypotheticalproteinTE42_09075[CandidatusSynechococcusspongiarumSP3]/11598    ---------IRL-------------------------RNVAEGKTLT-----VDEQGKMI
gi|737409843|ref|WP_035390956.1|hypotheticalprotein[Ferrimicrobiumacidiphilum]_gi|765544266|gb|KJE76241.1|typeIrestrictionenzym    TLHS----VLTD-------------------------S----DSEIK-----LDDAAKLL
gi|654906925|ref|WP_028358281.1|damageinducibleprotein[Brackiellaoedipodis]/11679    HLQQ----LLSD-------------------------E----NNQLV-----IDDAAKIV
gi|502809901|ref|WP_013044877.1|hypotheticalprotein[CandidatusPuniceispirillummarinum]_gi|292663146|gb|ADE38247.1|hypotheticalp    TMQD----AISD-------------------------K-----NELS-----VDDAAKIV
gi|251771370|gb|EES51950.1|typeIIIrestrictionenzyme,ressubunit[Leptospirillumferrodiazotrophum]/11628    --------LANA-------------------------YSLESGKAIQ-----ARFAAKIV
gi|498328480|ref|WP_010642636.1|DNAdamageinducibleprotein[Acidithiobacillusthiooxidans]/11627    IANE----YNNI-------------------------YKLEEKKGIT-----VEFATRII
gi|647633864|ref|WP_025906698.1|damageinducibleprotein[Prochlorococcussp.scB245a_519B7]/11622    SIQN----QFTD-------------------------G-----SELQ-----LDDATKMI
gi|647619599|ref|WP_025893729.1|damageinducibleprotein[Prochlorococcussp.scB241_527N11]/11621    RIQN----SFSE-------------------------G-----SELE-----LDDATKMI
gi|755034887|ref|WP_042387498.1|hypotheticalprotein[Clostridiumsp.CL6]/11606    ELQT----LLMD-------------------------K----NHELK-----MNDSVKIM
gi|498139798|ref|WP_010453954.1|helicase[SuccinivibrionaceaebacteriumWG1]/11662    NIRE----QVED-------------------------S---SEKSIN-----TDDTLKLI
gi|527501386|gb|EPT34571.1|typeIIIrestrictionenzyme,ressubunit[Bacteroidetesbacteriumoraltaxon272str.F0290]/11789    DMKT----KIK------------------------------GNDELT-----LDDASKLV
gi|496047275|ref|WP_008771782.1|helicase[Bacteroidessp.2_1_33B]_gi|262296443|gb|EEY84373.1|helicaseCterminaldomainprotein[Bact/    SILE----DVKD-------------------------K---QQKEIK-----MDDASKLI
PDI8503III/11664    SILE----DVKD-------------------------K---QQKEIK-----MDDASKLI
gi|738719431|ref|WP_036613715.1|helicase[Parabacteroidesdistasonis]_gi|649542518|gb|KDS49387.1|restrictionendonucleasefamilypro    SILE----DVKD-------------------------K---QQKEIK-----MDDASKLI
gi|659417033|gb|KEJ86133.1|hypotheticalproteinHMPREF1002_00341[Porphyromonassp.31_2]/11656    SILE----DVKD-------------------------K---QQKEIK-----MDDASKLI
gi|567456807|gb|ETK11358.1|hypotheticalproteinT235_15845[Tannerellasp.oraltaxonBU063isolateCell8/11]/11433    ELMK----QVKD-------------------------T---DAKELN-----YDDTGRLI
gi|545440279|ref|WP_021677877.1|helicase[Porphyromonasgingivalis]_gi|543985765|gb|ERJ89108.1|helicaseprotein[Porphyromonasgingi    ELMK----QVKD-------------------------K---NAKELN-----YDDTGRLI
gi|497438056|ref|WP_009752254.1|helicase[Prevotellasp.oraltaxon473]_gi|429144866|gb|EKX87968.1|helicaseprotein[Prevotellasp.ora    TLMQ----QVKD-------------------------L---QAKELN-----YDDTGRLI
gi|490423474|ref|WP_004295683.1|MULTISPECIES:helicase[Bacteroides]_gi|156110098|gb|EDO11843.1|helicaseCterminaldomainprotein[B/    NILS----DIKD-------------------------P---NNVELN-----FDDTSKLI
gi|941777229|ref|WP_055173506.1|helicase[Bacteroidescaccae]_gi|933263395|emb|CUQ00567.1|Predictedhelicase[Bacteroidescaccae]/1/    NIKQ----DITN-------------------------S----TTELN-----FDDTSKLI
gi|492413898|ref|WP_005836892.1|helicase[Bacteroidesuniformis]_gi|392678824|gb|EIY72229.1|hypotheticalproteinHMPREF1072_03583[B    NIKQ----DITN-------------------------S----TTELN-----FDDTSKLI
gi|695455095|ref|WP_032566191.1|helicase[Bacteroidesfragilis]_gi|596095426|gb|EXZ59996.1|restrictionendonucleasefamilyprotein[B    NIKQ----DITN-------------------------S----TTELN-----FDDTSKLI
gi|933227941|emb|CUQ02348.1|Predictedhelicase[Bacteroidesuniformis]/11658    NIKQ----DITN-------------------------S----TTELN-----FDDTSKLI
gi|494823013|ref|WP_007558421.1|helicase[Bacteroidesplebeius]_gi|198272167|gb|EDY96436.1|helicaseCterminaldomainprotein[Bacter/    NIKQ----DITN-------------------------S----TTELN-----FDDTSKLI
gi|495427212|ref|WP_008151909.1|helicase[Parabacteroidesjohnsonii]_gi|218222381|gb|EEC95031.1|helicaseCterminaldomainprotein[P/    NIRR----DVTD-------------------------T----TTELN-----FDDTSKLI
gi|933196855|emb|CUO94545.1|typeIrestrictionenzymeEcoKIsubunitR[Bacteroidesuniformis]_gi|933220098|emb|CUN86874.1|typeIrestrict    NIKR----DITD-------------------------T----TTELN-----FDDTSKLI
gi|823687748|ref|WP_047101121.1|helicase[Brachyspirahyodysenteriae]_gi|823334537|gb|KLI17932.1|helicase[Brachyspirahyodysenteri    ALQT----MLKN-------------------------K----NEVID-----TDDISKLI
gi|496436691|ref|WP_009145538.1|helicase[Phascolarctobacteriumsuccinatutens]_gi|322414007|gb|EFY04839.1|helicaseCterminaldomai/    DLLQ----AVQD-------------------------K----NQEIN-----ADDAVKLV
gi|503372880|ref|WP_013607541.1|helicase[Sphaerochaetaglobosa]_gi|324026933|gb|ADY13692.1|typeIIIrestrictionproteinressubunit[S    ELQQ----SI-----------------------------ANEDSEIP-----TDDALKLI
```

```
gi|505821748|ref|WP_015707145.1|helicase[Treponemaprimitia]_gi|299483503|gb|ADJ19584.1|helicasedomaincontainingprotein[Trepone/      KLKA----AIEK-------------------------HNSNENKEIE-----VEDALKII
gi|505822741|ref|WP_015708134.1|helicase[Treponemaprimitia]_gi|333739639|gb|AEF85129.1|endonucleaseandmethylaseLlaGI[Treponemap     KLKA----SIEK-------------------------HNTNENKEIE-----VEEALKII
gi|488791994|ref|WP_002704400.1|helicase[Treponemasaccharophilum]_gi|380767691|gb|EIC01689.1|typeIIIrestrictionproteinressubuni     KIQK----IILD-------------------------QKTEDNKEIK-----AVDAIKLI
gi|524671454|emb|CDD81750.1|putativeseptumsitedeterminingproteinMinC[Bacteroidessp.CAG:462]/11642                                  ALRD----VITN-------------------------E----DGTIT-----VDDASKFV
gi|524573865|emb|CDC95766.1|putativeseptumsitedeterminingproteinMinC[Alistipessp.CAG:268]/11639                                    TLQN----ALTR-------------------------E----DGTID-----ADDPSKLI
gi|658617970|ref|WP_029759328.1|helicase[Fusobacteriumnucleatum]/11606                                                             ELQA----IIAN-------------------------G----ESEFK-----VDDATKLI
gi|494412037|ref|WP_007216782.1|helicase[Bacteroidescellulosilyticus]_gi|392638921|gb|EIY32752.1|hypotheticalproteinHMPREF1062_     ALQA----ALAN-------------------------H----ELEIK-----TDDAAKLI
gi|383107102|gb|AFG37435.1|putativehelicase[SpirochaetaafricanaDSM8902]/11616                                                      PVQN----MVAD-------------------------K----DKEIN-----IEDAAKLI
SAF8902III/11616                                                                                                                   PVQN----MVAD-------------------------K----DKEIN-----IEDAAKLI
gi|652538894|ref|WP_026932796.1|helicase[Gramellaechinicola]/11633                                                                 AIQA----MVAN-------------------------S----DQEIN-----SDDASKLI
gi|639238223|ref|WP_024568839.1|helicase[Elizabethkingiaanophelis]/11628                                                           SVQS----MIAG-------------------------S----DNEIS-----SDDASKLI
gi|652520381|ref|WP_026914582.1|helicase[Gramellaportivictoriae]/11623                                                             SVQK----MVSN-------------------------P----SNEIT-----SDDASKLI
gi|494922183|ref|WP_007648219.1|Helicase[Formosasp.AK20]_gi|469825489|gb|EMQ95550.1|Helicasedomainprotein[Formosasp.AK20]/11626    AVQK----MVAN-------------------------S----ENEIN-----SDDASKLI
gi|522083382|ref|WP_020594591.1|hypotheticalprotein[Spirosomapanaciterrae]/11637                                                   AVQK----MIAD-------------------------K----ESEIN-----TDDASKLI
gi|490455352|ref|WP_004326157.1|helicase[Bacteroidesovatus]_gi|392669277|gb|EIY62768.1|hypotheticalproteinHMPREF1070_03635[Bact    AVQK----MVAN-------------------------P----ENEIS-----SDDVSKLI
gi|499516035|ref|WP_011202675.1|helicase[Bacteroidesfragilis]_gi|53713270|ref|YP_099262.1|helicasedomainprotein[Bacteroidesfrag    AVQK----MVAN-------------------------P----ENEIS-----SDDVSKLI
gi|651338332|ref|WP_026461320.1|helicase[Adhaeribacteraquaticus]/11620                                                            AVQK----MIAG-------------------------E----ESEIN-----TDDASKLI
gi|806964642|gb|AKC17139.1|helicase[Flavobacteriumpsychrophilum]/11639                                                            DIQK----MIAD-------------------------D----DHEID-----LDDASKLI
gi|752684374|ref|WP_041332322.1|helicase[Flavobacteriumpsychrophilum]_gi|666882889|gb|AIG30715.1|helicase[Flavobacteriumpsychro    DIQK----MIAD-------------------------D----DHEID-----LDDASKLI
gi|933196595|emb|CUP04894.1|DNAorRNAhelicasesofsuperfamilyII[Bacteroidesuniformis]/11631                                          AVQK----MIAD-------------------------D----TNEIN-----ADDASKLI
gi|670509109|ref|WP_031456918.1|helicase[Flavobacteriumchungangense]/11625                                                         AIQK----MIAD-------------------------E----NSEIS-----TDDASKLI
                                                                                                                                                                                       .

gi|490190857|ref|WP_004089451.1|hypotheticalprotein[Helicobacterbilis]_gi|476630638|gb|EMZ37196.1|hypotheticalproteinC826_02193    GTHKGLVKSDLMTL---------------------------DSS--IQEDTEYKTQSDNI
gi|488896133|ref|WP_002807242.1|MULTISPECIES:restrictionendonucleaseEco57I[Campylobacter]_gi|380541096|gb|EIA65377.1|hypothetic    GTHKGLARNDLVAL---------------------------DEN--NQIDNDFLEEMDSN
gi|746602240|ref|WP_039628365.1|restrictionendonucleaseEco57I[Campylobacterlari]_gi|744819395|gb|AJD03391.1|putativehelicase[Ca    GTHKGLARNDLVAL---------------------------DEN--NQIDNDFLEEMDSN
gi|727317135|ref|WP_033772558.1|restrictionendonucleaseEco57I[Helicobacterpylori]/11622                                            GTHKGLAKQDLIAL---------------------------DDE--NKEDNDLQDKTDTI
gi|727103490|ref|WP_033618954.1|restrictionendonucleaseEco57I[Helicobacterpylori]/11616                                            GTHKGLAKQDVIAL---------------------------DDE--NQEDNDLQDKTDTI
gi|727340205|ref|WP_033794408.1|restrictionendonucleaseEco57I[Helicobacterpylori]/11609                                            GTHKGLAKQDLIAL---------------------------DDE--NKEDNDLQDKTDTI
gi|727292407|ref|WP_033749975.1|restrictionendonucleaseEco57I[Helicobacterpylori]/11611                                            GTHKGLAKQDLIAL---------------------------DDE--NKEDNDLQDKTDTI
gi|727289795|ref|WP_033747591.1|restrictionendonucleaseEco57I[Helicobacterpylori]/11612                                            GTHKGLAKQDVIAL---------------------------DDE--NKEDNDLQDKTDTF
gi|727319676|ref|WP_033775047.1|restrictionendonucleaseEco57I[Helicobacterpylori]/11612                                            GTHKGLAKQDLIAL---------------------------DDE--NKEDNDLQDKTDTI
gi|447212910|ref|WP_001290166.1|restrictionendonucleaseEco57I[Helicobacterpylori]_gi|308063540|gb|ADO05427.1|hypotheticalprotei    GTHKGLAKQDLIAL---------------------------DNE--NKEDYDLQNKNDTT
gi|447212901|ref|WP_001290157.1|restrictionendonucleaseEco57I[Helicobacterpylori]_gi|384557329|gb|AFH97797.1|hypotheticalprotei    GTHKGLAKQDLIAL---------------------------DDE--NKKDYDLQNKNDTT
gi|515849671|ref|WP_017280424.1|hypotheticalprotein[Helicobacterpylori]/11615                                                      GTHKGLAKQDLIAL---------------------------DDE--NKKDHNLQNKNDTT
gi|447212900|ref|WP_001290156.1|restrictionendonucleaseEco57I[Helicobacterpylori]_gi|317178970|dbj|BAJ56758.1|TypeIIGrestrictio    GTHKGLAKQDLIAL---------------------------DDE--NKKDHNLQNKNDTT
gi|447079018|ref|WP_001156274.1|hypotheticalprotein[Helicobacterpylori]/11614                                                      GTHKGLAKQDIIAL---------------------------DDE--NKEDNDLQNKKDAV
gi|447212800|ref|WP_001290056.1|hypotheticalprotein[Helicobacterpylori]_gi|532153248|gb|EQL67316.1|restrictionendonucleaseEco57    GTHKGLAKQDIIAL---------------------------NNE--NQEDNDLQNKKDAV
gi|505457237|ref|WP_015644339.1|hypotheticalprotein[Helicobacterpylori]_gi|499061160|gb|AGL70074.1|restrictionendonucleaseEco57    GTHKGLAKQDLIVL---------------------------DNE--NQGDHNLQNKYDTV
HPYUM037X/11591                                                                                                                    GTHKGLAKQDLIVL---------------------------DNE--NQGDHNLQNKYDTV
gi|920026297|ref|WP_052937176.1|restrictionendonuclease[Helicobacterpylori]_gi|912528678|gb|KNX48238.1|restrictionendonucleaseE    GTHKGLAKQDLIVL---------------------------DNE--NKEDHNLQNKYDTA
gi|915539050|ref|WP_050841327.1|restrictionendonuclease[Helicobacterpylori]_gi|908890887|gb|KNE06183.1|restrictionendonucleaseE    GTHKGLAKQDLIAL---------------------------DDE--NKEDNDLQNKYDTA
gi|727101397|ref|WP_033616957.1|restrictionendonucleaseEco57I[Helicobacterpylori]/11592                                            GTHKGLAKQDLIVL---------------------------DNE--NKEDHNLQNKYDTA
gi|562814875|emb|CDK13002.1|TypeISPRMsystem[Helicobacterpylori26695]/11588                                                        GTHKGLAKQDLIVL---------------------------NEK--NKEDHNLQNQYDTA
HPYAXVIII/11588                                                                                                                    GTHKGLAKQDLIVL---------------------------NEK--NKEDHNLQNQYDTA
gi|447079029|ref|WP_001156285.1|restrictionendonucleaseEco57I[Helicobacterpylori]_gi|393085956|gb|EJB86635.1|hypotheticalprotei    GTHKGLAKQDLIVL---------------------------DDE--NKEDNDLQNKADTF
gi|489105953|ref|WP_003015814.1|restrictionendonuclease[Helicobacterpylori]_gi|477503388|gb|ENH59950.1|restrictionendonucleasef    GTHKGLAKQDLIVL---------------------------DEE--NKEDNDLKDKKDTA
gi|447079006|ref|WP_001156262.1|restrictionendonucleaseEco57I[Helicobacterpylori]_gi|393067470|gb|EJB68280.1|hypotheticalprotei    GTHKGLAKQDLIVL---------------------------DEK--NKEDHNLQHQYDTT
gi|727314589|ref|WP_033770298.1|restrictionendonucleaseEco57I[Helicobacterpylori]/11586                                            GTHKGLAKQDLIVL---------------------------DDE--NKEDHNLQNKDDTA
gi|447079019|ref|WP_001156275.1|restrictionendonucleaseEco57I[Helicobacterpylori]_gi|308065028|gb|ADO06920.1|hypotheticalprotei    GTHKGLAKQDLIVL---------------------------DDE--NREDNDLKNKRDTF
gi|447079028|ref|WP_001156284.1|restrictionendonucleaseEco57I[Helicobacterpylori]_gi|393076030|gb|EJB76784.1|hypotheticalprotei    GTHKGLAKQDLIVL---------------------------DDE--NKEDHNLQNKADTF
gi|393034009|gb|EJB35070.1|hypotheticalproteinHPNQ4053_0631[HelicobacterpyloriNQ4053]/11514                                       GTHKGLAKQDLIVL---------------------------DDE--NKEDNDLKNKADTF
gi|447079004|ref|WP_001156260.1|restrictionendonucleaseEco57I[Helicobacterpylori]_gi|384552418|gb|AFI07366.1|hypotheticalprotei    GTHKGLAKQDLIAL---------------------------NNE--NKEDHNLQNQYDTA
gi|746011306|ref|WP_039088215.1|restrictionendonucleaseEco57I[Helicobacterpylori]_gi|733488720|gb|KHL85979.1|restrictionendonuc    GTHKGLAKQDLIVL---------------------------DDE--NKEDHNLQNKADTF
gi|531081162|gb|EQD92166.1|restrictionendonucleaseEco57I[HelicobacterpyloriPZ5080]/11509                                          GTHKGLAKQDLIAL---------------------------DDE--NKEDNDLKDKKDTF
gi|544760796|ref|WP_021187395.1|restrictionendonucleaseEco57I[Helicobacterpylori]_gi|531086508|gb|EQD96745.1|restrictionendonuc    GTHKGLAKQDLIVL---------------------------DEK--NKEDNDLKNKADTF
gi|447079012|ref|WP_001156268.1|restrictionendonucleaseEco57I[Helicobacterpylori]_gi|393028199|gb|EJB29286.1|hypotheticalprotei    GTHKGLAKQDLIAL---------------------------DDE--NKEDNDLKNKADTF
gi|746008458|ref|WP_039085555.1|restrictionendonucleaseEco57I[Helicobacterpylori]_gi|733480358|gb|KHL78026.1|restrictionendonuc    GTHKGLAKQDVIAL---------------------------DDE--NKEDNDLKNKADTF
gi|447079011|ref|WP_001156267.1|restrictionendonucleaseEco57I[Helicobacterpylori]_gi|393038534|gb|EJB39568.1|hypotheticalprotei    GTHKGLAKQDVIAL---------------------------DDE--NKEDNDLKNKADTF
gi|393037163|gb|EJB38199.1|hypotheticalproteinHPNQ4161_0422[HelicobacterpyloriNQ4161]/11556                                       GTHKGLAKQDVIAL---------------------------DDE--NQEDNDLKNKADTF
gi|447079007|ref|WP_001156263.1|restrictionendonucleaseEco57I[Helicobacterpylori]_gi|393051817|gb|EJB52768.1|hypotheticalprotei    GTHKGLAKQDLIVL---------------------------DDE--NKEDHNLQNQYDTA
gi|487867094|ref|WP_001940560.1|helicase[Helicobacterpylori]_gi|459505657|gb|EMG98418.1|helicaseprotein[HelicobacterpyloriGAM21    GTHKGLAKQDVIAL---------------------------DDE--NKEDNDLKNKADTF
gi|487872094|ref|WP_001945560.1|helicase[Helicobacterpylori]_gi|459512104|gb|EMH04523.1|helicaseprotein[HelicobacterpyloriGAM24    GTHKGLAKQDLIAL---------------------------DDE--NKEDNDLKNKADTF
gi|487880228|ref|WP_001953694.1|helicase[Helicobacterpylori]_gi|459529131|gb|EMH20734.1|helicaseprotein[HelicobacterpyloriGAM26    GTHKGLAKQDVIAL---------------------------DDE--NKEDNDLKNKADTF
gi|487882593|ref|WP_001956059.1|helicase[Helicobacterpylori]_gi|459533462|gb|EMH24859.1|helicaseprotein[HelicobacterpyloriGAM26    GTHKGLAKQDLIAL---------------------------NDE--NKEDNDLKNKADTF
gi|487845430|ref|WP_001918896.1|helicase[Helicobacterpylori]_gi|410713871|gb|EKQ71363.1|helicaseprotein[HelicobacterpyloriGAM10    GTHKGLAKQDLIAL---------------------------DDE--NKEDNDLKNKADTF
gi|447079005|ref|WP_001156261.1|restrictionendonucleaseEco57I[Helicobacterpylori]_gi|393069830|gb|EJB70625.1|hypotheticalprotei    GTHKGLAKQDLIAL---------------------------DDE--NKEDNDLKNKADTF
```

gi|459506808|gb|EMG99528.1|helicaseprotein[HelicobacterpyloriGAM121Aii]/11545	GTHKGLAKQDLIAL---------------------------DDE--NKEDNDLKNKADTF
gi|487825283|ref|WP_001898749.1|restrictionendonucleaseEco57I[Helicobacterpylori]_gi|393107526|gb|EJC08068.1|hypotheticalprotei	GTHKGLAKQDVIAL---------------------------DDE--NQEDNDLKNKADTF
gi|447079008|ref|WP_001156264.1|restrictionendonucleaseEco57I[Helicobacterpylori]_gi|393058636|gb|EJB59524.1|hypotheticalprotei	GTHKGLAKQDVIAL---------------------------DDE--NQEDNDLKNKADTF
gi|447079015|ref|WP_001156271.1|restrictionendonucleaseEco57I[Helicobacterpylori]_gi|307637357|gb|ADN79807.1|hypotheticalprotei	GTHKGLAKQDVIAL---------------------------DDE--NKEDNDLKNKADTF
gi|487884861|ref|WP_001958327.1|helicase[Helicobacterpylori]_gi|459535535|gb|EMH26834.1|helicaseprotein[HelicobacterpyloriGAM26	GTHKGLAKQDLIVL---------------------------DDE--NKEDNDLKNKADTF
gi|487886365|ref|WP_001959831.1|helicase[Helicobacterpylori]_gi|459538727|gb|EMH29861.1|helicaseprotein[HelicobacterpyloriGAM27	GTHKGLAKQDVIAL---------------------------DDE--NKEDNDLKNKADTF
gi|545301637|ref|WP_021581412.1|helicase[Helicobacterpylori]_gi|535931889|gb|ERA61745.1|helicaseprotein[HelicobacterpyloriGAM11	GTHKGLAKQDVIAL---------------------------DDE--NKEDNDLKNKTDTF
gi|447079010|ref|WP_001156266.1|restrictionendonucleaseEco57I[Helicobacterpylori]_gi|393095477|gb|EJB96081.1|hypotheticalprotei	GTHKGLAKQDVIAL---------------------------DDE--NQEDNDLKNKADTF
gi|447079009|ref|WP_001156265.1|restrictionendonucleaseEco57I[Helicobacterpylori]_gi|393056294|gb|EJB57206.1|hypotheticalprotei	GTHKGLAKQDVIAL---------------------------NDE--NQEDNDLKNKADTF
gi|471386985|gb|EMR58906.1|restrictionendonucleasefamilyprotein[HelicobacterpyloriHpH1]/11504	GTHKGLAKQDVIAL---------------------------DDE--NKEDSDLKNKADTF
gi|487875679|ref|WP_001949145.1|helicase[Helicobacterpylori]_gi|459516928|gb|EMH09091.1|helicaseprotein[HelicobacterpyloriGAM25	GTHKGLAKQDVIAL---------------------------DDE--NKEDNDLKNKADTF
gi|496130066|ref|WP_008854573.1|DNAdamageinducibleprotein[Commensalibacterintestini]_gi|353671689|gb|EHD13391.1|helicasedomain/	GCWKALSKQGLFEK---------------------------------------DH-NLAN
gi|924898894|ref|WP_053556546.1|damageinducibleprotein[Sphingopyxissp.113P3]_gi|924523996|gb|ALC14732.1|damageinducibleprotei/1	GCYKALTKVDFAGE---------------------------------------A--GHPR
gi|780802950|gb|KJS08562.1|damageinducibleprotein[Hoefleasp.BRH_c9]/11633	GCYKALTKADLKQD---------------------------------------VT-ADPL
gi|944671432|gb|KQN22886.1|damageinducibleprotein[Sphingomonassp.Leaf32]/11415	GCYKALTKADFGGD---------------------------------------VA-NDTS
gi|944612953|gb|KQM64772.1|damageinducibleprotein[Sphingomonassp.Leaf16]/11726	GCYKALTKADFGGD---------------------------------------VA-NDTS
gi|764353851|ref|WP_044331468.1|damageinducibleprotein[Sphingomonashengshuiensis]_gi|761897338|gb|AJP71707.1|damageinduciblep/1	GCYKALTKHGLKDE---------------------------------------LL-TDPQ
gi|945874472|gb|KQZ27973.1|damageinducibleprotein[Mesorhizobiumsp.Root552]/11651	GCYKALTKHGLKNE---------------------------------------LL-TDPQ
gi|759408081|ref|WP_043132403.1|damageinducibleprotein[Paracoccussanguinis]_gi|694217086|gb|KGJ14281.1|damageinducibleprotein/1	GCYKALTKADLVED---------------------------------------LA-FDPR
gi|654342671|ref|WP_027836116.1|damageinducibleprotein[Maritaleamyrionectae]/11632	GCYKALTKSDIKAD---------------------------------------LG-FDPS
gi|126717687|gb|EBA14409.1|hypotheticalproteinRSK20926_02032[Roseobactersp.SK20926]/11603	GCYKALTKTDLAAD---------------------------------------LE-FDPR
gi|765301116|ref|WP_044628780.1|damageinducibleprotein[Halomonasmeridiana]_gi|764089679|gb|KJD19875.1|damageinducibleprotein[/1	GCYKALTKSDIRSD---------------------------------------LE-FDPQ
gi|518053006|ref|WP_019223214.1|DNAdamageinducibleprotein[Bartonellarattaustraliani]/11654	GCYRALTKIDKEAT---------------------------------------VDDDDTE
gi|493637806|ref|WP_006589578.1|DNAdamageinducibleprotein[Bartonellabirtlesii]_gi|395410345|gb|EJF76900.1|hypotheticalproteinM/	GCYRALTKIDKEAV---------------------------------------VDNDDTE
gi|501188603|ref|WP_012231622.1|DNAdamageinducibleprotein[Bartonellatribocorum]_gi|161017843|emb|CAK01401.1|helicase/methyltra/	GCYRALTKIDKQAA---------------------------------------IDDDDTE
gi|651388616|ref|WP_026500615.1|damageinducibleprotein[Bartonellagrahamii]/11655	GCYRALTKIDKQAA---------------------------------------VDDDDTE
gi|501188669|ref|WP_012231687.1|DNAdamageinducibleprotein[Bartonellatribocorum]_gi|161017922|emb|CAK01480.1|helicase/methyltra/	GCYRALTKIDKQAA---------------------------------------IDDDDTE
gi|518048832|ref|WP_019219040.1|DNAdamageinducibleprotein[Bartonellaflorencae]/11655	GCYKALAQIEKKE--DDGIK
gi|518053617|ref|WP_019223825.1|DNAdamageinducibleprotein[Bartonellarattaustraliani]/11607	GCYKALSQIEKKDD--EDNDIK
gi|506336449|ref|WP_015856168.1|DNAdamageinducibleprotein[Bartonellagrahamii]_gi|240267492|gb|ACS51080.1|helicase/methyltransf/	GCYKALAQIEKKE--DDGIK
gi|494588832|ref|WP_007347427.1|DNAdamageinducibleprotein[Bartonellarattimassiliensis]_gi|395419053|gb|EJF85361.1|hypothetical/	GCYKALSQIEKKE--EDGIK
gi|501188650|ref|WP_012231668.1|DNAdamageinducibleprotein[Bartonellatribocorum]_gi|161017902|emb|CAK01460.1|helicase/methyltra/	GCYKALSQIEKKE--EDGIK
gi|501188624|ref|WP_012231643.1|DNAdamageinducibleprotein[Bartonellatribocorum]_gi|161017866|emb|CAK01424.1|helicase/methyltra/	GCYQALAKLDLKTD---------------------------------------LG-DDTA
gi|518053856|ref|WP_019224064.1|DNAdamageinducibleprotein[Bartonellarattaustraliani]/11625	GCYQALTKLDLKTD---------------------------------------LG-DDTS
gi|501188053|ref|WP_012231081.1|DNAdamageinducibleprotein[Bartonellatribocorum]_gi|161017434|emb|CAK00992.1|helicase/methyltra/	GCYQALTKLDLKND---------------------------------------LG-DDTA
gi|651388630|ref|WP_026500626.1|damageinducibleprotein[Bartonellagrahamii]/11657	GCYRALTKMDLKLD---------------------------------------LE-DDPK
gi|518048855|ref|WP_019219063.1|DNAdamageinducibleprotein[Bartonellaflorencae]/11658	GCYRALTKMDLKLD---------------------------------------LE-DDPK
gi|490995101|ref|WP_004856829.1|DNAdamageinducibleprotein[Bartonelladoshiae]_gi|395413167|gb|EJF79645.1|hypotheticalproteinMCS/	GCYRALTKMDLKID---------------------------------------LE-DDPK
gi|491002558|ref|WP_004864279.1|DNAdamageinducibleprotein[Bartonellavinsonii]_gi|395421392|gb|EJF87643.1|hypotheticalproteinME/	GCYQALSKIDLKLD---------------------------------------LN-DDPN
gi|491003373|ref|WP_004865094.1|DNAdamageinducibleprotein[Bartonellavinsonii]_gi|395432864|gb|EJF98838.1|hypotheticalproteinME/	GCYQALSKIDLKLD---------------------------------------LN-DDPN
gi|491002426|ref|WP_004864148.1|DNAdamageinducibleprotein[Bartonellavinsonii]_gi|395421767|gb|EJF88003.1|hypotheticalproteinME/	GCYQALSKIDLKVD---------------------------------------LS-DDPN
gi|491004024|ref|WP_004865745.1|DNAdamageinducibleprotein[Bartonellavinsonii]_gi|395432542|gb|EJF98522.1|hypotheticalproteinME/	GCYQALSKIDLKVD---------------------------------------LS-DDPN
gi|648396442|ref|WP_026088193.1|damageinducibleprotein[Bartonellarattaustraliani]/11593	GCYQALSKIDLKVD---------------------------------------LD-DDPN
gi|748190174|ref|WP_039761290.1|damageinducibleprotein[Bartonellaqueenslandensis]/11658	GCYQALSKIDLKVD---------------------------------------LD-DDPT
gi|494817599|ref|WP_007553007.1|DNAdamageinducibleprotein[Bartonellasp.DB56]_gi|395411312|gb|EJF77837.1|hypotheticalproteinMC/1	GCYQALTKMDLKID---------------------------------------LG-DDTR
gi|515763376|ref|WP_017195976.1|DNAdamageinducibleprotein[Bartonellabirtlesii]/11656	GCYQALTKMDLKID---------------------------------------LG-DDTR
gi|494589420|ref|WP_007348014.1|DNAdamageinducibleprotein[Bartonellarattimassiliensis]_gi|395416278|gb|EJF82667.1|hypothetical/	GCYQALAKIDLKVD---------------------------------------LS-DDPN
gi|506336469|ref|WP_015856188.1|DNAdamageinducibleprotein[Bartonellagrahamii]_gi|240267512|gb|ACS51100.1|helicase/methyltransf/	GCYQALAKIDLKVD---------------------------------------LS-DDPN
gi|749308087|ref|WP_040132227.1|damageinducibleprotein[Bartonellatribocorum]_gi|605051878|emb|CDO49988.1|helicase/methyltransf/	GCYQALAKIDLKVD---------------------------------------LN-DDPN
gi|515763800|ref|WP_017196400.1|DNAdamageinducibleprotein[Bartonellabirtlesii]/11658	GCYQALSKIDLKVD---------------------------------------LS-DDPN
gi|492186994|ref|WP_005773380.1|DNAdamageinducibleprotein[Bartonellaelizabethae]_gi|395418361|gb|EJF84688.1|hypotheticalprotei/	GCYQALSKIDLKVD---------------------------------------LS-DDPN
gi|501188615|ref|WP_012231634.1|DNAdamageinducibleprotein[Bartonellatribocorum]_gi|161017856|emb|CAK01414.1|helicase/methyltra/	GCYQALSKIDLKVD---------------------------------------LS-DDPN
gi|501187470|ref|WP_012230499.1|DNAdamageinducibleprotein[Bartonellatribocorum]_gi|161017090|emb|CAK00648.1|helicase/methyltra/	GCYQALSKIDLKVD---------------------------------------LS-DDPN
gi|501188636|ref|WP_012231655.1|DNAdamageinducibleprotein[Bartonellatribocorum]_gi|161017879|emb|CAK01437.1|helicase/methyltra/	GCYQALSKIDLKVD---------------------------------------LS-DDPN
gi|930151941|emb|CTQ34818.1|UvsWhelicase[Jannaschiarubra]/11641	GCYKALTKTDLKID---------------------------------------VG-TDTG
gi|928977106|ref|WP_054001461.1|damageinducibleprotein[Shimiasp.SK013]_gi|927698852|gb|KPA22698.1|typeIrestrictionenzymeEcoKIs/	GCYKALIKQDGDGD---------------------------------------FA-VDAQ
gi|738872465|ref|WP_036760884.1|damageinducibleprotein[Leisingeradaeponensis]/11643	GCYKALTKVDIAAD---------------------------------------LG-ADTQ
gi|518050881|ref|WP_019221089.1|DNAdamageinducibleprotein[Bartonellasenegalensis]/11652	GCYKALTKQDIKAD---------------------------------------LG-TDPY
gi|493980596|ref|WP_006923523.1|DNAdamageinducibleprotein[Bartonellawashoensis]_gi|395413322|gb|EJF79796.1|hypotheticalprotein/	GCYKALTKQDMKAD---------------------------------------VG-TDPY
gi|493983106|ref|WP_006925984.1|DNAdamageinducibleprotein[Bartonellawashoensis]_gi|395418127|gb|EJF84455.1|hypotheticalprotein/	GCYKALTKQDLKAD---------------------------------------VG-ADRH
gi|499494671|ref|WP_011181311.1|DNAdamageinducibleprotein[Bartonellahenselae]_gi|49239008|emb|CAF28308.1|hypotheticalproteinBH/	GCYKALTKQDMKAD---------------------------------------IQ-TDPY
gi|651388426|ref|WP_026500441.1|damageinducibleprotein[Bartonellagrahamii]/11652	GCYKALTKQDLKAD---------------------------------------VG-ADPH
gi|506337280|ref|WP_015856999.1|DNAdamageinducibleprotein[Bartonellagrahamii]_gi|240268402|gb|ACS51990.1|helicase/methyltransf/	GCYKALTKQDLKAD---------------------------------------VG-ADSH
gi|494817535|ref|WP_007552943.1|DNAdamageinducibleprotein[Bartonellasp.DB56]_gi|395412002|gb|EJF78518.1|hypotheticalproteinMC/1	GCYKALTKQDLKAD---------------------------------------IG-TDPH
gi|493638099|ref|WP_006589864.1|DNAdamageinducibleprotein[Bartonellabirtlesii]_gi|395409809|gb|EJF76394.1|hypotheticalproteinM/	GCYKALTKQDLKAD---------------------------------------VG-ADPY
gi|748189589|ref|WP_039760707.1|damageinducibleprotein[Bartonellaqueenslandensis]/11651	GCYKALTKKDIKAD---------------------------------------VG-VDSY
gi|501189483|ref|WP_012232501.1|DNAdamageinducibleprotein[Bartonellatribocorum]_gi|161018902|emb|CAK02460.1|helicase/methyltra/	GCYKALTKQDMKAD---------------------------------------VG-ADPY

```
gi|518049181|ref|WP_019219389.1|DNAdamageinducibleprotein[Bartonellaflorencae]/11652    GCYKALTKQNMKAD---------------------------------------VG-ADLY
gi|492193255|ref|WP_005774528.1|DNAdamageinducibleprotein[Bartonellaelizabethae]_gi|395416403|gb|EJF82779.1|hypotheticalprotei/    GCYKALTKQDMKAD---------------------------------------VG-MDQY
gi|498387325|ref|WP_010701288.1|helicase/methyltransferase[Bartonellabovis]_gi|478418724|gb|ENN92426.1|helicase/methyltransfera    GCYKALIKTDMKVD---------------------------------------VN-FDPA
gi|319408697|emb|CBI82352.1|conservedhypotheticalprotein[BartonellaschoenbuchensisR1]/11661    GCYKALTKTDIKAD---------------------------------------VS-FDPA
gi|494741726|ref|WP_007477134.1|DNAdamageinducibleprotein[Bartonellamelophagi]_gi|395423925|gb|EJF90113.1|hypotheticalproteinM/    GCYKALNKTDMKAD---------------------------------------VK-TDPA
gi|737261405|ref|WP_035245349.1|damageinducibleprotein[Alcanivoraxjadensis]_gi|686989709|gb|KGD62556.1|hypotheticalproteinT9A_/    GCYKALTKATLKDD---------------------------------------IQ-YDAK
gi|916687564|ref|WP_051294655.1|damageinducibleprotein[Gemmobacternectariphilus]/11641    GCYKALTKADMKED---------------------------------------VA-ADPH
gi|740294127|ref|WP_038132495.1|damageinducibleprotein[Thioclavasp.DT234]_gi|660524826|gb|KEO54689.1|hypotheticalproteinDT23_/1    GCYKALTKQDLQQD---------------------------------------IS-FDPQ
gi|568331062|ref|WP_024099615.1|helicase[Phaeobactergallaeciensis]_gi|568232121|gb|AHD12007.1|putativehelicase[Phaeobactergalla    GCYKALTKTDMKAD---------------------------------------VA-ADPH
gi|518810459|ref|WP_019966413.1|DNAdamageinducibleprotein[Pannonibacterphragmitetus]/11635    GCYKALTKIDLKAD---------------------------------------VS-ADPH
gi|495305760|ref|WP_008030510.1|DNAdamageinducibleprotein[Rhodobactersp.SW2]_gi|259021698|gb|EEW25000.1|typeIIIrestrictionprot/    GCYKALTKIDLKAD---------------------------------------VS-TDPH
gi|506403303|ref|WP_015923022.1|DNAdamageinducibleprotein[Rhodobactersphaeroides]_gi|221162951|gb|ACM03922.1|HypotheticalProte/    GCYKALTKVDLKAD---------------------------------------VA-ADPH
gi|738818386|ref|WP_036708790.1|damageinducibleprotein[Paracoccussanguinis]_gi|694226786|gb|KGJ22541.1|damageinducibleprotein/1    GCYKALTKVDLKAD---------------------------------------VA-ADPH
gi|746096057|ref|WP_039162453.1|damageinducibleprotein[Gallibacteriumanatis]_gi|703617775|gb|KGQ63868.1|damageinducibleprotei/1    GCWKALSKYGVEDD---------------------------------------LA-DDSQ
gi|737716785|ref|WP_035685631.1|damageinducibleprotein[Avibacteriumparagallinarum]/11643    GCWKALSKYGVEDE---------------------------------------LT-DDSQ
gi|540072553|gb|ERF78672.1|hypotheticalproteinN561_04930[Gallibacteriumanatis12656/12}/11651    GCWKALSKYGVEDE---------------------------------------LA-DDSQ
gi|917509519|ref|WP_052115936.1|damageinducibleprotein[Gallibacteriumanatis]_gi|702414161|gb|KGQ42233.1|damageinducibleprotei/1    GCWKALSKYGVEDE---------------------------------------LA-DDSQ
gi|746082942|ref|WP_039150268.1|damageinducibleprotein[Gallibacteriumanatis]_gi|703617078|gb|KGQ63194.1|damageinducibleprotei/1    GCWKALSKYGVEDD---------------------------------------LA-DDSQ
gi|822522885|ref|WP_046952840.1|damageinducibleprotein[Haemophilushaemolyticus]_gi|821501762|gb|KKZ59267.1|damageinduciblepro/1    GCWKALSKYGIMNE---------------------------------------LG-D-DE
gi|404381014|gb|EJZ77501.1|putativehelicase[Pasteurellamultocidasubsp.gallicidaP1059]/11681    GCWKALSKYGIMES---------------------------------------LG-D-NE
gi|298281660|gb|EFI23149.1|helicaseCterminaldomainprotein[Neisseriasp.oraltaxon014str.F0314]/11493    GCWKALSKYGLSGE---------------------------------------EG-ALYN
gi|348013055|gb|EGY51980.1|helicasedomainprotein[Neisseriashayeganii871]/11481    GCWKALSKYGLSGD---------------------------------------DG---YH
gi|496407140|ref|WP_009116004.1|DNAdamageinducibleprotein[Neisseriawadsworthii]_gi|349794756|gb|EGZ48566.1|helicasedomainprote/    GCWKALSKYGLSGE---------------------------------------DG-ALYN
gi|517216441|ref|WP_018405259.1|DNAdamageinducibleprotein[Marinobacterlipolyticus]/11656    GCWKALSKQGLFEV---------------------------------------AG-QQAN
gi|738230264|ref|WP_036185691.1|damageinducibleprotein[Marinobactersp.ELB17]/11671    GCWKALSKQGLFEV---------------------------------------AG-QQAN
gi|447197741|ref|WP_001274997.1|DNAdamageinducibleprotein[Acinetobacterbaumannii]_gi|408512324|gb|EKK13969.1|typeIIIrestrictio/    GCWKALAKQGLYNP---------------------------------------DG-SFSS
gi|820775897|ref|WP_046739909.1|damageinducibleprotein[Acinetobactersp.AG1]_gi|819909758|gb|KKW75938.1|damageinducibleprotein/1    GCWKALAKQGLYNP---------------------------------------DG-TIAK
gi|584595163|ref|WP_024160846.1|helicase[Acinetobacterpittii]_gi|576702530|emb|CDI28049.1|helicasedomaincontainingprotein[Acin/    GCWKALAKQGLYNP---------------------------------------DG-TVAK
gi|514963042|ref|WP_016651366.1|hypotheticalprotein[Acinetobactersp.NIPH2036]_gi|514162665|gb|EPG41391.1|hypotheticalproteinF90    GCWKALAKQGLYNP---------------------------------------DG-TVAK
gi|515965564|ref|WP_017396147.1|DNAdamageinducibleprotein[Acinetobacterhaemolyticus]/11667    GCWKALAKHGLSNP---------------------------------------DG-TAAK
gi|491346810|ref|WP_005204747.1|MULTISPECIES:hypotheticalprotein[Acinetobacter]_gi|480335823|gb|ENX56033.1|hypotheticalproteinF    GCWKALAKQGLYNP---------------------------------------DG-TVAK
gi|693044472|ref|WP_032215268.1|damageinducibleprotein[Escherichiacoli]_gi|607725731|gb|EZE08230.1|damageinducibleprotein[Esc/1    GCWKALAKQGLASS---------------------------------------DG-VYPE
gi|742382177|ref|WP_038861304.1|damageinducibleprotein[Cronobactermuytjensii]_gi|924333747|gb|ALB69766.1|damageinducibleprote/1    GCWKALAKQGLAGT---------------------------------------DG-VHSD
gi|915912804|ref|WP_050927035.1|damageinducibleprotein[Yersiniaenterocolitica]_gi|899805802|emb|CQH32042.1|typeIrestrictionenz/    GCWKALAKQGLAGT---------------------------------------DG-VHPH
gi|921111327|gb|KON62781.1|typeIrestrictionenzymeEcoKIsubunitR[Komagataeibactereuropaeus]/11492    GCWKALATGGLPRE---------------------------------------GT-AIPE
gi|582024278|gb|AHI27615.1|DNAhelicaserestrictionenzymeTypeIIIRsubunit[KomagataeibacterxylinusE25]/11636    GCWKALATGGLPRE---------------------------------------GT-TIPE
gi|347662673|dbj|BAK86128.1|DNAhelicaserestrictionenzymeTypeIIIRsubunit[KomagataeibactermedellinensisNBRC3288]/11737    GCWKALATGGLPRE---------------------------------------GT-TVPE
gi|917445053|ref|WP_052051471.1|damageinducibleprotein[Acetobactertropicalis]/11429    GCWKALAKIGLSQE---------------------------------------GV-DDPA
gi|453329445|dbj|GAC88472.1|DNAhelicaserestrictionenzymetypeIIIRsubunit[GluconobacterthailandicusNBRC3255]_gi|530287981|dbj|GAD    GCWKALAKIGLSQD---------------------------------------GV-EDPE
gi|889770922|ref|WP_048844278.1|damageinducibleprotein[Gluconobacterfrateurii]/11419    GCWKALAKIGLSHD---------------------------------------GV-EDPE
gi|504886208|ref|WP_015073310.1|hypotheticalprotein[Gluconobacteroxydans]_gi|411027954|gb|AFW01209.1|hypotheticalproteinB932_16    GCWKALAKIGLSQD---------------------------------------GV-GDPE
gi|480040211|gb|ENV79751.1|hypotheticalproteinF942_01641[AcinetobacterursingiiANC3649]/11432    GCWKALSKQNSETD---------------------------------------LQ-DDPN
gi|822498222|ref|WP_046936504.1|damageinducibleprotein[Xanthomonasgardneri]_gi|821751224|gb|KLB07223.1|damageinducibleprotein/1    GCWKALAKLGIHED---------------------------------------GV-ESPE
gi|654059559|ref|WP_027705556.1|damageinducibleprotein[Zymobacterpalmae]/11635    GCWKALSKIGLVGE---------------------------------------EG-GYSE
gi|643535620|ref|WP_025224315.1|damageinducibleprotein[Methylophilaceaebacterium11]_gi|577704400|gb|EUJ10546.1|putativehelicas/    GCWKALSKQDTQED---------------------------------------IV-DDLN
MBA11I/11635    GCWKALSKQDTQED---------------------------------------IV-DDLN
gi|655355857|ref|WP_028762287.1|damageinducibleprotein[Shewanellacolwelliana]/11632    GCWKALSKQDTQED---------------------------------------LS-DDLH
gi|753204236|ref|WP_041510579.1|damageinducibleprotein[Shewanellasp.cp20]_gi|752457765|gb|KIO36326.1|damageinducibleprotein[S/1    GCWKALSKHQMKEG---------------------------------------LE-LDPE
KPNNIH30III/11619    GCWKALSKHQMKEG---------------------------------------LD-IDPD
gi|749295994|ref|WP_040120352.1|damageinducibleprotein[Klebsiellapneumoniae]_gi|723249300|gb|AIX81926.1|damageinducibleprotei/1    GCWKALSKHQMKEG---------------------------------------LD-IDPD
gi|641639739|emb|CDQ54010.1|unnamedproteinproduct[Klebsiellapneumoniae]/11641    GCWKALSKHQMKEG---------------------------------------LD-IDPD
gi|490797922|ref|WP_004660060.1|hypotheticalprotein[Acinetobactersp.NIPH236]_gi|479876779|gb|ENU27973.1|hypotheticalproteinF992    GCWKALSKQGIHAD---------------------------------------LE-EDTA
gi|916750926|ref|WP_051357982.1|damageinducibleprotein[Acinetobactertjernbergiae]/11453    GCWKALSKQGIHAD---------------------------------------VE-EDTA
gi|691098662|ref|WP_032025060.1|damageinducibleprotein[Acinetobacterbaumannii]_gi|593567521|gb|EXR50735.1|restrictionendonucle/    GCWKALSKQGIHSD---------------------------------------VE-EDTA
gi|736669405|ref|WP_034676049.1|damageinducibleprotein[Acinetobacterbaumannii]/11643    GCWKALSKQGIHAD---------------------------------------VE-EDTN
gi|491368928|ref|WP_005226826.1|hypotheticalprotein[Acinetobactersp.NIPH1847]_gi|480173259|gb|ENX09797.1|hypotheticalproteinF89    GCWKALSKQGIHAD---------------------------------------VE-EDTN
gi|796853993|ref|WP_045795953.1|damageinducibleprotein[Acinetobacterindicus]_gi|787929761|gb|KJV44219.1|damageinducibleprotei/1    GCWKALSKQGFHAD---------------------------------------VE-EDTA
gi|295797463|gb|ADG32253.1|typeIIrestrictionproteinressubunit[ThiomonasintermediaK12]/11676    GCWKALSKQDLSKD---------------------------------------LI-GDDA
gi|499871179|ref|WP_011551913.1|DNAdamageinducibleprotein[Myxococcusxanthus]_gi|108467544|gb|ABF92729.1|putativerestriction/mo/    GCWKALSKQGLQEG---------------------------------------IT-DDHA
gi|495313852|ref|WP_008038600.1|DNAdamageinducibleprotein[Bartonellatamiae]_gi|395424181|gb|EJF90368.1|hypotheticalproteinME5_/    GCWKALSKQNLTES---------------------------------------LE-GDVS
gi|501453335|ref|WP_012476784.1|DNAdamageinducibleprotein[Leptospirabiflexa]_gi|167777824|gb|ABZ96123.1|Helicase[Leptospirabif/    GCWKALSKQGSKEN---------------------------------------SL-REED
gi|738104613|ref|WP_036063193.1|damageinducibleprotein[Leptospiranoguchii]/11652    GCWKALSKLGSKES---------------------------------------LS-DDTG
gi|410001039|gb|EKO51661.1|typeIIIrestrictionenzyme,ressubunit[Leptospirakirschneristr.200802841]/11651    GCWKALSKLGSKES---------------------------------------LS-DDTG
gi|490901851|ref|WP_004763774.1|typeIIIrestrictionenzyme,ressubunit[Leptospirakirschneri]_gi|464209249|gb|EMN24014.1|typeIIIres    GCWKALSKLGSKES---------------------------------------LS-DDTG
gi|464398131|gb|EMO65902.1|typeIIIrestrictionenzyme,ressubunit[Leptospirakirschneristr.200803703]/11648    GCWKALSKLGSKES---------------------------------------LS-DDTG
gi|945029024|gb|KQQ84900.1|damageinducibleprotein[Xanthomonassp.Leaf131]/11423    GCWKALAKLDITED---------------------------------------GS-DKPD
gi|896511314|ref|WP_049418183.1|damageinducibleprotein[Stenotrophomonasmaltophilia]/11639    GCWKALAKLGLHED---------------------------------------GS-DTPE
```

gi|896513082|ref|WP_049419616.1|damageinducibleprotein[Stenotrophomonasmaltophilia]/11638 GCWKALAKLGIHED---------------------------------------GS-ETPD
gi|896580616|ref|WP_049469425.1|damageinducibleprotein[Stenotrophomonasmaltophilia]/11641 GCWKALAKLGIHEG---------------------------------------AG-ESAE
gi|648289350|ref|WP_026070499.1|damageinducibleprotein[Stenotrophomonasmaltophilia]/11639 GCWKALAKLGIHEG---------------------------------------AG-ESAE
gi|656272786|ref|WP_029218442.1|damageinducibleprotein[Xanthomonascassavae]/11639 GCWKALAKLGIREG---------------------------------------AC-ESAE
gi|406976236|gb|EKD98747.1|hypotheticalproteinACD_23C00290G0002[unculturedbacterium]/11679 GCWKALAKQGLHDD---------------------------------------LL-GDGA
gi|828145737|ref|WP_047238120.1|damageinducibleprotein[Chromobacteriumsubtsugae]/11645 GCWKALSKQDTRED---------------------------------------LG-DDAA
gi|654311174|ref|WP_027824327.1|damageinducibleprotein[Laribacterhongkongensis]/11641 GCWKALSKQDTRED---------------------------------------LG-DDAA
gi|757242173|gb|KIR15516.1|typeIrestrictionenzymeEcoKIsubunitR[Pseudomonasfluorescens]/11778 GCWKALSKQDTRED---------------------------------------LV-DDGD
gi|653605161|ref|WP_027616682.1|damageinducibleprotein[Pseudomonassp.URHB0015]/11639 GCWKALSKQDTRED---------------------------------------LV-DDGD
gi|520868025|ref|WP_020308060.1|helicase[Pseudomonassyringae]_gi|519159034|gb|EPM49935.1|helicasedomaincontainingprotein[Pseud/ GCWKALSKQDTRED---------------------------------------LV-DDGD
gi|940156567|gb|KPX71668.1|Helicasedomaincontainingprotein[Pseudomonasamygdalipv.lachrymans]/11677 GCWKALSKQDTRED---------------------------------------LV-DDGD
gi|505460508|ref|WP_015647610.1|helicase[Pseudomonassp.GLE121]_gi|499110212|ref|YP_007974374.1|helicasedomaincontainingprotein/ GCWKALAKQDLTDD---------------------------------------LM-GDNE
gi|940235551|gb|KPY45311.1|hypotheticalproteinALO48_200154[Pseudomonassyringaepv.rhaphiolepidis]/11631 GCWKALAKQDLTDN---------------------------------------LV-SDNE
gi|520880808|ref|WP_020312235.1|helicase[Pseudomonassyringae]_gi|519172158|gb|EPM62027.1|helicasedomaincontainingprotein[Pseud/ GCWKALAKQGMADD---------------------------------------LI-GDNQ
gi|829046479|ref|WP_047282259.1|damageinducibleprotein[Pseudomonasfluorescens]/11630 GCWKALAKQGLAEN---------------------------------------LV-GDDQ
gi|499415493|ref|WP_011102960.1|DNAdamageinducibleprotein[Pseudomonassyringaegroupgenomosp.3]_gi|28867279|ref|NP_789898.1|heli/ GCWKALAKQGLAEN---------------------------------------LV-GDDQ
gi|730238299|ref|WP_033942897.1|damageinducibleprotein[Pseudomonasaeruginosa]/11629 GCWKALAKQGLHEQ---------------------------------------LI-GDDE
gi|495310125|ref|WP_008034873.1|DNAdamageinducibleprotein[Pseudomonassp.GM60]_gi|398186685|gb|EJM74052.1|putativehelicase[Pseu/ GCWKALAKQGLTEQ---------------------------------------LV-GDGE
gi|779992653|ref|WP_045428690.1|damageinducibleprotein[Pseudomonassp.MT1]_gi|699967872|dbj|BAP80334.1|DNAhelicaserestrictione/1 GCWKALAKQGLAEQ---------------------------------------LV-GDGQ
gi|786037289|ref|WP_045733274.1|damageinducibleprotein[Pseudomonaspseudoalcaligenes]_gi|782987904|gb|KJU79152.1|damageinducib/1 GCWKALAKQGLHEQ---------------------------------------LI-GDDE
gi|749666366|ref|WP_040265445.1|damageinducibleprotein[Pseudomonasrhodesiae]/11632 GCWKALAKQGLAEN---------------------------------------LV-GDDQ
gi|734902433|ref|WP_034133698.1|damageinducibleprotein[Pseudomonasfluorescens]/11635 GCWKALAKQGLAEN---------------------------------------LV-GDDQ
gi|490551031|ref|WP_004416146.1|typeIIrestrictionmodificationsystemrestriction/helicasesubunit[Pseudomonassyringae]_gi|4432823/ GCWKALAKQGLAEN---------------------------------------LV-GDDQ
gi|489199911|ref|WP_003109152.1|DNAdamageinducibleprotein[Pseudomonasaeruginosa]_gi|552648708|gb|ERY63184.1|hypotheticalprotei/ GCWKALAKQGLHEQ---------------------------------------LI-GDDE
gi|730641237|ref|WP_034061328.1|damageinducibleprotein[Pseudomonasaeruginosa]/11636 GCWKALAKQGLAEN---------------------------------------LV-GDDQ
gi|751304980|ref|WP_041013386.1|damageinducibleprotein[Pseudomonasxanthomarina]_gi|703407611|emb|CEG51014.1|Helicasedomaincon/1 GCWKALAKQGLAEN---------------------------------------LV-GDNQ
gi|939335207|ref|WP_054881520.1|damageinducibleprotein[Pseudomonassp.NBRC111128]/11632 GCWKALAKQGLAEN---------------------------------------LV-GDDQ
gi|757693555|ref|WP_042923792.1|damageinducibleprotein[Pseudomonaschloritidismutans]_gi|780910610|gb|KJS78302.1|damageinducib/1 GCWKALAKQGLAEN---------------------------------------LV-GDDQ
gi|665953944|ref|WP_031319989.1|damageinducibleprotein[Pseudomonastolaasii]/11626 GCWKALAKQGLHEQ---------------------------------------LV-GDDE
gi|771600573|ref|WP_045213746.1|damageinducibleprotein[Pseudomonassp.21]_gi|770081123|gb|KJJ98634.1|damageinducibleprotein[Ps/1 GCWKALAKQGLAEQ---------------------------------------LV-GDDQ
gi|740871396|ref|WP_038656644.1|damageinducibleprotein[Pseudomonasstutzeri]_gi|627772214|gb|AHY41437.1|damageinducibleprotein/1 GCWKALAKQGLAEN---------------------------------------LV-GDDQ
gi|946242205|gb|KRC93782.1|damageinducibleprotein[Pseudomonassp.Root9]/11634 GCWKALAKQGLAEN---------------------------------------LV-GDDQ
gi|553777322|ref|WP_023109107.1|MULTISPECIES:hypotheticalprotein[Pseudomonas]_gi|552638674|gb|ERY53184.1|hypotheticalproteinQ05 GCWKALAKQGLAEN---------------------------------------LV-GDDQ
gi|896383370|ref|WP_049324618.1|damageinducibleprotein[Pseudomonasstutzeri]/11627 GCWKALAKQGLHEQ---------------------------------------LI-GDDE
gi|696158510|gb|AIT41787.1|putativehelicase[Pseudomonassp.LM8]/11630 GCWKALAKQGLAEN---------------------------------------LV-GDDQ
gi|504634892|ref|WP_014821994.1|DNAdamageinducibleprotein[Pseudomonasstutzeri]_gi|390985249|gb|AFM35242.1|helicasedomainconta/1 GCWKALAKQGLAEN---------------------------------------LV-GDDQ
gi|544783974|ref|WP_021206729.1|DNAdamageinducibleprotein[Pseudomonasstutzeri]_gi|533224779|gb|EQM79688.1|DNAdamageinduciblep/1 GCWKALAKQGLHEQ---------------------------------------LI-GDDQ
gi|504567385|ref|WP_014754487.1|MULTISPECIES:DNAdamageinducibleprotein[Pseudomonas]_gi|388559493|gb|AFK68634.1|helicasedomain/1 GCWKALAKQGLAEN---------------------------------------LV-GDDQ
gi|654441469|ref|WP_027912052.1|damageinducibleprotein[Pseudomonassp.URIL14HWK12:I7]/11630 GCWKALAKQGLAEN---------------------------------------LV-GDDQ
gi|941470066|ref|WP_055127894.1|damageinducibleprotein[Pseudomonasmediterranea]/11631 GCWKALAKQGLAEN---------------------------------------LV-GDDQ
gi|557815599|ref|WP_023446184.1|DNAdamageinducibleprotein[Pseudomonaschloritidismutans]_gi|557364081|gb|ESQ98190.1|DNAdamagei/1 GCWKALAKQGLAEN---------------------------------------LV-GDDQ
gi|727814791|ref|WP_033898247.1|damageinducibleprotein[Pseudomonassp.FH1]_gi|567648005|gb|ETK23229.1|helicasedomaincontaining/1 GCWKALAKQGLAEN---------------------------------------LV-GDDQ
gi|503278702|ref|WP_013513363.1|DNAdamageinducibleprotein[Desulfovibrioaespoeensis]_gi|316942375|gb|ADU61426.1|typeIIIrestrict/ GCWKALSKQDVTTD---------------------------------------LV-DDTN
gi|505228796|ref|WP_015415898.1|typeIIIrestrictionendonucleasesubunitR[Desulfovibriopiezophilus]_gi|451896976|emb|CCH49855.1|Ty GCWKALSKQDVTDD---------------------------------------LV-DDAA
gi|517732543|ref|WP_018902751.1|hypotheticalprotein[Variovoraxparadoxus]/11675 GCWKALSKQGLSND---------------------------------------LA-GDDE
gi|550909475|ref|WP_022662570.1|DNAdamageinducibleprotein[Desulfovibriolongus]/11637 GCWKALSKQGVTDD---------------------------------------LV-GDSD
gi|945494542|gb|KQV50024.1|damageinducibleprotein[Massiliasp.Root335]/11647 GCWKALAKQGLTED---------------------------------------LN-GDHE
gi|334196917|gb|AEG70102.1|helicasedomainprotein[RalstoniasolanacearumPo82]/11663 GCWKALSKQDLTED---------------------------------------LV-GDNA
gi|656006746|ref|WP_029048170.1|damageinducibleprotein[Cupriavidussp.amp6]/11661 GCWKALSKQDLTGD---------------------------------------LV-GDDA
BPS2196ORF3054P/11659 GCWKALSKQGLTED---------------------------------------LV-GDGD
gi|740932312|ref|WP_038717332.1|damageinducibleprotein[Burkholderiapseudomallei]_gi|760211882|gb|KIX58314.1|damageinduciblepr/1 GCWKALSKQGLTED---------------------------------------LV-GDGD
gi|755810813|ref|WP_042583861.1|damageinducibleprotein[Burkholderiasp.MSHR3999]_gi|755174952|gb|KIP19790.1|DEAD/DEAHboxhelicas/ GCWKALSKQGLTED---------------------------------------LV-GDGG
gi|484586570|gb|AGK48195.1|DEAD/DEAHboxhelicasefamilyprotein[BurkholderiathailandensisMSMB121]/11646 GCWKALSKQGLTED---------------------------------------LV-GDGD
gi|737582935|ref|WP_035553941.1|damageinducibleprotein[Burkholderiasp.9120]/11658 GCWKALSKQGLTED---------------------------------------LV-GDGD
gi|740989872|ref|WP_038773225.1|damageinducibleprotein[Burkholderiapseudomallei]_gi|705645291|gb|KGS70691.1|DEAD/DEAHboxhelica/ GCWKALSKQGLTED---------------------------------------LV-GDGD
gi|654912525|ref|WP_028363424.1|damageinducibleprotein[Burkholderiasp.JPY366]/11659 GCWKALSKQGLTED---------------------------------------LV-GDGD
gi|740955879|ref|WP_038740512.1|damageinducibleprotein[Burkholderiapseudomallei]_gi|714684932|gb|KGW74438.1|DEAD/DEAHboxhelica/ GCWKALSKQGLTED---------------------------------------LV-GDGD
gi|919203714|ref|WP_052759215.1|damageinducibleprotein[Xanthomonasaxonopodis]/11388 GCWNGLSKRLATTD-----------------------------------GY-STAQLDPQ
gi|522152381|ref|WP_020663589.1|hypotheticalprotein[Amycolatopsisbenzoatilytica]/11605 GCWNGLSKRSGRTP-----------------------------------DG-EGFAPGSP
gi|663196013|ref|WP_030233899.1|damageinducibleprotein[Streptomycessp.NRRLS455]/11641 GCWNGLAKRTGTFG-----------------------------------DG-THFEPGEE
gi|750429553|ref|WP_040711444.1|damageinducibleprotein[Nocardiopsispotens]/11622 GCWNAMAKHTGTFA-----------------------------------DG-TGFGPEEA
gi|518656139|ref|WP_019819475.1|hypotheticalprotein[Saccharomonosporasaliphila]/11623 GCWNALAKRTGTFA-----------------------------------DG-SGFSEDET
gi|759722933|ref|WP_043433758.1|damageinducibleprotein[Streptomycespluripotens]_gi|733404978|gb|KHK94361.1|damageinduciblepro/1 GCWNALAKRTGTFA-----------------------------------DG-SGFGKDEA
gi|663424167|ref|WP_030419030.1|damageinducibleprotein[Streptomycessp.NRRLF5065]/11627 GCWNALAKRTGTFA-----------------------------------DG-SGFGKDEA
gi|663412917|ref|WP_030408353.1|damageinducibleprotein[Streptomycessp.NRRLF5917]/11630 GCWNALAKRTGTFA-----------------------------------DG-SGFGADEA
gi|671521483|ref|WP_031506489.1|damageinducibleprotein[Streptomycesmegasporus]/11630 GCWNALAKRTGTFA-----------------------------------DG-SGFGKDEA
gi|740053144|ref|WP_037902776.1|damageinducibleprotein[Streptomycessp.TAA204]/11626 GCWNAMAKRTGTFA-----------------------------------DG-SGFAEEEA

```
gi|663409375|ref|WP_030404974.1|MULTISPECIES:damageinducibleprotein[Streptomyces]/11627    GCWNAMAKRTGTFA-----------------------------------DG-SGFAEDDA
gi|746628608|ref|WP_039654277.1|damageinducibleprotein[Streptomycessp.MUSC125]_gi|745644381|gb|KIE25557.1|damageinducibleprot/1    GCWNAMAKRTGTFA-----------------------------------DG-SGFAEDEA
gi|727322066|ref|WP_033777320.1|damageinducibleprotein[Salinisporapacifica]/11627    GCWNAMAKRTGTFA-----------------------------------DG-SGFAEGEA
gi|653653242|ref|WP_027655726.1|damageinducibleprotein[Salinisporapacifica]/11618    GCWNAMAKRTGTFA-----------------------------------DG-SGFAEGEA
gi|648632543|ref|WP_026324294.1|damageinducibleprotein[Salinisporapacifica]/11621    GCWNAMAKRTGTFA-----------------------------------DG-SGFAEGEA
gi|881018002|ref|WP_048738463.1|damageinducibleprotein[Corynebacteriumfalsenii]/11676    GAWNGLAKRSGGAQ-----------------------------------GTKLGFEVGAK
gi|505465526|ref|WP_015652417.1|helicase[Corynebacteriumcallunae]_gi|459385704|gb|AGG67995.1|helicase[CorynebacteriumcallunaeDS    GAWNGLAKRSGKEQ-----------------------------------DTKTGFSSSDA
gi|41223062|emb|CAF19007.1|DNAorRNAhelicaseofsuperfamilyII[CorynebacteriumglutamicumATCC13032]/11646    GAWNGLAKRSGKEQ-----------------------------------DTKTGFSSSDA
CGL13032I/11643    GAWNGLAKRSGKEQ-----------------------------------DTKTGFSSSDA
gi|751257081|ref|WP_040967675.1|damageinducibleprotein[Corynebacteriumglutamicum]_gi|748811620|gb|AJE67931.1|damageinduciblep/1    GTWNGLTKRSGREQ-----------------------------------GTKSGFEHGAA
gi|749040539|ref|WP_040086524.1|damageinducibleprotein[Corynebacteriumhumireducens]/11663    GTWTALAKRSGREQ-----------------------------------GTKSGFEEDAA
gi|491662899|ref|WP_005519615.1|helicase[Corynebacteriummatruchotii]_gi|224946756|gb|EEG27965.1|helicaseCterminaldomainprotein/    GAWNGVAKRSGKEQ-----------------------------------GTSSGFAVDAE
gi|746717312|ref|WP_039676231.1|damageinducibleprotein[Corynebacteriumminutissimum]_gi|734991118|gb|KHO29219.1|damageinducibl/1    GAWNGLAKRSGAEQ-----------------------------------DTKSGFEANAQ
gi|908654892|emb|CQD15529.1|SuperfamilyIIDNA/RNAhelicase(modularprotein)[Corynebacteriumstriatum]/11674    GAWNGLAKRSGAEQ-----------------------------------DTKSGFEANAQ
gi|895994446|ref|WP_049064418.1|damageinducibleprotein[Corynebacteriumaurimucosum]/11667    GAWNGLAKRSGAEQ-----------------------------------DTKSGFEANAQ
gi|896123331|ref|WP_049145390.1|damageinducibleprotein[Corynebacteriumaurimucosum]/11669    GAWNGLAKRSGAEQ-----------------------------------DTKSGFEANAQ
gi|817681178|ref|WP_046645422.1|damageinducibleprotein[Corynebacteriumstriatum]_gi|817551679|gb|KKO79868.1|damageinduciblepro/1    GAWNGLAKRSGAEQ-----------------------------------DTKSGFEAYAL
gi|896139239|ref|WP_049159146.1|damageinducibleprotein[Corynebacteriumaurimucosum]/11675    GAWNGLAKRSGAEQ-----------------------------------DTKSGFEANAQ
gi|489943947|ref|WP_003847254.1|helicase[Corynebacteriumammoniagenes]_gi|295968218|gb|EFG81467.1|helicaseCterminaldomainprotei/    GAWNALAKRSGKLQ-----------------------------------GQKDGFDVGAA
gi|516653464|ref|WP_018022453.1|helicase[Corynebacteriumdoosanense]_gi|697778282|gb|AIT61018.1|damageinducibleprotein[Coryneba/    GAWNALAKRSGKLQ-----------------------------------GSKDGFELNAA
gi|493894179|ref|WP_006840129.1|helicase[Corynebacteriumlipophiloflavum]_gi|227078819|gb|EEI16782.1|helicaseCterminaldomainpro/    GAWNGLAKRSGKEQ-----------------------------------GTKSGFEHGAD
gi|740831472|ref|WP_038616755.1|damageinducibleprotein[Corynebacteriumpseudotuberculosis]_gi|666103058|gb|AIG06935.1|putativeh/    GAWNALAKRSGTLQ-----------------------------------GKKGGFGADDQ
gi|518023322|ref|WP_019193530.1|helicase[Corynebacteriumtimonense]/11655    GAWNALAKRSGKLQ-----------------------------------GGKDGFEVGAA
gi|552767697|ref|WP_023019559.1|MULTISPECIES:hypotheticalprotein[Corynebacterium]_gi|550739013|gb|ERS38609.1|hypotheticalprotei    GAWNALAKRSGKLQ-----------------------------------GTKDGFEVNAK
gi|652659121|ref|WP_027017812.1|damageinducibleprotein[Corynebacteriumpseudodiphtheriticum]/11644    GAWNALAKRSGKLQ-----------------------------------GTKDGFEVNAK
gi|749039309|ref|WP_040085390.1|damageinducibleprotein[Corynebacteriumhumireducens]/11657    GAWNGLAKRSGKEQ-----------------------------------GTKSGFDHGAE
gi|740835591|ref|WP_038620874.1|damageinducibleprotein[Corynebacteriumulcerans]_gi|698973854|gb|AIU33664.1|HelicaseCterminald/1    GAWNGLAKRSGKLQ-----------------------------------GNKGGFEKDAA
gi|805307599|ref|WP_046096452.1|damageinducibleprotein[Corynebacteriumulcerans]_gi|803357323|gb|AKA97638.1|Damageinduciblepro/1    GAWNGLAKRSGKLQ-----------------------------------GSKGGFEKNVA
gi|504083555|ref|WP_014317549.1|helicase[Corynebacteriumdiphtheriae]_gi|371584249|gb|AEX47914.1|superfamilyIIDNA/RNAhelicase[Co    GAWNGLAKRSGALQ-----------------------------------GKKGGFDEGSE
gi|896182193|ref|WP_049193580.1|damageinducibleprotein[Corynebacteriumjeikeium]/11645    GAWNGLAKRSGALQ-----------------------------------GKKGGFDKGSE
gi|917636459|ref|WP_052205460.1|damageinducibleprotein[Corynebacteriumriegelii]_gi|914295811|gb|AKV59187.1|damageinduciblepro/1    GAWNALAKRSGKLQ-----------------------------------GAKGGFDEGAT
gi|737013522|ref|WP_035009205.1|damageinducibleprotein[Corynebacteriumjeikeium]/11651    GAWNALAKRSGKLQ-----------------------------------GEKGGFEVNAA
gi|737006291|ref|WP_035002094.1|damageinducibleprotein[Corynebacteriumjeikeium]/11658    GAWNGLAKRSGDLQ-----------------------------------GQKGGFDIDAA
gi|737010378|ref|WP_035006133.1|damageinducibleprotein[Corynebacteriumjeikeium]/11650    GAWNGLAKRSGDLQ-----------------------------------GQKGGFDTNAA
gi|737010473|ref|WP_035006228.1|damageinducibleprotein[Corynebacteriumjeikeium]/11657    GAWNGLAKRSGKEQ-----------------------------------GTRLGFEESAK
gi|737006250|ref|WP_035002053.1|damageinducibleprotein[Corynebacteriumjeikeium]/11658    GAWNGLAKRSGKEQ-----------------------------------GTRLGFEESAK
gi|923018168|ref|WP_053412254.1|damageinducibleprotein[Corynebacteriumlactis]_gi|922845184|gb|ALA67504.1|helicase[Corynebacter/    GAWNGLAKRSGALQ-----------------------------------GKKGGFDEGAA
gi|923018885|ref|WP_053412970.1|damageinducibleprotein[Corynebacteriumlactis]_gi|922845845|gb|ALA68165.1|helicase[Corynebacter/    GAWNGLAKRSGALQ-----------------------------------GKRGGFDEGAA
gi|512464870|ref|WP_016422869.1|hypotheticalprotein[Corynebacteriumsp.HFH0082]_gi|512047830|gb|EPD47505.1|hypotheticalproteinHM    GAWNGLAKRSGSLQ-----------------------------------GKKGGFEEDAE
gi|881012697|ref|WP_048733406.1|damageinducibleprotein[Corynebacteriumurealyticum]/11670    GAWNGLAKRSGSLQ-----------------------------------GKKGGFEEDAA
gi|755608478|ref|WP_042530519.1|damageinducibleprotein[Corynebacteriumsingulare]_gi|754340239|gb|AJI78730.1|putativehelicase[C/    GAWNGLAKRSGELQ-----------------------------------GKKGGFDEDAQ
gi|491421796|ref|WP_005279593.1|helicase[Corynebacteriumaccolens]_gi|227077180|gb|EEI15143.1|helicaseCterminaldomainprotein[Co/    GAWNGLAKRSGELQ-----------------------------------GKKGGFDENAR
gi|550752286|gb|ERS51451.1|hypotheticalproteinHMPREF1282_00033[Corynebacteriumsp.KPL1856]_gi|550759961|gb|ERS58964.1|hypothetic    GAWNGLAKRSGELQ-----------------------------------GKKGGFEENAQ
gi|497955053|ref|WP_010269209.1|helicase[Corynebacteriumbovis]/11599    GCWNGLA---------------------------------------------GGFDAAAR
gi|860580040|ref|WP_048470569.1|hypotheticalprotein[Mycobacteriumchlorophenolicum]_gi|859098575|gb|KMO76871.1|typeIrestrictione    GCWNGLAKRAGASV-----------------------------------DGEPSFAPGEL
gi|262085905|gb|ACY21873.1|typeIIIrestrictionproteinressubunit[GordoniabronchialisDSM43247]/11632    GCWNGLAKRAGTTP-----------------------------------DGT-GFAPDEP
gi|495996338|ref|WP_008720917.1|typeIIIrestrictionendonucleasesubunitR[Rhodococcussp.AW25M09]_gi|443412350|emb|CCQ18455.1|TypeI    GCWNGLAKRAGRSP-----------------------------------DGD-GFAIGAP
gi|739284636|ref|WP_037147410.1|damageinducibleprotein[Rhodococcusfascians]/11641    GCWNGLAKRAGRSP-----------------------------------DGD-GFAIGAP
gi|739328195|ref|WP_037190574.1|damageinducibleprotein[Rhodococcusfascians]/11643    GCWNGLAKRAGRSP-----------------------------------DGD-GFAIGAP
gi|811698335|emb|CMN21041.1|helicase[Mycobacteriumtuberculosis]/11725    GCWNGLAKRSG----------------------------------------T-GIVAGEP
gi|801577898|emb|CFK23773.1|helicase[Mycobacteriumtuberculosis]/11675    GCWNGLAKRSG----------------------------------------T-GIVAGEP
MTU18II/11606    GCWNGLAKRSG----------------------------------------T-GIVAGEP
gi|887719858|emb|CMT27886.1|helicase[Mycobacteriumtuberculosis]/11667    GCWNGLAKRSG----------------------------------------T-GIVAGEP
gi|621694743|gb|KAS87156.1|hypotheticalproteinAL70_01801[MycobacteriumtuberculosisTKK_03_0115]/11604    GCWNGLAKRSG----------------------------------------T-GIVAGEP
gi|888128072|emb|CLQ84422.1|helicase[Mycobacteriumtuberculosis]_gi|889917090|emb|CLY86893.1|helicase[Mycobacteriumtuberculosis]    GCWNGLAKRSG----------------------------------------T-GIVAGEP
gi|884823424|emb|CKL96672.1|helicase[Mycobacteriumtuberculosis]/11466    GCWNGLAKRSG----------------------------------------T-GIVAGEP
gi|505103239|ref|WP_015290341.1|hypotheticalprotein[Mycobacteriumcanettii]_gi|432162733|emb|CCK60117.1|Conservedproteinofunknow    GCWNGLAKRSG----------------------------------------T-GVAAGEP
gi|505116250|ref|WP_015303352.1|hypotheticalprotein[Mycobacteriumcanettii]_gi|432158726|emb|CCK56026.1|Conservedproteinofunknow    GCWNGLAKRSG----------------------------------------T-GIVAGEP
gi|432154735|emb|CCK51974.1|Conservedproteinofunknownfunction(part1)[MycobacteriumcanettiiCIPT140060008]/11588    GCWNGLAKRSG----------------------------------------T-GIVAGEP
gi|809008027|ref|WP_046295375.1|hypotheticalprotein[Mycobacteriumsp.UM_Kg17]/11624    GCWNGLAKRAGTAP-----------------------------------DGS-GFAPGEV
gi|808802633|ref|WP_046284139.1|hypotheticalprotein[Mycobacteriumsp.UM_NZ2]/11624    GCWNGLAKRAGTAP-----------------------------------DGS-GFAPGEA
gi|750125774|ref|WP_040429852.1|damageinducibleprotein[Corynebacteriumnuruki]/11668    GCWNGLAKRAGTAP-----------------------------------DG-SSFSPGEI
gi|602237614|gb|EYT60960.1|damageinducibleprotein[Dietziasp.UCDTHP]/11473    GCWNGLAKRAGATA-----------------------------------SG-DSFPPGAQ
gi|665897412|ref|WP_031264618.1|damageinducibleprotein[Dietziasp.UCDTHP]_gi|602237599|gb|EYT60945.1|helicase[Dietziasp.UCDTH/11    GCWNGLAKRAGATA-----------------------------------SG-ESFPPGAQ
gi|502903674|ref|WP_013138650.1|helicase[Segniliparusrotundus]_gi|296181291|gb|ADG98197.1|typeIIIrestrictionproteinressubunit[S    GCWNGLAKRAGAAV-----------------------------------DG-TGFAPGEV
gi|780248306|ref|WP_045538930.1|hypotheticalprotein[Gordoniasp.no.9]_gi|778142514|gb|KJR06917.1|hypotheticalproteinUG54_12320[G    GCWNGLAKRAGAAI-----------------------------------DG-STFTPGEN
gi|665897368|ref|WP_031264574.1|damageinducibleprotein[Dietziasp.UCDTHP]_gi|602239137|gb|EYT62483.1|helicase[Dietziasp.UCDTH/11    GCWNGLAKRAGTSP-----------------------------------TG-EGFAPGEP
```

gi|493372923|ref|WP_006329236.1|helicase[Gordoniarhizosphera]_gi|403207917|dbj|GAB88168.1|hypotheticalproteinGORHZ_006_00370[Go	GCWNGLAKRSGTTP----------------------------------GGEQGFTPGER
gi|764904870|ref|WP_044487821.1|hypotheticalprotein[Mycobacteriumtuberculosis]_gi|625005353|gb|KBZ57120.1|hypotheticalproteinK8	GCWNGLAKRAGKTP----------------------------------DG-HGFTSGEA
gi|927068212|ref|WP_053778441.1|damageinducibleprotein[Gordoniasp.NB41Y]_gi|926660541|gb|EMP14526.2|damageinducibleprotein[G/11	GAWNGLAKRAGKTP----------------------------------DG-TGFTPGEP
gi|935514169|ref|WP_054429894.1|damageinducibleprotein[Mycobacteriumimmunogenum]_gi|929801120|gb|KPG29247.1|damageinduciblepr/1	GCWNGLAKRAGKTP----------------------------------DG-QGFAPGEA
gi|941317728|ref|WP_055118574.1|hypotheticalprotein[Mycobacteriumperegrinum]/11657	GCWNGLAKRAGKTP----------------------------------DG-QGFAPGEA
gi|933683673|gb|ALG86377.1|hypotheticalproteinACH46_20120[Gordoniasp.QH11]/11636	GCWNGLAKRAGKTP----------------------------------EG-TGFAPGEA
gi|873553947|emb|CRY84110.1|Predictedhelicase[Nocardiafarcinica]/11688	GCWNGLAKRAGKTP----------------------------------DG-VGFPPGQP
gi|499525749|ref|WP_011212389.1|helicase[Nocardiafarcinica]_gi|54019339|dbj|BAD60707.1|hypotheticalprotein[NocardiafarcinicaIFM	GCWNGLAKRAGKTP----------------------------------EG-VGFPPGQP
gi|640109609|ref|WP_024794187.1|damageinducibleprotein[Tomitellabiformata]/11653	GCWNGLAKRAGQTP----------------------------------DG-TGFQPGEL
gi|640109991|ref|WP_024794334.1|hypotheticalprotein[Tomitellabiformata]/11639	GCWNGLAKRAGQTP----------------------------------DG-TGFAPGEL
gi|939125496|ref|WP_054831438.1|damageinducibleprotein[Rhodococcuserythropolis]/11669	GCWNGLAKRAGETP----------------------------------EG-TGFAPGEA
gi|910258550|ref|WP_050062711.1|hypotheticalprotein[Rhodococcussp.RD6.2]_gi|846871967|emb|CRK49225.1|conservedhypotheticalprote	GCWNGLAKRAGRTP----------------------------------DG-TGFAPGEA
gi|797148189|gb|KJY52358.1|putativeHelicase[Bifidobacteriumasteroides]/11801	GAWNGLLTRGVHTNSALESVDIDTGLGEQVQFAVMGNQSQ-------RQSGGETRTETVQ
gi|535476570|gb|EFG27357.2|hypotheticalproteinHMPREF9020_00999[ScardoviainopinataF0304]/11827	GAWNGLMTKGIHRQE-QQDIDIDTGLGEQVQFAILNDNDV-------DSHSGL--TSSIE
gi|737482729|ref|WP_035462496.1|hypotheticalprotein[Alicyclobacillusmacrosporangiidus]/11512	ALWKTLHD---TSVQP
gi|685434354|gb|KGA95679.1|helicase[BacillusalcalophilusATCC27647=CGMCC1.3604]/11575	GCWNGLVKRKSNSD--ETLGD
gi|945370253|gb|KQU27251.1|helicase[Bacillussp.Leaf13]/11557	GCWNGLVRRRSNSD--EALGN
gi|880997015|ref|WP_048719116.1|helicase[Enterococcushirae]/11563	GCWNGLIKRKNNSN--ILVGK
gi|945356929|gb|KQU14100.1|helicase[Bacillussp.Leaf49]/11547	GCWNGLVRRQSDSD--KTLGP
gi|763482876|gb|KIZ53217.1|helicase[Bacillussafensis]/11563	GCWNGLVKRKGNTN--ETIGQ
gi|852223292|ref|WP_048309707.1|helicase[Anaerobacillusmacyae]_gi|850322910|gb|KMM38590.1|helicase[Anaerobacillusmacyae]/11573	GCWNGLVKRRSNSN--VISAN
gi|739708665|ref|WP_037562619.1|helicase[Sporolactobacillusterrae]/11573	GCWNGLIKRKSNSN--AISAN
gi|496687561|ref|WP_009329104.1|MULTISPECIES:helicase[Bacillus]_gi|317390867|gb|EFV71668.1|endonucleaseandmethylaseLlaGI[Bacill	GCWNGLIKRKSNSN--VISAN
gi|930213599|gb|KPH74225.1|helicase[Oceanobacilluscaeni]/11569	GCWNGLIKRKSNTN--VISAN
gi|516366003|ref|WP_017756036.1|helicase[Bacillussp.ZYK]/11571	GCWNGLIKRRSNSN--VISAN
gi|488909977|ref|WP_002821052.1|helicase[Oenococcusoeni]_gi|399965230|gb|EJN99855.1|endonucleaseandmethylaseLlaGI[Oenococcusoen	GVWNAMIKRKSFSD--AVSGQ
gi|495019967|ref|WP_007745780.1|helicase[Oenococcuskitaharae]_gi|366983682|gb|EHN59081.1|typeIIIrestrictionenzyme,ressubunit[Oe	GVWNAMIKRKSFSD--AVSGQ
gi|489666119|ref|WP_003570406.1|typeIIIrestrictionendonuclease[Lactobacilluscasei]_gi|410527834|gb|EKQ02696.1|typeIIIrestrictio	GVWNAMMKRESFSN--KVNGA
gi|511447623|gb|EPC78110.1|superfamilyIIDNA/RNAhelicase[Lactobacillusparacaseisubsp.paracaseiLpp221}/11615	GVWNAMMKRESFSN--KTTGA
gi|497700411|ref|WP_010014595.1|helicase[Lactobacilluscoryniformis]/11585	GVWNAMMKRESFSN--KTAGN
gi|489784048|ref|WP_003687939.1|helicase[Lactobacillusmali]_gi|394428539|gb|EJF01080.1|superfamilyIIDNA/RNAhelicase[Lactobacill	GVWNAMMKRESFSN--KVSGK
gi|446780825|ref|WP_000858081.1|helicase[Staphylococcusaureus]_gi|374396604|gb|EHQ67835.1|typeIIIrestrictionenzyme,ressubunit[S	GIWNGMIKRQGTSG--KVNGR
gi|510796658|ref|WP_016171456.1|hypotheticalprotein[Enterococcusdispar]_gi|508167532|gb|EOT44083.1|hypotheticalproteinOMK_00227	GVWNAMIKREGATG--KVSGT
gi|612613473|gb|EZS30411.1|hypotheticalproteinW603_02482[StaphylococcusaureusVET0353R]_gi|612881917|gb|EZU93992.1|hypotheticalp	GVWNAMIKREGATG--KVSGV
gi|488332893|ref|WP_002402278.1|helicase[Enterococcusfaecalis]_gi|306513864|gb|EFM82466.1|helicaseCterminaldomainprotein[Enter/	GVWNGLMKRSSVD---KE--------------------------------------AIFEGK
gi|921166029|ref|WP_053111806.1|helicase[Enterococcusdurans]_gi|915771660|gb|AKX85928.1|helicase[Enterococcusdurans]/11562	GVWNGLMKRSSVD---KE--------------------------------------AVFEGK
gi|498525358|ref|WP_010825030.1|hypotheticalprotein[Enterococcusfaecalis]_gi|487059631|gb|EOJ86727.1|hypotheticalproteinWOC_005	GVWNGLMKRSSVD---KD--------------------------------------AVFEGK
gi|498412624|ref|WP_010722070.1|hypotheticalprotein[Enterococcusfaecium]_gi|486634654|gb|EOF75253.1|hypotheticalproteinSGC_0253	GVWNGLMKRSSVD---KD--------------------------------------AVFEGK
gi|498408534|ref|WP_010718364.1|hypotheticalprotein[Enterococcusfaecium]_gi|486609608|gb|EOF50873.1|hypotheticalproteinSCW_0257	GVWNGLMKRSSVD---KD--------------------------------------AVFEGK
gi|498407475|ref|WP_010717483.1|hypotheticalprotein[Enterococcusfaecalis]_gi|486594855|gb|EOF36348.1|hypotheticalproteinSCK_005	GVWNGLMKRSSVD---KE--------------------------------------AVFEGK
gi|514885392|ref|WP_016615344.1|helicase[Enterococcusfaecalis]_gi|514505843|gb|EPH71676.1|helicaseprotein[Enterococcusfaecalis0	GVWNGLMKRSSVD---KE--------------------------------------AIFEGK
gi|325157603|gb|EGC69759.1|helicaseCterminaldomainprotein[EnterococcuscasseliflavusATCC12755]/11561	GVWNGLMKRSSVD---KG--------------------------------------AVFEGK
gi|895856206|ref|WP_048967668.1|helicase[Enterococcusfaecalis]/11551	GVWNGLMKRSSVD---KE--------------------------------------AVFEGK
gi|639458836|ref|WP_024636366.1|helicase[Enterococcusfaecium]_gi|658176502|gb|KEI51159.1|helicase[EnterococcusfaeciumUC7256]/1/	GVWNGLMKRSSVD---KE--------------------------------------AVFEGK
gi|488266511|ref|WP_002337719.1|hypotheticalprotein[Enterococcusfaecium]_gi|430581409|gb|ELB19854.1|hypotheticalproteinOIQ_0351	GVWNGLMKRSSVD---KD--------------------------------------AVFEGK
gi|446353356|ref|WP_000431211.1|helicase[Staphylococcusaureus]_gi|253726329|gb|EES95058.1|helicaseCterminaldomainprotein[Staph/	GIWNGMMRRNGYKNPIKN--------------------------------------SPFDGA
gi|672712068|emb|CDM14336.1|DNAorRNAhelicaseofsuperfamilyII[StaphylococcusepidermidisPM221]/11464	GIWNGMMRRNGYKNPIKN--------------------------------------SPYDGA
gi|896298990|ref|WP_049279905.1|helicase[Staphylococcusaureus]/11571	GIWNGMMRRNGYKNPIKN--------------------------------------SPYDGA
gi|541903432|gb|ERH36190.1|hypotheticalproteinSEQU_00085[StaphylococcusequorumUMCCNS924]/11578	GIWNGMMRRNGYKNPIKN--------------------------------------SPYDGA
gi|488289747|ref|WP_002360955.1|helicase[Enterococcusfaecalis]_gi|256682216|gb|EEU21911.1|endonucleaseandmethylaseLlaGI[Enteroc	GIWNGMMRRNGYKNPIKN--------------------------------------SPYDGA
gi|494392045|ref|WP_007207894.1|helicase[Enterococcusitalicus]_gi|315483798|gb|EFU74284.1|helicaseCterminaldomainprotein[Enter/	GIWNGMMRRNGYKNPVKN--------------------------------------SPYDGA
gi|446350692|ref|WP_000428547.1|helicase[Bacilluscereus]_gi|228727580|gb|EEL78721.1|RestrictionmodificationsystemLlaBIII[Bacil/	GIWNGMMRRNGYKNPVKS--------------------------------------SPYSGA
gi|488255302|ref|WP_002326510.1|hypotheticalprotein[Enterococcusfaecium]_gi|430480276|gb|ELA57459.1|hypotheticalproteinOGE_0473	GIWNGMMRRNGYKNPIKN--------------------------------------SPYDGA
gi|843253770|ref|WP_047916698.1|helicase[Lactococcuspiscium]_gi|802124677|emb|CEN29502.1|EndonucleaseandmethylaseLlaGI,TypeISPr	GIWNGMMRRNGYKNPIKN--------------------------------------SPYDGA
LlaBIII/11578	GIWNGMMRRNGYKNPIKN--------------------------------------SPYDGA
gi|499320531|ref|WP_011011023.1|helicase[Listeriainnocua]_gi|16415805|emb|CAC42019.1|pli0021[ListeriainnocuaClip11262]/11569	GIWNGMMRRNGYKNPIKN--------------------------------------SPYDGA
gi|760026859|ref|WP_043709973.1|helicase[Weissellacibaria]_gi|758337165|gb|KIU20061.1|UvsWhelicase[Weissellacibaria]/11567	GIWNGMMRRNGYKNPVKN--------------------------------------SPYDGA
LlaGI/11570	GIWNGMMRRNGYKNPIKN--------------------------------------SPYDGA
gi|499988414|ref|WP_011669132.1|helicase[Lactococcuslactis]_gi|116109019|gb|ABJ74139.1|DNAorRNAhelicaseofsuperfamilyII[Lactococ	GIWNGMMRRNGYKNPIKN--------------------------------------SPYDGA
gi|685938360|ref|WP_031670319.1|helicase[Listeriamonocytogenes]_gi|590403387|gb|EXL23261.1|hypotheticalproteinX846_1956[Listeri	GIWNGMMRRNGYKNPIKN--------------------------------------SPYDGA
gi|530793219|gb|EQC56240.1|helicase[Lactococcuslactissubsp.cremorisTIFN5]_gi|530793247|gb|EQC56263.1|helicase[Lactococcuslactis	GIWNGMMRRNGYKNPIKN--------------------------------------SPYDGA
gi|695270006|ref|WP_032492075.1|hypotheticalprotein[Listeriamonocytogenes]_gi|695212872|ref|YP_009081522.1|hypotheticalproteinp	GIWNGMMRRNGYKNPIKN--------------------------------------SPYDGA
gi|504101310|ref|WP_014335297.1|helicase[Leuconostocmesenteroides]_gi|859247905|gb|AKO63885.1|helicase(plasmid)[Leuconostocmese	GIWNGMMRRNGYKNPVKN--------------------------------------SPYDGA
gi|488905190|ref|WP_002816265.1|helicase[Leuconostocmesenteroides]_gi|227351522|gb|EEJ41777.1|helicaseCterminaldomainprotein[L/	GVWNGMMRRNGYNNPVKN--------------------------------------SPYDGA
gi|518121285|ref|WP_019291493.1|MULTISPECIES:hypotheticalprotein[Lactococcus]/11588	GVWNGMIKRNSDSD--TYWGA
gi|754857213|ref|WP_042217902.1|helicase[Lactococcusgarvieae]/11549	GVWNGMLKRETFSN--NVSGS
gi|640048455|ref|WP_024784762.1|helicase[Streptococcusmutans]/11564	GVWNGMIKRESFTD--NVSGE

```
gi|512695361|ref|WP_016476323.1|hypotheticalprotein[Veillonellasp.HPA0037]_gi|512079853|gb|EPD79018.1|hypotheticalproteinHMPREF    GVWNGMVKRETFSD-----------------------------------------KVSGE
gi|636963514|ref|WP_024403747.1|helicase[Streptococcussuis]/11574    GVWNGMIKRESFTD-----------------------------------------KVSGE
gi|881115312|ref|WP_048802949.1|helicase[Staphylococcuspasteuri]/11573    GVWNGMIKREGFTN-----------------------------------------KVSGE
gi|446063996|ref|WP_000141851.1|helicase[Streptococcusmitis]_gi|307618725|gb|EFN97864.1|endonucleaseandmethylaseLlaGI[Streptoco    GVWNGMIKRESFAD-----------------------------------------KVSGE
gi|488206609|ref|WP_002277817.1|restrictionmodificationsystemLlaBIII[Streptococcusmutans]_gi|449173033|gb|EMB75626.1|restricti/    GVWNGMIKRESFAD-----------------------------------------KVSGE
gi|640023290|ref|WP_024781621.1|helicase[Streptococcusmutans]/11566    GVWNGIIKRESFAD-----------------------------------------KVSGE
gi|814946581|ref|WP_046393781.1|helicase[Streptococcusuberis]_gi|810600885|gb|KKF57499.1|helicase[StreptococcusuberisB362]/1156    GIWNGMVKRETFAD-----------------------------------------KVSGE
gi|765470762|ref|WP_044720336.1|helicase[Streptococcussuis]/11559    GVWNAMVKRESFTD-----------------------------------------KVSGE
gi|654507127|ref|WP_027976443.1|helicase[Streptococcusdevriesei]/11558    GVWNGMIKRESFSD-----------------------------------------KVSGE
gi|530812902|gb|EQC72481.1|DNAorRNAhelicaseofsuperfamilyII[Streptococcussp.HSISS2]/11570    GVWNGMIKRESFAD-----------------------------------------KVSGE
gi|489158846|ref|WP_003068504.1|helicase[Streptococcusintermedius]_gi|355367114|gb|EHG14827.1|hypotheticalproteinHMPREF9682_002    GVWNGMIKRESFAD-----------------------------------------KVSGE
RM.Lru27782ORF13200P/11620    GIWNAMTKRNGMNG-----------------------------------------AISGA
gi|492676546|ref|WP_005919236.1|DNAphosphorothioationsystemrestrictionenzyme[Pediococcusacidilactici]_gi|482517924|gb|EOA08103.    GVWNAMVKRNGLNG-----------------------------------------EIKGA
gi|498183116|ref|WP_010497272.1|helicase[Lactobacillusacidipiscis]/11563    GVWNAMVKRNGING-----------------------------------------EISGA
gi|822100485|ref|WP_046921865.1|helicase[Lactobacillusruminis]_gi|821684442|gb|KLA43321.1|restrictionmodificationsystemLlaBIII/    GIWNAMTKRNGMNG-----------------------------------------AITGA
gi|935565832|ref|WP_054450311.1|helicase[Lactobacilluskunkeei]/11573    GVWNAMVKRNGITG-----------------------------------------EVTGA
gi|752296887|ref|WP_041152901.1|helicase[Lactobacilluskunkeei]_gi|751663520|gb|KIM18358.1|helicase[Lactobacilluskunkeei]/11577    GVWNAMVKRNGITG-----------------------------------------EVTGA
gi|495745291|ref|WP_008469870.1|helicase[Lactobacillushominis]_gi|394484442|emb|CCI81207.1|RestrictionmodificationsystemLlaBII/    GVWNAMVKRNGVTG-----------------------------------------EITGA
gi|918672414|ref|WP_052543134.1|helicase[Lactobacillussp.]/11564    GVWNAMVKRDGITG-----------------------------------------EIDGA
gi|799141631|ref|WP_045927261.1|helicase[Lactobacilluskullabergensis]_gi|797155335|gb|KJY59264.1|putativerestrictionendonucleas    GVWNAMVKRDGITG-----------------------------------------EITGA
gi|504379990|ref|WP_014567092.1|helicase[Lactobacillusjohnsonii]_gi|329666726|gb|AEB92674.1|putativerestrictionendonuclease[Lac    GVWNAMVKRNGITD-----------------------------------------EINGA
gi|489880179|ref|WP_003783646.1|DNAdamageinducibleprotein[Actinomycesnaeslundii]_gi|399901561|gb|EJN84440.1|typeIIIrestriction/    GCWNALAKRSVNT---------------------------------------ADYGRDIQ
gi|496524497|ref|WP_009232780.1|DNAdamageinducibleprotein[Actinomycessp.oraltaxon849]_gi|365265413|gb|EHM95177.1|hypotheticalp/    GCWNALAKRSVDT---------------------------------------ADYGKDIQ
gi|915397782|ref|WP_050783167.1|damageinducibleprotein[Actinomycesoris]/11694    GCWNALAKRSVDT---------------------------------------ADYGKDIQ
gi|502925248|ref|WP_013160224.1|helicase[Propionibacteriumfreudenreichii]_gi|296921272|emb|CBL55824.1|helicase[Propionibacteriu    GCWNGLAKRTTDL----------------------------------------DFGKNPA
gi|732164352|emb|CEG87481.1|helicase[Propionibacteriumfreudenreichii]/11618    GCWNGLAKRTADQ----------------------------------------DFGPDPV
gi|739047402|ref|WP_036925058.1|damageinducibleprotein[Propionibacteriumthoenii]/11596    GCWNGLAKRTTDM----------------------------------------DFGEHPT
gi|739112839|ref|WP_036982533.1|damageinducibleprotein[Propionibacteriumjensenii]/11594    GCWNGLAKRTTDM----------------------------------------DFGDHPT
gi|918023558|ref|WP_052314121.1|damageinducibleprotein[Nocardiathailandica]/11612    GCWNGLAKNFGPE---------------------------IG------------TAGDRT
gi|498380271|ref|WP_010694427.1|DNAdamageinducibleprotein[Saccharopolysporaspinosa]/11605    GCWNGLAKHFGTD---------------------------E-------------QVTDRT
gi|749991459|ref|WP_040339809.1|damageinducibleprotein[CandidatusBlastococcusmassiliensis]/11597    GCWNGLAKNFGPS---------------------------ATD--------GQPSVVGRA
gi|754177801|ref|WP_041796231.1|damageinducibleprotein[Modestobactermarinus]/11592    GCWNGLAKHFGPV---------------------------ATD--------GEA-PADKV
gi|504188248|ref|WP_014375350.1|DNAdamageinducibleprotein[Blastococcussaxobsidens]_gi|378782790|emb|CCG02456.1|putativeHelicas/    GCWNGLAKNFGPG---------------------------ASD--------DDARAVDKA
gi|946476713|gb|KRF26798.1|damageinducibleprotein[Phycicoccussp.Soil803]/11609    GCWNGLAKRFGDA---------------------------ATA--------DDLDGDDLT
gi|700176672|gb|KGN37509.1|DNAdamageinducibleprotein[KnoelliasubterraneaKCTC19937]/11633    GCWNGLAKRFGDA---------------------------LTS--------DDLDGDDLT
gi|700168841|gb|KGN29914.1|hypotheticalproteinN802_10215[KnoelliasinensisKCTC19936]/11631    GCWNGLAKRFGDA---------------------------ATA--------DDLDGDDLT
gi|946426241|gb|KRE76610.1|damageinducibleprotein[Arthrobactersp.Soil763]/11606    GCWNGLAKKGHIE---------------------------------------HNFDADLE
gi|517318525|ref|WP_018500927.1|hypotheticalprotein[Frankiasp.BCU110501]/11592    GCWNGLAKRGKLE---------------------------------------HSFEPDAR
gi|499072429|ref|WP_010851327.1|Helicase[Tetrasphaeraelongata]_gi|478756579|emb|CCH71501.1|putativeHelicase[Tetrasphaeraelongat    GCWNGLAKRGSAE---------------------------------------HSFDADPA
gi|750080666|ref|WP_040385350.1|damageinducibleprotein[Demetriaterragena]/11625    GCWNGLAKHFDTL-----------------------------------------EGESRA
gi|752558292|ref|WP_041229523.1|damageinducibleprotein[Dermacoccusnishinomiyaensis]_gi|664689315|gb|AIF41904.1|damageinducibl/1    GCWNGLAKHFDTV-----------------------------------------GGEDLA
gi|829089213|ref|WP_047310701.1|damageinducibleprotein[Dermacoccussp.PE3]_gi|828384812|gb|KLO63770.1|damageinducibleprotein[D/1    GCWQGLAKHFDTV-----------------------------------------EGESLA
gi|748756635|ref|WP_040014750.1|damageinducibleprotein[Dermacoccussp.Ellin185]/11596    GCWQGLAKHFDTV-----------------------------------------EGESLA
gi|916534724|ref|WP_051141848.1|damageinducibleprotein[Actinomycesneuii]/11632    GCWNAMAKRRSGE---------------------------ID----------VSYGQDTA
gi|921140030|ref|WP_053090276.1|damageinducibleprotein[Rothiamucilaginosa]/11616    GCWNGLAKRRSGE---------------------------LE----------ASFGDDLA
gi|896406751|ref|WP_049343749.1|damageinducibleprotein[Rothiamucilaginosa]/11647    GCWNGLAKRRSGE---------------------------LE----------ASFGDDLE
gi|723608234|gb|AIY01610.1|putativehelicase[Arthrobactersp.PAMC25486]/11613    GVWNGLAKRRAGE---------------------------FQ----------DGFGQDWQ
gi|946394527|gb|KRE45160.1|damageinducibleprotein[Arthrobactersp.Soil736]/11616    GCWNGLAKRRSGV---------------------------HE----------VSFGNDLA
gi|515765304|ref|WP_017197904.1|hypotheticalprotein[Arthrobactersp.M2012083]/11648    GCWNGLAKRRSGA---------------------------HE----------VSFGNDLA
gi|945315587|gb|KQT73127.1|damageinducibleprotein[Microbacteriumsp.Leaf436]/11612    GTWNGLSKRSVIP---------------------------G------------EYEDDPL
gi|759908497|ref|WP_043594599.1|damageinducibleprotein[Frigoribacteriumsp.MEB024]_gi|758271359|gb|KIU03346.1|damageinduciblep/1    GIYNGLAKRGVHG---------------------------IDT----------TAPNAHQ
gi|945072850|gb|KQR34237.1|hypotheticalproteinASF75_00055[Curtobacteriumsp.Leaf154]/11597    GIYNGLAKRGVHG---------------------------IDT----------TTPNAHQ
gi|551263152|ref|WP_022883363.1|DNAdamageinducibleprotein[Glaciibactersuperstes]/11621    GIYNGLAKRGVQG---------------------------LGN----------Q-AADRT
gi|945452341|gb|KQV08491.1|hypotheticalproteinASC63_10170[Leifsoniasp.Root112D2]/11623    GIYNGLAKRGVQG---------------------------LGP----------W-PMDIA
gi|742759483|gb|KIC69912.1|damageinducibleprotein[Kocuriarhizophila]/11610    GCWKGLSTRGSAP---------------------------GGR-----DDDAVPHLGAPA
gi|737919995|ref|WP_035885304.1|damageinducibleprotein[Kocuriarhizophila]/11611    GCWKGLSTRGSAP---------------------------GGR-----DDA-APRPGAPA
gi|737997701|ref|WP_035959325.1|damageinducibleprotein[Kocuriamarina]_gi|725823884|gb|KHE75603.1|damageinducibleprotein[Kocur/1    GCWKGLSTRGSGQ---------------------------GGR-----GDAVASHSGAPA
gi|664521070|ref|WP_031037650.1|damageinducibleprotein[Streptomycessp.NRRLWC3774]/11624    GCWNAMAKRTGTF---------------------------AD--------GSGFG-EEEA
gi|500101034|ref|WP_011777041.1|DNAdamageinducibleprotein[Arthrobacteraurescens]_gi|119951547|gb|ABM10457.1|putativeHelicase[A/    GCWNGLAKRGVD--------------------------------------QSRLEV-TGA
gi|916871953|ref|WP_051479009.1|damageinducibleprotein[Arthrobactersp.H5]/11588    GCWNGLAKRGVD--------------------------------------QHRLEV-SGT
gi|612084649|gb|EZP43369.1|TypeIIIrestrictionenzyme,ressubunit[Micrococcusluteus]/11625    GCWNGLAKRGVD--------------------------------------GGRLDEVDAA
gi|759739708|ref|WP_043450422.1|damageinducibleprotein[Arthrobacterphenanthrenivorans]_gi|742758264|gb|KIC68716.1|damageinduc/1    GCWNGLSKRGVN--------------------------------------GERLEISDGS
gi|636845448|ref|WP_024366823.1|damageinducibleprotein[Arthrobactersp.TB26]/11605    GCWNGLSKRGVN--------------------------------------GERLSIGDTS
gi|500009025|ref|WP_011689743.1|DNAdamageinducibleprotein[Arthrobactersp.FB24]_gi|116613051|gb|ABK05760.1|typeIIIrestrictionen/    GCWNGLSKRGVN--------------------------------------GERLSIGDTS
gi|821249847|gb|KKZ12083.1|hypotheticalproteinTQ37_06090[CandidatusSynechococcuspongiarum15L]/11436    GCLRALAKMDGE----------------------------------------QFPEGDRA
gi|739446902|ref|WP_037306885.1|damageinducibleprotein[Ruegeriahalocynthiae]/11592    GCYKALQKHGDN----------------------------------------QEFAVDAD
gi|640620081|ref|WP_025048131.1|damageinducibleprotein[Sulfitobactermediterraneus]_gi|751956650|gb|KIN77213.1|TypeIIIrestricti/    GCYKALIKQGGD----------------------------------------DDFAVDAQ
```

```
gi|740247634|ref|WP_038088583.1|damageinducibleprotein[Thiobacillusprosperus]_gi|684049748|gb|KFZ90853.1|putativehelicase[Thio/      GSWKGLSKQGLKV---------------------------ID------DGEVSDLEEDTA
gi|518420321|ref|WP_019590528.1|hypotheticalprotein[Thioalkalivibriosp.ALE20]/11603                                                 GAWKGLSKRGLVA---------------------------VDE-----QGAEEAVTEDTN
gi|500156826|ref|WP_011831496.1|DNAdamageinducibleprotein[Methylibiumpetroleiphilum]_gi|124261892|gb|ABM96881.1|adeninespecifi/     GAWKGLSKHGLVE---------------------------VDE-----TGDQAALQEDLQ
gi|769978537|ref|WP_045094522.1|damageinducibleprotein[Legionellafallonii]_gi|698841736|emb|CEG55682.1|Putativeproteinwithboxh/      GCWKGLSKDGLVT---------------------------INE-----QE-AQELTEDIL
gi|553752541|ref|WP_023085344.1|hypotheticalprotein[Pseudomonasaeruginosa]_gi|552201952|gb|ERU62653.1|hypotheticalproteinQ088_0     GSWKGLSKRGLVS---------------------------VGE-----DGQEEALVEDVA
gi|880980418|ref|WP_048704461.1|damageinducibleprotein[Thauerahumireducens]_gi|875132785|gb|KMU70944.1|damageinducibleprotein/1     GSWKGLSKQGLVA---------------------------VGE-----DGQEETLTEDVE
gi|740135732|ref|WP_037982815.1|damageinducibleprotein[Thauerasp.27]/11603                                                          GSWKGLSKRGLVA---------------------------VGE-----DGQEETLTEDVE
gi|738017371|ref|WP_035978332.1|damageinducibleprotein[Burkholderiaglumae]/11380                                                    GSWKGLSKRGLVL---------------------------VGE-----DGQEEALSDDTA
gi|498506003|ref|WP_010806671.1|typeIIIrestrictionenzyme,ressubunit[Pandoraeasp.SD62]_gi|490314561|gb|EON12879.1|typeIIIrestri/     GSWKGLSKRGLVL---------------------------VGE-----DGQEDALSEDTA
gi|500117748|ref|WP_011793753.1|DNAdamageinducibleprotein[Acidovoraxcitrulli]_gi|120587742|gb|ABM31182.1|typeIIIrestrictionenz/     GSWKGLSKKGLVA---------------------------VDD-----DGQEDALNEDAA
gi|651617328|ref|WP_026611444.1|damageinducibleprotein[Methylocaldumszegediense]/11601                                              GTWKGLSKRGLVL---------------------------VAD-----DGEQENLTEDTA
gi|737210208|ref|WP_035194994.1|damageinducibleprotein[Acidithiobacillusferrivorans]_gi|669953220|emb|CDQ11755.1|Adeninespecif/     GCWNGLAKRGAL-------------------------------------GSVD-FGGDAQ
gi|935697386|ref|WP_054506180.1|damageinducibleprotein[Achromobactersp.]_gi|928716153|emb|CUK12836.1|Hefnuclease[Achromobacter/     GCWNGLGKHVSI------------------------------------DDGMD-VSADPQ
gi|429502517|gb|ELA00827.1|adeninespecificDNAmethyltransferase[Cupriavidussp.HMR1]/11599                                            GCWNGLGKHVSV------------------------------------DDGMD-VSVDPQ
gi|506356642|ref|WP_015876361.1|DNAdamageinducibleprotein[Burkholderiaglumae]_gi|237877158|gb|ACR29491.1|AdeninespecificDNAmet/     GCWNGLGKHVSV------------------------------------EDGLD-VSADPQ
gi|696406787|ref|WP_032978003.1|damageinducibleprotein[Bordetellahinzii]_gi|625335076|gb|KCB49343.1|typeIIIrestrictionenzyme,r/     GCWNGLGKHVAA------------------------------------EDGMD-VSVDPQ
gi|817529125|gb|KKO72587.1|damageinducibleprotein[Kerstersiagyiorum]/11592                                                         GCWNGLGKHVAA------------------------------------EDGMD-VSVDPQ
gi|737048910|ref|WP_035044118.1|damageinducibleprotein[Aquabacteriumsp.NJ1]_gi|699052896|gb|KGM38706.1|damageinducibleprotein/1     GCWNGLGKHAAI------------------------------------EDGND-FSVDPL
gi|822492519|ref|WP_046933319.1|MULTISPECIES:damageinducibleprotein[Xanthomonas]_gi|821703525|gb|KLA61196.1|damageinduciblepr/1     GCWNGLGKHVAA------------------------------------EDGMD-ISADPQ
gi|751252861|ref|WP_040963740.1|damageinducibleprotein[Xanthomonasfuscans]_gi|749808363|gb|KIJ03887.1|damageinducibleprotein[/1     GCWNGLGKHVAA------------------------------------EDGMD-ISGDPQ
gi|496774980|ref|WP_009368792.1|DNAdamageinducibleprotein[Bilophilasp.4_1_30]_gi|345039902|gb|EGW44205.1|hypotheticalproteinHM/     GCWTGLGKRLAE------------------------------------DDKAD-VASDPK
gi|499241912|ref|WP_010939452.1|DNAdamageinducibleprotein[Desulfovibriovulgaris]_gi|46580580|ref|YP_011388.1|adeninespecificDN/     GCWNGLGKRLAE------------------------------------DDTAD-KHSDPL
gi|504274913|ref|WP_014462015.1|DNAdamageinducibleprotein[Bacillusmegaterium]_gi|345446919|gb|AEN91936.1|AdeninespecificDNAmet/     GCWNGLSKKEAN------------------------------------SR-----KTFGP
gi|504895966|ref|WP_015083068.1|typeIIrestrictionenzymeandmethyltransferasesubtypeG[Anabaenasp.90]_gi|413972424|gb|AFW96512.1|t     GCWNGLAKRILN------------------------------------DEEGDDT-EDKT
gi|518327765|ref|WP_019497972.1|hypotheticalprotein[Calothrixsp.PCC7103]/11600                                                      GCWNGLSKRLAT------------------------------------DADGKEIEHDTT
gi|652765503|ref|WP_027079917.1|damageinducibleprotein[Luteimonasmephitis]/11593                                                    GCWNGLSKRIAP------------------------------------VEGDNTAETDPN
gi|896539951|ref|WP_049442123.1|damageinducibleprotein[Stenotrophomonasmaltophilia]/11591                                           GCWNGLSKRLAT------------------------------------TDGDNTAQLDPH
gi|500217900|ref|WP_011888043.1|DNAdamageinducibleprotein[Geobacillusthermodenitrificans]_gi|134267954|gb|ABO68149.1|Conserved/     GCWNALSKRVMP------------------------------------DG-TS-MLEDPR
gi|696570298|ref|WP_033102014.1|damageinducibleprotein[Thermoactinomycesdaqus]/11594                                                GCWNALSKRFME------------------------------------NE-SD-QAADLM
gi|251772067|gb|EES52637.1|putativehelicase[Leptospirillumferrodiazotrophum]/11618                                                  GCWNGLSKKMVS------------------------------------ED-GE-IRSDLS
gi|738124101|ref|WP_036082108.1|damageinducibleprotein[Leptospirillumferriphilum]_gi|685243306|gb|KGA94177.1|putativehelicase[/     GCWNGLSKKMVL------------------------------------ED-GE-VRSDLP
gi|558699223|ref|WP_023524766.1|helicase[Leptospirillumsp.GroupII'CF1']_gi|206603764|gb|EDZ40244.1|Putativehelicase[Leptospiri/     GCWNGLSKKMVS------------------------------------ED-GE-VRSDPF
gi|406774462|gb|AFS53887.1|putativehelicase[LeptospirillumferriphilumML04]/11610                                                    GCWNGLSKRVFE------------------------------------NSHEE-NLTDPA
gi|740719463|ref|WP_038504749.1|damageinducibleprotein[Leptospirillumferriphilum]_gi|640838125|gb|AIA30173.1|damageinduciblep/1     GCWNGLSKRVFD------------------------------------NGHEE-NLTDPV
gi|517810766|ref|WP_018980974.1|hypotheticalprotein[Saccharibacterfloricola]/11733                                                  GCWRAMLGHDLVT-------------------------ETGA---------EGAEKPIPQ
gi|502471675|ref|WP_012798533.1|helicase[Slackiaheliotrinireducens]_gi|256791761|gb|ACV22431.1|predictedhelicase[Slackiaheliotr     GCWKGLLDQGRK--------------------------------------------EGGT
gi|657871713|ref|WP_029576297.1|hypotheticalprotein[Bifidobacteriumthermacidophilum]_gi|673007521|gb|KFJ02535.1|helicase[Bifido     GCWKALFDRRHAR--GVEDIV-----------------QIGR-----HARREDTNDDAKR
gi|673004757|gb|KFI99791.1|DNAhelicaserestrictionenzymetypeIIIRessubunit[Bifidobacteriumthermacidophilumsubsp.porcinum]/11682       GCWKALFDRRHAR--GVEDIV-----------------QIGR-----HARREDTNDDAKR
gi|917316092|ref|WP_051922804.1|hypotheticalprotein[Bifidobacteriumstellenboschense]_gi|673003774|gb|KFI98812.1|helicase[Bifido     GCWKALFDRRHSK--SVDELV-----------------SRGR-----HAAETAP-DDSKR
gi|705415694|ref|WP_033502332.1|hypotheticalprotein[Bifidobacteriumpseudolongum]_gi|672982411|gb|KFI77685.1|DNAdamageinducible/     GCWKAMFDRRHST--SVADLA-----------------TRGR-----HAETTVGDDESNR
gi|916591418|ref|WP_051198509.1|hypotheticalprotein[Bifidobacteriumsp.AGR2158]/11704                                                GCWKALFDRRHAS--NLNGV-------------------VGR-----HAQAGLEDDESRQ
gi|772242982|gb|AJW78255.1|hypotheticalproteinVO01_03165[Clavibactermichiganensissubsp.insidiosus]/11642                            GCYNALAKRNAGQ---------------------------L----------AGGFGADLK
gi|916649818|ref|WP_051256909.1|damageinducibleprotein[Actinomycesgraevenitzii]/11677                                               GCWNALAKRKNPG---------------------------S----------DVDYGSDCS
gi|496504578|ref|WP_009212861.1|helicase[Actinomycessp.oraltaxon180]_gi|315312695|gb|EFU60776.1|helicase[Actinomycessp.oraltaxo     GCWNALAKRKNRD---------------------------S----------DVDYGEDRA
gi|494862206|ref|WP_007588306.1|helicase[Actinomycessp.ICM39]_gi|398362799|gb|EJN46470.1|typeIIIrestrictionenzyme,ressubunit[Ac     GCWNALAKRKNRG---------------------------S----------DVDYGEDRA
gi|881057111|ref|WP_048774974.1|damageinducibleprotein[Sanguibacterkeddieii]/11697                                                  GCWNALAKRKNRG---------------------------S----------DVDYGEDRA
gi|881020459|ref|WP_048740808.1|damageinducibleprotein[Kytococcussedentarius]/11704                                                 GCWNALAKRKNRG---------------------------S----------DVDYGEDRA
gi|881052313|ref|WP_048770555.1|damageinducibleprotein[Cellulomonasflavigena]/11705                                                 GCWNALAKRKNPG---------------------------S----------DVDYGEDRA
gi|490108682|ref|WP_004009424.1|helicase[Mobiluncuscurtisii]_gi|315490465|gb|EFU80089.1|helicaseCterminaldomainprotein[Mobilun/     GCWNALSKRKNRF---------------------------A----------DMQYGNDTE
gi|490111935|ref|WP_004012603.1|helicase[Mobiluncusmulieris]_gi|227843812|gb|EEJ53982.1|helicaseCterminaldomainprotein[Mobilun/     GCWNALAKRKNSF---------------------------T----------DTQYGDDTN
gi|490114228|ref|WP_004014848.1|helicase[Mobiluncusmulieris]_gi|269934432|gb|EEZ90993.1|helicaseCterminaldomainprotein[Mobilun/     GCWNALAKRKNSF---------------------------T----------DTQYGDDTN
gi|493333551|ref|WP_006290636.1|helicase[Parascardoviadenticolens]_gi|315119487|gb|EFT82620.1|helicaseCterminaldomainprotein[P/     GTYKGLATHGVEAQARLNQLEDSHEL--TPDFLLINKVELNNDSSAEQETSGIIEGQDIQ
gi|495817067|ref|WP_008541646.1|helicase,partial[Sutterellaparvirubra]_gi|378598583|gb|EHY31712.1|helicaseprotein,partial[Sutte     GAWRALSKCD--F---------------------------KGE---------RSMWDDLK
gi|491572696|ref|WP_005430278.1|helicase[Sutterellawadsworthensis]_gi|319805116|gb|EFW01943.1|DNAhelicaserestrictionenzymeTypeI     GCWRALSKLD--L---------------------------VNE---------VSMSDDRQ
gi|748686321|ref|WP_039944445.1|hypotheticalprotein[Thermicanusaegyptius]/11573                                                     GTWRALSGHV--R---------------------------GEE---------------IP
gi|916875698|ref|WP_051482754.1|hypotheticalprotein[Thioalkalivibriosp.HK1]/11366                                                   GCWRALHDPENLA---------------------------GKASSRSRPDSQTEPRPKPR
gi|821248604|gb|KKZ10960.1|hypotheticalproteinTE42_09075[CandidatusSynechococcusspongiarumSP3]/11598                                 GCLRALAKMD---------------------------------G-------EQFPEGDRA
gi|737409843|ref|WP_035390956.1|hypotheticalprotein[Ferrimicrobiumacidiphilum]_gi|765544266|gb|KJE76241.1|typeIrestrictionenzym     GCWMGLAKIS-------------------------------DSQ--------EEFANDPD
gi|654906925|ref|WP_028358281.1|damageinducibleprotein[Brackiellaoedipodis]/11679                                                   GCWKALAKYQ--L---------------------------DSKS--------NNSNVDLT
gi|502809901|ref|WP_013044877.1|hypotheticalprotein[CandidatusPuniceispirillummarinum]_gi|292663146|gb|ADE38247.1|hypotheticalp     GCYNALRNRP--------------------------------------------EESDSK
gi|251771370|gb|EES51950.1|typeIIIrestrictionenzyme,ressubunit[Leptospirillumferrodiazotrophum]/11628                               GAWKALAKEGVTT---------------------------DDGH--------PAFDPGDP
gi|498328480|ref|WP_010642636.1|DNAdamageinducibleprotein[Acidithiobacillusthiooxidans]/11627                                       GAWKGLSNHNVQV---------------------------IDGA-------EVTETSNAP
gi|647633864|ref|WP_025906698.1|damageinducibleprotein[Prochlorococcussp.scB245a_519B7]/11622                                       GCYKALAKIG--I---------------------------IDEQ--------NAKQKENK
gi|647619599|ref|WP_025893729.1|damageinducibleprotein[Prochlorococcussp.scB241_527N11]/11621                                       GCYKALAKIG--I---------------------------QKNS--------VGINEDKI
gi|755034887|ref|WP_042387498.1|hypotheticalprotein[Clostridiumsp.CL6]/11606                                                        GCWNGLSKKS--I---------------------------ESED--------DSFKVDPY
gi|498139798|ref|WP_010453954.1|helicase[SuccinivibrionaceaebacteriumWG1]/11662                                                     GILNGLSKNI--R---------------------------YDDG--------KTWQVDPQ
gi|527501386|gb|EPT34571.1|typeIIIrestrictionenzyme,ressubunit[Bacteroidetesbacteriumoraltaxon272str.F0290]/11789                   GCVNALSKRI--K---------------------------GDKG--------ITAAEDPG
```

```
gi|496047275|ref|WP_008771782.1|helicase[Bacteroidessp.2_1_33B]_gi|262296443|gb|EEY84373.1|helicaseCterminaldomainprotein[Bact/    GCINGLSKRI--K---------------------------GDKG--------VTKEADPV
PDI8503III/11664    GCINGLSKRI--K---------------------------GDKG--------VTKEADPV
gi|738719431|ref|WP_036613715.1|helicase[Parabacteroidesdistasonis]_gi|649542518|gb|KDS49387.1|restrictionendonucleasefamilypro    GCINGLSKRI--K---------------------------GDKG--------VTKEADPV
gi|659417033|gb|KEJ86133.1|hypotheticalproteinHMPREF1002_00341[Porphyromonassp.31_2]/11656    GCINGLSKRI--K---------------------------GDKG--------VTKEADPV
gi|567456807|gb|ETK11358.1|hypotheticalproteinT235_15845[Tannerellasp.oraltaxonBU063isolateCell8/11]/11433    GVINGLSKKI--L---------------------------GDKG--------ITWESDPR
gi|545440279|ref|WP_021677877.1|helicase[Porphyromonasgingivalis]_gi|543985765|gb|ERJ89108.1|helicaseprotein[Porphyromonasgingi    GVINGLSKKI--L---------------------------GDKG--------VTWESDPH
gi|497438056|ref|WP_009752254.1|helicase[Prevotellasp.oraltaxon473]_gi|429144866|gb|EKX87968.1|helicaseprotein[Prevotellasp.ora    GVINGLSKKI--M---------------------------GDKG--------VTWDADPR
gi|490423474|ref|WP_004295683.1|MULTISPECIES:helicase[Bacteroides]_gi|156110098|gb|EDO11843.1|helicaseCterminaldomainprotein[B/    GIVNGLSKII--R---------------------------GDKH--------ATWDADPT
gi|941777229|ref|WP_055173506.1|helicase[Bacteroidescaccae]_gi|933263395|emb|CUQ00567.1|Predictedhelicase[Bacteroidescaccae]/1/    GVINGLSKMI--H---------------------------GDDH--------RTWDADPR
gi|492413898|ref|WP_005836892.1|helicase[Bacteroidesuniformis]_gi|392678824|gb|EIY72229.1|hypotheticalproteinHMPREF1072_03583[B    GVINGLSKMI--H---------------------------GDDH--------RTWDADPR
gi|695455095|ref|WP_032566191.1|helicase[Bacteroidesfragilis]_gi|596095426|gb|EXZ59996.1|restrictionendonucleasefamilyprotein[B    GVINGLSKMI--Q---------------------------GDDH--------RTWDADPR
gi|933227941|emb|CUQ02348.1|Predictedhelicase[Bacteroidesuniformis]/11658    GVINGLSKMI--Q---------------------------GDDH--------RTWDADPR
gi|494823013|ref|WP_007558421.1|helicase[Bacteroidesplebeius]_gi|198272167|gb|EDY96436.1|helicaseCterminaldomainprotein[Bacter/    GVINGLSKMI--Q---------------------------GDDH--------RTWDADPR
gi|495427212|ref|WP_008151909.1|helicase[Parabacteroidesjohnsonii]_gi|218222381|gb|EEC95031.1|helicaseCterminaldomainprotein[P/    GVINGLSKMI--R---------------------------GDDH--------RTWDADPH
gi|933196855|emb|CUO94545.1|typeIrestrictionenzymeEcoKIsubunitR[Bacteroidesuniformis]_gi|933220098|emb|CUN86874.1|typeIrestrict    GVINGLSKMI--Q---------------------------GDDH--------RTWDADPH
gi|823687748|ref|WP_047101121.1|helicase[Brachyspirahyodysenteriae]_gi|823334537|gb|KLI17932.1|helicase[Brachyspirahyodysenteri    GCINGLSKRI--I---------------------------GDDG--------LLKDKDPL
gi|496436691|ref|WP_009145538.1|helicase[Phascolarctobacteriumsuccinatutens]_gi|322414007|gb|EFY04839.1|helicaseCterminaldomai/    GVINALSKRV--V---------------------------PDPD--------IVKSVDPA
gi|503372880|ref|WP_013607541.1|helicase[Sphaerochaetaglobosa]_gi|324026933|gb|ADY13692.1|typeIIIrestrictionproteinressubunit[S    GCINALSKRT--K----------------------------ETS--------TFSDVDPG
gi|505821748|ref|WP_015707145.1|helicase[Treponemaprimitia]_gi|299483503|gb|ADJ19584.1|helicasedomaincontainingprotein[Trepone/    GCINALSKKS--L---------------------------TDKE--------IFENIDPQ
gi|505822741|ref|WP_015708134.1|helicase[Treponemaprimitia]_gi|333739639|gb|AEF85129.1|endonucleaseandmethylaseLlaGI[Treponemap    GCINALSKKS--L---------------------------TDKE--------IFENIDPQ
gi|488791994|ref|WP_002704400.1|helicase[Treponemasaccharophilum]_gi|380767691|gb|EIC01689.1|typeIIIrestrictionproteinressubuni    GCINVLSKRTNYL---------------------------TDK--------ELFTDEDPE
gi|524671454|emb|CDD81750.1|putativeseptumsitedeterminingproteinMinC[Bacteroidessp.CAG:462]/11642    GCINALSKRV--L---------------------------GD-E-------GLIKAADPL
gi|524573865|emb|CDC95766.1|putativeseptumsitedeterminingproteinMinC[Alistipessp.CAG:268]/11639    GCINALSKKV--L---------------------------GADE-------EFVKGSDPL
gi|658617970|ref|WP_029759328.1|helicase[Fusobacteriumnucleatum]/11606    GCINGLSKQI--L---------------------------ETEG--------IIKSTDPE
gi|494412037|ref|WP_007216782.1|helicase[Bacteroidescellulosilyticus]_gi|392638921|gb|EIY32752.1|hypotheticalproteinHMPREF1062_    GCINALSKKT--L---------------------------LDSK--------LLKETDPA
gi|383107102|gb|AFG37435.1|putativehelicase[SpirochaetaafricanaDSM8902]/11616    GCINALSKQV--I---------------------------GDEG--------TIKGTDPM
SAF8902III/11616    GCINALSKQV--I---------------------------GDEG--------TIKGTDPM
gi|652538894|ref|WP_026932796.1|helicase[Gramellaechinicola]/11633    GCINALSKQV--L---------------------------GDEG--------LIKSSDPN
gi|639238223|ref|WP_024568839.1|helicase[Elizabethkingiaanophelis]/11628    GCINALSKQV--L---------------------------GDEG--------LLKGSDPE
gi|652520381|ref|WP_026914582.1|helicase[Gramellaportivictoriae]/11623    GCLNAMSKQV--L---------------------------GDNG--------LIKDVDPE
gi|494922183|ref|WP_007648219.1|Helicase[Formosasp.AK20]_gi|469825489|gb|EMQ95550.1|Helicasedomainprotein[Formosasp.AK20]/11626    GCLNALSKQV--L---------------------------GDNG--------LIKEVDPE
gi|522083382|ref|WP_020594591.1|hypotheticalprotein[Spirosomapanaciterrae]/11637    GTINALSKQI--L---------------------------GDEG--------IIKETDPE
gi|490455352|ref|WP_004326157.1|helicase[Bacteroidesovatus]_gi|392669277|gb|EIY62768.1|hypotheticalproteinHMPREF1070_03635[Bact    GCINALSKQI--L---------------------------GDAG--------IIKDSDPE
gi|499516035|ref|WP_011202675.1|helicase[Bacteroidesfragilis]_gi|53713270|ref|YP_099262.1|helicasedomainprotein[Bacteroidesfrag    GCINALSKQI--L---------------------------GDAG--------IIKDSDPE
gi|651338332|ref|WP_026461320.1|helicase[Adhaeribacteraquaticus]/11620    GCVNALSKKM--L---------------------------GDAG--------IMKESDPE
gi|806964642|gb|AKC17139.1|helicase[Flavobacteriumpsychrophilum]/11639    GCINALSKQI--L---------------------------GDAG--------FILSSDPE
gi|752684374|ref|WP_041332322.1|helicase[Flavobacteriumpsychrophilum]_gi|666882889|gb|AIG30715.1|helicase[Flavobacteriumpsychro    GCINALSKQI--L---------------------------GDAG--------FILSSDPE
gi|933196595|emb|CUP04894.1|DNAorRNAhelicasesofsuperfamilyII[Bacteroidesuniformis]/11631    GCINALSKKM--L---------------------------GDEN-------DNIKSTDPQ
gi|670509109|ref|WP_031456918.1|helicase[Flavobacteriumchungangense]/11625    GCINALSKQV--L---------------------------GDEG--------IIKDSDPL
                                                                                                                                    .    :

gi|490190857|ref|WP_004089451.1|hypotheticalprotein[Helicobacterbilis]_gi|476630638|gb|EMZ37196.1|hypotheticalproteinC826_02193    PSKRALNFCR-NIATS--------------KNIKESFQTIIECYDEEMRKK---------
gi|488896133|ref|WP_002807242.1|MULTISPECIES:restrictionendonucleaseEco57I[Campylobacter]_gi|380541096|gb|EIA65377.1|hypothetic    LSRRVINFCK-SIRDS--------------KNISNSFKIIMQCYDEALKKD---------
gi|746602240|ref|WP_039628365.1|restrictionendonucleaseEco57I[Campylobacterlari]_gi|744819395|gb|AJD03391.1|putativehelicase[Ca    ISRRVINFCK-SIRDS--------------KNITNSFKIIMQCYDEALKKD---------
gi|727317135|ref|WP_033772558.1|restrictionendonucleaseEco57I[Helicobacterpylori]/11622    PSKRAISFCK-SIKTS--------------KNIKDSFQTIIECYDEELKKK---------
gi|727103490|ref|WP_033618954.1|restrictionendonucleaseEco57I[Helicobacterpylori]/11616    PSKRAISFCK-SIKTS--------------KNIKDSFQTIIECYDEELKKK---------
gi|727340205|ref|WP_033794408.1|restrictionendonucleaseEco57I[Helicobacterpylori]/11609    PSKRAISFCK-SIKTS--------------KNIKDSFQTIIECYDEELKKK---------
gi|727292407|ref|WP_033749975.1|restrictionendonucleaseEco57I[Helicobacterpylori]/11611    PSKRAISFCK-SIKTS--------------KNIKDSFYTIMECYDEELKKK---------
gi|727289795|ref|WP_033747591.1|restrictionendonucleaseEco57I[Helicobacterpylori]/11612    VSQRAISFCK-SIQTS--------------KNIKDSFQTIMECYDEELKKK---------
gi|727319676|ref|WP_033775047.1|restrictionendonucleaseEco57I[Helicobacterpylori]/11612    PSKRTISFCK-SIKTS--------------KNIKDSFETIMECYNEELKKK---------
gi|447212910|ref|WP_001290166.1|restrictionendonucleaseEco57I[Helicobacterpylori]_gi|308063540|gb|ADO05427.1|hypotheticalprotei    PSQRAISFCK-SINTS--------------KHIKESFETIMECYNEELKKK---------
gi|447212901|ref|WP_001290157.1|restrictionendonucleaseEco57I[Helicobacterpylori]_gi|384557329|gb|AFH97797.1|hypotheticalprotei    PSQRAISFCK-SINTS--------------KHIKESFETIMECYNEELKKK---------
gi|515849671|ref|WP_017280424.1|hypotheticalprotein[Helicobacterpylori]/11615    PSQRAISFCK-SINTS--------------KRIKDSFETIMECYDEELKKK---------
gi|447212900|ref|WP_001290156.1|restrictionendonucleaseEco57I[Helicobacterpylori]_gi|317178970|dbj|BAJ56758.1|TypeIIGrestrictio    PSQRAISFCK-SISTS--------------KRIKESFETIMECYNEELKKK---------
gi|447079018|ref|WP_001156274.1|hypotheticalprotein[Helicobacterpylori]/11614    ASQRAINFCK-SIDTS--------------KRIKDSFETIMECYDEELKKK---------
gi|447212800|ref|WP_001290056.1|hypotheticalprotein[Helicobacterpylori]_gi|532153248|gb|EQL67316.1|restrictionendonucleaseEco57    ASQRAISFCK-SIDTS--------------KRIKDSFETIMECYDEELKKK---------
gi|505457237|ref|WP_015644339.1|hypotheticalprotein[Helicobacterpylori]_gi|499061160|gb|AGL70074.1|restrictionendonucleaseEco57    PSQRAISFCK-SINTS--------------KNIKDSFETIMECYDEELKKK---------
HPYUM037X/11591    PSQRAISFCK-SINTS--------------KNIKDSFETIMECYDEELKKK---------
gi|920026297|ref|WP_052937176.1|restrictionendonuclease[Helicobacterpylori]_gi|912528678|gb|KNX48238.1|restrictionendonucleaseE    PSQRAISFCK-SIQTS--------------KNITDSFETIMECYDEELKKK---------
gi|915539050|ref|WP_050841327.1|restrictionendonuclease[Helicobacterpylori]_gi|908890887|gb|KNE06183.1|restrictionendonucleaseE    PSQRAINFCK-SIQTS--------------KNIKDSFETIMECYDEELKKK---------
gi|727101397|ref|WP_033616957.1|restrictionendonucleaseEco57I[Helicobacterpylori]/11592    PSQRAISFCK-SIQTS--------------KNIKDSFETIMECYDEELKKK---------
gi|562814875|emb|CDK13002.1|TypeISPRMsystem[Helicobacterpylori26695]/11588    PSQRAINFCK-SINTS--------------KNIKDSFETIMECYDEELKKK---------
HPYAXVIII/11588    PSQRAINFCK-SINTS--------------KNIKDSFETIMECYDEELKKK---------
gi|447079029|ref|WP_001156285.1|restrictionendonucleaseEco57I[Helicobacterpylori]_gi|393085956|gb|EJB86635.1|hypotheticalprotei    VSQRAISFCK-SIQTS--------------KNIKDSFETIMECYDEELKKK---------
gi|489105953|ref|WP_003015814.1|restrictionendonuclease[Helicobacterpylori]_gi|477503388|gb|ENH59950.1|restrictionendonucleasef    PSQRAINFCK-SIQTS--------------KNIKDSFETIMECYDEELKKK---------
gi|447079006|ref|WP_001156262.1|restrictionendonucleaseEco57I[Helicobacterpylori]_gi|393067470|gb|EJB68280.1|hypotheticalprotei    PSQRAISFCK-SINTS--------------KNIKDSFETIMECYDEELKKK---------
gi|727314589|ref|WP_033770298.1|restrictionendonucleaseEco57I[Helicobacterpylori]/11586    PSQRAINFCK-SINTS--------------KNIKDSFETIMECYDEELKKK---------
```

```
gi|447079019|ref|WP_001156275.1|restrictionendonucleaseEco57I[Helicobacterpylori]_gi|308065028|gb|ADO06920.1|hypotheticalprotei      VSQRAISFCK-SIQTS--------------KNIKDSFETIMECYDEELKKK---------
gi|447079028|ref|WP_001156284.1|restrictionendonucleaseEco57I[Helicobacterpylori]_gi|393076030|gb|EJB76784.1|hypotheticalprotei      VSQRAISFCK-SIQTS--------------KNIKDSFETIMECYDEELKKK---------
gi|393034009|gb|EJB35070.1|hypotheticalproteinHPNQ4053_0631[HelicobacterpyloriNQ4053]/11514                                          VSQRAISFCK-SIQTS--------------KNIKDSFETIMECYDEELKKK---------
gi|447079004|ref|WP_001156260.1|restrictionendonucleaseEco57I[Helicobacterpylori]_gi|384552418|gb|AFI07366.1|hypotheticalprotei      PSQRAINFCK-SINTS--------------KNIKDSFETIMECYDEELKKK---------
gi|746011306|ref|WP_039088215.1|restrictionendonucleaseEco57I[Helicobacterpylori]_gi|733488720|gb|KHL85979.1|restrictionendonuc      VSQRAISFCK-SIQTS--------------KNIKDSFETIMECYDEELKKK---------
gi|531081162|gb|EQD92166.1|restrictionendonucleaseEco57I[HelicobacterpyloriPZ5080]/11509                                             VSQRAINFCK-SINTS--------------KNIKDSFETIMECYDEELKKK---------
gi|544760796|ref|WP_021187395.1|restrictionendonucleaseEco57I[Helicobacterpylori]_gi|531086508|gb|EQD96745.1|restrictionendonuc      VSQRAISFCK-SIQTS--------------KNIKDSFETIMECYDEELKKK---------
gi|447079012|ref|WP_001156268.1|restrictionendonucleaseEco57I[Helicobacterpylori]_gi|393028199|gb|EJB29286.1|hypotheticalprotei      VSQRAISFCK-SIQTS--------------KNITDSFETIMECYDEELKKK---------
gi|746008458|ref|WP_039085555.1|restrictionendonucleaseEco57I[Helicobacterpylori]_gi|733480358|gb|KHL78026.1|restrictionendonuc      VSQRAISFCK-SIQTS--------------KNIKDSFETIMECYDEELKKK---------
gi|447079011|ref|WP_001156267.1|restrictionendonucleaseEco57I[Helicobacterpylori]_gi|393038534|gb|EJB39568.1|hypotheticalprotei      VSQRAISFCK-SIQTS--------------KNIKDSFETIMECYDEELKKK---------
gi|393037163|gb|EJB38199.1|hypotheticalproteinHPNQ4161_0422[HelicobacterpyloriNQ4161]/11556                                          VSQRAISFCK-SIQTS--------------KNIKDSFETIMECYDEELKKK---------
gi|447079007|ref|WP_001156263.1|restrictionendonucleaseEco57I[Helicobacterpylori]_gi|393051817|gb|EJB52768.1|hypotheticalprotei      PSQRAISFCK-SIQTS--------------KNIKDSFETIIECYDEELKKK---------
gi|487867094|ref|WP_001940560.1|helicase[Helicobacterpylori]_gi|459505657|gb|EMG98418.1|helicaseprotein[HelicobacterpyloriGAM21      VSQRAISFCK-SIQTS--------------KNITDSFQTIMECYDEELKKK---------
gi|487872094|ref|WP_001945560.1|helicase[Helicobacterpylori]_gi|459512104|gb|EMH04523.1|helicaseprotein[HelicobacterpyloriGAM24      VSQRAISFCK-SIQTS--------------KNIKDSFETIMECYDEELKKK---------
gi|487880228|ref|WP_001953694.1|helicase[Helicobacterpylori]_gi|459529131|gb|EMH20734.1|helicaseprotein[HelicobacterpyloriGAM26      VSQRAISFCK-SIQTS--------------KNIKDSFETIMECYDEELKKK---------
gi|487882593|ref|WP_001956059.1|helicase[Helicobacterpylori]_gi|459533462|gb|EMH24859.1|helicaseprotein[HelicobacterpyloriGAM26      VSQRAISFCK-SIQTS--------------KNIKDSFETIMECYDEELKKK---------
gi|487845430|ref|WP_001918896.1|helicase[Helicobacterpylori]_gi|410713871|gb|EKQ71363.1|helicaseprotein[HelicobacterpyloriGAM10      VSQRAISFCK-SIQTS--------------KNITDSFQTIMECYDEELKKK---------
gi|447079005|ref|WP_001156261.1|restrictionendonucleaseEco57I[Helicobacterpylori]_gi|393069830|gb|EJB70625.1|hypotheticalprotei      VSQRAISFCK-SIQTS--------------KNIKDSFETIMECYDEELKKK---------
gi|459506808|gb|EMG99528.1|helicaseprotein[HelicobacterpyloriGAM121Aii]/11545                                                        VSQRAISFCK-SIQTS--------------KNIKDSFETIMECYDEELKKK---------
gi|487825283|ref|WP_001898749.1|restrictionendonucleaseEco57I[Helicobacterpylori]_gi|393107526|gb|EJC08068.1|hypotheticalprotei      VSQRAISFCK-SIQTS--------------KNIKDSFETIMECYDEELKKK---------
gi|447079008|ref|WP_001156264.1|restrictionendonucleaseEco57I[Helicobacterpylori]_gi|393058636|gb|EJB59524.1|hypotheticalprotei      VSQRAISFCK-SIQTS--------------KNIKDSFETIMECYDEELKKK---------
gi|447079015|ref|WP_001156271.1|restrictionendonucleaseEco57I[Helicobacterpylori]_gi|307637357|gb|ADN79807.1|hypotheticalprotei      VSQRAISFCK-SIQTS--------------KNIKDSFETIMECYDEELKKK---------
gi|487884861|ref|WP_001958327.1|helicase[Helicobacterpylori]_gi|459535535|gb|EMH26834.1|helicaseprotein[HelicobacterpyloriGAM26      VSQRAISFCK-SIQTS--------------KNIKDSFETIMECYDEELKKK---------
gi|487886365|ref|WP_001959831.1|helicase[Helicobacterpylori]_gi|459538727|gb|EMH29861.1|helicaseprotein[HelicobacterpyloriGAM27      VSQRAISFCK-SIQTS--------------KNIKDSFETIMECYDEELKKK---------
gi|545301637|ref|WP_021581412.1|helicase[Helicobacterpylori]_gi|535931889|gb|ERA61745.1|helicaseprotein[HelicobacterpyloriGAM11      VSQRAISFCK-SIQTS--------------KNIKDSFETIMECYDEELKKK---------
gi|447079010|ref|WP_001156266.1|restrictionendonucleaseEco57I[Helicobacterpylori]_gi|393095477|gb|EJB96081.1|hypotheticalprotei      VSQRAISFCK-SIQTS--------------KNIKDSFETIMECYDEELKKK---------
gi|447079009|ref|WP_001156265.1|restrictionendonucleaseEco57I[Helicobacterpylori]_gi|393056294|gb|EJB57206.1|hypotheticalprotei      VSQRAISFCK-SIQTS--------------KNIKDSFQTIMECYDEELKKK---------
gi|471386985|gb|EMR58906.1|restrictionendonucleasefamilyprotein[HelicobacterpyloriHpH1]/11504                                        VSQRAISFCK-SIQTS--------------KNIKDSFETIMECYDEELKKK---------
gi|487875679|ref|WP_001949145.1|helicase[Helicobacterpylori]_gi|459516928|gb|EMH09091.1|helicaseprotein[HelicobacterpyloriGAM25      VSQRAISFCK-SIQTS--------------KNIKDSFETIMECYDEELKKK---------
gi|496130066|ref|WP_008854573.1|DNAdamageinducibleprotein[Commensalibacterintestini]_gi|353671689|gb|EHD13391.1|helicasedomain/      PMQRAVAFCQ-VIESTY----KGKIHKVSSKLIAEMFGSVVEQYQAYETKELLKE-----
gi|924898894|ref|WP_053556546.1|damageinducibleprotein[Sphingopyxissp.113P3]_gi|924523996|gb|ALC14732.1|damageinducibleprotei/1      PMRRAIAFAK-SIA--------------ASKTVAAEFGRVAADWRASLEAAAPK------
gi|780802950|gb|KJS08562.1|damageinducibleprotein[Hoefleasp.BRH_c9]/11633                                                           PMKRALAFCR-DIK--------------SSKLITNEFTKVIDEYLDSANIMDGD------
gi|944671432|gb|KQN22886.1|damageinducibleprotein[Sphingomonassp.Leaf32]/11415                                                      HMGRALAFCR-DIA--------------SSKLVRDEFAAVVDEYLASVAN----------
gi|944612953|gb|KQM64772.1|damageinducibleprotein[Sphingomonassp.Leaf16]/11726                                                      HMGRALAFCR-DIA--------------SSKLVRDEFAAVVDEYLASVAN----------
gi|764353851|ref|WP_044331468.1|damageinducibleprotein[Sphingomonashengshuiensis]_gi|761897338|gb|AJP71707.1|damageinduciblep/1      PMKRALAFCK-DIA--------------TSKLVQSEFAAVIDEYLASDEGKEA-------
gi|945874472|gb|KQZ27973.1|damageinducibleprotein[Mesorhizobiumsp.Root552]/11651                                                    PMKRALAFCR-DIA--------------TSELIEEEFAAVVSEYLASDEGKQA-------
gi|759408081|ref|WP_043132403.1|damageinducibleprotein[Paracoccussanguinis]_gi|694217086|gb|KGJ14281.1|damageinducibleprotein/1      PMRRALAFCQ-SIA--------------KSKIIEDEFTKVVDEYTGSDLIE---------
gi|654342671|ref|WP_027836116.1|damageinducibleprotein[Maritaleamyrionectae]/11632                                                  PMRRALAFCQ-SIE--------------KSKIIEDEFAQVADEYATNELVN---------
gi|126717687|gb|EBA14409.1|hypotheticalproteinRSK20926_02032[Roseobactersp.SK20926]/11603                                           PMKRALAFCQ-SIK--------------KSTIIEEEFTQVVDEYIGNELID---------
gi|765301116|ref|WP_044628780.1|damageinducibleprotein[Halomonasmeridiana]_gi|764089679|gb|KJD19875.1|damageinducibleprotein[/1      PMRRALAFCQ-SIA--------------KSKIIEDEFANVVEEYTGSELTE---------
gi|518053006|ref|WP_019223214.1|DNAdamageinducibleprotein[Bartonellarattaustraliani]/11654                                          PMHTALAFCK-DID--------------TSKYVRNAFNKVIKSH----IND---------
gi|493637806|ref|WP_006589578.1|DNAdamageinducibleprotein[Bartonellabirtlesii]_gi|395410345|gb|EJF76900.1|hypotheticalproteinM/      PMRCALAFCK-DIN--------------TSRYITKLFNKIIKSH----IND---------
gi|501188603|ref|WP_012231622.1|DNAdamageinducibleprotein[Bartonellatribocorum]_gi|161017843|emb|CAK01401.1|helicase/methyltra/      PMQCALAFCK-DIN--------------TSQYITKLFNKIIKSH----IDD---------
gi|651388616|ref|WP_026500615.1|damageinducibleprotein[Bartonellagrahamii]/11655                                                    PMHSALAFCK-DIN--------------TSQYITKLFNKIIKSH----IDD---------
gi|501188669|ref|WP_012231687.1|DNAdamageinducibleprotein[Bartonellatribocorum]_gi|161017922|emb|CAK01480.1|helicase/methyltra/      PMQCALAFCK-DIN--------------TSQYITKLFNKIIKSH----IDD---------
gi|518048832|ref|WP_019219040.1|DNAdamageinducibleprotein[Bartonellaflorencae]/11655                                                PMRRSLAFCK-DIK--------------TSERVSEIFNDAIDEY----HNF---------
gi|518053617|ref|WP_019223825.1|DNAdamageinducibleprotein[Bartonellarattaustraliani]/11607                                          PMRRSLAFCK-DIN--------------TSQRVSEIFNDAIDEY----HKF---------
gi|506336449|ref|WP_015856168.1|DNAdamageinducibleprotein[Bartonellagrahamii]_gi|240267492|gb|ACS51080.1|helicase/methyltransf/      PMRRSLAFCK-DIK--------------TSERVSEIFNDAIDEY----HNF---------
gi|494588832|ref|WP_007347427.1|DNAdamageinducibleprotein[Bartonellarattimassiliensis]_gi|395419053|gb|EJF85361.1|hypothetical/      PMRRSLAFCK-NIN--------------TSQLVSELFNDAIDEY----HKF---------
gi|501188650|ref|WP_012231668.1|DNAdamageinducibleprotein[Bartonellatribocorum]_gi|161017902|emb|CAK01460.1|helicase/methyltra/      PMRRSLAFCK-NIK--------------TSQLVSELFNDAIDEY----HKF---------
gi|501188624|ref|WP_012231643.1|DNAdamageinducibleprotein[Bartonellatribocorum]_gi|161017866|emb|CAK01424.1|helicase/methyltra/      PMRRALAFCK-DIK--------------TSKQIRDTFQGKGVKRIFNRLYQ---------
gi|518053856|ref|WP_019224064.1|DNAdamageinducibleprotein[Bartonellarattaustraliani]/11625                                          PMRRALAFCK-DIK--------------TSKRIRDTFQGKGVKRIFNRLYQ---------
gi|501188053|ref|WP_012231081.1|DNAdamageinducibleprotein[Bartonellatribocorum]_gi|161017434|emb|CAK00992.1|helicase/methyltra/      PMRRALAFCK-DIK--------------TSKNIRDTFQGKGVKRIFNRLYQ---------
gi|651388630|ref|WP_026500626.1|damageinducibleprotein[Bartonellagrahamii]/11657                                                    PMRRALAFCK-DID--------------TSKRICKRFKKEKVQESLRALHK---------
gi|518048855|ref|WP_019219063.1|DNAdamageinducibleprotein[Bartonellaflorencae]/11658                                                PMRRALAFCK-DID--------------TSKRICKRFKKEKVQESLRALHK---------
gi|490995101|ref|WP_004856829.1|DNAdamageinducibleprotein[Bartonelladoshiae]_gi|395413167|gb|EJF79645.1|hypotheticalproteinMCS/      PMRRALAFCK-DID--------------TSKRICKRFKKEKVQESLRALHK---------
gi|491002558|ref|WP_004864279.1|DNAdamageinducibleprotein[Bartonellavinsonii]_gi|395421392|gb|EJF87643.1|hypotheticalproteinME/      PMRRALAFCK-DIK--------------TSERIRDTFNSDEIQSELRTLYE---------
gi|491003373|ref|WP_004865094.1|DNAdamageinducibleprotein[Bartonellavinsonii]_gi|395432864|gb|EJF98838.1|hypotheticalproteinME/      PMRRALAFCK-DIK--------------TSERIRDTFNSDEIQDELRTLYE---------
gi|491002426|ref|WP_004864148.1|DNAdamageinducibleprotein[Bartonellavinsonii]_gi|395421767|gb|EJF88003.1|hypotheticalproteinME/      PMRRALAFCK-DIE--------------TSERICKAFNSDKTKERLHALYE---------
gi|491004024|ref|WP_004865745.1|DNAdamageinducibleprotein[Bartonellavinsonii]_gi|395432542|gb|EJF98522.1|hypotheticalproteinME/      PMRRALAFCK-DIE--------------TSERICRAFNSDKTKERLHALYE---------
gi|648396442|ref|WP_026088193.1|damageinducibleprotein[Bartonellarattaustraliani]/11593                                             PMRHALAFCR-DIE--------------SSKRICRTFNSQEIQDELYNLHK---------
gi|748190174|ref|WP_039761290.1|damageinducibleprotein[Bartonellaqueenslandensis]/11658                                             PMRHALAFCR-DIE--------------SSKRICRTFNSQEIQDELYNLHK---------
gi|494817599|ref|WP_007553007.1|DNAdamageinducibleprotein[Bartonellasp.DB56]_gi|395411312|gb|EJF77837.1|hypotheticalproteinMC/1      PMHRALAFCR-DID--------------TSERICRTFNDEEVKKDLLNLHK---------
gi|515763376|ref|WP_017195976.1|DNAdamageinducibleprotein[Bartonellabirtlesii]/11656                                                PMHRALAFCR-NIK--------------TSERIRDTFNDEEVKKDLLDLHE---------
gi|494589420|ref|WP_007348014.1|DNAdamageinducibleprotein[Bartonellarattimassiliensis]_gi|395416278|gb|EJF82667.1|hypothetical/      PMRRALAFCK-DIK--------------TSERIRDTFNSEEIQEELFNLHK---------
gi|506336469|ref|WP_015856188.1|DNAdamageinducibleprotein[Bartonellagrahamii]_gi|240267512|gb|ACS51100.1|helicase/methyltransf/      PMRRALAFCK-DIK--------------TSKRIRKTFNSEEIQEELFNLHQ---------
gi|749308087|ref|WP_040132227.1|damageinducibleprotein[Bartonellatribocorum]_gi|605051878|emb|CDO49988.1|helicase/methyltransf/      PMRRALAFCK-DIK--------------TSKRICKTFNSEEIQKELFNLHK---------
```

gi|515763800|ref|WP_017196400.1|DNAdamageinducibleprotein[Bartonellabirtlesii]/11658	PMRRALAFCR-DIK--------------TSERIRDTFNSPEIQKELSDLHK---------
gi|492186994|ref|WP_005773380.1|DNAdamageinducibleprotein[Bartonellaelizabethae]_gi|395418361|gb|EJF84688.1|hypotheticalprotei/	PMRRALAFCK-DIK--------------TSERIRDTFNSQNIQKELSGIHK---------
gi|501188615|ref|WP_012231634.1|DNAdamageinducibleprotein[Bartonellatribocorum]_gi|161017856|emb|CAK01414.1|helicase/methyltra/	PMRRALAFCK-DIK--------------TSERIRDTFNSQEIQKELYNLHK---------
gi|501187470|ref|WP_012230499.1|DNAdamageinducibleprotein[Bartonellatribocorum]_gi|161017090|emb|CAK00648.1|helicase/methyltra/	PMRRALAFCK-DIK--------------TSERIRDTFNSQEIQKELYNLHK---------
gi|501188636|ref|WP_012231655.1|DNAdamageinducibleprotein[Bartonellatribocorum]_gi|161017879|emb|CAK01437.1|helicase/methyltra/	PMRRALAFCK-DIK--------------TSERIRDTFNSQEIQKELYNLHK---------
gi|930151941|emb|CTQ34818.1|UvsWhelicase[Jannaschiarubra]/11641	AMRRALIFCR-DIK--------------SSRIIEDEFAAVAEEFRSQNP-----------
gi|928977106|ref|WP_054001461.1|damageinducibleprotein[Shimiasp.SK013]_gi|927698852|gb|KPA22698.1|typeIrestrictionenzymeEcoKIs/	PMRRAIAFCK-DIR--------------SSKLIEREFGAVVDEFNGTTVLDDD-------
gi|738872465|ref|WP_036760884.1|damageinducibleprotein[Leisingeradaeponensis]/11643	PMRRALAFCR-DIK--------------SSQLVEREFSAVVSDFLGDDEVIETE------
gi|518050881|ref|WP_019221089.1|DNAdamageinducibleprotein[Bartonellasenegalensis]/11652	PIQRALAFCK-DIE--------------SSKLVRDEFSVVVKEYLEYINKENT-------
gi|493980596|ref|WP_006923523.1|DNAdamageinducibleprotein[Bartonellawashoensis]_gi|395413322|gb|EJF79796.1|hypotheticalprotein/	PMHRALAFCK-TIE--------------NSKLVRNEFSAVVKEYLDYINKENT-------
gi|493983106|ref|WP_006925984.1|DNAdamageinducibleprotein[Bartonellawashoensis]_gi|395418127|gb|EJF84455.1|hypotheticalprotein/	PMHRALAFCK-TIE--------------SSKLVRDEFSAVVREYLDYINKENT-------
gi|499494671|ref|WP_011181311.1|DNAdamageinducibleprotein[Bartonellahenselae]_gi|49239008|emb|CAF28308.1|hypotheticalproteinBH/	PMHRALAFCK-DIR--------------SSKLVRDEFSAVVKEYLDYTNREDT-------
gi|651388426|ref|WP_026500441.1|damageinducibleprotein[Bartonellagrahamii]/11652	PMRRALAFCK-TIE--------------SSKLVCGEFSAVVREYLDYTK-ENT-------
gi|506337280|ref|WP_015856999.1|DNAdamageinducibleprotein[Bartonellagrahamii]_gi|240268402|gb|ACS51990.1|helicase/methyltransf/	PMRRALAFCK-TIE--------------SSKLVCGEFSAVVREYLDYTK-ENT-------
gi|494817535|ref|WP_007552943.1|DNAdamageinducibleprotein[Bartonellasp.DB56]_gi|395412002|gb|EJF78518.1|hypotheticalproteinMC/1	PMHRALAFCR-TIE--------------SSKLVRDEFSAVVREYLEYTK-ENT-------
gi|493638099|ref|WP_006589864.1|DNAdamageinducibleprotein[Bartonellabirtlesii]_gi|395409809|gb|EJF76394.1|hypotheticalproteinM/	PMHRALAFCK-TIQ--------------SSKVVRDEFFAVVKEYLEYTK-KNT-------
gi|748189589|ref|WP_039760707.1|damageinducibleprotein[Bartonellaqueenslandensis]/11651	PMHRALAFCK-TIE--------------SSKLVRREFSAVVEEYLEYTQ-GNA-------
gi|501189483|ref|WP_012232501.1|DNAdamageinducibleprotein[Bartonellatribocorum]_gi|161018902|emb|CAK02460.1|helicase/methyltra/	PMHRALAFCK-DIR--------------SSKLVRDEFSAVVEEYLEYTQ-ENT-------
gi|518049181|ref|WP_019219389.1|DNAdamageinducibleprotein[Bartonellaflorencae]/11652	PMQRALAFCK-DIR--------------SSKLVRDEFSAVVEEYLEYTQ-ENA-------
gi|492193255|ref|WP_005774528.1|DNAdamageinducibleprotein[Bartonellaelizabethae]_gi|395416403|gb|EJF82779.1|hypotheticalprotei/	PMHRALAFCK-DIR--------------SSKLVRDEFSAVVEEYLEYTQ-GNT-------
gi|498387325|ref|WP_010701288.1|helicase/methyltransferase[Bartonellabovis]_gi|478418724|gb|ENN92426.1|helicase/methyltransfera	PMQRVLAFCK-DIK--------------SSELLRDEFSAVVEEYFDYINKKDT-------
gi|319408697|emb|CBI82352.1|conservedhypotheticalprotein[BartonellaschoenbuchensisR1]/11661	PMQRVLTFCK-DIE--------------SSKLLRDEFSAVVEEYLDYINKENT-------
gi|494741726|ref|WP_007477134.1|DNAdamageinducibleprotein[Bartonellamelophagi]_gi|395423925|gb|EJF90113.1|hypotheticalproteinM/	PMQRVLAFCK-DIK--------------SSELLRDEFSAVVEEYLDYINKKNT-------
gi|737261405|ref|WP_035245349.1|damageinducibleprotein[Alcanivoraxjadensis]_gi|686989709|gb|KGD62556.1|hypotheticalproteinT9A_/	PMRRALAFCR-DIR--------------SSKLIQSQFAEVVEEYVNYHSEED--------
gi|916687564|ref|WP_051294655.1|damageinducibleprotein[Gemmobacternectariphilus]/11641	PMRRALAFCK-DIR--------------SSKLVRDEFSAVVDEFLGVDPVLEEE------
gi|740294127|ref|WP_038132495.1|damageinducibleprotein[Thioclavasp.DT234]_gi|660524826|gb|KEO54689.1|hypotheticalproteinDT23_/1	PMRRALAFCK-SIA--------------ASKLIRDEFTQVVAEYTGDDALIEDD------
gi|568331062|ref|WP_024099615.1|helicase[Phaeobactergallaeciensis]_gi|568232121|gb|AHD12007.1|putativehelicase[Phaeobactergalla	PMRRALAFCK-DIR--------------SSKLIRDEFSAVVDEFLGDDPILDDI------
gi|518810459|ref|WP_019966413.1|DNAdamageinducibleprotein[Pannonibacterphragmitetus]/11635	PMRRALAFAK-DIR--------------SSKLIRDEFTAVVDEYLGQDSLIDDD------
gi|495305760|ref|WP_008030510.1|DNAdamageinducibleprotein[Rhodobactersp.SW2]_gi|259021698|gb|EEW25000.1|typeIIIrestrictionprot/	PMRRALAFAK-DIR--------------SSKLIRDEFTAVVDEYLGQDSLIDDD------
gi|506403303|ref|WP_015923022.1|DNAdamageinducibleprotein[Rhodobactersphaeroides]_gi|221162951|gb|ACM03922.1|HypotheticalProte/	PMRRALAFAR-DIR--------------SSKLIRDEFTAVVDEYLGQDSLIDDD------
gi|738818386|ref|WP_036708790.1|damageinducibleprotein[Paracoccussanguinis]_gi|694226786|gb|KGJ22541.1|damageinducibleprotein/1	PMRRALAFAR-DIR--------------SSKLIRDEFTAVVDEYLGQDSLIDDD------
gi|746096057|ref|WP_039162453.1|damageinducibleprotein[Gallibacteriumanatis]_gi|703617775|gb|KGQ63868.1|damageinducibleprotei/1	PMKRAVAFCQ-VIEKDY----RGSKHKVSSKAIAEMFDTVVTAYQEQEIEALNAI-----
gi|737716785|ref|WP_035685631.1|damageinducibleprotein[Avibacteriumparagallinarum]/11643	PMKRAVAFCQ-VIEKDY----RGSKHKVSSKAIAEMFDTVVTAYQEQEIEALKAI-----
gi|540072553|gb|ERF78672.1|hypotheticalproteinN561_04930[Gallibacteriumanatis12656/12]/11651	PMKRAVAFCQ-VIEKDY----RGSKHKVSSKAIAEMFDTVVTAYQEQEIEALNEI-----
gi|917509519|ref|WP_052115936.1|damageinducibleprotein[Gallibacteriumanatis]_gi|702414161|gb|KGQ42233.1|damageinducibleprotei/1	PMKRAVAFCQ-VIEKDY----RGSKHKVSSKAIAEMFDTVVTAYQEQEIEALNEI-----
gi|746082942|ref|WP_039150268.1|damageinducibleprotein[Gallibacteriumanatis]_gi|703617078|gb|KGQ63194.1|damageinducibleprotei/1	PMKRAVAFCQ-VIEKDY----KGSKHKVSSKAIAEMFDTVVTAYQEQEIEALNEI-----
gi|822522885|ref|WP_046952840.1|damageinducibleprotein[Haemophilushaemolyticus]_gi|821501762|gb|KKZ59267.1|damageinduciblepro/1	PMKRAVAFCQ-VIEKEY----KGKTHKVSSKNIAEIFSNVVDAYQKQEIAALEEQ-----
gi|404381014|gb|EJZ77501.1|putativehelicase[Pasteurellamultocidasubsp.gallicidaP1059]/11681	PMKRAVAFCQ-VIEKEY----KGKTHKVSSKAISEIFANVVDAYQMQEIEALKAQ-----
gi|298281660|gb|EFI23149.1|helicaseCterminaldomainprotein[Neisseriasp.oraltaxon014str.F0314]/11493	PMRRAVAFCQ-VIEKEY----KGSRHKVSSKLIADEFSKVVKKYQQKETEEWLKD-----
gi|348013055|gb|EGY51980.1|helicasedomainprotein[Neisseriashayeganii871]/11481	PMQRAVAFCQ-VIEKEY----KGGKHKVSSKLIAESFEAVVAAYQQSERAVRLKE-----
gi|496407140|ref|WP_009116004.1|DNAdamageinducibleprotein[Neisseriawadsworthii]_gi|349794756|gb|EGZ48566.1|helicasedomainprote/	PMRRAVAFCQ-VIEKEY----KGSKHKVSSKLIAEMFQAVIEEYQQAESKALLEH-----
gi|517216441|ref|WP_018405259.1|DNAdamageinducibleprotein[Marinobacterlipolyticus]/11656	PMKRAVAFCQ-VIDKEY----KGKNHKVSSKLISEMFGAVVAQYQEAEIASIREK-----
gi|738230264|ref|WP_036185691.1|damageinducibleprotein[Marinobactersp.ELB17]/11671	PMKRAVAFCQ-VIDKEY----KGKNHKVSSKLISEMFGAVVAQYQEAEIASIREK-----
gi|447197741|ref|WP_001274997.1|DNAdamageinducibleprotein[Acinetobacterbaumannii]_gi|408512324|gb|EKK13969.1|typeIIIrestrictio/	PMKRAVAFCQ-VIEKEY----KGKKHKVSSKQITEMFSAVVEAYQEAEIDHLYRT-----
gi|820775897|ref|WP_046739909.1|damageinducibleprotein[Acinetobactersp.AG1]_gi|819909758|gb|KKW75938.1|damageinducibleprotein/1	PMERAVAFCQ-VIEKEY----KGKKHKVSSIQIRDMFGPVIEAYKEAEMDHLYQT-----
gi|584595163|ref|WP_024160846.1|helicase[Acinetobacterpittii]_gi|576702530|emb|CDI28049.1|helicasedomaincontainingprotein[Acin/	PMERAVAFCQ-VIEKEY----KGKKHKVSSKQITEMFSAVVEAYQQAEMDHLYRT-----
gi|514963042|ref|WP_016651366.1|hypotheticalprotein[Acinetobactersp.NIPH2036]_gi|514162665|gb|EPG41391.1|hypotheticalproteinF90	PMERAVAFCQ-VIEKEY----KGKKHKVSSKQITEMFSAVVEAYQQAEMDHLYRT-----
gi|515965564|ref|WP_017396147.1|DNAdamageinducibleprotein[Acinetobacterhaemolyticus]/11667	PMERAVAFCQ-VIEKEY----KGKKHKVSSKQITEMFSAVVEAYQQAEMDHLYRT-----
gi|491346810|ref|WP_005204747.1|MULTISPECIES:hypotheticalprotein[Acinetobacter]_gi|480335823|gb|ENX56033.1|hypotheticalproteinF	PMERAVAFCQ-VIEKEY----KGKKHKVSSKQITEMFSAVVEAYQQAEMDHLYRT-----
gi|693044472|ref|WP_032215268.1|damageinducibleprotein[Escherichiacoli]_gi|607725731|gb|EZE08230.1|damageinducibleprotein[Esc/1	AMKRAVAFCQ-VIEKDF----RGKTHKVSSKLITEMFGAVVEAYQASEIEHLRET-----
gi|742382177|ref|WP_038861304.1|damageinducibleprotein[Cronobactermuytjensii]_gi|924333747|gb|ALB69766.1|damageinducibleprote/1	AMKRAVAFCQ-VIEKDF----RGKTHKVSSKLITDMFGAVVAEYQKSEIEHIRET-----
gi|915912804|ref|WP_050927035.1|damageinducibleprotein[Yersiniaenterocolitica]_gi|899805802|emb|CQH32042.1|typeIrestrictionenz/	AMKRAVAFCQ-VIEE-S----KGKVHKVSSKLITEMFGAVVEAYQASEIEYLRET-----
gi|921111327|gb|KON62781.1|typeIrestrictionenzymeEcoKIsubunitR[Komagataeibactereuropaeus]/11492	PMKRAVAFCQ-VISPDY----KGRTPKVSSIQIADMFQQVVEEYQEQEDIEPE-------
gi|582024278|gb|AHI27615.1|DNAhelicaserestrictionenzymeTypeIIIRsubunit[KomagataeibacterxylinusE25]/11636	PMKRAVAFCQ-VISPGY----RGRTPKVSSIQIADMFQQVVEEYQEQEDIEPE-------
gi|347662673|dbj|BAK86128.1|DNAhelicaserestrictionenzymeTypeIIIRsubunit[KomagataeibactermedellinensisNBRC3288]/11737	PMKRAVAFCQ-VISPDY----KGRTPKVSSIQIADMFQQVVEEYQEQEGIEPE-------
gi|917445053|ref|WP_052051471.1|damageinducibleprotein[Acetobactertropicalis]/11429	PMKRAVAFCQ-VISPDY----KGRAHKVSSIQIADMFQKVVEEYQKGDGIEPE-------
gi|453329445|dbj|GAC88472.1|DNAhelicaserestrictionenzymetypeIIIRsubunit[GluconobacterthailandicusNBRC3255]_gi|530287981|dbj|GAD	PMKRAVAFCQ-VISPDY----KGRAHKVSSIQIADMFQKVVEEYQLQDNIEPE-------
gi|889770922|ref|WP_048844278.1|damageinducibleprotein[Gluconobacterfrateurii]/11419	PMKRAVAFCQ-VISPDY----KGRAHKVSSIQIADMFQKVVEEYQRQDGIEPE-------
gi|504886208|ref|WP_015073310.1|hypotheticalprotein[Gluconobacteroxydans]_gi|411027954|gb|AFW01209.1|hypotheticalproteinB932_16	PMKRAVAFCQ-VISPDY----KGRAHKVSSIQIADMFQKVVEEYQRQDGIEPE-------
gi|480040211|gb|ENV79751.1|hypotheticalproteinF942_01641[AcinetobacterursingiiANC3649]/11432	PMQRAVAFCQ-VIEPHT-SKGKSKQHKVSSKNIAAMFQAVVEQYQTYEIQHNPQ------
gi|822498222|ref|WP_046936504.1|damageinducibleprotein[Xanthomonasgardneri]_gi|821751224|gb|KLB07223.1|damageinducibleprotein/1	PMKRAVAFCQ-VIEPGK----SGKAHKVSSKEIAGMFQQVVEAYQEAGDIEDA-------
gi|654059559|ref|WP_027705556.1|damageinducibleprotein[Zymobacterpalmae]/11635	HMKRAVAFCQ-VIEAQY----AGSAHKVSSKHIAEMFKAVVEAYQEAEEIEAT-------
gi|643535620|ref|WP_025224315.1|damageinducibleprotein[Methylophilaceaebacterium11]_gi|577704400|gb|EUJ10546.1|putativehelicas/	PMRRAVAFCQ-VIEPSI----KATSHKVSSKNIASMFAKVVEAYQEQEETFDSN------
MBA11I/11635	PMRRAVAFCQ-VIEPSI----KATSHKVSSKNIASMFAKVVEAYQEQEETFDSN------
gi|655355857|ref|WP_028762287.1|damageinducibleprotein[Shewanellacolwelliana]/11632	PMQKAVAFCQ-VIEPQSTSKRKSAKHKVSSKNIASMFQSVVEAYQEASDIE---------
gi|753204236|ref|WP_041510579.1|damageinducibleprotein[Shewanellasp.cp20]_gi|752457765|gb|KIO36326.1|damageinducibleprotein[S/1	PMKRAVAFCQ-VIERNT-----GKTHKVSSKQIAEMFQAVVESYQEHENDA---------
KPNNIH30III/11619	PMKRAVAFCQ-VIEPST-----GTKHKVSSKQIAEMFQAVVEAYQEHESDA---------

gi|749295994|ref|WP_040120352.1|damageinducibleprotein[Klebsiellapneumoniae]_gi|723249300|gb|AIX81926.1|damageinducibleprotei/1	PMKRAVAFCQ-VIEPST-----GTKHKVSSKQIAEMFQAVVEAYQEHESDA---------
gi|641639739|emb|CDQ54010.1|unnamedproteinproduct[Klebsiellapneumoniae]/11641	PMKRAVAFCQ-VIEAST-----GTKHKVSSKQIAEMFQAVVEAYQEHESDA---------
gi|490797922|ref|WP_004660060.1|hypotheticalprotein[Acinetobactersp.NIPH236]_gi|479876779|gb|ENU27973.1|hypotheticalproteinF992	PMQRALAFCQ-VIDQTA----KARKHQVSSNRIASIFQTVVEAYQKAEAKE---------
gi|916750926|ref|WP_051357982.1|damageinducibleprotein[Acinetobactertjernbergiae]/11453	PMQRALAFCQ-VIDQSE----KARKHQVSSTRIANIFQTVVEAYQNAEIKE---------
gi|691098662|ref|WP_032025060.1|damageinducibleprotein[Acinetobacterbaumannii]_gi|593567521|gb|EXR50735.1|restrictionendonucle/	PMQRALAFCQ-VIEQSE----KARKHQVSSTRIANIFQTVVEAYQEAEAKE---------
gi|736669405|ref|WP_034676049.1|damageinducibleprotein[Acinetobacterbaumannii]/11643	PMQRALAFCQ-VIDQTE----KARKHQVSSTRIADIFHKVVEAYQKAEDKE---------
gi|491368928|ref|WP_005226826.1|hypotheticalprotein[Acinetobactersp.NIPH1847]_gi|480173259|gb|ENX09797.1|hypotheticalproteinF89	PMQRALAFCQ-VIDQSE----KARKHQVSSTRIANIFQTVVEAYQDAEVKE---------
gi|796853993|ref|WP_045795953.1|damageinducibleprotein[Acinetobacterindicus]_gi|787929761|gb|KJV44219.1|damageinducibleprotei/1	PMQRALAFCQ-VIDQTA----KARKHQVSSTRIANIFQTVVEAYQEAEALE---------
gi|295797463|gb|ADG32253.1|typeIIIrestrictionproteinressubunit[ThiomonasintermediaK12]/11676	PMKRAVAFCQ-VIEVQK----GGKTHKVSSKQIKAMFQAVVEAYQEQEHASDDR------
gi|499871179|ref|WP_011551913.1|DNAdamageinducibleprotein[Myxococcusxanthus]_gi|108467544|gb|ABF92729.1|putativerestriction/mo/	PMKRAVAFCQ-VIERPT----GAKVHKVGSKNIADMFQAVVEAYQQSEPA--DG------
gi|495313852|ref|WP_008038600.1|DNAdamageinducibleprotein[Bartonellatamiae]_gi|395424181|gb|EJF90368.1|hypotheticalproteinME5_/	PMQRAVAFCQ-VIDRKK-------SNKVGSLQIANMFQDVVKEYQRTATD--E-------
gi|501453335|ref|WP_012476784.1|DNAdamageinducibleprotein[Leptospirabiflexa]_gi|167777824|gb|ABZ96123.1|Helicase[Leptospirabif/	SMKRAVAFCQ-VINYQA----GAKTHKVSSKVIANMFAEVVRAYQEKE------------
gi|738104613|ref|WP_036063193.1|damageinducibleprotein[Leptospiranoguchii]/11652	PMRRAVSFAQ-VIEYNK----GAKTHKVSSKVIASMFQTVVDAYLEHE------------
gi|410001039|gb|EKO51661.1|typeIIIrestrictionenzyme,ressubunit[Leptospirakirschneristr.200802841]/11651	PMRRAVSFAQ-VIEYNK----GAKTHKVSSKVIASMFQTVVDAYLEQE------------
gi|490901851|ref|WP_004763774.1|typeIIIrestrictionenzyme,ressubunit[Leptospirakirschneri]_gi|464209249|gb|EMN24014.1|typeIIIres	PMRRAVSFAQ-VIEYNK----GAKTHKVSSKVIASMFQTVVDAYLEQE------------
gi|464398131|gb|EMO65902.1|typeIIIrestrictionenzyme,ressubunit[Leptospirakirschneristr.200803703]/11648	PMRRAVSFAQ-VIEYNK----GAKTHKVSSKVIASMFQTVVDAYLEQE------------
gi|945029024|gb|KQQ84900.1|damageinducibleprotein[Xanthomonassp.Leaf131]/11423	PMKRAVAFCQ-VIEPGK----GGKTHKVSSKEISGMFQAVVEAYQEA-------------
gi|896511314|ref|WP_049418183.1|damageinducibleprotein[Stenotrophomonasmaltophilia]/11639	PMKRAVAFCQ-VIEPGK----GGKTHKVSSKEISGMFQAVVEAYQDA-------------
gi|896513082|ref|WP_049419616.1|damageinducibleprotein[Stenotrophomonasmaltophilia]/11638	PMKRAVAFCQ-VIEPGK----GGKTHKVSSKEIAGMFQAVVEAYQDA-------------
gi|896580616|ref|WP_049469425.1|damageinducibleprotein[Stenotrophomonasmaltophilia]/11641	PMKRAVAFCQ-VIEPSR----GGKTHKVSSKEIADMFKAVVDAYQDA-------------
gi|648289350|ref|WP_026070499.1|damageinducibleprotein[Stenotrophomonasmaltophilia]/11639	PMKRAVAFCQ-VIEPSR----GGKTHKVSSKEIADMFKAVVDAYQDA-------------
gi|656272786|ref|WP_029218442.1|damageinducibleprotein[Xanthomonascassavae]/11639	PMKRAVAFCQ-VIEPSR----GGKTHKVSSKEIADMFKAVVDAYQDA-------------
gi|406976236|gb|EKD98747.1|hypotheticalproteinACD_23C00290G0002[unculturedbacterium]/11679	PMKRAVAFCQ-VIEHKK----GSKTHKVSSKQIAGMFQAVVEAYQTSEPELSDE------
gi|828145737|ref|WP_047238120.1|damageinducibleprotein[Chromobacteriumsubtsugae]/11645	AMRRAVAFCQ-VIEYQS----NAKTHKISSKKIADMFSAVVEEYKASNPD----------
gi|654311174|ref|WP_027824327.1|damageinducibleprotein[Laribacterhongkongensis]/11641	AMRRAVAFCQ-VIEYQS----NAKTHKISSKKIADMFAAVVEEYKASNPD----------
gi|757242173|gb|KIR15516.1|typeIrestrictionenzymeEcoKIsubunitR[Pseudomonasfluorescens]/11778	PMRRAVAFCQ-VIEYQR----FAKTHKVSSKNIASMFDQVVEAYKASNP-----------
gi|653605161|ref|WP_027616682.1|damageinducibleprotein[Pseudomonassp.URHB0015]/11639	PMRRAVAFCQ-VIEYQK----FAKTHKVSSKNIASMFDQVVEAYKTSNP-----------
gi|520868025|ref|WP_020308060.1|helicase[Pseudomonassyringae]_gi|519159034|gb|EPM49935.1|helicasedomaincontainingprotein[Pseud/	PMRRAVAFCQ-VIEYQK----FAKTHKVSSKNIASMFDQVVESYKASNP-----------
gi|940156567|gb|KPX71668.1|Helicasedomaincontainingprotein[Pseudomonasamygdalipv.lachrymans]/11677	PMRRAVAFCQ-VIEYQR----FAKTHKVSSKNIASMFDQVVEAYKTSNP-----------
gi|505460508|ref|WP_015647610.1|helicase[Pseudomonassp.GLE121]_gi|499110212|ref|YP_007974374.1|helicasedomaincontainingprotein/	PMKRAVAFCQ-VIEASH---NKKGTHKVSSKNIASMFQAVVEAYQES-------------
gi|940235551|gb|KPY45311.1|hypotheticalproteinALO48_200154[Pseudomonassyringaepv.rhaphiolepidis]/11631	PMKRAVAFCQ-VIEPNH---NKKGTHKVSSKNIARMFQSVVESYQAS-------------
gi|520880808|ref|WP_020312235.1|helicase[Pseudomonassyringae]_gi|519172158|gb|EPM62027.1|helicasedomaincontainingprotein[Pseud/	PMKRAVAFCQ-VISPDY----KGSKHKVSSKNIAGMFQSVVEAYQES-------------
gi|829046479|ref|WP_047282259.1|damageinducibleprotein[Pseudomonasfluorescens]/11630	PMKRAVAFCQ-VIAPNY----KGTKHKVSSINIASMFKSVVEAYQES-------------
gi|499415493|ref|WP_011102960.1|DNAdamageinducibleprotein[Pseudomonassyringaegroupgenomosp.3]_gi|28867279|ref|NP_789898.1|heli/	PMKRAVAFCQ-VISPSY----KGTKHKVSSINIASMFQSVVEAYQES-------------
gi|730238299|ref|WP_033942897.1|damageinducibleprotein[Pseudomonasaeruginosa]/11629	PMKRAVAFCQ-VISPNY----KGTKHKVSSINIASMFQSVVEAYQES-------------
gi|495310125|ref|WP_008034873.1|DNAdamageinducibleprotein[Pseudomonassp.GM60]_gi|398186685|gb|EJM74052.1|putativehelicase[Pseu/	PMRRAVAFCQ-VISPNY----KGTKHKVSSINIASMFQSVVEAYQES-------------
gi|779992653|ref|WP_045428690.1|damageinducibleprotein[Pseudomonassp.MT1]_gi|699967872|dbj|BAP80334.1|DNAhelicaserestrictione/1	PMKRAVAFCQ-VISPNY----KGTKHKVSSVNIADTFQKVVEAYQDA-------------
gi|786037289|ref|WP_045733274.1|damageinducibleprotein[Pseudomonaspseudoalcaligenes]_gi|782987904|gb|KJU79152.1|damageinducib/1	PMKRAVAFCQ-VISPNY----KGTKHKVSSINIASMFQSVVEAYQES-------------
gi|749666366|ref|WP_040265445.1|damageinducibleprotein[Pseudomonasrhodesiae]/11632	PMKRAVAFCQ-VISPNY----KGTKHKVSSINIASMFQSVVEAYQES-------------
gi|734902433|ref|WP_034133698.1|damageinducibleprotein[Pseudomonasfluorescens]/11635	PMKRAVAFCQ-VISPNY----KGTKHKVSSINIASMFQSVVEAYQES-------------
gi|490551031|ref|WP_004416146.1|typeIIrestrictionmodificationsystemrestriction/helicasesubunit[Pseudomonassyringae]_gi|4432823/	PMRRAVAFCQ-VISPNY----KGTKHKVSSISIASMFQSVVEAYQES-------------
gi|489199911|ref|WP_003109152.1|DNAdamageinducibleprotein[Pseudomonasaeruginosa]_gi|552648708|gb|ERY63184.1|hypotheticalprotei/	PMKRAVAFCQ-VISPNY----KGTKHKVSSINIASMFQAVVEAYQES-------------
gi|730641237|ref|WP_034061328.1|damageinducibleprotein[Pseudomonasaeruginosa]/11636	PMKRAVAFCQ-VISPNY----KGTKHKVSSINIASMFQSVVEAYQES-------------
gi|751304980|ref|WP_041013386.1|damageinducibleprotein[Pseudomonasxanthomarina]_gi|703407611|emb|CEG51014.1|Helicasedomaincon/1	PMKRAVAFCQ-VIAPNY----KGTKHKVSSISIASMFQSVVEAYQES-------------
gi|939335207|ref|WP_054881520.1|damageinducibleprotein[Pseudomonassp.NBRC111128]/11632	PMRRAVAFCQ-VISPNY----KGTKHKVSSINIASMFQSVVEAYQES-------------
gi|757693555|ref|WP_042923792.1|damageinducibleprotein[Pseudomonaschloritidismutans]_gi|780910610|gb|KJS78302.1|damageinducib/1	PMKRAVAFCQ-VISPNY----KGTKHKVSSVNIASMFQSVVEAYQEA-------------
gi|665953944|ref|WP_031319989.1|damageinducibleprotein[Pseudomonastolaasii]/11626	PMKRAVAFCQ-VISPNY----KGTKHKVSSVNIADMFQSVVEAYQES-------------
gi|771600573|ref|WP_045213746.1|damageinducibleprotein[Pseudomonassp.21]_gi|770081123|gb|KJJ98634.1|damageinducibleprotein[Ps/1	PMKRAVAFCQ-VISPNY----KGTKHKVSSINIASMFKSVVEAYQES-------------
gi|740871396|ref|WP_038656644.1|damageinducibleprotein[Pseudomonasstutzeri]_gi|627772214|gb|AHY41437.1|damageinducibleprotein/1	PMKRAVAFCQ-VISPNY----KGTKHKVSSINIASMFQSVVEAYQES-------------
gi|946242205|gb|KRC93782.1|damageinducibleprotein[Pseudomonassp.Root9]/11634	PMKRAVAFCQ-VISPNY----KGTKHKVSSINIASMFQSVVEAYQES-------------
gi|553777322|ref|WP_023109107.1|MULTISPECIES:hypotheticalprotein[Pseudomonas]_gi|552638674|gb|ERY53184.1|hypotheticalproteinQ05	PMRRAVAFCQ-VISPNY----KGTKHKVSSINIASMFQSVVEAYQES-------------
gi|896383370|ref|WP_049324618.1|damageinducibleprotein[Pseudomonasstutzeri]/11627	PMRRAVAFCQ-VISPNY----KGTKHKVSSINIASMFQSVVEAYQQS-------------
gi|696158510|gb|AIT41787.1|putativehelicase[Pseudomonassp.LM8]/11630	PMKRAVAFCQ-VISPNY----KGTKHKVSSINIASMFQSVVEAYQES-------------
gi|504634892|ref|WP_014821994.1|DNAdamageinducibleprotein[Pseudomonasstutzeri]_gi|390985249|gb|AFM35242.1|helicasedomainconta/1	PMKRAVAFCQ-VISPNY----KGTKHKVSSINIASMFQSVVEAYQES-------------
gi|544783974|ref|WP_021206729.1|DNAdamageinducibleprotein[Pseudomonasstutzeri]_gi|533224779|gb|EQM79688.1|DNAdamageinduciblep/1	PMKRAVAFCQ-VISPNY----KGTKHKVSSINIASMFQSVVEAYQES-------------
gi|504567385|ref|WP_014754487.1|MULTISPECIES:DNAdamageinducibleprotein[Pseudomonas]_gi|388559493|gb|AFK68634.1|helicasedomain/1	PMKRAVAFCQ-VISPNY----KGTKHKVSSINIASMFQSVVEAYQES-------------
gi|654441469|ref|WP_027912052.1|damageinducibleprotein[Pseudomonassp.URIL14HWK12:I7]/11630	PMRRAVAFCQ-VISPNY----KGTKHKVSSINIASMFQSVVEAYQES-------------
gi|941470066|ref|WP_055127894.1|damageinducibleprotein[Pseudomonasmediterranea]/11631	PMRRAVAFCQ-VISPNY----KGTKHKVSSINIASMFQSVVEAYQES-------------
gi|557815599|ref|WP_023446184.1|DNAdamageinducibleprotein[Pseudomonaschloritidismutans]_gi|557364081|gb|ESQ98190.1|DNAdamagei/1	PMKRAVAFCQ-VISPNY----KGTKHKVSSVNIASMFQSVVEAYQES-------------
gi|727814791|ref|WP_033898247.1|damageinducibleprotein[Pseudomonassp.FH1]_gi|567648005|gb|ETK23229.1|helicasedomaincontaining/1	PMKRAVAFCQ-VISPNY----KGTKHKVSSINIASMFQSVVEAYQES-------------
gi|503278702|ref|WP_013513363.1|DNAdamageinducibleprotein[Desulfovibrioaespoeensis]_gi|316942375|gb|ADU61426.1|typeIIrestrict/	PMKRAVAFCQ-VIEIAR----NARKHKVSSKNIAAMFEEVVQEYQKTEQD----------
gi|505228796|ref|WP_015415898.1|typeIIrestrictionendonucleasesubunitR[Desulfovibriopiezophilus]_gi|451896976|emb|CCH49855.1|Ty	PMKRAVAFCQ-VIEIAR----NARKHKVSSKNIANMFQKVVKSYQESETE----------
gi|517732543|ref|WP_018902751.1|hypotheticalprotein[Variovoraxparadoxus]/11675	AMKRAVAFCQ-VIEVSK----GAKTHKVSSKNIAAMFQSVVEAYQETEEE----------
gi|550909475|ref|WP_022662570.1|DNAdamageinducibleprotein[Desulfovibriolongus]/11637	PMRRAVAFCQ-VIEVQR----KAKVHKVSSKNIAAMFQAVVEAYQDS-------------
gi|945494542|gb|KQV50024.1|damageinducibleprotein[Massiliasp.Root335]/11647	SMKRAVAFCQ-VIEVSK----GAKTHKVSSKNIAGMFQAVVEAYQESEED----------
gi|334196917|gb|AEG70102.1|helicasedomainprotein[RalstoniasolanacearumPo82]/11663	AMKRAVAFCQ-VIEVSK----GAKTHKVSSKQIAGMFQAVVEAYQESEET----------
gi|656006746|ref|WP_029048170.1|damageinducibleprotein[Cupriavidussp.amp6]/11661	AMKRAVAFCQ-VIEVSK----GAKTHKVSSKQIAGMFQAVVEAYQESEAT----------

```
BPS2196ORF3054P/11659                                                                                             PMSRAVAFCQ-VIEISK----GAKSHKVSSKQIAGMFQAVVEAYQESEET----------
gi|740932312|ref|WP_038717332.1|damageinducibleprotein[Burkholderiapseudomallei]_gi|760211882|gb|KIX58314.1|damageinduciblepr/1    PMSRAVAFCQ-VIEISK----GAKSHKVSSKQIAGMFQAVVEAYQESEET----------
gi|755810813|ref|WP_042583861.1|damageinducibleprotein[Burkholderiasp.MSHR3999]_gi|755174952|gb|KIP19790.1|DEAD/DEAHboxhelicas/    PMSRAVAFCQ-VIEVSK----GAKTHKVSSKQIASMFQAVVEAYQEE-------------
gi|484586570|gb|AGK48195.1|DEAD/DEAHboxhelicasefamilyprotein[BurkholderiathailandensisMSMB121]/11646    PMSRAVAFCQ-VIEVSK----GSKSHKVSSKQIAAMFQAVVEAYQESEET----------
gi|737582935|ref|WP_035553941.1|damageinducibleprotein[Burkholderiasp.9120]/11658    PMKRAVAFCQ-VIEVSK----GAKTHKVSSKQIKNMFQAVVEAYQESEET----------
gi|740989872|ref|WP_038773225.1|damageinducibleprotein[Burkholderiapseudomallei]_gi|705645291|gb|KGS70691.1|DEAD/DEAHboxhelica/    PMSRAVAFCQ-VIEVAK----GAKTHKVSSKQIAGMFQAVVEAYQESEET----------
gi|654912525|ref|WP_028363424.1|damageinducibleprotein[Burkholderiasp.JPY366]/11659    PMSRAVAFCQ-VIEVSK----GAKTHKVSSKQIAGMFQAVVEAYQESEET----------
gi|740955879|ref|WP_038740512.1|damageinducibleprotein[Burkholderiapseudomallei]_gi|714684932|gb|KGW74438.1|DEAD/DEAHboxhelica/    PMSRAVAFCQ-VIEVSK----GAKSHKVSSKQIAGMFQAVVEAYQESEET----------
gi|919203714|ref|WP_052759215.1|damageinducibleprotein[Xanthomonasaxonopodis]/11388    PMRRAVAFAR-DIKSS--------------KHLAQLFPEVVDQYIEGSDE----------
gi|522152381|ref|WP_020663589.1|hypotheticalprotein[Amycolatopsisbenzoatilytica]/11605    PMRRAVAFSR-SIKES--------------KKLTDSFAEVIGAYDA-ADE----------
gi|663196013|ref|WP_030233899.1|damageinducibleprotein[Streptomycessp.NRRLS455]/11641    PMRRAVAFAR-SIKDS--------------QAIADHFNAIVSSYED-ADD----------
gi|750429553|ref|WP_040711444.1|damageinducibleprotein[Nocardiopsispotens]/11622    PMQRAVAFAR-SIADS--------------KAITERFQEVVDAYDD-ADE----------
gi|518656139|ref|WP_019819475.1|hypotheticalprotein[Saccharomonosporasaliphila]/11623    TMKRAVAFAR-SIADS--------------KTITERFNEVVDAYDD-ADD----------
gi|759722933|ref|WP_043433758.1|damageinducibleprotein[Streptomycespluripotens]_gi|733404978|gb|KHK94361.1|damageinduciblepro/1    PMKRAVAFSR-SIADS--------------KAIMERFNEVVDAYDD-ADD----------
gi|663424167|ref|WP_030419030.1|damageinducibleprotein[Streptomycessp.NRRLF5065]/11627    PMKRAVAFSR-SIADS--------------KAIMERFNEVVDAYDD-ADD----------
gi|663412917|ref|WP_030408353.1|damageinducibleprotein[Streptomycessp.NRRLF5917]/11630    PMKRAVAFSR-SIADS--------------KAIVERFNQVIDAYDD-ADD----------
gi|671521483|ref|WP_031506489.1|damageinducibleprotein[Streptomycesmegasporus]/11630    PMKRAVAFSR-SIADS--------------KAIMERFNEVVDAYDD-ADD----------
gi|740053144|ref|WP_037902776.1|damageinducibleprotein[Streptomycessp.TAA204]/11626    PMKRAVAFAR-SIADS--------------KSIAARFNEVVDAYDD-ADD----------
gi|663409375|ref|WP_030404974.1|MULTISPECIES:damageinducibleprotein[Streptomyces]/11627    PMKRAVAFAR-SIADS--------------KSIAERFKEVVDAYDD-ADD----------
gi|746628608|ref|WP_039654277.1|damageinducibleprotein[Streptomycessp.MUSC125]_gi|745644381|gb|KIE25557.1|damageinducibleprot/1    PMKRAVAFAR-SIADS--------------KSIADRFNEVVDAYDD-ADD----------
gi|727322066|ref|WP_033777320.1|damageinducibleprotein[Salinisporapacifica]/11627    PMKRAVAFAR-SIADS--------------TAIARRFTEVVDAYDD-ADD----------
gi|653653242|ref|WP_027655726.1|damageinducibleprotein[Salinisporapacifica]/11618    PMKRAVAFAR-SIADS--------------TAIAKRFNEVVDAYDD-ADD----------
gi|648632543|ref|WP_026324294.1|damageinducibleprotein[Salinisporapacifica]/11621    PMKRAVAFAR-SIADS--------------TAIAKRFNDVVDAYDN-ADD----------
gi|881018002|ref|WP_048738463.1|damageinducibleprotein[Corynebacteriumfalsenii]/11676    PMQRAVAFAK-DIRES--------------KQIAESYPALVGAFQQSLRDQWEE------
gi|505465526|ref|WP_015652417.1|helicase[Corynebacteriumcallunae]_gi|459385704|gb|AGG67995.1|helicase[CorynebacteriumcallunaeDS    AMERAVAFAR-DIKTS--------------QQIAESFPRVVNAYTTELEV--K-------
gi|41223062|emb|CAF19007.1|DNAorRNAhelicaseofsuperfamilyII[CorynebacteriumglutamicumATCC13032]/11646    AMERAVAFAR-DIKTS--------------QQIAESFPRVVNAYTTELEV--K-------
CGL13032I/11643    AMERAVAFAR-DIKTS--------------QQIAESFPRVVNAYTTELEV--K-------
gi|751257081|ref|WP_040967675.1|damageinducibleprotein[Corynebacteriumglutamicum]_gi|748811620|gb|AJE67931.1|damageinduciblep/1    PMQRTVAFAR-NIKTS--------------KQITDAFPTLIANHVDALNEAAA-------
gi|749040539|ref|WP_040086524.1|damageinducibleprotein[Corynebacteriumhumireducens]/11663    PMQRTVAFAR-DIKTS--------------KKIAESFPTLISNFTDTLREKAA-------
gi|491662899|ref|WP_005519615.1|helicase[Corynebacteriummatruchotii]_gi|224946756|gb|EEG27965.1|helicaseCterminaldomainprotein/    PMRRTVAFAK-DIKTS--------------KEITETYPALIRNYQSLLLEASA-------
gi|746717312|ref|WP_039676231.1|damageinducibleprotein[Corynebacteriumminutissimum]_gi|734991118|gb|KHO29219.1|damageinducibl/1    PMRRTVAFAK-DIKAS--------------KTIQETFPTLIRQYQEQLKDHAA-------
gi|908654892|emb|CQD15529.1|SuperfamilyIIDNA/RNAhelicase(modularprotein)[Corynebacteriumstriatum]/11674    PMLRTVAFAK-DIKAS--------------KTIQETFPTLIRQYQEQLKDHAA-------
gi|895994446|ref|WP_049064418.1|damageinducibleprotein[Corynebacteriumaurimucosum]/11667    PMLRTVAFAK-DIKAS--------------KTIQETFPTLIRQYQEQLKDHAA-------
gi|896123331|ref|WP_049145390.1|damageinducibleprotein[Corynebacteriumaurimucosum]/11669    PMRRTVAFAK-DIKAS--------------KIIQETFPTLIRQYQEQLKDHAA-------
gi|817681178|ref|WP_046645422.1|damageinducibleprotein[Corynebacteriumstriatum]_gi|817551679|gb|KKO79868.1|damageinduciblepro/1    AMRRTVAFAK-DIKAS--------------KIIQETFPTLIRQYQEQLKDHAA-------
gi|896139239|ref|WP_049159146.1|damageinducibleprotein[Corynebacteriumaurimucosum]/11675    PMRRTVAFAK-DIKAS--------------KIIQETFPTLIRQYQEQLKDHAA-------
gi|489943947|ref|WP_003847254.1|helicase[Corynebacteriumammoniagenes]_gi|295968218|gb|EFG81467.1|helicaseCterminaldomainprotei/    PMQRTVAFAK-DIKAS--------------KQIAESFPSLIETHKAQLIEHQA-------
gi|516653464|ref|WP_018022453.1|helicase[Corynebacteriumdoosanense]_gi|697778282|gb|AIT61018.1|damageinducibleprotein[Coryneba/    PMRRTVAFAK-DIKTS--------------KQIAESFPSLISSHKVQLGFQQN-------
gi|493894179|ref|WP_006840129.1|helicase[Corynebacteriumlipophiloflavum]_gi|227078819|gb|EEI16782.1|helicaseCterminaldomainpro/    PMRRSVAFAK-DIKTS--------------KQIAESYPALIANYQQSLREASA-------
gi|740831472|ref|WP_038616755.1|damageinducibleprotein[Corynebacteriumpseudotuberculosis]_gi|666103058|gb|AIG06935.1|putativeh/    PMRRTVAFAK-DIKHS--------------RQIAESFPALIAAHQELLHQNAVN------
gi|518023322|ref|WP_019193530.1|helicase[Corynebacteriumtimonense]/11655    PMQRTVAFAK-DIRHS--------------QQIADAFPTLVSAHQEMLRQHAE-------
gi|552767697|ref|WP_023019559.1|MULTISPECIES:hypotheticalprotein[Corynebacterium]_gi|550739013|gb|ERS38609.1|hypotheticalprotei    PMQRTVVFAK-DIKHS--------------KQIADSFPALVTAHQESLHEHAA-------
gi|652659121|ref|WP_027017812.1|damageinducibleprotein[Corynebacteriumpseudodiphtheriticum]/11644    PMQRTVAFAK-DIKHS--------------KQIADSFPALVAAHQESLHEHAA-------
gi|749039309|ref|WP_040085390.1|damageinducibleprotein[Corynebacteriumhumireducens]/11657    PMRRTVAFAK-DINTS--------------KQIAESFPALISQHQETLRDHAV-------
gi|740835591|ref|WP_038620874.1|damageinducibleprotein[Corynebacteriumulcerans]_gi|698973854|gb|AIU33664.1|HelicaseCterminald/1    PMKRTVAFAK-DIKTS--------------KLISESFPALIASHQDTLRSHAA-------
gi|805307599|ref|WP_046096452.1|damageinducibleprotein[Corynebacteriumulcerans]_gi|803357323|gb|AKA97638.1|Damageinduciblepro/1    PMKRTVAFAK-DIKTS--------------KLIAESFPALIASHQDTLRSHAA-------
gi|504083555|ref|WP_014317549.1|helicase[Corynebacteriumdiphtheriae]_gi|371584249|gb|AEX47914.1|superfamilyIIDNA/RNAhelicase[Co    PMRRAVAFAR-DIKTS--------------KQIAESYPLLISNYQQSLKKKAA-------
gi|896182193|ref|WP_049193580.1|damageinducibleprotein[Corynebacteriumjeikeium]/11645    PMRRTVAFAR-DIKTS--------------KQIAESYPLLIDNYQQSMKEKAA-------
gi|917636459|ref|WP_052205460.1|damageinducibleprotein[Corynebacteriumriegelii]_gi|914295811|gb|AKV59187.1|damageinduciblepro/1    PMRRTVAFAK-DIKTS--------------KLIANTYPGLIASHQETLREHAA-------
gi|737013522|ref|WP_035009205.1|damageinducibleprotein[Corynebacteriumjeikeium]/11651    PMQRTVAFAK-DIKTS--------------KLIAETYPGLIASHQEALREHGV-------
gi|737006291|ref|WP_035002094.1|damageinducibleprotein[Corynebacteriumjeikeium]/11658    PMQRAVAFAQ-DIKTS--------------KQIAESFPSLIFTYQQQLREASV-------
gi|737010378|ref|WP_035006133.1|damageinducibleprotein[Corynebacteriumjeikeium]/11650    SMQRAVAFAR-DIKTS--------------KQISESFPSLISAYQQQLREAAV-------
gi|737010473|ref|WP_035006228.1|damageinducibleprotein[Corynebacteriumjeikeium]/11657    PMGRSVAFAR-DIRTS--------------QQIAEFYPKLISSYQDMLKDKAA-------
gi|737006250|ref|WP_035002053.1|damageinducibleprotein[Corynebacteriumjeikeium]/11658    PMGRSVAFAR-DIRTS--------------QQIAEFYPKLISSYQDMLKDKAA-------
gi|923018168|ref|WP_053412254.1|damageinducibleprotein[Corynebacteriumlactis]_gi|922845184|gb|ALA67504.1|helicase[Corynebacter/    AMRRAVAFAK-DIKTS--------------TQIAESFPALVATHQELLKEHAV-------
gi|923018885|ref|WP_053412970.1|damageinducibleprotein[Corynebacteriumlactis]_gi|922845845|gb|ALA68165.1|helicase[Corynebacter/    AMQRAVAFAK-DIKTS--------------TQIAESFPALVSTHQELLKEHAV-------
gi|512464870|ref|WP_016422869.1|hypotheticalprotein[Corynebacteriumsp.HFH0082]_gi|512047830|gb|EPD47505.1|hypotheticalproteinHM    PMPRAVAFAR-DIKTS--------------QAIADSYPQLVNTHRELLEEKAV-------
gi|881012697|ref|WP_048733406.1|damageinducibleprotein[Corynebacteriumurealyticum]/11670    PMQRAVAFAR-DIKTS--------------QAIADSYPQLVNMHRELLEEKAV-------
gi|755608478|ref|WP_042530519.1|damageinducibleprotein[Corynebacteriumsingulare]_gi|754340239|gb|AJI78730.1|putativehelicase[C/    PMQRAVAFAK-DIKTS--------------QLIAETYPSLIGTHQELLKEKAV-------
gi|491421796|ref|WP_005279593.1|helicase[Corynebacteriumaccolens]_gi|227077180|gb|EEI15143.1|helicaseCterminaldomainprotein[Co/    PMQRAVAFAK-DIKTS--------------EQIAESFPSLIRTHQELLKDKAA-------
gi|550752286|gb|ERS51451.1|hypotheticalproteinHMPREF1282_00033[Corynebacteriumsp.KPL1856]_gi|550759961|gb|ERS58964.1|hypothetic    AMQRAVAFAK-DIKSS--------------QLIADTYPSLIATHQELLKDKAV-------
gi|497955053|ref|WP_010269209.1|helicase[Corynebacteriumbovis]/11599    PMQRAVAFAV-DIRAS--------------RRVAETFPRITAG-----------------
gi|860580040|ref|WP_048470569.1|hypotheticalprotein[Mycobacteriumchlorophenolicum]_gi|859098575|gb|KMO76871.1|typeIrestrictione    PMQRAVAFAR-DIAAS--------------KQIASLFPRVVEAYQSSLDAQEND------
gi|262085905|gb|ACY21873.1|typeIIIrestrictionproteinressubunit[GordoniabronchialisDSM43247]/11632    PMRRAVAFAK-DIAAS--------------KQVAEVFPAVVDAYRDLLDDTDPL------
gi|495996338|ref|WP_008720917.1|typeIIIrestrictionendonucleasesubunitR[Rhodococcussp.AW25M09]_gi|443412350|emb|CCQ18455.1|TypeI    PMKRAVAFAK-DIAAS--------------KQVAEVFPDVVDAYRALLDENEND------
gi|739284636|ref|WP_037147410.1|damageinducibleprotein[Rhodococcusfascians]/11641    PMKRAVAFAK-DIAAS--------------KQVAEVFPDVVDAYRALLEENEND------
gi|739328195|ref|WP_037190574.1|damageinducibleprotein[Rhodococcusfascians]/11643    PMKRAVAFAK-DIAAS--------------KQVAEVFPDVVDAYRALLDENENE------
```

```
gi|811698335|emb|CMN21041.1|helicase[Mycobacteriumtuberculosis]/11725    PMRRAVAFAK-DIKTS--------------KQVAELFPKVVEAYRELVDD----------
gi|801577898|emb|CFK23773.1|helicase[Mycobacteriumtuberculosis]/11675    PMRRAVAFAK-DIKTS--------------KQVAELFPKVVEAYRELVDD----------
MTU18II/11606    PMRRAVAFAK-DIKTS--------------KQVAELFPKVVEAYRELVDD----------
gi|887719858|emb|CMT27886.1|helicase[Mycobacteriumtuberculosis]/11667    PMRRAVAFAK-DIKTS--------------KQVAELFPKVVEAYRELVDD----------
gi|621694743|gb|KAS87156.1|hypotheticalproteinAL70_01801[MycobacteriumtuberculosisTKK_03_0115]/11604    PMRRAVAFAK-DIKTS--------------KQVAELFPKVVEAYRELVDD----------
gi|888128072|emb|CLQ84422.1|helicase[Mycobacteriumtuberculosis]_gi|889917090|emb|CLY86893.1|helicase[Mycobacteriumtuberculosis]    PMRRAVAFAK-DIKTS--------------KQVAELFPKVVEAYRELVDD----------
gi|884823424|emb|CKL96672.1|helicase[Mycobacteriumtuberculosis]/11466    PMRRAVAFAK-DIKTS--------------KQVAELFPKVVEAYRELVDD----------
gi|505103239|ref|WP_015290341.1|hypotheticalprotein[Mycobacteriumcanettii]_gi|432162733|emb|CCK60117.1|Conservedproteinofunknow    PMRRAVAFAK-DIKTS--------------KQVAEMFPKVVEAYRELVDD----------
gi|505116250|ref|WP_015303352.1|hypotheticalprotein[Mycobacteriumcanettii]_gi|432158726|emb|CCK56026.1|Conservedproteinofunknow    PMRRAVAFAK-DIKTS--------------KQVAELFPKVVEAYRELVDD----------
gi|432154735|emb|CCK51974.1|Conservedproteinofunknownfunction(part1)[MycobacteriumcanettiiCIPT140060008]/11588    PMRRAVAFAK-DIKTS--------------KQVAELFPKVVEAYRELVDD----------
gi|809008027|ref|WP_046295375.1|hypotheticalprotein[Mycobacteriumsp.UM_Kg17]/11624    PMRRAVAFAR-DIKSS--------------KQVAEMFPIVVTRYQQLLDEHEND------
gi|808802633|ref|WP_046284139.1|hypotheticalprotein[Mycobacteriumsp.UM_NZ2]/11624    PMRRAVAFAR-DIKTS--------------KQVAEMFPVVVTRYQELLDAHEDD------
gi|750125774|ref|WP_040429852.1|damageinducibleprotein[Corynebacteriumnuruki]/11668    PMRRAVAFAK-DIKSS--------------KQVAEMFPQVVTAYQDLLNEQEDK------
gi|602237614|gb|EYT60960.1|damageinducibleprotein[Dietziasp.UCDTHP]/11473    PMKRAVAFAK-DIASS--------------KQIEAMFPAVVDAYQDLLDDAEDD------
gi|665897412|ref|WP_031264618.1|damageinducibleprotein[Dietziasp.UCDTHP]_gi|602237599|gb|EYT60945.1|helicase[Dietziasp.UCDTH/11    PMKRAVAFAK-DIASS--------------KQIEAMFPAVVGAYQDLLDDAEDD------
gi|502903674|ref|WP_013138650.1|helicase[Segniliparusrotundus]_gi|296181291|gb|ADG98197.1|typeIIIrestrictionproteinressubunit[S    PMRRAVAFAR-DIDSS--------------KMVAEMFPRVVDAYRDTLDDEHGE------
gi|780248306|ref|WP_045538930.1|hypotheticalprotein[Gordoniasp.no.9]_gi|778142514|gb|KJR06917.1|hypotheticalproteinUG54_12320[G    PMRRAVAFAK-DIASS--------------QSVAAMFPAVVDAYRDTLDDEDSD------
gi|665897368|ref|WP_031264574.1|damageinducibleprotein[Dietziasp.UCDTHP]_gi|602239137|gb|EYT62483.1|helicase[Dietziasp.UCDTH/11    PMRRAVAFAR-DIKSS--------------KQVAEMLPRVVESYQDLLRDAEVD------
gi|493372923|ref|WP_006329236.1|helicase[Gordoniarhizosphera]_gi|403207917|dbj|GAB88168.1|hypotheticalproteinGORHZ_006_00370[Go    PMQRAVAFAR-DIADS--------------KHLATVFPAVVESYQDLLTDSETE------
gi|764904870|ref|WP_044487821.1|hypotheticalprotein[Mycobacteriumtuberculosis]_gi|625005353|gb|KBZ57120.1|hypotheticalproteinK8    PMRRAVAFAK-DIAAS--------------KQVAELFPKVVDAYREMLSNSQDD------
gi|927068212|ref|WP_053778441.1|damageinducibleprotein[Gordoniasp.NB41Y]_gi|926660541|gb|EMP14526.2|damageinducibleprotein[G/11    PMRRAVAFAK-DIAAS--------------KKIAELFPRVVDTYRDMLTDSINE------
gi|935514169|ref|WP_054429894.1|damageinducibleprotein[Mycobacteriumimmunogenum]_gi|929801120|gb|KPG29247.1|damageinduciblepr/1    PMRRAVAFAK-DIAAS--------------KQVAELFPKVVEAYREMLSDSQDD------
gi|941317728|ref|WP_055118574.1|hypotheticalprotein[Mycobacteriumperegrinum]/11657    PMRRAVAFAK-DIAAS--------------KQVAELFPKVVEAYREMLTDSLDD------
gi|933683673|gb|ALG86377.1|hypotheticalproteinACH46_20120[Gordoniasp.QH11]/11636    PMRRAVVFAK-DIAAS--------------KQVSEVFPAVVDAYRDLLTEHQDD------
gi|873553947|emb|CRY84110.1|Predictedhelicase[Nocardiafarcinica]/11688    PMKRAVVFAK-DIAAS--------------KQVAEVFPAVVDAYRELLADREDD------
gi|499525749|ref|WP_011212389.1|helicase[Nocardiafarcinica]_gi|54019339|dbj|BAD60707.1|hypotheticalprotein[NocardiafarcinicaIFM    PMRRAVVFAK-DIAAS--------------KQVAEVFPAVVDAYRELLADREDD------
gi|640109609|ref|WP_024794187.1|damageinducibleprotein[Tomitellabiformata]/11653    PMRRAVAFAK-DIAAS--------------KQVAEVFPAVVDAYRDLLATSEDD------
gi|640109991|ref|WP_024794334.1|hypotheticalprotein[Tomitellabiformata]/11639    PMRRAVAFAK-DIAAS--------------KQVAEVFPAVVDAYRDLLATSEDD------
gi|939125496|ref|WP_054831438.1|damageinducibleprotein[Rhodococcuserythropolis]/11669    PMRRAVAFAK-DIAAS--------------KQVAEVFPTVVDAYRDLLENSEND------
gi|910258550|ref|WP_050062711.1|hypotheticalprotein[Rhodococcussp.RD6.2]_gi|846871967|emb|CRK49225.1|conservedhypotheticalprote    PMRRAVVFAK-DIAAS--------------KQVAEVFPSVVDAYRDLLAENEND------
gi|797148189|gb|KJY52358.1|putativeHelicase[Bifidobacteriumasteroides]/11801    PLRRAVAFTR-RIAD--------------SKALADGFNDVIFRYLEQQIKSG--------
gi|535476570|gb|EFG27357.2|hypotheticalproteinHMPREF9020_00999[ScardoviainopinataF0304]/11827    HMHTAVAFTR-TIHD--------------SKILADGFSDVISEYIQQSEKQG--------
gi|737482729|ref|WP_035462496.1|hypotheticalprotein[Alicyclobacillusmacrosporangiidus]/11512    PLQRLIAFVN-SRAR--------------SREIVETWH----KYADKM------------
gi|685434354|gb|KGA95679.1|helicase[BacillusalcalophilusATCC27647=CGMCC1.3604]/11575    PMKRAIAFTG-TIRE--------------SKLIANMFSEVVETYINESGN----------
gi|945370253|gb|KQU27251.1|helicase[Bacillussp.Leaf13]/11557    PMKRAIAFTG-TIRE--------------SKLITDMFSHVVDQYLYQDTED---------
gi|880997015|ref|WP_048719116.1|helicase[Enterococcushirae]/11563    PMKRAIAFTG-TIKE--------------SIMIKDMFKEVVDLYINASQD----------
gi|945356929|gb|KQU14100.1|helicase[Bacillussp.Leaf49]/11547    PMKRAIAFTG-TIKD--------------SKLIKDMFNHVVNEYLYQDVDE---------
gi|763482876|gb|KIZ53217.1|helicase[Bacillussafensis]/11563    PMKRAIAFTG-TIKD--------------SKLITNMFSSVVDEYINEVND----------
gi|852223292|ref|WP_048309707.1|helicase[Anaerobacillusmacyae]_gi|850322910|gb|KMM38590.1|helicase[Anaerobacillusmacyae]/11573    PMKRAIAFAG-TIRE--------------SKLIKEMFTEVVDMYINESGD----------
gi|739708665|ref|WP_037562619.1|helicase[Sporolactobacillusterrae]/11573    PMKRAIAFAG-TIRE--------------SKLIKEMFTEVVDMYINESGN----------
gi|496687561|ref|WP_009329104.1|MULTISPECIES:helicase[Bacillus]_gi|317390867|gb|EFV71668.1|endonucleaseandmethylaseLlaGI[Bacill    PMKRAIAFAG-TIRE--------------SKLIKDMFTEVVDMYINTSGD----------
gi|930213599|gb|KPH74225.1|helicase[Oceanobacilluscaeni]/11569    PMKRAIAFTG-TIRD--------------SKLIKEMFTEVVDMYINTSGD----------
gi|516366003|ref|WP_017756036.1|helicase[Bacillussp.ZYK]/11571    PMKRAIAFAG-TIRE--------------SKLIKEMFTEVVDMYINGSGD----------
gi|488909977|ref|WP_002821052.1|helicase[Oenococcusoeni]_gi|399965230|gb|EJN99855.1|endonucleaseandmethylaseLlaGI[Oenococcusoen    PMKRAIAFTN-TIAH--------------SKKIAQEFNQVVNDYLGDKA-----------
gi|495019967|ref|WP_007745780.1|helicase[Oenococcuskitaharae]_gi|366983682|gb|EHN59081.1|typeIIIrestrictionenzyme,ressubunit[Oe    PMKRAIAFTN-TIAN--------------SKKIAKEFNQVVNDYLGDKA-----------
gi|489666119|ref|WP_003570406.1|typeIIIrestrictionendonuclease[Lactobacilluscasei]_gi|410527834|gb|EKQ02696.1|typeIIIrestrictio    PMQRAIAFAS-VIDNQ----RGHGAGKVGSKQIAQEFNHVVNEYLGDDN-----------
gi|511447623|gb|EPC78110.1|superfamilyIIDNA/RNAhelicase[Lactobacillusparacaseisubsp.paracaseiLpp221}/11615    PMQRAIAFAS-VIDNQ----RGHRAGKVGSKQIAHEFSHVVNEYLGNDN-----------
gi|497700411|ref|WP_010014595.1|helicase[Lactobacilluscoryniformis]/11585    PMQRAIAFAS-VIDNQ----RGHGAGKVGSKQIAQEFSHVVNEYLGTDN-----------
gi|489784048|ref|WP_003687939.1|helicase[Lactobacillusmali]_gi|394428539|gb|EJF01080.1|superfamilyIIDNA/RNAhelicase[Lactobacill    PMQRAIAFAS-VIDNQ----RGHGAGKVGSKQIAREFNHVVNEYLGKDN-----------
gi|446780825|ref|WP_000858081.1|helicase[Staphylococcusaureus]_gi|374396604|gb|EHQ67835.1|typeIIIrestrictionenzyme,ressubunit[S    PMKRAISFID-SIEN--------------SKKIAEKFNSVVNEYLGDTA-----------
gi|510796658|ref|WP_016171456.1|hypotheticalprotein[Enterococcusdispar]_gi|508167532|gb|EOT44083.1|hypotheticalproteinOMK_00227    PMKRAISFID-TIKN--------------SEKISEKFNAVVNEYLGDNA-----------
gi|612613473|gb|EZS30411.1|hypotheticalproteinW603_02482[StaphylococcusaureusVET0353R]_gi|612881917|gb|EZU93992.1|hypotheticalp    PMKRAISFID-TINN--------------SEKIAKKFNAVVNEYLGDNA-----------
gi|488332893|ref|WP_002402278.1|helicase[Enterococcusfaecalis]_gi|306513864|gb|EFM82466.1|helicaseCterminaldomainprotein[Enter/    PMQRAISFIN-TINN--------------SKKISSQFNEVVNEYLEGNE-----------
gi|921166029|ref|WP_053111806.1|helicase[Enterococcusdurans]_gi|915771660|gb|AKX85928.1|helicase[Enterococcusdurans]/11562    PMQRAISFIN-TINN--------------SKKISSQFNEVVNEYLDGNE-----------
gi|498525358|ref|WP_010825030.1|hypotheticalprotein[Enterococcusfaecalis]_gi|487059631|gb|EOJ86727.1|hypotheticalproteinWOC_005    PMQRAISFIN-TINN--------------SKKISSQFNEVVNEYLDGNE-----------
gi|498412624|ref|WP_010722070.1|hypotheticalprotein[Enterococcusfaecium]_gi|486634654|gb|EOF75253.1|hypotheticalproteinSGC_0253    PMQRAISFIN-TINN--------------SKKISSQFNEVVNEYLDGNE-----------
gi|498408534|ref|WP_010718364.1|hypotheticalprotein[Enterococcusfaecium]_gi|486609608|gb|EOF50873.1|hypotheticalproteinSCW_0257    PMQRAISFIN-TINN--------------SKKISSQFNEVVNEYLDGNE-----------
gi|498407475|ref|WP_010717483.1|hypotheticalprotein[Enterococcusfaecalis]_gi|486594855|gb|EOF36348.1|hypotheticalproteinSCK_005    PMQRAISFIN-TINN--------------SKKISSQFNEVVNEYLEGNE-----------
gi|514885392|ref|WP_016615344.1|helicase[Enterococcusfaecalis]_gi|514505843|gb|EPH71676.1|helicaseprotein[Enterococcusfaecalis0    PMQRAISFIN-TINN--------------SKKISSQFNEVVNEYLEGNE-----------
gi|325157603|gb|EGC69759.1|helicaseCterminaldomainprotein[EnterococcuscasseliflavusATCC12755]/11561    PMQRAISFIN-TINN--------------SKKISSQFNEVVNEYLDGNE-----------
gi|895856206|ref|WP_048967668.1|helicase[Enterococcusfaecalis]/11551    PMQRAISFIN-TINN--------------SKKISSQFNEVVNEYLDGNE-----------
gi|639458836|ref|WP_024636366.1|helicase[Enterococcusfaecium]_gi|658176502|gb|KEI51159.1|helicase[EnterococcusfaeciumUC7256]/1/    PMQRAISFIN-TINN--------------SKKISSQFNEVVNEYLDGNE-----------
gi|488266511|ref|WP_002337719.1|hypotheticalprotein[Enterococcusfaecium]_gi|430581409|gb|ELB19854.1|hypotheticalproteinOIQ_0351    PMQRAISFIN-TINN--------------SKKISSQFNEVVNEYLEGNE-----------
gi|446353356|ref|WP_000431211.1|helicase[Staphylococcusaureus]_gi|253726329|gb|EES95058.1|helicaseCterminaldomainprotein[Staph/    PLERAIAFTR-TIED--------------SKKVSHQFEEVVNEYIGADI-----------
gi|672712068|emb|CDM14336.1|DNAorRNAhelicaseofsuperfamilyII[StaphylococcusepidermidisPM221]/11464    PLERAIAFTR-TIED--------------SKKVSKQFEEVVNEYIGAST-----------
gi|896298990|ref|WP_049279905.1|helicase[Staphylococcusaureus]/11571    PLERAIAFTR-AIED--------------SKKVSKQFEEVVNEYIGAST-----------
gi|541903432|gb|ERH36190.1|hypotheticalproteinSEQU_00085[StaphylococcusequorumUMCCNS924]/11578    PLERAIAFTR-TIED--------------SKKVSKQFEEVVNEYIGSST-----------
```

```
gi|488289747|ref|WP_002360955.1|helicase[Enterococcusfaecalis]_gi|256682216|gb|EEU21911.1|endonucleaseandmethylaseLlaGI[Enteroc    PLERAIAFTR-TIAD--------------SKKVSQQFEEVVNDYIGSEL-----------
gi|494392045|ref|WP_007207894.1|helicase[Enterococcusitalicus]_gi|315483798|gb|EFU74284.1|helicaseCterminaldomainprotein[Enter/    PLERAIAFTR-TIAD--------------SKKVSQQFEEVVNDYIGSEI-----------
gi|446350692|ref|WP_000428547.1|helicase[Bacilluscereus]_gi|228727580|gb|EEL78721.1|RestrictionmodificationsystemLlaBIII[Bacil/    PLERAIAFTR-TIDD--------------SKKVSQQFEEVVNEYIGDTM-----------
gi|488255302|ref|WP_002326510.1|hypotheticalprotein[Enterococcusfaecium]_gi|430480276|gb|ELA57459.1|hypotheticalproteinOGE_0473    PLERAIAFTR-TIAD--------------SKKVTQQFEEVVNEYLGAEL-----------
gi|843253770|ref|WP_047916698.1|helicase[Lactococcuspiscium]_gi|802124677|emb|CEN29502.1|EndonucleaseandmethylaseLlaGI,TypeISPr    PLERAIAFTR-TIEE--------------SKKVSSQFEEVVNEYISEAI-----------
LlaBIII/11578                                                                                                                       PLERAIAFTR-TIEE--------------SKKVSSQFEEVVNEYISEAI-----------
gi|499320531|ref|WP_011011023.1|helicase[Listeriainnocua]_gi|16415805|emb|CAC42019.1|pli0021[ListeriainnocuaClip11262]/11569        PLERAIAFTR-TIEE--------------SKKVSGQFEEVVNEYISEAI-----------
gi|760026859|ref|WP_043709973.1|helicase[Weissellacibaria]_gi|758337165|gb|KIU20061.1|UvsWhelicase[Weissellacibaria]/11567         PLERAIAFTR-TIEE--------------SKKVSDQFEEVVNEYIGTAV-----------
LlaGI/11570                                                                                                                         PLERAIAFTR-TIEE--------------SKKVSSQFEEVVNEYISEAI-----------
gi|499988414|ref|WP_011669132.1|helicase[Lactococcuslactis]_gi|116109019|gb|ABJ74139.1|DNAorRNAhelicaseofsuperfamilyII[Lactococ    PLERAIAFTR-TIEE--------------SKKVSGQFEEVVNEYISEAI-----------
gi|685938360|ref|WP_031670319.1|helicase[Listeriamonocytogenes]_gi|590403387|gb|EXL23261.1|hypotheticalproteinX846_1956[Listeri    PLERAIAFTR-TIEE--------------SKKVSSQFEEVVNEYISEAI-----------
gi|530793219|gb|EQC56240.1|helicase[Lactococcuslactissubsp.cremorisTIFN5]_gi|530793247|gb|EQC56263.1|helicase[Lactococcuslactis    PLERAIAFTR-TIEE--------------SKKVSSQFEEVVNEYISEAI-----------
gi|695270006|ref|WP_032492075.1|hypotheticalprotein[Listeriamonocytogenes]_gi|695212872|ref|YP_009081522.1|hypotheticalproteinp    PLERAIAFTR-TIEE--------------SKKVSSQFEEVVNEYISEAI-----------
gi|504101310|ref|WP_014335297.1|helicase[Leuconostocmesenteroides]_gi|859247905|gb|AKO63885.1|helicase(plasmid)[Leuconostocmese    PLERAIAFTR-TIDE--------------SKKVSDQFEEVVNEYIGTAV-----------
gi|488905190|ref|WP_002816265.1|helicase[Leuconostocmesenteroides]_gi|227351522|gb|EEJ41777.1|helicaseCterminaldomainprotein[L/    PLERAIAFTK-KIKE--------------SKKVAAQFEEVVNEYIGNAV-----------
gi|518121285|ref|WP_019291493.1|MULTISPECIES:hypotheticalprotein[Lactococcus]/11588                                                 PMKRAIAFTR-TIAD--------------SKLITDKFDMVVNDYLSES------------
gi|754857213|ref|WP_042217902.1|helicase[Lactococcusgarvieae]/11549                                                                 PMKRAIAFSR-TIKD--------------SKALTEQFESVVNDYLENTD-----------
gi|640048455|ref|WP_024784762.1|helicase[Streptococcusmutans]/11564                                                                 PMKRAIAFSR-TIND--------------SKRLSDQFEKVVNEYLDSDE-----------
gi|512695361|ref|WP_016476323.1|hypotheticalprotein[Veillonellasp.HPA0037]_gi|512079853|gb|EPD79018.1|hypotheticalproteinHMPREF    PMQRAIAFSR-TIKD--------------SQRISEQFENVVNDYLNSGD-----------
gi|636963514|ref|WP_024403747.1|helicase[Streptococcussuis]/11574                                                                   PMKRAIAFSR-TIDD--------------SKRLSDQFENVVNDYLNSEE-----------
gi|881115312|ref|WP_048802949.1|helicase[Staphylococcuspasteuri]/11573                                                              PMKRAIAFSR-TIKD--------------SKRLSEQFENVVNDYLDNEN-----------
gi|446063996|ref|WP_000141851.1|helicase[Streptococcusmitis]_gi|307618725|gb|EFN97864.1|endonucleaseandmethylaseLlaGI[Streptoco    PMQRAIAFSR-TIED--------------SKRLSTQFENVVNEYLNSDE-----------
gi|488206609|ref|WP_002277817.1|restrictionmodificationsystemLlaBIII[Streptococcusmutans]_gi|449173033|gb|EMB75626.1|restricti/    PMKRAIAFSR-TIKD--------------SQRLSEQFENVVNDYLDSEE-----------
gi|640023290|ref|WP_024781621.1|helicase[Streptococcusmutans]/11566                                                                 PMKRAIAFSR-TIKD--------------SQRLSEQFENVVNDYLDSEE-----------
gi|814946581|ref|WP_046393781.1|helicase[Streptococcusuberis]_gi|810600885|gb|KKF57499.1|helicase[StreptococcusuberisB362]/1156     PMRRAIAFSR-TIAD--------------SKRISEEFENVVNDYLESED-----------
gi|765470762|ref|WP_044720336.1|helicase[Streptococcussuis]/11559                                                                   PMKRAIAFSR-TIAD--------------SKRLSAQFENVVNDYLNSED-----------
gi|654507127|ref|WP_027976443.1|helicase[Streptococcusdevriesei]/11558                                                              PMKRAIAFSR-TIKD--------------SKRLSEQFENVVNDYLNSEE-----------
gi|530812902|gb|EQC72481.1|DNAorRNAhelicaseofsuperfamilyII[Streptococcussp.HSISS2]/11570                                             PMKRAIAFSR-TIKD--------------SQRLSEQFENVVNDYLDSDK-----------
gi|489158846|ref|WP_003068504.1|helicase[Streptococcusintermedius]_gi|355367114|gb|EHG14827.1|hypotheticalproteinHMPREF9682_002    PMKRAIAFSR-TIKD--------------SQRLSAQFENVVNDYLNSED-----------
RM.Lru27782ORF13200P/11620                                                                                                          PMKRAIAFTD-TIEH--------------SKQIADEFNDVVNDYLGEYA-----------
gi|492676546|ref|WP_005919236.1|DNAphosphorothioationsystemrestrictionenzyme[Pediococcusacidilactici]_gi|482517924|gb|EOA08103.    PMKRAIAFTD-TIAH--------------SKEIAKEFNNVVNDYLGKEA-----------
gi|498183116|ref|WP_010497272.1|helicase[Lactobacillusacidipiscis]/11563                                                            PMKRAIAFTD-TIKH--------------SQEIADEFNNVVNDYLGAQA-----------
gi|822100485|ref|WP_046921865.1|helicase[Lactobacillusruminis]_gi|821684442|gb|KLA43321.1|restrictionmodificationsystemLlaBIII/    PMKRAIAFTD-TIKH--------------SEQVAKEFNNVVNDYLGKQA-----------
gi|935565832|ref|WP_054450311.1|helicase[Lactobacilluskunkeei]/11573                                                                PMKRAIAFSD-TIKH--------------SKAISKEFSSVVNEYLDTQS-----------
gi|752296887|ref|WP_041152901.1|helicase[Lactobacilluskunkeei]_gi|751663520|gb|KIM18358.1|helicase[Lactobacilluskunkeei]/11577     PMKRAIAFSD-TIKH--------------SKAISKEFSSVVNEYLDTQS-----------
gi|495745291|ref|WP_008469870.1|helicase[Lactobacillushominis]_gi|394484442|emb|CCI81207.1|RestrictionmodificationsystemLlaBII/    PLKRAIAFTD-TIKH--------------SKTIANEFNQVVNEYLDTQS-----------
gi|918672414|ref|WP_052543134.1|helicase[Lactobacillussp.]/11564                                                                    PMKRAIAFTD-TIKH--------------SKIISNEFNQVVNDYLDSDTDDN--------
gi|799141631|ref|WP_045927261.1|helicase[Lactobacilluskullabergensis]_gi|797155335|gb|KJY59264.1|putativerestrictionendonucleas    PMKRAIAFSD-TIKH--------------SKIIADEFNQVVNDYLDSDDDA---------
gi|504379990|ref|WP_014567092.1|helicase[Lactobacillusjohnsonii]_gi|329666726|gb|AEB92674.1|putativerestrictionendonuclease[Lac    PLKRAIAFTD-TIKH--------------SKTISEEFNQVVNEYLDTQS-----------
gi|489880179|ref|WP_003783646.1|DNAdamageinducibleprotein[Actinomycesnaeslundii]_gi|399901561|gb|EJN84440.1|typeIIIrestriction/    PMRTAVAFAR-DIKTS--------------DSFASSFSKVVEDYRLMLTDES------EG
gi|496524497|ref|WP_009232780.1|DNAdamageinducibleprotein[Actinomycessp.oraltaxon849]_gi|365265413|gb|EHM95177.1|hypotheticalp/    PMRTAVAFAR-DIKTS--------------KRFASSFRNVAEDYRLTLTDEP------EV
gi|915397782|ref|WP_050783167.1|damageinducibleprotein[Actinomycesoris]/11694                                                       PMRTAVAFAR-DIKTS--------------KRFASSFRNVAEDYRLTLTDDP------EV
gi|502925248|ref|WP_013160224.1|helicase[Propionibacteriumfreudenreichii]_gi|296921272|emb|CBL55824.1|helicase[Propionibacteriu    PMQRAVAFAQ-NIKAS--------------KGFAQAFPELTSALADDADED---------
gi|732164352|emb|CEG87481.1|helicase[Propionibacteriumfreudenreichii]/11618                                                         PMKRAVAFLS-SIKAS--------------RRFAEALPQAVDTLTAREQDSG--------
gi|739047402|ref|WP_036925058.1|damageinducibleprotein[Propionibacteriumthoenii]/11596                                              PMQRAVAFAA-NIKAS--------------KAFAGALPQVVDQVIGDDA-----------
gi|739112839|ref|WP_036982533.1|damageinducibleprotein[Propionibacteriumjensenii]/11594                                             PMQRAVAFAA-NIKAS--------------KAFAGALPQVVDQVIGDDA-----------
gi|918023558|ref|WP_052314121.1|damageinducibleprotein[Nocardiathailandica]/11612                                                   PMRRAVAFAK-DIRSS--------------KAASAAFPALIDRALQDDA-----------
gi|498380271|ref|WP_010694427.1|DNAdamageinducibleprotein[Saccharopolysporaspinosa]/11605                                           PMRTAVAFAR-DINAS--------------QRAAEGFPILVDRVLEDENPD---------
gi|749991459|ref|WP_040339809.1|damageinducibleprotein[CandidatusBlastococcusmassiliensis]/11597                                    PMRTAVAFAQ-HIKAS--------------KTAAAAFPDLADRAQIDA------------
gi|754177801|ref|WP_041796231.1|damageinducibleprotein[Modestobactermarinus]/11592                                                  PMRTAVAFAQ-HIKAS--------------KAAAAAFPDLADRAQIDA------------
gi|504188248|ref|WP_014375350.1|DNAdamageinducibleprotein[Blastococcussaxobsidens]_gi|378782790|emb|CCG02456.1|putativeHelicas/    PMRTAVAFAQ-HIKAS--------------KAAASAFPDLADRAQIDA------------
gi|946476713|gb|KRF26798.1|damageinducibleprotein[Phycicoccussp.Soil803]/11609                                                      PMKTAVAFAA-NIKAS--------------KAAREAFPSIVDRLISTYADEQ--------
gi|700176672|gb|KGN37509.1|DNAdamageinducibleprotein[KnoelliasubterraneaKCTC19937]/11633                                            PMKTAVAFAA-NIKAS--------------KAAREAFPSIVDRLISTYAEDE--------
gi|700168841|gb|KGN29914.1|hypotheticalproteinN802_10215[KnoelliasinensisKCTC19936]/11631                                           PMKTAVAFAA-NIKAS--------------KAATEAFPSIVDGLISTYAEEE--------
gi|946426241|gb|KRE76610.1|damageinducibleprotein[Arthrobactersp.Soil763]/11606                                                     PMRRAVAFAG-TIKAS--------------KKVEGMFSNLVDQYVAVHETDD--------
gi|517318525|ref|WP_018500927.1|hypotheticalprotein[Frankiasp.BCU110501]/11592                                                      PMRRAIAFAG-NIKDS--------------KRIEDLFQAVTDYYVGATDQEQ--------
gi|499072429|ref|WP_010851327.1|Helicase[Tetrasphaeraelongata]_gi|478756579|emb|CCH71501.1|putativeHelicase[Tetrasphaeraelongat    PMRRAVAFAG-TIKDS--------------KHIESLFAETVRQYAAAHDLDD--------
gi|750080666|ref|WP_040385350.1|damageinducibleprotein[Demetriaterragena]/11625                                                     PMKRAVAFAK-DIKTS--------------KSVAEAFPTIVDRHVDTVDLTE--------
gi|752558292|ref|WP_041229523.1|damageinducibleprotein[Dermacoccusnishinomiyaensis]_gi|664689315|gb|AIF41904.1|damageinducibl/1    PMQRAVAFAK-DIKTS--------------RAIADAFPTIVQRHITEGEFAD--------
gi|829089213|ref|WP_047310701.1|damageinducibleprotein[Dermacoccussp.PE3]_gi|828384812|gb|KLO63770.1|damageinducibleprotein[D/1    PMKRAVAFAK-DIKTS--------------KAIAQAFPDVVNKHVAAVDLPD--------
gi|748756635|ref|WP_040014750.1|damageinducibleprotein[Dermacoccussp.Ellin185]/11596                                                PMKRAVAFAK-DIKTS--------------KAIAQAFPDVVNKHVAAVDLPD--------
gi|916534724|ref|WP_051141848.1|damageinducibleprotein[Actinomycesneuii]/11632                                                      PMRRAVAFAK-DIKTS--------------KRIAHDFADLVRIHLQDLD-----------
gi|921140030|ref|WP_053090276.1|damageinducibleprotein[Rothiamucilaginosa]/11616                                                    PMKRAVAFSR-SIKDS--------------QKIEREFEDLVRIHLRNRE-----------
gi|896406751|ref|WP_049343749.1|damageinducibleprotein[Rothiamucilaginosa]/11647                                                    PMKRAVAFSR-SIKDS--------------QKIEREFEDLVRINLRNRE-----------
gi|723608234|gb|AIY01610.1|putativehelicase[Arthrobactersp.PAMC25486]/11613                                                         PMRRAVAFNR-SIPAS--------------KKIEAEFQELVRVHLTDLA-----------
gi|946394527|gb|KRE45160.1|damageinducibleprotein[Arthrobactersp.Soil736]/11616                                                     PMKRAVAFNR-DIKSS--------------KLVESEFEDLVRVHLTNLD-----------
gi|515765304|ref|WP_017197904.1|hypotheticalprotein[Arthrobactersp.M2012083]/11648                                                  PMKRAVAFNR-DIKSS--------------RLVESEFEELVRVHLTNLD-----------
```

gi|945315587|gb|KQT73127.1|damageinducibleprotein[Microbacteriumsp.Leaf436]/11612 PMQRAVMFAK-DIATS--------------RKLADAFQQVV---------------EGEI
gi|759908497|ref|WP_043594599.1|damageinducibleprotein[Frigoribacteriumsp.MEB024]_gi|758271359|gb|KIU03346.1|damageinduciblep/1 PLRRAVAFSR-SIKDS--------------KAVQGLLDGYDGVDGVRSD-----------
gi|945072850|gb|KQR34237.1|hypotheticalproteinASF75_00055[Curtobacteriumsp.Leaf154]/11597 PLRRAVAFSR-SIKDS--------------KTVRGFLDGYEGVDGVRPD-----------
gi|551263152|ref|WP_022883363.1|DNAdamageinducibleprotein[Glaciibactersuperstes]/11621 PLRRAVAFSR-SIKDS--------------KKVQSMLNGYDGQFTQPHGVSFASEAEHAA
gi|945452341|gb|KQV08491.1|hypotheticalproteinASC63_10170[Leifsoniasp.Root112D2]/11623 PMRRAVAFSR-SIKDS--------------QKVSSMLDGFSGGPAASGVPSFPTAAEQER
gi|742759483|gb|KIC69912.1|damageinducibleprotein[Kocuriarhizophila]/11610 PMRRAVAFAA-NIRES--------------QRVADALERVSEQLTQDNP-----------
gi|737919995|ref|WP_035885304.1|damageinducibleprotein[Kocuriarhizophila]/11611 PMRRAVAFAA-NIRES--------------QRVADALERVSEQLTQDNP-----------
gi|737997701|ref|WP_035959325.1|damageinducibleprotein[Kocuriamarina]_gi|725823884|gb|KHE75603.1|damageinducibleprotein[Kocur/1 PMRRAVAFAS-NIRES--------------QRVADALERVSEQLTQDNP-----------
gi|664521070|ref|WP_031037650.1|damageinducibleprotein[Streptomycessp.NRRLWC3774]/11624 PMKRAVAFAR-SIADS--------------KSIADRFNEVVDAYDDADD-----------
gi|500101034|ref|WP_011777041.1|DNAdamageinducibleprotein[Arthrobacteraurescens]_gi|119951547|gb|ABM10457.1|putativeHelicase[A/ PMKRAVAFAQ-NIKES--------------KKIASMFAEVTDQLAHEHE-----------
gi|916871953|ref|WP_051479009.1|damageinducibleprotein[Arthrobactersp.H5]/11588 PMRRAVAFAQ-NIKES--------------KKIAGMFADVTEQLAREQE-----------
gi|612084649|gb|EZP43369.1|TypeIIIrestrictionenzyme,ressubunit[Micrococcusluteus]/11625 PMRRAVAFAR-NIKES--------------KQLALQFEQVSRQLATSTD-----------
gi|759739708|ref|WP_043450422.1|damageinducibleprotein[Arthrobacterphenanthrenivorans]_gi|742758264|gb|KIC68716.1|damageinduc/1 PMRRAVAFAR-NIKES--------------KKLAADFELVGRQLLINQS-----------
gi|636845448|ref|WP_024366823.1|damageinducibleprotein[Arthrobactersp.TB26]/11605 PMNRAVAFAR-NIKES--------------KKLAEQFELIGRQLLVEDD-----------
gi|500009025|ref|WP_011689743.1|DNAdamageinducibleprotein[Arthrobactersp.FB24]_gi|116613051|gb|ABK05760.1|typeIIIrestrictionen/ PMNRAVAFAR-NIKES--------------KKLAEQFELIGRQLLVEDD-----------
gi|821249847|gb|KKZ12083.1|hypotheticalproteinTQ37_06090[CandidatusSynechococcusspongiarum15L]/11436 PMSRAIAFCN-LVKSS--------------RQLEATIGEVATAYGQQVCG----------
gi|739446902|ref|WP_037306885.1|damageinducibleprotein[Ruegeriahalocynthiae]/11592 PMRRAIAFCK-DIRSS--------------KLIAREFAAVVDEYNGASVV----------
gi|640620081|ref|WP_025048131.1|damageinducibleprotein[Sulfitobactermediterraneus]_gi|751956650|gb|KIN77213.1|TypeIIIrestricti/ PMRRAIAFCK-DIRSS--------------KLIEREFGAVVDEFNGTTVL----------
gi|740247634|ref|WP_038088583.1|damageinducibleprotein[Thiobacillusprosperus]_gi|684049748|gb|KFZ90853.1|putativehelicase[Thio/ PMRRAVAFSK-SIKAS--------------HEATKVFADLVRLYRQEHE-----------
gi|518420321|ref|WP_019590528.1|hypotheticalprotein[Thioalkalivibriosp.ALE20]/11603 PMQRAVAFSR-SIKAS--------------KQTTEAFSSMAELYSNALP-----------
gi|500156826|ref|WP_011831496.1|DNAdamageinducibleprotein[Methylibiumpetroleiphilum]_gi|124261892|gb|ABM96881.1|adeninespecifi/ PMKRAVAFSR-SIRDS--------------KQTTEVFGQLVGLYHRMHA-----------
gi|769978537|ref|WP_045094522.1|damageinducibleprotein[Legionellafallonii]_gi|698841736|emb|CEG55682.1|Putativeproteinwithboxh/ PMRRAIAFSR-SIKDS--------------KQKVEIFSALLERYHQLPN-----------
gi|553752541|ref|WP_023085344.1|hypotheticalprotein[Pseudomonasaeruginosa]_gi|552201952|gb|ERU62653.1|hypotheticalproteinQ088_0 PMRRAVAFSK-SIKLS--------------RQTTEIFKDLVGVYKQAHD-----------
gi|880980418|ref|WP_048704461.1|damageinducibleprotein[Thauerahumireducens]_gi|875132785|gb|KMU70944.1|damageinducibleprotein/1 PMRRAVAFSK-SIKLS--------------KQTTDIFKDLVAVYQQAHD-----------
gi|740135732|ref|WP_037982815.1|damageinducibleprotein[Thauerasp.27]/11603 PMRRAVAFSK-SIKLS--------------KQTTDIFKDLVAVYQQAHD-----------
gi|738017371|ref|WP_035978332.1|damageinducibleprotein[Burkholderiaglumae]/11380 PMRRAVAFSR-SIKDS--------------QQMRDTFTRLVGLYQEAHD-----------
gi|498506003|ref|WP_010806671.1|typeIIIrestrictionenzyme,ressubunit[Pandoraeasp.SD62]_gi|490314561|gb|EON12879.1|typeIIIrestri/ PMRRAVAFSK-SIKDS--------------QQMRDTFTRLVGLYQEAHD-----------
gi|500117748|ref|WP_011793753.1|DNAdamageinducibleprotein[Acidovoraxcitrulli]_gi|120587742|gb|ABM31182.1|typeIIIrestrictionenz/ PMRRAVAFSK-SIKDS--------------KQMQDVFGQLVQTYQQVHQQQG--------
gi|651617328|ref|WP_026611444.1|damageinducibleprotein[Methylocaldumszegediense]/11601 PMRRAVAFSK-SIKDS--------------RQTTEIFSQLVELYRQSQQ-----------
gi|737210208|ref|WP_035194994.1|damageinducibleprotein[Acidithiobacillusferrivorans]_gi|669953220|emb|CDQ11755.1|Adeninespecif/ PMRTAVAFAG-TIPHS--------------KLLAREFMAVVDELREDE------------
gi|935697386|ref|WP_054506180.1|damageinducibleprotein[Achromobactersp.]_gi|928716153|emb|CUK12836.1|Hefnuclease[Achromobacter/ PMRTALAFAQ-SIKHS--------------KLVRSEFERISNDLSDDL------------
gi|429502517|gb|ELA00827.1|adeninespecificDNAmethyltransferase[Cupriavidussp.HMR1]/11599 PMRTALAFAQ-SIKHS--------------KLVRSELERISNDLSDDL------------
gi|506356642|ref|WP_015876361.1|DNAdamageinducibleprotein[Burkholderiaglumae]_gi|237877158|gb|ACR29491.1|AdeninespecificDNAmet/ PMRTAIAFAQ-SIKHS--------------KLLRSEFERISNDLSDDL------------
gi|696406787|ref|WP_032978003.1|damageinducibleprotein[Bordetellahinzii]_gi|625335076|gb|KCB49343.1|typeIIIrestrictionenzyme,r/ PMRTAIAFAQ-SIKHS--------------KLLTTEFERISDDLGDQI------------
gi|817529125|gb|KKO72587.1|damageinducibleprotein[Kerstersiagyiorum]/11592 PMRTAIAFAQ-SIKHS--------------KLLTAEFERISDDLGDQI------------
gi|737048910|ref|WP_035044118.1|damageinducibleprotein[Aquabacteriumsp.NJ1]_gi|699052896|gb|KGM38706.1|damageinducibleprotein/1 PMRTAIAFAQ-SIKHS--------------KLLTAEFERISNDLGDDI------------
gi|822492519|ref|WP_046933319.1|MULTISPECIES:damageinducibleprotein[Xanthomonas]_gi|821703525|gb|KLA61196.1|damageinduciblepr/1 AMRTAIAFAQ-SIKHS--------------KLLTAEFERISNDLADEI------------
gi|751252861|ref|WP_040963740.1|damageinducibleprotein[Xanthomonasfuscans]_gi|749808363|gb|KIJ03887.1|damageinducibleprotein[/1 PMRTAIAFAQ-SIKHS--------------KLLTAEFEKVSNDLGDQI------------
gi|496774980|ref|WP_009368792.1|DNAdamageinducibleprotein[Bilophilasp.4_1_30]_gi|345039902|gb|EGW44205.1|hypotheticalproteinHM/ PMRTALAFAG-NIKHS--------------KLVAKEFSRIADELGGKV------------
gi|499241912|ref|WP_010939452.1|DNAdamageinducibleprotein[Desulfovibriovulgaris]_gi|46580580|ref|YP_011388.1|adeninespecificDN/ PMRTALAFAG-NIKNS--------------KRLAGEFVRIAAELGDAV------------
gi|504274913|ref|WP_014462015.1|DNAdamageinducibleprotein[Bacillusmegaterium]_gi|345446919|gb|AEN91936.1|AdeninespecificDNAmet/ PMKRAVAFTK-SIKDS--------------KLIADMFENLVDEFIQLD-D----------
gi|504895966|ref|WP_015083068.1|typeIIrestrictionenzymeandmethyltransferasesubtypeG[Anabaenasp.90]_gi|413972424|gb|AFW96512.1|t PMKRAVAFCR-TIEDS--------------EKIVKLFAKNINDYQKLNPE----------
gi|518327765|ref|WP_019497972.1|hypotheticalprotein[Calothrixsp.PCC7103]/11600 PMRRAVAFSR-SIKDS--------------QKIEKLFSEIIEQYRQTH-P----------
gi|652765503|ref|WP_027079917.1|damageinducibleprotein[Luteimonasmephitis]/11593 PMHRAVAFAK-DIKAS--------------IKVSEQFPVIVNQYIDGQ-D----------
gi|896539951|ref|WP_049442123.1|damageinducibleprotein[Stenotrophomonasmaltophilia]/11591 PMRRAVAFAR-DIKSS--------------KHLAQLFPEVVDQYIDGS-D----------
gi|500217900|ref|WP_011888043.1|DNAdamageinducibleprotein[Geobacillusthermodenitrificans]_gi|134267954|gb|ABO68149.1|Conserved/ PMKRAVAFTT-SIKNS--------------KKVTELFEQTVEDYCRSLPE----------
gi|696570298|ref|WP_033102014.1|damageinducibleprotein[Thermoactinomycesdaqus]/11594 PMRRAVAFSN-SIKNS--------------KRVTELFARTIEDYCASLSN----------
gi|251772067|gb|EES52637.1|putativehelicase[Leptospirillumferrodiazotrophum]/11618 PMRRAVAFSR-SIKDS--------------DALAKRFSHIVDQYLESLSV----------
gi|738124101|ref|WP_036082108.1|damageinducibleprotein[Leptospirillumferriphilum]_gi|685243306|gb|KGA94177.1|putativehelicase[/ PMRRAVAFSR-SIKDS--------------DALAKRFSHIVDQYLESLSM----------
gi|558699223|ref|WP_023524766.1|helicase[Leptospirillumsp.GroupII'CF1']_gi|206603764|gb|EDZ40244.1|Putativehelicase[Leptospiri/ PMRRAVAFSR-SIKDS--------------DALAKRFSHIVDQYLESLSV----------
gi|406774462|gb|AFS53887.1|putativehelicase[LeptospirillumferriphilumML04]/11610 PMKRAVAFAR-SIADS--------------KQVSSQFDHIVTQYLDSFDP----------
gi|740719463|ref|WP_038504749.1|damageinducibleprotein[Leptospirillumferriphilum]_gi|640838125|gb|AIA30173.1|damageinduciblep/1 PMKRAVAFAR-SIADS--------------RQISDQFDHIVTQYLDSFDP----------
gi|517810766|ref|WP_018980974.1|hypotheticalprotein[Saccharibacterfloricola]/11733 QMKRAVAFCG-AIEEDT--------ALVSSKKIARVFSHVANLYDQENG-----------
gi|502471675|ref|WP_012798533.1|helicase[Slackiaheliotrinireducens]_gi|256791761|gb|ACV22431.1|predictedhelicase[Slackiaheliotr RLKNAVAFCS-TIRES--------------QRIEKFFTRTVASYINYEREEKG-------
gi|657871713|ref|WP_029576297.1|hypotheticalprotein[Bifidobacteriumthermacidophilum]_gi|673007521|gb|KFJ02535.1|helicase[Bifido PLHHAIAFAS-SIKAS--------------KQLSEEFQSVIDAYTNNETDSSQP------
gi|673004757|gb|KFI99791.1|DNAhelicaserestrictionenzymetypeIIIRessubunit[Bifidobacteriumthermacidophilumsubsp.porcinum]/11682 PLHHAIAFAS-SIKAS--------------KQLSAEFQSVIDAYTNNETDSSQP------
gi|917316092|ref|WP_051922804.1|hypotheticalprotein[Bifidobacteriumstellenboschense]_gi|673003774|gb|KFI98812.1|helicase[Bifido VLHHAIAFAA-SIKDS--------------KALSAKFRTVIDDYTRNLEDTDAN------
gi|705415694|ref|WP_033502332.1|hypotheticalprotein[Bifidobacteriumpseudolongum]_gi|672982411|gb|KFI77685.1|DNAdamageinducible/ VLHHAIAFAA-SIKDS--------------KRLTDKFQTVIDDYTESLEDTSEN------
gi|916591418|ref|WP_051198509.1|hypotheticalprotein[Bifidobacteriumsp.AGR2158]/11704 VLHHAIAFAS-TIKDS--------------ERLKDKFQAVIDDYTSSLEDTEEN------
gi|772242982|gb|AJW78255.1|hypotheticalproteinVO01_03165[Clavibactermichiganensissubsp.insidiosus]/11642 PMRKAIAFAK-DIKTS--------------KGVANDFETLVDG--HLSN---IL------
gi|916649818|ref|WP_051256909.1|damageinducibleprotein[Actinomycesgraevenitzii]/11677 PMRRAVAFCR-DIKAS--------------KQVTDQFPDLVNGTSGLSN---LT------
gi|496504578|ref|WP_009212861.1|helicase[Actinomycessp.oraltaxon180]_gi|315312695|gb|EFU60776.1|helicase[Actinomycessp.oraltaxo PMRRAVAFCK-NIKAS--------------KQVTEQFPDLVNGPHGLSD---LS------
gi|494862206|ref|WP_007588306.1|helicase[Actinomycessp.ICM39]_gi|398362799|gb|EJN46470.1|typeIIIrestrictionenzyme,ressubunit[Ac PMRRAVAFCK-DIKAS--------------KAVATQFPDLVNGPFGLSD---LS------
gi|881057111|ref|WP_048774974.1|damageinducibleprotein[Sanguibacterkeddieii]/11697 PMRRAVAFCK-DIKAS--------------KAVAMQFPDLVNGPFGLSD---LS------
gi|881020459|ref|WP_048740808.1|damageinducibleprotein[Kytococcussedentarius]/11704 PMRRAVAFCK-DIKAS--------------KAVATQFPDLVNGPFGLSD---LS------
gi|881052313|ref|WP_048770555.1|damageinducibleprotein[Cellulomonasflavigena]/11705 PMRRAVAFCK-DIKAS--------------KAVATQFPDLVNGPFGLSD---LS------
gi|490108682|ref|WP_004009424.1|helicase[Mobiluncuscurtisii]_gi|315490465|gb|EFU80089.1|helicaseCterminaldomainprotein[Mobilun/ PMRRAVAFAK-DIKTS--------------KQITSEFPILVRD--QLQD---LE------

```
gi|490111935|ref|WP_004012603.1|helicase[Mobiluncusmulieris]_gi|227843812|gb|EEJ53982.1|helicaseCterminaldomainprotein[Mobilun/  PMRRAVAFVK-DIKTS--------------KLVATEFQNLVNQ--HLQN---LT------
gi|490114228|ref|WP_004014848.1|helicase[Mobiluncusmulieris]_gi|269934432|gb|EEZ90993.1|helicaseCterminaldomainprotein[Mobilun/  PMRRAVAFVK-DIKTS--------------KLVATEFQNLVNQ--HLQN---LT------
gi|493333551|ref|WP_006290636.1|helicase[Parascardoviadenticolens]_gi|315119487|gb|EFT82620.1|helicaseCterminaldomainprotein[P/  PLHRAVGFCS-TIKES--------------KRMDEVFANVVRHYQEASGDT---------
gi|495817067|ref|WP_008541646.1|helicase,partial[Sutterellaparvirubra]_gi|378598583|gb|EHY31712.1|helicaseprotein,partial[Sutte  LMKRAVGFAQ-VIEPPK---PGQNLDRTSSKLFTANFAKTIEDFKRDHFNT-LNEKNR--
gi|491572696|ref|WP_005430278.1|helicase[Sutterellawadsworthensis]_gi|319805116|gb|EFW01943.1|DNAhelicaserestrictionenzymeTypeI  PMRRAVGFAQ-IIEPNI-----KYLDRTSSKRFTENFESTIEEFKDQQFEE-LSKKDK--
gi|748686321|ref|WP_039944445.1|hypotheticalprotein[Thermicanusaegyptius]/11573  AARRAVVFAGQAIRDS--------------RNFAKAFQNTVSAFTEKTG-----------
gi|916875698|ref|WP_051482754.1|hypotheticalprotein[Thioalkalivibriosp.HK1]/11366  PLRRAIAFTN-TIGDS--------------KRLEKHWPKIVERAQAD-----LP------
gi|821248604|gb|KKZ10960.1|hypotheticalproteinTE42_09075[CandidatusSynechococcusspongiarumSP3]/11598  PMGRAIAFCN-LVKSS--------------KALEASIGDVAEAYGRQV-----C------
gi|737409843|ref|WP_035390956.1|hypotheticalprotein[Ferrimicrobiumacidiphilum]_gi|765544266|gb|KJE76241.1|typeIrestrictionenzym  PMKTGVIFVN-TINGS--------------KEVQKTFTDVAAQAQLQ-G-----------
gi|654906925|ref|WP_028358281.1|damageinducibleprotein[Brackiellaoedipodis]/11679  PMKRAVAFCQ-VIEDTY---KGK-GHRVSSKLIARMFSEVVKAYQAQEKAVTPD------
gi|502809901|ref|WP_013044877.1|hypotheticalprotein[CandidatusPuniceispirillummarinum]_gi|292663146|gb|ADE38247.1|hypotheticalp  KLKRAVSFSS-TIKKS--------------KHVRDHFQAVVDKMDEQE------------
gi|251771370|gb|EES51950.1|typeIIIrestrictionenzyme,ressubunit[Leptospirillumferrodiazotrophum]/11628  PMKSGVAFSH-TIKSS--------------RELAACFQEVVNLYLDNHGDG---------
gi|498328480|ref|WP_010642636.1|DNAdamageinducibleprotein[Acidithiobacillusthiooxidans]/11627  AMKRAVGFCK-SIKASE--------------QVRDAFGKVVALALQEEGSEDADS-----
gi|647633864|ref|WP_025906698.1|damageinducibleprotein[Prochlorococcussp.scB245a_519B7]/11622  PMKTALAFCR-RISQS--------------QTFAEKFADVIEEYNSN-----EVI-----
gi|647619599|ref|WP_025893729.1|damageinducibleprotein[Prochlorococcussp.scB241_527N11]/11621  PMKKALAFCQ-NIRTS--------------KIFSSEFVEVIDEYTSN-----EKI-----
gi|755034887|ref|WP_042387498.1|hypotheticalprotein[Clostridiumsp.CL6]/11606  PMKRAVAFCN-QIKDS--------------KRLVEMFNVIQEHIRGN-----NP------
gi|498139798|ref|WP_010453954.1|helicase[SuccinivibrionaceaebacteriumWG1]/11662  LMHRAMIFCP-KIGKED---V-----PGSSKNIAHILPKICDMYTKN-----MS------
gi|527501386|gb|EPT34571.1|typeIIIrestrictionenzyme,ressubunit[Bacteroidetesbacteriumoraltaxon272str.F0290]/11789  MMKRAVVFCS-TINRSK---RF---SGIASTDFAMQFPELAKYYKEN-----DVE-----
gi|496047275|ref|WP_008771782.1|helicase[Bacteroidessp.2_1_33B]_gi|262296443|gb|EEY84373.1|helicaseCterminaldomainprotein[Bact/  LMRRAVAFCS-TINPSE---RG---SGISSKGFAAVMPTMVRKYKES-----LS------
PDI8503III/11664  LMRRAVAFCS-TINPSE---RG---SGISSKGFAAVMPTMVRKYKES-----LS------
gi|738719431|ref|WP_036613715.1|helicase[Parabacteroidesdistasonis]_gi|649542518|gb|KDS49387.1|restrictionendonucleasefamilypro  LMRRAVAFCS-TINPSE---RG---SGISSKGFAAVMPTMVRKYKES-----LS------
gi|659417033|gb|KEJ86133.1|hypotheticalproteinHMPREF1002_00341[Porphyromonassp.31_2]/11656  LMRRAVAFCS-TINPSE---RG---SGISSKGFAAVMPTMVRKYKES-----LS------
gi|567456807|gb|ETK11358.1|hypotheticalproteinT235_15845[Tannerellasp.oraltaxonBU063isolateCell8/11]/11433  LMHRALAFTH-KIGQAD---E-----PGTSKNIATVLPKISAIYNES-----LP------
gi|545440279|ref|WP_021677877.1|helicase[Porphyromonasgingivalis]_gi|543985765|gb|ERJ89108.1|helicaseprotein[Porphyromonasgingi  LMRRALAFTH-KIGHSD---E-----PGTSRNIEHVLPRISEIYNES-----LP------
gi|497438056|ref|WP_009752254.1|helicase[Prevotellasp.oraltaxon473]_gi|429144866|gb|EKX87968.1|helicaseprotein[Prevotellasp.ora  LMRRALAFTH-KIGKAD---E-----PGSSRNIEHVLPRISALYNES-----LS------
gi|490423474|ref|WP_004295683.1|MULTISPECIES:helicase[Bacteroides]_gi|156110098|gb|EDO11843.1|helicaseCterminaldomainprotein[B/  VMHRAIAFCS-AIGNNH---S-----PGTSVNTAEVLPTICEKYRDS-----IS------
gi|941777229|ref|WP_055173506.1|helicase[Bacteroidescaccae]_gi|933263395|emb|CUQ00567.1|Predictedhelicase[Bacteroidescaccae]/1/  MMRRAVAFCS-AIGNET---K-----AGTSKYVASVLPRISGKYEEN-----LD------
gi|492413898|ref|WP_005836892.1|helicase[Bacteroidesuniformis]_gi|392678824|gb|EIY72229.1|hypotheticalproteinHMPREF1072_03583[B  MMRRAVAFCS-AIGNET---K-----AGTSKYVASVLPRISGKYEEN-----LD------
gi|695455095|ref|WP_032566191.1|helicase[Bacteroidesfragilis]_gi|596095426|gb|EXZ59996.1|restrictionendonucleasefamilyprotein[B  MMRRAVAFCS-AIGNET---K-----AGTSKYVASVLPRISGKYEEN-----LD------
gi|933227941|emb|CUQ02348.1|Predictedhelicase[Bacteroidesuniformis]/11658  MMRRAVAFCS-AIGNET---K-----AGTSKYVASVLPRISRKYEES-----ID------
gi|494823013|ref|WP_007558421.1|helicase[Bacteroidesplebeius]_gi|198272167|gb|EDY96436.1|helicaseCterminaldomainprotein[Bacter/  MMRRAVAFCS-AIGNET---K-----AGTSKYVASVLPRISGKYEEN-----LD------
gi|495427212|ref|WP_008151909.1|helicase[Parabacteroidesjohnsonii]_gi|218222381|gb|EEC95031.1|helicaseCterminaldomainprotein[P/  MMRRAVAFCS-SIDRSA---SR---TGIASKYVASVLPQISEKYDEN-----LD------
gi|933196855|emb|CUO94545.1|typeIrestrictionenzymeEcoKIsubunitR[Bacteroidesuniformis]_gi|933220098|emb|CUN86874.1|typeIrestrict  MMRRAVAFCS-SIDKSA---SR---TGIASKYVASVLPQISEKYDKN-----LD------
gi|823687748|ref|WP_047101121.1|helicase[Brachyspirahyodysenteriae]_gi|823334537|gb|KLI17932.1|helicase[Brachyspirahyodysenteri  PMKRAVAFCQ-SIKVS--------------KKITDIFNDISKDYYNS-----LS------
gi|496436691|ref|WP_009145538.1|helicase[Phascolarctobacteriumsuccinatutens]_gi|322414007|gb|EFY04839.1|helicaseCterminaldomai/  LMHRAVAFCS-KISVS--------------KVISNSFTNFGKSIQEN-----FQ------
gi|503372880|ref|WP_013607541.1|helicase[Sphaerochaetaglobosa]_gi|324026933|gb|ADY13692.1|typeIIrestrictionproteinressubunit[S  LMHSAVAFCQ-NIKIS--------------KRTVEAMNACRDAYYKT-----LP------
gi|505821748|ref|WP_015707145.1|helicase[Treponemaprimitia]_gi|299483503|gb|ADJ19584.1|helicasedomaincontainingprotein[Trepone/  PMHSAVAFCQ-NIAIS--------------KATAEAFNDVREAYFES-----LT------
gi|505822741|ref|WP_015708134.1|helicase[Treponemaprimitia]_gi|333739639|gb|AEF85129.1|endonucleaseandmethylaseLlaGI[Treponemap  PMHSAVAFCQ-NIAIS--------------KATAEAFNDVREAYFES-----LT------
gi|488791994|ref|WP_002704400.1|helicase[Treponemasaccharophilum]_gi|380767691|gb|EIC01689.1|typeIIrestrictionproteinressubuni  PMKSAVAFCS-NIENS--------------KYLRDSFNVCQKAYYEN-----MS------
gi|524671454|emb|CDD81750.1|putativeseptumsitedeterminingproteinMinC[Bacteroidessp.CAG:462]/11642  PMRRAVAFCS-TIKAS--------------KATARVFTDCKSAYMED-----VS------
gi|524573865|emb|CDC95766.1|putativeseptumsitedeterminingproteinMinC[Alistipessp.CAG:268]/11639  PMRRAVAFCS-SIKAS--------------KAIANAFTDYKDLYMED-----IR------
gi|658617970|ref|WP_029759328.1|helicase[Fusobacteriumnucleatum]/11606  PMKRAVAFCR-DIKTS--------------KRITNNLNNYSKDYILS-----LK------
gi|494412037|ref|WP_007216782.1|helicase[Bacteroidescellulosilyticus]_gi|392638921|gb|EIY32752.1|hypotheticalproteinHMPREF1062_  PMRRAVAFCQ-SIKIS--------------KQTVTELNGCRESYYDT-----LS------
gi|383107102|gb|AFG37435.1|putativehelicase[SpirochaetaafricanaDSM8902]/11616  PMKRAVAFCQ-KIADS--------------KTISHNFNETSDIYLDS-----LS------
SAF8902III/11616  PMKRAVAFCQ-KIADS--------------KTISHNFNETSDIYLDS-----LS------
gi|652538894|ref|WP_026932796.1|helicase[Gramellaechinicola]/11633  PMKRAVAFCQ-NINVS--------------RKITRTFNDVSEQYLDS-----IP------
gi|639238223|ref|WP_024568839.1|helicase[Elizabethkingiaanophelis]/11628  PMKRAVAFCQ-NIKVS--------------NHITNTLNQTKGIYLDS-----LT------
gi|652520381|ref|WP_026914582.1|helicase[Gramellaportivictoriae]/11623  PMKRAVAFCQ-NIKVS--------------KRITGTFNDTSEIYLDS-----VK------
gi|494922183|ref|WP_007648219.1|Helicase[Formosasp.AK20]_gi|469825489|gb|EMQ95550.1|Helicasedomainprotein[Formosasp.AK20]/11626  PMKRAVAFCQ-NIKIS--------------KKITNTFNDTSDVYLDS-----VE------
gi|522083382|ref|WP_020594591.1|hypotheticalprotein[Spirosomapanaciterrae]/11637  PMRRAVAFCQ-SIAAS--------------RKITAIYNTASVPYISE-----LP------
gi|490455352|ref|WP_004326157.1|helicase[Bacteroidesovatus]_gi|392669277|gb|EIY62768.1|hypotheticalproteinHMPREF1070_03635[Bact  PMKRAVAFCP-NIQAS--------------KKITNTFNSTLESYYSE-----LS------
gi|499516035|ref|WP_011202675.1|helicase[Bacteroidesfragilis]_gi|53713270|ref|YP_099262.1|helicasedomainprotein[Bacteroidesfrag  PMKRAVAFCP-NIQAS--------------KKITNTFNSTLESYYSE-----LS------
gi|651338332|ref|WP_026461320.1|helicase[Adhaeribacteraquaticus]/11620  PMRRALAFCR-NIDES--------------KKITANFMTATDSYIDS-----LP------
gi|806964642|gb|AKC17139.1|helicase[Flavobacteriumpsychrophilum]/11639  PMKRAVAFCQ-SIKVS--------------KKITNTFNTTSDAYINS-----LP------
gi|752684374|ref|WP_041332322.1|helicase[Flavobacteriumpsychrophilum]_gi|666882889|gb|AIG30715.1|helicase[Flavobacteriumpsychro  PMKRAVAFCQ-SIKVS--------------KKITNTFNTTSDAYINS-----LP------
gi|933196595|emb|CUP04894.1|DNAorRNAhelicasesofsuperfamilyII[Bacteroidesuniformis]/11631  PMKRAVAFCQ-SIRVS--------------KRITETYNTATSAYVES-----LP------
gi|670509109|ref|WP_031456918.1|helicase[Flavobacteriumchungangense]/11625  PMKRAVAFCS-NIKVS--------------RKITETYNTASDSYIDS-----LP------
     : *

gi|490190857|ref|WP_004089451.1|hypotheticalprotein[Helicobacterbilis]_gi|476630638|gb|EMZ37196.1|hypotheticalproteinC826_02193  -----------------------T-KSHTINIDHIDGTMNANTRFTKLSQLDQ---E---
gi|488896133|ref|WP_002807242.1|MULTISPECIES:restrictionendonucleaseEco57I[Campylobacter]_gi|380541096|gb|EIA65377.1|hypothetic  -----------------------SFKNLNISIDHVDGTMNSNVRLNKLNNLNS---P---
gi|746602240|ref|WP_039628365.1|restrictionendonucleaseEco57I[Campylobacterlari]_gi|744819395|gb|AJD03391.1|putativehelicase[Ca  -----------------------SFKNLDINIDHVDGTMNSNIRLNKLNNLNS---P---
gi|727317135|ref|WP_033772558.1|restrictionendonucleaseEco57I[Helicobacterpylori]/11622  -----------------------SFKNLEISIDHIDGTMNCKVRLDKLETLNQ---P---
gi|727103490|ref|WP_033618954.1|restrictionendonucleaseEco57I[Helicobacterpylori]/11616  -----------------------SFKNLEISIDHIDGTMNCKVRLDKLETLNQ---P---
gi|727340205|ref|WP_033794408.1|restrictionendonucleaseEco57I[Helicobacterpylori]/11609  -----------------------SFKNLEISIDHIDGTMNCKVRLDKLETLNQ---P---
gi|727292407|ref|WP_033749975.1|restrictionendonucleaseEco57I[Helicobacterpylori]/11611  -----------------------SFKNLEISIDHIDGTMNCKVRLDKLETLNQ---P---
gi|727289795|ref|WP_033747591.1|restrictionendonucleaseEco57I[Helicobacterpylori]/11612  -----------------------SFKNLQISIDHIDGTMNCKERLDKLENLNQ---F---
```

gi|727319676|ref|WP_033775047.1|restrictionendonucleaseEco57I[Helicobacterpylori]/11612	-----------------------SFKNLTISIDHIDGTMNCKERLEKLEELNE---F---
gi|447212910|ref|WP_001290166.1|restrictionendonucleaseEco57I[Helicobacterpylori]_gi|308063540|gb|ADO05427.1|hypotheticalprotei	-----------------------SFKNLTISIDHIDGTMNCKVRLEKLEELNT---F---
gi|447212901|ref|WP_001290157.1|restrictionendonucleaseEco57I[Helicobacterpylori]_gi|384557329|gb|AFH97797.1|hypotheticalprotei	-----------------------SFKNLTISIDHIDGTMNCKVRLEKLEELNA---F---
gi|515849671|ref|WP_017280424.1|hypotheticalprotein[Helicobacterpylori]/11615	-----------------------SFKNLTISIDHIDGTMNCKVRLEKLEELNA---F---
gi|447212900|ref|WP_001290156.1|restrictionendonucleaseEco57I[Helicobacterpylori]_gi|317178970|dbj|BAJ56758.1|TypeIIGrestrictio	-----------------------SFKNLTISIDHIDGTMNCKVRLEKLEELNE---F---
gi|447079018|ref|WP_001156274.1|hypotheticalprotein[Helicobacterpylori]/11614	-----------------------SFKNLQIKIDHIDGTMNCKVRLEKLEELNE---F---
gi|447212800|ref|WP_001290056.1|hypotheticalprotein[Helicobacterpylori]_gi|532153248|gb|EQL67316.1|restrictionendonucleaseEco57	-----------------------SFKNLQISIDHIDGTMNCKVRLEKLEELNE---F---
gi|505457237|ref|WP_015644339.1|hypotheticalprotein[Helicobacterpylori]_gi|499061160|gb|AGL70074.1|restrictionendonucleaseEco57	-----------------------SFKNLKISIDHIDGTMNCKDRLEKLEKLNQ---F---
HPYUM037X/11591	-----------------------SFKNLKISIDHIDGTMNCKDRLEKLEKLNQ---F---
gi|920026297|ref|WP_052937176.1|restrictionendonuclease[Helicobacterpylori]_gi|912528678|gb|KNX48238.1|restrictionendonucleaseE	-----------------------SFKNLQIKIDHIDGTMNCKDRLEKLEELNQ---F---
gi|915539050|ref|WP_050841327.1|restrictionendonuclease[Helicobacterpylori]_gi|908890887|gb|KNE06183.1|restrictionendonucleaseE	-----------------------SFKNLEIKIDHIDGTMNCKERLEKLENLNQ---F---
gi|727101397|ref|WP_033616957.1|restrictionendonucleaseEco57I[Helicobacterpylori]/11592	-----------------------SFKNLKISIDHIDGTMNCKERLEKLEELNQ---F---
gi|562814875|emb|CDK13002.1|TypeISPRMsystem[Helicobacterpylori26695]/11588	-----------------------SFKNLKISIDHIDGTMNCKDRLEKLEELNQ---F---
HPYAXVIII/11588	-----------------------SFKNLKISIDHIDGTMNCKDRLEKLEELNQ---F---
gi|447079029|ref|WP_001156285.1|restrictionendonucleaseEco57I[Helicobacterpylori]_gi|393085956|gb|EJB86635.1|hypotheticalprotei	-----------------------SFKNLKISIDHIDGTMNCKDRLKKLEELNE---F---
gi|489105953|ref|WP_003015814.1|restrictionendonuclease[Helicobacterpylori]_gi|477503388|gb|ENH59950.1|restrictionendonucleasef	-----------------------SFKNLKISIDHIDGTMNCKERLEKLENLNA---F---
gi|447079006|ref|WP_001156262.1|restrictionendonucleaseEco57I[Helicobacterpylori]_gi|393067470|gb|EJB68280.1|hypotheticalprotei	-----------------------SFKNLKISIDHIDGTMNCKDRLEKLEELNE---F---
gi|727314589|ref|WP_033770298.1|restrictionendonucleaseEco57I[Helicobacterpylori]/11586	-----------------------SFKNLEIKIDHIDGTMNCKERLKKLEKLNQ---F---
gi|447079019|ref|WP_001156275.1|restrictionendonucleaseEco57I[Helicobacterpylori]_gi|308065028|gb|ADO06920.1|hypotheticalprotei	-----------------------SFKNLEIKIDHVDGTMNCKDRLDKLEELNE---L---
gi|447079028|ref|WP_001156284.1|restrictionendonucleaseEco57I[Helicobacterpylori]_gi|393076030|gb|EJB76784.1|hypotheticalprotei	-----------------------SFKNLKISIDHVDGTMNCKERLEKLEELNE---F---
gi|393034009|gb|EJB35070.1|hypotheticalproteinHPNQ4053_0631[HelicobacterpyloriNQ4053]/11514	-----------------------SFKNLKISIDHVDGTMNCKERLDKLENLNT---F---
gi|447079004|ref|WP_001156260.1|restrictionendonucleaseEco57I[Helicobacterpylori]_gi|384552418|gb|AFI07366.1|hypotheticalprotei	-----------------------SFKNLQITIDHVDGTMNCKERLEKLENLNQ---F---
gi|746011306|ref|WP_039088215.1|restrictionendonucleaseEco57I[Helicobacterpylori]_gi|733488720|gb|KHL85979.1|restrictionendonuc	-----------------------SFKNLKINIDHVDGTMNCKERLEKLEKLNQ---F---
gi|531081162|gb|EQD92166.1|restrictionendonucleaseEco57I[HelicobacterpyloriPZ5080]/11509	-----------------------SFKNLKISIDHIDGTMNCKERLDKLENLNQ---F---
gi|544760796|ref|WP_021187395.1|restrictionendonucleaseEco57I[Helicobacterpylori]_gi|531086508|gb|EQD96745.1|restrictionendonuc	-----------------------SFKNLQITIDHIDGTMNCKERLDKLENLNQ---F---
gi|447079012|ref|WP_001156268.1|restrictionendonucleaseEco57I[Helicobacterpylori]_gi|393028199|gb|EJB29286.1|hypotheticalprotei	-----------------------SFKNLLIKIDHVDGTMNCKERLEKLEELNQ---F---
gi|746008458|ref|WP_039085555.1|restrictionendonucleaseEco57I[Helicobacterpylori]_gi|733480358|gb|KHL78026.1|restrictionendonuc	-----------------------SFKNLQITIDHVDGTMNCKERLEKLENLNQ---F---
gi|447079011|ref|WP_001156267.1|restrictionendonucleaseEco57I[Helicobacterpylori]_gi|393038534|gb|EJB39568.1|hypotheticalprotei	-----------------------SFKNLQIRIDHIDGTMNCKERLEKLENLNQ---F---
gi|393037163|gb|EJB38199.1|hypotheticalproteinHPNQ4161_0422[HelicobacterpyloriNQ4161]/11556	-----------------------SFKNLKISIDHVDGTMNCKERLDKLEELNT---F---
gi|447079007|ref|WP_001156263.1|restrictionendonucleaseEco57I[Helicobacterpylori]_gi|393051817|gb|EJB52768.1|hypotheticalprotei	-----------------------SFKNLKISIDHIDGTMNCKDRLEKLEKLNQ---F---
gi|487867094|ref|WP_001940560.1|helicase[Helicobacterpylori]_gi|459505657|gb|EMG98418.1|helicaseprotein[HelicobacterpyloriGAM21	-----------------------SFKNLQISIDHVDGTMNCKERLDKLENLNA---F---
gi|487872094|ref|WP_001945560.1|helicase[Helicobacterpylori]_gi|459512104|gb|EMH04523.1|helicaseprotein[HelicobacterpyloriGAM24	-----------------------SFKNLKINIDHVDGTMNCKKRLDKLEELNK---F---
gi|487880228|ref|WP_001953694.1|helicase[Helicobacterpylori]_gi|459529131|gb|EMH20734.1|helicaseprotein[HelicobacterpyloriGAM26	-----------------------SFKNLKISIDHVDGTMNCKDRLEKLEKLNK---F---
gi|487882593|ref|WP_001956059.1|helicase[Helicobacterpylori]_gi|459533462|gb|EMH24859.1|helicaseprotein[HelicobacterpyloriGAM26	-----------------------SFKNLKINIDHVDGTMNCKERLEKLENLNQ---F---
gi|487845430|ref|WP_001918896.1|helicase[Helicobacterpylori]_gi|410713871|gb|EKQ71363.1|helicaseprotein[HelicobacterpyloriGAM10	-----------------------SFKNLKISIDHVDGTMNCKERLEKLEELNK---F---
gi|447079005|ref|WP_001156261.1|restrictionendonucleaseEco57I[Helicobacterpylori]_gi|393069830|gb|EJB70625.1|hypotheticalprotei	-----------------------SFKNLQITIDHVDGTMNCKERLDKLENLNA---F---
gi|459506808|gb|EMG99528.1|helicaseprotein[HelicobacterpyloriGAM121Aii]/11545	-----------------------SFKNLKINIDHVDGTMNCKERLEKLEELNK---F---
gi|487825283|ref|WP_001898749.1|restrictionendonucleaseEco57I[Helicobacterpylori]_gi|393107526|gb|EJC08068.1|hypotheticalprotei	-----------------------SFKNLKINIDHVDGTMNCKERLDKLEELNK---F---
gi|447079008|ref|WP_001156264.1|restrictionendonucleaseEco57I[Helicobacterpylori]_gi|393058636|gb|EJB59524.1|hypotheticalprotei	-----------------------SFKNLKISIDHVDGTMNCKDRLEKLEELNK---F---
gi|447079015|ref|WP_001156271.1|restrictionendonucleaseEco57I[Helicobacterpylori]_gi|307637357|gb|ADN79807.1|hypotheticalprotei	-----------------------SFKNLKISIDHVDGTMNCKERLDKLENLNT---F---
gi|487884861|ref|WP_001958327.1|helicase[Helicobacterpylori]_gi|459535535|gb|EMH26834.1|helicaseprotein[HelicobacterpyloriGAM26	-----------------------SFKNLKINIDHVDGTMNCKERLDKLENLNA---F---
gi|487886365|ref|WP_001959831.1|helicase[Helicobacterpylori]_gi|459538727|gb|EMH29861.1|helicaseprotein[HelicobacterpyloriGAM27	-----------------------SFKNLQITIDHVDGTMNCKERLDKLENLNA---F---
gi|545301637|ref|WP_021581412.1|helicase[Helicobacterpylori]_gi|535931889|gb|ERA61745.1|helicaseprotein[HelicobacterpyloriGAM11	-----------------------SFKNLQITIDHVDGTMNCKDRLDKLENLNQ---F---
gi|447079010|ref|WP_001156266.1|restrictionendonucleaseEco57I[Helicobacterpylori]_gi|393095477|gb|EJB96081.1|hypotheticalprotei	-----------------------SFKNLKISIDHVDGTMNCKDRLDKLENLNQ---F---
gi|447079009|ref|WP_001156265.1|restrictionendonucleaseEco57I[Helicobacterpylori]_gi|393056294|gb|EJB57206.1|hypotheticalprotei	-----------------------SFKNLKISIDHVDGTMNCKERLDKLENLNA---F---
gi|471386985|gb|EMR58906.1|restrictionendonucleasefamilyprotein[HelicobacterpyloriHpH1]/11504	-----------------------SFKNLQIRIDHVDGTMNCKERLDKLENLNQ---F---
gi|487875679|ref|WP_001949145.1|helicase[Helicobacterpylori]_gi|459516928|gb|EMH09091.1|helicaseprotein[HelicobacterpyloriGAM25	-----------------------SFKNLKISIDHVDGTMNCKERLDKLEKLNQ---F---
gi|496130066|ref|WP_008854573.1|DNAdamageinducibleprotein[Commensalibacterintestini]_gi|353671689|gb|EHD13391.1|helicasedomain/	----------------TPDAPIDPALTMICEAEHVDGGMNASEKEQKLDWLKA---E---
gi|924898894|ref|WP_053556546.1|damageinducibleprotein[Sphingopyxissp.113P3]_gi|924523996|gb|ALC14732.1|damageinducibleprotei/1	--------------------LAEQMPPLEVTVEHVDGGFNAKARDHLLGWLKA---D---
gi|780802950|gb|KJS08562.1|damageinducibleprotein[Hoefleasp.BRH_c9]/11633	---------------------PADKYRLHCEWDHVDGTFNAKTRTNLLQWLKE---D---
gi|944671432|gb|KQN22886.1|damageinducibleprotein[Sphingomonassp.Leaf32]/11415	----------------------DDDDALRCEVHHVDGTFNAKTRTRELEWLKA---E---
gi|944612953|gb|KQM64772.1|damageinducibleprotein[Sphingomonassp.Leaf16]/11726	----------------------DDDDALRCEVHHVDGTFNAKTRTRELEWLKA---E---
gi|764353851|ref|WP_044331468.1|damageinducibleprotein[Sphingomonashengshuiensis]_gi|761897338|gb|AJP71707.1|damageinduciblep/1	---------------------EGDTQPLECQLEHVDGTFNAKARNRLLDWLKE---E---
gi|945874472|gb|KQZ27973.1|damageinducibleprotein[Mesorhizobiumsp.Root552]/11651	---------------------EGNAKPLECEIEHVDGTFNAKARTRLLDWLKE---D---
gi|759408081|ref|WP_043132403.1|damageinducibleprotein[Paracoccussanguinis]_gi|694217086|gb|KGJ14281.1|damageinducibleprotein/1	-----------------------DERYLATEVRHIDGSFNATAREEMLNWLKA---D---
gi|654342671|ref|WP_027836116.1|damageinducibleprotein[Maritaleamyrionectae]/11632	-----------------------EMPHLKTEVRHVDGSYNATARDEMLKWLKA---E---
gi|126717687|gb|EBA14409.1|hypotheticalproteinRSK20926_02032[Roseobactersp.SK20926]/11603	-----------------------DNRHLHTEVRHVDGTFNASTRDEMLNWLKA---D---
gi|765301116|ref|WP_044628780.1|damageinducibleprotein[Halomonasmeridiana]_gi|764089679|gb|KJD19875.1|damageinducibleprotein[/1	-----------------------EVGYLRTEVRHVDGSYNATAREEMLSWLKK---G---
gi|518053006|ref|WP_019223214.1|DNAdamageinducibleprotein[Bartonellarattaustraliani]/11654	--------------------TLKNVPSLECEIKHIDGTQSVKTRNQALDWLKE---D---
gi|493637806|ref|WP_006589578.1|DNAdamageinducibleprotein[Bartonellabirtlesii]_gi|395410345|gb|EJF76900.1|hypotheticalproteinM/	--------------------FLQTVPFLNCEVRHIDGTQSVKKRNEELDWLKQ---D---
gi|501188603|ref|WP_012231622.1|DNAdamageinducibleprotein[Bartonellatribocorum]_gi|161017843|emb|CAK01401.1|helicase/methyltra/	--------------------FLQTVPFLNCEVRHIDGTQSVKKRNEELDWLKE---D---
gi|651388616|ref|WP_026500615.1|damageinducibleprotein[Bartonellagrahamii]/11655	--------------------FLQTVPFLNCEVRHIDGTQSVKKRNEELDWLKE---D---
gi|501188669|ref|WP_012231687.1|DNAdamageinducibleprotein[Bartonellatribocorum]_gi|161017922|emb|CAK01480.1|helicase/methyltra/	--------------------FLQTVPFLNCEVRHIDGTQSVKKRNEELDWLKE---D---
gi|518048832|ref|WP_019219040.1|DNAdamageinducibleprotein[Bartonellaflorencae]/11655	--------------------VPESKASLIFDVKHIDGTFNGKDRNKLLDWLKE---D---
gi|518053617|ref|WP_019223825.1|DNAdamageinducibleprotein[Bartonellarattaustraliani]/11607	--------------------VPESKTSLIFDVKHIDGRFSGKDRNKLLDWLKE---D---
gi|506336449|ref|WP_015856168.1|DNAdamageinducibleprotein[Bartonellagrahamii]_gi|240267492|gb|ACS51080.1|helicase/methyltransf/	--------------------VPESKASLIFDVKHIDGTFNGKDRNKLLDWLKE---D---
gi|494588832|ref|WP_007347427.1|DNAdamageinducibleprotein[Bartonellarattimassiliensis]_gi|395419053|gb|EJF85361.1|hypothetical/	--------------------VPESKTSLIFDVKHIDGSFNGKNRNKLLDWLKE---E---

```
gi|501188650|ref|WP_012231668.1|DNAdamageinducibleprotein[Bartonellatribocorum]_gi|161017902|emb|CAK01460.1|helicase/methyltra/      --------------------VPESKTSLIFDVKHIDGSFNGKNRNKLLDWLKE---D---
gi|501188624|ref|WP_012231643.1|DNAdamageinducibleprotein[Bartonellatribocorum]_gi|161017866|emb|CAK01424.1|helicase/methyltra/      --------------------NHPDTPPLICEVDHIDGKDGAKERSRKLDWLEE---N---
gi|518053856|ref|WP_019224064.1|DNAdamageinducibleprotein[Bartonellarattaustraliani]/11625      --------------------NHPDTPPLICEVDHIDGKDGAKERTRKLDWLEE---N---
gi|501188053|ref|WP_012231081.1|DNAdamageinducibleprotein[Bartonellatribocorum]_gi|161017434|emb|CAK00992.1|helicase/methyltra/      --------------------KHPDTPRLICEIDHIDGKDGAKERSRKLDWLEE---N---
gi|651388630|ref|WP_026500626.1|damageinducibleprotein[Bartonellagrahamii]/11657      --------------------NYKDTPPLNCTLAHIDGTFSAKERTKQLDWLKE---D---
gi|518048855|ref|WP_019219063.1|DNAdamageinducibleprotein[Bartonellaflorencae]/11658      --------------------NYKDTPPLNCTLAHIDGTFSTKERTKQLDWLKE---D---
gi|490995101|ref|WP_004856829.1|DNAdamageinducibleprotein[Bartonelladoshiae]_gi|395413167|gb|EJF79645.1|hypotheticalproteinMCS/      --------------------NYKDTPPLNCTLAHIDGTFSTKERTKQLDWLKE---D---
gi|491002558|ref|WP_004864279.1|DNAdamageinducibleprotein[Bartonellavinsonii]_gi|395421392|gb|EJF87643.1|hypotheticalproteinME/      --------------------SHKKTPALHCRFEHIDGTCSAKERTKKLDWLKE---D---
gi|491003373|ref|WP_004865094.1|DNAdamageinducibleprotein[Bartonellavinsonii]_gi|395432864|gb|EJF98838.1|hypotheticalproteinME/      --------------------SHKKTPALHCRFEHIDGTCSAKERTKKLDWLKE---D---
gi|491002426|ref|WP_004864148.1|DNAdamageinducibleprotein[Bartonellavinsonii]_gi|395421767|gb|EJF88003.1|hypotheticalproteinME/      --------------------NRKETPPLNCTFAHIDGKQSAKRRNELLDWLRE---D---
gi|491004024|ref|WP_004865745.1|DNAdamageinducibleprotein[Bartonellavinsonii]_gi|395432542|gb|EJF98522.1|hypotheticalproteinME/      --------------------NRKETPPLNCTFAHIDGKQSAKRRNELLDWLRE---D---
gi|648396442|ref|WP_026088193.1|damageinducibleprotein[Bartonellarattaustraliani]/11593      --------------------IHKDTTPLDCTFEHIDGTFSAKERTKRLDWLKE---D---
gi|748190174|ref|WP_039761290.1|damageinducibleprotein[Bartonellaqueenslandensis]/11658      --------------------IHKDTPPLDCTFEHIDGTFSAKERTKRLDWLKE---D---
gi|494817599|ref|WP_007553007.1|DNAdamageinducibleprotein[Bartonellasp.DB56]_gi|395411312|gb|EJF77837.1|hypotheticalproteinMC/1      --------------------NHKNIPPLNCQADHINGKSGAKDRTIKLDWLKE---D---
gi|515763376|ref|WP_017195976.1|DNAdamageinducibleprotein[Bartonellabirtlesii]/11656      --------------------NHKNIPPLNCQADHINGKSGAKDRTIKLDWLKE---D---
gi|494589420|ref|WP_007348014.1|DNAdamageinducibleprotein[Bartonellarattimassiliensis]_gi|395416278|gb|EJF82667.1|hypothetical/      --------------------VYKDTPPLDCTFDHIDGTQSAKTRHKALDWLKE---D---
gi|506336469|ref|WP_015856188.1|DNAdamageinducibleprotein[Bartonellagrahamii]_gi|240267512|gb|ACS51100.1|helicase/methyltransf/      --------------------LYKDTPSLHCTFDHIDGTQSAKMRNKALDWLKE---D---
gi|749308087|ref|WP_040132227.1|damageinducibleprotein[Bartonellatribocorum]_gi|605051878|emb|CDO49988.1|helicase/methyltransf/      --------------------VYKDTPPLHCTFDHIDGTQSAKTRHKALDWLKE---D---
gi|515763800|ref|WP_017196400.1|DNAdamageinducibleprotein[Bartonellabirtlesii]/11658      --------------------LYKETPPLQCTFAHIDGTQSAKKRNEALDWLKE---D---
gi|492186994|ref|WP_005773380.1|DNAdamageinducibleprotein[Bartonellaelizabethae]_gi|395418361|gb|EJF84688.1|hypotheticalprotei/      --------------------LYKDTPPLQCTFAHIDGTQSAKKRNEALDWLKE---D---
gi|501188615|ref|WP_012231634.1|DNAdamageinducibleprotein[Bartonellatribocorum]_gi|161017856|emb|CAK01414.1|helicase/methyltra/      --------------------LYKETPPLQCTFAHIDGTQSAKKRNEALDWLKE---D---
gi|501187470|ref|WP_012230499.1|DNAdamageinducibleprotein[Bartonellatribocorum]_gi|161017090|emb|CAK00648.1|helicase/methyltra/      --------------------LYKETPPLQCTFAHIDGTQSAKKRNEALDWLKE---D---
gi|501188636|ref|WP_012231655.1|DNAdamageinducibleprotein[Bartonellatribocorum]_gi|161017879|emb|CAK01437.1|helicase/methyltra/      --------------------LYKETPPLQCTFAHIDGTQSAKKRNEALDWLKE---D---
gi|930151941|emb|CTQ34818.1|UvsWhelicase[Jannaschiarubra]/11641      ----------------------DDALTLDCEVRHVDGTFNADSRGRLLDWLKA---D---
gi|928977106|ref|WP_054001461.1|damageinducibleprotein[Shimiasp.SK013]_gi|927698852|gb|KPA22698.1|typeIrestrictionenzymeEcoKIs/      -----------------------DAEHVSCEVKHVDGTFNAKTRGELLHWLSD---D---
gi|738872465|ref|WP_036760884.1|damageinducibleprotein[Leisingeradaeponensis]/11643      --------------------ADIAQHQLNCQIRHVDGTFNAKSRGECLDWLKA---E---
gi|518050881|ref|WP_019221089.1|DNAdamageinducibleprotein[Bartonellasenegalensis]/11652      ----------------------QNTPFLQCEVKHVDGTFNAKDRGALLDWLKA---E---
gi|493980596|ref|WP_006923523.1|DNAdamageinducibleprotein[Bartonellawashoensis]_gi|395413322|gb|EJF79796.1|hypotheticalprotein/      ----------------------ENEPFLKCEIKHVDGTFRAKDRGALLDWLKA---D---
gi|493983106|ref|WP_006925984.1|DNAdamageinducibleprotein[Bartonellawashoensis]_gi|395418127|gb|EJF84455.1|hypotheticalprotein/      ----------------------ENEPFLKCEIKHVDGTFNAKDRGALLDWLKA---D---
gi|499494671|ref|WP_011181311.1|DNAdamageinducibleprotein[Bartonellahenselae]_gi|49239008|emb|CAF28308.1|hypotheticalproteinBH/      ----------------------ENEPFLQCEIEHVDGTCNAKDRGVLLDWMKA---D---
gi|651388426|ref|WP_026500441.1|damageinducibleprotein[Bartonellagrahamii]/11652      ----------------------ENEHFLKCEIEHVDGTFNAKDRGALLDWLKA---D---
gi|506337280|ref|WP_015856999.1|DNAdamageinducibleprotein[Bartonellagrahamii]_gi|240268402|gb|ACS51990.1|helicase/methyltransf/      ----------------------EKEHFLKCEIEHVDGTFNAKDRGALLDWLKA---D---
gi|494817535|ref|WP_007552943.1|DNAdamageinducibleprotein[Bartonellasp.DB56]_gi|395412002|gb|EJF78518.1|hypotheticalproteinMC/1      ----------------------ENASFLKCEIEHVDGTCNAKDRGALLDWLKA---D---
gi|493638099|ref|WP_006589864.1|DNAdamageinducibleprotein[Bartonellabirtlesii]_gi|395409809|gb|EJF76394.1|hypotheticalproteinM/      ----------------------ENESFLKCEVEHVDGTFNAKDRGALLDWLKA---D---
gi|748189589|ref|WP_039760707.1|damageinducibleprotein[Bartonellaqueenslandensis]/11651      ----------------------ENEPFLKCAVEHVDGKFNAKDRSALLDWLKA---E---
gi|501189483|ref|WP_012232501.1|DNAdamageinducibleprotein[Bartonellatribocorum]_gi|161018902|emb|CAK02460.1|helicase/methyltra/      ----------------------ENENFLKCEIEHVDGKFNAKDRSALLDWLKA---E---
gi|518049181|ref|WP_019219389.1|DNAdamageinducibleprotein[Bartonellaflorencae]/11652      ----------------------ENAPFLKCEVEHVDGTFNAKDRGALLDWLKA---E---
gi|492193255|ref|WP_005774528.1|DNAdamageinducibleprotein[Bartonellaelizabethae]_gi|395416403|gb|EJF82779.1|hypotheticalprotei/      ----------------------ENENFLQCEIEHVDGTFNAKDRGALLDWLKA---D---
gi|498387325|ref|WP_010701288.1|helicase/methyltransferase[Bartonellabovis]_gi|478418724|gb|ENN92426.1|helicase/methyltransfera      ----------------------ENEPFLKCEVEHVDGTFNAKERSILLDWLKA---D---
gi|319408697|emb|CBI82352.1|conservedhypotheticalprotein[BartonellaschoenbuchensisR1]/11661      ----------------------ENEPSLKCEVKHVDGTFNAKERSILLDWLKA---D---
gi|494741726|ref|WP_007477134.1|DNAdamageinducibleprotein[Bartonellamelophagi]_gi|395423925|gb|EJF90113.1|hypotheticalproteinM/      ----------------------ENKPFLKCEVKHVDGKFNAKERSILLDWLKS---D---
gi|737261405|ref|WP_035245349.1|damageinducibleprotein[Alcanivoraxjadensis]_gi|686989709|gb|KGD62556.1|hypotheticalproteinT9A_/      -----------------------GAPELQCSLEHVDGTFNAKTRNRLLDWLKA---E---
gi|916687564|ref|WP_051294655.1|damageinducibleprotein[Gemmobacternectariphilus]/11641      --------------------D--EDERLRCEIEHVDGTYNAKERGRLLDWLKA---D---
gi|740294127|ref|WP_038132495.1|damageinducibleprotein[Thioclavasp.DT234]_gi|660524826|gb|KEO54689.1|hypotheticalproteinDT23_/1      --------------------APSSPDRLDIEIEHVDGTFNAKSRNELLDWLKA---D---
gi|568331062|ref|WP_024099615.1|helicase[Phaeobactergallaeciensis]_gi|568232121|gb|AHD12007.1|putativehelicase[Phaeobactergalla      --------------------EE-DDDQLRCEIEHVDGTFNAKERGRLLDWLKA---D---
gi|518810459|ref|WP_019966413.1|DNAdamageinducibleprotein[Pannonibacterphragmitetus]/11635      --------------------T--PSDHLQCQIEHVDGTFNAKTRGALLDWLKA---D---
gi|495305760|ref|WP_008030510.1|DNAdamageinducibleprotein[Rhodobactersp.SW2]_gi|259021698|gb|EEW25000.1|typeIIIrestrictionprot/      --------------------T--PSDHLQCQIEHVDGTFNAKTRGALLDWLKA---D---
gi|506403303|ref|WP_015923022.1|DNAdamageinducibleprotein[Rhodobactersphaeroides]_gi|221162951|gb|ACM03922.1|HypotheticalProte/      --------------------T--PSDHLQCEIEHVDGTFNAKTRGALLDWLKA---D---
gi|738818386|ref|WP_036708790.1|damageinducibleprotein[Paracoccussanguinis]_gi|694226786|gb|KGJ22541.1|damageinducibleprotein/1      --------------------I--PSDHLQCEIEHVDGTFNAKTRGALLDWLKA---D---
gi|746096057|ref|WP_039162453.1|damageinducibleprotein[Gallibacteriumanatis]_gi|703617775|gb|KGQ63868.1|damageinducibleprotei/1      ----------------DPNLTRDQSLTLKCEAEHVDGSMNASEKAGKINWLKA-------
gi|737716785|ref|WP_035685631.1|damageinducibleprotein[Avibacteriumparagallinarum]/11643      ----------------DPNLTRDQSLTLKCKAEHVDGSMNASEKAGKINWLKA-------
gi|540072553|gb|ERF78672.1|hypotheticalproteinN561_04930[Gallibacteriumanatis12656/12]/11651      ----------------NPELSLNQSLTLKCEAEHVDGSMNASEKSSKINWLKA-------
gi|917509519|ref|WP_052115936.1|damageinducibleprotein[Gallibacteriumanatis]_gi|702414161|gb|KGQ42233.1|damageinducibleprotei/1      ----------------NPELSLNQSLTLKCEAEHVDGSMNASEKSSKINWLKA-------
gi|746082942|ref|WP_039150268.1|damageinducibleprotein[Gallibacteriumanatis]_gi|703617078|gb|KGQ63194.1|damageinducibleprotei/1      ----------------DPKLSLNQSLTLKCEAEHVDGSMNASEKSSKINWLKE-------
gi|822522885|ref|WP_046952840.1|damageinducibleprotein[Haemophilushaemolyticus]_gi|821501762|gb|KKZ59267.1|damageinduciblepro/1      ----------------NPDFRPDNSLLLKCEAEHVDGGMNASEKSAKIEWLKA-------
gi|404381014|gb|EJZ77501.1|putativehelicase[Pasteurellamultocidasubsp.gallicidaP1059]/11681      ----------------DPDFEPDLSLQLKCQAEHVDGSMNASEKSAKINWLKA-------
gi|298281660|gb|EFI23149.1|helicaseCterminaldomainprotein[Neisseriasp.oraltaxon014str.F0314]/11493      ----------------NPDLLTPPSLAMICEAKHVDGGMNAGEKESRLQWLKA-------
gi|348013055|gb|EGY51980.1|helicasedomainprotein[Neisseriashayeganii871]/11481      ----------------QPDLKLPPQLAMICQADHVDGGMSAGEKEARLDWLKA---SD--
gi|496407140|ref|WP_009116004.1|DNAdamageinducibleprotein[Neisseriawadsworthii]_gi|349794756|gb|EGZ48566.1|helicasedomainprote/      ----------------NPDLKLPPQLAMKCEAEHVDGSMNAGEKESKLQWLKA-------
gi|517216441|ref|WP_018405259.1|DNAdamageinducibleprotein[Marinobacterlipolyticus]/11656      ----------------EPDAPLDSALTMLCAAEHVDGTMNASQKEAKLEWLKA-------
gi|738230264|ref|WP_036185691.1|damageinducibleprotein[Marinobactersp.ELB17]/11671      ----------------DPDAPLDSALTMLCAAEHVDGTMNASQKEAKLEWLKA-------
gi|447197741|ref|WP_001274997.1|DNAdamageinducibleprotein[Acinetobacterbaumannii]_gi|408512324|gb|EKK13969.1|typeIIIrestrictio/      ----------------EEDPYIDPSLKLACQVKHVDGGMNATEKKEKITWLKD-------
gi|820775897|ref|WP_046739909.1|damageinducibleprotein[Acinetobactersp.AG1]_gi|819909758|gb|KKW75938.1|damageinducibleprotein/1      ----------------EEDPYIDPSLKLNCEVKHVDGGMNATEKKEKITWLKE-------
gi|584595163|ref|WP_024160846.1|helicase[Acinetobacterpittii]_gi|576702530|emb|CDI28049.1|helicasedomaincontainingprotein[Acin/      ----------------EEDPYIDPSLKLNCHVKHVDGGMNATEKKEKITWLKE-------
gi|514963042|ref|WP_016651366.1|hypotheticalprotein[Acinetobactersp.NIPH2036]_gi|514162665|gb|EPG41391.1|hypotheticalproteinF90      ----------------EEYPYIDPSLKLNCEVKHVDGGMNATEKKDKITWLKE-------
gi|515965564|ref|WP_017396147.1|DNAdamageinducibleprotein[Acinetobacterhaemolyticus]/11667      ----------------EEDPYIDPSLKLNCHVKHVDGGMNATEKKEKITWLKE-------
gi|491346810|ref|WP_005204747.1|MULTISPECIES:hypotheticalprotein[Acinetobacter]_gi|480335823|gb|ENX56033.1|hypotheticalproteinF      ----------------EEDPYIDLSLKLNCQVKHVDGGMNATEKKEKITWLKE-------
```

```
gi|693044472|ref|WP_032215268.1|damageinducibleprotein[Escherichiacoli]_gi|607725731|gb|EZE08230.1|damageinducibleprotein[Esc/1      ----------------APGKAIDPSLKLQCQVKHVDGSMNATEKKSKIEWLKA-------
gi|742382177|ref|WP_038861304.1|damageinducibleprotein[Cronobactermuytjensii]_gi|924333747|gb|ALB69766.1|damageinducibleprote/1     ----------------SPDKEIDPSLMLQCQVKHVDGSMNATEKKSKIEWLKA-------
gi|915912804|ref|WP_050927035.1|damageinducibleprotein[Yersiniaenterocolitica]_gi|899805802|emb|CQH32042.1|typeIrestrictionenz/     ----------------APEQEFDLSLKLQCEVKHVDGSMNATEKKSKIEWLKA-------
gi|921111327|gb|KON62781.1|typeIrestrictionenzymeEcoKIsubunitR[Komagataeibactereuropaeus]/11492                                       -------------------------ARLVCEAEHVDGGMNASEKEGKLAWLKD---E---
gi|582024278|gb|AHI27615.1|DNAhelicaserestrictionenzymeTypeIIIRsubunit[KomagataeibacterxylinusE25]/11636                              -------------------------ARLVCEAEHVDGGMNASEKEGKLAWLKD---E---
gi|347662673|dbj|BAK86128.1|DNAhelicaserestrictionenzymeTypeIIIRsubunit[KomagataeibactermedellinensisNBRC3288]/11737                  -------------------------ARLICEAEHVDGGMNASEKEGKLAWLKD---E---
gi|917445053|ref|WP_052051471.1|damageinducibleprotein[Acetobactertropicalis]/11429                                                   -------------------------AYLTCEAEHVDGGMNASEKEGKLSWLKE---E---
gi|453329445|dbj|GAC88472.1|DNAhelicaserestrictionenzymetypeIIIRsubunit[GluconobacterthailandicusNBRC3255]_gi|530287981|dbj|GAD      -------------------------ARLTCEAKHVDGGMNASEKEAKLSWLKD---KPEE
gi|889770922|ref|WP_048844278.1|damageinducibleprotein[Gluconobacterfrateurii]/11419                                                  -------------------------ARLTCEAKHVDGGMNASEKEAKLSWLKE---G---
gi|504886208|ref|WP_015073310.1|hypotheticalprotein[Gluconobacteroxydans]_gi|411027954|gb|AFW01209.1|hypotheticalproteinB932_16      -------------------------ARLTCEAKHVDGGMNASEKEAKLSWLKE---E---
gi|480040211|gb|ENV79751.1|hypotheticalproteinF942_01641[AcinetobacterursingiiANC3649]/11432                                          -------------------DADLIENSLQCIAEHIDGGMNATTKEEKLQWLKA---Q---
gi|822498222|ref|WP_046936504.1|damageinducibleprotein[Xanthomonasgardneri]_gi|821751224|gb|KLB07223.1|damageinducibleprotein/1      -------------------------ARLTCEAEHVDGGMNASQKEAKLDWLKA---E---
gi|654059559|ref|WP_027705556.1|damageinducibleprotein[Zymobacterpalmae]/11635                                                        -------------------------DRITCEAEHVDGSMNASEKEAKLDWLKA---E---
gi|643535620|ref|WP_025224315.1|damageinducibleprotein[Methylophilaceaebacterium11]_gi|577704400|gb|EUJ10546.1|putativehelicas/      -------------------D-QPISIKLKCEAEHIDGGMNASQKETKLTWLKA---E---
MBA11I/11635                                                                                                                          -------------------D-QPISIKLKCEAEHIDGGMNASQKETKLTWLKA---E---
gi|655355857|ref|WP_028762287.1|damageinducibleprotein[Shewanellacolwelliana]/11632                                                   -------------------------DGLFCEAEHIDGSMNASEKGEKISWLKE---K---
gi|753204236|ref|WP_041510579.1|damageinducibleprotein[Shewanellasp.cp20]_gi|752457765|gb|KIO36326.1|damageinducibleprotein[S/1      -------------------------DDLVCEVQHVDGSMNATEKESKINWLKA---Q---
KPNNIH30III/11619                                                                                                                     -------------------------DDLICEVEHVDGGMNATEKESKINWLKA---Q---
gi|749295994|ref|WP_040120352.1|damageinducibleprotein[Klebsiellapneumoniae]_gi|723249300|gb|AIX81926.1|damageinducibleprotei/1      -------------------------DDLICEVEHVDGGMNATEKESKINWLKA---Q---
gi|641639739|emb|CDQ54010.1|unnamedproteinproduct[Klebsiellapneumoniae]/11641                                                        -------------------------DDLICEVEHVDGGMNATEKESKINWLKA---Q---
gi|490797922|ref|WP_004660060.1|hypotheticalprotein[Acinetobactersp.NIPH236]_gi|479876779|gb|ENU27973.1|hypotheticalproteinF992      --------------------GNEISHRLFCQAQHVDGGMGADKKEQRLNWLKA---TPEP
gi|916750926|ref|WP_051357982.1|damageinducibleprotein[Acinetobactertjernbergiae]/11453                                               --------------------GNEISHRLICEADHVDGGMGADKKEQKLNWLKA---IPKQ
gi|691098662|ref|WP_032025060.1|damageinducibleprotein[Acinetobacterbaumannii]_gi|593567521|gb|EXR50735.1|restrictionendonucle/      --------------------GNEISHRLICEAQHVDGAMGADKKEQKLNWLKA---TPEP
gi|736669405|ref|WP_034676049.1|damageinducibleprotein[Acinetobacterbaumannii]/11643                                                  --------------------GNEISHRLVCEAQHVDGGMGADLKEQKLNWLKA---TPEP
gi|491368928|ref|WP_005226826.1|hypotheticalprotein[Acinetobactersp.NIPH1847]_gi|480173259|gb|ENX09797.1|hypotheticalproteinF89      --------------------GNEISHRLICEAQHVDGGMGADQKEQKLNWLKA---TPEL
gi|796853993|ref|WP_045795953.1|damageinducibleprotein[Acinetobacterindicus]_gi|787929761|gb|KJV44219.1|damageinducibleprotei/1      --------------------GNEISHRLICEAQHVDGGMGADLKEQKLNWLKA---TPEP
gi|295797463|gb|ADG32253.1|typeIIIrestrictionproteinressubunit[ThiomonasintermediaK12]/11676                                         --------------------SEQPAVALTCEAEHVDGGMNASQKEAKLDWLKA---ET--
gi|499871179|ref|WP_011551913.1|DNAdamageinducibleprotein[Myxococcusxanthus]_gi|108467544|gb|ABF92729.1|putativerestriction/mo/      --------------------NAATSAALRCQAEHVDGSMNASEKEAKISWLKA---ET--
gi|495313852|ref|WP_008038600.1|DNAdamageinducibleprotein[Bartonellatamiae]_gi|395424181|gb|EJF90368.1|hypotheticalproteinME5_/      ---------------------EETSVKLHCEARHVDGGMNASEKEEKIHWLKS---KT--
gi|501453335|ref|WP_012476784.1|DNAdamageinducibleprotein[Leptospirabiflexa]_gi|167777824|gb|ABZ96123.1|Helicase[Leptospirabif/      ----------------------ENPHPLLCEAEHVDGGMNAGEKEAKLQWLKS---ET--
gi|738104613|ref|WP_036063193.1|damageinducibleprotein[Leptospiranoguchii]/11652                                                      ----------------------TIENPLHVEVDHVDGSMNATEKESKLQWLKE---DT--
gi|410001039|gb|EKO51661.1|typeIIIrestrictionenzyme,ressubunit[Leptospirakirschneristr.200802841]/11651                              ----------------------TIENPLRVEVDHVDGSMNATDKESKLQWLKE---DT--
gi|490901851|ref|WP_004763774.1|typeIIIrestrictionenzyme,ressubunit[Leptospirakirschneri]_gi|464209249|gb|EMN24014.1|typeIIIres      ----------------------TIENPLRVEVDHVDGSMNATDKESKLQWLKE---DT--
gi|464398131|gb|EMO65902.1|typeIIIrestrictionenzyme,ressubunit[Leptospirakirschneristr.200803703]/11648                              ----------------------TIENPLRVEVDHVDGSMNATDKESKLQWLKE---DT--
gi|945029024|gb|KQQ84900.1|damageinducibleprotein[Xanthomonassp.Leaf131]/11423                                                        -------------------EDIEEGARLTCEAAHVDGGMNASEKEAKLDWLKA---E---
gi|896511314|ref|WP_049418183.1|damageinducibleprotein[Stenotrophomonasmaltophilia]/11639                                             -------------------EDIEDGARLTCEAEHVDGGMNAGQKEAKLDWLKA---P---
gi|896513082|ref|WP_049419616.1|damageinducibleprotein[Stenotrophomonasmaltophilia]/11638                                             -------------------EDIEDATRLTCEAAHVDGSMNAGQKEAKLDWLKA---D---
gi|896580616|ref|WP_049469425.1|damageinducibleprotein[Stenotrophomonasmaltophilia]/11641                                             -------------------EDIEDAARMTCEAKHVDGSMNAGEKEAKLDWLKA---P---
gi|648289350|ref|WP_026070499.1|damageinducibleprotein[Stenotrophomonasmaltophilia]/11639                                             -------------------EDIEDAARMTCEAKHVDGSMNAGEKEAKLDWLKA---P---
gi|656272786|ref|WP_029218442.1|damageinducibleprotein[Xanthomonascassavae]/11639                                                     -------------------EDIEDAARMTCEAKHVDGSMNAGEKEAKLDWLKA---P---
gi|406976236|gb|EKD98747.1|hypotheticalproteinACD_23C00290G0002[unculturedbacterium]/11679                                            -------------------ERISPSFNLACEAAHVDGSMNATEKDTLLSWLKE---E---
gi|828145737|ref|WP_047238120.1|damageinducibleprotein[Chromobacteriumsubtsugae]/11645                                                ----------------------EDCATLMCEADHVDGGMNAGEKESKLAWLKS---P---
gi|654311174|ref|WP_027824327.1|damageinducibleprotein[Laribacterhongkongensis]/11641                                                 ----------------------EDCATLKCEADHIDGGMNAGEKDSKLAWLKA---P---
gi|757242173|gb|KIR15516.1|typeIrestrictionenzymeEcoKIsubunitR[Pseudomonasfluorescens]/11778                                          -------------------ENTDTAATLICEASHVDGGMTASQKEAELDWLKA---E---
gi|653605161|ref|WP_027616682.1|damageinducibleprotein[Pseudomonassp.URHB0015]/11639                                                  -------------------ENNDTAATLICEASHVDGGMTASQKEAELDWLKA---E---
gi|520868025|ref|WP_020308060.1|helicase[Pseudomonassyringae]_gi|519159034|gb|EPM49935.1|helicasedomaincontainingprotein[Pseud/      -------------------ESTDTAATLICEASHVDGGMTASQKEAELDWLKA---E---
gi|940156567|gb|KPX71668.1|Helicasedomaincontainingprotein[Pseudomonasamygdalipv.lachrymans]/11677                                   -------------------ENTNTAATLICEASHVDGGMTASQKEAELDWLKA---E---
gi|505460508|ref|WP_015647610.1|helicase[Pseudomonassp.GLE121]_gi|499110212|ref|YP_007974374.1|helicasedomaincontainingprotein/      -------------------EEIEEASRLVCQAEHVDGSMNASEKEAKLAWIKA---D---
gi|940235551|gb|KPY45311.1|hypotheticalproteinALO48_200154[Pseudomonassyringaepv.rhaphiolepidis]/11631                               -------------------EEIEEASRLICEAQHVDGSMNASEKETKLTWLKA---D---
gi|520880808|ref|WP_020312235.1|helicase[Pseudomonassyringae]_gi|519172158|gb|EPM62027.1|helicasedomaincontainingprotein[Pseud/      -------------------EDVDEAARLTCEADHVDGGMNASQKDEKLTWLKA---E---
gi|829046479|ref|WP_047282259.1|damageinducibleprotein[Pseudomonasfluorescens]/11630                                                  -------------------EEIDEASRIICEAEHVDGGMNASQKEAKLDWLKE---E---
gi|499415493|ref|WP_011102960.1|DNAdamageinducibleprotein[Pseudomonassyringaegroupgenomosp.3]_gi|28867279|ref|NP_789898.1|heli/      -------------------ENIDEASRIICEAAHVDGCMNASQKEAKLNWLKE---E---
gi|730238299|ref|WP_033942897.1|damageinducibleprotein[Pseudomonasaeruginosa]/11629                                                   -------------------EEIDEASRLICEAEHVDGGMNASAKEAKLNWLKE---E---
gi|495310125|ref|WP_008034873.1|DNAdamageinducibleprotein[Pseudomonassp.GM60]_gi|398186685|gb|EJM74052.1|putativehelicase[Pseu/      -------------------EEIDEASRIVCEAEHVDGGMNASQKEAKLNWLKE---E---
gi|779992653|ref|WP_045428690.1|damageinducibleprotein[Pseudomonassp.MT1]_gi|699967872|dbj|BAP80334.1|DNAhelicaserestrictione/1      -------------------ENIDEASRLICKAEHVDGGMNASQKEAKLAWLKE---E---
gi|786037289|ref|WP_045733274.1|damageinducibleprotein[Pseudomonaspseudoalcaligenes]_gi|782987904|gb|KJU79152.1|damageinducib/1      -------------------ESIDEASRLICEAEHVDGGMNASAKEAKLNWLKE---E---
gi|749666366|ref|WP_040265445.1|damageinducibleprotein[Pseudomonasrhodesiae]/11632                                                    -------------------EEIDEGSRIICEAAHVDGGMNASQKEAKLNWLKE---E---
gi|734902433|ref|WP_034133698.1|damageinducibleprotein[Pseudomonasfluorescens]/11635                                                  -------------------EEIDEGSRIICEATHVDGGMNASQKEAKLNWLKE---E---
gi|490551031|ref|WP_004416146.1|typeIIrestrictionmodificationsystemrestriction/helicasesubunit[Pseudomonassyringae]_gi|4432823/      -------------------ENIDEASRIICEAAHVDGGMNASAKEVKLNWLKE---E---
gi|489199911|ref|WP_003109152.1|DNAdamageinducibleprotein[Pseudomonasaeruginosa]_gi|552648708|gb|ERY63184.1|hypotheticalprotei/      -------------------EEIDEASRLICEAEHVDGGMNASAKEAKLNWLKE---E---
gi|730641237|ref|WP_034061328.1|damageinducibleprotein[Pseudomonasaeruginosa]/11636                                                   -------------------ENIDEASRIICEAAHVDGGMNASAKETKLNWLKE---E---
gi|751304980|ref|WP_041013386.1|damageinducibleprotein[Pseudomonasxanthomarina]_gi|703407611|emb|CEG51014.1|Helicasedomaincon/1      -------------------ENIDEASRIICEAEHVDGSMNASQKEAKLNWLKD---E---
gi|939335207|ref|WP_054881520.1|damageinducibleprotein[Pseudomonassp.NBRC111128]/11632                                                -------------------ENIDEASRIICEAAHVDGGMNASAKEAKLTWLKE---E---
gi|757693555|ref|WP_042923792.1|damageinducibleprotein[Pseudomonaschloritidismutans]_gi|780910610|gb|KJS78302.1|damageinducib/1      -------------------EDIDEASRIICEAAHVDGGMNASQKEAKLNWLKE---E---
gi|665953944|ref|WP_031319989.1|damageinducibleprotein[Pseudomonastolaasii]/11626                                                     -------------------EEIDEAARLTCEAEHVDGGMNASQKEAKLKWLKA---E---
gi|771600573|ref|WP_045213746.1|damageinducibleprotein[Pseudomonassp.21]_gi|770081123|gb|KJJ98634.1|damageinducibleprotein[Ps/1      -------------------VEIEEASRIICEAEHVDGGMNASQKEAKLNWLKE---E---
gi|740871396|ref|WP_038656644.1|damageinducibleprotein[Pseudomonasstutzeri]_gi|627772214|gb|AHY41437.1|damageinducibleprotein/1      -------------------ENIDEVSRITCQAEHVDGGMNASQKEAKLSWLKE---E---
```

```
gi|946242205|gb|KRC93782.1|damageinducibleprotein[Pseudomonassp.Root9]/11634    -------------------ENIDEASRIICEAAHVDGGMNASAKEAKLNWLKE---E---
gi|553777322|ref|WP_023109107.1|MULTISPECIES:hypotheticalprotein[Pseudomonas]_gi|552638674|gb|ERY53184.1|hypotheticalproteinQ05    -------------------ENIDEASRIICEAAHVDGGMNASAKEAKLNWLKE---E---
gi|896383370|ref|WP_049324618.1|damageinducibleprotein[Pseudomonasstutzeri]/11627    -------------------ENIDEASRLICEAEHVDGGMNASAKEAKLNWLKE---E---
gi|696158510|gb|AIT41787.1|putativehelicase[Pseudomonassp.LM8]/11630    -------------------ENIDEASRIICEAEHVDGGMNASQKEAKLKWLKD---E---
gi|504634892|ref|WP_014821994.1|DNAdamageinducibleprotein[Pseudomonasstutzeri]_gi|390985249|gb|AFM35242.1|helicasedomainconta/1    -------------------ENIDEVSRITCQAEHVDGGMNASQKEAKLSWLKE---E---
gi|544783974|ref|WP_021206729.1|DNAdamageinducibleprotein[Pseudomonasstutzeri]_gi|533224779|gb|EQM79688.1|DNAdamageinduciblep/1    -------------------ENIDEASRLICQAEHVDGGMNASQKEAKLKWLKD---E---
gi|504567385|ref|WP_014754487.1|MULTISPECIES:DNAdamageinducibleprotein[Pseudomonas]_gi|388559493|gb|AFK68634.1|helicasedomain/1    -------------------ENIDEASRIICEAAHVDGGMNASAKEAKLNWLKE---E---
gi|654441469|ref|WP_027912052.1|damageinducibleprotein[Pseudomonassp.URIL14HWK12:I7]/11630    -------------------ENIDESSRIICEAAHVDGGMNASAKEAKLNWLKE---E---
gi|941470066|ref|WP_055127894.1|damageinducibleprotein[Pseudomonasmediterranea]/11631    -------------------ENIDESSRIICEAAHVDGGMNASAKEAKLNWLKE---E---
gi|557815599|ref|WP_023446184.1|DNAdamageinducibleprotein[Pseudomonaschloritidismutans]_gi|557364081|gb|ESQ98190.1|DNAdamagei/1    -------------------ENIDEASRIICEAAHVDGGMNASAKEAKLNWLKE---E---
gi|727814791|ref|WP_033898247.1|damageinducibleprotein[Pseudomonassp.FH1]_gi|567648005|gb|ETK23229.1|helicasedomaincontaining/1    -------------------EEIDEGSRIICEAAHVDGGMNASQKEAKLNWLKE---E---
gi|503278702|ref|WP_013513363.1|DNAdamageinducibleprotein[Desulfovibrioaespoeensis]_gi|316942375|gb|ADU61426.1|typeIIrestrict/    ----------------------ELASTLICKAEHVDGSMNASAKEEKLDWLRA---E---
gi|505228796|ref|WP_015415898.1|typeIIIrestrictionendonucleasesubunitR[Desulfovibriopiezophilus]_gi|451896976|emb|CCH49855.1|Ty    ----------------------ELVSSLTCEADHVDGSMNASTKESKLDWLRA---E---
gi|517732543|ref|WP_018902751.1|hypotheticalprotein[Variovoraxparadoxus]/11675    ----------------------ENAPKLACEAAHVDGGMNASEKEEKLSWLKA---P---
gi|550909475|ref|WP_022662570.1|DNAdamageinducibleprotein[Desulfovibriolongus]/11637    -------------------EEFEPSARLTCEAAHVDGSMNASEKEAKLQWLKA---E---
gi|945494542|gb|KQV50024.1|damageinducibleprotein[Massiliasp.Root335]/11647    ----------------------DAASTLACEAEHVDGSMNASEKESKLAWLKA---D---
gi|334196917|gb|AEG70102.1|helicasedomainprotein[RalstoniasolanacearumPo82]/11663    -------------------EEFEQITRLTCEAEHVDGGMNASEKEAKLAWLKS---E---
gi|656006746|ref|WP_029048170.1|damageinducibleprotein[Cupriavidussp.amp6]/11661    -------------------EEFEQAARLRCEAEHVDGGMNASEKEAKLAWLKS---E---
BPS2196ORF3054P/11659    -------------------EEFEQVARLHCEAEHVDGGMNASEKEAKLAWLKA---E---
gi|740932312|ref|WP_038717332.1|damageinducibleprotein[Burkholderiapseudomallei]_gi|760211882|gb|KIX58314.1|damageinduciblepr/1    -------------------EEFEQVARLHCEAEHVDGGMNASEKEAKLAWLKA---E---
gi|755810813|ref|WP_042583861.1|damageinducibleprotein[Burkholderiasp.MSHR3999]_gi|755174952|gb|KIP19790.1|DEAD/DEAHboxhelicas/    -------------------EEFEQAVRLHCEAEHVDGGMNASEKEAKLAWLKA---E---
gi|484586570|gb|AGK48195.1|DEAD/DEAHboxhelicasefamilyprotein[BurkholderiathailandensisMSMB121]/11646    -------------------EEFEQAARLHCEAEHVDGGMNASEKEAKLAWLKA---E---
gi|737582935|ref|WP_035553941.1|damageinducibleprotein[Burkholderiasp.9120]/11658    -------------------EEFEQAARLHCEAEHVDGGMNASEKEAKLAWLKA---E---
gi|740989872|ref|WP_038773225.1|damageinducibleprotein[Burkholderiapseudomallei]_gi|705645291|gb|KGS70691.1|DEAD/DEAHboxhelica/    -------------------EEFEQAARLRCEAEHVDGGMNASEKEAKLAWLKA---E---
gi|654912525|ref|WP_028363424.1|damageinducibleprotein[Burkholderiasp.JPY366]/11659    -------------------EEFEQVARLHCEAEHVDGGMNASEKEAKLAWLKA---E---
gi|740955879|ref|WP_038740512.1|damageinducibleprotein[Burkholderiapseudomallei]_gi|714684932|gb|KGW74438.1|DEAD/DEAHboxhelica/    -------------------EEFEQAARLRCEAEHVDGGMNASEKEAKLAWLKA---E---
gi|919203714|ref|WP_052759215.1|damageinducibleprotein[Xanthomonasaxonopodis]/11388    -------------------------DLLRCDVQHVDGTYNVLLRNQKLDWLK---AESE-
gi|522152381|ref|WP_020663589.1|hypotheticalprotein[Amycolatopsisbenzoatilytica]/11605    -------------------------ATLRCEVRHVDGSYNALQRNVELDWLK---ADAE-
gi|663196013|ref|WP_030233899.1|damageinducibleprotein[Streptomycessp.NRRLS455]/11641    -------------------------ELLNCEVEHIDGTFNTLRRNDLLDWLK---QDPG-
gi|750429553|ref|WP_040711444.1|damageinducibleprotein[Nocardiopsispotens]/11622    -------------------------KALRCEIRHVDGTFNTLRRNERLDWLK---QEPD-
gi|518656139|ref|WP_019819475.1|hypotheticalprotein[Saccharomonosporasaliphila]/11623    -------------------------EVLHCEVDHVDGTFNALQRNRLLDWLK---QDPG-
gi|759722933|ref|WP_043433758.1|damageinducibleprotein[Streptomycespluripotens]_gi|733404978|gb|KHK94361.1|damageinduciblepro/1    -------------------------DVLHCEVEHVDGTFNTLRRNQLLDWLK---QDPG-
gi|663424167|ref|WP_030419030.1|damageinducibleprotein[Streptomycessp.NRRLF5065]/11627    -------------------------DVLHCEVQHVDGTFNTLRRNQLLDWLK---QDPG-
gi|663412917|ref|WP_030408353.1|damageinducibleprotein[Streptomycessp.NRRLF5917]/11630    -------------------------DVLHCEVEHVDGTFNTLQRNQLLDWLK---QDPG-
gi|671521483|ref|WP_031506489.1|damageinducibleprotein[Streptomycesmegasporus]/11630    -------------------------DVLHCEVEHVDGTFNTLRRNQLLDWLK---QDPG-
gi|740053144|ref|WP_037902776.1|damageinducibleprotein[Streptomycessp.TAA204]/11626    -------------------------DVLHCEVDHVDGTFNTLQRNRLLDWLK---QDPG-
gi|663409375|ref|WP_030404974.1|MULTISPECIES:damageinducibleprotein[Streptomyces]/11627    -------------------------DVLHCDVDHVDGTFNTLQRNRLLDWLK---QDPG-
gi|746628608|ref|WP_039654277.1|damageinducibleprotein[Streptomycessp.MUSC125]_gi|745644381|gb|KIE25557.1|damageinducibleprot/1    -------------------------DVLHCEVGHVDGTFNTLQRNRLLDWLK---QDPG-
gi|727322066|ref|WP_033777320.1|damageinducibleprotein[Salinisporapacifica]/11627    -------------------------EVLRCEVEHVDGTFNTLRRNKLLDWLK---QDPG-
gi|653653242|ref|WP_027655726.1|damageinducibleprotein[Salinisporapacifica]/11618    -------------------------EVLRCEVEHVDGTYNTLRRNKLLDWLK---QDPG-
gi|648632543|ref|WP_026324294.1|damageinducibleprotein[Salinisporapacifica]/11621    -------------------------EVLRCEVEHVDGTFNMLRRNKLLDWLK---QDPG-
gi|881018002|ref|WP_048738463.1|damageinducibleprotein[Corynebacteriumfalsenii]/11676    ------GVDATDSPGSVSREFALHNVDLDIAARHVDGGMNAMERNSRLSWLE---SDVP-
gi|505465526|ref|WP_015652417.1|helicase[Corynebacteriumcallunae]_gi|459385704|gb|AGG67995.1|helicase[CorynebacteriumcallunaeDS    -----------------NDEVDEHNLNLSVACQHVDGSMNALERNSRLTWLK---APTQ-
gi|41223062|emb|CAF19007.1|DNAorRNAhelicaseofsuperfamilyII[CorynebacteriumglutamicumATCC13032]/11646    -----------------NDDVDEHNLNLSVACQHVDGSMNALERNSRLTWLK---APTQ-
CGL13032I/11643    -----------------NDDVDEHNLNLSVACQHVDGSMNALERNSRLTWLK---APTQ-
gi|751257081|ref|WP_040967675.1|damageinducibleprotein[Corynebacteriumglutamicum]_gi|748811620|gb|AJE67931.1|damageinduciblep/1    -----------------TNDVSLHNVDVGIAANHVDGTMNAMQRESLLTWLE---APIP-
gi|749040539|ref|WP_040086524.1|damageinducibleprotein[Corynebacteriumhumireducens]/11663    -----------------TNDISLHNIDLEVGAHHVDGGMNAMERSSHLTWLE---APLR-
gi|491662899|ref|WP_005519615.1|helicase[Corynebacteriummatruchotii]_gi|224946756|gb|EEG27965.1|helicaseCterminaldomainprotein/    -----------------VNDISLHHVDLRIAAQHVDGGMNAMQRNTKLSWLQ---STMP-
gi|746717312|ref|WP_039676231.1|damageinducibleprotein[Corynebacteriumminutissimum]_gi|734991118|gb|KHO29219.1|damageinducibl/1    -----------------TNDVSLLNIDLQVAVEHVDGTMNALERGNKISWLE---SSIP-
gi|908654892|emb|CQD15529.1|SuperfamilyIIDNA/RNAhelicase(modularprotein)[Corynebacteriumstriatum]/11674    -----------------TNDVSLLNIDLQVAVEHVDGTMNALERGNKISWLE---SSIP-
gi|895994446|ref|WP_049064418.1|damageinducibleprotein[Corynebacteriumaurimucosum]/11667    -----------------TNDVSLLNIDLQVAVEHVDGTMNALERGNKISWLE---SSIP-
gi|896123331|ref|WP_049145390.1|damageinducibleprotein[Corynebacteriumaurimucosum]/11669    -----------------TNDVSLLNIDLQVAVEHVDGTMNALERGNKISWLE---SSIS-
gi|817681178|ref|WP_046645422.1|damageinducibleprotein[Corynebacteriumstriatum]_gi|817551679|gb|KKO79868.1|damageinduciblepro/1    -----------------TNDVSLLNIDLQVAVEHVDGTMNALERGNKISWLE---SSIP-
gi|896139239|ref|WP_049159146.1|damageinducibleprotein[Corynebacteriumaurimucosum]/11675    -----------------TNDVSLLNIDLQVAVEHVDGTMNALERGNKISWLE---SSIP-
gi|489943947|ref|WP_003847254.1|helicase[Corynebacteriumammoniagenes]_gi|295968218|gb|EFG81467.1|helicaseCterminaldomainprotei/    -----------------LSGVDAQNINLEIAADHVDGTMNALARSSRISWLE---ADMP-
gi|516653464|ref|WP_018022453.1|helicase[Corynebacteriumdoosanense]_gi|697778282|gb|AIT61018.1|damageinducibleprotein[Coryneba/    -----------------VDEEAKHNIDLEVAAKHVDGTMNALIRSSSISWLE---ADMP-
gi|493894179|ref|WP_006840129.1|helicase[Corynebacteriumlipophiloflavum]_gi|227078819|gb|EEI16782.1|helicaseCterminaldomainpro/    -----------------MSDISELNLELDVEAQHVDGSMNALARNNRISWLE---SSFT-
gi|740831472|ref|WP_038616755.1|damageinducibleprotein[Corynebacteriumpseudotuberculosis]_gi|666103058|gb|AIG06935.1|putativeh/    ------V-------EPSLVDATLHHVDLSVTAQHVDGTMNALERSGKITWLE---ADFP-
gi|518023322|ref|WP_019193530.1|helicase[Corynebacteriumtimonense]/11655    -----------------PGEASLHNIDLSVSAQHVDGTMNALERSNKITWLE---ADIP-
gi|552767697|ref|WP_023019559.1|MULTISPECIES:hypotheticalprotein[Corynebacterium]_gi|550739013|gb|ERS38609.1|hypotheticalprotei    -----------------LEDISLHNTDVAVAAQHVDGTMNAMQRSSRINWLE---ADIA-
gi|652659121|ref|WP_027017812.1|damageinducibleprotein[Corynebacteriumpseudodiphtheriticum]/11644    -----------------LEDISLHNTDVAVAAQHVDGTMNAMQRSSRINWLE---ADIA-
gi|749039309|ref|WP_040085390.1|damageinducibleprotein[Corynebacteriumhumireducens]/11657    -----------------LNDVDLSNADLDIQADHVDGSMNAQTRDRKISWLE---STMP-
gi|740835591|ref|WP_038620874.1|damageinducibleprotein[Corynebacteriumulcerans]_gi|698973854|gb|AIU33664.1|HelicaseCterminald/1    -----------------VNDVSLHNVDVGVAAEHVDGGMNAMVRGKKISWLE---ADID-
gi|805307599|ref|WP_046096452.1|damageinducibleprotein[Corynebacteriumulcerans]_gi|803357323|gb|AKA97638.1|Damageinduciblepro/1    -----------------VNDVSLHNVDVGVAAEHVDGGMNAMVRGKKISWLE---ADID-
gi|504083555|ref|WP_014317549.1|helicase[Corynebacteriumdiphtheriae]_gi|371584249|gb|AEX47914.1|superfamilyIIDNA/RNAhelicase[Co    -----------------LNDVSLHNIDLRIAVKHVDGGMNAMERNNRLFWIE---SPIA-
gi|896182193|ref|WP_049193580.1|damageinducibleprotein[Corynebacteriumjeikeium]/11645    -----------------LNDVSTQNIDLRVAVQHVDGGMNAMERSTRLSWIE---APVD-
gi|917636459|ref|WP_052205460.1|damageinducibleprotein[Corynebacteriumriegelii]_gi|914295811|gb|AKV59187.1|damageinduciblepro/1    -----------------TNDVSLHNVDLSVAVQHVDGGMNAMERGNKISWLE---ASMD-
```

```
gi|737013522|ref|WP_035009205.1|damageinducibleprotein[Corynebacteriumjeikeium]/11651  -----------------LNDVSLHNLDLSVAVQHVDGSMNAMERGNKIAWLE---ASMN-
gi|737006291|ref|WP_035002094.1|damageinducibleprotein[Corynebacteriumjeikeium]/11658  -----------------LGDVSLHNVDLRIASQHVDGAMNAMERGSKLTWLE---STPD-
gi|737010378|ref|WP_035006133.1|damageinducibleprotein[Corynebacteriumjeikeium]/11650  -----------------MGDVSLHNVDLRVTSQHVDGAMNAMERGSRLSWLE---STPD-
gi|737010473|ref|WP_035006228.1|damageinducibleprotein[Corynebacteriumjeikeium]/11657  -----------------LNDISLLNVDLNIATDHVDGSMNAMERNTKLAWLE---SALP-
gi|737006250|ref|WP_035002053.1|damageinducibleprotein[Corynebacteriumjeikeium]/11658  -----------------LNDVSLLNVDLSIATDHVDGSMNAMERNTKLAWLE---GALP-
gi|923018168|ref|WP_053412254.1|damageinducibleprotein[Corynebacteriumlactis]_gi|922845184|gb|ALA67504.1|helicase[Corynebacter/  -----------------LSDVSLHNVDLAVAAQHVDGGMNALERGTRLSWLE---SPAV-
gi|923018885|ref|WP_053412970.1|damageinducibleprotein[Corynebacteriumlactis]_gi|922845845|gb|ALA68165.1|helicase[Corynebacter/  -----------------LGDVSLHNINLAVAAQHVDGGMNALERGTRLSWLE---SPAV-
gi|512464870|ref|WP_016422869.1|hypotheticalprotein[Corynebacteriumsp.HFH0082]_gi|512047830|gb|EPD47505.1|hypotheticalproteinHM  -----------------LNDVSLHNVDVSISAQHVDGGMNALERGTKLSWLE---SPAV-
gi|881012697|ref|WP_048733406.1|damageinducibleprotein[Corynebacteriumurealyticum]/11670  -----------------LNDVSLHNVDVSISAQHVDGSMNALERGTKLSWLE---SPAA-
gi|755608478|ref|WP_042530519.1|damageinducibleprotein[Corynebacteriumsingulare]_gi|754340239|gb|AJI78730.1|putativehelicase[C/  -----------------LNDVSLTNIDLNVAAQHVDGGMNAMERGTRLSWLE---SPAG-
gi|491421796|ref|WP_005279593.1|helicase[Corynebacteriumaccolens]_gi|227077180|gb|EEI15143.1|helicaseCterminaldomainprotein[Co/  -----------------LSDVSLTNVDLHIAAQHVDGGMNAMERGTKLSWIE---SPAA-
gi|550752286|gb|ERS51451.1|hypotheticalproteinHMPREF1282_00033[Corynebacteriumsp.KPL1856]_gi|550759961|gb|ERS58964.1|hypothetic  -----------------LNDVSLTNVDLRVAAQHVDGGMNAMERGTKLSWIE---SPAD-
gi|497955053|ref|WP_010269209.1|helicase[Corynebacteriumbovis]/11599  -------------------------KEPEVDARHVDGTMNALERGRLLQWLK---APVP-
gi|860580040|ref|WP_048470569.1|hypotheticalprotein[Mycobacteriumchlorophenolicum]_gi|859098575|gb|KMO76871.1|typeIrestrictione  ------G----------H-RVAETNRDLICEVHHVDGTYNALERNAELAWLKS---PVA-
gi|262085905|gb|ACY21873.1|typeIIIrestrictionproteinressubunit[GordoniabronchialisDSM43247]/11632  ------N----------D---ATGNRDLTVSARHVDGTMNALQRNEALRWLRS--GSIG-
gi|495996338|ref|WP_008720917.1|typeIIIrestrictionendonucleasesubunitR[Rhodococcussp.AW25M09]_gi|443412350|emb|CCQ18455.1|TypeI  ------G----------E-QVDATNRDLVVSARHVDGTFNALKRNEQLSWLKA---PVP-
gi|739284636|ref|WP_037147410.1|damageinducibleprotein[Rhodococcusfascians]/11641  ------G----------E-QVDATNRDLVVSARHVDGTFNALKRNEQLSWLKA---PVP-
gi|739328195|ref|WP_037190574.1|damageinducibleprotein[Rhodococcusfascians]/11643  ------G----------E-QVDATNRDLVVSARHVDGTFNALKRNEQLSWLKA---PVP-
gi|811698335|emb|CMN21041.1|helicase[Mycobacteriumtuberculosis]/11725  ------------------------GPGLACSVRHVDGTFNALVRNEQLAWLKG---VVA-
gi|801577898|emb|CFK23773.1|helicase[Mycobacteriumtuberculosis]/11675  ------------------------GPGLACSVRHVDGTFNALVRNEQLAWLKG---VVA-
MTU18II/11606  ------------------------GPGLACSVRHVDGTFNALVRNEQLAWLKG---VVA-
gi|887719858|emb|CMT27886.1|helicase[Mycobacteriumtuberculosis]/11667  ------------------------GPGLACSVRHVDGTFNALVRNEQLAWLKG---VVA-
gi|621694743|gb|KAS87156.1|hypotheticalproteinAL70_01801[MycobacteriumtuberculosisTKK_03_0115]/11604  ------------------------GPGLACSVRHVDGTFNALVRNEQLAWLKG---VVA-
gi|888128072|emb|CLQ84422.1|helicase[Mycobacteriumtuberculosis]_gi|889917090|emb|CLY86893.1|helicase[Mycobacteriumtuberculosis]  ------------------------GPGLACSVRHVDGTFNALVRNEQLAWLKG---VVA-
gi|884823424|emb|CKL96672.1|helicase[Mycobacteriumtuberculosis]/11466  ------------------------GPGLACSVRHVDGTFNALVRNEQLAWLKG---VVA-
gi|505103239|ref|WP_015290341.1|hypotheticalprotein[Mycobacteriumcanettii]_gi|432162733|emb|CCK60117.1|Conservedproteinofunknow  ------------------------GTGLTCSVRHVDGTFNALVRNEQLAWLKG---VVA-
gi|505116250|ref|WP_015303352.1|hypotheticalprotein[Mycobacteriumcanettii]_gi|432158726|emb|CCK56026.1|Conservedproteinofunknow  ------------------------GPGLACSVRHVDGTFNALVRNEQLAWLKG---VVA-
gi|432154735|emb|CCK51974.1|Conservedproteinofunknownfunction(part1)[MycobacteriumcanettiiCIPT140060008]/11588  ------------------------GPGLACSVRHVDGTFNALVRNEQLAWLKG---VVA-
gi|809008027|ref|WP_046295375.1|hypotheticalprotein[Mycobacteriumsp.UM_Kg17]/11624  ------G----------Q-NVEATNRGLTCAVHHVDGTFNALQRNAELTWLKG---VVA-
gi|808802633|ref|WP_046284139.1|hypotheticalprotein[Mycobacteriumsp.UM_NZ2]/11624  ------G----------Q-LVSATNRGLTCSVHHVDGTFNALQRNAELTWLKG---VVA-
gi|750125774|ref|WP_040429852.1|damageinducibleprotein[Corynebacteriumnuruki]/11668  ------G----------H-SIEGTNRDLRCEVHHVDGTFNALQRNEELTWLK---APVA-
gi|602237614|gb|EYT60960.1|damageinducibleprotein[Dietziasp.UCDTHP]/11473  ------G----------R-EILETNRNLSCHVHHVDGTYNAMRRNEELSWLK---APVA-
gi|665897412|ref|WP_031264618.1|damageinducibleprotein[Dietziasp.UCDTHP]_gi|602237599|gb|EYT60945.1|helicase[Dietziasp.UCDTH/11  ------G----------R-EILETNRNLSCQVHHVDGTYNAMRRNEELSWLK---APVA-
gi|502903674|ref|WP_013138650.1|helicase[Segniliparusrotundus]_gi|296181291|gb|ADG98197.1|typeIIIrestrictionproteinressubunit[S  ------S----------DENAAANNPDLYCSVEHVDGTFNALKRNARLGWLQ---APLA-
gi|780248306|ref|WP_045538930.1|hypotheticalprotein[Gordoniasp.no.9]_gi|778142514|gb|KJR06917.1|hypotheticalproteinUG54_12320[G  -------------------DPADNNADLYCSVRHVDGTYNALQRTSRINWLKGISEPLS-
gi|665897368|ref|WP_031264574.1|damageinducibleprotein[Dietziasp.UCDTHP]_gi|602239137|gb|EYT62483.1|helicase[Dietziasp.UCDTH/11  ------G----------V-DVADTNRSLNCSVHHVDGTFNALKRNEELTWLK---AEPD-
gi|493372923|ref|WP_006329236.1|helicase[Gordoniarhizosphera]_gi|403207917|dbj|GAB88168.1|hypotheticalproteinGORHZ_006_00370[Go  ------G----------H-QIDTTNRDLTCDIKHVDGTFNALRRNEALSWLK---APVP-
gi|764904870|ref|WP_044487821.1|hypotheticalprotein[Mycobacteriumtuberculosis]_gi|625005353|gb|KBZ57120.1|hypotheticalproteinK8  ------G----------I-DINETNLGLTVGVRHVDGTFNALERNRELQWLK---APLP-
gi|927068212|ref|WP_053778441.1|damageinducibleprotein[Gordoniasp.NB41Y]_gi|926660541|gb|EMP14526.2|damageinducibleprotein[G/11  ------G----------H-DVNTTNLDLGVQVHHVDGTYNALERNRELQWLK---APLP-
gi|935514169|ref|WP_054429894.1|damageinducibleprotein[Mycobacteriumimmunogenum]_gi|929801120|gb|KPG29247.1|damageinduciblepr/1  ------G----------V-TINETNLDLAVQVRHVDGTFNALERNRELQWLK---APLP-
gi|941317728|ref|WP_055118574.1|hypotheticalprotein[Mycobacteriumperegrinum]/11657  ------G----------V-AINETNLDLAVQVHHVDGTYNALERNRELQWLK---APLP-
gi|933683673|gb|ALG86377.1|hypotheticalproteinACH46_20120[Gordoniasp.QH11]/11636  ------G----------S-DVDSTNLDLRCEVHHVDGTFNALERNAELAWLK---APVP-
gi|873553947|emb|CRY84110.1|Predictedhelicase[Nocardiafarcinica]/11688  ------G----------H-EVASTNLDLSCSVHHVDGTFNALQRNAELAWLK---APVP-
gi|499525749|ref|WP_011212389.1|helicase[Nocardiafarcinica]_gi|54019339|dbj|BAD60707.1|hypotheticalprotein[NocardiafarcinicaIFM  ------G----------H-EITATNLDLSCSVHHVDGTFNALQRNAELAWLK---APVP-
gi|640109609|ref|WP_024794187.1|damageinducibleprotein[Tomitellabiformata]/11653  ------G----------N-RVDATNLDLTCAVRHVDGTFNALERNRELAWLK---APLP-
gi|640109991|ref|WP_024794334.1|hypotheticalprotein[Tomitellabiformata]/11639  ------G----------N-RVDATNLDLTCAVRHVDGTFNALERNRELAWLK---APLP-
gi|939125496|ref|WP_054831438.1|damageinducibleprotein[Rhodococcuserythropolis]/11669  ------G----------G-AVDSTNLDLTCSVRHVDGTFNALQRNEQLSWLK---APIP-
gi|910258550|ref|WP_050062711.1|hypotheticalprotein[Rhodococcussp.RD6.2]_gi|846871967|emb|CRK49225.1|conservedhypotheticalprote  ------G----------H-AVSSTNLDLACSVHHVDGTFNALQRNAELAWLK---APVP-
gi|797148189|gb|KJY52358.1|putativeHelicase[Bifidobacteriumasteroides]/11801  ---------------------HKVEGLLKTSVKHVDGSMPARKRNDLLSWLGE---PP--
gi|535476570|gb|EFG27357.2|hypotheticalproteinHMPREF9020_00999[ScardoviainopinataF0304]/11827  ---------------------ASLEGTIIPKVEHVDGSMPTKERRKLLNWLAD---RQE-
gi|737482729|ref|WP_035462496.1|hypotheticalprotein[Alicyclobacillusmacrosporangiidus]/11512  ------------------------SDEYRTFVAHMDGTMSMRERSRLLRCLET---LSD-
gi|685434354|gb|KGA95679.1|helicase[BacillusalcalophilusATCC27647=CGMCC1.3604]/11575  -----------------------NSNAFRVEVDHADGSMNALEKNKKISWLKS---D-V-
gi|945370253|gb|KQU27251.1|helicase[Bacillussp.Leaf13]/11557  -----------------------YAHQYEIEIDHADGSMNALEKNKKISWLKD---E-V-
gi|880997015|ref|WP_048719116.1|helicase[Enterococcushirae]/11563  -----------------------QATPYKVEIDHADGTMNALQKNEKINWLKS---D-V-
gi|945356929|gb|KQU14100.1|helicase[Bacillussp.Leaf49]/11547  -----------------------HEHQYKIEIDHADGSMNALEKNKKISWLKD---D-V-
gi|763482876|gb|KIZ53217.1|helicase[Bacillussafensis]/11563  -----------------------QSDIFNIEIEHADGSMNALQKNEKISWLKA---G-V-
gi|852223292|ref|WP_048309707.1|helicase[Anaerobacillusmacyae]_gi|850322910|gb|KMM38590.1|helicase[Anaerobacillusmacyae]/11573  -----------------------QVDPFQVEIDHADGSMNAMQKNEKISWLKS---D-V-
gi|739708665|ref|WP_037562619.1|helicase[Sporolactobacillusterrae]/11573  -----------------------QSEPVRVEIEHADGSMNALQKNEKISWLKA---N-V-
gi|496687561|ref|WP_009329104.1|MULTISPECIES:helicase[Bacillus]_gi|317390867|gb|EFV71668.1|endonucleaseandmethylaseLlaGI[Bacill  -----------------------QREPVRVEIDHADGSMNALQKNEKISWLKG---E-V-
gi|930213599|gb|KPH74225.1|helicase[Oceanobacilluscaeni]/11569  -----------------------QREPVRVEIEHADGSMNALQKNEKISWLKS---D-V-
gi|516366003|ref|WP_017756036.1|helicase[Bacillussp.ZYK]/11571  -----------------------QAEPVRVEIDHADGSMNALQKNEKISWLKA---E-V-
gi|488909977|ref|WP_002821052.1|helicase[Oenococcusoeni]_gi|399965230|gb|EJN99855.1|endonucleaseandmethylaseLlaGI[Oenococcusoen  ------------------------DDSYSIDVKHVDGTLNALQKKDALDWLAD---DTI-
gi|495019967|ref|WP_007745780.1|helicase[Oenococcuskitaharae]_gi|366983682|gb|EHN59081.1|typeIIIrestrictionenzyme,ressubunit[Oe  ------------------------DDSYAIDVRHVDGTLNALQKKDALDWLAD---DTI-
gi|489666119|ref|WP_003570406.1|typeIIIrestrictionendonuclease[Lactobacilluscasei]_gi|410527834|gb|EKQ02696.1|typeIIIrestrictio  ------------------------PNSFHVDVKHVDGSMNALQKKDAIDWLAT---DL--
gi|511447623|gb|EPC78110.1|superfamilyIIDNA/RNAhelicase[Lactobacillusparacaseisubsp.paracaseiLpp221}/11615  ------------------------PNSFRVDVKHVDGSMNALQKKDAIDWLAS---DL--
gi|497700411|ref|WP_010014595.1|helicase[Lactobacilluscoryniformis]/11585  ------------------------SNNFHVDVKHVDGSMNALQKKDAIDWLAA---DL--
gi|489784048|ref|WP_003687939.1|helicase[Lactobacillusmali]_gi|394428539|gb|EJF01080.1|superfamilyIIDNA/RNAhelicase[Lactobacill  ------------------------PNSFHVHVKHVDGSMNALQKKDAIDWLAS---DL--
```

```
gi|446780825|ref|WP_000858081.1|helicase[Staphylococcusaureus]_gi|374396604|gb|EHQ67835.1|typeIIIrestrictionenzyme,ressubunit[S   ------------------------KESFQIDVRHVDGKLNALRKKEALDWLTD---D-I-
gi|510796658|ref|WP_016171456.1|hypotheticalprotein[Enterococcusdispar]_gi|508167532|gb|EOT44083.1|hypotheticalproteinOMK_00227   ------------------------EDSFHIEVKHVDGTLNALQKKDAIDWLAG---D-I-
gi|612613473|gb|EZS30411.1|hypotheticalproteinW603_02482[StaphylococcusaureusVET0353R]_gi|612881917|gb|EZU93992.1|hypotheticalp   ------------------------EDSFHIEVKHVDGTLNALQKKEAIDWLAG---D-V-
gi|488332893|ref|WP_002402278.1|helicase[Enterococcusfaecalis]_gi|306513864|gb|EFM82466.1|helicaseCterminaldomainprotein[Enter/   ------------------------TIQQSINVRHVDGAMNTLEKKEALDWLSE---D-F-
gi|921166029|ref|WP_053111806.1|helicase[Enterococcusdurans]_gi|915771660|gb|AKX85928.1|helicase[Enterococcusdurans]/11562   ------------------------SIQQSINVRHVDGMMNTLEKKEALDWLSE---D-F-
gi|498525358|ref|WP_010825030.1|hypotheticalprotein[Enterococcusfaecalis]_gi|487059631|gb|EOJ86727.1|hypotheticalproteinWOC_005   ------------------------SIQQSINVRHVDGAMNTLEKKEALDWLSE---D-F-
gi|498412624|ref|WP_010722070.1|hypotheticalprotein[Enterococcusfaecium]_gi|486634654|gb|EOF75253.1|hypotheticalproteinSGC_0253   ------------------------SIQQSINVRHVDGMMNTLEKKEALDWLSE---D-F-
gi|498408534|ref|WP_010718364.1|hypotheticalprotein[Enterococcusfaecium]_gi|486609608|gb|EOF50873.1|hypotheticalproteinSCW_0257   ------------------------SIQQSINVRHVDGMMNTLEKKEALDWLSE---D-F-
gi|498407475|ref|WP_010717483.1|hypotheticalprotein[Enterococcusfaecalis]_gi|486594855|gb|EOF36348.1|hypotheticalproteinSCK_005   ------------------------SIQQSINVRHVDGMMNTLEKKEALDWLSE---D-F-
gi|514885392|ref|WP_016615344.1|helicase[Enterococcusfaecalis]_gi|514505843|gb|EPH71676.1|helicaseprotein[Enterococcusfaecalis0   ------------------------TIQQSINVRHVDGAMNTLEKKEALDWLSE---D-F-
gi|325157603|gb|EGC69759.1|helicaseCterminaldomainprotein[EnterococcuscasseliflavusATCC12755]/11561   ------------------------SIQQSINVRHVDGMMNTLEKKEALDWLSE---D-F-
gi|895856206|ref|WP_048967668.1|helicase[Enterococcusfaecalis]/11551   ------------------------SIQQSINVRHVDGMMNTLEKKEALDWLSE---D-F-
gi|639458836|ref|WP_024636366.1|helicase[Enterococcusfaecium]_gi|658176502|gb|KEI51159.1|helicase[EnterococcusfaeciumUC7256]/1/   ------------------------SIQQSINVRHVDGMMNTLEKKEALDWLSE---D-F-
gi|488266511|ref|WP_002337719.1|hypotheticalprotein[Enterococcusfaecium]_gi|430581409|gb|ELB19854.1|hypotheticalproteinOIQ_0351   ------------------------TIQQSINVRHVDGMMNTLEKKEALDWLSE---D-F-
gi|446353356|ref|WP_000431211.1|helicase[Staphylococcusaureus]_gi|253726329|gb|EES95058.1|helicaseCterminaldomainprotein[Staph/   -----------------------EEQSKKLSMRHADGTMNALQKGELLDWLAD---PNK-
gi|672712068|emb|CDM14336.1|DNAorRNAhelicaseofsuperfamilyII[StaphylococcusepidermidisPM221]/11464   -----------------------DQQTTKLSMKHADGSMNSLQKGEVLDWLAD---QNK-
gi|896298990|ref|WP_049279905.1|helicase[Staphylococcusaureus]/11571   -----------------------DQQTTKLSMKHADGSMNALQKGEVLDWLAD---PNK-
gi|541903432|gb|ERH36190.1|hypotheticalproteinSEQU_00085[StaphylococcusequorumUMCCNS924]/11578   -----------------------DQQTTKLSMKHADGSMNALQKGEVLDWLAD---SNK-
gi|488289747|ref|WP_002360955.1|helicase[Enterococcusfaecalis]_gi|256682216|gb|EEU21911.1|endonucleaseandmethylaseLlaGI[Enteroc   -----------------------EEESIHLSMRHADGTMNALQKGEILDWLAD---PEK-
gi|494392045|ref|WP_007207894.1|helicase[Enterococcusitalicus]_gi|315483798|gb|EFU74284.1|helicaseCterminaldomainprotein[Enter/   -----------------------EDESIHLSMRHADGTMNALQKGEVLDWLAD---PEK-
gi|446350692|ref|WP_000428547.1|helicase[Bacilluscereus]_gi|228727580|gb|EEL78721.1|RestrictionmodificationsystemLlaBIII[Bacil/   ------------------------DESVHLSMRHADGSMNALQKGEILDWLAD---PKK-
gi|488255302|ref|WP_002326510.1|hypotheticalprotein[Enterococcusfaecium]_gi|430480276|gb|ELA57459.1|hypotheticalproteinOGE_0473   -----------------------EEESIELSMRHADGSMNALQKGEVLDWLAD---PNK-
gi|843253770|ref|WP_047916698.1|helicase[Lactococcuspiscium]_gi|802124677|emb|CEN29502.1|EndonucleaseandmethylaseLlaGI,TypeISPr   -----------------------EDESIHLSMRHADGQMNALQKGEVLDWLAN---PNK-
LlaBIII/11578   -----------------------EDESIHLSMRHADGQMNALQKGEVLDWLAN---PNK-
gi|499320531|ref|WP_011011023.1|helicase[Listeriainnocua]_gi|16415805|emb|CAC42019.1|pli0021[ListeriainnocuaClip11262]/11569   -----------------------EDESIHLSMRHADGQMNALQKGEILDWLAN---PNK-
gi|760026859|ref|WP_043709973.1|helicase[Weissellacibaria]_gi|758337165|gb|KIU20061.1|UvsWhelicase[Weissellacibaria]/11567   -----------------------DEQSVHLSMKHADGQMNALQKGEILDWLAN---PNK-
LlaGI/11570   -----------------------EDESIHLSMRHADGKMNALQKGEILDWLAD---PNK-
gi|499988414|ref|WP_011669132.1|helicase[Lactococcuslactis]_gi|116109019|gb|ABJ74139.1|DNAorRNAhelicaseofsuperfamilyII[Lactococ   -----------------------EDESIHLSMRHADGQMNALQKGEILDWLAN---PNK-
gi|685938360|ref|WP_031670319.1|helicase[Listeriamonocytogenes]_gi|590403387|gb|EXL23261.1|hypotheticalproteinX846_1956[Listeri   -----------------------EDESIHLSMRHADGQMNALQKGEVLDWLAN---PNK-
gi|530793219|gb|EQC56240.1|helicase[Lactococcuslactissubsp.cremorisTIFN5]_gi|530793247|gb|EQC56263.1|helicase[Lactococcuslactis   -----------------------EDESIHLSMRHADGQMNALQKGEILDWLAN---PNK-
gi|695270006|ref|WP_032492075.1|hypotheticalprotein[Listeriamonocytogenes]_gi|695212872|ref|YP_009081522.1|hypotheticalproteinp   -----------------------EDESIHLSMRHADGQMNALQKGEVLDWLAN---PNK-
gi|504101310|ref|WP_014335297.1|helicase[Leuconostocmesenteroides]_gi|859247905|gb|AKO63885.1|helicase(plasmid)[Leuconostocmese   -----------------------DEQPVHLSMRHADGQMNALQKGEILDWLAN---PNK-
gi|488905190|ref|WP_002816265.1|helicase[Leuconostocmesenteroides]_gi|227351522|gb|EEJ41777.1|helicaseCterminaldomainprotein[L/   -----------------------YNQSVHLSMRHADGKMNALQKGEILDWLAD---PNK-
gi|518121285|ref|WP_019291493.1|MULTISPECIES:hypotheticalprotein[Lactococcus]/11588   ------------------------DNEFSVDVHHADGTMNALQKNTELDWLKS---DDI-
gi|754857213|ref|WP_042217902.1|helicase[Lactococcusgarvieae]/11549   ------------------------DTHFTAKVHHADGTMSALEKNRELDWLAD---KDL-
gi|640048455|ref|WP_024784762.1|helicase[Streptococcusmutans]/11564   --------------------------GYSVDVRHVDGGMNALQKSEALDWLAS---DDI-
gi|512695361|ref|WP_016476323.1|hypotheticalprotein[Veillonellasp.HPA0037]_gi|512079853|gb|EPD79018.1|hypotheticalproteinHMPREF   --------------------------SYSVAVRHVDGGMNALEKNEALDWLAS---NDI-
gi|636963514|ref|WP_024403747.1|helicase[Streptococcussuis]/11574   --------------------------GYSVNVRHVDGNMNALEKNEALDWLAS---DDI-
gi|881115312|ref|WP_048802949.1|helicase[Staphylococcuspasteuri]/11573   --------------------------GYFVDVRHVDGNMNALEKNEALDWLAS---DDI-
gi|446063996|ref|WP_000141851.1|helicase[Streptococcusmitis]_gi|307618725|gb|EFN97864.1|endonucleaseandmethylaseLlaGI[Streptoco   --------------------------GYSVNVRHVDGSMNALEKNEALDWLAS---DDI-
gi|488206609|ref|WP_002277817.1|restrictionmodificationsystemLlaBIII[Streptococcusmutans]_gi|449173033|gb|EMB75626.1|restricti/   --------------------------GYSVAVRHVDGGMNALQKNEALDWLAA---DDI-
gi|640023290|ref|WP_024781621.1|helicase[Streptococcusmutans]/11566   --------------------------GYSVNVRHVDGSMNALQKNEALDWLAA---DDI-
gi|814946581|ref|WP_046393781.1|helicase[Streptococcusuberis]_gi|810600885|gb|KKF57499.1|helicase[StreptococcusuberisB362]/1156   --------------------------GYSVNVRHVDGTMNAIEKNEALDWLAS---DDI-
gi|765470762|ref|WP_044720336.1|helicase[Streptococcussuis]/11559   --------------------------GYSVNVRHVDGNMNALEKNEALDWLAS---DDI-
gi|654507127|ref|WP_027976443.1|helicase[Streptococcusdevriesei]/11558   --------------------------GYSVNVRHVDGGMNALEKNEALDWLGS---DDI-
gi|530812902|gb|EQC72481.1|DNAorRNAhelicaseofsuperfamilyII[Streptococcussp.HSISS2]/11570   --------------------------GYSVNVRHVDGGMNALEKNGALDWLAS---DDI-
gi|489158846|ref|WP_003068504.1|helicase[Streptococcusintermedius]_gi|355367114|gb|EHG14827.1|hypotheticalproteinHMPREF9682_002   --------------------------GYSVNVRHVDGGMNALEKNEALDWLAS---DDI-
RM.Lru27782ORF13200P/11620   ------------------------EGSFSVDVHHVDGGLNAMEKKTELDWLAS----DM-
gi|492676546|ref|WP_005919236.1|DNAphosphorothioationsystemrestrictionenzyme[Pediococcusacidilactici]_gi|482517924|gb|EOA08103.   ------------------------DESFSVDVKHVDGSLNALEKKNALDWLAN----DM-
gi|498183116|ref|WP_010497272.1|helicase[Lactobacillusacidipiscis]/11563   ------------------------DESFSVDVHHVDGSLNALQKKNQLDWLAN----EI-
gi|822100485|ref|WP_046921865.1|helicase[Lactobacillusruminis]_gi|821684442|gb|KLA43321.1|restrictionmodificationsystemLlaBIII/   ------------------------EDSFSVDVHHVDGGLNALEKKSQLDWLAG----SI-
gi|935565832|ref|WP_054450311.1|helicase[Lactobacilluskunkeei]/11573   ------------------------SESFMVDVHHVDGGLNALQKTEQIDWLGD---EHI-
gi|752296887|ref|WP_041152901.1|helicase[Lactobacilluskunkeei]_gi|751663520|gb|KIM18358.1|helicase[Lactobacilluskunkeei]/11577   ------------------------SESFMVDVHHVDGGLNALQKTEQIDWLGD---EHI-
gi|495745291|ref|WP_008469870.1|helicase[Lactobacillushominis]_gi|394484442|emb|CCI81207.1|RestrictionmodificationsystemLlaBII/   ------------------------TESFKVDVHHADGSFNAMEKETEISWLGD---KNI-
gi|918672414|ref|WP_052543134.1|helicase[Lactobacillussp.]/11564   ---------------------SISENSYHVDVHHVDGGLNALEKENEIDWLGN---DDI-
gi|799141631|ref|WP_045927261.1|helicase[Lactobacilluskullabergensis]_gi|797155335|gb|KJY59264.1|putativerestrictionendonucleas   ---------------------NIAEDSFHVDVHHVDGGLNSMEKNSQIDWLGS---DDI-
gi|504379990|ref|WP_014567092.1|helicase[Lactobacillusjohnsonii]_gi|329666726|gb|AEB92674.1|putativerestrictionendonuclease[Lac   ------------------------TESFKVDVHHVDGGLNALEKEEQIDWLGN---NDI-
gi|489880179|ref|WP_003783646.1|DNAdamageinducibleprotein[Actinomycesnaeslundii]_gi|399901561|gb|EJN84440.1|typeIIIrestriction/   PELPDD---ADGT----SSDAPAQKRLGSVEVRHVDGSMNIMERNGLLSWLSG---DLGA
gi|496524497|ref|WP_009232780.1|DNAdamageinducibleprotein[Actinomycessp.oraltaxon849]_gi|365265413|gb|EHM95177.1|hypotheticalp/   PELPDD---ADGV----DSDTPSQKRLGSVKVRHVDGSMNIMERTSLLSWLSG---DLGA
gi|915397782|ref|WP_050783167.1|damageinducibleprotein[Actinomycesoris]/11694   PELPDD---ADGV----DSDTPSQKRLGSVEVRHVDGSMNIMERTSLLSWLSG---DLGA
gi|502925248|ref|WP_013160224.1|helicase[Propionibacteriumfreudenreichii]_gi|296921272|emb|CBL55824.1|helicase[Propionibacteriu   ------------------------TPQLDVAVHHVDGGMNALKRSEELAWLKA-------
gi|732164352|emb|CEG87481.1|helicase[Propionibacteriumfreudenreichii]/11618   -----------EQ----DSGGQGGAVEPVCSARHVDGTMNALQRSEALAWLKA-------
gi|739047402|ref|WP_036925058.1|damageinducibleprotein[Propionibacteriumthoenii]/11596   --------------------------GLQVEAHHVDGTMNALTRGEELAWLKA-------
gi|739112839|ref|WP_036982533.1|damageinducibleprotein[Propionibacteriumjensenii]/11594   --------------------------GLQVDAHHVDGTMNALTRADELAWLKA-------
gi|918023558|ref|WP_052314121.1|damageinducibleprotein[Nocardiathailandica]/11612   ---------------------RAGSNSLRVEAAHVDGGMGIHERNKHLDWLKA-------
gi|498380271|ref|WP_010694427.1|DNAdamageinducibleprotein[Saccharopolysporaspinosa]/11605   ---------------------DESRNPLRVEAQHVDGTMRIHTRNDKLAWLKQ-------
gi|749991459|ref|WP_040339809.1|damageinducibleprotein[CandidatusBlastococcusmassiliensis]/11597   ---------------------PEDRPRLRVQAAHVDGTMGVHERNTHLAWLKE-------
```

```
gi|754177801|ref|WP_041796231.1|damageinducibleprotein[Modestobactermarinus]/11592    ---------------------PEDRPRLRVQAAHVDGTMGVHERNAHLAWLKE-------
gi|504188248|ref|WP_014375350.1|DNAdamageinducibleprotein[Blastococcussaxobsidens]_gi|378782790|emb|CCG02456.1|putativeHelicas/    ---------------------PDDRPKLRVEAAHVDGTMGVHERNTHLAWLKE-------
gi|946476713|gb|KRF26798.1|damageinducibleprotein[Phycicoccussp.Soil803]/11609    -----------DS----GD--ARERNRMQVEIRHVDGTMNAVERGNELAWLKS---GS--
gi|700176672|gb|KGN37509.1|DNAdamageinducibleprotein[KnoelliasubterraneaKCTC19937]/11633    -----------DT----DA--VRERNRMQVQIRHVDGTMNAVDRGNELAWLKS---GA--
gi|700168841|gb|KGN29914.1|hypotheticalproteinN802_10215[KnoelliasinensisKCTC19936]/11631    -----------DS----GA--ARERNRMQVEIRHVDGTMNAVDRGNELAWLKS---GS--
gi|946426241|gb|KRE76610.1|damageinducibleprotein[Arthrobactersp.Soil763]/11606    -----------D---------GEPVNVLDCEVRHVDGTFNALERNTRLDWLRA-------
gi|517318525|ref|WP_018500927.1|hypotheticalprotein[Frankiasp.BCU110501]/11592    -----------PD----G------SNPLKCTVQHVDGGMNALERNTKLDWLRA-------
gi|499072429|ref|WP_010851327.1|Helicase[Tetrasphaeraelongata]_gi|478756579|emb|CCH71501.1|putativeHelicase[Tetrasphaeraelongat    -----------AD----G------EPVLTCEIQHVDGTFNALERNTRLDWLRA-------
gi|750080666|ref|WP_040385350.1|damageinducibleprotein[Demetriaterragena]/11625    -----------PE----ADDRGHRSSDLRVECEHVDGTYNALERNKKLSWLKE-------
gi|752558292|ref|WP_041229523.1|damageinducibleprotein[Dermacoccusnishinomiyaensis]_gi|664689315|gb|AIF41904.1|damageinducibl/1    -----------DG----A----ALSTDLRLEVEHVDGTFNALERNKYLDWLKA-------
gi|829089213|ref|WP_047310701.1|damageinducibleprotein[Dermacoccussp.PE3]_gi|828384812|gb|KLO63770.1|damageinducibleprotein[D/1    -----------DA----AD---AQSNDLLIEIKHVDGSFNALARNERLDWLKA-------
gi|748756635|ref|WP_040014750.1|damageinducibleprotein[Dermacoccussp.Ellin185]/11596    -----------DA----AE---EQSTDLKIEIQHVDGSFNALARNERLDWLKA-------
gi|916534724|ref|WP_051141848.1|damageinducibleprotein[Actinomycesneuii]/11632    --------------------NTDTTDNLGVQCQHVDGTMNAIERGEKLDWLKE---GESS
gi|921140030|ref|WP_053090276.1|damageinducibleprotein[Rothiamucilaginosa]/11616    --------------------NDDPTDDLGVQVRHIDGGFNAQSRAELLNWLKQ---EPN-
gi|896406751|ref|WP_049343749.1|damageinducibleprotein[Rothiamucilaginosa]/11647    --------------------NDDPTDDLGVQVRHIDGGFNAQSRAELLSWLKQ---EPN-
gi|723608234|gb|AIY01610.1|putativehelicase[Arthrobactersp.PAMC25486]/11613    --------------------NDDPTDDLRVEAKHVDGTFNAVRRGERLDWLQA---DPEV
gi|946394527|gb|KRE45160.1|damageinducibleprotein[Arthrobactersp.Soil736]/11616    --------------------NDDPTDDLKVQVRHIDGGFNAVARAERLDWLKE---DFNH
gi|515765304|ref|WP_017197904.1|hypotheticalprotein[Arthrobactersp.M2012083]/11648    --------------------NDDPTDDLKVEVKHVDGGFNAITRAERLDWLKE---DLDT
gi|945315587|gb|KQT73127.1|damageinducibleprotein[Microbacteriumsp.Leaf436]/11612    GRIEGSGED--------------TSRLLHAEARHVDGTQSMLVRNEKLDWLKE---AS--
gi|759908497|ref|WP_043594599.1|damageinducibleprotein[Frigoribacteriumsp.MEB024]_gi|758271359|gb|KIU03346.1|damageinduciblep/1    -SIAGRRID---------DTDYG-QQTYALEARHVDGTMNVLERNTHLDWLKA---DTE-
gi|945072850|gb|KQR34237.1|hypotheticalproteinASF75_00055[Curtobacteriumsp.Leaf154]/11597    -SIAGRRID---------LSEDGDAQTYRLEARHVDGGMNILERNAHLDWLKA---DTS-
gi|551263152|ref|WP_022883363.1|DNAdamageinducibleprotein[Glaciibactersuperstes]/11621    TQVTGQGAL--------------GDPRLRLEAKHVDGTMNVMIRNEMLDWLKA---DTD-
gi|945452341|gb|KQV08491.1|hypotheticalproteinASC63_10170[Leifsoniasp.Root112D2]/11623    LEIAGENADATQR----AAIDKATVQPLGIQAKHVDGTMNVLVRNTMLDWLKE---ETD-
gi|742759483|gb|KIC69912.1|damageinducibleprotein[Kocuriarhizophila]/11610    --------------------Q-----DLLLRAEHVDGTMNVAQRGSKLRWLEA---EPE-
gi|737919995|ref|WP_035885304.1|damageinducibleprotein[Kocuriarhizophila]/11611    --------------------Q-----DLLLRAEHVDGTMNVAQRGSKLRWLEA---EPE-
gi|737997701|ref|WP_035959325.1|damageinducibleprotein[Kocuriamarina]_gi|725823884|gb|KHE75603.1|damageinducibleprotein[Kocur/1    --------------------Q-----DLLLRAEHVDGTMNVAQRGEKLRWLEA---EPE-
gi|664521070|ref|WP_031037650.1|damageinducibleprotein[Streptomycessp.NRRLWC3774]/11624    --------------------D-----VLHCEVGHVDGTFNTLQRNRLLDWLKQ---DPG-
gi|500101034|ref|WP_011777041.1|DNAdamageinducibleprotein[Arthrobacteraurescens]_gi|119951547|gb|ABM10457.1|putativeHelicase[A/    --------------------G-----SLRCEGEHVDGTFNVLERNEKLDWLKA---DTAS
gi|916871953|ref|WP_051479009.1|damageinducibleprotein[Arthrobactersp.H5]/11588    --------------------G-----SLRCEGEHVDGTFNVLDRSAKLDWLKA---DTT-
gi|612084649|gb|EZP43369.1|TypeIIIrestrictionenzyme,ressubunit[Micrococcusluteus]/11625    --------------------P--MTPDLRVEASHVDGTFPMAQRVRELDWLKQ---P-A-
gi|759739708|ref|WP_043450422.1|damageinducibleprotein[Arthrobacterphenanthrenivorans]_gi|742758264|gb|KIC68716.1|damageinduc/1    --------------------DVDAVPGLKLEAEHVDGTFNVLERSAKLDWLKA---EDA-
gi|636845448|ref|WP_024366823.1|damageinducibleprotein[Arthrobactersp.TB26]/11605    --------------------D-----ALKLEAEHVDGTFNVLERSAKLDWLQD---ETK-
gi|500009025|ref|WP_011689743.1|DNAdamageinducibleprotein[Arthrobactersp.FB24]_gi|116613051|gb|ABK05760.1|typeIIIrestrictionen/    --------------------D-----ALKLEAEHVDGTFNVLERSAKLDWLQD---ETK-
gi|821249847|gb|KKZ12083.1|hypotheticalproteinTQ37_06090[CandidatusSynechococcusspongiarum15L]/11436    ----------------------NGEAVPSVSARHVDGTFNATARADALAFLEE---TP--
gi|739446902|ref|WP_037306885.1|damageinducibleprotein[Ruegeriahalocynthiae]/11592    --------------------DDETEDMLSCAVEHVDGTFNAKTRGELLHWLGD---DV--
gi|640620081|ref|WP_025048131.1|damageinducibleprotein[Sulfitobactermediterraneus]_gi|751956650|gb|KIN77213.1|TypeIIIrestricti/    --------------------DDEDAEHISCEVKHVDGTFNAKTRGELLHWLSD---DV--
gi|740247634|ref|WP_038088583.1|damageinducibleprotein[Thiobacillusprosperus]_gi|684049748|gb|KFZ90853.1|putativehelicase[Thio/    ----------------------DDDSMVNATLHHVDGTMNMARRKDELDWLKD---EP--
gi|518420321|ref|WP_019590528.1|hypotheticalprotein[Thioalkalivibriosp.ALE20]/11603    --------------------EKHQQGLVPCELRHVDGTMNAHQRQVELEWLKE---SS--
gi|500156826|ref|WP_011831496.1|DNAdamageinducibleprotein[Methylibiumpetroleiphilum]_gi|124261892|gb|ABM96881.1|adeninespecifi/    ---------------------GEETKLVECDLDHVDGGMNALVRLNKLNWLRA---NP--
gi|769978537|ref|WP_045094522.1|damageinducibleprotein[Legionellafallonii]_gi|698841736|emb|CEG55682.1|Putativeproteinwithboxh/    ---------------------NQHLKMVPCAVRHIDGSMNSQTRKQELDWLKE---E---
gi|553752541|ref|WP_023085344.1|hypotheticalprotein[Pseudomonasaeruginosa]_gi|552201952|gb|ERU62653.1|hypotheticalproteinQ088_0    --------------------GDESDGMVRCDLDHVDGTMNALKRKQALDWLKA---DA--
gi|880980418|ref|WP_048704461.1|damageinducibleprotein[Thauerahumireducens]_gi|875132785|gb|KMU70944.1|damageinducibleprotein/1    --------------------GAEPTGMVRCNLDHVDGTMNALTRKRALDWLKA---DL--
gi|740135732|ref|WP_037982815.1|damageinducibleprotein[Thauerasp.27]/11603    --------------------GAEPTGMVRCDLDHVDGTMNALTRKRALDWLKA---DL--
gi|738017371|ref|WP_035978332.1|damageinducibleprotein[Burkholderiaglumae]/11380    --------------------GDENTGMVNCTLDHVDGSMNALIRQNKLDWLKS---GV--
gi|498506003|ref|WP_010806671.1|typeIIIrestrictionenzyme,ressubunit[Pandoraeasp.SD62]_gi|490314561|gb|EON12879.1|typeIIIrestri/    --------------------DDEDTGMVNCSLDHVDGGMNALIRLNKLEWLKA---GV--
gi|500117748|ref|WP_011793753.1|DNAdamageinducibleprotein[Acidovoraxcitrulli]_gi|120587742|gb|ABM31182.1|typeIIIrestrictionenz/    -EPEGQ--------------DEALQDMVACQLQHVDGTMNALKRQTSLDWLKA---EV--
gi|651617328|ref|WP_026611444.1|damageinducibleprotein[Methylocaldumszegediense]/11601    ----GE--------------EDASDGMVGCDLEHVDGTMNALLRQTYLDWLKA---DP--
gi|737210208|ref|WP_035194994.1|damageinducibleprotein[Acidithiobacillusferrivorans]_gi|669953220|emb|CDQ11755.1|Adeninespecif/    ------------------------DGSMALEAEHVDGTMDVLTRNHKLDWLKA---NTGS
gi|935697386|ref|WP_054506180.1|damageinducibleprotein[Achromobactersp.]_gi|928716153|emb|CUK12836.1|Hefnuclease[Achromobacter/    ------------------------EYLPALEAKHVDGTMNVVDRNRSLSWLKE---NIGQ
gi|429502517|gb|ELA00827.1|adeninespecificDNAmethyltransferase[Cupriavidussp.HMR1]/11599    ------------------------EYLPALEAKHVDGTMNVVDRNRSLSWLKE---NIGQ
gi|506356642|ref|WP_015876361.1|DNAdamageinducibleprotein[Burkholderiaglumae]_gi|237877158|gb|ACR29491.1|AdeninespecificDNAmet/    ------------------------EYLPALEAKHVDGTMNVVERNQKLSWLKS---NIGS
gi|696406787|ref|WP_032978003.1|damageinducibleprotein[Bordetellahinzii]_gi|625335076|gb|KCB49343.1|typeIIIrestrictionenzyme,r/    ------------------------DYLPALEAKHVDGTMNVVERNQKLSWLKD---NIGK
gi|817529125|gb|KKO72587.1|damageinducibleprotein[Kerstersiagyiorum]/11592    ------------------------DYLPALEAKHVDGTMNVVERNQKLSWLKD---NIGK
gi|737048910|ref|WP_035044118.1|damageinducibleprotein[Aquabacteriumsp.NJ1]_gi|699052896|gb|KGM38706.1|damageinducibleprotein/1    ------------------------DYLPALEAKHVDGTMNVVERNQKLSWLKD---NIAK
gi|822492519|ref|WP_046933319.1|MULTISPECIES:damageinducibleprotein[Xanthomonas]_gi|821703525|gb|KLA61196.1|damageinduciblepr/1    ------------------------DYLPALEAKHVDGTMNVVERNQKLSWLKG---NIGK
gi|751252861|ref|WP_040963740.1|damageinducibleprotein[Xanthomonasfuscans]_gi|749808363|gb|KIJ03887.1|damageinducibleprotein[/1    ------------------------DYLPALEAKHVDGTMNVVERNQRLSWLKG---NIGK
gi|496774980|ref|WP_009368792.1|DNAdamageinducibleprotein[Bilophilasp.4_1_30]_gi|345039902|gb|EGW44205.1|hypotheticalproteinHM/    -------------------------NLPRLFAEHVDGTMNVVERNQKLSWLKA---NAVS
gi|499241912|ref|WP_010939452.1|DNAdamageinducibleprotein[Desulfovibriovulgaris]_gi|46580580|ref|YP_011388.1|adeninespecificDN/    -------------------------PLPKLEAQHVDGTMNVVERNQKLAWLKE---NANA
gi|504274913|ref|WP_014462015.1|DNAdamageinducibleprotein[Bacillusmegaterium]_gi|345446919|gb|AEN91936.1|AdeninespecificDNAmet/    -----------------------SDSSLRCEVEHVDGTFNALDRNKKLDWLKE---ET--
gi|504895966|ref|WP_015083068.1|typeIIrestrictionenzymeandmethyltransferasesubtypeG[Anabaenasp.90]_gi|413972424|gb|AFW96512.1|t    -----------------------DETILQCELQHVDGKQNALQRNEKLEWLKA---EPLH
gi|518327765|ref|WP_019497972.1|hypotheticalprotein[Calothrixsp.PCC7103]/11600    -----------------------DEDILHCEVKHVDGTQNSLQRNQKLDWLRE---DT--
gi|652765503|ref|WP_027079917.1|damageinducibleprotein[Luteimonasmephitis]/11593    -----------------------DDSLLRCEVEHVDGTFNVLRRNAKLDWLKE---DT--
gi|896539951|ref|WP_049442123.1|damageinducibleprotein[Stenotrophomonasmaltophilia]/11591    -----------------------D-DLLRCEVQHVDGTYNVLLRNQKLDWLKE---DA--
gi|500217900|ref|WP_011888043.1|DNAdamageinducibleprotein[Geobacillusthermodenitrificans]_gi|134267954|gb|ABO68149.1|Conserved/    -----D-----------------DENLLMCEVRHVDGTQNIIERNANLQWLKE---E---
gi|696570298|ref|WP_033102014.1|damageinducibleprotein[Thermoactinomycesdaqus]/11594    -----E-----------------VENLLVCEVNHVDGTHNIVERNSKLQWLRE---N---
gi|251772067|gb|EES52637.1|putativehelicase[Leptospirillumferrodiazotrophum]/11618    -----D--------------T-DEDSPLKCEVRHVDGTFNILQRNSLLEWLKS---DE--
gi|738124101|ref|WP_036082108.1|damageinducibleprotein[Leptospirillumferriphilum]_gi|685243306|gb|KGA94177.1|putativehelicase[/    -----D--------------T-EDDPPLNCEVRHVDGTFNILQRNSLLKWLKS---DE--
```

```
gi|558699223|ref|WP_023524766.1|helicase[Leptospirillumsp.GroupII'CF1']_gi|206603764|gb|EDZ40244.1|Putativehelicase[Leptospiri/      -----D--------------T-EEDPPLKCEVRHVDGTFNILQRNSLLEWLKS---DE--
gi|406774462|gb|AFS53887.1|putativehelicase[LeptospirillumferriphilumML04]/11610                                                     -----H--------------EEIRESPLKCEVNHVDGTFNILLRNSLLDWLKS---D---
gi|740719463|ref|WP_038504749.1|damageinducibleprotein[Leptospirillumferriphilum]_gi|640838125|gb|AIA30173.1|damageinduciblep/1      -----H--------------EEIRESPLKCEVNHVDGTYNILLRNSLLDWLKS---D---
gi|517810766|ref|WP_018980974.1|hypotheticalprotein[Saccharibacterfloricola]/11733                                                   ------------------------LTDKQAEARHVDGSMTGSEREEALRWLKA---DTP-
gi|502471675|ref|WP_012798533.1|helicase[Slackiaheliotrinireducens]_gi|256791761|gb|ACV22431.1|predictedhelicase[Slackiaheliotr      ----------------------VDLPEFYCDIQHVDGSMNAKDRKDKLAWLAD----VD-
gi|657871713|ref|WP_029576297.1|hypotheticalprotein[Bifidobacteriumthermacidophilum]_gi|673007521|gb|KFJ02535.1|helicase[Bifido      ------------------E---GQAQPLRVTIDHIDGTMDSELRKEKLAWLAA---DEG-
gi|673004757|gb|KFI99791.1|DNAhelicaserestrictionenzymetypeIIIRessubunit[Bifidobacteriumthermacidophilumsubsp.porcinum]/11682       ------------------E---RQAQPLHVTIDHIDGTMDSELRKEKLAWLAA---DEG-
gi|917316092|ref|WP_051922804.1|hypotheticalprotein[Bifidobacteriumstellenboschense]_gi|673003774|gb|KFI98812.1|helicase[Bifido      ------------------RDVIAANHEVHVEIDHVDGGMDAATRRGKLDWLAA---KTA-
gi|705415694|ref|WP_033502332.1|hypotheticalprotein[Bifidobacteriumpseudolongum]_gi|672982411|gb|KFI77685.1|DNAdamageinducible/      ------------------AATIHANNVVTVDIKHVDGTMDAQTRADRLDWLSA---DTP-
gi|916591418|ref|WP_051198509.1|hypotheticalprotein[Bifidobacteriumsp.AGR2158]/11704                                                 ------------------RETISNGRMVKVEIDHVDGTMDAEERREKLDWLAE---DMP-
gi|772242982|gb|AJW78255.1|hypotheticalproteinVO01_03165[Clavibactermichiganensissubsp.insidiosus]/11642                             ---N--------------DDPSDDL---TVQARHIDGTMNATERGTLLDWLKS---EGVT
gi|916649818|ref|WP_051256909.1|damageinducibleprotein[Actinomycesgraevenitzii]/11677                                                 ---N--------------ADASDNL---QVECRHVDGTMNAAVRAQEMAWLTE---GSGT
gi|496504578|ref|WP_009212861.1|helicase[Actinomycessp.oraltaxon180]_gi|315312695|gb|EFU60776.1|helicase[Actinomycessp.oraltaxo      ---N--------------DDASDNL---QVECHHVDGTMNAAVRSREMDWLTE---GAGT
gi|494862206|ref|WP_007588306.1|helicase[Actinomycessp.ICM39]_gi|398362799|gb|EJN46470.1|typeIIIrestrictionenzyme,ressubunit[Ac      ---N--------------DDASDNL---QVECRHVDGTMNAAVRAREMDWLTE---GAGT
gi|881057111|ref|WP_048774974.1|damageinducibleprotein[Sanguibacterkeddieii]/11697                                                   ---N--------------DDASDNL---QVECRHVDGTMNAAVRAREMDWLTE---GAGT
gi|881020459|ref|WP_048740808.1|damageinducibleprotein[Kytococcussedentarius]/11704                                                  ---N--------------DDASDNL---QIECRHVDGTMNAAVRAREMDWLTE---GAGT
gi|881052313|ref|WP_048770555.1|damageinducibleprotein[Cellulomonasflavigena]/11705                                                  ---N--------------DDASDNL---QVECRHVDGTMNAAVRAREMDWLTE---GAGT
gi|490108682|ref|WP_004009424.1|helicase[Mobiluncuscurtisii]_gi|315490465|gb|EFU80089.1|helicaseCterminaldomainprotein[Mobilun/      ---N--------------DDESDNL---EVQCQHVDGTMNAVERGEALDWLKE---DSNT
gi|490111935|ref|WP_004012603.1|helicase[Mobiluncusmulieris]_gi|227843812|gb|EEJ53982.1|helicaseCterminaldomainprotein[Mobilun/      ---N--------------ADPSDNL---AVQCRHVDGTMNAVQRGEALDWLKA---DPGE
gi|490114228|ref|WP_004014848.1|helicase[Mobiluncusmulieris]_gi|269934432|gb|EEZ90993.1|helicaseCterminaldomainprotein[Mobilun/      ---N--------------ADPSDNL---AVQCRHVDGTMNAVQRGEALDWLKA---DPGE
gi|493333551|ref|WP_006290636.1|helicase[Parascardoviadenticolens]_gi|315119487|gb|EFT82620.1|helicaseCterminaldomainprotein[P/      -------------------------LKLNCELQHVDGSMNSSERSEKLNWLAG---GKD-
gi|495817067|ref|WP_008541646.1|helicase,partial[Sutterellaparvirubra]_gi|378598583|gb|EHY31712.1|helicaseprotein,partial[Sutte      -SYS--------------HAAFDAEYPLVCDCRHIDGSMNATEKGALLDWLRA---EPE-
gi|491572696|ref|WP_005430278.1|helicase[Sutterellawadsworthensis]_gi|319805116|gb|EFW01943.1|DNAhelicaserestrictionenzymeTypeI      -NLS--------------REAYDLAYPLKCETRHIDGSMNATEKDALLNWLRE---EPE-
gi|748686321|ref|WP_039944445.1|hypotheticalprotein[Thermicanusaegyptius]/11573                                                      ---------------------EKTH---HFKVRHVDGTMTATERKEHLDWLRG---EPD-
gi|916875698|ref|WP_051482754.1|hypotheticalprotein[Thioalkalivibriosp.HK1]/11366                                                    ---P--------------ERKEEFL---RCQTSHVDGQHNALDRKKRIEALKN---VPQ-
gi|821248604|gb|KKZ10960.1|hypotheticalproteinTE42_09075[CandidatusSynechococcusspongiarumSP3]/11598                                 ------------------GGGEAVP---SVSARHVDGTFNATARAEALAFLEE---TPP-
gi|737409843|ref|WP_035390956.1|hypotheticalprotein[Ferrimicrobiumacidiphilum]_gi|765544266|gb|KJE76241.1|typeIrestrictionenzym      ---I--------------GTSRNLP---GVEIKHIDGTMGALERNGDLNWLRE---ST--
gi|654906925|ref|WP_028358281.1|damageinducibleprotein[Brackiellaoedipodis]/11679                                                    ---Y--------------VD-PNPVLDMVCEAEHVDGSMNATKKEEKINWLKG---ENGQ
gi|502809901|ref|WP_013044877.1|hypotheticalprotein[CandidatusPuniceispirillummarinum]_gi|292663146|gb|ADE38247.1|hypotheticalp      ---------------------HDGF---TCQVDHVDGTNTALERKNKLDWLRE---PAGA
gi|251771370|gb|EES51950.1|typeIIIrestrictionenzyme,ressubunit[Leptospirillumferrodiazotrophum]/11628                                ------------------------L---TCHFEHVDGGMNIDRRLERIRWLDA---AQE-
gi|498328480|ref|WP_010642636.1|DNAdamageinducibleprotein[Acidithiobacillusthiooxidans]/11627                                        ---P--------------ETPHTFL---QAKTRHVDGDMNMGERLTLLDWLKE---EPE-
gi|647633864|ref|WP_025906698.1|damageinducibleprotein[Prochlorococcussp.scB245a_519B7]/11622                                        ---L--------------NKNKCDL---QVEVHHVDGTFNAEEKNDELAWLKE---ETN-
gi|647619599|ref|WP_025893729.1|damageinducibleprotein[Prochlorococcussp.scB241_527N11]/11621                                        ---F--------------ANNNTNL---EVELFHVDGTFNADQRNEKLNWLKD---ETE-
gi|755034887|ref|WP_042387498.1|hypotheticalprotein[Clostridiumsp.CL6]/11606                                                         ------------------KEAESLV---NIEIQHVDGSFNALEKKEKINWLKE---DSE-
gi|498139798|ref|WP_010453954.1|helicase[SuccinivibrionaceaebacteriumWG1]/11662                                                      ---D--------------EQKERFISLTPDTVHHVDGSMNTATRAGLIAELKD---DLK-
gi|527501386|gb|EPT34571.1|typeIIIrestrictionenzyme,ressubunit[Bacteroidetesbacteriumoraltaxon272str.F0290]/11789                    ---E--------------SERNQVV---NVEARHIDGGMSAEIRAEAIDWLKD---ESI-
gi|496047275|ref|WP_008771782.1|helicase[Bacteroidessp.2_1_33B]_gi|262296443|gb|EEY84373.1|helicaseCterminaldomainprotein[Bact/      ---E--------------EVREEVV---DIEVQHIDGAMNAATREEKIAWLKE---ETG-
PDI8503III/11664                                                                                                                      ---E--------------EVREEVV---DIEVQHIDGAMNAATQEEKIAWLKE---ETG-
gi|738719431|ref|WP_036613715.1|helicase[Parabacteroidesdistasonis]_gi|649542518|gb|KDS49387.1|restrictionendonucleasefamilypro      ---E--------------EAREEVV---DIEVQHIDGGMNAATREEKIAWLKE---ETG-
gi|659417033|gb|KEJ86133.1|hypotheticalproteinHMPREF1002_00341[Porphyromonassp.31_2]/11656                                           ---E--------------EVREEVV---DIEVQHIDGAMNAATREEKIAWLKE---ETG-
gi|567456807|gb|ETK11358.1|hypotheticalproteinT235_15845[Tannerellasp.oraltaxonBU063isolateCell8/11]/11433                           ---E--------------AEKSEVV---HVLTRHVDGSMGAAERNDALAWLAA---DPD-
gi|545440279|ref|WP_021677877.1|helicase[Porphyromonasgingivalis]_gi|543985765|gb|ERJ89108.1|helicaseprotein[Porphyromonasgingi      ---E--------------DERTGVV---HITARHVDGGMGATERNAALAWLAE---DAT-
gi|497438056|ref|WP_009752254.1|helicase[Prevotellasp.oraltaxon473]_gi|429144866|gb|EKX87968.1|helicaseprotein[Prevotellasp.ora      ---D--------------QERESVV---HIKARHVDGSMGAAQRNDALAWLAE---DTT-
gi|490423474|ref|WP_004295683.1|MULTISPECIES:helicase[Bacteroides]_gi|156110098|gb|EDO11843.1|helicaseCterminaldomainprotein[B/      ---T--------------EEREHVV---EVQARHIDGSMNSQARNEQLAWLAD---ENI-
gi|941777229|ref|WP_055173506.1|helicase[Bacteroidescaccae]_gi|933263395|emb|CUQ00567.1|Predictedhelicase[Bacteroidescaccae]/1/      ---S--------------ENLSHTV---SVTTRHIDGSMNSQERNGILQWLAE---ESD-
gi|492413898|ref|WP_005836892.1|helicase[Bacteroidesuniformis]_gi|392678824|gb|EIY72229.1|hypotheticalproteinHMPREF1072_03583[B      ---S--------------ESLLHTV---SVTTRHIDGSMNSQERNGILQWLAE---ESD-
gi|695455095|ref|WP_032566191.1|helicase[Bacteroidesfragilis]_gi|596095426|gb|EXZ59996.1|restrictionendonucleasefamilyprotein[B      ---S--------------ESLSHTV---SVTTRHIDGSMNSQERNGILQWLAE---ESD-
gi|933227941|emb|CUQ02348.1|Predictedhelicase[Bacteroidesuniformis]/11658                                                           ---S--------------ESLSRTV---SVTTRHIDGSMNSQERNGILQWLAE---ESD-
gi|494823013|ref|WP_007558421.1|helicase[Bacteroidesplebeius]_gi|198272167|gb|EDY96436.1|helicaseCterminaldomainprotein[Bacter/      ---S--------------ESLSHTV---SVTTRHIDGSMNSQKRNGILQWLAE---ESD-
gi|495427212|ref|WP_008151909.1|helicase[Parabacteroidesjohnsonii]_gi|218222381|gb|EEC95031.1|helicaseCterminaldomainprotein[P/      ---E--------------ESLSHTV---SITAKHIDGSMNSQERNGILQWLAD---EPD-
gi|933196855|emb|CUO94545.1|typeIrestrictionenzymeEcoKIsubunitR[Bacteroidesuniformis]_gi|933220098|emb|CUN86874.1|typeIrestrict      ---A--------------ESLSHTV---SITAKHIDGSMNSQERNGILQWLSD---EPD-
gi|823687748|ref|WP_047101121.1|helicase[Brachyspirahyodysenteriae]_gi|823334537|gb|KLI17932.1|helicase[Brachyspirahyodysenteri      ---D--------------EVKKEIV---NITSQHIDGTMNATVRSDLLSWLKR---DEEN
gi|496436691|ref|WP_009145538.1|helicase[Phascolarctobacteriumsuccinatutens]_gi|322414007|gb|EFY04839.1|helicaseCterminaldomai/      ---E--------------DAQEDTV---IATAKHIDGSMNANERNELVSWLRN---APT-
gi|503372880|ref|WP_013607541.1|helicase[Sphaerochaetaglobosa]_gi|324026933|gb|ADY13692.1|typeIIIrestrictionproteinressubunit[S      ---E--------------EERREIV---SIEADHVDGTMGATTRDAKLSKLKK---VSR-
gi|505821748|ref|WP_015707145.1|helicase[Treponemaprimitia]_gi|299483503|gb|ADJ19584.1|helicasedomaincontainingprotein[Trepone/      ---E--------------EQRKEIV---TVESKHVDGTMGAQTRERKLQWLKS---ADT-
gi|505822741|ref|WP_015708134.1|helicase[Treponemaprimitia]_gi|333739639|gb|AEF85129.1|endonucleaseandmethylaseLlaGI[Treponemap      ---E--------------EQRKEIV---TVESDHVDGTMGAQTRERKLQWLKS---ADT-
gi|488791994|ref|WP_002704400.1|helicase[Treponemasaccharophilum]_gi|380767691|gb|EIC01689.1|typeIIIrestrictionproteinressubuni      ---E--------------DDRADLV---VVEADHVDGSMGSSVREEKLDWLKK---ADS-
gi|524671454|emb|CDD81750.1|putativeseptumsitedeterminingproteinMinC[Bacteroidessp.CAG:462]/11642                                    ---A--------------EDKAQMV---DVVAEHVDGTMSATERDQKLMWLKG---QTE-
gi|524573865|emb|CDC95766.1|putativeseptumsitedeterminingproteinMinC[Alistipessp.CAG:268]/11639                                      ---E--------------EDRATMV---DVVAHHVDGSMSATKRDEELMWLKE---QPE-
gi|658617970|ref|WP_029759328.1|helicase[Fusobacteriumnucleatum]/11606                                                               ---E--------------EAKKKMV---DISSKHIDGGMNALEREDLLSWLKE---DVE-
gi|494412037|ref|WP_007216782.1|helicase[Bacteroidescellulosilyticus]_gi|392638921|gb|EIY32752.1|hypotheticalproteinHMPREF1062_      ---E--------------EERSEIV---SVEADHVDGTMNASIRDRKLSWLKS---TPA-
gi|383107102|gb|AFG37435.1|putativehelicase[SpirochaetaafricanaDSM8902]/11616                                                        ---E--------------EKKEAMV---SPRARHVDGTMSAPERDELLSWLKG---SED-
SAF8902III/11616                                                                                                                      ---E--------------EKKEAMV---SPRARHVDGTMSAPERDELLSWLKG---SED-
gi|652538894|ref|WP_026932796.1|helicase[Gramellaechinicola]/11633                                                                   ---L--------------TKRENLV---TVSSKHIDGSMSAPERDDLLGWLKS---TSA-
gi|639238223|ref|WP_024568839.1|helicase[Elizabethkingiaanophelis]/11628                                                             ---A--------------EAREKMV---NVSSKHIDGSMNAPTREELLEWLKS---SDT-
gi|652520381|ref|WP_026914582.1|helicase[Gramellaportivictoriae]/11623                                                               ---P--------------KHREKLV---NVSSNHIDGSMNAPQRDELLGWLKS---QSA-
gi|494922183|ref|WP_007648219.1|Helicase[Formosasp.AK20]_gi|469825489|gb|EMQ95550.1|Helicasedomainprotein[Formosasp.AK20]/11626      ---P--------------SQREKLV---NVSSNHIDGSMNAPQRDELLGWLKS---QSA-
```

```
gi|522083382|ref|WP_020594591.1|hypotheticalprotein[Spirosomapanaciterrae]/11637      ---D--------------EKKHQMQ---SVASRHIDGTMDATQRNELLAWLKA---EPE-
gi|490455352|ref|WP_004326157.1|helicase[Bacteroidesovatus]_gi|392669277|gb|EIY62768.1|hypotheticalproteinHMPREF1070_03635[Bact      ---E--------------EQKDKIV---AVASDHIDGTMAATIRQEKLSWLKS---VPE-
gi|499516035|ref|WP_011202675.1|helicase[Bacteroidesfragilis]_gi|53713270|ref|YP_099262.1|helicasedomainprotein[Bacteroidesfrag      ---E--------------EQKDKIV---AVASDHIDGTMAATIRQEKLSWLKS---VPE-
gi|651338332|ref|WP_026461320.1|helicase[Adhaeribacteraquaticus]/11620      ---E--------------EKKEQMV---SVASKHIDGTMTAPQRDELLGWLKA---ESE-
gi|806964642|gb|AKC17139.1|helicase[Flavobacteriumpsychrophilum]/11639      ---Q--------------EKKSKMV---SMASEHIDGTMSAPRRDELLSWLKE---ETP-
gi|752684374|ref|WP_041332322.1|helicase[Flavobacteriumpsychrophilum]_gi|666882889|gb|AIG30715.1|helicase[Flavobacteriumpsychro      ---Q--------------EKKSKMV---SMASEHIDGTMSAPRRDELLSWLKE---ETP-
gi|933196595|emb|CUP04894.1|DNAorRNAhelicasesofsuperfamilyII[Bacteroidesuniformis]/11631      ---E--------------EERRKMV---SVSSKHIDGTMSAPQRDELLGWLKA---NPE-
gi|670509109|ref|WP_031456918.1|helicase[Flavobacteriumchungangense]/11625      ---K--------------EKRDTMV---SIASKHIDGSMSAPERDELLGWIKS---DIE-
                                            * :*      :   :  :

gi|490190857|ref|WP_004089451.1|hypotheticalprotein[Helicobacterbilis]_gi|476630638|gb|EMZ37196.1|hypotheticalproteinC826_02193      -------------------KESICELITNAKCLSEGVDIPALDSVVFFDGRSAMVDIIQA
gi|488896133|ref|WP_002807242.1|MULTISPECIES:restrictionendonucleaseEco57I[Campylobacter]_gi|380541096|gb|EIA65377.1|hypothetic      -------------------KENTCNILSNARCLSEGVDVPALDSVVFFDGRNSMVDIIQS
gi|746602240|ref|WP_039628365.1|restrictionendonucleaseEco57I[Campylobacterlari]_gi|744819395|gb|AJD03391.1|putativehelicase[Ca      -------------------KENTCNILSNARCLSEGVDVPALDSVVFFDGRNSMVDIIQS
gi|727317135|ref|WP_033772558.1|restrictionendonucleaseEco57I[Helicobacterpylori]/11622      -------------------EQNTCKVLSNARCLSEGVDVPALDSIVFFDGKSAMVDIIQA
gi|727103490|ref|WP_033618954.1|restrictionendonucleaseEco57I[Helicobacterpylori]/11616      -------------------EQNTCKVLSNARCLSEGVDVPALDSIVFFDGKSAMVDIIQA
gi|727340205|ref|WP_033794408.1|restrictionendonucleaseEco57I[Helicobacterpylori]/11609      -------------------EQNTCKVLSNARCLSEGVDVPALDSIVFFDGKSAMVDIIQA
gi|727292407|ref|WP_033749975.1|restrictionendonucleaseEco57I[Helicobacterpylori]/11611      -------------------EQNTCKVLSNARCLSEGVDVPALDSIVFFDGKSAMVDIIQA
gi|727289795|ref|WP_033747591.1|restrictionendonucleaseEco57I[Helicobacterpylori]/11612      -------------------EPNTCKVLSNARCLSEGVDVPALDSVIFFDGKSAMVDIIQA
gi|727319676|ref|WP_033775047.1|restrictionendonucleaseEco57I[Helicobacterpylori]/11612      -------------------KPNTCKVLSNARCLSEGVDVPALDSVIFFDGKSAMVDIIQA
gi|447212910|ref|WP_001290166.1|restrictionendonucleaseEco57I[Helicobacterpylori]_gi|308063540|gb|ADO05427.1|hypotheticalprotei      -------------------KPNTCKVLSNARCLSEGVDVPALDSIVFFDGKSAMVDIIQA
gi|447212901|ref|WP_001290157.1|restrictionendonucleaseEco57I[Helicobacterpylori]_gi|384557329|gb|AFH97797.1|hypotheticalprotei      -------------------KPNTCKVLSNARCLSEGVDVPALDSIVFFDGKSAMVDIIQA
gi|515849671|ref|WP_017280424.1|hypotheticalprotein[Helicobacterpylori]/11615      -------------------KPNTCKVLSNARCLSEGVDVPALDSIVFFDGKSAMVDIIQA
gi|447212900|ref|WP_001290156.1|restrictionendonucleaseEco57I[Helicobacterpylori]_gi|317178970|dbj|BAJ56758.1|TypeIIGrestrictio      -------------------KPDTCKVLSNARCLSEGVDVPALDSIVFFDGKSAMVDIIQA
gi|447079018|ref|WP_001156274.1|hypotheticalprotein[Helicobacterpylori]/11614      -------------------QPNTCKVLSNARCLSEGVDVPALDTIVFFDGKSAMVDIIQA
gi|447212800|ref|WP_001290056.1|hypotheticalprotein[Helicobacterpylori]_gi|532153248|gb|EQL67316.1|restrictionendonucleaseEco57      -------------------QPNTCKVLSNARCLSEGVDVPALDSIVFFDGKSAMVDIIQA
gi|505457237|ref|WP_015644339.1|hypotheticalprotein[Helicobacterpylori]_gi|499061160|gb|AGL70074.1|restrictionendonucleaseEco57      -------------------QPNTCKVLSNARCLSEGVDVPALDSIVFFDGKSAMVDIIQA
HPYUM037X/11591      -------------------QPNTCKVLSNARCLSEGVDVPALDSIVFFDGKSAMVDIIQA
gi|920026297|ref|WP_052937176.1|restrictionendonuclease[Helicobacterpylori]_gi|912528678|gb|KNX48238.1|restrictionendonucleaseE      -------------------QPNTCKVLSNARCLSEGVDVPALDSIVFFDGKSAMVDIIQA
gi|915539050|ref|WP_050841327.1|restrictionendonuclease[Helicobacterpylori]_gi|908890887|gb|KNE06183.1|restrictionendonucleaseE      -------------------QPNTCKVLSNARCLSEGVDVPALDSIVFFDGKSAMVDIIQA
gi|727101397|ref|WP_033616957.1|restrictionendonucleaseEco57I[Helicobacterpylori]/11592      -------------------EPNTCKVLSNARCLSEGVDVPALDSIVFFDGKSAMVDIIQA
gi|562814875|emb|CDK13002.1|TypeISPRMsystem[Helicobacterpylori26695]/11588      -------------------EPNTCKVLSNARCLSEGVDVPALDSIVFFDGKSAMVDIIQA
HPYAXVIII/11588      -------------------EPNTCKVLSNARCLSEGVDVPALDSIVFFDGKSAMVDIIQA
gi|447079029|ref|WP_001156285.1|restrictionendonucleaseEco57I[Helicobacterpylori]_gi|393085956|gb|EJB86635.1|hypotheticalprotei      -------------------QPNTCKVLSNARCLSEGVDVPALDSIVFFDGKSAMVDIIQA
gi|489105953|ref|WP_003015814.1|restrictionendonuclease[Helicobacterpylori]_gi|477503388|gb|ENH59950.1|restrictionendonucleasef      -------------------EPNTCKVLSNARCLSEGVDVPALDSVVFFDGKSAMVDIIQA
gi|447079006|ref|WP_001156262.1|restrictionendonucleaseEco57I[Helicobacterpylori]_gi|393067470|gb|EJB68280.1|hypotheticalprotei      -------------------QPNTCKVLSNARCLSEGVDVPALDSIVFFDGKSAMVDIIQA
gi|727314589|ref|WP_033770298.1|restrictionendonucleaseEco57I[Helicobacterpylori]/11586      -------------------EPNTCKILSNARCLSEGVDVPALDSIVFFDGKSAMVDIIQA
gi|447079019|ref|WP_001156275.1|restrictionendonucleaseEco57I[Helicobacterpylori]_gi|308065028|gb|ADO06920.1|hypotheticalprotei      -------------------QPNTCKVLSNARCLSEGVDVPALDSVIFFDGKSAMVDIIQA
gi|447079028|ref|WP_001156284.1|restrictionendonucleaseEco57I[Helicobacterpylori]_gi|393076030|gb|EJB76784.1|hypotheticalprotei      -------------------QPNTCKVLSNARCLSEGVDVPALDSVIFFDGRSAMVDIIQA
gi|393034009|gb|EJB35070.1|hypotheticalproteinHPNQ4053_0631[HelicobacterpyloriNQ4053]/11514      -------------------KPNTCKVLSNARCLSEGVDVPALDSIIFFDGKSAMVDIIQA
gi|447079004|ref|WP_001156260.1|restrictionendonucleaseEco57I[Helicobacterpylori]_gi|384552418|gb|AFI07366.1|hypotheticalprotei      -------------------QPNTCKVLSNARCLSEGVDVPALDSVIFFDGKSAMVDIIQA
gi|746011306|ref|WP_039088215.1|restrictionendonucleaseEco57I[Helicobacterpylori]_gi|733488720|gb|KHL85979.1|restrictionendonuc      -------------------EPNTCKVLSNARCLSEGVDVPALDSVIFFDGKSAMVDIIQA
gi|531081162|gb|EQD92166.1|restrictionendonucleaseEco57I[HelicobacterpyloriPZ5080]/11509      -------------------KPNTCKVLSNARCLSEGVDVPALDSIVFFDGKSAMVDIIQA
gi|544760796|ref|WP_021187395.1|restrictionendonucleaseEco57I[Helicobacterpylori]_gi|531086508|gb|EQD96745.1|restrictionendonuc      -------------------EPNTCKVLSNARCLSEGVDVPALDSVIFFDGKSAMVDIIQA
gi|447079012|ref|WP_001156268.1|restrictionendonucleaseEco57I[Helicobacterpylori]_gi|393028199|gb|EJB29286.1|hypotheticalprotei      -------------------QPNTCKVLSNARCLSEGVDVPALDSVIFFDGKSAMVDIIQA
gi|746008458|ref|WP_039085555.1|restrictionendonucleaseEco57I[Helicobacterpylori]_gi|733480358|gb|KHL78026.1|restrictionendonuc      -------------------EPNTCKVLSNARCLSEGVDVPALDSVIFFDGKSAMVDIIQA
gi|447079011|ref|WP_001156267.1|restrictionendonucleaseEco57I[Helicobacterpylori]_gi|393038534|gb|EJB39568.1|hypotheticalprotei      -------------------QPNTCKVLSNARCLSEGVDVPALDSVIFFDGKSAMVDIIQA
gi|393037163|gb|EJB38199.1|hypotheticalproteinHPNQ4161_0422[HelicobacterpyloriNQ4161]/11556      -------------------EPNTCKVLSNARCLSEGVDVPALDSVIFFDGKSAMVDIIQA
gi|447079007|ref|WP_001156263.1|restrictionendonucleaseEco57I[Helicobacterpylori]_gi|393051817|gb|EJB52768.1|hypotheticalprotei      -------------------EPNTCKVLSNARCLSEGVDVPALDSIVFFDGKSAMVDIIQA
gi|487867094|ref|WP_001940560.1|helicase[Helicobacterpylori]_gi|459505657|gb|EMG98418.1|helicaseprotein[HelicobacterpyloriGAM21      -------------------KPNTCKVLSNARCLSEGVDVPALDSVIFFDGRSAMVDIIQA
gi|487872094|ref|WP_001945560.1|helicase[Helicobacterpylori]_gi|459512104|gb|EMH04523.1|helicaseprotein[HelicobacterpyloriGAM24      -------------------EPNICKVLSNARCLSEGVDVPALDSVIFFDGRSAMVDIIQA
gi|487880228|ref|WP_001953694.1|helicase[Helicobacterpylori]_gi|459529131|gb|EMH20734.1|helicaseprotein[HelicobacterpyloriGAM26      -------------------EPNTCKVLSNARCLSEGVDVPALDSVIFFDGKSAMVDIIQA
gi|487882593|ref|WP_001956059.1|helicase[Helicobacterpylori]_gi|459533462|gb|EMH24859.1|helicaseprotein[HelicobacterpyloriGAM26      -------------------KPNTCKVLSNARCLSEGVDVPALDSVIFFDGRSAMVDIIQA
gi|487845430|ref|WP_001918896.1|helicase[Helicobacterpylori]_gi|410713871|gb|EKQ71363.1|helicaseprotein[HelicobacterpyloriGAM10      -------------------EPNTCKVLSNARCLSEGVDVPALDSVIFFDGRSAMVDIIQA
gi|447079005|ref|WP_001156261.1|restrictionendonucleaseEco57I[Helicobacterpylori]_gi|393069830|gb|EJB70625.1|hypotheticalprotei      -------------------EPNTCKVLSNARCLSEGVDVPALDSVIFFDGRSAMVDIIQA
gi|459506808|gb|EMG99528.1|helicaseprotein[HelicobacterpyloriGAM121Aii]/11545      -------------------EPNTCKVLSNARCLSEGVDVPALDSVIFFDGKSAMVDIIQA
gi|487825283|ref|WP_001898749.1|restrictionendonucleaseEco57I[Helicobacterpylori]_gi|393107526|gb|EJC08068.1|hypotheticalprotei      -------------------EPNICKVLSNARCLSEGVDVPALDSVIFFDGKSAMVDIIQA
gi|447079008|ref|WP_001156264.1|restrictionendonucleaseEco57I[Helicobacterpylori]_gi|393058636|gb|EJB59524.1|hypotheticalprotei      -------------------EPNICKVLSNARCLSEGVDVPALDSVIFFDGKSAMVDIIQA
gi|447079015|ref|WP_001156271.1|restrictionendonucleaseEco57I[Helicobacterpylori]_gi|307637357|gb|ADN79807.1|hypotheticalprotei      -------------------EPNICKVLSNARCLSEGVDVPALDSVIFFDGRSAMVDIIQA
gi|487884861|ref|WP_001958327.1|helicase[Helicobacterpylori]_gi|459535535|gb|EMH26834.1|helicaseprotein[HelicobacterpyloriGAM26      -------------------EPNTCKVLSNARCLSEGVDVPALDSVIFFDGRSAMVDIIQA
gi|487886365|ref|WP_001959831.1|helicase[Helicobacterpylori]_gi|459538727|gb|EMH29861.1|helicaseprotein[HelicobacterpyloriGAM27      -------------------EPNTCKVLSNARCLSEGVDVPALDSVIFFDGKSAMVDIIQA
gi|545301637|ref|WP_021581412.1|helicase[Helicobacterpylori]_gi|535931889|gb|ERA61745.1|helicaseprotein[HelicobacterpyloriGAM11      -------------------EPNTCKVLSNARCLSEGVDVPALDSVIFFDGRSAMVDIIQA
gi|447079010|ref|WP_001156266.1|restrictionendonucleaseEco57I[Helicobacterpylori]_gi|393095477|gb|EJB96081.1|hypotheticalprotei      -------------------EPNICKVLSNARCLSEGVDVPALDSVIFFDGRSAMVDIIQA
gi|447079009|ref|WP_001156265.1|restrictionendonucleaseEco57I[Helicobacterpylori]_gi|393056294|gb|EJB57206.1|hypotheticalprotei      -------------------EPNICKVLSNARCLSEGVDVPALDSVIFFDGKSAMVDIIQA
gi|471386985|gb|EMR58906.1|restrictionendonucleasefamilyprotein[HelicobacterpyloriHpH1]/11504      -------------------EPNICKVLSNARCLSEGVDVPALDSVIFFDGRSAMVDIIQA
gi|487875679|ref|WP_001949145.1|helicase[Helicobacterpylori]_gi|459516928|gb|EMH09091.1|helicaseprotein[HelicobacterpyloriGAM25      -------------------EPNICKVLSNARCLSEGVDVPALDSVIFFDGRSAMVDIIQA
gi|496130066|ref|WP_008854573.1|DNAdamageinducibleprotein[Commensalibacterintestini]_gi|353671689|gb|EHD13391.1|helicasedomain/      -----------------TP-ENTCRILSNVRCLSEGVDVPALDAVLFLTPRSSQVDVVQS
gi|924898894|ref|WP_053556546.1|damageinducibleprotein[Sphingopyxissp.113P3]_gi|924523996|gb|ALC14732.1|damageinducibleprotei/1      -----------------TGRDDECRVLTNARCLSEGVDVPALDAVMFLHPRKSQIDVVQA
```

```
gi|780802950|gb|KJS08562.1|damageinducibleprotein[Hoefleasp.BRH_c9]/11633                                      -----------------TD-GDTCRILSNARCLSEGVDVPALDAIMFLHPRKSQIDVVQS
gi|944671432|gb|KQN22886.1|damageinducibleprotein[Sphingomonassp.Leaf32]/11415                                 -----------------HE-DHSCRILSNARCLSEGVDVPALDAIMFLHPRKSQIDVVQS
gi|944612953|gb|KQM64772.1|damageinducibleprotein[Sphingomonassp.Leaf16]/11726                                 -----------------HE-DHSCRILSNARCLSEGVDVPALDAIMFLHPRKSQIDVVQS
gi|764353851|ref|WP_044331468.1|damageinducibleprotein[Sphingomonashengshuiensis]_gi|761897338|gb|AJP71707.1|damageinduciblep/1   -----------------HG-DHACRILTNARCLSEGVDVPALDAILFMHPRKSQIDVVQS
gi|945874472|gb|KQZ27973.1|damageinducibleprotein[Mesorhizobiumsp.Root552]/11651                               -----------------HG-NHACRILTNARCLSEGVDVPALDAILFMHPRKSQIDVVQS
gi|759408081|ref|WP_043132403.1|damageinducibleprotein[Paracoccussanguinis]_gi|694217086|gb|KGJ14281.1|damageinducibleprotein/1   -----------------AG-EDTCRILTNAKVLSEGVDVPALDAIMFMHPRKSQIDVVQS
gi|654342671|ref|WP_027836116.1|damageinducibleprotein[Maritaleamyrionectae]/11632                             -----------------TD-DDTCRILTNAKVLSEGVDVPALDAIMFMHPRKSQIDVVQS
gi|126717687|gb|EBA14409.1|hypotheticalproteinRSK20926_02032[Roseobactersp.SK20926]/11603                       -----------------AG-DDTCRILTNAKVLSEGVDVPALDAIMFMHPRKSQIDVVQS
gi|765301116|ref|WP_044628780.1|damageinducibleprotein[Halomonasmeridiana]_gi|764089679|gb|KJD19875.1|damageinducibleprotein[/1   -----------------GE-DDTCRILTNAKVLSEGVDVPALDAIMFMHPRKSQIDVVQS
gi|518053006|ref|WP_019223214.1|DNAdamageinducibleprotein[Bartonellarattaustraliani]/11654                    -----------------AG-KNVCRILSNVRCLSEGIDVPALDAIMFLHPRNSQVDVIQA
gi|493637806|ref|WP_006589578.1|DNAdamageinducibleprotein[Bartonellabirtlesii]_gi|395410345|gb|EJF76900.1|hypotheticalproteinM/   -----------------AE-NNVCRILSNVRCLSEGIDVPALDAIMFLHPRNSQVDVIQA
gi|501188603|ref|WP_012231622.1|DNAdamageinducibleprotein[Bartonellatribocorum]_gi|161017843|emb|CAK01401.1|helicase/methyltra/   -----------------TG-QNVCRILSNVRCLSEGIDVPALDAIMFLHPRNSQVDVIQA
gi|651388616|ref|WP_026500615.1|damageinducibleprotein[Bartonellagrahamii]/11655                               -----------------AG-NNVCRILSNVRCLSEGIDVPALDAIMFLHPRNSQVDVIQA
gi|501188669|ref|WP_012231687.1|DNAdamageinducibleprotein[Bartonellatribocorum]_gi|161017922|emb|CAK01480.1|helicase/methyltra/   -----------------TG-QNVCRILSNVRCLSEGIDVPALDAIMFLHPRNSQVDVIQA
gi|518048832|ref|WP_019219040.1|DNAdamageinducibleprotein[Bartonellaflorencae]/11655                          -----------------TD-ENNCRVLTNVRCLSEGVDVPALDAVLFLHPRKSHVDVIQA
gi|518053617|ref|WP_019223825.1|DNAdamageinducibleprotein[Bartonellarattaustraliani]/11607                    -----------------TD-ENNCRILTNVRCLSEGVDVPALDAILFLHPRKSHVDVIQA
gi|506336449|ref|WP_015856168.1|DNAdamageinducibleprotein[Bartonellagrahamii]_gi|240267492|gb|ACS51080.1|helicase/methyltransf/   -----------------TE-ENNCRVLTNVRCLSEGVDVPALDAVLFLHPRKSHVDVIQA
gi|494588832|ref|WP_007347427.1|DNAdamageinducibleprotein[Bartonellarattimassiliensis]_gi|395419053|gb|EJF85361.1|hypothetical/   -----------------TD-ENNCRVLTNVRCLSEGVDVPALDAVMFLHPRKSHVDVIQA
gi|501188650|ref|WP_012231668.1|DNAdamageinducibleprotein[Bartonellatribocorum]_gi|161017902|emb|CAK01460.1|helicase/methyltra/   -----------------TD-ENNCRVLTNVRCLSEGVDVPALDAVMFLHPRKSHVDVIQA
gi|501188624|ref|WP_012231643.1|DNAdamageinducibleprotein[Bartonellatribocorum]_gi|161017866|emb|CAK01424.1|helicase/methyltra/   -----------------MG-ENHCRVLTNVRCLSEGVDVPSLDAVMFLHPRKSQVDVIQA
gi|518053856|ref|WP_019224064.1|DNAdamageinducibleprotein[Bartonellarattaustraliani]/11625                    -----------------VG-ENHCRVLTNVRCLSEGVDVPTLDAVMFLHPRKSQVDVIQA
gi|501188053|ref|WP_012231081.1|DNAdamageinducibleprotein[Bartonellatribocorum]_gi|161017434|emb|CAK00992.1|helicase/methyltra/   -----------------VG-ENHCRVLTNVRCLSEGVDVPTLDAVMFLHPRKSQVDVIQA
gi|651388630|ref|WP_026500626.1|damageinducibleprotein[Bartonellagrahamii]/11657                               -----------------AG-ENTCRVLTNVRCLSEGVDVPALDAVLFLHPRKSQIEIIQA
gi|518048855|ref|WP_019219063.1|DNAdamageinducibleprotein[Bartonellaflorencae]/11658                          -----------------AG-ENTCRVLTNVRCLSEGVDVPALDAVLFLHPRKSQIEIIQA
gi|490995101|ref|WP_004856829.1|DNAdamageinducibleprotein[Bartonelladoshiae]_gi|395413167|gb|EJF79645.1|hypotheticalproteinMCS/   -----------------AG-ENTCRVLTNVRCLSEGVDVPALDAVLFLHPRKSQIEIIQA
gi|491002558|ref|WP_004864279.1|DNAdamageinducibleprotein[Bartonellavinsonii]_gi|395421392|gb|EJF87643.1|hypotheticalproteinME/   -----------------AG-KNICRVLSNVRCLSEGVDVPALDAVMFLHPRKSHVDVIQA
gi|491003373|ref|WP_004865094.1|DNAdamageinducibleprotein[Bartonellavinsonii]_gi|395432864|gb|EJF98838.1|hypotheticalproteinME/   -----------------AG-ENTCRVLTNVRCLSEGVDVPALDAVMFLHPRKSHVDVIQA
gi|491002426|ref|WP_004864148.1|DNAdamageinducibleprotein[Bartonellavinsonii]_gi|395421767|gb|EJF88003.1|hypotheticalproteinME/   -----------------AG-ENTCRVLSNVRCLSEGVDVPALDAVMFLHPRKSHVDVIQA
gi|491004024|ref|WP_004865745.1|DNAdamageinducibleprotein[Bartonellavinsonii]_gi|395432542|gb|EJF98522.1|hypotheticalproteinME/   -----------------AE-ENTCRVLSNVRCLSEGVDVPALDAVMFLHPRKSHVDVIQA
gi|648396442|ref|WP_026088193.1|damageinducibleprotein[Bartonellarattaustraliani]/11593                        -----------------AG-ENTCRVLSNVRCLSEGIDVPALDAVMFLHPRKSQVDVIQA
gi|748190174|ref|WP_039761290.1|damageinducibleprotein[Bartonellaqueenslandensis]/11658                        -----------------AG-ENTCRVLSNVRCLSEGVDVPALDAVMFLHPRKSHVDVIQA
gi|494817599|ref|WP_007553007.1|DNAdamageinducibleprotein[Bartonellasp.DB56]_gi|395411312|gb|EJF77837.1|hypotheticalproteinMC/1   -----------------AG-ENICRILTNVRCLSEGVDVPSLDAVMFLHPRKSQVDVIQA
gi|515763376|ref|WP_017195976.1|DNAdamageinducibleprotein[Bartonellabirtlesii]/11656                          -----------------AG-ENVCRILTNVRCLSEGVDVPSLDAVMFLHPRKSQVDVIQA
gi|494589420|ref|WP_007348014.1|DNAdamageinducibleprotein[Bartonellarattimassiliensis]_gi|395416278|gb|EJF82667.1|hypothetical/   -----------------AG-ENHCRVLTNVRCLSEGVDVPALDAIMFLHPRKSYVDVIQA
gi|506336469|ref|WP_015856188.1|DNAdamageinducibleprotein[Bartonellagrahamii]_gi|240267512|gb|ACS51100.1|helicase/methyltransf/   -----------------AG-GNSCRVLTNVRCLSEGVDVPALDAIMFLHPRKSYVDVIQA
gi|749308087|ref|WP_040132227.1|damageinducibleprotein[Bartonellatribocorum]_gi|605051878|emb|CDO49988.1|helicase/methyltransf/   -----------------AG-ENTCRVLTNVRCLSEGVDVPALDAIMFLHPRKSHVDIIQA
gi|515763800|ref|WP_017196400.1|DNAdamageinducibleprotein[Bartonellabirtlesii]/11658                          -----------------AG-EHVCRVLTNVRCLSEGVDVPALDAIMFLHPRKSQVDVIQA
gi|492186994|ref|WP_005773380.1|DNAdamageinducibleprotein[Bartonellaelizabethae]_gi|395418361|gb|EJF84688.1|hypotheticalprotei/   -----------------AG-EHACRVLTNVRCLSEGVDVPALDAIMFLHPRKSQVDVIQA
gi|501188615|ref|WP_012231634.1|DNAdamageinducibleprotein[Bartonellatribocorum]_gi|161017856|emb|CAK01414.1|helicase/methyltra/   -----------------AG-ENTCRVLTNVRCLSEGVDVPALDAIMFLHPRKSQVDVIQA
gi|501187470|ref|WP_012230499.1|DNAdamageinducibleprotein[Bartonellatribocorum]_gi|161017090|emb|CAK00648.1|helicase/methyltra/   -----------------AG-EHVCRVLTNVRCLSEGVDVPALDAIMFLHPRKSQVDVIQA
gi|501188636|ref|WP_012231655.1|DNAdamageinducibleprotein[Bartonellatribocorum]_gi|161017879|emb|CAK01437.1|helicase/methyltra/   -----------------AG-ENTCRVLTNVRCLSEGVDVPALDAIMFLHPRKSQVDVIQA
gi|930151941|emb|CTQ34818.1|UvsWhelicase[Jannaschiarubra]/11641                                                -----------------PG-EDNCRILTNARCLSEGVDVPALDGIMFLHPRKSQIDVVQS
gi|928977106|ref|WP_054001461.1|damageinducibleprotein[Shimiasp.SK013]_gi|927698852|gb|KPA22698.1|typeIrestrictionenzymeEcoKIs/   -----------------VA-NDSCKILSNARCLSEGVDVPALDAIMFLHPRKSQIDVVQS
gi|738872465|ref|WP_036760884.1|damageinducibleprotein[Leisingeradaeponensis]/11643                           -----------------SE-ENTCRILTNARCLSEGVDVPALDAIMFLHPRKSQIDVVQS
gi|518050881|ref|WP_019221089.1|DNAdamageinducibleprotein[Bartonellasenegalensis]/11652                        -----------------SG-DDVCRILTNARCLSEGVDVPALDAIMFLNPRKSQIDVVQS
gi|493980596|ref|WP_006923523.1|DNAdamageinducibleprotein[Bartonellawashoensis]_gi|395413322|gb|EJF79796.1|hypotheticalprotein/   -----------------SG-EDVCRILTNARCLSEGVDVPALDAIMFLNARKSQIDVVQS
gi|493983106|ref|WP_006925984.1|DNAdamageinducibleprotein[Bartonellawashoensis]_gi|395418127|gb|EJF84455.1|hypotheticalprotein/   -----------------SG-EDVCRILTNARCLSEGVDVPALDAIMFLNARKSQIDVVQS
gi|499494671|ref|WP_011181311.1|DNAdamageinducibleprotein[Bartonellahenselae]_gi|49239008|emb|CAF28308.1|hypotheticalproteinBH/   -----------------SG-DGVCRILTNARCLSEGVDVPALDAIMFLNARKSQIDVVQS
gi|651388426|ref|WP_026500441.1|damageinducibleprotein[Bartonellagrahamii]/11652                               -----------------SG-EDVCRILTNARCLSEGVDVPALDAIMFLNPRKSQIDVVQS
gi|506337280|ref|WP_015856999.1|DNAdamageinducibleprotein[Bartonellagrahamii]_gi|240268402|gb|ACS51990.1|helicase/methyltransf/   -----------------SG-EDVCRILTNARCLSEGVDVPALDAIMFLNPRKSQIDVVQA
gi|494817535|ref|WP_007552943.1|DNAdamageinducibleprotein[Bartonellasp.DB56]_gi|395412002|gb|EJF78518.1|hypotheticalproteinMC/1   -----------------SG-DDVCRILTNARCLSEGVDVPALDAIMFLNPRKSQIDVVQS
gi|493638099|ref|WP_006589864.1|DNAdamageinducibleprotein[Bartonellabirtlesii]_gi|395409809|gb|EJF76394.1|hypotheticalproteinM/   -----------------GE-DDVCRILTNARCLSEGVDVPALDAIMFLNPRKSQIDVVQS
gi|748189589|ref|WP_039760707.1|damageinducibleprotein[Bartonellaqueenslandensis]/11651                        -----------------SG-DGVCRILTNARCLSEGVDVPALDAIMFLNPRKSQIDVVQA
gi|501189483|ref|WP_012232501.1|DNAdamageinducibleprotein[Bartonellatribocorum]_gi|161018902|emb|CAK02460.1|helicase/methyltra/   -----------------SG-GDVCRILTNARCLSEGVDVPALDAIMFLNPRKSQIDVVQA
gi|518049181|ref|WP_019219389.1|DNAdamageinducibleprotein[Bartonellaflorencae]/11652                          -----------------CD-DDVCRVLTNARCLSEGVDVPALDAIMFLNPRKSQIDVVQA
gi|492193255|ref|WP_005774528.1|DNAdamageinducibleprotein[Bartonellaelizabethae]_gi|395416403|gb|EJF82779.1|hypotheticalprotei/   -----------------SG-DEVCRILTNARCLSEGVDVPALDAIMFLNPRKSQIDVVQA
gi|498387325|ref|WP_010701288.1|helicase/methyltransferase[Bartonellabovis]_gi|478418724|gb|ENN92426.1|helicase/methyltransfera   -----------------AG-DNVCRVLTNARCLSEGVDVPALDAIIFLHPRKSLIDVVQA
gi|319408697|emb|CBI82352.1|conservedhypotheticalprotein[BartonellaschoenbuchensisR1]/11661                    -----------------AG-DNVCRVLTNARCLSEGVDVPALDAIIFLHPRKSVIDVVQS
gi|494741726|ref|WP_007477134.1|DNAdamageinducibleprotein[Bartonellamelophagi]_gi|395423925|gb|EJF90113.1|hypotheticalproteinM/   -----------------AE-DNVCRVLTNARCLSEGVDVPALDAIIFLHPRKSLIDVVQS
gi|737261405|ref|WP_035245349.1|damageinducibleprotein[Alcanivoraxjadensis]_gi|686989709|gb|KGD62556.1|hypotheticalproteinT9A_/   -----------------PE-PNECRILTNARCLSEGVDVPALDSIMFLHPRKSQIDVIQS
gi|916687564|ref|WP_051294655.1|damageinducibleprotein[Gemmobacternectariphilus]/11641                         -----------------SG-DHICRILTNARCLSEGVDVPALDAIMFLHPRKSQIDVVQS
gi|740294127|ref|WP_038132495.1|damageinducibleprotein[Thioclavasp.DT234]_gi|660524826|gb|KEO54689.1|hypotheticalproteinDT23_/1   -----------------AE-GNTARILTNARCLSEGVDVPSLDAIMFLHPRKSLIDVVQS
gi|568331062|ref|WP_024099615.1|helicase[Phaeobactergallaeciensis]_gi|568232121|gb|AHD12007.1|putativehelicase[Phaeobactergalla   -----------------AS-DHTCRILTNARCLSEGVDVPALDAIMFLHPRKSQIDVVQS
gi|518810459|ref|WP_019966413.1|DNAdamageinducibleprotein[Pannonibacterphragmitetus]/11635                     -----------------AG-DNVCRILTNARCLSEGVDVPALDAIMFLHPRKSQIDVVQS
gi|495305760|ref|WP_008030510.1|DNAdamageinducibleprotein[Rhodobactersp.SW2]_gi|259021698|gb|EEW25000.1|typeIIIrestrictionprot/   -----------------AG-DNVCRILTNARCLSEGVDVPALDAIMFLHPRKSQIDVVQS
gi|506403303|ref|WP_015923022.1|DNAdamageinducibleprotein[Rhodobactersphaeroides]_gi|221162951|gb|ACM03922.1|HypotheticalProte/   -----------------AG-DNVCRILTNARCLSEGVDVPALDAIMFLHPRKSQIDVVQS
gi|738818386|ref|WP_036708790.1|damageinducibleprotein[Paracoccussanguinis]_gi|694226786|gb|KGJ22541.1|damageinducibleprotein/1   -----------------AG-DNVCRILTNARCLSEGVDVPALDAIMFLHPRKSQIDVVQS
```

```
gi|746096057|ref|WP_039162453.1|damageinducibleprotein[Gallibacteriumanatis]_gi|703617775|gb|KGQ63868.1|damageinducibleprotei/1      ----------------ETE-ENTCRILSNVRCLSEGVDVPALDAVLFLTARNSQVDVVQS
gi|737716785|ref|WP_035685631.1|damageinducibleprotein[Avibacteriumparagallinarum]/11643      ----------------ETE-ENTCRILSNVRCLSEGVDVPALDAVLFLTARNSQVDVVQS
gi|540072553|gb|ERF78672.1|hypotheticalproteinN561_04930[Gallibacteriumanatis12656/12}/11651      ----------------DSG-EKTCRILSNVRCLSEGVDVPALDAVLFLTARNSQVDVVQS
gi|917509519|ref|WP_052115936.1|damageinducibleprotein[Gallibacteriumanatis]_gi|702414161|gb|KGQ42233.1|damageinducibleprotei/1      ----------------DPG-EKTCRILSNVRCLSEGVDVPALDAVLFLTARNSQVDVVQS
gi|746082942|ref|WP_039150268.1|damageinducibleprotein[Gallibacteriumanatis]_gi|703617078|gb|KGQ63194.1|damageinducibleprotei/1      ----------------DAG-ENTCRILSNVRCLSEGVDVPALDAVLFLTARNSQVDVVQS
gi|822522885|ref|WP_046952840.1|damageinducibleprotein[Haemophilushaemolyticus]_gi|821501762|gb|KKZ59267.1|damageinduciblepro/1      ----------------EQE-DNTCRILSNVRCLSEGVDVPALDAVLFLTPRSSQVDVVQS
gi|404381014|gb|EJZ77501.1|putativehelicase[Pasteurellamultocidasubsp.gallicidaP1059]/11681      ----------------ENE-ANTCRILSNVRCLSEGVDVPALDAVLFLTPRSSQVDVVQS
gi|298281660|gb|EFI23149.1|helicaseCterminaldomainprotein[Neisseriasp.oraltaxon014str.F0314]/11493      ----------------DIP-DNTCRILSNVRCLSEGVDVPALDAVLFLTPRNSKVDVVQS
gi|348013055|gb|EGY51980.1|helicasedomainprotein[Neisseriashayeganii871]/11481      ----------------GLP-ENTCRILSNVRCLSEGVDVPSLDAVLFLTPRNSQVDVVQS
gi|496407140|ref|WP_009116004.1|DNAdamageinducibleprotein[Neisseriawadsworthii]_gi|349794756|gb|EGZ48566.1|helicasedomainprote/      ----------------ELP-DNTCRILSNVRCLSEGVDVPALDAVLFLTPRNSQVDVVQS
gi|517216441|ref|WP_018405259.1|DNAdamageinducibleprotein[Marinobacterlipolyticus]/11656      ----------------TTE-DNTCRILSNVRCLSEGVDVPALDAVLFLTPRSSQVDVVQS
gi|738230264|ref|WP_036185691.1|damageinducibleprotein[Marinobactersp.ELB17]/11671      ----------------TTE-DNTCRILSNVRCLSEGVDVPALDAVLFLTPRSSQVDVVQS
gi|447197741|ref|WP_001274997.1|DNAdamageinducibleprotein[Acinetobacterbaumannii]_gi|408512324|gb|EKK13969.1|typeIIIrestrictio/      ----------------ETP-DNTCRILSNVRCLSEGVDVPSLDAVLFLTPRNSQVDVVQS
gi|820775897|ref|WP_046739909.1|damageinducibleprotein[Acinetobactersp.AG1]_gi|819909758|gb|KKW75938.1|damageinducibleprotein/1      ----------------QSP-ENTCRILSNVRCLSEGVDVPSLDAVLFLTPRNSQVDVVQS
gi|584595163|ref|WP_024160846.1|helicase[Acinetobacterpittii]_gi|576702530|emb|CDI28049.1|helicasedomaincontainingprotein[Acin/      ----------------QAP-ENTCRILSNVRCLSEGVDVPSLDAVLFLTPRNSQVDVVQS
gi|514963042|ref|WP_016651366.1|hypotheticalprotein[Acinetobactersp.NIPH2036]_gi|514162665|gb|EPG41391.1|hypotheticalproteinF90      ----------------TAP-ENTCRILSNVRCLSEGVDVPSLDAVLFLTPRNSQVDVVQS
gi|515965564|ref|WP_017396147.1|DNAdamageinducibleprotein[Acinetobacterhaemolyticus]/11667      ----------------QAP-ENTCRILSNVRCLSEGVDVPSLDAVLFLTPRNSQVDVVQS
gi|491346810|ref|WP_005204747.1|MULTISPECIES:hypotheticalprotein[Acinetobacter]_gi|480335823|gb|ENX56033.1|hypotheticalproteinF      ----------------QAP-ENTCRILSNVRCLSEGVDVPSLDAVLFLTPRNSQVDVVQS
gi|693044472|ref|WP_032215268.1|damageinducibleprotein[Escherichiacoli]_gi|607725731|gb|EZE08230.1|damageinducibleprotein[Esc/1      ----------------DTD-GNVCRILSNVRCLSEGVDVPSLDAVLFLTPRGSQVDVVQS
gi|742382177|ref|WP_038861304.1|damageinducibleprotein[Cronobactermuytjensii]_gi|924333747|gb|ALB69766.1|damageinducibleprote/1      ----------------ETE-DNVCRILSNVRCLSEGVDVPSLDAVLFLTPRSSQVDVVQS
gi|915912804|ref|WP_050927035.1|damageinducibleprotein[Yersiniaenterocolitica]_gi|899805802|emb|CQH32042.1|typeIrestrictionenz/      ----------------EAD-ENVCRILSNVRCLSEGVDVPSLDAVLFLTPRSSQVDVVQS
gi|921111327|gb|KON62781.1|typeIrestrictionenzymeEcoKIsubunitR[Komagataeibactereuropaeus]/11492      -----------------TP-EGICRVLSNVRCLSEGVDVPALDAVLFLTPRNSQVDVVQS
gi|582024278|gb|AHI27615.1|DNAhelicaserestrictionenzymeTypeIIIRsubunit[KomagataeibacterxylinusE25]/11636      -----------------TP-EGICRVLSNVRCLSEGVDVPALDAVLFLTPRNSQVDVVQS
gi|347662673|dbj|BAK86128.1|DNAhelicaserestrictionenzymeTypeIIIRsubunit[KomagataeibactermedellinensisNBRC3288]/11737      -----------------TP-EGICRVLSNVRCLSEGVDVPALDAVLFLTPRNSQVDVVQS
gi|917445053|ref|WP_052051471.1|damageinducibleprotein[Acetobactertropicalis]/11429      -----------------TR-DKVCRVLSNVRCLSEGVDVPALDAVLFLTPRNSQVDVVQS
gi|453329445|dbj|GAC88472.1|DNAhelicaserestrictionenzymetypeIIIRsubunit[GluconobacterthailandicusNBRC3255]_gi|530287981|dbj|GAD      -----------------NK-GKICRVLSNVRCLSEGVDVPALDAVLFLTPRNSQVDVVQS
gi|889770922|ref|WP_048844278.1|damageinducibleprotein[Gluconobacterfrateurii]/11419      -----------------TK-EKTCRVLSNVRCLSEGVDVPALDAVLFLTPRNSQVDVVQS
gi|504886208|ref|WP_015073310.1|hypotheticalprotein[Gluconobacteroxydans]_gi|411027954|gb|AFW01209.1|hypotheticalproteinB932_16      -----------------TK-EKACRVLSNVRCLSEGVDVPALDAVLFLTPRNSQVDVVQS
gi|480040211|gb|ENV79751.1|hypotheticalproteinF942_01641[AcinetobacterursingiiANC3649]/11432      -----------------PE-ENQCKILSNVRCLSEGVDVPSLDAVLFLTPRNSQVDVVQS
gi|822498222|ref|WP_046936504.1|damageinducibleprotein[Xanthomonasgardneri]_gi|821751224|gb|KLB07223.1|damageinducibleprotein/1      -----------------MP-ADTCRVLSNVRCLSEGVDVPALDAVLFLTPRNSQVDVVQS
gi|654059559|ref|WP_027705556.1|damageinducibleprotein[Zymobacterpalmae]/11635      -----------------PA-KNTCRILSNVRCLSEGVDVPALDAVLFLTPRNSQVDVVQS
gi|643535620|ref|WP_025224315.1|damageinducibleprotein[Methylophilaceaebacterium11]_gi|577704400|gb|EUJ10546.1|putativehelicas/      -----------------TP-DDTCRILSNVRCLSEGVDVPALDAVLFLTPRNSQVDVVQS
MBA11I/11635      -----------------TP-DDTCRILSNVRCLSEGVDVPALDAVLFLTPRNSQVDVVQS
gi|655355857|ref|WP_028762287.1|damageinducibleprotein[Shewanellacolwelliana]/11632      -----------------TE-DNVCRILSNVRCLSEGVDVPALDAVLFLTPRNSQVDVVQS
gi|753204236|ref|WP_041510579.1|damageinducibleprotein[Shewanellasp.cp20]_gi|752457765|gb|KIO36326.1|damageinducibleprotein[S/1      -----------------PE-ENNCKILSNVRCLSEGVDVPALDAVLFLTPRNSQVDVVQS
KPNNIH30III/11619      -----------------PE-EGSCKILSNVRCLSEGVDVPALDAVLFLTPRNSQVDVVQS
gi|749295994|ref|WP_040120352.1|damageinducibleprotein[Klebsiellapneumoniae]_gi|723249300|gb|AIX81926.1|damageinducibleprotei/1      -----------------PE-EGSCKILSNVRCLSEGVDVPALDAVLFLTPRNSQVDVVQS
gi|641639739|emb|CDQ54010.1|unnamedproteinproduct[Klebsiellapneumoniae]/11641      -----------------PE-EGSCKILSNVRCLSEGVDVPALDAVLFLTPRNSQVDVVQS
gi|490797922|ref|WP_004660060.1|hypotheticalprotein[Acinetobactersp.NIPH236]_gi|479876779|gb|ENU27973.1|hypotheticalproteinF992      Q----------HDDD-GAE-RPVCRVLSNVRCLSEGVDVPALDAVLFLTPRNSQVDVVQS
gi|916750926|ref|WP_051357982.1|damageinducibleprotein[Acinetobactertjernbergiae]/11453      Q----------QDED-GTE-RPICRVLSNVRCLSEGVDVPALDAVLFLTPRNSQVDVVQS
gi|691098662|ref|WP_032025060.1|damageinducibleprotein[Acinetobacterbaumannii]_gi|593567521|gb|EXR50735.1|restrictionendonucle/      Q----------FDEN-GVE-RPICRVLSNVRCLSEGVDVPALDSVLFLTPRNSQVDVVQS
gi|736669405|ref|WP_034676049.1|damageinducibleprotein[Acinetobacterbaumannii]/11643      Q----------YDED-GKE-RPICRVLSNVRCLSEGVDVPALDAVLFLTPRNSQVDVVQS
gi|491368928|ref|WP_005226826.1|hypotheticalprotein[Acinetobactersp.NIPH1847]_gi|480173259|gb|ENX09797.1|hypotheticalproteinF89      K----------IDEN-GVE-RPICRVLSNVRCLSEGVDVPALDAVLFLTPRSSQVDVVQS
gi|796853993|ref|WP_045795953.1|damageinducibleprotein[Acinetobacterindicus]_gi|787929761|gb|KJV44219.1|damageinducibleprotei/1      Q----------VDEN-GVE-RPICRVLSNVRCLSEGVDVPALDAVLFLTPRSSQVDVVQS
gi|295797463|gb|ADG32253.1|typeIIIrestrictionproteinressubunit[ThiomonasintermediaK12]/11676      ------------------P-DHTCRILSNVRCLSEGVDVPALDAVLFLTPRNSQVDVVQS
gi|499871179|ref|WP_011551913.1|DNAdamageinducibleprotein[Myxococcusxanthus]_gi|108467544|gb|ABF92729.1|putativerestriction/mo/      ------------------P-EDTCRILSNVRCLSEGVDVPALDAVLFLTPRKSQVDVVQS
gi|495313852|ref|WP_008038600.1|DNAdamageinducibleprotein[Bartonellatamiae]_gi|395424181|gb|EJF90368.1|hypotheticalproteinME5_/      ------------------P-EDTCRILSNVRCLSEGVDVPALDAVLFLTARNSQVDVVQS
gi|501453335|ref|WP_012476784.1|DNAdamageinducibleprotein[Leptospirabiflexa]_gi|167777824|gb|ABZ96123.1|Helicase[Leptospirabif/      ------------------E-ENVCRILSNVRCLSEGVDVPALDAVLFLTPRNSQVDVVQS
gi|738104613|ref|WP_036063193.1|damageinducibleprotein[Leptospiranoguchii]/11652      ------------------Q-ENSCRILSNVRCLSEGVDVPALDAVLFLSPRQSQVDVVQS
gi|410001039|gb|EKO51661.1|typeIIIrestrictionenzyme,ressubunit[Leptospirakirschneristr.200802841]/11651      ------------------Q-EYSCRILSNVRCLSEGVDVPALDAVLFLSPRQSQVDVVQS
gi|490901851|ref|WP_004763774.1|typeIIIrestrictionenzyme,ressubunit[Leptospirakirschneri]_gi|464209249|gb|EMN24014.1|typeIIIres      ------------------Q-EYSCRILSNVRCLSEGVDVPALDAVLFLSPRQSQVDVVQS
gi|464398131|gb|EMO65902.1|typeIIIrestrictionenzyme,ressubunit[Leptospirakirschneristr.200803703]/11648      ------------------Q-EYSCRILSNVRCLSEGVDVPALDAVLFLSPRQSQVDVVQS
gi|945029024|gb|KQQ84900.1|damageinducibleprotein[Xanthomonassp.Leaf131]/11423      -----------------AP-VNTCRVLSNVRCLSEGVDVPALDAVLFLTPRNSQVDVVQS
gi|896511314|ref|WP_049418183.1|damageinducibleprotein[Stenotrophomonasmaltophilia]/11639      -----------------TP-EDTCRILSNVRCLSEGVDVPALDAVLFLTPRNSQVDVVQS
gi|896513082|ref|WP_049419616.1|damageinducibleprotein[Stenotrophomonasmaltophilia]/11638      -----------------TP-PDTCRVLSNVRCLSEGVDVPALDAVLFLTPRNSQVDVVQS
gi|896580616|ref|WP_049469425.1|damageinducibleprotein[Stenotrophomonasmaltophilia]/11641      -----------------TP-PDTCRVLSNVRCLSEGVDVPALDAVLFLTPRNSQVDVVQS
gi|648289350|ref|WP_026070499.1|damageinducibleprotein[Stenotrophomonasmaltophilia]/11639      -----------------TP-PDTCRVLSNVRCLSEGVDVPALDAVLFLTPRNSQVDVVQS
gi|656272786|ref|WP_029218442.1|damageinducibleprotein[Xanthomonascassavae]/11639      -----------------TP-PDTCRVLSNVRCLSEGVDVPALDAVLFLTPRNSQVDVVQS
gi|406976236|gb|EKD98747.1|hypotheticalproteinACD_23C00290G0002[unculturedbacterium]/11679      -----------------PP-ENTCRVLSNVRCLSEGVDVPALDAVLFLTPRNSQVDVVQS
gi|828145737|ref|WP_047238120.1|damageinducibleprotein[Chromobacteriumsubtsugae]/11645      -----------------VP-EDTCRILSNVRCLSEGVDVPALDAVLFLTPRSSQVDVVQS
gi|654311174|ref|WP_027824327.1|damageinducibleprotein[Laribacterhongkongensis]/11641      -----------------VP-DDTCRILSNVRCLSEGVDVPALDAVLFLTPRSSQVDVVQS
gi|757242173|gb|KIR15516.1|typeIrestrictionenzymeEcoKIsubunitR[Pseudomonasfluorescens]/11778      -----------------VP-DNTCRILSNVRCLSEGVDVPALDAVLFLTPRNSQVDVVQS
gi|653605161|ref|WP_027616682.1|damageinducibleprotein[Pseudomonassp.URHB0015]/11639      -----------------VP-DNTCRILSNVRCLSEGVDVPALDAVLFLTPRNSQVDVVQS
gi|520868025|ref|WP_020308060.1|helicase[Pseudomonassyringae]_gi|519159034|gb|EPM49935.1|helicasedomaincontainingprotein[Pseud/      -----------------VP-DNTCRILSNVRCLSEGVDVPALDAVLFLTPRNSQVDVVQS
gi|940156567|gb|KPX71668.1|Helicasedomaincontainingprotein[Pseudomonasamygdalipv.lachrymans]/11677      -----------------VP-DNTCRILSNVRCLSEGVDVPALDAVLFLTPRNSQVDVVQS
gi|505460508|ref|WP_015647610.1|helicase[Pseudomonassp.GLE121]_gi|499110212|ref|YP_007974374.1|helicasedomaincontainingprotein/      -----------------IP-EHTCRILSNVRCLTEGVDVPALDAVLFLTPRNSLVDVVQS
gi|940235551|gb|KPY45311.1|hypotheticalproteinALO48_200154[Pseudomonassyringaepv.rhaphiolepidis]/11631      -----------------AP-EHTCRILSNVRCLSEGVDVPALDAVLFLTPRNSQVDVVQS
```

```
gi|520880808|ref|WP_020312235.1|helicase[Pseudomonassyringae]_gi|519172158|gb|EPM62027.1|helicasedomaincontainingprotein[Pseud/      -----------------AP-ENTCRILSNVRCLSEGVDVPALDAVLFLTPRNSQVDVVQS
gi|829046479|ref|WP_047282259.1|damageinducibleprotein[Pseudomonasfluorescens]/11630      -----------------AP-VNTCRILSNVRCLSEGVDVPALDAVLFLTPRNSQVDVVQS
gi|499415493|ref|WP_011102960.1|DNAdamageinducibleprotein[Pseudomonassyringaegroupgenomosp.3]_gi|28867279|ref|NP_789898.1|heli/      -----------------PP-ANTCRILSNVRCLSEGVDVPALDAVLFLTPRNSQVDVVQS
gi|730238299|ref|WP_033942897.1|damageinducibleprotein[Pseudomonasaeruginosa]/11629      -----------------PP-ANTCRILSNVRCLSEGVDVPALDAVLFLTPRNSQVDVVQS
gi|495310125|ref|WP_008034873.1|DNAdamageinducibleprotein[Pseudomonassp.GM60]_gi|398186685|gb|EJM74052.1|putativehelicase[Pseu/      -----------------SP-ANTCRILSNVRCLSEGVDVPALDAVLFLTPRNSQVDVVQS
gi|779992653|ref|WP_045428690.1|damageinducibleprotein[Pseudomonassp.MT1]_gi|699967872|dbj|BAP80334.1|DNAhelicaserestrictione/1      -----------------SP-ANTCRILSNVRCLSEGVDVPALDAVLFLTPRNSQVDVVQS
gi|786037289|ref|WP_045733274.1|damageinducibleprotein[Pseudomonaspseudoalcaligenes]_gi|782987904|gb|KJU79152.1|damageinducib/1      -----------------PP-ANTCRILSNVRCLSEGVDVPALDAVLFLTPRNSQVDVVQS
gi|749666366|ref|WP_040265445.1|damageinducibleprotein[Pseudomonasrhodesiae]/11632      -----------------PP-TNTCRILSNVRCLSEGVDVPALDAVLFLTPRNSQVDVVQS
gi|734902433|ref|WP_034133698.1|damageinducibleprotein[Pseudomonasfluorescens]/11635      -----------------PP-TNTCRILSNVRCLSEGVDVPALDAVLFLTPRNSQVDVVQS
gi|490551031|ref|WP_004416146.1|typeIIrestrictionmodificationsystemrestriction/helicasesubunit[Pseudomonassyringae]_gi|4432823/      -----------------PP-ANTCRILSNVRCLSEGVDVPALDAVLFLTPRNSQVDVVQS
gi|489199911|ref|WP_003109152.1|DNAdamageinducibleprotein[Pseudomonasaeruginosa]_gi|552648708|gb|ERY63184.1|hypotheticalprotei/      -----------------PL-ANTCRILSNVRCLSEGVDVPALDAVLFLTPRNSQVDVVQS
gi|730641237|ref|WP_034061328.1|damageinducibleprotein[Pseudomonasaeruginosa]/11636      -----------------PP-ANTCRILSNVRCLSEGVDVPALDAVLFLTPRNSQVDVVQS
gi|751304980|ref|WP_041013386.1|damageinducibleprotein[Pseudomonasxanthomarina]_gi|703407611|emb|CEG51014.1|Helicasedomaincon/1      -----------------PP-ANTCRILSNVRCLSEGVDVPALDAVLFLTPRNSQVDVVQS
gi|939335207|ref|WP_054881520.1|damageinducibleprotein[Pseudomonassp.NBRC111128]/11632      -----------------PP-ANTCRILSNVRCLSEGVDVPALDAVLFLTPRNSQVDVVQS
gi|757693555|ref|WP_042923792.1|damageinducibleprotein[Pseudomonaschloritidismutans]_gi|780910610|gb|KJS78302.1|damageinducib/1      -----------------AP-ANTCRILSNVRCLSEGVDVPALDAVLFLTPRNSQVDVVQS
gi|665953944|ref|WP_031319989.1|damageinducibleprotein[Pseudomonastolaasii]/11626      -----------------AP-ANTCRILSNVRCLSEGVDVPALDAVLFLTPRNSQVDVVQS
gi|771600573|ref|WP_045213746.1|damageinducibleprotein[Pseudomonassp.21]_gi|770081123|gb|KJJ98634.1|damageinducibleprotein[Ps/1      -----------------PP-ANTCRILSNVRCLSEGVDVPALDAVLFLTPRNSQVDVVQS
gi|740871396|ref|WP_038656644.1|damageinducibleprotein[Pseudomonasstutzeri]_gi|627772214|gb|AHY41437.1|damageinducibleprotein/1      -----------------PP-ANTCRILSNVRCLSEGVDVPALDAVLFLTPRNSQVDVVQS
gi|946242205|gb|KRC93782.1|damageinducibleprotein[Pseudomonassp.Root9]/11634      -----------------PP-ANTCRILSNVRCLSEGVDVPALDAVLFLTPRNSQVDVVQS
gi|553777322|ref|WP_023109107.1|MULTISPECIES:hypotheticalprotein[Pseudomonas]_gi|552638674|gb|ERY53184.1|hypotheticalproteinQ05      -----------------PP-ANTCRILSNVRCLSEGVDVPALDAVLFLTPRNSQVDVVQS
gi|896383370|ref|WP_049324618.1|damageinducibleprotein[Pseudomonasstutzeri]/11627      -----------------PP-VNTCRILSNVRCLSEGVDVPALDAVLFLTPRNSQVDVVQS
gi|696158510|gb|AIT41787.1|putativehelicase[Pseudomonassp.LM8]/11630      -----------------PP-ANTCRILSNVRCLSEGVDVPALDAVLFLTPRNSQVDVVQS
gi|504634892|ref|WP_014821994.1|DNAdamageinducibleprotein[Pseudomonasstutzeri]_gi|390985249|gb|AFM35242.1|helicasedomainconta/1      -----------------PP-ANTCRILSNVRCLSEGVDVPALDAVLFLTPRNSQVDVVQS
gi|544783974|ref|WP_021206729.1|DNAdamageinducibleprotein[Pseudomonasstutzeri]_gi|533224779|gb|EQM79688.1|DNAdamageinduciblep/1      -----------------PP-ANTCRILSNVRCLSEGVDVPALDAVLFLTPRNSQVDVVQS
gi|504567385|ref|WP_014754487.1|MULTISPECIES:DNAdamageinducibleprotein[Pseudomonas]_gi|388559493|gb|AFK68634.1|helicasedomain/1      -----------------PP-ANTCRILSNVRCLSEGVDVPALDAVLFLTPRNSQVDVVQS
gi|654441469|ref|WP_027912052.1|damageinducibleprotein[Pseudomonassp.URIL14HWK12:I7]/11630      -----------------PP-ANTCRILSNVRCLSEGVDVPALDAVLFLTPRNSQVDVVQS
gi|941470066|ref|WP_055127894.1|damageinducibleprotein[Pseudomonasmediterranea]/11631      -----------------PP-ANTCRILSNVRCLSEGVDVPALDAVLFLTPRNSQVDVVQS
gi|557815599|ref|WP_023446184.1|DNAdamageinducibleprotein[Pseudomonaschloritidismutans]_gi|557364081|gb|ESQ98190.1|DNAdamagei/1      -----------------PP-ANTCRILSNVRCLSEGVDVPALDAVLFLTPRNSQVDVVQS
gi|727814791|ref|WP_033898247.1|damageinducibleprotein[Pseudomonassp.FH1]_gi|567648005|gb|ETK23229.1|helicasedomaincontaining/1      -----------------PP-TNTCRILSNVRCLSEGVDVPALDAVLFLTPRNSQVDVVQS
gi|503278702|ref|WP_013513363.1|DNAdamageinducibleprotein[Desulfovibrioaespoeensis]_gi|316942375|gb|ADU61426.1|typeIIIrestrict/      -----------------VP-ENTCRILSNVRCLSEGVDVPALDAVLFLTPRNSQVDVVQS
gi|505228796|ref|WP_015415898.1|typeIIIrestrictionendonucleasesubunitR[Desulfovibriopiezophilus]_gi|451896976|emb|CCH49855.1|Ty      -----------------SS-ENTCRILSNVRCLSEGVDVPALDAVLFLTPRNSQVDVVQS
gi|517732543|ref|WP_018902751.1|hypotheticalprotein[Variovoraxparadoxus]/11675      -----------------SP-DNTCRILSNVRCLSEGVDVPALDAVLFLTPRNSQVDVVQS
gi|550909475|ref|WP_022662570.1|DNAdamageinducibleprotein[Desulfovibriolongus]/11637      -----------------TP-ANTCRILSNVRCLSEGVDVPALDAVLFLTPRNSQVDVVQS
gi|945494542|gb|KQV50024.1|damageinducibleprotein[Massiliasp.Root335]/11647      -----------------AD-DNTCRILSNVRCLSEGVDVPALDAVLFLTPRNSQVDVVQS
gi|334196917|gb|AEG70102.1|helicasedomainprotein[RalstoniasolanacearumPo82]/11663      -----------------TP-ENTCRILSNVRCLSEGVDVPALDAVLFLTPRNSQVDVVQS
gi|656006746|ref|WP_029048170.1|damageinducibleprotein[Cupriavidussp.amp6]/11661      -----------------TP-ENTCRILSNVRCLSEGVDVPALDAVLFLTPRNSQVDVVQS
BPS2196ORF3054P/11659      -----------------TP-ENTCRILSNVRCLSEGVDVPALDAVLFLTPRNSQVDVVQS
gi|740932312|ref|WP_038717332.1|damageinducibleprotein[Burkholderiapseudomallei]_gi|760211882|gb|KIX58314.1|damageinduciblepr/1      -----------------TP-ENTCRILSNVRCLSEGVDVPALDAVLFLTPRNSQVDVVQS
gi|755810813|ref|WP_042583861.1|damageinducibleprotein[Burkholderiasp.MSHR3999]_gi|755174952|gb|KIP19790.1|DEAD/DEAHboxhelicas/      -----------------TP-ENTCRILSNVRCLSEGVDVPALDAVLFLTPRNSQVDVVQS
gi|484586570|gb|AGK48195.1|DEAD/DEAHboxhelicasefamilyprotein[BurkholderiathailandensisMSMB121]/11646      -----------------MP-ENTCRILSNVRCLSEGVDVPALDAVLFLTPRNSQVDVVQS
gi|737582935|ref|WP_035553941.1|damageinducibleprotein[Burkholderiasp.9120]/11658      -----------------TP-ENTCRILSNVRCLSEGVDVPALDAVLFLTPRNSQVDVVQS
gi|740989872|ref|WP_038773225.1|damageinducibleprotein[Burkholderiapseudomallei]_gi|705645291|gb|KGS70691.1|DEAD/DEAHboxhelica/      -----------------TP-ENTCRILSNVRCLSEGVDVPALDAVLFLTPRNSQVDVVQS
gi|654912525|ref|WP_028363424.1|damageinducibleprotein[Burkholderiasp.JPY366]/11659      -----------------TP-ENTCRILSNVRCLSEGVDVPALDAVLFLTPRNSQVDVVQS
gi|740955879|ref|WP_038740512.1|damageinducibleprotein[Burkholderiapseudomallei]_gi|714684932|gb|KGW74438.1|DEAD/DEAHboxhelica/      -----------------TP-ENTCRILSNVRCLSEGVDVPALDAVLFLTPRNSQVDVVQS
gi|919203714|ref|WP_052759215.1|damageinducibleprotein[Xanthomonasaxonopodis]/11388      --------------------GNSCRILSNARCLSEGVDVPALDAVLFLNPRDSVVDVVQS
gi|522152381|ref|WP_020663589.1|hypotheticalprotein[Amycolatopsisbenzoatilytica]/11605      --------------------ANSCRILSNARCLSEGVDVPDLDAVLFLNPRNSVVDVVQS
gi|663196013|ref|WP_030233899.1|damageinducibleprotein[Streptomycessp.NRRLS455]/11641      --------------------PNNARILSNARCLSEGVDVPNLDAVLFLQPRNSVVDVVQS
gi|750429553|ref|WP_040711444.1|damageinducibleprotein[Nocardiopsispotens]/11622      --------------------SGSARILSNARCLSEGVDVPSLDAVLFLHPRNSVVDVVQS
gi|518656139|ref|WP_019819475.1|hypotheticalprotein[Saccharomonosporasaliphila]/11623      --------------------PANARILSNARCLSEGVDVPSLDAVLFLHPRNSVVDVVQS
gi|759722933|ref|WP_043433758.1|damageinducibleprotein[Streptomycespluripotens]_gi|733404978|gb|KHK94361.1|damageinduciblepro/1      --------------------QNNARILSNARCLSEGVDVPSLDAVLFLNPRNSVVDVVQS
gi|663424167|ref|WP_030419030.1|damageinducibleprotein[Streptomycessp.NRRLF5065]/11627      --------------------QHNARILTNARCLSEGVDVPSLDAVLFLNPRNSVVDVVQS
gi|663412917|ref|WP_030408353.1|damageinducibleprotein[Streptomycessp.NRRLF5917]/11630      --------------------SNNARILSNARCLSEGVDVPSLDAVLFLNPRNSVVDVVQS
gi|671521483|ref|WP_031506489.1|damageinducibleprotein[Streptomycesmegasporus]/11630      --------------------QNNARILSNARCLSEGVDVPSLDAVLFLNPRNSVVDVVQS
gi|740053144|ref|WP_037902776.1|damageinducibleprotein[Streptomycessp.TAA204]/11626      --------------------QNQARILSNARCLSEGVDVPSLDAVLFLHPRNSVVDVVQS
gi|663409375|ref|WP_030404974.1|MULTISPECIES:damageinducibleprotein[Streptomyces]/11627      --------------------QNQARILSNARCLSEGVDVPSLDAVLFLHPRNSVVDVVQS
gi|746628608|ref|WP_039654277.1|damageinducibleprotein[Streptomycessp.MUSC125]_gi|745644381|gb|KIE25557.1|damageinducibleprot/1      --------------------QNQARILSNARCLSEGVDVPSLDAVLFLHPRNSVVDVVQS
gi|727322066|ref|WP_033777320.1|damageinducibleprotein[Salinisporapacifica]/11627      --------------------PANARILSNARCLSEGVDVPSLDAVLFLHPRNSVVDVVQS
gi|653653242|ref|WP_027655726.1|damageinducibleprotein[Salinisporapacifica]/11618      --------------------PANARILSNARCLSEGVDVPSLDAVLFLHPRNSVVDVVQS
gi|648632543|ref|WP_026324294.1|damageinducibleprotein[Salinisporapacifica]/11621      --------------------PANARILSNARCLSEGVDVPSLDAVLFLHPRNSVVDVVQS
gi|881018002|ref|WP_048738463.1|damageinducibleprotein[Corynebacteriumfalsenii]/11676      --------------------EDQTRLLTNARCLSEGVDVPALDSIIFFNPRNSMVDVVQS
gi|505465526|ref|WP_015652417.1|helicase[Corynebacteriumcallunae]_gi|459385704|gb|AGG67995.1|helicase[CorynebacteriumcallunaeDS      --------------------SMETKILTNARCLSEGVDVPALDSVIFFNPRNSMVDVVQS
gi|41223062|emb|CAF19007.1|DNAorRNAhelicaseofsuperfamilyII[CorynebacteriumglutamicumATCC13032]/11646      --------------------SMETKILTNARCLSEGVDVPALDSVIFFNPRNSMVDVVQS
CGL13032I/11643      --------------------SMETKILTNARCLSEGVDVPALDSVIFFNPRNSMVDVVQS
gi|751257081|ref|WP_040967675.1|damageinducibleprotein[Corynebacteriumglutamicum]_gi|748811620|gb|AJE67931.1|damageinduciblep/1      --------------------ETETRMLTNARCLSEGVDVPALDAVVFFNPRNSMVDVVQS
gi|749040539|ref|WP_040086524.1|damageinducibleprotein[Corynebacteriumhumireducens]/11663      --------------------ENKTRILTNARCLSEGVDVPALDSVVFFNPRNSMVDVVQS
gi|491662899|ref|WP_005519615.1|helicase[Corynebacteriummatruchotii]_gi|224946756|gb|EEG27965.1|helicaseCterminaldomainprotein/      --------------------TDETRILTNARCLSEGVDVPALDAVVFFHPRNSMVDVVQS
gi|746717312|ref|WP_039676231.1|damageinducibleprotein[Corynebacteriumminutissimum]_gi|734991118|gb|KHO29219.1|damageinducibl/1      --------------------EDETRILTNARCLSEGVDVPALDSVIFFHPRNSMVDVVQS
```

gi|908654892|emb|CQD15529.1|SuperfamilyIIDNA/RNAhelicase(modularprotein)[Corynebacteriumstriatum]/11674 --------------------EDETRILTNARCLSEGVDVPALDSVIFFHPRNSMVDVVQS
gi|895994446|ref|WP_049064418.1|damageinducibleprotein[Corynebacteriumaurimucosum]/11667 --------------------EDETRILTNARCLSEGVDVPALDSVIFFHPRNSMVDVVQS
gi|896123331|ref|WP_049145390.1|damageinducibleprotein[Corynebacteriumaurimucosum]/11669 --------------------EDETRILTNARCLSEGVDVPALDSVIFFHPRNSMVDVVQS
gi|817681178|ref|WP_046645422.1|damageinducibleprotein[Corynebacteriumstriatum]_gi|817551679|gb|KKO79868.1|damageinduciblepro/1 --------------------EDETRILTNARCLSEGVDVPALDSVIFFHPRNSMVDVVQS
gi|896139239|ref|WP_049159146.1|damageinducibleprotein[Corynebacteriumaurimucosum]/11675 --------------------EDETRILTNARCLSEGVDVPALDSVIFFHPRNSMVDVVQS
gi|489943947|ref|WP_003847254.1|helicase[Corynebacteriumammoniagenes]_gi|295968218|gb|EFG81467.1|helicaseCterminaldomainprotei/ --------------------SQESRVLTNARCLSEGVDVPGLDSVIFFNPRNSMVDVVQS
gi|516653464|ref|WP_018022453.1|helicase[Corynebacteriumdoosanense]_gi|697778282|gb|AIT61018.1|damageinducibleprotein[Coryneba/ --------------------ANESRILANARCLSEGVDVPGLDSVIFFNPRNSMVDVVQS
gi|493894179|ref|WP_006840129.1|helicase[Corynebacteriumlipophiloflavum]_gi|227078819|gb|EEI16782.1|helicaseCterminaldomainpro/ --------------------GTETRVLTNARCLSEGVDVPALDSVIFFNPRNSMVDVVQS
gi|740831472|ref|WP_038616755.1|damageinducibleprotein[Corynebacteriumpseudotuberculosis]_gi|666103058|gb|AIG06935.1|putativeh/ --------------------SDESRVLTNARCLSEGVDVPGLDSVIFFNPRNSMVDVVQS
gi|518023322|ref|WP_019193530.1|helicase[Corynebacteriumtimonense]/11655 --------------------ATESRVLTNARCLSEGVDVPGLDSVIFFNPRNSMVDVVQS
gi|552767697|ref|WP_023019559.1|MULTISPECIES:hypotheticalprotein[Corynebacterium]_gi|550739013|gb|ERS38609.1|hypotheticalprotei --------------------PTESRVLTNARCLSEGVDVPGLDSVIFFQPRNSMVDVVQS
gi|652659121|ref|WP_027017812.1|damageinducibleprotein[Corynebacteriumpseudodiphtheriticum]/11644 --------------------PTESRVLTNARCLSEGVDVPGLDSVIFFQPRNSMVDVVQS
gi|749039309|ref|WP_040085390.1|damageinducibleprotein[Corynebacteriumhumireducens]/11657 --------------------DNETRLLTNARCLSEGVDVPALDSVVFFNPRNSMVDVVQS
gi|740835591|ref|WP_038620874.1|damageinducibleprotein[Corynebacteriumulcerans]_gi|698973854|gb|AIU33664.1|HelicaseCterminald/1 --------------------DDETRILTNARCLSEGVDVPGLDAVIFFNPRNSMVDVVQS
gi|805307599|ref|WP_046096452.1|damageinducibleprotein[Corynebacteriumulcerans]_gi|803357323|gb|AKA97638.1|Damageinduciblepro/1 --------------------DDETRILTNARCLSEGVDVPGLDAVIFFNPRNSMVDVVQS
gi|504083555|ref|WP_014317549.1|helicase[Corynebacteriumdiphtheriae]_gi|371584249|gb|AEX47914.1|superfamilyIIDNA/RNAhelicase[Co --------------------EDETRILTNARCLSEGVDVPALDAVVFFNPRNSMVDVVQS
gi|896182193|ref|WP_049193580.1|damageinducibleprotein[Corynebacteriumjeikeium]/11645 --------------------EDETRILTNARCLSEGVDVPALDSVIFFNPRNSMVDVVQS
gi|917636459|ref|WP_052205460.1|damageinducibleprotein[Corynebacteriumriegelii]_gi|914295811|gb|AKV59187.1|damageinduciblepro/1 --------------------EEETRILTNARCLSEGVDVPGLDAVIFFNPRNSMVDVVQS
gi|737013522|ref|WP_035009205.1|damageinducibleprotein[Corynebacteriumjeikeium]/11651 --------------------EDETRILTNARCLSEGVDVPGLDSVIFFNPRNSMVDVVQS
gi|737006291|ref|WP_035002094.1|damageinducibleprotein[Corynebacteriumjeikeium]/11658 --------------------EDQTRLLTNARCLSEGVDVPALDSVIFFNPRNSMVDVVQS
gi|737010378|ref|WP_035006133.1|damageinducibleprotein[Corynebacteriumjeikeium]/11650 --------------------EDQTRVLTNARCLSEGVDVPALDSVIFFNPRNSMVDVVQS
gi|737010473|ref|WP_035006228.1|damageinducibleprotein[Corynebacteriumjeikeium]/11657 --------------------ENETRVLTNARCLSEGVDVPALDSVIFFNPRNSMVDVVQS
gi|737006250|ref|WP_035002053.1|damageinducibleprotein[Corynebacteriumjeikeium]/11658 --------------------ENETRVLTNARCLSEGVDVPALDSVIFFNPRNSMVDVVQS
gi|923018168|ref|WP_053412254.1|damageinducibleprotein[Corynebacteriumlactis]_gi|922845184|gb|ALA67504.1|helicase[Corynebacter/ --------------------EDETRILTNARCLSEGVDVPALDAVIFFNPRNSMVDVVQS
gi|923018885|ref|WP_053412970.1|damageinducibleprotein[Corynebacteriumlactis]_gi|922845845|gb|ALA68165.1|helicase[Corynebacter/ --------------------EDETRILTNARCLSEGVDVPALDAVIFFNPRNSMVDVVQS
gi|512464870|ref|WP_016422869.1|hypotheticalprotein[Corynebacteriumsp.HFH0082]_gi|512047830|gb|EPD47505.1|hypotheticalproteinHM --------------------DQHTRVLTNARCLSEGVDVPALDAVIFFNPRNSMVDVVQS
gi|881012697|ref|WP_048733406.1|damageinducibleprotein[Corynebacteriumurealyticum]/11670 --------------------EQHTRVLTNARCLSEGVDVPALDAVIFFNPRNSMVDVVQS
gi|755608478|ref|WP_042530519.1|damageinducibleprotein[Corynebacteriumsingulare]_gi|754340239|gb|AJI78730.1|putativehelicase[C/ --------------------EHESRMLTNARCLSEGVDVPALDSVIFFNPRNSMVDVVQS
gi|491421796|ref|WP_005279593.1|helicase[Corynebacteriumaccolens]_gi|227077180|gb|EEI15143.1|helicaseCterminaldomainprotein[Co/ --------------------ENEARILTNARCLSEGVDVPALDSVIFFNPRNSMVDVVQS
gi|550752286|gb|ERS51451.1|hypotheticalproteinHMPREF1282_00033[Corynebacteriumsp.KPL1856]_gi|550759961|gb|ERS58964.1|hypothetic --------------------ENESRMLTNARCLSEGVDVPALDAVVFFNPRNSMVDVVQS
gi|497955053|ref|WP_010269209.1|helicase[Corynebacteriumbovis]/11599 --------------------DGECRVLTNARCLSEGVDVPALDAVMFLNPRSSTVDVVQS
gi|860580040|ref|WP_048470569.1|hypotheticalprotein[Mycobacteriumchlorophenolicum]_gi|859098575|gb|KMO76871.1|typeIrestrictione --------------------QGECRILTNARCLSEGVDVPALDAVLFLHPRNSIVDVVQS
gi|262085905|gb|ACY21873.1|typeIIIrestrictionproteinressubunit[GordoniabronchialisDSM43247]/11632 --------------------DDECRILSNARCLSEGVDVPALDAVLFLHPRNSVVDVVQS
gi|495996338|ref|WP_008720917.1|typeIIIrestrictionendonucleasesubunitR[Rhodococcussp.AW25M09]_gi|443412350|emb|CCQ18455.1|TypeI --------------------EGECRILSNARCLSEGVDVPALDAVLFLHPRNSVVDVVQS
gi|739284636|ref|WP_037147410.1|damageinducibleprotein[Rhodococcusfascians]/11641 --------------------EGECRILSNARCLSEGVDVPALDAVLFLHPRNSVVDVVQS
gi|739328195|ref|WP_037190574.1|damageinducibleprotein[Rhodococcusfascians]/11643 --------------------EGECRILSNARCLSEGVDVPALDAVLFLHPRNSVVDVVQS
gi|811698335|emb|CMN21041.1|helicase[Mycobacteriumtuberculosis]/11725 --------------------EDECRILSNARCLSEGVDVPALDAVLFLNPRNSIVDVVQS
gi|801577898|emb|CFK23773.1|helicase[Mycobacteriumtuberculosis]/11675 --------------------EDECRILSNARCLSEGVDVPALDAVLFLNPRNSIVDVVQS
MTU18II/11606 --------------------EDECRILSNARCLSEGVDVPALDAVLFLNPRNSIVDVVQS
gi|887719858|emb|CMT27886.1|helicase[Mycobacteriumtuberculosis]/11667 --------------------EDECRILSNARCLSEGVDVPALDAVLFLNPRNSIVDVVQS
gi|621694743|gb|KAS87156.1|hypotheticalproteinAL70_01801[MycobacteriumtuberculosisTKK_03_0115]/11604 --------------------EDECRILSNARCLSEGVDVPALDAVLFLNPRNSIVDVVQS
gi|888128072|emb|CLQ84422.1|helicase[Mycobacteriumtuberculosis]_gi|889917090|emb|CLY86893.1|helicase[Mycobacteriumtuberculosis] --------------------EDECRILSNARCLSEGVDVPALDAVLFLNPRNSIVDVVQS
gi|884823424|emb|CKL96672.1|helicase[Mycobacteriumtuberculosis]/11466 --------------------EDECRILSNARCLSEGVDVPALDAVLFLNPRNSIVDVVQS
gi|505103239|ref|WP_015290341.1|hypotheticalprotein[Mycobacteriumcanettii]_gi|432162733|emb|CCK60117.1|Conservedproteinofunknow --------------------EDECRILSNARCLSEGVDVPALDAVLFLNPRNSIVDVVQS
gi|505116250|ref|WP_015303352.1|hypotheticalprotein[Mycobacteriumcanettii]_gi|432158726|emb|CCK56026.1|Conservedproteinofunknow --------------------EDECRILSNARCLSEGVDVPALDAVLFLNPRNSIVDVVQS
gi|432154735|emb|CCK51974.1|Conservedproteinofunknownfunction(part1)[MycobacteriumcanettiiCIPT140060008]/11588 --------------------EDECRILSNARCLSEGVDVPALDAVLFLNPRNSIVDVVQS
gi|809008027|ref|WP_046295375.1|hypotheticalprotein[Mycobacteriumsp.UM_Kg17]/11624 --------------------EDECRILTNARCLSEGVDVPALDAVLFLHPRNSVVDVVQS
gi|808802633|ref|WP_046284139.1|hypotheticalprotein[Mycobacteriumsp.UM_NZ2]/11624 --------------------EDECRILTNARCLSEGVDVPALDAVLFLHPRNSVVDVVQS
gi|750125774|ref|WP_040429852.1|damageinducibleprotein[Corynebacteriumnuruki]/11668 --------------------DDECRILTNARCLSEGVDVPALDAVLFLHPRNSVVDVVQS
gi|602237614|gb|EYT60960.1|damageinducibleprotein[Dietziasp.UCDTHP]/11473 --------------------DDECRILTNARCLSEGVDVPALDAVLFLHPRNSVVDVVQS
gi|665897412|ref|WP_031264618.1|damageinducibleprotein[Dietziasp.UCDTHP]_gi|602237599|gb|EYT60945.1|helicase[Dietziasp.UCDTH/11 --------------------DDECRILTNARCLSEGVDVPALDAVLFLHPRNSVVDVVQS
gi|502903674|ref|WP_013138650.1|helicase[Segniliparusrotundus]_gi|296181291|gb|ADG98197.1|typeIIIrestrictionproteinressubunit[S --------------------ENECRILTNARCLSEGVDVPALDAVLFLHPRNSVVDVVQS
gi|780248306|ref|WP_045538930.1|hypotheticalprotein[Gordoniasp.no.9]_gi|778142514|gb|KJR06917.1|hypotheticalproteinUG54_12320[G --------------------DNECRILTNARCLSEGVDVPALDAVLFLHPRNSIVDVVQA
gi|665897368|ref|WP_031264574.1|damageinducibleprotein[Dietziasp.UCDTHP]_gi|602239137|gb|EYT62483.1|helicase[Dietziasp.UCDTH/11 --------------------ANECRILTNARCLSEGVDVPALDAVLFLHPRNSVVDVVQS
gi|493372923|ref|WP_006329236.1|helicase[Gordoniarhizosphera]_gi|403207917|dbj|GAB88168.1|hypotheticalproteinGORHZ_006_00370[Go --------------------EDECRILTNARCLSEGVDVPALDAVLFLQPRNSIVDVVQS
gi|764904870|ref|WP_044487821.1|hypotheticalprotein[Mycobacteriumtuberculosis]_gi|625005353|gb|KBZ57120.1|hypotheticalproteinK8 --------------------ENECRILSNARCLSEGVDVPALDAVMFLHPRNSVVDVVQS
gi|927068212|ref|WP_053778441.1|damageinducibleprotein[Gordoniasp.NB41Y]_gi|926660541|gb|EMP14526.2|damageinducibleprotein[G/11 --------------------DNECRILTNARCLSEGVDVPALDSVMFLHPRNSVVDVVQS
gi|935514169|ref|WP_054429894.1|damageinducibleprotein[Mycobacteriumimmunogenum]_gi|929801120|gb|KPG29247.1|damageinduciblepr/1 --------------------ENECRILSNARCLSEGVDVPALDAVMFLHPRNSVVDVVQS
gi|941317728|ref|WP_055118574.1|hypotheticalprotein[Mycobacteriumperegrinum]/11657 --------------------ENECRILSNARCLSEGVDVPALDAVMFLHPRNSVVDVVQS
gi|933683673|gb|ALG86377.1|hypotheticalproteinACH46_20120[Gordoniasp.QH11]/11636 --------------------DGDCRILTNARCLSEGVDVPALDAVMFLNPRNSVVDVVQS
gi|873553947|emb|CRY84110.1|Predictedhelicase[Nocardiafarcinica]/11688 --------------------EGECRILTNARCLSEGVDVPALDAVMFLNPRNSVVDVVQS
gi|499525749|ref|WP_011212389.1|helicase[Nocardiafarcinica]_gi|54019339|dbj|BAD60707.1|hypotheticalprotein[NocardiafarcinicaIFM --------------------EGECRILTNARCLSEGVDVPALDAVMFLNPRNSVVDVVQS
gi|640109609|ref|WP_024794187.1|damageinducibleprotein[Tomitellabiformata]/11653 --------------------EGECRILTNARCLSEGVDVPALDAVMFLNPRNSVVDVVQS
gi|640109991|ref|WP_024794334.1|hypotheticalprotein[Tomitellabiformata]/11639 --------------------EDECRILTNARCLSEGVDVPALDAVMFLNPRNSVVDVVQS
gi|939125496|ref|WP_054831438.1|damageinducibleprotein[Rhodococcuserythropolis]/11669 --------------------EGEARILTNARCLSEGVDVPALDAVMFLNPRNSVVDVVQS
gi|910258550|ref|WP_050062711.1|hypotheticalprotein[Rhodococcussp.RD6.2]_gi|846871967|emb|CRK49225.1|conservedhypotheticalprote --------------------EGECRILTNARCLSEGVDVPALDAVMFLNPRNSVVDVVQS
gi|797148189|gb|KJY52358.1|putativeHelicase[Bifidobacteriumasteroides]/11801 ----------------D---EGECRMLSNAKCLTEGVDLPQLDAVIFFQPRASQVDIVQA

```
gi|535476570|gb|EFG27357.2|hypotheticalproteinHMPREF9020_00999[ScardoviainopinataF0304]/11827    ----------------D---PDGCRILSNAKCLTEGVDLPLLDAVIFFQPRASQVDIVQA
gi|737482729|ref|WP_035462496.1|hypotheticalprotein[Alicyclobacillusmacrosporangiidus]/11512    ----------------PEGNLVEHVLITNARVLTEGIDVPDLDAVVFLEPRKSQIDVIQA
gi|685434354|gb|KGA95679.1|helicase[BacillusalcalophilusATCC27647=CGMCC1.3604]/11575    ----------------P---DHTCRILSNVRFLTEGVDVPDLDAVMFLKPRKSKIDIAQA
gi|945370253|gb|KQU27251.1|helicase[Bacillussp.Leaf13]/11557    ----------------P---DNTCRILSNARFLTEGVDVPDLDAIMFLKPRKSKIDIAQA
gi|880997015|ref|WP_048719116.1|helicase[Enterococcushirae]/11563    ----------------P---ENTCRILSNARFLTEGVDVPDLDAVMFLKPRKSKIDIAQA
gi|945356929|gb|KQU14100.1|helicase[Bacillussp.Leaf49]/11547    ----------------P---SNTCRILSNARFLTEGVDVPDLDAVMFLKPRRSKIDIAQA
gi|763482876|gb|KIZ53217.1|helicase[Bacillussafensis]/11563    ----------------N---DRTCRILSNARFLTEGVDVPDLDAVMFLKPRKSKIDIAQA
gi|852223292|ref|WP_048309707.1|helicase[Anaerobacillusmacyae]_gi|850322910|gb|KMM38590.1|helicase[Anaerobacillusmacyae]/11573    ----------------P---SNTCRILSNARFLTEGVDVPDLDAVMFLKPRKSRIDIAQA
gi|739708665|ref|WP_037562619.1|helicase[Sporolactobacillusterrae]/11573    ----------------P---DNTCRILSNARFLTEGVDIPDLDAVMFLKPRKSRIDVAQA
gi|496687561|ref|WP_009329104.1|MULTISPECIES:helicase[Bacillus]_gi|317390867|gb|EFV71668.1|endonucleaseandmethylaseLlaGI[Bacill    ----------------P---PNTCRILSNARFLTEGVDVPDLDAVMFLKPRKSRIDIAQA
gi|930213599|gb|KPH74225.1|helicase[Oceanobacilluscaeni]/11569    ----------------P---SNTCRILSNARFLTEGVDVPDLDAVMFLKPRKSRIDIAQA
gi|516366003|ref|WP_017756036.1|helicase[Bacillussp.ZYK]/11571    ----------------P---PSTCRILSNARFLTEGVDVPDLDAVMFLKPRKSRIDIAQA
gi|488909977|ref|WP_002821052.1|helicase[Oenococcusoeni]_gi|399965230|gb|EJN99855.1|endonucleaseandmethylaseLlaGI[Oenococcusoen    ----------------D---DNQARVLSNVKFLTEGIDVPNLDAVIFFAPKHSQVDIVQA
gi|495019967|ref|WP_007745780.1|helicase[Oenococcuskitaharae]_gi|366983682|gb|EHN59081.1|typeIIIrestrictionenzyme,ressubunit[Oe    ----------------D---DNQARVLSNVKFLTEGIDVPNLDAVIFFAPKKSQVDIVQA
gi|489666119|ref|WP_003570406.1|typeIIIrestrictionendonuclease[Lactobacilluscasei]_gi|410527834|gb|EKQ02696.1|typeIIIrestrictio    ----------------P---DNEARVLSNVKFLTEGIDVPNLDAIIFFAPKQSQIDIVQA
gi|511447623|gb|EPC78110.1|superfamilyIIDNA/RNAhelicase[Lactobacillusparacaseisubsp.paracaseiLpp221}/11615    ----------------P---DNEARLLSNVKFLTEGIDVPNLDAIIFFAPKKSQIDIVQA
gi|497700411|ref|WP_010014595.1|helicase[Lactobacilluscoryniformis]/11585    ----------------P---DDEARVLSNVKFLTEGIDVPNLDAIIFFAPKQSQIDIVQA
gi|489784048|ref|WP_003687939.1|helicase[Lactobacillusmali]_gi|394428539|gb|EJF01080.1|superfamilyIIDNA/RNAhelicase[Lactobacill    ----------------P---DDEARLLSNVKFLTEGIDVPNLDAIIFFAPKQSQIDIVQA
gi|446780825|ref|WP_000858081.1|helicase[Staphylococcusaureus]_gi|374396604|gb|EHQ67835.1|typeIIIrestrictionenzyme,ressubunit[S    ----------------D---NNEARVLSNVKFLTEGIDVPNLDAVIFFAPKKSQVDIVQA
gi|510796658|ref|WP_016171456.1|hypotheticalprotein[Enterococcusdispar]_gi|508167532|gb|EOT44083.1|hypotheticalproteinOMK_00227    ----------------D---IDEARVLSNVKFLTEGIDVPNLDAVIFFSPKKSQVDIVQA
gi|612613473|gb|EZS30411.1|hypotheticalproteinW603_02482[StaphylococcusaureusVET0353R]_gi|612881917|gb|EZU93992.1|hypotheticalp    ----------------D---IDEARVLSNVKFLTEGIDVPNLDAVIFFSPKKSQVDIVQA
gi|488332893|ref|WP_002402278.1|helicase[Enterococcusfaecalis]_gi|306513864|gb|EFM82466.1|helicaseCterminaldomainprotein[Enter/    ----------------A---EDETRVLSNVKFLTEGIDVPNLDAVIFLAPKKSQVDIVQA
gi|921166029|ref|WP_053111806.1|helicase[Enterococcusdurans]_gi|915771660|gb|AKX85928.1|helicase[Enterococcusdurans]/11562    ----------------A---EDETRVLSNVKFLTEGIDVPNLDAVIFLAPKKSQVDIVQA
gi|498525358|ref|WP_010825030.1|hypotheticalprotein[Enterococcusfaecalis]_gi|487059631|gb|EOJ86727.1|hypotheticalproteinWOC_005    ----------------A---EDETRVLSNVKFLTEGIDVPNLDAVIFLAPKKSQVDIVQA
gi|498412624|ref|WP_010722070.1|hypotheticalprotein[Enterococcusfaecium]_gi|486634654|gb|EOF75253.1|hypotheticalproteinSGC_0253    ----------------A---EDETRVLSNVKFLTEGIDVPNLDAVIFLAPKKSQVDIVQA
gi|498408534|ref|WP_010718364.1|hypotheticalprotein[Enterococcusfaecium]_gi|486609608|gb|EOF50873.1|hypotheticalproteinSCW_0257    ----------------A---EDETRVLSNVKFLTEGIDVPNLDAVIFLAPKKSQVDIVQA
gi|498407475|ref|WP_010717483.1|hypotheticalprotein[Enterococcusfaecalis]_gi|486594855|gb|EOF36348.1|hypotheticalproteinSCK_005    ----------------A---EDETRVLSNVKFLTEGIDVPNLDAVIFLAPKKSQVDIVQA
gi|514885392|ref|WP_016615344.1|helicase[Enterococcusfaecalis]_gi|514505843|gb|EPH71676.1|helicaseprotein[Enterococcusfaecalis0    ----------------A---EDETRVLSNVKFLTEGIDVPNLDAVIFLAPKKSQVDIVQA
gi|325157603|gb|EGC69759.1|helicaseCterminaldomainprotein[EnterococcuscasseliflavusATCC12755]/11561    ----------------A---EDETRVLSNVKFLTEGIDVPNLDAVIFLAPKKSQVDIVQA
gi|895856206|ref|WP_048967668.1|helicase[Enterococcusfaecalis]/11551    ----------------A---EDETRVLSNVKFLTEGIDVPNLDAVIFLAPKKSQVDIVQA
gi|639458836|ref|WP_024636366.1|helicase[Enterococcusfaecium]_gi|658176502|gb|KEI51159.1|helicase[EnterococcusfaeciumUC7256]/1/    ----------------A---EDETRVLSNVKFLTEGIDVPNLDAVIFLAPKKSQVDIVQA
gi|488266511|ref|WP_002337719.1|hypotheticalprotein[Enterococcusfaecium]_gi|430581409|gb|ELB19854.1|hypotheticalproteinOIQ_0351    ----------------A---EDETRVLSNVKFLTEGIDVPNLDAVIFLAPKKSQVDIVQA
gi|446353356|ref|WP_000431211.1|helicase[Staphylococcusaureus]_gi|253726329|gb|EES95058.1|helicaseCterminaldomainprotein[Staph/    ----------------S---SDEARIVSNVRFLTEGIDVPTLDAVIFLAPKKSQVDIVQA
gi|672712068|emb|CDM14336.1|DNAorRNAhelicaseofsuperfamilyII[StaphylococcusepidermidisPM221]/11464    ----------------P---SDEARIVSNVRFLTEGIDVPTLDAVIFLAPKKSQVDIVQA
gi|896298990|ref|WP_049279905.1|helicase[Staphylococcusaureus]/11571    ----------------P---SDEARIVSNVRFLTEGIDVPTLDAVIFLAPKKSQVDIVQA
gi|541903432|gb|ERH36190.1|hypotheticalproteinSEQU_00085[StaphylococcusequorumUMCCNS924]/11578    ----------------P---LDEARIVSNVRFLTEGIDVPTLDAVIFLAPKKSQVDIVQA
gi|488289747|ref|WP_002360955.1|helicase[Enterococcusfaecalis]_gi|256682216|gb|EEU21911.1|endonucleaseandmethylaseLlaGI[Enteroc    ----------------P---SDEARIVSNVRFLTEGIDVPTLDAVIFLAPKKSQVDIVQA
gi|494392045|ref|WP_007207894.1|helicase[Enterococcusitalicus]_gi|315483798|gb|EFU74284.1|helicaseCterminaldomainprotein[Enter/    ----------------P---SDEARIVSNVRFLTEGIDVPTLDAVIFLAPKKSQVDIVQA
gi|446350692|ref|WP_000428547.1|helicase[Bacilluscereus]_gi|228727580|gb|EEL78721.1|RestrictionmodificationsystemLlaBIII[Bacil/    ----------------P---SDEARIVSNVRFLTEGIDVPTLDAIIFLAPKKSQVDIVQA
gi|488255302|ref|WP_002326510.1|hypotheticalprotein[Enterococcusfaecium]_gi|430480276|gb|ELA57459.1|hypotheticalproteinOGE_0473    ----------------P---EDEARIVSNVRFLTEGIDVPTLDAVIFLAPKKSQVDIVQA
gi|843253770|ref|WP_047916698.1|helicase[Lactococcuspiscium]_gi|802124677|emb|CEN29502.1|EndonucleaseandmethylaseLlaGI,TypeISPr    ----------------P---ADEARIVSNVRFLTEGIDIPTLDAVIFLSPKKSQVDIVQA
LlaBIII/11578    ----------------P---ADEARIVSNVRFLTEGIDIPTLDAVIFLSPKKSQVDIVQA
gi|499320531|ref|WP_011011023.1|helicase[Listeriainnocua]_gi|16415805|emb|CAC42019.1|pli0021[ListeriainnocuaClip11262]/11569    ----------------P---ANEARIVSNVRFLTEGIDIPTLDAVIFLSPKKSQVDIVQA
gi|760026859|ref|WP_043709973.1|helicase[Weissellacibaria]_gi|758337165|gb|KIU20061.1|UvsWhelicase[Weissellacibaria]/11567    ----------------P---ADEARIVSNVRFLTEGIDIPTLDAVIFLSPKKSQVDIVQA
LlaGI/11570    ----------------P---ADEARIVSNVRFLTEGIDIPTLDAVIFLSPKKSQVDIVQA
gi|499988414|ref|WP_011669132.1|helicase[Lactococcuslactis]_gi|116109019|gb|ABJ74139.1|DNAorRNAhelicaseofsuperfamilyII[Lactococ    ----------------P---ADEARIVSNVRFLTEGIDIPTLDAVIFLSPKKSQVDIVQA
gi|685938360|ref|WP_031670319.1|helicase[Listeriamonocytogenes]_gi|590403387|gb|EXL23261.1|hypotheticalproteinX846_1956[Listeri    ----------------P---ADEARIVSNVRFLTEGIDIPTLDAVIFLSPKKSQVDIVQA
gi|530793219|gb|EQC56240.1|helicase[Lactococcuslactissubsp.cremorisTIFN5]_gi|530793247|gb|EQC56263.1|helicase[Lactococcuslactis    ----------------P---ADEARIVSNVRFLTEGIDIPTLDAVIFLSPKKSQVDIVQA
gi|695270006|ref|WP_032492075.1|hypotheticalprotein[Listeriamonocytogenes]_gi|695212872|ref|YP_009081522.1|hypotheticalproteinp    ----------------P---ADEARIVSNVRFLTEGIDIPTLDAVIFLSPKKSQVDIVQA
gi|504101310|ref|WP_014335297.1|helicase[Leuconostocmesenteroides]_gi|859247905|gb|AKO63885.1|helicase(plasmid)[Leuconostocmese    ----------------P---ADEARIVSNVRFLTEGIDIPTLDAVIFLSPKKSQVDIVQA
gi|488905190|ref|WP_002816265.1|helicase[Leuconostocmesenteroides]_gi|227351522|gb|EEJ41777.1|helicaseCterminaldomainprotein[L/    ----------------P---DDEARIVSNVKFLTEGIDIPTLDAVIFLSPKKSQVDIVQA
gi|518121285|ref|WP_019291493.1|MULTISPECIES:hypotheticalprotein[Lactococcus]/11588    ----------------P---ENEARILSNVRFLTEGIDVPNLDAIIFLSSRKSQVDIVQA
gi|754857213|ref|WP_042217902.1|helicase[Lactococcusgarvieae]/11549    ----------------P---ENEARILSNVRFLTEGIDVPNLDAIIFLSPRKSQVDIVQA
gi|640048455|ref|WP_024784762.1|helicase[Streptococcusmutans]/11564    ----------------P---DNSARILSNVRFLTEGIDVPNLDAIVFLSPRKSQVDIVQA
gi|512695361|ref|WP_016476323.1|hypotheticalprotein[Veillonellasp.HPA0037]_gi|512079853|gb|EPD79018.1|hypotheticalproteinHMPREF    ----------------P---ENSARILSNVRFLTEGIDVPNLDAIIFLSPRKSQIDIVQA
gi|636963514|ref|WP_024403747.1|helicase[Streptococcussuis]/11574    ----------------P---EDSARILSNVRFLTEGIDVPNLDAIIFLSPRKSQVDIVQA
gi|881115312|ref|WP_048802949.1|helicase[Staphylococcuspasteuri]/11573    ----------------P---EDSARILSNVRFLTEGVDVPNLDAIVFLSPRKSQVDIVQA
gi|446063996|ref|WP_000141851.1|helicase[Streptococcusmitis]_gi|307618725|gb|EFN97864.1|endonucleaseandmethylaseLlaGI[Streptoco    ----------------P---EDSARILSNVRFLTEGIDVPNLDAIIFLSPRKSQVDIVQA
gi|488206609|ref|WP_002277817.1|restrictionmodificationsystemLlaBIII[Streptococcusmutans]_gi|449173033|gb|EMB75626.1|restricti/    ----------------P---ENSARILSNVRFLTEGIDVPNLDAIIFLSPRKSQVDIVQA
gi|640023290|ref|WP_024781621.1|helicase[Streptococcusmutans]/11566    ----------------P---ENSARILSNVRFLTEGIDVPNLDAIIFLSPRKSQVDIVQA
gi|814946581|ref|WP_046393781.1|helicase[Streptococcusuberis]_gi|810600885|gb|KKF57499.1|helicase[StreptococcusuberisB362]/1156    ----------------P---ENSARILSNVRFLTEGIDVPNLDAIVFLSPRKSQIDIVQA
gi|765470762|ref|WP_044720336.1|helicase[Streptococcussuis]/11559    ----------------P---EESARILSNVRFLTEGIDVPNLDAIIFLSPRKSQVDIVQA
gi|654507127|ref|WP_027976443.1|helicase[Streptococcusdevriesei]/11558    ----------------P---EDSARILSNVRFLTEGIDVPNLDAIIFLSPRKSQVDIVQA
gi|530812902|gb|EQC72481.1|DNAorRNAhelicaseofsuperfamilyII[Streptococcussp.HSISS2]/11570    ----------------P---EDSARILSNVRFLTEGIDVPNLDAIIFLSPRKSQVDIVQA
gi|489158846|ref|WP_003068504.1|helicase[Streptococcusintermedius]_gi|355367114|gb|EHG14827.1|hypotheticalproteinHMPREF9682_002    ----------------P---EDSARILSNVRFLTEGIDVPNLDAIIFLSPRKSQVDIVQA
RM.Lru27782ORF13200P/11620    ----------------P---ENTARVLSNVRFLTEGIDVPNLDAIIFMSPRKSQVDIVQA
gi|492676546|ref|WP_005919236.1|DNAphosphorothioationsystemrestrictionenzyme[Pediococcusacidilactici]_gi|482517924|gb|EOA08103.    ----------------P---ENHARVLSNVRFLTEGIDVPSLDAVIFFSPKKSQVDIVQA
```

gi|498183116|ref|WP_010497272.1|helicase[Lactobacillusacidipiscis]/11563	----------------P---DNQARVLSNVRFLTEGIDVPNLDGVIFFSPKKSQVDIVQA
gi|822100485|ref|WP_046921865.1|helicase[Lactobacillusruminis]_gi|821684442|gb|KLA43321.1|restrictionmodificationsystemLlaBIII/	----------------P---ENQARVLSNVRFLTEGIDVPNLDAIIFMSPKKSQVDIVQA
gi|935565832|ref|WP_054450311.1|helicase[Lactobacilluskunkeei]/11573	----------------E---DNRARILSNVRFLTEGIDVPNLDAIIFFSPKKSQVDIVQA
gi|752296887|ref|WP_041152901.1|helicase[Lactobacilluskunkeei]_gi|751663520|gb|KIM18358.1|helicase[Lactobacilluskunkeei]/11577	----------------E---ENRARILSNVRFLTEGIDVPNLDAIIFFSPKKSQVDIVQA
gi|495745291|ref|WP_008469870.1|helicase[Lactobacillushominis]_gi|394484442|emb|CCI81207.1|RestrictionmodificationsystemLlaBII/	----------------E---NNQARILSNVRFLTEGIDVPNLDGIIFFSPKKSQVDIVQA
gi|918672414|ref|WP_052543134.1|helicase[Lactobacillussp.]/11564	----------------E---DNHARILSNVRFLTEGIDVPNLDAIIFFSPKKSQVDIVQA
gi|799141631|ref|WP_045927261.1|helicase[Lactobacilluskullabergensis]_gi|797155335|gb|KJY59264.1|putativerestrictionendonucleas	----------------E---ENHARVLSNVRFLTEGIDVPNLDAIIFFSPKKSQVDIVQA
gi|504379990|ref|WP_014567092.1|helicase[Lactobacillusjohnsonii]_gi|329666726|gb|AEB92674.1|putativerestrictionendonuclease[Lac	----------------E---ENHARVLSNVRFLTEGIDVPNLDAIIFFSPKKSQVDIVQA
gi|489880179|ref|WP_003783646.1|DNAdamageinducibleprotein[Actinomycesnaeslundii]_gi|399901561|gb|EJN84440.1|typeIIIrestriction/	-PAPTRQASSASES---EETSVSCRILSNARCLSEGVDVPALDAVMFLSPRKSQVDIVQS
gi|496524497|ref|WP_009232780.1|DNAdamageinducibleprotein[Actinomycessp.oraltaxon849]_gi|365265413|gb|EHM95177.1|hypotheticalp/	-PAPTRQASSASES---EETSVSCRILSNARCLSEGVDVPALDAVMFLSPRKSQVDIVQS
gi|915397782|ref|WP_050783167.1|damageinducibleprotein[Actinomycesoris]/11694	-PAPTRQASSTAESEGPEGTSVSCRILSNARCLSEGVDVPALDAVMFLSPRKSQVDIVQS
gi|502925248|ref|WP_013160224.1|helicase[Propionibacteriumfreudenreichii]_gi|296921272|emb|CBL55824.1|helicase[Propionibacteriu	-----------------PVPEGECRILSNARCLSEGVDVPALDAVLFLSPRNSLVDVVQS
gi|732164352|emb|CEG87481.1|helicase[Propionibacteriumfreudenreichii]/11618	-----------------PIPDGETRVVSNARCLAEGVDVPALDAVLFLHPRNSMVDVIQS
gi|739047402|ref|WP_036925058.1|damageinducibleprotein[Propionibacteriumthoenii]/11596	-----------------PVPEGECRILSNARCLAEGVDVPALDAVLFLSPRNSLVDVVQS
gi|739112839|ref|WP_036982533.1|damageinducibleprotein[Propionibacteriumjensenii]/11594	-----------------PVPEGECRILSNARCLAEGVDVPALDAVLFLSPRNSLVDVVQS
gi|918023558|ref|WP_052314121.1|damageinducibleprotein[Nocardiathailandica]/11612	-----------------EADPDVCRVLTNARCLSEGVDVPALDAVMFLTPRGSQVDVVQS
gi|498380271|ref|WP_010694427.1|DNAdamageinducibleprotein[Saccharopolysporaspinosa]/11605	-----------------EPESNVCRILTNARCLSEGVDVPSLDAVLFLTPRSSQVDVVQS
gi|749991459|ref|WP_040339809.1|damageinducibleprotein[CandidatusBlastococcusmassiliensis]/11597	-----------------PAPDGVCRVLTNARCLSEGVDVPSLDAVLFLTPRGSQVDVVQS
gi|754177801|ref|WP_041796231.1|damageinducibleprotein[Modestobactermarinus]/11592	-----------------SHTTGVCRVLTNARCLSEGVDVPALDAVLFLTPRGSQVDVVQS
gi|504188248|ref|WP_014375350.1|DNAdamageinducibleprotein[Blastococcussaxobsidens]_gi|378782790|emb|CCG02456.1|putativeHelicas/	-----------------PAGDGVCRVLTNARCLSEGVDVPALDAVLFLTPRGSQVDVVQS
gi|946476713|gb|KRF26798.1|damageinducibleprotein[Phycicoccussp.Soil803]/11609	-----------------EETDNVCRVLTNVRCLSEGVDVPALDAVMFLTPRGSQVDIVQS
gi|700176672|gb|KGN37509.1|DNAdamageinducibleprotein[KnoelliasubterraneaKCTC19937]/11633	-----------------EETDNVCRVLTNVRCLSEGVDVPALDAVMFLTPRGSQVDIVQS
gi|700168841|gb|KGN29914.1|hypotheticalproteinN802_10215[KnoelliasinensisKCTC19936]/11631	-----------------EETDNVCRVLTNVRCLSEGVDVPALDAVMFLTPRGSQVDIVQS
gi|946426241|gb|KRE76610.1|damageinducibleprotein[Arthrobactersp.Soil763]/11606	-----------------EVPDGQCRILTNARCLSEGVDVPGLDAVMFMAPRKSVVDVVQS
gi|517318525|ref|WP_018500927.1|hypotheticalprotein[Frankiasp.BCU110501]/11592	-----------------EPRDGVCRILTNARCLSEGIDVPALDAVMFLSPRKSVVDIVQS
gi|499072429|ref|WP_010851327.1|Helicase[Tetrasphaeraelongata]_gi|478756579|emb|CCH71501.1|putativeHelicase[Tetrasphaeraelongat	-----------------DVPNGVCRILTNARCLSEGVDVPALDAVMFLSPRKSVVDIVQS
gi|750080666|ref|WP_040385350.1|damageinducibleprotein[Demetriaterragena]/11625	-----------------EPAEGTCRVLSNARCLSEGVDVPSLDTVMFLTARSSEVDVVQS
gi|752558292|ref|WP_041229523.1|damageinducibleprotein[Dermacoccusnishinomiyaensis]_gi|664689315|gb|AIF41904.1|damageinducibl/1	-----------------EPEANTCRVLTNARCLSEGVDVPSLDAVMFLSPRGSEVDIVQS
gi|829089213|ref|WP_047310701.1|damageinducibleprotein[Dermacoccussp.PE3]_gi|828384812|gb|KLO63770.1|damageinducibleprotein[D/1	-----------------EPADNTCRILTNARCLSEGVDVPNLDAVLFLTPRSSEVDVVQS
gi|748756635|ref|WP_040014750.1|damageinducibleprotein[Dermacoccussp.Ellin185]/11596	-----------------EPADNTCRILTNARCLSEGVDVPNLDAVLFLTPRSSEADVVQS
gi|916534724|ref|WP_051141848.1|damageinducibleprotein[Actinomycesneuii]/11632	------------------KDSPVCRVLTNARCLSEGVDVPTLDAVFFLNPRRSQVDVIQA
gi|921140030|ref|WP_053090276.1|damageinducibleprotein[Rothiamucilaginosa]/11616	------------------EEGPICRILTNARCLTEGVDVPNLDAVLFLNPRNSMVDVIQA
gi|896406751|ref|WP_049343749.1|damageinducibleprotein[Rothiamucilaginosa]/11647	------------------EEGPICRILTNARCLTEGVDVPNLDAVLFLNPRNSMVDVIQA
gi|723608234|gb|AIY01610.1|putativehelicase[Arthrobactersp.PAMC25486]/11613	LLVDDRTAPRNADGSWPLKEQPTCRILTNARCLTEGVDVPSLDAVLFMNPRNSMVDVIQS
gi|946394527|gb|KRE45160.1|damageinducibleprotein[Arthrobactersp.Soil736]/11616	-----------------DGGQPTCRILTNARCLTEGVDVPSLDAVLFLNPRNSMVDVIQA
gi|515765304|ref|WP_017197904.1|hypotheticalprotein[Arthrobactersp.M2012083]/11648	-----------------PEDQPVCRILTNARCLTEGVDVPSLDAVLFLNPRNSMVDVIQA
gi|945315587|gb|KQT73127.1|damageinducibleprotein[Microbacteriumsp.Leaf436]/11612	-------------------PDSTVRILSNAKCLSEGVDVPALDAVMFMNPRKSVVDVVQS
gi|759908497|ref|WP_043594599.1|damageinducibleprotein[Frigoribacteriumsp.MEB024]_gi|758271359|gb|KIU03346.1|damageinduciblep/1	-------------------GDNVARILTNARCLSEGVDVPSLDAVIFLNSRDSQVDVVQS
gi|945072850|gb|KQR34237.1|hypotheticalproteinASF75_00055[Curtobacteriumsp.Leaf154]/11597	-------------------DQNVCRILTNARCLSEGVDVPALDAVIFLNSRDSQVDVVQS
gi|551263152|ref|WP_022883363.1|DNAdamageinducibleprotein[Glaciibactersuperstes]/11621	-P-----------------EDNLCRILTNARCLSEGVDVPALDAVIFLNSRDSQVDVVQS
gi|945452341|gb|KQV08491.1|hypotheticalproteinASC63_10170[Leifsoniasp.Root112D2]/11623	-P-----------------TENGCRILTNARCLSEGVDVPALDAVIFLNSRDSQVDVVQS
gi|742759483|gb|KIC69912.1|damageinducibleprotein[Kocuriarhizophila]/11610	--------------------QGECRILTNARCLSEGVDVPSLDAVMFLNPRNSQVDVVQS
gi|737919995|ref|WP_035885304.1|damageinducibleprotein[Kocuriarhizophila]/11611	--------------------QGECRILTNARCLSEGVDVPSLDAVMFLNPRNSQVDVVQS
gi|737997701|ref|WP_035959325.1|damageinducibleprotein[Kocuriamarina]_gi|725823884|gb|KHE75603.1|damageinducibleprotein[Kocur/1	--------------------QGECRILTNARCLSEGVDVPSLDAVMFLNPRNSQVDVVQS
gi|664521070|ref|WP_031037650.1|damageinducibleprotein[Streptomycessp.NRRLWC3774]/11624	--------------------QNQARILSNARCLSEGVDVPSLDAVLFLHPRNSVVDVVQS
gi|500101034|ref|WP_011777041.1|DNAdamageinducibleprotein[Arthrobacteraurescens]_gi|119951547|gb|ABM10457.1|putativeHelicase[A/	NQ-----------------DGDVCRVLSNARCLSEGVDVPALDAVLFLNPRNSQVDVVQS
gi|916871953|ref|WP_051479009.1|damageinducibleprotein[Arthrobactersp.H5]/11588	-G-----------------DGGVCRVLSNARCLSEGVDVPALDAVLFLNPRNSQVDVVQS
gi|612084649|gb|EZP43369.1|TypeIIIrestrictionenzyme,ressubunit[Micrococcusluteus]/11625	-------------------GENTVRVLSNAKCLSEGVDVPTLDAVLFLSPRNSQVDVVQS
gi|759739708|ref|WP_043450422.1|damageinducibleprotein[Arthrobacterphenanthrenivorans]_gi|742758264|gb|KIC68716.1|damageinduc/1	-------------------GANSCRILTNARCLSEGVDVPSLDAVLFLNPRNSQVDVVQS
gi|636845448|ref|WP_024366823.1|damageinducibleprotein[Arthrobactersp.TB26]/11605	--------------------GNVCRILSNAKCLTEGVDVPSLDAVLFLNPRNSQVDVVQA
gi|500009025|ref|WP_011689743.1|DNAdamageinducibleprotein[Arthrobactersp.FB24]_gi|116613051|gb|ABK05760.1|typeIIIrestrictionen/	--------------------GNVCRILSNAKCLTEGVDVPSLDAVLFLNPRNSQVDVVQA
gi|821249847|gb|KKZ12083.1|hypotheticalproteinTQ37_06090[CandidatusSynechococcusspongiarum15L]/11436	--------------------PGDIRILTNARCLTEGVDVPALDGILFMHPRKSQIEVVQA
gi|739446902|ref|WP_037306885.1|damageinducibleprotein[Ruegeriahalocynthiae]/11592	-------------------EHNGCKILSNARCLSEGVDVPSLDAILFLHPRKSQIDVVQS
gi|640620081|ref|WP_025048131.1|damageinducibleprotein[Sulfitobactermediterraneus]_gi|751956650|gb|KIN77213.1|TypeIIIrestricti/	-------------------ANDSCKILSNARCLSEGVDVPALDAIMFLHPRKSQIDVVQS
gi|740247634|ref|WP_038088583.1|damageinducibleprotein[Thiobacillusprosperus]_gi|684049748|gb|KFZ90853.1|putativehelicase[Thio/	-------------------DAGQCRILSNARCLSEGVDVPALDAVVFFDTRESIVDIVQS
gi|518420321|ref|WP_019590528.1|hypotheticalprotein[Thioalkalivibriosp.ALE20]/11603	-------------------EDTSCRILSNARCLSEGVDVPALDAVVFFDTRESIVDIVQS
gi|500156826|ref|WP_011831496.1|DNAdamageinducibleprotein[Methylibiumpetroleiphilum]_gi|124261892|gb|ABM96881.1|adeninespecifi/	-------------------GDGSCRILSNARCLSEGIDVPALDAVAFFDTRESVVDIVQS
gi|769978537|ref|WP_045094522.1|damageinducibleprotein[Legionellafallonii]_gi|698841736|emb|CEG55682.1|Putativeproteinwithboxh/	--------------------SDECRILSNARCLSEGIDVPALDAVVFFDTRESIVDIVQS
gi|553752541|ref|WP_023085344.1|hypotheticalprotein[Pseudomonasaeruginosa]_gi|552201952|gb|ERU62653.1|hypotheticalproteinQ088_0	-------------------EPGECRILSNARCLSEGIDVPTLDAVVFFDTRESIVDIVQS
gi|880980418|ref|WP_048704461.1|damageinducibleprotein[Thauerahumireducens]_gi|875132785|gb|KMU70944.1|damageinducibleprotein/1	-------------------EAGDCRILSNARCLSEGIDVPTLDAVVFFDTRESIVDIVQS
gi|740135732|ref|WP_037982815.1|damageinducibleprotein[Thauerasp.27]/11603	-------------------EAGDCRILSNARCLSEGIDVPTLDAVVFFDTRESIVDIVQS
gi|738017371|ref|WP_035978332.1|damageinducibleprotein[Burkholderiaglumae]/11380	-------------------AEGHCRILSNARCLSEGIDVPALDAVVFFDTRESIVDIVQS
gi|498506003|ref|WP_010806671.1|typeIIIrestrictionenzyme,ressubunit[Pandoraeasp.SD62]_gi|490314561|gb|EON12879.1|typeIIIrestri/	-------------------DEGHCRILSNARCLSEGIDVPALDAVVFFDTRESIVDIVQS
gi|500117748|ref|WP_011793753.1|DNAdamageinducibleprotein,Acidovoraxcitrulli]_gi|120587742|gb|ABM31182.1|typeIIIrestrictionenz/	-------------------GEGQCRILTNARCLSEGIDVPALDAVVFFDTRESIVDIVQS
gi|651617328|ref|WP_026611444.1|damageinducibleprotein[Methylocaldumszegediense]/11601	-------------------GEGQCRILSNARCLSEGIDVPALDAVVFFDTRESIVDIVQS
gi|737210208|ref|WP_035194994.1|damageinducibleprotein[Acidithiobacillusferrivorans]_gi|669953220|emb|CDQ11755.1|Adeninespecif/	-------------------GENTCRILTNVRCLSEGVDVPALDAVIFLNPRDSIVDVVQS
gi|935697386|ref|WP_054506180.1|damageinducibleprotein[Achromobactersp.]_gi|928716153|emb|CUK12836.1|Hefnuclease[Achromobacter/	-------------------HDEVCRILTNARCLTEGVDVPALDAAIFLNPRDSVVDVVQS

gi|429502517|gb|ELA00827.1|adeninespecificDNAmethyltransferase[Cupriavidussp.HMR1]/11599	-------------------DEDVCRILTNARCLTEGVDVPALDAAIFLNPRDSVVDVVQS
gi|506356642|ref|WP_015876361.1|DNAdamageinducibleprotein[Burkholderiaglumae]_gi|237877158|gb|ACR29491.1|AdeninespecificDNAmet/	-------------------DEDVCRILTNARCLSEGVDVPALDAAIFLNPRDSVVDVVQS
gi|696406787|ref|WP_032978003.1|damageinducibleprotein[Bordetellahinzii]_gi|625335076|gb|KCB49343.1|typeIIIrestrictionenzyme,r/	-------------------EEPVCRILTNARCLSEGVDVPALDAAIFLNPRDSVVDVVQS
gi|817529125|gb|KKO72587.1|damageinducibleprotein[Kerstersiagyiorum]/11592	-------------------EEPVCRILTNARCLSEGVDVPALDAAIFLNPRDSVVDVVQS
gi|737048910|ref|WP_035044118.1|damageinducibleprotein[Aquabacteriumsp.NJ1]_gi|699052896|gb|KGM38706.1|damageinducibleprotein/1	-------------------DEALCRILTNARCLSEGVDVPALDAAIFLNPRDSVVDVVQS
gi|822492519|ref|WP_046933319.1|MULTISPECIES:damageinducibleprotein[Xanthomonas]_gi|821703525|gb|KLA61196.1|damageinduciblepr/1	-------------------DEPVCRILTNARCLSEGVDVPALDAAIFLNPRDSVVDVVQS
gi|751252861|ref|WP_040963740.1|damageinducibleprotein[Xanthomonasfuscans]_gi|749808363|gb|KIJ03887.1|damageinducibleprotein[/1	-------------------EEPVCRILTNARCLSEGVDVPALDAAIFLNPRDSVVDVVQS
gi|496774980|ref|WP_009368792.1|DNAdamageinducibleprotein[Bilophilasp.4_1_30]_gi|345039902|gb|EGW44205.1|hypotheticalproteinHM/	-------------------EENECRILTNAKCLSEGVDVPALDCAIFLNPRDSVVDVVQS
gi|499241912|ref|WP_010939452.1|DNAdamageinducibleprotein[Desulfovibriovulgaris]_gi|46580580|ref|YP_011388.1|adeninespecificDN/	AP---------------ADAPATCRILTNAKCLSEGVDVPALDCAIFLSPRDSVVDVVQS
gi|504274913|ref|WP_014462015.1|DNAdamageinducibleprotein[Bacillusmegaterium]_gi|345446919|gb|AEN91936.1|AdeninespecificDNAmet/	------------------GKENLCRILSNARCLTEGVDVPDLDAVMFLNPRNSVVDVVQA
gi|504895966|ref|WP_015083068.1|typeIIrestrictionenzymeandmethyltransferasesubtypeG[Anabaenasp.90]_gi|413972424|gb|AFW96512.1|t	SP-------PSQ---GGVRGGNICRILSNARCLSEGVDVPALDAVIFFTPRNSVVDVVQS
gi|518327765|ref|WP_019497972.1|hypotheticalprotein[Calothrixsp.PCC7103]/11600	-----------------NGNGKICRILSNARCLSEGVDVPSLDAVMFLTPRNSVVDVVQS
gi|652765503|ref|WP_027079917.1|damageinducibleprotein[Luteimonasmephitis]/11593	-----------------SDEGNICRILTNARCLSEGVDVPALDAVLFLNPRDSVVDVVQS
gi|896539951|ref|WP_049442123.1|damageinducibleprotein[Stenotrophomonasmaltophilia]/11591	-------------------PGNTCRILSNARCLSEGVDVPALDAVLFLNPRDSVVDVVQS
gi|500217900|ref|WP_011888043.1|DNAdamageinducibleprotein[Geobacillusthermodenitrificans]_gi|134267954|gb|ABO68149.1|Conserved/	------------------PGENRCRILSNARCLTEGVDVPALDAVIFLNPRNSQVDVVQA
gi|696570298|ref|WP_033102014.1|damageinducibleprotein[Thermoactinomycesdaqus]/11594	------------------TDENTCRILSNARCLTEGVDVPTLDAVIFLNPRESQVDVVQA
gi|251772067|gb|EES52637.1|putativehelicase[Leptospirillumferrodiazotrophum]/11618	------------------IGDNECRILSNARCLSEGVDVPALDSVIFLNPRDSVVDVVQS
gi|738124101|ref|WP_036082108.1|damageinducibleprotein[Leptospirillumferriphilum]_gi|685243306|gb|KGA94177.1|putativehelicase[/	------------------IGDNECRILSNARCLSEGVDVPALDGVIFLNPRDSVVDVVQS
gi|558699223|ref|WP_023524766.1|helicase[Leptospirillumsp.GroupII'CF1']_gi|206603764|gb|EDZ40244.1|Putativehelicase[Leptospiri/	------------------IGDNECRILSNARCLSEGVDVPALDSVIFLNPRDSVVDVVQS
gi|406774462|gb|AFS53887.1|putativehelicase[LeptospirillumferriphilumML04]/11610	------------------PGPNACRILSNARCLSEGVDVPSLDAVIFLNPRDSVIDVVQS
gi|740719463|ref|WP_038504749.1|damageinducibleprotein[Leptospirillumferriphilum]_gi|640838125|gb|AIA30173.1|damageinduciblep/1	------------------SGPNSCRILSNARCLSEGVDVPSLDAVIFLNPRDSVIDVVQS
gi|517810766|ref|WP_018980974.1|hypotheticalprotein[Saccharibacterfloricola]/11733	--------------------EGTSRILSNVRCLSEGIDVPSLDAALFLSPRNSTADIIQS
gi|502471675|ref|WP_012798533.1|helicase[Slackiaheliotrinireducens]_gi|256791761|gb|ACV22431.1|predictedhelicase[Slackiaheliotr	--------------------EERCRILSNARCLAEGIDVPNLDAVLFLQPKKSQIDIIQA
gi|657871713|ref|WP_029576297.1|hypotheticalprotein[Bifidobacteriumthermacidophilum]_gi|673007521|gb|KFJ02535.1|helicase[Bifido	--------------------EYQCHILSNARCLAEGIDVPDLDAVIYLSSRRSKVDIIQS
gi|673004757|gb|KFI99791.1|DNAhelicaserestrictionenzymetypeIIIRessubunit[Bifidobacteriumthermacidophilumsubsp.porcinum]/11682	--------------------EYQCHILSNARCLAEGIDVPDLDAVIYLSSRRSKVDIIQS
gi|917316092|ref|WP_051922804.1|hypotheticalprotein[Bifidobacteriumstellenboschense]_gi|673003774|gb|KFI98812.1|helicase[Bifido	--------------------DNECHILSNARCLAEGIDVPDLDAVMYLNARRSKVDIIQS
gi|705415694|ref|WP_033502332.1|hypotheticalprotein[Bifidobacteriumpseudolongum]_gi|672982411|gb|KFI77685.1|DNAdamageinducible/	--------------------ANECRILSNARCLAEGIDVPDLDAVIYLSARKSKVDIIQS
gi|916591418|ref|WP_051198509.1|hypotheticalprotein[Bifidobacteriumsp.AGR2158]/11704	--------------------SNECHILSNARCLAEGVDVPDLDAVIYLHARKSHVDIIQS
gi|772242982|gb|AJW78255.1|hypotheticalproteinVO01_03165[Clavibactermichiganensissubsp.insidiosus]/11642	D----------------RHDRAVARVLTNARCLSEGVDVPSLDAVLFLHPRKSQVDVVQA
gi|916649818|ref|WP_051256909.1|damageinducibleprotein[Actinomycesgraevenitzii]/11677	D------------------EEPVCRILTNARCLSEGVDVPTLDAVLFLSPRNSQVDVIQA
gi|496504578|ref|WP_009212861.1|helicase[Actinomycessp.oraltaxon180]_gi|315312695|gb|EFU60776.1|helicase[Actinomycessp.oraltaxo	D------------------EVPVCRILTNARCLSEGVDVPTLDAVLFLAPRKSQVDVIQA
gi|494862206|ref|WP_007588306.1|helicase[Actinomycessp.ICM39]_gi|398362799|gb|EJN46470.1|typeIIIrestrictionenzyme,ressubunit[Ac	H------------------EVPVCRILTNARCLSEGVDVPTLDAVLFLSPRKSQVDVIQA
gi|881057111|ref|WP_048774974.1|damageinducibleprotein[Sanguibacterkeddieii]/11697	D------------------EVPVCRILTNARCLSEGVDVPTLDAVLFLSPRKSQVDVIQA
gi|881020459|ref|WP_048740808.1|damageinducibleprotein[Kytococcussedentarius]/11704	D------------------EVPVCRILTNARCLSEGVDVPTLDAVLFLSPRKSQVDVIQA
gi|881052313|ref|WP_048770555.1|damageinducibleprotein[Cellulomonasflavigena]/11705	D------------------EVPVCRILTNARCLSEGVDVPTLDAVLFLSPRKSQVDVIQA
gi|490108682|ref|WP_004009424.1|helicase[Mobiluncuscurtisii]_gi|315490465|gb|EFU80089.1|helicaseCterminaldomainprotein[Mobilun/	-------------------DHPVCRILTNARCLSEGIDVPTLDAVLFLNPRKSQVDVIQA
gi|490111935|ref|WP_004012603.1|helicase[Mobiluncusmulieris]_gi|227843812|gb|EEJ53982.1|helicaseCterminaldomainprotein[Mobilun/	-------------------NYPVCRILTNARCLSEGVDVPTLDAVLFLNPRKSFVDVIQA
gi|490114228|ref|WP_004014848.1|helicase[Mobiluncusmulieris]_gi|269934432|gb|EEZ90993.1|helicaseCterminaldomainprotein[Mobilun/	-------------------NYPVCRILTNARCLSEGVDVPTLDAVLFLNPRKSFVDVIQA
gi|493333551|ref|WP_006290636.1|helicase[Parascardoviadenticolens]_gi|315119487|gb|EFT82620.1|helicaseCterminaldomainprotein[P/	--------------------ENECRILTNARCLAEGVDVPSLDAVIFFAPKKSEVDVVQA
gi|495817067|ref|WP_008541646.1|helicase,partial[Sutterellaparvirubra]_gi|378598583|gb|EHY31712.1|helicaseprotein,partial[Sutte	--------------------KNHCKILFNVRCLSEGVDVPSLDAVIFLSPRKSVVDVVQT
gi|491572696|ref|WP_005430278.1|helicase[Sutterellawadsworthensis]_gi|319805116|gb|EFW01943.1|DNAhelicaserestrictionenzymeTypeI	--------------------ENVCKILFNVRCLSEGVDVPSLDAVLFLSPRKSQVEVVQT
gi|748686321|ref|WP_039944445.1|hypotheticalprotein[Thermicanusaegyptius]/11573	--------------------EGEVRVLSNARVLTEGIDVPSLDAVVFLKPRKSQVDVVQA
gi|916875698|ref|WP_051482754.1|hypotheticalprotein[Thioalkalivibriosp.HK1]/11366	---------------------GECHILSNARCLSEGVDVPALDAVLFMAERKSTVDVVQA
gi|821248604|gb|KKZ10960.1|hypotheticalproteinTE42_09075[CandidatusSynechococcusspongiarumSP3]/11598	---------------------GEIRILTNARCLTEGVDVPALDGILFMHPRKSQIEVVQA
gi|737409843|ref|WP_035390956.1|hypotheticalprotein[Ferrimicrobiumacidiphilum]_gi|765544266|gb|KJE76241.1|typeIrestrictionenzym	---------------------EECRLLTNAKCLSEGVDVPSLDAVMFLQPRKSQVDVVQA
gi|654906925|ref|WP_028358281.1|damageinducibleprotein[Brackiellaoedipodis]/11679	I------------------PDNECRILTNVRCLSEGVDIPALDAVMFMTPRNSLVDVVQS
gi|502809901|ref|WP_013044877.1|hypotheticalprotein[CandidatusPuniceispirillummarinum]_gi|292663146|gb|ADE38247.1|hypotheticalp	N-----------------EHGEICRILSNAKCLTEGVDVPALDAAIFMNPRKSQVDVVQA
gi|251771370|gb|EES51950.1|typeIIrestrictionenzyme,ressubunit[Leptospirillumferrodiazotrophum]/11628	-------------------ASAECRVLTNARCLSEGVDVPSLDAVLFFDARDSMVDVVQS
gi|498328480|ref|WP_010642636.1|DNAdamageinducibleprotein[Acidithiobacillusthiooxidans]/11627	--------------------FGSCHLLTNARCLSEGVDVPALDAVIFFDARDSMVDIVQS
gi|647633864|ref|WP_025906698.1|damageinducibleprotein[Prochlorococcussp.scB245a_519B7]/11622	--------------------PNNCRVLSNARCLSEGIDVPSLDAVMFLHPRKSQIDVVQS
gi|647619599|ref|WP_025893729.1|damageinducibleprotein[Prochlorococcussp.scB241_527N11]/11621	--------------------PNICRVLSNARCLSEGVDVPSLDAIMFLHPRRSQIDVVQS
gi|755034887|ref|WP_042387498.1|hypotheticalprotein[Clostridiumsp.CL6]/11606	--------------------ENTCRILTNARCLSEGVNVPALDAVMFLNPRSSMVDIIQS
gi|498139798|ref|WP_010453954.1|helicase[SuccinivibrionaceaebacteriumWG1]/11662	-------------------TPNECRILTNVRCLSEGVDVPALDAVVFLSPKNSKVDVVQS
gi|527501386|gb|EPT34571.1|typeIIIrestrictionenzyme,ressubunit[Bacteroidetesbacteriumoraltaxon272str.F0290]/11789	-------------------ADGQCRILSNVRCLSEGVDVPALDAVIFASARDSQVDVVQS
gi|496047275|ref|WP_008771782.1|helicase[Bacteroidessp.2_1_33B]_gi|262296443|gb|EEY84373.1|helicaseCterminaldomainprotein[Bact/	-------------------NPHECRILSNVRCLSEGVDVPALDAVLFVAARNSEVDVVQS
PDI8503III/11664	-------------------NPNECRILSNVRCLSEGVDVPALDAVLFVAARNSEVDVVQS
gi|738719431|ref|WP_036613715.1|helicase[Parabacteroidesdistasonis]_gi|649542518|gb|KDS49387.1|restrictionendonucleasefamilypro	-------------------NPNECRILSNVRCLSEGVDVPALDAVLFVAARNSEVDVVQS
gi|659417033|gb|KEJ86133.1|hypotheticalproteinHMPREF1002_00341[Porphyromonassp.31_2]/11656	-------------------NPNECRILSNVRCLSEGVDVPALDAVLFVAARNSEVDVVQS
gi|567456807|gb|ETK11358.1|hypotheticalproteinT235_15845[Tannerellasp.oraltaxonBU063isolateCell8/11]/11433	-------------------DPQECRVVSNVRCLSEGVDVPALDAVLFLSARNSQVDVVQS
gi|545440279|ref|WP_021677877.1|helicase[Porphyromonasgingivalis]_gi|543985765|gb|ERJ89108.1|helicaseprotein[Porphyromonasgingi	-------------------DPQECRVVTNVRCLSEGVDVPALDAVLFLSARNSQVDVVQS
gi|497438056|ref|WP_009752254.1|helicase[Prevotellasp.oraltaxon473]_gi|429144866|gb|EKX87968.1|helicaseprotein[Prevotellasp.ora	-------------------DPQECRVVTNVRCLSEGVDVPALDAVLFLSARNSQVDVVQS
gi|490423474|ref|WP_004295683.1|MULTISPECIES:helicase[Bacteroides]_gi|156110098|gb|EDO11843.1|helicaseCterminaldomainprotein[B/	-------------------GENECRVLTNVRCLSEGIDVPALDAVLFLSSRNSQVDVVQS
gi|941777229|ref|WP_055173506.1|helicase[Bacteroidescaccae]_gi|933263395|emb|CUQ00567.1|Predictedhelicase[Bacteroidescaccae]/1/	-------------------NERECRVVTNVRCLSEGVDVPSLDAVLFLSARNSQVDVVQS
gi|492413898|ref|WP_005836892.1|helicase[Bacteroidesuniformis]_gi|392678824|gb|EIY72229.1|hypotheticalproteinHMPREF1072_03583[B	-------------------NERECRVVTNVRCLSEGVDVPSLDAVLFLSARNSQVDVVQS
gi|695455095|ref|WP_032566191.1|helicase[Bacteroidesfragilis]_gi|596095426|gb|EXZ59996.1|restrictionendonucleasefamilyprotein[B	-------------------NERECRIVTNVRCLSEGVDVPSLDAVLFLSARNSQVDVVQS
gi|933227941|emb|CUQ02348.1|Predictedhelicase[Bacteroidesuniformis]/11658	-------------------NERECRVVTNVRCLSEGVDVPSLDAVLFLSARNSQVDVVQS
gi|494823013|ref|WP_007558421.1|helicase[Bacteroidesplebeius]_gi|198272167|gb|EDY96436.1|helicaseCterminaldomainprotein[Bacter/	-------------------NERECRIVTNVRCLSEGVDVPSLDAVLFLSARNSQVDVVQS

```
gi|495427212|ref|WP_008151909.1|helicase[Parabacteroidesjohnsonii]_gi|218222381|gb|EEC95031.1|helicaseCterminaldomainprotein[P/      -------------------NDRECRVVTNVRCLSEGVDVPSLDAVLFLSARNSQVDVVQS
gi|933196855|emb|CUO94545.1|typeIrestrictionenzymeEcoKIsubunitR[Bacteroidesuniformis]_gi|933220098|emb|CUN86874.1|typeIrestrict      -------------------NDRECRVVTNVRCLSEGVDVPSLDAVLFLSARNSQVDVVQS
gi|823687748|ref|WP_047101121.1|helicase[Brachyspirahyodysenteriae]_gi|823334537|gb|KLI17932.1|helicase[Brachyspirahyodysenteri      A-----------------EKENECKILTNVKCLSEGVDVPSLDAVLFLSAKNSQVDVVQS
gi|496436691|ref|WP_009145538.1|helicase[Phascolarctobacteriumsuccinatutens]_gi|322414007|gb|EFY04839.1|helicaseCterminaldomai/      -------------------DGNECRILTNVRCLSEGVDVPSLDAVIFLSKRNSQVDVVQS
gi|503372880|ref|WP_013607541.1|helicase[Sphaerochaetaglobosa]_gi|324026933|gb|ADY13692.1|typeIIIrestrictionproteinressubunit[S      -------------------EGSECQILMNVRCLSEGVDVPTLDAILFLSARNSQIDVVQS
gi|505821748|ref|WP_015707145.1|helicase[Treponemaprimitia]_gi|299483503|gb|ADJ19584.1|helicasedomaincontainingprotein[Trepone/      -------------------GKQDCHILNNVRCLSEGVDVPSLDAVMFLSARNSQIDVVQS
gi|505822741|ref|WP_015708134.1|helicase[Treponemaprimitia]_gi|333739639|gb|AEF85129.1|endonucleaseandmethylaseLlaGI[Treponemap      -------------------GKQDCHILNNVRCLSEGVDVPSLDAVMFLSARNSQIDVVQS
gi|488791994|ref|WP_002704400.1|helicase[Treponemasaccharophilum]_gi|380767691|gb|EIC01689.1|typeIIIrestrictionproteinressubuni      -------------------GKKECRILNNVRCLSEGVDVPSLDAILFLSSRNSEVDVVQS
gi|524671454|emb|CDD81750.1|putativeseptumsitedeterminingproteinMinC[Bacteroidessp.CAG:462]/11642      -------------------NDMECRMLTNARCLSEGVDVPSLDAVVFVSPKNSQVDVVQS
gi|524573865|emb|CDC95766.1|putativeseptumsitedeterminingproteinMinC[Alistipessp.CAG:268]/11639      -------------------NERECRMLTNARCLSEGVDVPSLDAVVFISPKNSQVDVVQS
gi|658617970|ref|WP_029759328.1|helicase[Fusobacteriumnucleatum]/11606      -------------------E-KECRILTNARCLSEGVDVPNLDAVLFLSAKNSQVDVVQS
gi|494412037|ref|WP_007216782.1|helicase[Bacteroidescellulosilyticus]_gi|392638921|gb|EIY32752.1|hypotheticalproteinHMPREF1062_      -------------------DGRECRILCNVRCLSEGVDVPSLDAVLFLSARNSQVDVVQS
gi|383107102|gb|AFG37435.1|putativehelicase[SpirochaetaafricanaDSM8902]/11616      -------------------DSSDCRILTNVRCLSEGVDVPSLDAVLFLSARNSHVDVVQS
SAF8902III/11616      -------------------DSSDCRILTNVRCLSEGVDVPSLDAVLFLSARNSHVDVVQS
gi|652538894|ref|WP_026932796.1|helicase[Gramellaechinicola]/11633      -------------------GTNESRILTNVRCLSEGVDVPSLDAVMFLSARNSQVDVVQS
gi|639238223|ref|WP_024568839.1|helicase[Elizabethkingiaanophelis]/11628      -------------------EPNECRILTNVRCLSEGVDVPSLDAVLFLSARNSQVDVVQS
gi|652520381|ref|WP_026914582.1|helicase[Gramellaportivictoriae]/11623      -------------------DSNECRVLTNVRCLSEGVDVPSLDAVLFLSARNSEVDVVQS
gi|494922183|ref|WP_007648219.1|Helicase[Formosasp.AK20]_gi|469825489|gb|EMQ95550.1|Helicasedomainprotein[Formosasp.AK20]/11626      -------------------DSNECRILTNVRCLSEGVDVPSLDAVLFLSARNSQVDVVQS
gi|522083382|ref|WP_020594591.1|hypotheticalprotein[Spirosomapanaciterrae]/11637      -------------------E-NECRILTNVRCLSEGVDVPSLDAVLFLSARNSQVDVVQS
gi|490455352|ref|WP_004326157.1|helicase[Bacteroidesovatus]_gi|392669277|gb|EIY62768.1|hypotheticalproteinHMPREF1070_03635[Bact      -------------------DEKECRVLTNVRCLSEGVDVPSLDAVLFLSARNSQVDVVQS
gi|499516035|ref|WP_011202675.1|helicase[Bacteroidesfragilis]_gi|53713270|ref|YP_099262.1|helicasedomainprotein[Bacteroidesfrag      -------------------DEKECRVLTNVRCLSEGVDVPSLDAVLFLSARNSQVDVVQS
gi|651338332|ref|WP_026461320.1|helicase[Adhaeribacteraquaticus]/11620      -------------------D-NECRVLTNVRCLSEGVDVPSLDAVMFLSARNSQVDVVQS
gi|806964642|gb|AKC17139.1|helicase[Flavobacteriumpsychrophilum]/11639      -------------------Q-NACRVLTNVRCLSEGVDVPSLDAVLFLSARNSQVDVVQS
gi|752684374|ref|WP_041332322.1|helicase[Flavobacteriumpsychrophilum]_gi|666882889|gb|AIG30715.1|helicase[Flavobacteriumpsychro      -------------------Q-NACRVLTNVRCLSEGVDVPSLDAVLFLSARNSQVDVVQS
gi|933196595|emb|CUP04894.1|DNAorRNAhelicasesofsuperfamilyII[Bacteroidesuniformis]/11631      -------------------D-GECRVLTNVRCLSEGVDVPALDAVMFLSARNSQVDVVQS
gi|670509109|ref|WP_031456918.1|helicase[Flavobacteriumchungangense]/11625      -------------------E-GDCRILTNVRCLSEGVDVPSLDAVLFLSARNSQVDVVQS
                                                  :: *.: *:**:::* **   :   : :  :: *:

gi|490190857|ref|WP_004089451.1|hypotheticalprotein[Helicobacterbilis]_gi|476630638|gb|EMZ37196.1|hypotheticalproteinC826_02193      VGRVMRKA------P--NK--EVGYIILPVSLDETE--------L---KNLDSAV--NNT
gi|488896133|ref|WP_002807242.1|MULTISPECIES:restrictionendonucleaseEco57I[Campylobacter]_gi|380541096|gb|EIA65377.1|hypothetic      VGRVMRKA------P--NK--KTGYIILPLALSENE--------L---SNLDEAI--KNT
gi|746602240|ref|WP_039628365.1|restrictionendonucleaseEco57I[Campylobacterlari]_gi|744819395|gb|AJD03391.1|putativehelicase[Ca      VGRVMRKA------P--NK--KTGYIILPLALSENE--------L---SNLDEAI--KNT
gi|727317135|ref|WP_033772558.1|restrictionendonucleaseEco57I[Helicobacterpylori]/11622      VGRVMRKA------K--GK--KRGYIILPIALEESE--------I---KNLNKAV--NNT
gi|727103490|ref|WP_033618954.1|restrictionendonucleaseEco57I[Helicobacterpylori]/11616      VGRVMRKA------K--GK--KRGYIILPIALEESE--------I---KNLNEAV--NNT
gi|727340205|ref|WP_033794408.1|restrictionendonucleaseEco57I[Helicobacterpylori]/11609      VGRVMRKA------K--GK--KRGYIILPIALEESE--------I---KNLNEAI--NNT
gi|727292407|ref|WP_033749975.1|restrictionendonucleaseEco57I[Helicobacterpylori]/11611      VGRVMRKA------K--GK--KRGYIILPIALEESE--------I---KNLNEAI--NNT
gi|727289795|ref|WP_033747591.1|restrictionendonucleaseEco57I[Helicobacterpylori]/11612      VGRVMRKA------K--HK--KRGYIILPIALEESE--------I---KNLDEAV--KNT
gi|727319676|ref|WP_033775047.1|restrictionendonucleaseEco57I[Helicobacterpylori]/11612      VGRVMRKA------K--HK--KRGYIILPIALEESE--------I---KNLDEAV--KNT
gi|447212910|ref|WP_001290166.1|restrictionendonucleaseEco57I[Helicobacterpylori]_gi|308063540|gb|ADO05427.1|hypotheticalprotei      VGRVMRKA------K--HK--KRGYIILPIALEESE--------I---QNLDEAV--NNT
gi|447212901|ref|WP_001290157.1|restrictionendonucleaseEco57I[Helicobacterpylori]_gi|384557329|gb|AFH97797.1|hypotheticalprotei      VGRVMRKA------K--HK--KRGYIILPIALEESE--------I---QNLDEAV--NNT
gi|515849671|ref|WP_017280424.1|hypotheticalprotein[Helicobacterpylori]/11615      VGRVMRKA------K--HK--KRGYIILPIALEESE--------I---QNLDEAV--NNT
gi|447212900|ref|WP_001290156.1|restrictionendonucleaseEco57I[Helicobacterpylori]_gi|317178970|dbj|BAJ56758.1|TypeIIGrestrictio      VGRVMRKA------K--HK--KRGYIILPIALEESE--------I---QNLDEAV--NNT
gi|447079018|ref|WP_001156274.1|hypotheticalprotein[Helicobacterpylori]/11614      VGRVMRKA------K--RK--QRGYIILPIALEESE--------I---KNLDEAV--NNT
gi|447212800|ref|WP_001290056.1|hypotheticalprotein[Helicobacterpylori]_gi|532153248|gb|EQL67316.1|restrictionendonucleaseEco57      VGRVMRKA------K--RK--QRGYIILPIALEESE--------I---QNLDEAV--NNT
gi|505457237|ref|WP_015644339.1|hypotheticalprotein[Helicobacterpylori]_gi|499061160|gb|AGL70074.1|restrictionendonucleaseEco57      VGRVMRKA------K--RK--QRGYIILPIALEEHE--------I---QNLDEAV--NNT
HPYUM037X/11591      VGRVMRKA------K--RK--QRGYIILPIALEEHE--------I---QNLDEAV--NNT
gi|920026297|ref|WP_052937176.1|restrictionendonuclease[Helicobacterpylori]_gi|912528678|gb|KNX48238.1|restrictionendonucleaseE      VGRVMRKA------K--RK--KRGYIILPIALEESE--------I---KNLDEAV--NNT
gi|915539050|ref|WP_050841327.1|restrictionendonuclease[Helicobacterpylori]_gi|908890887|gb|KNE06183.1|restrictionendonucleaseE      VGRVMRKA------K--NK--KRGYIILPIALEESE--------I---KNLDEAV--NNT
gi|727101397|ref|WP_033616957.1|restrictionendonucleaseEco57I[Helicobacterpylori]/11592      VGRVMRKA------K--NK--KRGYIILPIALEESE--------I---KNLDEAV--NNT
gi|562814875|emb|CDK13002.1|TypeISPRMsystem[Helicobacterpylori26695]/11588      VGRVMRKA------K--RK--KRGYIILPIALEESE--------I---QNLDEAV--NNT
HPYAXVIII/11588      VGRVMRKA------K--RK--KRGYIILPIALEESE--------I---QNLDEAV--NNT
gi|447079029|ref|WP_001156285.1|restrictionendonucleaseEco57I[Helicobacterpylori]_gi|393085956|gb|EJB86635.1|hypotheticalprotei      VGRVMRKA------K--RK--KRGYIILPIALEESE--------I---KNLDEAV--NNT
gi|489105953|ref|WP_003015814.1|restrictionendonuclease[Helicobacterpylori]_gi|477503388|gb|ENH59950.1|restrictionendonucleasef      VGRVMRKA------K--RK--KRGYIILPIALEESE--------I---QNLDEAV--NNT
gi|447079006|ref|WP_001156262.1|restrictionendonucleaseEco57I[Helicobacterpylori]_gi|393067470|gb|EJB68280.1|hypotheticalprotei      VGRVMRKS------K--RK--KRGYIILPIALEESE--------I---KNLDEAV--NNT
gi|727314589|ref|WP_033770298.1|restrictionendonucleaseEco57I[Helicobacterpylori]/11586      VGRVMRKA------K--NK--KRGYIILPIALEESE--------I---KNLDEAV--NNT
gi|447079019|ref|WP_001156275.1|restrictionendonucleaseEco57I[Helicobacterpylori]_gi|308065028|gb|ADO06920.1|hypotheticalprotei      VGRVMRKA------K--NK--KRGYIILPIALRESE--------I---KNLDEAV--NNT
gi|447079028|ref|WP_001156284.1|restrictionendonucleaseEco57I[Helicobacterpylori]_gi|393076030|gb|EJB76784.1|hypotheticalprotei      VGRVMRKA------K--NK--KRGYIILPIALKESE--------I---KNLDEAV--NNT
gi|393034009|gb|EJB35070.1|hypotheticalproteinHPNQ4053_0631[HelicobacterpyloriNQ4053]/11514      VGRVMRKA------K--HK--KRGYIILPIALEESE--------I---KNLDEAV--NNT
gi|447079004|ref|WP_001156260.1|restrictionendonucleaseEco57I[Helicobacterpylori]_gi|384552418|gb|AFI07366.1|hypotheticalprotei      VGRVMRKA------K--NK--KRGYIILPIALEESE--------I---KNLDEAV--KNT
gi|746011306|ref|WP_039088215.1|restrictionendonucleaseEco57I[Helicobacterpylori]_gi|733488720|gb|KHL85979.1|restrictionendonuc      VGRVMRKA------K--HK--KRGYIILPIALEESE--------I---KNLDEAV--NNT
gi|531081162|gb|EQD92166.1|restrictionendonucleaseEco57I[HelicobacterpyloriPZ5080]/11509      VGRVMRKA------K--NK--KRGYIILPIALEESE--------I---KNLDEAV--NNT
gi|544760796|ref|WP_021187395.1|restrictionendonucleaseEco57I[Helicobacterpylori]_gi|531086508|gb|EQD96745.1|restrictionendonuc      VGRVMRKA------K--HK--KRGYIILPIALEESE--------I---KNLDEAV--NNT
gi|447079012|ref|WP_001156268.1|restrictionendonucleaseEco57I[Helicobacterpylori]_gi|393028199|gb|EJB29286.1|hypotheticalprotei      VGRVMRKA------K--NK--KRGYIILPIALEESE--------I---KNLDEAV--NNT
gi|746008458|ref|WP_039085555.1|restrictionendonucleaseEco57I[Helicobacterpylori]_gi|733480358|gb|KHL78026.1|restrictionendonuc      VGRVMRKA------K--HK--KRGYIILPIALEESE--------I---KNLDEAV--NNT
gi|447079011|ref|WP_001156267.1|restrictionendonucleaseEco57I[Helicobacterpylori]_gi|393038534|gb|EJB39568.1|hypotheticalprotei      VGRVMRKA------K--NK--KRGYIILPIALEESE--------I---KNLDEAV--NNT
gi|393037163|gb|EJB38199.1|hypotheticalproteinHPNQ4161_0422[HelicobacterpyloriNQ4161]/11556      VGRVMRKA------K--NK--KRGYIILPIALRESE--------I---KNLDEAV--KNT
gi|447079007|ref|WP_001156263.1|restrictionendonucleaseEco57I[Helicobacterpylori]_gi|393051817|gb|EJB52768.1|hypotheticalprotei      VGRVMRKA------K--RK--KRGYIILPIALEESE--------I---NNLDEAV--KNT
gi|487867094|ref|WP_001940560.1|helicase[Helicobacterpylori]_gi|459505657|gb|EMG98418.1|helicaseprotein[HelicobacterpyloriGAM21      VGRVMRKA------K--NK--KRGYIILPIALRESE--------I---KNLDEAV--KNT
```

```
gi|487872094|ref|WP_001945560.1|helicase[Helicobacterpylori]_gi|459512104|gb|EMH04523.1|helicaseprotein[HelicobacterpyloriGAM24      VGRVMRKA------K--NK--KRGYIILPIALKESE--------I---KNLDEAV--KNT
gi|487880228|ref|WP_001953694.1|helicase[Helicobacterpylori]_gi|459529131|gb|EMH20734.1|helicaseprotein[HelicobacterpyloriGAM26      VGRVMRKA------K--NK--KRGYIILPIALRESE--------I---KNLDEAV--KNT
gi|487882593|ref|WP_001956059.1|helicase[Helicobacterpylori]_gi|459533462|gb|EMH24859.1|helicaseprotein[HelicobacterpyloriGAM26      VGRVMRKA------K--NK--KRGYIILPIALRESE--------I---QNLDEAV--KNT
gi|487845430|ref|WP_001918896.1|helicase[Helicobacterpylori]_gi|410713871|gb|EKQ71363.1|helicaseprotein[HelicobacterpyloriGAM10      VGRVMRKA------K--NK--KRGYIILPIALRESE--------I---KNLDEAV--KNT
gi|447079005|ref|WP_001156261.1|restrictionendonucleaseEco57I[Helicobacterpylori]_gi|393069830|gb|EJB70625.1|hypotheticalprotei      VGRVMRKA------K--NK--KRGYIILPIALRESE--------I---QNLDEAV--KNT
gi|459506808|gb|EMG99528.1|helicaseprotein[HelicobacterpyloriGAM121Aii]/11545                                                       VGRVMRKA------K--NK--KRGYIILPIALRESE--------I---KNLDEAV--KNT
gi|487825283|ref|WP_001898749.1|restrictionendonucleaseEco57I[Helicobacterpylori]_gi|393107526|gb|EJC08068.1|hypotheticalprotei      VGRVMRKA------K--NK--KRGYIILPIALRESE--------I---KNLDEAV--KNT
gi|447079008|ref|WP_001156264.1|restrictionendonucleaseEco57I[Helicobacterpylori]_gi|393058636|gb|EJB59524.1|hypotheticalprotei      VGRVMRKA------K--NK--KRGYIILPIALRESE--------I---KNLDEAV--KNT
gi|447079015|ref|WP_001156271.1|restrictionendonucleaseEco57I[Helicobacterpylori]_gi|307637357|gb|ADN79807.1|hypotheticalprotei      VGRVMRKA------K--NK--KRGYIILPIALRESE--------I---KNLDEAV--KNT
gi|487884861|ref|WP_001958327.1|helicase[Helicobacterpylori]_gi|459535535|gb|EMH26834.1|helicaseprotein[HelicobacterpyloriGAM26      VGRVMRKA------K--NK--KRGYIILPIALAENE--------I---KNLDEAV--KNT
gi|487886365|ref|WP_001959831.1|helicase[Helicobacterpylori]_gi|459538727|gb|EMH29861.1|helicaseprotein[HelicobacterpyloriGAM27      VGRVMRKA------K--NK--KRGYIILPIALRESE--------I---KNLDEAV--KNT
gi|545301637|ref|WP_021581412.1|helicase[Helicobacterpylori]_gi|535931889|gb|ERA61745.1|helicaseprotein[HelicobacterpyloriGAM11      VGRVMRKA------K--NK--KRGYIILPIALAENE--------I---KNLDEAV--KNT
gi|447079010|ref|WP_001156266.1|restrictionendonucleaseEco57I[Helicobacterpylori]_gi|393095477|gb|EJB96081.1|hypotheticalprotei      VGRVMRKA------K--NK--KRGYIILPIALRESE--------I---KNLDEAV--KNT
gi|447079009|ref|WP_001156265.1|restrictionendonucleaseEco57I[Helicobacterpylori]_gi|393056294|gb|EJB57206.1|hypotheticalprotei      VGRVMRKA------K--NK--KRGYIILPIALKESE--------I---KNLDEAV--KNT
gi|471386985|gb|EMR58906.1|restrictionendonucleasefamilyprotein[HelicobacterpyloriHpH1]/11504                                       VGRVMRKA------K--NK--KRGYIILPIALAENE--------I---KNLDEAV--KNT
gi|487875679|ref|WP_001949145.1|helicase[Helicobacterpylori]_gi|459516928|gb|EMH09091.1|helicaseprotein[HelicobacterpyloriGAM25      VGRVMRKA------K--NK--KRGYIILPIALKESE--------I---KNLDEAV--KNT
gi|496130066|ref|WP_008854573.1|DNAdamageinducibleprotein[Commensalibacterintestini]_gi|353671689|gb|EHD13391.1|helicasedomain/     VGRVMRKA------P--NK--QLGYVILPVVIPAGI--------E--A---DKAL-DNNQ
gi|924898894|ref|WP_053556546.1|damageinducibleprotein[Sphingopyxissp.113P3]_gi|924523996|gb|ALC14732.1|damageinducibleprotei/1     VGRVMRRA------P--GK--ELGYVILPVGIPAGV--------A--A---EDAL-NNNE
gi|780802950|gb|KJS08562.1|damageinducibleprotein[Hoefleasp.BRH_c9]/11633                                                          VGRVMRRA------P--GK--KMGYVILPVGIPAGV--------A--P---EEAL-NNNE
gi|944671432|gb|KQN22886.1|damageinducibleprotein[Sphingomonassp.Leaf32]/11415                                                      VGRVMRRA------P--GK--KMGYVILPVGIPAGV--------A--P---EEAL-NNNE
gi|944612953|gb|KQM64772.1|damageinducibleprotein[Sphingomonassp.Leaf16]/11726                                                      VGRVMRRA------P--GK--KMGYVILPVGIPAGV--------A--P---EEAL-NNNE
gi|764353851|ref|WP_044331468.1|damageinducibleprotein[Sphingomonashengshuiensis]_gi|761897338|gb|AJP71707.1|damageinduciblep/1     VGRVMRRA------T--GK--NMGYVILPIGVPAGV--------T--P---EEAL-NDNE
gi|945874472|gb|KQZ27973.1|damageinducibleprotein[Mesorhizobiumsp.Root552]/11651                                                   VGRVMRRA------P--GK--NMGYVILPIGVPAGM--------T--P---EEAL-NDNE
gi|759408081|ref|WP_043132403.1|damageinducibleprotein[Paracoccussanguinis]_gi|694217086|gb|KGJ14281.1|damageinducibleprotein/1     VGRVMRRA------E--GK--KMGYVILPVAIPPDT--------T--P---EDAL-KDNE
gi|654342671|ref|WP_027836116.1|damageinducibleprotein[Maritaleamyrionectae]/11632                                                 VGRVMRKA------E--GK--KLGYVILPVAIPPNV--------K--P---EEAL-NDNE
gi|126717687|gb|EBA14409.1|hypotheticalproteinRSK20926_02032[Roseobactersp.SK20926]/11603                                           VGRVMRRA------E--GK--KLGYVILPVAIPPNT--------K--P---EDAL-NDNE
gi|765301116|ref|WP_044628780.1|damageinducibleprotein[Halomonasmeridiana]_gi|764089679|gb|KJD19875.1|damageinducibleprotein[/1     VGRVMRRA------E--GK--KLGYVILPVAIPPNT--------K--P---EEAL-NDNE
gi|518053006|ref|WP_019223214.1|DNAdamageinducibleprotein[Bartonellarattaustraliani]/11654                                         VGRVMRRA------E--GK--KTGYIILPVVVPSYI--------D--A---KESV-KHNS
gi|493637806|ref|WP_006589578.1|DNAdamageinducibleprotein[Bartonellabirtlesii]_gi|395410345|gb|EJF76900.1|hypotheticalproteinM/    VGRVMRRA------P--NK--KTGYIILPIIIPSHL--------E--A---KKAL-RNNK
gi|501188603|ref|WP_012231622.1|DNAdamageinducibleprotein[Bartonellatribocorum]_gi|161017843|emb|CAK01401.1|helicase/methyltra/   VGRVMRRA------P--NK--KTGYIILPIIIPSHL--------E--A---KKAL-KNNK
gi|651388616|ref|WP_026500615.1|damageinducibleprotein[Bartonellagrahamii]/11655                                                   VGRVMRRA------P--NK--KTGYIILPIIIPSHL--------E--A---KKAL-KNNK
gi|501188669|ref|WP_012231687.1|DNAdamageinducibleprotein[Bartonellatribocorum]_gi|161017922|emb|CAK01480.1|helicase/methyltra/   VGRVMRRA------P--NK--KTGYIILPIIIPSHL--------E--A---KKAL-KNNK
gi|518048832|ref|WP_019219040.1|DNAdamageinducibleprotein[Bartonellaflorencae]/11655                                               VGRVMRRA------E--GK--TTGYIILPVGIPFNI--------P--P---QQAL-ENNT
gi|518053617|ref|WP_019223825.1|DNAdamageinducibleprotein[Bartonellarattaustraliani]/11607                                         VGRVMRRA------E--GK--TTGYIILPVGIPFNI--------P--P---QKAL-ANNT
gi|506336449|ref|WP_015856168.1|DNAdamageinducibleprotein[Bartonellagrahamii]_gi|240267492|gb|ACS51080.1|helicase/methyltransf/    VGRVMRRA------E--GK--TTGYIILPVGIPFNI--------P--P---QQAL-ENNT
gi|494588832|ref|WP_007347427.1|DNAdamageinducibleprotein[Bartonellarattimassiliensis]_gi|395419053|gb|EJF85361.1|hypothetical/   VGRVMRRA------E--GK--TTGYIILPVGIPFNI--------P--P---QQAL-ENNT
gi|501188650|ref|WP_012231668.1|DNAdamageinducibleprotein[Bartonellatribocorum]_gi|161017902|emb|CAK01460.1|helicase/methyltra/   VGRVMRRA------E--GK--TTGYIILPVGIPFNI--------P--P---QQAL-ENNT
gi|501188624|ref|WP_012231643.1|DNAdamageinducibleprotein[Bartonellatribocorum]_gi|161017866|emb|CAK01424.1|helicase/methyltra/   VGRVMRRA------P--GK--KRGYIILPVGVPAGI--------D--P---EKAL-QNNK
gi|518053856|ref|WP_019224064.1|DNAdamageinducibleprotein[Bartonellarattaustraliani]/11625                                         VGRVMRRA------P--GK--KRGYIILPVGVPAGI--------D--P---EKAL-KNNK
gi|501188053|ref|WP_012231081.1|DNAdamageinducibleprotein[Bartonellatribocorum]_gi|161017434|emb|CAK00992.1|helicase/methyltra/   VGRVMRRA------K--GK--KRGYIILPVGVPAGI--------A--P---EKAL-KNNP
gi|651388630|ref|WP_026500626.1|damageinducibleprotein[Bartonellagrahamii]/11657                                                   IGRVMRRA------E--GK--KRGYIILPVGIPADI--------P--P---EIAL-EKNQ
gi|518048855|ref|WP_019219063.1|DNAdamageinducibleprotein[Bartonellaflorencae]/11658                                               IGRVMRRA------E--GK--KRGYIILPIGIPANI--------P--P---EIAL-ENNQ
gi|490995101|ref|WP_004856829.1|DNAdamageinducibleprotein[Bartonelladoshiae]_gi|395413167|gb|EJF79645.1|hypotheticalproteinMCS/    IGRVMRRA------E--GK--KRGYIILPIGIPADI--------P--P---EIAL-ENNQ
gi|491002558|ref|WP_004864279.1|DNAdamageinducibleprotein[Bartonellavinsonii]_gi|395421392|gb|EJF87643.1|hypotheticalproteinME/    VGRIMRRA------K--GK--KRGYIILPVGVPAGI--------S--A---EQAL-RHNK
gi|491003373|ref|WP_004865094.1|DNAdamageinducibleprotein[Bartonellavinsonii]_gi|395432864|gb|EJF98838.1|hypotheticalproteinME/    VGRIMRRA------K--GK--KRGYIILPVGVPAGI--------S--A---EQAL-RHNK
gi|491002426|ref|WP_004864148.1|DNAdamageinducibleprotein[Bartonellavinsonii]_gi|395421767|gb|EJF88003.1|hypotheticalproteinME/    VGRIMRRA------K--GK--KRGYIILPVGVPAGI--------S--A---EQAL-RHNK
gi|491004024|ref|WP_004865745.1|DNAdamageinducibleprotein[Bartonellavinsonii]_gi|395432542|gb|EJF98522.1|hypotheticalproteinME/    VGRIMRRA------K--GK--KRGYIILPVGIPAGI--------S--A---EQAL-RHNK
gi|648396442|ref|WP_026088193.1|damageinducibleprotein[Bartonellarattaustraliani]/11593                                            VGRIMRRA------K--GK--KRGYIILPVGIPAGI--------S--A---ENAL-RYNE
gi|748190174|ref|WP_039761290.1|damageinducibleprotein[Bartonellaqueenslandensis]/11658                                            VGRIMRRA------K--GK--KRGYIILPVGIPAGI--------S--A---ENAL-RYNE
gi|494817599|ref|WP_007553007.1|DNAdamageinducibleprotein[Bartonellasp.DB56]_gi|395411312|gb|EJF77837.1|hypotheticalproteinMC/1    VGRVMRRA------K--GK--KMGYIILPVGVPAGV--------S--A---EQAL-KNNK
gi|515763376|ref|WP_017195976.1|DNAdamageinducibleprotein[Bartonellabirtlesii]/11656                                               VGRVMRRA------K--GK--KMGYIILPVGVPAGV--------S--P---EEAL-KNNK
gi|494589420|ref|WP_007348014.1|DNAdamageinducibleprotein[Bartonellarattimassiliensis]_gi|395416278|gb|EJF82667.1|hypothetical/   VGRIMRRA------K--GK--KRGYIILPVGIPAGV--------S--A---EQAL-KYNK
gi|506336469|ref|WP_015856188.1|DNAdamageinducibleprotein[Bartonellagrahamii]_gi|240267512|gb|ACS51100.1|helicase/methyltransf/    VGRIMRRA------K--GK--KRGYIILPVGIPAGV--------S--A---EHAL-RYNK
gi|749308087|ref|WP_040132227.1|damageinducibleprotein[Bartonellatribocorum]_gi|605051878|emb|CDO49988.1|helicase/methyltransf/    VGRIMRRA------R--GK--KRGYIILPVGVPAGV--------S--A---EHAL-RYNK
gi|515763800|ref|WP_017196400.1|DNAdamageinducibleprotein[Bartonellabirtlesii]/11658                                               VGRIMRRA------K--GK--KRGYIILPVGVPAGI--------S--A---EEAL-KYNK
gi|492186994|ref|WP_005773380.1|DNAdamageinducibleprotein[Bartonellaelizabethae]_gi|395418361|gb|EJF84688.1|hypotheticalprotei/   VGRIMRRS------P--GK--KRGYIILPVGIPAGV--------S--A---EQVL-KYNK
gi|501188615|ref|WP_012231634.1|DNAdamageinducibleprotein[Bartonellatribocorum]_gi|161017856|emb|CAK01414.1|helicase/methyltra/   VGRIMRRA------K--GK--KRGYIILPVGVPAGI--------S--A---EEAL-KYNK
gi|501187470|ref|WP_012230499.1|DNAdamageinducibleprotein[Bartonellatribocorum]_gi|161017090|emb|CAK00648.1|helicase/methyltra/   VGRIMRRA------K--GK--KRGYIILPVGVPAGI--------S--A---EEAL-KYNK
gi|501188636|ref|WP_012231655.1|DNAdamageinducibleprotein[Bartonellatribocorum]_gi|161017879|emb|CAK01437.1|helicase/methyltra/   VGRIMRRA------K--GK--KRGYIILPVGVPAGI--------S--A---EEAL-KYNK
gi|930151941|emb|CTQ34818.1|UvsWhelicase[Jannaschiarubra]/11641                                                                    VGRVMRQT------PDRSK--KMGYVILPVAIPAGT--------S--P---EEAL-NDNE
gi|928977106|ref|WP_054001461.1|damageinducibleprotein[Shimiasp.SK013]_gi|927698852|gb|KPA22698.1|typeIrestrictionenzymeEcoKIs/    VGRVMRRA------E--GK--KLGYVILPVVIPANV--------T--P---EAAL-NDNE
gi|738872465|ref|WP_036760884.1|damageinducibleprotein[Leisingeradaeponensis]/11643                                                VGRVMRRA------P--GK--RMGYVILPVGVPADK--------T--P---EEAL-ADND
gi|518050881|ref|WP_019221089.1|DNAdamageinducibleprotein[Bartonellasenegalensis]/11652                                            VGRVMRRS------E--GK--KMGYVILPIGIPSGI--------P--V---EQAL-NNNE
gi|493980596|ref|WP_006923523.1|DNAdamageinducibleprotein[Bartonellawashoensis]_gi|395413322|gb|EJF79796.1|hypotheticalprotein/   VGRVMRRS------E--GK--KMGYVILPIGIPSGI--------P--V---EQAL-NNND
gi|493983106|ref|WP_006925984.1|DNAdamageinducibleprotein[Bartonellawashoensis]_gi|395418127|gb|EJF84455.1|hypotheticalprotein/   VGRVMRRS------E--GK--KMGYVILPIGIPSGI--------P--V---EQAL-NNND
gi|499494671|ref|WP_011181311.1|DNAdamageinducibleprotein[Bartonellahenselae]_gi|49239008|emb|CAF28308.1|hypotheticalproteinBH/    VGRVMRRS------E--GK--KMGYVILPIGIPSGT--------P--V---EQAL-NNND
gi|651388426|ref|WP_026500441.1|damageinducibleprotein[Bartonellagrahamii]/11652                                                   VGRVMRRS------E--GK--KMGYVILPIGIPSGI--------P--V---EQAL-NNND
```

```
gi|506337280|ref|WP_015856999.1|DNAdamageinducibleprotein[Bartonellagrahamii]_gi|240268402|gb|ACS51990.1|helicase/methyltransf/    VGRVMRRS------E--GK--KMGYVILPIGIPSGI--------P--V---EQAL-NNND
gi|494817535|ref|WP_007552943.1|DNAdamageinducibleprotein[Bartonellasp.DB56]_gi|395412002|gb|EJF78518.1|hypotheticalproteinMC/1    VGRVMRRS------E--GK--KMGYVILPIGIPSRT--------P--V---EQAL-NNND
gi|493638099|ref|WP_006589864.1|DNAdamageinducibleprotein[Bartonellabirtlesii]_gi|395409809|gb|EJF76394.1|hypotheticalproteinM/    VGRVMRRS------E--GK--KMGYVILPIGIPSGT--------P--V---EQAL-NNND
gi|748189589|ref|WP_039760707.1|damageinducibleprotein[Bartonellaqueenslandensis]/11651    VGRVMRRS------E--GK--KMGYVILPIGIPSGI--------P--V---EQAL-NNND
gi|501189483|ref|WP_012232501.1|DNAdamageinducibleprotein[Bartonellatribocorum]_gi|161018902|emb|CAK02460.1|helicase/methyltra/    VGRVMRRS------E--GK--KMGYVILPIGVPSGI--------P--V---EQAL-NNND
gi|518049181|ref|WP_019219389.1|DNAdamageinducibleprotein[Bartonellaflorencae]/11652    VGRVMRRS------E--GK--KMGYVILPIGVPFDT--------P--A---EQAL-NNND
gi|492193255|ref|WP_005774528.1|DNAdamageinducibleprotein[Bartonellaelizabethae]_gi|395416403|gb|EJF82779.1|hypotheticalprotei/    VGRVMRRS------E--GK--KMGYVILPIGVPFDT--------P--A---EQAL-NNND
gi|498387325|ref|WP_010701288.1|helicase/methyltransferase[Bartonellabovis]_gi|478418724|gb|ENN92426.1|helicase/methyltransfera    VGRVMRRS------E--GK--KMGYIILPIGIPFGI--------P--V---KQAL-NNNE
gi|319408697|emb|CBI82352.1|conservedhypotheticalprotein[BartonellaschoenbuchensisR1]/11661    VGRVMRRA------E--GK--KMGYVILPVGIPYGI--------P--V---EQAL-NNNE
gi|494741726|ref|WP_007477134.1|DNAdamageinducibleprotein[Bartonellamelophagi]_gi|395423925|gb|EJF90113.1|hypotheticalproteinM/    VGRVMRRS------E--GK--KMGYVILPIGIPFGI--------P--V---EQAL-NNNE
gi|737261405|ref|WP_035245349.1|damageinducibleprotein[Alcanivoraxjadensis]_gi|686989709|gb|KGD62556.1|hypotheticalproteinT9A_/    VGRVMRKA------G-PSK--RMGYVILPIGVPHGV--------T--P---EEAL-NDNE
gi|916687564|ref|WP_051294655.1|damageinducibleprotein[Gemmobacternectariphilus]/11641    VGRVMRRA------D--NK--RMGYVILPVGVPAGV--------P--P---EQAL-ADNE
gi|740294127|ref|WP_038132495.1|damageinducibleprotein[Thioclavasp.DT234]_gi|660524826|gb|KEO54689.1|hypotheticalproteinDT23_/1    VGRVMRRA------E--GK--KMGYVILPVGVPAGV--------E--P---EVAL-KDND
gi|568331062|ref|WP_024099615.1|helicase[Phaeobactergallaeciensis]_gi|568232121|gb|AHD12007.1|putativehelicase[Phaeobactergalla    VGRVMRKT------D--TK--KMGYVILPVGVPAGV--------P--P---EQAL-ADNE
gi|518810459|ref|WP_019966413.1|DNAdamageinducibleprotein[Pannonibacterphragmitetus]/11635    VGRVMRRA------E--GK--KMGYVILPVGVPAGV--------P--P---EQAL-NDNE
gi|495305760|ref|WP_008030510.1|DNAdamageinducibleprotein[Rhodobactersp.SW2]_gi|259021698|gb|EEW25000.1|typeIIIrestrictionprot/    VGRVMRRA------D--GK--KMGYVILPVGVPAGV--------P--P---EQAL-ADNE
gi|506403303|ref|WP_015923022.1|DNAdamageinducibleprotein[Rhodobactersphaeroides]_gi|221162951|gb|ACM03922.1|HypotheticalProte/    VGRVMRRA------D--SK--KMGYVILPVGVPAGV--------P--P---ELAL-NDNE
gi|738818386|ref|WP_036708790.1|damageinducibleprotein[Paracoccussanguinis]_gi|694226786|gb|KGJ22541.1|damageinducibleprotein/1    VGRVMRRA------D--SK--KMGYVILPVGVPAGV--------P--P---EQAL-NDNE
gi|746096057|ref|WP_039162453.1|damageinducibleprotein[Gallibacteriumanatis]_gi|703617775|gb|KGQ63868.1|damageinducibleprotei/1    VGRVMRKA------E--GK--KRGYVILPVVIPANK--------T--P---EEAL-NDNE
gi|737716785|ref|WP_035685631.1|damageinducibleprotein[Avibacteriumparagallinarum]/11643    VGRVMRKA------E--GK--KRGYVILPVVIPANK--------T--P---EEAL-NDNE
gi|540072553|gb|ERF78672.1|hypotheticalproteinN561_04930[Gallibacteriumanatis12656/12}/11651    VGRVMRKA------E--GK--KRGYVILPVVIPANK--------T--P---EEAL-NDNE
gi|917509519|ref|WP_052115936.1|damageinducibleprotein[Gallibacteriumanatis]_gi|702414161|gb|KGQ42233.1|damageinducibleprotei/1    VGRVMRKA------E--GK--KRGYVILPVVIPANK--------T--P---EEAL-NDNE
gi|746082942|ref|WP_039150268.1|damageinducibleprotein[Gallibacteriumanatis]_gi|703617078|gb|KGQ63194.1|damageinducibleprotei/1    VGRVMRKA------E--GK--KRGYVILPVVIPANK--------T--P---EEAL-NDNE
gi|822522885|ref|WP_046952840.1|damageinducibleprotein[Haemophilushaemolyticus]_gi|821501762|gb|KKZ59267.1|damageinduciblepro/1    VGRVMRNA------P--GK--KRGYVILPVVIPAGI--------E--P---DKAL-EKNE
gi|404381014|gb|EJZ77501.1|putativehelicase[Pasteurellamultocidasubsp.gallicidaP1059]/11681    VGRVMRNS------E--GK--KRGYVILPVVIPAGL--------S--P---DEAL-DKNE
gi|298281660|gb|EFI23149.1|helicaseCterminaldomainprotein[Neisseriasp.oraltaxon014str.F0314]/11493    VGRVMRRA------E--GK--KQGYVILPVVIPPGI--------E--P---EAAL-DKNE
gi|348013055|gb|EGY51980.1|helicasedomainprotein[Neisseriashayeganii871]/11481    VGRVMRRA------E--GK--KQGYVILPVVIPAHV--------A--A---EEAL-DKNE
gi|496407140|ref|WP_009116004.1|DNAdamageinducibleprotein[Neisseriawadsworthii]_gi|349794756|gb|EGZ48566.1|helicasedomainprote/    VGRVMRRA------E--GK--KQGYVILPVVIPAGI--------E--A---EDAL-DKNE
gi|517216441|ref|WP_018405259.1|DNAdamageinducibleprotein[Marinobacterlipolyticus]/11656    VGRVMRLA------P--GK--ELGYVILPVVIPAGV--------E--P---EEAL-NRNE
gi|738230264|ref|WP_036185691.1|damageinducibleprotein[Marinobactersp.ELB17]/11671    VGRVMRLA------P--GK--ELGYVILPVVIPAGV--------E--P---EEAL-NRNE
gi|447197741|ref|WP_001274997.1|DNAdamageinducibleprotein[Acinetobacterbaumannii]_gi|408512324|gb|EKK13969.1|typeIIIrestrictio/    VGRVMRKA------P--NK--DLGYVILPVVIPAGV--------E--P---EQAL-NNNE
gi|820775897|ref|WP_046739909.1|damageinducibleprotein[Acinetobactersp.AG1]_gi|819909758|gb|KKW75938.1|damageinducibleprotein/1    VGRVMRKA------P--GK--QLGYVILPVVIPAGV--------E--P---EQAL-NSNE
gi|584595163|ref|WP_024160846.1|helicase[Acinetobacterpittii]_gi|576702530|emb|CDI28049.1|helicasedomaincontainingprotein[Acin/    VGRVMRKA------E--GK--QLGYVILPVVIPAGV--------E--P---EQAL-NSNE
gi|514963042|ref|WP_016651366.1|hypotheticalprotein[Acinetobactersp.NIPH2036]_gi|514162665|gb|EPG41391.1|hypotheticalproteinF90    VGRVMRKA------D--GK--QLGYVILPVVIPAGV--------E--P---EQAL-NSNE
gi|515965564|ref|WP_017396147.1|DNAdamageinducibleprotein[Acinetobacterhaemolyticus]/11667    VGRVMRKA------E--GK--QLGYVILPVVIPAGV--------E--P---EQAL-NSNE
gi|491346810|ref|WP_005204747.1|MULTISPECIES:hypotheticalprotein[Acinetobacter]_gi|480335823|gb|ENX56033.1|hypotheticalproteinF    VGRVMRKA------P--NK--ELGYVILPVVIPAGV--------E--P---EQAL-NNNE
gi|693044472|ref|WP_032215268.1|damageinducibleprotein[Escherichiacoli]_gi|607725731|gb|EZE08230.1|damageinducibleprotein[Esc/1    VGRVMRRA------E--GK--KLGYVILPVVIPAGE--------E--P---ENAL-NNNQ
gi|742382177|ref|WP_038861304.1|damageinducibleprotein[Cronobactermuytjensii]_gi|924333747|gb|ALB69766.1|damageinducibleprote/1    VGRVMRRA------E--GK--ELGYVILPVVIPAGE--------E--P---ENAL-NNNQ
gi|915912804|ref|WP_050927035.1|damageinducibleprotein[Yersiniaenterocolitica]_gi|899805802|emb|CQH32042.1|typeIrestrictionenz/    VGRVMRRA------E--GK--ELGYVILPVVIPPDE--------E--P---ENAL-NNNQ
gi|921111327|gb|KON62781.1|typeIrestrictionenzymeEcoKIsubunitR[Komagataeibactereuropaeus]/11492    VGRVMRVA------P--GK--KRGYVVLPVVIPAGV--------E--P---DQAL-DNNK
gi|582024278|gb|AHI27615.1|DNAhelicaserestrictionenzymeTypeIIIRsubunit[KomagataeibacterxylinusE25]/11636    VGRVMRVA------P--GK--KRGYVVLPVVIPVGV--------E--P---DQAL-DNNK
gi|347662673|dbj|BAK86128.1|DNAhelicaserestrictionenzymeTypeIIIRsubunit[KomagataeibactermedellinensisNBRC3288]/11737    VGRVMRVA------P--GK--KRGYVVLPVVIPAGV--------E--P---DQAL-DNNK
gi|917445053|ref|WP_052051471.1|damageinducibleprotein[Acetobactertropicalis]/11429    VGRVMRNA------P--GK--KRGYVVLPVVIPAGT--------P--P---EQSL-DNNQ
gi|453329445|dbj|GAC88472.1|DNAhelicaserestrictionenzymetypeIIIRsubunit[GluconobacterthailandicusNBRC3255]_gi|530287981|dbj|GAD    VGRVMRNA------P--GK--TRGYVVLPVVIPAGT--------P--P---EDSL-DNNQ
gi|889770922|ref|WP_048844278.1|damageinducibleprotein[Gluconobacterfrateurii]/11419    VGRVMRNA------P--GK--TRGYVVLPVVIPAGT--------P--P---EQSL-DNNQ
gi|504886208|ref|WP_015073310.1|hypotheticalprotein[Gluconobacteroxydans]_gi|411027954|gb|AFW01209.1|hypotheticalproteinB932_16    VGRVMRNA------P--GK--TRGYVVLPVVIPAGT--------S--P---EQSL-DNNQ
gi|480040211|gb|ENV79751.1|hypotheticalproteinF942_01641[AcinetobacterursingiiANC3649]/11432    VGRVMRLA------P--NK--KRGYIILPVVIPAGV--------E--P---HEAL-NDNK
gi|822498222|ref|WP_046936504.1|damageinducibleprotein[Xanthomonasgardneri]_gi|821751224|gb|KLB07223.1|damageinducibleprotein/1    VGRVMRTA------L--GK--KRGYVVLPVVIPSGV--------E--P---HLAL-NDNK
gi|654059559|ref|WP_027705556.1|damageinducibleprotein[Zymobacterpalmae]/11635    VGRVMRNA------P--GK--KRGYVVLPVVIPAGV--------E--P---NAAL-DDNK
gi|643535620|ref|WP_025224315.1|damageinducibleprotein[Methylophilaceaebacterium11]_gi|577704400|gb|EUJ10546.1|putativehelicas/    VGRVMRKS------E--GK--KRGYVVLPVVIPAGM--------E--A---HEAL-SDNK
MBA11I/11635    VGRVMRKS------E--GK--KRGYVVLPVVIPAGM--------E--A---HEAL-SDNK
gi|655355857|ref|WP_028762287.1|damageinducibleprotein[Shewanellacolwelliana]/11632    VGRVMRKS------E--GK--TRGYVILPVVVPTGM--------E--P---DEAL-NDNQ
gi|753204236|ref|WP_041510579.1|damageinducibleprotein[Shewanellasp.cp20]_gi|752457765|gb|KIO36326.1|damageinducibleprotein[S/1    VGRVMRNA------P--GK--VRGYVILPVVIPNGM--------S--P---NEAL-NDNQ
KPNNIH30III/11619    VGRVMRNA------P--GK--VRGYVILPVVIPTGM--------S--P---EAAL-DDNQ
gi|749295994|ref|WP_040120352.1|damageinducibleprotein[Klebsiellapneumoniae]_gi|723249300|gb|AIX81926.1|damageinducibleprotei/1    VGRVMRNA------P--GK--VRGYVILPVVIPTGM--------S--P---EAAL-DDNQ
gi|641639739|emb|CDQ54010.1|unnamedproteinproduct[Klebsiellapneumoniae]/11641    VGRVMRNA------P--GK--VRGYVILPVVIPTGM--------S--P---EAAL-DDNQ
gi|490797922|ref|WP_004660060.1|hypotheticalprotein[Acinetobactersp.NIPH236]_gi|479876779|gb|ENU27973.1|hypotheticalproteinF992    VGRVMRLA------P--NK--TMGYVILPVVIPPNV--------D--A---NKAL-DDNQ
gi|916750926|ref|WP_051357982.1|damageinducibleprotein[Acinetobactertjernbergiae]/11453    VGRVMRLA------E--GK--KRGYVILPVVIPPNV--------D--A---NKAL-DDNM
gi|691098662|ref|WP_032025060.1|damageinducibleprotein[Acinetobacterbaumannii]_gi|593567521|gb|EXR50735.1|restrictionendonucle/    VGRVMRLA------P--NK--KRGYVILPVVIPPGV--------D--P---NKAL-DDNQ
gi|736669405|ref|WP_034676049.1|damageinducibleprotein[Acinetobacterbaumannii]/11643    VGRVMRLA------P--NK--TRGYVILPVVIPPNV--------D--A---NKAL-DDNQ
gi|491368928|ref|WP_005226826.1|hypotheticalprotein[Acinetobactersp.NIPH1847]_gi|480173259|gb|ENX09797.1|hypotheticalproteinF89    VGRVMRLA------P--NK--KRGYVILPVVIPPGV--------E--P---NKAL-DDNL
gi|796853993|ref|WP_045795953.1|damageinducibleprotein[Acinetobacterindicus]_gi|787929761|gb|KJV44219.1|damageinducibleprotei/1    VGRVMRLA------P--NK--KRGYVILPVVIPPGV--------E--P---HKAL-DDNQ
gi|295797463|gb|ADG32253.1|typeIIIrestrictionproteinressubunit[ThiomonasintermediaK12]/11676    VGRVMRNA------P--GK--KRGYVILPVVIPAGV--------E--P---HEAL-NDNK
gi|499871179|ref|WP_011551913.1|DNAdamageinducibleprotein[Myxococcusxanthus]_gi|108467544|gb|ABF92729.1|putativerestriction/mo/    VGRVMRKP------ADSSK--KRGYIVLPVVIPAGV--------E--P---HDAL-NDNQ
gi|495313852|ref|WP_008038600.1|DNAdamageinducibleprotein[Bartonellatamiae]_gi|395424181|gb|EJF90368.1|hypotheticalproteinME5_/    VGRVMRKA------E--GK--TRGYIVLPVVIPSGV--------E--P---HEAL-NDNK
gi|501453335|ref|WP_012476784.1|DNAdamageinducibleprotein[Leptospirabiflexa]_gi|167777824|gb|ABZ96123.1|Helicase[Leptospirabif/    VGRVMRNA------P--GK--KRGYVILPVVIPAGI--------E--P---HEAL-NDNK
gi|738104613|ref|WP_036063193.1|damageinducibleprotein[Leptospiranoguchii]/11652    VGRVMRMA------P--NK--KRGYVILPVVIPAGV--------E--P---HEAL-NDNK
```

```
gi|410001039|gb|EKO51661.1|typeIIIrestrictionenzyme,ressubunit[Leptospirakirschneristr.200802841]/11651    VGRVMRMA------P--NK--KRGYVILPIVIPAGV--------E--P---HEAL-NDNK
gi|490901851|ref|WP_004763774.1|typeIIIrestrictionenzyme,ressubunit[Leptospirakirschneri]_gi|464209249|gb|EMN24014.1|typeIIIres    VGRVMRMA------P--NK--KRGYVILPIVIPAGV--------E--P---HEAL-NDNK
gi|464398131|gb|EMO65902.1|typeIIIrestrictionenzyme,ressubunit[Leptospirakirschneristr.200803703]/11648    VGRVMRMA------P--NK--KRGYVILPIVIPAGV--------E--P---HEAL-NDNK
gi|945029024|gb|KQQ84900.1|damageinducibleprotein[Xanthomonassp.Leaf131]/11423    VGRVMRNA------P--GK--KRGYVVLPVVIPAGV--------E--P---HEAL-NDNK
gi|896511314|ref|WP_049418183.1|damageinducibleprotein[Stenotrophomonasmaltophilia]/11639    VGRVMRNA------P--GK--RRGYVVLPVVIPAGV--------E--P---ERAL-DDNK
gi|896513082|ref|WP_049419616.1|damageinducibleprotein[Stenotrophomonasmaltophilia]/11638    VGRVMRNA------P--GK--KRGYVVLPVVIPAGV--------E--P---HQAL-DDNK
gi|896580616|ref|WP_049469425.1|damageinducibleprotein[Stenotrophomonasmaltophilia]/11641    VGRVMRNA------P--GK--QRGYVVLPVVIPAGI--------E--P---HEAL-NDNR
gi|648289350|ref|WP_026070499.1|damageinducibleprotein[Stenotrophomonasmaltophilia]/11639    VGRVMRNA------P--GK--QRGYVVLPVVIPAGI--------E--P---HEAL-NDNR
gi|656272786|ref|WP_029218442.1|damageinducibleprotein[Xanthomonascassavae]/11639    VGRVMRNA------P--GK--QRGYVVLPVVIPAGI--------E--P---HEAL-NDNR
gi|406976236|gb|EKD98747.1|hypotheticalproteinACD_23C00290G0002[unculturedbacterium]/11679    VGRVMRNA------P--GK--KRGYVVLPVVIPAGV--------E--P---HEAL-NDNQ
gi|828145737|ref|WP_047238120.1|damageinducibleprotein[Chromobacteriumsubtsugae]/11645    VGRVMRLA------E--GK--KRGYVILPVVIPAGM--------A--P---DEAL-NDNK
gi|654311174|ref|WP_027824327.1|damageinducibleprotein[Laribacterhongkongensis]/11641    VGRVMRLA------E--GK--KRGYVILPVVIPSGM--------A--P---DEAL-NDNK
gi|757242173|gb|KIR15516.1|typeIrestrictionenzymeEcoKIsubunitR[Pseudomonasfluorescens]/11778    VGRVMRKA------E--GK--KRGYVILPVVIPSGM--------A--P---EEAL-NDNK
gi|653605161|ref|WP_027616682.1|damageinducibleprotein[Pseudomonassp.URHB0015]/11639    VGRVMRKA------E--GK--KRGYVILPVVIPSNM--------A--P---EEAL-NDNK
gi|520868025|ref|WP_020308060.1|helicase[Pseudomonassyringae]_gi|519159034|gb|EPM49935.1|helicasedomaincontainingprotein[Pseud/    VGRVMRKA------E--GK--KRGYVILPVVIPSGM--------A--P---DEAL-NDNK
gi|940156567|gb|KPX71668.1|Helicasedomaincontainingprotein[Pseudomonasamygdalipv.lachrymans]/11677    VGRVMRKA------E--GK--KRGYVILPVVIPAGM--------A--P---DEAL-NDNK
gi|505460508|ref|WP_015647610.1|helicase[Pseudomonassp.GLE121]_gi|499110212|ref|YP_007974374.1|helicasedomaincontainingprotein/    VGRVMRNA------P--GK--KRGYVVLPVVIPAGM--------E--P---HEAL-NDNQ
gi|940235551|gb|KPY45311.1|hypotheticalproteinALO48_200154[Pseudomonassyringaepv.rhaphiolepidis]/11631    VGRVMRNA------P--GK--KRGYVVLPVVIPAGV--------E--A---HEAL-NDNQ
gi|520880808|ref|WP_020312235.1|helicase[Pseudomonassyringae]_gi|519172158|gb|EPM62027.1|helicasedomaincontainingprotein[Pseud/    VGRVMRNA------P--GK--KRGYVVLPVVIPAGM--------E--P---HEAL-NDNQ
gi|829046479|ref|WP_047282259.1|damageinducibleprotein[Pseudomonasfluorescens]/11630    VGRVMRNA------P--GK--KRGYVVLPVVIPAGM--------E--P---HEAL-NDNQ
gi|499415493|ref|WP_011102960.1|DNAdamageinducibleprotein[Pseudomonassyringaegroupgenomosp.3]_gi|28867279|ref|NP_789898.1|heli/    VGRVMRNA------P--GK--RRGYVVLPVVIPAGM--------E--P---HEAL-NDNQ
gi|730238299|ref|WP_033942897.1|damageinducibleprotein[Pseudomonasaeruginosa]/11629    VGRVMRNA------P--GK--KRGYVVLPVVIPAGM--------E--P---HEAL-NDNQ
gi|495310125|ref|WP_008034873.1|DNAdamageinducibleprotein[Pseudomonassp.GM60]_gi|398186685|gb|EJM74052.1|putativehelicase[Pseu/    VGRVMRNA------P--GK--KRGYVVLPVVIPAGM--------E--P---HEAL-NDNQ
gi|779992653|ref|WP_045428690.1|damageinducibleprotein[Pseudomonassp.MT1]_gi|699967872|dbj|BAP80334.1|DNAhelicaserestrictione/1    VGRVMRNA------P--GK--KRGYVVLPVVIPARM--------E--A---HEAL-NDNQ
gi|786037289|ref|WP_045733274.1|damageinducibleprotein[Pseudomonaspseudoalcaligenes]_gi|782987904|gb|KJU79152.1|damageinducib/1    VGRVMRNA------P--GK--KRGYVVLPVVIPAGM--------E--P---HEAL-NDNQ
gi|749666366|ref|WP_040265445.1|damageinducibleprotein[Pseudomonasrhodesiae]/11632    VGRVMRNA------P--GK--KRGYVVLPVVIPANT--------E--P---HVAL-NDNQ
gi|734902433|ref|WP_034133698.1|damageinducibleprotein[Pseudomonasfluorescens]/11635    VGRVMRNA------P--GK--KRGYVVLPVVIPANT--------E--P---HVAL-NDNQ
gi|490551031|ref|WP_004416146.1|typeIIrestrictionmodificationsystemrestriction/helicasesubunit[Pseudomonassyringae]_gi|4432823/    VGRVMRNA------P--GK--KRGYVVLPVVIPAGM--------E--P---HEAL-NDNQ
gi|489199911|ref|WP_003109152.1|DNAdamageinducibleprotein[Pseudomonasaeruginosa]_gi|552648708|gb|ERY63184.1|hypotheticalprotei/    VGRVMRNA------P--GK--KRGYVVLPVVIPAGM--------E--P---HEAL-NDNQ
gi|730641237|ref|WP_034061328.1|damageinducibleprotein[Pseudomonasaeruginosa]/11636    VGRVMRNA------P--GK--KRGYVVLPVVIPAGM--------E--P---HEAL-NDNQ
gi|751304980|ref|WP_041013386.1|damageinducibleprotein[Pseudomonasxanthomarina]_gi|703407611|emb|CEG51014.1|Helicasedomaincon/1    VGRVMRNA------P--GK--KRGYVVLPVVIPAGM--------E--P---HEAL-NDNQ
gi|939335207|ref|WP_054881520.1|damageinducibleprotein[Pseudomonassp.NBRC111128]/11632    VGRVMRNA------P--GK--KRGYVVLPVVIPAGM--------E--P---HEAL-NDNQ
gi|757693555|ref|WP_042923792.1|damageinducibleprotein[Pseudomonaschloritidismutans]_gi|780910610|gb|KJS78302.1|damageinducib/1    VGRVMRNA------P--GK--KRGYVVLPVVIPAGM--------E--P---HEAL-NDNQ
gi|665953944|ref|WP_031319989.1|damageinducibleprotein[Pseudomonastolaasii]/11626    VGRVMRNA------P--GK--KRGYVVLPVVIPAGM--------E--P---HEAL-NDNQ
gi|771600573|ref|WP_045213746.1|damageinducibleprotein[Pseudomonassp.21]_gi|770081123|gb|KJJ98634.1|damageinducibleprotein[Ps/1    VGRVMRNA------P--GK--KRGYVVLPVVIPAGM--------E--P---HDAL-NDNQ
gi|740871396|ref|WP_038656644.1|damageinducibleprotein[Pseudomonasstutzeri]_gi|627772214|gb|AHY41437.1|damageinducibleprotein/1    VGRVMRNA------P--GK--KRGYVVLPVVIPAGM--------E--P---HEAL-NDNQ
gi|946242205|gb|KRC93782.1|damageinducibleprotein[Pseudomonassp.Root9]/11634    VGRVMRNA------P--GK--KRGYVVLPVVIPAGM--------E--P---HEAL-NDNQ
gi|553777322|ref|WP_023109107.1|MULTISPECIES:hypotheticalprotein[Pseudomonas]_gi|552638674|gb|ERY53184.1|hypotheticalproteinQ05    VGRVMRNA------P--GK--KRGYVVLPVVIPAGM--------E--P---HEAL-NDNQ
gi|896383370|ref|WP_049324618.1|damageinducibleprotein[Pseudomonasstutzeri]/11627    VGRVMRNA------P--GK--KRGYVVLPVVIPAGM--------E--P---HEAL-NDNQ
gi|696158510|gb|AIT41787.1|putativehelicase[Pseudomonassp.LM8]/11630    VGRVMRNA------P--GK--KRGYVVLPVVIPTGM--------E--P---HEAL-NDNQ
gi|504634892|ref|WP_014821994.1|DNAdamageinducibleprotein[Pseudomonasstutzeri]_gi|390985249|gb|AFM35242.1|helicasedomainconta/1    VGRVMRNA------P--GK--KRGYVVLPVVIPAGM--------E--P---HEAL-NDNQ
gi|544783974|ref|WP_021206729.1|DNAdamageinducibleprotein[Pseudomonasstutzeri]_gi|533224779|gb|EQM79688.1|DNAdamageinduciblep/1    VGRVMRNA------P--GK--KRGYVVLPVVIPAGM--------E--P---HEAL-NDNQ
gi|504567385|ref|WP_014754487.1|MULTISPECIES:DNAdamageinducibleprotein[Pseudomonas]_gi|388559493|gb|AFK68634.1|helicasedomain/1    VGRVMRNA------P--GK--KRGYVVLPVVIPAGM--------E--P---HEAL-NDNQ
gi|654441469|ref|WP_027912052.1|damageinducibleprotein[Pseudomonassp.URIL14HWK12:I7]/11630    VGRVMRNA------P--GK--KRGYVVLPVVIPAGM--------E--P---HEAL-NDNQ
gi|941470066|ref|WP_055127894.1|damageinducibleprotein[Pseudomonasmediterranea]/11631    VGRVMRNA------P--GK--KRGYVVLPVVIPAGM--------E--P---HEAL-NDNQ
gi|557815599|ref|WP_023446184.1|DNAdamageinducibleprotein[Pseudomonaschloritidismutans]_gi|557364081|gb|ESQ98190.1|DNAdamagei/1    VGRVMRNA------P--GK--KRGYVVLPVVIPAGM--------E--P---HEAL-NDNQ
gi|727814791|ref|WP_033898247.1|damageinducibleprotein[Pseudomonassp.FH1]_gi|567648005|gb|ETK23229.1|helicasedomaincontaining/1    VGRVMRNA------P--GK--KRGYVVLPVVIPANT--------E--P---HVAL-NDNQ
gi|503278702|ref|WP_013513363.1|DNAdamageinducibleprotein[Desulfovibrioaespoeensis]_gi|316942375|gb|ADU61426.1|typeIIrestrict/    VGRVMRNA------P--GK--KRGYVILPVVIPAGK--------E--P---HEAL-NDNV
gi|505228796|ref|WP_015415898.1|typeIIIrestrictionendonucleasesubunitR[Desulfovibriopiezophilus]_gi|451896976|emb|CCH49855.1|Ty    VGRVMRNA------P--GK--KRGYVILPVVIPAGK--------K--P---HEAL-NDNV
gi|517732543|ref|WP_018902751.1|hypotheticalprotein[Variovoraxparadoxus]/11675    VGRVMRNA------P--GK--ERGYVILPVVIPAGV--------E--P---HDAL-NDNK
gi|550909475|ref|WP_022662570.1|DNAdamageinducibleprotein[Desulfovibriolongus]/11637    VGRVMRTA------P--GK--KRGYVILPVVIPAGM--------E--A---HEAL-NDNK
gi|945494542|gb|KQV50024.1|damageinducibleprotein[Massiliasp.Root335]/11647    VGRVMRNA------P--GK--KRGYVVLPVVIPAGV--------E--P---HDAL-NDNK
gi|334196917|gb|AEG70102.1|helicasedomainprotein[RalstoniasolanacearumPo82]/11663    VGRVMRNA------P--GK--KRGYVILPVVIPAGV--------E--P---HEAL-NDNK
gi|656006746|ref|WP_029048170.1|damageinducibleprotein[Cupriavidussp.amp6]/11661    VGRVMRIA------P--GK--KRGYVVLPVVIPAGV--------E--P---HEAL-NDNK
BPS2196ORF3054P/11659    VGRVMRNA------P--GK--KRGYVVLPVVIPAGV--------E--A---HEAL-NDNK
gi|740932312|ref|WP_038717332.1|damageinducibleprotein[Burkholderiapseudomallei]_gi|760211882|gb|KIX58314.1|damageinduciblepr/1    VGRVMRNA------P--GK--KRGYVVLPVVIPAGV--------E--A---HEAL-NDNK
gi|755810813|ref|WP_042583861.1|damageinducibleprotein[Burkholderiasp.MSHR3999]_gi|755174952|gb|KIP19790.1|DEAD/DEAHboxhelicas/    VGRVMRNA------P--GK--KRGYVVLPVVIPAGV--------E--P---HEAL-NDNK
gi|484586570|gb|AGK48195.1|DEAD/DEAHboxhelicasefamilyprotein[BurkholderiathailandensisMSMB121]/11646    VGRVMRNA------Q--GK--KRGYVVLPVVIPAGV--------E--P---HEAL-NDNK
gi|737582935|ref|WP_035553941.1|damageinducibleprotein[Burkholderiasp.9120]/11658    VGRVMRNA------P--GK--KRGYVVLPVVIPVDV--------E--P---HEAL-NDNK
gi|740989872|ref|WP_038773225.1|damageinducibleprotein[Burkholderiapseudomallei]_gi|705645291|gb|KGS70691.1|DEAD/DEAHboxhelica/    VGRVMRNA------P--GK--KRGYVVLPVVIPAGV--------E--P---HEAL-NDNK
gi|654912525|ref|WP_028363424.1|damageinducibleprotein[Burkholderiasp.JPY366]/11659    VGRVMRNA------P--GK--KRGYVVLPVVIPAGV--------E--A---HEAL-NDNK
gi|740955879|ref|WP_038740512.1|damageinducibleprotein[Burkholderiapseudomallei]_gi|714684932|gb|KGW74438.1|DEAD/DEAHboxhelica/    VGRVMRNA------P--GK--KRGYVVLPVVIPAGV--------E--P---HEAL-NDNK
gi|919203714|ref|WP_052759215.1|damageinducibleprotein[Xanthomonasaxonopodis]/11388    VGRVMRRA------E--GK--KYGYIILPIGVPADL--------T--P---EEAL-RDNK
gi|522152381|ref|WP_020663589.1|hypotheticalprotein[Amycolatopsisbenzoatilytica]/11605    VGRVMRKA------E--GK--DYGYIILPVGVPTDV--------A--P---DKAL-SDNK
gi|663196013|ref|WP_030233899.1|damageinducibleprotein[Streptomycessp.NRRLS455]/11641    VGRVMRLA------P--GK--RYGYIILPVAVPAGM--------S--P---EEAL-ADNR
gi|750429553|ref|WP_040711444.1|damageinducibleprotein[Nocardiopsispotens]/11622    VGRVMRTA------P--GK--KYGYIILPVAVPAGM--------P--P---EKAL-ADNQ
gi|518656139|ref|WP_019819475.1|hypotheticalprotein[Saccharomonosporasaliphila]/11623    VGRVMRVA------P--GK--NYGYIILPVAIPAGT--------P--P---EKAL-ADNR
```

gi|759722933|ref|WP_043433758.1|damageinducibleprotein[Streptomycespluripotens]_gi|733404978|gb|KHK94361.1|damageinduciblepro/1	VGRVMRVA------P--GK--NYGYIILPVAVPAGV--------S--P---EKAL-ADNQ
gi|663424167|ref|WP_030419030.1|damageinducibleprotein[Streptomycessp.NRRLF5065]/11627	VGRVMRVA------P--GK--NYGYIILPVAVPAGV--------S--P---EKAL-TDNH
gi|663412917|ref|WP_030408353.1|damageinducibleprotein[Streptomycessp.NRRLF5917]/11630	VGRVMRVA------P--GK--NYGYIILPVAVPAGV--------A--P---EKAL-ADNQ
gi|671521483|ref|WP_031506489.1|damageinducibleprotein[Streptomycesmegasporus]/11630	VGRVMRVA------P--GK--NYGYIILPVAVPAGV--------S--P---EKAL-ADNQ
gi|740053144|ref|WP_037902776.1|damageinducibleprotein[Streptomycessp.TAA204]/11626	VGRVMRLA------P--GK--NYGYIILPVAIPAGM--------S--P---EAAL-QKNE
gi|663409375|ref|WP_030404974.1|MULTISPECIES:damageinducibleprotein[Streptomyces]/11627	VGRVMRLA------P--GK--NYGYIILPVAIPAGM--------S--P---EVAL-QKNE
gi|746628608|ref|WP_039654277.1|damageinducibleprotein[Streptomycessp.MUSC125]_gi|745644381|gb|KIE25557.1|damageinducibleprot/1	VGRVMRLA------P--GK--NYGYIILPVAIPAGM--------S--P---EAAL-QKNE
gi|727322066|ref|WP_033777320.1|damageinducibleprotein[Salinisporapacifica]/11627	VGRVMRLA------P--GK--NYGYIILPVAIPAGM--------A--P---EKAL-ADNQ
gi|653653242|ref|WP_027655726.1|damageinducibleprotein[Salinisporapacifica]/11618	VGRVMRLA------P--GK--NYGYIILPVAIPAGM--------A--P---EKAL-ADNQ
gi|648632543|ref|WP_026324294.1|damageinducibleprotein[Salinisporapacifica]/11621	VGRVMRLA------S--GK--NYGYIILPVAIPAGM--------A--P---EKAL-ADNQ
gi|881018002|ref|WP_048738463.1|damageinducibleprotein[Corynebacteriumfalsenii]/11676	VGRVMRKA------K--GK--DYGYIILPVAVPPGV--------S--P---AAAL-NDNK
gi|505465526|ref|WP_015652417.1|helicase[Corynebacteriumcallunae]_gi|459385704|gb|AGG67995.1|helicase[CorynebacteriumcallunaeDS	VGRVMRKS------P--GK--DYGYIILPVAVPPGV--------A--P---SAAL-NDSR
gi|41223062|emb|CAF19007.1|DNAorRNAhelicaseofsuperfamilyII[CorynebacteriumglutamicumATCC13032]/11646	VGRVMRKS------P--GK--NYGYIILPVAVPPGV--------A--P---SAAL-NDSR
CGL13032I/11643	VGRVMRKS------P--GK--NYGYIILPVAVPPGV--------A--P---SAAL-NDSR
gi|751257081|ref|WP_040967675.1|damageinducibleprotein[Corynebacteriumglutamicum]_gi|748811620|gb|AJE67931.1|damageinduciblep/1	VGRVMRKA------E--GK--DYGYIILPVAVPPGA--------E--P---STVL-NDNK
gi|749040539|ref|WP_040086524.1|damageinducibleprotein[Corynebacteriumhumireducens]/11663	VGRVMRKS------E--GK--DYGYIILPVAVPPGV--------S--P---SQAL-NDNV
gi|491662899|ref|WP_005519615.1|helicase[Corynebacteriummatruchotii]_gi|224946756|gb|EEG27965.1|helicaseCterminaldomainprotein/	VGRVMRKS------E--GK--DYGYIILPVAVPPNE--------S--P---AAVL-DDNK
gi|746717312|ref|WP_039676231.1|damageinducibleprotein[Corynebacteriumminutissimum]_gi|734991118|gb|KHO29219.1|damageinducibl/1	VGRVMRKA------E--GK--DYGYIILPVAIPPGV--------S--P---SQAL-NDNT
gi|908654892|emb|CQD15529.1|SuperfamilyIIDNA/RNAhelicase(modularprotein)[Corynebacteriumstriatum]/11674	VGRVMRKA------E--GK--DYGYIILPVAIPPGV--------S--P---SQAL-NDNT
gi|895994446|ref|WP_049064418.1|damageinducibleprotein[Corynebacteriumaurimucosum]/11667	VGRVMRKA------E--GK--DYGYIILPVAIPPGV--------S--P---SQAL-NDNT
gi|896123331|ref|WP_049145390.1|damageinducibleprotein[Corynebacteriumaurimucosum]/11669	VGRVMRKA------E--GK--DYGYIILPVAIPPGV--------S--P---SQAL-NDNT
gi|817681178|ref|WP_046645422.1|damageinducibleprotein[Corynebacteriumstriatum]_gi|817551679|gb|KKO79868.1|damageinduciblepro/1	VGRVMRKA------E--GK--DYGYIILPVAIPPGV--------S--P---SQAL-NDNT
gi|896139239|ref|WP_049159146.1|damageinducibleprotein[Corynebacteriumaurimucosum]/11675	VGRVMRKA------E--GK--DYGYIILPVAIPPGV--------S--P---SQAL-NDNT
gi|489943947|ref|WP_003847254.1|helicase[Corynebacteriumammoniagenes]_gi|295968218|gb|EFG81467.1|helicaseCterminaldomainprotei/	VGRVMRKA------E--GK--DYGYIILPVAVAPGV--------S--P---SQAL-NDNS
gi|516653464|ref|WP_018022453.1|helicase[Corynebacteriumdoosanense]_gi|697778282|gb|AIT61018.1|damageinducibleprotein[Coryneba/	VGRVMRKA------E--GK--DYGYIILPVAVAPGT--------S--P---SQAL-NDNA
gi|493894179|ref|WP_006840129.1|helicase[Corynebacteriumlipophiloflavum]_gi|227078819|gb|EEI16782.1|helicaseCterminaldomainpro/	VGRVMRKS------E--GK--DYGYIILPVAVPPGK--------S--P---SEAL-NDNT
gi|740831472|ref|WP_038616755.1|damageinducibleprotein[Corynebacteriumpseudotuberculosis]_gi|666103058|gb|AIG06935.1|putativeh/	VGRVMRKA------E--GK--DYGYIILPVAVSPGV--------S--P---AQAL-NDNA
gi|518023322|ref|WP_019193530.1|helicase[Corynebacteriumtimonense]/11655	VGRVMRKA------E--GK--DYGYIILPVAVPPGV--------S--P---SQAL-NDNA
gi|552767697|ref|WP_023019559.1|MULTISPECIES:hypotheticalprotein[Corynebacterium]_gi|550739013|gb|ERS38609.1|hypotheticalprotei	VGRVMRKA------E--GK--DYGYIILPVAVPPGV--------S--P---SQAL-NDNT
gi|652659121|ref|WP_027017812.1|damageinducibleprotein[Corynebacteriumpseudodiphtheriticum]/11644	VGRVMRKA------E--GK--DYGYIILPVAVPPGV--------S--P---SQAL-NDNT
gi|749039309|ref|WP_040085390.1|damageinducibleprotein[Corynebacteriumhumireducens]/11657	VGRVMRKS------E--GK--DYGYIILPVAVPPGA--------S--P---SEAL-NDNV
gi|740835591|ref|WP_038620874.1|damageinducibleprotein[Corynebacteriumulcerans]_gi|698973854|gb|AIU33664.1|HelicaseCterminald/1	VGRVMRKK------E--GK--DYGYIILPVAVPPGK--------S--P---SEAL-NDNT
gi|805307599|ref|WP_046096452.1|damageinducibleprotein[Corynebacteriumulcerans]_gi|803357323|gb|AKA97638.1|Damageinduciblepro/1	VGRVMRKK------E--GK--DYGYIILPVAVPPGK--------S--P---SEAL-NDNT
gi|504083555|ref|WP_014317549.1|helicase[Corynebacteriumdiphtheriae]_gi|371584249|gb|AEX47914.1|superfamilyIIDNA/RNAhelicase[Co	VGRVMRKS------K--GK--DYGYIILPVAVPPNV--------S--P---SQAL-NDNA
gi|896182193|ref|WP_049193580.1|damageinducibleprotein[Corynebacteriumjeikeium]/11645	VGRVMRKS------E--GK--DYGYIILPVAVAPDA--------S--P---SEAL-NDNQ
gi|917636459|ref|WP_052205460.1|damageinducibleprotein[Corynebacteriumriegelii]_gi|914295811|gb|AKV59187.1|damageinduciblepro/1	VGRVMRKS------E--GK--DYGYIILPVAVPPGK--------S--P---SEAL-NDNT
gi|737013522|ref|WP_035009205.1|damageinducibleprotein[Corynebacteriumjeikeium]/11651	VGRVMRKA------E--GK--DYGYIILPVAVPPGK--------A--P---SEAL-NDNT
gi|737006291|ref|WP_035002094.1|damageinducibleprotein[Corynebacteriumjeikeium]/11658	VGRVMRKS------E--GK--DYGYIILPVAVSPNV--------L--P---AQAL-NDNQ
gi|737010378|ref|WP_035006133.1|damageinducibleprotein[Corynebacteriumjeikeium]/11650	VGRVMRKS------E--GK--DYGYIILPVAVAPNV--------S--P---AQAL-NDNQ
gi|737010473|ref|WP_035006228.1|damageinducibleprotein[Corynebacteriumjeikeium]/11657	VGRVMRKS------E--GK--DYGYIILPVAVPPNM--------S--P---SEAL-NDNT
gi|737006250|ref|WP_035002053.1|damageinducibleprotein[Corynebacteriumjeikeium]/11658	VGRVMRKS------E--GK--DYGYIILPVAVPPST--------S--P---SEAL-NDNT
gi|923018168|ref|WP_053412254.1|damageinducibleprotein[Corynebacteriumlactis]_gi|922845184|gb|ALA67504.1|helicase[Corynebacter/	VGRVMRKS------A--GK--DYGYIILPVAVAPDV--------S--P---AQAL-NDNT
gi|923018885|ref|WP_053412970.1|damageinducibleprotein[Corynebacteriumlactis]_gi|922845845|gb|ALA68165.1|helicase[Corynebacter/	VGRVMRKS------E--GK--DYGYIILPVAVAPDV--------S--P---AQAL-GDNT
gi|512464870|ref|WP_016422869.1|hypotheticalprotein[Corynebacteriumsp.HFH0082]_gi|512047830|gb|EPD47505.1|hypotheticalproteinHM	VGRVMRKS------E--GK--DYGYIILPVAVAPDV--------S--P---AQAL-NDNV
gi|881012697|ref|WP_048733406.1|damageinducibleprotein[Corynebacteriumurealyticum]/11670	VGRVMRKS------E--GK--DYGYIILPVAIAPDV--------S--P---AQAL-NDNV
gi|755608478|ref|WP_042530519.1|damageinducibleprotein[Corynebacteriumsingulare]_gi|754340239|gb|AJI78730.1|putativehelicase[C/	VGRVMRKS------E--GK--DYGYIILPVAVASNV--------S--P---AEAL-NDNQ
gi|491421796|ref|WP_005279593.1|helicase[Corynebacteriumaccolens]_gi|227077180|gb|EEI15143.1|helicaseCterminaldomainprotein[Co/	VGRVMRKS------A--RK--DYGYIILPVAVAQDV--------S--P---SQAL-NDNQ
gi|550752286|gb|ERS51451.1|hypotheticalproteinHMPREF1282_00033[Corynebacteriumsp.KPL1856]_gi|550759961|gb|ERS58964.1|hypothetic	VGRVMRKS------A--GK--DYGYIILPVAVPPNV--------S--P---SEAL-NDNQ
gi|497955053|ref|WP_010269209.1|helicase[Corynebacteriumbovis]/11599	VGRVMRRS------P--DK--DYGYIILPVGVPAGT--------S--P---AEAL-KDNR
gi|860580040|ref|WP_048470569.1|hypotheticalprotein[Mycobacteriumchlorophenolicum]_gi|859098575|gb|KMO76871.1|typeIrestrictione	VGRVMRLS------P--DK--DYGYVILPVAVPACM--------N--P---SEAL-ADNQ
gi|262085905|gb|ACY21873.1|typeIIIrestrictionproteinressubunit[GordoniabronchialisDSM43247]/11632	VGRVMRLS------P--GK--DYGYIILPVAVPSGV--------S--P---AAAL-SDNR
gi|495996338|ref|WP_008720917.1|typeIIIrestrictionendonucleasesubunitR[Rhodococcussp.AW25M09]_gi|443412350|emb|CCQ18455.1|TypeI	VGRVMRLS------P--GK--DYGYIILPVAVPAGV--------S--P---SQAL-ADNR
gi|739284636|ref|WP_037147410.1|damageinducibleprotein[Rhodococcusfascians]/11641	VGRVMRLS------P--GK--DYGYIILPVAVPAGV--------S--P---SQAL-ADNR
gi|739328195|ref|WP_037190574.1|damageinducibleprotein[Rhodococcusfascians]/11643	VGRVMRLS------P--GK--DYGYIILPVAVPAGV--------S--P---SQAL-ADNR
gi|811698335|emb|CMN21041.1|helicase[Mycobacteriumtuberculosis]/11725	VGRVMRKS------P--GK--DYGYVILPVAVPEGV--------E--P---SAAL-ADNK
gi|801577898|emb|CFK23773.1|helicase[Mycobacteriumtuberculosis]/11675	VGRVMRKS------P--GK--DYGYVILPVAVPEGV--------E--P---SAAL-ADNK
MTU18II/11606	VGRVMRKS------P--GK--DYGYVILPVAVPEGV--------E--P---SAAL-ADNK
gi|887719858|emb|CMT27886.1|helicase[Mycobacteriumtuberculosis]/11667	VGRVMRKS------P--GK--DYGYVILPVAVPEGV--------E--P---SAAL-ADNK
gi|621694743|gb|KAS87156.1|hypotheticalproteinAL70_01801[MycobacteriumtuberculosisTKK_03_0115]/11604	VGRVMRKS------P--GK--DYGYVILPVAVPEGV--------E--P---SAAL-ADNK
gi|888128072|emb|CLQ84422.1|helicase[Mycobacteriumtuberculosis]_gi|889917090|emb|CLY86893.1|helicase[Mycobacteriumtuberculosis]	VGRVMRKS------P--GK--DYGYVILPVAVPEGV--------E--P---SAAL-ADNK
gi|884823424|emb|CKL96672.1|helicase[Mycobacteriumtuberculosis]/11466	VGRVMRKS------P--GK--DYGYVILPVAVPEGV--------E--P---SAAL-ADNK
[truncated: 3,016,592 more chars]
